# D_4_-Symmetric Dirhodium Tetrakis(binaphthylphosphate) Catalysts for Enantioselective Functionalization of Unactivated C–H Bonds

**DOI:** 10.1021/jacs.4c06023

**Published:** 2024-07-03

**Authors:** Ziyi Chen, Kristin Shimabukuro, John Bacsa, Djamaladdin G. Musaev, Huw M. L. Davies

**Affiliations:** † Department of Chemistry, Emory University, 1515 Dickey Drive, Atlanta, Georgia 30322, United States; ‡ Cherry L. Emerson Center for Scientific Computation, Emory University, 1521 Dickey Drive, Atlanta, Georgia 30322, United States

## Abstract

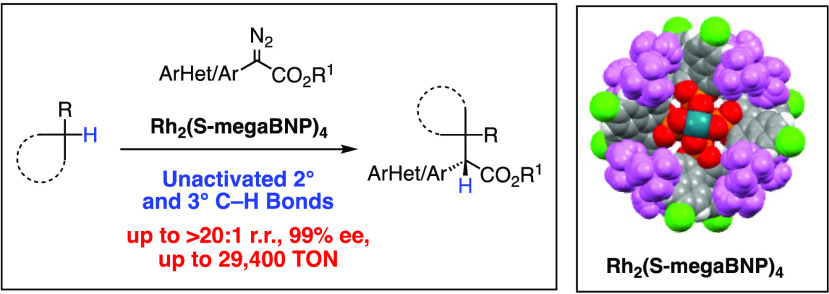

Dirhodium tetrakis(2,2′-binaphthylphosphate) catalysts were successfully developed for asymmetric C–H functionalization with trichloroethyl aryldiazoacetates as the carbene precursors. The 2,2′-binaphthylphosphate (BNP) ligands were modified by introduction of aryl and/or chloro functionality at the 4,4′,6,6′ positions. As the BNP ligands are C_2_-symmetric, the resulting dirhodium tetrakis(2,2′-binaphthylphosphate) complexes were expected to be D_4_-symmetric, but X-ray crystallographic and computational studies revealed this is not always the case because of internal T-shaped CH−π and aryl–aryl interactions between the ligands. The optimum catalyst is Rh_2_(*S*-megaBNP)_4_, with 3,5-di(*tert*-butyl)phenyl substituents at the 4,4′ positions and chloro substituents at the 6,6′ positions. This catalyst adopts a D_4_-symmetric arrangement and is ideally suited for site-selective C–H functionalization at unactivated tertiary sites with high levels of enantioselectivity, outperforming the best dirhodium tetracarboxylate catalyst developed for this reaction. The standard reactions were conducted with a catalyst loading of 1 mol % but lower catalyst loadings can be used if desired, as illustrated in the C–H functionalization of cyclohexane in 91% ee with 0.0025 mol % catalyst loading (29,400 turnover numbers). These studies further illustrate the effectiveness of donor/acceptor carbenes in site-selective intermolecular C–H functionalization and expand the toolbox of catalysts available for catalyst-controlled C–H functionalization.

## Introduction

**Figure 1:**
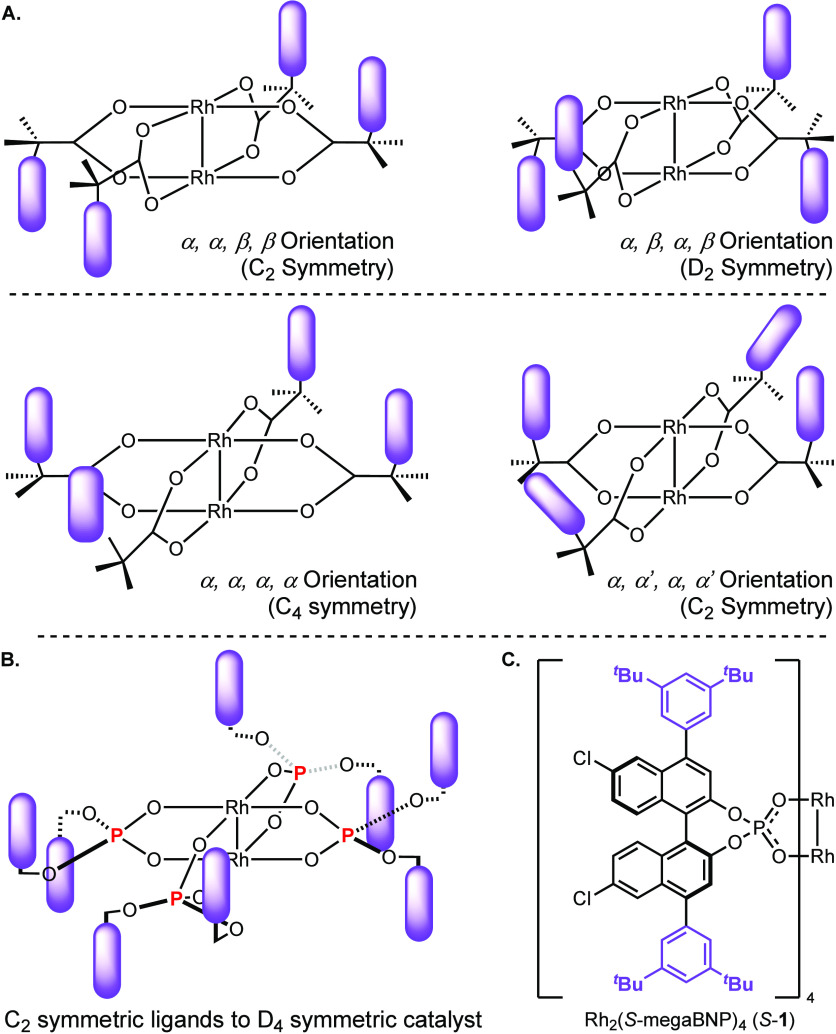
**A**. High symmetry orientations of dirhodium tetracarboxylates. **B**. Model of the D_4_-symmetric arrangement with C_2_-symmetric phosphate ligands. **C**. Structure of the optimum catalyst, Rh_2_(*S*-megaBNP)_4_.

Homoleptic chiral dirhodium tetracarboxylates have been shown to be tremendously effective catalysts, especially for carbene and nitrene transfer reactions.^1−4^ Depending on the nature of the chiral ligands, they can self-assemble during formation of the dirhodium complexes to generate catalysts with higher symmetry than the ligands themselves, C_2_, C_4_, or D_2_ symmetric, as illustrated in Figure 1A.^2,3^ As the catalysts have two rhodium coordination sites, the high symmetry arrangement is advantageous because it would limit the number of different orientations when the carbene binds to the dirhodium complex. We and others have designed a wide range of high symmetry dirhodium tetracarboxylate catalysts^2−4^ that have shown broad applicability in the reactions of donor/acceptor^1a,1c,2,3^ and donor/donor carbenes.^1d,1e^ Our most recent work has focused on C_4_-symmetric bowl-shaped catalysts, which require blocking of one of the rhodium coordination sites to make them effective chiral catalysts.^3k,4b^ Therefore, we decided to explore whether appropriately designed C_2_-symmetric binaphthylphosphate (BNP) ligands,^5^ which would be expected to generate Rh_2_(BNP)_4_ complexes of D_4_ symmetry with both rhodium sites being identical (Figure 1B), would have distinctive characteristics and broaden the scope of enantioselective C–H functionalization reactions. In this paper, we describe a new binaphthylphosphate dirhodium catalyst, Rh_2_(*S*-megaBNP)_4_ (*S*-**1**) (Figure 1C), and demonstrate that even though its conformational mobility is more complex than had been anticipated, it is very effective for asymmetric C–H functionalization with donor/acceptor carbenes.

**Figure 2:**
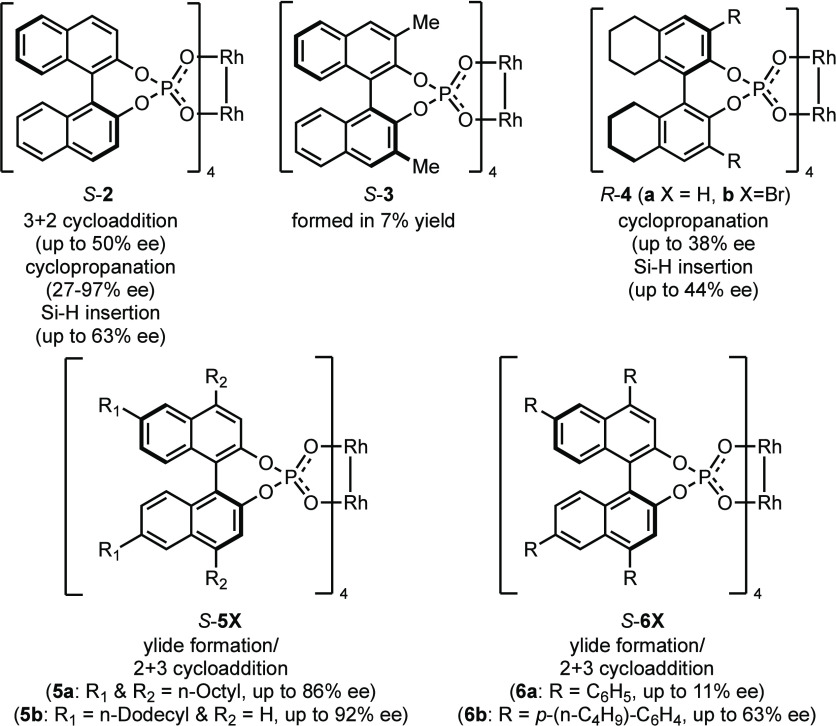
Representative examples of previously studied dirhodium tetrakis(binaphthylphosphonate) catalysts (*R*-**X** or *S*-**X**, depending on which enantiomer of the ligand is used).

The use of chiral binaphthylphosphate ligands in dirhodium catalysis started in the early 1990s,^6^ but their application is far less developed compared to the dirhodium tetracarboxylate and carboxamidate catalysts.^1−4^ A few examples are known where reasonably high levels of asymmetric induction were achieved, but the scope of these reactions is limited. Figure 2 illustrates the most significant catalysts that have been developed.^6a,7^ In the pioneering studies by Pirrung,^6a^ the parent Rh_2_(*S*-BNP)_4_, *S*-**2**, complex was shown to be capable of up to 50% ee in cycloaddition reactions (Figure 2). Later studies by Davies showed that, in the cyclopropanation reactions with aryldiazoacetates, *R*-**2** was capable of relatively high levels of asymmetric induction but only when methoxy substituents were present in the aryl ring.^7p^ The main challenge associated with the binaphthylphosphate ligands is how to modify their structure to enhance the asymmetric induction exhibited by the dirhodium catalysts. Typically, when dirhodium complexes of binaphthylphosphonic acids themselves are used as chiral protic catalysts, far superior performances can be obtained when bulky substituents are introduced at 3,3′ positions in the binaphthyl.^8^ However, the introduction of bulky substituents at this position is not feasible for these dirhodium complexes because the C3 substituents of one ligand will sterically interfere with the adjacent ligand. Consequently, the dirhodium tetrakis(binaphthylphosphate) catalysts can only be formed when the C3 substituent is either hydrogen or methyl, and the yield for formation of the C3 methyl-substituted complex *S*-**3** is very low (7%).^7o^ Another option is to use partially hydrogenated ligands, but catalyst *R*-**4a** still gives only moderate levels of asymmetric introduction (up to 44% ee).^7f^ To date, the most promising studies are those by Hodgson who examined asymmetric cycloaddition of oxonium ylides derived from the dirhodium(tetra-binaphthylphosphate)-carbene intermediates. The Rh_2_[*S*-4,4′,6,6′-tetra-*n*-octyl-BNP]_4_ catalyst *S*-**5**, with bulky *N*-octyl substituents, designed for increased solubility,^7e,7l^ similar to the tactic used with the dirhodium tetraprolinate catalysts,^3b^ performs well in enantioselective cycloaddition reactions, resulting in up to 86% (92%) ee.^7l^ However, the corresponding tetraphenyl catalyst *S*-**6a**, which is closely related to the current catalyst design, results in much lower levels of enantioselectivity (11% ee), although the *p*-*n*-butylphenyl derivative *S*-**6b** gave up to 63% ee.^7e^ It should be noted that another C_2_-symmetric phosphate ligand class that have been successfully applied to generate D_4_-symmetric dirhodium catalysts are the spiro ligands developed by Zhou,^9^ but we decided to focus on binaphthylphosphates because of their ease of synthesis.

**Figure 3:**
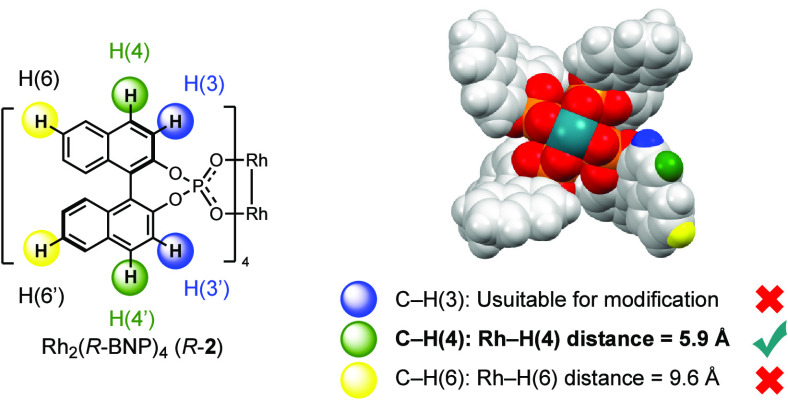
Rationale for catalysts optimization studies illustrated on the X-ray structure of *R*-**2**.

In order for the dirhodium tetrakis(binaphthylphosphate) catalysts to match the range of highly asymmetric transformations possible with dirhodium tetracarboxylates, we reasoned that further ligand optimization is needed. The X-ray crystallographic structure (Figure 3)^7f^ and our computational studies (see Figure S7.2) of Rh_2_(*R*-BNP)_4_, *R*-**2**, indicate that this complex adopts a structure that is D_4_-symmetric. This complex, however, has a relatively flat structure, with no major components of the ligands pointing directly toward the carbene binding site, which may explain why the asymmetric induction with *R*-**2** is typically modest. Therefore, we decided to explore whether introduction of large functionality into the BNP ligands would improve the asymmetric induction exhibited by this class of catalysts. As mentioned above, large functionality at C3 of the naphthyl group (color coded blue) cannot be accommodated because groups at this position would interfere with the adjacent ligands. The C4 position (colored green) appears best for introduction of sterically influencing groups, whereas the C6 position (colored yellow) is too far away from the rhodium. Even substituents at C4 would need to be large because it is still located relatively far away from the rhodium coordination site. On the basis of this initial analysis, the tetraphenyl derivative *S*-**6a** appeared to be a promising starting point because the phenyl groups are highly amenable for modification into larger groups by means of metal-catalyzed cross-coupling reactions. Therefore, we decided to begin our studies by evaluating *S*-**6a** in a standard C–H functionalization reaction as a reference reaction and then analyzing its structure to understand its limitations. Then, we examined a series of more bulky derivatives, following our central hypothesis that bulky C4 substituents would be a requirement for enhanced enantioselectivity.

## Results and Discussion

**Scheme 1:**
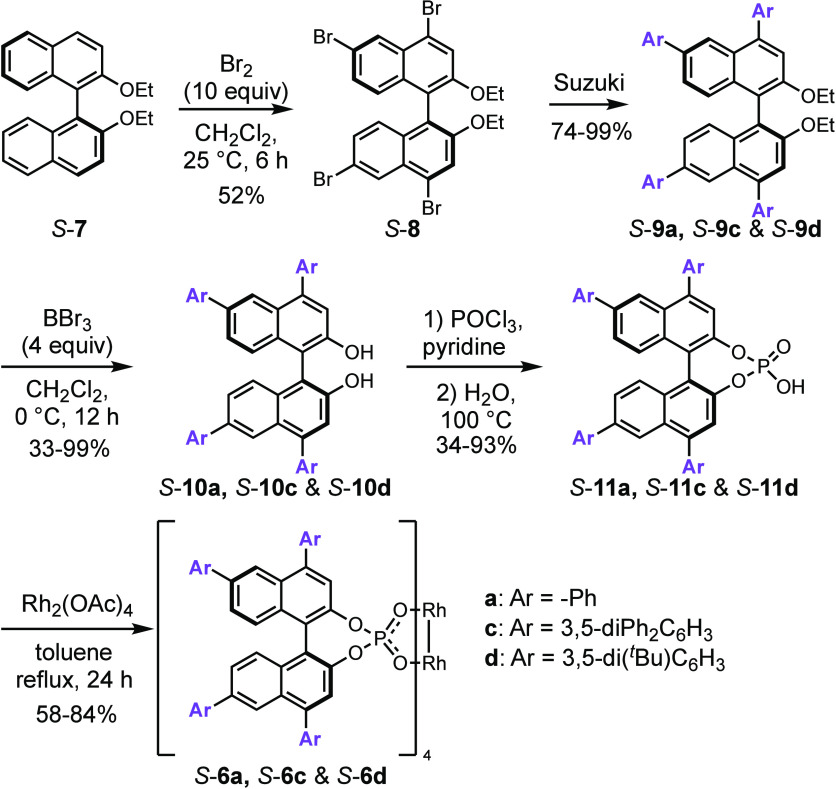
Synthesis of Tetraarylbinaphthylphosphate Catalysts *S*-6a,c,d

The synthetic route to a series of the 4,4′,6,6′-tetraarylbinaphthylphosphate catalysts is summarized in Scheme 1, following a procedure adapted from the one that has been used previously for the synthesis of tetraphenyl derivative *S*-**6a**.^7e^ Bromination of the binaphthyl ether *S*-**7** preferentially occurs at the 6,6′ positions, but the 4,4′ position can also be brominated under more forcing conditions to generate the tetrabromo derivative *S*-**8**. Tetra-fold Suzuki coupling on *S*-**8** generated a series of tetraaryl derivatives *S*-**9a**,**c**,**d**, which on de-etherification to form *S*-**10a**,**c**,**d**, followed by generation of the phosphonic acid *S*-**11a**,**c**,**d** and ligand exchange with dirhodium tetraacetate, generated the desired binaphthylphosphate catalysts *S*-**6a**,**c**,**d**.

The binaphthylphosphate catalysts *S*-**2**, *S*-**6a**, *S*-**6c**, and *S*-**6d** were tested for their effectiveness at asymmetric induction in a standard C–H functionalization of cyclohexane (Table 1) using the bromoaryldiazoacetate **12a** as the carbene source to form the functionalized product **13a**.^10^ The parent catalyst *S*-**2** generated **13a** in only 27% ee, while the previously known tetraphenyl catalyst *S*-**6a**^7e^ gave **13a** in 44% ee. Gratifyingly, a significant enhancement was obtained with the 3,5-disubstituted aryl catalysts *S*-**6c** and *S*-**6d**, which generated **13a** in 79% and 85% ee, respectively.

**Table 1:**
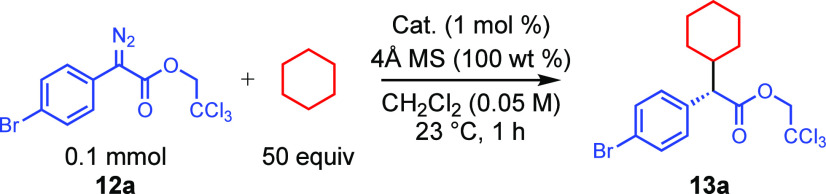
Initial Catalyst Screening of C–H Functionalization of Cyclohexane^a^

Entry	Catalyst	NMR yield^b^ (%)	ee (%)
1	*S*-**2**	54	**27**
2	*S*-**6a**	66	**44**
3	*S*-**6c**	66	**79**
4	*S*-**6d**	61	**85**

a Reaction conditions: catalyst (1 mol %), cyclohexane (50 equiv), 4 Å MS (100 wt %), 1 mL of CH_2_Cl_2_ in a 4 mL vial, diazo (0.1 mmol) in 1 mL of CH_2_Cl_2_ was added over 1 h via syringe pump at 23 °C. The ee values were determined by chiral HPLC analysis.

b NMR yields were determined with trichloroethylene as internal standard (6.47 ppm)

**Figure 4:**
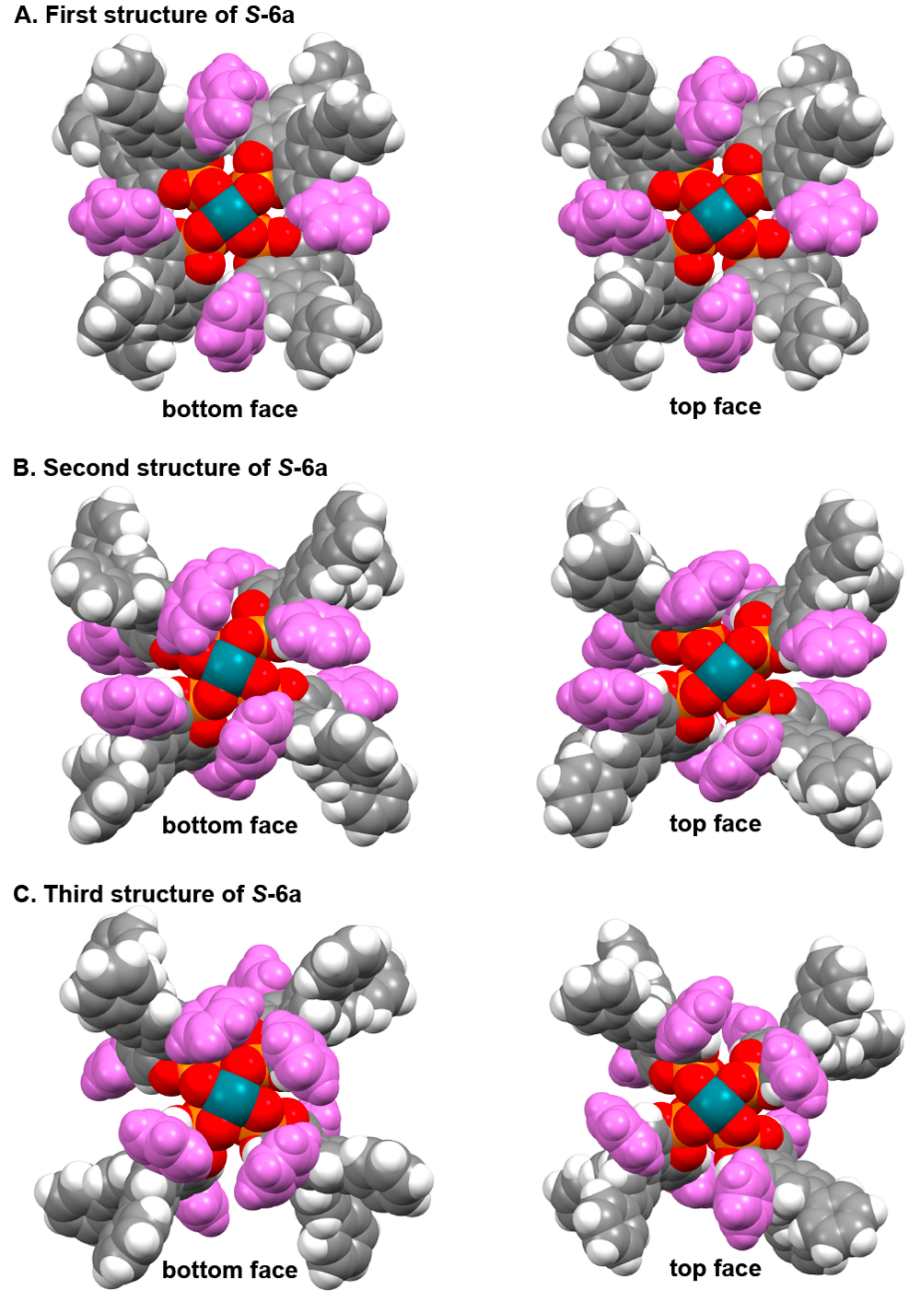
Three distinct conformers of *S*-**6a** in the crystal structure unit cell. Two views from each conformer are given (the 4,4′-phenyl substituents are colored in purple to enhance the visualization). None of the conformers is in a high symmetry arrangement.

Even though the enantioselectivity in the C–H functionalization improved with increasing the size of the aryl substituent on the catalyst, the results are still below what would have been possible with the chiral dirhodium tetracarboxylate catalysts.^10^ To gain further insight into these catalysts, we prepared suitable crystals of the tetraphenyl catalyst *S*-**6a** for X-ray crystallographic analysis. At the onset of this work, we expected all the catalysts to adopt a D_4_-symmetric orientation, as had been reported for the parent catalyst, *S*-**2**,^7f^ but this was definitely not the case for *S*-**6a**. The unit cell contained three molecules of *S*-**6a**, and they were in different conformations, none of which had D_4_ symmetry (Figure 4). This result indicates that the expectation that all of the catalysts would routinely be D_4_ symmetric is not a given outcome.

**Figure 5:**
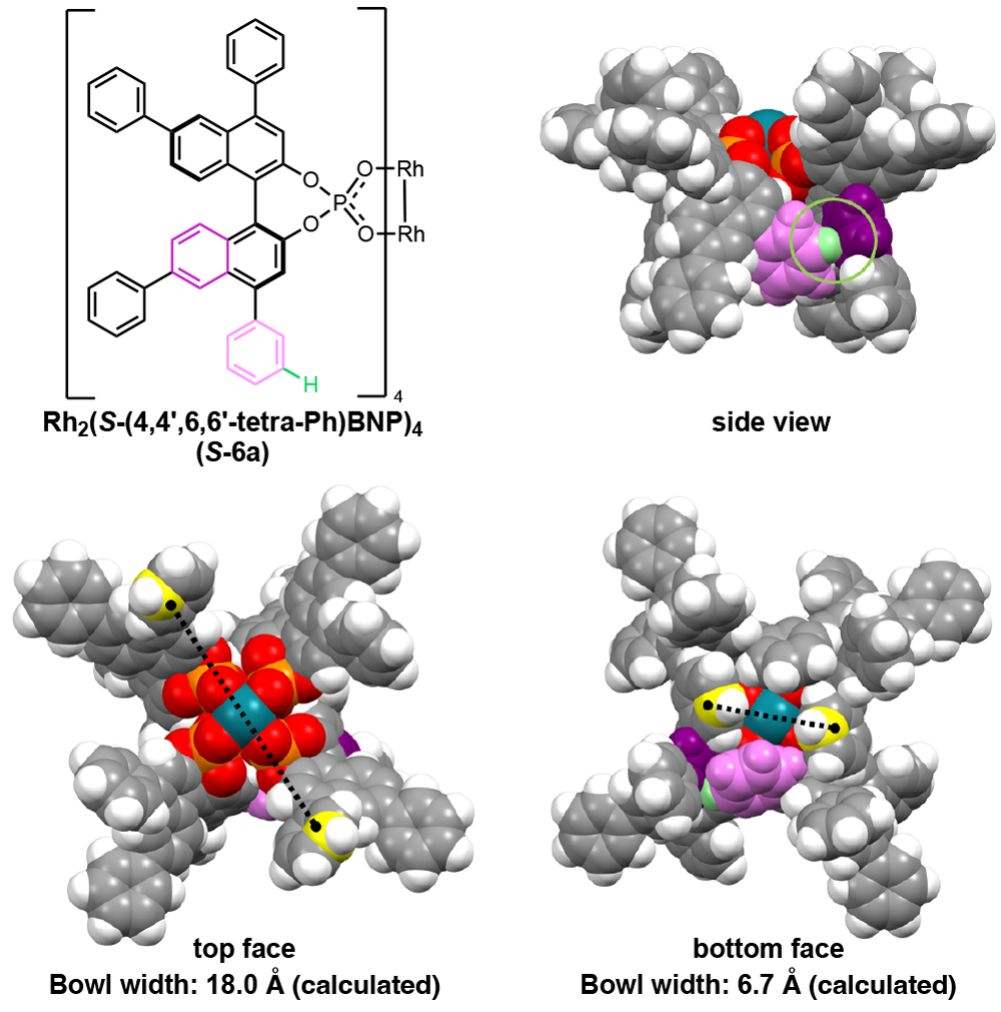
Computationally minimized structure of *S*-**6a** showing evidence of T-shaped CH−π interaction, which disrupts the expected D_4_ symmetry of the catalysts. Bowl width values are measured across the catalyst bowl between the innermost *meta*-positioned carbon atoms (yellow) of the 4,4′-aryl substituents.

The unexpected variability in how the BNP ligands of *S*-**6a** orient themselves in the crystal packing would likely also influence the catalyst structure in solution. In order to evaluate this expectation, we carried out computational studies on *S*-**6a** and its various derivatives (see the Supporting Information). These calculations were conducted at the {[B3LYP-D3(BJ)] + PCM(DCM)}/[6-31G(d,p) + Lanl2dz] level of theory (see the Supporting Information for details).^11,12^ The calculations on *S*-**6a** converged to the structure shown in Figure 5. As seen from this figure, the calculated structure of *S*-**6a** is not D_4_ symmetric and exhibits several T-shaped CH−π and π–π interactions between the Ph-rings, as well as the Rh−π(Ph) interactions, which result in closing of one side of the catalyst versus the other site and decreasing symmetry of the catalyst to either C_2_ (Figure 4A) or no simplified symmetry at all (Figure 4B,C). The calculated bowl widths are 6.7 and 18.0 Å for the top and bottom sides of the catalyst *S*-**6a**, respectively (see Figure 5). Interestingly, the use of B3LYP instead of the B3LYP-D3BJ approach reduces the difference between the top- and bottom-site bowl width of catalyst *S*-**6a** from 11.3 to 3.3 Å (the B3LYP calculated top-site and bottom-site bowl widths are 12.0 and 15.3 Å, respectively). Comparison of the above presented findings at the B3LYP and B3LYP-D3BJ levels of theory illustrates the critical importance of weak interaction in defining the structure of the Rh_2_(*R*-tetraarylbinaphthylphosphate) complexes,^13^ and the structural flexibility of the BNP ligands in *S*-**6a** (for details, see the Supporting Information, Figure S7.6). We also used computation to explore whether the 6,6′ phenyl groups in *S*-**6a** have a major influence on the catalyst structure and found that the unsubstituted 6,6′-H and 6,6′-Cl-substituted analogues of this catalyst adopt an almost identical orientation to *S*-**6a** (see the Supporting Information for details, Figures S7.4 and S7.8).

**Scheme 2:**
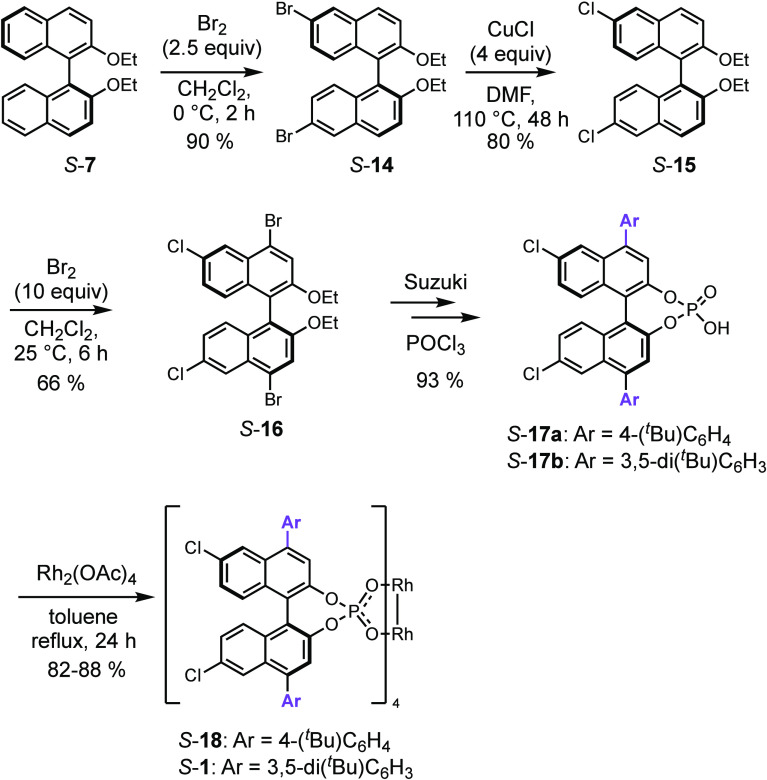
Synthesis of the Rh_2_-[6,6′-dichloro-4,4′-diarylbinaphthylphosphate] Catalysts *S*-18 and Rh_2_(*S*-megaBNP)_4_ (*S*-1)

The computational studies show that the 4,4′-diaryl substituents play a pivotal role in determining whether the catalysts adopt a high symmetry structure and the 6,6′ substitutions have a limited effect. Therefore, we decided to adjust the design of the next catalysts to focus on bulky 4,4′-diaryl substituents, while maintaining the same groups at the 6,6′ positions. The direct synthesis of 4,4′-disubstituted binaphthols with no substituents at the 6,6′ positions is challenging because the 6,6′ positions are favored for electrophilic aromatic substitution.^7b−7e^ Therefore, we embarked on the synthesis of binaphthylphosphate catalysts with bulky aryl substituents at C4, C4′ and smaller chlorine substituents at C6, C6′, as illustrated in Scheme 2. Bromination of *S*-**7** under mild conditions^7b−7d^ resulted in the selective formation of the 6,6′-dibromo derivative *S*-**14**. Treatment of *S*-**14** with copper(I) chloride generated the 6,6′-dichloro derivative *S*-**15**,^14^ which then could be dibrominated at the 4,4′ positions to form *S*-**16**. Double Suzuki coupling of *S*-**16** only occurred at the bromide and subsequent reactions that were used in Scheme 1, generated the desired ligands *S*-**17a** and *S*-**17b** with 4,4′-aryl substituents. The ligand exchange with dirhodium tetraacetates generated the desired catalysts *S*-**18** and Rh_2_(*S*-megaBNP)_4_ (*S*-**1**). Although the initial plan was to prepare a library of catalysts in this series, the excellent performance of *S*-**1** precluded the necessity to prepare an extended library.

The two new catalysts were evaluated in the standard C–H functionalization with cyclohexane, and the results are summarized in Table 2. The *para*-*tert*-butylphenyl derivative *S*-**18** was an effective catalyst but the enantioselectivity during the formation of **13a** remained moderate (54% ee). In contrast, the 3,5-di-*tert*-butylphenyl catalyst *S*-**1** was exceptional, generating **13a** in 99% ee. In these initial studies, a vast excess of cyclohexane was used, but good yield and enantioselectivity were still achieved with 10 equiv of cyclohexane, generating **13a** in an 85% isolated yield and 99% ee. Less than 10 equiv of cyclohexane led to a slightly decreased yield and enantioselectivity, but even with just 1 equiv of cyclohexane, **13a** was formed in 70% NMR yield and 97% ee (Table 2, entry 4).

**Table 2:**
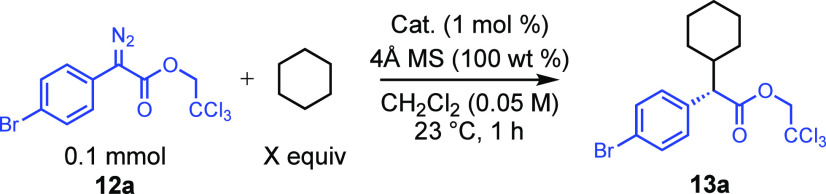
C–H Functionalization of Cyclohexane Using *S*-18 and *S*-1 as Catalysts^a^

Entry	Catalyst	X equiv	NMR yield^c^ (%)	ee (%)
1	*S*-18	50	72	54
2	*S*-1	50	94	99
3	*S***-1**	**10**	**94(85**^d^**)**	**99**
4^b^	*S*-1	5	76(71^d^)	97
5^b^	*S*-1	1	70(63^d^)	97

a Reaction conditions and analysis were the same as described in Table 1.

b 0.5 mol % catalyst was used.

c NMR yields were determined with trichloroethylene as internal standard (6.47 ppm).

d Isolated yield.

**Figure 6:**
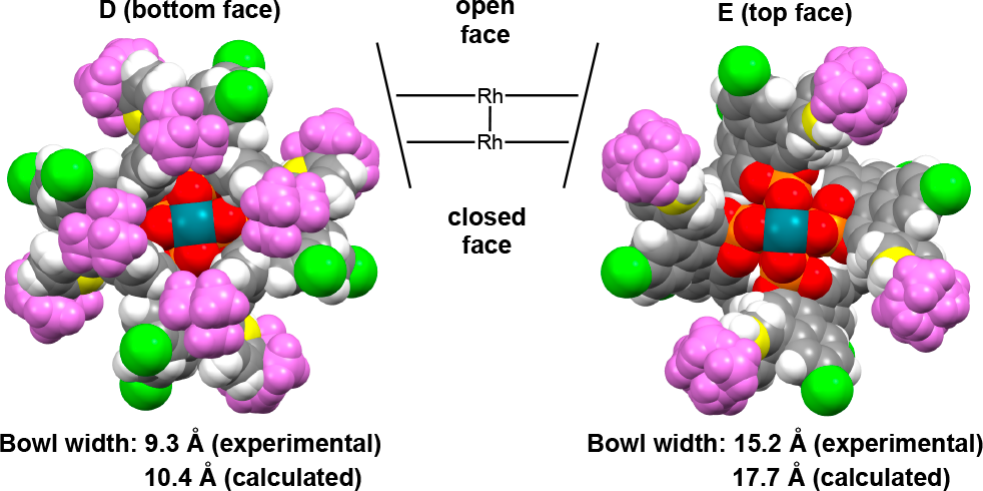
C_4_-symmetric crystal structure of *S*-**18** showing a bottom view **D** and a top view **E**. (The 4,4′-aryl substituent is colored in purple to enhance the visualization.) One face of the catalyst is open, and the other is closed. Bowl width values are measured across the catalyst bowl between the innermost *meta*-positioned carbon atoms (yellow) of the 4,4′-aryl substituents.

The difference in enantioselectivity observed with catalysts *S*-**18** and *S*-**1** is dramatic, and so, further structural analyses of these catalysts were performed to understand what were the stereochemical controlling factors. X-ray and computational (see the Supporting Information, Figure S7.10, for details) studies of *S*-**18** (see Figure 6) show that it has a more ordered ligand orientation than the tetraphenyl catalyst *S*-**6a** but it still does not adopt a D_4_-symmetric structure. Instead, it adopts C_4_ symmetry. One face of the catalyst has the four *tert*-butylphenyl groups attracted toward each other (structure **D**), with a bowl-width of 9.7 Å (the calculated value is 10.4 Å), whereas on the other face (structure **E**) four *tert*-butylphenyl groups are spread apart with a bowl-width of 14.3 Å (the calculated value is 17.7 Å), Thus, one face (structure **E**) of catalyst *S*-**18** is quite open and the other face (structure **D**) is still relatively closed. In other words, either the catalyst will have two distinctive faces for carbene binding and/or the ligands will have conformational mobility between the two structures. In either case, the overall effect is that this catalyst would not have a well-defined orientation for the carbene coordination, and this is presumably reflected in the moderate enantioselectivity it exhibited.

**Figure 7:**
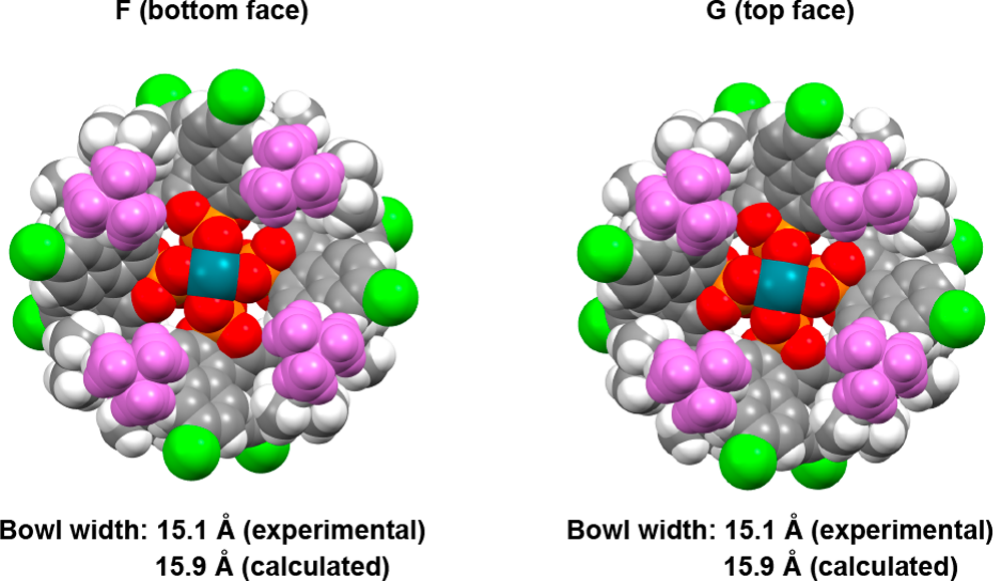
D_4_-symmetric crystal structure of *S*-**1** showing a bottom view **F** and a top view **G**. (The 4,4′-aryl substituent is colored in purple to enhance the visualization.) Bowl width values are measured across the catalyst bowl between the innermost *meta*-positioned carbon atoms (yellow) of the 4,4′-aryl substituents.

In contrast to the results above, the X-ray structure of the optimum catalyst, Rh_2_(*S*-megaBNP)_4_ (*S*- **1**), indicates that it adopts a D_4_-symmetric arrangement (Figure 7). The sterically more demanding 3,5-di-*tert*-butylphenyl does not appear to accommodate a closer approach of this functionality on one face of the catalyst versus the other, and hence, both faces of the catalysts are identical. DFT structural optimization studies were conducted, starting from the X-ray structure of *S*-**1**, but the structure remained virtually unchanged (see the Supporting Information, Figure S12, for details). This indicates that the solid-state orientation is likely to be the same in solution. Due to the high symmetry, the four potential binding orientations for each face of the catalyst are identical (or almost identical), which leads to a greater likelihood for the catalyst to be capable of achieving high asymmetric induction. Furthermore, the C_4_ or D_4_ symmetry of catalysts *S*-**18** and Rh_2_(*S*-megaBNP)_4_ (*S*-**1**), supports the hypothesis that having large aryl groups at the 4,4′ positions favors organization of the complex in a high symmetry orientation.

**Figure 8:**
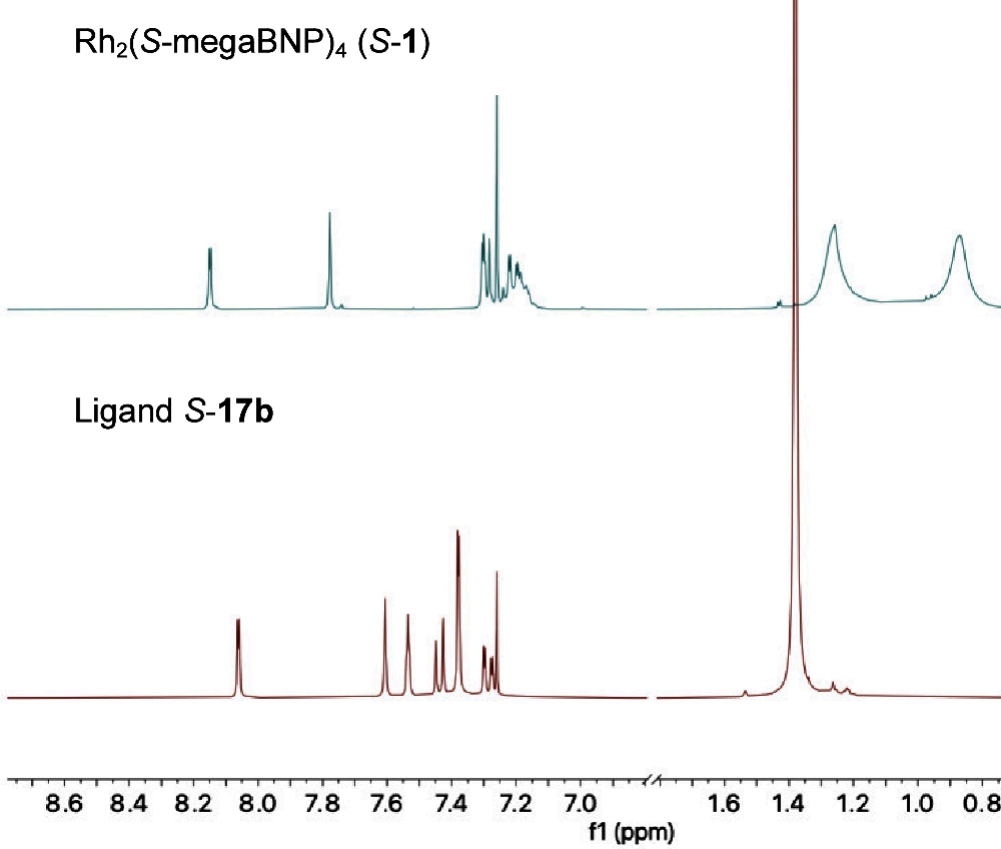
Proton NMR spectra of catalyst Rh_2_(*S*-megaBNP)_4_ (*S*-**1**) and ligand *S*-**17b**. When the complex *S*-**1** is formed, the spectra are considerably broadened, and the signals for the *tert*-butyl groups occur at 1.2 and 0.8 ppm, with one of them considerably shielded.

Even though the X-ray crystallographic and computational studies of Rh_2_(*S*-megaBNP)_4_ (*S*-**1**) indicated that it maintains a D_4_-symmetric structure, the proton NMR spectrum indicated that *S*-**1** has hindered conformational mobility. This can be readily seen by comparing the NMR spectra of ligand *S*-**17b** and catalyst *S*-**1** (Figure 8). The proton NMR signals for the ligand are sharp, whereas the signals for the catalyst are broad, indicating the existence of significant conformational barriers. Furthermore, there are some major changes in the chemical shifts with some signals deshielded, most notably the *tert*-butyl group from 1.4 ppm in the ligand **17b** to two signals at 1.3 and 0.9 ppm in the complex *S*-**1**. Therefore, further NMR studies were conducted to determine whether the solid-state structure of *S*-**1** was a realistic view of the solution structure or whether other hindered rotation issues were in play.

**Figure 9:**
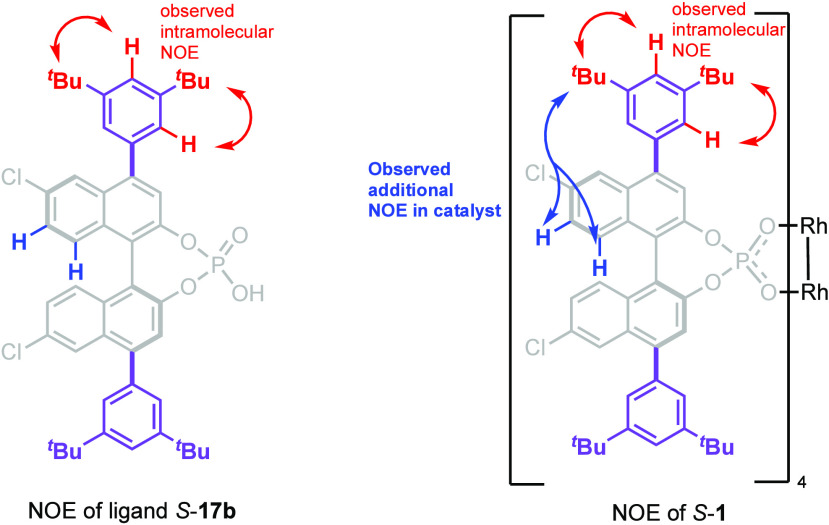
NOE enhancements observed in the ligand *S*-**17b** and catalyst *S*-**1**. (See the Supporting Information, Figures S6.1–S6.6, for the detailed spectral data.)

Variable NMR studies revealed that the conformational barrier was about 13 kcal/mol (see the Supporting Information in Figure S6.1 for the details of the variable temperature NMR experiments). In order to determine what was likely causing the conformational barrier, nuclear Overhauser effect (NOE) studies were conducted (see Supporting Information, Figures S6.3–S6.6 for details). Of particular significance to this analysis were the data obtained for the NOE exhibited by the *tert*-butyl groups as shown in Figure 9. In the free ligand *S*-**17b** the *tert*-butyl group had the expected positive NOE to the *ortho*-hydrogens on the benzene ring and the C3 and C5 hydrogens on the naphthyl ring. In complex *S*-**1**, NOE enhancements were seen not only to these same aromatic protons but also to the C7 and C8 protons on the naphthyl ring, which should be too far removed from the *tert*-butyl group for NOE.

**Figure 10:**
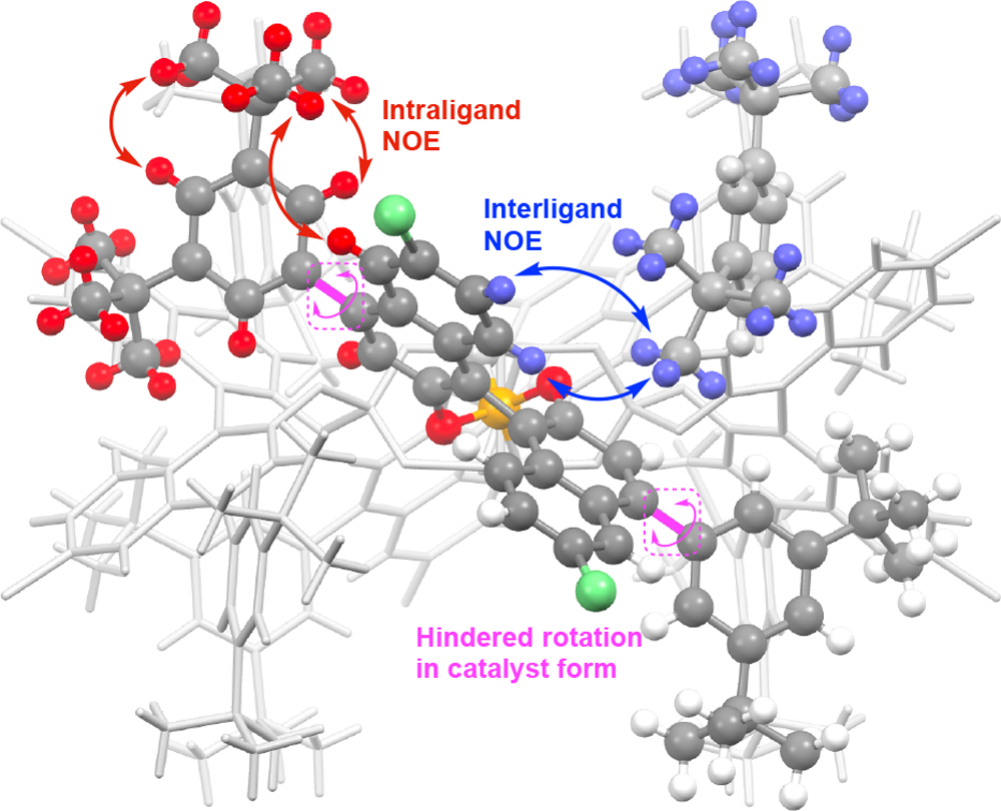
Key NOE enhancements between the *tert*-butyl groups and aromatic protons. Red arrows indicate the enhancement of NOE within the same ligand. Blue arrows indicate the enhancement of NOE with protons in the adjacent ligands.

On examining the crystal structure of *S*-**1**, it is clear that a *tert*-butyl group of one ligand is closely aligned to the naphthyl ring of the adjacent ligand, and we propose that the catalyst in solution adopts a similar structure to the X-ray structure and the additional NOEs seen in **1** compared to the ligand are due to intermolecular interaction between the *tert*-butyl group and the adjacent ligands. Of particular significance is that both *tert*-butyl groups cause the NOE effect, even though only one is in close proximity to the naphthyl group of the adjacent ligand. This would indicate that the hindered rotation is occurring between the binaphthyl and the di-*tert*-butylphenyl bond (Figure. 10). The barrier for rotation is less in the ambient temperature NMR studies of the ligand *S*-**17b** but becomes greater in the complex *S*-**1** because of additional intermolecular interactions between the *tert*-butyl group of one ligand and the binaphthyl and the di-*tert*-butylphenyl fragments of adjacent ligands.

Having established that Rh_2_(*S*-megaBNP)_4_ (*S*-**1**) is the optimum catalyst and developed a reasonable understanding of why it is so effective, we then began to explore its synthetic potential in C–H functionalization reactions. The first series of experiments examined the influence of *p*-substituted aryldiazoacetates and a few heteroaryldiazoacetates on the enantioselectivity of the C–H functionalization reaction (Table 3). In general, with the dirhodium tetracarboxylates, we have found that donor/acceptor carbenes with trihaloethyl esters are better than those with a standard methyl ester in the functionalization of unactivated C–H bonds and often result in higher levels of enantioselectivity.^15^ The reaction of aryldiazoacetates to form products **13a**–**c** compare the influence of the ester group on the reactions catalyzed by *S*-**1**. All three give effective transformations, but the enantioselectivity with the trichloroethyl ester (99% ee) is higher than the methyl ester (90% ee) and the trifluoroethyl ester (91% ee). High enantioselectivity can be obtained when the *p*-substituent is electron withdrawing, as seen with **13d**–**f** (92–95% ee), but the trifluoromethanesulfonyl derivative does not do as well, forming **13i** in 78% ee. A *p*-phenyl substituent generates **13g** with high enantioselectivity (92% ee), but there is a slight drop in the enantioselectivity with the *p*-tolyl derivative, forming **13h** in 86% ee. Other aromatic systems were also examined to form products **13j**–**l**. The 2-naphthyl and 4-chloropyridyl diazo derivatives perform well, forming **13j** in 90% ee and **13k** in 94% ee, respectively, but the chloropyrimidine derivative generated **13l** with only 65% ee. The diazo compounds that performed the worst were the ones with an electron donating methoxy group (61% ee, product **13m**), a bulky *tert*-butyl group (41% ee, product **13n**), and the parent phenyl derivative lacking a *para* substituent (56% ee, product **13o**).

**Table 3:**
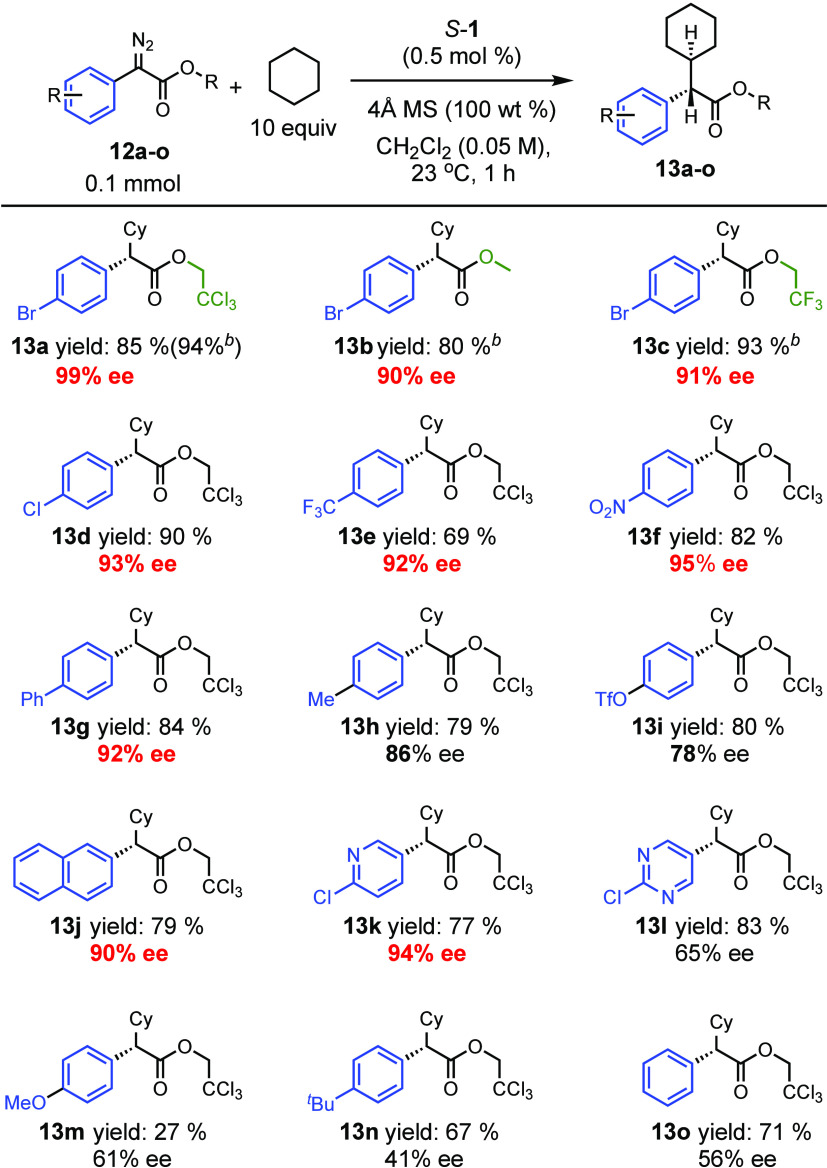
C–H Functionalization of Cyclohexane with *p*-Substituted Aryldiazoacetates^a^

a Reaction conditions: catalyst (0.5 mol %), cyclohexane (10 equiv), 4 Å MS (100 wt %), 1 mL of CH_2_Cl_2_ in a 4 mL vial, diazo (0.1 mmol) in 1 mL of CH_2_Cl_2_ was added over 1 h via syringe pump at 23 °C. Isolated yields were given. The ee values were determined by chiral HPLC analysis.

b NMR yields were determined with trichloroethylene as internal standard (6.47 ppm).

**Figure 11:**
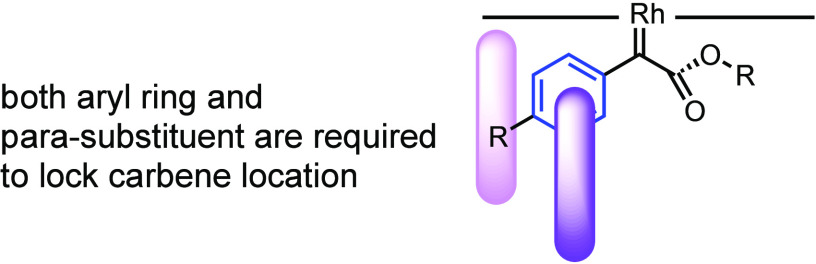
Working hypothesis: electron-withdrawing *para* substituted aryl rings are needed to lock the rhodium carbene in a defined position in the catalyst.

The variable enantioselectivity, depending on the nature of the *para* substituent, leads to the hypothesis that even though the catalyst is likely to be D_4_ symmetric in solution, it is still necessary for there to be a well-defined interaction between the *p*-substituted aryl group and the wall of the catalyst to lock the ligand/carbene interaction (Figure 11). As the aromatic rings in the catalyst are electron rich, it would be reasonable that the most effective aryl group on the carbene would be electron withdrawing. An aryl group plus the *para* substituent appears to be a requirement for an effective interaction with the catalyst and not just a phenyl group. If the group is too large, such as *tert*-butyl (**13n**) or is absent (**13o**), there is a considerable drop in the level of asymmetric induction. Computational studies were attempted on the structures of the carbene-[both Ph-trichloroethyl and (*p*-Br)Ph-trichloroethyl] bound *S*-**1** complexes. These carbene complexes were too big for complete frequency analyses, but their optimized structures (see the Supporting Information, Figure S7.13) show that the *para* substituent causes the carbene to orientate itself between two adjacent ligands, whereas the orientation is not so stringent when an unsubstituted phenyl ring is present.

In order to test the working hypothesis further, control experiments were conducted with differentially substituted aryldiazoacetates. The reference substrate was the *p*-chloro derivative, which had been shown to generate **13d** in 93% ee (Table 4, entry 1). When the reaction was conducted on the *m*-chloro or *o*-chloro derivatives, the enantioselectivity was considerably lower (48% ee for **13p** and 60% ee for **13q**, respectively). Low enantioselectivity was also observed with a variety of *meta* substituents as shown in the formation of **13r**–**t** (18–49% ee). Interestingly, even though 3,5-dibromo derivative **13u** was formed with low levels on enantioselectivity (11% ee), the 3,4-dichloro and 3,4-dibromo derivatives, **13v** and **13w**, were both formed in 91% ee. These control studies further support the hypothesis that an aryl group with a *para* substituent is a crucial component for achieving high asymmetric induction in the C–H functionalization reactions with *S*-**1**.

**Table 4:**
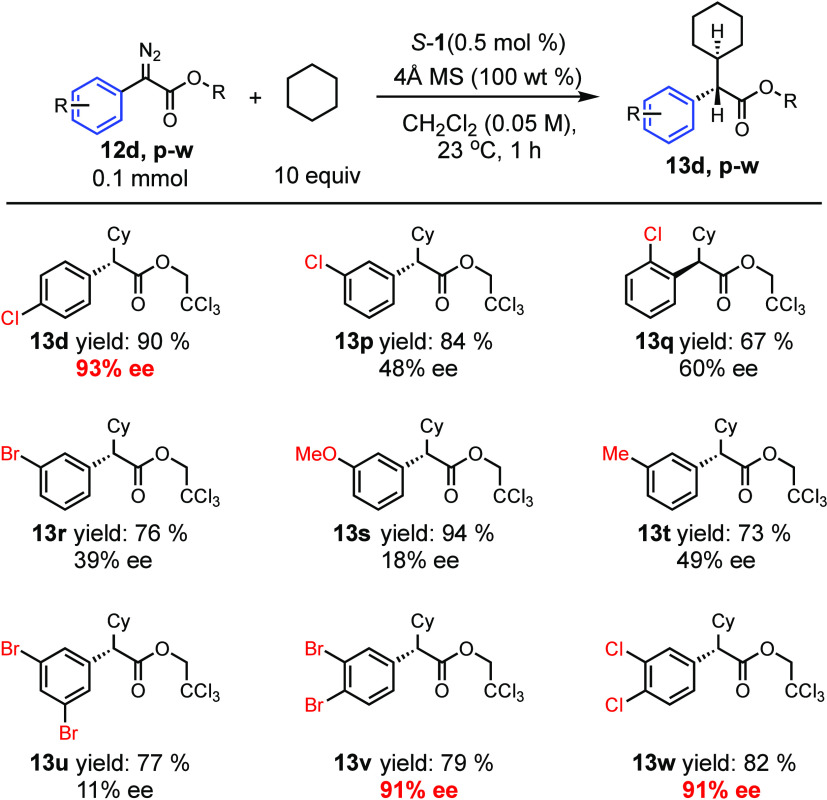
Testing the Working Hypothesis with Differentially Substituted Haloaryldiazoacetates^a^

a Reaction conditions: catalyst (0.5 mol %), cyclohexane (10 equiv), 4 Å MS (100 wt %), 1 mL of CH_2_Cl_2_ in a 4 mL vial, diazo (0.1 mmol) in 1 mL of CH_2_Cl_2_ was added over 1 h via syringe pump at 23 °C. Isolated yields were given. The ee values were determined by chiral HPLC analysis.

**Scheme 3:**
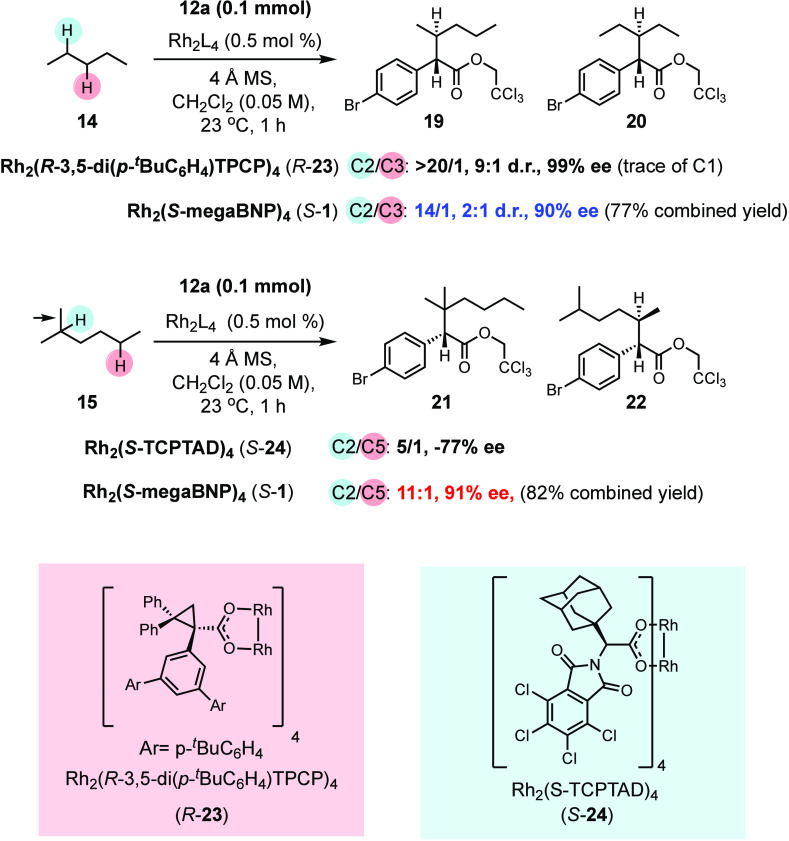
Comparison of Rh_2_(*S*-megaBNP)_4_ (*S*-1) with the Established Chiral Dirhodium Tetracarboxylate Catalysts

Having established that Rh_2_(*S*-megaBNP)_4_ (*S*-**1**) is capable of high levels of asymmetric induction, we then examined its influence on catalyst-controlled site-selective and enantioselective functionalization of unactivated C–H bonds. The dirhodium tetracarboxylate catalysts are capable of exceptional site selectivity, and so, we decided to challenge *S*-**1** and see how it would compete against some of the best dirhodium tetracarboxylate catalysts (Scheme 3). Pentane (**14**) and 2-methylhexane (**15**) were used as the two test substrates. The bulky D_2_-symmetric catalyst, Rh_2_(*R*-3,5-di(*p*-^*t*^BuC_6_H_4_)TPCP)_4_ (*R*-**23**), has been shown to drive the C–H functionalization of donor/acceptor carbenes toward the most accessible secondary C–H bond.^3i^ In the case of pentane, a clean reaction occurs at C2, favoring **19**, with no observed reaction occurring at C3 to form **20**. The only regioisomer formed is a trace amount of C–H functionalization at the methyl group. Furthermore, the C–H functionalization to form **19** proceeds with 9:1 dr and in 99% ee. The reaction of pentane with *S*-**1**, as catalyst, gave a 14:1 site selectivity for C2 functionalization (**19**) over C3 functionalization (**20**), indicating that it is not as sterically demanding as Rh_2_(*R*-3,5-di(*p*-^*t*^BuC_6_H_4_)TPCP)_4_ and thus does not distinguish as well between the two methylene sites. Furthermore, the C2 diastereoselectivity for the formation of **19** is inferior (2:1 d.r) to that of the *R*-**23**-catalyzed reaction (9:1 d.r). The second comparison is against the best tertiary selective catalyst, Rh_2_(S-TCPTAD)_4_ (*S*-**24**). This catalyst is less sterically demanding than *R*-**23** and preferentially reacts at the most accessible tertiary C–H bond.^3j^ The head-to-head comparison using 2-methylhexane (**15**) as a substrate reveals that *S*-**1** competes very well with *S*-**24**. Not only does it give enhanced site selectivity for the tertiary site to preferentially form **21** over **22** (11:1 r.r. versus 5:1 r.r.), but also the level of asymmetric induction at the tertiary group to form **21** is enhanced (91% ee for *S*-**1**, versus 77% ee for *S*-**24**).

As Rh_2_(*S*-megaBNP)_4_ (*S*-**1**) competes well with *S*-**24** for site-selective tertiary C–H functionalization, a detailed study was conducted on a range of substrates **25a**–**m**, and the results are described in Table 5. The parallel reactions with Rh_2_(*S*-TCPTAD)_4_ are included in the Supporting Information for comparison purposes (see Table S4.2.1). The reactions were conducted under two reaction conditions. Condition A uses an excess of trap, and this is very effective for cheap volatile hydrocarbons. Condition B uses 2 equiv of the aryldiazoacetates and was preferred when more elaborate substrates were used. *S*-**1**-catalyzed reactions strongly prefer the most accessible tertiary C–H bonds (**25a**–**d**) although a readily accessible secondary C–H bond can still be a competitive site (**25b**). The reaction can be carried out in the presence of other functionality, as illustrated with **25e**–**i**. Bromo, phthalimido, *p*-substituted phenoxy, and boronates are compatible with these reactions. In all cases, the enantioselectivity is high, ranging from 80 to 95% ee. The reaction can also be conducted on other cyclic substrates, as illustrated with **25j**–**l**. The reaction with adamantane is particularly impressive, as C–H functionalization product **26l** is formed in 96% ee (entry 3).

**Table 5:**
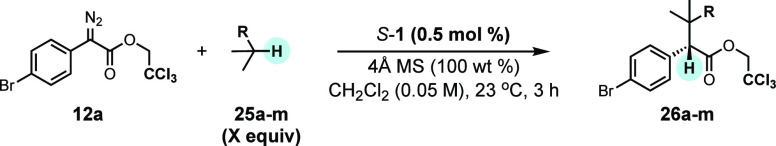

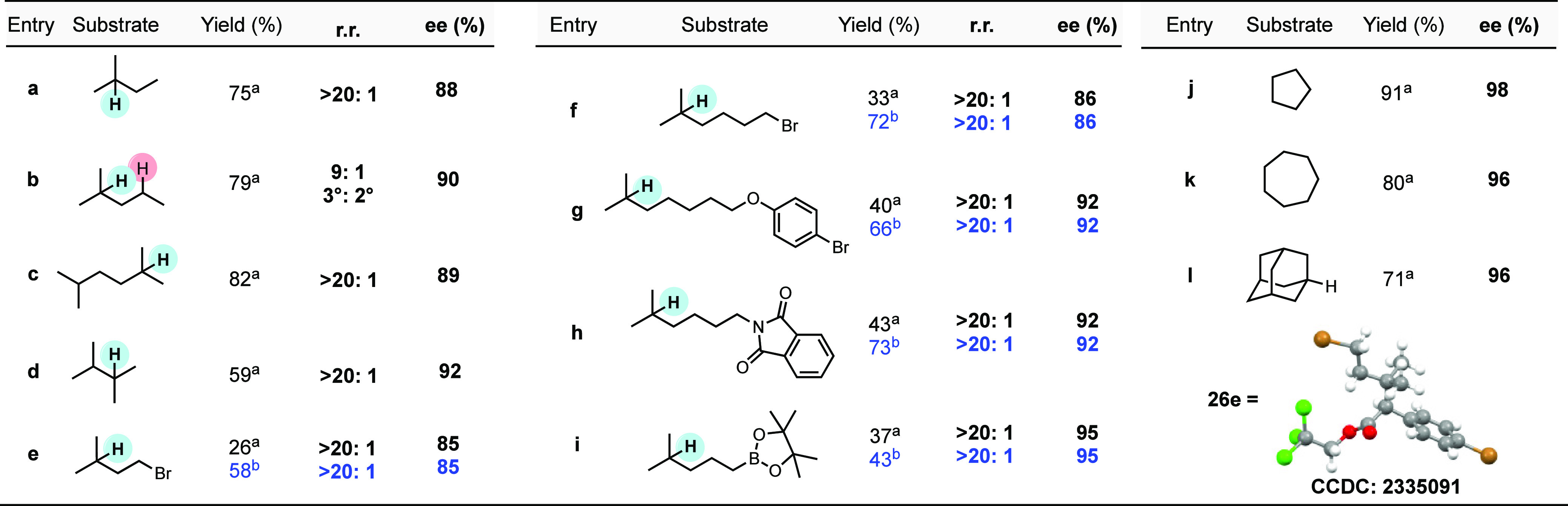
*S*-1 Catalyzed Selective C–H Functionalization at Tertiary C–H Bonds

a Reactions conditions: catalyst (0.5 mol %), **25a**–**f** and **25j**–**l** (1 mmol, 10 equiv) or **25g**–**i** (2 equiv), 4 Å MS (100 wt %), 1 mL of CH_2_Cl_2_ in a 4 mL vial, **12a** (0.1 mmol, 1 equiv) in 1 mL of CH_2_Cl_2_ was added over 3 h via syringe pump at 23 °C. Isolated yields were given. The ee values were determined by chiral HPLC analysis.

b Reactions conditions: catalyst (0.5 mol %), **25e**–**i** (0.1 mol, 1 equiv), 4 Å MS (100 wt %), 1 mL of CH_2_Cl_2_ in a 4 mL vial, **12a** (0.2 mmol, 2 equiv) in 1 mL of CH_2_Cl_2_ was added over 3 h via syringe pump at 23 °C. Isolated yields were given. The ee values were determined by chiral HPLC analysis.

**Scheme 4:**
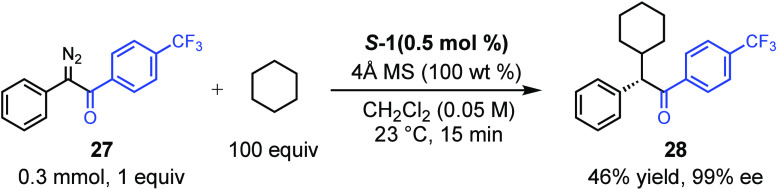
Rh_2_(*S*-megaBNP)_4_ (*S*-1) Catalyzed C–H Functionalization with Diazoketone 27

The studies so far have been conducted using aryldiazoacetates, which are the most widely used carbene precursors in the C–H functionalization reactions. However, it is possible to extend the chemistry to other acceptor groups such as aryl diazo ketones,^3k^ shown in Scheme 4. For an effective reaction with the diazoketone **27**, it was necessary to use a large excess of cyclohexane but under these conditions the C–H functionalization product **28** was generated with very high levels of asymmetric induction (99% ee), albeit in moderate yield (46%).

**Scheme 5:**
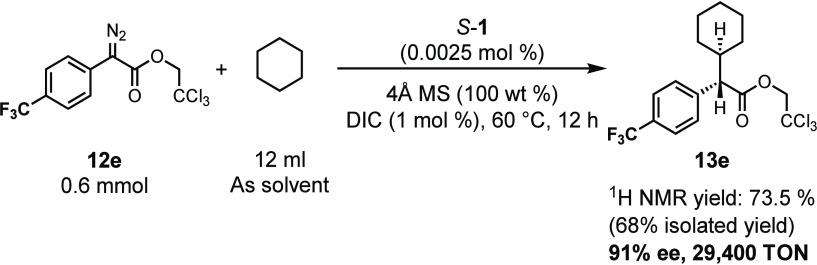
Rh_2_(*S*-megaBNP)_4_ (*S*-1) Catalyzed C–H Functionalization under Low Catalyst Loading

The dirhodium tetracarboxylate catalysts are capable of achieving very high turnover numbers (TONs) in the reactions of donor/acceptor carbenes.^10b,16^ Therefore, we conducted a brief study to evaluate the kinetic efficiency of *S*-**1** in the reaction of the aryldiazoacetate **12e** with cyclohexane. Previously, we had shown that the optimum reaction conditions for high TON C–H functionalization with dirhodium tetracarboxylates were conducted at elevated temperature (60 °C) and used cyclohexane as solvent and an aryldiazoacetate with an electron withdrawing group on the aryl ring. Furthermore, the presence of small amount of DCC or DIC enhanced the TONs. As a test reaction, we conducted a reaction using the optimized conditions with a catalyst loading of 0.0025 mol % (Scheme 5). Under these conditions, the C–H functionalization product **13e** was formed in 68% isolated yield (29,400 TON) and in 91% ee. This brief evaluation indicates that the phosphonate catalysts are capable of high TON’s if desired.

## Conclusion

In summary, we have prepared a series of chiral dirhodium tetrakis(binaphthylphosphate) catalysts and demonstrated their utility in site-selective and enantioselective functionalization of unactivated C–H bonds. At the onset of this work, all the catalysts were expected to be D_4_-symmetric, but these studies revealed that this is not necessarily the case, The catalysts need to be carefully designed for them to adopt a D_4_-symmetric structure and avoid symmetry-breaking T-shaped CH−π interactions. Among these catalysts, Rh_2_(*S*-megaBNP)_4_ (*S*-**1**) displays excellent site selectivity and enantioselectivity for functionalization of unactivated secondary and tertiary C–H bonds of cyclic alkanes and unactivated tertiary C–H bonds of various acyclic substrates. This work broadens the scope of chiral dirhodium catalysts capable of selective C–H functionalization by donor/acceptor carbenes.

## Data Availability

The data underlying this study are available in the published article and its online Supporting Information.

## Supporting Information Available

Complete experimental procedures, materials, computational details and findings, compound characterizations, and Cartesian coordinates of all calculated systems (PDF)
