# Supplementary material for: D4-Symmetric Dirhodium Tetrakis(binaphthylphosphate) Catalysts for Enantioselective Functionalization of Unactivated C–H Bonds
Source: J Am Chem Soc. 2024 Jul 3;146(28):19460–73. doi: 10.1021/jacs.4c06023 (PMC11258696; doi:10.1021/jacs.4c06023)
Supplement: Supplementary file 1 — ja4c06023_si_001.pdf [file ja4c06023_si_001.pdf]

Supporting Information

D_4-Symmetric Dirhodium Tetrakis(binaphthylphosphate) Catalysts for Enantioselective Functionalization of Unactivated C–H Bonds

Ziyi Chen[1], Kristin Shimabukuro[1], John Bacsa[1], Djamaladdin G. Musaev*[,1,2] & Huw M. L. Davies*[,1]

[1]Department of Chemistry, Emory University, 1515 Dickey Drive, Atlanta, 30322, Georgia, USA

[2]Cherry L. Emerson Center for Scientific Computation, Emory University, 1521 Dickey Drive, Atlanta, Georgia 30322, United States

Email: hmdavie@emory.edu, dmusaev@emory.edu

Table of Contents

1. General Information ***1***

2. General Procedures ***3***

3. Procedure of the synthesis of intermediates and catalysts and their characterization ***8***

4. Characterization of C–H Insertion Products ***24***

4.1 Crude NMR Data for Determination of Regioselectivity and Diastereoselectivity **24**

4.2 Data Analysis for Scheme 3 .... **34**

4.3 Procedure and Characterization of C–H Insertion Products **42**

5. High Turnover Number Study ***64***

6. VT-NMR Study for S-1 and ^{1}H, NOESY NMR study for S- 17b and S- 1 .... ***65***

7. Calculation ***73***

8. NMR Spectra ***127***

9. HPLC and SFC Chromatograms ***190***

10. X-Ray Crystallographic Data ***235***

10.1 X-Ray Crystallographic Data for *S*-6a **235**

10.2 X-Ray Crystallographic Data for *S*-18 .... **368**

10.3 X-Ray Crystallographic Data for S-1 .... **394**

10.4 X-Ray Crystallographic Data for 26e **421**

11. Reference ***430***

1. General Information

All reagents and solvents were used as purchase from commercial sources (Sigma-Aldrich, Oakwood, TCI, Ambeed, Strem) for substrate synthesis unless otherwise noted. Dichloromethane used in C–H insertion reactions was prepared from solvent purification system and stored over activated 4 Å molecular sieves, which were activated at 220 °C for 4 hours under vacuum and stored in an oven over 100 °C. All column chromatography was performed on silica gel (SiliaFlash® P60, 40-63 μm). Thin layer chromatographic (TLC) analysis was performed with aluminum-sheet silica gel plates.

2-Methylpentane (**25b**) and 2,3-dimethylbutane (**25d**) were distilled before using.

^{1}H, ^{13}C, ^{19}F and ^{31}P NMR spectra were recorded at 800 MHz (^{13}C at 201 MHz) on Bruker-800 spectrometer, 600 MHz (^{13}C at 151 MHz) on Bruker-600 spectrometer or Varian IVONA-600 spectrometer, 500 MHz on Varian INOVA-500 spectrometer, or 400 MHz (^{13}C at 101 MHz, ^{19}F at 376 MHz, ^{31}P at 162 MHz) on Bruker-400 spectrometer and all were reported in parts per million (ppm). Unless otherwise noted, ^{1}H, ^{13}C, ^{19}F and ^{31}P NMR spectra were performed in solutions of deuterated chloroform ($CDCl_3$) with the residue chloroform set as an internal standard (7.26 ppm for ^{1}H NMR, and 77.16 ppm for ^{13}C NMR). Abbreviations for signal multiplicity are as follow: br = broad, s = singlet, d = doublet, t = triplet, q = quartet, sept = septet, m = multiplet, dd = doublet of doublet, ddd = doublet of doublet of doublet, etc. Coupling constants (J values) were calculated directly from the spectra.

IR spectra were collected on a Nicolet iS10 FT-IR spectrometer.

Mass spectra were taken on a Thermo Finnigan LTQ-FTMS spectrometer with APCI, ESI.

Optical rotation were determined by Autopol IV(Rudolph Research Analytical).

Enantiomeric excess data were obtained from either **Agilent 1100 series instrument High Performance Liquid Chromatography (HPLC)**, **Agilent 1290 series instrument Ultra-High Performance Liquid Chromatography (UPLC)** or Waters ACQUITY UPC^2 Supercritical Fluid Chromatography (SFC). The HPLC system operated with HPLC grade isopropanol/n-hexane gradient and commercial ChiralPak/ChiralCel columns from Daicel Chemical Industries, notably ChiralPak AD-H (5 μm particle size, 4.6 mm vs. 250 mm), ChiralCel OD-H (5 μm particle size, 4.6 mm vs. 250 mm), ChiralPak AS-H (5 μm particle size, 4.6 mm vs. 250 mm), and Regis (R,R) Whelk-O 1 from Regis Technologies (5 μm particle size, 4.6 mm vs. 250 mm). The UPLC system operated with HPLC grade isopropanol/n-hexane gradient and the commercial ChiralPak column

from Daicel Chemical Industries, notably Regis (S,S) Whelk-O 1 from Regis Technologies (1.8 µm particle size Kromasil, 4.6 mm vs. 100 mm). The SFC system operated with supercritical CO_2 and HPLC grade (50% methanol in isopropanol with 0.2% formic acid) and commercial ChiralPak/ChiralCel columns from Daicel Chemical Industries, notably ChiralCel OJ-3 (3 µm particle size, 3.0 mm vs. 150 mm), Regis (S,S) Whelk-O 1 from Regis Technologies (3.5 µm particle size, 3.0 mm vs. 150 mm), and Trefoil AMY1 from Waters (2.5 µm particle size, 3.0 mm vs. 150 mm).

Chiral HPLC or SFC conditions were determined by obtaining separation of the racemic products using Rh_2(*R/S*-BNP)$_4$, Rh_2(*R/S*-TCPTAD)$_4$ or Rh_2(*R/S*-megaBNP)$_4$ as catalyst for C–H insertion reactions.

Warning: Diazo compounds are known to have potential thermal stability issues[1a] and are explosive hazards. Even though we did not have problems in our work, working with diazo compounds should be carefully performed in a well-ventilated hood, require the use of PPE, and careful handling of the reagents.

2. General Procedures

General Procedure A

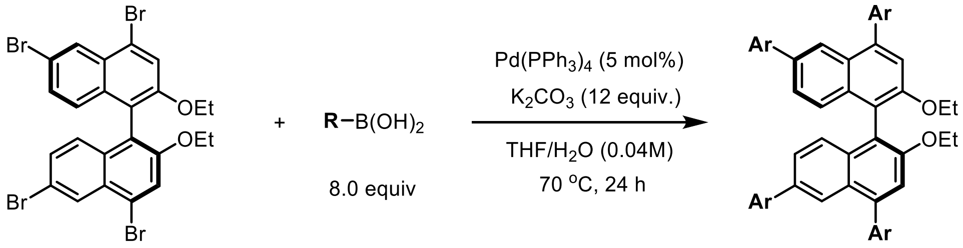

To a 100 mL three-necked round-bottom flask with a condenser in the middle, containing a magnet stir bar, was added (*S*)-4,4',6,6'-tetrabromo-2,2'-diethoxy-1,1'-binaphthalene (1.0 equiv) and tetrakis(triphenylphosphine)palladium(0) (0.05 equiv). Then the flask was vacuumed and refiled with N_2 for three times and half of the THF (degassed) was added in the flask to start stirring the solution at 70 °C for 15 min. The corresponding aryl boronic acid (8.0 equiv) dissolved in the other half of THF (degassed), followed by potassium carbonate (12.0 equiv) dissolved in deionized water (degassed), was added in the flask via syringe. Overall concentration of the solution is 0.04 M and $V_{THF} : V_{water} = 1 : 1$. After stirring at 70 °C for 24 h, the flask was removed from heating and was cooled to room temperature before the solution was filtered and washed with ethyl acetate to remove the palladium black. The filtrate was concentrated to remove most of THF and was then extracted with ethyl acetate (same volume of the solution) for three times. The combined organic layer was dried over anhydrous sodium sulfate and then was concentrated as the crude product, which was eventually purified by column chromatography with silica gel to afford the product.

General Procedure B

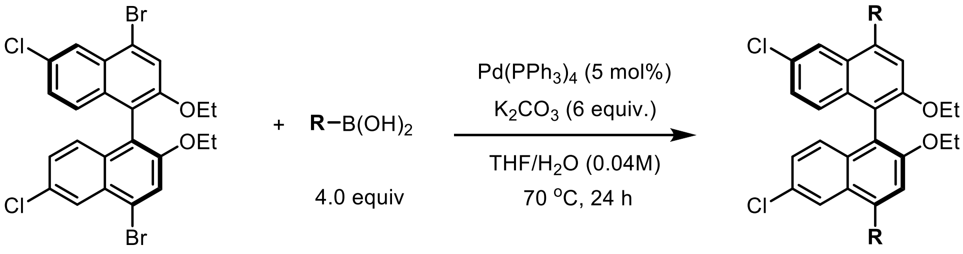

To a 100 mL three-necked round-bottom flask with a condenser in the middle, containing a magnet stir bar, was added (*S*)-4,4'-dibromo-6,6'-dichloro-2,2'-diethoxy-1,1'-binaphthalene (1.0 equiv) and tetrakis(triphenylphosphine)palladium(0) (0.05 equiv). After that, the flask was vacuumed and refiled with N_2 for three times and half of the THF (degassed) was added in the flask to start stirring

the solution at 70 °C for 15 min. The corresponding aryl boronic acid (4.0 equiv) dissolved in the other half of THF (degassed), followed by potassium carbonate (6.0 equiv) dissolved in deionized water (degassed), was added in the flask via syringe. Overall concentration of the solution is 0.04 M and $V_{THF} : V_{water} = 1 : 1$. After stirring at 70 °C for 24 hours, the flask was removed from heating and was cooled to room temperature before the solution was filtered and washed with ethyl acetate to remove the palladium black. The filtrate was concentrated to remove most of THF and was then extracted with ethyl acetate (same volume of the solution) for three times. The combined organic layer was dried over anhydrous sodium sulfate and then was concentrated to afford the crude product, which was eventually purified by column chromatography with silica gel.

General Procedure C

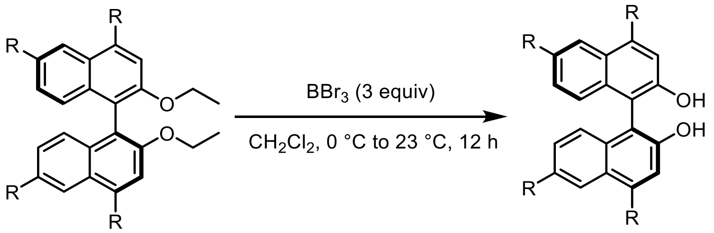

To a 100 mL round-bottom flask with a magnetic stir bar was added the corresponding ethyl protected binaphthol. 30 mL of dry DCM was added in the flask to dissolve the protected binaphthol. The solution was stirred at 0 °C in ice bath for 15 min before the dropwise addition of a DCM solution of BBr_3 (3.0 equiv). After that, the ice bath was removed, and the reaction was gradually warmed up and stirred for 12 hours. Then the solution was cooled at 0 °C to be quenched with slow addition of water (10 mL). The solution was transferred to separatory funnel to be extracted with DCM (50 mL X 3). All combined organic layer was dried over anhydrous sodium sulfate, concentrated and purified via column chromatography to afford the product.

General Procedure D

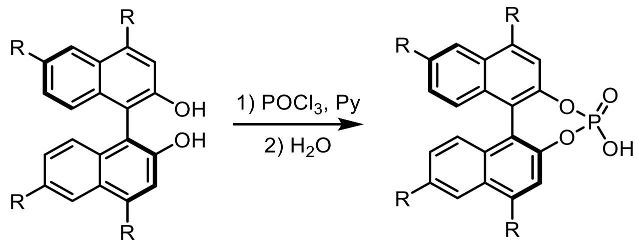

To a 100 mL oven-dried, two-necked, round-bottom flask, containing a magnetic stir bar with an attached vertical condenser was added the corresponding diol. 3 mL anhydrous pyridine was added to the flask via syringe and the solution was stirred for 5 min at room temperature. Then distilled phosphoryl trichloride (1.2 equiv) was added to the stirring solution, and the reaction was stirred at 90 °C for 2.5 hours (until the full conversion of diol by TLC analysis). After that, the flask was removed from heating for about 10 mins before the addition of 1mL of deionized water and the reaction was heated to 90 °C again for 2 hours. Later, the flask was removed from heating for about 10 mins before the slow addition of 25 mL of deionized water, and the reaction was heated to 100 °C for 2 h. Finally, the suspension was cooled to room temperature then transferred to a separatory funnel to be extracted with CH_2Cl_2 (50 mL X 3). Combined organic layer was dried over anhydrous sodium sulfate, evaporated and purified by column chromatography to afford the corresponding phosphoric acid.

General Procedure E

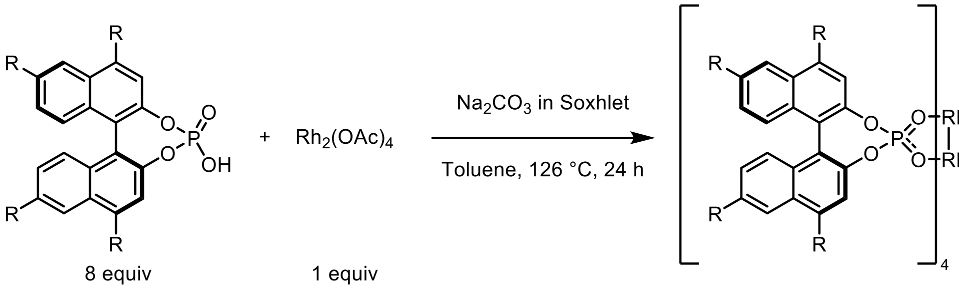

To a 50 mL round-bottom flask with a magnetic stir bar was added the corresponding phosphoric acid (8.0 equiv) and dirhodium tetraacetate (1.0 equiv). A Soxhlet containing sand and anhydrous sodium carbonate was placed between the flask and a vertical condenser. Then 15 mL of dry toluene was added before the solution was vigorously stirred at 126 °C to ensure that efficiently condensed liquid could soak through the solid base inside of the Soxhlet. After 24 h, the flask was

removed from heating and cooled to room temperature before being concentrated and directly purified by column chromatography (if necessary, excess ligand can be recovered by column chromatography).

General Procedure F

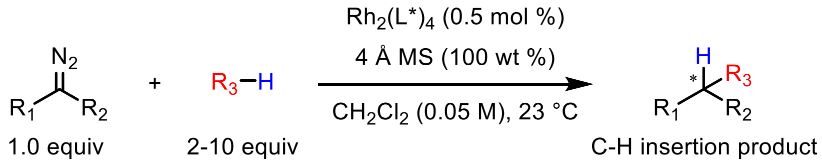

To a 4 mL vial with a magnetic stir bar was added 4 Å Molecular Sieves. After being flame-dried under vacuum, vacuum-refilled with N_2 for 3 times and cooled to room temperature, the corresponding catalyst (0.5 mol %) was added in the vial followed by the addition of **alkyl substrate (2-10 equiv).** 1 mL of dry dichloromethane was added to the vial and the solution was stirred at 23 °C, followed by slow addition of a solution of corresponding diazo compound **(0.1 mmol, 1 equiv)** in 1 mL dichloromethane via syringe pump over 1 h (for secondary C–H insertion) or 3 h (for tertiary C–H insertion). After full consumption of the diazo compound (monitored by TLC analysis), the reaction was filtered through a short pipette of silica gel and was concentrated for crude NMR analysis. Then, the crude sample was purified via column chromatography (Both UV detection and CAM stain were used for identification of C–H insertion products).

General Procedure G

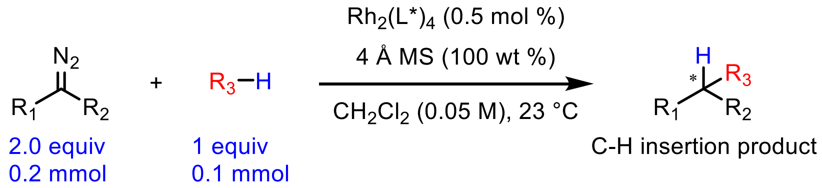

To a 4 mL vial with a magnetic stir bar was added 4 Å Molecular Sieves. After being flame-dried under vacuum, vacuum-refilled with N_2 for 3 times and cooled to room temperature, the corresponding catalyst (0.5 mol %) was added in the vial followed by the addition of alkyl substrate (1 equiv, 0.1 mmol). 1 mL of dry dichloromethane was added to the vial and the solution was stirred at 23 °C, followed by slow addition of a solution of corresponding diazo compound (0.2 mmol, 2 equiv) in 1 mL dichloromethane via syringe pump over 1 h (for secondary C–H insertion) or 3 h (for tertiary C–H insertion). After full consumption of the diazo compound (monitored by

TLC analysis), the reaction was filtered through a short pipette of silica gel and was concentrated for crude NMR analysis. Then, the crude sample was purified via column chromatography (Both UV detection and CAM stain were used for identification of C–H insertion products).

3. Procedure of the synthesis of intermediates and catalysts and their characterization

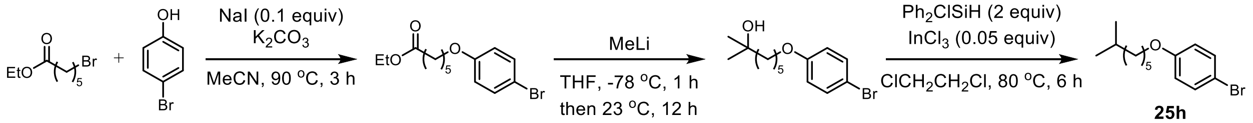

25h

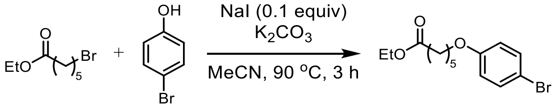

Ethyl 6-bromohexanoate (2.6 g, 11.7 mmol, 1 equiv), 4-bromophenol (2.0 g, 11.8 mmol, 1.01 equiv), sodium iodide (174 mg, 1.2 mmol, 0.1 equiv) and potassium carbonate (1.6 g, 11.7 mmol, 1 equiv) were added in the 50 mL round-bottom flask with a magnetic stir bar. Then 20 mL of dry acetonitrile was added to the flask and the mixture was stirred at 55 °C (in a heating mantle) for 3 h before it was filtrated and concentrated as the crude material, which was purified by silica gel chromatography to afford a colorless liquid in 75% yield (2.75 g).

^{1}H NMR (400 MHz, $CDCl_3$) δ 7.35 (d, J = 9.1 Hz, 2H), 6.76 (d, J = 9.1 Hz, 2H), 4.13 (q, J = 7.2 Hz, 2H), 3.91 (t, J = 6.4 Hz, 2H), 2.33 (t, J = 7.5 Hz, 2H), 1.83 – 1.74 (m, 2H), 1.74 – 1.64 (m, 2H), 1.25 (t, J = 7.2 Hz, 3H); **^{13}C NMR** (101 MHz, $CDCl_3$) δ 173.7, 158.2, 132.3, 116.4, 112.7, 68.0, 60.4, 34.3, 29.0, 25.7, 24.8, 14.4; **IR** (neat) 2979, 2940, 2868, 1731, 1590, 1577, 1488, 1472, 1240, 1170, 1101, 1070, 1031, 1001, 977, 822 cm^{-1}; **HRMS** (FTMS +p APCI) calcd for $C_{14}H_{20}O_3{}^{79}Br$ $[M+H]^+$ 315.0590 found 315.0590.

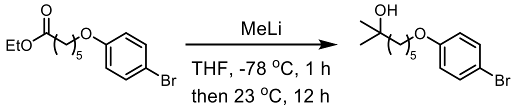

Ethyl 6-(4-bromophenoxy)hexanoate (2.75 g, 8.7 mmol, 1 equiv) was added in a 100 mL flame-dried round-bottom flask with a magnetic stir bar followed by the addition of 30 mL dry THF. Then the stirring mixture was cooled to -78 °C followed by an addition of MeLi solution (1.6M, 12.5 mL, 20 mmol, 2.3 equiv) over 20 min and kept stirring for 12 h while gradually warmed up to 23 °C. The reaction was quenched with sat. NH_4Cl solution at 0 °C and was extracted with ethyl acetate, washed with brine, dried over anhydrous sodium sulfate and concentrated as the crude

material, which was purified by silica gel chromatography to afford a colorless liquid in 46% yield (1.20 g).

^{1}H NMR (400 MHz, $CDCl_3$) δ 7.35 (d, J = 9.0 Hz, 2H), 6.76 (d, J = 9.0 Hz, 2H), 3.91 (t, J = 6.5 Hz, 2H), 1.78 (dq, J = 8.3, 6.4 Hz, 2H), 1.52 – 1.38 (m, 6H), 1.32 (br s, 1H), 1.21 (s, 6H); **^{13}C NMR** (101 MHz, $CDCl_3$) δ 158.3, 132.3, 116.4, 112.7, 71.1, 68.2, 43.9, 29.4, 29.3, 26.7, 24.2; **IR** (neat) 3367, 2966, 2936, 2862, 1590, 1578, 1488, 1470, 1377, 1285, 1242, 1170, 1071, 1001, 907, 820, 640 cm^{-1}; **HRMS** (FTMS +p APCI) calcd for $C_{14}H_{21}O_2{}^{79}Br$ $[M]^+$ 300.0719 found 300.0719.

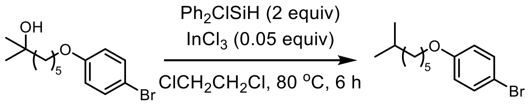

25g

To a 16 mL vial was added 7-(4-bromophenoxy)-2-methylheptan-2-ol (600 mg, 2.0 mmol, 1.0 equiv) with a magnetic stir bar followed by the addition of 4 mL dry 1,2-DCE followed by an addition of Ph_2ClSiH (0.87 mL, 4.2 mmol, 2.1 equiv) dropwise. Then the stirring mixture was heated to 80 °C (in a heating mantle) for 6 h. After that, the mixture was cooled to 23 °C and was quenched with 2 mL deionized water, extracted with ethyl acetate, washed with brine, dried over anhydrous sodium sulfate and concentrated as the crude material, which was purified by silica gel chromatography to afford 1-bromo-4-((6-methylheptyl)oxy)benzene (**25g**) as a colorless liquid in 70% yield (400 mg).

^{1}H NMR (400 MHz, $CDCl_3$) δ 7.36 (d, J = 9.0 Hz, 2H), 6.77 (d, J = 9.0 Hz, 2H), 3.91 (t, J = 6.6 Hz, 2H), 1.77 (dq, J = 7.9, 6.5 Hz, 2H), 1.59 – 1.48 (m, 1H), 1.46 – 1.38 (m, 2H), 1.38 – 1.29 (m, 2H), 1.23 – 1.15 (m, 2H), 0.87 (d, J = 6.6 Hz, 6H); **^{13}C NMR** (101 MHz, $CDCl_3$) δ 158.4, 132.3, 116.4, 112.7, 68.4, 39.0, 29.3, 28.1, 27.3, 26.4, 22.8; **IR** (neat) 2951, 2929, 2867, 1590, 1578, 1488, 1468, 1384, 1366, 1285, 1242, 1170, 1072, 1002, 843, 820, 641 cm^{-1}; **HRMS** (FTMS +p ESI) calcd for $C_{14}H_{21}O^{79}Br$ $[M]^+$ 284.0770 found 284.0769.

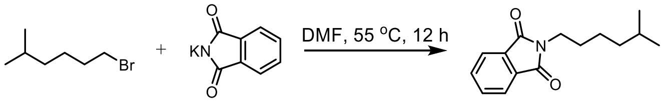

25h

2-(5-methylhexyl)isoindoline-1,3-dione (**25h**) was prepared according to a modified literature procedure.[1] Potassium 1,3-dioxoisoindolin-2-ide (950 mg, 5.1 mmol, 1 equiv) and 1-bromo-5-methylhexane (1.0 g, 5.6 mmol, 1.1 equiv) were added to a 50 mL round-bottom flask with a

magnetic stir bar. After that, DMF (20 mL) was added in the flask and the reaction was stirred at 55 °C (in a heating mantle) for 12 h before it was cooled down to room temperature and poured into 50 mL ice water. The mixture was then extracted with dichloromethane (50 mL X 3), washed with brine, dried over anhydrous sodium sulfate, concentrated and purified by silica gel chromatography to afford a colorless oil (898 mg, 71% yield).

^{1}H NMR (400 MHz, $CDCl_3$) δ 7.84 (dd, J = 5.5, 3.0 Hz, 2H), 7.70 (dd, J = 5.4, 3.1 Hz, 2H), 3.67 (t, J = 7.3 Hz, 2H), 1.70 – 1.60 (m, 2H), 1.51 (dq, J = 13.3, 6.7 Hz, 1H), 1.39 – 1.29 (m, 2H), 1.25 – 1.16 (m, 2H), 0.86 (d, J = 6.7 Hz, 6H); **^{13}C NMR** (101 MHz, $CDCl_3$) δ 168.6, 134.0, 132.3, 123.3, 38.6, 38.2, 29.0, 28.0, 24.8, 22.7; **IR** (neat) 2956, 2931, 2867, 1771, 1695, 1614, 1594, 1463, 1432, 1396, 1364, 1338, 1288, 1225, 1189, 1133, 1055, 1004, 970, 898, 854, 792, 718, 709, 625, 530 cm^{-1}; **HRMS** (FTMS +p ESI) calcd for $C_{15}H_{20}O_2N$ $[M+H]^+$ 246.1489 found 246.1488 (Δ = -0.09).

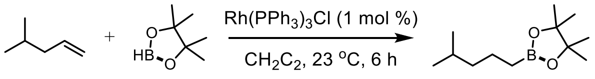

25i

To a 4 mL vial with septa cap was added $Rh(PPh_3)_3Cl$ (27.8 mg, 1 mol %). After the vial was vacuum-refilled with nitrogen for three times, 4-methyl-1-pentene (252 mg, 1.0 equiv) was added in the vial with 1 mL degassed CH_2Cl_2. Then 4,4,5,5-tetramethyl-1,3,2-dioxaborolane (HBpin, 422 mg, 1.1 equiv) was added to the stirring mixture at 23 °C. After 6 h, 1 drop of water was added to the mixture to quench the reaction and the solvent was removed via rotary evaporation to afford the crude product, which was then purified via silica gel chromatography. The purified product, 4,4,5,5-tetramethyl-2-(4-methylpentyl)-1,3,2-dioxaborolane (**25i**), was obtained as a colorless liquid in 26% yield (m = 164 mg, volatile).

^{1}H NMR (400 MHz, $CDCl_3$) δ 1.53 (sept, J = 6.7 Hz, 1H), 1.44 – 1.35 (m, 2H), 1.24 (s, 12H), 1.20 – 1.13 (m, 2H), 0.85 (d, J = 6.7 Hz, 6H), 0.74 (t, J = 7.9 Hz, 3H); **^{13}C NMR** (151 MHz, $CDCl_3$) δ 83.0, 42.1, 27.9, 25.0, 22.8, 21.9; **IR** (neat) 2978, 2954, 2928, 2869, 1468, 1408, 1371, 1313, 1268, 1238, 1189, 969, 849 cm^{-1}; **HRMS** (FTMS +p APCI) calcd for $C_{12}H_{26}O_2{}^{10}B$ $[M]^+$ 212.2056 found 212.2060.

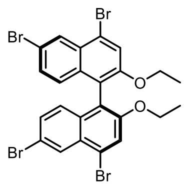

S-**8**

(*S*)-4,4',6,6'-tetrabromo-2,2'-diethoxy-1,1'-binaphthalene **(*S*-8)** was prepared from the reported procedure (12 g, 52% yield). Spectroscopic data match the reported literature.[2]

^{1}H NMR (600 MHz, $CDCl_3$) δ 8.40 (d, J = 2.0 Hz, 2H), 7.72 (s, 2H), 7.30 (dd, J = 9.0, 1.9 Hz, 2H), 6.93 (d, J = 9.0 Hz, 2H), 4.08 – 4.00 (m, 4H), 1.08 (t, J = 7.0 Hz, 6H).

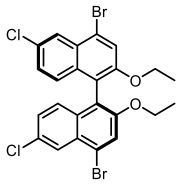

S-**16**

(*S*)-4,4'-dibromo-6,6'-dichloro-2,2'-diethoxy-1,1'-binaphthalene **(*S*-16)** was prepared from the reported procedure. Spectroscopic data matches with these in the reported literature.[3, 4]

^{1}H NMR (400 MHz, $CDCl_3$) δ 8.23 (dd, J = 2.1, 0.6 Hz, 2H), 7.73 (s, 2H), 7.18 (dd, J = 9.0, 2.1 Hz, 2H), 7.01 (dd, J = 9.0, 0.6 Hz, 2H), 4.04 (q, J = 7.0 Hz, 4H), 1.08 (t, J = 7.0 Hz, 6H).

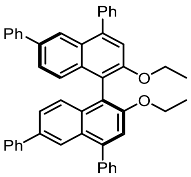

S-**9a**

Using ***general procedure A*** with (*S*)-2,2'-diethoxy-4,4',6,6'-tetrabromo-1,1'-binaphthalene (1.50 g, 2.28 mmol, 1.0 equiv) and phenylboronic acid (2.22 g, 18.24 mmol, 8.0 equiv), (S)-2,2'-diethoxy-4,4',6,6'-tetraphenyl-1,1'-binaphthalene **(*S*-9a)** was obtained as a white solid (1.20 g, 74 % yield) after purification by column chromatography.

$[\alpha]^{20}_D$: +11.0°(c = 1.18, $CHCl_3$); **^{1}H NMR** (400 MHz, $CDCl_3$) δ 8.16 (d, J = 1.8 Hz, 2H), 7.76 – 7.69 (m, 4H), 7.62 – 7.48 (m, 12H), 7.46 – 7.37 (m, 8H), 7.33 – 7.27 (m, 2H), 4.16 (q, J = 7.0 Hz, 4H), 1.17 (t, J = 7.0 Hz, 6H); **^{13}C NMR** (101 MHz, $CDCl_3$) δ 154.1, 142.0, 141.6, 141.1, 136.3, 134.1, 130.4, 128.9, 128.6, 127.9, 127.6, 127.4, 127.0, 126.7, 125.9, 124.3, 120.0, 117.5, 65.4, 15.3; **IR** (neat) 3055, 2978, 2929, 1951, 1789, 1586, 1571, 1486, 1372, 1329, 1264, 1212, 1056,

950, 831, 758, 697 cm^{-1}; **HRMS** (FTMS +p APCI) calcd for $C_{48}H_{39}O_2$ $[M+H]^+$ 647.2945 found 647.2940 (Δ = -0.64).

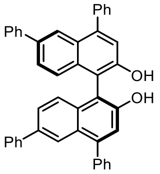

S-**10a**

Using ***general procedure C*** with (S)-2,2'-diethoxy-4,4',6,6'-tetraphenyl-1,1'-binaphthalene (1.20 g), (S)-4,4',6,6'-tetraphenyl-[1,1'-binaphthalene]-2,2'-diol **(*S*-10a)** was obtained as a light-yellow foam solid (1.03 g, yield: 94 %). Spectroscopic data match the reported literature.[5]

^{1}H NMR (400 MHz, $CDCl_3$) δ 8.20 (d, J = 1.6 Hz, 2H), 7.71 – 7.67 (m, 4H), 7.65 (dd, J = 8.7, 1.9 Hz, 2H), 7.62 – 7.55 (m, 8H), 7.55 – 7.50 (m, 2H), 7.47 (dd, J = 8.7, 0.6 Hz, 2H), 7.45 – 7.39 (m, 6H), 7.36 – 7.30 (m, 2H), 5.25 (s, 2H)**.**

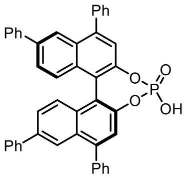

S-**11a**

Using ***general procedure D*** with (S)-4,4',6,6'-tetraphenyl-[1,1'-binaphthalene]-2,2'-diol (1.00 g, 1.69 mmol), (11bS)-4-hydroxy-1,7,9,14-tetraphenyldinaphtho[2,1-d:1',2'-f][1,3,2]dioxaphosphepine 4-oxide **(*S*-11a)** was obtained as a light-yellow foam solid (973 mg, 88 % yield). Spectroscopic data match the reported literature.[5]

^{1}H NMR (400 MHz, DMSO) δ 8.15 (d, J = 1.9 Hz, 1H), 7.82 – 7.68 (m, 3H), 7.67 – 7.52 (m, 6H), 7.45 (t, J = 7.6 Hz, 2H), 7.42 – 7.32 (m, 2H); **^{13}C NMR** (101 MHz, DMSO) δ 149.9 (d, J =), 141.8, 139.9, 139.3, 136.4, 131.9, 129.9, 129.1, 128.8, 128.6, 128.0, 127.6, 127.5, 126.7, 125.5, 123.9, 123.4, 121.2; **^{31}P NMR** (162 MHz, DMSO) δ 4.57.

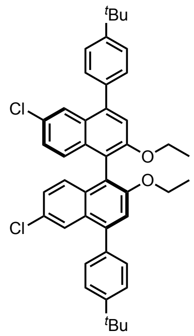

Using ***general procedure B*** with (S)-4,4'-dibromo-6,6'-dichloro-2,2'-diethoxy-1,1'-binaphthalene (1.50 g, 2.28 mmol, 1.0 equiv) and (4-(tert-butyl)phenyl)boronic acid (200 mg, 351 μmol, 4.0 equiv), (S)-4,4'-bis(4-(tert-butyl)phenyl)-6,6'-dichloro-2,2'-diethoxy-1,1'-binaphthalene was obtained as a white solid (230 mg, 99 % yield) after purification by column chromatography.

[α]$^{20}_{D}$: -53.8°(c = 0.44, $CHCl_3$); **^{1}H NMR** (500 MHz, $CDCl_3$) δ 7.92 (dd, J = 1.7, 0.9 Hz, 2H), 7.60 – 7.57 (m, 4H), 7.56 – 7.53 (m, 4H), 7.39 (s, 2H), 7.21 – 7.16 (m, 4H), 4.11 – 4.07 (m, 4H), 1.45 (s, 18H), 1.11 (t, J = 7.0 Hz, 6H); **^{13}C NMR** (101 MHz, $CDCl_3$) δ 154.0, 150.8, 141.1, 137.3, 133.0, 129.9, 129.6, 128.4, 127.6, 127.0, 125.6, 125.2, 119.4, 117.7, 65.2, 34.9, 31.6, 15.1; **IR** (neat) 2962, 2903, 2868, 1912, 1585, 1393, 1347, 1108, 840 cm^{-1}; **HRMS** (FTMS +p ESI) calcd for $C_{44}H_{44}O_2{}^{35}Cl_2$ [M]$^+$ 674.2718 found 674.2703 (Δ = -1.42).

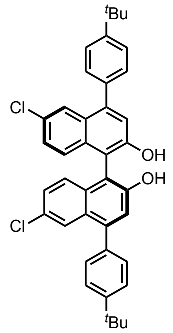

Using ***general procedure C*** with (S)-4,4'-bis(4-(tert-butyl)phenyl)-6,6'-dichloro-2,2'-diethoxy-1,1'-binaphthalene, (S)-4,4'-bis(4-(tert-butyl)phenyl)-6,6'-dichloro-[1,1'-binaphthalene]-2,2'-diolwas obtained as a light-yellow foam solid (161 mg, yield: 73 %).

[α]$^{20}_{D}$: +3.3°(c = 0.16, $CHCl_3$); **^{1}H NMR** (400 MHz, $CDCl_3$) δ 7.98 (d, J = 2.1 Hz, 2H), 7.62 – 7.57 (m, 4H), 7.54 – 7.49 (m, 4H), 7.39 (s, 2H), 7.29 (dd, J = 9.0, 2.1 Hz, 2H), 7.21 (d, J = 9.0 Hz, 2H), 5.10 (s, 2H), 1.45 (s, 18H); **^{13}C NMR** (101 MHz, $CDCl_3$) δ 152.5, 151.3, 143.5, 136.2, 132.4, 130.4, 129.6, 129.0, 128.4, 126.3, 126.0, 125.8, 119.8, 110.1, 34.9, 31.6; **IR** (neat) 3520, 2962,

1589, 1498, 1380, 1144, 947, 838 cm^{-1}; **HRMS** (FTMS +p ESI) calcd for $C_{40}H_{36}O_2{}^{35}Cl_2$ $[M]^+$ 618.2087 found 618.2080 (Δ = -1.07).

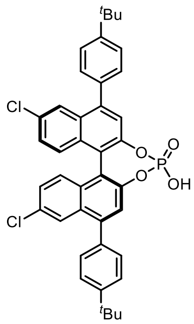

S-**17a**

Using ***general procedure D*** with (S)-4,4'-bis(4-(tert-butyl)phenyl)-6,6'-dichloro-[1,1'-binaphthalene]-2,2'-diol (393 mg, 634 μmol), (11bS)-1,7-bis(4-(tert-butyl)phenyl)-9,14-dichloro-4-hydroxydinaphtho[2,1-d:1',2'-f][1,3,2]dioxaphosphepine 4-oxide **(*S*-17a)** was obtained as a light-yellow foam solid (401 mg, 93 % yield).

$[\alpha]^{20}_{D}$: +40.2°(c = 0.10, $CHCl_3$); **^{1}H NMR** (500 MHz, $CDCl_3$) δ 8.90 (brs, 1H, P(O)OH), 8.06 (d, J = 2.2 Hz, 2H), 7.57 – 7.54 (m, 2H), 7.50 (d, J = 8.4 Hz, 4H), 7.48 – 7.43 (m, 6H), 7.30 (dd, J = 9.1, 2.2 Hz, 2H), 1.39 (s, 18H); **^{13}C NMR** (101 MHz, $CDCl_3$) δ 151.5, 146.6(d, $^2J_{C\text{-}P}$ = 9.1 Hz), 143.6, 135.5, 132.3, 131.4(d, $^4J_{C\text{-}P}$ = 1.3 Hz), 131.2(d, $^4J_{C\text{-}P}$ = 0.7 Hz), 129.7, 129.1, 127.8, 126.0, 125.8, 122.5(d, $^3J_{C\text{-}P}$ = 3.0 Hz), 120.2(d, $^3J_{C\text{-}P}$ = 2.2 Hz), 34.9, 31.5; **^{31}P NMR** (162 MHz, $CDCl_3$) δ 4.7; **IR** (neat) 2961, 1585, 1494, 1361, 1176, 1019, 816, 714 cm^{-1}; **HRMS** (FTMS +p ESI) calcd for $C_{40}H_{36}O_4{}^{35}Cl_2P$ $[M+H]^+$ 681.1723 found 681.1721 (Δ = 0.21).

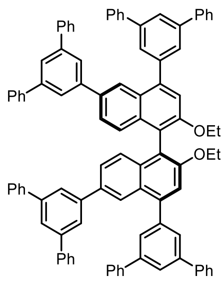

S-**9c**

Using ***general procedure A*** with (S)-4,4',6,6'-tetrabromo-2,2'-diethoxy-1,1'-binaphthalene (700 mg, 1.06 mmol, 1.0 equiv) and [1,1':3',1''-terphenyl]-5'-ylboronic acid (2.33 g, 8.51 mmol, 8.0 equiv), (S)-4,4',6,6'-tetra([1,1':3',1''-terphenyl]-5'-yl)-2,2'-diethoxy-1,1'-binaphthalene **(*S*-9c)** was obtained as a white solid. (Note that normal phase column chromatography is not able to fully

purify the product. Hence, the product was directly used for the following deprotection and was then further purified.)

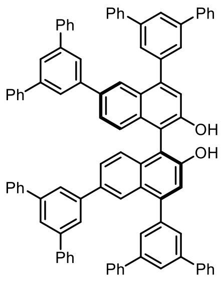

S-**10c**

Using ***general procedure C*** with (S)-4,4',6,6'-tetra([1,1':3',1''-terphenyl]-5'-yl)-2,2'-diethoxy-1,1'-binaphthalene (900 mg, 717 μmol), (S)-4,4',6,6'-tetra([1,1':3',1''-terphenyl]-5'-yl)-[1,1'-binaphthalene]-2,2'-diol **(*S*-10c)** was obtained as a white foam solid (853 mg, 74 % yield, two steps) after purification by column chromatography (0-20% ethyl acetate/n-hexane for 20 column volume, then 20% ethyl acetate/n-hexane for 20 column volume).

[α]^{20}$_D$: +52.1°(c = 0.31, $CHCl_3$); **^{1}H NMR** (600 MHz, $CDCl_3$) δ 8.48 (d, J = 1.7 Hz, 2H), 7.97 (s, 6H), 7.81 – 7.77 (m, 14H), 7.75 (t, J = 1.7 Hz, 2H), 7.63 – 7.60 (m, 10H), 7.58 (d, J = 8.7 Hz, 2H), 7.50 – 7.46 (m, 8H), 7.44 – 7.40 (m, 12H), 7.39 – 7.35 (m, 4H), 5.33 (s, 2H); **^{13}C NMR** (101 MHz, CDCl3) δ 152.7, 144.3, 142.5, 142.4, 142.3, 141.1, 140.92, 140.89, 137.5, 133.6, 129.1, 129.0, 128.4, 127.9, 127.8, 127.7, 127.6, 127.5, 127.4, 125.9, 125.6, 125.5, 125.4, 125.3, 119.6, 110.7; **IR** (neat) 3308, 3033, 1591, 1575, 1496, 874, 754, 692 cm^{-1}; **HRMS** (FTMS +p ESI) calcd for $C_{92}H_{62}O_2$ $[M]^+$ 1198.4744 found 1198.4715 (Δ = -2.42).

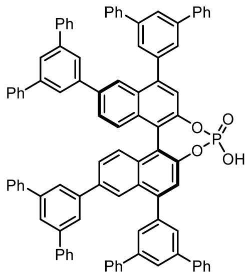

S-**11c**

Using ***general procedure D*** with (S)-4,4',6,6'-tetra([1,1':3',1''-terphenyl]-5'-yl)-[1,1'-binaphthalene]-2,2'-diol (950 mg, 792 μmol), (11bS)-1,7,9,14-tetra([1,1':3',1''-terphenyl]-5'-yl)-4-hydroxydinaphtho[2,1-d:1',2'-f][1,3,2]dioxaphosphepine 4-oxide **(*S*-11c)** was obtained as a white foam solid (340 mg, 34 % yield) after purification by column chromatography.

[α]$^{20}_{D}$: +65.2°(c = 0.51, $CHCl_3$); **^{1}H NMR** (400 MHz, $CDCl_3$) δ 8.52 (s, 2H), 7.91 (s, 6H), 7.82 – 7.76 (m, 12H), 7.73 (d, J = 7.3 Hz, 8H), 7.61 (dd, J = 8.1, 1.3 Hz, 8H), 7.45 – 7.32 (m, 24H), 5.68 (s, 3H, P(O)OH H–bonded with H_2O); **^{13}C NMR** (101 MHz, $CDCl_3$) δ 146.7(d, J = 9.4 Hz), 144.1, 142.6, 142.3, 142.0, 141.1, 140.8, 140.2, 138.9, 132.4, 130.8, 129.0, 128.9, 128.6, 127.9, 127.74, 127.66, 127.5, 127.4, 126.8, 126.1, 125.7, 125.5, 125.0, 122.1(d, J = 2.5 Hz), 120.9(d, J = 2.2 Hz); **^{31}P NMR** (162 MHz, $CDCl_3$) δ 5.41; **IR** (neat) 3033, 1593, 1575, 1496, 1180, 1027, 876, 755, 695 cm^{-1}; **HRMS** (FTMS -p ESI) calcd for $C_{92}H_{60}O_4P$ [M-H]$^-$ 1259.4235 found 1259.4256 (Δ = 1.69).

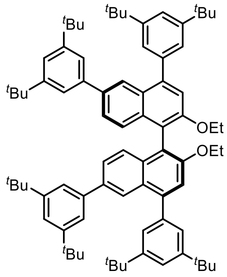

S-**9d**

Using ***general procedure A*** with (S)-4,4',6,6'-tetrabromo-2,2'-diethoxy-1,1'-binaphthalene (2.00 g, 3.04 mmol), (S)-4,4',6,6'-tetrakis(3,5-di-tert-butylphenyl)-2,2'-diethoxy-1,1'-binaphthalene **(*S*-9d)** was obtained as a pale-yellow foam solid (1.79 g, 54 % yield) after purification by column chromatography.

[α]$^{20}_{D}$: +10.5°(c = 0.18, $CHCl_3$); **^{1}H NMR** (400 MHz, $CDCl_3$) δ 8.09 (d, J = 1.7 Hz, 2H), 7.57 – 7.52 (m, 6H), 7.50 (dd, J = 8.8, 1.7 Hz, 2H), 7.46 (s, 2H), 7.43 (d, J = 8.8 Hz, 2H), 7.37 (s, 6H), 4.18 (q, J = 7.0 Hz, 4H), 1.45 (s, 36H), 1.32 (s, 36H), 1.21 (t, J = 7.0 Hz, 6H); **^{13}C NMR** (101 MHz, $CDCl_3$) δ 153.9, 151.0, 150.7, 143.1, 141.6, 140.4, 137.8, 133.9, 128.2, 126.7, 126.6, 124.6(two signals), 122.2, 121.5, 121.1, 119.9, 117.5, 65.4, 35.2, 35.1, 31.9, 31.7, 15.4; **IR** (neat) 3064, 2962, 2903, 2868, 1593, 1362, 1247, 875, 714 cm^{-1}; **HRMS** (FTMS +p ESI) calcd for $C_{80}H_{102}O_2$ [M]$^+$ 1094.7874 found 1094.7833 (Δ= -3.75).

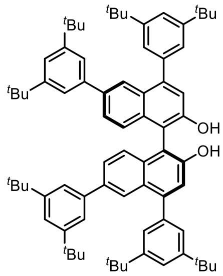

S-**10d**

Using ***general procedure C*** with (S)-4,4',6,6'-tetrakis(3,5-di-tert-butylphenyl)-2,2'-diethoxy-1,1'-binaphthalene (1.79 g, 1.63 mmol), (S)-4,4',6,6'-tetrakis(3,5-di-tert-butylphenyl)-[1,1'-binaphthalene]-2,2'-diol **(*S*-10d)** was obtained as a white foam solid (1.69 g, 99 % yield) after purification by column chromatography.

[α]^{20}$_{D}$: +55.4°(c = 0.48, $CHCl_3$); **^{1}H NMR** (400 MHz, $CDCl_3$) δ 8.18 (d, J = 1.7 Hz, 2H), 7.62 (dd, J = 8.7, 1.7 Hz, 2H), 7.55 (dd, J = 4.1, 2.3 Hz, 6H), 7.51 (d, J = 8.7 Hz, 2H), 7.47 (s, 2H), 7.42 (t, J = 1.7 Hz, 2H), 7.38 (d, J = 1.7 Hz, 4H), 5.25 (s, 2H), 1.45 (s, 36H), 1.35 (s, 36H); **^{13}C NMR** (101 MHz, CDCl3) δ 152.4, 151.2, 150.9, 145.6, 141.2, 139.3, 138.7, 133.2, 128.7, 128.1, 125.4, 125.3, 124.3, 122.3, 121.9, 121.5, 119.2, 110.3, 35.2, 35.1, 31.8, 31.7; **IR** (neat) 3536, 3063, 2962, 2904, 2867, 1592, 1393, 1264, 874, 739 cm^{-1}, **HRMS** (FTMS +p ESI) calcd for $C_{76}H_{94}O_2$ [M]$^+$ 1038.7248 found 1038.7231 (Δ= -1.7).

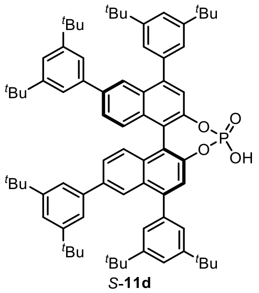

S-**11d**

Using ***general procedure D*** with (S)-4,4',6,6'-tetrakis(3,5-di-tert-butylphenyl)-[1,1'-binaphthalene]-2,2'-diol (1.74 g, 1.67 mmol), (11bS)-1,7,9,14-tetrakis(3,5-di-tert-butylphenyl)-4-hydroxydinaphtho[2,1-d:1',2'-f][1,3,2]dioxaphosphepine 4-oxide **(*S*-11d)** was obtained as a light-yellow foam solid (1.73 g, 94 %) after purification by column chromatography.

[α]^{20}$_{D}$: +98.9°(c = 0.26, $CHCl_3$); **^{1}H NMR** (400 MHz, $CDCl_3$) δ 8.22 (d, J = 1.5 Hz, 2H), 7.73 (d, J = 8.8 Hz, 2H), 7.65 – 7.57 (m, 4H), 7.49 (d, J = 7.4 Hz, 6H), 7.41 (dd, J = 12.4, 1.5 Hz, 6H), 6.11 (s, 3H, P(O)OH hydrogen bonded with one H_2O), 1.40 (s, 36H), 1.34 (s, 36H); **^{13}C NMR** (101 MHz, $CDCl_3$) δ 151.3, 150.9, 146.5(d, $^{2}J_{C\text{-}P}$ = 8.4 Hz), 145.3, 140.8, 139.9, 138.7, 132.1, 131.0,

128.3, 127.1, 125.0, 124.3, 122.2, 122.0, 121.8, 121.7, 120.5, 35.11, 35.09, 31.8, 31.6; **^{31}P NMR** (162 MHz, $CDCl_3$) δ 5.61; **IR** (neat) 2961, 2904, 2867, 1593, 1362, 1264, 1247, 1022, 876, 739 cm^{-1}, **HRMS** (FTMS -p ESI) calcd for $C_{76}H_{92}O_4P$ $[M-H]^-$ 1099.6739 found 1099.6730 (Δ= -0.75).

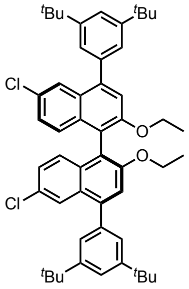

Using ***general procedure B*** with (S)-4,4'-dibromo-6,6'-dichloro-2,2'-diethoxy-1,1'-binaphthalene (1.00 g, 1.76 mmol), (S)-6,6'-dichloro-4,4'-bis(3,5-di-tert-butylphenyl)-2,2'-diethoxy-1,1'-binaphthalene was obtained as a white solid (1.45 g, 99% yield).

$[\alpha]^{20}_D$: -44.5°(c = 2.22, $CHCl_3$); **^{1}H NMR** (400 MHz, $CDCl_3$) δ 7.94 (d, J = 2.1 Hz, 2H), 7.59 (t, J = 1.8 Hz, 2H), 7.50 – 7.45 (m, 6H), 7.23 (d, J = 9.0 Hz, 2H), 7.19 (dd, J = 9.0, 2.1 Hz, 2H), 4.15 (q, J = 7.0 Hz, 4H), 1.47 (s, 36H), 1.17 (t, J = 7.0 Hz, 6H); **^{13}C NMR** (101 MHz, $CDCl_3$) δ 154.0, 151.0, 142.2, 139.4, 133.0, 129.6, 128.5, 127.6, 126.9, 125.4, 124.6, 121.7, 119.4, 117.6, 65.3, 35.2, 31.7, 15.2; **IR** (neat) 3067, 2962, 2903, 2867, 1580, 1490, 1477, 1373, 1362, 1247, 1206, 1089, 879, 738 cm^{-1}; **HRMS** (FTMS +p APCI) calcd for $C_{52}H_{61}O_2{}^{35}Cl_2$ $[M+H]^+$ 787.4043 found 787.4054 (Δ= 1.38).

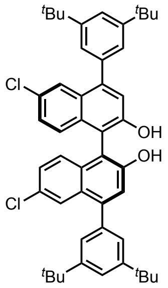

Using ***general procedure C*** with (S)-6,6'-dichloro-4,4'-bis(3,5-di-tert-butylphenyl)-2,2'-diethoxy-1,1'-binaphthalene (1.40 g, 1.78 mmol), (S)-6,6'-dichloro-4,4'-bis(3,5-di-tert-butylphenyl)-[1,1'-binaphthalene]-2,2'-diol was obtained as a white foam solid (1.05 g, 81% yield) after further purification by column chromatography.

[α]$^{20}_{D}$: +4.0°(c = 0.34, $CHCl_3$); **^{1}H NMR** (400 MHz, $CDCl_3$) δ 8.00 (d, J = 2.0 Hz, 2H), 7.58 (t, J = 1.7 Hz, 2H), 7.44 (d, J = 2.0 Hz, 6H), 7.29 (dd, J = 9.0, 2.1 Hz, 2H), 7.24 (d, J = 9.0 Hz, 2H), 5.16 (s, 2H), 1.45 (s, 36H); **^{13}C NMR** (101 MHz, $CDCl_3$) δ 152.5, 151.2, 144.6, 138.3, 132.5, 130.4, 129.1, 128.3, 126.4, 126.2, 124.4, 122.1, 119.7, 110.1, 35.2, 31.7; **IR** (neat) 3535, 3067, 2962, 2904, 2868, 1772, 1585, 1380, 1247, 1143, 907, 721 cm^{-1}; **HRMS** (FTMS -p ESI) calcd for $C_{48}H_{51}O_2{}^{35}Cl_2$ $[M-H]^-$ 729.3272 found 729.3295 (Δ= 3.22).

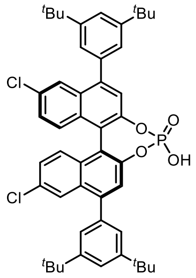

S-**17b**

Using ***general procedure D*** with (S)-6,6'-dichloro-4,4'-bis(3,5-di-tert-butylphenyl)-[1,1'-binaphthalene]-2,2'-diol (1.04 g, 1.41 mmol), (11bS)-9,14-dichloro-1,7-bis(3,5-di-tert-butylphenyl)-4-hydroxydinaphtho[2,1-d:1',2'-f][1,3,2]dioxaphosphepine 4-oxide **(*S*-17b)** was obtained as a light yellow foam solid (1.05 g, 93% yield) after purification by column chromatography.

[α]$^{20}_{D}$: +87.1°(c = 0.25, $CHCl_3$); **^{1}H NMR** (400 MHz, $CDCl_3$) δ 8.06 (d, J = 2.1 Hz, 2H), 7.61 (s, 2H), 7.54 (t, J = 1.6 Hz, 2H), 7.44 (d, J = 9.1 Hz, 2H), 7.38 (d, J = 1.7 Hz, 4H), 7.29 (dd, J = 9.1, 2.1 Hz, 2H), 1.38 (s, 36H); **^{13}C NMR** (101 MHz, $CDCl_3$) δ 151.2, 146.7(d, $^{2}J_{C-P}$ = 9.3 Hz), 144.6, 137.7, 132.2, 131.5, 131.3, 129.1, 127.7, 126.2, 124.5, 122.3, 120.2, 35.1, 31.7; **^{31}P NMR** (162 MHz, $CDCl_3$) δ 5.00; **IR** (neat) 2962, 2867, 2359, 1737, 1581, 1263, 1018, 917, 737, 717 cm^{-1}, **HRMS** (FTMS -p ESI) calcd for $C_{48}H_{50}O_4{}^{35}Cl_2P$ $[M-H]^-$ 791.2829 found 791.2845 (Δ= 1.97).

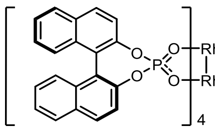

S-**2**

Using ***general procedure E*** with (*S*)-binaphthol phosphoric acid (756 mg, 2.17 mmol), ***S*-2** was obtained as a green solid (360 mg, 83 % yield) after purification by column chromatography. Characterization data matched reported data.[6]

^{1}H NMR (400 MHz, $CDCl_3$) δ 7.89 (dd, J = 7.9, 1.7 Hz, 1H), 7.85 (d, J = 8.8 Hz, 1H), 7.60 (d, J = 8.8 Hz, 1H), 7.46 (t, J = 7.9 Hz, 2H), 7.32 (td, J = 7.9, 1.1 Hz, 1H); **^{13}C NMR** (151 MHz, $CDCl_3$) δ 148.0, 147.9 (d, J = 4.7 Hz,), 132.5, 132.0, 131.3, 128.6, 127.4, 126.6, 125.6, 121.8, 121.4; **^{31}P NMR** (243 MHz, $CDCl_3$) δ 20.74;

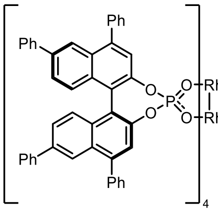

S-**6a**

Using ***general procedure E*** with (*S*)-4,4',6,6'-tetraphenylbinaphtholphosphoric acid (425 mg, 652 μmol), *S*-**6a** was obtained as a green solid (176 mg, 84 % yield) after purification by column chromatography. Characterization data matched main reported data.[5] (note that reported data indicates different conformations from **^{13}C NMR**)

^{1}H NMR indicates possible different conformations; **^{31}P NMR** (162 MHz, $CDCl_3$) δ 19.39 (major), 17.35 (minor). The structure was determined by X-ray crystallography, which showed that *S*-**6a** can exist in different conformations.

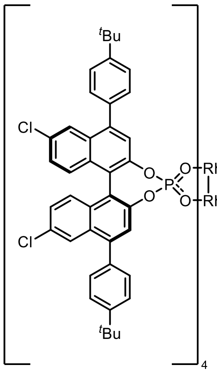

S-**18**

Using ***general procedure E*** with (*S*)-6,6'-dichloro-4,4'-bis((4-tertbutyl)phenyl)binaphtholphosphoric acid (345 mg, 507 μmol), *S*-**18** was obtained as a green solid (110 mg, 82 % yield) after purification by column chromatography.

^{1}H NMR (400 MHz, $CDCl_3$) δ 7.97 (d, J = 2.1 Hz, 2H), 7.46 (s, 2H), 7.37 (d, J = 9.1 Hz, 2H), 7.29 (s, 8H), 7.24 (dd, J = 9.1, 2.1 Hz, 2H), 1.28 (s, 18H); **^{13}C NMR** (101 MHz, $CDCl_3$) δ 151.5, 146.6 (d, $^{2}J_{C-P}$ = 9.1 Hz), 143.6, 135.5, 132.3, 131.4 (d, $^{4}J_{C-P}$ = 1.5 Hz), 131.2 (d, $^{4}J_{C-P}$ = 1 Hz), 129.7, 129.1, 127.8, 126.0, 125.8, 122.5 (d, $^{3}J_{C-P}$ = 3.3 Hz), 120.2 (d, $^{3}J_{C-P}$ = 2.5 Hz), 34.9, 31.5; **^{31}P NMR** (162 MHz, $CDCl_3$) δ 19.00; **IR** (neat) 2963, 1680, 1585, 1490, 1362, 1264, 962, 734 cm^{-1}; HRMS cannot be obtained by ESI or APCI. The structure was confirmed by X-ray crystallography.

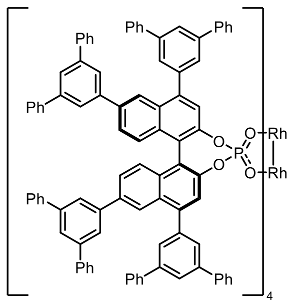

S-**6c**

Using ***general procedure E*** with (*S*)-4,4',6,6'-tetra[(1,1':3',1'')-terphenyl-5'-yl]binaphtholphosphoric acid (310 mg, 246 μmol), *S*-**6c** was obtained as a green solid (126 mg, 75 % yield) after purification by column chromatography.

^{1}H NMR (400 MHz, $CDCl_3$) δ 8.55 (d, J = 2.0 Hz, 2H), 8.21 (s, 2H), 7.77 – 7.59 (m, 22H), 7.50 – 7.34 (m, 20H), 7.24 – 6.99 (m, 10H), 6.95 – 6.57 (m, 4H); **^{13}C** NMR (201 MHz, $CDCl_3$) δ 147.7 (d, J = 9.6 Hz), 144.3, 142.6, 142.2, 142.0, 141.2, 140.7, 140.3, 138.7, 132.7, 130.5, 129.1, 129.0, 128.8, 127.6, 127.5, 127.4, 127.3, 127.2, 126.5, 126.1, 125.7, 125.5, 124.7, 122.9, 121.5; **^{31}P NMR** (162 MHz, $CDCl_3$) δ 24.20; **IR** (neat) 3034, 1593, 1575, 1497, 1412, 1368, 1313, 1264, 1213, 1183, 1156, 1059, 1013, 974, 899, 878, 855, 756, 696, 537 cm^{-1}; HRMS cannot be obtained by ESI or APCI.

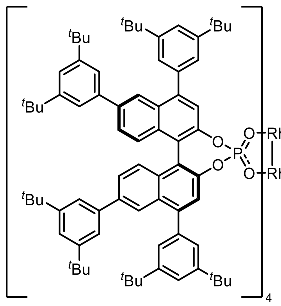

S-**6d**

Using ***general procedure E*** with (S)-4,4',6,6'-tetra(3,5-*di*-tert-butyl-phenyl)binaphtholphosphoric acid (2.07 g, 1.88 mmol), *S*-**6d** was obtained as a green solid (626 mg, 58 % yield) after purification by column chromatography.

^{1}H NMR (400 MHz, $CDCl_3$) δ 8.39 – 8.33 (m, 1H), 7.79 (s, 1H), 7.64 (d, J = 8.9 Hz, 1H), 7.60 – 7.54 (m, 1H), 7.48 (dd, J = 12.6, 1.6 Hz, 3H), 7.37 (s, 2H), 7.31 – 7.26 (m, 1H), 1.40 (s, 18H), 1.31 – 1.01 (m, 18H); **^{13}C NMR** (101 MHz, $CDCl_3$) δ 151.3, 147.6 (d, J = 9.8 Hz), 145.3, 140.9, 139.2, 138.7, 132.2, 130.5, 129.1, 128.9, 126.4, 126.2, 124.4, 122.5, 122.1, 121.9, 121.7, 120.8, 35.1, 34.8, 31.7, 31.6; **^{31}P NMR** (162 MHz, $CDCl_3$) δ 22.99; **IR** (neat) 2953, 1593, 1497, 1476, 1393, 1362, 1315, 1247, 1211, 1186, 1157, 1057, 972, 896, 876, 824, 756, 737, 714, 695, 599, 540 cm^{-1}; HRMS cannot be obtained by ESI or APCI.

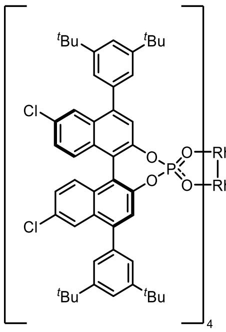

S-**1**

Using ***general procedure E*** with (S)-6,6'-dichloro-4,4'-bis(3,5-*di*-tert-butyl-phenyl)binaphtholphosphoric acid (1.05 g, 1.32 mmol), *S*-**1** was obtained as a green solid (496 mg, 89 % yield) after purification by column chromatography.

^{1}H NMR (400 MHz, $CDCl_3$) δ 8.15 (d, J = 2.1 Hz, 2H), 7.78 (s, 2H), 7.32 – 7.27 (m, 4H), 7.25 – 7.13 (m, 6H), 1.26 (brs, 18H), 0.87 (brs, 18H); **^{31}P NMR** (162 MHz, $CDCl_3$) δ 23.69; **^{13}C NMR** (151 MHz, $CDCl_3$) δ 151.6 and 150.4 (each broad signals due to hindered rotation), 147.7 (d, J = 10.1 Hz), 144.8, 137.6, 131.9, 131.4, 131.0, 129.4, 127.2, 125.8, 125.7 and 123.7 (each broad

signals due to hindered rotation), 122.8, 122.2, 120.5, 34.8, 31.4; **IR** (neat) 2962, 1581, 1497, 1394, 1292, 1200, 1060, 990, 971, 915, 775, 735, 601 cm^{-1}; HRMS cannot be obtained by ESI or APCI. The structure was confirmed by X-ray crystallography.

4. Characterization of C–H Insertion Products

4.1 Crude NMR Data for Determination of Regioselectivity and Diastereoselectivity

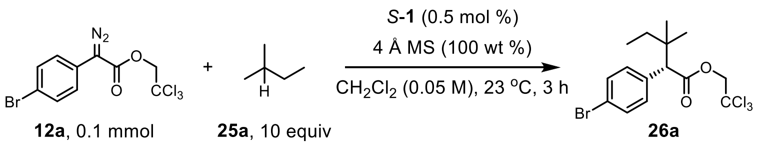

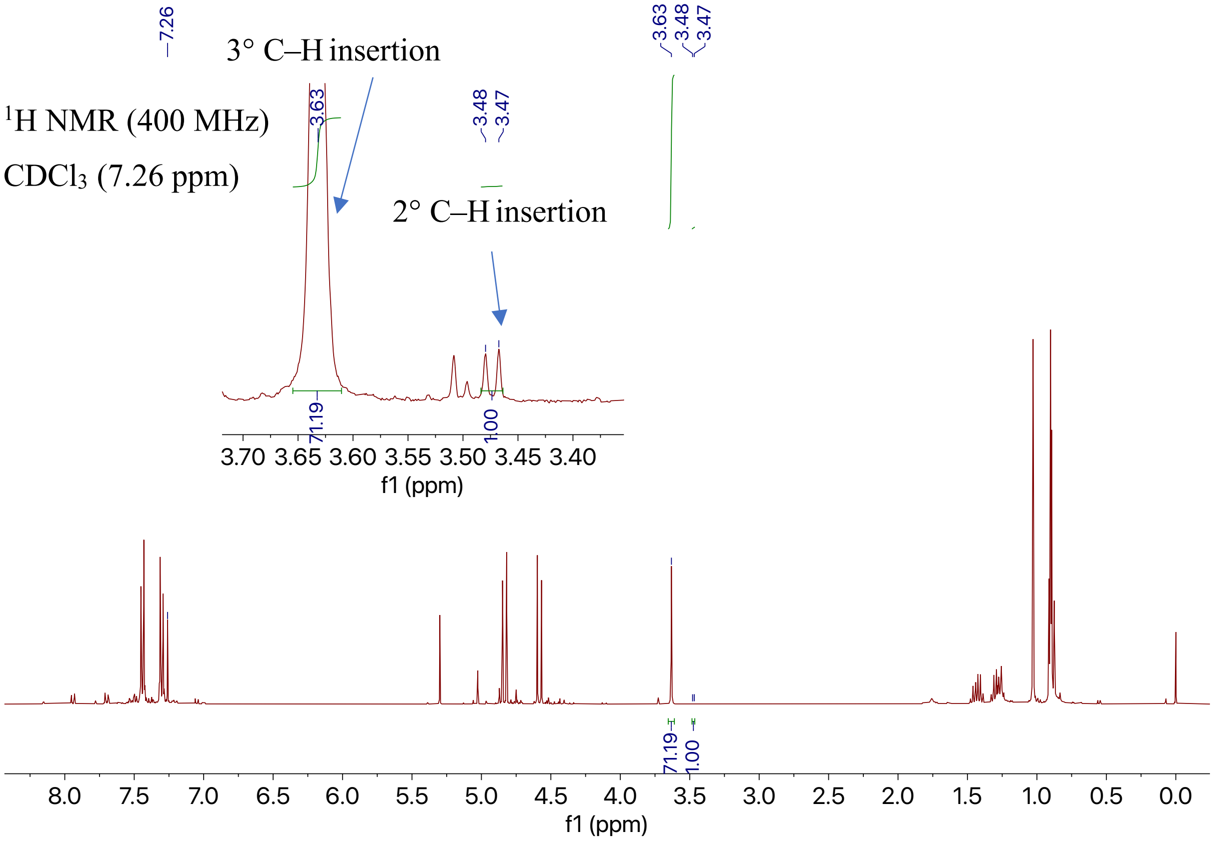

The 3° C–H insertion regio-selectivity is assigned based on the singlet at 3.63 (benzylic H) as previously discussed for related systems.[8]

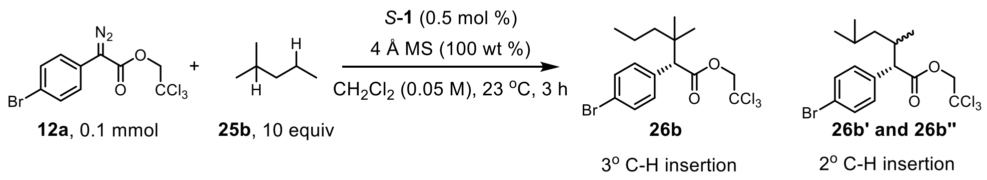

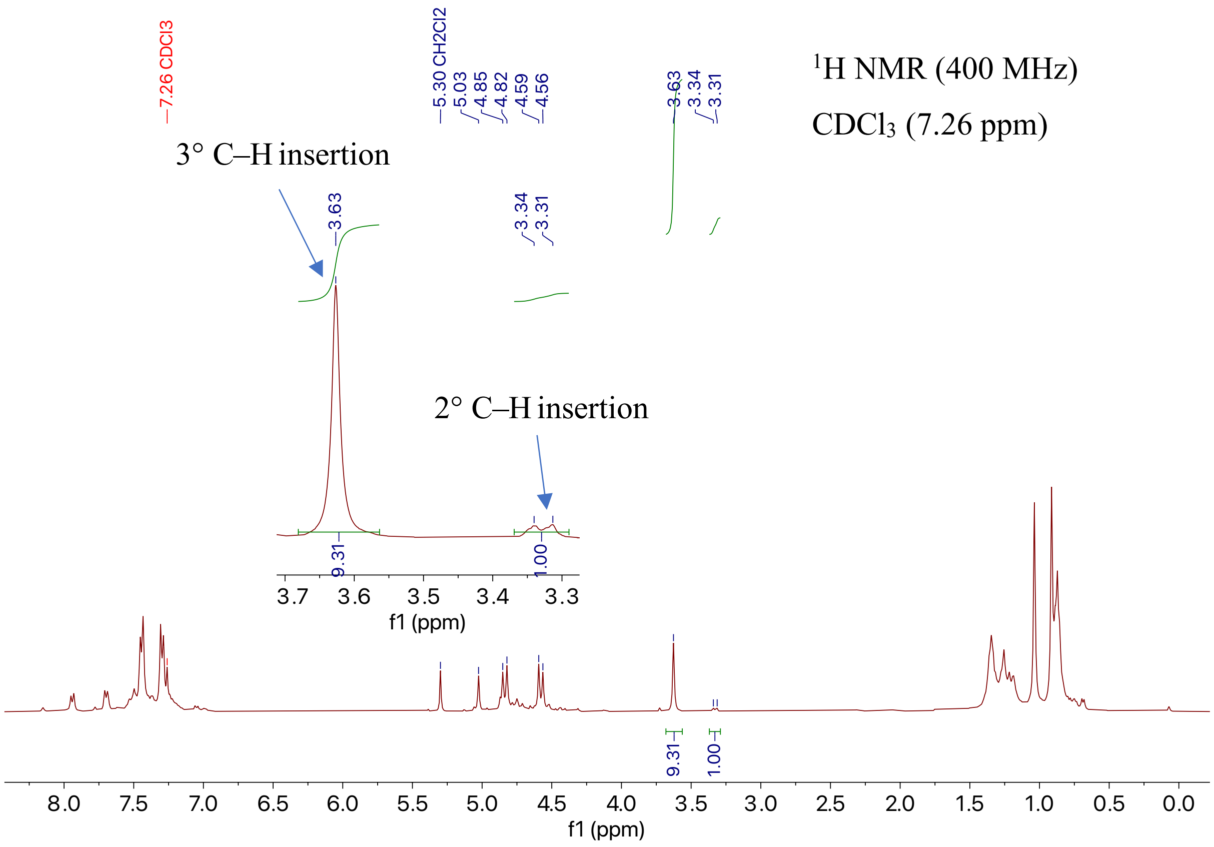

The 3° C–H insertion regio-selectivity is assigned based on the singlet at 3.63 (benzylic H) as previously discussed for related systems.[8]

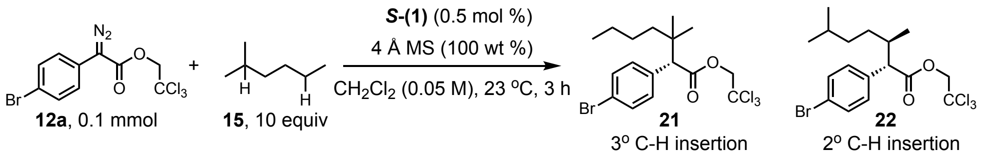

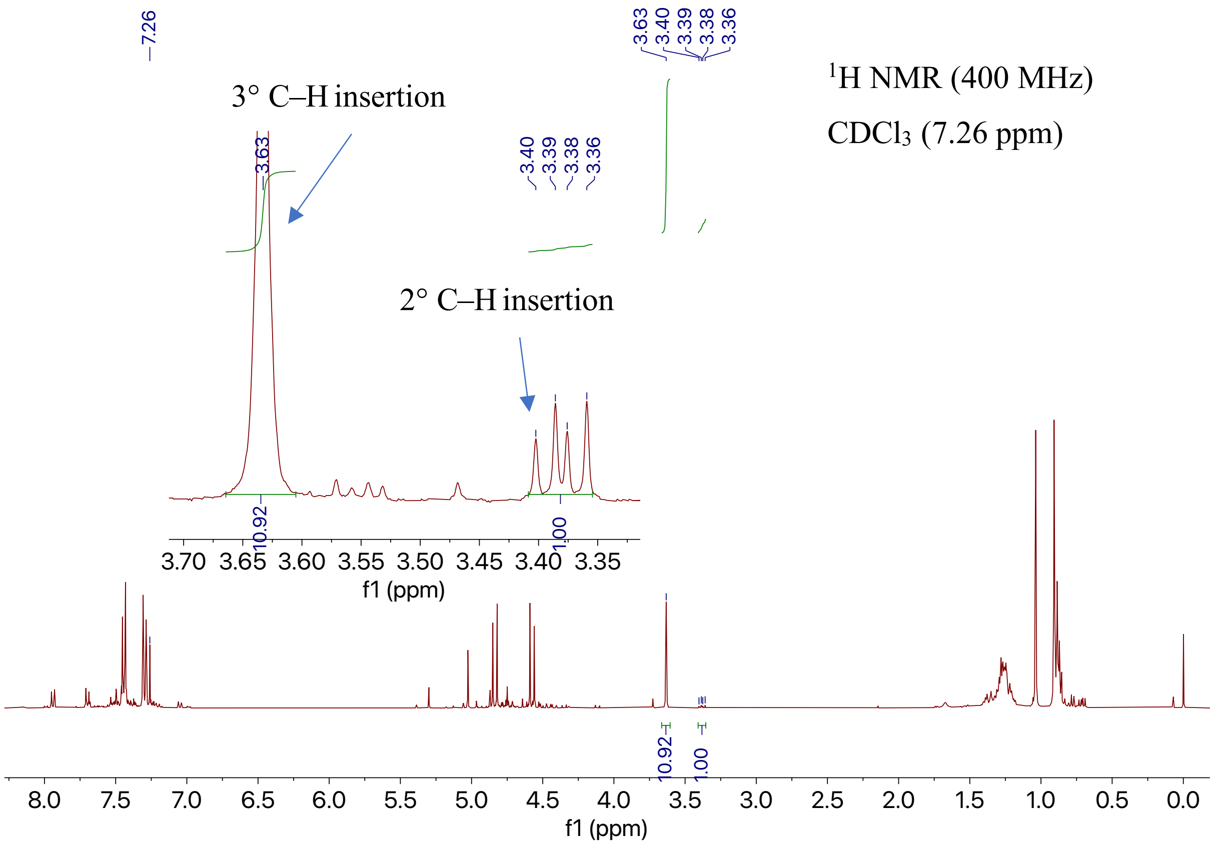

The 3° C–H insertion regio-selectivity is assigned based on the singlet at 3.63 (benzylic H) as previously discussed for related systems.[8]

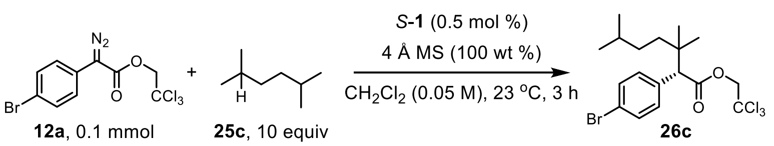

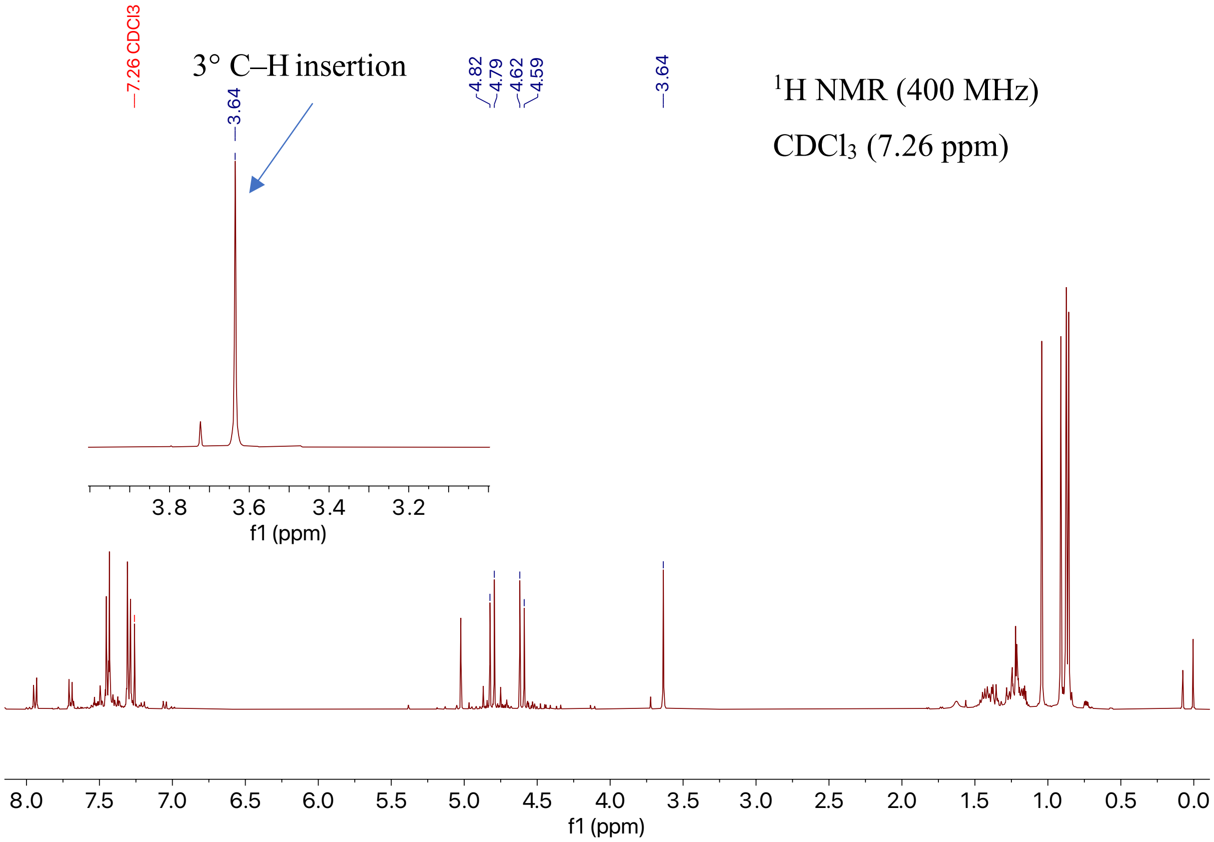

The 3° C–H insertion regio-selectivity is assigned based on the singlet at 3.64 (benzylic H) as previously discussed for related systems.[8]

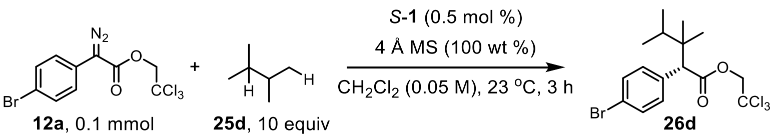

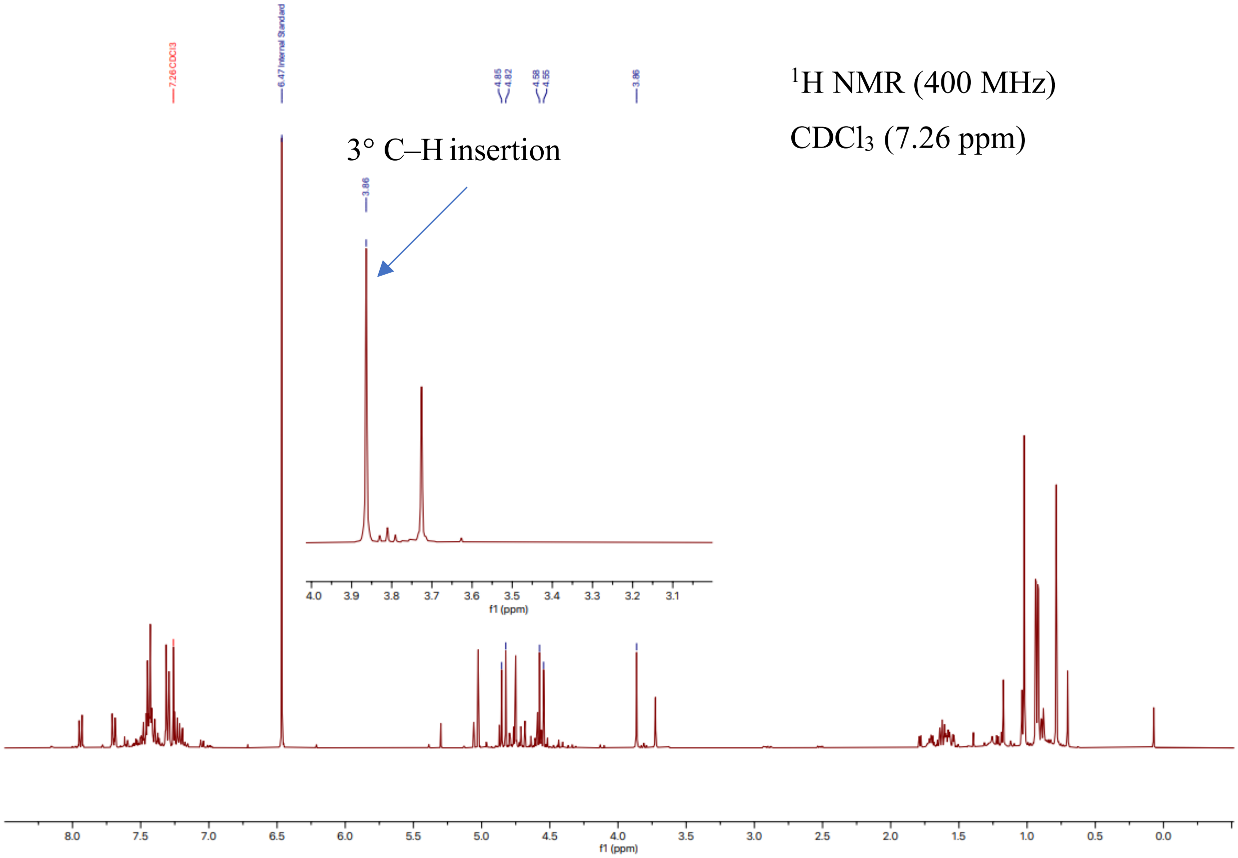

The 3° C–H insertion regio-selectivity is assigned based on the singlet at 3.86 (benzylic H) as previously discussed for related systems.[8]

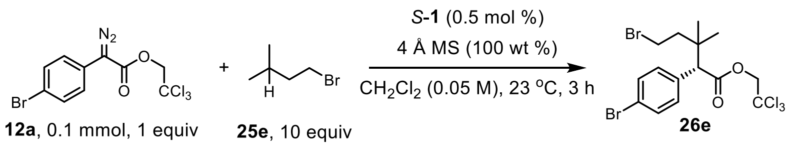

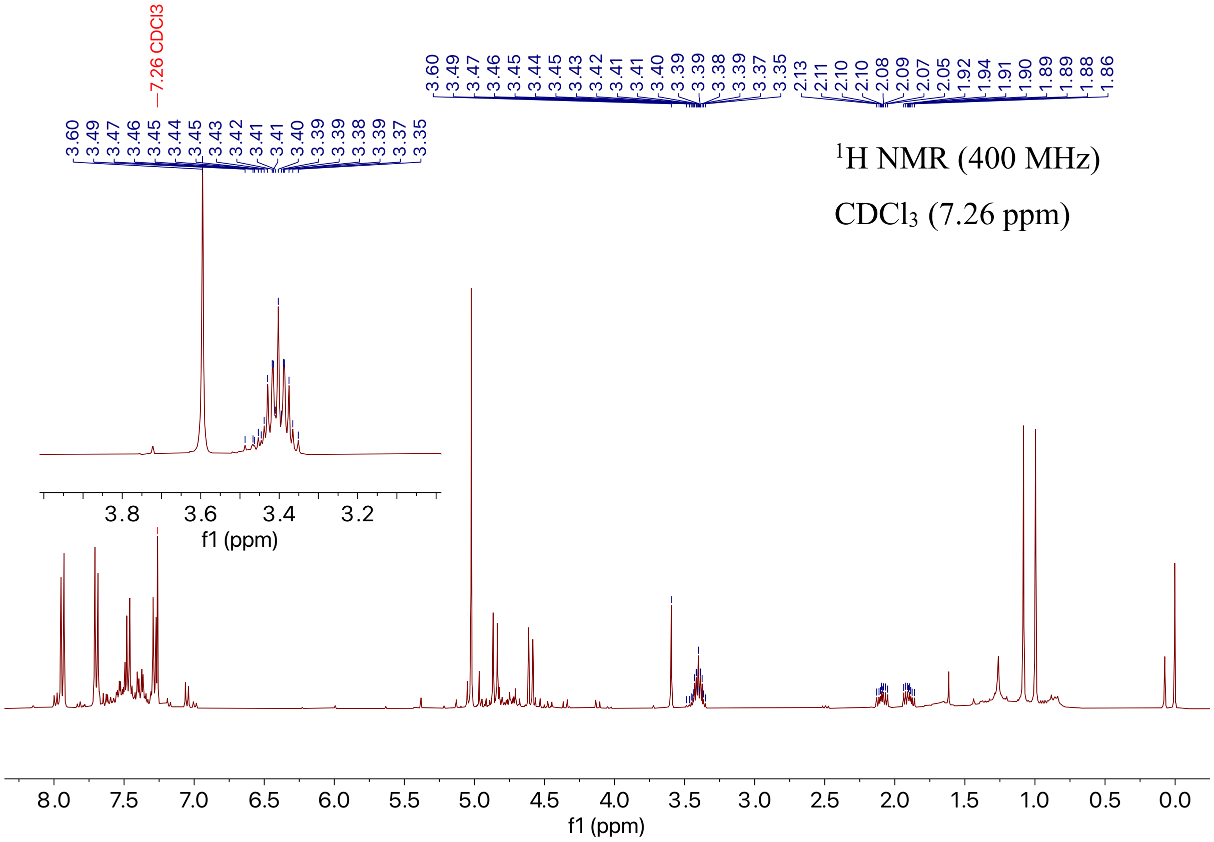

The 3° C–H insertion regio-selectivity is assigned based on the singlet at 3.60 (benzylic H) as previously discussed for related systems.[8]

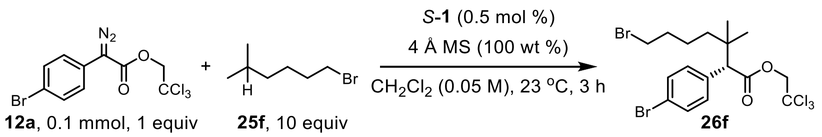

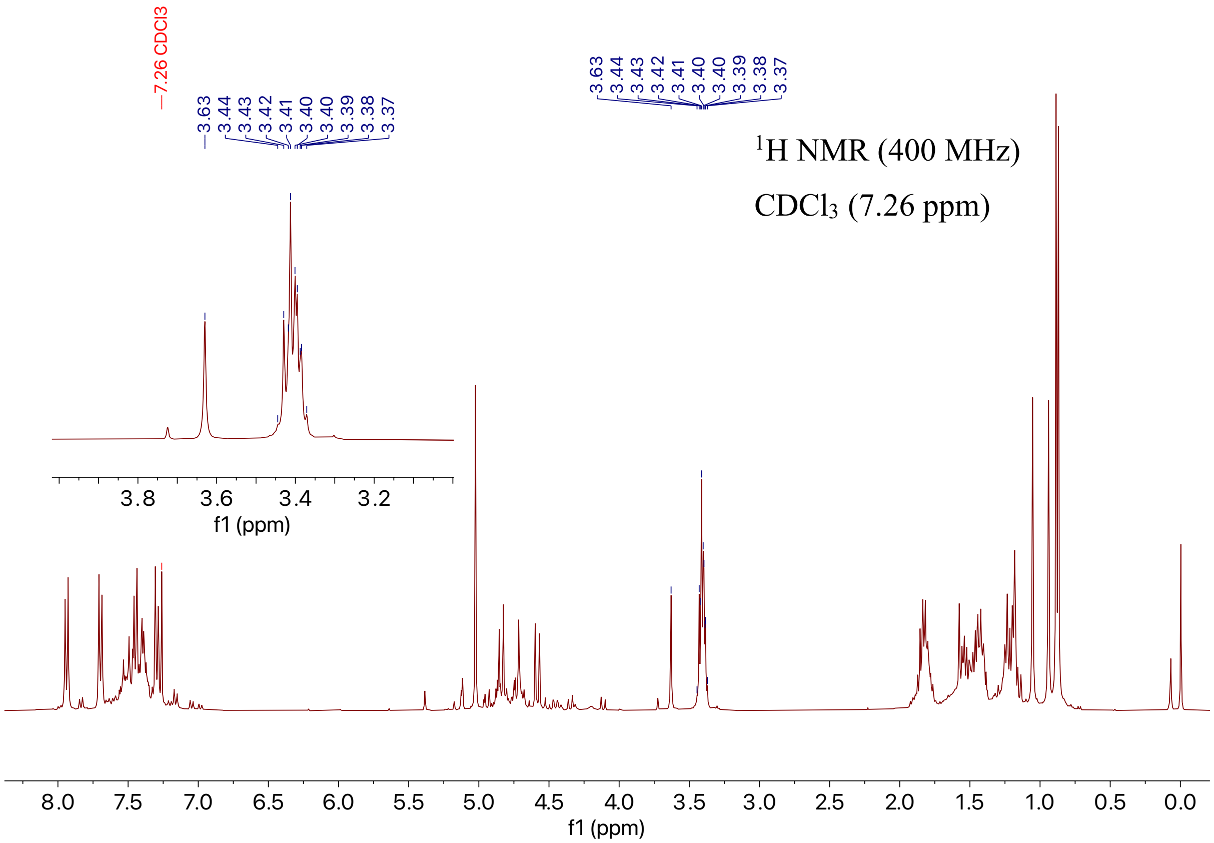

The 3° C–H insertion regio-selectivity is assigned based on the singlet at 3.63 (benzylic H) as previously discussed for related systems.[8]

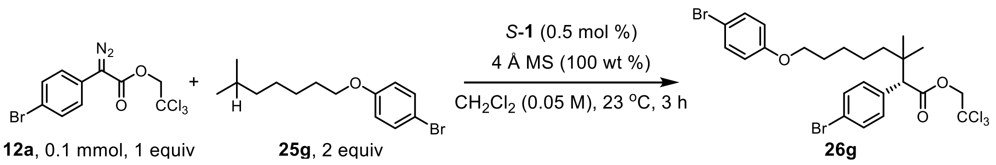

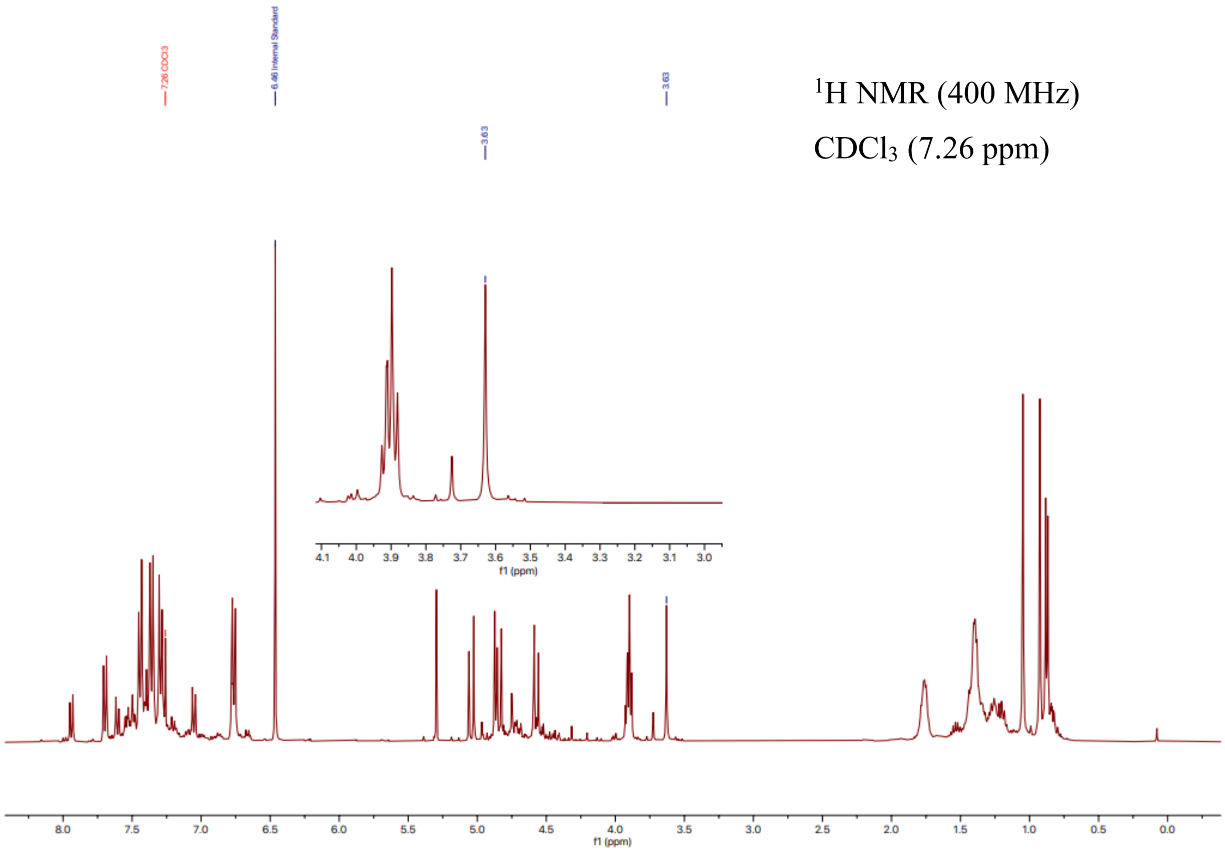

^{1}H NMR (400 MHz)

$CDCl_3$ (7.26 ppm)

The 3° C–H insertion regio-selectivity is assigned based on the singlet at 3.63 (benzylic H) as previously discussed for related systems.[8]

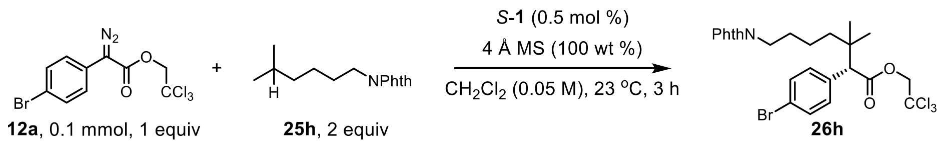

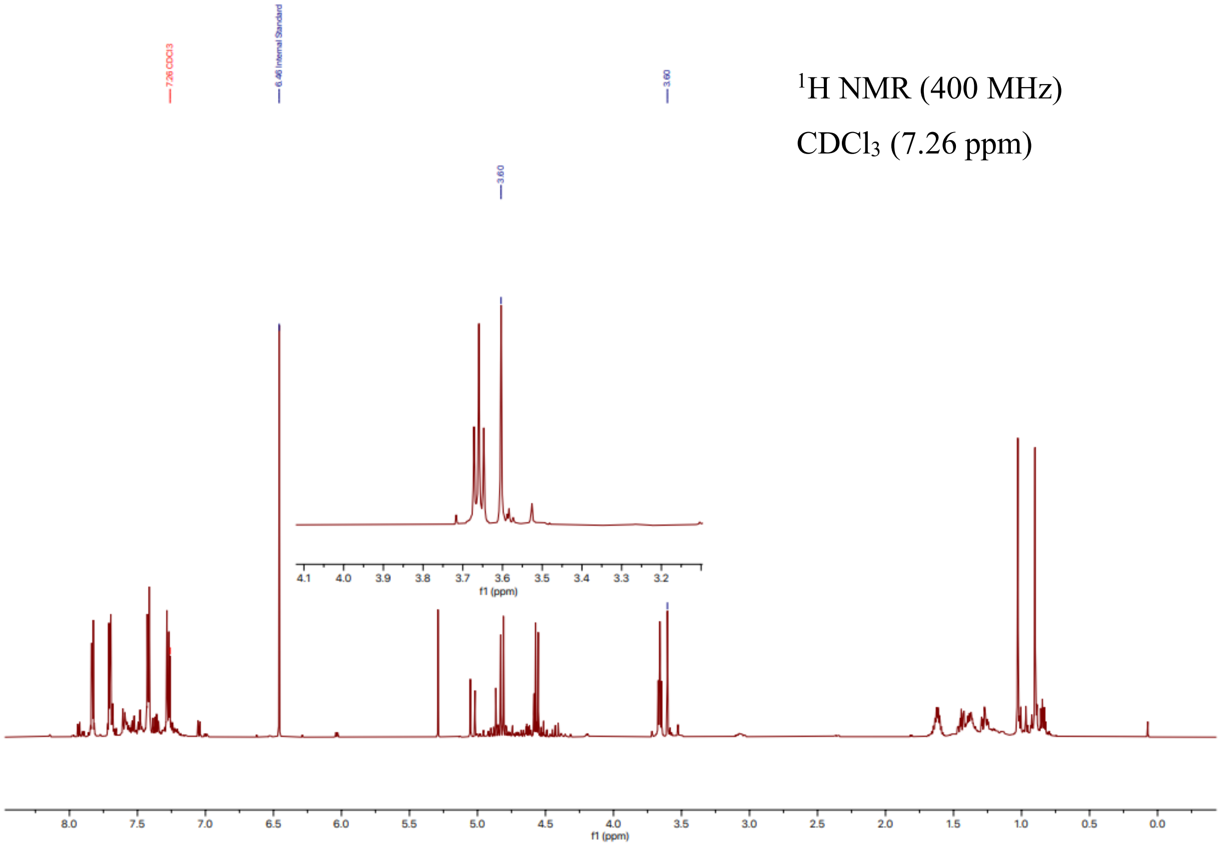

The 3° C–H insertion regio-selectivity is assigned based on the singlet at 3.60 (benzylic H) as previously discussed for related systems.[8]

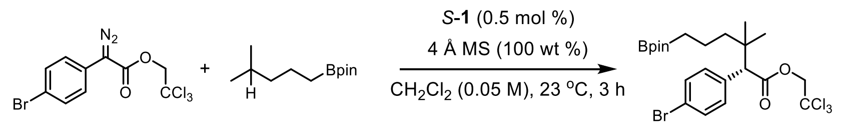

12a, 0.1 mmol, 1 equiv **25i**, 2 equiv **26i**

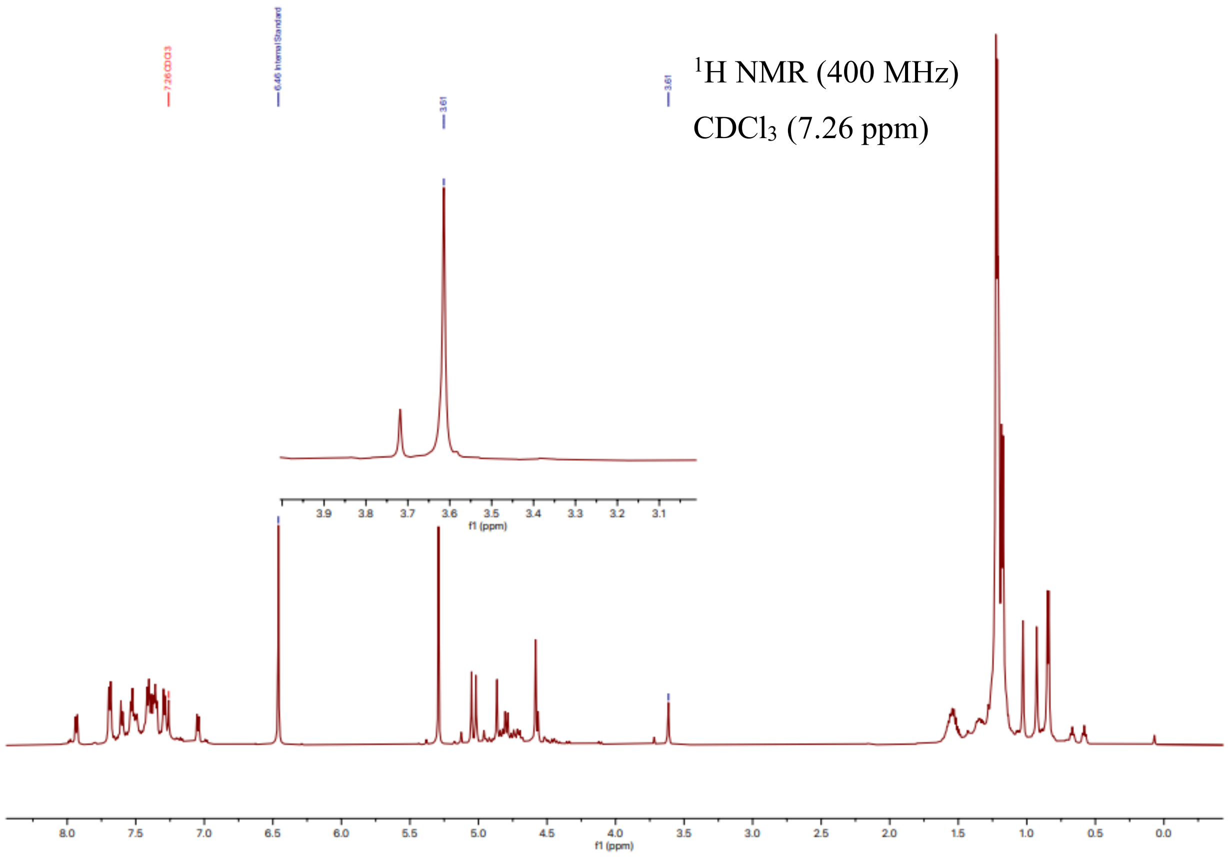

The 3° C–H insertion regio-selectivity is assigned based on the singlet at 3.61 (benzylic H) as previously discussed for related systems.[8]

4.2 Data Analysis for Scheme 3

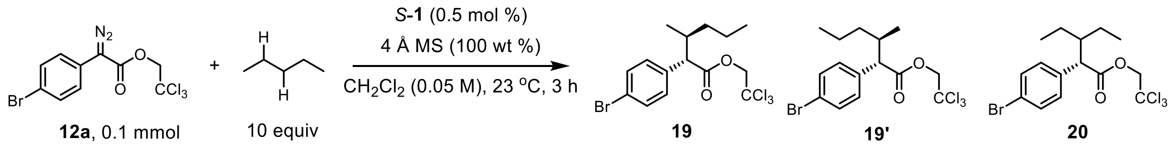

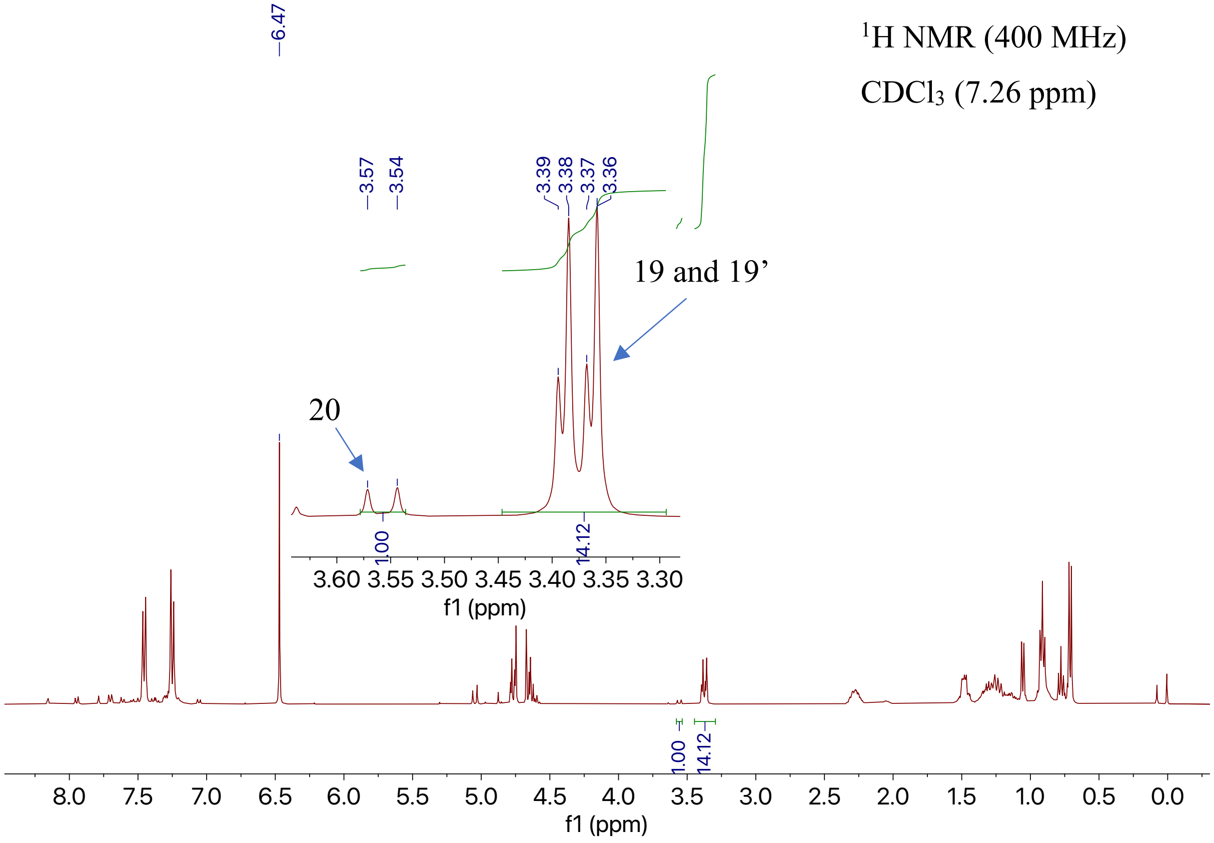

The 2° C–H insertion regio-selectivity is assigned based on the singlet at 3.38 and 3.37 (benzylic H) as previously discussed for related systems.[8]

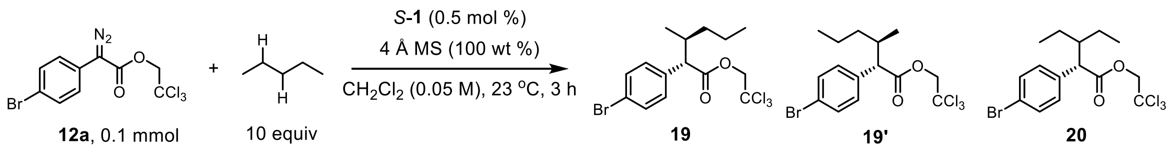

Using ***general procedure F*** with 2,2,2-trichloroethyl 2-(4-bromophenyl)-2-diazoacetate (**12a**, 0.1 mmol, 32.8 mg), **S-(1)** (0.5 mol%) and *n*-pentane (**14**, 10 equiv.), 2,2,2-trichloroethyl (2*S*,3*S*)-2-(4-bromophenyl)-3-methylhexanoate (**19**), 2,2,2-trichloroethyl (2*S*,3*R*)-2-(4-bromophenyl)-3-methylhexanoate (**19'**) and 2,2,2-trichloroethyl (*S*)-2-(4-bromophenyl)-3-ethylpentanoate (**20**) were obtained as a colorless liquid (32.1 mg, 77 % combined yield, **19/19'&20 = 14/1**, determined by crude ^{1}H NMR) after purification by column chromatography.

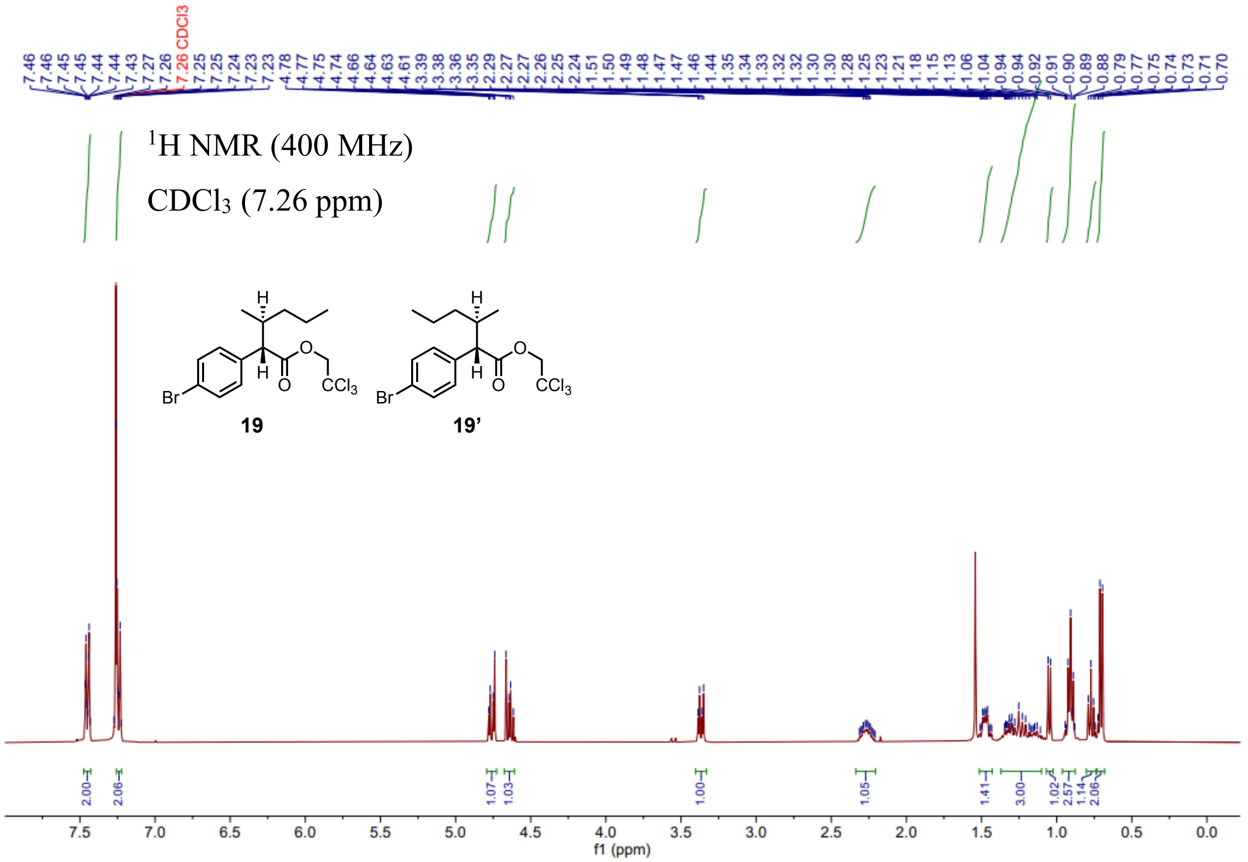

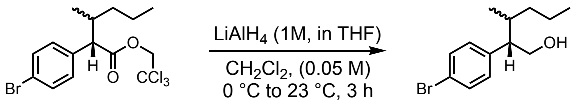

19 and 19' **19-1 and 19'-1**

For determination of enantioselectivity, ester groups were reduced to alcohol for better resolution. To an oven-dried 20 ml vial with a magnetic stir bar was added crude products, which was then dissolved in 4 ml dry dichloromethane. The vial was sealed by a septum and was vacuumed and refilled with N_2. Then 0.1 mL $LiAlH_4$ in tetrahydrofuran (1 M) was added to the vial at 0 °C and was stirred at room temperature for 12 hours before quenching with Na_2SO_4•$10H_2O$. The crude material was dry-loaded to be purified by column chromatography (16.5 mg, 75 % combined yield, mixture of **19-1/19'-1**). Spectroscopic data matches with these in the reported literature[7].

^1H NMR shows a mixture of diastereomers **19-1 (major)** and **19'-1 (minor)**

(19-1) HPLC (Regis (S,S) Whelk-O 1, 1 mL/min, 1% isoproponal in hexane, λ 230 nm), retention times of 43.74 min (major) and 76.00 min (minor), 90% ee.

(19'-1) HPLC (Regis (S,S) Whelk-O 1, 1 mL/min, 1% isoproponal in hexane, λ 230 nm), retention times of 39.68 min (major) and 71.35 min (minor), 96% ee.

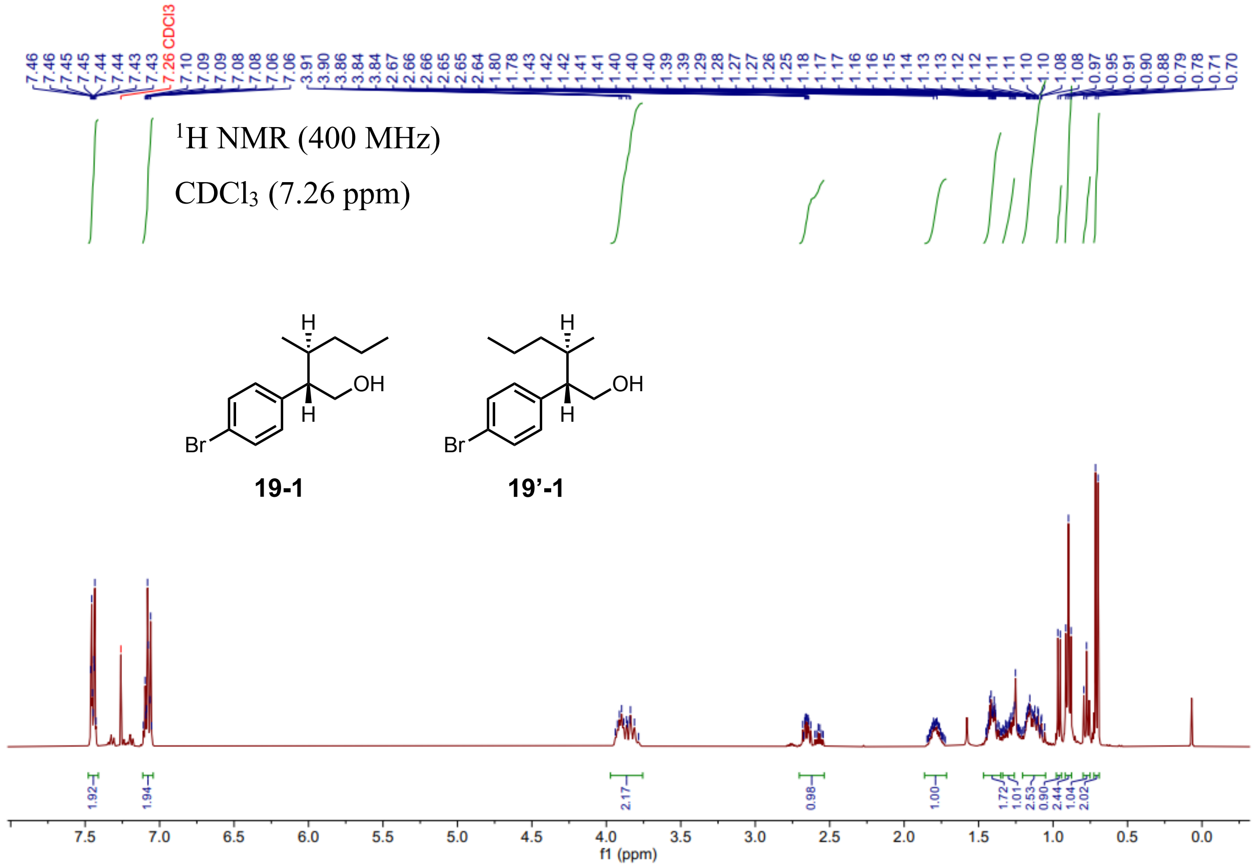

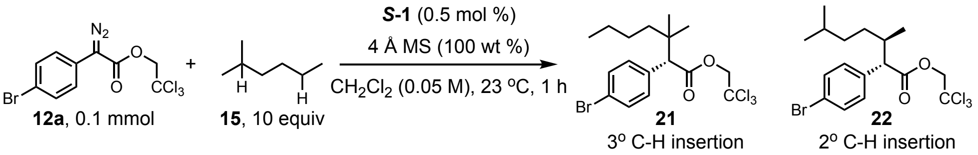

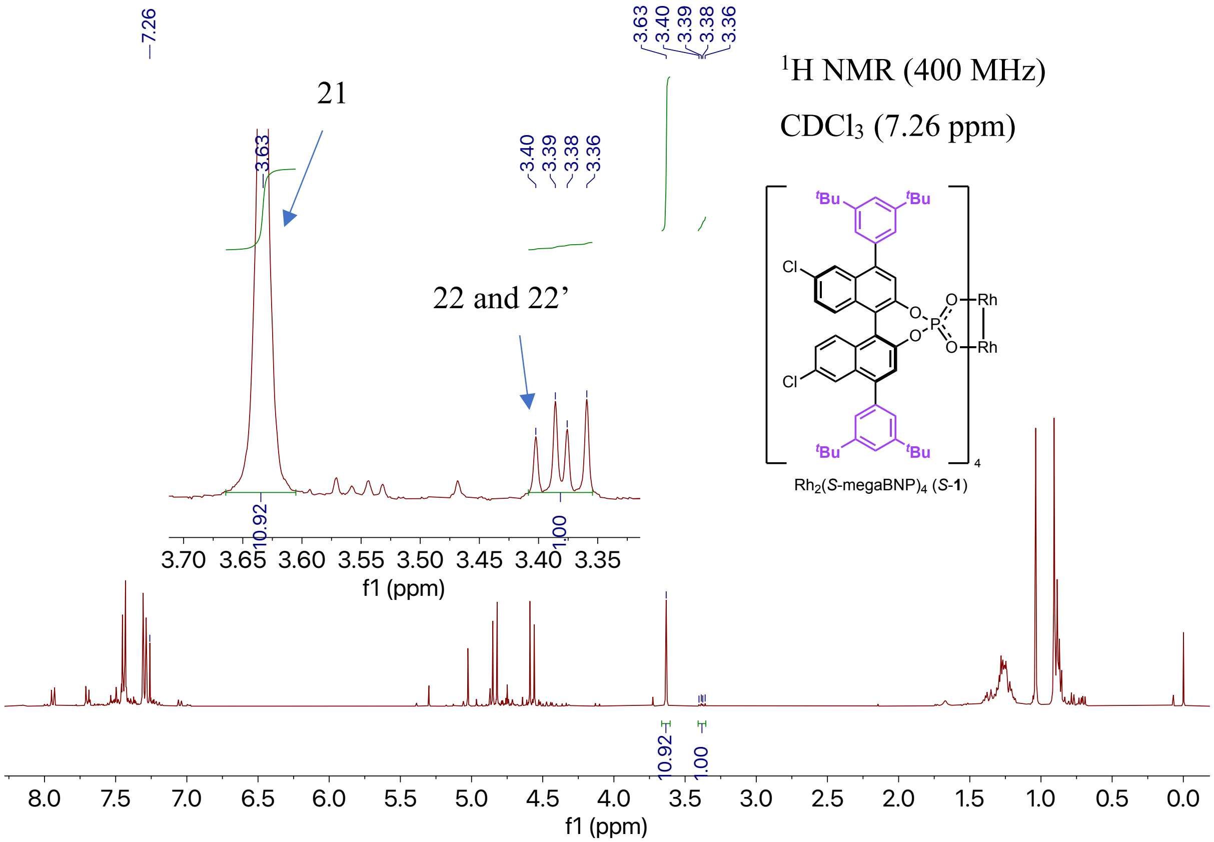

The 3° C–H insertion regio-selectivity is assigned based on the singlet at 3.63 (benzylic H) as previously discussed for related systems.[8]

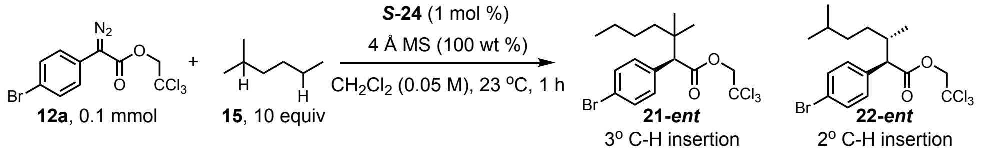

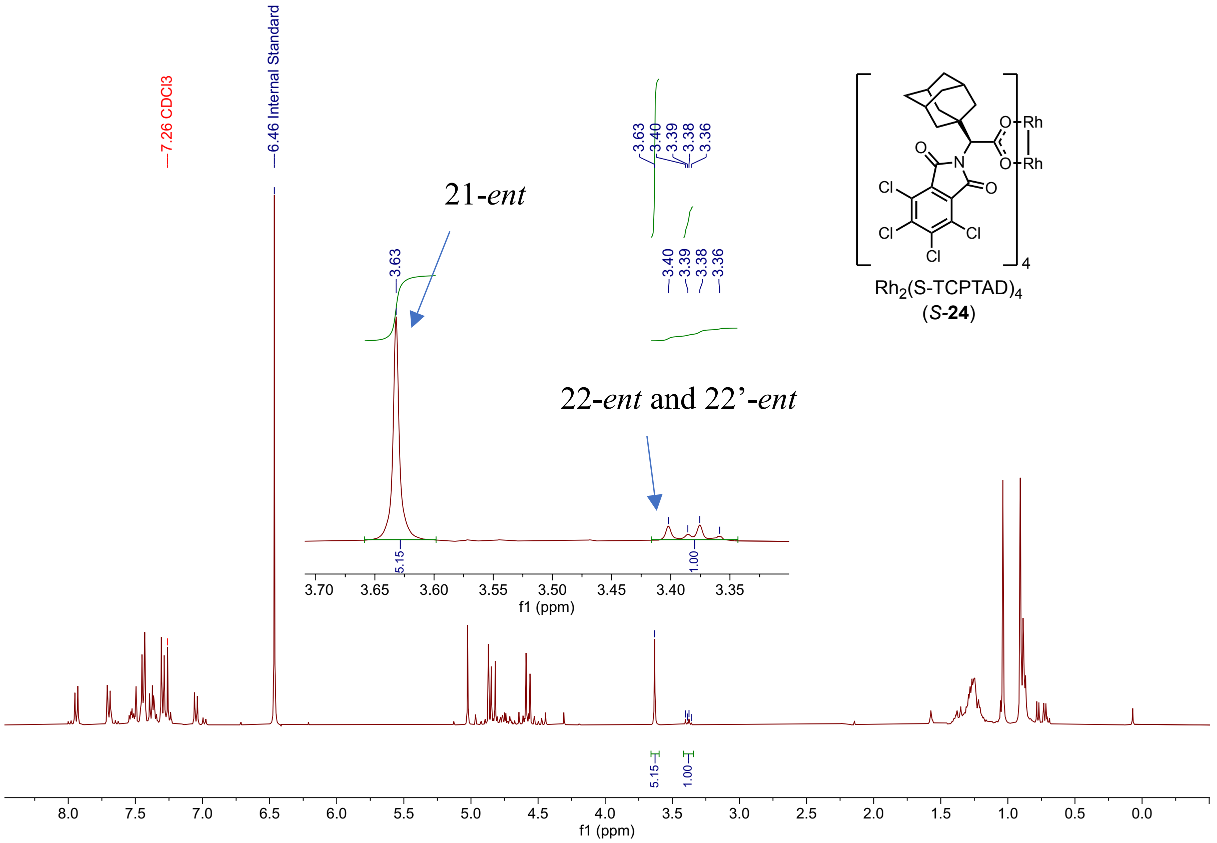

The 3° C–H insertion regio-selectivity is assigned based on the singlet at 3.63 (benzylic H) as previously discussed for related systems.[8]

Table S4.2.1 Comparison between *S*-**1** and *S*-**24** for C–H functionalization of 2-methylhexane

Entry	Catalyst	r.r.(3°:2°)	ee(%)
A	***S*-1**	11:1	99
B	***S*-24**	5:1	-77*

*favoring the opposite enantiomer (as **21-*ent***)

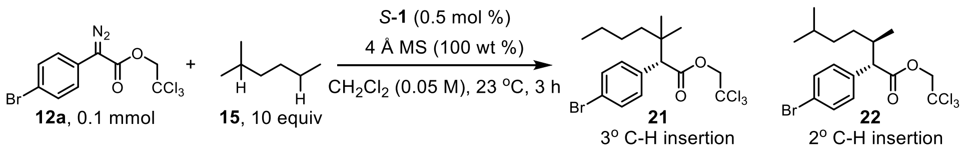

Using ***general procedure F*** with 2,2,2-trichloroethyl 2-(4-bromophenyl)-2-diazoacetate (**12a**, 0.1 mmol, 37.2 mg), **S-(1)** (1.7 mg, 0.5 mol %) and 2-methylhexane (**15**, 10 equiv), 2,2,2-trichloroethyl (*S*)-2-(4-bromophenyl)-3,3-dimethylheptanoate (**21**), 2,2,2-trichloroethyl (2*S*,3*R*)-2-(4-bromophenyl)-3,5-dimethylheptanoate (**22**) and 2,2,2-trichloroethyl (2*S*,3*S*)-2-(4-bromophenyl)-3,5-dimethylheptanoate (**22'**) were obtained as a colorless liquid (36.5 mg, 82 % combined yield, **21/(22 and 22') = 11/1**, determined by crude ^{1}H NMR) after purification by column chromatography.

Characterization of 21:

$[\alpha]^{20}_{D}$: -6.6°(c = 1.67, $CHCl_3$, 91% ee); **^{1}H NMR** (400 MHz, $CDCl_3$) δ 7.47 – 7.42 (m, 2H), 7.32 – 7.28 (m, 2H), 4.83 (d, J = 12.0 Hz, 1H), 4.57 (d, J = 12.0 Hz, 1H), 3.63 (s, 1H), 1.33 – 1.19 (m, 6H), 1.04 (s, 3H), 0.93 – 0.86 (m, 6H); **^{13}C NMR** (101 MHz, $CDCl_3$) δ 171.3, 134.3, 132.0, 131.2, 121.8, 94.9, 74.3, 59.7, 40.6, 37.4, 26.2, 24.7, 24.3, 23.6, 14.3; **IR** (neat) 2956, 2931, 2871, 1747, 1589, 1488, 1467, 1411, 1369, 1304, 1260, 1217, 1161, 1119, 1074, 1029, 1011, 902, 828, 762, 719, 574 cm^{-1}; **HRMS** (FTMS +p APCI) calcd for $C_{17}H_{23}O_2{}^{79}Br^{35}Cl_3$ $[M+H]^+$ 442.9942 found 442.9942.

HPLC (Regis (S,S) Whelk-O 1, 0.5 mL/min, 0.5% isoproponal in hexane, *λ* 230 nm), retention times of 11.49 min (minor) and 12.58 min (major), 91% ee.

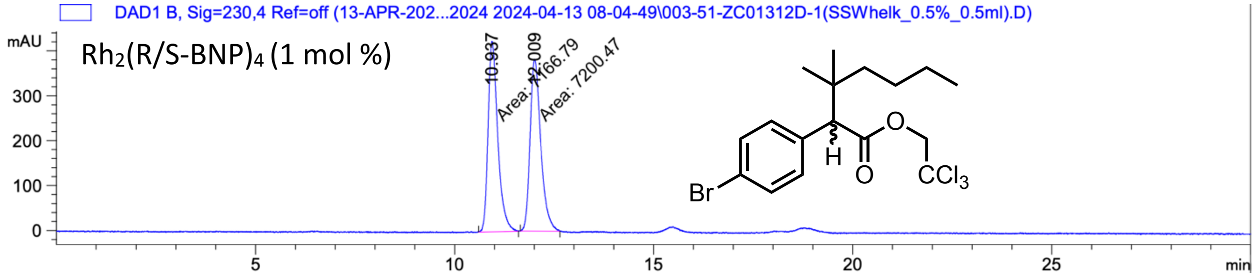

Signal 2: DAD1 B, Sig=230,4 Ref=off

Peak #	RetTime [min]	Type	Width [min]	Area [mAU*s]	Height [mAU]	Area %
1	10.937	MM	0.2815	7166.79395	424.31329	49.8828
2	12.009	MM	0.3131	7200.46533	383.25952	50.1172
Totals :				1.43673e4	807.57281	

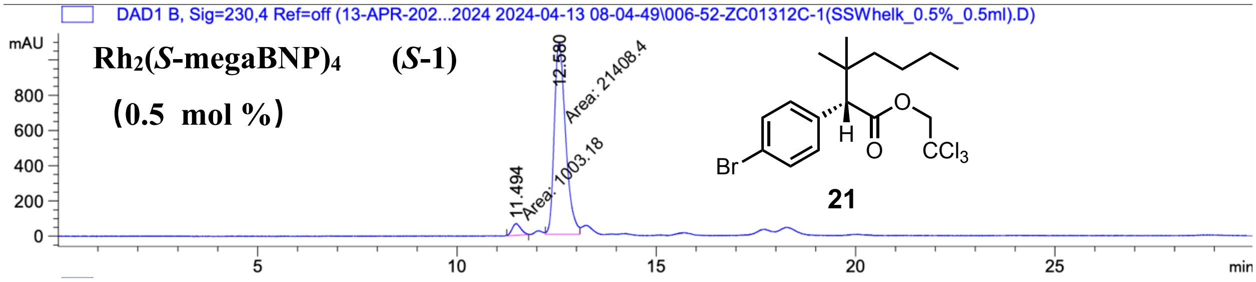

Signal 2: DAD1 B, Sig=230,4 Ref=off

Peak #	RetTime [min]	Type	Width [min]	Area [mAU*s]	Height [mAU]	Area %
1	11.494	MM	0.2518	1003.18427	66.41367	4.4762
2	12.580	MM	0.3293	2.14084e4	1083.60535	95.5238
Totals :				2.24116e4	1150.01901	

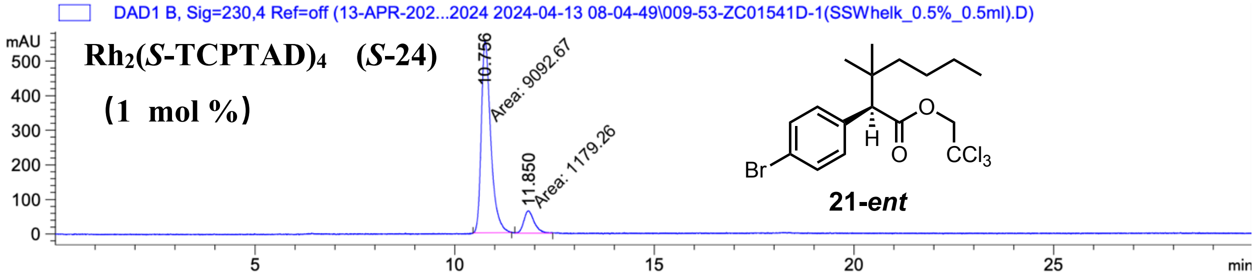

Signal 2: DAD1 B, Sig=230,4 Ref=off

Peak #	RetTime [min]	Type	Width [min]	Area [mAU*s]	Height [mAU]	Area %
1	10.756	MM	0.2741	9092.67090	552.86047	88.5196
2	11.850	MM	0.3039	1179.26160	64.68212	11.4804
Totals :				1.02719e4	617.54259	

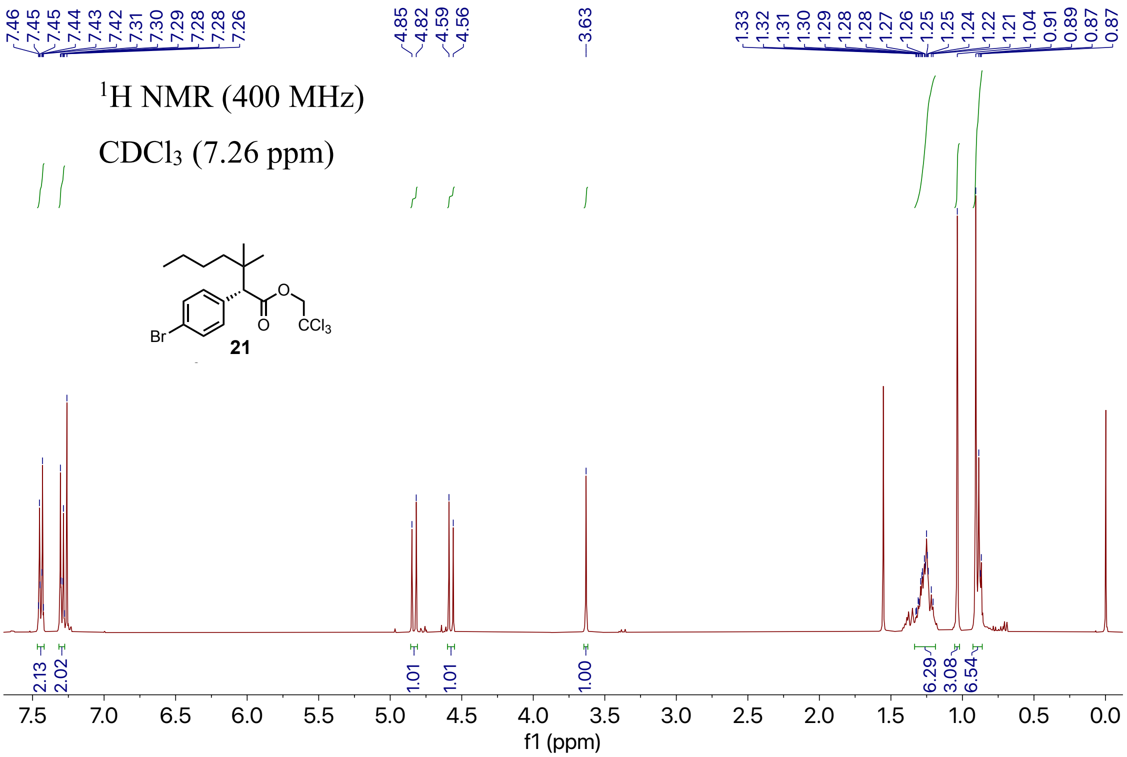

7.46 7.45 7.45 7.44 7.43 7.42 7.31 7.30 7.29 7.28 7.28 7.26
4.85 4.82 4.59 4.56
3.63
1.33 1.32 1.31 1.30 1.29 1.28 1.28 1.27 1.26 1.25 1.25 1.24 1.22 1.21 1.04 0.91 0.89 0.87 0.87
¹H NMR (400 MHz)
CDCl₃ (7.26 ppm)
Br
O
O
CCl₃
21
2.13 2.02 1.01 1.01 1.00 6.29 3.08 6.54
7.5 7.0 6.5 6.0 5.5 5.0 4.5 4.0 3.5 3.0 2.5 2.0 1.5 1.0 0.5 0.0
f1 (ppm)

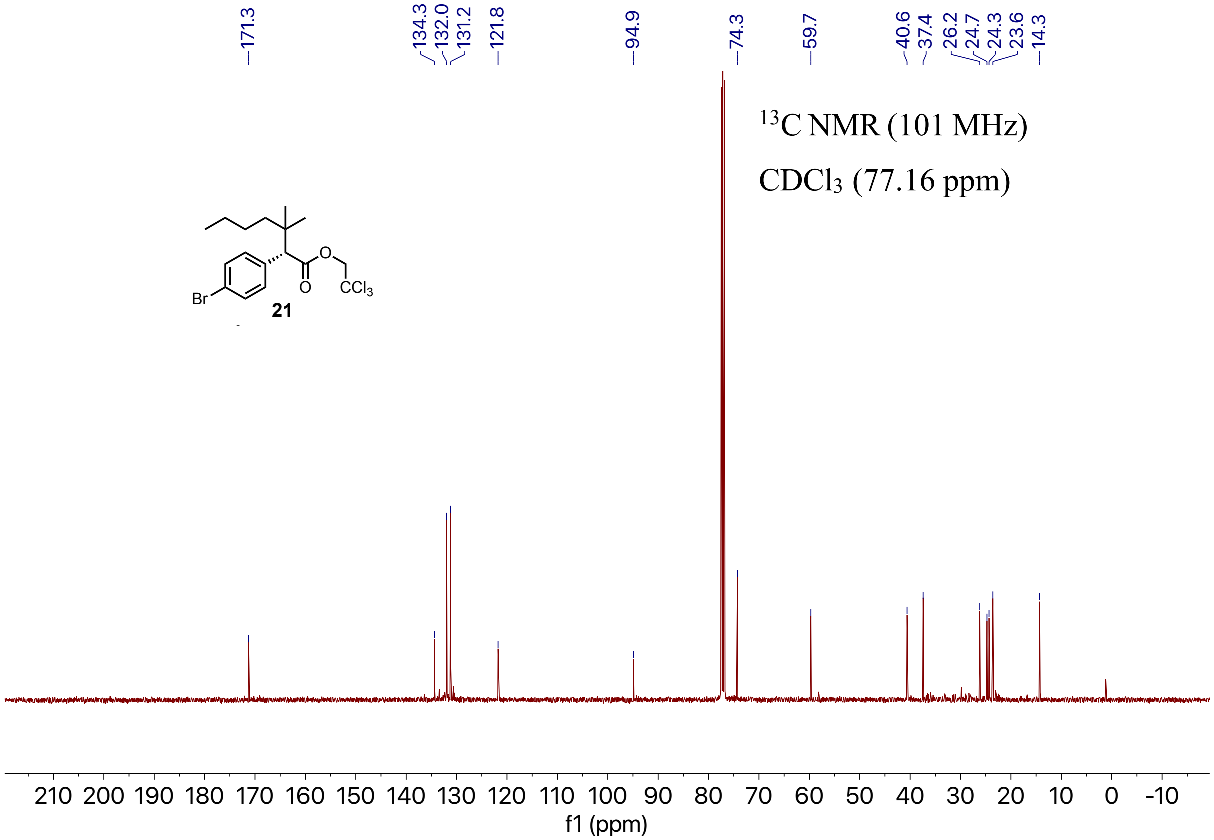

171.3
134.3 132.0 131.2 121.8
94.9
74.3
59.7
40.6 37.4 26.2 24.7 24.3 23.6 14.3
¹³C NMR (101 MHz)
CDCl₃ (77.16 ppm)
Br
O
O
CCl₃
21
210 200 190 180 170 160 150 140 130 120 110 100 90 80 70 60 50 40 30 20 10 0 -10
f1 (ppm)

4.3 Procedure and Characterization of C–H Insertion Products

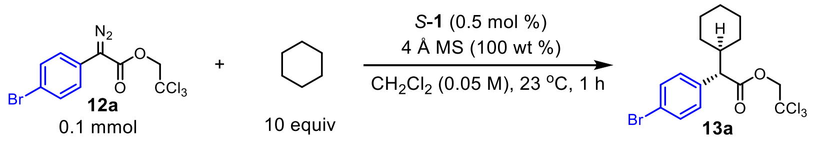

Using ***general procedure F*** with 2,2,2-trichloroethyl 2-(4-bromophenyl)-2-diazoacetate (**12a**, 0.1 mmol, 37.2 mg), *S*-**1** (1.7 mg, 0.5 mol %) and cyclohexane (10 equiv), 2,2,2-trichloroethyl (*S*)-2-(4-bromophenyl)-2-cyclohexylacetate (**13a**) was obtained as a colorless liquid (36.4 mg, 85 % yield) after purification by column chromatography. Spectroscopic data matches with these in the reported literature.[8]

^{1}H NMR (500 MHz, $CDCl_3$) δ 7.47 – 7.42 (m, 2H), 7.25 – 7.22 (m, 2H), 4.76 (d, J = 12.0 Hz, 1H), 4.63 (d, J = 12.0 Hz, 1H), 3.34 (d, J = 10.7 Hz, 1H), 2.05 (qt, J = 11.0, 3.3 Hz, 1H), 1.89 – 1.82 (m, 1H), 1.79 – 1.71 (m, 1H), 1.68 – 1.60 (m, 2H), 1.38 – 1.31 (m, 1H), 1.34 – 1.24 (m, 1H), 1.19 – 1.10 (m, 2H), 1.13 – 1.04 (m, 1H), 0.82 – 0.71 (m, 1H).

HPLC (ChiralPak AD-H, 1 mL/min, 0.1% isoproponal in hexane, *λ* 230 nm), retention times of 8.58 min (major) and 15.37 min (minor), 99% ee.

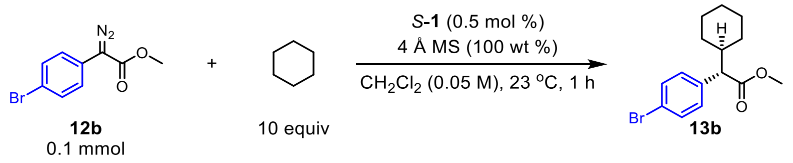

Using ***general procedure F*** with methyl 2-(4-bromophenyl)-2-diazoacetate (**12b**, 0.1 mmol, 25.5 mg), *S*-**1** (1.7 mg, 0.5 mol %) and cyclohexane (10 equiv), methyl (*S*)-2-(4-bromophenyl)-2-cyclohexylacetate (**13b**) was obtained as a colorless liquid (80 % ^{1}H NMR yield; 23.0 mg, 74 % isolation yield) after purification by column chromatography. Spectroscopic data matches with these in the reported literature.[9]

^{1}H NMR (400 MHz, $CDCl_3$) δ 7.43 (d, J = 8.3 Hz, 2H), 7.20 (d, J = 8.3 Hz, 2H), 3.64 (s, 3H), 3.19 (d, J = 10.6 Hz, 1H), 1.96 (qt, J = 11.1, 3.3 Hz, 1H), 1.82 – 1.69 (m, 2H), 1.68 – 1.57 (m, 2H), 1.36 – 1.24 (m, 2H), 1.20 – 0.97 (m, 3H), 0.72 (qd, J = 12.1, 3.6 Hz, 1H).

UPLC (Regis (S,S) Whelk-O 1, 1 mL/min, 1% isoproponal in hexane, *λ* 230 nm), retention times of 6.28 min (major) and 9.47 min (minor), 90% ee.

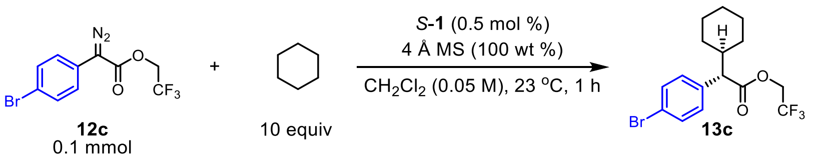

Using ***general procedure F*** with 2,2,2-trifluoroethyl 2-(4-bromophenyl)-2-diazoacetate (**12c**, 0.1 mmol, 32.3 mg), *S*-**1** (1.7 mg, 0.5 mol %) and cyclohexane (10 equiv), 2,2,2-trifluoroethyl (*S*)-2-(4-bromophenyl)-2-cyclohexylacetate (**13c**) was obtained as a colorless liquid (93 % ^{1}H NMR yield; 33.0 mg, 87 % isolation yield) after purification by column chromatography. Spectroscopic data matches with these in the reported literature.[10]

^{1}H NMR (400 MHz, $CDCl_3$) δ 7.45 (d, J = 8.4 Hz, 2H), 7.20 (d, J = 8.4 Hz, 2H), 4.54 (dq, J = 12.7, 8.4 Hz($^3J_{H-F}$), 1H), 4.32 (dq, J = 12.7, 8.4 Hz($^3J_{H-F}$), 1H), 3.30 (d, J = 10.6 Hz, 1H), 1.99 (qt, J = 11.2, 3.3 Hz, 1H), 1.81 – 1.71 (m, 2H), 1.70 – 1.58 (m, 2H), 1.37 – 1.26 (m, 2H), 1.20 – 1.01 (m, 3H), 0.75 (qd, J = 12.1, 3.4 Hz, 1H).

HPLC (ChiralPak AD-H, 1 mL/min, 0.1% isopropanal in hexane, *λ* 230 nm), retention times of 6.72 min (major) and 8.17 min (minor), 91% ee.

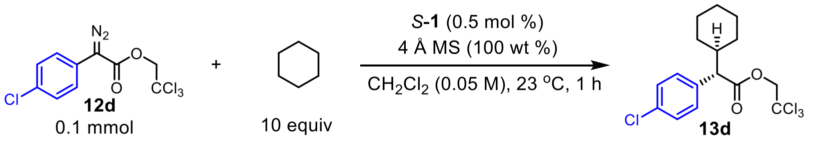

Using ***general procedure F*** with 2,2,2-trichloroethyl 2-(4-chlorophenyl)-2-diazoacetate (**12d**, 0.1 mmol, 37.2 mg), *S*-**1** (1.7 mg, 0.5 mol %) and cyclohexane (10 equiv), 2,2,2-trichloroethyl (*S*)-2-(4-chlorophenyl)-2-cyclohexylacetate (**13d**) was obtained as a colorless liquid (34.6 mg, 90 % yield) after purification by column chromatography.

$[\alpha]^{20}_D$: -5.5°(c = 1.52, $CHCl_3$, 93% ee); **^{1}H NMR** (400 MHz, $CDCl_3$) δ 7.30 (s, 4H), 4.76 (d, J = 12.0 Hz, 1H), 4.63 (d, J = 12.0 Hz, 1H), 3.36 (d, J = 10.7 Hz, 1H), 2.05 (qt, J = 11.0, 3.3 Hz, 1H), 1.90 – 1.82 (m, 1H), 1.79 – 1.71 (m, 1H), 1.70 – 1.57 (m, 2H), 1.39 – 1.24 (m, 2H), 1.19 – 1.04 (m, 3H), 0.76 (qd, J = 12.3, 3.4 Hz, 1H); **^{13}C NMR** (101 MHz, $CDCl_3$) δ 171.9, 135.6, 133.6, 130.2, 128.9, 95.0, 74.3, 58.2, 41.0, 32.0, 30.4, 26.3, 26.01, 25.97; **IR** (neat) 2925, 2852, 1748, 1491, 1218, 1143, 1122, 1016, 831, 764 cm^{-1}; **HRMS** (FTMS +p APCI) calcd for $C_{14}H_{17}O_2N_2{}^{35}Cl_4$ $[M+H]^+$: 383.0134 found 383.0135.

HPLC (ChiralPak AD-H, 0.5 mL/min, 0.5% isopropanal in hexane, *λ* 230 nm), retention times of 11.69 min (major) and 16.78 min (minor), 93% ee.

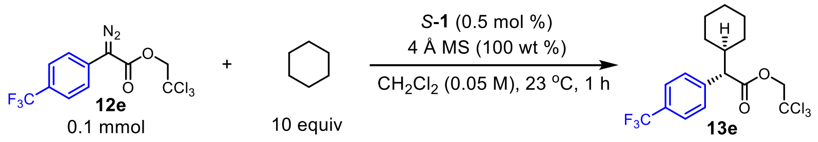

Using ***general procedure F*** with 2,2,2-trichloroethyl 2-(4-(trifluoromethyl)phenyl)-2-diazoacetate (**12e**, 0.1 mmol, 36.2 mg), *S*-**1** (1.7 mg, 0.5 mol %) and cyclohexane (10 equiv), 2,2,2-trichloroethyl (*S*)-2-(4-(trifluoromethyl)phenyl)-2-cyclohexylacetate (**13e**) was obtained as a colorless liquid (33.4 mg, 81 % yield) after purification by column chromatography. Spectroscopic data matches with these in the reported literature.[10]

^{1}H NMR (400 MHz, $CDCl_3$) δ 7.59 (d, J = 8.1 Hz, 2H), 7.49 (d, J = 8.1 Hz, 2H), 4.77 (d, J = 12.0 Hz, 1H), 4.64 (d, J = 12.0 Hz, 1H), 3.46 (d, J = 10.7 Hz, 1H), 2.11 (qt, J = 11.1, 3.4 Hz, 1H), 1.89 (d, J = 12.6 Hz, 1H), 1.81 – 1.73 (m, 1H), 1.70 – 1.59 (m, 2H), 1.38 – 1.27 (m, 2H), 1.21 – 1.05 (m, 3H), 0.79 (qd, J = 12.2, 3.4 Hz, 1H).

HPLC (ChiralPak AD-H, 1 mL/min, 0.1% isopropanal in hexane, *λ* 230 nm), retention times of 6.68 min (major) and 11.46 min (minor), 92% ee.

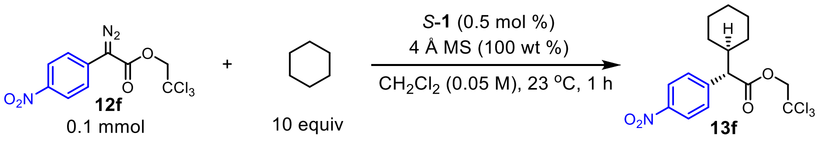

Using ***general procedure F*** with 2,2,2-trichloroethyl 2-diazo-2-(4-nitrophenyl)acetate (**12f**, 0.1 mmol, 33.9 mg), *S*-**1** (1.7 mg, 0.5 mol %) and cyclohexane (10 equiv), 2,2,2-trichloroethyl (*S*)-2-cyclohexyl-2-(4-nitrophenyl)acetate (**13f**) was obtained as a colorless liquid (32.4 mg, 82 % yield) after purification by column chromatography. Spectroscopic data matches with these in the reported literature[10].

^{1}H NMR (400 MHz, $CDCl_3$) δ 8.22 – 8.16 (m, 2H), 7.59 – 7.52 (m, 2H), 4.76 (d, J = 12.0 Hz, 1H), 4.67 (d, J = 12.0 Hz, 1H), 3.53 (d, J = 10.5 Hz, 1H), 2.13 (qt, J = 11.0, 3.3 Hz, 1H), 1.89 (d, J = 12.6 Hz, 1H), 1.82 – 1.73 (m, 1H), 1.65 (dd, J = 8.9, 4.3 Hz, 2H), 1.41 – 1.25 (m, 2H), 1.24 – 1.08 (m, 3H), 0.81 (qd, J = 12.2, 3.4 Hz, 1H).

HPLC (ChiralPak AD-H, 1 mL/min, 1% isopropanal in hexane, *λ* 230 nm), retention times of 10.52 min (major) and 14.54 min (minor), 95% ee.

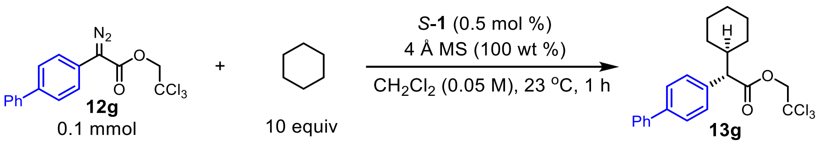

Using ***general procedure F*** with 2,2,2-trichloroethyl 2-([1,1'-biphenyl]-4-yl)-2-diazoacetate (**12g**, 0.1 mmol, 37.0 mg), *S*-**1** (1.7 mg, 0.5 mol %) and cyclohexane (10 equiv), 2,2,2-trichloroethyl (*S*)-2-([1,1'-biphenyl]-4-yl)-2-cyclohexylacetate (**13g**) was obtained as a colorless liquid (35.8 mg, 84 % yield) after purification by column chromatography.

[α]^{20}$_{D}$: +0.8°(c = 0.10, $CHCl_3$, 92% ee); **^{1}H NMR** (400 MHz, $CDCl_3$) δ 7.58 (ddd, J = 11.4, 7.6, 1.6 Hz, 4H), 7.44 (ddd, J = 7.8, 4.4, 2.1 Hz, 4H), 7.37 – 7.32 (m, 1H), 4.81 (d, J = 12.0 Hz, 1H), 4.64 (d, J = 12.0 Hz, 1H), 3.44 (d, J = 10.7 Hz, 1H), 2.15 (qt, J = 11.0, 3.3 Hz, 1H), 1.92 (d, J = 12.6 Hz, 1H), 1.82 – 1.74 (m, 1H), 1.66 (d, J = 8.5 Hz, 2H), 1.49 – 1.41 (m, 1H), 1.40 – 1.29 (m, 1H), 1.25 – 1.09 (m, 3H), 0.89 – 0.77 (m, 1H); **^{13}C NMR** (101 MHz, $CDCl_3$) δ 172.3, 140.8, 140.5, 136.1, 129.3, 128.9, 127.5, 127.4, 127.2, 95.1, 74.3, 58.5, 41.0, 32.1, 30.5, 26.4, 26.1, 26.0; **IR** (neat) 3030, 2925, 2851, 1747, 1486, 1448, 1284, 1142, 1121, 1022, 836, 757, 697 cm^{-1}; **HRMS** (FTMS +p APCI) calcd for $C_{22}H_{23}O_2{}^{35}Cl_3$ $[M]^+$ 424.0758 found 424.0762.

HPLC (Regis (S,S) Whelk-O 1, 1 mL/min, 1% isoproponal in hexane, *λ* 230 nm), retention times of 12.91 min (minor) and 13.86 min (major), 92% ee.

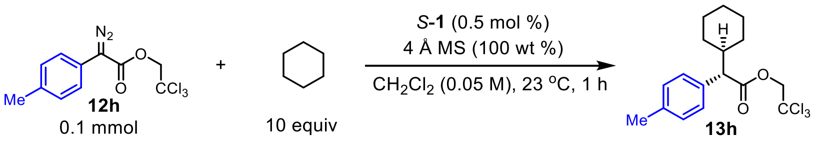

Using ***general procedure F*** with 2,2,2-trichloroethyl 2-diazo-2-(p-tolyl)acetate (**12h**, 0.1 mmol, 30.8 mg), *S*-**1** (1.7 mg, 0.5 mol %) and cyclohexane (10 equiv), 2,2,2-trichloroethyl (*S*)-2-cyclohexyl-2-(*p*-tolyl)acetate (**13h**) was obtained as a colorless liquid (28.8 mg, 79 % yield) after purification by column chromatography.

[α]^{20}$_{D}$: +7.1°(c = 0.63, $CHCl_3$, 86% ee); **^{1}H NMR** (400 MHz, $CDCl_3$) δ 7.27 – 7.22 (m, 2H), 7.13 (d, J = 7.8 Hz, 2H), 4.78 (d, J = 12.0 Hz, 1H), 4.60 (d, J = 12.0 Hz, 1H), 3.34 (d, J = 10.8 Hz, 1H), 2.33 (s, 3H), 2.07 (qt, J = 11.0, 3.3 Hz, 1H), 1.92 – 1.83 (m, 1H), 1.79 – 1.70 (m, 1H), 1.63 (ddd, J = 11.3, 6.1, 3.4 Hz, 2H), 1.41 – 1.34 (m, 1H), 1.34 – 1.25 (m, 1H), 1.19 – 1.04 (m, 3H), 0.77 (qd, J = 12.3, 3.4 Hz, 1H); **^{13}C NMR** (101 MHz, $CDCl_3$) δ 172.5, 137.3, 134.0, 129.4, 128.7, 95.0, 74.2, 58.4, 40.9, 32.1, 30.4, 26.4, 26.1, 26.0, 21.2; **IR** (neat) 2923, 2851, 1749, 1687, 1606, 1513, 1448,

1142, 1121, 1022, 935, 826, 750, 720 cm^{-1}, **HRMS** (FTMS +p ESI) calcd for $C_{17}H_{22}O_2{}^{35}Cl_3$ $[M+H]^+$ 363.0680 found 363.0678 (Δ= -0.57).

HPLC (Regis (S,S) Whelk-O 1, 1 mL/min, 1% isopropanal in hexane, λ 230 nm), retention times of 6.30 min (minor) and 7.84 min (major), 86% ee.

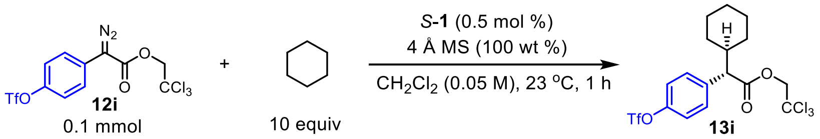

Using ***general procedure F*** with 2,2,2-trichloroethyl 2-diazo-2-(4-(((trifluoromethyl)sulfonyl)oxy)phenyl)acetate (**12i**, 0.1 mmol, 44.2 mg), *S*-**1** (1.7 mg, 0.5 mol %) and cyclohexane (10 equiv), 2,2,2-trichloroethyl (*S*)-2-cyclohexyl-2-(4-(((trifluoromethyl)sulfonyl)oxy)phenyl)acetate (**13i**) was obtained as a white solid (39.8 mg, 80 % yield) after purification by column chromatography.

$[\alpha]^{20}_D$: -3.2°(c = 0.32, $CHCl_3$, 78% ee); **^{1}H NMR** (400 MHz, $CDCl_3$) δ 7.49 – 7.42 (m, 2H), 7.26 – 7.20 (m, 2H), 4.76 (d, J = 12.0 Hz, 1H), 4.65 (d, J = 12.0 Hz, 1H), 3.44 (d, J = 10.6 Hz, 1H), 2.08 (qt, J = 11.0, 3.3 Hz, 1H), 1.88 (d, J = 12.6 Hz, 1H), 1.82 – 1.72 (m, 1H), 1.71 – 1.59 (m, 2H), 1.40 – 1.24 (m, 2H), 1.22 – 1.07 (m, 3H), 0.79 (qd, J = 12.2, 3.2 Hz, 1H); **^{13}C NMR** (101 MHz, $CDCl_3$) δ 171.6, 149.0, 137.6, 130.7, 121.6, 118.85 (q, J = 320.8 Hz), 94.8, 74.3, 58.1, 41.1, 31.9, 30.4, 26.2, 25.94, 25.90; ^{19}F NMR (376 MHz, $CDCl_3$) δ -72.8; **IR** (neat) 2928, 2853, 1750, 1500, 1421, 1250, 1210, 1140, 1018, 888, 844, 722 cm^{-1}; **HRMS** (FTMS +p APCI) calcd for $C_{17}H_{19}O_5{}^{35}Cl_3F_3{}^{32}S$ $[M+H]^+$ 496.9965 found 496.9970.

HPLC (ChiralPak AD-H, 1 mL/min, 1% isopropanal in hexane, λ 230 nm), retention times of 5.56 min (major) and 6.39 min (minor), 78% ee.

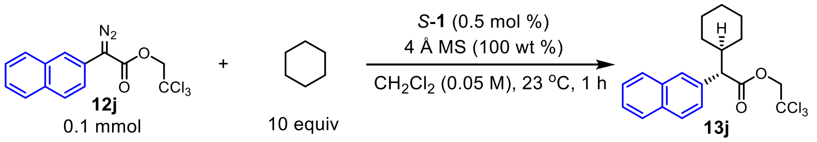

Using ***general procedure F*** with 2,2,2-trichloroethyl 2-diazo-2-(naphthalen-2-yl)acetate (**12j**, 0.1 mmol, 34.4 mg), *S*-**1** (1.7 mg, 0.5 mol %) and cyclohexane (10 equiv), 2,2,2-trichloroethyl (*S*)-2-cyclohexyl-2-(naphthalen-2-yl)acetate (**13j**) was obtained as a white solid (31.6 mg, 79 % yield)

after purification by column chromatography. Spectroscopic data matches with these in the reported literature[10].

^{1}H NMR (400 MHz, $CDCl_3$) δ 7.85 – 7.78 (m, 4H), 7.54 (dd, J = 8.6, 1.4 Hz, 1H), 7.51 – 7.44 (m, 2H), 4.81 (d, J = 12.0 Hz, 1H), 4.62 (d, J = 12.0 Hz, 1H), 3.56 (d, J = 10.8 Hz, 1H), 2.22 (qt, J = 11.0, 3.3 Hz, 1H), 1.95 (d, J = 12.6 Hz, 1H), 1.79 (d, J = 11.3 Hz, 1H), 1.71 – 1.55 (m, 2H), 1.45 – 1.29 (m, 2H), 1.71 – 1.55 (m, 3H), 0.83 (qd, J = 12.2, 3.5 Hz, 1H).

SFC (ChiralCel OJ-3, 2.5 mL/min, 3% (50% methanol in isopropanol with 0.2% Formic Acid), *λ* 230 nm), retention times of 1.87 min (minor) and 2.08 min (major), 90% ee.

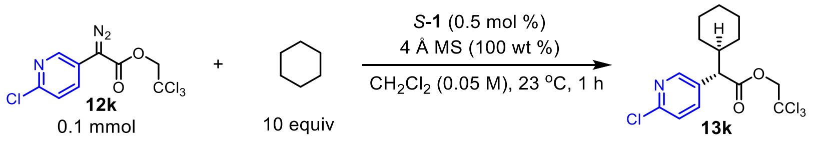

Using ***general procedure F*** with 2,2,2-trichloroethyl 2-(6-chloropyridin-3-yl)-2-diazoacetate (**12k**, 0.1 mmol, 32.9 mg), *S*-**1** (1.7 mg, 0.5 mol %) and cyclohexane (10 equiv), 2,2,2-trichloroethyl (*S*)-2-(6-chloropyridin-3-yl)-2-cyclohexylacetate (**13k**) was obtained as a colorless liquid (29.7 mg, 77 % yield) after purification by column chromatography. Spectroscopic data matches with these in the reported literature[10].

^{1}H NMR (400 MHz, $CDCl_3$) δ 8.32 (d, J = 2.5 Hz, 1H), 7.74 (dd, J = 8.3, 2.5 Hz, 1H), 7.31 (d, J = 8.3 Hz, 1H), 4.76 (d, J = 12.0 Hz, 1H), 4.66 (d, J = 12.0 Hz, 1H), 3.42 (d, J = 10.4 Hz, 1H), 2.05 (qt, J = 11.1, 3.3 Hz, 1H), 1.86 (d, J = 12.6 Hz, 1H), 1.81 – 1.72 (m, 1H), 1.70 – 1.61 (m, 2H), 1.39 – 1.24 (m, 2H), 1.20 – 1.06 (m, 3H), 0.80 (qd, J = 12.0, 2.9 Hz, 1H).

HPLC (Regis (R,R) Whelk-O 1, 0.5 mL/min, 1% isoproponal in hexane, *λ* 230 nm), retention times of 19.37 min (major) and 22.97 min (minor), 94% ee.

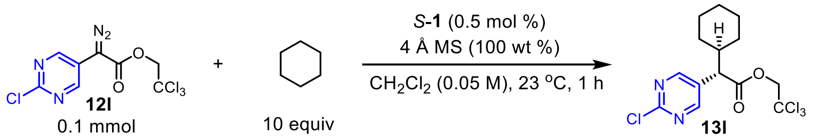

Using ***general procedure F*** with 2,2,2-trichloroethyl 2-(2-chloropyrimidin-5-yl)-2-diazoacetate (**12l**, 0.1 mmol, 33.0 mg), *S*-**1** (1.7 mg, 0.5 mol %) and cyclohexane (10 equiv), 2,2,2-trichloroethyl (*S*)-2-(2-chloropyrimidin-5-yl)-2-cyclohexylacetate (**13l**) was obtained as a white solid (32.0 mg, 83 % yield) after purification by column chromatography.

[α]$^{20}_{D}$: -24.5°(c = 0.75, $CHCl_3$, 65% ee); **^{1}H NMR** (400 MHz, $CDCl_3$) δ 8.64 (s, 2H), 4.78 (d, J = 12.0 Hz, 1H), 4.70 (d, J = 12.0 Hz, 1H), 3.44 (d, J = 9.9 Hz, 1H), 2.08 (dtd, J = 13.2, 8.1, 3.4 Hz, 1H), 1.85 (d, J = 12.7 Hz, 1H), 1.81 – 1.74 (m, 1H), 1.73 – 1.64 (m, 2H), 1.44 – 1.37 (m, 1H), 1.37 – 1.25 (m, 1H), 1.22 – 1.05 (m, 3H), 0.84 (qd, J = 12.2, 3.4 Hz, 1H); **^{13}C NMR** (101 MHz, $CDCl_3$) δ 170.3, 160.9, 159.8, 129.3, 94.4, 74.6, 53.4, 41.2, 31.7, 30.3, 25.9, 25.8, 25.7; **IR** (neat) 2929, 2854, 1750, 1577, 1547, 1449, 1398, 1151, 1122, 1024, 934, 777, 717 cm^{-1}; **HRMS** (FTMS +p APCI) calcd for $C_{14}H_{17}O_2N_2{}^{35}Cl_4$ $[M+H]^+$ 385.0041 found 385.0039.

HPLC (ChiralPak AD-H, 1 mL/min, 1% isopropanal in hexane, *λ* 230 nm), retention times of 23.96 min (major) and 28.12 min (minor), 65% ee.

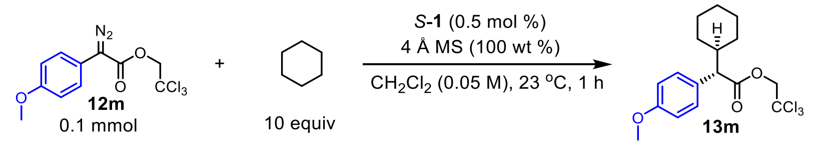

Using ***general procedure F*** with 2,2,2-trichloroethyl 2-diazo-2-(4-methoxyphenyl)acetate (**12m**, 0.1 mmol, 32.4 mg), *S*-**1** (1.7 mg, 0.5 mol %) and cyclohexane (10 equiv), 2,2,2-trichloroethyl (*S*)-2-cyclohexyl-2-(4-methoxyphenyl)acetate (**13m**) was obtained as a colorless liquid (10.3 mg, 27 % yield) after purification by column chromatography.

[α]$^{20}_{D}$: -0.5°(c = 0.10, $CHCl_3$, 61% ee); **^{1}H NMR** (400 MHz, $CDCl_3$) δ 7.30 – 7.24 (m, 2H), 6.88 – 6.82 (m, 2H), 4.76 (d, J = 12.0 Hz, 1H), 4.62 (d, J = 12.0 Hz, 1H), 3.79 (s, 3H), 3.32 (d, J = 10.7 Hz, 1H), 2.05 (qt, J = 11.0, 3.3 Hz, 1H), 1.87 (d, J = 12.7 Hz, 1H), 1.75 (d, J = 11.5 Hz, 1H), 1.69 – 1.60 (m, 2H), 1.44 – 1.36 (m, 1H), 1.34 – 1.28 (m, 1H), 1.20 – 1.05 (m, 3H), 0.77 (qd, J = 12.1, 2.7 Hz, 1H); **^{13}C NMR** (101 MHz, $CDCl_3$) δ 172.6, 159.2, 129.9, 129.2, 114.1, 95.1, 74.2, 58.0, 55.4, 40.9, 32.1, 30.5, 26.4, 26.08, 26.05; **IR** (neat) 2924, 2851, 1748, 1610, 1511, 1448, 1372, 1247, 1177, 1120, 1035, 833, 754 cm^{-1}; **HRMS** (FTMS +p ESI) calcd for $C_{17}H_{22}O_3{}^{35}Cl_3$ $[M+H]^+$ 379.0629 found 379.0628 (Δ= -0.36).

HPLC (Regis (S,S) Whelk-O 1, 1 mL/min, 1% isopropanal in hexane, *λ* 230 nm), retention times of 13.41 min (minor) and 14.50 min (major), 61% ee.

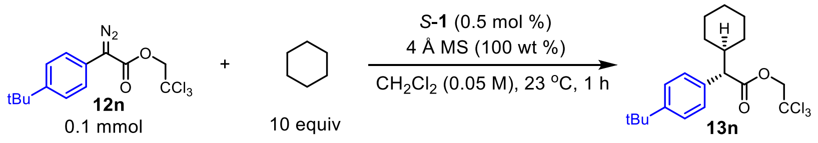

Using ***general procedure F*** with 2,2,2-trichloroethyl 2-(4-(*tert*-butyl)phenyl)-2-diazoacetate (**12n**, 0.1 mmol, 35.0 mg), *S*-**1** (1.7 mg, 0.5 mol %) and cyclohexane (10 equiv), 2,2,2-trichloroethyl (*S*)-2-cyclohexyl-2-(4-(*tert*-butyl)phenyl)acetate (**13n**) was obtained as a colorless liquid (27.2 mg, 67 % yield) after purification by column chromatography. Spectroscopic data matches with these in the reported literature[10].

^{1}H NMR (400 MHz, $CDCl_3$) δ 7.36 – 7.29 (m, 2H), 7.32 – 7.24 (m, 2H), 4.79 (d, J = 12.0 Hz, 1H), 4.58 (d, J = 12.0 Hz, 1H), 3.35 (d, J = 10.8 Hz, 1H), 2.08 (qt, J = 11.0, 3.3 Hz, 1H), 1.88 (d, J = 12.6 Hz, 1H), 1.79 – 1.71 (m, 1H), 1.69 – 1.59 (m, 2H), 1.40 (d, J = 12.9 Hz, 1H), 1.30 (s, 10H), 1.20 – 1.05 (m, 1H).

HPLC (Regis (S,S) Whelk-O 1, 0.5 mL/min, 0.1% isoproponal in hexane, λ 230 nm), retention times of 17.91 min (major) and 21.01 min (minor), 41% ee.

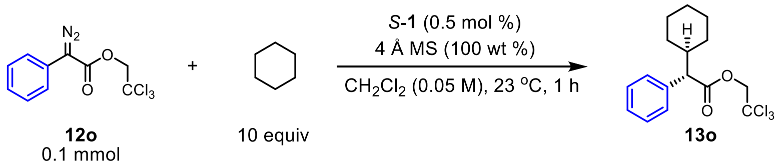

Using ***general procedure F*** with 2,2,2-trichloroethyl 2-diazo-2-phenylacetate (**12o**, 0.1 mmol, 29.4 mg), *S*-**1** (1.7 mg, 0.5 mol %) and cyclohexane (10 equiv), 2,2,2-trichloroethyl (S)-2-cyclohexyl-2-phenylacetate (**13o**) was obtained as a colorless liquid (24.8 mg, 71 % yield) after purification by column chromatography.

$[\alpha]^{20}_D$: -9.8°(c = 2.11, $CHCl_3$, 56% ee); **^{1}H NMR** (400 MHz, $CDCl_3$) δ 7.38 – 7.24 (m, 5H), 4.78 (d, J = 12.0 Hz, 1H), 4.62 (d, J = 12.0 Hz, 1H), 3.38 (d, J = 10.8 Hz, 1H), 2.10 (qt, J = 11.0, 3.3 Hz, 1H), 1.93 – 1.85 (m, 1H), 1.80 – 1.70 (m, 1H), 1.70 – 1.57 (m, 2H), 1.40 – 1.24 (m, 2H), 1.22 – 1.06 (m, 3H), 0.83 – 0.72 (m, 1H); **^{13}C NMR** (101 MHz, $CDCl_3$) δ 172.3, 137.1, 128.9, 128.7, 127.6, 95.0, 74.2, 58.9, 40.9, 32.1, 30.4, 26.4, 26.05, 26.01; **IR** (neat) 3064, 3030, 2924, 2851, 1748, 1449, 1285, 1260, 1219, 1172, 1141, 1120, 1093, 809, 720, 699cm^{-1}; **HRMS** (FTMS -p APCI) calcd for $C_{16}H_{18}{}^{35}Cl_3O_2$ $[M-H]^-$ 347.0378 found 347.0378.

HPLC (Regis (S,S) Whelk-O 1, 1 mL/min, 1% isoproponal in hexane, λ 210 nm), retention times of 6.08 min (minor) and 7.14 min (major), 56% ee.

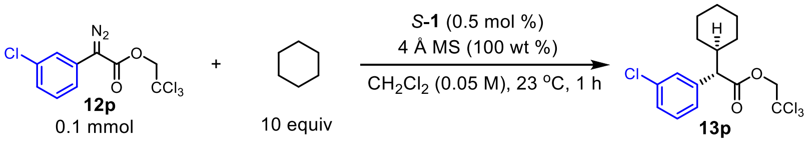

Using ***general procedure F*** with 2,2,2-trichloroethyl 2-(3-chlorophenyl)-2-diazoacetate (**12p**, 0.1 mmol, 37.2 mg), *S*-**1** (1.7 mg, 0.5 mol %) and cyclohexane (10 equiv), 2,2,2-trichloroethyl (*S*)-2-(3-chlorophenyl)-2-cyclohexylacetate (**13p**) was obtained as a colorless liquid (32.3 mg, 84 % yield) after purification by column chromatography.

[α]^{20}$_D$: -4.1°(c = 2.85, $CHCl_3$, 48% ee); **^{1}H NMR** (400 MHz, $CDCl_3$) δ 7.39 (s, 1H), 7.24 (s, 3H), 4.78 (d, J = 12.0 Hz, 1H), 4.63 (d, J = 12.0 Hz, 1H), 3.36 (d, J = 10.6 Hz, 1H), 2.07 (qt, J = 11.0, 3.4 Hz, 1H), 1.92 – 1.83 (m, 1H), 1.80 – 1.72 (m, 1H), 1.71 – 1.59 (m, 2H), 1.42 – 1.25 (m, 2H), 1.22 – 1.05 (m, 3H), 0.86 – 0.74 (m, 1H); **^{13}C NMR** (101 MHz, $CDCl_3$) δ 171.7, 139.1, 134.6, 129.9, 129.0, 127.9, 127.2, 94.9, 74.3, 58.5, 41.0, 32.0, 30.5, 26.3, 26.00, 25.95; **IR** (neat) 2925, 2852, 1749, 1595, 1574, 1476, 1448, 1431, 1371, 1190, 1174, 1144, 1122, 1025, 842, 818, 717, 576 cm^{-1}; **HRMS** (FTMS +p APCI) calcd for $C_{16}H_{19}O_2{}^{35}Cl_4$ $[M+H]^+$ 383.0134 found 383.0136. **HPLC** (Regis (S,S) Whelk-O 1, 0.5 mL/min, 0.5% isoproponal in hexane, λ 230 nm), retention times of 14.14 min (major) and 18.73 min (minor), 48% ee.

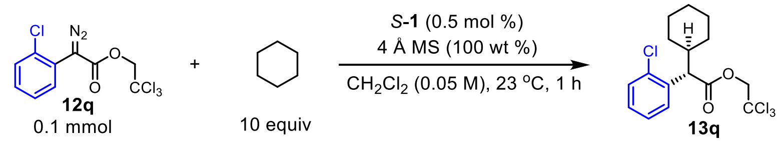

Using ***general procedure F*** with 2,2,2-trichloroethyl 2-(2-chlorophenyl)-2-diazoacetate (**12q**, 0.1 mmol, 37.2 mg), *S*-**1** (1.7 mg, 0.5 mol %) and cyclohexane (10 equiv), 2,2,2-trichloroethyl (*S*)-2-(2-chlorophenyl)-2-cyclohexylacetate (**13q**) was obtained as a colorless liquid (25.7 mg, 67 % yield) after purification by column chromatography. Spectroscopic data matches with these in the reported literature.[10]

^{1}H NMR (400 MHz, $CDCl_3$) δ 7.55 (dd, J = 7.8, 1.7 Hz, 1H), 7.38 (dd, J = 7.9, 1.4 Hz, 1H), 7.25 (td, J = 7.6, 1.3 Hz, 1H), 7.19 (td, J = 7.6, 1.7 Hz, 1H), 4.74 (d, J = 12.0 Hz, 1H), 4.67 (d, J = 12.0 Hz, 1H), 4.18 (d, J = 10.7 Hz, 1H), 2.11 (qt, J = 11.0, 3.3 Hz, 1H), 1.93 (d, J = 12.5 Hz, 1H), 1.81 – 1.73 (m, 1H), 1.70 – 1.59 (m, 2H), 1.38 – 1.25 (m, 2H), 1.23 – 1.09 (m, 3H), 0.91 (qd, J = 12.2, 3.2 Hz, 1H).

HPLC (ChiralCel OD-H, 0.5 mL/min, 0% isoproponal in hexane, λ 230 nm), retention times of 13.17 min (major) and 15.69 min (minor), 60% ee.

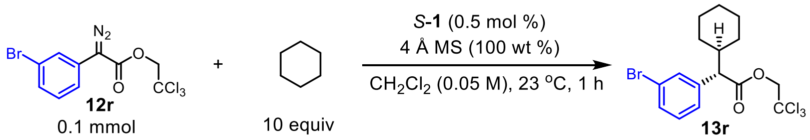

Using ***general procedure F*** with 2,2,2-trichloroethyl 2-(3-bromophenyl)-2-diazoacetate (**12r**, 0.1 mmol, 37.2 mg), *S*-**1** (1.7 mg, 0.5 mol %) and cyclohexane (10 equiv), 2,2,2-trichloroethyl (*S*)-2-(3-bromophenyl)-2-cyclohexylacetate (**13r**) was obtained as a colorless liquid (32.6 mg, 76 % yield) after purification by column chromatography.

[α]^{20}$_D$: -11.2°(c = 2.44, $CHCl_3$, 39% ee); **^{1}H NMR** (400 MHz, $CDCl_3$) δ 7.54 (t, J = 1.8 Hz, 1H), 7.41 (ddd, J = 7.9, 1.9, 1.1 Hz, 1H), 7.31 – 7.27 (m, 1H), 7.19 (t, J = 7.8 Hz, 1H), 4.78 (d, J = 12.0 Hz, 1H), 4.63 (d, J = 12.0 Hz, 1H), 3.34 (d, J = 10.6 Hz, 1H), 2.07 (qt, J = 11.0, 3.4 Hz, 1H), 1.91 – 1.82 (m, 1H), 1.80 – 1.72 (m, 1H), 1.69 – 1.61 (m, 2H), 1.42 – 1.35 (m, 1H), 1.35 – 1.25 (m, 1H), 1.22 – 1.05 (m, 3H), 0.79 (qd, J = 12.1, 3.3 Hz, 1H); **^{13}C NMR** (101 MHz, $CDCl_3$) δ 171.7, 139.3, 131.8, 130.8, 130.2, 127.6, 122.8, 94.9, 74.3, 58.5, 41.0, 32.0, 30.4, 26.3, 26.0, 25.9; **IR** (neat) 2925, 2851, 1749, 1592, 1474, 1448, 1144, 1121, 1074, 818, 722, 575 cm^{-1}; **HRMS** (FTMS +p ESI) calcd for $C_{16}H_{19}O_2{}^{79}Br^{35}Cl_3$ $[M+H]^+$ 426.9629 found 426.9632 (Δ= 0.79).

HPLC (Regis (S,S) Whelk-O 1, 1 mL/min, 1% isoproponal in hexane, λ 230 nm), retention times of 6.29 min (minor) and 8.13 min (major), 39% ee.

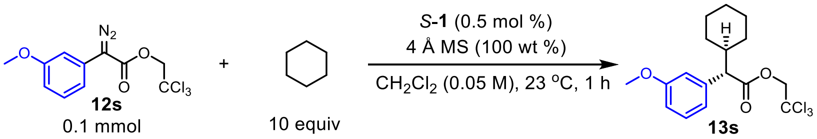

Using ***general procedure F*** with 2,2,2-trichloroethyl 2-diazo-2-(3-methoxyphenyl)acetate (**12s**, 0.1 mmol, 32.4 mg), *S*-**1** (1.7 mg, 0.5 mol %) and cyclohexane (10 equiv), 2,2,2-trichloroethyl (*S*)-2-cyclohexyl-2-(3-methoxyphenyl)acetate (**13s**) was obtained as a colorless liquid (35.7 mg, 94 % yield) after purification by column chromatography.

[α]^{20}$_D$: -17.3°(c = 3.05, $CHCl_3$, 18% ee); **^{1}H NMR** (400 MHz, $CDCl_3$) δ 7.22 (t, J = 8.1 Hz, 1H), 6.94 (dd, J = 7.1, 1.2 Hz, 2H), 6.83 – 6.78 (m, 1H), 4.78 (d, J = 12.0 Hz, 1H), 4.62 (d, J = 12.0 Hz, 1H), 3.80 (s, 3H), 3.35 (d, J = 10.7 Hz, 1H), 2.08 (qt, J = 11.0, 3.3 Hz, 1H), 1.92 – 1.84 (m, 1H),

1.80 – 1.72 (m, 1H), 1.69 – 1.58 (m, 2H), 1.44 – 1.36 (m, 1H), 1.35 – 1.25 (m, 1H), 1.21 – 1.05 (m, 3H), 0.79 (qd, J = 12.3, 3.3 Hz, 1H); **^{13}C NMR** (101 MHz, $CDCl_3$) δ 172.2, 159.9, 138.6, 129.6, 121.4, 114.5, 113.0, 95.1, 74.3, 58.9, 55.4, 40.9, 32.1, 30.5, 26.4, 26.1, 26.0; **IR** (neat) 2924, 2851, 1748, 1599, 1585, 1489, 1449, 1372, 1263, 1142, 1120, 1050, 792, 746, 717, 573 cm^{-1}; **HRMS** (FTMS +p APCI) calcd for $C_{17}H_{21}O_3{}^{35}Cl_3$ (M^+): 378.0551 found 378.0553.

HPLC (Regis (S,S) Whelk-O 1, 1 mL/min, 1% isopropanal in hexane, *λ* 230 nm), retention times of 7.90 min (major) and 11.82 min (minor), 18% ee.

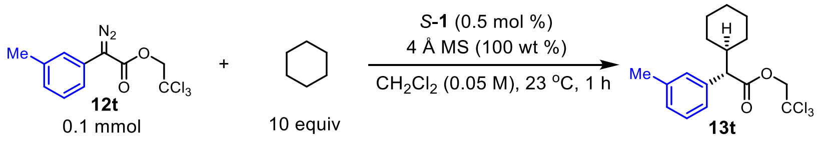

Using ***general procedure F*** with 2,2,2-trichloroethyl 2-diazo-2-(*m*-tolyl)acetate (**12t**, 0.1 mmol, 30.8 mg), *S*-**1** (1.7 mg, 0.5 mol %) and cyclohexane (10 equiv), 2,2,2-trichloroethyl (S)-2-cyclohexyl-2-(*m*-tolyl)acetate (**13t**) was obtained as a colorless liquid (26.6 mg, 73 % yield) after purification by column chromatography.

$[\alpha]^{20}_D$: -10.7°(c = 1.24, $CHCl_3$, 49% ee); **^{1}H NMR** (400 MHz, $CDCl_3$) δ 7.23 – 7.13 (m, 3H), 7.08 (d, J = 7.2 Hz, 1H), 4.80 (d, J = 12.0 Hz, 1H), 4.59 (d, J = 12.0 Hz, 1H), 3.33 (d, J = 10.8 Hz, 1H), 2.34 (s, 3H), 2.09 (qt, J = 11.0, 3.3 Hz, 1H), 1.88 (dd, J = 12.6, 3.5 Hz, 1H), 1.80 – 1.71 (m, 1H), 1.69 – 1.56 (m, 2H), 1.42 – 1.33 (m, 1H), 1.30 (ddt, J = 17.7, 8.3, 4.3 Hz, 1H), 1.20 – 1.05 (m, 3H), 0.77 (qd, J = 12.4, 3.4 Hz, 1H); **^{13}C NMR** (101 MHz, $CDCl_3$) δ 172.4, 138.3, 136.9, 129.5, 128.5, 128.4, 126.0, 95.1, 74.2, 58.8, 40.9, 32.1, 30.5, 26.4, 26.1, 26.0, 21.6; **IR** (neat) 2923, 2851, 1748, 1606, 1488, 1448, 1731, 1284, 1186, 1170, 1119, 1094, 879, 744, 718, 577 cm^{-1}; **HRMS** (FTMS +p APCI) calcd for $C_{17}H_{22}O_2{}^{35}Cl_3$ $[M+H]^+$ 363.0680 found 363.0681.

HPLC (Regis (S,S) Whelk-O 1, 0.5 mL/min, 1% isopropanal in hexane, *λ* 230 nm), retention times of 5.81 min (minor) and 8.06 min (major), 49% ee.

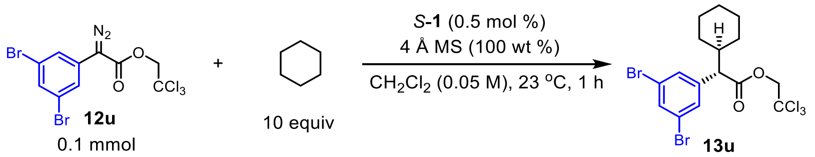

Using ***general procedure F*** with 2,2,2-trichloroethyl 2-(3,5-dibromophenyl)-2-diazoacetate (**12u**, 0.1 mmol, 45.1 mg), *S*-**1** (1.7 mg, 0.5 mol %) and cyclohexane (10 equiv), 2,2,2-trichloroethyl

(*S*)-2-(3,5-dibromophenyl)-2-cyclohexylacetate (**13u**) was obtained as a colorless liquid (39.1 mg, 77 % yield) after purification by column chromatography.

[α]^{20}$_D$: -3.9°(c = 3.05, $CHCl_3$, 11% ee); **^{1}H NMR** (400 MHz, $CDCl_3$) δ 7.58 (t, J = 1.8 Hz, 1H), 7.47 (d, J = 1.7 Hz, 2H), 4.81 (d, J = 12.0 Hz, 1H), 4.62 (d, J = 12.0 Hz, 1H), 3.30 (d, J = 10.6 Hz, 1H), 2.02 (qt, J = 11.1, 3.4 Hz, 1H), 1.89 – 1.80 (m, 1H), 1.80 – 1.71 (m, 1H), 1.70 – 1.61 (m, 2H), 1.42 – 1.34 (m, 1H), 1.33 – 1.23 (m, 1H), 1.22 – 1.04 (m, 3H), 0.85 – 0.74 (m, 1H); **^{13}C NMR** (101 MHz, $CDCl_3$) δ 171.1, 140.8, 133.4, 130.7, 123.1, 94.7, 74.4, 58.3, 41.1, 31.8, 30.4, 26.2, 25.9, 25.8; **IR** (neat) 3076, 2927, 2852, 1749, 1582, 1556, 1447, 1423, 1173, 1144, 1122, 856, 742, 720cm^{-1}; **HRMS** (FTMS +p APCI) calcd for $C_{16}H_{18}O_2{}^{79}Br_2{}^{35}Cl_3$ $[M+H]^+$ 504.8734 found 504.8723.
HPLC (Regis (S,S) Whelk-O 1, 1 mL/min, 1% isoproponal in hexane, λ 230 nm), retention times of 5.96 min (minor) and 8.00 min (major), 11% ee.

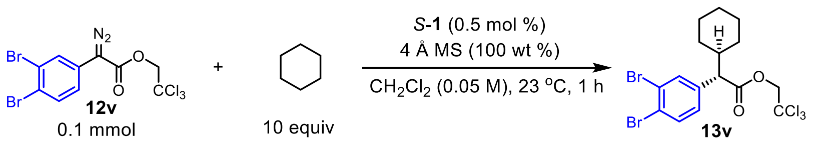

Using ***general procedure F*** with 2,2,2-trichloroethyl 2-diazo-2-(3,4-dibromophenyl)acetate (**12v**, 0.1 mmol, 45.1 mg), *S*-**1** (1.7 mg, 0.5 mol %) and cyclohexane (10 equiv), 2,2,2-trichloroethyl (*S*)-2-cyclohexyl-2-(3,4-dibromophenyl)acetate (**13v**) was obtained as a colorless liquid (40.0 mg, 79 % yield) after purification by column chromatography.

[α]^{20}$_D$: -12.0°(c = 2.27, $CHCl_3$, 91% ee); **^{1}H NMR** (400 MHz, $CDCl_3$) δ 7.65 (d, J = 2.1 Hz, 1H), 7.56 (d, J = 8.3 Hz, 1H), 7.17 (dd, J = 8.3, 2.1 Hz, 1H), 4.78 (d, J = 12.0 Hz, 1H), 4.62 (d, J = 12.0 Hz, 1H), 3.32 (d, J = 10.6 Hz, 1H), 2.03 (qt, J = 11.1, 3.3 Hz, 1H), 1.84 (d, J = 12.5 Hz, 1H), 1.80 – 1.71 (m, 1H), 1.65 (d, J = 9.5 Hz, 2H), 1.41 – 1.33 (m, 1H), 1.33 – 1.24 (m, 1H), 1.21 – 1.03 (m, 3H), 0.84 – 0.73 (m, 1H); **^{13}C NMR** (101 MHz, $CDCl_3$) δ 171.3, 138.0, 133.9, 133.8, 129.1, 125.1, 124.0, 94.8, 74.4, 57.9, 41.0, 31.9, 30.4, 26.2, 25.92, 25.87; **IR** (neat) 2926, 2851, 1747, 1555, 1461, 1448, 1370, 1173, 1117, 1014, 826, 718 cm^{-1}, **HRMS** (FTMS +p APCI) calcd for $C_{16}H_{18}O_2{}^{79}Br_2{}^{35}Cl_3$ $[M+H]^+$ 504.8734 found 504.8737.
HPLC (Regis (S,S) Whelk-O 1, 1 mL/min, 1% isoproponal in hexane, λ 230 nm), retention times of 8.23 min (minor) and 10.15 min (major), 91% ee.

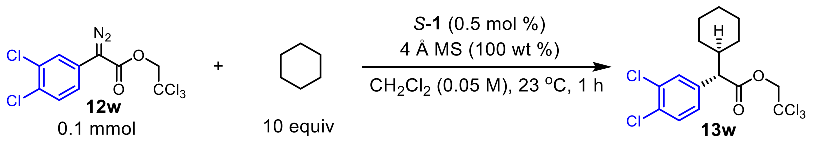

Using ***general procedure F*** with 2,2,2-trichloroethyl 2-(3,4-dichlorophenyl)-2-diazoacetate (**12w**, 0.1 mmol, 36.2 mg), *S*-**1** (1.7 mg, 0.5 mol %) and cyclohexane (10 equiv), 2,2,2-trichloroethyl (*S*)-2-(3,4-dichlorophenyl)-2-cyclohexylacetate (**13w**) was obtained as a colorless liquid (34.3 mg, 82 % yield) after purification by column chromatography.

$[\alpha]^{20}_{D}$: -3.8°(c = 2.71, $CHCl_3$, 91% ee); **^{1}H NMR** (400 MHz, $CDCl_3$) δ 7.49 (d, J = 2.2 Hz, 1H), 7.39 (d, J = 8.2 Hz, 1H), 7.20 (dd, J = 8.3, 2.1 Hz, 1H), 4.78 (d, J = 12.0 Hz, 1H), 4.63 (d, J = 12.0 Hz, 1H), 3.34 (d, J = 10.6 Hz, 1H), 2.03 (qt, J = 11.0, 3.4 Hz, 1H), 1.85 (dt, J = 12.6, 3.5 Hz, 1H), 1.79 – 1.71 (m, 1H), 1.65 (ddt, J = 10.1, 3.7, 1.9 Hz, 2H), 1.41 – 1.24 (m, 2H), 1.21 – 1.04 (m, 3H), 0.84 – 0.73 (m, 1H); **^{13}C NMR** (101 MHz, $CDCl_3$) δ 171.4, 137.2, 132.8, 131.8, 130.7, 130.6, 128.3, 94.8, 74.3, 58.0, 41.1, 31.9, 30.4, 26.2, 25.93, 25.87; **IR** (neat) 2927, 2852, 1749, 1562, 1471, 1449, 1403, 1371, 1330, 1301, 1282, 1252, 1222, 1206, 1174, 1144, 1119, 1032, 943, 918, 883, 828, 794, 717, 575 cm^{-1}; **HRMS** (FTMS -p APCI) calcd for $C_{16}H_{16}{}^{35}Cl_5O_2$ $[M-H]^-$ 414.9598 found 414.9597.

HPLC (Regis (S,S) Whelk-O 1, 1 mL/min, 1% isoproponal in hexane, λ 230 nm), retention times of 7.00 min (minor) and 8.23 min (major), 91% ee.

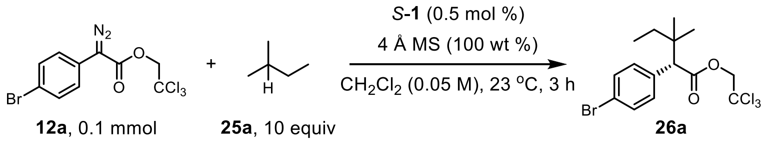

Using ***general procedure F*** with 2,2,2-trichloroethyl 2-(4-bromophenyl)-2-diazoacetate (**12a**, 0.1 mmol, 37.2 mg), *S*-**1** (1.7 mg, 0.5 mol %) and 2-methylbutane (**25a**, 10 equiv), 2,2,2-trichloroethyl (*S*)-2-(4-bromophenyl)-3,3-dimethylpentanoate (**26a**) was obtained as a colorless liquid (31.2 mg, 75 % yield) after purification by column chromatography.

$[\alpha]^{20}_D$: -14.1°(c = 2.74, $CHCl_3$, 88% ee); **^{1}H NMR** (400 MHz, $CDCl_3$) δ 7.47 – 7.42 (m, 2H), 7.32 – 7.28 (m, 2H), 4.83 (d, J = 12.0 Hz, 1H), 4.58 (d, J = 12.0 Hz, 1H), 3.63 (s, 1H), 1.49 – 1.38 (m, 1H), 1.34 – 1.23 (m, 1H), 1.03 (s, 3H), 0.93 – 0.86 (m, 6H); **^{13}C NMR** (101 MHz, $CDCl_3$) δ 171.2, 134.3, 132.0, 131.2, 121.8, 94.9, 74.3, 59.4, 37.6, 33.1, 24.0, 23.9, 8.4; **IR** (neat) 2965, 2880, 1747, 1589, 1488, 1464, 1411, 1369, 1340, 1301, 1257, 1120, 1076, 1062, 1012, 827, 762, 722, 575 cm^{-1}; **HRMS** (FTMS +p APCI) calcd for $C_{15}H_{19}O_2{}^{79}Br^{35}Cl_3$ $[M+H]^+$ 414.9629 found 414.9634.

HPLC (ChiralPak AD-H, 0.5 mL/min, 0.5% isoproponal in hexane, λ 230 nm), retention times of 9.30 min (major) and 10.86 min (minor), 88% ee.

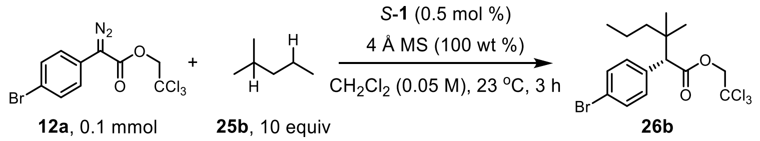

Using ***general procedure F*** with 2,2,2-trichloroethyl 2-(4-bromophenyl)-2-diazoacetate (**12a**, 0.1 mmol, 37.2 mg), *S*-**1** (1.7 mg, 0.5 mol %) and distilled 2-methylpentane (**25b**, 10 equiv), 2,2,2-trichloroethyl (*S*)-2-(4-bromophenyl)-3,3-dimethylhexanoate (**26b**), 2,2,2-trichloroethyl (2*S*,3*R*)-2-(4-bromophenyl)-3,5-dimethylhexanoate (**26b'**) and 2,2,2-trichloroethyl (2*S*,3*S*)-2-(4-bromophenyl)-3,5-dimethylhexanoate (**26b"**) were obtained as a colorless liquid (24.3 mg, 59 % combined yield, **26b/(26b' and 26b") = 9.3/1**, determined by crude ^{1}H NMR) after purification by column chromatography. Spectroscopic data matches with these in the reported literature.[11]

^{1}H NMR (400 MHz, $CDCl_3$) δ 77.44 (d, J = 8.6 Hz, 2H), 7.30 (d, J = 8.5 Hz, 2H), 4.83 (d, J = 12.0 Hz, 1H), 4.58 (d, J = 12.0 Hz, 1H), 3.62 (s, 1H), 1.41 – 1.28 (m, 3H), 1.27 – 1.16 (m, 1H), 1.04 (s, 3H), 0.91 (s, 3H), 0.87 (t, J = 7.1 Hz, 3H);); **^{13}C NMR** (101 MHz, $CDCl_3$) δ 171.3, 134.4, 132.0,

131.2, 121.8, 94.9, 74.3, 59.8, 43.2, 37.5, 24.7, 24.4, 17.2, 14.9. (**reported before: 10.1038/nature24641**)

HPLC (Regis (S,S) Whelk-O 1, 0.1 mL/min, 0.1% isoproponal in hexane, λ 230 nm), retention times of 58.28 min (minor) and 63.25 min (major), 90% ee.

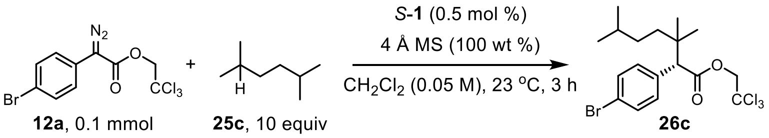

Using ***general procedure F*** with 2,2,2-trichloroethyl 2-(4-bromophenyl)-2-diazoacetate (**12a**, 0.1 mmol, 37.2 mg), *S*-**1** (1.7 mg, 0.5 mol %) and 2,5-dimethylhexane (**25c**, 10 equiv), 2,2,2-trichloroethyl (*S*)-2-(4-bromophenyl)-3,3,6-trimethylheptanoate (**26c**) was obtained as a colorless liquid (33.0 mg, 72 % yield) after purification by column chromatography.

$[\alpha]^{20}_{D}$: -2.3°(c = 2.08, $CHCl_3$, 89% ee); **^{1}H NMR** (400 MHz, $CDCl_3$) δ 7.47 – 7.42 (m, 2H), 7.32 – 7.27 (m, 2H), 4.81 (d, J = 12.0 Hz, 1H), 4.60 (d, J = 12.0 Hz, 1H), 3.63 (s, 1H), 1.48 – 1.31 (m, 2H), 1.30 – 1.12 (m, 3H), 1.04 (s, 3H), 0.91 (s, 3H), 0.86 (d, J = 6.6 Hz, 6H); **^{13}C NMR** (101 MHz, $CDCl_3$) δ 171.3, 134.3, 132.0, 131.2, 121.8, 94.9, 74.3, 59.7, 38.6, 37.3, 32.9, 28.8, 24.8, 24.3, 22.9, 22.8; **IR** (neat) 2954, 2931, 2869, 1747, 1589, 1489, 1468, 1411, 1368, 1341, 1305, 1259, 1217, 1160, 1123, 1076, 1030, 1012, 907, 831, 763, 723, 575 cm^{-1}; **HRMS** (FTMS +p APCI) calcd for $C_{18}H_{23}O_2{}^{79}Br^{35}Cl_3$ $[M+H]^+$ 454.9942 found 454.9947.

HPLC (Regis (S,S) Whelk-O 1, 0.5 mL/min, 0.5% isoproponal in hexane, λ 230 nm), retention times of 11.00 min (minor) and 12.19 min (major), 89% ee.

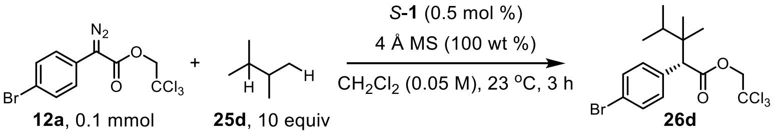

Using ***general procedure F*** with 2,2,2-trichloroethyl 2-(4-bromophenyl)-2-diazoacetate (**12a**, 0.1 mmol, 37.2 mg), *S*-**1** (1.7 mg, 0.5 mol %) and distilled 2,3-dimethylbutane (**25d**, 10 equiv), 2,2,2-trichloroethyl (S)-2-(4-bromophenyl)-3,3,4-trimethylpentanoate (**26d**) was obtained as a colorless liquid (13.8 mg, 32 % yield) after purification by column chromatography. (Note that the racemic sample was prepared from Rh_2(*R/S*-TCPTAD)$_4$)

^{1}H NMR (400 MHz, $CDCl_3$) δ 7.47 – 7.42 (m, 2H), 7.33 – 7.28 (m, 2H), 4.83 (d, J = 12.0 Hz, 1H), 4.56 (d, J = 12.0 Hz, 1H), 3.86 (s, 1H), 1.62 (hept, J = 7.1 Hz, 1H), 1.02 (s, 3H), 0.94 (d, J = 2.1 Hz, 3H), 0.92 (d, J = 2.1 Hz, 3H), 0.79 (s, 3H); **^{13}C NMR** (101 MHz, $CDCl_3$) δ 171.3, 134.5, 132.2, 131.2, 121.8, 94.9, 74.3, 57.5, 40.1, 34.4, 20.7, 20.5, 18.0, 17.5; **IR** (neat) 2964, 1747, 1589, 1489, 1411, 1396, 1371, 1333, 1306, 1251, 1172, 1146, 1113, 1076, 1062, 1029, 1011, 926, 903, 822, 762, 719, 575 cm^{-1}; **HRMS** (FTMS +p APCI) calcd for $C_{16}H_{21}O_2{}^{79}Br^{35}Cl_3$ $[M+H]^+$ 428.9785 found 428.9790 (Δ = 1.23 ppm).

UPLC (ChiralPak AD-H, 0.5 mL/min, 0.01% isoproponal in hexane, *λ* 230 nm), retention times of 10.61 min (major) and 11.48 min (minor), 92% ee.

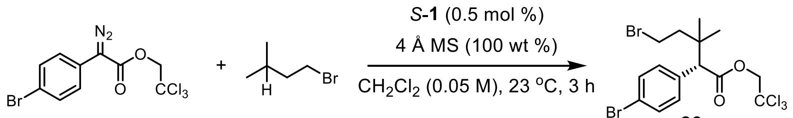

12a, 0.1 mmol, 1 equiv
0.2 mmol, 2 equiv

25e, 10 equiv
1 equiv

26e

Using ***general procedure F*** with 2,2,2-trichloroethyl 2-(4-bromophenyl)-2-diazoacetate (**12a**, 0.1 mmol, 37.2 mg), *S*-**1** (1.7 mg, 0.5 mol %) and 1-bromo-3-methylbutane (**25e**, 10 equiv), 2,2,2-trichloroethyl (*S*)-5-bromo-2-(4-bromophenyl)-3,3-dimethylpentanoate (**26e**) was obtained as a white solid (12.9 mg, 26 % yield) after purification by column chromatography.

Using ***general procedure G*** with 2,2,2-trichloroethyl 2-(4-bromophenyl)-2-diazoacetate (**12a**, 0.2 mmol, 2 equiv, 74.4 mg), *S*-**1** (1.7 mg, 0.5 mol %) and 1-bromo-3-methylbutane (**25e**, 0.1 mmol, 1 equiv), 2,2,2-trichloroethyl (*S*)-5-bromo-2-(4-bromophenyl)-3,3-dimethylpentanoate (**26e**) was obtained as a white solid (28.8 mg, 58 % yield) after purification by column chromatography.

^{1}H NMR (800 MHz, $CDCl_3$) δ 7.47 (d, J = 8.6 Hz, 2H), 7.28 (d, J = 8.5 Hz, 2H), 4.85 (d, J = 11.9 Hz, 1H), 4.59 (d, J = 12.1 Hz, 1H), 3.59 (s, 1H), 3.45 – 3.35 (m, 2H), 2.09 (ddd, J = 13.7, 11.9, 5.5 Hz, 1H), 1.89 (ddd, J = 13.8, 11.8, 5.3 Hz, 1H), 1.08 (s, 3H), 0.99 (s, 3H); **^{13}C NMR** (201 MHz, $CDCl_3$) δ 169.6, 132.4, 130.9, 130.5, 121.3, 93.7, 73.4, 58.9, 42.9, 37.6, 27.2, 23.4, 23.3; **IR** (neat) X cm^{-1}; **HRMS** (FTMS +p APCI) calcd for $C_{15}H_{18}O_2{}^{79}Br_2{}^{35}Cl_3$ $[M+H]^+$ 492.8734 found 492.8731.

SFC (Regis (S,S) Whelk-O 1, 2.5 mL/min, 2% (50% methanol in isopropanol with 0.2% Formic Acid)), retention times of 2.96 min (minor) and 3.17 min (major), 85% ee.

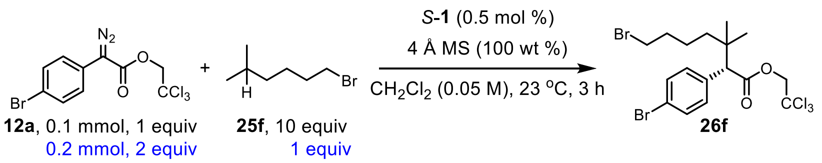

Using ***general procedure F*** with 2,2,2-trichloroethyl 2-(4-bromophenyl)-2-diazoacetate (**12a**, 0.1 mmol, 37.2 mg), *S*-**1** (1.7 mg, 0.5 mol %) and 1-bromo-5-methylhexane (**25f**, 10 equiv), 2,2,2-trichloroethyl (*S*)-7-bromo-2-(4-bromophenyl)-3,3-dimethylheptanoate (**26f**) was obtained as a colorless liquid (17.3 mg, 33 % yield) after purification by column chromatography.

Using ***general procedure G*** with 2,2,2-trichloroethyl 2-(4-bromophenyl)-2-diazoacetate (**12a**, 0.2 mmol, 2 equiv, 74.4 mg), *S*-**1** (1.7 mg, 0.5 mol %) and 1-bromo-5-methylhexane (**25f**, 0.1 mmol, 1 equiv), 2,2,2-trichloroethyl (*S*)-7-bromo-2-(4-bromophenyl)-3,3-dimethylheptanoate (**26f**) was obtained as a colorless liquid (37.7 mg, 72 % yield) after purification by column chromatography. **$[\alpha]^{20}_D$**: +2.8°(c = 0.10, $CHCl_3$, 86% ee); **^{1}H NMR** (400 MHz, $CDCl_3$) δ 77.45 (d, J = 8.5 Hz, 2H), 7.30 (d, J = 8.5 Hz, 2H), 4.84 (d, J = 12.0 Hz, 1H), 4.59 (d, J = 12.0 Hz, 1H), 3.63 (s, 1H), 3.40 (t, J = 6.7 Hz, 2H), 1.80 (pd, J = 6.9, 3.9 Hz, 2H), 1.53 – 1.38 (m, 3H), 1.25 – 1.19 (m, 1H), 1.05 (s, 3H), 0.94 (s, 3H); **^{13}C NMR** (101 MHz, $CDCl_3$) δ 171.1, 134.1, 131.9, 131.3, 121.9, 94.9, 74.3, 59.7, 39.7, 37.4, 33.9, 33.4, 24.7, 24.4, 22.6; **IR** (neat) 2945, 1746, 1589, 1488, 1473, 1411, 1370, 1342, 1258, 1205, 1121, 1075, 1034, 1011, 905, 829, 762, 720, 649, 547 cm^{-1}; **HRMS** (FTMS +p APCI) calcd for $C_{17}H_{22}O_2{}^{79}Br_2{}^{35}Cl_3$ $[M+H]^+$ 520.9047 found 520.9055.

HPLC (Regis (S,S) Whelk-O 1, 0.5 mL/min, 0.5% isoproponal in hexane, λ 230 nm), retention times of 16.98 min (minor) and 19.28 min (major), 86% ee.

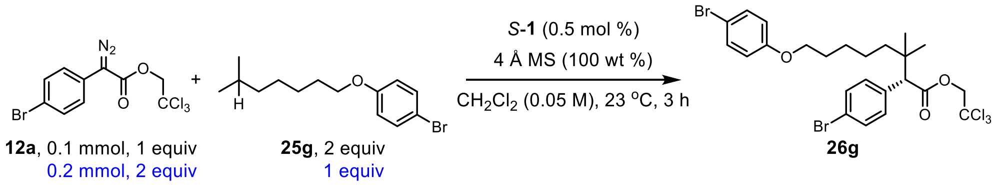

Using ***general procedure F*** with 2,2,2-trichloroethyl 2-(4-bromophenyl)-2-diazoacetate (**12a**, 0.1 mmol, 37.2 mg), *S*-**1** (1.7 mg, 0.5 mol %) and 1-bromo-4-((6-methylheptyl)oxy)benzene (**25g**, 2 equiv), 2,2,2-trichloroethyl (*S*)-8-(4-bromophenoxy)-2-(4-bromophenyl)-3,3-dimethyloctanoate (**26g**) was obtained as a colorless liquid in 40% yield (25.2 mg) after purification by column chromatography.

Using ***general procedure G*** with 2,2,2-trichloroethyl 2-(4-bromophenyl)-2-diazoacetate (**12a**, 0.2 mmol, 2 equiv, 74.4 mg), *S*-**1** (1.7 mg, 0.5 mol %) and 1-bromo-4-((6-methylheptyl)oxy)benzene (**25g**, 1 equiv, 0.1 mmol), 2,2,2-trichloroethyl (*S*)-8-(4-bromophenoxy)-2-(4-bromophenyl)-3,3-dimethyloctanoate (**26g**) was obtained as a colorless liquid in 66% yield (41.6 mg) after purification by column chromatography. (Note that the racemic sample was prepared from Rh_2(*R/S*-TCPTAD)$_4$)

$[\alpha]^{20}_D$: -5.3°(c = 1.93, $CHCl_3$, 92% ee); **^{1}H NMR** (400 MHz, $CDCl_3$) δ 7.44 (d, J = 8.7 Hz, 2H), 7.36 (d, J = 9.0 Hz, 2H), 7.29 (d, J = 8.6 Hz, 2H), 6.76 (d, J = 9.0 Hz, 2H), 4.84 (d, J = 12.0 Hz, 1H), 4.57 (d, J = 12.1 Hz, 1H), 3.90 (t, J = 6.4 Hz, 2H), 3.62 (s, 1H), 1.76 (p, J = 6.3 Hz, 2H), 1.49 – 1.31 (m, 6H), 1.04 (s, 3H), 0.92 (s, 3H); **^{13}C NMR** (101 MHz, $CDCl_3$) δ 171.2, 158.3, 134.2, 132.4, 132.0, 131.2, 121.9, 116.4, 112.8, 94.9, 74.3, 68.2, 59.8, 40.7, 37.4, 29.3, 26.9, 24.7, 24.4, 23.8; **IR** (neat) 2931, 2858, 1748, 1590, 1488, 1410, 1370, 1284, 1242, 1170, 1121, 1073, 1011, 822, 762, 720, 641, 574, 507 cm^{-1}; **HRMS** (FTMS +p ESI) calcd for $C_{24}H_{28}O_3{}^{79}Br_2{}^{35}Cl_3$ $[M+H]^+$ 626.9465 found 626.9463 (Δ= -0.41).

HPLC (Regis (S,S) Whelk-O 1, 1 mL/min, 1% isoproponal in hexane, λ 230 nm), retention times of 11.13 min (minor) and 12.37 min (major), 92% ee.

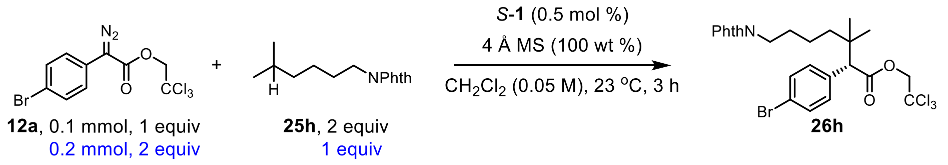

Using ***general procedure F*** with 2,2,2-trichloroethyl 2-(4-bromophenyl)-2-diazoacetate (**12a**, 0.1 mmol, 37.2 mg), *S*-**1** (1.7 mg, 0.5 mol %) and 2-(5-methylhexyl)isoindoline-1,3-dione (**25h**, 2 equiv), 2,2,2-trichloroethyl (*S*)-2-(4-bromophenyl)-7-(1,3-dioxoisoindolin-2-yl)-3,3-dimethylheptanoate (**26h**) was obtained as a white solid in 43% yield (25.4 mg) after purification by column chromatography.

Using ***general procedure G*** with 2,2,2-trichloroethyl 2-(4-bromophenyl)-2-diazoacetate (**12a**, 0.2 mmol, 2 equiv, 74.4 mg), *S*-**1** (1.7 mg, 0.5 mol %) and 2-(5-methylhexyl)isoindoline-1,3-dione (**25h**, 0.1 mmol, 1 equiv), 2,2,2-trichloroethyl (*S*)-2-(4-bromophenyl)-7-(1,3-dioxoisoindolin-2-yl)-3,3-dimethylheptanoate (**26h**) was obtained as a white solid in 73% yield (43.1 mg) after purification by column chromatography. (Note that the racemic sample was prepared from Rh_2(*R/S*-TCPTAD)$_4$)

[α]^{20}$_{D}$: +6.5°(c = 2.31, $CHCl_3$, 92% ee); **^{1}H NMR** (400 MHz, $CDCl_3$) δ 7.84 (dd, J = 5.4, 3.1 Hz, 2H), 7.71 (dd, J = 5.4, 3.1 Hz, 2H), 7.42 (d, J = 8.5 Hz, 2H), 7.28 (d, J = 8.6 Hz, 2H), 4.82 (d, J = 12.0 Hz, 1H), 4.56 (d, J = 12.0 Hz, 1H), 3.66 (t, J = 7.3 Hz, 2H), 3.60 (s, 1H), 1.62 (dtd, J = 7.6, 6.3, 4.3 Hz, 2H), 1.50 – 1.33 (m, 3H), 1.32 – 1.26 (m, 1H), 1.03 (s, 3H), 0.90 (s, 3H); **^{13}C NMR** (101 MHz, $CDCl_3$) δ 171.1, 168.5, 134.2, 134.0, 132.3, 131.9, 131.2, 123.3, 121.9, 94.9, 74.3, 59.8, 40.3, 38.1, 37.4, 29.5, 24.7, 24.3, 21.4; **IR** (neat) 2938, 1771, 1747, 1708, 1615, 1488, 1467, 1436, 1395, 1368, 1267, 1188, 1121, 1075, 1040, 1011, 904, 830, 762, 719, 626, 574, 530 cm^{-1}; **HRMS** (FTMS +p ESI) calcd for $C_{25}H_{26}O_4N^{79}Br^{35}Cl_3$ $[M+H]^+$ 588.0105 found 588.0111 (Δ= 0.88).
SFC (ChiralCel OJ-3, 2.5 mL/min, 2% (50% methanol in isopropanol with 0.2% Formic Acid)), retention times of 7.49 min (major) and 8.41 min (minor), 92% ee.

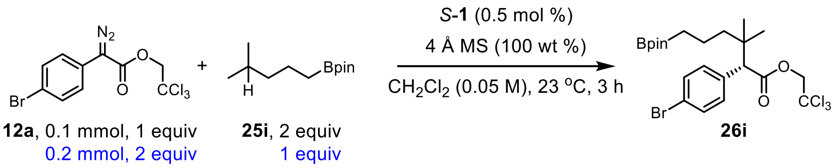

Using ***general procedure F*** with 2,2,2-trichloroethyl 2-(4-bromophenyl)-2-diazoacetate (**12a**, 0.1 mmol, 37.2 mg), *S*-**1** (1.7 mg, 0.5 mol %) and 4,4,5,5-tetramethyl-2-(4-methylpentyl)-1,3,2-dioxaborolane (**25i**, 2.0 equiv), ((dichloro-λ^3-methyl)-λ^2-chloraneyl)methyl (S)-2-(4-bromophenyl)-3,3-dimethyl-6-(4,4,5,5-tetramethyl-1,3,2-dioxaborolan-2-yl)hexanoate (**26i**) was obtained as a colorless liquid in 37% yield (20.6 mg) after purification by column chromatography.
Using ***general procedure G*** with 2,2,2-trichloroethyl 2-(4-bromophenyl)-2-diazoacetate (**12a**, 0.2 mmol, 2 equiv, 74.4 mg), *S*-**1** (1.7 mg, 0.5 mol %) and 4,4,5,5-tetramethyl-2-(4-methylpentyl)-1,3,2-dioxaborolane (**25i**, 0.1 mmol, 1 equiv), ((dichloro-λ^3-methyl)-λ^2-chloraneyl)methyl (S)-2-(4-bromophenyl)-3,3-dimethyl-6-(4,4,5,5-tetramethyl-1,3,2-dioxaborolan-2-yl)hexanoate (**26i**) was obtained as a colorless liquid in 43% yield (23.9 mg) after purification by column chromatography.
(Note that the racemic sample was prepared from ***R/S*-1**.
[α]^{20}$_{D}$: +3.4°(c = 0.56, $CHCl_3$, 95% ee); **^{1}H NMR** (600 MHz, $CDCl_3$) δ 7.43 (d, J = 8.5 Hz, 2H), 7.29 (d, J = 8.5 Hz, 2H), 4.82 (d, J = 12.0 Hz, 1H), 4.57 (d, J = 12.0 Hz, 1H), 3.62 (s, 1H), 1.48 – 1.40 (m, 2H), 1.40 – 1.32 (m, 1H), 1.24 – 1.18 (m, 13H), 1.03 (s, 3H), 0.92 (s, 3H), 0.71 (t, J = 7.5 Hz, 1H); **^{13}C NMR** (101 MHz, $CDCl_3$) δ 171.2, 134.4, 132.0, 131.1, 121.7, 94.9, 83.1, 74.3, 59.7, 43.8, 37.6, 25.0, 24.9, 24.6, 24.4, 18.4; **^{11}B NMR** (128 MHz, $CDCl_3$) δ 34.46; **IR** (neat) 2975,

2936, 2873, 1748, 1488, 1411, 1371, 1322, 1253, 1125, 1075, 1011, 968, 831, 763, 719 cm^{-1}; **HRMS** (FTMS +p ESI) calcd for $C_{22}H_{31}O_4{}^{10}B^{79}Br^{35}Cl_3{}^{23}Na$ $[M+Na]^+$ 576.0493 found 576.0500 (Δ= 1.2).

HPLC (ChiralPak AD-H, 1 mL/min, 0.5% isopropanal in hexane, *λ* 230 nm), retention times of 5.33 min (major) and 6.19 min (minor), 95% ee.

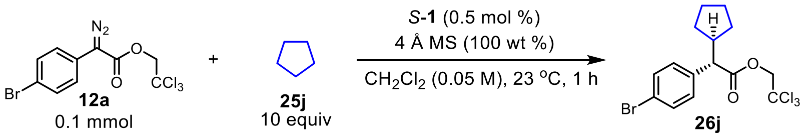

Using ***general procedure F*** with 2,2,2-trichloroethyl 2-(4-bromophenyl)-2-diazoacetate (**12a**, 0.1 mmol, 37.2 mg), *S*-**1** (1.7 mg, 0.5 mol %) and cyclopentane (**25j**, 10 equiv), 2,2,2-trichloroethyl (*S*)-2-(4-bromophenyl)-2-cyclopentylacetate (**26j**) was obtained as a colorless liquid (37.7 mg, 91 % yield) after purification by column chromatography. Spectroscopic data matches with these in the reported literature[8].

^{1}H NMR (400 MHz, $CDCl_3$) δ 7.47 – 7.42 (m, 2H), 7.28 – 7.23 (m, 2H), 4.76 (d, J = 12.0 Hz, 1H), 4.65 (d, J = 12.0 Hz, 1H), 3.39 (d, J = 11.1 Hz, 1H), 2.60 (ddt, J = 15.9, 11.1, 8.0 Hz, 1H), 1.96 (dtd, J = 12.2, 7.6, 4.6 Hz, 1H), 1.75 – 1.55 (m, 3H), 1.49 (dtd, J = 15.4, 8.8, 2.7 Hz, 2H), 1.32 (dq, J = 12.7, 8.3 Hz, 1H), 1.09 – 0.95 (m, 1H).

HPLC (ChiralPak AD-H, 1 mL/min, 0.5% isopropanal in hexane, *λ* 230 nm), retention times of 6.49 min (major) and 7.40 min (minor), 98% ee.

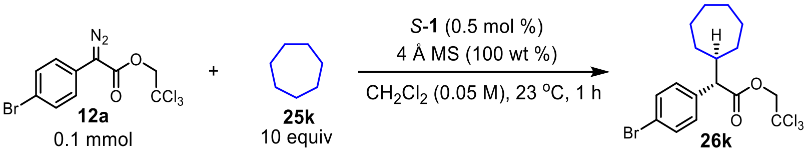

Using ***general procedure F*** with 2,2,2-trichloroethyl 2-(4-bromophenyl)-2-diazoacetate (**12a**, 0.1 mmol, 37.2 mg), *S*-**1** (1.7 mg, 0.5 mol %) and cycloheptane (**25k**, 10 equiv), 2,2,2-trichloroethyl (*S*)-2-(4-bromophenyl)-2-cycloheptylacetate (**26k**) was obtained as a colorless liquid (35.4 mg, 80 % yield) after purification by column chromatography. Spectroscopic data matches with these in the reported literature[8].

^{1}H NMR (400 MHz, $CDCl_3$) δ 7.47 – 7.41 (m, 2H), 7.27 – 7.22 (m, 2H), 4.73 (d, J = 12.0 Hz, 1H), 4.64 (d, J = 12.0 Hz, 1H), 3.43 (d, J = 10.9 Hz, 1H), 2.36 – 2.24 (m, 1H), 1.83 (ddd, J = 13.0, 6.4,

3.3 Hz, 1H), 1.70 (ddt, J = 13.5, 9.5, 5.1 Hz, 1H), 1.58 – 1.46 (m, 5H), 1.45 – 1.25 (m, 4H), 1.07 – 0.96 (m, 1H).

HPLC (Regis (S,S) Whelk-O 1, 1 mL/min, 1% isopropanal in hexane, λ 230 nm), retention times of 7.98 min (minor) and 8.93 min (major), 96% ee.

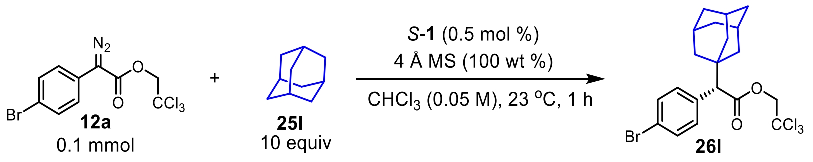

Using ***general procedure F*** with 2,2,2-trichloroethyl 2-(4-bromophenyl)-2-diazoacetate (**12a**, 0.1 mmol, 37.2 mg), *S*-**1** (1.7 mg, 0.5 mol %) and adamantane (**25l**, 10 equiv) in **Chloroform** (0.05 M), 2,2,2-trichloroethyl (S)-2-((3*S*,5*S*,7*S*)-adamantan-1-yl)-2-(4-bromophenyl)acetate (**26l**) was obtained as a colorless liquid (25.7 mg, 71 % yield) after purification by column chromatography. Spectroscopic data matches with these in the reported literature[8].

^{1}H NMR (400 MHz, $CDCl_3$) δ 7.47 – 7.41 (m, 2H), 7.31 – 7.26 (m, 2H), 4.80 (d, J = 12.0 Hz, 1H), 4.62 (d, J = 12.0 Hz, 1H), 3.39 (s, 1H), 1.97 (p, J = 3.3 Hz, 3H), 1.75 – 1.63 (m, 6H), 1.61 – 1.52 (m, 6H).

HPLC (Regis (S,S) Whelk-O 1, 1 mL/min, 1% isopropanal in hexane, λ 230 nm), retention times of 6.30 min (minor) and 6.98 min (major), 96% ee.

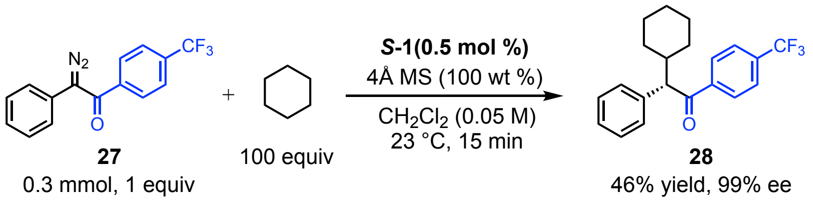

Using the modified reported procedure[21] with 2-diazo-2-phenyl-1-(4-(trifluoromethyl)-phenyl)ethan-1-one (**27**, 1 equiv, 0.3 mmol, 87.0 mg) and cyclohexane (100 equiv, 1.0 mL) in a flame-dried 4 mL vial under nitrogen atmosphere, ***S*-1** (5.1 mg, 0.5 mol %) in 1 mL dry CH_2Cl_2 was added to the reaction vial in one portion and the reaction was stirred at room temperature for another 15 mins. (*S*)-2-cyclohexyl-2-phenyl-1-(4-(trifluoromethyl)phenyl)ethan-1-one (**28**) was obtained as a colorless liquid (47.8 mg, 46 % yield) after purification by column chromatography. NMR spectra matched with reported data[21].

^{1}H NMR (400 MHz, $CDCl_3$) δ 8.05 (d, J = 8.1 Hz, 2H), 7.66 (d, J = 8.2 Hz, 2H), 7.33 – 7.26 (m, 4H), 7.25 – 7.18 (m, 1H), 4.27 (d, J = 10.1 Hz, 1H), 2.30 (dtd, J = 14.4, 11.1, 3.3 Hz, 1H), 1.83 (dt, J = 12.5, 3.1 Hz, 1H), 1.73 – 1.59 (m, 3H), 1.40 – 1.27 (m, 2H), 1.22 – 1.08 (m, 2H), 0.97 (tdd, J = 12.5, 11.0, 3.4 Hz, 1H), 0.85 (qd, J = 11.9, 3.0 Hz, 1H).

HPLC (Regis (S, S) Whelk-O 1, 0.5 mL/min, 0.5% isoproponal in hexane, λ 230 nm), retention times of 12.7 min (major) and 16.3 min (minor), 99% ee.

5. High Turnover Number Study

Procedure for High Turnover Number Study: To a flame dried 4 mL vial with 4 Å Molecular Sieves (100 wt %) and a magnetic stir bar was added 1 mL distilled cyclohexane and the vial was sealed with a septum. *S*-**1** from a stock solution in dichloromethane (0.0025 mol %) and 1 mol % diisopropylcarbodiimide (DIC) in dichloromethane were added to the vial syringe. The mixture was stirred and heated at 60 °C for 2 mins before the slow addition of 2,2,2-trichloroethyl 2-(4-(trifluoromethyl)phenyl)-2-diazoacetate (36.2 mg, 0.1 mmol, 1 equiv) in 1 mL distilled cyclohexane with 2 drops of dry dichloromethane via syringe over 3 hours, which was rinsed with dry dichloromethane and injected into the vial. The reaction was kept at 60 °C and monitored by TLC. After full consumption of diazo compound, the reaction mixture was transferred into a 20 mL vial and the solvent was removed to afford the crude material for NMR analysis (73.5% NMR yield), then the crude material was purified by silica gel chromatography to afford the purified product (28.4 mg, 68% yield) for determination of yield and HPLC analysis.

6. VT-NMR Study for *S*-1 and ^{1}H, NOESY NMR study for *S*- 17b and *S*- 1

The Variable-temperature ^{1}H NMR was used for estimation of the rotation barriers that was observed for the catalyst *S*-**1**. The data were obtained using **Varian INOVA-600 MHz** spectrometer and analyzed using **MestRenova 15.0.1**

Solvent: **Toluene-*d*$_8$**

Number of scans: **16**

Temperature measurement: Temperature of the probe

Spectra were taken after the samples spin for **15 mins** under the corresponding temperature.

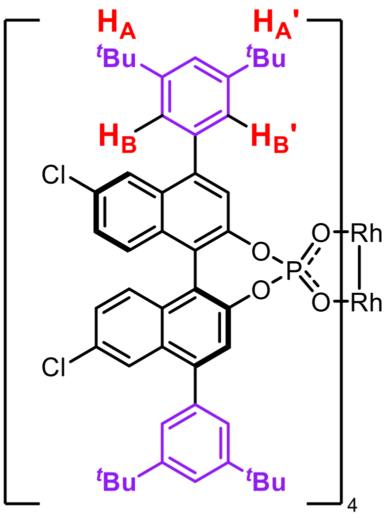

S-**1**

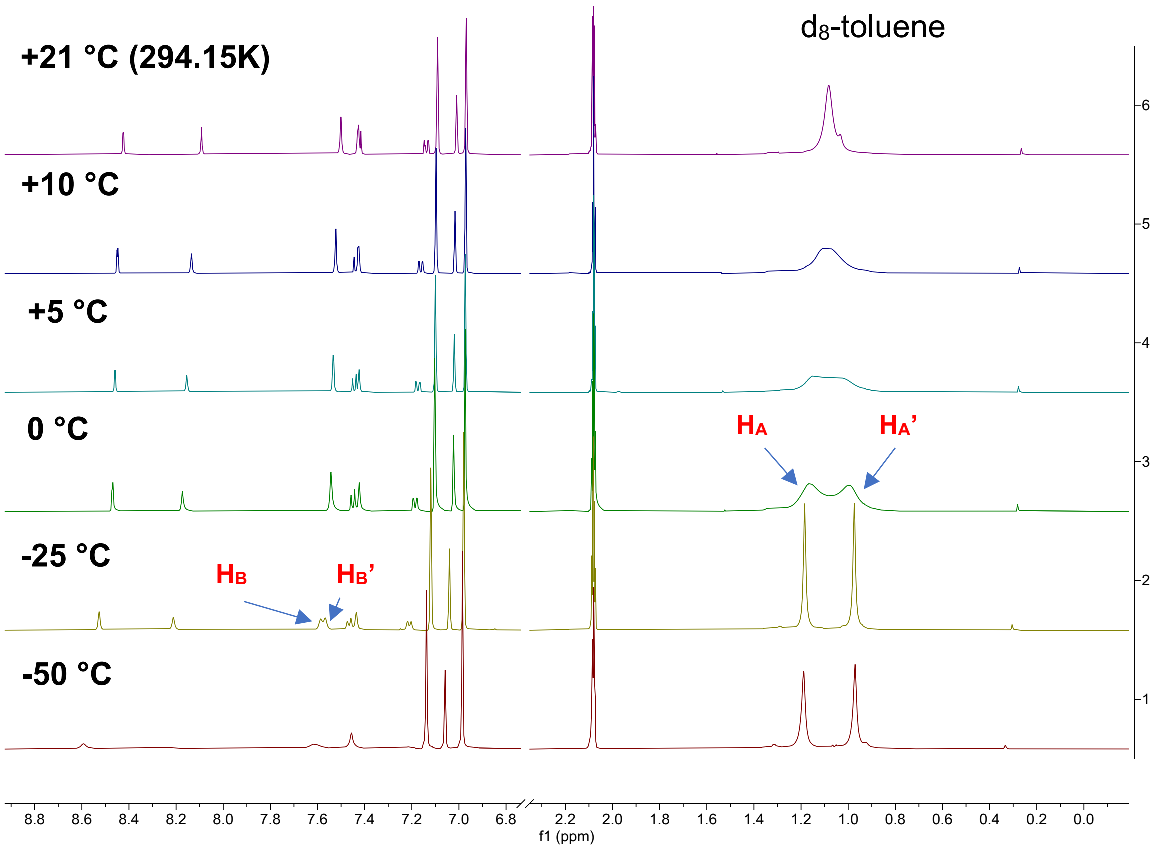

Figure S6.1. ^{1}H-NMR spectra of *S*-**1** (range δ 8.8-0 ppm, -50 °C-+21°C, range δ 6.8-2.2 ppm was cut off for better clarity and there is no noticeable signal within this region)

Calculations:

The proton H_A and H_B are isolated systems, so the crude estimation of rate constants, *k*, at coalescence were obtained using approximate formula.[12, 13]

$$k = \frac{\pi \Delta \upsilon}{\sqrt{2}}$$

Table S6.1. Parameters from VT-NMR spectra and calculated *k* values.

Compound	$\Delta\upsilon$ (H_A), Hz	T_c (H_A), K	*k* (H_A), s^{-1}	-	$\Delta\upsilon$ (H_B), Hz	T_c (H_B), K	*k* (H_B), s^{-1}
S-(1)	130.75	278.15	290.45	-	11.58	248.15	25.72

Eyring plots, ln(*k*/T) vs 1/T, were then plotted using two data points (H_A and H_B at coalescence) for compound *S*-**1**.

$$\ln\left(\frac{k}{\mathrm{T}}\right) = -\frac{\Delta H^{\ddagger}}{R} \bullet \frac{1}{\mathrm{T}} + \frac{\Delta S^{\ddagger}}{R} + \ln\left(\frac{k_B}{h}\right)$$

$$\Delta G^{\ddagger} = \Delta H^{\ddagger} - T\Delta S^{\ddagger}$$

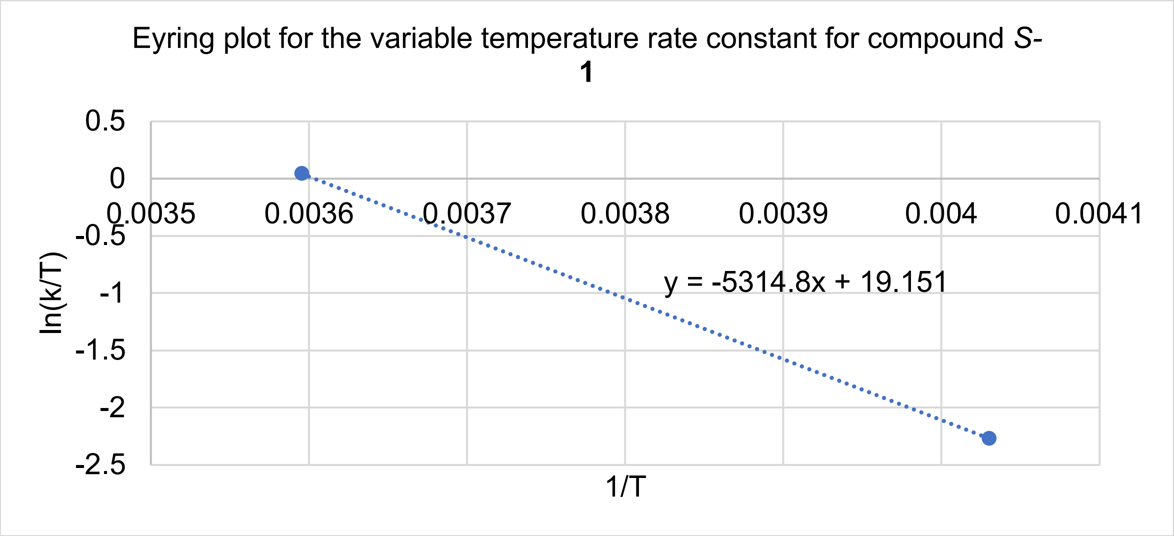

Figure S6.2. Eyring plots, ln(*k*/T) vs 1/T, of compound *S*-**1**

Table S6.2. Calculated energies of compound *S*-**1**

Compound	$\Delta H^{\ddagger}$, (kcal/mol)	$\Delta S^{\ddagger}$, (kcal/mol•K)	$\Delta G^{\ddagger}$, (kcal/mol, at 273.15 K)
S-**1**	10.56	-0.009	13.06

The low temperature ^{1}H and NOESY NMR was used for understanding the possible slow rotation that was observed for the catalyst *S*-**1**. The data were obtained using Bruker-800 MHz spectrometer and analyzed using MestRenova 15.0.1

Solvent: Toluene-d_8 or $CDCl_3$ (The usage of Toluene-d_8 is to deconvolute aromatic signals. When using Toluene-d_8, NOESY spectra data was obtained at 21 °C, which is the same temperature for NMR analysis for ligand *S*-**17b**)

Number of scans: 4

Temperature measurement: Temperature of the probe

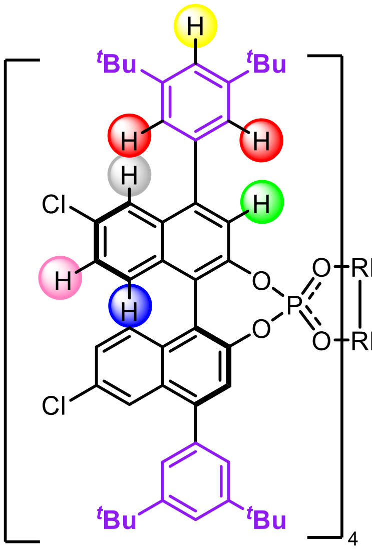

S-**1**

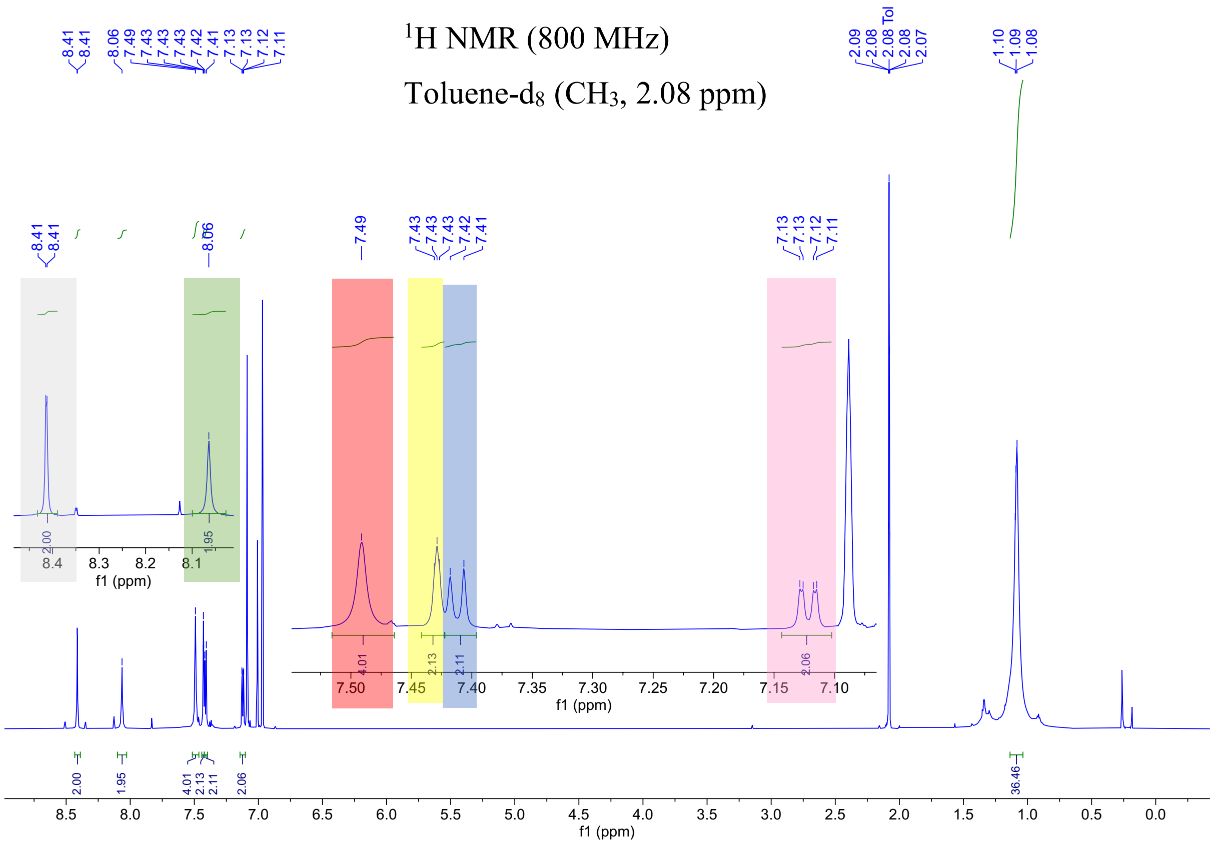

Figure S6.3. ^{1}H NMR spectra of ligand *S*-**1** (range δ 9.0 - -0.5 ppm, **21 °C**)

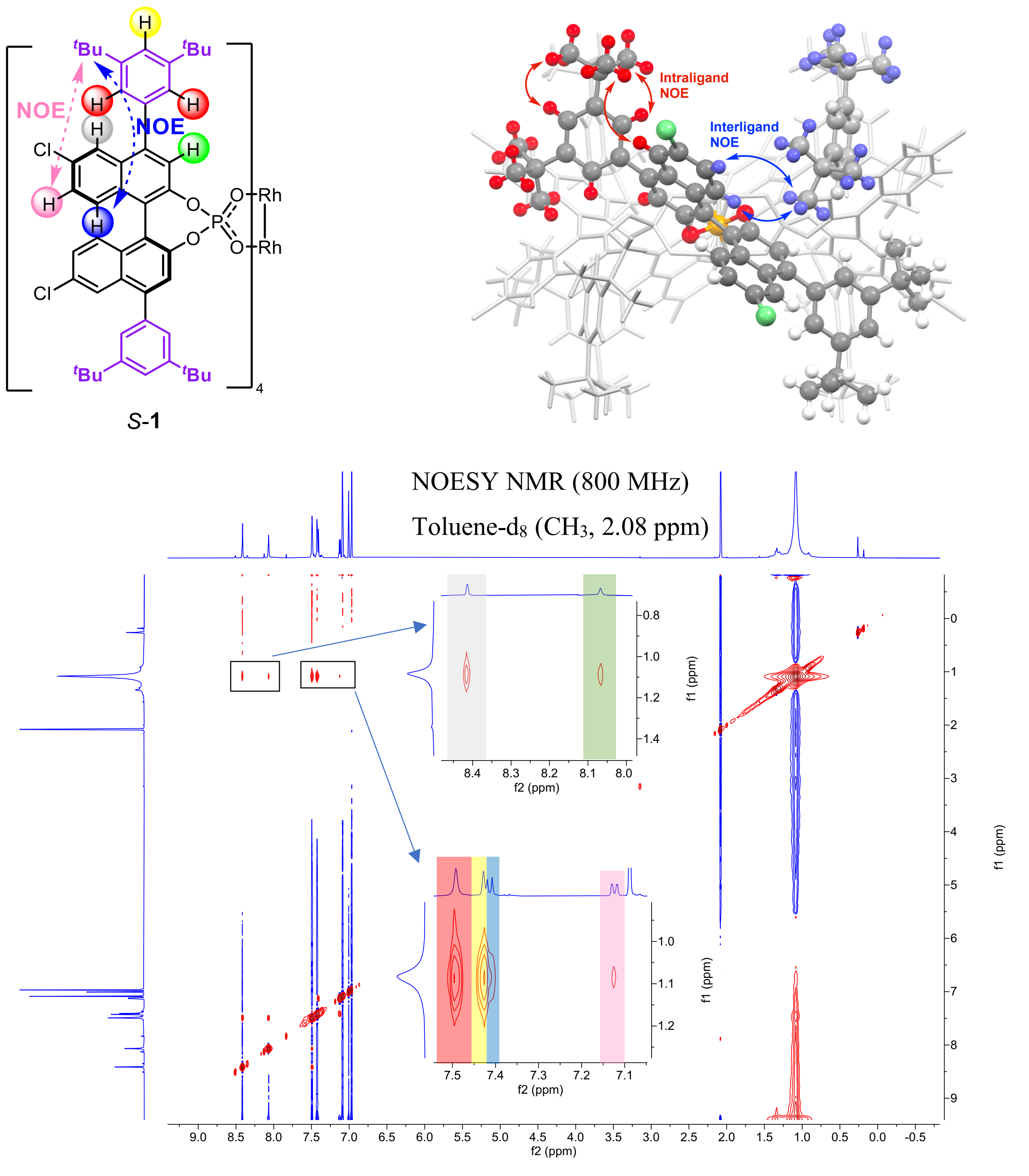

Figure S6.4. NOESY NMR spectra of complex *S*-**1** (range δ 9.0 - -0.5 ppm, **21 °C**) and the corresponding model of obsereved intraligand and interligand NOE (Top right)

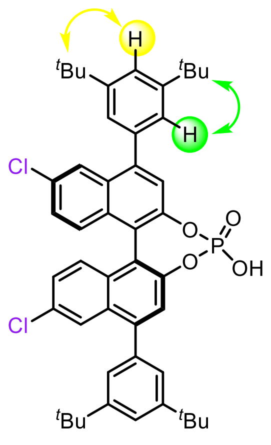

S-**17b**

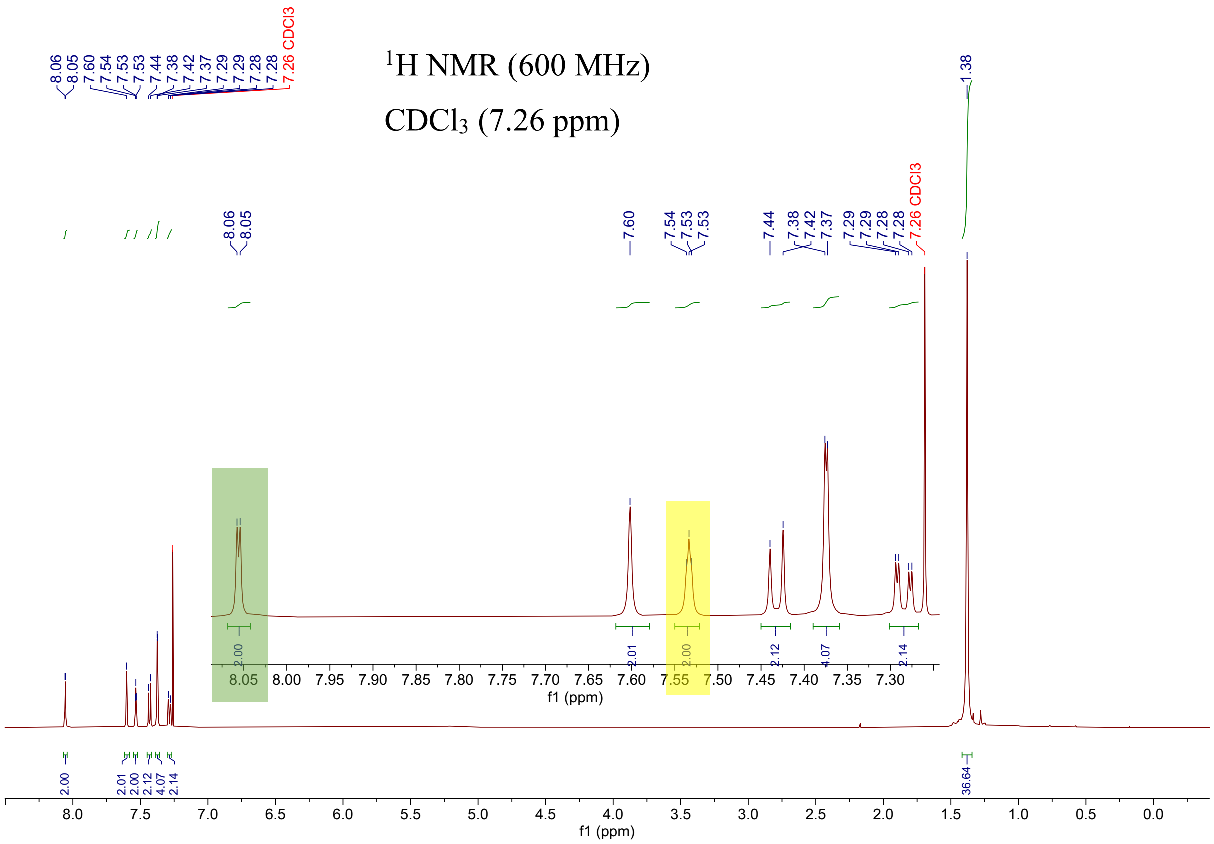

Figure S6.5. ^{1}H NMR spectra of ligand *S*-**17b** (range δ 8.5 - -0.5 ppm, **21 °C**)

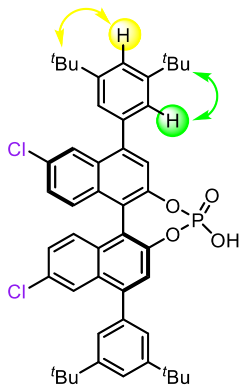

S-**17b**

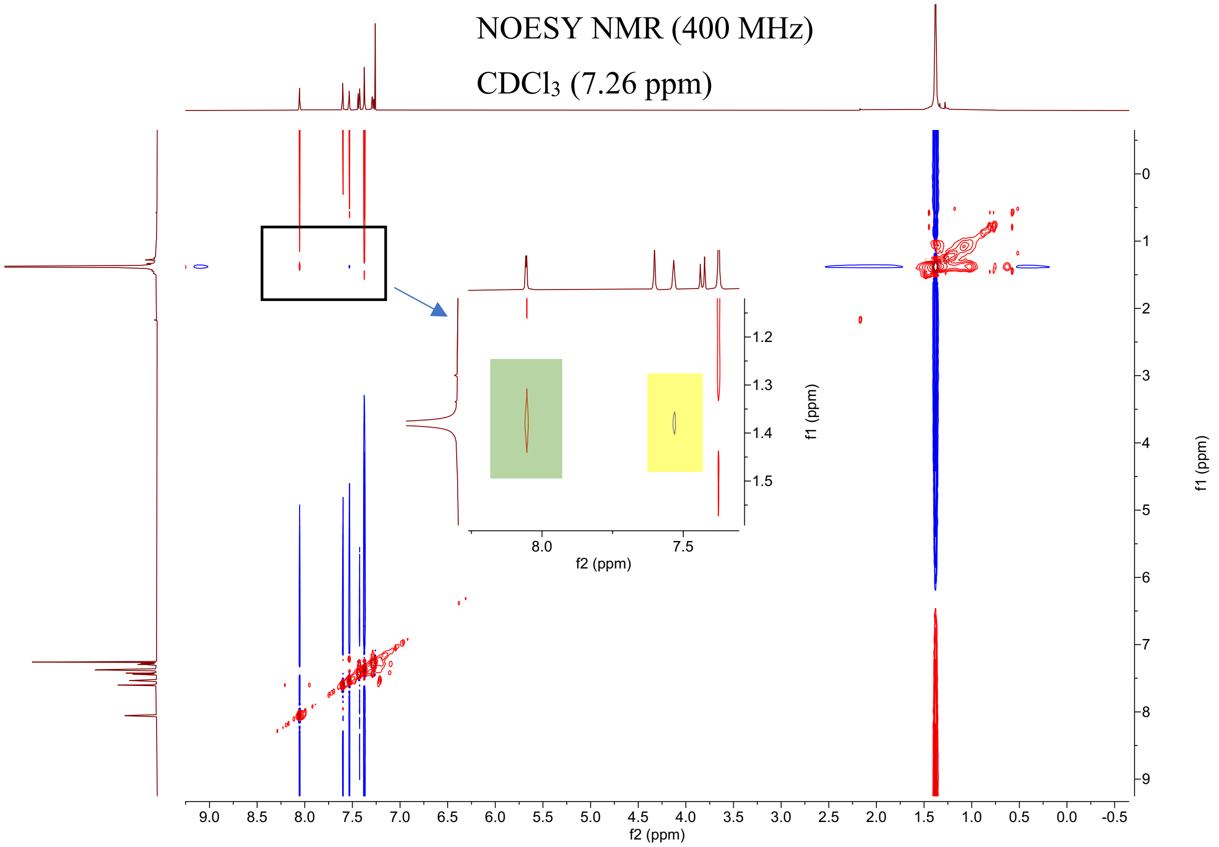

Figure S6.6. NOESY NMR spectra of ligand *S*-**17b** (range δ 9.0 - -0.5 ppm, zoomed in δ 8.2-7.0 ppm, **21 °C**)

7. Calculation

I. Computational Procedure

Geometry optimization and frequency calculations (where that were technologically possible) for all reported structures were performed with the Gaussian 16 suite of programs[1] at the both [B3LYP-D3(BJ)] and B3LYP levels of density functional approaches with the Lanl2dz version of Hay-Wadt effective core potential and basis sets for Rh, and the 6-31G(d,p) basis sets for all other atoms (we labeled the utilized basis sets as [6-31G(d,p) + Lanl2dz]). It is necessary to mention that [B3LYP-D3(BJ)] approach includes Grimme's empirical dispersion-correction (D3) and Becke-Johnson (BJ) damping[2-5] and is expected better account dispersive and long-range non-covalent interactions between various fragments of the calculated complexes. Bulk solvent effects are incorporated in all calculations using the polarizable continuum solvation model (PCM).[6] As a solvent we chose dichloromethane. Below, these computational methods were labeled as {[B3LYP + PCM(DCM)]/[6-31G(d,p) + Lanl2dz]} and {[B3LYP-D3(BJ)] + PCM(DCM)}/[6-31G(d,p) + Lanl2dz], or in short as a B3LYP and [B3LYP-D3(BJ)]. Structures of the calculated complexes and their important geometry parameters are given in **Figures S7.1-S7.13** of the Supporting Information. The cartesian coordinates of all computed structures are included in Section IV of the Supporting Information.

A. Validation of the selected computational approaches.

At first, the presented calculations shown that the overall structure and specific geometry parameters of parent catalyst Rh_2(*R*-BNP)$_4$ *R*-**2**, calculated at the B3LYP and [B3LYP-D3(BJ)] levels, are fully consistent with each other and with their values from the X-ray crystallographic studies (see Figure S1). These collective computational and X-ray studies indicate that complex *R*-**2** adopts a relatively flat structure (with no components of the ligands pointing directly towards the carbene binding sites) and has a pseudo-D_4 symmetry. The calculated (2.526 Å and 2.530 Å, at the B3LYP and [B3LYP-D3(BJ)] levels) Rh-Rh bond distance in *R*-**2** is in excellent agreement with its experimental value of 2.541 Å. Remarkably, the Rh-Rh bond distance in *R*-**2** is significantly (by *ca* 0.15 Å) longer than that in the dirhodium tetracarboxylates, for example in $Rh_2(OAc)_4$.[7] Furthermore, comparison of the calculated and experimental geometry parameters of *R*-**2** show that [B3LYP-D3(BJ)] approache does slightly better describe core structural parameters of the dirhodium tetrakis(binaphthylphosphate) complex than the B3LYP.

B. Importance of dispersive interactions.

To elucidate roles of the dispersive and non-covalent interactions in defining of structures of the aryl-substituted dirhodium tetrakis(binaphthylphosphate) complex, we calculated structures of the Rh_2(*S*-BNP)$_4$ *S*-**2**, Rh_2(*R*-BNP)$_4$ *R*-**2**, Rh_2[*S*-(4,4'-diPh)-BNP]$_4$, Rh_2(*S*-(4,4',6,6'-tetra-Ph)BNP)$_4$ *S*-**6a**, Rh_2(*S*-(6,6'-dichloro-4,4'-(diPh))BNP)$_4$, Rh_2(*S*-(6,6'-dichloro-4,4'-(*p*-di^{t}Bu-Ph))BNP)$_4$ *S*-**18**, and Rh_2(*S*-metaBNP)$_4$ *S*-**1** complexes at both the B3LYP and [B3LYP-D3(BJ)] levels of theory. As seen in **Figures S7.1-S7.12**, these two computational approaches clearly provide dramatically different structural motifs for all calculated complexes. In general, the B3LYP approach consistently leads to the pseudo-D_4-symmetric structures for all calculated dirhodium tetrakis(binaphthylphosphate) complexes with a similar bowl width of top and bottom faces of the catalysts. These conclusions from the B3LYP calculations are inconsistent with the observed enantioselectivity of the studied complexes (see main text). In contrast, at the [B3LYP-D3(BJ)] level of theory, the top and bottom faces of catalysts have very different bowl widths, and the difference between them decreases when the size of substituents on the 4,4'-aryl rings increases. Our largest Rh_2(*S*-metaBNP)$_4$ catalyst has almost symmetrical top and bottom faces, i.e. overall pseudo-D_4 symmetry. Conclusions of the [B3LYP-D3(BJ)] calculations are fully consistent with observed enantioselectivity of the studied dirhodium tetrakis(binaphthylphosphate) catalysts. Based on these analyses we conclude that

the use of density functionals incorporating dispersive and non-covalent interactions into the calculation is absolutely necessary for a better description of dirhodium tetrakis(binaphthylphosphate) catalysts.

Therefore, in this paper we discuss only the [B3LYP-D3(BJ)] calculated results. In most cases, we discuss the Gibbs free energies calculated at 298.15 K temperature and 1 atm. However, for a few larger systems (see below), where the performance of frequency calculations at the [B3LYP-D3(BJ)] level is technically impossible, we use only electronic energies (ΔE) in our discussion.

II. Structures of the calculated catalysts.

Rh_2(*R*-BNP)$_4$ (*R*-**2**) and Rh_2(*S*-BNP)$_4$ (*S*-**2**)

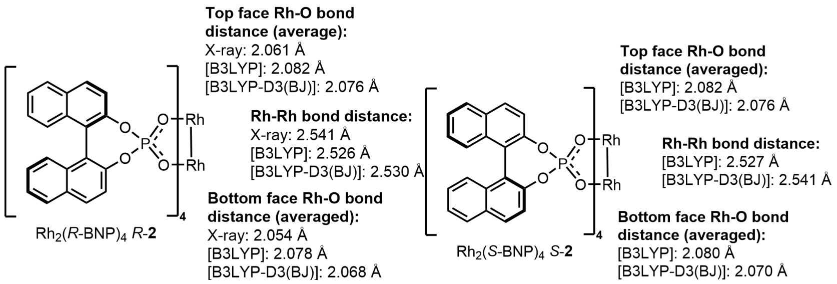

Figure S7.1. Measured and calculated (at the [B3LYP] and [B3LYP-D3(BJ)] levels of theory) important geometry parameters of the Rh_2(*R*-BNP)$_4$ *R*-**2** and Rh_2(*S*-BNP)$_4$ *S*-**2** complexes.

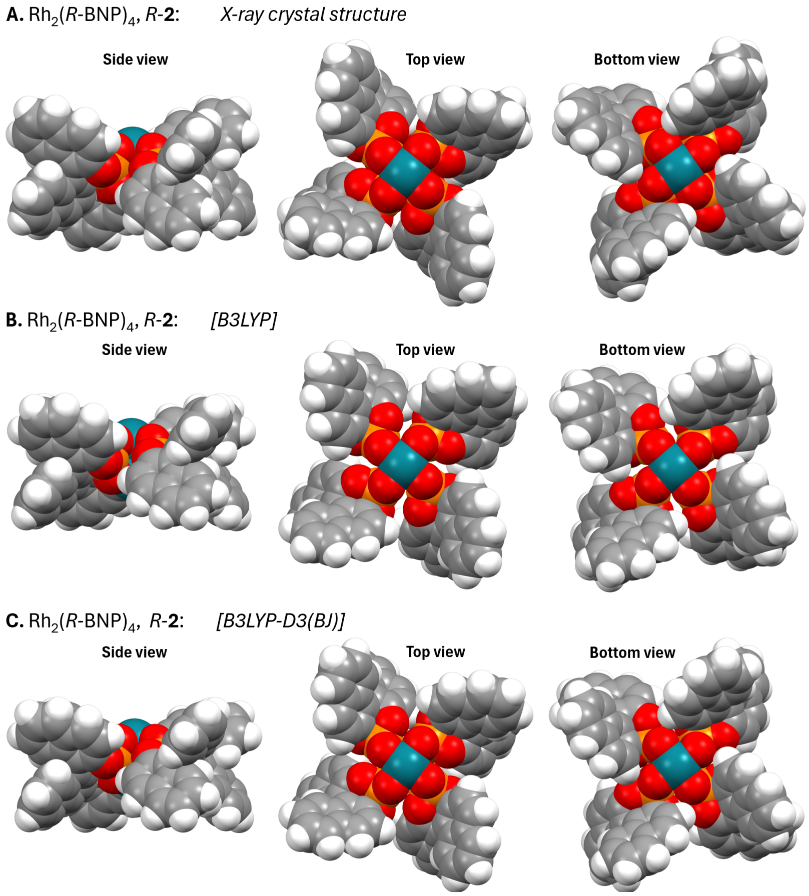

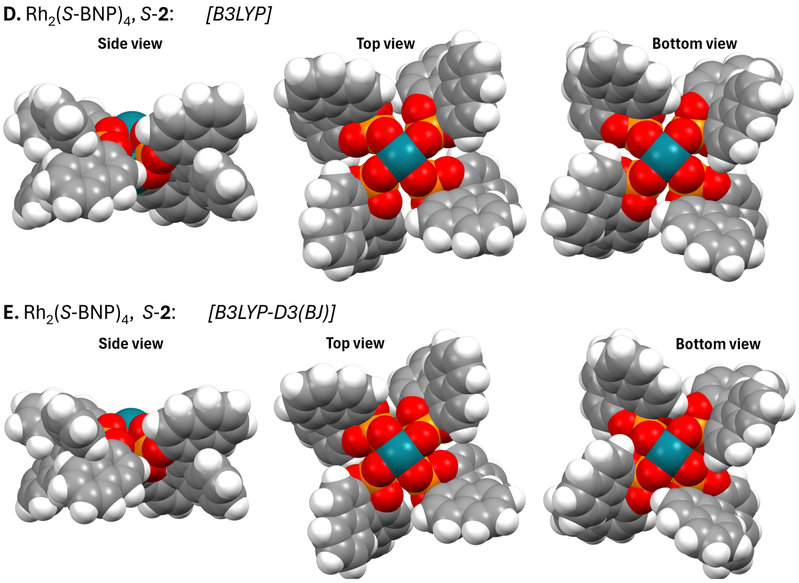

Figure S7.2. (**A**) Experimental structure of the Rh_2(*R*-BNP)$_4$ *R*-**2** complex, and the [B3LYP] and [B3LYP-D3(BJ)] calculated structures of Rh_2(*R*-BNP)$_4$ *R*-**2**, (**B** and **C**, respectively) and Rh_2(*S*-BNP)$_4$, *S*-**2**, (**D** and **E**, respectively).

Rh_2(*S*-(4,4'-diPh)-BNP)$_4$

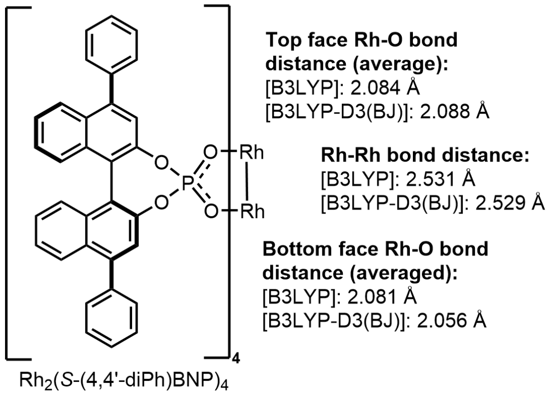

Figure S7.3. Important geometry parameters calculated at the [B3LYP] and [B3LYP-D3(BJ)] levels of theory of the Rh_2(*S*-(4,4'-diPh)BNP)$_4$ catalyst complex.

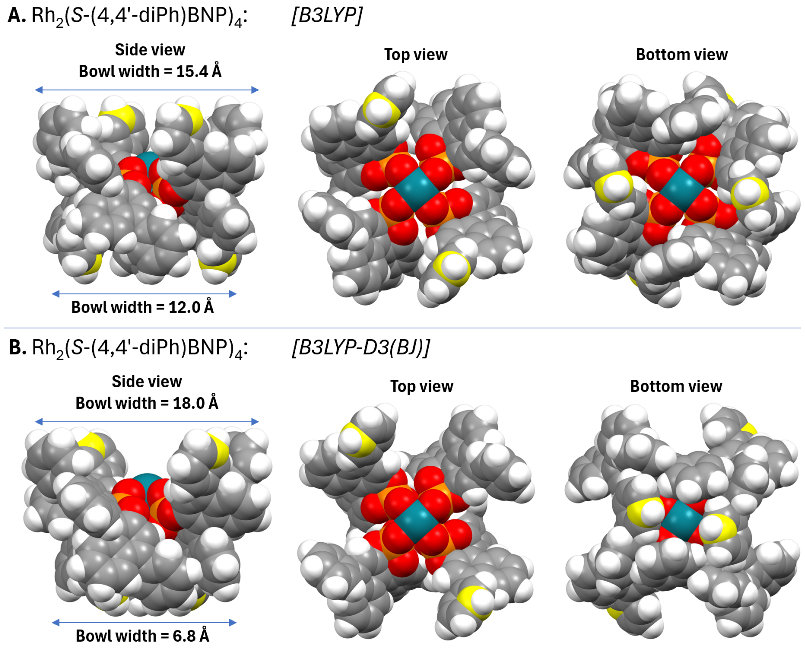

Figure S7.4. Structures of the Rh_2(*S*-(4,4'-diPh)BNP)$_4$ complex calculated at the (**A**) B3LYP and (**B**) [B3LYP-D3(BJ)] levels of theory. Atoms highlighted in yellow indicate the carbon atoms, the distance between which is taken as the bowl width value.

Rh$_2$(*S*-(4,4',6,6'-tetra-Ph)BNP)$_4$ *S*-**6a**

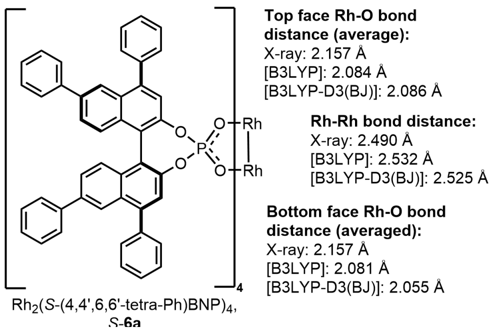

Figure S7.5. Important measured and calculated geometry parameters of the Rh$_2$(*S*-(4,4';6,6'-tetra-Ph)BNP)$_4$ *S*-**6a** complex at the [B3LYP] and [B3LYP-D3(BJ)] levels of theory.

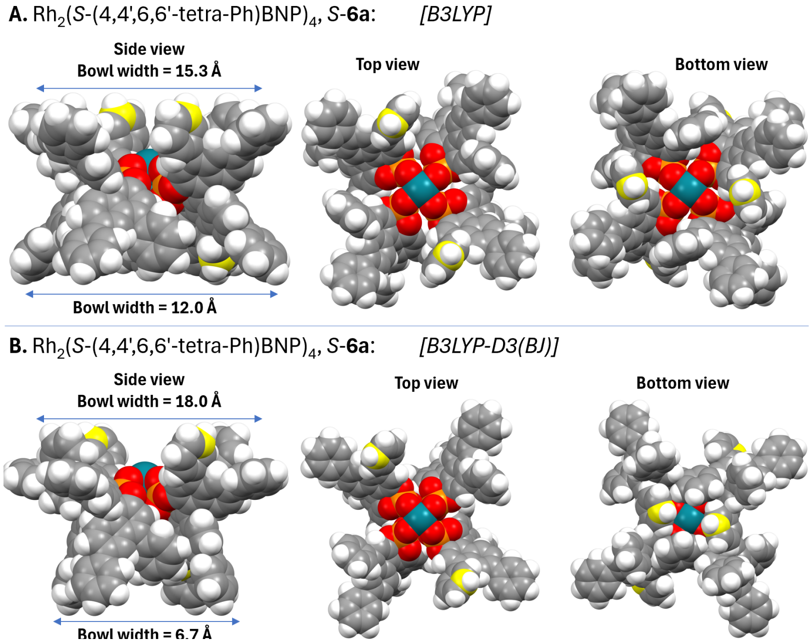

Figure S7.6. Structures of the Rh$_2$(*S*-(4,4',6,6'-tetra-Ph)BNP)$_4$ *S*-**6a** complex calculated at the (**A**) B3LYP and (**B**) [B3LYP-D3(BJ)] levels of theory. Atoms highlighted in yellow indicate the carbon atoms, distance between which is taken as the bowl width value.

Rh$_2$(*S*-(6,6'-dichloro-4,4'-(diPh))BNP)$_4$

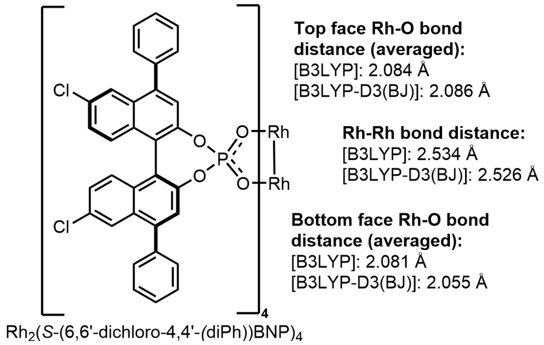

Figure S7.7. Important geometry parameters of the Rh$_2$(*S*-(6,6'-dichloro-4,4'-(diPh)BNP)$_4$ complex calculated at the [B3LYP] and [B3LYP-D3(BJ)] levels of theory.

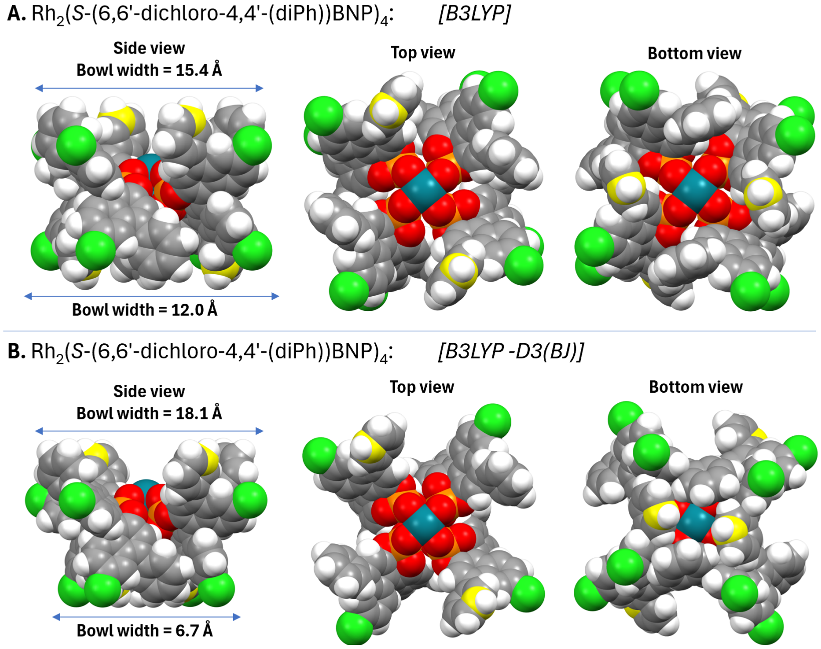

Figure S7.8. Structures of the Rh$_2$(*S*-(6,6'-dichloro-4,4'-(diPh))BNP)$_4$ complex calculated at the (**A**) B3LYP and (**B**) [B3LYP-D3(BJ)] levels of theory. Atoms highlighted in yellow indicate the carbon atoms, distance between which is taken as the bowl width value.

Rh_2(*S*-(6,6'-dichloro-4,4'-(*p*-*t*Bu-Ph))BNP)$_4$ (*S*-**18**)

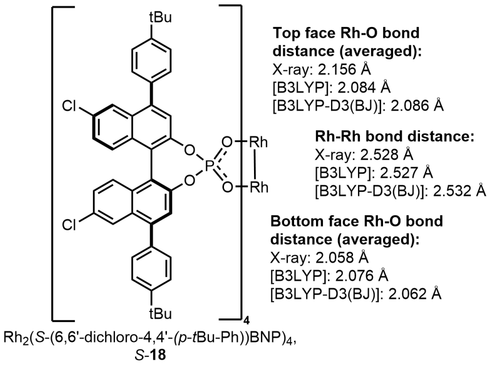

Figure S7.9. Important geometry parameters of the Rh_2(*S*-(6,6'-dichloro-4,4'-(*p*-*t*Bu-Ph)BNP)$_4$ *S*-**18** complex measured and calculated at the [B3LYP] and [B3LYP-D3(BJ)] levels of theory.

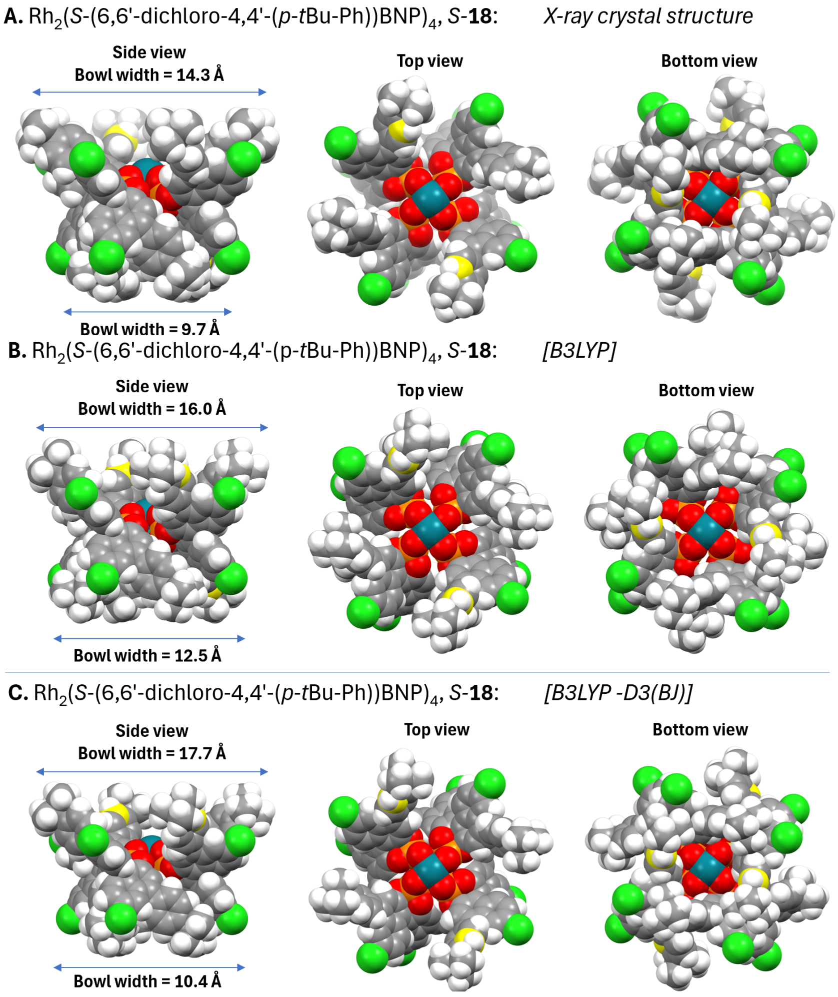

Figure S7.10. (**A**) Experimental and calculated at the (**B**) B3LYP and (**C**) [B3LYP-D3(BJ)] levels of theory, structures of Rh_2(*S*-(6,6'-dichloro-4,4'-(*p*-di^{t}But-Ph))BNP)$_4$ (*S*-**18**). Atoms highlighted in yellow indicate the carbon atoms, distance between which is taken as the bowl width value.

Rh_2(*S*-megaBNP)$_4$ (*S*-**1**)

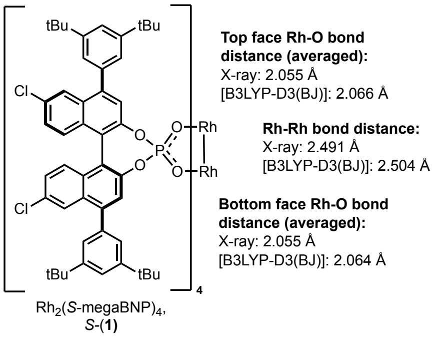

Figure S7.11. Important geometry parameters of the Rh_2(*S*-megaBNP)$_4$ (*S*-**1**) complex measured and calculated at the [B3LYP-D3(BJ)] level of theory.

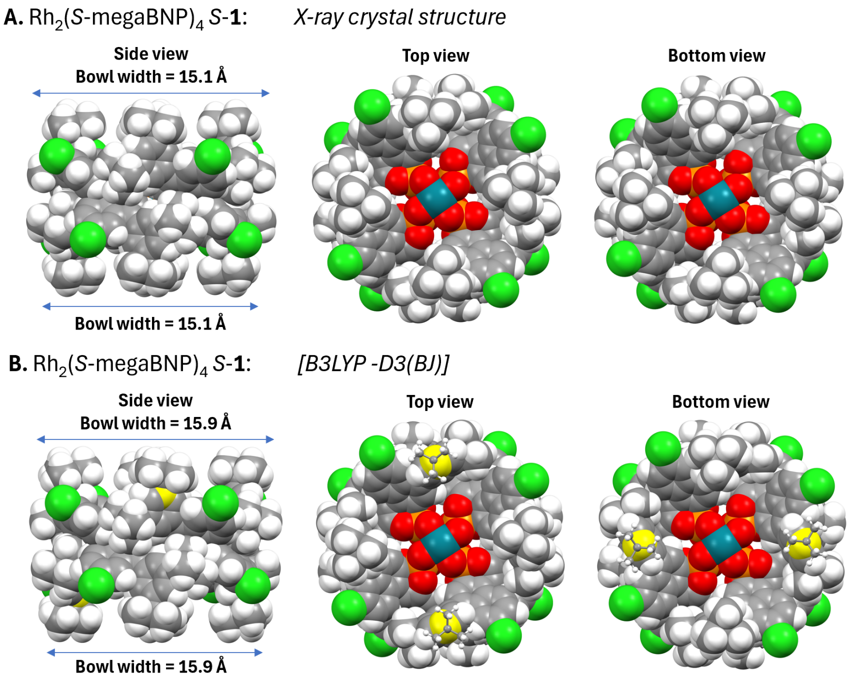

Figure S7.12. (**A**) Experimental and (**B**) calculated structures of Rh_2(*S*-megaBNP)$_4$ (*S*-**1**) at the [B3LYP-D3(BJ)] level of theory. Atoms highlighted in yellow indicate the carbon atoms, distance between which is taken as the bowl width value.

III. Structural analyses of the carbene-catalyst complexes

Preliminary analyses into the geometry of two different carbenes and their isomers were conducted (see Figure S14). Due to the large size of the studied systems (>444 atoms), we failed to calculate frequencies of these complexes at the B3LYP-D3(BJ) level of theory, therefore, here we report only geometries and ΔE-based relative energies of the calculated structures. Furthermore, since these species have several isomeric forms, here we discuss only the energetically lowest isomers. For both {Rh_2(*S*-megaBNP)$_4$}-[Ph-TCE] and {Rh_2(*S*-megaBNP)$_4$}-[(*p*-BrPh)-TCE] carbenes (see Figure S7.13), the energetically lowest isomers are the structures with the Ph [or (*p*-BrPh)] and TCE fragments on the Rh-Rh-Carbene plane. However, as seen in Figure S7.13, aryl rings of these carbenes are slightly tilted [the calculated (Rh,C^1,C^2,C^3) dihedral angles are -69.3 and -89.0 degrees] relative to the Rh-Rh-Carbene plane. The calculated (C^4,O^1,C^5,C^6) dihedral angles are 162.5 and 117.8 degrees in the {Rh_2(*S*-megaBNP)$_4$}-[Ph-TCE] and {Rh_2(*S*-megaBNP)$_4$}-[(*p*-BrPh)-TCE] carbenes, respectively. Close examinations of these structures indicate that the "reactive pocket" is more congested in {Rh_2(*S*-megaBNP)$_4$}-[Ph-TCE] than {Rh_2(*S*-megaBNP)$_4$}-[(*p*-BrPh)-TCE].

A. **B.**

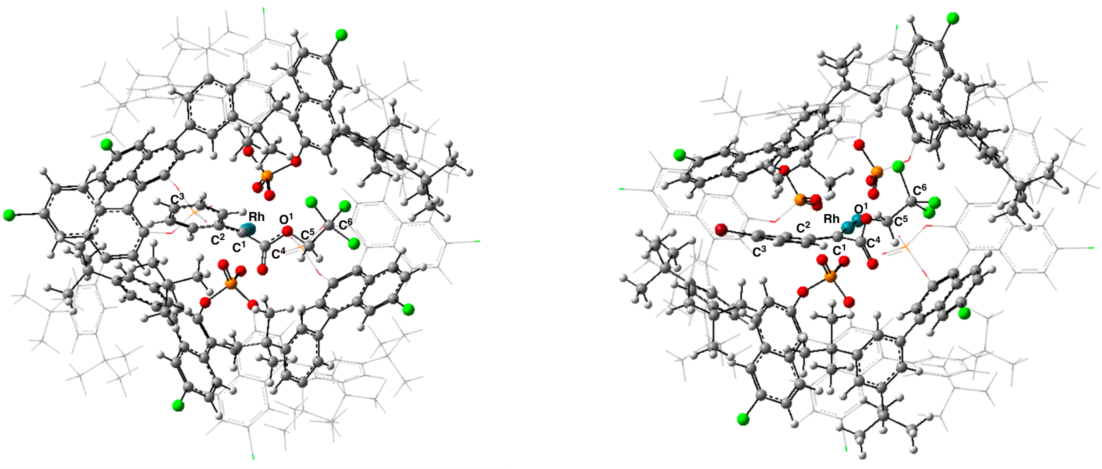

Figure S7.13. The calculated lowest-energy structures of the (**A**) {Rh_2(*S*-megaBNP)$_4$}-[Ph-TCE] and (**B**) {Rh_2(*S*-megaBNP)$_4$}-[(*p*-BrPh)-TCE] carbene complexes.

IV. Superimposed x-ray crystal and DFT-calculated structures for catalysts *S*-1 and *S*-18

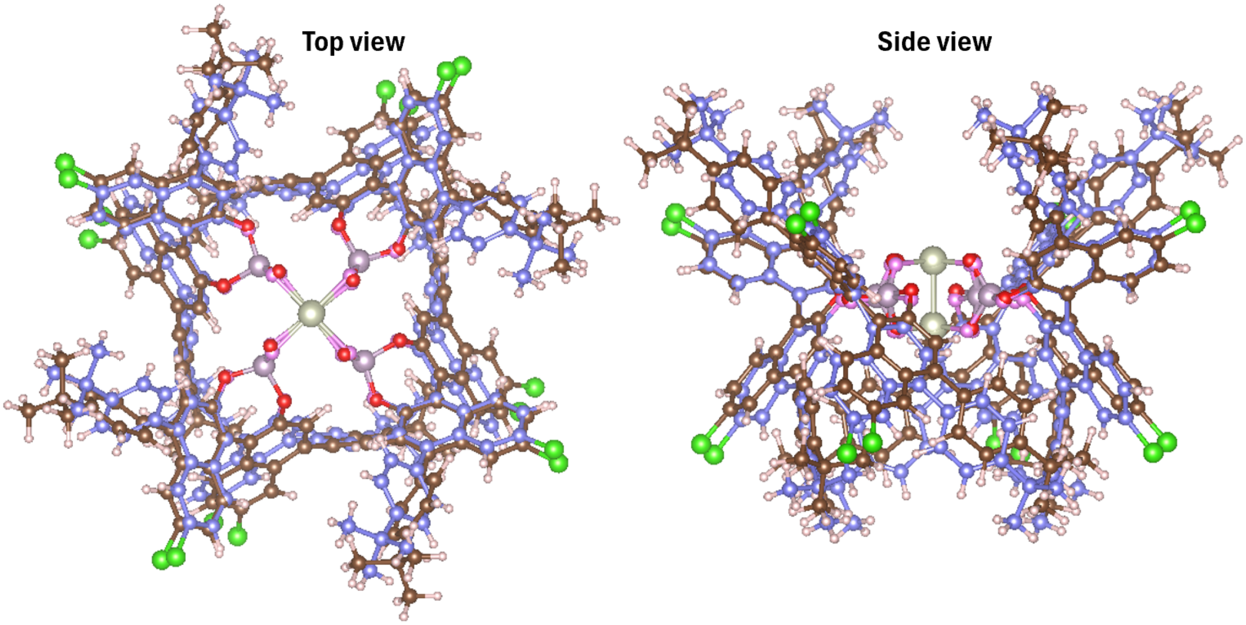

Figure S7.14. The superimposed experimental x-ray crystal (purple) and the calculated (brown) structures of Rh_2(*S*-(6,6'-dichloro-4,4'-(p-tBu-Ph)BNP$)_4$ (*S*-**18**).

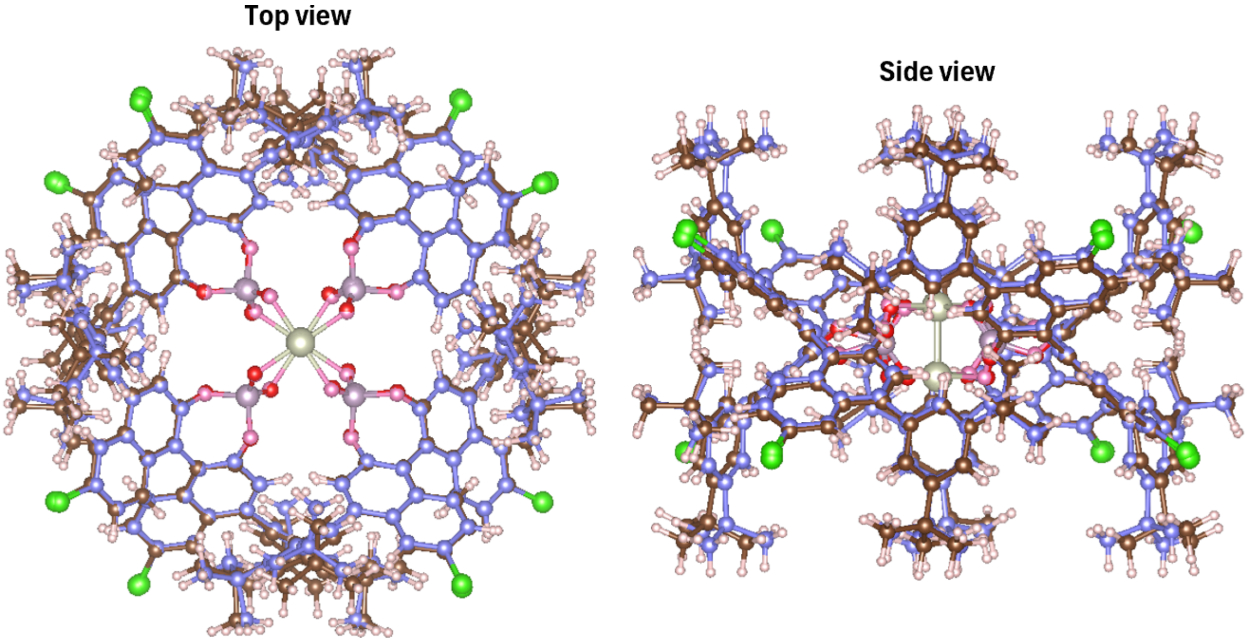

Figure S7.15. The superimposed experimental x-ray crystal (purple) and the calculated (brown) structures of catalyst Rh_2(*S*-megaBNP$)_4$ (*S*-**1**).

V. Cartesian coordinates of all calculated structures

Rh_2(*R*-BNP)$_4$, (*R*-2) [B3LYP-D3(BJ)]

	X	Y	Z
Rh	0.0160960	-0.2808710	-0.0109630
Rh	0.0160890	-0.2808810	2.5191220
P	2.5532130	0.8816360	1.2545460
P	1.1784550	-2.8179910	1.2545730
O	2.0125650	0.2879190	-0.0454060
O	1.9909860	0.3312650	2.5663700
O	2.3676050	2.4874120	1.1204900
O	4.1488150	0.6852670	1.3937980
O	0.5844410	-2.2774750	-0.0453000
O	0.6280910	-2.2558410	2.5664440
O	0.9823840	-4.4136190	1.3937820
O	2.7841590	-2.6320220	1.1204000
C	4.3709450	3.4463520	2.0287490
C	5.1425200	2.8325250	0.9149690
C	5.0199090	1.4821730	0.6353320
C	6.6420000	1.5448640	-1.1288160
H	7.2319090	1.0538580	-1.8963940
C	6.7723000	2.9475880	-0.9506900
C	6.0007140	3.6092960	0.0615070
C	7.6350720	3.7129480	-1.7801980
H	8.2213620	3.1952980	-2.5338970
C	7.7168520	5.0793560	-1.6448990
H	8.3760630	5.6548410	-2.2869570
C	6.9231720	5.7393780	-0.6771000
H	6.9686170	6.8205910	-0.5918330
C	6.0876980	5.0254080	0.1525840
H	5.4788300	5.5462440	0.8806440
C	2.9986300	3.2822930	2.0889090
C	4.9969060	4.1942550	3.0843530
C	4.1751010	4.8214930	4.0791140
C	2.7643710	4.6591980	4.0322800
H	2.1544140	5.1417310	4.7898040
C	2.1787290	3.8791500	3.0651420
H	1.1103970	3.7059890	3.0232440
C	6.9734590	5.0322820	4.2340640
H	8.0540800	5.0994640	4.3124670
C	6.1600150	5.6855250	5.1900550
H	6.6194820	6.2601460	5.9880080
C	4.7908670	5.5748940	5.1142170
H	4.1552540	6.0535850	5.8535890
C	1.7794040	-5.2844900	0.6351530
C	3.1297940	-5.4068670	0.9146760
C	3.9066650	-6.2648710	0.0611180
C	3.2450320	-7.0364660	-0.9511200
C	1.8422810	-6.9063570	-1.1291860
H	1.3513490	-7.4962480	-1.8968260
C	1.1194150	-6.0243450	-0.3643650
H	0.0531840	-5.8823370	-0.4983990
C	5.3227850	-6.3516860	0.1521710
H	5.8435640	-5.7428090	0.8802620
C	6.0368440	-7.1870120	-0.6775890
H	7.1180640	-7.2323250	-0.5923350
C	5.3769090	-7.9807000	-1.6454390
H	5.9524610	-8.6398070	-2.2875420
C	4.0104870	-7.8990760	-1.7807110
H	3.4928940	-8.4853700	-2.5344460
C	3.7435940	-4.6352410	2.0284360
C	3.5793020	-3.2629530	2.0886790
C	4.1761940	-2.4430050	3.0648460
H	4.0028700	-1.3746990	3.0230330
C	4.9564860	-3.0285640	4.0318430
H	5.4390500	-2.4185520	4.7893000
C	5.1190260	-4.4392660	4.0785900
C	4.4917840	-5.2611210	3.0838760
C	5.8726800	-5.0549730	5.1135460
H	6.3513760	-4.4193240	5.8528840
C	5.9835440	-6.4241040	5.1892820
H	6.5583480	-6.8835380	5.9871230
C	5.3303070	-7.2376040	4.2333300
H	5.3976880	-8.3182170	4.3116800
C	4.6038290	-6.6725250	3.2090010
H	4.1011370	-7.3075800	2.4902850
C	6.4083190	4.3060340	3.2095980
H	7.0433560	3.8033380	2.4908730
C	5.7597730	0.8221150	-0.3641300
H	5.6176010	-0.2440860	-0.4982370
P	-2.5210280	-1.4433880	1.2545230
P	-1.1462700	2.2562380	1.2545860
O	-1.9803730	-0.8496620	-0.0454220
O	-1.9588090	-0.8930260	2.5663540
O	-2.3354190	-3.0491630	1.1204570
O	-4.1166310	-1.2470190	1.3937660
O	-0.5522470	1.7157330	-0.0452870
O	-0.5959150	1.6940800	2.5664570
O	-0.9502040	3.8518660	1.3938080

O	-2.7519740	2.0702690	1.1204020
C	-4.3387630	-4.0081150	2.0286970
C	-5.1103410	-3.3942730	0.9149290
C	-4.9877250	-2.0439200	0.6352950
C	-6.6098280	-2.1065990	-1.1288430
H	-7.1997400	-1.6155890	-1.8964160
C	-6.7401490	-3.5093200	-0.9507050
C	-5.9685640	-4.1710320	0.0614890
C	-7.6029470	-4.2746730	-1.7801930
H	-8.1892350	-3.7570210	-2.5338920
C	-7.6847580	-5.6410780	-1.6448720
H	-8.3439890	-6.2165560	-2.2869130
C	-6.8910880	-6.3011020	-0.6770650
H	-6.9365620	-7.3823130	-0.5917780
C	-6.0555880	-5.5871390	0.1525990
H	-5.4467290	-6.1079760	0.8806660
C	-2.9664480	-3.8440540	2.0888650
C	-4.9647260	-4.7560560	3.0842700
C	-4.1429260	-5.3833060	4.0790280
C	-2.7321970	-5.2209930	4.0322140
H	-2.1222440	-5.7035380	4.7897330
C	-2.1465520	-4.4409250	3.0650940
H	-1.0782200	-4.2677600	3.0232050
C	-6.9412840	-5.5941560	4.2339170
H	-8.0219040	-5.6613670	4.3122930
C	-6.1278450	-6.2473990	5.1899130
H	-6.5873160	-6.8220460	5.9878440
C	-4.7586970	-6.1367400	5.1141030
H	-4.1230890	-6.6154360	5.8534760
C	-1.7472180	4.7227310	0.6351670
C	-3.0976140	4.8450940	0.9146680
C	-3.8744960	5.7030110	0.0610260
C	-3.2128540	6.4745960	-0.9512150
C	-1.8100920	6.3445400	-1.1292230
H	-1.3191560	6.9344160	-1.8968730
C	-1.0872220	5.4625700	-0.3643580
H	-0.0209850	5.3205800	-0.4983650
C	-5.2906300	5.7897300	0.1519720
H	-5.8114210	5.1808400	0.8800420
C	-6.0046900	6.6249780	-0.6778650
H	-7.0859190	6.6702210	-0.5926850
C	-5.3447450	7.4186780	-1.6456980
H	-5.9202990	8.0777230	-2.2878620
C	-3.9783110	7.3371310	-1.7808830
H	-3.4607080	7.9234240	-2.5346130
C	-3.7113960	4.0734850	2.0284530
C	-3.5471130	2.7011960	2.0886850
C	-4.1439880	1.8812440	3.0648590
H	-3.9706780	0.8129360	3.0230300
C	-4.9242430	2.4668030	4.0318840
H	-5.4067900	1.8567890	4.7893510
C	-5.0867310	3.8775080	4.0786730
C	-4.4595010	4.6993690	3.0839560
C	-5.8403080	4.4932110	5.1136900
H	-6.3189990	3.8575560	5.8530250
C	-5.9510960	5.8623440	5.1894900
H	-6.5258370	6.3217740	5.9873780
C	-5.2978470	6.6758490	4.2335510
H	-5.3651560	7.7564620	4.3119570
C	-4.5714450	6.1107750	3.2091650
H	-4.0687400	6.7458380	2.4904660
C	-6.3761380	-4.8678740	3.2094770
H	-7.0111690	-4.3651820	2.4907440
C	-5.7275900	-1.3838570	-0.3641620
H	-5.5854130	-0.3176570	-0.4982690

Rh_2(*R*-BNP)$_4$, (*R*-2) [B3LYP]

Rh	-1.4545420	1.7090950	0.1464650
Rh	-1.4545370	1.7090800	2.6724180
P	1.0142500	3.1309700	1.4080920
P	-0.0326620	-0.7596510	1.4080840
O	0.4503420	2.5495330	0.1103230
O	0.4160950	2.6119270	2.7173420
O	0.9013240	4.7433950	1.2563190
O	2.6054380	2.8805550	1.5529270
O	-0.6143330	-0.1958800	0.1103490
O	-0.5518570	-0.1616510	2.7173510
O	-0.2826490	-2.3508830	1.5528820
O	1.5797280	-0.6462410	1.2562730
C	2.9831980	5.6426880	2.0665490
C	3.6922320	4.9519430	0.9511870
C	3.4927470	3.5979060	0.7341160
C	5.0591330	3.4997000	-1.0804510
H	5.5991670	2.9444860	-1.8416100
C	5.2592690	4.9023680	-0.9759620
C	4.5572500	5.6472120	0.0313650
C	6.1225420	5.5853570	-1.8758500
H	6.6542210	5.0039450	-2.6241240
C	6.2707720	6.9520070	-1.8154420
H	6.9282420	7.4636950	-2.5116600
C	5.5471910	7.6946480	-0.8523570
H	5.6436940	8.7759380	-0.8240170
C	4.7153490	7.0615950	0.0456040
H	4.1618660	7.6492730	0.7678820
C	1.6059150	5.5380700	2.1742760
C	3.6706140	6.3981380	3.0829010
C	2.9052810	7.0974210	4.0767780
C	1.4871910	7.0045940	4.0674140

H	0.9183600	7.5456080	4.8178970
C	0.8426890	6.2174270	3.1450890
H	-0.2357750	6.1052800	3.1320460
C	5.7131510	7.1735160	4.1683600
H	6.7976960	7.1874970	4.2221900
C	4.9553590	7.8952790	5.1209940
H	5.4603210	8.4692510	5.8919700
C	3.5807560	7.8512670	5.0752500
H	2.9856780	8.3831460	5.8125820
C	0.4349990	-3.2380460	0.7341290
C	1.7890900	-3.4370890	0.9511950
C	2.4845950	-4.3019570	0.0313900
C	1.7399350	-5.0042990	-0.9758440
C	0.3371830	-4.8046420	-1.0802710
H	-0.2178950	-5.3449620	-1.8413260
C	-0.3060590	-3.9162680	-0.2545470
H	-1.3699800	-3.7225820	-0.3338650
C	3.8990260	-4.4595830	0.0455750
H	4.4865490	-3.9058770	0.7678090
C	4.5323180	-5.2912630	-0.8523750
H	5.6136410	-5.3874050	-0.8240850
C	3.7898730	-6.0151400	-1.8153800
H	4.3017490	-6.6724520	-2.5116070
C	2.4231640	-5.8673840	-1.8757220
H	1.8419000	-6.3993070	-2.6239370
C	2.4796550	-2.7278300	2.0665230
C	2.3746290	-1.3505700	2.1742260
C	3.0537630	-0.5871350	3.1450240
H	2.9413090	0.4912980	3.1319720
C	3.8411250	-1.2314030	4.0673510
H	4.3819590	-0.6624010	4.8178350
C	3.9343880	-2.6494560	4.0767240
C	3.2353290	-3.4150120	3.0828540
C	4.6884720	-3.3246900	5.0751820
H	5.2201790	-2.7294430	5.8125030
C	4.7329330	-4.6992770	5.1209250
H	5.3070870	-5.2040470	5.8918920
C	4.0113900	-5.4573010	4.1683080
H	4.0257200	-6.5418410	4.2221280
C	3.2844340	-4.8341090	3.1771600
H	2.7298580	-5.4316410	2.4636560
C	5.0897230	6.4467900	3.1771900
H	5.6870640	5.8920530	2.4636500
C	4.1706330	2.8566940	-0.2546700
H	3.9766040	1.7928360	-0.3340430
P	-3.9233300	0.2872060	1.4080850
P	-2.8764190	4.1778260	1.4081180
O	-3.3594270	0.8686580	0.1103200
O	-3.3251700	0.8062330	2.7173390
O	-3.8104080	-1.3252180	1.2562950
O	-5.5145180	0.5376150	1.5529280
O	-2.2947520	3.6140700	0.1103750
O	-2.3572170	3.5798110	2.7173770
O	-2.6264330	5.7690570	1.5529330
O	-4.4888090	4.0644190	1.2563130
C	-5.8922820	-2.2244900	2.0665440
C	-6.6012840	-1.5337730	0.9511390
C	-6.4018140	-0.1797280	0.7340980
C	-7.9681080	-0.0815140	-1.0805430
H	-8.5081130	0.4737040	-1.8417200
C	-8.1681560	-1.4842020	-0.9761500
C	-7.4661550	-2.2290600	0.0311820
C	-9.0312990	-2.1671910	-1.8761620
H	-9.5629700	-1.5857640	-2.6244300
C	-9.1793960	-3.5338610	-1.8158960
H	-9.8367590	-4.0455500	-2.5122130
C	-8.4557920	-4.2765190	-0.8528430
H	-8.5521730	-5.3578230	-0.8246250
C	-7.6240820	-3.6434670	0.0452400
H	-7.0705760	-4.2311660	0.7674820
C	-4.5149960	-2.1198850	2.1742600
C	-6.5797030	-2.9798170	3.0829940
C	-5.8143630	-3.6790790	4.0768820
C	-4.3962690	-3.5863280	4.0674550
H	-3.8274370	-4.1273150	4.8179570
C	-3.7517670	-2.7992150	3.1450870
H	-2.6733010	-2.6870840	3.1320250
C	-8.6222290	-3.7549180	4.1686870
H	-9.7067710	-3.7687750	4.2226190
C	-7.8644310	-4.4767130	5.1212900
H	-8.3693840	-5.0505950	5.8923380
C	-6.4898280	-4.4328150	5.0754440
H	-5.8947410	-4.9646980	5.8127660
C	-3.3440860	6.6562180	0.7341820
C	-4.6981790	6.8552460	0.9512440
C	-5.3937090	7.7200240	0.0313660
C	-4.6490540	8.4223550	-0.9758800
C	-3.2462910	8.2227550	-1.0802670
H	-2.6912210	8.7630590	-1.8413380
C	-2.6030350	7.3344260	-0.2545080
H	-1.5391090	7.1407570	-0.3338140
C	-6.8081540	7.8775450	0.0454610
H	-7.3956800	7.3238240	0.7676810
C	-7.4414580	8.7091410	-0.8525560
H	-8.5227900	8.8052060	-0.8243280
C	-6.6990170	9.4330310	-1.8155530
H	-7.2109040	10.0902780	-2.5118340
C	-5.3322960	9.2853600	-1.8758250

H	-4.7510320	9.8172830	-2.6240400
C	-5.3887130	6.1460000	2.0666040
C	-5.2836940	4.7687370	2.1742860
C	-5.9627970	4.0052930	3.1450980
H	-5.8503570	2.9268590	3.1320220
C	-6.7501080	4.6495550	4.0674710
H	-7.2909130	4.0805460	4.8179710
C	-6.8433180	6.0676100	4.0768990
C	-6.1442850	6.8331780	3.0830190
C	-7.5973070	6.7428320	5.0754390
H	-8.1289980	6.1475740	5.8127620
C	-7.6416880	8.1174170	5.1212600
H	-8.2157660	8.6221780	5.8922910
C	-6.9201460	8.8754520	4.1686510
H	-6.9344010	9.9599900	4.2225390
C	-6.1932860	8.2522730	3.1774250
H	-5.6387050	8.8498170	2.4639350
C	-7.9988110	-3.0283070	3.1774260
H	-8.5961630	-2.4735280	2.4639280
C	-7.0796720	0.5614940	-0.2546980
H	-6.8856630	1.6253580	-0.3340360

Rh_2(*S*-BNP)$_4$, (*S*-2) [B3LYP-D3(BJ)]

C	-0.7636310	-0.4559280	6.6315360
C	0.6127130	-0.1755030	6.1431940
C	0.8032300	0.3140320	4.8636020
C	3.1721560	0.5237400	5.1323020
H	4.1514190	0.8074430	4.7590360
C	3.0684610	-0.0396040	6.4324500
C	1.7819770	-0.4168000	6.9431650
C	4.2242550	-0.2631060	7.2275670
H	5.1899490	0.0384200	6.8321850
C	4.1280510	-0.8612650	8.4627200
H	5.0185100	-1.0317630	9.0594150
C	2.8645740	-1.2724240	8.9496650
H	2.7956650	-1.7682270	9.9129180
C	1.7225550	-1.0563850	8.2111080
H	0.7636350	-1.3850640	8.5919390
C	-1.6173310	-1.2687980	5.9054180
C	-1.2780730	0.1353100	7.8373450
C	-2.5877450	-0.2274610	8.2960900
C	-3.3769710	-1.1240700	7.5283520
H	-4.3662940	-1.3933990	7.8847490
C	-2.9108390	-1.6209090	6.3361000
H	-3.5032080	-2.2838510	5.7159510
C	-1.0693140	1.6463880	9.7365350
H	-0.4971410	2.3880720	10.2850840
C	-2.3429030	1.2527990	10.2112030
H	-2.7351250	1.6833800	11.1270380
C	-3.0861940	0.3402530	9.4992600
H	-4.0749280	0.0460710	9.8392930
C	-0.5510880	1.1041530	8.5817430
H	0.4201430	1.4239120	8.2260960
C	2.0592890	0.6771110	4.3418920
H	2.1104050	1.0624890	3.3313050
O	-1.1921340	-1.8290280	4.6916970
O	-0.3119020	0.4704270	4.0250240
P	-0.9413800	-0.8899350	3.4038290
O	-2.2499870	-0.4860100	2.7270950
O	0.0706380	-1.6685480	2.5620130
Rh	-2.0928380	-0.0240650	0.7072430
Rh	0.1744480	-1.1616170	0.5589120
C	-1.7121950	-0.4846310	-5.2984000
C	-1.0753760	-1.7971300	-5.0089610
C	-1.1435850	-2.3294790	-3.7338510
C	-0.0025690	-4.3416620	-4.3483020
H	0.3929280	-5.3236570	-4.1075040
C	0.1661020	-3.8364790	-5.6655570
C	-0.3529090	-2.5420810	-6.0025260
C	0.8661730	-4.5818370	-6.6518070
H	1.2436000	-5.5650120	-6.3858760
C	1.0754110	-4.0676740	-7.9105850
H	1.6152170	-4.6445710	-8.6547940
C	0.6003030	-2.7738080	-8.2312810
H	0.7902530	-2.3609270	-9.2171100
C	-0.0942940	-2.0305770	-7.3032750
H	-0.4443540	-1.0383720	-7.5592430
C	-1.4128900	0.6247140	-4.5264750
C	-2.6889490	-0.3216160	-6.3408440
C	-3.2199210	0.9836770	-6.6094820
C	-2.8100390	2.0863700	-5.8141540
H	-3.2168940	3.0699880	-6.0268820
C	-1.9362860	1.9087360	-4.7697090
H	-1.6301780	2.7260550	-4.1268560
C	-4.1218270	-1.2266710	-8.0903960
H	-4.4910930	-2.0797440	-8.6510030
C	-4.6116040	0.0683340	-8.3826490
H	-5.3428770	0.2038860	-9.1731280
C	-4.1724260	1.1470770	-7.6508690
H	-4.5561300	2.1436820	-7.8490360
C	-3.1874020	-1.4174020	-7.0971290
H	-2.8301570	-2.4158160	-6.8789460
C	-0.6277690	-3.5912410	-3.3822800
H	-0.7407930	-3.9308770	-2.3599930
O	-0.5183680	0.5069800	-3.4505200
O	-1.7748580	-1.5890830	-2.7238680
P	-0.9727290	-0.3083750	-2.1341710
O	-1.9879140	0.4745820	-1.3042660

O	0.3409440	-0.6966720	-1.4528460
C	-3.8806240	-5.8154370	0.8982400
C	-2.6392470	-5.9947790	1.6972280
C	-1.9800850	-4.8889080	2.2034270
C	-0.3283130	-6.2017580	3.3332380
H	0.5439920	-6.2890430	3.9737190
C	-0.9070960	-7.3825460	2.7952910
C	-2.0621630	-7.2861290	1.9499420
C	-0.3430680	-8.6598640	3.0571230
H	0.5245250	-8.7184160	3.7080400
C	-0.8711870	-9.7961510	2.4891060
H	-0.4287640	-10.7662160	2.6922690
C	-1.9850820	-9.6978770	1.6218340
H	-2.3827590	-10.5934250	1.1546380
C	-2.5652090	-8.4769860	1.3591150
H	-3.4114820	-8.4153420	0.6863660
C	-3.8938370	-4.9704660	-0.1981400
C	-5.1156000	-6.4615130	1.2516410
C	-6.2617610	-6.2995190	0.4045340
C	-6.1752200	-5.4673600	-0.7427760
H	-7.0481110	-5.3527610	-1.3777880
C	-5.0152670	-4.7908150	-1.0302230
H	-4.9303720	-4.1260960	-1.8822170
C	-6.4581660	-7.8369930	2.7477180
H	-6.5471300	-8.4138630	3.6629700
C	-7.5750390	-7.7113460	1.8879780
H	-8.5095100	-8.2020540	2.1409150
C	-7.4766130	-6.9528330	0.7446620
H	-8.3319370	-6.8293010	0.0867050
C	-5.2618130	-7.2290290	2.4394030
H	-4.4193600	-7.3260710	3.1123320
C	-0.8387550	-4.9644870	3.0239990
H	-0.3970060	-4.0437340	3.3840150
O	-2.7328480	-4.2662800	-0.5533090
O	-2.4701650	-3.6098450	1.8978880
P	-2.2021930	-3.0699750	0.3911750
O	-3.0482740	-1.8068700	0.2371710
O	-0.7102600	-2.9645700	0.0665230
C	1.4040210	4.9046130	0.3934870
C	2.2000940	4.0790780	-0.5532990
C	1.6675390	2.9089730	-1.0641930
C	3.5612300	2.4787550	-2.4633860
H	4.0786160	1.8824430	-3.2086810
C	4.2054570	3.6296470	-1.9352320
C	3.5349490	4.4312580	-0.9522780
C	5.5184840	3.9862400	-2.3439460
H	6.0088310	3.3770190	-3.0977800
C	6.1627560	5.0676880	-1.7889280
H	7.1678750	5.3278040	-2.1053580
C	5.5192460	5.8333780	-0.7878880
H	6.0409860	6.6694780	-0.3327560
C	4.2407990	5.5243700	-0.3801380
H	3.7648720	6.1132590	0.3940700
C	0.8591910	4.3386000	1.5332250
C	1.1277640	6.2942720	0.1476580
C	0.4123190	7.0542790	1.1315820
C	-0.0547580	6.4144790	2.3100770
H	-0.5936660	6.9990300	3.0491060
C	0.1428130	5.0689590	2.5004660
H	-0.2288720	4.5486650	3.3757360
C	1.2262400	8.2776940	-1.2627690
H	1.5177900	8.7505470	-2.1953750
C	0.5595040	9.0362050	-0.2715850
H	0.3551430	10.0883190	-0.4426570
C	0.1567680	8.4318980	0.8966510
H	-0.3757720	8.9960120	1.6568690
C	1.5024260	6.9440530	-1.0599470
H	2.0038810	6.3745690	-1.8320340
C	2.3173420	2.1028230	-2.0180380
H	1.8158870	1.2104780	-2.3715310
O	1.0418200	2.9711800	1.7916950
O	0.3994190	2.4948910	-0.6299950
P	0.2982800	1.8776060	0.8667860
O	-1.1937230	1.7834500	1.1848030
O	1.1363830	0.6084080	1.0359340

Rh_2(*S*-BNP)$_4$, (*S*-2) [B3LYP]

Rh	-1.4545420	1.7090950	0.1464650
Rh	-1.4545370	1.7090800	2.6724180
P	1.0142500	3.1309700	1.4080920
P	-0.0326620	-0.7596510	1.4080840
O	0.4503420	2.5495330	0.1103230
O	0.4160950	2.6119270	2.7173420
O	0.9013240	4.7433950	1.2563190
O	2.6054380	2.8805550	1.5529270
O	-0.6143330	-0.1958800	0.1103490
O	-0.5518570	-0.1616510	2.7173510
O	-0.2826490	-2.3508830	1.5528820
O	1.5797280	-0.6462410	1.2562730
C	2.9831980	5.6426880	2.0665490
C	3.6922320	4.9519430	0.9511870
C	3.4927470	3.5979060	0.7341160
C	5.0591330	3.4997000	-1.0804510
H	5.5991670	2.9444860	-1.8416100
C	5.2592690	4.9023680	-0.9759620
C	4.5572500	5.6472120	0.0313650
C	6.1225420	5.5853570	-1.8758500
H	6.6542210	5.0039450	-2.6241240

C	6.2707720	6.9520070	-1.8154420
H	6.9282420	7.4636950	-2.5116600
C	5.5471910	7.6946480	-0.8523570
H	5.6436940	8.7759380	-0.8240170
C	4.7153490	7.0615950	0.0456040
H	4.1618660	7.6492730	0.7678820
C	1.6059150	5.5380700	2.1742760
C	3.6706140	6.3981380	3.0829010
C	2.9052810	7.0974210	4.0767780
C	1.4871910	7.0045940	4.0674140
H	0.9183600	7.5456080	4.8178970
C	0.8426890	6.2174270	3.1450890
H	-0.2357750	6.1052800	3.1320460
C	5.7131510	7.1735160	4.1683600
H	6.7976960	7.1874970	4.2221900
C	4.9553590	7.8952790	5.1209940
H	5.4603210	8.4692510	5.8919700
C	3.5807560	7.8512670	5.0752500
H	2.9856780	8.3831460	5.8125820
C	0.4349990	-3.2380460	0.7341290
C	1.7890900	-3.4370890	0.9511950
C	2.4845950	-4.3019570	0.0313900
C	1.7399350	-5.0042990	-0.9758440
C	0.3371830	-4.8046420	-1.0802710
H	-0.2178950	-5.3449620	-1.8413260
C	-0.3060590	-3.9162680	-0.2545470
H	-1.3699800	-3.7225820	-0.3338650
C	3.8990260	-4.4595830	0.0455750
H	4.4865490	-3.9058770	0.7678090
C	4.5323180	-5.2912630	-0.8523750
H	5.6136410	-5.3874050	-0.8240850
C	3.7898730	-6.0151400	-1.8153800
H	4.3017490	-6.6724520	-2.5116070
C	2.4231640	-5.8673840	-1.8757220
H	1.8419000	-6.3993070	-2.6239370
C	2.4796550	-2.7278300	2.0665230
C	2.3746290	-1.3505700	2.1742260
C	3.0537630	-0.5871350	3.1450240
H	2.9413090	0.4912980	3.1319720
C	3.8411250	-1.2314030	4.0673510
H	4.3819590	-0.6624010	4.8178350
C	3.9343880	-2.6494560	4.0767240
C	3.2353290	-3.4150120	3.0828540
C	4.6884720	-3.3246900	5.0751820
H	5.2201790	-2.7294430	5.8125030
C	4.7329330	-4.6992770	5.1209250
H	5.3070870	-5.2040470	5.8918920
C	4.0113900	-5.4573010	4.1683080
H	4.0257200	-6.5418410	4.2221280
C	3.2844340	-4.8341090	3.1771600
H	2.7298580	-5.4316410	2.4636560
C	5.0897230	6.4467900	3.1771900
H	5.6870640	5.8920530	2.4636500
C	4.1706330	2.8566940	-0.2546700
H	3.9766040	1.7928360	-0.3340430
P	-3.9233300	0.2872060	1.4080850
P	-2.8764190	4.1778260	1.4081180
O	-3.3594270	0.8686580	0.1103200
O	-3.3251700	0.8062330	2.7173390
O	-3.8104080	-1.3252180	1.2562950
O	-5.5145180	0.5376150	1.5529280
O	-2.2947520	3.6140700	0.1103750
O	-2.3572170	3.5798110	2.7173770
O	-2.6264330	5.7690570	1.5529330
O	-4.4888090	4.0644190	1.2563130
C	-5.8922820	-2.2244900	2.0665440
C	-6.6012840	-1.5337730	0.9511390
C	-6.4018140	-0.1797280	0.7340980
C	-7.9681080	-0.0815140	-1.0805430
H	-8.5081130	0.4737040	-1.8417200
C	-8.1681560	-1.4842020	-0.9761500
C	-7.4661550	-2.2290600	0.0311820
C	-9.0312990	-2.1671910	-1.8761620
H	-9.5629700	-1.5857640	-2.6244300
C	-9.1793960	-3.5338610	-1.8158960
H	-9.8367590	-4.0455500	-2.5122130
C	-8.4557920	-4.2765190	-0.8528430
H	-8.5521730	-5.3578230	-0.8246250
C	-7.6240820	-3.6434670	0.0452400
H	-7.0705760	-4.2311660	0.7674820
C	-4.5149960	-2.1198850	2.1742600
C	-6.5797030	-2.9798170	3.0829940
C	-5.8143630	-3.6790790	4.0768820
C	-4.3962690	-3.5863280	4.0674550
H	-3.8274370	-4.1273150	4.8179570
C	-3.7517670	-2.7992150	3.1450870
H	-2.6733010	-2.6870840	3.1320250
C	-8.6222290	-3.7549180	4.1686870
H	-9.7067710	-3.7687750	4.2226190
C	-7.8644310	-4.4767130	5.1212900
H	-8.3693840	-5.0505950	5.8923380
C	-6.4898280	-4.4328150	5.0754440
H	-5.8947410	-4.9646980	5.8127660
C	-3.3440860	6.6562180	0.7341820
C	-4.6981790	6.8552460	0.9512440
C	-5.3937090	7.7200240	0.0313660
C	-4.6490540	8.4223550	-0.9758800
C	-3.2462910	8.2227550	-1.0802670

H	-2.6912210	8.7630590	-1.8413380
C	-2.6030350	7.3344260	-0.2545080
H	-1.5391090	7.1407570	-0.3338140
C	-6.8081540	7.8775450	0.0454610
H	-7.3956800	7.3238240	0.7676810
C	-7.4414580	8.7091410	-0.8525560
H	-8.5227900	8.8052060	-0.8243280
C	-6.6990170	9.4330310	-1.8155530
H	-7.2109040	10.0902780	-2.5118340
C	-5.3322960	9.2853600	-1.8758250
H	-4.7510320	9.8172830	-2.6240400
C	-5.3887130	6.1460000	2.0666040
C	-5.2836940	4.7687370	2.1742860
C	-5.9627970	4.0052930	3.1450980
H	-5.8503570	2.9268590	3.1320220
C	-6.7501080	4.6495550	4.0674710
H	-7.2909130	4.0805460	4.8179710
C	-6.8433180	6.0676100	4.0768990
C	-6.1442850	6.8331780	3.0830190
C	-7.5973070	6.7428320	5.0754390
H	-8.1289980	6.1475740	5.8127620
C	-7.6416880	8.1174170	5.1212600
H	-8.2157660	8.6221780	5.8922910
C	-6.9201460	8.8754520	4.1686510
H	-6.9344010	9.9599900	4.2225390
C	-6.1932860	8.2522730	3.1774250
H	-5.6387050	8.8498170	2.4639350
C	-7.9988110	-3.0283070	3.1774260
H	-8.5961630	-2.4735280	2.4639280
C	-7.0796720	0.5614940	-0.2546980
H	-6.8856630	1.6253580	-0.3340360

Rh$_2$(*S*-(4,4’-diPh)-BNP)$_4$ [B3LYP-D3(BJ)]

C	-0.9440040	-0.6611140	6.6854540
C	0.3516760	-0.4842980	5.9783070
C	0.3786750	0.2042580	4.7831490
C	2.7478790	-0.0866050	4.4523990
C	2.8043420	-0.8109980	5.6904910
C	1.5976210	-1.0336220	6.4417030
C	4.0296000	-1.3097340	6.2097110
H	4.9451310	-1.1008530	5.6696750
C	4.0698230	-2.0394620	7.3763250
H	5.0182330	-2.4055850	7.7563980
C	2.8731410	-2.3242710	8.0720370
H	2.9010770	-2.9300830	8.9722750
C	1.6703610	-1.8338040	7.6143020
H	0.7587700	-2.0549750	8.1546550
C	-2.0384670	-1.2020700	6.0321490
C	-1.1401430	-0.2289330	8.0424270
C	-2.3884980	-0.4773070	8.7086820
C	-3.4688130	-1.0813140	7.9828210
C	-3.2759340	-1.4211360	6.6587410
H	-4.0814500	-1.8457170	6.0708120
C	-0.2942710	0.8429740	10.0632700
H	0.4957060	1.3802530	10.5786720
C	-1.4887020	0.5198800	10.7451790
H	-1.6045270	0.7843060	11.7915020
C	-2.5094870	-0.1194820	10.0798140
H	-3.4272980	-0.3587900	10.6022600
C	-0.1246430	0.4723360	8.7491670
H	0.7960330	0.7179090	8.2355010
C	1.5386400	0.4240200	4.0313790
H	1.4425540	0.9217930	3.0769860
O	-1.9590230	-1.6094810	4.6948220
O	-0.8390180	0.6252940	4.2108860
P	-1.6254180	-0.5936040	3.4836090
O	-2.8868870	-0.0142580	2.8482960
O	-0.6987120	-1.3950440	2.5688910
Rh	-2.8310760	0.3397170	0.7891730
Rh	-0.5749810	-0.7892950	0.6082700
C	-1.6959980	-0.1938170	-5.3480710
C	-0.9711180	-1.3740380	-4.8079470
C	-1.3382980	-1.8864980	-3.5807010
C	0.3426980	-3.6132930	-3.5888160
C	0.7819360	-3.1422570	-4.8717860
C	0.1405590	-2.0017100	-5.4700200
C	1.8345410	-3.7796410	-5.5828020
H	2.2853930	-4.6676730	-5.1567360
C	2.2798680	-3.2945360	-6.7917250
H	3.0821350	-3.8011900	-7.3186510
C	1.7012300	-2.1278010	-7.3404340
H	2.0767280	-1.7253840	-8.2759940
C	0.6594340	-1.5000120	-6.6945790
H	0.2198860	-0.6083320	-7.1231600
C	-1.8244500	0.9609750	-4.5952520
C	-2.3440450	-0.2140530	-6.6309680
C	-2.9833660	0.9684510	-7.1380550
C	-3.0458970	2.1447570	-6.3187500
C	-2.4745870	2.1145500	-5.0622670
H	-2.5346830	2.9730270	-4.4033690
C	-2.9722940	-1.4053940	-8.6641810
H	-2.9996040	-2.3242200	-9.2415640
C	-3.5232130	-0.2195300	-9.1992560
H	-3.9483270	-0.2214240	-10.1979560
C	-3.5311970	0.9346410	-8.4500760
H	-3.9613080	1.8403430	-8.8591000
C	-2.3937650	-1.3989130	-7.4158760
H	-1.9680120	-2.3100520	-7.0154050

C	-0.7217760	-2.9877790	-2.9754970
H	-1.0441720	-3.2732500	-1.9842790
O	-1.2661880	1.0576510	-3.3144680
O	-2.3168920	-1.2096700	-2.8245920
P	-1.7176000	0.1067950	-2.0888750
O	-2.8430300	0.7243230	-1.2620550
O	-0.4118510	-0.2046160	-1.3561680
C	-4.0112260	-5.8142130	0.6216680
C	-2.6749100	-5.7816230	1.2722070
C	-2.2643050	-4.6298850	1.9113430
C	-0.1163700	-5.5221840	2.5422820
C	-0.4713250	-6.7570860	1.9022170
C	-1.7431280	-6.8766940	1.2402300
C	0.3984700	-7.8809690	1.9102680
H	1.3347350	-7.8087000	2.4502850
C	0.0703250	-9.0462620	1.2548710
H	0.7486220	-9.8932060	1.2790070
C	-1.1443660	-9.1347330	0.5379890
H	-1.3860110	-10.0408600	-0.0085850
C	-2.0257770	-8.0765440	0.5323920
H	-2.9555490	-8.1537400	-0.0167540
C	-4.3727960	-4.8455790	-0.2991940
C	-5.0022030	-6.7998640	0.9577270
C	-6.2582260	-6.8309700	0.2609420
C	-6.5515210	-5.8185740	-0.7125990
C	-5.6087940	-4.8422630	-0.9655560
H	-5.8117080	-4.0422590	-1.6680980
C	-5.7104510	-8.7193460	2.2851810
H	-5.5186200	-9.4278210	3.0849570
C	-6.9079420	-8.8038720	1.5402600
H	-7.6224130	-9.5937000	1.7496550
C	-7.1736830	-7.8781900	0.5574250
H	-8.0958530	-7.9399030	-0.0068520
C	-4.7816670	-7.7463250	1.9960140
H	-3.8637980	-7.6920750	2.5673010
C	-1.0268840	-4.4870570	2.5498310
H	-0.7744610	-3.5226840	2.9673100
O	-3.4913920	-3.8176070	-0.6556880
O	-3.0775360	-3.4807650	1.8345160
P	-2.9384020	-2.7295910	0.4028200
O	-3.8101480	-1.4771480	0.4532260
O	-1.4726980	-2.5381020	0.0102150
C	1.3848950	4.9732290	0.6929160
C	2.0669040	3.9306390	-0.1182390
C	1.3123770	2.9481610	-0.7255970
C	3.2128720	1.8220790	-1.6893880
C	4.0670020	2.8082140	-1.0904930
C	3.4937630	3.8493620	-0.2796010
C	5.4749770	2.7948910	-1.2828950
H	5.9028580	2.0407530	-1.9322420
C	6.2914520	3.7171090	-0.6680410
H	7.3637380	3.6898180	-0.8336500
C	5.7342600	4.6917020	0.1903960
H	6.3819170	5.3962660	0.7025830
C	4.3712970	4.7539640	0.3777870
H	3.9524230	5.5052890	1.0350910
C	0.5200150	4.6208020	1.7150220
C	1.5373390	6.3763740	0.4197760
C	0.9082590	7.3516770	1.2666060
C	0.0555350	6.9152350	2.3348830
C	-0.1291350	5.5612500	2.5310570
H	-0.7944150	5.1972220	3.3054220
C	2.4712500	8.1782160	-0.9328200
H	3.0441330	8.5016300	-1.7962880
C	1.9260100	9.1361820	-0.0490730
H	2.1035890	10.1935040	-0.2184030
C	1.1602560	8.7293550	1.0193780
H	0.7363780	9.4657110	1.6907210
C	2.2854710	6.8350750	-0.6993360
H	2.7118720	6.1072120	-1.3777000
C	1.8496540	1.9202670	-1.5092220
H	1.1787050	1.1644570	-1.8916520
O	0.2717070	3.2781740	2.0258030
O	-0.0727950	2.8951130	-0.4679930
P	-0.4012400	2.2451210	0.9821390
O	-1.9203580	2.1847370	1.1267370
O	0.3655430	0.9400790	1.2000800
C	1.2168700	-5.2552500	3.1391980
C	1.3058050	-4.6908770	4.4218400
C	2.3969400	-5.4443350	2.4008900
C	2.5407720	-4.3370710	4.9608380
H	0.3977380	-4.5275010	4.9942160
C	3.6330560	-5.0882370	2.9406160
H	2.3377980	-5.8347710	1.3905330
C	3.7093650	-4.5391260	4.2230880
H	2.5904530	-3.9018870	5.9515760
H	4.5352560	-5.2291460	2.3527740
H	4.6705890	-4.2624250	4.6456190
C	-7.8446640	-5.7612970	-1.4421850
C	-7.8536450	-5.7006450	-2.8440990
C	-9.0693470	-5.7092770	-0.7568030
C	-9.0557660	-5.6021750	-3.5444900
H	-6.9114940	-5.7426010	-3.3819870
C	-10.2704670	-5.6086290	-1.4575270
H	-9.0750360	-5.7289410	0.3281540
C	-10.2679930	-5.5576280	-2.8533720
H	-9.0448690	-5.5628520	-4.6294780
H	-11.2082240	-5.5620340	-0.9122820

H	-11.2040980	-5.4806850	-3.3978130
C	3.9085860	0.0842900	3.5419780
C	4.2049610	1.3537100	3.0201170
C	4.6490950	-1.0212750	3.0915790
C	5.2233950	1.5194310	2.0839870
H	3.6279080	2.2118730	3.3510710
C	5.6676870	-0.8547220	2.1529980
H	4.3987170	-2.0136890	3.4510000
C	5.9612020	0.4155770	1.6506460
H	5.4373820	2.5059400	1.6907050
H	6.2231870	-1.7207300	1.8053890
H	6.7545220	0.5478120	0.9211040
C	-4.8005560	-1.3319220	8.5922470
C	-5.3656130	-2.6149890	8.5321740
C	-5.5410490	-0.2957470	9.1841180
C	-6.6330980	-2.8599530	9.0602060
H	-4.7990190	-3.4223250	8.0784070
C	-6.8088620	-0.5409150	9.7098910
H	-5.1269300	0.7068360	9.2135760
C	-7.3578130	-1.8240080	9.6524390
H	-7.0525260	-3.8602170	9.0110690
H	-7.3712990	0.2727850	10.1575550
H	-8.3445020	-2.0138120	10.0635940
C	1.0192200	-4.6969050	-2.8317390
C	0.2579030	-5.7302900	-2.2624700
C	2.3984270	-4.6499910	-2.5688060
C	0.8582400	-6.7010910	-1.4636510
H	-0.8112820	-5.7664500	-2.4481120
C	2.9986210	-5.6216520	-1.7677230
H	2.9892030	-3.8297920	-2.9625560
C	2.2321650	-6.6521260	-1.2173590
H	0.2550540	-7.4905480	-1.0316940
H	4.0637940	-5.5652290	-1.5638290
H	2.6977190	-7.4100660	-0.5945270
C	-3.7282660	3.3896240	-6.7574080
C	-3.0436040	4.6137920	-6.7153380
C	-5.0724730	3.3821340	-7.1641320
C	-3.6814700	5.7989080	-7.0813640
H	-2.0035310	4.6284060	-6.4042790
C	-5.7100580	4.5674870	-7.5278040
H	-5.6212980	2.4460800	-7.1759830
C	-5.0161670	5.7791320	-7.4904570
H	-3.1348580	6.7364830	-7.0491750
H	-6.7518500	4.5454050	-7.8328150
H	-5.5133940	6.7012400	-7.7752640
C	3.7131770	0.6415150	-2.4387490
C	3.1513570	0.3171150	-3.6841270
C	4.6584980	-0.2319650	-1.8760570
C	3.5332440	-0.8404480	-4.3588820
H	2.4087570	0.9786040	-4.1199260
C	5.0395000	-1.3915970	-2.5516400
H	5.0631190	-0.0181520	-0.8925460
C	4.4827580	-1.6964070	-3.7963890
H	3.0880140	-1.0756650	-5.3181560
H	5.7626600	-2.0630490	-2.0982700
H	4.7786650	-2.5979830	-4.3243160
C	-0.6622690	7.8648780	3.2240980
C	-0.5355810	7.7497010	4.6168740
C	-1.5172880	8.8499780	2.7036510
C	-1.2357730	8.6042190	5.4682690
H	0.1250650	6.9923860	5.0276710
C	-2.2184800	9.7024400	3.5553810
H	-1.6446400	8.9326470	1.6291830
C	-2.0779020	9.5845080	4.9400570
H	-1.1210440	8.5055080	6.5434240
H	-2.8810730	10.4539890	3.1370560
H	-2.6233190	10.2499360	5.6021450

Rh$_2$(*S*-(4,4'-diPh)-BNP)$_4$ [B3LYP]

Rh	14.7957570	7.5876180	-0.5217710
Rh	14.7966460	7.5869680	2.0094220
P	17.2737580	8.9669140	0.7498920
P	16.1757930	5.1096510	0.7493600
O	16.7101760	8.4090170	-0.5578730
O	16.7233830	8.3709760	2.0482860
O	17.0909610	10.5787170	0.6688800
O	18.8763830	8.7769120	0.8539220
O	15.6172240	5.6732620	-0.5581010
O	15.5809030	5.6603490	2.0480910
O	15.9853610	3.5070940	0.8537340
O	17.7875930	5.2919270	0.6671560
C	19.1320530	11.5210050	1.5259900
C	19.8612410	10.9295580	0.3683030
C	19.7195300	9.5848270	0.0743620
C	21.2570820	9.6491420	-1.7968070
C	21.4117320	11.0622820	-1.5840200
C	20.6837430	11.7069980	-0.5228080
C	22.2742250	11.8544830	-2.3934260
H	22.8660870	11.3662130	-3.1582960
C	22.3656090	13.2178060	-2.2259680
C	21.5870560	13.8608300	-1.2380070
H	21.6320900	14.9403960	-1.1295030
C	20.7752410	13.1226640	-0.4064080
H	20.1860290	13.6256480	0.3503920
C	17.7639190	11.3483790	1.6304220
C	19.7811080	12.2425470	2.5894010
C	18.9969130	12.8468520	3.6346590
C	17.5702290	12.6664760	3.6507990

C	16.9832440	11.9115230	2.6535130
H	15.9166290	11.7172150	2.6504500
C	21.8146530	13.0687760	3.6603800
H	22.8984120	13.1301970	3.6921900
C	21.0411330	13.7314140	4.6393340
C	19.6691740	13.6176880	4.6248760
H	19.0807660	14.1212970	5.3824110
C	16.7923200	2.6636200	0.0735480
C	18.1372540	2.5214830	0.3663450
C	18.9136530	1.6986660	-0.5253860
C	18.2677760	0.9708090	-1.5859850
C	16.8545020	1.1259040	-1.7975570
C	16.1486400	1.9781710	-0.9707460
H	15.0891770	2.1511750	-1.1233660
C	20.3293910	1.6067130	-0.4102150
H	20.8332290	2.1958200	0.3460990
C	21.0665660	0.7945930	-1.2423950
H	22.1462120	0.7492170	-1.1348300
C	20.4224280	0.0161760	-2.2297360
C	19.0589900	0.1079930	-2.3960140
H	18.5698610	-0.4837640	-3.1604160
C	18.7299400	3.2505310	1.5234910
C	18.5578710	4.6187300	1.6280260
C	19.1222880	5.3992650	2.6505280
H	18.9284310	6.4659660	2.6475540
C	19.8779600	4.8120610	3.6471380
C	20.0577440	3.3853040	3.6309490
C	19.4522000	2.6012720	2.5862900
C	20.8291850	2.7128160	4.6205410
H	21.3336710	3.3010850	5.3776010
C	20.9423960	1.3408150	4.6349920
C	20.2786100	0.5674830	3.6566670
H	20.3396510	-0.5162970	3.6884910
C	19.5603050	1.1839400	2.6568100
H	19.0600150	0.5823850	1.9076780
C	21.1984030	12.3511220	2.6599290
H	21.8001000	11.8517230	1.9103170
C	20.4051170	8.9422780	-0.9705370
H	20.2324560	7.8828900	-1.1240610
P	12.3185560	6.2077260	0.7507600
P	13.4167210	10.0650640	0.7512800
O	12.8812980	6.7662340	-0.5571030
O	12.8699720	6.8028610	2.0490810
O	12.5009570	4.5959340	0.6686780
O	10.7160540	6.3980030	0.8561690
O	13.9741680	9.5019850	-0.5568800
O	14.0124800	9.5135770	2.0492850
O	13.6076050	11.6675230	0.8563760
O	11.8048090	9.8832070	0.6703620
C	10.4603940	3.6535490	1.5269730
C	9.7302880	4.2458270	0.3702880
C	9.8720080	5.5906950	0.0769720
C	8.3324750	5.5278270	-1.7926210
C	8.1777780	4.1145860	-1.5805390
C	8.9067420	3.4690850	-0.5204740
C	7.3142880	3.3230450	-2.3895290
H	6.7217240	3.8118960	-3.1534830
C	7.2228180	1.9596370	-2.2228090
C	8.0022770	1.3158620	-1.2360550
H	7.9571560	0.2362380	-1.1281670
C	8.8150980	2.0533650	-0.4048490
H	9.4050030	1.5498050	0.3510280
C	11.8286570	3.8258180	1.6303150
C	9.8121230	2.9314960	2.5905230
C	10.5971130	2.3263270	3.6346830
C	12.0238560	2.5063320	3.6496430
C	12.6101160	3.2618190	2.6523380
H	13.6767750	3.4558710	2.6484480
C	7.7793460	2.1051040	3.6628380
H	6.6956010	2.0439410	3.6956000
C	8.5535890	1.4416440	4.6406620
C	9.9255630	1.5550300	4.6250230
H	10.5145370	1.0507950	5.3817020
C	12.7995050	12.5119870	0.0784770
C	11.4550390	12.6539890	0.3735080
C	10.6774190	13.4780810	-0.5159810
C	11.3217950	14.2072360	-1.5765970
C	12.7347200	14.0521890	-1.7905160
C	13.4416910	13.1987130	-0.9659030
H	14.5008980	13.0257600	-1.1203460
C	9.2618720	13.5701090	-0.3985330
H	8.7590880	12.9800800	0.3577650
C	8.5235620	14.3834550	-1.2285060
H	7.4440890	14.4288640	-1.1192330
C	9.1663230	15.1630790	-2.2157920
C	10.5294910	15.0712580	-2.3842700
H	11.0175520	15.6639570	-3.1486240
C	10.8639520	11.9236160	1.5306300
C	11.0359280	10.5552620	1.6331830
C	10.4729700	9.7735330	2.6555690
H	10.6667250	8.7068210	2.6510110
C	9.7189120	10.3596110	3.6540660
C	9.5393130	11.7864160	3.6399210
C	10.1434080	12.5716620	2.5953330
C	8.7694660	12.4577920	4.6315070
H	8.2659970	11.8686600	5.3885730
C	8.6565260	13.8297950	4.6478630
C	9.3189910	14.6042460	3.6695280

H	9.2581990	15.6879950	3.7028180
C	10.0356710	13.9889230	2.6678070
H	10.5349390	14.5913330	1.9186810
C	8.3948650	2.8232370	2.6622790
H	7.7926080	3.3232690	1.9135410
C	9.1854710	6.2340070	-0.9668330
H	9.3581800	7.2934550	-1.1198890
C	12.9008010	1.9373430	4.7128340
C	13.9970780	1.1334260	4.3584170
C	12.6988540	2.2347970	6.0719530
C	14.8597210	0.6311860	5.3336630
H	14.1656780	0.8966970	3.3119350
C	13.5636060	1.7346110	7.0460710
H	11.8731250	2.8768690	6.3629660
C	14.6447990	0.9286460	6.6810350
H	15.6992500	0.0079810	5.0395330
H	13.3963480	1.9821090	8.0903820
H	15.3165680	0.5394320	7.4404770
C	7.6326260	6.2632620	-2.8847280
C	7.7909170	5.9016390	-4.2338900
C	6.8401600	7.3820360	-2.5787350
C	7.1677450	6.6342260	-5.2446920
H	8.4205430	5.0554300	-4.4913920
C	6.2144390	8.1121590	-3.5902680
H	6.7079140	7.6714220	-1.5402220
C	6.3751700	7.7398810	-4.9265370
H	7.3083220	6.3447560	-6.2820940
H	5.6005480	8.9704970	-3.3330380
H	5.8896340	8.3083000	-5.7143470
C	9.1510280	9.4824140	4.7176370
C	8.3463640	8.3864900	4.3638240
C	9.4502650	9.6837570	6.0764550
C	7.8451260	7.5236120	5.3393770
H	8.1082650	8.2183530	3.3175790
C	8.9510790	8.8187700	7.0508770
H	10.0929310	10.5092000	6.3669710
C	8.1443520	7.7379400	6.6864530
H	7.2213180	6.6843660	5.0457180
H	9.1999520	8.9855620	8.0949350
H	7.7559190	7.0659860	7.4461320
C	13.4689710	14.7521800	-2.8833270
C	13.1055410	14.5944850	-4.2320730
C	14.5884000	15.5441750	-2.5785110
C	13.8370120	15.2177800	-5.2436070
H	12.2587970	13.9652220	-4.4886970
C	15.3174080	16.1700190	-3.5907730
H	14.8791820	15.6759590	-1.5403300
C	14.9433350	16.0098860	-4.9266130
H	13.5461410	15.0776670	-6.2806810
H	16.1762780	16.7835430	-3.3344430
H	15.5108820	16.4955200	-5.7149910
C	20.4474020	5.6880120	4.7109040
C	21.2515620	6.7843410	4.3572070
C	20.1501690	5.4850480	6.0699200
C	21.7542570	7.6460440	5.3330510
H	21.4881240	6.9537180	3.3108130
C	20.6508080	6.3488620	7.0446360
H	19.5079270	4.6592600	6.3603920
C	21.4570150	7.4301150	6.6803090
H	22.3776560	8.4856230	5.0394740
H	20.4034800	6.1808260	8.0888620
H	21.8465850	8.1011520	7.4402150
C	16.1187150	0.4272750	-2.8902080
C	16.4800340	0.5869400	-4.2392900
C	14.9999120	-0.3653650	-2.5847800
C	15.7471210	-0.0350700	-5.2505720
H	17.3262630	1.2167250	-4.4963310
C	14.2694630	-0.9899250	-3.5967960
H	14.7107560	-0.4986640	-1.5463380
C	14.6414390	-0.8278340	-4.9329860
H	16.0363590	0.1065650	-6.2878960
H	13.4111050	-1.6039740	-3.3400070
H	14.0727640	-1.3124620	-5.7211700
C	16.6941130	13.2344970	4.7151890
C	15.5972140	14.0382530	4.3623350
C	16.8974600	12.9362470	6.0739260
C	14.7353260	14.5395690	5.3387230
H	15.4275290	14.2755850	3.3161650
C	16.0334680	13.4355150	7.0491880
H	17.7236850	12.2942720	6.3637430
C	14.9516440	14.2413330	6.6857000
H	13.8952880	15.1626510	5.0457880
H	16.2018130	13.1874110	8.0931800
H	14.2804660	14.6298270	7.4460320
C	21.9558510	8.9145460	-2.8901690
C	21.7959480	9.2770170	-4.2389140
C	22.7488850	7.7957310	-2.5858090
C	22.4181060	8.5452090	-5.2509050
H	21.1658650	10.1232710	-4.4951430
C	23.3735910	7.0663880	-3.5985340
H	22.8823740	7.5056940	-1.5476370
C	23.2112580	7.4395020	-4.9343760
H	22.2762860	8.8353280	-6.2879570
H	23.9879430	6.2080020	-3.3425650
H	23.6960000	6.8716900	-5.7231110
H	8.0653420	14.3168660	5.4174900
H	8.0663780	0.8493430	5.4093400
H	21.0035480	-0.6471250	-2.8632580

H	6.5589830	1.3782640	-2.8555390
H	23.0286720	13.7996940	-2.8590330
H	8.5843500	15.8273220	-2.8475410
H	21.5348160	0.8528770	5.4031190
H	21.5288950	14.3233520	5.4079430

Rh_2(*S*-(4,4',6,6'-tetra-Ph)BNP)$_4$, (*S*-6a) [B3LYP-D3(BJ)]

Rh	-0.4926108	1.4039409	0.0000000
Rh	-0.4979248	1.4093439	-2.5244910
P	2.0307772	2.6327189	-1.3034470
P	0.7308162	-1.1192221	-1.3083770
O	1.4254452	2.2234509	0.0372820
O	1.4899522	1.9302699	-2.5499000
O	1.9033352	4.2461899	-1.4269080
O	3.6235452	2.3685719	-1.3589790
O	0.3270382	-0.5140111	0.0340830
O	0.0233792	-0.5784221	-2.5520190
O	0.4666302	-2.7120131	-1.3630910
O	2.3437462	-0.9915421	-1.4381520
C	3.7421102	4.8115759	-2.8658250
C	4.6642512	4.5281959	-1.7351190
C	4.5720882	3.3423439	-1.0256340
C	6.4277782	3.8821109	0.4350650
C	6.5573642	5.1443819	-0.2366760
C	5.6427972	5.4801039	-1.2909230
C	7.5699602	6.0750999	0.1094820
H	8.2768232	5.8037129	0.8826550
C	7.6641402	7.3211669	-0.4852790
C	6.6964002	7.6726169	-1.4650110
H	6.7427452	8.6495049	-1.9346440
C	5.7270862	6.7820239	-1.8558040
H	5.0134982	7.0710849	-2.6167850
C	2.3794642	4.7200549	-2.6663330
C	4.1851012	5.1294139	-4.1949020
C	3.2229012	5.3843679	-5.2318670
C	1.8201972	5.2792779	-4.9428950
C	1.4239952	4.9651879	-3.6605800
H	0.3813502	4.8169959	-3.4167350
C	5.9749942	5.4672739	-5.8173220
H	7.0358472	5.4943019	-6.0432750
C	5.0353102	5.7729629	-6.8387960
C	3.6884282	5.7275409	-6.5251010
H	2.9576172	5.9255659	-7.2986500
C	1.4417182	-3.6603011	-1.0327880
C	2.6250152	-3.7525271	-1.7465160
C	3.5786942	-4.7307411	-1.3053760
C	3.2469492	-5.6448611	-0.2494760
C	1.9871052	-5.5151921	0.4267940
C	1.1154462	-4.5221181	0.0270530
H	0.1698052	-4.3750911	0.5359200
C	4.8785582	-4.8151101	-1.8749580
H	5.1647402	-4.1018581	-2.6373380
C	5.7707352	-5.7840751	-1.4869140
H	6.7459222	-5.8304881	-1.9600670
C	5.4230202	-6.7513661	-0.5054110
C	4.1790952	-6.6571191	0.0938040
H	3.9106602	-7.3636351	0.8683220
C	2.9040922	-2.8307041	-2.8785550
C	2.8130252	-1.4680061	-2.6791340
C	3.0542192	-0.5127981	-3.6745690
H	2.9067132	0.5298779	-3.4304620
C	3.3635462	-0.9093161	-4.9579500
C	3.4678472	-2.3120741	-5.2468890
C	3.2170672	-3.2740151	-4.2086770
C	3.8061602	-2.7778991	-6.5413090
H	4.0009902	-2.0472421	-7.3158140
C	3.8507112	-4.1248561	-6.8547780
C	3.5490832	-5.0643171	-5.8318680
H	3.5754502	-6.1252221	-6.0576410
C	3.2439062	-4.6515561	-4.5567000
H	3.0219432	-5.3898021	-3.7967440
C	5.5625312	5.1572989	-4.5432070
H	6.3009692	4.9323369	-3.7843200
C	5.4343412	3.0121039	0.0326300
H	5.2873732	2.0646539	0.5381400
P	-3.0212868	0.1803309	-1.2966500
P	-1.7219528	3.9325009	-1.2917370
O	-2.4105458	0.5844039	0.0431980
O	-2.4858098	0.8880609	-2.5423760
O	-2.8937548	-1.4325701	-1.4271310
O	-4.6143438	0.4441389	-1.3446540
O	-1.3124618	3.3217549	0.0464640
O	-1.0186418	3.3974559	-2.5401530
O	-1.4590238	5.5256649	-1.3403650
O	-3.3352658	3.8042749	-1.4164070
C	-4.7385028	-1.9930771	-2.8603890
C	-5.6559748	-1.7143311	-1.7247440
C	-5.5611788	-0.5312021	-1.0110580
C	-7.4106418	-1.0771271	0.4552340
C	-7.5426958	-2.3368201	-0.2208510
C	-6.6324208	-2.6682181	-1.2801710
C	-8.5536248	-3.2691341	0.1258810
H	-9.2573518	-3.0009281	0.9030140
C	-8.6499608	-4.5129131	-0.4733040
C	-7.6862028	-4.8603171	-1.4583890
H	-7.7342458	-5.8354011	-1.9315910
C	-6.7187388	-3.9679611	-1.8497430

H	-6.0082408	-4.2538851	-2.6147880
C	-3.3750458	-1.9018551	-2.6663200
C	-5.1869998	-2.3061571	-4.1887640
C	-4.2291128	-2.5571461	-5.2306730
C	-2.8252428	-2.4526041	-4.9472480
C	-2.4237408	-2.1430081	-3.6654930
H	-1.3801198	-1.9953281	-3.4256560
C	-6.9835948	-2.6386561	-5.8048890
H	-8.0453808	-2.6651321	-6.0264840
C	-6.0481378	-2.9406441	-6.8313420
C	-4.6999648	-2.8959491	-6.5231340
H	-3.9723508	-3.0911101	-7.3004110
C	-2.4336398	6.4720479	-1.0033820
C	-3.6192058	6.5663909	-1.7130480
C	-4.5719828	7.5424499	-1.2652090
C	-4.2373498	8.4528059	-0.2069730
C	-2.9753488	8.3211799	0.4648810
C	-2.1044968	7.3300079	0.0587080
H	-1.1571968	7.1815369	0.5640600
C	-5.8736758	7.6283299	-1.8303650
H	-6.1619898	6.9177389	-2.5944200
C	-6.7650108	8.5955229	-1.4360220
H	-7.7416748	8.6432609	-1.9059800
C	-6.4146218	9.5593969	-0.4521140
C	-5.1688218	9.4634199	0.1429240
H	-4.8982368	10.1671899	0.9191920
C	-3.9014808	5.6487989	-2.8477210
C	-3.8091708	4.2853639	-2.6539120
C	-4.0538098	3.3339839	-3.6521780
H	-3.9051818	2.2903679	-3.4128650
C	-4.3681868	3.7353749	-4.9328090
C	-4.4738348	5.1391969	-5.2159830
C	-4.2192708	6.0971599	-4.1750170
C	-4.8171288	5.6099469	-6.5073050
H	-5.0149338	4.8822649	-7.2838510
C	-4.8628958	6.9580819	-6.8154510
C	-4.5574298	7.8936429	-5.7901060
H	-4.5846718	8.9554039	-6.0117300
C	-4.2474818	7.4760269	-4.5176710
H	-4.0227378	8.2113819	-3.7557300
C	-6.5658698	-2.3330911	-4.5314160
H	-7.3011888	-2.1109651	-3.7686760
C	-6.4190988	-0.2053011	0.0520620
H	-6.2702878	0.7402329	0.5606110
C	-1.7674888	-2.5994511	-5.9795140
C	-0.6875978	-3.4654991	-5.7476940
C	-1.7671068	-1.8157151	-7.1454960
C	0.3575192	-3.5615281	-6.6634960
H	-0.6728298	-4.0649131	-4.8424720
C	-0.7175028	-1.9106691	-8.0603110
H	-2.5744358	-1.1112231	-7.3167780
C	0.3451542	-2.7873911	-7.8255080
H	1.1823052	-4.2344091	-6.4659030
H	-0.7266098	-1.2908831	-8.9520340
H	1.1625562	-2.8661091	-8.5359950
C	-6.5007358	-3.2458121	-8.2079540
C	-7.6048628	-2.5774941	-8.7631580
C	-5.8244818	-4.1908411	-8.9976000
C	-8.0177708	-2.8440931	-10.0679120
H	-8.1259558	-1.8262541	-8.1780260
C	-6.2372448	-4.4565951	-10.3019650
H	-4.9841728	-4.7339591	-8.5765740
C	-7.3358848	-3.7845041	-10.8428800
H	-8.8679978	-2.3104941	-10.4819830
H	-5.7056358	-5.1956481	-10.8938110
H	-7.6578578	-3.9924681	-11.8585940
C	-8.2874198	-0.6831091	1.5879520
C	-8.4105578	-1.4883551	2.7321410
C	-8.9713718	0.5415369	1.5487580
C	-9.2054818	-1.0820851	3.8029710
H	-7.8665438	-2.4256971	2.7865970
C	-9.7675998	0.9465789	2.6198960
H	-8.8838408	1.1689629	0.6670540
C	-9.8887858	0.1349639	3.7493610
H	-9.2848128	-1.7133561	4.6827320
H	-10.2950488	1.8943119	2.5702700
H	-10.5086548	0.4493549	4.5833490
C	-9.7206608	-5.4629391	-0.0912360
C	-9.4779408	-6.8462941	-0.0551800
C	-11.0037278	-5.0016211	0.2487910
C	-10.4838628	-7.7385241	0.3131520
H	-8.4882158	-7.2235281	-0.2925840
C	-12.0088288	-5.8935511	0.6184140
H	-11.2190848	-3.9388711	0.1997320
C	-11.7536098	-7.2663671	0.6520160
H	-10.2729808	-8.8033151	0.3426070
H	-12.9953888	-5.5165911	0.8708940
H	-12.5371108	-7.9613771	0.9378340
C	-4.5189528	2.6774879	-5.9643890
C	-5.3843928	1.5978269	-5.7292280
C	-3.7394528	2.6767129	-7.1332040
C	-5.4839098	0.5525269	-6.6444470
H	-5.9805628	1.5833989	-4.8218640
C	-3.8378778	1.6269119	-8.0474310
H	-3.0355048	3.4839199	-7.3072630
C	-4.7139248	0.5644579	-7.8092200
H	-6.1562468	-0.2720721	-6.4442360
H	-3.2213108	1.6357069	-8.9413850

H	-4.7953608	-0.2530791	-8.5192510
C	-5.1727018	7.4105799	-8.1910590
C	-4.5060378	8.5144699	-8.7487090
C	-6.1205888	6.7344279	-8.9773610
C	-4.7770108	8.9272499	-10.0526030
H	-3.7526698	9.0354709	-8.1662360
C	-6.3907318	7.1470769	-10.2808620
H	-6.6624318	5.8942869	-8.5543620
C	-5.7202488	8.2454859	-10.8242350
H	-4.2446338	9.7772929	-10.4686190
H	-7.1319338	6.6155549	-10.8700940
H	-5.9316258	8.5673619	-11.8392750
C	-2.5778848	9.1979809	1.5963730
C	-3.3794028	9.3207549	2.7432160
C	-1.3535818	9.8823089	1.5532310
C	-2.9698378	10.1156609	3.8128020
H	-4.3164038	8.7764639	2.8006900
C	-0.9452438	10.6785199	2.6231290
H	-0.7290368	9.7950719	0.6694550
C	-1.7531698	10.7993279	3.7552770
H	-3.5982378	10.1947039	4.6946430
H	0.0021662	11.2062509	2.5704460
H	-1.4362178	11.4191819	4.5883050
C	-7.3636778	10.6298459	-0.0669410
C	-8.7468528	10.3867959	-0.0264090
C	-6.9015798	11.9130379	0.2715580
C	-9.6381338	11.3925199	0.3447510
H	-9.1246268	9.3969749	-0.2625520
C	-7.7925558	12.9179389	0.6440150
H	-5.8390478	12.1286589	0.2190580
C	-9.1651958	12.6623959	0.6820360
H	-10.7027748	11.1813819	0.3776370
H	-7.4150208	13.9045979	0.8952410
H	-9.8594718	13.4457439	0.9700500
C	3.5103912	0.1523779	-5.9861920
C	4.3772572	1.2308319	-5.7507580
C	2.7259322	0.1577169	-7.1516660
C	4.4733512	2.2793969	-6.6626120
H	4.9772362	1.2417389	-4.8458560
C	2.8209342	1.2107889	-8.0624880
H	2.0209342	-0.6485941	-7.3256470
C	3.6984392	2.2720089	-7.8241500
H	5.1468192	3.1030259	-6.4622280
H	2.2005962	1.2054819	-8.9538590
H	3.7771812	3.0920949	-8.5315370
C	4.1554352	-4.5721621	-8.2332270
C	3.4869922	-5.6741941	-8.7924380
C	5.1002262	-3.8928821	-9.0205480
C	3.7532652	-6.0821451	-10.0988140

H	2.7359242	-6.1975221	-8.2090870
C	5.3656532	-4.3006921	-10.3265420
H	5.6434242	-3.0541621	-8.5964730
C	4.6934652	-5.3973021	-10.8714300
H	3.2195942	-6.9308161	-10.5159720
H	6.1045342	-3.7668291	-10.9165720
H	4.9011862	-5.7154201	-11.8884080
C	1.5927942	-6.3960161	1.5562640
C	2.3977782	-6.5232601	2.7001900
C	0.3681342	-7.0797761	1.5143580
C	1.9912572	-7.3220001	3.7680770
H	3.3351212	-5.9794671	2.7567960
C	-0.0371658	-7.8798161	2.5825560
H	-0.2590988	-6.9890671	0.6328370
C	0.7741952	-8.0050671	3.7117590
H	2.6223322	-7.4044941	4.6476880
H	-0.9849098	-8.4070441	2.5308400
H	0.4596022	-8.6279031	4.5434560
C	6.3728792	-7.8234881	-0.1269330
C	7.7562282	-7.5809501	-0.0894910
C	5.9113922	-9.1077621	0.2082750
C	8.6482862	-8.5882281	0.2755410
H	8.1335872	-6.5903861	-0.3231650
C	6.8031482	-10.1142241	0.5745990
H	4.8486532	-9.3229041	0.1580470
C	8.1759602	-9.8591741	0.6096380
H	9.7130732	-8.3774901	0.3061590
H	6.4260612	-11.1016751	0.8233740
H	8.8708362	-10.6437251	0.8928910
C	0.7582092	5.4302749	-5.9702150
C	-0.3200148	6.2964069	-5.7310590
C	0.7522762	4.6502689	-7.1386760
C	-1.3689638	6.3960929	-6.6420800
H	-0.3304518	6.8929659	-4.8238940
C	-0.3011688	4.7488609	-8.0486790
H	1.5584062	3.9458259	-7.3157010
C	-1.3621908	5.6255719	-7.8065400
H	-2.1924278	7.0689019	-6.4387750
H	-0.2963308	4.1319049	-8.9423950
H	-2.1825748	5.7071009	-8.5132640
C	5.4822482	6.0825919	-8.2162600
C	6.5837162	5.4156329	-8.7783450
C	4.8031282	7.0305819	-8.9998670
C	6.9912732	5.6864419	-10.0839130
H	7.1068972	4.6621739	-8.1979470
C	5.2105572	7.3005609	-10.3050440
H	3.9648322	7.5726389	-8.5734950
C	6.3066092	6.6298039	-10.8528210
H	7.8394972	5.1538439	-10.5033450

H	4.6768282	8.0418569	-10.8921590
H	6.6244212	6.8410589	-11.8691660
C	7.3090682	3.4834919	1.5626560
C	7.4371172	4.2842859	2.7094300
C	7.9924372	2.2587849	1.5159840
C	8.2362322	3.8736389	3.7754570
H	6.8936402	5.2215839	2.7697040
C	8.7928552	1.8493599	2.5823220
H	7.9011152	1.6347839	0.6322350
C	8.9188902	2.6565759	3.7144060
H	8.3193372	4.5014959	4.6573120
H	9.3197642	0.9016479	2.5269140
H	9.5420162	2.3387699	4.5446640
C	8.7366842	8.2694279	-0.1039950
C	8.4945312	9.6527029	-0.0615700
C	10.0210282	7.8064419	0.2288780
C	9.5022522	10.5432169	0.3060010
H	7.5039392	10.0311519	-0.2933540
C	11.0279302	8.6966509	0.5977470
H	10.2358682	6.7438309	0.1748050
C	10.7732662	10.0693989	0.6377160
H	9.2918092	11.6079449	0.3404530
H	12.0154232	8.3184389	0.8446420
H	11.5581622	10.7630779	0.9229370

Rh$_2$(*S*-(4,4',6,6'-tetra-Ph)BNP)$_4$, (*S*-6a) [B3LYP]

Rh	14.7974000	7.5883000	0.1358000
Rh	14.7938000	7.5883000	-2.3965000
P	17.2911000	8.9372000	-1.1390000
P	16.1445000	5.0929000	-1.1377000
O	16.7223000	8.3853000	0.1690000
O	16.7286000	8.3530000	-2.4374000
O	17.1345000	10.5517000	-1.0539000
O	18.8904000	8.7221000	-1.2472000
O	15.5944000	5.6634000	0.1702000
O	15.5583000	5.6535000	-2.4361000
O	15.9296000	3.4934000	-1.2432000
O	17.7590000	5.2500000	-1.0554000
C	19.1910000	11.4659000	-1.9055000
C	19.9123000	10.8555000	-0.7530000
C	19.7479000	9.5115000	-0.4651000
C	21.2889000	9.5413000	1.4053000
C	21.4705000	10.9526000	1.1953000
C	20.7500000	11.6117000	0.1395000
C	22.3469000	11.7222000	2.0061000
H	22.9081000	11.2145000	2.7808000
C	22.4824000	13.0935000	1.8631000
C	21.6979000	13.7413000	0.8698000
H	21.7741000	14.8169000	0.7454000
C	20.8716000	13.0255000	0.0372000
H	20.2992000	13.5491000	-0.7186000
C	17.8199000	11.3147000	-2.0113000
C	19.8490000	12.1837000	-2.9637000
C	19.0781000	12.8074000	-4.0056000
C	17.6483000	12.6453000	-4.0268000
C	17.0497000	11.8946000	-3.0332000
H	15.9801000	11.7169000	-3.0323000
C	21.8899000	12.9905000	-4.0357000
H	22.9736000	13.0503000	-4.0473000
C	21.1392000	13.6847000	-5.0238000
C	19.7587000	13.5728000	-4.9898000
H	19.1708000	14.0649000	-5.7547000
C	16.7205000	2.6374000	-0.4610000
C	18.0640000	2.4729000	-0.7508000
C	18.8220000	1.6370000	0.1419000
C	18.1649000	0.9182000	1.2002000
C	16.7539000	1.0999000	1.4122000
C	16.0645000	1.9634000	0.5833000
H	15.0079000	2.1543000	0.7343000
C	20.2356000	1.5155000	0.0374000
H	20.7578000	2.0866000	-0.7204000
C	20.9530000	0.6910000	0.8703000
H	22.0285000	0.6149000	0.7443000
C	20.3071000	-0.0918000	1.8662000
C	18.9361000	0.0436000	2.0113000
H	18.4299000	-0.5162000	2.7880000
C	18.6723000	3.1925000	-1.9054000
C	18.5207000	4.5633000	-2.0131000
C	19.0985000	5.3321000	-3.0372000
H	18.9206000	6.4016000	-3.0377000
C	19.8475000	4.7322000	-4.0312000
C	20.0100000	3.3025000	-4.0081000
C	19.3884000	2.5330000	-2.9639000
C	20.7738000	2.6204000	-4.9926000
H	21.2644000	3.2073000	-5.7593000
C	20.8860000	1.2399000	-5.0247000
C	20.1937000	0.4906000	-4.0341000
H	20.2537000	-0.5931000	-4.0442000
C	19.4809000	1.1155000	-3.0392000
H	18.9788000	0.5155000	-2.2900000
C	21.2664000	12.2760000	-3.0411000
H	21.8674000	11.7724000	-2.2938000
C	20.4236000	8.8536000	0.5767000
H	20.2328000	7.7968000	0.7263000
P	12.3006000	6.2391000	-1.1332000
P	13.4465000	10.0835000	-1.1344000
O	12.8725000	6.7918000	0.1731000

O	12.8589000	6.8236000	-2.4332000
O	12.4591000	4.6248000	-1.0479000
O	10.7007000	6.4525000	-1.2364000
O	14.0007000	9.5131000	0.1719000
O	14.0287000	9.5231000	-2.4345000
O	13.6607000	11.6831000	-1.2404000
O	11.8322000	9.9262000	-1.0464000
C	10.3987000	3.7074000	-1.8866000
C	9.6832000	4.3197000	-0.7315000
C	9.8479000	5.6644000	-0.4478000
C	8.3182000	5.6375000	1.4319000
C	8.1370000	4.2254000	1.2264000
C	8.8516000	3.5646000	0.1677000
C	7.2668000	3.4568000	2.0447000
H	6.7100000	3.9657000	2.8218000
C	7.1322000	2.0850000	1.9060000
C	7.9109000	1.4358000	0.9090000
H	7.8351000	0.3597000	0.7878000
C	8.7309000	2.1505000	0.0694000
H	9.2989000	1.6258000	-0.6888000
C	11.7691000	3.8597000	-2.0003000
C	9.7356000	2.9867000	-2.9396000
C	10.5014000	2.3617000	-3.9846000
C	11.9308000	2.5253000	-4.0141000
C	12.5342000	3.2785000	-3.0253000
H	13.6036000	3.4572000	-3.0307000
C	7.6897000	2.1752000	-3.9982000
H	6.6059000	2.1141000	-4.0035000
C	8.4355000	1.4798000	-4.9893000
C	9.8161000	1.5935000	-4.9634000
H	10.4002000	1.1005000	-5.7306000
C	12.8745000	12.5378000	-0.4519000
C	11.5293000	12.7029000	-0.7337000
C	10.7764000	13.5366000	0.1654000
C	11.4395000	14.2528000	1.2216000
C	12.8518000	14.0710000	1.4250000
C	13.5365000	13.2094000	0.5900000
H	14.5940000	13.0184000	0.7344000
C	9.3622000	13.6581000	0.0693000
H	8.8358000	13.0889000	-0.6869000
C	8.6495000	14.4801000	0.9087000
H	7.5733000	14.5564000	0.7892000
C	9.3010000	15.2603000	1.9030000
C	10.6730000	15.1251000	2.0396000
H	11.1836000	15.6829000	2.8148000
C	10.9146000	11.9859000	-1.8866000
C	11.0659000	10.6153000	-1.9985000
C	10.4824000	9.8490000	-3.0212000
H	10.6602000	8.7795000	-3.0253000
C	9.7278000	10.4513000	-4.0096000
C	9.5653000	11.8809000	-3.9821000
C	10.1926000	12.6479000	-2.9394000
C	8.7958000	12.5652000	-4.9606000
H	8.3012000	11.9802000	-5.7260000
C	8.6830000	13.9458000	-4.9885000
C	9.3806000	14.6927000	-3.9998000
H	9.3202000	15.7765000	-4.0067000
C	10.0993000	14.0655000	-3.0106000
H	10.6054000	14.6637000	-2.2627000
C	8.3179000	2.8925000	-3.0087000
H	7.7206000	3.3969000	-2.2590000
C	9.1777000	6.3242000	0.5965000
H	9.3683000	7.3815000	0.7424000
C	12.7921000	1.9391000	-5.0806000
C	13.8767000	1.1177000	-4.7305000
C	12.5874000	2.2373000	-6.4392000
C	14.7253000	0.5992000	-5.7096000
H	14.0471000	0.8802000	-3.6845000
C	13.4382000	1.7208000	-7.4171000
H	11.7709000	2.8925000	-6.7270000
C	14.5078000	0.8977000	-7.0564000
H	15.5559000	-0.0374000	-5.4190000
H	13.2691000	1.9689000	-8.4609000
H	15.1687000	0.4959000	-7.8187000
C	7.7491000	0.6662000	-6.0254000
C	6.5192000	1.0777000	-6.5692000
C	8.3119000	-0.5359000	-6.4905000
C	5.8777000	0.3170000	-7.5468000
H	6.0733000	2.0125000	-6.2426000
C	7.6709000	-1.2959000	-7.4685000
H	9.2468000	-0.8885000	-6.0651000
C	6.4506000	-0.8731000	-8.0013000
H	4.9324000	0.6588000	-7.9586000
H	8.1209000	-2.2247000	-7.8075000
H	5.9504000	-1.4660000	-8.7615000
C	7.6372000	6.3914000	2.5232000
C	7.8024000	6.0394000	3.8742000
C	6.8573000	7.5181000	2.2140000
C	7.1977000	6.7891000	4.8837000
H	8.4227000	5.1870000	4.1339000
C	6.2503000	8.2654000	3.2244000
H	6.7202000	7.8002000	1.1741000
C	6.4175000	7.9026000	4.5624000
H	7.3432000	6.5066000	5.9223000
H	5.6459000	9.1297000	2.9648000
H	5.9465000	8.4844000	5.3493000
C	6.2134000	1.3092000	2.7781000
C	6.5552000	0.0185000	3.2196000

C	4.9794000	1.8461000	3.1867000
C	5.6957000	-0.7074000	4.0443000
H	7.5116000	-0.4106000	2.9360000
C	4.1205000	1.1205000	4.0118000
H	4.6814000	2.8296000	2.8357000
C	4.4746000	-0.1599000	4.4446000
H	5.9843000	-1.6993000	4.3802000
H	3.1692000	1.5525000	4.3091000
H	3.8049000	-0.7256000	5.0857000
C	9.1389000	9.5889000	-5.0737000
C	8.3168000	8.5058000	-4.7204000
C	9.4352000	9.7909000	-6.4331000
C	7.7958000	7.6562000	-5.6973000
H	8.0809000	8.3374000	-3.6737000
C	8.9161000	8.9391000	-7.4087000
H	10.0907000	10.6063000	-6.7233000
C	8.0923000	7.8710000	-7.0449000
H	7.1587000	6.8268000	-5.4042000
H	9.1627000	9.1062000	-8.4532000
H	7.6886000	7.2093000	-7.8055000
C	7.8681000	14.6311000	-6.0243000
C	8.2795000	15.8599000	-6.5708000
C	6.6648000	14.0684000	-6.4864000
C	7.5176000	16.5004000	-7.5480000
H	9.2151000	16.3057000	-6.2465000
C	5.9035000	14.7084000	-7.4641000
H	6.3123000	13.1345000	-6.0589000
C	6.3263000	15.9276000	-7.9996000
H	7.8593000	17.4447000	-7.9620000
H	4.9739000	14.2585000	-7.8008000
H	5.7324000	16.4269000	-8.7596000
C	13.6081000	14.7536000	2.5136000
C	13.2584000	14.5914000	3.8655000
C	14.7348000	15.5321000	2.2008000
C	14.0103000	15.1976000	4.8724000
H	12.4060000	13.9723000	4.1280000
C	15.4844000	16.1407000	3.2086000
H	15.0153000	15.6668000	1.1602000
C	15.1238000	15.9764000	4.5477000
H	13.7296000	15.0545000	5.9119000
H	16.3487000	16.7439000	2.9463000
H	15.7073000	16.4486000	5.3325000
C	8.5274000	16.1814000	2.7746000
C	7.2371000	15.8414000	3.2190000
C	9.0658000	17.4158000	3.1799000
C	6.5133000	16.7030000	4.0432000
H	6.8069000	14.8848000	2.9380000
C	8.3422000	18.2768000	4.0046000
H	10.0490000	17.7124000	2.8267000
C	7.0624000	17.9245000	4.4402000
H	5.5217000	16.4158000	4.3813000
H	8.7754000	19.2284000	4.2993000
H	6.4982000	18.5958000	5.0809000
C	20.4304000	5.5973000	-5.0964000
C	21.2538000	6.6799000	-4.7450000
C	20.1271000	5.3982000	-6.4547000
C	21.7692000	7.5321000	-5.7226000
H	21.4952000	6.8461000	-3.6992000
C	20.6407000	6.2525000	-7.4311000
H	19.4704000	4.5832000	-6.7434000
C	21.4658000	7.3202000	-7.0691000
H	22.4075000	8.3612000	-5.4310000
H	20.3886000	6.0877000	-8.4747000
H	21.8652000	7.9839000	-7.8303000
C	21.6948000	0.5570000	-6.0669000
C	21.2797000	-0.6701000	-6.6144000
C	22.8958000	1.1204000	-6.5342000
C	22.0359000	-1.3083000	-7.5976000
H	20.3457000	-1.1163000	-6.2862000
C	23.6513000	0.4826000	-7.5178000
H	23.2511000	2.0530000	-6.1061000
C	23.2250000	-0.7349000	-8.0542000
H	21.6914000	-2.2514000	-8.0122000
H	24.5793000	0.9330000	-7.8584000
H	23.8143000	-1.2325000	-8.8188000
C	16.0039000	0.4150000	2.5038000
C	16.3619000	0.5736000	3.8539000
C	14.8749000	-0.3622000	2.1959000
C	15.6158000	-0.0347000	4.8639000
H	17.2161000	1.1917000	4.1128000
C	14.1312000	-0.9729000	3.2068000
H	14.5881000	-0.4943000	1.1567000
C	14.5000000	-0.8121000	4.5440000
H	15.9029000	0.1058000	5.9020000
H	13.2650000	-1.5751000	2.9483000
H	13.9210000	-1.2860000	5.3313000
C	21.0856000	-1.0158000	2.7304000
C	22.3787000	-0.6778000	3.1680000
C	20.5490000	-2.2512000	3.1351000
C	23.1071000	-1.5424000	3.9850000
H	22.8077000	0.2795000	2.8873000
C	21.2772000	-3.1152000	3.9527000
H	19.5636000	-2.5462000	2.7869000
C	22.5599000	-2.7649000	4.3815000
H	24.1009000	-1.2567000	4.3180000
H	20.8454000	-4.0675000	4.2470000
H	23.1276000	-3.4384000	5.0166000
C	16.7817000	13.2304000	-5.0896000

C	15.6999000	14.0536000	-4.7349000
C	16.9784000	12.9294000	-6.4487000
C	14.8463000	14.5710000	-5.7102000
H	15.5355000	14.2931000	-3.6884000
C	16.1227000	13.4450000	-7.4228000
H	17.7927000	12.2729000	-6.7399000
C	15.0559000	14.2699000	-7.0576000
H	14.0180000	15.2091000	-5.4161000
H	16.2857000	13.1948000	-8.4671000
H	14.3911000	14.6709000	-7.8170000
C	21.8207000	14.4953000	-6.0656000
C	23.0468000	14.0810000	-6.6158000
C	21.2568000	15.6973000	-6.5298000
C	23.6836000	14.8389000	-7.5986000
H	23.4934000	13.1463000	-6.2899000
C	21.8932000	16.4545000	-7.5130000
H	20.3249000	16.0520000	-6.0997000
C	23.1098000	16.0289000	-8.0521000
H	24.6260000	14.4949000	-8.0152000
H	21.4424000	17.3831000	-7.8513000
H	23.6063000	16.6195000	-8.8164000
C	21.9758000	8.7892000	2.4942000
C	21.8200000	9.1449000	3.8452000
C	22.7520000	7.6605000	2.1828000
C	22.4301000	8.3969000	4.8527000
H	21.2026000	9.9989000	4.1068000
C	23.3644000	6.9148000	3.1911000
H	22.8819000	7.3755000	1.1428000
C	23.2065000	7.2813000	4.5293000
H	22.2918000	8.6822000	5.8915000
H	23.9658000	6.0489000	2.9298000
H	23.6818000	6.7009000	5.3145000
C	23.4084000	13.8701000	2.7267000
C	23.0717000	15.1626000	3.1671000
C	24.6444000	13.3325000	3.1282000
C	23.9381000	15.8894000	3.9837000
H	22.1140000	15.5924000	2.8890000
C	25.5102000	14.0591000	3.9452000
H	24.9384000	12.3476000	2.7777000
C	25.1611000	15.3411000	4.3769000
H	23.6533000	16.8827000	4.3188000
H	26.4629000	13.6264000	4.2370000
H	25.8362000	15.9076000	5.0116000

Rh$_2$(*S*-(6,6'-dichloro-4,4'-(diPh))BNP)$_4$ [B3LYP-D3(BJ)]

Rh	14.7968000	7.5885000	1.4566000
Rh	14.7962000	7.5875000	-1.0692000
P	17.3481000	8.7558000	0.1550000
P	15.9643000	5.0363000	0.1569000
O	16.7366000	8.3557000	1.4949000
O	16.7947000	8.0664000	-1.0930000
O	17.2488000	10.3718000	0.0324000
O	18.9365000	8.4658000	0.0957000
O	15.5640000	5.6487000	1.4963000
O	15.2753000	5.5891000	-1.0917000
O	15.6738000	3.4479000	0.0985000
O	17.5803000	5.1350000	0.0344000
C	19.1004000	10.9123000	-1.3971000
C	20.0131000	10.6089000	-0.2644000
C	19.8998000	9.4204000	0.4376000
C	21.7596000	9.9205000	1.9079000
C	21.9066000	11.1856000	1.2469000
C	21.0037000	11.5438000	0.1899000
C	22.9357000	12.0965000	1.6082000
H	23.6588000	11.8157000	2.3618000
C	23.0116000	13.3268000	1.0041000
C	22.0790000	13.7309000	0.0260000
H	22.1391000	14.7226000	-0.4067000
C	21.1026000	12.8478000	-0.3686000
H	20.3871000	13.1551000	-1.1202000
C	17.7360000	10.8401000	-1.2030000
C	19.5532000	11.2362000	-2.7216000
C	18.5972000	11.5141000	-3.7588000
C	17.1925000	11.4215000	-3.4790000
C	16.7889000	11.1028000	-2.2002000
H	15.7436000	10.9649000	-1.9625000
C	21.3689000	11.5854000	-4.3186000
H	22.4254000	11.5900000	-4.5591000
C	20.4167000	11.9221000	-5.3037000
C	19.0686000	11.8846000	-5.0459000
H	18.3581000	12.1362000	-5.8218000
C	16.6281000	2.4844000	0.4406000
C	17.8166000	2.3705000	-0.2611000
C	18.7511000	1.3797000	0.1936000
C	18.3925000	0.4773000	1.2508000
C	17.1275000	0.6249000	1.9117000
C	16.2771000	1.6309000	1.4989000
H	15.3288000	1.7946000	1.9976000
C	20.0552000	1.2803000	-0.3646000
H	20.3630000	1.9954000	-1.1164000
C	20.9380000	0.3038000	0.0304000
H	21.9298000	0.2432000	-0.4021000
C	20.5334000	-0.6284000	1.0087000
C	19.3031000	-0.5520000	1.6127000
H	19.0220000	-1.2747000	2.3665000
C	18.1205000	3.2826000	-1.3942000
C	18.0487000	4.6471000	-1.2007000

C	18.3118000	5.5937000	-2.1982000
H	18.1741000	6.6392000	-1.9610000
C	18.6307000	5.1895000	-3.4767000
C	18.7231000	3.7847000	-3.7559000
C	18.4446000	2.8292000	-2.7185000
C	19.0939000	3.3127000	-5.0427000
H	19.3460000	4.0228000	-5.8188000
C	19.1312000	1.9645000	-5.2999000
C	18.7938000	1.0127000	-4.3146000
H	18.7981000	-0.0438000	-4.5547000
C	18.4575000	1.4481000	-3.0535000
H	18.1936000	0.7192000	-2.2982000
C	20.9342000	11.2495000	-3.0572000
H	21.6635000	10.9861000	-2.3021000
C	20.7537000	9.0698000	1.4956000
H	20.5905000	8.1215000	1.9944000
P	12.2448000	6.4202000	0.1572000
P	13.6286000	10.1397000	0.1553000
O	12.8569000	6.8213000	1.4965000
O	12.7975000	7.1088000	-1.0916000
O	12.3441000	4.8041000	0.0356000
O	10.6563000	6.7102000	0.0984000
O	14.0295000	9.5283000	1.4950000
O	14.3170000	9.5860000	-1.0932000
O	13.9191000	11.7281000	0.0955000
O	12.0125000	10.0409000	0.0338000
C	10.4918000	4.2625000	-1.3926000
C	9.5796000	4.5668000	-0.2596000
C	9.6932000	5.7557000	0.4414000
C	7.8342000	5.2566000	1.9130000
C	7.6869000	3.9911000	1.2530000
C	8.5892000	3.6322000	0.1958000
C	6.6580000	3.0804000	1.6155000
H	5.9353000	3.3617000	2.3692000
C	6.5819000	1.8497000	1.0122000
C	7.5140000	1.4449000	0.0339000
H	7.4536000	0.4529000	-0.3980000
C	8.4901000	2.3278000	-0.3618000
H	9.2053000	2.0199000	-1.1136000
C	11.8563000	4.3349000	-1.1992000
C	10.0383000	3.9377000	-2.7167000
C	10.9938000	3.6591000	-3.7541000
C	12.3987000	3.7519000	-3.4750000
C	12.8029000	4.0714000	-2.1966000
H	13.8483000	4.2093000	-1.9595000
C	8.2219000	3.5873000	-4.3125000
H	7.1653000	3.5824000	-4.5525000
C	9.1736000	3.2500000	-5.2979000
C	10.5219000	3.2878000	-5.0407000
H	11.2320000	3.0357000	-5.8169000
C	12.9649000	12.6918000	0.4375000
C	11.7760000	12.8051000	-0.2636000
C	10.8417000	13.7961000	0.1910000
C	11.2008000	14.6993000	1.2474000
C	12.4663000	14.5523000	1.9076000
C	13.3165000	13.5460000	1.4950000
H	14.2651000	13.3827000	1.9932000
C	9.5373000	13.8951000	-0.3666000
H	9.2292000	13.1794000	-1.1177000
C	8.6546000	14.8718000	0.0283000
H	7.6626000	14.9320000	-0.4036000
C	9.0597000	15.8047000	1.0057000
C	10.2904000	15.7288000	1.6090000
H	10.5719000	16.4520000	2.3622000
C	11.4715000	11.8922000	-1.3959000
C	11.5435000	10.5278000	-1.2014000
C	11.2799000	9.5805000	-2.1981000
H	11.4178000	8.5352000	-1.9602000
C	10.9604000	9.9838000	-3.4768000
C	10.8679000	11.3884000	-3.7570000
C	11.1468000	12.3447000	-2.7203000
C	10.4965000	11.8595000	-5.0439000
H	10.2440000	11.1488000	-5.8194000
C	10.4590000	13.2075000	-5.3021000
C	10.7968000	14.1600000	-4.3175000
H	10.7922000	15.2164000	-4.5584000
C	11.1336000	13.7255000	-3.0563000
H	11.3978000	14.4550000	-2.3016000
C	8.6572000	3.9241000	-3.0515000
H	7.9282000	4.1879000	-2.2962000
C	8.8398000	6.1071000	1.4996000
H	9.0032000	7.0557000	1.9976000
C	13.4540000	3.5908000	-4.5067000
C	14.5401000	2.7356000	-4.2626000
C	13.4473000	4.3590000	-5.6828000
C	15.5856000	2.6358000	-5.1777000
H	14.5590000	2.1473000	-3.3503000
C	14.4974000	4.2608000	-6.5961000
H	12.6361000	5.0568000	-5.8620000
C	15.5663000	3.3953000	-6.3496000
H	16.4128000	1.9669000	-4.9751000
H	14.4838000	4.8685000	-7.4958000
H	16.3819000	3.3139000	-7.0617000
C	6.9626000	5.6758000	3.0402000
C	6.8284000	4.8851000	4.1931000
C	6.2970000	6.9099000	2.9857000
C	6.0396000	5.3156000	5.2591000
H	7.3602000	3.9414000	4.2599000

C	5.5068000	7.3384000	4.0518000
H	6.3933000	7.5255000	2.0967000
C	5.3742000	6.5417000	5.1907000
H	5.9514000	4.6962000	6.1463000
H	4.9927000	8.2927000	3.9911000
H	4.7590000	6.8748000	6.0208000
C	10.7992000	8.9277000	-4.5077000
C	9.9441000	7.8417000	-4.2627000
C	11.5672000	8.9336000	-5.6839000
C	9.8441000	6.7956000	-5.1771000
H	9.3559000	7.8234000	-3.3503000
C	11.4689000	7.8828000	-6.5965000
H	12.2651000	9.7446000	-5.8637000
C	10.6035000	6.8141000	-6.3491000
H	9.1753000	5.9685000	-4.9737000
H	12.0765000	7.8958000	-7.4963000
H	10.5220000	5.9980000	-7.0606000
C	12.8857000	15.4246000	3.0340000
C	12.0951000	15.5600000	4.1868000
C	14.1200000	16.0898000	2.9789000
C	12.5259000	16.3496000	5.2521000
H	11.1513000	15.0285000	4.2541000
C	14.5488000	16.8809000	4.0444000
H	14.7356000	15.9926000	2.0900000
C	13.7521000	17.0146000	5.1831000
H	11.9065000	16.4388000	6.1392000
H	15.5032000	17.3946000	3.9833000
H	14.0853000	17.6304000	6.0127000
C	18.7915000	6.2449000	-4.5085000
C	19.6467000	7.3310000	-4.2646000
C	18.0231000	6.2381000	-5.6844000
C	19.7463000	8.3764000	-5.1798000
H	20.2352000	7.3500000	-3.3524000
C	18.1211000	7.2881000	-6.5978000
H	17.3251000	5.4269000	-5.8633000
C	18.9866000	8.3570000	-6.3515000
H	20.4153000	9.2037000	-4.9773000
H	17.5131000	7.2744000	-7.4973000
H	19.0678000	9.1726000	-7.0637000
C	16.7087000	-0.2467000	3.0390000
C	17.5000000	-0.3813000	4.1914000
C	15.4743000	-0.9119000	2.9850000
C	17.0698000	-1.1701000	5.2575000
H	18.4438000	0.1502000	4.2578000
C	15.0461000	-1.7022000	4.0513000
H	14.8583000	-0.8152000	2.0964000
C	15.8435000	-1.8351000	5.1897000
H	17.6896000	-1.2587000	6.1443000
H	14.0917000	-2.2159000	3.9911000
H	15.5107000	-2.4504000	6.0198000
C	16.1367000	11.5819000	-4.5103000
C	15.0506000	12.4371000	-4.2662000
C	16.1429000	10.8129000	-5.6858000
C	14.0048000	12.5363000	-5.1809000
H	15.0321000	13.0259000	-3.3542000
C	15.0924000	10.9105000	-6.5987000
H	16.9540000	10.1149000	-5.8649000
C	14.0236000	11.7761000	-6.3523000
H	13.1776000	13.2053000	-4.9782000
H	15.1056000	10.3022000	-7.4981000
H	13.2076000	11.8570000	-7.0641000
C	22.6317000	9.5021000	3.0350000
C	22.7665000	10.2936000	4.1873000
C	23.2972000	8.2680000	2.9810000
C	23.5558000	9.8638000	5.2531000
H	22.2347000	11.2373000	4.2537000
C	24.0880000	7.8402000	4.0471000
H	23.2005000	7.6518000	2.0925000
C	24.2211000	8.6377000	5.1853000
H	23.6445000	10.4838000	6.1399000
H	24.6020000	6.8858000	3.9868000
H	24.8367000	8.3052000	6.0153000
Cl	10.0092000	13.7735000	-6.9062000
Cl	8.6065000	2.8003000	-6.9016000
Cl	21.6491000	-1.9105000	1.4658000
Cl	5.3003000	0.7338000	1.4701000
Cl	24.2934000	14.4429000	1.4606000
Cl	7.9442000	17.0870000	1.4626000
Cl	19.5804000	1.3973000	-6.9038000
Cl	20.9830000	12.3707000	-6.9080000

Rh_2(*S*-(6,6'-dichloro-4,4'-(diPh))BNP)$_4$ [B3LYP]

Rh	14.7958700	7.5878600	0.6047500
Rh	14.7966950	7.5872050	-1.9269910
P	17.2991780	8.9226220	-0.6693340
P	16.1314150	5.0846500	-0.6688200
O	16.7243280	8.3810720	0.6405660
O	16.7387390	8.3293410	-1.9647190
O	17.1472870	10.5377710	-0.5989480
O	18.8974740	8.7009480	-0.7748910
O	15.5890280	5.6593790	0.6407880
O	15.5389530	5.6452130	-1.9645240
O	15.9098070	3.4863730	-0.7747470
O	17.7465100	5.2365350	-0.5972670
C	19.1758930	11.4224440	-1.5428780
C	19.9294360	10.8531270	-0.3897130
C	19.7719030	9.5217790	-0.0459310
C	21.3642800	9.6188060	1.7788790

C	21.5397790	11.0205720	1.5116860
C	20.7926020	11.6433030	0.4505640
C	22.4423280	11.8222320	2.2662080
H	23.0480430	11.3477760	3.0288530
C	22.5554690	13.1768670	2.0487420
C	21.7591980	13.8016290	1.0632480
H	21.8217000	14.8757410	0.9158770
C	20.9079310	13.0521410	0.2827450
H	20.3055110	13.5410350	-0.4728760
C	17.8022750	11.2741230	-1.5993580
C	19.8025050	12.0985890	-2.6488150
C	18.9958430	12.6899130	-3.6847640
C	17.5655600	12.5395670	-3.6491960
C	16.9992120	11.8228390	-2.6120480
H	15.9306070	11.6452330	-2.5706160
C	21.8160400	12.8446020	-3.8130740
H	22.8991940	12.8805270	-3.8829450
C	21.0237410	13.4976010	-4.7838400
C	19.6506770	13.4170850	-4.7185710
H	19.0474510	13.9139560	-5.4687880
C	16.7299260	2.6118730	-0.0450760
C	18.0615870	2.4543150	-0.3876280
C	18.8509480	1.5911000	0.4533730
C	18.2271860	0.8438610	1.5138490
C	16.8251610	1.0193410	1.7796910
C	16.1093440	1.9083850	1.0010850
H	15.0592110	2.0966560	1.1946230
C	20.2599490	1.4757790	0.2869230
H	20.7495800	2.0782400	-0.4681870
C	21.0086770	0.6244740	1.0681140
H	22.0829330	0.5619850	0.9217890
C	20.3829570	-0.1718520	2.0529550
C	19.0281090	-0.0587290	2.2691020
H	18.5529100	-0.6644820	3.0312540
C	18.6320030	3.2078390	-1.5402660
C	18.4837670	4.5814610	-1.5969380
C	19.0335840	5.3844840	-2.6090630
H	18.8560280	6.4531030	-2.5677980
C	19.7513910	4.8181060	-3.6454450
C	19.9016860	3.3878150	-3.6808580
C	19.3092410	2.5811900	-2.6455190
C	20.6299060	2.7329410	-4.7139010
H	21.1275940	3.3361390	-5.4635990
C	20.7104210	1.3598730	-4.7790800
C	20.0563640	0.5676120	-3.8089950
H	20.0923030	-0.5155430	-3.8788250
C	19.3825230	1.1651350	-2.7672460
H	18.8909210	0.5495880	-2.0236440
C	21.2185560	12.1718200	-2.7706190

H	21.8341300	11.6810270	-2.0265040
C	20.4753280	8.9022150	1.0008770
H	20.2870610	7.8522660	1.1954100
P	12.2933930	6.2523780	-0.6701240
P	13.4611660	10.0904300	-0.6706360
O	12.8673530	6.7947140	0.6398400
O	12.8547290	6.8449120	-1.9654660
O	12.4452410	4.6372840	-0.5985520
O	10.6951880	6.4739940	-0.7770940
O	14.0026730	9.5163560	0.6396190
O	14.0545200	9.5292230	-1.9656610
O	13.6828290	11.6886570	-0.7772420
O	11.8460190	9.9385580	-0.6002200
C	10.4174220	3.7518430	-1.5434720
C	9.6628270	4.3220610	-0.3914380
C	9.8200020	5.6536710	-0.0484940
C	8.2253540	5.5581390	1.7744230
C	8.0503040	4.1561210	1.5082640
C	8.7987700	3.5325390	0.4485500
C	7.1469520	3.3550380	2.2624360
H	6.5403160	3.8301010	3.0239680
C	7.0341950	2.0002010	2.0460270
C	7.8316590	1.3746580	1.0619960
H	7.7694320	0.3004120	0.9154920
C	8.6837510	2.1235470	0.2818170
H	9.2870870	1.6340510	-0.4726820
C	11.7910830	3.9001370	-1.5989080
C	9.7917830	3.0747380	-2.6493870
C	10.5993540	2.4824520	-3.6840800
C	12.0296060	2.6328000	-3.6473760
C	12.5950280	3.3505040	-2.6104010
H	13.6635950	3.5281260	-2.5681800
C	7.7792760	2.3277110	-3.8147810
H	6.6961850	2.2917580	-3.8855950
C	8.5724270	1.6737930	-4.7842290
C	9.9454330	1.7543390	-4.7178020
H	10.5493220	1.2567630	-5.4670170
C	12.8617840	12.5637650	-0.0493580
C	11.5304840	12.7208950	-0.3935290
C	10.7401370	13.5848890	0.4457410
C	11.3626830	14.3333070	1.5060950
C	12.7644470	14.1582650	1.7735950
C	13.4811680	13.2684330	0.9967240
H	14.5311190	13.0804910	1.1915650
C	9.3313040	13.6998840	0.2776560
H	8.8425490	13.0965760	-0.4773470
C	8.5816450	14.5519230	1.0571520
H	7.5075400	14.6141390	0.9096130
C	9.2062250	15.3493490	2.0418270

C	10.5608530	15.2366030	2.2595410
H	11.0351690	15.8432110	3.0215630
C	10.9613690	11.9662670	-1.5460820
C	11.1097630	10.5926100	-1.6013160
C	10.5612310	9.7886090	-2.6133570
H	10.7389140	8.7200570	-2.5709580
C	9.8445860	10.3539780	-3.6510960
C	9.6941680	11.7842210	-3.6879680
C	10.2853370	12.5918480	-2.6526820
C	8.9670770	12.4380800	-4.7224480
H	8.4703110	11.8341490	-5.4721670
C	8.8865100	13.8110800	-4.7889780
C	9.5393780	14.6042890	-3.8188690
H	9.5034250	15.6873770	-3.8897360
C	10.2120670	14.0077840	-2.7757930
H	10.7027560	14.6240600	-2.0321930
C	8.3758410	3.0014340	-2.7724060
H	7.7596060	3.4929280	-2.0293030
C	9.1152860	6.2740790	0.9969480
H	9.3032480	7.3242090	1.1907970
C	12.9354310	2.0766270	-4.6910450
C	14.0291920	1.2768370	-4.3188090
C	12.7864110	2.3790040	-6.0521830
C	14.9238010	0.7957640	-5.2703220
H	14.1733090	1.0256530	-3.2718330
C	13.6895150	1.8949990	-7.0012900
H	11.9709450	3.0193220	-6.3750720
C	14.7788000	1.0903890	-6.6375010
H	15.7521120	0.1791930	-4.9340570
H	13.5338110	2.1669980	-8.0389170
C	7.5086770	6.2707460	2.8690250
C	7.5952080	5.8612020	4.2071990
C	6.7651330	7.4294800	2.5898240
C	6.9574510	6.5767030	5.2227810
H	8.1840830	4.9858860	4.4646880
C	6.1285720	8.1356170	3.6068180
H	6.6773870	7.7718710	1.5625410
C	6.2059060	7.7288210	4.9505750
H	7.0639450	6.2220120	6.2414360
H	5.5589850	9.0213510	3.3412980
C	9.2896310	9.4481240	-4.6953880
C	8.4895410	8.3542740	-4.3240620
C	9.5934880	9.5972130	-6.0561890
C	8.0096070	7.4596400	-5.2761280
H	8.2372220	8.2101030	-3.2773670
C	9.1106240	8.6940820	-7.0058510
H	10.2340780	10.4127520	-6.3783530
C	8.3057280	7.6047040	-6.6429780
H	7.3927510	6.6312580	-4.9405640

H	9.3837550	8.8498390	-8.0431730
C	13.4759420	14.8748220	2.8689970
C	13.0650070	14.7881430	4.2067350
C	14.6349680	15.6183930	2.5910850
C	13.7794430	15.4257930	5.2231310
H	12.1894220	14.1992400	4.4632460
C	15.3400450	16.2548430	3.6088850
H	14.9784290	15.7062530	1.5641690
C	14.9318420	16.1773710	4.9522080
H	13.4236830	15.3191930	6.2414020
H	16.2260570	16.8244590	3.3443530
C	20.3077360	5.7229680	-4.6898570
C	21.1075910	6.8169830	-4.3185110
C	20.0054630	5.5727760	-6.0508890
C	21.5888380	7.7107050	-5.2707710
H	21.3586950	6.9619970	-3.2716400
C	20.4896430	6.4749930	-7.0007480
H	19.3651000	4.7570820	-6.3731150
C	21.2943280	7.5645190	-6.6378490
H	22.2054610	8.5392510	-4.9351820
H	20.2177270	6.3183980	-8.0382630
C	16.1124500	0.3041660	2.8752060
C	16.5220470	0.3923830	4.2132550
C	14.9535510	-0.4395010	2.5970230
C	15.8064180	-0.2438380	5.2297060
H	17.3974990	0.9814100	4.4699380
C	14.2472850	-1.0745320	3.6148850
H	14.6111180	-0.5285470	1.5698660
C	14.6541080	-0.9954640	4.9585340
H	16.1611510	-0.1360520	6.2482100
H	13.3614170	-1.6442750	3.3501460
C	16.6606930	13.0946880	-4.6942550
C	15.5665890	13.8948570	-4.3238530
C	16.8109860	12.7909260	-6.0549440
C	14.6728720	14.3749670	-5.2766910
H	15.4215000	14.1471070	-3.2772670
C	15.9087710	13.2739670	-7.0053850
H	17.6267550	12.1502820	-6.3764200
C	14.8191510	14.0789510	-6.6434350
H	13.8442530	14.9918880	-4.9418290
H	16.0654440	13.0009120	-8.0425890
C	22.0793190	8.9072010	2.8752000
C	21.9909510	9.3181950	4.2128120
C	22.8229960	7.7479980	2.5983150
C	22.6270490	8.6036240	5.2300820
H	21.4019090	10.1939240	4.4685110
C	23.4578990	7.0427860	3.6169890
H	22.9121560	7.4044900	1.5715260
C	23.3786910	7.4510230	4.9602020

H	22.5191570	8.9594300	6.2482000
H	24.0276570	6.1566310	3.3532450
C	13.8047230	14.6270550	-7.6630410
C	13.8048330	16.1737140	-7.6071580
C	12.3910380	14.1025580	-7.3138670
C	14.1349020	14.1979020	-9.1052050
H	14.7921710	16.5747940	-7.8597560
H	13.5353330	16.5456240	-6.6141740
H	13.0807090	16.5787810	-8.3229140
H	12.3595670	13.0085910	-7.3534460
H	11.6567180	14.4898320	-8.0292440
H	12.0749410	14.4096430	-6.3126790
H	13.3896440	14.6134520	-9.7911020
H	14.1189110	13.1091690	-9.2204550
H	15.1170880	14.5619270	-9.4245340
C	7.7578480	6.5892690	-7.6617020
C	6.2111830	6.5892370	-7.6059750
C	8.2824840	5.1759720	-7.3111630
C	8.1871060	6.9181680	-9.1041270
H	5.8100050	7.5762900	-7.8595290
H	5.8392070	6.3206100	-6.6127800
H	5.8062790	5.8644000	-8.3211000
H	9.3764600	5.1446070	-7.3505730
H	7.8953960	4.4409450	-8.0259160
H	7.9753070	4.8607580	-6.3097260
H	7.7717080	6.1722320	-9.7893790
H	9.2758520	6.9021940	-9.2192540
H	7.8230010	7.9000180	-9.4243960
C	15.7941960	0.5412590	-7.6555910
C	15.7941330	-1.0053400	-7.5980590
C	17.2075250	1.0662190	-7.3056700
C	15.4653140	0.9688510	-9.0985140
H	14.8070520	-1.4067530	-7.8511290
H	16.0627550	-1.3761780	-6.6044360
H	16.5189350	-1.4111200	-8.3127240
H	17.2389710	2.1601440	-7.3464090
H	17.9425340	0.6782090	-8.0199400
H	17.5227030	0.7602410	-6.3038540
H	16.2112160	0.5526000	-9.7832860
H	15.4813630	2.0574600	-9.2149160
H	14.4834350	0.6044400	-9.4183440
C	21.8436730	8.5789440	-7.6567900
C	23.3902670	8.5787530	-7.5991020
C	21.3188410	9.9926470	-7.3081890
C	21.4161880	8.2488250	-9.0994620
H	23.7915890	7.5913980	-7.8512480
H	23.7610370	8.8482200	-6.6056830
H	23.7962050	9.3028660	-8.3143760
H	20.2249250	10.0241910	-7.3490970
H	21.7070330	10.7269750	-8.0230610
H	21.6247260	10.3086780	-6.3066120
H	21.8325860	8.9940730	-9.7848550
H	20.3275930	8.2648860	-9.2159860
H	21.7805270	7.2666230	-9.4183830
C	24.0956210	6.6415310	6.0557160
C	25.6147340	6.6128920	5.7611770
C	23.5492370	5.1935970	6.0653490
C	23.8885810	7.2438160	7.4583800
H	26.0320380	7.6254460	5.7566610
H	25.8332360	6.1552750	4.7917340
H	26.1387320	6.0332360	6.5293500
H	22.4753040	5.1817990	6.2791070
H	24.0558710	4.6027460	6.8366640
H	23.7057580	4.6925370	5.1054610
H	24.4170130	6.6359660	8.1998210
H	22.8310750	7.2644530	7.7415330
H	24.2815800	8.2635990	7.5267090
C	13.8434730	-1.7125330	6.0531110
C	13.8153060	-3.2316310	5.7584410
C	12.3954740	-1.1662950	6.0611780
C	14.4441840	-1.5055220	7.4564530
H	14.8279040	-3.6488390	5.7550270
H	13.3587920	-3.4501100	4.7884730
H	13.2348410	-3.7557340	6.5259330
H	12.3833290	-0.0923750	6.2749790
H	11.8038270	-1.6730310	6.8318160
H	11.8954830	-1.3228200	5.1007340
H	13.8355480	-2.0340350	8.1971920
H	14.4644300	-0.4480280	7.7396790
H	15.4639180	-1.8984540	7.5258970
C	15.7411340	16.8960640	6.0467130
C	15.7692710	18.4147790	5.7500710
C	17.1892460	16.3501680	6.0570690
C	15.1389470	16.6907710	7.4496760
H	14.7565800	18.8317450	5.7449980
H	16.2267960	18.6320900	4.7803180
H	16.3487710	18.9400290	6.5175070
H	17.2013970	15.2765340	6.2723040
H	17.7799450	16.8580550	6.8276750
H	17.6902390	16.5055300	5.0969580
H	15.7466530	17.2204120	8.1903730
H	15.1186410	15.6336500	7.7342870
H	14.1190450	17.0835520	7.5174910
C	5.4870930	8.5392640	6.0441500
C	3.9683910	8.5669490	5.7473880
C	6.0328640	9.9874340	6.0529380
C	5.6923590	7.9386460	7.4477880
H	3.5515070	7.5542200	5.7434180

H	3.7511030	9.0233760	4.7771120
H	3.4430500	9.1472630	6.5141470
H	7.1064860	9.9999180	6.2682080
H	5.5248880	10.5789340	6.8228710
H	5.8774980	10.4873620	5.0922720
H	5.1626500	8.5471420	8.1877870
H	6.7494690	7.9187180	7.7324640
H	5.2996310	6.9187980	7.5167180
Cl	8.2509533	16.4752775	2.9996062
Cl	7.9796451	14.5777987	-6.0879540
Cl	21.7916805	14.4045716	-6.0820207
Cl	23.6804541	14.1319215	3.0078454
Cl	21.6187081	0.5918797	-6.0763081
Cl	21.3370809	-1.2968805	3.0129333
Cl	7.8056288	0.7656411	-6.0822585
Cl	5.9082033	1.0458742	3.0046734

Rh_2(*S*-(6,6'-dichloro-4,4'-(*p*-tBu-Ph))BNP)$_4$, (*S*-18) [B3LYP-D3(BJ)]

Rh	-0.1456350	0.1270170	0.0420320
Rh	-0.1428350	0.1250580	-2.4901570
P	2.4545520	1.1569470	-1.2215950
P	0.8866660	-2.4727430	-1.2202850
O	1.8915890	0.5740940	0.0729160
O	1.8502740	0.6522790	-2.5329740
O	2.3649810	2.7677810	-1.0797140
O	4.0357760	0.8814830	-1.3976680
O	0.3018530	-1.9101270	0.0734890
O	0.3841150	-1.8681040	-2.5323160
O	0.6114510	-4.0539300	-1.3971250
O	2.4973090	-2.3832010	-1.0759760
C	4.2796320	3.6112900	-2.2590900
C	5.1547770	3.0245840	-1.2119890
C	5.0116790	1.7032270	-0.8214090
C	6.8603370	1.7828220	0.7421360
C	7.0336890	3.1707790	0.4191810
C	6.1489650	3.7992980	-0.5211730
C	8.0721750	3.9434950	1.0056840
H	8.7840440	3.4681110	1.6662970
C	8.1700440	5.2859170	0.7366400
C	7.2502250	5.9407580	-0.1085870
H	7.3242810	7.0096610	-0.2701750
C	6.2682820	5.2017130	-0.7238900
H	5.5632910	5.7029730	-1.3739620
C	2.9086100	3.4886060	-2.1540570
C	4.7765500	4.2970520	-3.4199770
C	3.8606250	4.9161330	-4.3416570
C	2.4452960	4.7205770	-4.1820270
C	2.0048260	3.9984830	-3.0904810
H	0.9573540	3.7690210	-2.9650770
C	6.6533580	5.0729660	-4.7783580
H	7.7160910	5.1086330	-4.9860840
C	5.7457100	5.7680690	-5.6036600
C	4.3894670	5.7001800	-5.4015470
H	3.7167810	6.2332240	-6.0591220
C	1.4324290	-5.0298680	-0.8198160
C	2.7543100	-5.1728630	-1.2086320
C	3.5281640	-6.1670790	-0.5169080
C	2.8984620	-7.0518600	0.4226020
C	1.5100890	-6.8785490	0.7437940
C	0.8115700	-5.8610490	0.1245240
H	-0.2277620	-5.6734980	0.3676880
C	4.9308370	-6.2863630	-0.7178600
H	5.4329020	-5.5813420	-1.3672770
C	5.6691180	-7.2683270	-0.1016790
H	6.7382240	-7.3423680	-0.2619290
C	5.0132210	-8.1881950	0.7426760
C	3.6704580	-8.0903640	1.0100260
H	3.1942600	-8.8022940	1.6699870
C	3.3423300	-4.2974920	-2.2548070
C	3.2195610	-2.9264920	-2.1495500
C	3.7305160	-2.0224600	-3.0851630
H	3.5009670	-0.9750060	-2.9596810
C	4.4536920	-2.4627130	-4.1760840
C	4.6494090	-3.8780160	-4.3358420
C	4.0293840	-4.7941570	-3.4150240
C	5.4346170	-4.4066250	-5.3949950
H	5.9683690	-3.7338000	-6.0518510
C	5.5028180	-5.7628270	-5.5972860
C	4.8068720	-6.6706740	-4.7729120
H	4.8428590	-7.7333690	-4.9807710
C	4.0959780	-6.1852000	-3.7002120
H	3.5725880	-6.8804710	-3.0565550
C	6.1676490	4.3632850	-3.7049610
H	6.8627710	3.8405670	-3.0605960
C	5.8428150	1.0835580	0.1237550
H	5.6552150	0.0445470	0.3682460
P	-2.7427030	-0.9052520	-1.2270390
P	-1.1755590	2.7245250	-1.2283350
O	-2.1830340	-0.3202700	0.0679050
O	-2.1358650	-0.4020530	-2.5378280
O	-2.6524340	-2.5158600	-1.0827400
O	-4.3237420	-0.6310210	-1.4067190
O	-0.5925450	2.1644840	0.0673370
O	-0.6703310	2.1179990	-2.5384830
O	-0.9009900	4.3055860	-1.4072650
O	-2.7863620	2.6343340	-1.0864150
C	-4.5648130	-3.3620200	-2.2638320

C	-5.4417480	-2.7743170	-1.2187870
C	-5.2998830	-1.4522830	-0.8301190
C	-7.1496650	-1.5307620	0.7321080
C	-7.3220420	-2.9192400	0.4108600
C	-6.4361950	-3.5485930	-0.5278880
C	-8.3606290	-3.6916950	0.9975270
H	-9.0732930	-3.2157820	1.6569040
C	-8.4576100	-5.0345220	0.7301870
C	-7.5367700	-5.6900340	-0.1134110
H	-7.6101900	-6.7591830	-0.2736620
C	-6.5546570	-4.9513330	-0.7288510
H	-5.8488930	-5.4531020	-1.3776890
C	-3.1940010	-3.2385140	-2.1569040
C	-5.0597160	-4.0494560	-3.4245550
C	-4.1421470	-4.6690940	-4.3442070
C	-2.7271400	-4.4726880	-4.1826970
C	-2.2885590	-3.7491850	-3.0913160
H	-1.2413560	-3.5191920	-2.9644800
C	-6.9341940	-4.8279400	-4.7846660
H	-7.9966140	-4.8644530	-4.9938330
C	-6.0249980	-5.5234370	-5.6079290
C	-4.6690770	-5.4545820	-5.4039910
H	-3.9951870	-5.9879930	-6.0600330
C	-1.7229650	5.2817460	-0.8317330
C	-3.0444730	5.4237740	-1.2221500
C	-3.8195550	6.4182270	-0.5321470
C	-3.1913250	7.3040050	0.4074170
C	-1.8032620	7.1315380	0.7303950
C	-1.1035440	6.1139340	0.1126580
H	-0.0644290	5.9269890	0.3572070
C	-5.2220430	6.5367610	-0.7348160
H	-5.7230590	5.8310290	-1.3842720
C	-5.9614530	7.5188820	-0.1202380
H	-7.0304040	7.5923470	-0.2817850
C	-5.3069360	8.4396640	0.7241950
C	-3.9644440	8.3426050	0.9931810
H	-3.4892970	9.0551960	1.6531890
C	-3.6309090	4.5470760	-2.2681080
C	-3.5075610	3.1762390	-2.1613640
C	-4.0171730	2.2710430	-3.0965770
H	-3.7873150	1.2238150	-2.9697910
C	-4.7395280	2.7098500	-4.1886220
C	-4.9357200	4.1248910	-4.3500360
C	-4.3170070	5.0422380	-3.4295260
C	-5.7200220	4.6520690	-5.4105690
H	-6.2527510	3.9783300	-6.0673210
C	-5.7885070	6.0080310	-5.6143560
C	-5.0938160	6.9170130	-4.7901790
H	-5.1299610	7.9794680	-4.9992320

C	-4.3839030	6.4329570	-3.7161960
H	-3.8615180	7.1291090	-3.0726710
C	-6.4503830	-4.1167840	-3.7113940
H	-7.1466920	-3.5937510	-3.0685660
C	-6.1321270	-0.8317290	0.1134750
H	-5.9452740	0.2077120	0.3567130
C	-1.6963830	-4.9220740	-5.1494050
C	-0.4662960	-5.4129040	-4.6739800
C	-1.8344490	-4.7468490	-6.5336070
C	0.5774650	-5.6984640	-5.5452170
H	-0.3265850	-5.5604220	-3.6078870
C	-0.7869440	-5.0507380	-7.4036780
H	-2.7476990	-4.3264670	-6.9385530
C	0.4444220	-5.5241230	-6.9340440
H	1.5154310	-6.0506380	-5.1287980
H	-0.9394070	-4.8865490	-8.4635380
C	-8.0042660	-0.8211530	1.7155230
C	-8.1811460	-1.2922280	3.0231540
C	-8.6175150	0.3919520	1.3647960
C	-8.9548680	-0.5824240	3.9420790
H	-7.6935970	-2.2106070	3.3343720
C	-9.3874730	1.0932960	2.2859330
H	-8.4975640	0.7764800	0.3564650
C	-9.5787930	0.6230010	3.5961200
H	-9.0586640	-0.9831590	4.9430730
H	-9.8508540	2.0236900	1.9737060
C	-5.1879140	1.6791620	-5.1558730
C	-5.6791210	0.4490530	-4.6808930
C	-5.0111570	1.8171390	-6.5398880
C	-5.9634390	-0.5948660	-5.5523330
H	-5.8278380	0.3094410	-3.6149610
C	-5.3138430	0.7694670	-7.4101940
H	-4.5904680	2.7304200	-6.9444550
C	-5.7874580	-0.4619690	-6.9409630
H	-6.3158710	-1.5328700	-5.1362200
H	-5.1484500	0.9218340	-8.4698800
C	-1.0948210	7.9861020	1.7146880
C	-1.5674020	8.1628360	3.0217930
C	0.1186590	8.5994330	1.3654090
C	-0.8586750	8.9364850	3.9416100
H	-2.4861180	7.6752190	3.3319070
C	0.8189120	9.3693380	2.2874210
H	0.5043550	8.4795830	0.3575120
C	0.3471130	9.5605070	3.5970900
H	-1.2605590	9.0401380	4.9421600
H	1.7496440	9.8327830	1.9762920
C	4.9034210	-1.4332890	-5.1440630
C	5.3946050	-0.2027840	-4.6700760
C	4.7280930	-1.5728770	-6.5281010

C	5.6803080	0.8399290	-5.5425090
H	5.5422300	-0.0618920	-3.6041590
C	5.0321510	-0.5264200	-7.3993810
H	4.3074700	-2.4864700	-6.9320030
C	5.5057920	0.7053840	-6.9311690
H	6.0327000	1.7782960	-5.1271840
H	4.8678600	-0.6800510	-8.4590580
C	0.8000250	-7.7323080	1.7276120
C	1.2709300	-7.9088440	3.0353480
C	-0.4132750	-8.3452570	1.3770280
C	0.5608060	-8.6820130	3.9544950
H	2.1894550	-7.4215020	3.3464570
C	-1.1149420	-9.1146510	2.2983870
H	-0.7976780	-8.2255560	0.3686200
C	-0.6447810	-9.3056940	3.6086610
H	0.9614410	-8.7855950	4.9555520
H	-2.0454620	-9.5778420	1.9862520
C	1.4159520	5.1693270	-5.1505500
C	0.1854020	5.6608410	-4.6770150
C	1.5555080	4.9925900	-6.5344110
C	-0.8574420	5.9453570	-5.5496640
H	0.0445740	5.8095970	-3.6112530
C	0.5089060	5.2954880	-7.4059390
H	2.4691580	4.5717190	-6.9379360
C	-0.7229900	5.7693050	-6.9381350
H	-1.7958650	6.2979650	-5.1346550
H	0.6624810	5.1300840	-8.4654510
C	7.7140680	1.0739860	1.7268670
C	7.8904120	1.5464350	3.0340710
C	8.3271520	-0.1396750	1.3777770
C	8.6635180	0.8374430	3.9541440
H	7.4029550	2.4652830	3.3440440
C	9.0965040	-0.8401970	2.3000440
H	8.2075890	-0.5252790	0.3698110
C	9.2873550	-0.3684900	3.6097920
H	8.7669180	1.2392570	4.9547490
H	9.5597980	-1.7710460	1.9890410
C	-1.9189170	6.0199910	-7.8643350
C	-2.5620900	7.3884690	-7.5557210
C	-2.9511690	4.8991320	-7.6110440
C	-1.5224020	5.9992610	-9.3495100
H	-1.8447040	8.2009370	-7.7099410
H	-2.9289070	7.4476340	-6.5286800
H	-3.4171130	7.5558540	-8.2187310
H	-2.5282020	3.9216700	-7.8654240
H	-3.8487760	5.0565750	-8.2191660
H	-3.2540570	4.8739320	-6.5627140
H	-2.4004760	6.2164130	-9.9653430
H	-1.1402010	5.0212180	-9.6568830
H	-0.7596400	6.7523230	-9.5726530
C	-6.0379950	-1.6589580	-7.8658370
C	-7.4061170	-2.3023370	-7.5560870
C	-4.9166630	-2.6905260	-7.6118570
C	-6.0179350	-1.2639610	-9.3514290
H	-8.2189180	-1.5854230	-7.7107470
H	-7.4647730	-2.6681160	-6.5286460
H	-7.5734070	-3.1581030	-8.2181580
H	-3.9394440	-2.2674500	-7.8669880
H	-5.0739510	-3.5888320	-8.2189880
H	-4.8910280	-2.9923170	-6.5632170
H	-6.2350970	-2.1427170	-9.9662840
H	-5.0401130	-0.8818190	-9.6595750
H	-6.7712810	-0.5016200	-9.5750520
C	1.6411600	-5.7761510	-7.8588270
C	2.2841320	-7.1441150	-7.5475390
C	2.6731250	-4.6548430	-7.6063890
C	1.2459160	-5.7577530	-9.3443800
H	1.5668910	-7.9568470	-7.7010360
H	2.6500910	-7.2016380	-6.5200970
H	3.1396960	-7.3125400	-8.2095780
H	2.2503040	-3.6778040	-7.8626190
H	3.5712870	-4.8131310	-8.2134750
H	2.9750840	-4.6280590	-6.5578270
H	2.1245180	-5.9758770	-9.9591120
H	0.8639800	-4.7802010	-9.6536410
H	0.4833400	-6.5111630	-9.5669870
C	5.7578920	1.9010930	-7.8572590
C	7.1261750	2.5439420	-7.5471190
C	4.6370030	2.9336720	-7.6054650
C	5.7388220	1.5043670	-9.3424040
H	7.9386180	1.8262780	-7.7001750
H	7.1841900	2.9109540	-6.5200810
H	7.2946380	3.3987680	-8.2101040
H	3.6597360	2.5109120	-7.8609270
H	4.7953800	3.8311560	-8.2135290
H	4.6106630	3.2367090	-6.5572050
H	5.9569300	2.3823060	-9.9580910
H	4.7610480	1.1223790	-9.6509010
H	6.4919510	0.7413810	-9.5645500
C	10.1447520	-1.1756440	4.5927360
C	11.5648520	-1.3472410	4.0100780
C	9.5039100	-2.5657810	4.7994620
C	10.2644880	-0.4879110	5.9622650
H	12.0390190	-0.3739110	3.8483920
H	11.5502200	-1.8782000	3.0541530
H	12.1894190	-1.9214730	4.7025370
H	8.4970370	-2.4710430	5.2185450
H	10.1083090	-3.1615290	5.4917930

H	9.4271220	-3.1191900	3.8592640
H	10.8880900	-1.0965630	6.6240660
H	9.2886140	-0.3684160	6.4431490
H	10.7307370	0.4990500	5.8801560
C	-1.4531940	-10.1630770	4.5905850
C	-1.6247120	-11.5829790	4.0074230
C	-2.8433100	-9.5217890	4.7960760
C	-0.7668640	-10.2833910	5.9607690
H	-0.6513910	-12.0574400	3.8465580
H	-2.1547580	-11.5679510	3.0509970
H	-2.1998200	-12.2075150	4.6991840
H	-2.7486340	-8.5150620	5.2155200
H	-3.4399830	-10.1261520	5.4876430
H	-3.3957290	-9.4445580	3.8553330
H	-1.3763900	-10.9069420	6.6218120
H	-0.6475050	-9.3076800	6.4420170
H	0.2200190	-10.7499600	5.8795200
C	1.1540500	10.4183740	4.5798040
C	1.3253380	11.8383530	3.9967630
C	2.5443280	9.7779000	4.7867300
C	0.4662750	10.5383250	5.9492930
H	0.3519040	12.3122610	3.8349420
H	1.8563100	11.8235820	3.0408470
H	1.8994260	12.4632330	4.6890600
H	2.4498080	8.7711180	5.2060780
H	3.1399250	10.3826180	5.4789130
H	3.0977750	9.7009850	3.8465650
H	1.0747800	11.1622410	6.6109320
H	0.3469990	9.5625520	6.4304390
H	-0.5207910	11.0043280	5.8670540
C	-10.4366520	1.4311390	4.5778550
C	-11.8564550	1.6023110	3.9943530
C	-9.7957770	2.8214150	4.7835630
C	-10.5571200	0.7447280	5.9479850
H	-12.3306230	0.6288730	3.8333330
H	-11.8413360	2.1324040	3.0379540
H	-12.4813080	2.1772260	4.6859890
H	-8.7891280	2.7269810	5.2032510
H	-10.4004660	3.4179020	5.4750040
H	-9.7184540	3.3738920	3.8428600
H	-11.1810110	1.3540550	6.6088900
H	-9.5814970	0.6256340	6.4294780
H	-11.0233860	-0.2422860	5.8665880
Cl	9.4633440	6.2285340	1.4691110
Cl	6.3779560	6.7083350	-6.9486300
Cl	6.4446120	-6.3947660	-6.9413240
Cl	5.9549310	-9.4815240	1.4762640
Cl	-6.6548540	-6.4655170	-6.9527550
Cl	-9.7510610	-5.9768010	1.4628230
Cl	-6.7290210	6.6382100	-6.9601310
Cl	-6.2500550	9.7331210	1.4557380

Rh_2(*S*-(6,6'-dichloro-4,4'-(*p*-tBu-Ph))BNP)$_4$, (*S*-18) [B3LYP]

Rh	14.7958890	7.5877680	0.5298280
Rh	14.7968270	7.5869180	-1.9967220
P	17.2490200	9.0277140	-0.7338120
P	16.2367300	5.1346610	-0.7329650
O	16.6986330	8.4381990	0.5653640
O	16.6662340	8.4881060	-2.0411790
O	17.0993110	10.6386970	-0.5915670
O	18.8459730	8.8144960	-0.8825790
O	15.6463010	5.6850030	0.5658120
O	15.6981190	5.7175610	-2.0406920
O	16.0235180	3.5377380	-0.8820570
O	17.8476070	5.2842510	-0.5894370
C	19.1155840	11.5729680	-1.5139530
C	19.8896410	10.9411470	-0.4085380
C	19.7383310	9.5930790	-0.1327190
C	21.3809130	9.5880460	1.6512000
C	21.5479450	11.0025880	1.4551090
C	20.7745170	11.6825530	0.4512070
C	22.4738590	11.7535290	2.2313880
H	23.1018120	11.2463820	2.9519080
C	22.5703660	13.1142770	2.0724700
C	21.7581330	13.8102570	1.1532550
H	21.8250510	14.8892470	1.0732850
C	20.8875990	13.0983970	0.3611030
H	20.2651600	13.6345460	-0.3442320
C	17.7404780	11.4289210	-1.5565230
C	19.7230110	12.3139450	-2.5873610
C	18.9002900	12.9674130	-3.5701260
C	17.4702910	12.8219720	-3.5195310
C	16.9223480	12.0428910	-2.5185660
H	15.8528930	11.8737550	-2.4657050
C	21.7233820	13.1272960	-3.7376900
H	22.8021700	13.1730140	-3.8341450
C	20.8990450	13.8299990	-4.6414050
C	19.5292520	13.7568830	-4.5726230
H	18.9213430	14.2994880	-5.2843970
C	16.8013960	2.6452250	-0.1316600
C	18.1496890	2.4938430	-0.4063420
C	18.8902710	1.6087870	0.4539310
C	18.2093690	0.8352350	1.4571070
C	16.7946630	1.0023310	1.6519760
C	16.1248630	1.9143400	0.8594120
H	15.0650690	2.0968200	0.9977570
C	20.3061850	1.4956240	0.3650500

H	20.8429910	2.1181430	-0.3397140
C	21.0172930	0.6249100	1.1576790
H	22.0963490	0.5579340	1.0786460
C	20.3204480	-0.1874330	2.0761420
C	18.9595670	-0.0908610	2.2338820
H	18.4517480	-0.7189070	2.9538470
C	18.7825330	3.2679590	-1.5111350
C	18.6386220	4.6430780	-1.5537310
C	19.2535500	5.4612460	-2.5151280
H	19.0844790	6.5307140	-2.4622970
C	20.0335010	4.9133370	-3.5154320
C	20.1788260	3.4833270	-3.5660560
C	19.5244170	2.6605710	-2.5839430
C	20.9690930	2.8543890	-4.5679400
H	21.5123610	3.4623180	-5.2791900
C	21.0421330	1.4845960	-4.6368000
C	20.3385890	0.6602370	-3.7337580
H	20.3843000	-0.4185460	-3.8302700
C	19.6048200	1.2472870	-2.7287340
H	19.0679020	0.6136930	-2.0336510
C	21.1363030	12.3943370	-2.7320920
H	21.7698770	11.8580620	-2.0364940
C	20.4691080	8.9174660	0.8590620
H	20.2866950	7.8577780	0.9983080
P	12.3436250	6.1470150	-0.7346650
P	13.3560270	10.0400540	-0.7355090
O	12.8930740	6.7373810	0.5645150
O	12.9274950	6.6856260	-2.0419660
O	12.4930180	4.5361300	-0.5910470
O	10.7468350	6.3603360	-0.8849990
O	13.9454830	9.4905610	0.5640670
O	13.8956330	9.4562970	-2.0424520
O	13.5693460	11.6368750	-0.8855170
O	11.7450370	9.8905330	-0.5931650
C	10.4775570	3.6013360	-1.5147040
C	9.7023840	4.2341210	-0.4106190
C	9.8535380	5.5823800	-0.1356360
C	8.2085880	5.5890420	1.6461110
C	8.0417230	4.1743440	1.4510050
C	8.8163700	3.4934970	0.4486390
C	7.1147860	3.4240990	2.2267340
H	6.4859570	3.9318680	2.9460510
C	7.0183870	2.0632270	2.0688120
C	7.8317030	1.3664380	1.1511700
H	7.7648020	0.2873890	1.0719900
C	8.7032720	2.0775940	0.3595240
H	9.3265370	1.5408340	-0.3446170
C	11.8527220	3.7451870	-1.5559860
C	9.8711650	2.8595380	-2.5881390
C	10.6948280	2.2051210	-3.5694870
C	12.1247950	2.3503890	-3.5175010
C	12.6717780	3.1302740	-2.5166380
H	13.7412010	3.2993000	-2.4627850
C	7.8718870	2.0455240	-3.7399070
H	6.7931950	1.9998850	-3.8374580
C	8.6970730	1.3419530	-4.6421700
C	10.0668040	1.4149110	-4.5719880
H	10.6753800	0.8716110	-5.2826580
C	12.7906850	12.5300120	-0.1366880
C	11.4426450	12.6810790	-0.4127880
C	10.7011950	13.5668860	0.4459680
C	11.3811060	14.3414050	1.4490630
C	12.7956500	14.1746310	1.6453610
C	13.4662500	13.2619060	0.8543040
H	14.5259390	13.0796720	0.9937800
C	9.2853580	13.6798740	0.3556570
H	8.7492560	13.0566890	-0.3490550
C	8.5734480	14.5512380	1.1468500
H	7.4944610	14.6180600	1.0667530
C	9.2693720	15.3644460	2.0652420
C	10.6301160	15.2681370	2.2243180
H	11.1372120	15.8968770	2.9441880
C	10.8108800	11.9059680	-1.5174970
C	10.9548780	10.5308180	-1.5587620
C	10.3409210	9.7117990	-2.5200570
H	10.5100190	8.6423900	-2.4661800
C	9.5618930	10.2588130	-3.5215730
C	9.4165020	11.6887690	-3.5735240
C	10.0699800	12.5123990	-2.5915250
C	8.6270800	12.3168160	-4.5766310
H	8.0844400	11.7082590	-5.2878230
C	8.5540380	13.6865470	-4.6467310
C	9.2567750	14.5117130	-3.7437990
H	9.2111240	15.5904100	-3.8412930
C	9.9896760	13.9255550	-2.7376220
H	10.5259730	14.5597660	-2.0426230
C	8.4580170	2.7792270	-2.7342970
H	7.8237870	3.3161640	-2.0398090
C	9.1215400	6.2588610	0.8546520
H	9.3038560	7.3186510	0.9932440
C	13.0440130	1.7184780	-4.5039980
C	14.1066890	0.9145910	-4.0582130
C	12.9343070	1.9470140	-5.8830960
C	15.0093890	0.3570840	-4.9589140
H	14.2181510	0.7183210	-2.9956500
C	13.8468490	1.3874780	-6.7804000
H	12.1431640	2.5869420	-6.2626970
C	14.9048880	0.5773070	-6.3439370

H	15.8105970	-0.2633090	-4.5687260
H	13.7225470	1.6033330	-7.8352310
C	7.4566930	6.3584720	2.6756980
C	7.5020400	6.0245610	4.0367670
C	6.7160770	7.4937330	2.3065020
C	6.8245610	6.7895120	4.9886530
H	8.0884150	5.1707980	4.3634910
C	6.0402570	8.2489080	3.2608250
H	6.6596830	7.7771190	1.2593080
C	6.0735250	7.9171030	4.6270290
H	6.8989890	6.4924710	6.0282600
H	5.4736150	9.1131560	2.9274250
C	8.9310320	9.3396610	-4.5088000
C	8.1269440	8.2767450	-4.0639490
C	9.1607720	9.4496900	-5.8876730
C	7.5703870	7.3741370	-4.9653330
H	7.9297540	8.1650220	-3.0015830
C	8.6021820	8.5372420	-6.7856610
H	9.8008910	10.2410260	-6.2665490
C	7.7918020	7.4789770	-6.3501410
H	6.9497880	6.5727420	-4.5758570
H	8.8189490	8.6618010	-7.8402750
C	13.5640530	14.9263730	2.6758270
C	13.2289840	14.8804710	4.0365920
C	14.6994160	15.6674360	2.3078530
C	13.9929090	15.5578600	4.9893620
H	12.3751050	14.2937430	4.3623770
C	15.4535540	16.3431830	3.2630530
H	14.9836920	15.7242590	1.2609240
C	15.1205750	16.3093700	4.6289580
H	13.6949910	15.4829980	6.0286870
H	16.3179080	16.9102090	2.9305820
C	20.6654490	5.8316220	-4.5027730
C	21.4694810	6.8945990	-4.0579730
C	20.4368290	5.7207110	-5.8817580
C	22.0270530	7.7964310	-4.9595040
H	21.6658160	7.0069820	-2.9955180
C	20.9964190	6.6323970	-6.7799010
H	19.7967970	4.9292960	-6.2606180
C	21.8067390	7.6907270	-6.3444220
H	22.6475650	8.5979060	-4.5700540
H	20.7804900	6.5071910	-7.8346100
C	16.0252390	0.2517680	2.6825390
C	16.3592540	0.2988330	4.0435260
C	14.8898600	-0.4891870	2.3143880
C	15.5942770	-0.3772980	4.9963450
H	17.2131150	0.8855210	4.3694310
C	14.1346560	-1.1636590	3.2696440
H	14.6063920	-0.5469120	1.2672890
C	14.4665510	-1.1286420	4.6357830
H	15.8914030	-0.3015590	6.0358330
H	13.2703050	-1.7306100	2.9370370
C	16.5520700	13.4528700	-4.5076030
C	15.4888650	14.2571170	-4.0637390
C	16.6632930	13.2230280	-5.8863610
C	14.5871270	14.8137380	-4.9659530
H	15.3762310	14.4543830	-3.0014830
C	15.7517020	13.7816710	-6.7851870
H	17.4548920	12.5827930	-6.2644920
C	14.6931570	14.5922160	-6.3506540
H	13.7854730	15.4344670	-4.5772170
H	15.8771550	13.5648170	-7.8396780
C	22.1313230	8.8196560	2.6826460
C	22.0838030	9.1549170	4.0433100
C	22.8726250	7.6841010	2.3157460
C	22.7598460	8.3909820	4.9970220
H	21.4968470	10.0089600	4.3682530
C	23.5470130	6.9299400	3.2718900
H	22.9307030	7.3996770	1.2689260
C	23.5115500	7.2630950	4.6377100
H	22.6837570	8.6890560	6.0362130
H	24.1142690	6.0654230	2.9402350
C	13.6696680	15.2233610	-7.3112200
C	13.7092260	16.7634580	-7.1641310
C	12.2523120	14.7111640	-6.9585130
C	13.9558710	14.8724120	-8.7834780
H	14.7002210	17.1554250	-7.4167470
H	13.4713940	17.0827270	-6.1450730
H	12.9793320	17.2263660	-7.8374760
H	12.1914020	13.6229650	-7.0656140
H	11.5106530	15.1597560	-7.6287570
H	11.9689610	14.9630880	-5.9320960
H	13.2050520	15.3443530	-9.4252120
H	13.9104420	13.7929580	-8.9612020
H	14.9388560	15.2317760	-9.1054150
C	7.1607490	6.4545390	-7.3097580
C	5.6206730	6.4937240	-7.1623590
C	7.6734730	5.0376250	-6.9560470
C	7.5112750	6.7397060	-8.7823190
H	5.2283330	7.4844020	-7.4156370
H	5.3016950	6.2565590	-6.1430550
H	5.1578460	5.7631740	-7.8350480
H	8.7616660	4.9769690	-7.0633620
H	7.2249510	4.2952910	-7.6255910
H	7.4218890	4.7550240	-5.9293400
H	7.0394010	5.9882560	-9.4233620
H	8.5907000	6.6944500	-8.9602590
H	7.1515580	7.7223400	-9.1049290

C	15.9294630	-0.0547190	-7.3027700
C	15.8899730	-1.5946680	-7.1541180
C	17.3463720	0.4580510	-6.9490990
C	15.6447430	0.2946490	-8.7756890
H	14.8992980	-1.9870410	-7.4073520
H	16.1268020	-1.9128510	-6.1344880
H	16.6206280	-2.0581780	-7.8262240
H	17.4072430	1.5461460	-7.0572770
H	18.0888070	0.0088620	-7.6180840
H	17.6286570	0.2072470	-5.9221130
H	16.3962720	-0.1778930	-9.4161460
H	15.6902420	1.3739200	-8.9544980
H	14.6621240	-0.0651520	-9.0982540
C	22.4388580	8.7143550	-7.3042030
C	23.9788240	8.6745270	-7.1558270
C	21.9265780	10.1316990	-6.9515670
C	22.0891040	8.4285700	-8.7768240
H	24.3708510	7.6835390	-7.4083770
H	24.2972710	8.9120420	-6.1364380
H	24.4424160	9.4045270	-7.8285870
H	20.8384770	10.1927990	-7.0595550
H	22.3758350	10.8734600	-7.6212520
H	22.1776930	10.4147540	-5.9248690
H	22.5617090	9.1794720	-9.4179710
H	21.0098080	8.4742140	-8.9554410
H	22.4485820	7.4456070	-9.0986960
C	24.2744840	6.4028890	5.6608150
C	25.7811360	6.4012640	5.3067050
C	23.7366840	4.9524920	5.6149460
C	24.1183010	6.9295630	7.0999510
H	26.1918740	7.4160320	5.3373170
H	25.9650930	5.9942360	4.3079080
H	26.3374490	5.7878090	6.0240850
H	22.6718970	4.9215060	5.8691330
H	24.2757100	4.3263820	6.3346490
H	23.8592070	4.5028390	4.6250350
H	24.6792600	6.2883090	7.7872190
H	23.0726420	6.9258290	7.4247660
H	24.5067580	7.9477960	7.2069050
C	13.6051630	-1.8915940	5.6578790
C	13.6034410	-3.3981630	5.3034120
C	12.1549640	-1.3533670	5.6107470
C	14.1305090	-1.7359200	7.0975550
H	14.6180560	-3.8092120	5.3349130
H	13.1973220	-3.5817780	4.3041840
H	12.9891290	-3.9544570	6.0200750
H	12.1240400	-0.2886360	5.8651740
H	11.5279970	-1.8923960	6.3297020
H	11.7062510	-1.4755080	4.6203630

H	13.4884870	-2.2969340	7.7840600
H	14.1266890	-0.6903510	7.4226610
H	15.1485580	-2.1246220	7.2053560
C	15.9807220	17.0738370	5.6509670
C	15.9814740	18.5801030	5.2951990
C	17.4313780	16.5367240	5.6052470
C	15.4545670	16.9189650	7.0904320
H	14.9665120	18.9903670	5.3257440
H	16.3880280	18.7631610	4.2960440
H	16.5949190	19.1375100	6.0117390
H	17.4629830	15.4722160	5.8605230
H	18.0574090	17.0768120	6.3242220
H	17.8807040	16.6584580	4.6150890
H	16.0957470	17.4809930	7.7768940
H	15.4589180	15.8736600	7.4163790
H	14.4361730	17.3070310	7.1972680
C	5.3090370	8.7784140	5.6480420
C	3.8029300	8.7796960	5.2915990
C	5.8469370	10.2287520	5.6014670
C	5.4629710	8.2532580	7.0879720
H	3.3921000	7.7649820	5.3227480
H	3.6205300	9.1855780	4.2920510
H	3.2455390	9.3939970	6.0074190
H	6.9113450	10.2599850	5.8572090
H	5.3068500	10.8556200	6.3197110
H	5.7258990	10.6773730	4.6109060
H	4.9009590	8.8952460	7.7736930
H	6.5081270	8.2573080	7.4144030
H	5.0743230	7.2351490	7.1953860
Cl	8.3570110	16.5195970	3.0338970
Cl	7.5692570	14.4450610	-5.8957790
Cl	21.6587100	14.8148340	-5.8897100
Cl	23.7245180	14.0265700	3.0423760
Cl	22.0279250	0.7249570	-5.8843650
Cl	21.2318090	-1.3418130	3.0466520
Cl	7.9385830	0.3562260	-5.8904850
Cl	5.8629780	1.1518040	3.0380420

Rh_2(*S*-megaBNP)$_4$, (*S*-1) [B3LYP-D3(BJ)]

Cl	-1.6205710	1.1747240	-0.0001440
Cl	-4.8156850	-6.6454160	3.1067680
P	2.2183630	-2.7453860	7.0986260
O	2.3486280	-3.2309060	5.5582180
O	0.6739210	-2.2682280	7.1995600
O	3.0686620	-1.5021230	7.3475130
O	2.4535620	-3.9802350	7.9654750
C	1.8127340	-2.3891070	4.5737520
C	2.7240740	-1.6260440	3.8310820
H	3.7743160	-1.6796860	4.0906260

C	2.2864220	-0.7966860	2.8165290
C	0.8736850	-0.7101820	2.5645990
C	-0.0488590	-1.4289010	3.4002300
C	0.4408430	-2.3356850	4.3985920
C	3.7826050	4.3085980	2.2547230
H	3.6079150	5.3806950	2.1144510
H	4.7253870	4.1834950	2.7969210
H	2.9778090	3.9172990	2.8836190
C	2.4968940	3.7887570	0.1480150
H	1.6554090	3.3859400	0.7189450
H	2.5134900	3.2880630	-0.8254330
H	2.3082040	4.8546320	-0.0186430
C	4.9523590	4.2403310	0.0469250
H	4.7343850	5.3034200	-0.0929870
H	5.0287050	3.7825690	-0.9444610
H	5.9278040	4.1628170	0.5374290
C	3.8393440	3.5934840	0.8870100
C	0.3548500	0.0663830	1.4933590
H	1.0336760	0.5460780	0.8024470
C	-1.0012270	0.2062260	1.3324750
C	-1.9194770	-0.3980140	2.2162440
H	-2.9835370	-0.2331350	2.0954390
C	-1.4409180	-1.2046140	3.2207200
H	-2.1422040	-1.6806920	3.8925650
C	3.2896360	-0.0181160	2.0495820
C	3.1291530	1.3530790	1.8336370
H	2.2605710	1.8508120	2.2489020
C	4.0760580	2.0936870	1.1125810
C	5.2027120	1.4226400	0.6330380
H	5.9412160	1.9738320	0.0658660
C	5.4203710	0.0528260	0.8634170
C	4.4487370	-0.6550060	1.5713930
H	4.5696610	-1.7143400	1.7575290
C	6.6890280	-0.6123850	0.3100470
C	6.8362530	-2.0646200	0.7874670
H	6.8689650	-2.1218450	1.8780330
H	7.7674480	-2.4898440	0.4014480
H	6.0179010	-2.7012950	0.4379520
C	7.9338910	0.1709430	0.7807260
H	7.9299210	1.2042440	0.4238250
H	8.8425170	-0.3069680	0.3990000
H	7.9932120	0.1910540	1.8729940
C	6.6265460	-0.6012210	-1.2325620
H	5.7559860	-1.1618970	-1.5880750
H	7.5265070	-1.0631560	-1.6527740
H	6.5546700	0.4180730	-1.6231970
C	-0.4443900	-3.1590560	5.2645790
C	-1.4346870	-4.0626620	4.7528220
C	-2.2889750	-4.7836600	5.6565370
C	-3.3540310	-5.5527200	5.1143960
H	-4.0599430	-6.0318540	5.7780080
C	-3.4903960	-5.6858250	3.7549480
C	-2.5869110	-5.0817970	2.8559930
H	-2.6879080	-5.2421730	1.7891950
C	-1.5888480	-4.2807070	3.3567580
H	-0.9026190	-3.8035990	2.6702760
C	-2.0605170	-4.7070950	7.0737900
C	-1.0530810	-3.8806070	7.5329760
H	-0.8117180	-3.8316460	8.5876150
C	-0.2959560	-3.1112790	6.6396540
C	-2.8436160	-5.4881460	8.0620360
C	-3.3172360	-4.8564840	9.2257220
H	-3.1207550	-3.8003020	9.3552650
C	-4.0323500	-5.5642260	10.1916120
C	-4.2812330	-6.9285560	9.9604750
H	-4.8559580	-7.4795940	10.6931860
C	-3.8092280	-7.5946660	8.8275220
C	-3.0758850	-6.8551890	7.8890410
H	-2.6629140	-7.3515220	7.0186680
C	-4.5715930	-4.9015860	11.4675250
C	-4.0514700	-3.4662050	11.6369960
H	-2.9592190	-3.4407850	11.6670720
H	-4.4223500	-3.0447640	12.5759700
H	-4.3843300	-2.8058870	10.8305010
C	-4.1290360	-5.7150950	12.7033080
H	-4.5194140	-6.7360160	12.6874420
H	-4.4968020	-5.2355290	13.6168480
H	-3.0381630	-5.7715910	12.7636020
C	-6.1135310	-4.8671310	11.3992230
H	-6.4495570	-4.2847580	10.5351860
H	-6.5241720	-4.4058820	12.3039690
H	-6.5326330	-5.8739110	11.3114740
C	-4.0549670	-9.0889410	8.5757370
C	-4.9051890	-9.7352040	9.6813650
H	-4.4144450	-9.6747660	10.6578940
H	-5.0600950	-10.7938760	9.4523140
H	-5.8900460	-9.2642320	9.7617340
C	-2.6974050	-9.8225570	8.5130980
H	-2.0622340	-9.4324480	7.7126750
H	-2.8527170	-10.8907830	8.3277600
H	-2.1545180	-9.7142530	9.4575470
C	-4.7952260	-9.2603850	7.2306520
H	-5.7615650	-8.7462310	7.2512310
H	-4.9768140	-10.3218320	7.0311060
H	-4.2177250	-8.8573980	6.3937070
Cl	13.3487890	0.9445070	3.1412870
Cl	10.0990050	-6.8671950	0.0602850
P	6.1757320	-2.7926690	7.0294950

O	7.7081090	-3.3007750	7.1680230
O	6.0847600	-2.3316990	5.4795220
O	5.9441080	-1.5367620	7.8658990
O	5.2899170	-4.0117330	7.2763200
C	8.7071660	-2.4840430	6.6208400
C	9.4634710	-1.7222510	7.5219410
H	9.2027380	-1.7571870	8.5726950
C	10.4928260	-0.9173130	7.0734170
C	10.7468400	-0.8552040	5.6597810
C	9.8988520	-1.5717290	4.7468290
C	8.8837340	-2.4529720	5.2484340
C	11.1522510	4.1984240	8.4848770
H	11.3163720	5.2641990	8.2932360
H	10.5992720	4.1005730	9.4245960
H	10.5225760	3.8065310	7.6810230
C	13.2599480	3.6141950	7.2283770
H	12.6889920	3.2081940	6.3883920
H	14.2229500	3.0945050	7.2630360
H	13.4501160	4.6730120	7.0228920
C	13.3468310	4.1054960	9.6769060
H	13.5088630	5.1621420	9.4434460
H	14.3287300	3.6306570	9.7695150
H	12.8462100	4.0526320	10.6488620
C	12.5043820	3.4568850	8.5667320
C	11.8327340	-0.1063080	5.1309290
H	12.5319370	0.3701310	5.8035370
C	11.9976770	0.0102940	3.7731350
C	11.1039190	-0.5911140	2.8627190
H	11.2291440	-0.4445660	1.7964870
C	10.0840000	-1.3715550	3.3518590
H	9.4042930	-1.8457260	2.6568940
C	11.2732900	-0.1391820	8.0664730
C	11.5152020	1.2253060	7.8871820
H	11.1113610	1.7183370	7.0105760
C	12.2476240	1.9657220	8.8255000
C	12.7116580	1.3019570	9.9629060
H	13.2866390	1.8530160	10.6954540
C	12.4561550	-0.0603770	10.1987970
C	11.7375020	-0.7685550	9.2353630
H	11.5315940	-1.8224490	9.3706370
C	12.9940190	-0.7175150	11.4782740
C	12.4954320	-2.1608430	11.6415920
H	11.4040830	-2.2024000	11.6720300
H	12.8727890	-2.5802550	12.5789590
H	12.8381880	-2.8122160	10.8319710
C	12.5282690	0.0882390	12.7106330
H	12.8984200	1.1167590	12.6946820
H	12.8997450	-0.3824980	13.6272150
H	11.4360770	0.1230220	12.7645990
C	14.5369190	-0.7293990	11.4236010
H	14.8887350	-1.3053140	10.5615210
H	14.9456430	-1.1868940	12.3310820
H	14.9431800	0.2830140	11.3419170
C	8.0033350	-3.2729810	4.3743350
C	8.4989870	-4.2006160	3.3981130
C	7.5825870	-4.9181300	2.5544550
C	8.1110630	-5.7139620	1.5022200
H	7.4392070	-6.1922610	0.8035770
C	9.4678750	-5.8746460	1.3694090
C	10.3772720	-5.2731480	2.2640830
H	11.4409600	-5.4550720	2.1668630
C	9.8908680	-4.4469850	3.2487180
H	10.5857550	-3.9721380	3.9280200
C	6.1668820	-4.8124720	2.7810160
C	5.7224250	-3.9627100	3.7756200
H	4.6687880	-3.8917470	4.0157880
C	6.6293190	-3.1981400	4.5213590
C	5.1649820	-5.5877610	2.0095980
C	4.0132620	-4.9428930	1.5248150
H	3.9023940	-3.8816550	1.7048230
C	3.0357920	-5.6441960	0.8190330
C	3.2431400	-7.0159570	0.5908760
H	2.5019390	-7.5626300	0.0228940
C	4.3631320	-7.6949280	1.0754090
C	5.3135960	-6.9611720	1.7989390
H	6.1742560	-7.4665590	2.2211810
C	1.7730240	-4.9666260	0.2674250
C	1.6248890	-3.5234560	0.7725920
H	1.5924730	-3.4868400	1.8644500
H	0.6937060	-3.0910510	0.3947540
H	2.4425940	-2.8793040	0.4355080
C	0.5219160	-5.7552220	0.7118850
H	0.5221470	-6.7795960	0.3303530
H	-0.3817930	-5.2636270	0.3358640
H	0.4558720	-5.8012450	1.8029130
C	1.8491460	-4.9489370	-1.2744150
H	2.7238420	-4.3836000	-1.6119340
H	0.9537580	-4.4778520	-1.6943140
H	1.9231280	-5.9613670	-1.6824120
C	4.5869390	-9.1976930	0.8561120
C	3.4760550	-9.8356390	0.0064570
H	2.4966400	-9.7488290	0.4873780
H	3.6857320	-10.9008520	-0.1299120
H	3.4133040	-9.3785100	-0.9861980
C	4.6221340	-9.9088800	2.2267020
H	5.4251910	-9.5244300	2.8620580
H	4.7860690	-10.9833680	2.0917570
H	3.6756040	-9.7713990	2.7592940

C	5.9347920	-9.4098260	0.1318790	H	6.8485720	-3.5858180	16.2227870
H	5.9339220	-8.9119650	-0.8431330	H	7.7597070	-3.2213120	17.6927100
H	6.1133220	-10.4782330	-0.0295740	H	6.0174650	-2.9604750	17.6552500
H	6.7747460	-9.0148480	0.7104570	C	7.8560490	-5.8881430	17.3651770
Cl	-1.7471770	-6.7205010	18.0265170	H	7.8270350	-6.9140120	17.7415970
Cl	-4.7743540	1.1766020	14.9517350	H	8.7736890	-5.4241570	17.7426360
P	2.2881770	-2.7536600	11.0549490	H	7.9197110	-5.9306400	16.2738650
O	2.3929830	-2.3011100	12.6065780	C	6.5546790	-5.0522790	19.3564820
O	0.7427350	-3.2208500	10.9166320	H	5.6966380	-4.4639510	19.6974320
O	3.1426630	-3.9937060	10.8023890	H	7.4635140	-4.6082200	19.7769210
O	2.5503400	-1.5008820	10.2227470	H	6.4527880	-6.0636920	19.7609360
C	1.8241410	-3.1539640	13.5627200	C	-0.4083460	-2.3433420	12.8376840
C	2.7089930	-3.9452360	14.3071930	C	-1.3976010	-1.4341310	13.3412130
H	3.7642000	-3.9039330	14.0668690	C	-2.2249100	-0.6920950	12.4297300
C	2.2407040	-4.7830740	15.3010020	C	-3.2889340	0.0866420	12.9600360
C	0.8226930	-4.8477440	15.5284180	H	-3.9740260	0.5844370	12.2883750
C	-0.0730690	-4.1036900	14.6856090	C	-3.4503520	0.2055530	14.3180340
C	0.4485510	-3.1897260	13.7100440	C	-2.5737100	-0.4222890	15.2271800
C	3.6229350	-9.9250410	15.8607740	H	-2.6943650	-0.2737990	16.2936690
H	3.4240520	-10.9930790	15.9997830	C	-1.5764840	-1.2306580	14.7365440
H	4.5699400	-9.8204400	15.3216080	H	-0.9102100	-1.7246600	15.4306890
H	2.8289680	-9.5155370	15.2297530	C	-1.9726020	-0.7583110	11.0159800
C	2.3431650	-9.3797910	17.9647550	C	-0.9669090	-1.5916570	10.5655870
H	1.5114670	-8.9602290	17.3914930	H	-0.7073150	-1.6314610	9.5147150
H	2.3674890	-8.8790660	18.9380380	C	-0.2330130	-2.3769990	11.4651570
H	2.1322710	-10.4414810	18.1314910	C	-2.7305660	0.0452960	10.0257650
C	4.7884410	-9.8830610	18.0713850	C	-3.2170190	-0.5672160	8.8570010
H	4.5480760	-10.9415640	18.2093390	H	-3.0429700	-1.6264550	8.7184130
H	4.8718530	-9.4282000	19.0635350	C	-3.9182340	0.1643520	7.8981500
H	5.7664590	-9.8251910	17.5833240	C	-4.1332300	1.5327420	8.1387740
C	3.6912610	-9.2123010	17.2291130	H	-4.6955050	2.1019170	7.4102910
C	0.2722370	-5.6273930	16.5814650	C	-3.6451130	2.1799950	9.2755750
H	0.9306320	-6.1246130	17.2797080	C	-2.9309440	1.4160690	10.2091060
C	-1.0885650	-5.7478560	16.7159610	H	-2.5092900	1.8945290	11.0854490
C	-1.9808300	-5.1205280	15.8218110	C	-4.4818450	-0.4733360	6.6200010
H	-3.0492940	-5.2711630	15.9202110	C	-4.0200090	-1.9276650	6.4465040
C	-1.4713840	-4.3094640	14.8362570	H	-2.9301350	-1.9959870	6.4102120
H	-2.1527300	-3.8147020	14.1574800	H	-4.4124180	-2.3326160	5.5089540
C	3.2197560	-5.5877880	16.0716220	H	-4.3744960	-2.5749380	7.2543420
C	3.0310280	-6.9563290	16.2823500	C	-4.0068330	0.3261430	5.3870900
H	2.1556940	-7.4362180	15.8604620	H	-4.3526820	1.3629550	5.4096670
C	3.9598040	-7.7174210	17.0059180	H	-4.3965790	-0.1322230	4.4717980
C	5.0992440	-7.0713000	17.4899060	H	-2.9146200	0.3357360	5.3244380
H	5.8245300	-7.6390450	18.0576900	C	-6.0240820	-0.4470620	6.6875600
C	5.3463800	-5.7061810	17.2621010	H	-6.4520570	-0.8886940	5.7810830
C	4.3898330	-4.9767010	16.5563590	H	-6.4035290	0.5749510	6.7783820
H	4.5319140	-3.9192160	16.3762000	H	-6.3828740	-1.0189080	7.5494600
C	6.6282270	-5.0663070	17.8144600	C	-3.8557090	3.6778210	9.5372380
C	6.8163010	-3.6260950	17.3145610	C	-4.6881340	4.3515990	8.4344100

H	-4.1978680	4.2840310	7.4581130
H	-4.8161610	5.4125890	8.6692400
H	-5.6845270	3.9062450	8.3503180
C	-2.4811890	4.3784070	9.6073070
H	-1.8564500	3.9660910	10.4047690
H	-2.6110880	5.4483950	9.8017990
H	-1.9399390	4.2654660	8.6624440
C	-4.5943170	3.8573210	10.8821270
H	-5.5720890	3.3654950	10.8563070
H	-4.7518560	4.9213190	11.0884330
H	-4.0277910	3.4354900	11.7172670
Cl	10.1862930	0.9501680	18.1232390
Cl	13.1536830	-6.9337630	14.9544280
P	6.2439440	-2.8006420	10.9858870
O	6.0977440	-3.2902810	12.5236180
O	7.8006940	-2.3638880	10.8905170
O	5.4257380	-1.5352400	10.7410550
O	5.9796960	-4.0250610	10.1125110
C	6.6568650	-2.4710220	13.5140040
C	5.7671220	-1.6878310	14.2619930
H	4.7159690	-1.7097650	14.0013810
C	6.2275720	-0.8789150	15.2829440
C	7.6418970	-0.8357500	15.5369890
C	8.5441740	-1.5744520	14.6966670
C	8.0296000	-2.4586060	13.6905740
C	4.8962360	4.2657720	15.8909410
H	5.1061170	5.3296750	16.0445720
H	3.9544000	4.1785640	15.3397790
H	5.6925830	3.8541150	15.2643940
C	6.1442190	3.6822840	18.0040270
H	6.3657480	4.7394420	18.1851750
H	6.9777760	3.2587370	17.4363960
H	6.1020030	3.1713630	18.9714190
C	3.7035320	4.2125010	18.0864780
H	3.6043590	3.7493390	19.0732950
H	2.7306310	4.1689420	17.5868090
H	3.9529060	5.2671650	18.2372910
C	4.8033570	3.5386940	17.2501390
C	8.1815720	-0.0842940	16.6157800
H	7.5159680	0.4094420	17.3096800
C	9.5408980	0.0129300	16.7806340
C	10.4424400	-0.6107760	15.8932400
H	11.5107200	-0.4793230	16.0177740
C	9.9419300	-1.3933860	14.8805050
H	10.6299510	-1.8844040	14.2057240
C	5.2468740	-0.0772390	16.0551910
C	5.4473110	1.2869500	16.2821310
H	6.3309380	1.7619530	15.8719610
C	4.5215800	2.0492510	17.0081290

C	3.3750520	1.4077010	17.4807930
H	2.6519490	1.9756840	18.0511950
C	3.1180150	0.0465480	17.2402470
C	4.0693060	-0.6838590	16.5277040
H	3.9175700	-1.7377310	16.3333820
C	1.8302960	-0.5851890	17.7885840
C	1.6428420	-2.0305690	17.3048030
H	1.6089450	-2.0826790	16.2139570
H	0.6999160	-2.4313620	17.6885130
H	2.4427960	-2.6913950	17.6521480
C	0.6087880	0.2348940	17.3192830
H	0.6416760	1.2665950	17.6793080
H	-0.3134790	-0.2182190	17.6985940
H	0.5513700	0.2599170	16.2270140
C	1.8904490	-0.5825820	19.3313460
H	2.7446660	-1.1684700	19.6858470
H	0.9773840	-1.0213510	19.7480840
H	1.9897400	0.4324980	19.7268860
C	8.8914120	-3.3011150	12.8192750
C	9.8536670	-4.2378240	13.3251990
C	10.6865060	-4.9777750	12.4168130
C	11.7273850	-5.7827080	12.9539670
H	12.4188240	-6.2784810	12.2873610
C	11.8584590	-5.9299820	14.3124520
C	10.9726510	-5.3057790	15.2151510
H	11.0675070	-5.4772690	16.2807860
C	9.9998150	-4.4710370	14.7196930
H	9.3276350	-3.9788990	15.4094130
C	10.4610880	-4.8847820	11.0000310
C	9.4795280	-4.0248930	10.5460920
H	9.2404990	-3.9621140	9.4916670
C	8.7455900	-3.2389880	11.4444650
C	11.2208150	-5.6834010	10.0075530
C	11.7164730	-5.0592940	8.8490460
H	11.5520270	-3.9969450	8.7252410
C	12.4136150	-5.7822850	7.8812270
C	12.6218860	-7.1544690	8.1054610
H	13.1836440	-7.7177150	7.3720280
C	12.1257570	-7.8132150	9.2323980
C	11.4110620	-7.0579920	10.1726930
H	10.9800980	-7.5468700	11.0385690
C	12.9778450	-5.1279700	6.6118560
C	12.4916620	-3.6805170	6.4438500
H	11.4003940	-3.6300970	6.4091840
H	12.8763620	-3.2652670	5.5076730
H	12.8358520	-3.0299030	7.2534370
C	12.5275610	-5.9264920	5.3691230
H	12.8962800	-6.9554570	5.3831180
H	12.9125850	-5.4507320	4.4607250

H	11.4362700	-5.9598680	5.3000030
C	14.5195260	-5.1293060	6.6931870
H	14.8614220	-4.5568960	7.5615520
H	14.9487900	-4.6758480	5.7931510
H	14.9136930	-6.1460010	6.7821100
C	12.3241460	-9.3161160	9.4741040
C	13.1657490	-9.9786430	8.3714220
H	12.6871970	-9.8954070	7.3905460
H	13.2859920	-11.0433970	8.5929360
H	14.1651080	-9.5369170	8.3048750
C	10.9440250	-10.0081450	9.5165820
H	10.3129660	-9.6043590	10.3134870
H	11.0642030	-11.0820720	9.6949840
H	10.4145600	-9.8760980	8.5675200
C	13.0445550	-9.5219870	10.8249750
H	14.0258020	-9.0365490	10.8192270
H	13.1922580	-10.5902400	11.0159080
H	12.4703920	-9.1100490	11.6598060
Rh	4.2166540	-4.0185260	9.0392000
Rh	4.2469390	-1.5145220	9.0440440

{Rh_2(*S*-megaBNP)$_4$}-[Ph-TCE] carbene complex

Cl	2.0411400	1.4109740	1.7886880
Cl	-3.2527160	-6.2850880	3.1766220
P	4.7196880	-5.0203730	7.1729040
O	4.5714680	-5.3145470	5.5843720
O	3.3184120	-4.2710300	7.5173070
O	5.8181730	-3.9692010	7.3669900
O	4.8410020	-6.3676980	7.8608110
C	4.2711560	-4.1717870	4.8296920
C	5.3523790	-3.5239640	4.2179480
H	6.3462130	-3.9261040	4.3526610
C	5.1546860	-2.3666170	3.5018260
C	3.8346730	-1.8230370	3.4141240
C	2.7399260	-2.4829640	4.0736580
C	2.9696980	-3.7084820	4.7873100
C	10.2857120	0.4303920	4.2848870
H	10.8475420	1.2156860	4.8021440
H	10.9098690	0.0529580	3.4726570
H	10.1198410	-0.3951980	4.9847740
C	8.1521930	1.5521070	4.9525860
H	7.9746260	0.7935010	5.7198640
H	7.1851360	1.9583290	4.6372150
H	8.7203930	2.3660420	5.4131160
C	9.2161210	2.1342820	2.7676840
H	9.7622520	2.9419190	3.2666640
H	8.2773160	2.5441500	2.3812490
H	9.8159850	1.8024530	1.9158680
C	8.9432270	0.9850360	3.7611910
C	3.5952540	-0.6212110	2.6983920
H	4.4207420	-0.1329930	2.1956120
C	2.3311090	-0.0880230	2.6621760
C	1.2506500	-0.6971730	3.3351220
H	0.2702260	-0.2369220	3.3141330
C	1.4618620	-1.8652750	4.0287150
H	0.6352120	-2.3206710	4.5567500
C	6.3533630	-1.7305730	2.8912670
C	6.9764110	-0.6455020	3.5229600
H	6.5217290	-0.2364440	4.4148640
C	8.1909000	-0.1479060	3.0493240
C	8.7524600	-0.7491960	1.9072220
H	9.6990520	-0.3736610	1.5398980
C	8.1433770	-1.8173310	1.2439460
C	6.9358260	-2.3054650	1.7644500
H	6.4442300	-3.1521760	1.2994060
C	8.7363050	-2.4816870	-0.0059260
C	8.9664950	-3.9817010	0.2757960
H	9.6299530	-4.1199150	1.1327770
H	9.4255590	-4.4695070	-0.5902710
H	8.0334830	-4.5067230	0.4959330
C	10.0750500	-1.8515210	-0.4202820
H	9.9661940	-0.7899500	-0.6634740
H	10.4624170	-2.3603530	-1.3079370
H	10.8246120	-1.9439570	0.3705710
C	7.7420030	-2.3306780	-1.1781240
H	6.7798310	-2.7992190	-0.9519180
H	8.1436290	-2.8065480	-2.0791810
H	7.5596580	-1.2743880	-1.4004720
C	1.9258840	-4.4351920	5.5589860
C	0.7171360	-4.9427960	4.9801940
C	-0.2551360	-5.6076060	5.8046080
C	-1.4917850	-5.9919170	5.2200870
H	-2.2660410	-6.4213570	5.8401290
C	-1.7110180	-5.8055970	3.8778250
C	-0.7295900	-5.2416050	3.0360600
H	-0.9149890	-5.1402870	1.9732910
C	0.4550090	-4.8173460	3.5888260
H	1.2079890	-4.3776000	2.9482560
C	0.0244740	-5.8386230	7.1948850
C	1.2251400	-5.3891120	7.7098160
H	1.4982310	-5.5862260	8.7396330
C	2.1407430	-4.7002850	6.9018100
C	-0.9103430	-6.5490410	8.1027000
C	-1.2022530	-5.9977520	9.3636470
H	-0.7586750	-5.0454890	9.6252140
C	-2.0456570	-6.6551600	10.2594470
C	-2.5980500	-7.8860250	9.8634920
H	-3.2593560	-8.3976080	10.5507050

C	-2.3221330	-8.4692840	8.6253160
C	-1.4629470	-7.7838530	7.7543230
H	-1.2000260	-8.2243640	6.7993780
C	-2.3903880	-6.0888480	11.6449600
C	-1.7095460	-4.7381010	11.9064140
H	-0.6216280	-4.8272100	11.8725990
H	-1.9827260	-4.3704190	12.9001100
H	-2.0112520	-3.9771380	11.1800190
C	-1.9170130	-7.0816710	12.7296950
H	-2.3900980	-8.0613480	12.6194090
H	-2.1664560	-6.6995100	13.7255820
H	-0.8325220	-7.2221180	12.6809690
C	-3.9181080	-5.8944890	11.7531640
H	-4.2736690	-5.1935830	10.9910290
H	-4.1775820	-5.4898380	12.7373370
H	-4.4590400	-6.8362990	11.6249300
C	-2.9207230	-9.8127990	8.1859340
C	-3.8278130	-10.4283430	9.2633630
H	-3.2780270	-10.6292170	10.1883090
H	-4.2309110	-11.3792500	8.9021280
H	-4.6746440	-9.7768440	9.5010470
C	-1.7799360	-10.8091360	7.8856330
H	-1.1232480	-10.4488760	7.0887450
H	-2.1942100	-11.7718160	7.5677600
H	-1.1672840	-10.9776580	8.7770690
C	-3.7618540	-9.5928540	6.9088590
H	-4.5783740	-8.8886420	7.0989960
H	-4.1984620	-10.5399280	6.5741110
H	-3.1577610	-9.1942230	6.0887090
Cl	14.1920060	-0.1523790	3.1481480
Cl	11.6645010	-8.1131150	-0.8569430
P	8.5938630	-5.5969320	7.2611020
O	10.1438770	-6.0253780	7.0592500
O	8.2104990	-4.9830680	5.8022040
O	8.5682430	-4.4187510	8.2439860
O	7.7870810	-6.8384620	7.5902700
C	10.9332990	-4.9877540	6.5276360
C	11.5403180	-4.1209640	7.4501610
H	11.3730630	-4.2893810	8.5063810
C	12.2099210	-3.0019760	7.0069690
C	12.3578850	-2.7951110	5.5954450
C	11.6860400	-3.6606510	4.6674450
C	10.9332840	-4.7828920	5.1605970
C	10.3990490	1.6980750	8.2335770
H	10.0214690	2.7193050	8.1165950
H	9.8111950	1.2047860	9.0102370
H	10.2200490	1.1756660	7.2918300
C	12.6914160	2.3797280	7.4537000
H	12.5861390	1.8022300	6.5299410
H	13.7575610	2.4341370	7.6966730
H	12.3303490	3.3963680	7.2630060
C	12.0558590	2.5963210	9.8706150
H	11.6194160	3.5844440	9.6960250
H	13.1068720	2.7444390	10.1360360
H	11.5449060	2.1491770	10.7291100
C	11.8957820	1.7313040	8.6091750
C	13.1462840	-1.7227780	5.1000200
H	13.6855490	-1.0937560	5.7955010
C	13.2179000	-1.4872120	3.7503800
C	12.4998900	-2.2739770	2.8273150
H	12.5471640	-2.0467380	1.7692530
C	11.7471460	-3.3289480	3.2865820
H	11.1995580	-3.9243760	2.5713710
C	12.6354380	-1.9783130	7.9898270
C	12.1367820	-0.6763560	7.8641820
H	11.4964050	-0.4483840	7.0207190
C	12.4302120	0.3066170	8.8122060
C	13.2553720	-0.0478610	9.8882790
H	13.4969450	0.7012650	10.6285210
C	13.7817710	-1.3398130	10.0376700
C	13.4493640	-2.3025340	9.0781440
H	13.8329260	-3.3116860	9.1592980
C	14.7516820	-1.6929990	11.1725470
C	14.4902450	-3.1249240	11.6803380
H	13.4319370	-3.2805350	11.9029970
H	15.0652930	-3.3182210	12.5910110
H	14.7870610	-3.8820550	10.9509430
C	14.6186290	-0.7183110	12.3559810
H	14.9453410	0.2921350	12.0944020
H	15.2440800	-1.0579240	13.1874380
H	13.5849790	-0.6604200	12.7065990
C	16.1926110	-1.6162680	10.6199860
H	16.3270390	-2.3110950	9.7847960
H	16.9160630	-1.8762280	11.4007560
H	16.4208620	-0.6070810	10.2624650
C	10.0082130	-5.5721040	4.3054580
C	10.4016170	-6.2603450	3.1130500
C	9.4062870	-6.8037710	2.2302960
C	9.8278110	-7.3521850	0.9890890
H	9.0905960	-7.6977170	0.2779980
C	11.1647960	-7.4357660	0.6892370
C	12.1596740	-6.9978710	1.5893970
H	13.2080940	-7.1133690	1.3411340
C	11.7730600	-6.4161640	2.7731040
H	12.5278270	-6.0648080	3.4670670
C	8.0208250	-6.7545570	2.6049370
C	7.6863930	-6.1979970	3.8241810
H	6.6600110	-6.2016400	4.1703570

C	8.6637530	-5.6098860	4.6393870
C	6.9246710	-7.2552970	1.7382790
C	5.8205880	-6.4192690	1.4879210
H	5.8217680	-5.4258330	1.9184050
C	4.7533980	-6.8520220	0.7008810
C	4.8170350	-8.1487410	0.1601440
H	3.9972770	-8.4897960	-0.4585800
C	5.8908360	-9.0093510	0.3965210
C	6.9408830	-8.5440520	1.2017900
H	7.7739770	-9.1983430	1.4325850
C	3.5262850	-5.9757420	0.4071470
C	3.6188890	-4.5943450	1.0727810
H	3.7048220	-4.6743170	2.1585110
H	2.7165350	-4.0165550	0.8499410
H	4.4723880	-4.0192330	0.7020970
C	2.2611390	-6.6816000	0.9428000
H	2.1193180	-7.6649180	0.4858740
H	1.3725850	-6.0796930	0.7252250
H	2.3206630	-6.8172620	2.0272330
C	3.3979790	-5.7704080	-1.1178570
H	4.2853410	-5.2672510	-1.5157890
H	2.5238160	-5.1500190	-1.3426780
H	3.2814710	-6.7195060	-1.6483010
C	5.9630850	-10.4297790	-0.1807420
C	4.7521140	-10.7637970	-1.0668290
H	3.8134130	-10.7172350	-0.5057730
H	4.8550060	-11.7805270	-1.4579000
H	4.6756620	-10.0843900	-1.9217900
C	6.0125200	-11.4468550	0.9803690
H	6.8820130	-11.2839770	1.6233780
H	6.0715880	-12.4675250	0.5876150
H	5.1141200	-11.3704840	1.6011990
C	7.2420920	-10.5632350	-1.0367270
H	7.2323000	-9.8455750	-1.8634450
H	7.3112920	-11.5711900	-1.4594530
H	8.1463010	-10.3854950	-0.4476150
Cl	0.4281230	-8.0222550	18.4573970
Cl	-1.7251340	-0.0156780	15.2764580
P	4.5153010	-4.9986360	11.0274880
O	4.7592560	-4.4909090	12.5510960
O	2.8970770	-5.1490500	10.9804850
O	5.0918010	-6.3838270	10.7893170
O	4.9477740	-3.8412480	10.1241510
C	4.1401900	-5.1959480	13.5866570
C	4.9661750	-6.0059620	14.3743030
H	6.0030660	-6.1234810	14.0911440
C	4.4769460	-6.6551290	15.4895210
C	3.0698520	-6.5803990	15.7580350
C	2.2157770	-5.8344220	14.8736150

C	2.7828330	-5.0515140	13.8110190
C	5.8416030	-11.7184990	16.5109000
H	5.6644590	-12.7678880	16.7696140
H	6.7318830	-11.6683170	15.8757890
H	4.9878890	-11.3700230	15.9226490
C	4.7641710	-10.9708200	18.6652850
H	3.8804910	-10.6024140	18.1364520
H	4.8794120	-10.3814440	19.5807140
H	4.5741930	-12.0112230	18.9497180
C	7.2098730	-11.4739250	18.5868430
H	6.9862700	-12.5130730	18.8466290
H	7.3868770	-10.9279680	19.5190250
H	8.1367550	-11.4696320	18.0046510
C	6.0355180	-10.8792420	17.7928530
C	2.4908820	-7.2256880	16.8834320
H	3.1279850	-7.7271160	17.5991280
C	1.1291560	-7.2154770	17.0590670
C	0.2663160	-6.5825630	16.1394020
H	-0.8073550	-6.6292980	16.2781340
C	0.8107290	-5.9011900	15.0769800
H	0.1529590	-5.4041300	14.3759890
C	5.4585500	-7.3869140	16.3265470
C	5.2809700	-8.7207430	16.6981000
H	4.3748110	-9.2347170	16.3982920
C	6.2748750	-9.4128510	17.4064540
C	7.4557740	-8.7328230	17.7151220
H	8.2403000	-9.2586960	18.2425670
C	7.6713580	-7.3900250	17.3568780
C	6.6548260	-6.7299030	16.6689440
H	6.7704970	-5.6941710	16.3757090
C	9.0181100	-6.7195710	17.6683330
C	8.9766440	-5.1977270	17.4461660
H	8.7885020	-4.9417140	16.4018240
H	9.9406360	-4.7596530	17.7227880
H	8.2047470	-4.7233900	18.0617890
C	10.0832730	-7.3225230	16.7245110
H	10.1551570	-8.4072260	16.8487320
H	11.0684270	-6.8914280	16.9311650
H	9.8349480	-7.1167330	15.6780640
C	9.4237490	-6.9763850	19.1345890
H	8.6702030	-6.5797850	19.8226590
H	10.3763810	-6.4810480	19.3492680
H	9.5518840	-8.0403820	19.3494400
C	1.9982830	-4.1163120	12.9663650
C	1.1749030	-3.0769430	13.5214940
C	0.4371260	-2.2048060	12.6533060
C	-0.4785770	-1.2792090	13.2233540
H	-1.0897080	-0.6658990	12.5764150
C	-0.5861810	-1.1639680	14.5859230

C	0.2132590	-1.9338440	15.4556130
H	0.1393150	-1.7939760	16.5273210
C	1.0739320	-2.8644200	14.9238270
H	1.6848260	-3.4526590	15.5930710
C	0.6261990	-2.2822170	11.2332150
C	1.4600730	-3.2599490	10.7277520
H	1.6607560	-3.3248480	9.6662950
C	2.1045990	-4.1654430	11.5850130
C	-0.0115630	-1.3153480	10.3096840
C	-0.6988980	-1.7685290	9.1776210
H	-0.7786870	-2.8350840	9.0113060
C	-1.2705320	-0.8641910	8.2745150
C	-1.1529240	0.5019610	8.5527470
H	-1.6135730	1.2122710	7.8764450
C	-0.4444680	0.9915900	9.6580640
C	0.1274140	0.0622990	10.5290450
H	0.7082690	0.3918370	11.3793720
C	-2.0082040	-1.3270270	7.0117830
C	-1.7252280	-2.8045840	6.7044100
H	-0.6515700	-2.9890960	6.6176540
H	-2.1914200	-3.0877590	5.7566650
H	-2.1198250	-3.4748020	7.4739160
C	-1.5364560	-0.4926760	5.8005900
H	-1.8335350	0.5563470	5.8789700
H	-1.9747540	-0.8901450	4.8789520
H	-0.4475090	-0.5258300	5.7102180
C	-3.5256020	-1.1325890	7.2131220
H	-4.0730370	-1.4455200	6.3172470
H	-3.7669910	-0.0836650	7.4113110
H	-3.8838710	-1.7285070	8.0588140
C	-0.3403330	2.5054950	9.8857530
C	-1.7493050	3.0494230	10.2099290
H	-2.4503890	2.8508080	9.3938050
H	-1.7120160	4.1324890	10.3694010
H	-2.1464370	2.5827400	11.1173240
C	0.1948510	3.1946940	8.6120050
H	1.1843930	2.8118420	8.3489670
H	0.2770580	4.2742740	8.7764740
H	-0.4657150	3.0394980	7.7547810
C	0.5998150	2.8530880	11.0521430
H	0.2408400	2.4438920	12.0017010
H	0.6594230	3.9398780	11.1635350
H	1.6131440	2.4783320	10.8763550
Cl	11.1482880	1.0942220	16.1080960
Cl	16.5066910	-6.5507940	15.5169220
P	8.5042960	-5.5679560	11.2118320
O	8.6303220	-5.8855490	12.7961930
O	10.0158220	-5.0877440	10.8411990
O	7.6339320	-4.3165910	11.0374430

O	8.0918510	-6.8411720	10.5010070
C	8.9529520	-4.7251770	13.5210030
C	7.8657350	-3.9908380	14.0061620
H	6.8675390	-4.3503420	13.8192050
C	8.0558530	-2.7809250	14.6213330
C	9.3845380	-2.2797380	14.7572710
C	10.4958020	-3.0361330	14.2432950
C	10.2651770	-4.3070580	13.6007060
C	3.9536000	-0.8470140	12.0367230
H	3.3905520	-0.3174530	11.2617600
H	3.4064080	-1.7538290	12.2993440
H	4.9094550	-1.1544630	11.6043970
C	4.9917680	1.2857180	12.8879930
H	4.4956470	1.8441420	12.0865250
H	5.9832190	0.9984360	12.5313060
H	5.1219260	1.9567960	13.7432690
C	2.7695280	0.5464150	13.7451670
H	2.8489900	1.1950750	14.6235970
H	2.1013640	-0.2812340	13.9894340
H	2.3012830	1.1289410	12.9466950
C	4.1476960	0.0542120	13.2772800
C	9.6051950	-1.0062910	15.3409480
H	8.7574370	-0.4475090	15.7180500
C	10.8766430	-0.4932610	15.4025130
C	11.9809310	-1.2061690	14.8911060
H	12.9733270	-0.7759800	14.9441000
C	11.7845410	-2.4438850	14.3210330
H	12.6385480	-2.9695840	13.9191190
C	6.8204660	-2.0555060	15.0278360
C	6.1553500	-1.2588900	14.0924800
H	6.6292820	-1.0797630	13.1331160
C	4.8781730	-0.7521560	14.3615020
C	4.2984360	-1.0536780	15.6028480
H	3.3056010	-0.6853300	15.8155290
C	4.9513750	-1.8351720	16.5672310
C	6.2225610	-2.3323440	16.2583420
H	6.7471150	-2.9735890	16.9583290
C	4.3046210	-2.2204330	17.9055240
C	4.0201320	-3.7395340	17.8940810
H	3.3514730	-4.0070040	17.0712760
H	3.5447100	-4.0480260	18.8314780
H	4.9384670	-4.3213240	17.7740370
C	2.9833000	-1.4748130	18.1503840
H	3.1281340	-0.3897750	18.1569070
H	2.5711460	-1.7665400	19.1209820
H	2.2371000	-1.7125650	17.3893290
C	5.2635580	-1.8891730	19.0689120
H	6.2062330	-2.4377920	18.9923250
H	4.8006490	-2.1578250	20.0242970

H	5.4955000	-0.8194750	19.0901460
C	11.3165470	-5.0905340	12.8968020
C	12.5299020	-5.5226730	13.5300710
C	13.5782950	-6.1339840	12.7599750
C	14.8151950	-6.4156990	13.3992120
H	15.6423270	-6.8006650	12.8195840
C	14.9656290	-6.1925600	14.7456750
C	13.9131440	-5.6865340	15.5367310
H	14.0464670	-5.5556130	16.6040430
C	12.7265720	-5.3539590	14.9275360
H	11.9191110	-4.9521140	15.5260010
C	13.3668080	-6.4120880	11.3665720
C	12.1464120	-6.0845810	10.8126760
H	11.9265640	-6.3207690	9.7783280
C	11.1543290	-5.4357630	11.5616370
C	14.3937590	-7.0163660	10.4821740
C	14.6372380	-6.4288140	9.2267760
H	14.0879130	-5.5344410	8.9631260
C	15.5603700	-6.9771400	8.3378520
C	16.2538440	-8.1326670	8.7403150
H	16.9814900	-8.5591030	8.0620140
C	16.0327490	-8.7495770	9.9735080
C	15.0831210	-8.1786540	10.8341120
H	14.8573260	-8.6560990	11.7807570
C	15.8448560	-6.3692210	6.9560360
C	14.9721210	-5.1380330	6.6665020
H	13.9078880	-5.3853430	6.6847720
H	15.2034890	-4.7477450	5.6708200
H	15.1482280	-4.3303950	7.3837110
C	15.5520830	-7.4243140	5.8662830
H	16.1687620	-8.3191500	5.9876150
H	15.7571190	-7.0072190	4.8744190
H	14.5014580	-7.7308820	5.8970140
C	17.3281280	-5.9471550	6.8833770
H	17.5529530	-5.1921970	7.6438760
H	17.5522390	-5.5195710	5.9002750
H	17.9995690	-6.7958840	7.0419510
C	16.7759520	-10.0182050	10.4145350
C	17.8007560	-10.4880450	9.3693300
H	17.3230810	-10.7386710	8.4169060
H	18.3074950	-11.3868730	9.7333470
H	18.5651970	-9.7275670	9.1805070
C	15.7539780	-11.1538310	10.6394640
H	15.0206630	-10.8916760	11.4072300
H	16.2667050	-12.0660830	10.9625830
H	15.2110870	-11.3753640	9.7150600
C	17.5252740	-9.7314580	11.7347030
H	18.2513100	-8.9229530	11.6013960
H	18.0650390	-10.6251070	12.0654560
H	16.8399350	-9.4402870	12.5358640
Rh	6.4345320	-6.6364260	9.1942610
Rh	6.7858270	-4.0483470	9.1859560
C	7.1379630	-2.0875180	9.1503090
C	8.0136470	-1.3679440	10.0042930
C	9.1930610	-1.9712060	10.5185260
C	7.7034290	-0.0268830	10.3704270
C	10.0265960	-1.2555150	11.3628990
H	9.4606750	-2.9640860	10.1935640
C	8.4978340	0.6388330	11.2907680
C	9.6629690	0.0292420	11.7803740
H	8.2360040	1.6419490	11.6079160
C	6.5275980	-1.3787000	8.0088460
O	5.2348210	-1.0664910	8.1087150
C	4.6911070	-0.4175810	6.9499020
H	4.4974720	-1.1545160	6.1684130
H	5.3833170	0.3407090	6.5834480
O	7.2259940	-1.1804170	7.0305710
H	10.9516140	-1.6963590	11.7111880
H	6.8123020	0.4462820	9.9711640
C	3.3838160	0.2482920	7.3592290
Cl	2.1565150	-0.9666560	7.8056290
Cl	2.8091550	1.1892280	5.9404130
Cl	3.6717190	1.3616730	8.7463950
H	10.2974010	0.5658220	12.4779280

{Rh$_2$(*S*-megaBNP)$_4$}-[(*p*-BrPh)-TCE] carbene complex

Cl	0.8999070	3.3360540	2.9491770
Cl	-4.9968980	-4.2207130	3.3265220
P	3.1309800	-3.6929760	7.3331330
O	2.9028240	-4.0035680	5.7542050
O	1.6981160	-3.0753620	7.7878800
O	4.1511580	-2.5578340	7.4695920
O	3.4139240	-5.0215280	8.0111630
C	2.6401390	-2.8246370	5.0343850
C	3.7342420	-2.2432760	4.3848620
H	4.6886620	-2.7504620	4.4130540
C	3.6265190	-0.9919250	3.8247000
C	2.3788170	-0.2986600	3.9153110
C	1.2533580	-0.9282180	4.5504560
C	1.3885890	-2.2460570	5.1046370
C	8.9838840	1.4496110	4.7080460
H	9.5894670	2.2047250	5.2206550
H	9.5617030	1.0862980	3.8563450
H	8.8320120	0.6074740	5.3901120
C	6.9097960	2.5508890	5.5456640
H	6.7076660	1.7283370	6.2383160
H	5.9578050	3.0367080	5.3092750

H	7.5367610	3.2817680	6.0661520
C	7.8918880	3.2719840	3.3576590
H	8.4775260	4.0325220	3.8856590
H	6.9483420	3.7262310	3.0386140
H	8.4474760	2.9823690	2.4608980
C	7.6337890	2.0608770	4.2793810
C	2.2441900	1.0204400	3.4092990
H	3.0889170	1.4883620	2.9193020
C	1.0566250	1.6929840	3.5608380
C	-0.0513130	1.1053340	4.2080090
H	-0.9693470	1.6672750	4.3297000
C	0.0535240	-0.1797430	4.6861170
H	-0.7932770	-0.6237290	5.1927630
C	4.8688080	-0.4151420	3.2494740
C	5.5529690	0.5996590	3.9354620
H	5.0996040	1.0084760	4.8288120
C	6.8198580	1.0065800	3.5162090
C	7.3688590	0.3969230	2.3719610
H	8.3521590	0.7089320	2.0433830
C	6.7027960	-0.6052390	1.6616660
C	5.4429020	-1.0084880	2.1276570
H	4.9020520	-1.8039320	1.6260750
C	7.2979700	-1.3040280	0.4317560
C	7.4655140	-2.8073910	0.7439600
H	8.1058570	-2.9541460	1.6170500
H	7.9234650	-3.3286150	-0.1033350
H	6.5077570	-3.2904740	0.9549200
C	8.6688960	-0.7287650	0.0440770
H	8.6046990	0.3343480	-0.2088840
H	9.0576210	-1.2594480	-0.8301340
H	9.3949730	-0.8413240	0.8531550
C	6.3420740	-1.1356600	-0.7689450
H	5.3592840	-1.5711600	-0.5672180
H	6.7541090	-1.6345080	-1.6526790
H	6.2000490	-0.0769110	-1.0083490
C	0.3036410	-2.9595230	5.8297430
C	-0.9425710	-3.3121530	5.2156240
C	-1.9392860	-4.0240590	5.9660440
C	-3.1976100	-4.2789690	5.3577700
H	-3.9807070	-4.7560990	5.9305290
C	-3.4260470	-3.9064640	4.0565910
C	-2.4327000	-3.2736420	3.2805060
H	-2.6297220	-3.0163360	2.2465450
C	-1.2202880	-2.9838960	3.8604950
H	-0.4579990	-2.4912410	3.2709070
C	-1.6616970	-4.4347260	7.3147210
C	-0.4468130	-4.0920990	7.8751060
H	-0.1862380	-4.3982990	8.8812820
C	0.5085080	-3.3799360	7.1334880
C	-2.6074330	-5.2586150	8.1086420
C	-2.9260210	-4.8992210	9.4297730
H	-2.5079180	-3.9842600	9.8316720
C	-3.7635360	-5.7011600	10.2074390
C	-4.2835560	-6.8731310	9.6301040
H	-4.9365350	-7.4957380	10.2280610
C	-3.9837500	-7.2629490	8.3236930
C	-3.1307960	-6.4396890	7.5759550
H	-2.8482720	-6.7284930	6.5699580
C	-4.1296970	-5.3589600	11.6583710
C	-3.4896840	-4.0436430	12.1194870
H	-2.3992800	-4.0892940	12.0660840
H	-3.7636080	-3.8398510	13.1588670
H	-3.8229090	-3.1923840	11.5186350
C	-3.6301590	-6.4876730	12.5876110
H	-4.0777450	-7.4524810	12.3333660
H	-3.8903980	-6.2606960	13.6275550
H	-2.5425410	-6.5925670	12.5233910
C	-5.6625640	-5.2261770	11.7865820
H	-6.0377430	-4.4315670	11.1335600
H	-5.9339840	-4.9775960	12.8181660
H	-6.1760930	-6.1538620	11.5190670
C	-4.5391140	-8.5430700	7.6849710
C	-5.4690560	-9.3143900	8.6355800
H	-4.9471490	-9.6323920	9.5436890
H	-5.8406020	-10.2131690	8.1340820
H	-6.3362780	-8.7148010	8.9300160
C	-3.3657470	-9.4684660	7.2953220
H	-2.6907120	-8.9880530	6.5815460
H	-3.7451080	-10.3859270	6.8329030
H	-2.7812230	-9.7466540	8.1781270
C	-5.3404980	-8.1683010	6.4185160
H	-6.1802830	-7.5125360	6.6703570
H	-5.7412710	-9.0700580	5.9434270
H	-4.7186680	-7.6506530	5.6824770
Cl	13.1831200	0.8084370	3.1543320
Cl	10.5508700	-7.0724950	-0.1024220
P	7.0826600	-3.9674420	7.6508850
O	8.6659030	-4.3003900	7.5771510
O	6.7483100	-3.5083640	6.1290750
O	6.9035440	-2.7019150	8.4952530
O	6.3707050	-5.2388670	8.0682080
C	9.4519740	-3.3190250	6.9528390
C	10.0743370	-2.3812850	7.7901170
H	9.8870700	-2.4333780	8.8553660
C	10.8572960	-1.3803860	7.2536410
C	11.0510210	-1.3454380	5.8304200
C	10.3169350	-2.2433640	4.9836080
C	9.5039610	-3.2745310	5.5705120

C	10.3829050	3.8921890	8.0073350
H	10.3344980	4.9089920	7.6040210
H	9.8790770	3.8874730	8.9760120
H	9.8244900	3.2382880	7.3326190
C	12.5630160	3.5686430	6.7905060
H	12.0786550	2.9477430	6.0309180
H	13.6082350	3.2518690	6.8657450
H	12.5430690	4.6059630	6.4394410
C	12.5420690	4.4084170	9.1479850
H	12.4521050	5.4371710	8.7860910
H	13.6083650	4.1853210	9.2528810
H	12.0803960	4.3601380	10.1388280
C	11.8547790	3.4526670	8.1599500
C	11.9600660	-0.4287460	5.2361290
H	12.5747810	0.1977820	5.8664940
C	12.0590060	-0.3387570	3.8712230
C	11.2573220	-1.1277350	3.0221380
H	11.3246330	-1.0102340	1.9473350
C	10.4062600	-2.0537550	3.5770850
H	9.7998450	-2.6588530	2.9196650
C	11.4499250	-0.3546480	8.1402410
C	11.3252200	1.0027970	7.8300890
H	10.7579130	1.2846910	6.9524520
C	11.9296730	1.9884860	8.6170020
C	12.6286860	1.5762050	9.7557930
H	13.1074910	2.3220900	10.3741810
C	12.7311420	0.2251610	10.1277640
C	12.1371790	-0.7315560	9.3015560
H	12.2207360	-1.7852280	9.5339320
C	13.5405170	-0.1880740	11.3641090
C	13.0937530	-1.5647410	11.8882490
H	12.0119300	-1.6006600	12.0417240
H	13.5795830	-1.7807510	12.8442160
H	13.3598680	-2.3759600	11.2054010
C	13.3643370	0.8437850	12.4954190
H	13.8246530	1.8054330	12.2530310
H	13.8387830	0.4782990	13.4118790
H	12.3056620	1.0194090	12.6974410
C	15.0321370	-0.2641610	10.9710620
H	15.1873460	-1.0040200	10.1791060
H	15.6414040	-0.5534900	11.8346170
H	15.3915520	0.7036040	10.6072260
C	8.6282960	-4.1675420	4.7681010
C	9.0848420	-4.9556010	3.6604820
C	8.1382880	-5.5956030	2.7868260
C	8.6228510	-6.2183750	1.6050130
H	7.9234730	-6.6229890	0.8872410
C	9.9730340	-6.2983080	1.3691460
C	10.9199640	-5.7828720	2.2798110
H	11.9801360	-5.9028500	2.0905050
C	10.4721260	-5.1171930	3.3955990
H	11.1908540	-4.7020590	4.0920110
C	6.7392130	-5.5686190	3.1121820
C	6.3502170	-4.9256140	4.2717170
H	5.3163090	-4.9412500	4.5943750
C	7.2738420	-4.2231670	5.0541640
C	5.6723630	-6.1746150	2.2787880
C	4.4985680	-5.4343090	2.0636740
H	4.4419790	-4.4318390	2.4686260
C	3.4189970	-5.9644670	1.3547510
C	3.5549310	-7.2587390	0.8284120
H	2.7361100	-7.6805200	0.2638880
C	4.7098850	-8.0267490	1.0192670
C	5.7582700	-7.4725580	1.7647410
H	6.6405810	-8.0656870	1.9749670
C	2.1030480	-5.1787340	1.2475300
C	2.3631110	-3.6694190	1.0588030
H	2.8625740	-3.2234040	1.9213400
H	1.4109840	-3.1438840	0.9338000
H	2.9758940	-3.4806820	0.1709650
C	1.3221290	-5.3943710	2.5649140
H	1.1038080	-6.4564750	2.7156250
H	0.3727190	-4.8496520	2.5448210
H	1.8970280	-5.0446100	3.4268010
C	1.2350130	-5.6630690	0.0720510
H	1.7767040	-5.6007930	-0.8773830
H	0.3411730	-5.0364880	-0.0047900
H	0.8965210	-6.6942720	0.2067330
C	4.8608450	-9.4481820	0.4579170
C	3.6336500	-9.8899260	-0.3558510
H	2.7261910	-9.9091300	0.2558090
H	3.7957350	-10.9011110	-0.7411860
H	3.4577970	-9.2310190	-1.2119830
C	5.0512720	-10.4392360	1.6272690
H	5.9409960	-10.2034230	2.2179410
H	5.1639500	-11.4592550	1.2444430
H	4.1864120	-10.4192230	2.2981370
C	6.0984270	-9.4989700	-0.4652320
H	5.9851930	-8.8038360	-1.3033720
H	6.2262300	-10.5076970	-0.8722410
H	7.0157100	-9.2355500	0.0691300
Cl	-0.4211660	-8.6145550	17.2653070
Cl	-4.2749980	-0.2204050	16.0366150
P	2.7495260	-3.2927660	11.1205740
O	2.8776260	-2.7785680	12.6639270
O	1.1535200	-3.5476910	10.9848210
O	3.3863430	-4.6615990	10.9404670
O	3.1938710	-2.1371300	10.2241560

C	2.4590580	-3.7571960	13.5829980
C	3.4662070	-4.5546760	14.1383460
H	4.4925560	-4.3580250	13.8562730
C	3.1532000	-5.6358560	14.9371300
C	1.7680190	-5.9089190	15.2054730
C	0.7465890	-5.1022830	14.5929790
C	1.1068140	-3.9694560	13.7884060
C	4.8736630	-10.4771490	13.6519870
H	4.7838280	-11.5509630	13.4555990
H	5.6640840	-10.0762480	13.0090990
H	3.9312430	-10.0036830	13.3631320
C	4.0689520	-10.8029110	16.0199250
H	3.1064620	-10.3343490	15.7957910
H	4.2828730	-10.6422740	17.0815690
H	3.9646570	-11.8800830	15.8513100
C	6.5015740	-10.9827630	15.4788020
H	6.3620480	-12.0527160	15.2968590
H	6.7807390	-10.8564600	16.5296770
H	7.3381360	-10.6468800	14.8579580
C	5.2033810	-10.2317410	15.1409840
C	1.3749760	-6.9765940	16.0564230
H	2.1300390	-7.5468420	16.5804000
C	0.0456850	-7.2846830	16.2107210
C	-0.9647930	-6.5693690	15.5346840
H	-2.0030270	-6.8618550	15.6379290
C	-0.6101940	-5.4963530	14.7519560
H	-1.3847320	-4.9417930	14.2386840
C	4.2724040	-6.5180410	15.3512020
C	4.2115860	-7.8988620	15.1437000
H	3.3009390	-8.3295410	14.7438790
C	5.3205330	-8.7215830	15.3898040
C	6.4985120	-8.1161960	15.8309440
H	7.3720150	-8.7303010	16.0062500
C	6.6015400	-6.7294450	16.0422290
C	5.4726990	-5.9459190	15.8092450
H	5.5053580	-4.8747690	15.9633500
C	7.9512430	-6.1291610	16.4594470
C	7.8461020	-4.6310980	16.7889550
H	7.5380480	-4.0419100	15.9217100
H	8.8239820	-4.2593070	17.1079110
H	7.1354010	-4.4455890	17.6008430
C	8.9450550	-6.3082420	15.2888180
H	9.0641090	-7.3635830	15.0262340
H	9.9281270	-5.9107630	15.5589440
H	8.5961170	-5.7734870	14.4001470
C	8.4937320	-6.8550110	17.7091130
H	7.8006580	-6.7537350	18.5506230
H	9.4552670	-6.4218820	18.0043060
H	8.6526060	-7.9215360	17.5290840

C	0.1250450	-3.0765300	13.1185530
C	-0.8896880	-2.3462880	13.8255820
C	-1.7664630	-1.4562970	13.1157430
C	-2.8263250	-0.8294230	13.8236230
H	-3.5385950	-0.2182340	13.2873360
C	-2.9549000	-1.0069560	15.1785540
C	-2.0477520	-1.8024530	15.9083750
H	-2.1477790	-1.8980720	16.9830600
C	-1.0442580	-2.4567480	15.2336860
H	-0.3502380	-3.0677900	15.7941840
C	-1.5656280	-1.2221240	11.7146630
C	-0.5866940	-1.9441150	11.0622140
H	-0.3832440	-1.7808980	10.0131530
C	0.2218390	-2.8525840	11.7545710
C	-2.3025330	-0.1965260	10.9419080
C	-2.7228640	-0.4833560	9.6325860
H	-2.5756950	-1.4859610	9.2525240
C	-3.3035300	0.4978130	8.8314100
C	-3.4837030	1.7794510	9.3804100
H	-3.9427820	2.5431230	8.7662130
C	-3.0709700	2.1032160	10.6745270
C	-2.4709870	1.0967880	11.4425620
H	-2.0886060	1.3296980	12.4289230
C	-3.7333600	0.2215220	7.3844640
C	-3.3247880	-1.1852850	6.9243700
H	-2.2454920	-1.3368970	7.0154320
H	-3.5939590	-1.3287440	5.8742420
H	-3.8222210	-1.9715510	7.5002450
C	-3.0545510	1.2416020	6.4444040
H	-3.3482730	2.2699020	6.6720880
H	-3.3343320	1.0366840	5.4050820
H	-1.9666780	1.1786500	6.5307170
C	-5.2664020	0.3568690	7.2717670
H	-5.5902430	0.1699460	6.2420120
H	-5.6027360	1.3588590	7.5549050
H	-5.7676080	-0.3655060	7.9241350
C	-3.2262410	3.5065140	11.2762320
C	-3.8744900	4.4938490	10.2927270
H	-3.2754330	4.6094230	9.3838490
H	-3.9592180	5.4780300	10.7634710
H	-4.8810440	4.1759980	10.0028830
C	-1.8353690	4.0539920	11.6663830
H	-1.3375210	3.4139280	12.4002520
H	-1.9331460	5.0528570	12.1050120
H	-1.1862780	4.1250820	10.7887880
C	-4.1137420	3.4171350	12.5371540
H	-5.1060550	3.0283060	12.2865370
H	-4.2376920	4.4087010	12.9858040
H	-3.6752770	2.7593520	13.2932040

Cl	9.9833780	3.3306550	15.7793740
Cl	14.6139480	-4.8005870	15.9296490
P	6.7295840	-3.6263730	11.5612310
O	6.8361350	-3.7018880	13.1767830
O	8.2464510	-3.2119750	11.1383280
O	5.8718660	-2.4079860	11.1990690
O	6.3255530	-4.9897800	11.0417860
C	7.2450530	-2.4655120	13.7186610
C	6.2177730	-1.5571220	14.0057280
H	5.1982690	-1.8603170	13.8065660
C	6.5120820	-0.2575550	14.3556300
C	7.8905990	0.0996060	14.5465960
C	8.9333720	-0.8368130	14.2173320
C	8.5913980	-2.1585620	13.7548380
C	3.9076200	3.9360730	11.4075810
H	3.9865330	4.8520620	10.8115710
H	2.8491720	3.6714470	11.4933160
H	4.4120650	3.1277970	10.8699280
C	6.0028730	4.5831830	12.6370340
H	6.0384540	5.5498800	12.1255630
H	6.5828580	3.8778250	12.0394540
H	6.4986730	4.6922170	13.6069210
C	3.7880590	5.2641720	13.5436310
H	4.1818440	5.3970410	14.5563010
H	2.7153880	5.0687910	13.6190940
H	3.9106260	6.2082680	13.0031020
C	4.5391450	4.1381110	12.8034330
C	8.2435300	1.3785850	15.0504740
H	7.4638530	2.0683960	15.3434470
C	9.5629980	1.7351990	15.1712110
C	10.5974060	0.8603630	14.7907780
H	11.6305770	1.1730680	14.8725990
C	10.2789390	-0.3906830	14.3156170
H	11.0826610	-1.0500720	14.0227880
C	5.3986340	0.7209750	14.3712270
C	5.5198310	1.9440300	13.6959610
H	6.4678410	2.1925660	13.2386760
C	4.4307100	2.8137590	13.5736450
C	3.2074380	2.4109770	14.1214510
H	2.3462540	3.0595040	14.0114390
C	3.0488930	1.1965870	14.8047150
C	4.1623150	0.3636670	14.9278940
H	4.0861300	-0.5801640	15.4525720
C	1.6753090	0.8223140	15.3746810
C	1.7011980	-0.5292940	16.1055050
H	1.9666310	-1.3501830	15.4318930
H	0.7096750	-0.7435760	16.5102460
H	2.4108250	-0.5230310	16.9388420
C	0.6576800	0.7350030	14.2171040
H	0.5753900	1.6858850	13.6821600
H	-0.3337460	0.4808880	14.6015500
H	0.9514370	-0.0379770	13.5007520
C	1.2182160	1.9059680	16.3749610
H	1.9204530	1.9851740	17.2111250
H	0.2319740	1.6530530	16.7786230
H	1.1448310	2.8894440	15.9022180
C	9.5733400	-3.1071060	13.1606460
C	10.7522380	-3.5715890	13.8322990
C	11.7347680	-4.3490900	13.1271490
C	12.9387670	-4.6933630	13.7974850
H	13.7194640	-5.2139130	13.2607370
C	13.1158920	-4.3606050	15.1177170
C	12.1249930	-3.6733460	15.8499970
H	12.2767740	-3.4533390	16.9000100
C	10.9747640	-3.2843110	15.2058780
H	10.2123140	-2.7498850	15.7581050
C	11.4977020	-4.7307250	11.7629280
C	10.3035740	-4.3611940	11.1785940
H	10.0584300	-4.6684780	10.1685890
C	9.3731660	-3.5733230	11.8690540
C	12.4686670	-5.4862660	10.9324800
C	12.7691620	-5.0069980	9.6438750
H	12.3125550	-4.0803890	9.3204700
C	13.6304690	-5.7021410	8.7961850
C	14.1934270	-6.9004580	9.2705750
H	14.8633550	-7.4474700	8.6198990
C	13.9154180	-7.4096100	10.5411020
C	13.0387170	-6.6857000	11.3634100
H	12.7721850	-7.0731000	12.3402510
C	13.9675510	-5.2157670	7.3783370
C	13.3188890	-3.8616360	7.0550230
H	12.2290450	-3.9219520	7.0837200
H	13.6040410	-3.5469720	6.0466230
H	13.6364890	-3.0769050	7.7491140
C	13.4448860	-6.2503610	6.3565900
H	13.8893650	-7.2370340	6.5152710
H	13.6864000	-5.9307090	5.3368950
H	12.3572790	-6.3521200	6.4324340
C	15.4973580	-5.0692610	7.2324780
H	15.8864650	-4.3406500	7.9511370
H	15.7468280	-4.7213700	6.2244460
H	16.0176230	-6.0169160	7.3970150
C	14.5248930	-8.7180020	11.0639430
C	15.4582490	-9.3795260	10.0372950
H	14.9272560	-9.6402070	9.1162900
H	15.8681520	-10.3028780	10.4576130
H	16.3000290	-8.7301280	9.7768860
C	13.3916780	-9.7115770	11.4008070

H	12.7177070	-9.3160070	12.1659580
H	13.8121410	-10.6498190	11.7779410
H	12.7957860	-9.9370370	10.5105820
C	15.3413780	-8.4165340	12.3403630
H	16.1533570	-7.7145900	12.1242750
H	15.7821350	-9.3381340	12.7350350
H	14.7191760	-7.9795400	13.1267530
Rh	4.8615990	-4.9770590	9.5198840
Rh	5.0561280	-2.3589480	9.3171310
C	5.3893470	-0.3790510	9.0848660
C	6.5355630	0.3387050	9.5031130
C	7.6390410	-0.3098790	10.1332670
C	6.5758760	1.7656060	9.4046060
C	8.6475960	0.4153690	10.7391050
H	7.6873760	-1.3826740	10.1406230
C	7.5779410	2.4938400	10.0101130
C	8.5813680	1.8129990	10.7203990
H	7.5799850	3.5748310	9.9610690
C	4.3608920	0.3842190	8.3410930
O	3.5331450	1.0492760	9.1788490
C	2.5453230	1.9070340	8.5995110
H	2.6505260	1.9361750	7.5150930
H	2.6868510	2.8987960	9.0293270
O	4.3125960	0.4133720	7.1307420
H	9.4668340	-0.0863930	11.2377790
H	5.7919420	2.2990980	8.8816960
Br	9.8379790	2.8112400	11.7067690
C	1.1504770	1.4016280	8.9657110
Cl	0.8529290	-0.1741320	8.1748990
Cl	-0.0313490	2.6179440	8.3687200
Cl	1.0094630	1.2329420	10.7459220

8. NMR Spectra

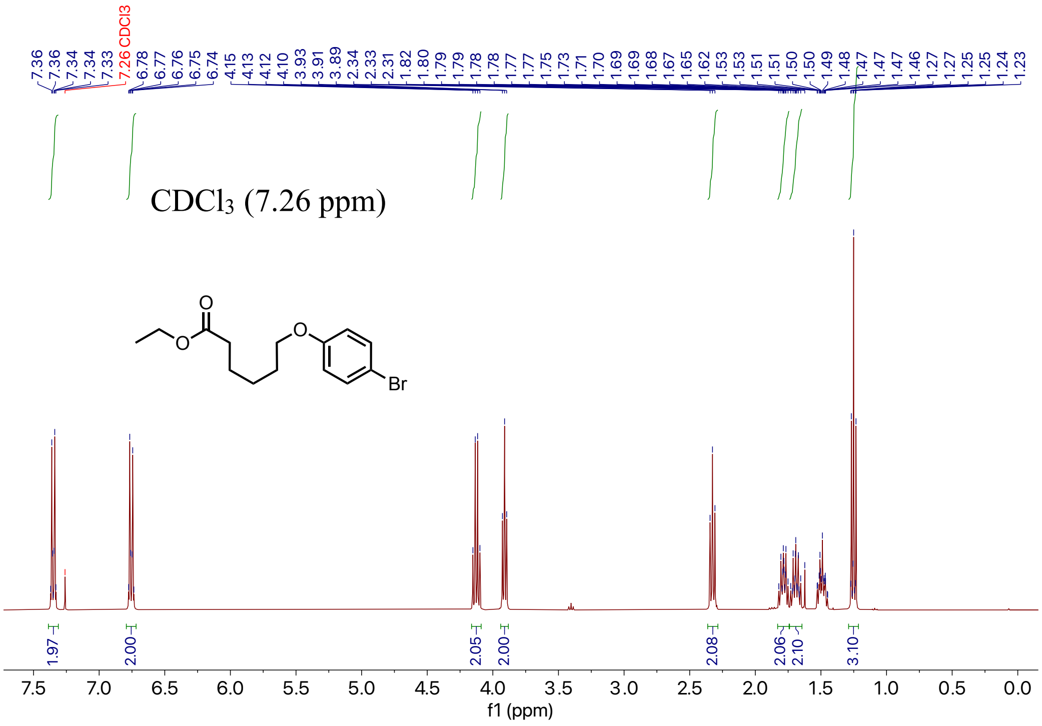

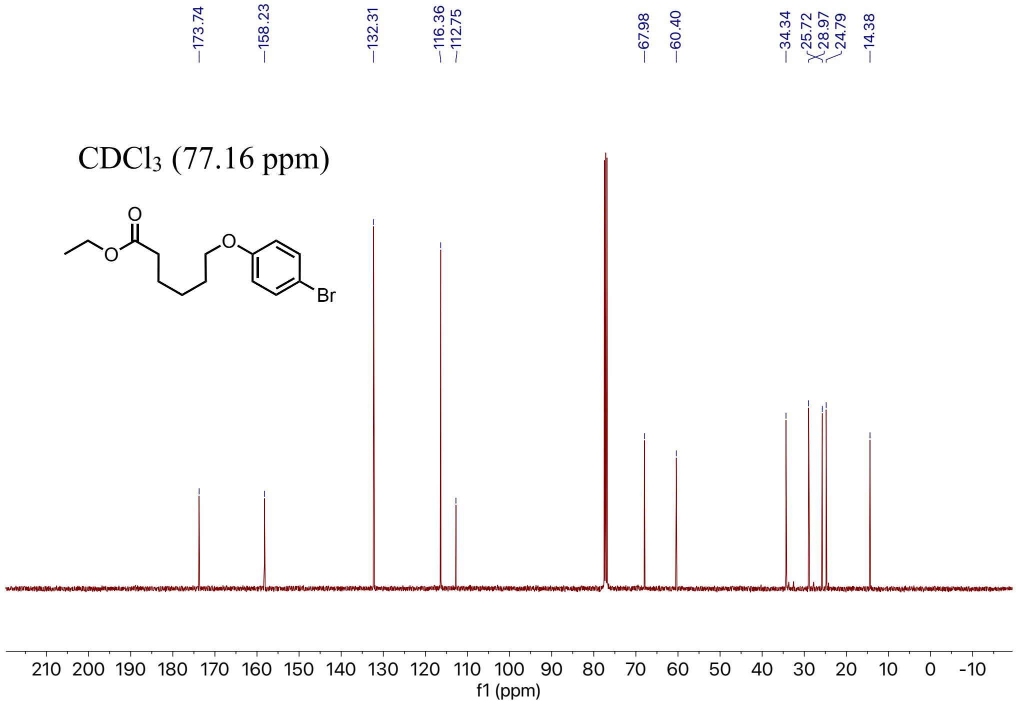

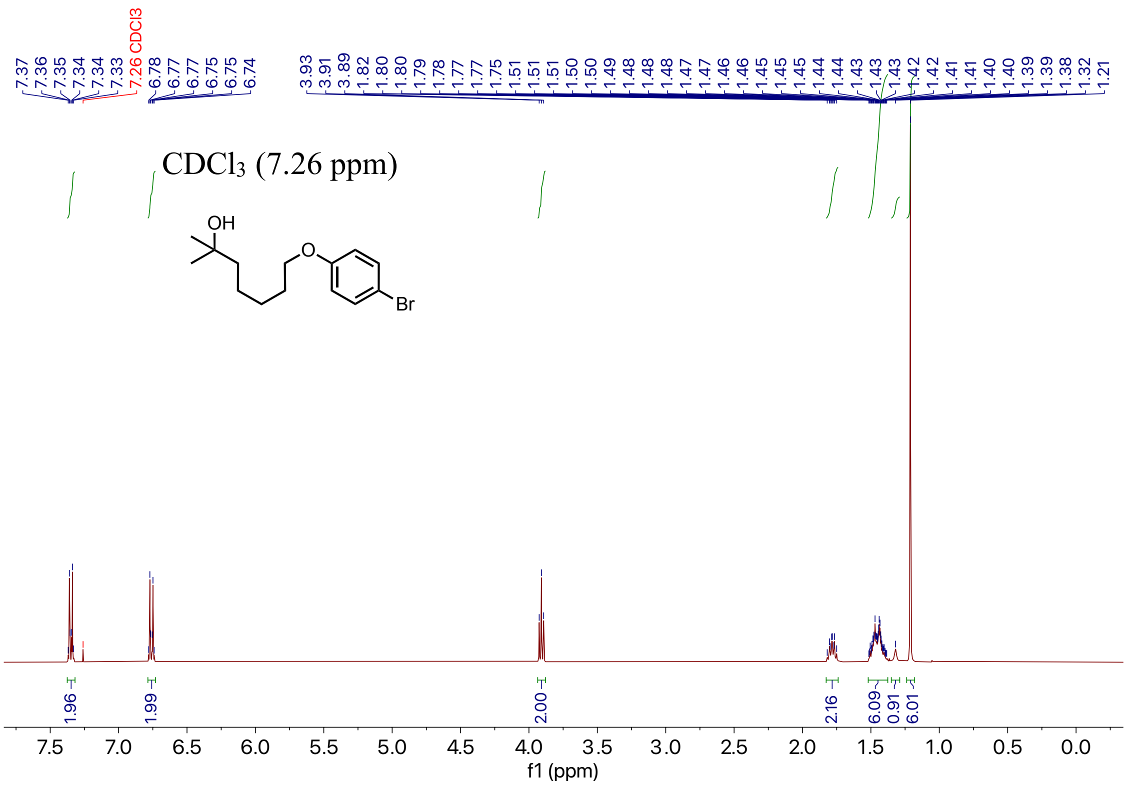

CDCl3 (7.26 ppm)
OH
O
Br
f1 (ppm)

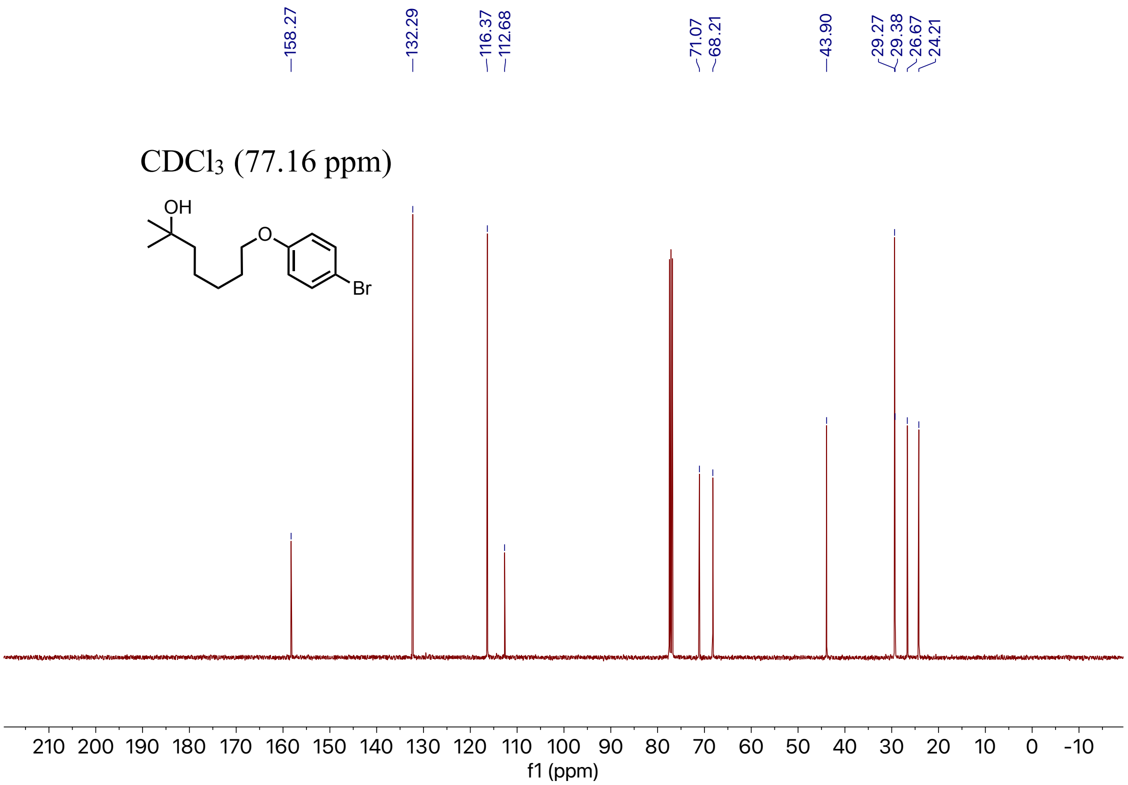

CDCl3 (77.16 ppm)
OH
O
Br
f1 (ppm)

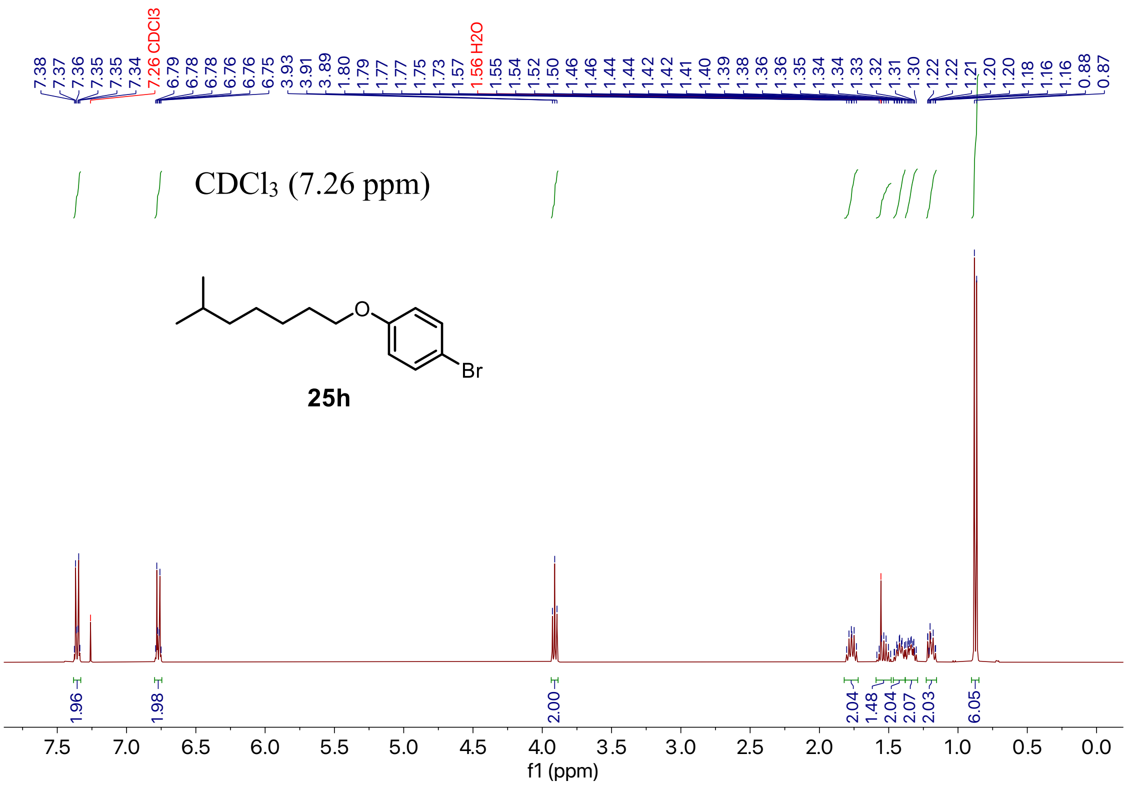
7.38
7.37
7.36
7.35
7.35
7.34
7.26 CDCl3
6.79
6.78
6.78
6.76
6.76
6.75
3.93
3.91
3.89
1.80
1.79
1.77
1.77
1.75
1.73
1.57
1.56 H2O
1.55
1.54
1.52
1.50
1.46
1.46
1.44
1.44
1.42
1.42
1.41
1.40
1.39
1.38
1.36
1.36
1.35
1.34
1.34
1.33
1.32
1.31
1.30
1.22
1.22
1.21
1.20
1.20
1.18
1.16
0.88
0.87
CDCl3 (7.26 ppm)
O
Br
25h
1.96
1.98
2.00
2.04
1.48
2.04
2.07
2.03
6.05
7.5 7.0 6.5 6.0 5.5 5.0 4.5 4.0 3.5 3.0 2.5 2.0 1.5 1.0 0.5 0.0
f1 (ppm)

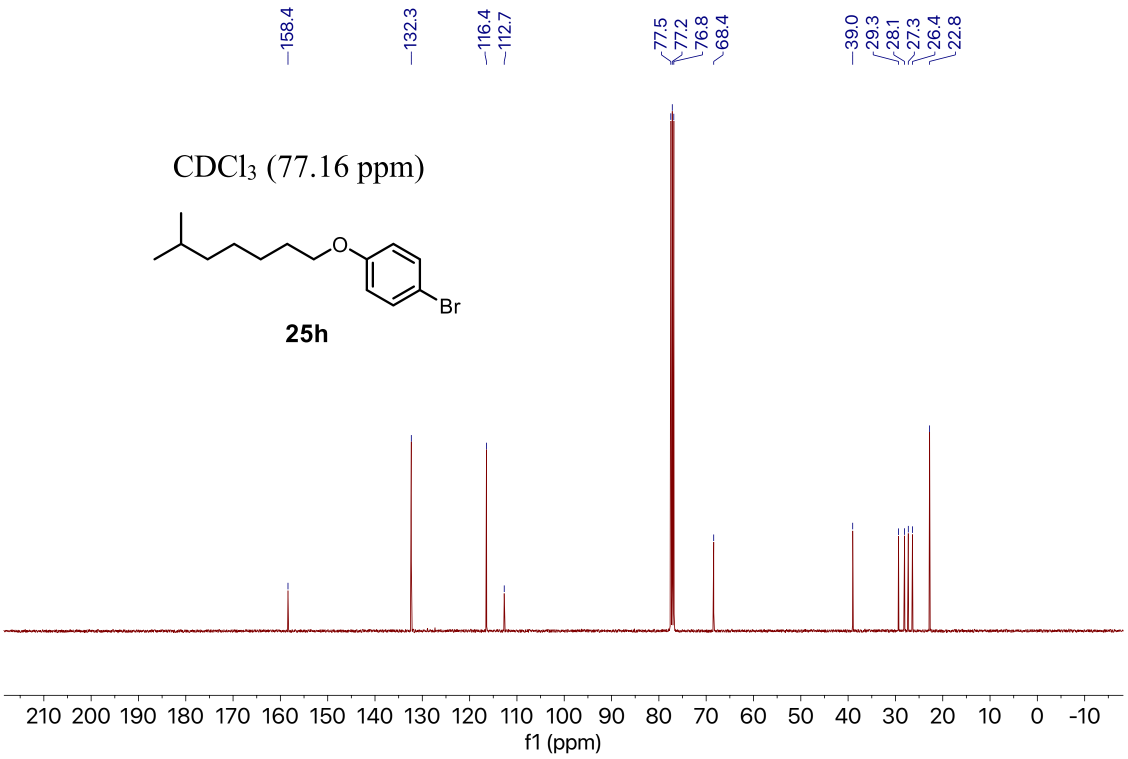
158.4
132.3
116.4
112.7
77.5
77.2
76.8
68.4
39.0
29.3
28.1
27.3
26.4
22.8
CDCl3 (77.16 ppm)
O
Br
25h
210 200 190 180 170 160 150 140 130 120 110 100 90 80 70 60 50 40 30 20 10 0 -10
f1 (ppm)

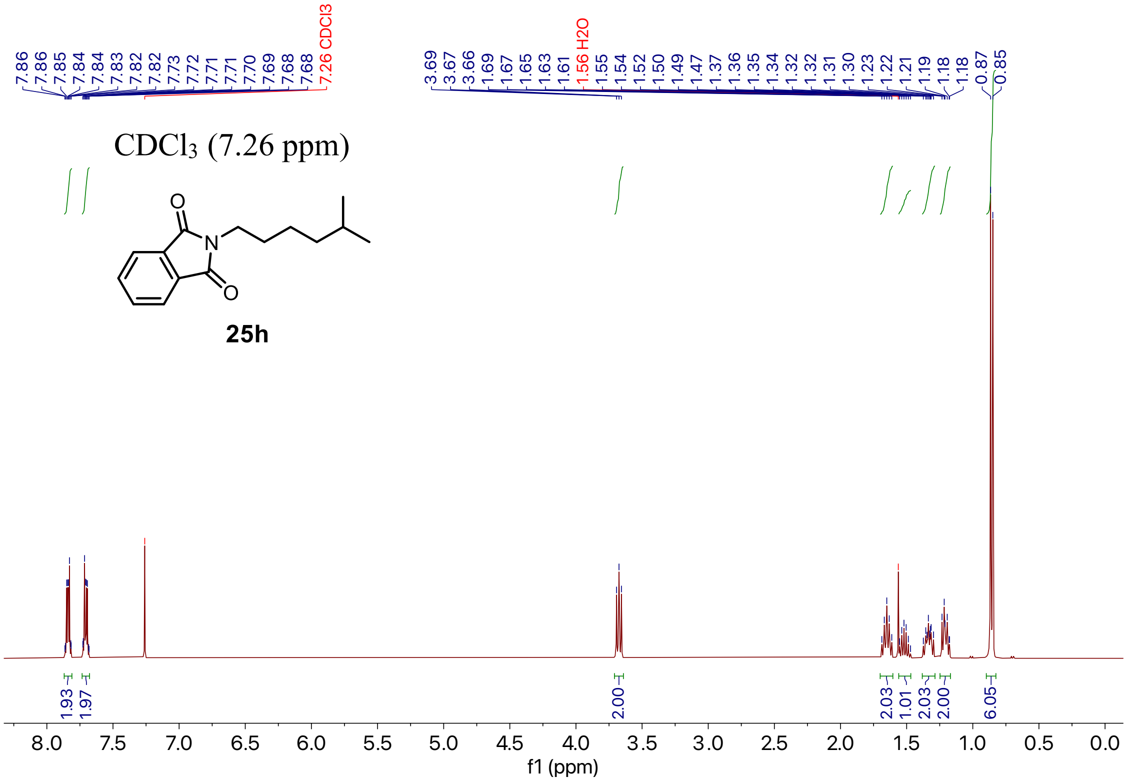
7.86
7.86
7.85
7.84
7.84
7.83
7.82
7.82
7.73
7.72
7.71
7.71
7.70
7.69
7.68
7.68
7.26 CDCl3
3.69
3.67
3.66
1.69
1.67
1.65
1.63
1.61
1.56 H2O
1.55
1.54
1.52
1.50
1.49
1.47
1.37
1.36
1.35
1.34
1.32
1.32
1.31
1.30
1.23
1.22
1.21
1.19
1.18
1.18
0.87
0.85
CDCl3 (7.26 ppm)
O
N
O
25h
1.93
1.97
2.00
2.03
1.01
2.03
2.00
6.05
8.0 7.5 7.0 6.5 6.0 5.5 5.0 4.5 4.0 3.5 3.0 2.5 2.0 1.5 1.0 0.5 0.0
f1 (ppm)

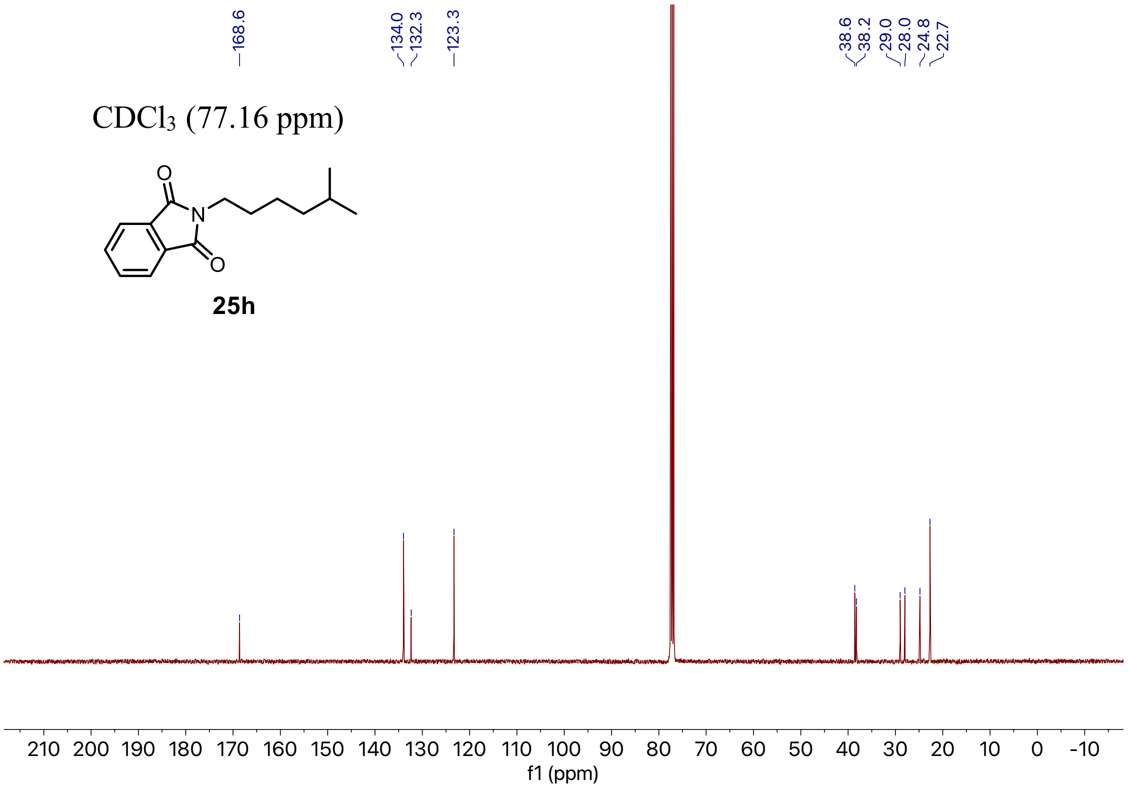
168.6
134.0
132.3
123.3
38.6
38.2
29.0
28.0
24.8
22.7
CDCl3 (77.16 ppm)
O
N
O
25h
210 200 190 180 170 160 150 140 130 120 110 100 90 80 70 60 50 40 30 20 10 0 -10
f1 (ppm)

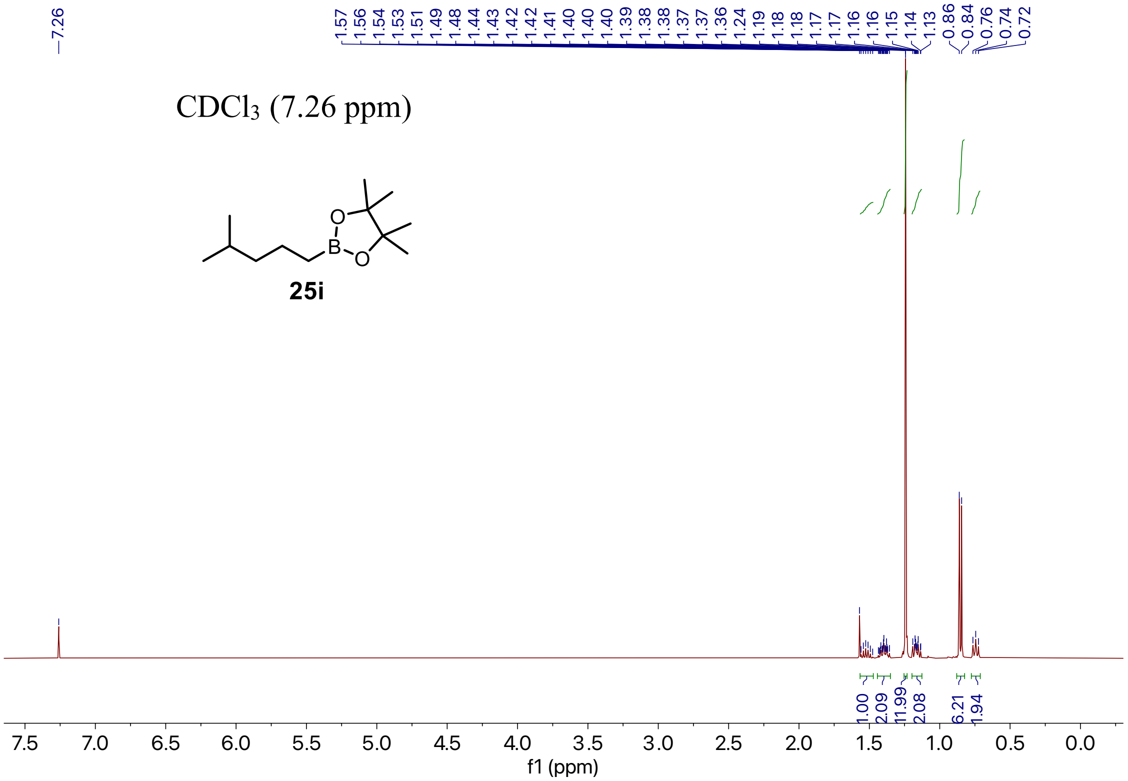
7.26
1.57 1.56 1.54 1.53 1.51 1.49 1.48 1.44 1.43 1.42 1.42 1.41 1.40 1.40 1.40 1.39 1.38 1.38 1.37 1.36 1.24 1.19 1.18 1.18 1.17 1.17 1.16 1.16 1.15 1.14 1.13 0.86 0.84 0.76 0.74 0.72
CDCl3 (7.26 ppm)
O
B
O
25i
1.00
2.09
11.99
2.08
6.21
1.94
7.5 7.0 6.5 6.0 5.5 5.0 4.5 4.0 3.5 3.0 2.5 2.0 1.5 1.0 0.5 0.0
f1 (ppm)

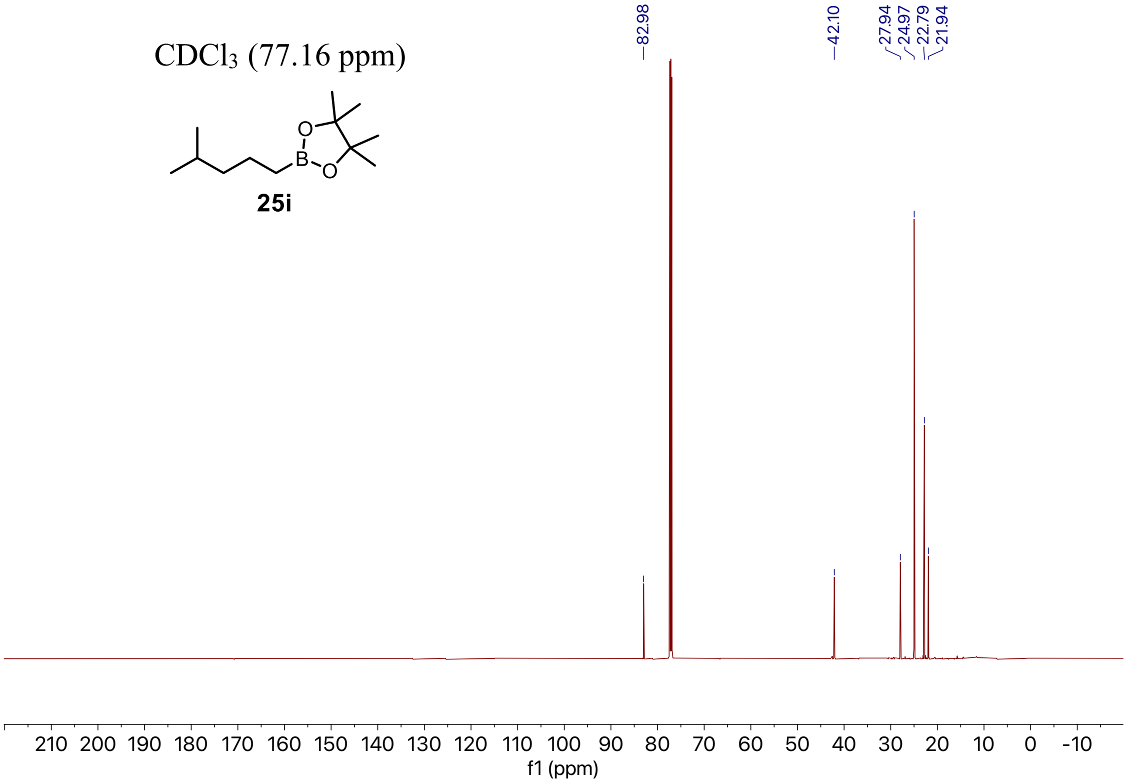
82.98
42.10
27.94 24.97 22.79 21.94
CDCl3 (77.16 ppm)
O
B
O
25i
210 200 190 180 170 160 150 140 130 120 110 100 90 80 70 60 50 40 30 20 10 0 -10
f1 (ppm)

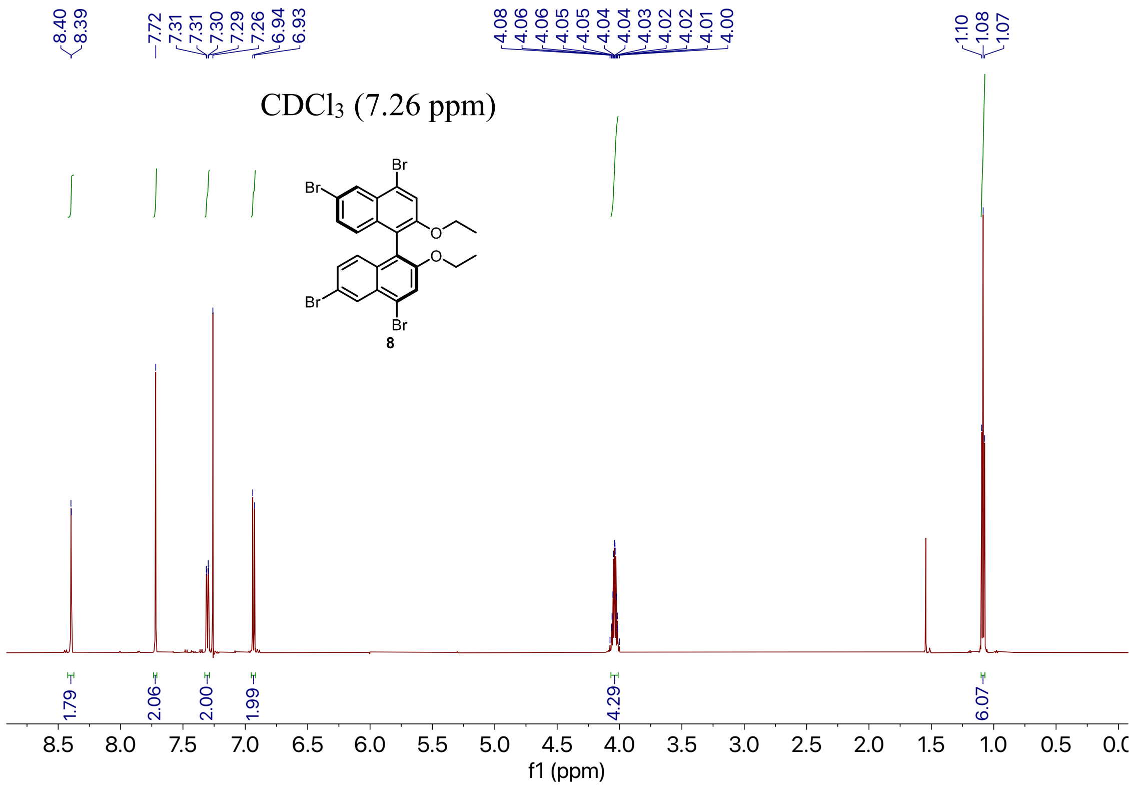
8.40
8.39
7.72
7.31
7.31
7.30
7.29
7.26
6.94
6.93
4.08
4.06
4.06
4.05
4.05
4.04
4.04
4.03
4.02
4.02
4.01
4.00
1.10
1.08
1.07
CDCl3 (7.26 ppm)
Br
Br
O
O
Br
Br
8
1.79
2.06
2.00
1.99
4.29
6.07
8.5 8.0 7.5 7.0 6.5 6.0 5.5 5.0 4.5 4.0 3.5 3.0 2.5 2.0 1.5 1.0 0.5 0.0
f1 (ppm)

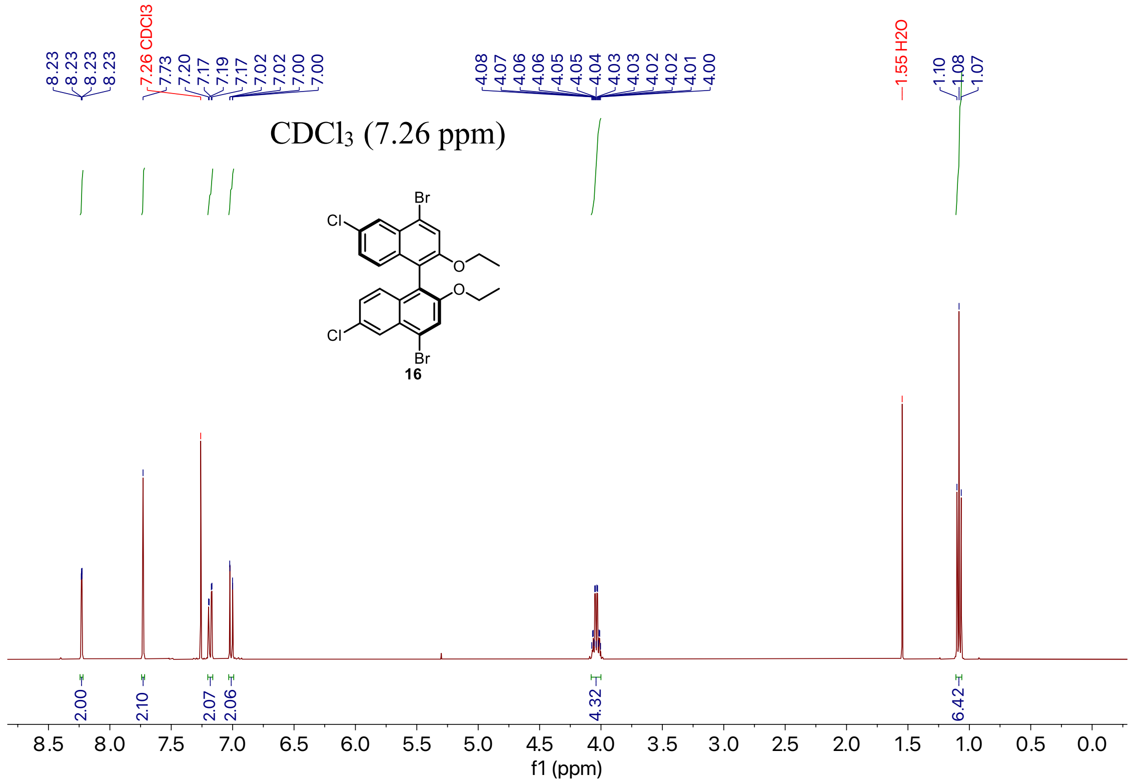
8.23
8.23
8.23
8.23
7.26 CDCl3
7.73
7.20
7.17
7.19
7.17
7.02
7.02
7.00
7.00
4.08
4.07
4.06
4.06
4.05
4.05
4.04
4.03
4.02
4.02
4.01
4.00
1.55 H2O
1.10
1.08
1.07
CDCl3 (7.26 ppm)
Br
Cl
O
O
Cl
Br
16
2.00
2.10
2.07
2.06
4.32
6.42
8.5 8.0 7.5 7.0 6.5 6.0 5.5 5.0 4.5 4.0 3.5 3.0 2.5 2.0 1.5 1.0 0.5 0.0
f1 (ppm)

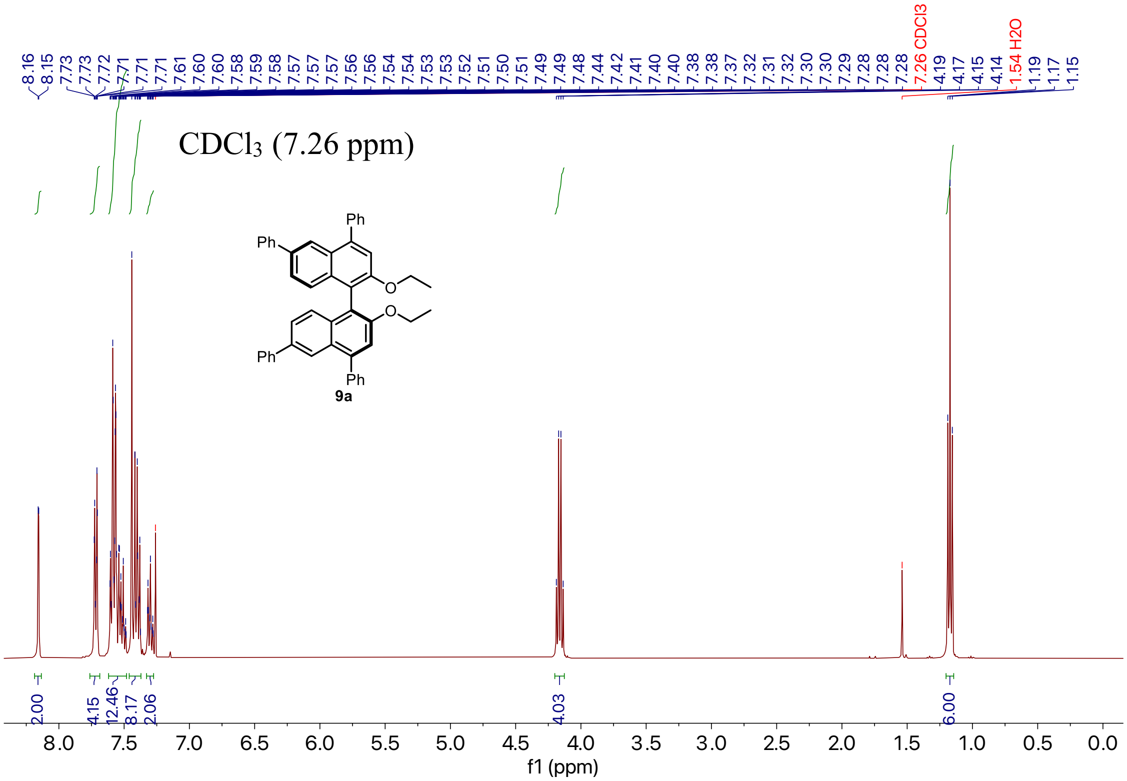
8.16
8.15
7.73
7.73
7.72
7.71
7.71
7.71
7.61
7.60
7.60
7.58
7.59
7.58
7.57
7.57
7.57
7.56
7.56
7.54
7.54
7.53
7.53
7.52
7.51
7.50
7.51
7.49
7.49
7.48
7.44
7.42
7.41
7.40
7.40
7.38
7.38
7.37
7.32
7.31
7.32
7.30
7.30
7.29
7.28
7.28
7.28
7.26 CDCl3
4.19
4.17
4.15
4.14
1.54 H2O
1.19
1.17
1.15
CDCl3 (7.26 ppm)
Ph
Ph
O
O
Ph
Ph
9a
2.00
4.15
12.46
8.17
2.06
4.03
6.00
f1 (ppm)

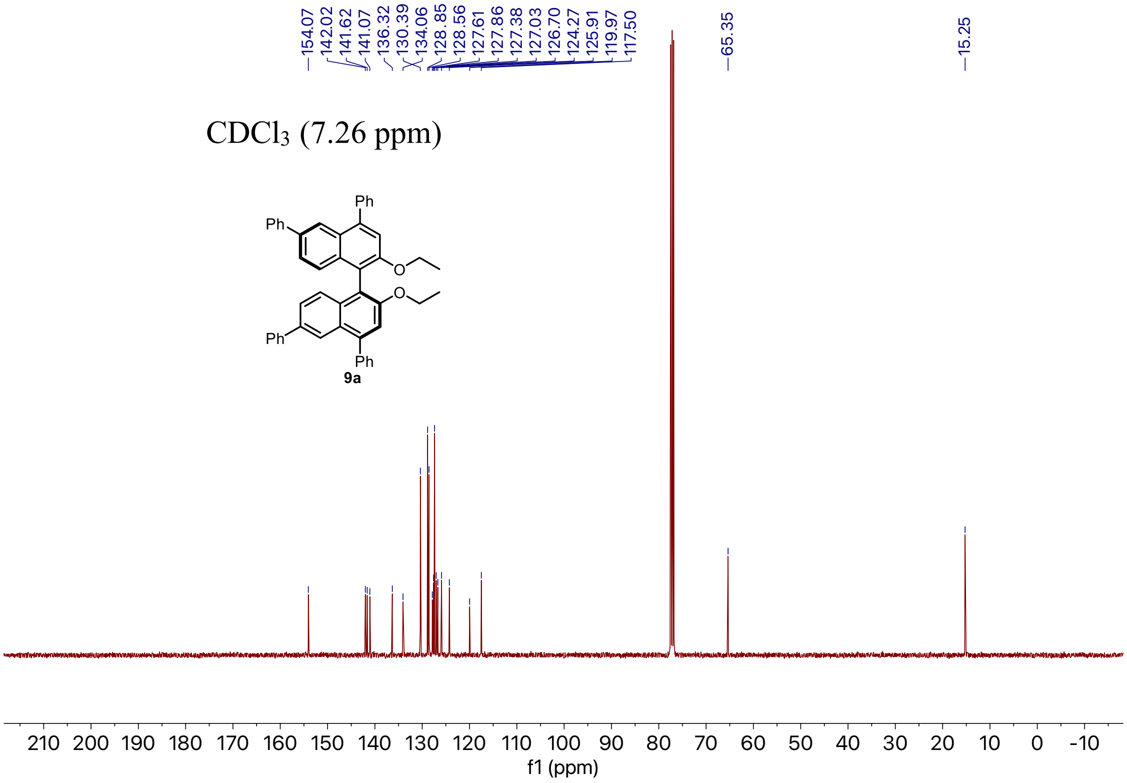
154.07
142.02
141.62
141.07
136.32
130.39
134.06
128.85
128.56
127.61
127.86
127.38
127.03
126.70
124.27
125.91
119.97
117.50
65.35
15.25
CDCl3 (7.26 ppm)
Ph
Ph
O
O
Ph
Ph
9a
f1 (ppm)

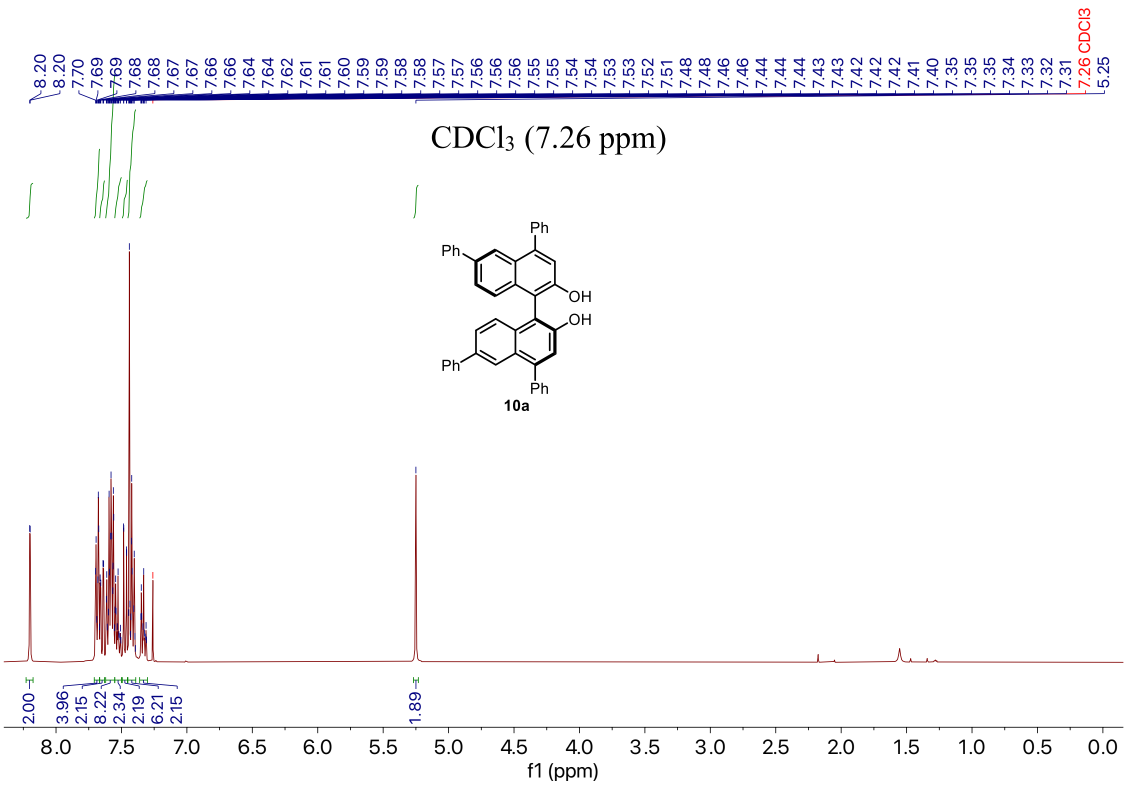

CDCl3 (7.26 ppm)
10a
f1 (ppm)

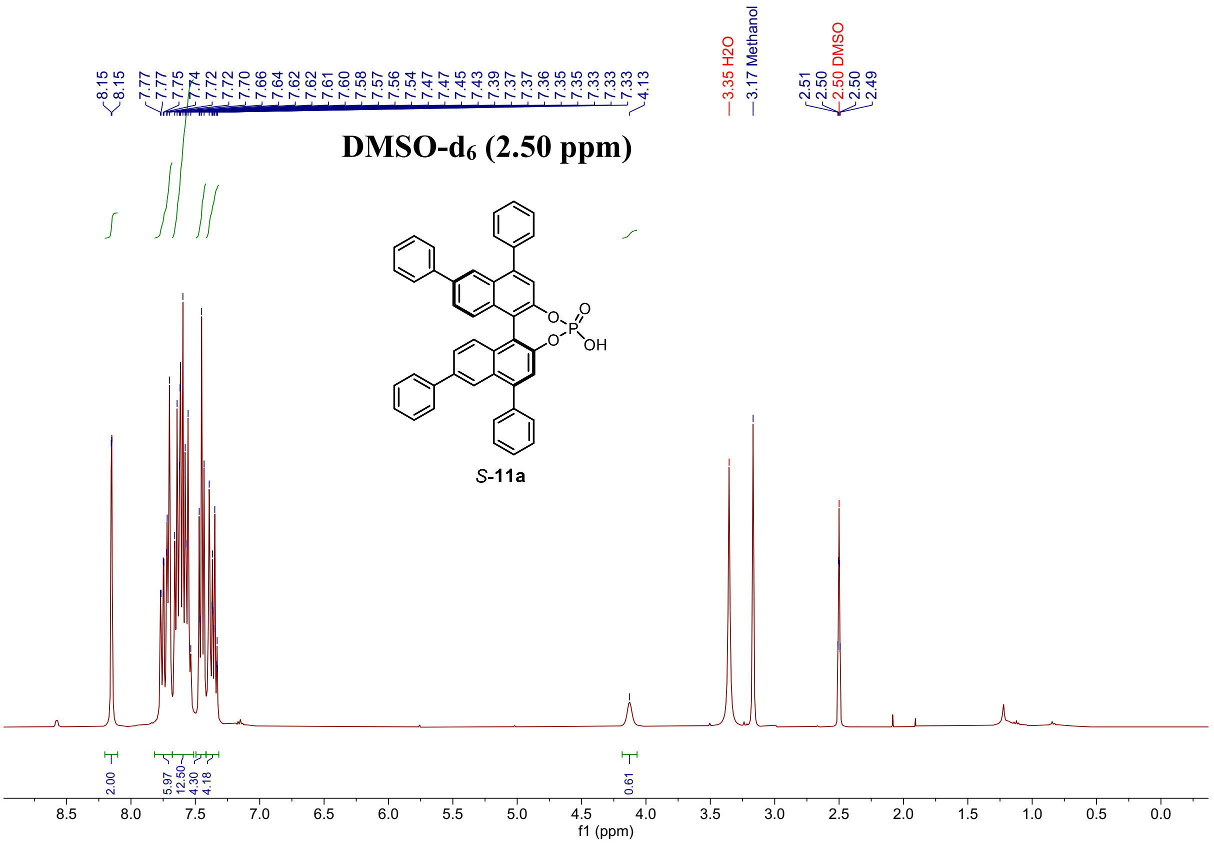

DMSO-d6 (2.50 ppm)
S-11a
f1 (ppm)

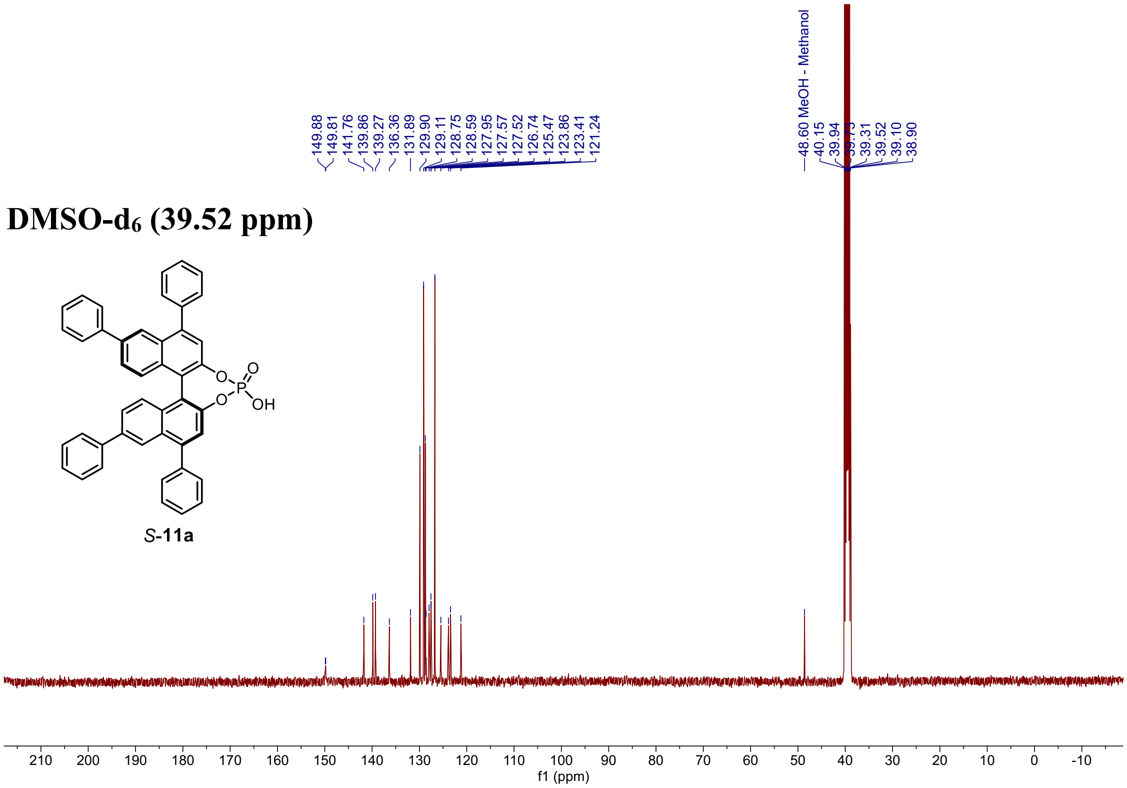

149.88
149.81
141.76
139.86
139.27
136.36
131.89
129.90
129.11
128.75
128.59
127.95
127.57
127.52
126.74
125.47
123.86
123.41
121.24
48.60 MeOH - Methanol
40.15
39.94
39.52
39.31
39.10
38.90
DMSO-d6 (39.52 ppm)
O
O
P
O
OH
S-11a
f1 (ppm)

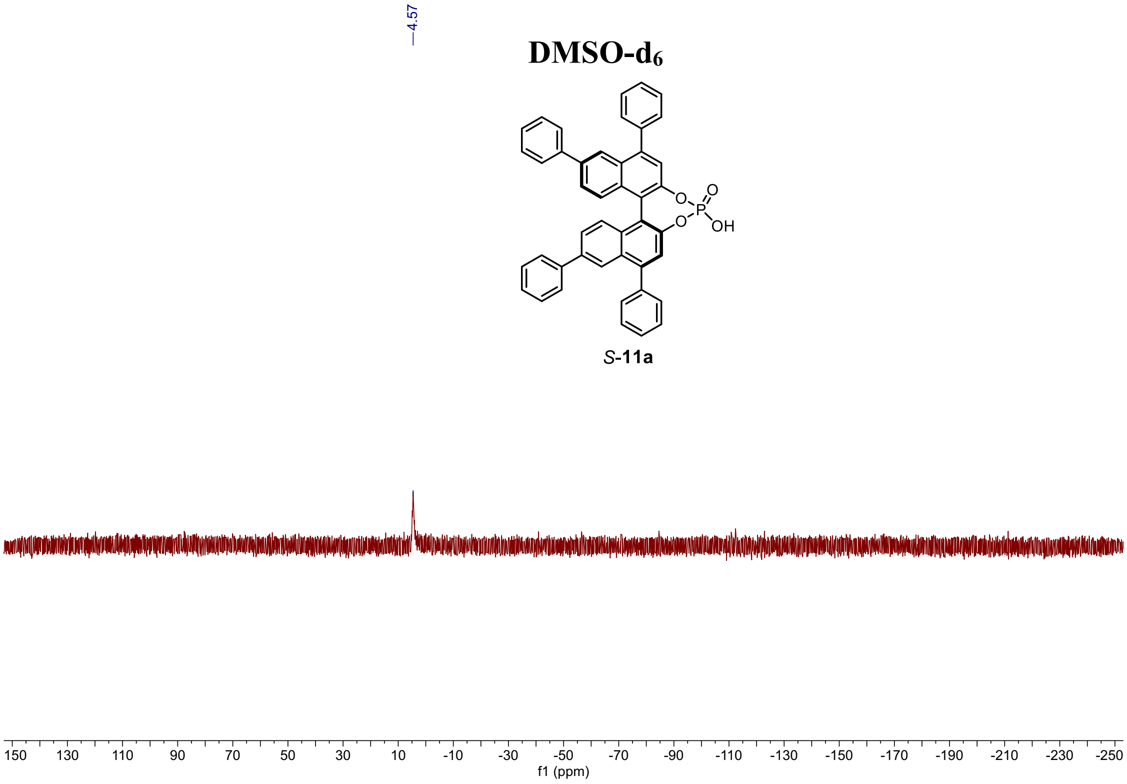

4.57
DMSO-d6
O
O
P
O
OH
S-11a
f1 (ppm)

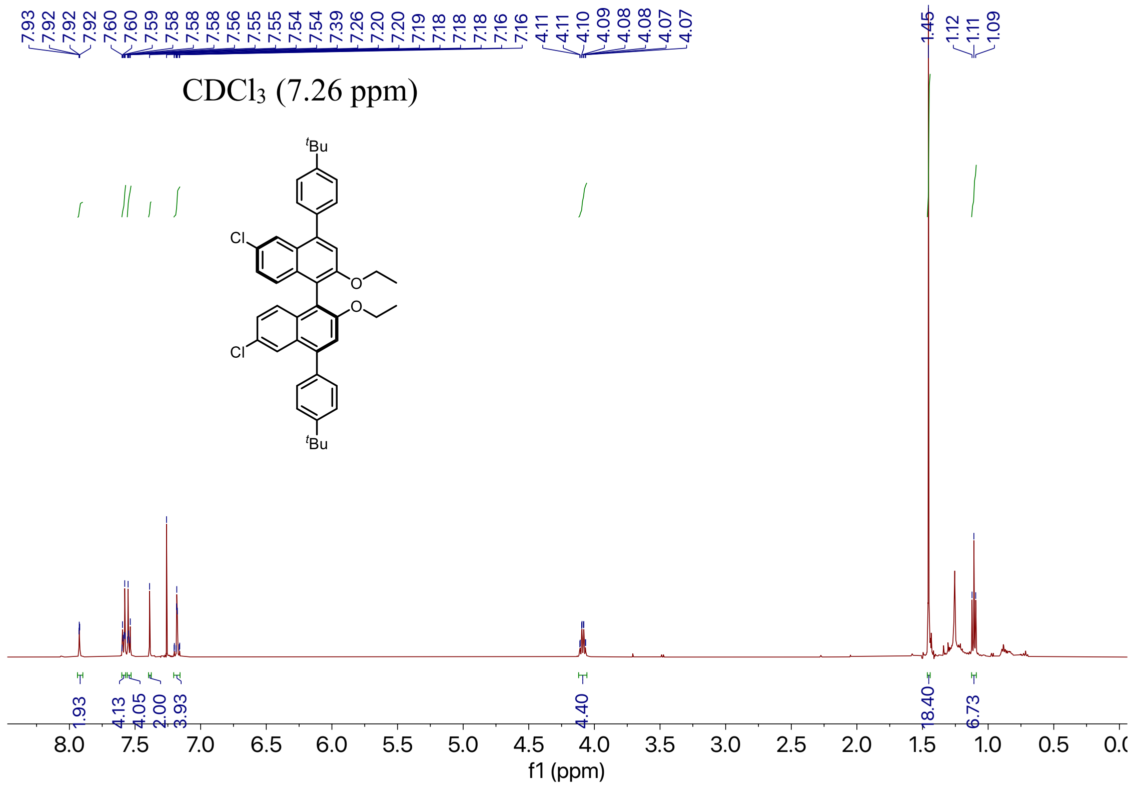
7.93 7.92 7.92 7.92 7.60 7.60 7.59 7.58 7.58 7.58 7.56 7.55 7.55 7.54 7.54 7.39 7.26 7.20 7.20 7.19 7.18 7.18 7.18 7.16 4.11 4.11 4.10 4.09 4.08 4.08 4.07 4.07
1.45 1.12 1.11 1.09
CDCl3 (7.26 ppm)
tBu
Cl
O
O
Cl
tBu
1.93 4.13 4.05 2.00 3.93 4.40 18.40 6.73
8.0 7.5 7.0 6.5 6.0 5.5 5.0 4.5 4.0 3.5 3.0 2.5 2.0 1.5 1.0 0.5
f1 (ppm)

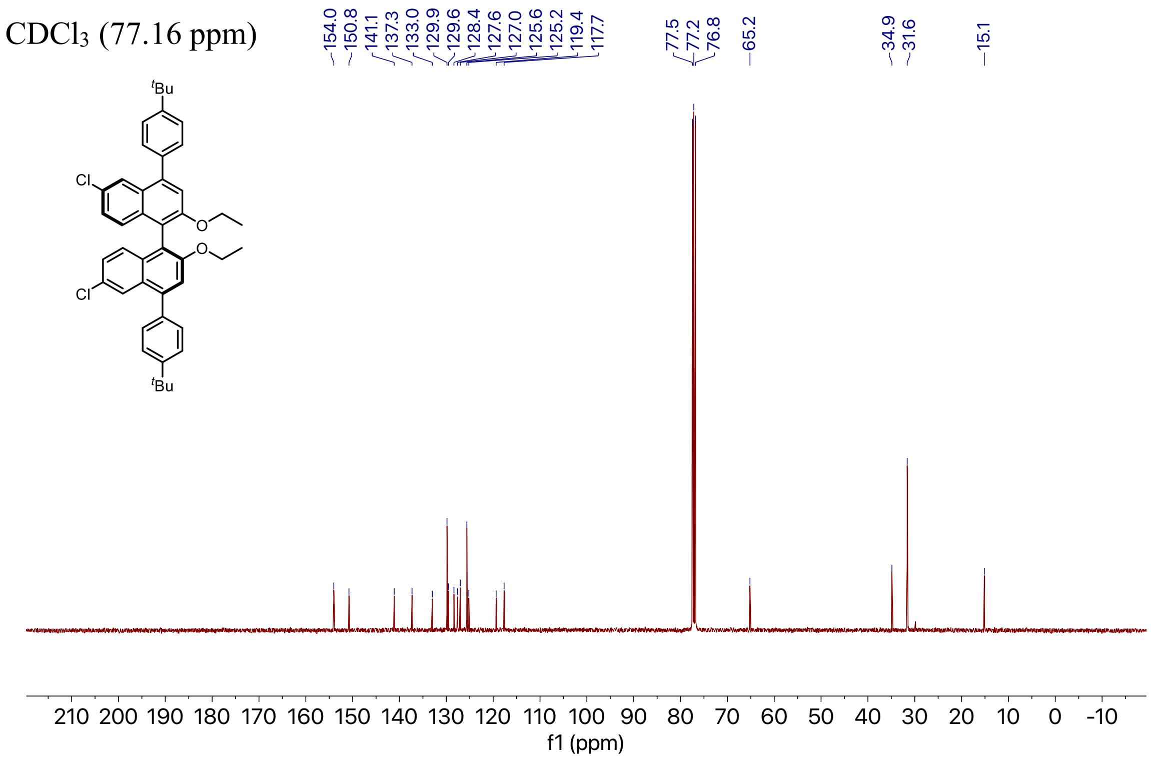
CDCl3 (77.16 ppm)
154.0 150.8 141.1 137.3 133.0 129.9 129.6 128.4 127.6 127.0 125.6 125.2 119.4 117.7
77.5 77.2 76.8 65.2 34.9 31.6 15.1
tBu
Cl
O
O
Cl
tBu
210 200 190 180 170 160 150 140 130 120 110 100 90 80 70 60 50 40 30 20 10 0 -10
f1 (ppm)

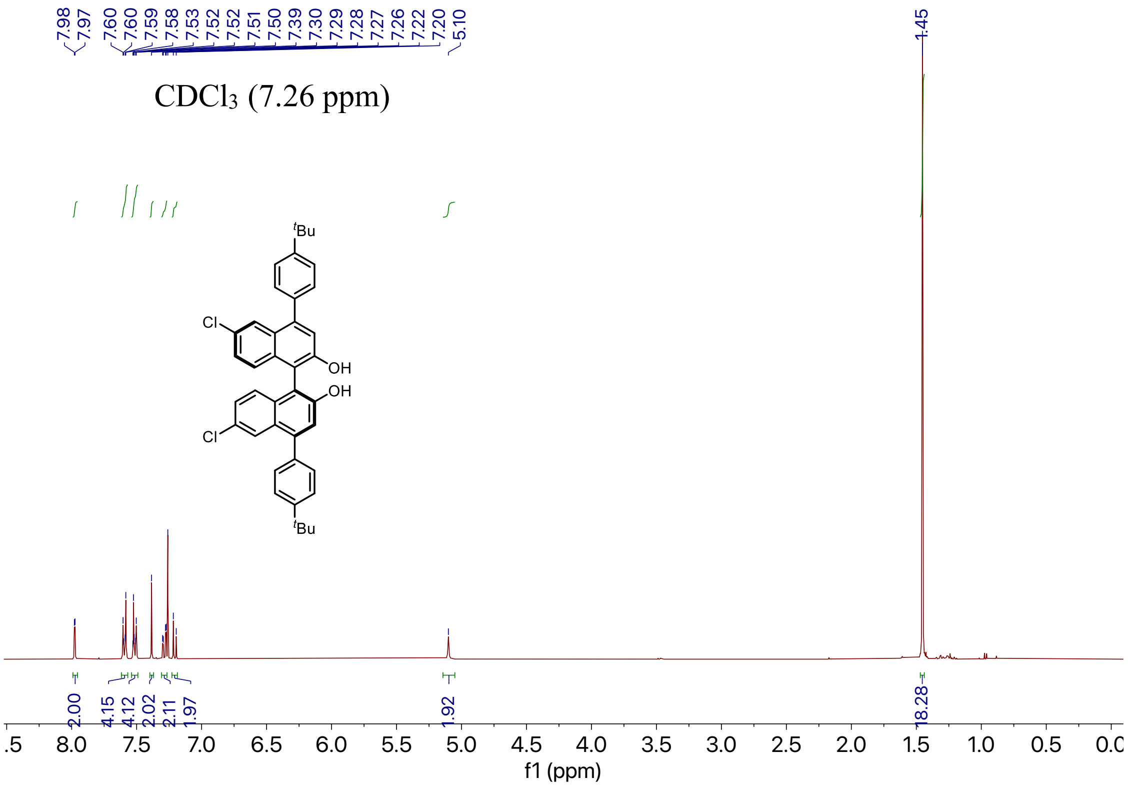
7.98
7.97
7.60
7.60
7.59
7.58
7.53
7.52
7.52
7.51
7.50
7.39
7.30
7.29
7.28
7.27
7.26
7.22
7.20
5.10
1.45
CDCl3 (7.26 ppm)
tBu
Cl
OH
OH
Cl
tBu
2.00
4.15
4.12
2.02
2.11
1.97
1.92
18.28
f1 (ppm)

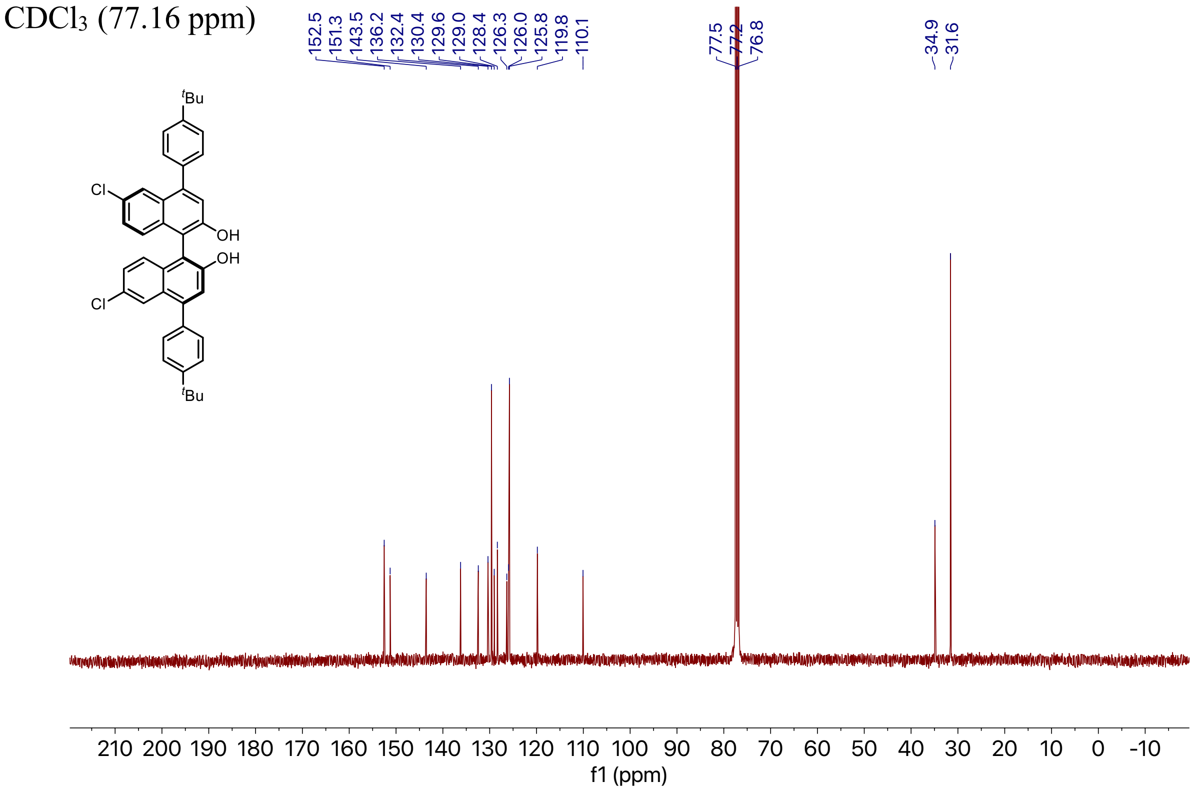
CDCl3 (77.16 ppm)
152.5
151.3
143.5
136.2
132.4
130.4
129.6
129.0
128.4
126.3
126.0
125.8
119.8
110.1
77.5
77.2
76.8
34.9
31.6
tBu
Cl
OH
OH
Cl
tBu
210 200 190 180 170 160 150 140 130 120 110 100 90 80 70 60 50 40 30 20 10 0 -10
f1 (ppm)

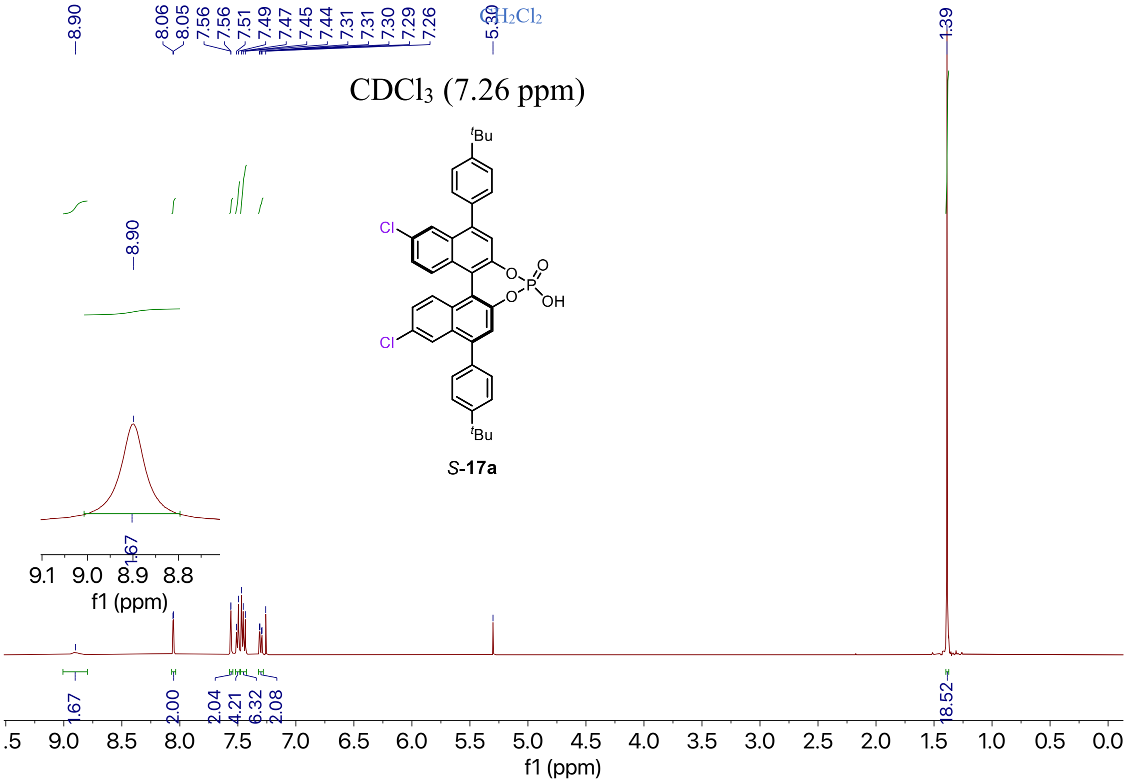
8.90
8.06
8.05
7.56
7.56
7.51
7.49
7.47
7.45
7.44
7.31
7.31
7.30
7.29
7.26
5.30
CH2Cl2
1.39
CDCl3 (7.26 ppm)
tBu
Cl
O
O
P
O
OH
Cl
tBu
S-17a
1.67
9.1 9.0 8.9 8.8
f1 (ppm)
1.67
2.00
2.04
4.21
6.32
2.08
18.52
.5 9.0 8.5 8.0 7.5 7.0 6.5 6.0 5.5 5.0 4.5 4.0 3.5 3.0 2.5 2.0 1.5 1.0 0.5 0.0
f1 (ppm)

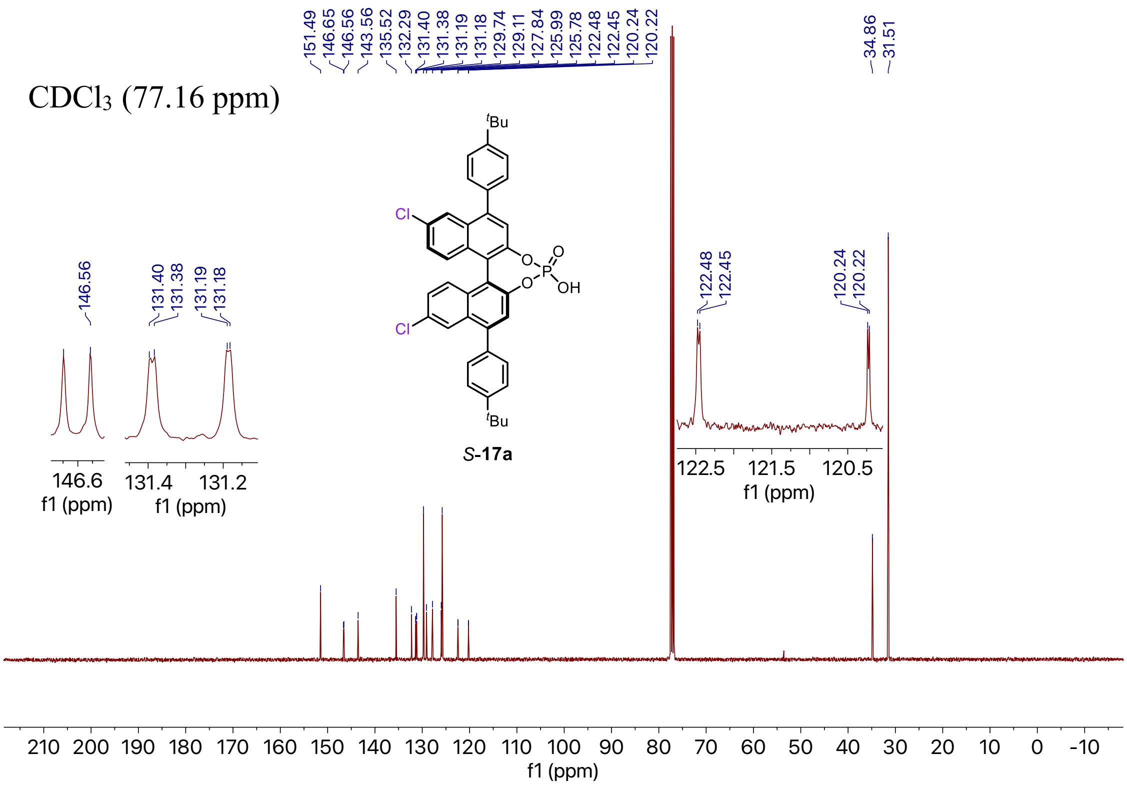

CDCl3 (77.16 ppm)
151.49
146.65
146.56
143.56
135.52
132.29
131.40
131.38
131.19
131.18
129.74
129.11
127.84
125.99
125.78
122.48
122.45
120.24
120.22
34.86
31.51
tBu
Cl
O
P
OH
S-17a
f1 (ppm)

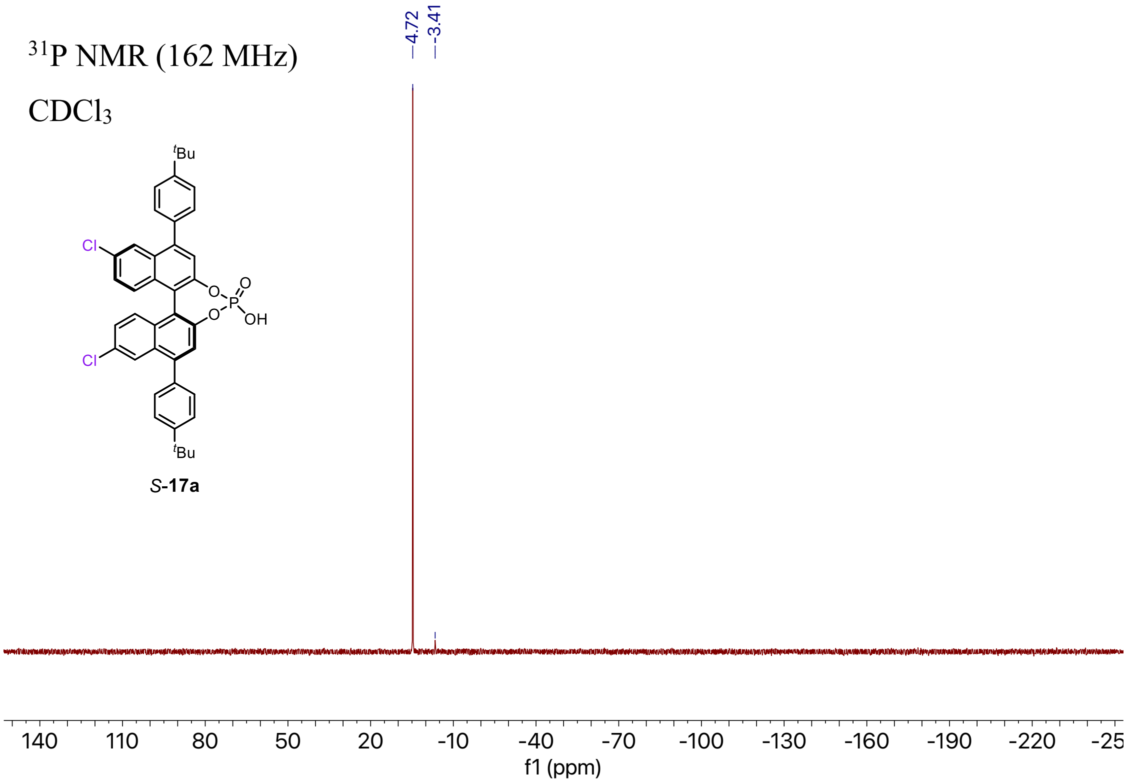

31P NMR (162 MHz)
CDCl3
4.72
-3.41
tBu
Cl
O
P
OH
S-17a
f1 (ppm)

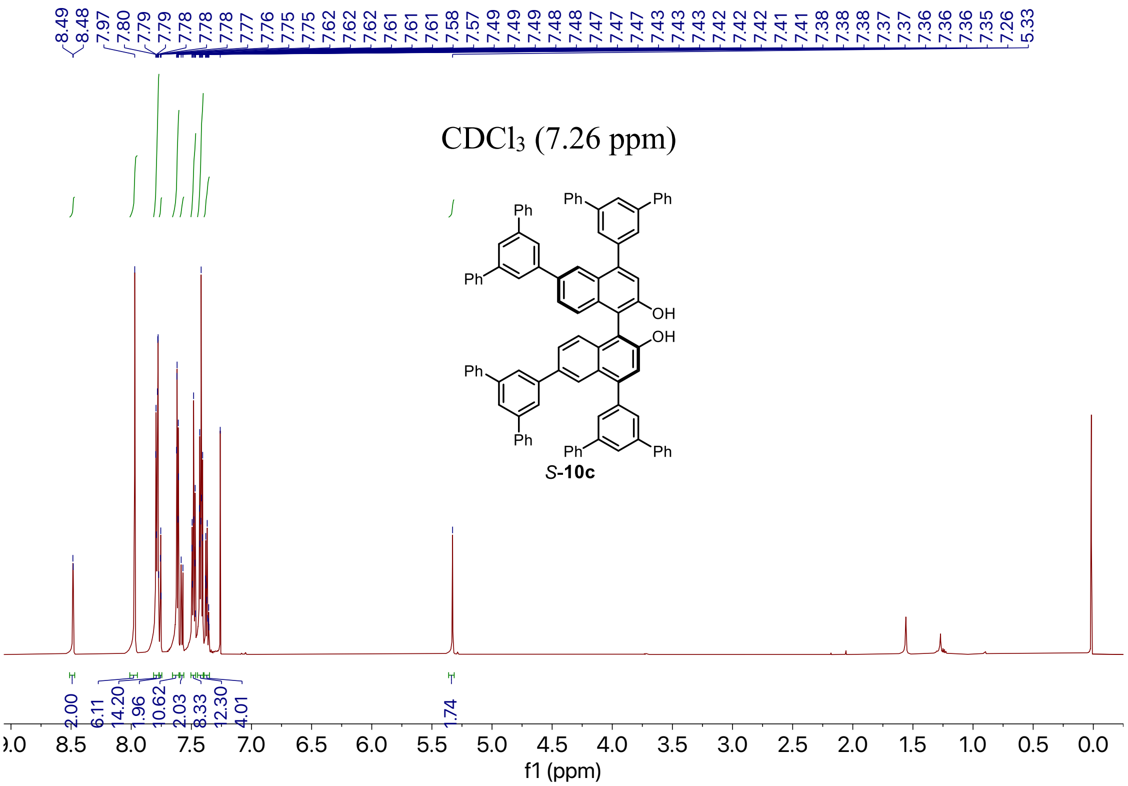
CDCl3 (7.26 ppm)
S-10c
f1 (ppm)

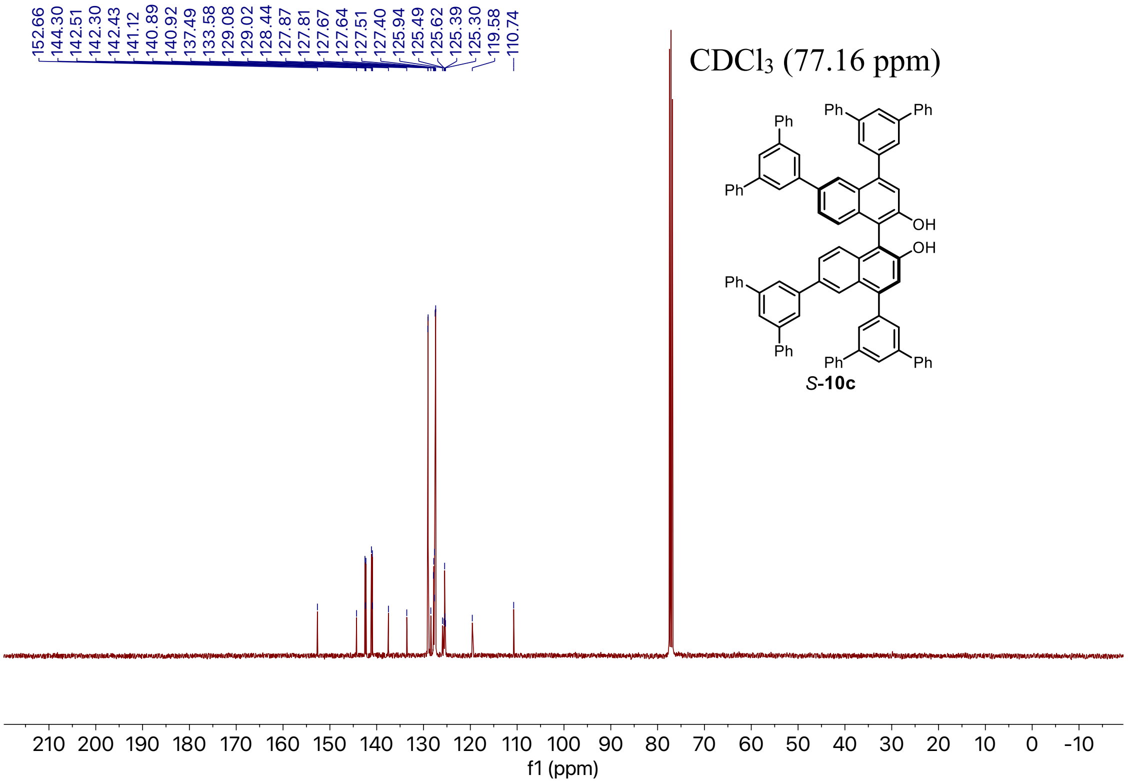
CDCl3 (77.16 ppm)
S-10c
f1 (ppm)

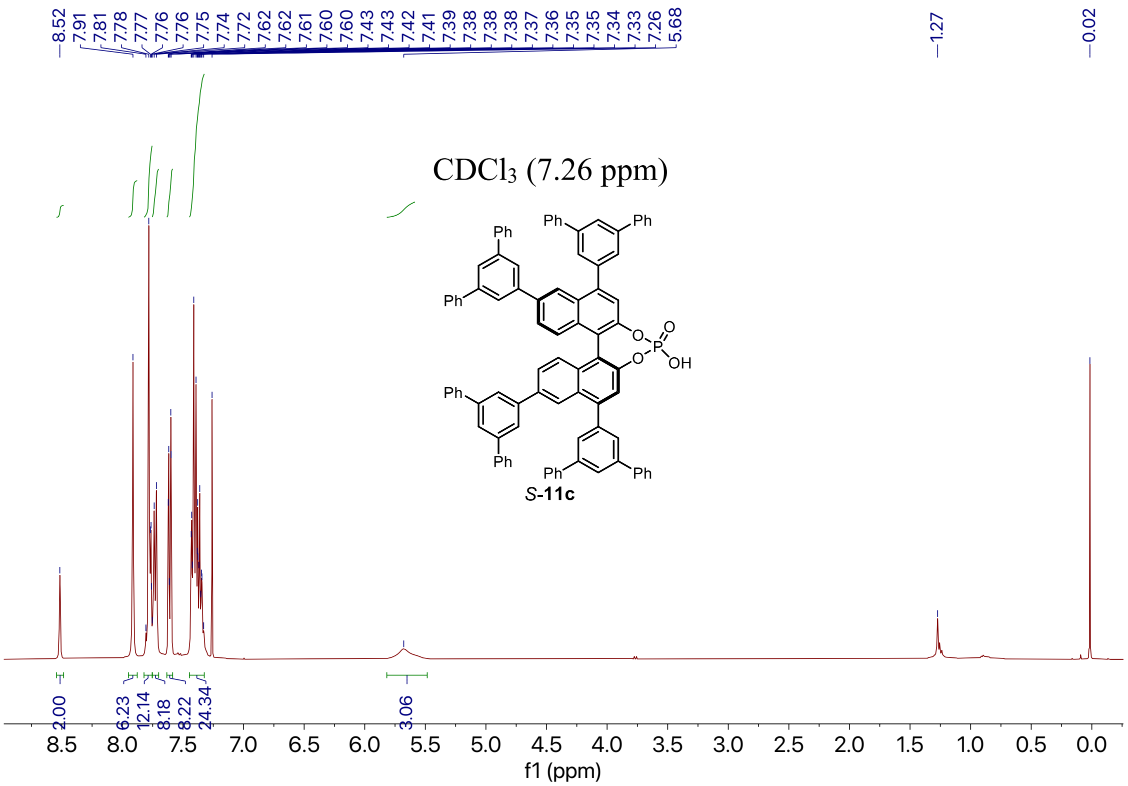
8.52
7.91
7.81
7.78
7.77
7.76
7.76
7.75
7.74
7.72
7.62
7.62
7.61
7.60
7.60
7.43
7.43
7.42
7.41
7.39
7.38
7.38
7.38
7.37
7.36
7.35
7.35
7.34
7.33
7.26
5.68
1.27
0.02
CDCl3 (7.26 ppm)
Ph
Ph
Ph
Ph
O
O
P
O
OH
Ph
Ph
Ph
Ph
S-11c
2.00
6.23
12.14
8.18
8.22
24.34
3.06
8.5 8.0 7.5 7.0 6.5 6.0 5.5 5.0 4.5 4.0 3.5 3.0 2.5 2.0 1.5 1.0 0.5 0.0
f1 (ppm)

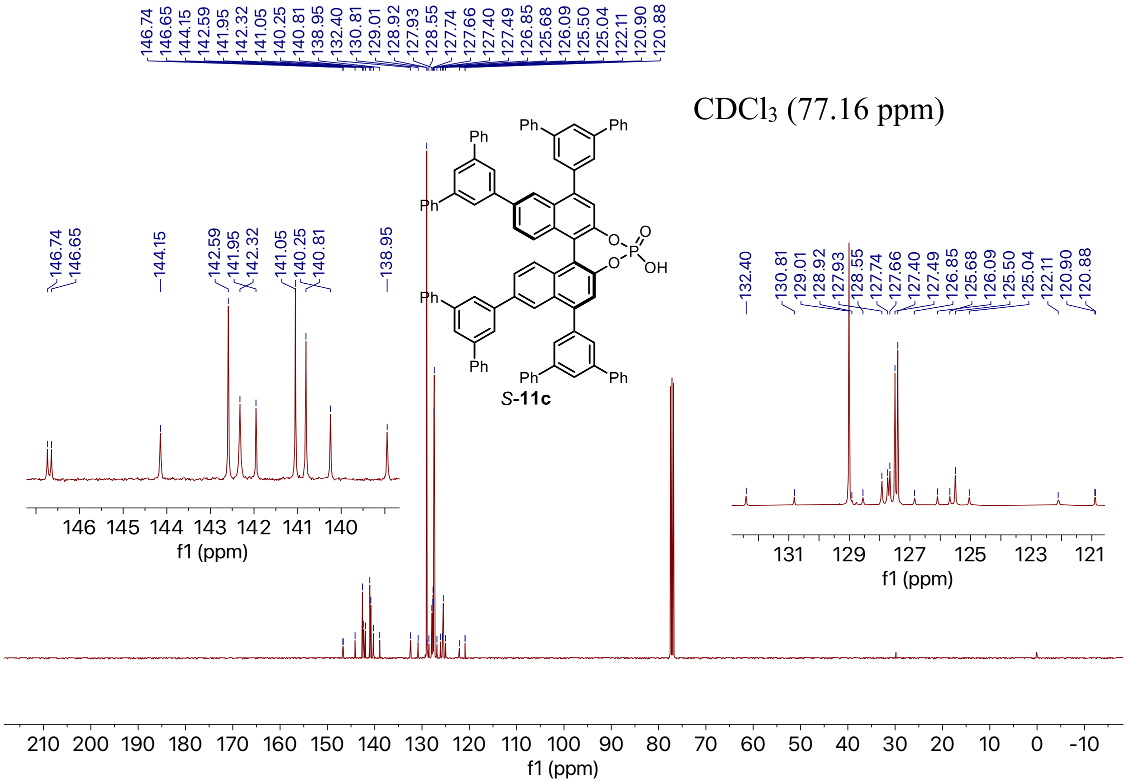
146.74
146.65
144.15
142.59
141.95
142.32
141.05
140.25
140.81
138.95
132.40
130.81
129.01
128.92
127.93
128.55
127.74
127.66
127.40
127.49
126.85
125.68
126.09
125.50
125.04
122.11
120.90
120.88
CDCl3 (77.16 ppm)
S-11c
146 145 144 143 142 141 140
f1 (ppm)
131 129 127 125 123 121
f1 (ppm)
210 200 190 180 170 160 150 140 130 120 110 100 90 80 70 60 50 40 30 20 10 0 -10
f1 (ppm)

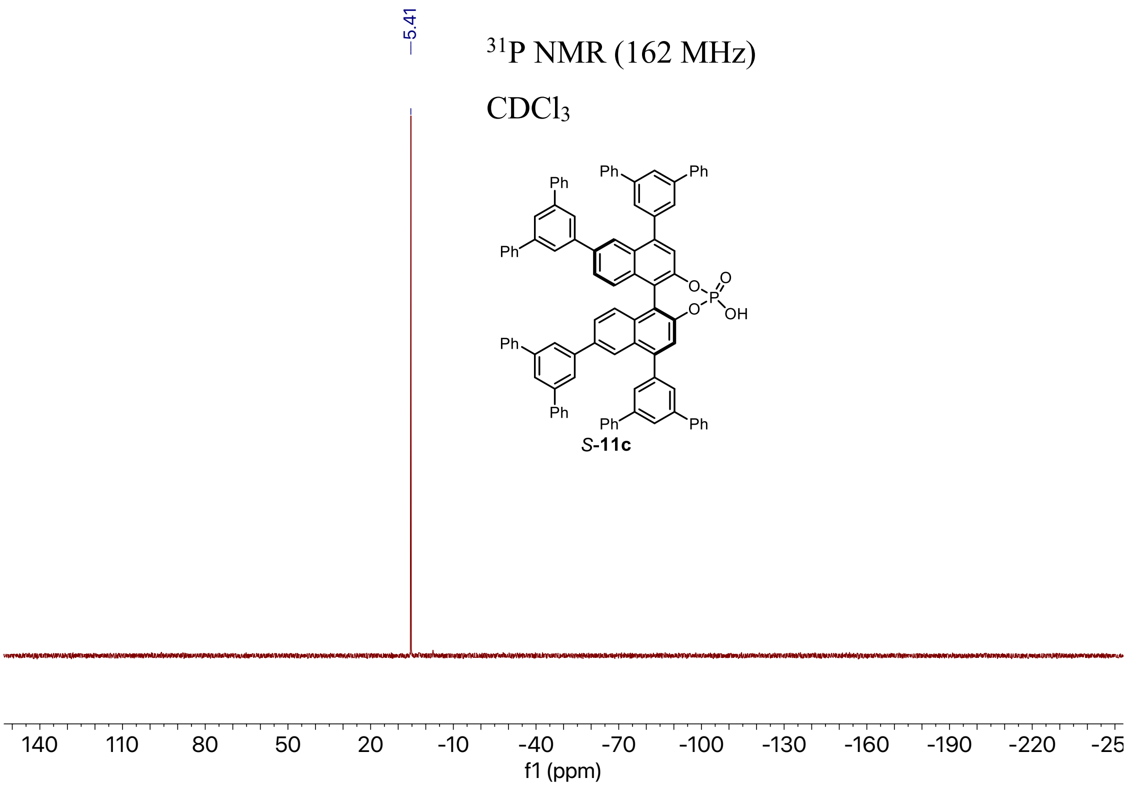

—5.41
^{31}P NMR (162 MHz)
$CDCl_3$
Ph
Ph
Ph
Ph
O
O
P
O
OH
Ph
Ph
Ph
Ph
S-**11c**
140 110 80 50 20 -10 -40 -70 -100 -130 -160 -190 -220 -25
f1 (ppm)

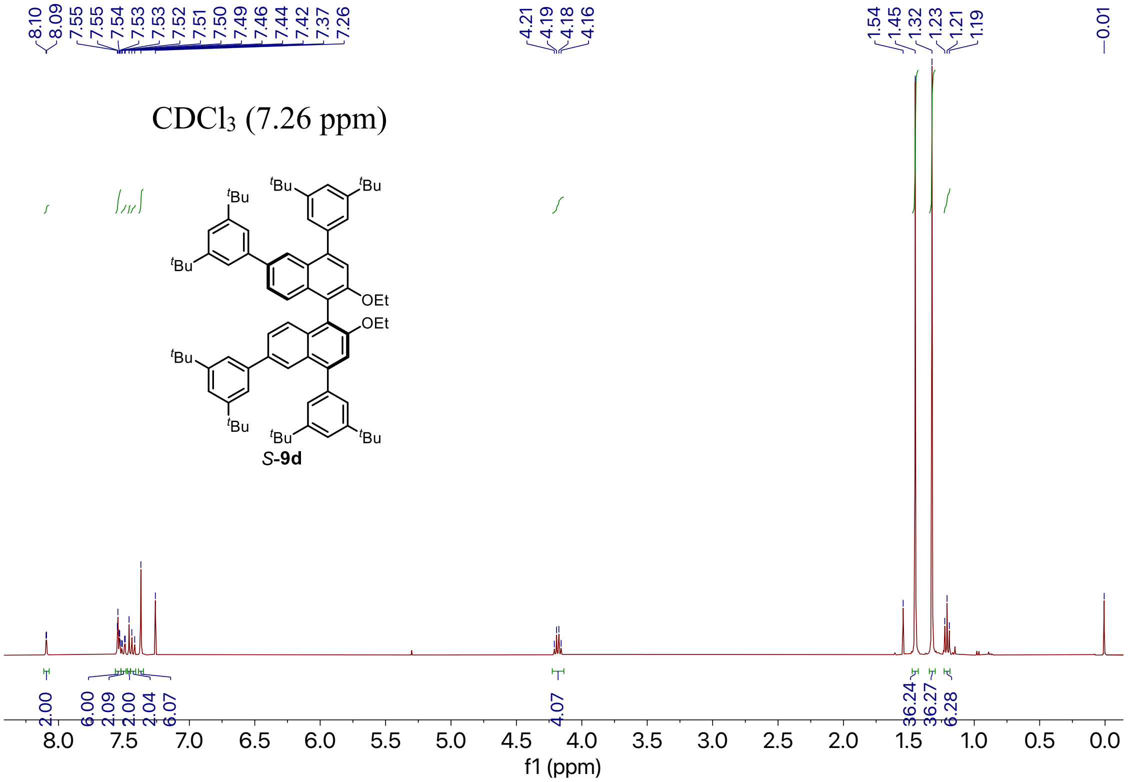
8.10
8.09
7.55
7.55
7.54
7.53
7.53
7.52
7.51
7.50
7.49
7.46
7.44
7.42
7.37
7.26
4.21
4.19
4.18
4.16
1.54
1.45
1.32
1.23
1.21
1.19
-0.01
CDCl3 (7.26 ppm)
S-9d
2.00
6.00
2.09
2.00
2.04
6.07
4.07
36.24
36.27
6.28
8.0 7.5 7.0 6.5 6.0 5.5 5.0 4.5 4.0 3.5 3.0 2.5 2.0 1.5 1.0 0.5 0.0
f1 (ppm)

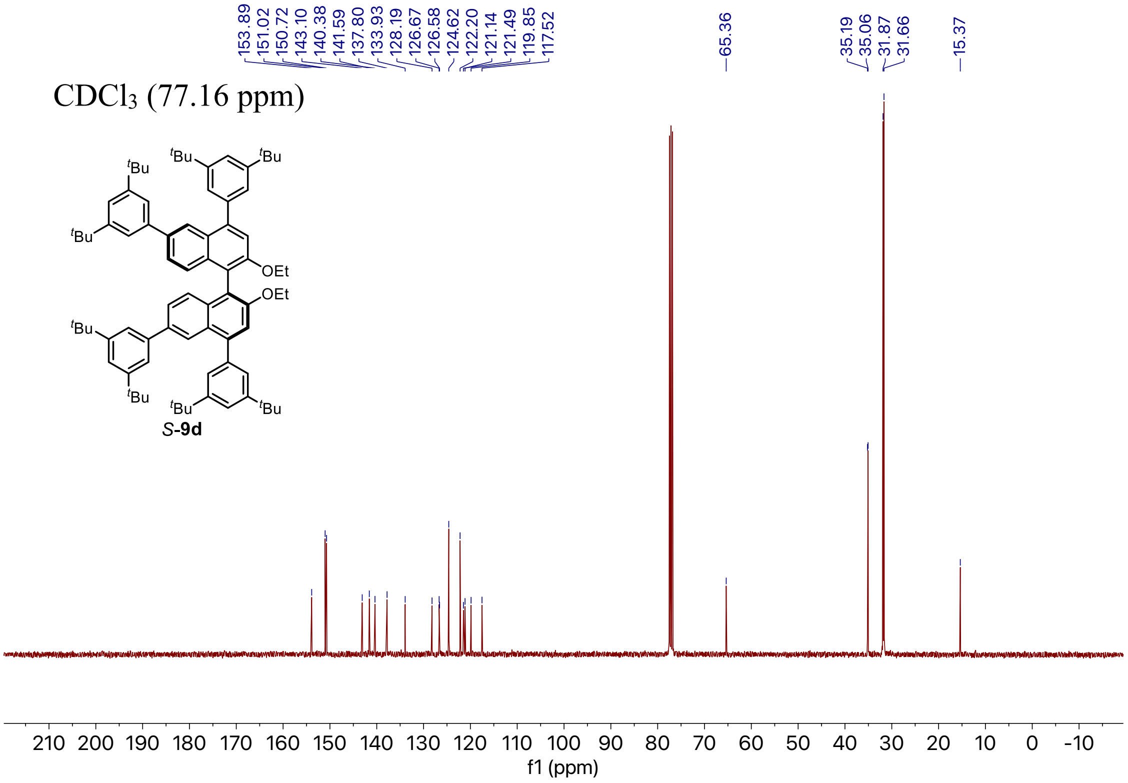
153.89
151.02
150.72
143.10
140.38
141.59
137.80
133.93
128.19
126.67
126.58
124.62
122.20
121.14
121.49
119.85
117.52
65.36
35.19
35.06
31.87
31.66
15.37
CDCl3 (77.16 ppm)
S-9d
210 200 190 180 170 160 150 140 130 120 110 100 90 80 70 60 50 40 30 20 10 0 -10
f1 (ppm)

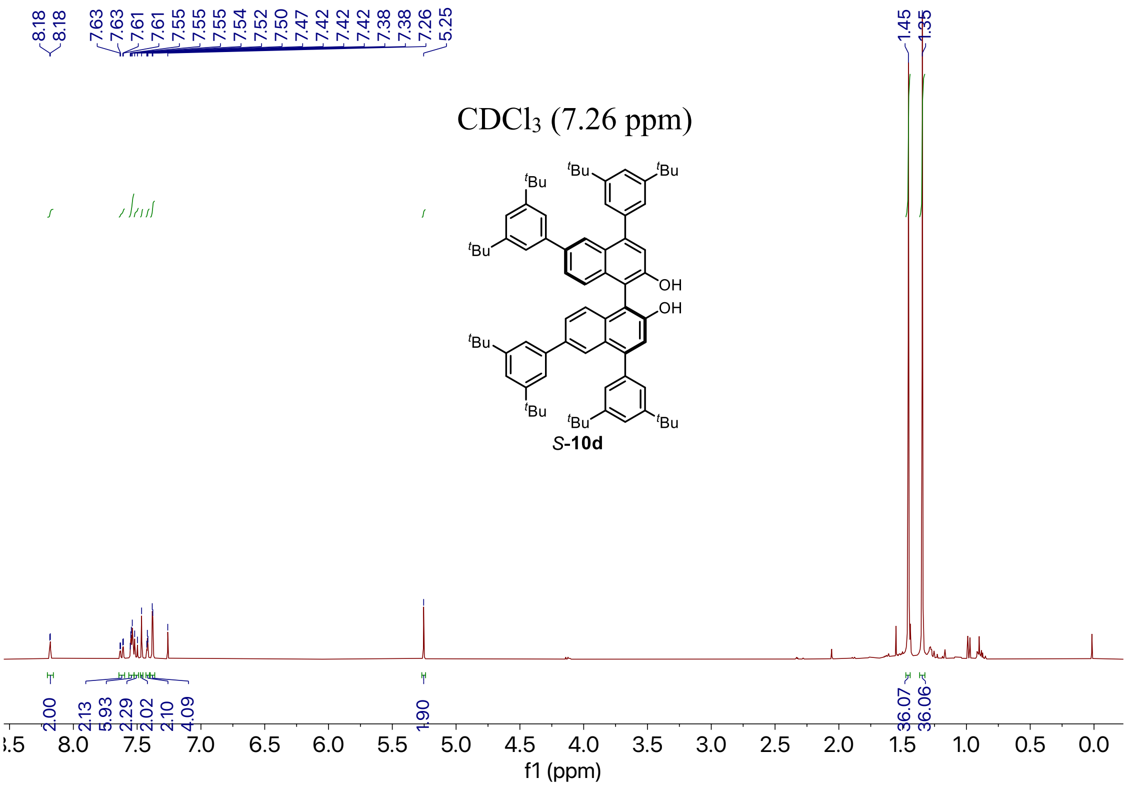
8.18
8.18
7.63
7.63
7.61
7.61
7.55
7.55
7.55
7.54
7.52
7.50
7.47
7.42
7.42
7.42
7.38
7.38
7.26
5.25
1.45
1.35
CDCl3 (7.26 ppm)
S-10d
OH
OH
2.00
2.13
5.93
2.29
2.02
2.10
4.09
1.90
36.07
36.06
f1 (ppm)

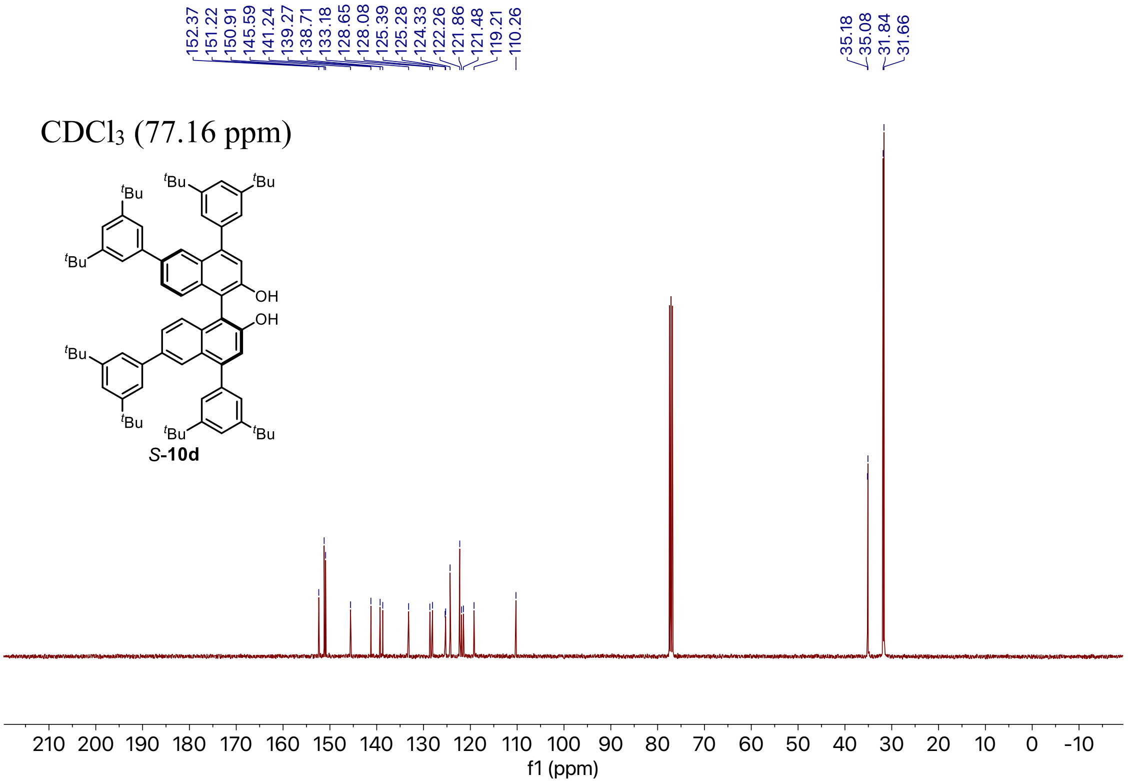
152.37
151.22
150.91
145.59
141.24
139.27
138.71
133.18
128.65
128.08
125.39
125.28
124.33
122.26
121.86
121.48
119.21
110.26
35.18
35.08
31.84
31.66
CDCl3 (77.16 ppm)
S-10d
OH
OH
f1 (ppm)

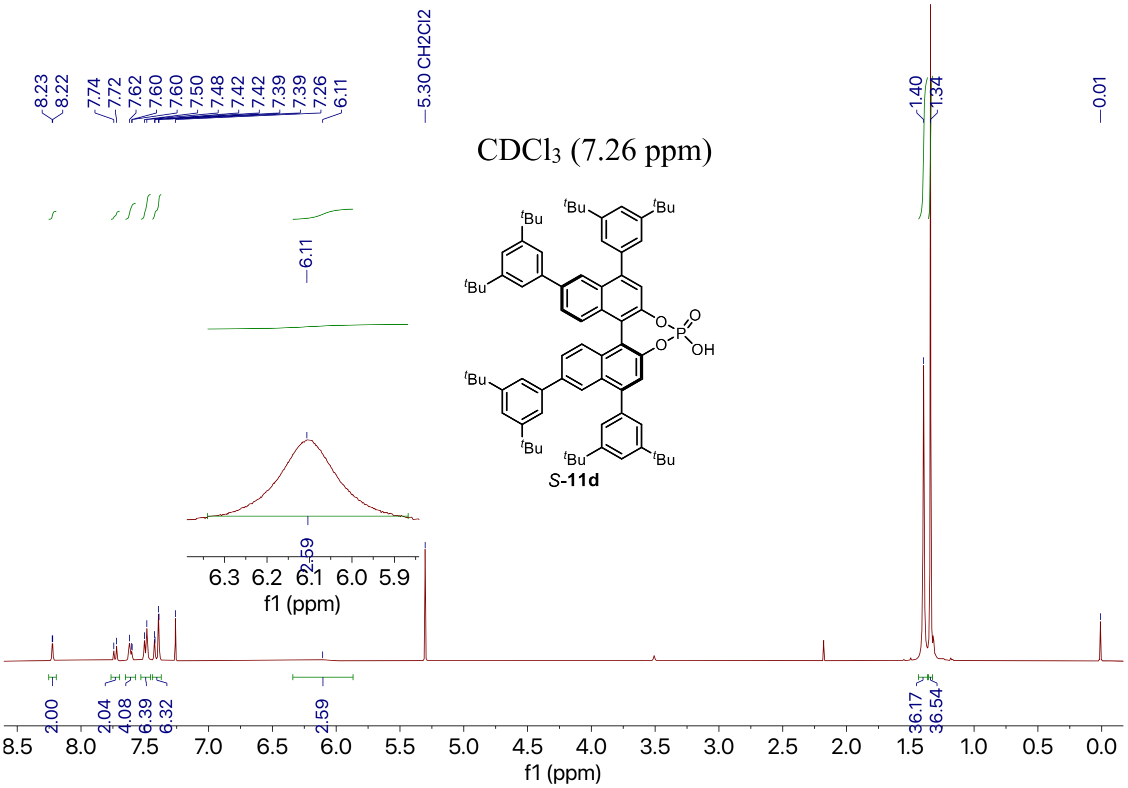
CDCl3 (7.26 ppm)
8.23
8.22
7.74
7.72
7.62
7.60
7.60
7.50
7.48
7.42
7.42
7.39
7.39
7.26
6.11
5.30 CH2Cl2
1.40
1.34
-0.01
6.11
2.59
6.3 6.2 6.1 6.0 5.9
f1 (ppm)
2.00
2.04
4.08
6.39
6.32
2.59
36.17
36.54
8.5 8.0 7.5 7.0 6.5 6.0 5.5 5.0 4.5 4.0 3.5 3.0 2.5 2.0 1.5 1.0 0.5 0.0
f1 (ppm)
tBu
OH
S-11d

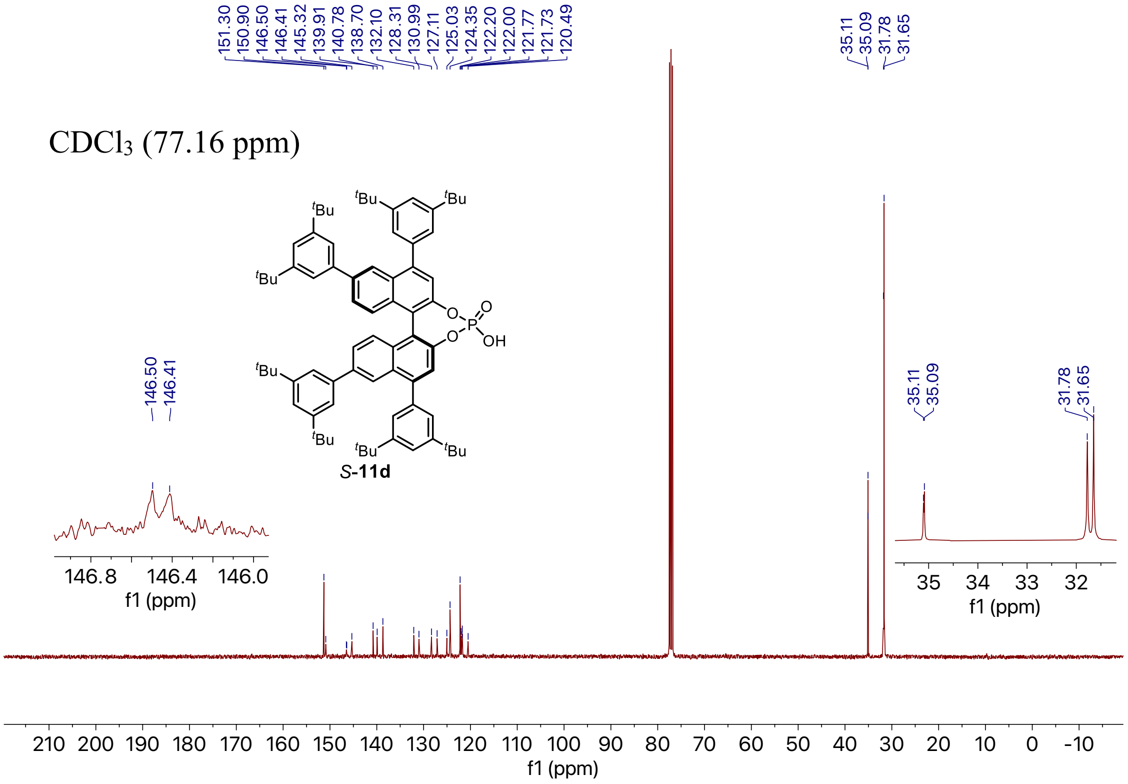
151.30
150.90
146.50
146.41
145.32
139.91
140.78
138.70
132.10
128.31
130.99
127.11
125.03
124.35
122.20
122.00
121.77
121.73
120.49
35.11
35.09
31.78
31.65
CDCl3 (77.16 ppm)
146.8 146.4 146.0
f1 (ppm)
35 34 33 32
f1 (ppm)
210 200 190 180 170 160 150 140 130 120 110 100 90 80 70 60 50 40 30 20 10 0 -10
f1 (ppm)
tBu
OH
S-11d

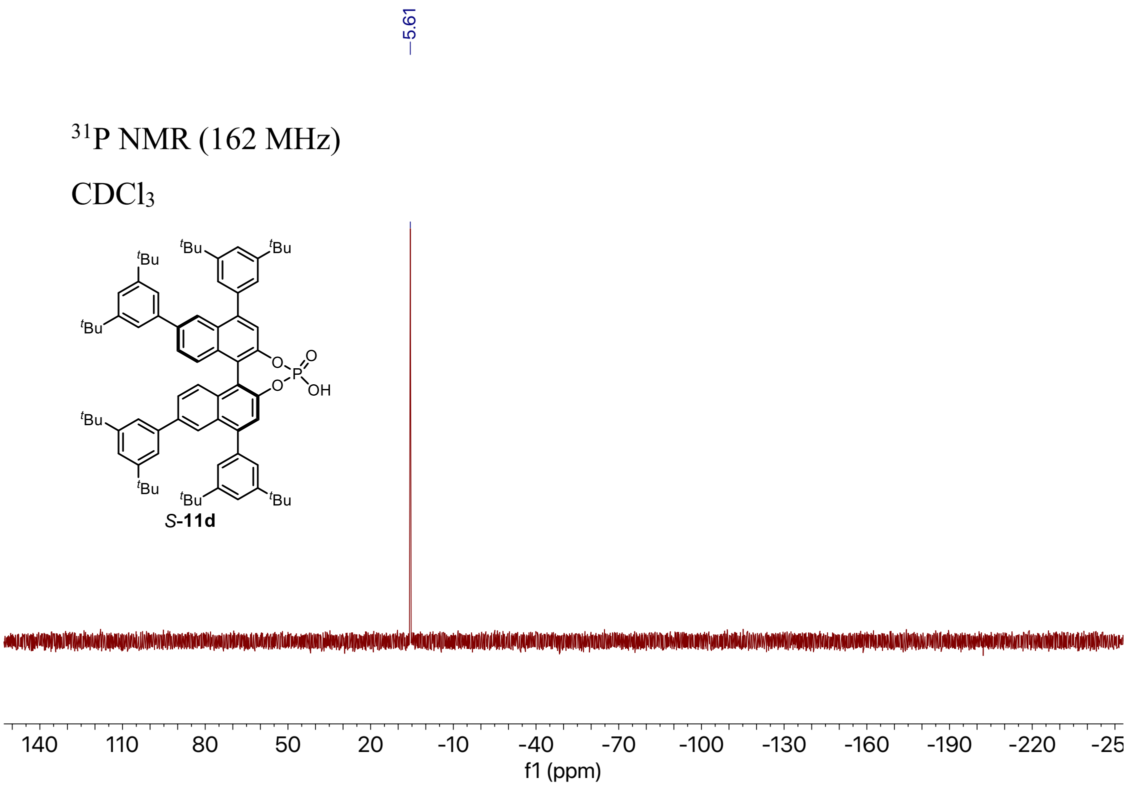
5.61
^{31}P NMR (162 MHz)
$CDCl_3$
tBu
O
P
OH
S-11d
140 110 80 50 20 -10 -40 -70 -100 -130 -160 -190 -220 -25
f1 (ppm)

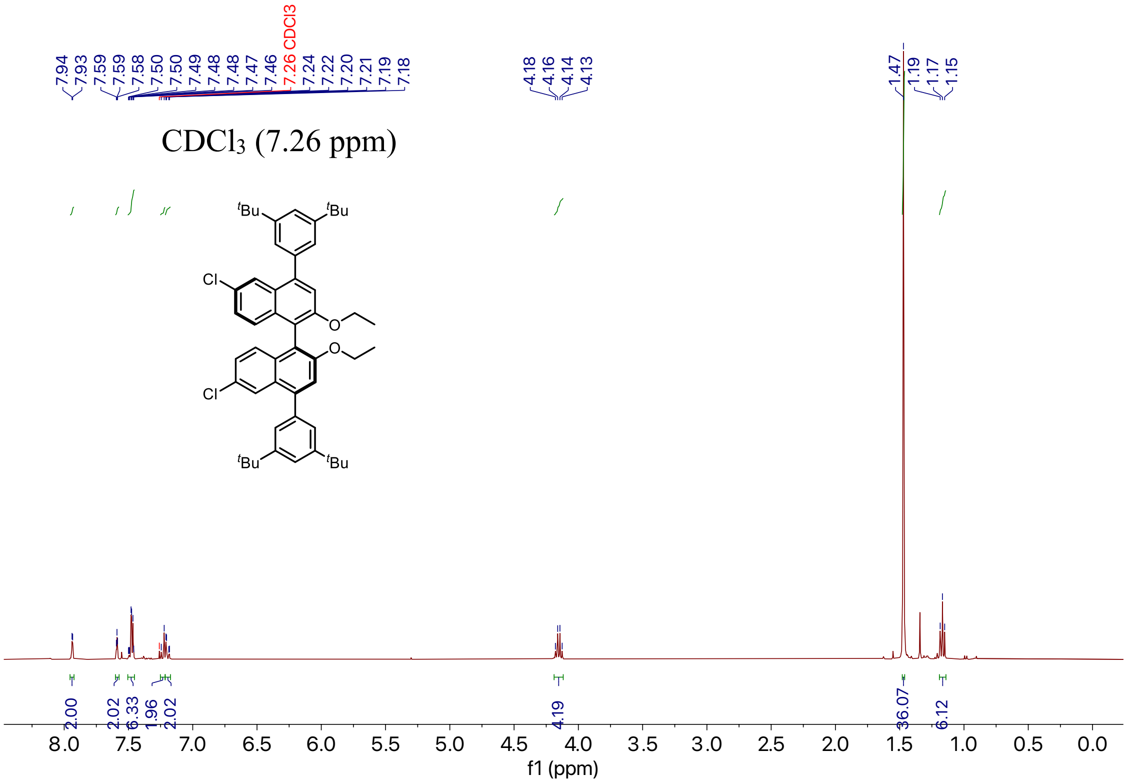
7.94 7.93 7.59 7.59 7.58 7.50 7.50 7.49 7.48 7.48 7.47 7.46 7.26 CDCl3 7.24 7.22 7.20 7.21 7.19 7.18
4.18 4.16 4.14 4.13
1.47 1.19 1.17 1.15
$CDCl_3$ (7.26 ppm)
tBu
Cl
O
2.00 2.02 6.33 1.96 2.02 4.19 36.07 6.12
8.0 7.5 7.0 6.5 6.0 5.5 5.0 4.5 4.0 3.5 3.0 2.5 2.0 1.5 1.0 0.5 0.0
f1 (ppm)

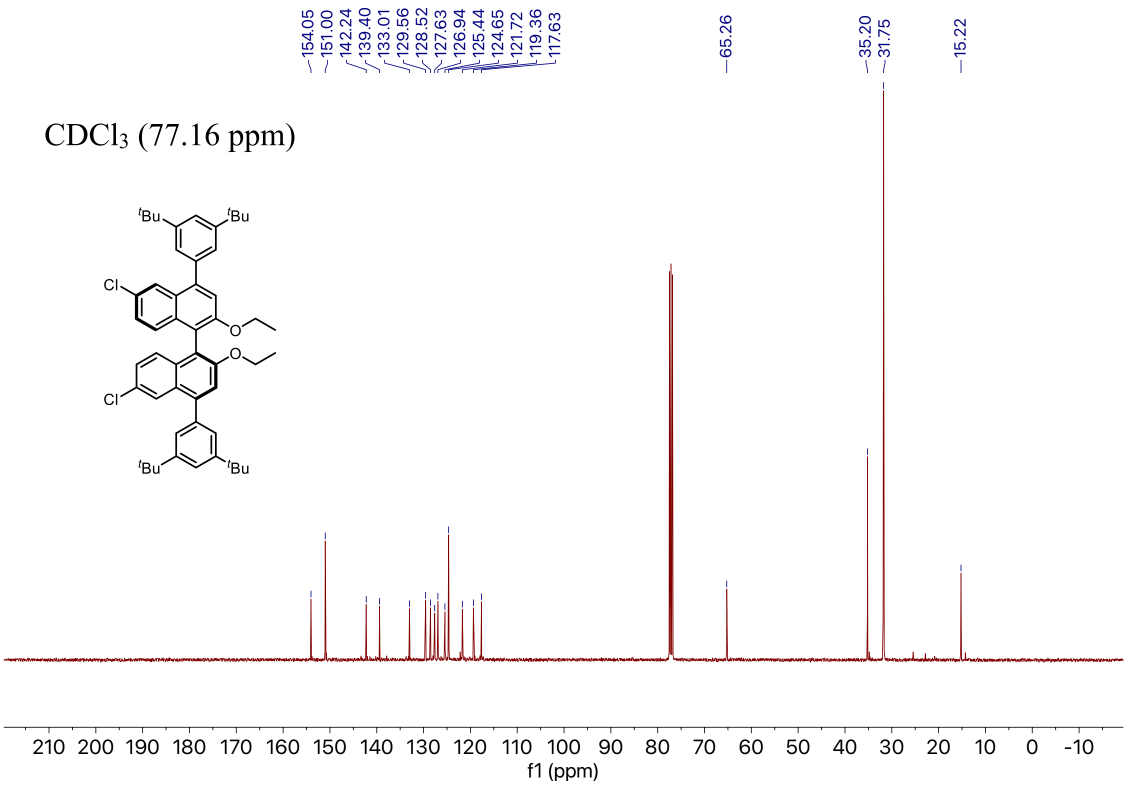
154.05
151.00
142.24
139.40
133.01
129.56
128.52
127.63
126.94
125.44
124.65
121.72
119.36
117.63
65.26
35.20
31.75
15.22
CDCl3 (77.16 ppm)
tBu
Cl
O
f1 (ppm)

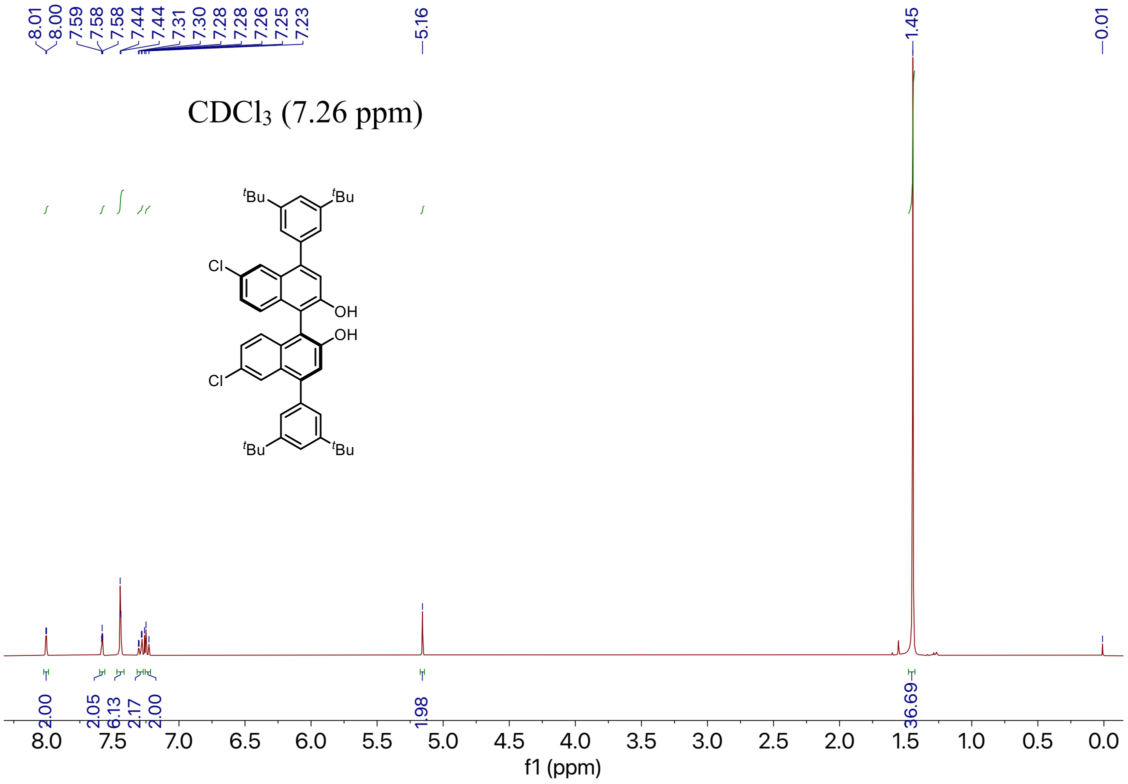
8.01
8.00
7.59
7.58
7.58
7.44
7.44
7.31
7.30
7.28
7.28
7.26
7.25
7.23
5.16
1.45
-0.01
CDCl3 (7.26 ppm)
tBu
Cl
OH
2.00
2.05
6.13
2.17
2.00
1.98
36.69
f1 (ppm)

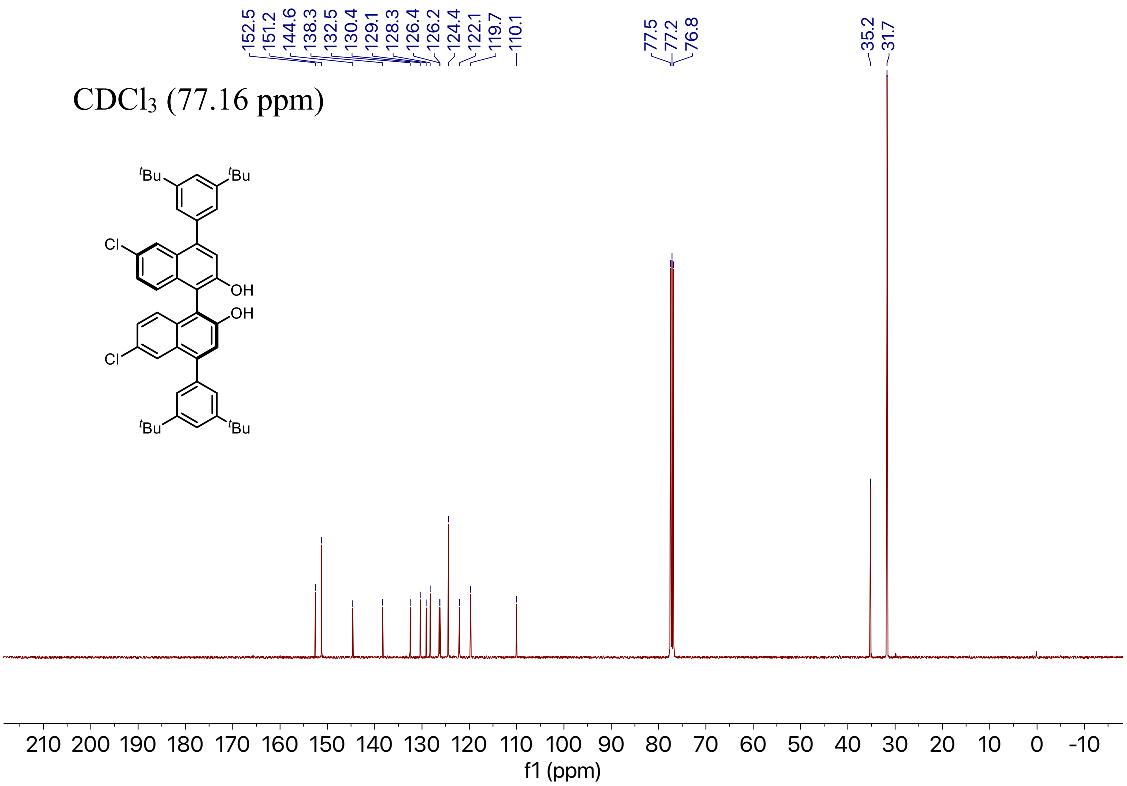
152.5
151.2
144.6
138.3
132.5
130.4
129.1
128.3
126.4
126.2
124.4
122.1
119.7
110.1
77.5
77.2
76.8
35.2
31.7
CDCl3 (77.16 ppm)
tBu
tBu
Cl
OH
OH
Cl
tBu
tBu
210 200 190 180 170 160 150 140 130 120 110 100 90 80 70 60 50 40 30 20 10 0 -10
f1 (ppm)

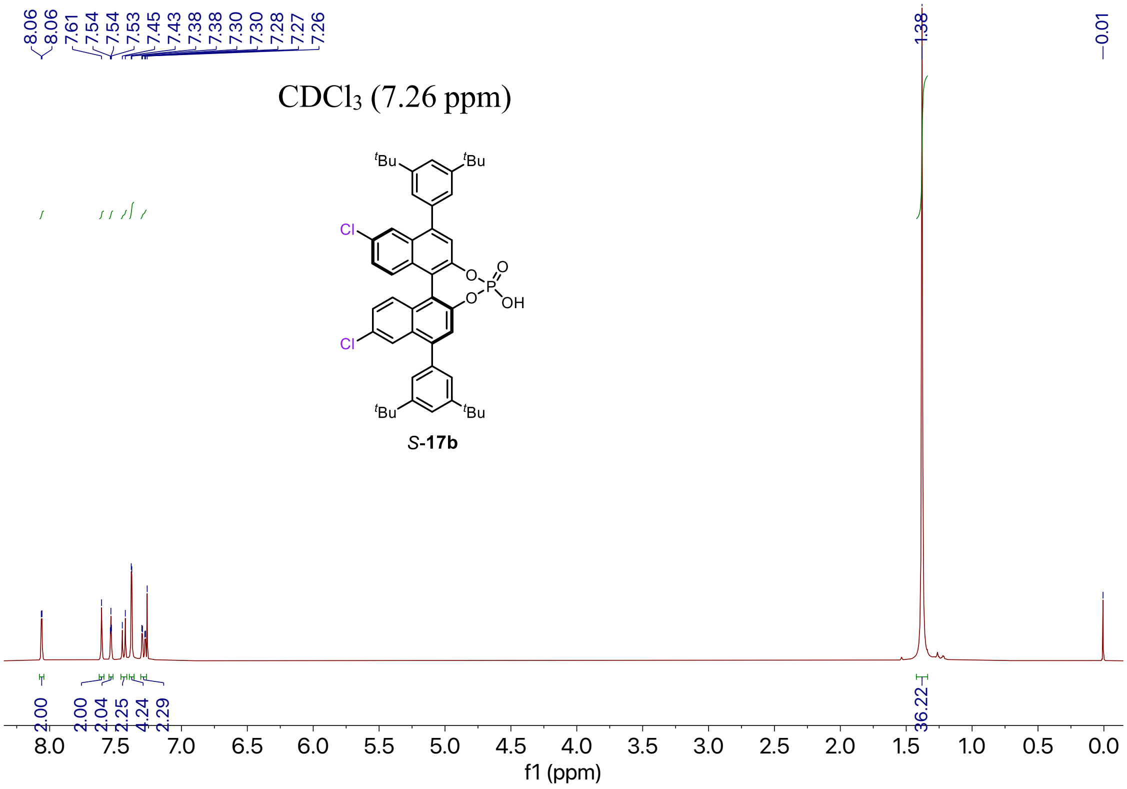
8.06
8.06
7.61
7.54
7.54
7.53
7.45
7.43
7.38
7.38
7.30
7.30
7.28
7.27
7.26
1.38
-0.01
CDCl3 (7.26 ppm)
tBu
tBu
Cl
O
O
P
O
OH
Cl
tBu
tBu
S-17b
2.00
2.00
2.04
2.25
4.24
2.29
36.22
8.0 7.5 7.0 6.5 6.0 5.5 5.0 4.5 4.0 3.5 3.0 2.5 2.0 1.5 1.0 0.5 0.0
f1 (ppm)

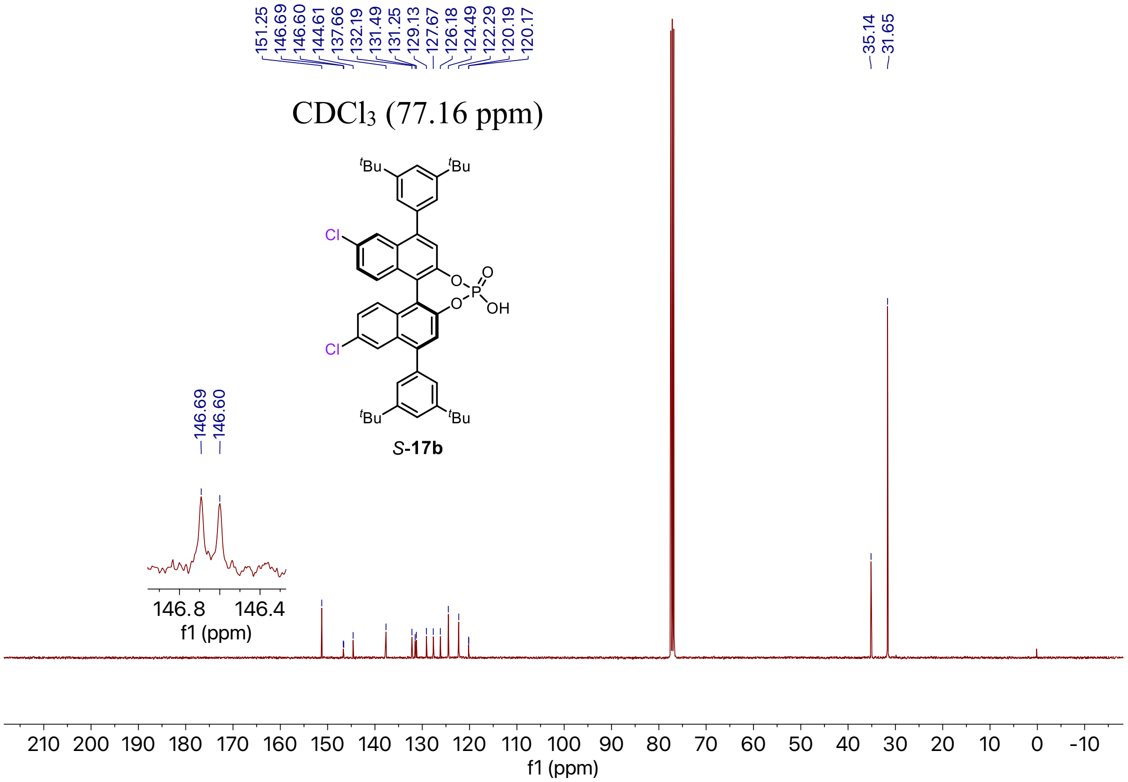
151.25
146.69
146.60
144.61
137.66
132.19
131.49
131.25
129.13
127.67
126.18
124.49
122.29
120.19
120.17
35.14
31.65
CDCl3 (77.16 ppm)
tBu
Cl
O
P
OH
S-17b
146.69
146.60
146.8
146.4
f1 (ppm)
210 200 190 180 170 160 150 140 130 120 110 100 90 80 70 60 50 40 30 20 10 0 -10
f1 (ppm)

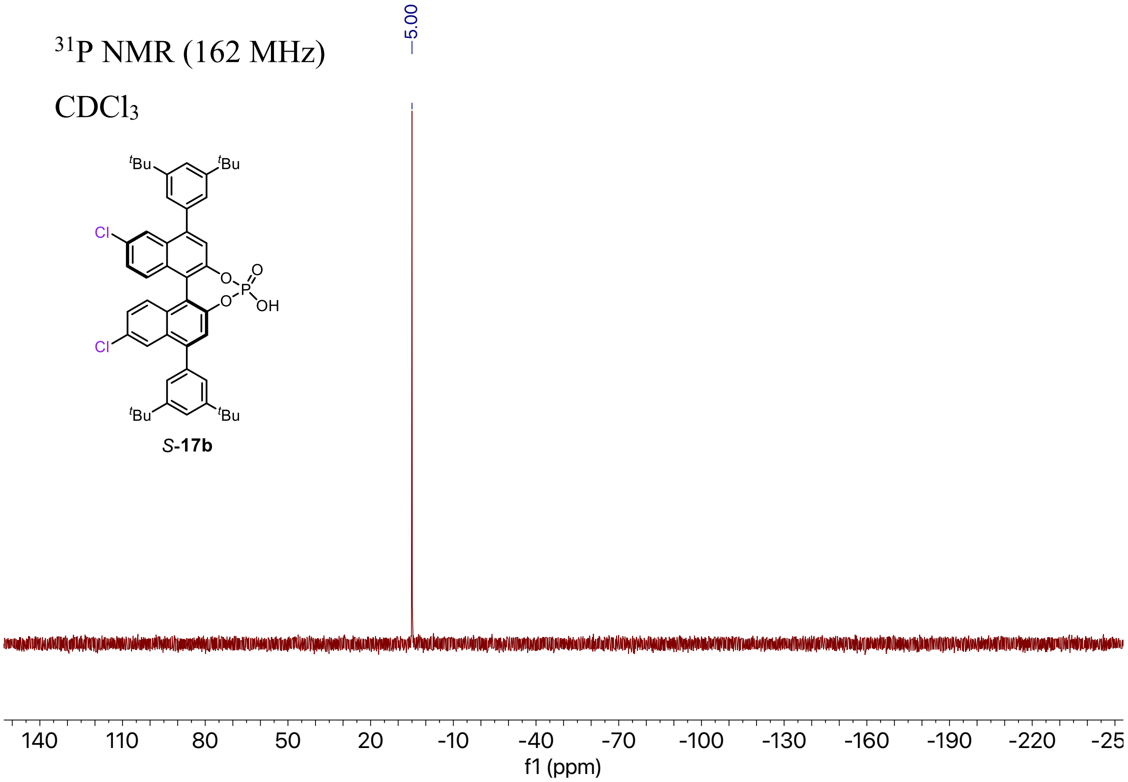
5.00
31P NMR (162 MHz)
CDCl3
tBu
Cl
O
P
OH
S-17b
140 110 80 50 20 -10 -40 -70 -100 -130 -160 -190 -220 -25
f1 (ppm)

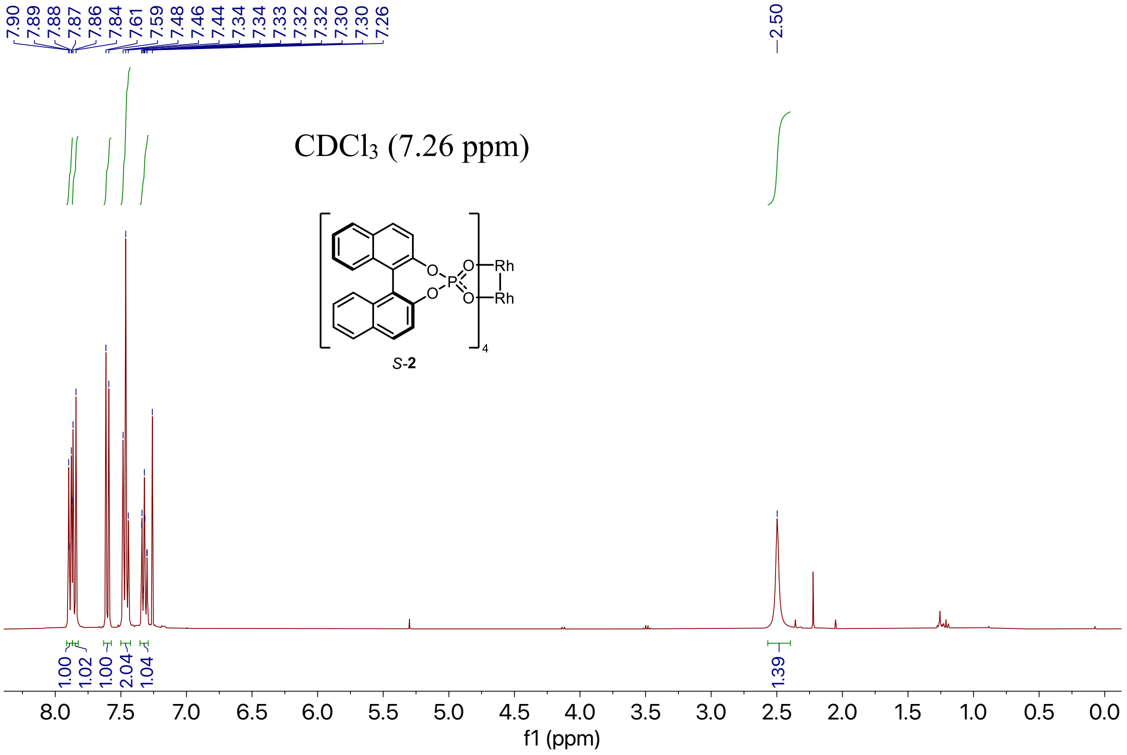
7.90
7.89
7.88
7.87
7.86
7.84
7.61
7.59
7.48
7.46
7.44
7.34
7.34
7.33
7.32
7.32
7.30
7.30
7.26
2.50
CDCl3 (7.26 ppm)
O
O
P
O
O
Rh
Rh
4
S-2
1.00
1.02
1.00
2.04
1.04
1.39
8.0 7.5 7.0 6.5 6.0 5.5 5.0 4.5 4.0 3.5 3.0 2.5 2.0 1.5 1.0 0.5 0.0
f1 (ppm)

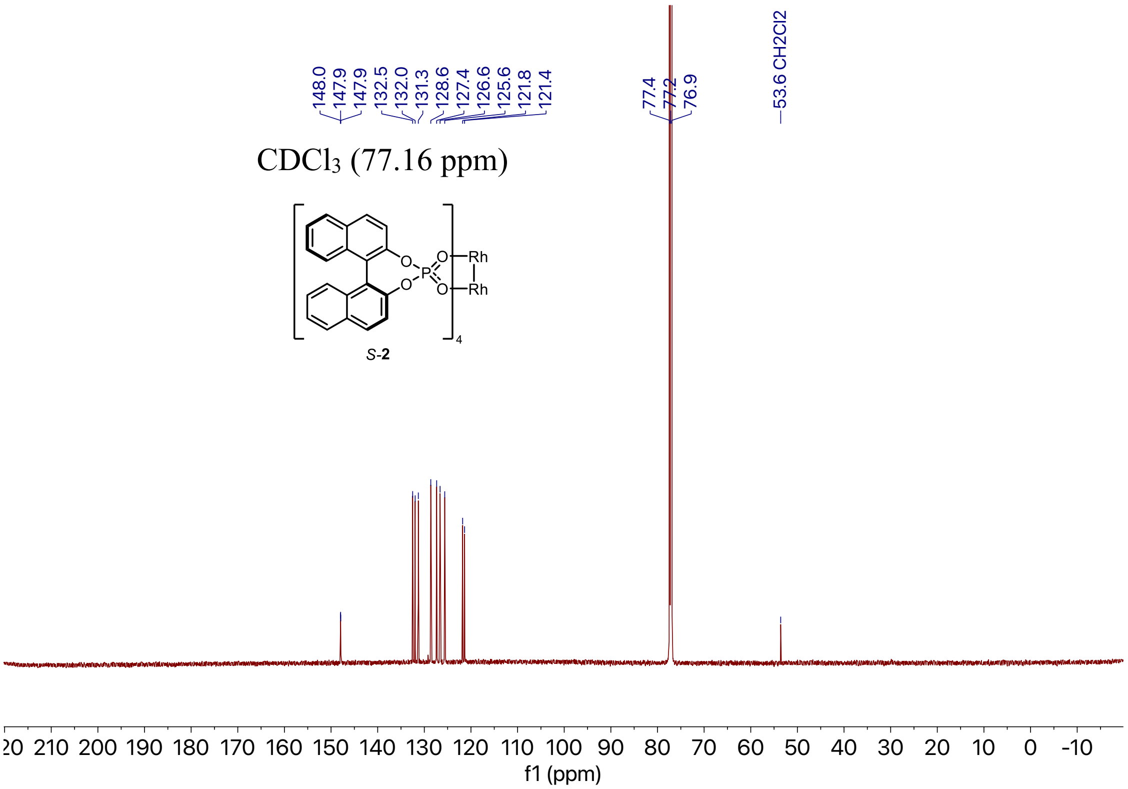
148.0
147.9
147.9
132.5
132.0
131.3
128.6
127.4
126.6
125.6
121.8
121.4
77.4
77.2
76.9
53.6 CH2Cl2
CDCl3 (77.16 ppm)
O
O
P
O
O
Rh
Rh
4
S-2
20 210 200 190 180 170 160 150 140 130 120 110 100 90 80 70 60 50 40 30 20 10 0 -10
f1 (ppm)

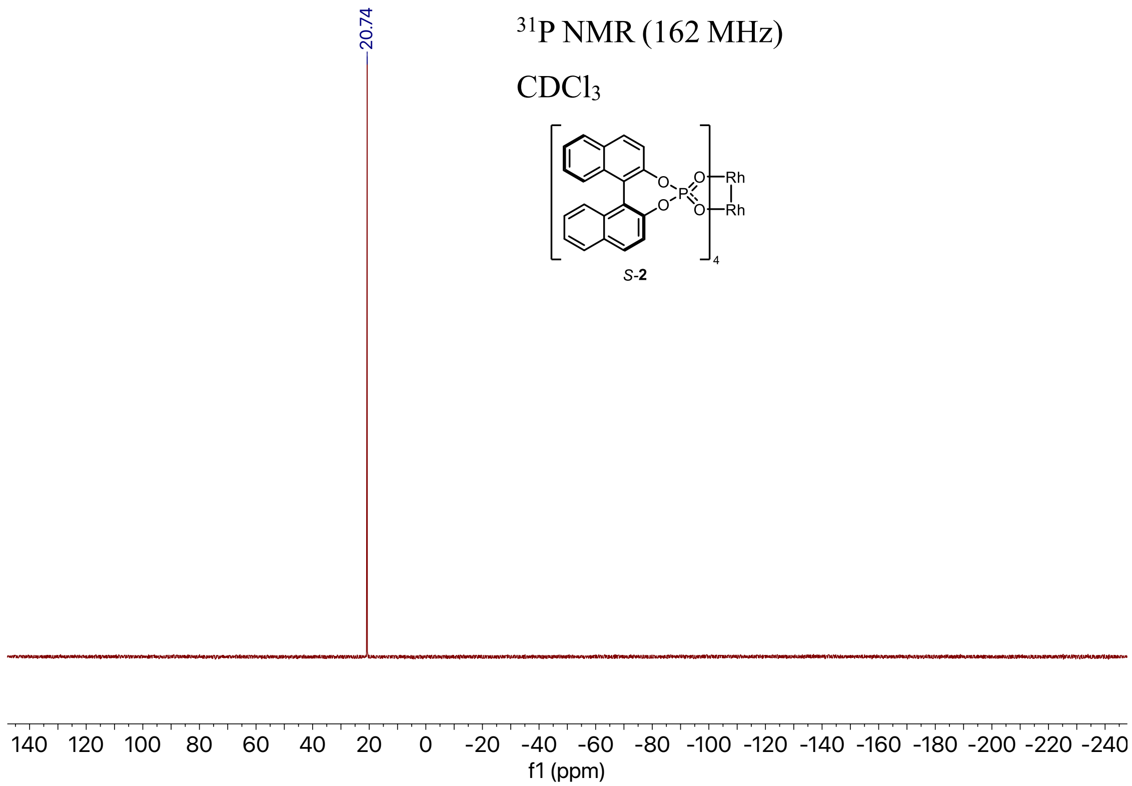
20.74
31P NMR (162 MHz)
CDCl3
O
P
O
O
O
Rh
Rh
4
S-2
140 120 100 80 60 40 20 0 -20 -40 -60 -80 -100 -120 -140 -160 -180 -200 -220 -240
f1 (ppm)

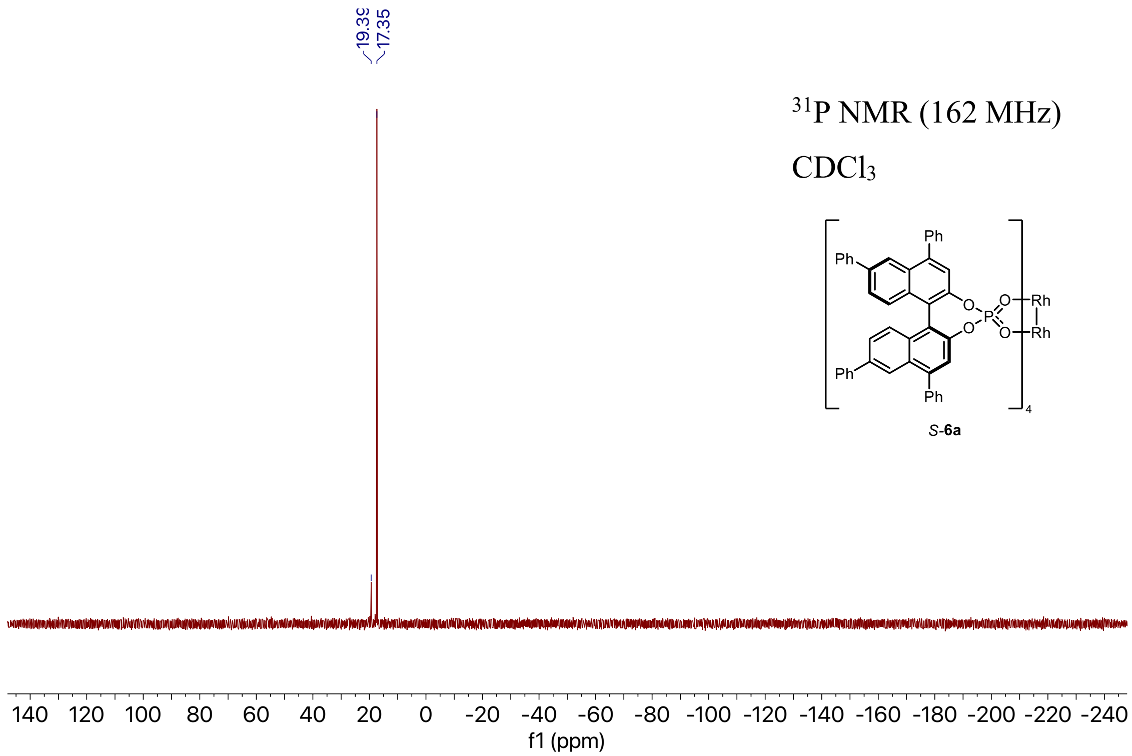
19.39
17.35
31P NMR (162 MHz)
CDCl3
Ph
Ph
Ph
Ph
O
P
O
O
O
Rh
Rh
4
S-6a
140 120 100 80 60 40 20 0 -20 -40 -60 -80 -100 -120 -140 -160 -180 -200 -220 -240
f1 (ppm)

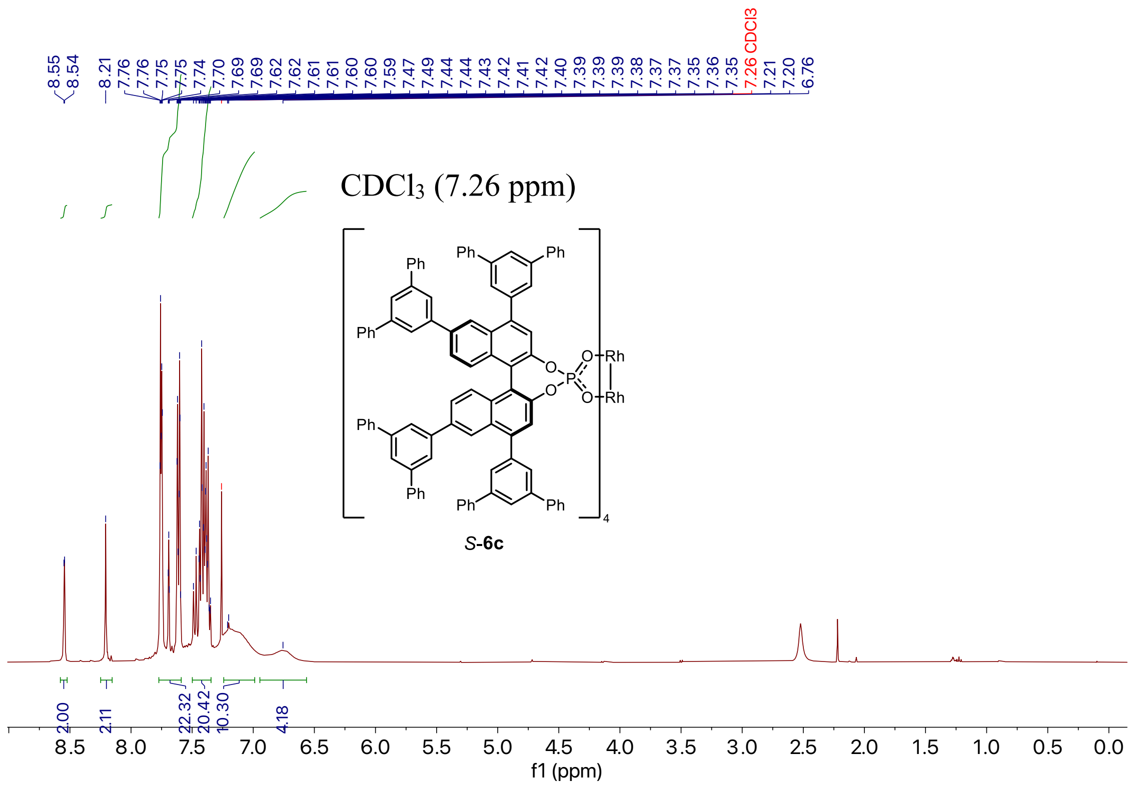
8.55
8.54
8.21
7.76
7.75
7.75
7.74
7.70
7.69
7.69
7.62
7.62
7.61
7.61
7.60
7.60
7.59
7.47
7.49
7.44
7.44
7.43
7.42
7.41
7.42
7.40
7.39
7.39
7.39
7.38
7.37
7.37
7.35
7.36
7.35
7.26 CDCl3
7.21
7.20
6.76
CDCl3 (7.26 ppm)
Ph
Rh
O
P
S-6c
4
2.00
2.11
22.32
20.42
10.30
4.18
8.5 8.0 7.5 7.0 6.5 6.0 5.5 5.0 4.5 4.0 3.5 3.0 2.5 2.0 1.5 1.0 0.5 0.0
f1 (ppm)

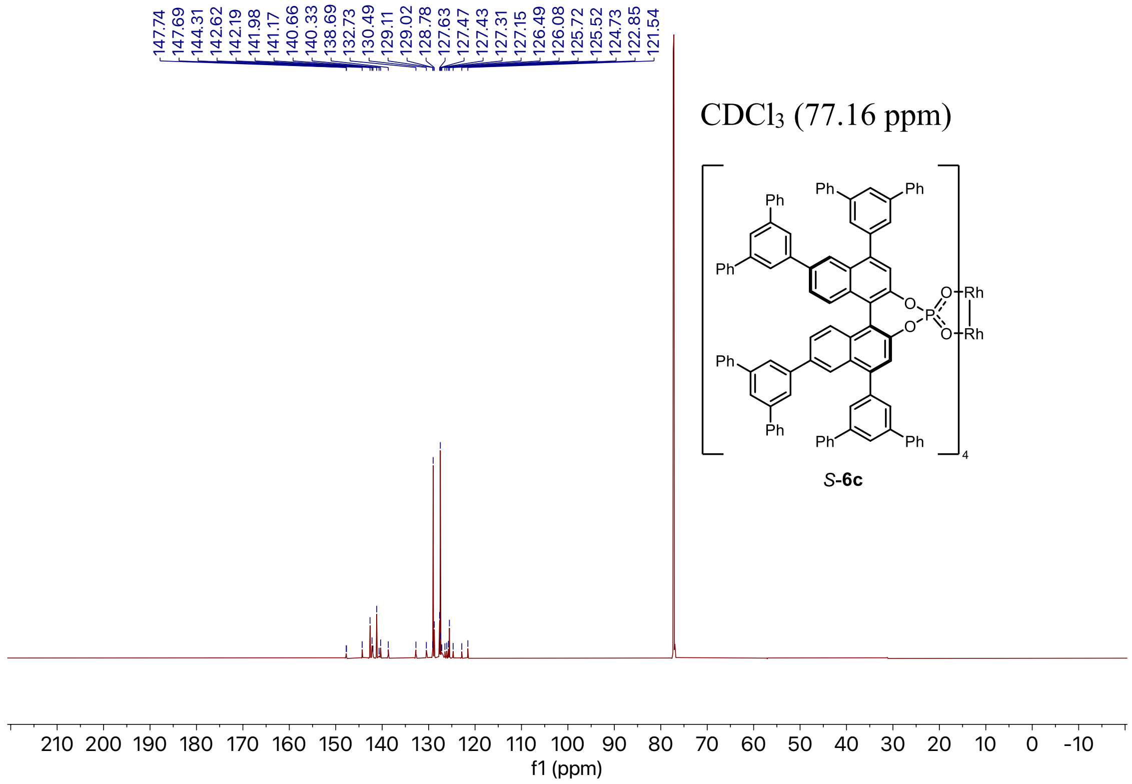
147.74
147.69
144.31
142.62
142.19
141.98
141.17
140.66
140.33
138.69
132.73
130.49
129.11
129.02
128.78
127.63
127.47
127.43
127.31
127.15
126.49
126.08
125.72
125.52
124.73
122.85
121.54
CDCl3 (77.16 ppm)
Ph
Rh
O
P
S-6c
4
210 200 190 180 170 160 150 140 130 120 110 100 90 80 70 60 50 40 30 20 10 0 -10
f1 (ppm)

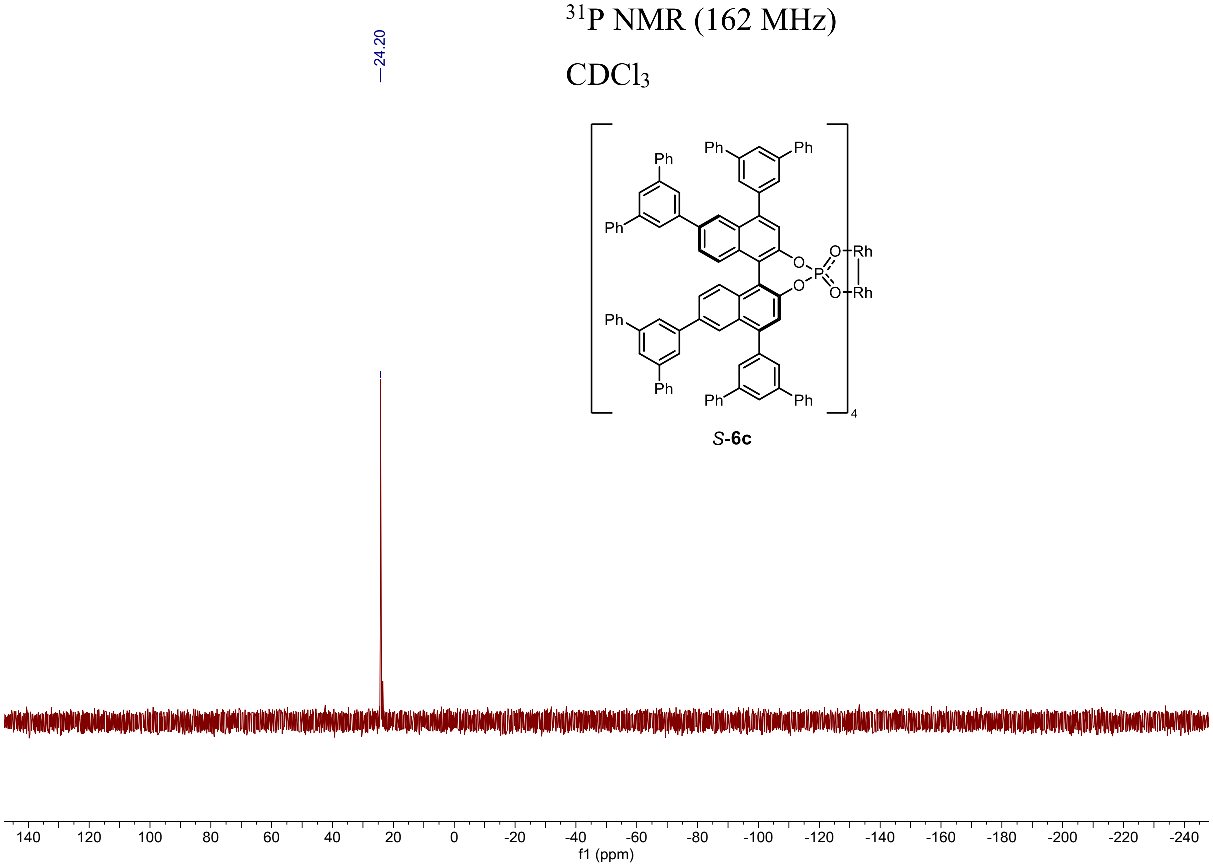

31P NMR (162 MHz)
CDCl3
24.20
S-6c
f1 (ppm)

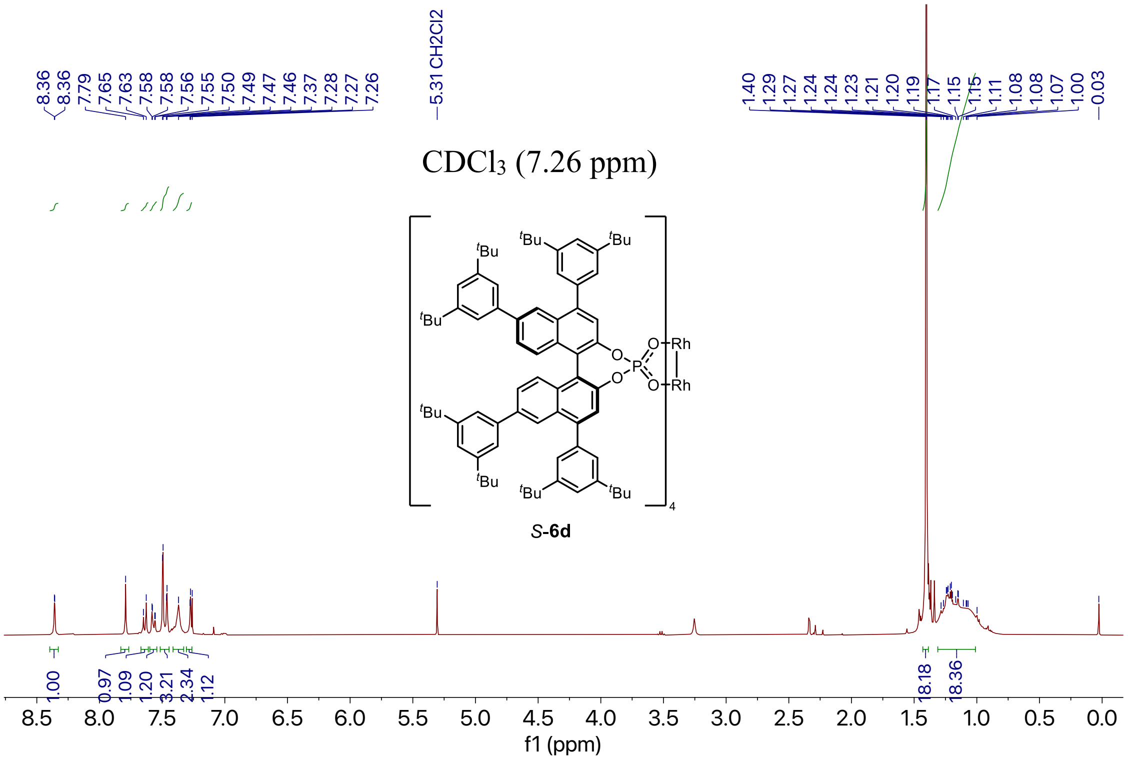

CDCl3 (7.26 ppm)
5.31 CH2Cl2
S-6d
f1 (ppm)

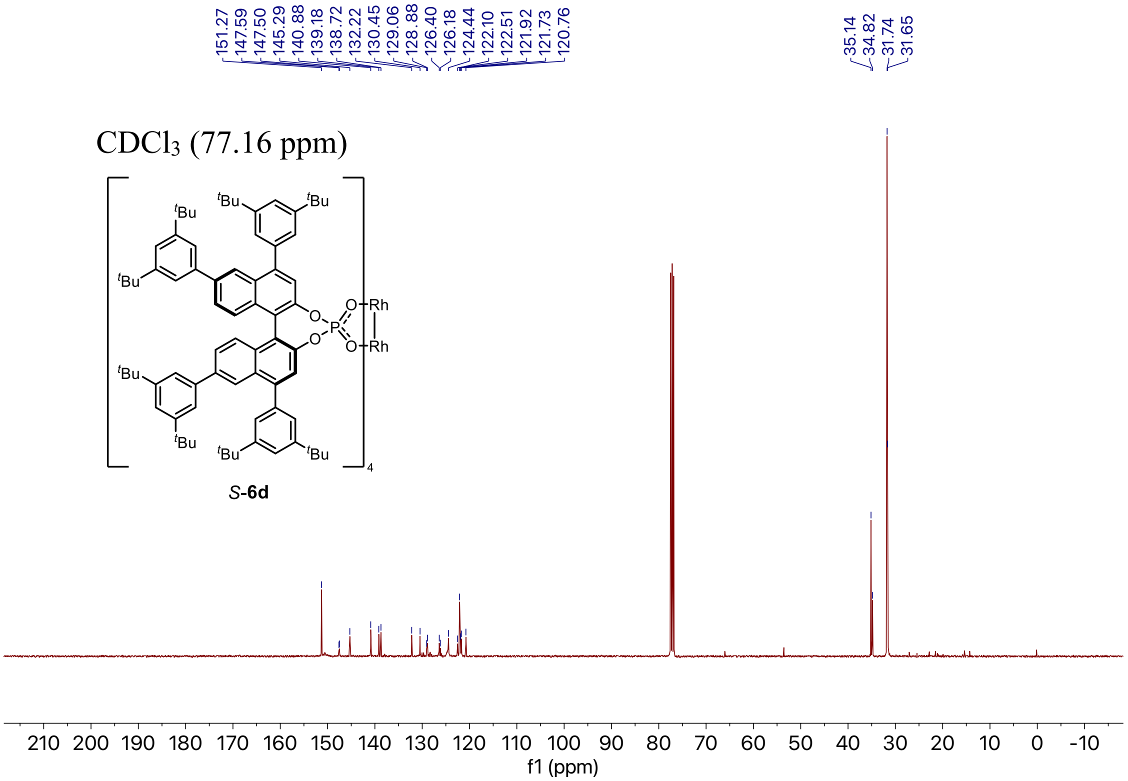
151.27
147.59
147.50
145.29
140.88
139.18
138.72
132.22
130.45
129.06
128.88
126.40
126.18
124.44
122.10
122.51
121.92
121.73
120.76
35.14
34.82
31.74
31.65
CDCl3 (77.16 ppm)
S-6d
210 200 190 180 170 160 150 140 130 120 110 100 90 80 70 60 50 40 30 20 10 0 -10
f1 (ppm)

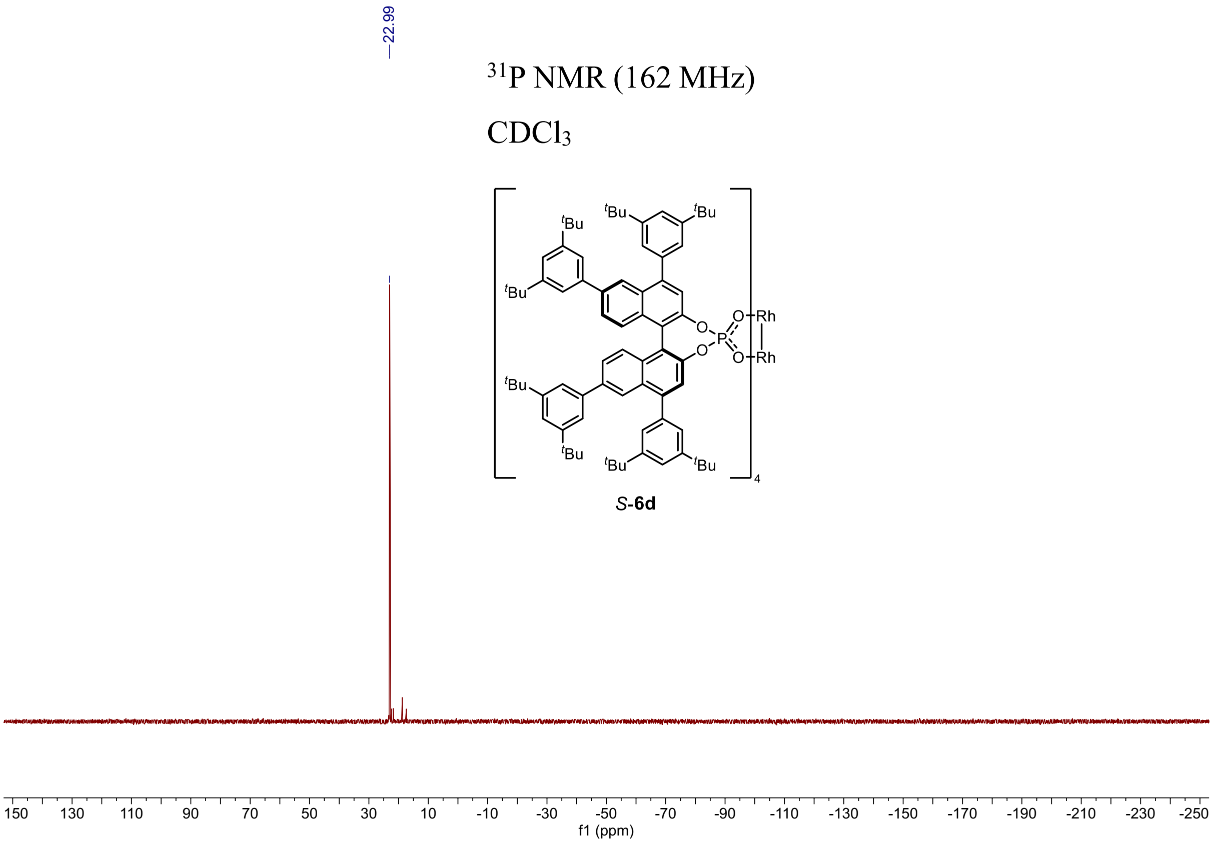
22.99
31P NMR (162 MHz)
CDCl3
S-6d
150 130 110 90 70 50 30 10 -10 -30 -50 -70 -90 -110 -130 -150 -170 -190 -210 -230 -250
f1 (ppm)

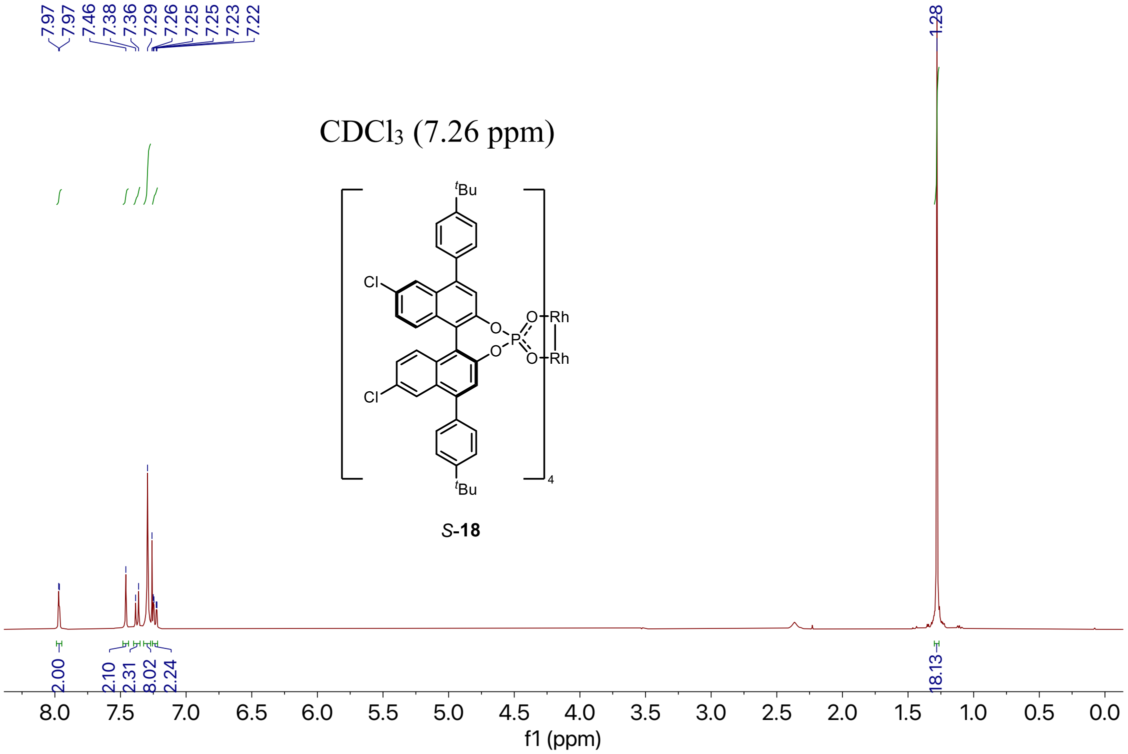

7.97
7.97
7.46
7.38
7.36
7.29
7.26
7.25
7.25
7.23
7.22
1.28
CDCl3 (7.26 ppm)
tBu
Cl
O
P
O
O
O
Rh
Rh
Cl
tBu
4
S-18
2.00
2.10
2.31
8.02
2.24
18.13
8.0 7.5 7.0 6.5 6.0 5.5 5.0 4.5 4.0 3.5 3.0 2.5 2.0 1.5 1.0 0.5 0.0
f1 (ppm)

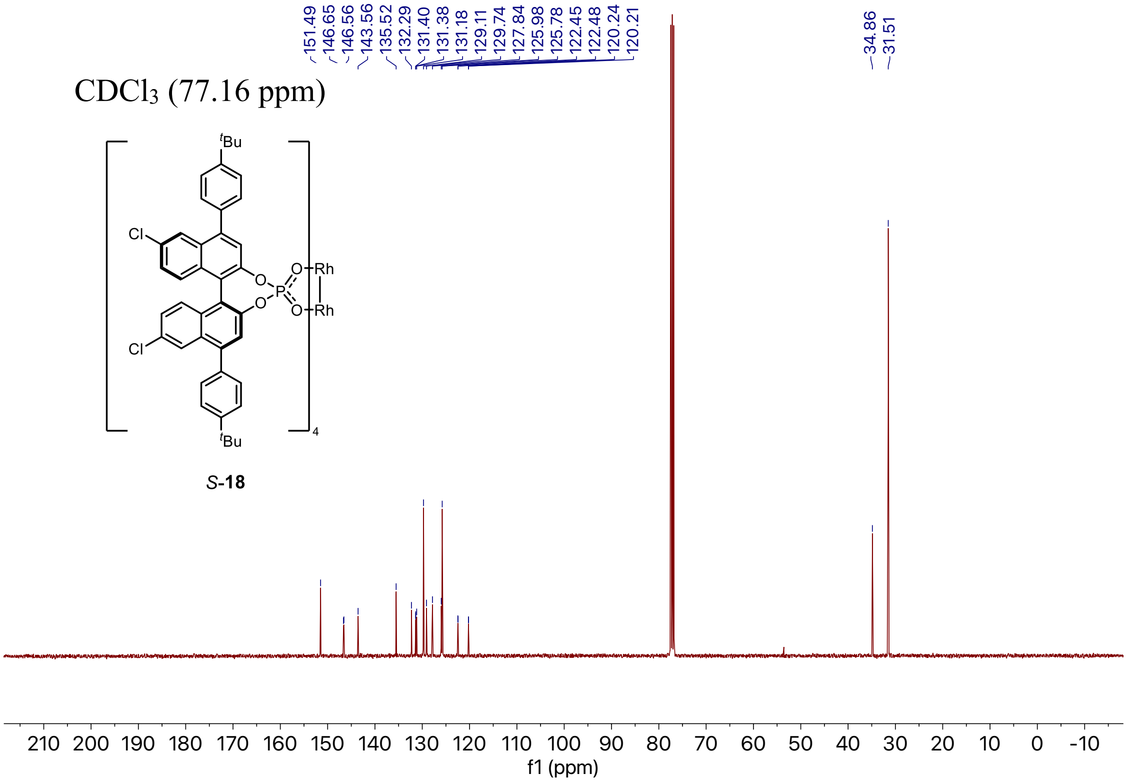

151.49
146.65
146.56
143.56
135.52
132.29
131.40
131.38
131.18
129.11
129.74
127.84
125.98
125.78
122.45
122.48
120.24
120.21
34.86
31.51
CDCl3 (77.16 ppm)
tBu
Cl
O
P
O
O
O
Rh
Rh
Cl
tBu
4
S-18
210 200 190 180 170 160 150 140 130 120 110 100 90 80 70 60 50 40 30 20 10 0 -10
f1 (ppm)

^{31}P NMR (162 MHz)

$CDCl_3$

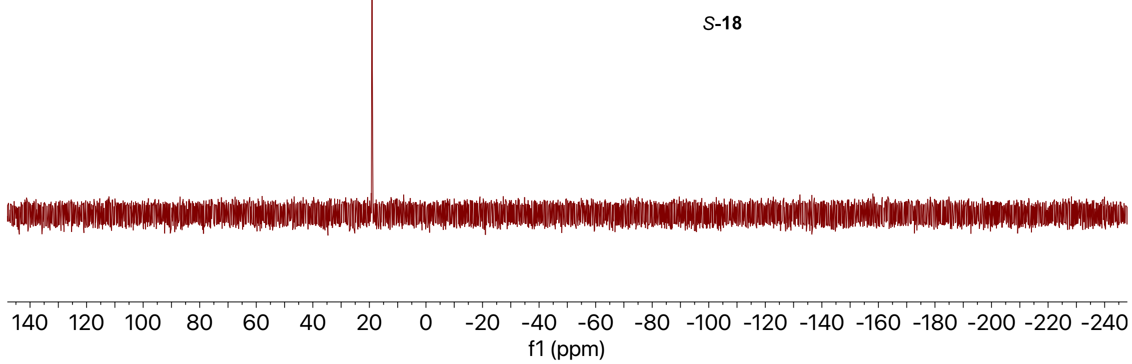

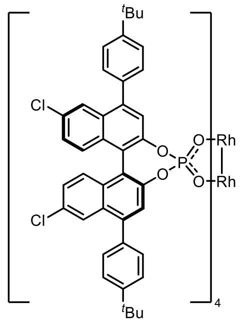

S-**18**

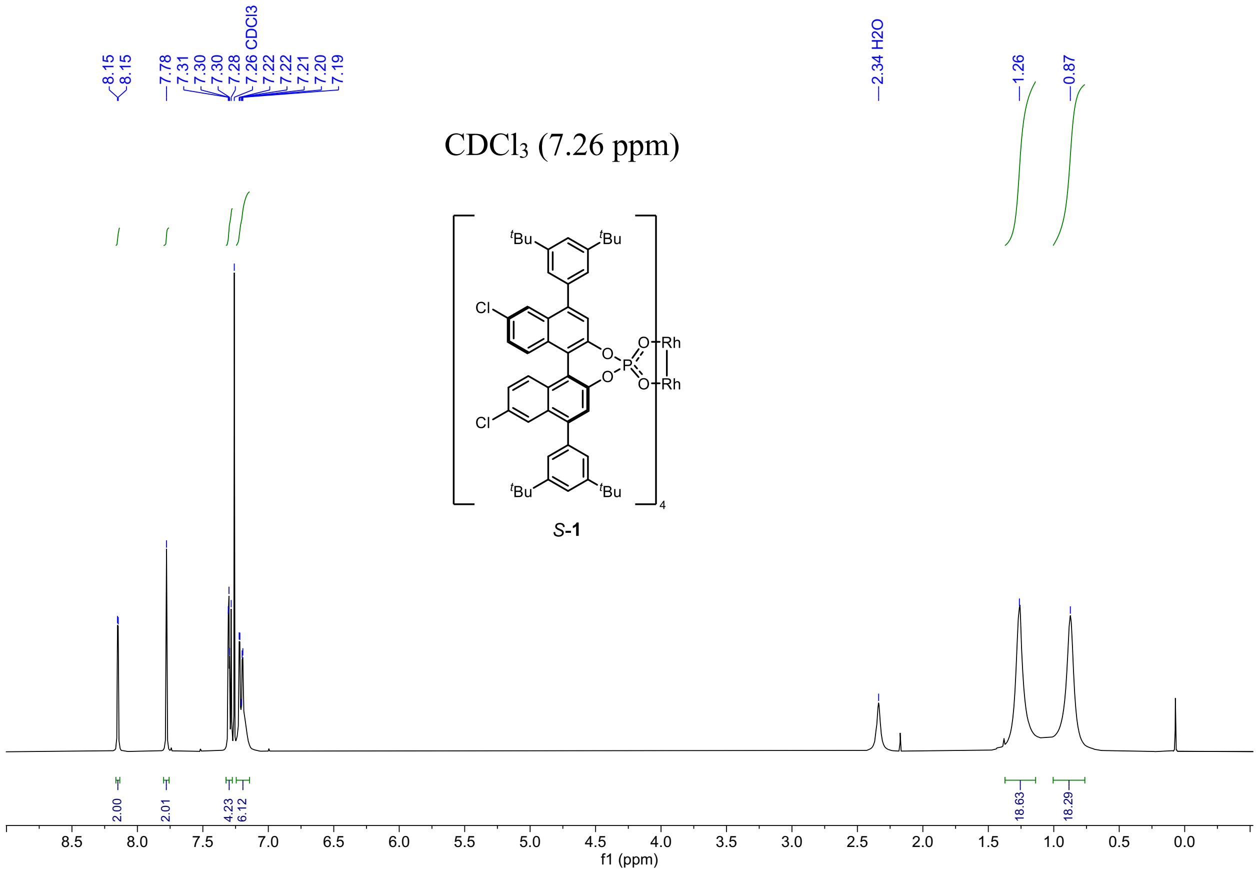
8.15
8.15
7.78
7.31
7.30
7.30
7.28
7.26 CDCl3
7.22
7.22
7.21
7.20
7.19
2.34 H2O
1.26
0.87
CDCl3 (7.26 ppm)
tBu
tBu
Cl
O
P
O
O
O
Rh
Rh
Cl
tBu
tBu
4
S-1
2.00
2.01
4.23
6.12
18.63
18.29
f1 (ppm)

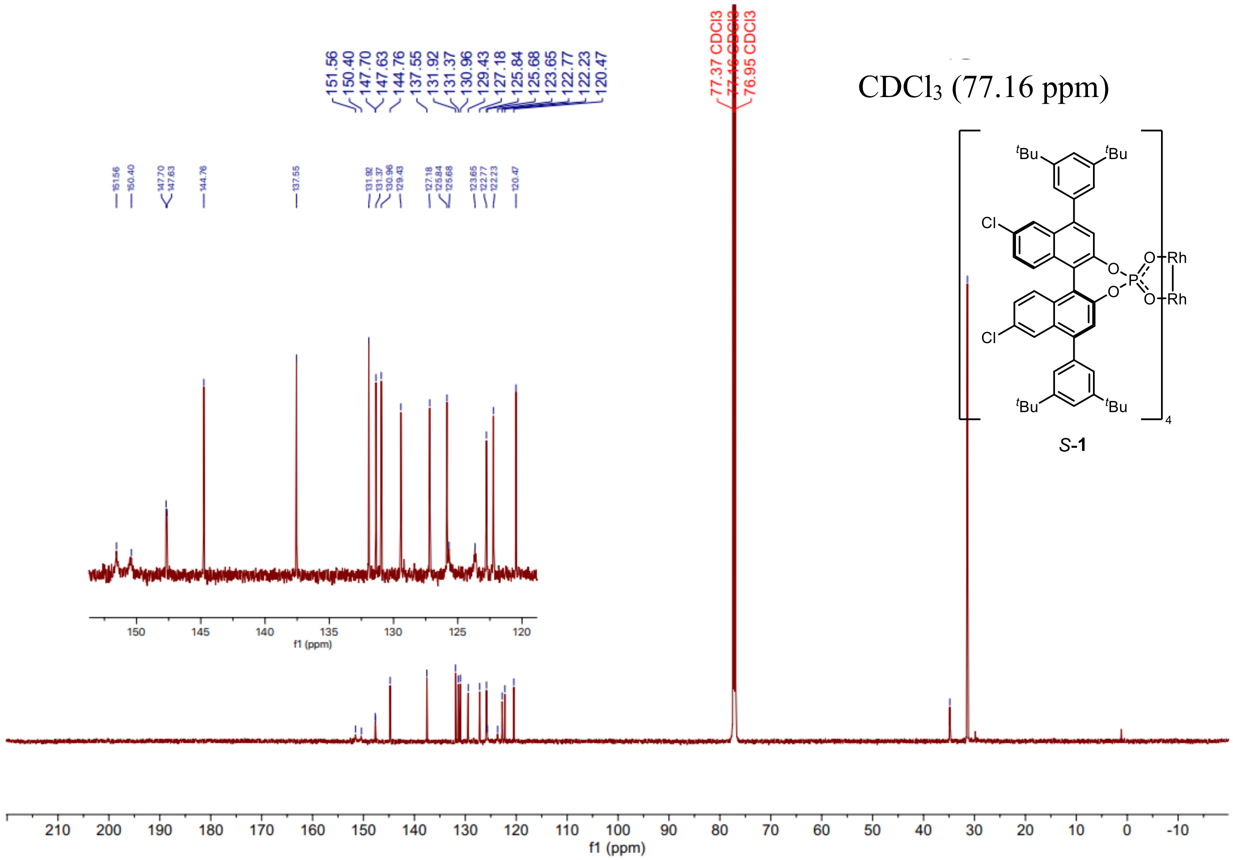
151.56
150.40
147.70
147.63
144.76
137.55
131.92
131.37
130.96
129.43
127.18
125.84
125.68
123.65
122.77
122.23
120.47
77.37 CDCl3
77.16 CDCl3
76.95 CDCl3
CDCl3 (77.16 ppm)
tBu
tBu
Cl
O
P
O
O
O
Rh
Rh
Cl
tBu
tBu
4
S-1
f1 (ppm)

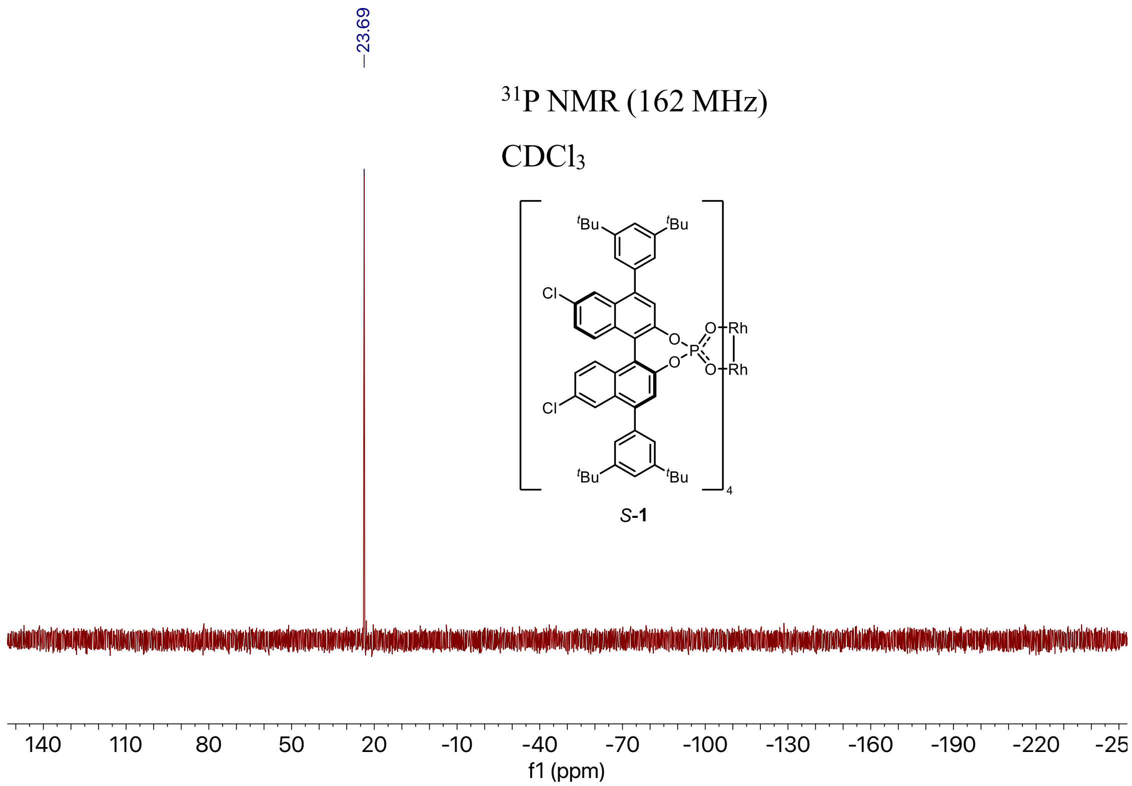

—23.69
^{31}P NMR (162 MHz)
CDCl$_3$
tBu
tBu
Cl
O
O
P
O
O
Rh
Rh
Cl
tBu
tBu
4
S-1
140
110
80
50
20
-10
-40
-70
-100
-130
-160
-190
-220
-25
f1 (ppm)

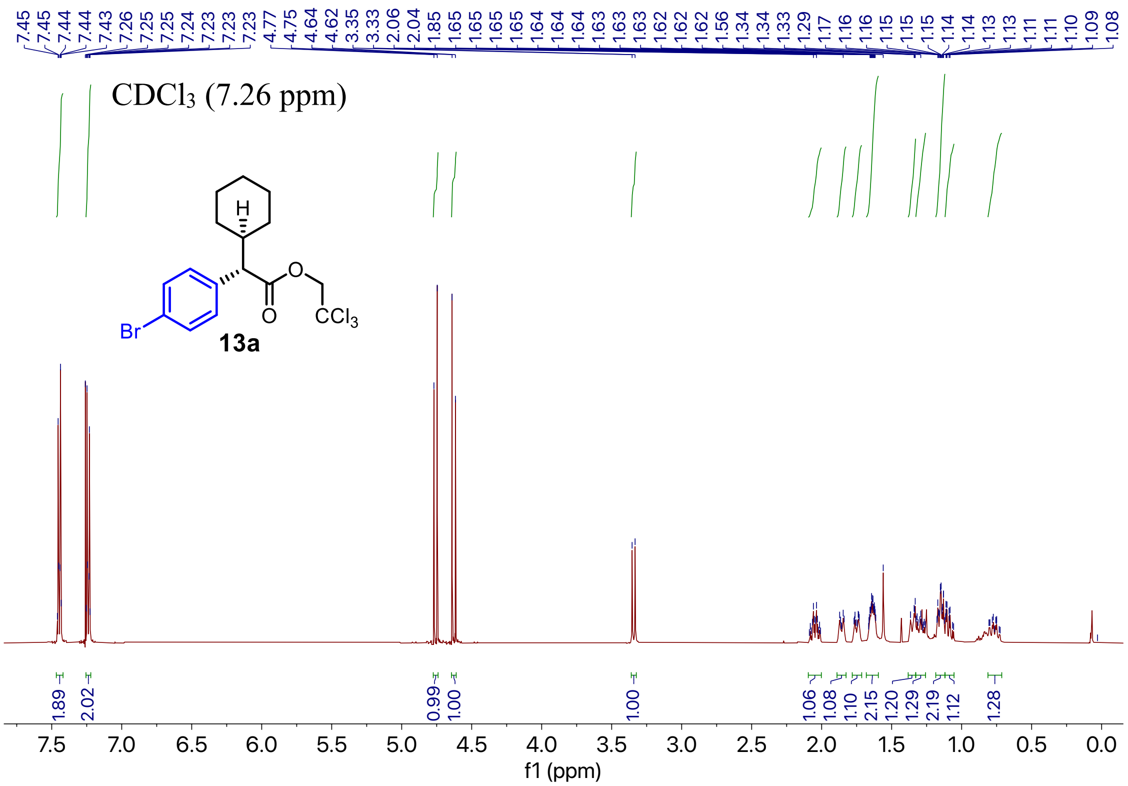

CDCl3 (7.26 ppm)
13a
f1 (ppm)

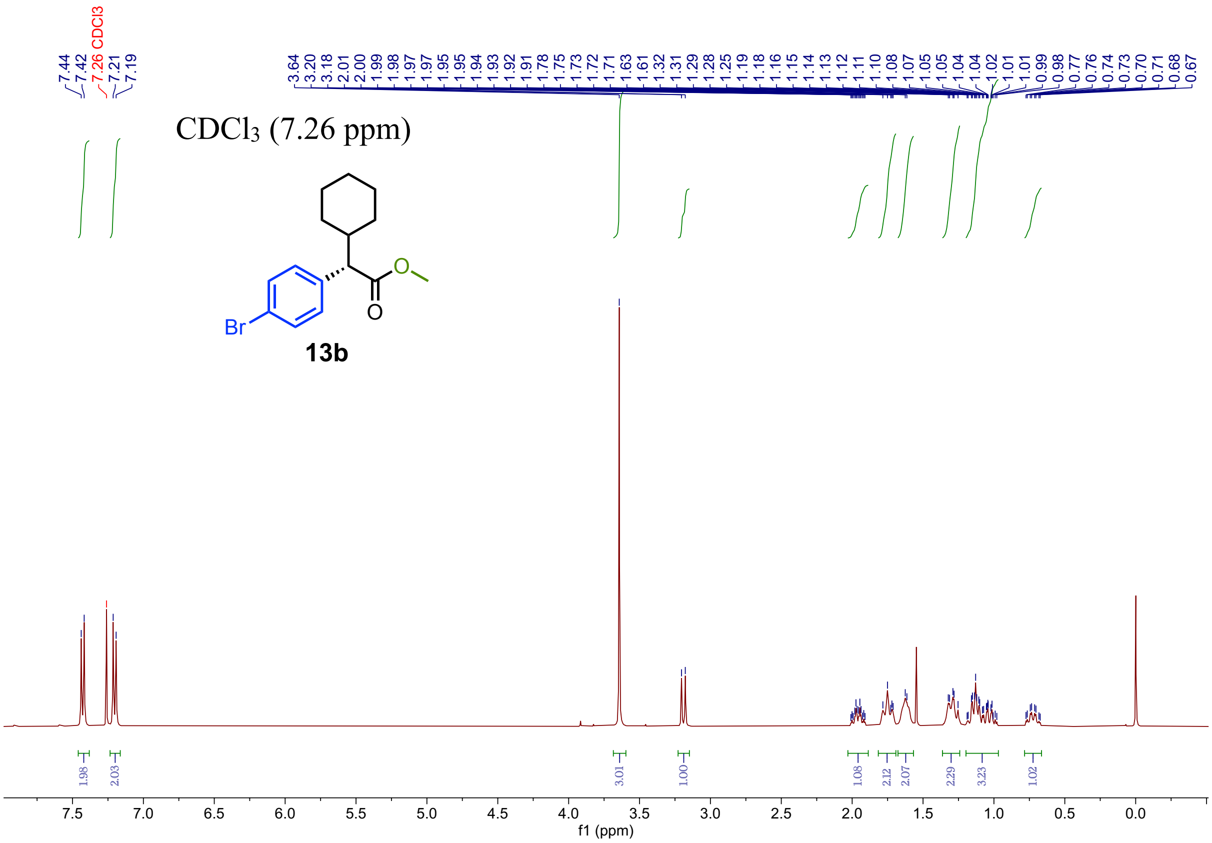

CDCl3 (7.26 ppm)
13b
f1 (ppm)

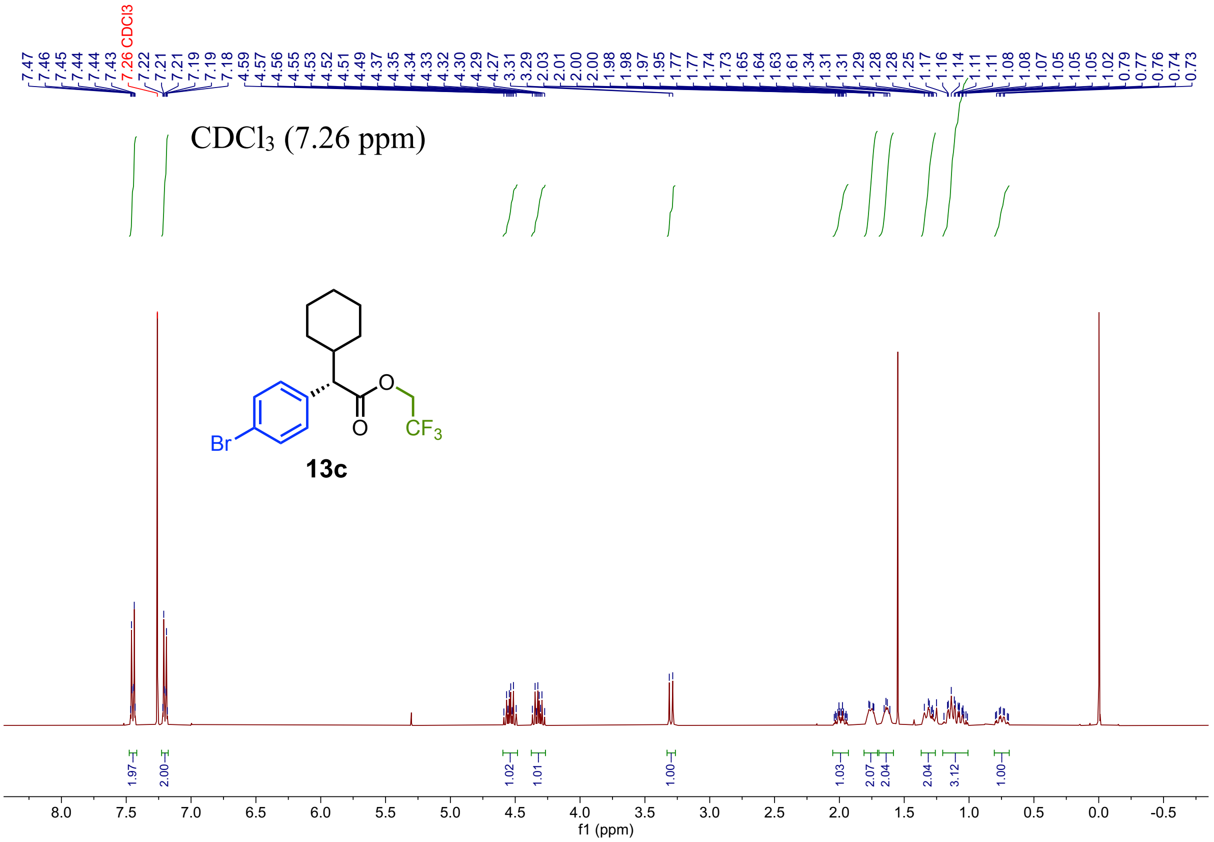

13c

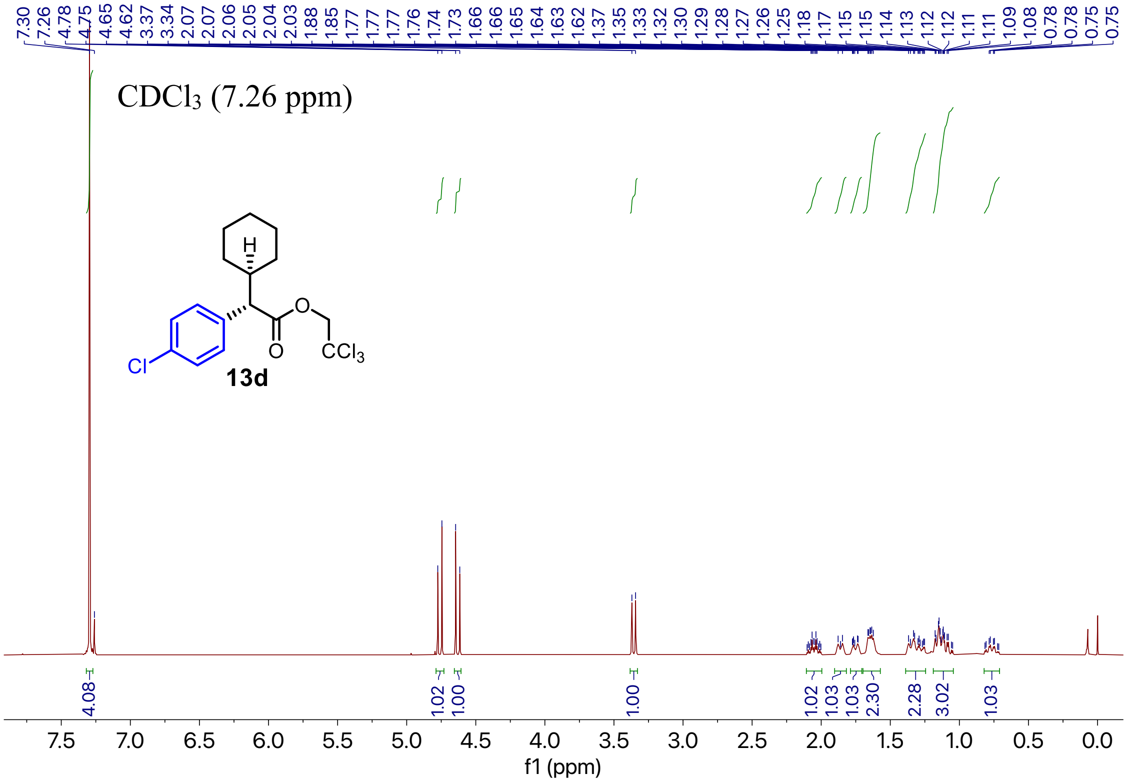

13d

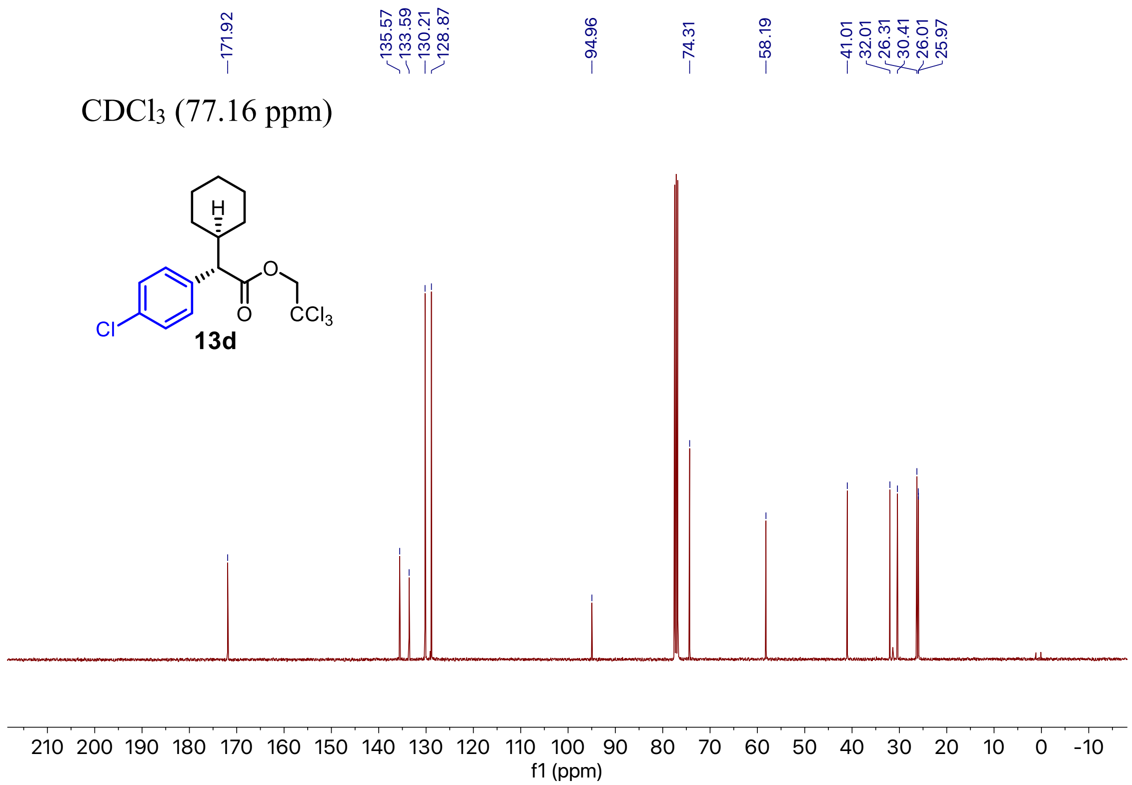
171.92
135.57
133.59
130.21
128.87
94.96
74.31
58.19
41.01
32.01
26.31
30.41
26.01
25.97
CDCl3 (77.16 ppm)
H
O
O
CCl3
Cl
13d
f1 (ppm)

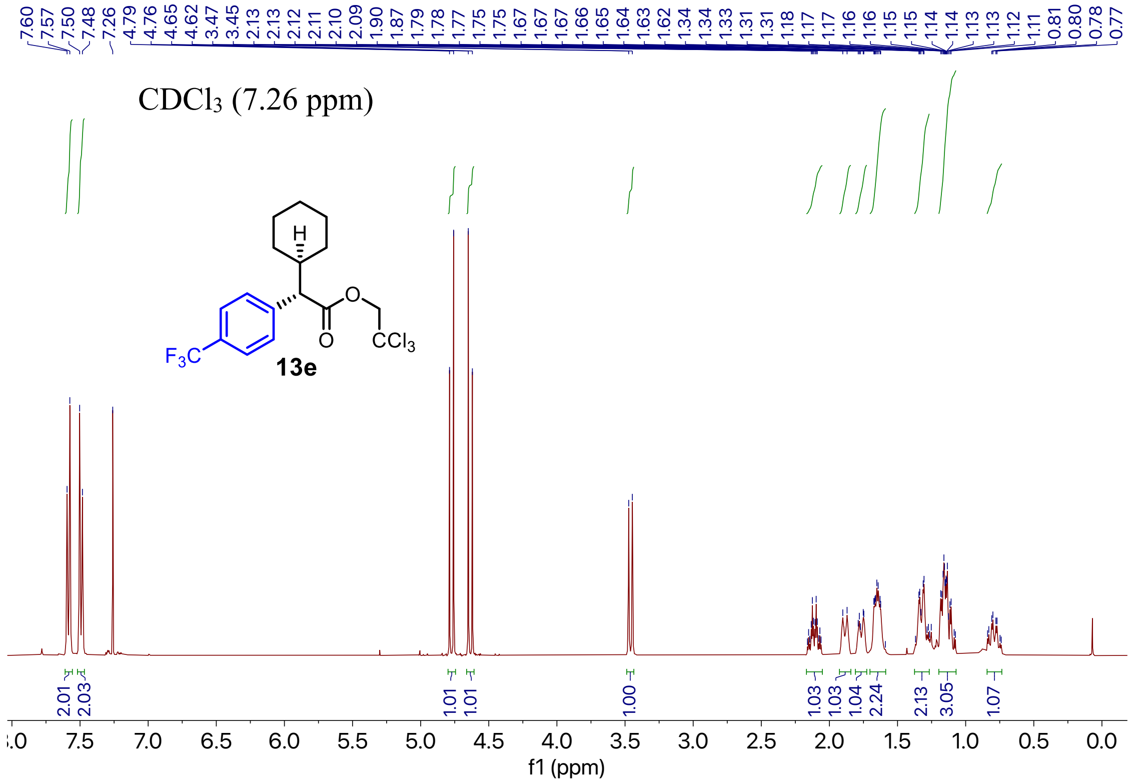
CDCl3 (7.26 ppm)
H
O
O
CCl3
F3C
13e
2.01
2.03
1.01
1.01
1.00
1.03
1.03
1.04
2.24
2.13
3.05
1.07
f1 (ppm)

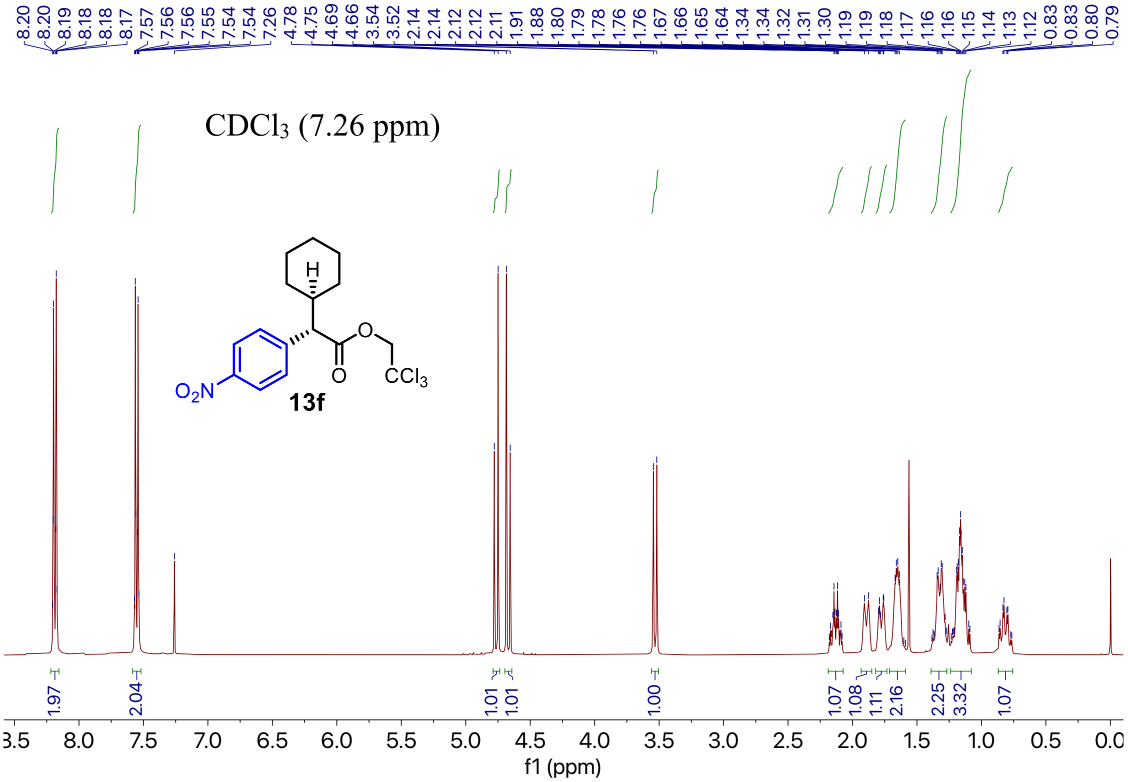

CDCl3 (7.26 ppm)
O2N
O
O
CCl3
H
13f
1.97
2.04
1.01
1.01
1.00
1.07
1.08
1.11
2.16
2.25
3.32
1.07
f1 (ppm)

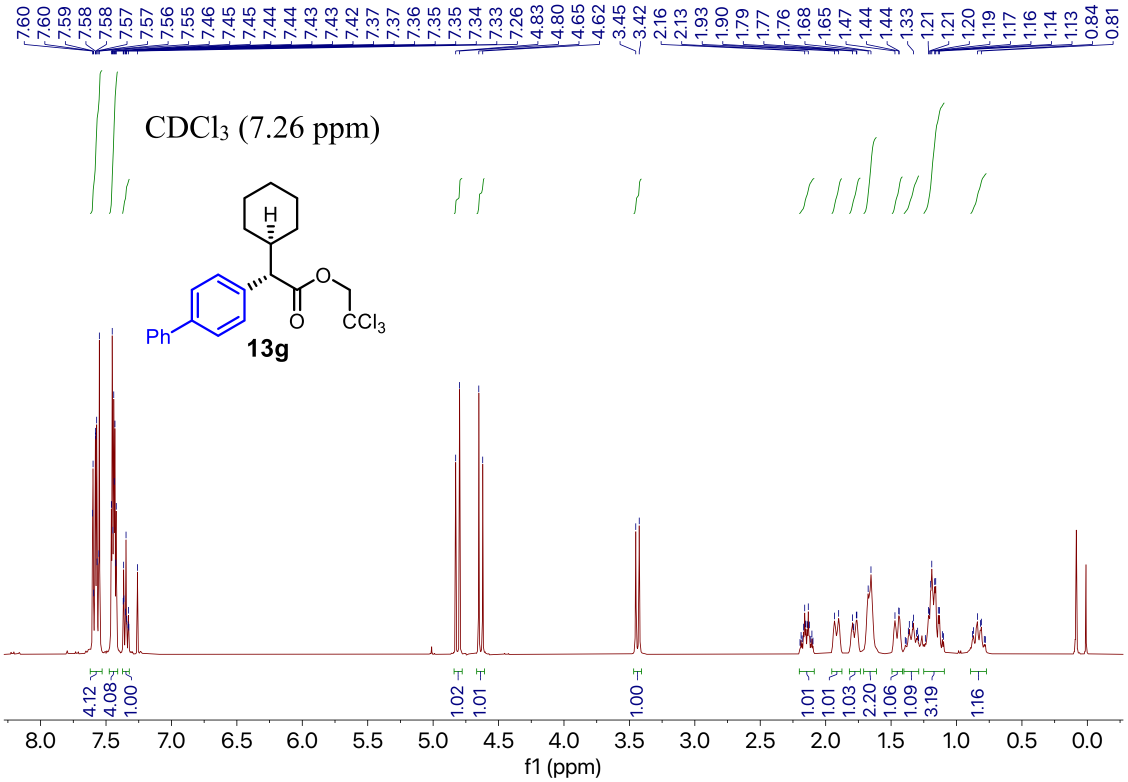

CDCl3 (7.26 ppm)
Ph
O
O
CCl3
H
13g
4.12
4.08
1.00
1.02
1.01
1.00
1.01
1.01
1.03
2.20
1.06
1.09
3.19
1.16
f1 (ppm)

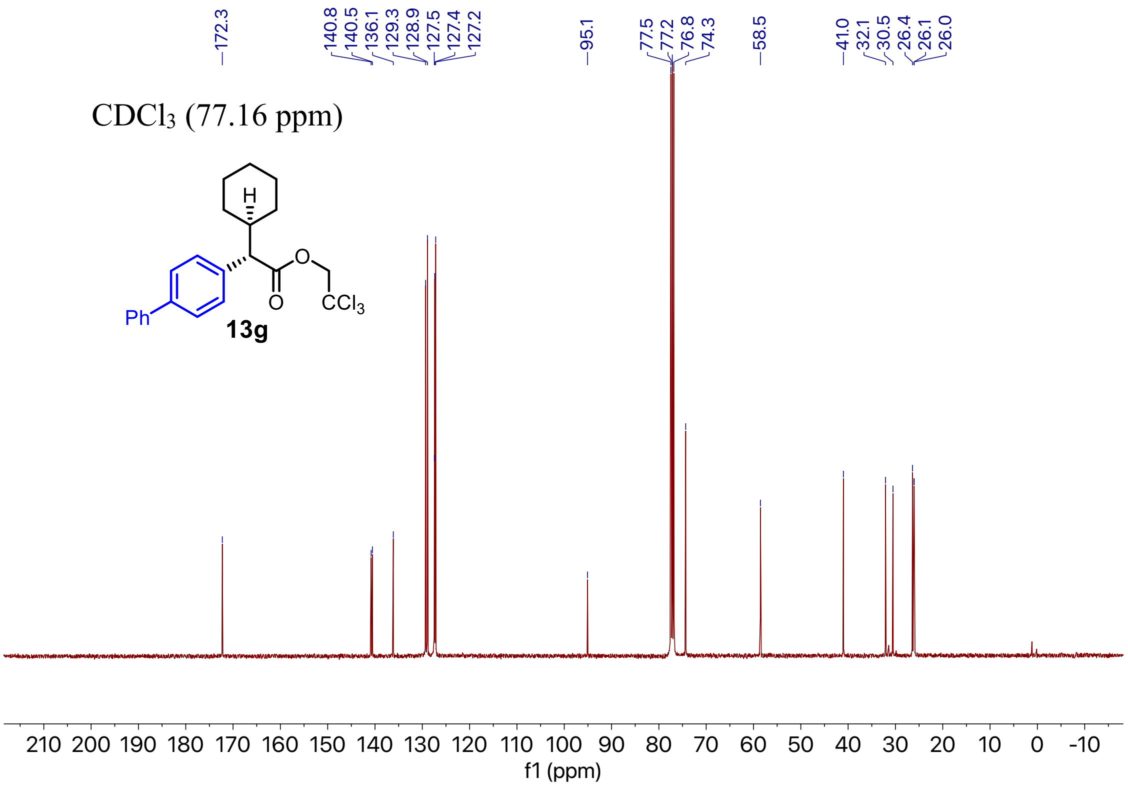
172.3
140.8
140.5
136.1
129.3
128.9
127.5
127.4
127.2
95.1
77.5
77.2
76.8
74.3
58.5
41.0
32.1
30.5
26.4
26.1
26.0
CDCl3 (77.16 ppm)
H
O
O
CCl3
Ph
13g
210 200 190 180 170 160 150 140 130 120 110 100 90 80 70 60 50 40 30 20 10 0 -10
f1 (ppm)

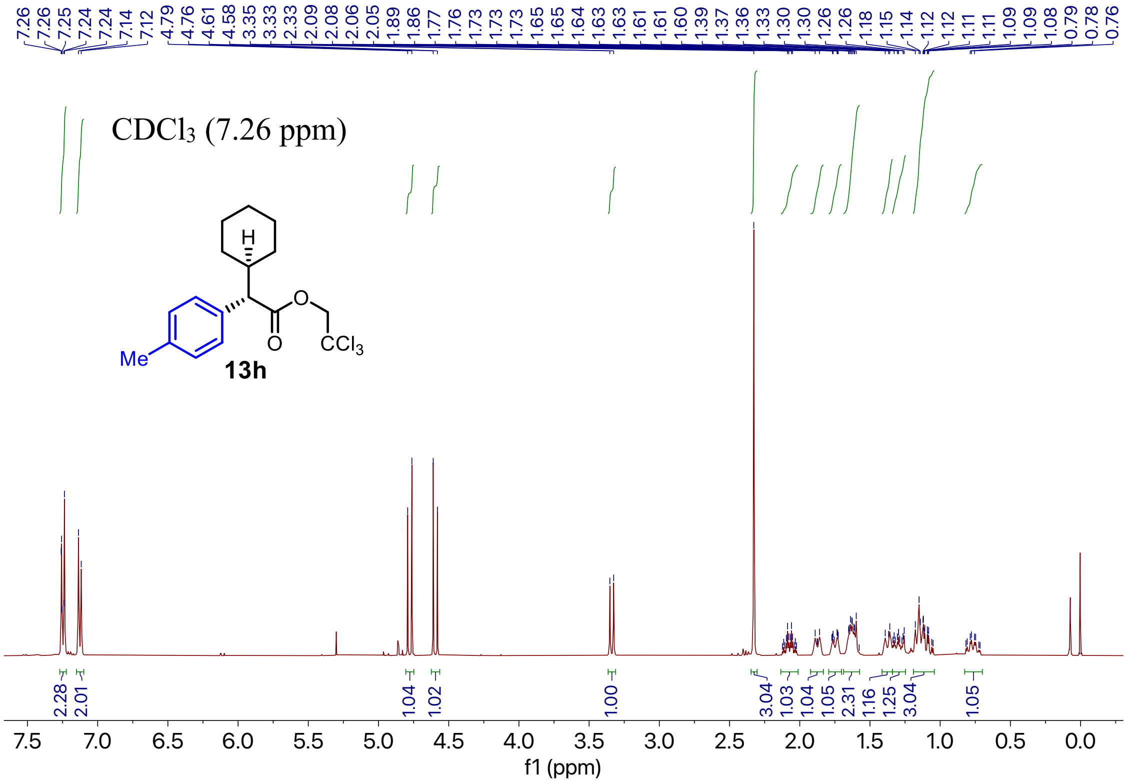
7.26
7.26
7.25
7.24
7.24
7.14
7.12
4.79
4.76
4.61
4.58
3.35
3.33
2.33
2.09
2.08
2.06
2.05
1.89
1.86
1.77
1.76
1.73
1.73
1.73
1.65
1.65
1.64
1.63
1.63
1.61
1.61
1.60
1.39
1.37
1.36
1.33
1.30
1.30
1.26
1.26
1.18
1.15
1.14
1.12
1.12
1.11
1.11
1.09
1.08
0.79
0.78
0.76
CDCl3 (7.26 ppm)
H
O
O
CCl3
Me
13h
2.28
2.01
1.04
1.02
1.00
3.04
1.03
1.04
1.05
2.31
1.16
1.25
3.04
1.05
7.5 7.0 6.5 6.0 5.5 5.0 4.5 4.0 3.5 3.0 2.5 2.0 1.5 1.0 0.5 0.0
f1 (ppm)

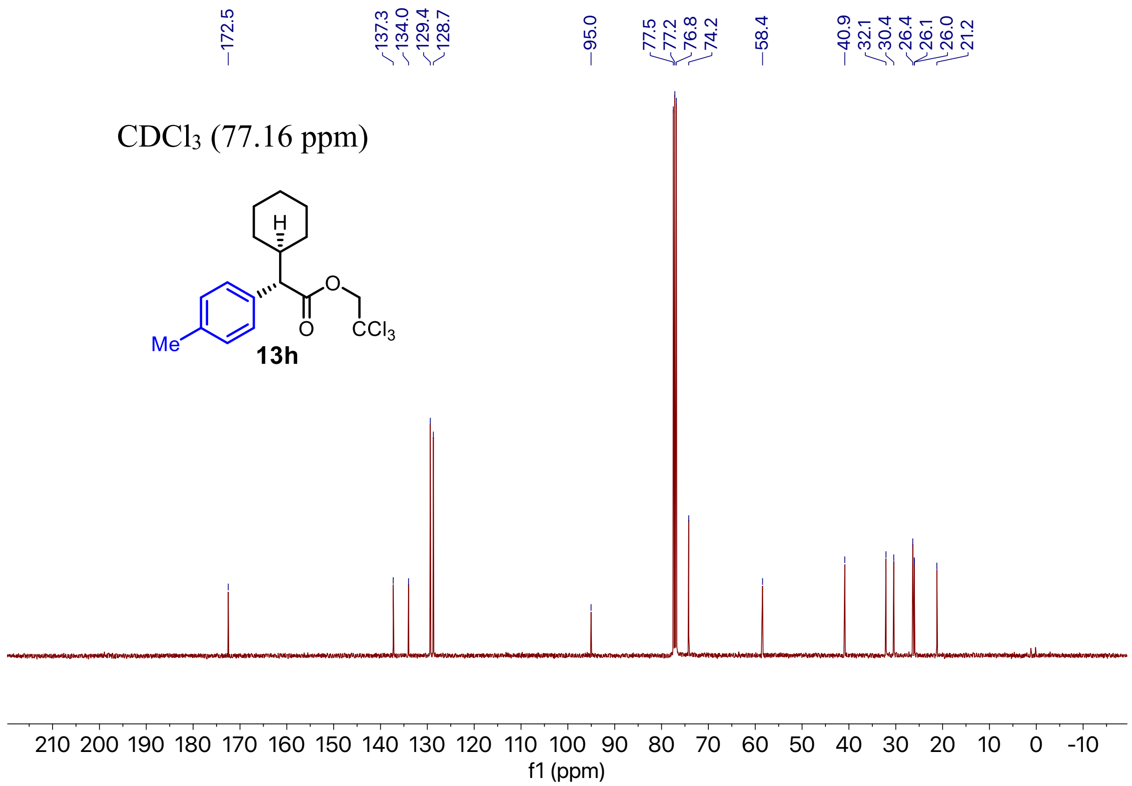

172.5
137.3
134.0
129.4
128.7
95.0
77.5
77.2
76.8
74.2
58.4
40.9
32.1
30.4
26.4
26.1
26.0
21.2
CDCl3 (77.16 ppm)
H
O
O
CCl3
Me
13h
210 200 190 180 170 160 150 140 130 120 110 100 90 80 70 60 50 40 30 20 10 0 -10
f1 (ppm)

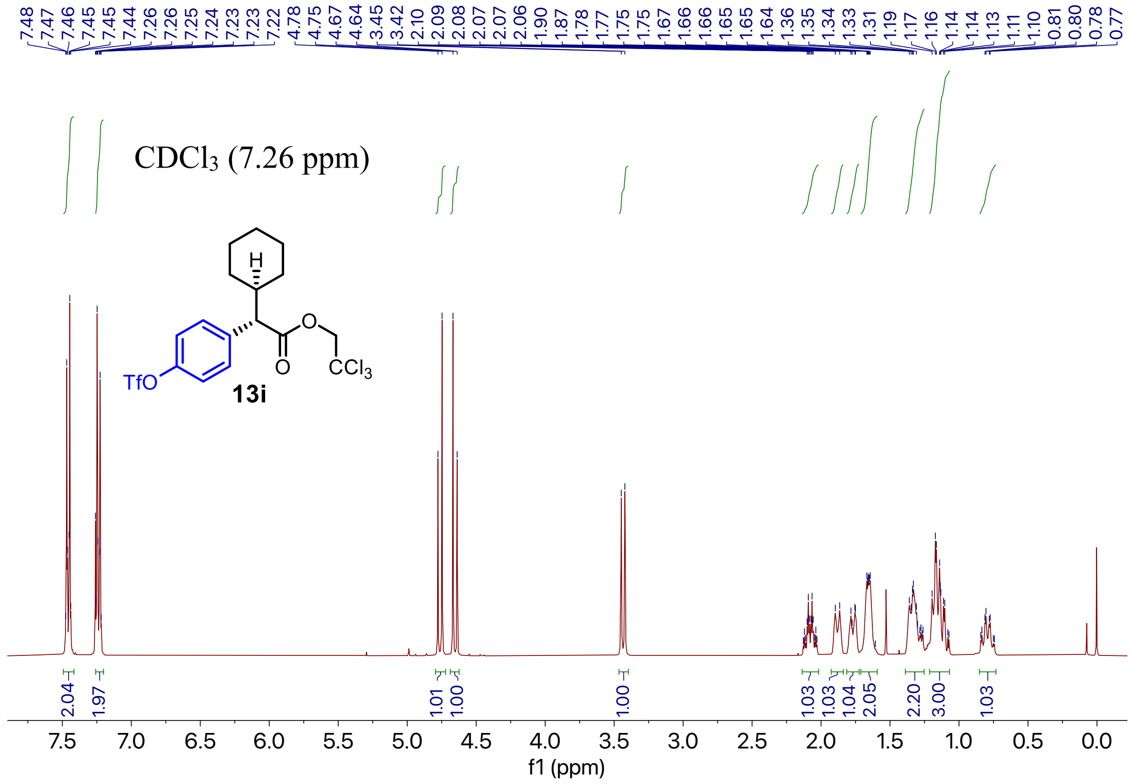

7.48
7.47
7.46
7.45
7.45
7.44
7.26
7.26
7.25
7.24
7.23
7.23
7.22
4.78
4.75
4.67
4.64
3.45
3.42
2.10
2.09
2.08
2.07
2.07
2.06
1.90
1.87
1.78
1.77
1.75
1.75
1.67
1.66
1.66
1.65
1.65
1.64
1.36
1.35
1.34
1.33
1.31
1.19
1.17
1.16
1.14
1.14
1.13
1.11
1.10
0.81
0.80
0.78
0.77
CDCl3 (7.26 ppm)
H
O
O
CCl3
TfO
13i
2.04
1.97
1.01
1.00
1.00
1.03
1.03
1.04
2.05
2.20
3.00
1.03
7.5 7.0 6.5 6.0 5.5 5.0 4.5 4.0 3.5 3.0 2.5 2.0 1.5 1.0 0.5 0.0
f1 (ppm)

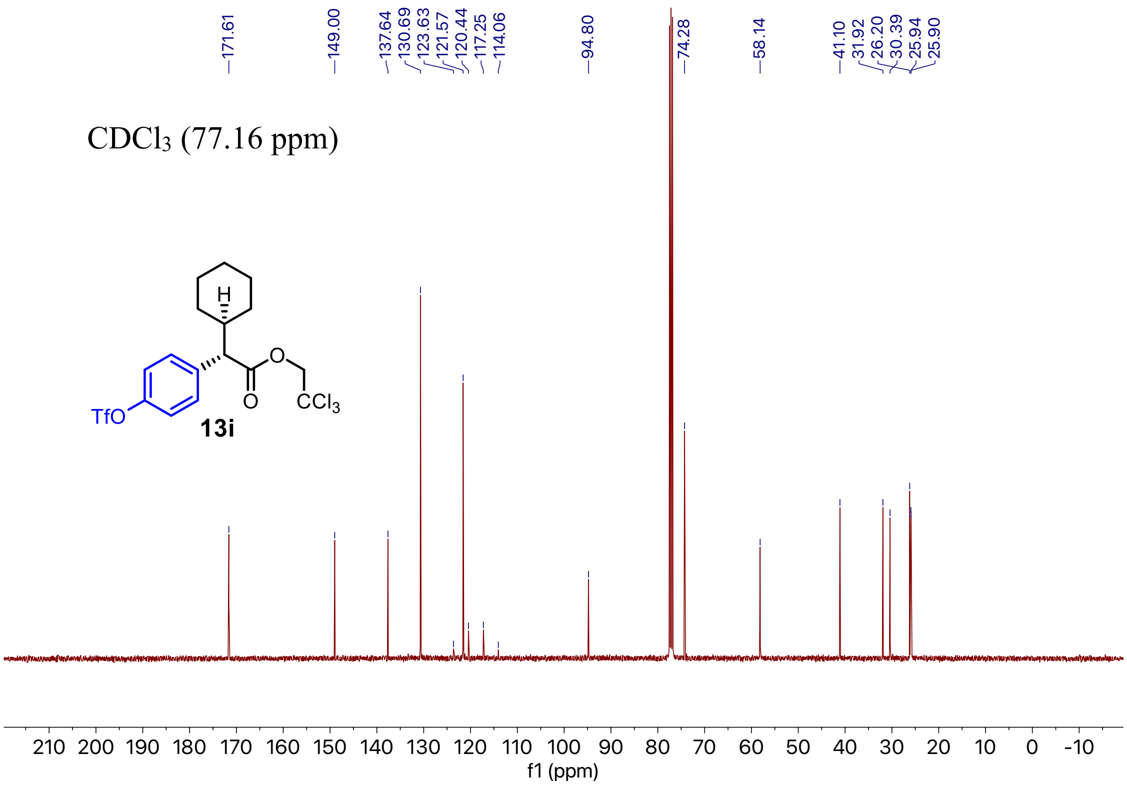

171.61
149.00
137.64
130.69
123.63
121.57
120.44
117.25
114.06
94.80
74.28
58.14
41.10
31.92
26.20
30.39
25.94
25.90
CDCl3 (77.16 ppm)
TfO
O
O
CCl3
H
13i
210 200 190 180 170 160 150 140 130 120 110 100 90 80 70 60 50 40 30 20 10 0 -10
f1 (ppm)

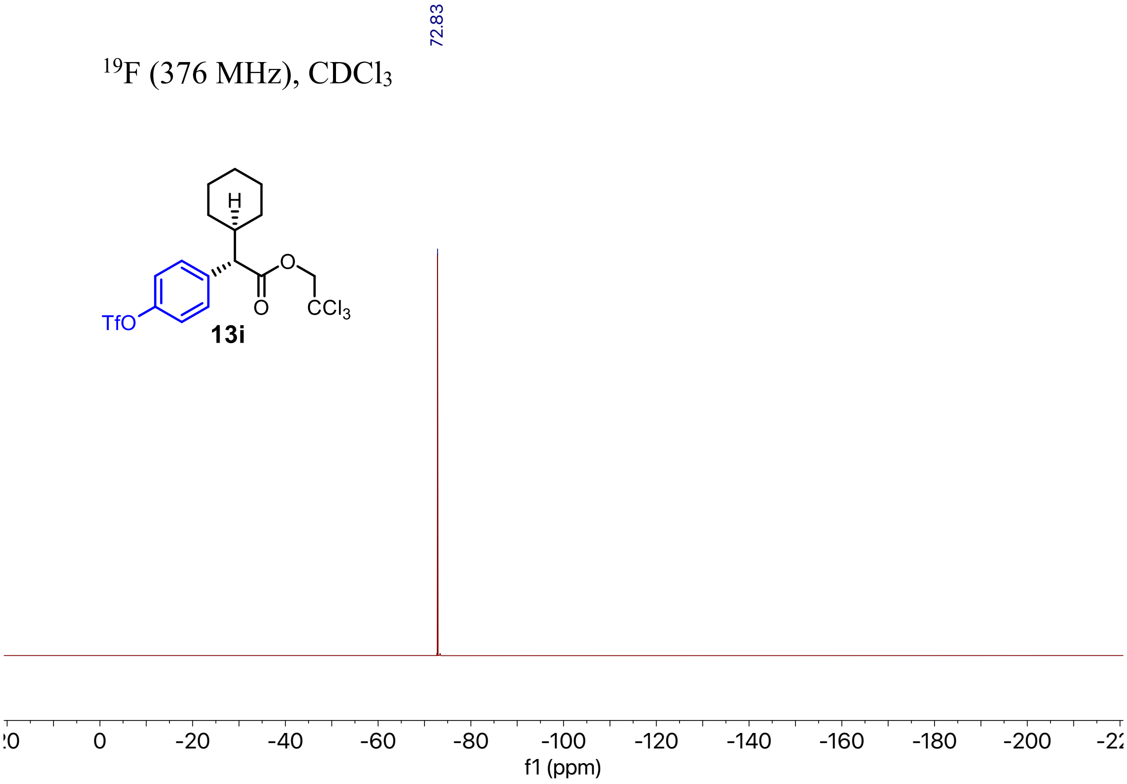

72.83
19F (376 MHz), CDCl3
TfO
O
O
CCl3
H
13i
0 -20 -40 -60 -80 -100 -120 -140 -160 -180 -200
f1 (ppm)

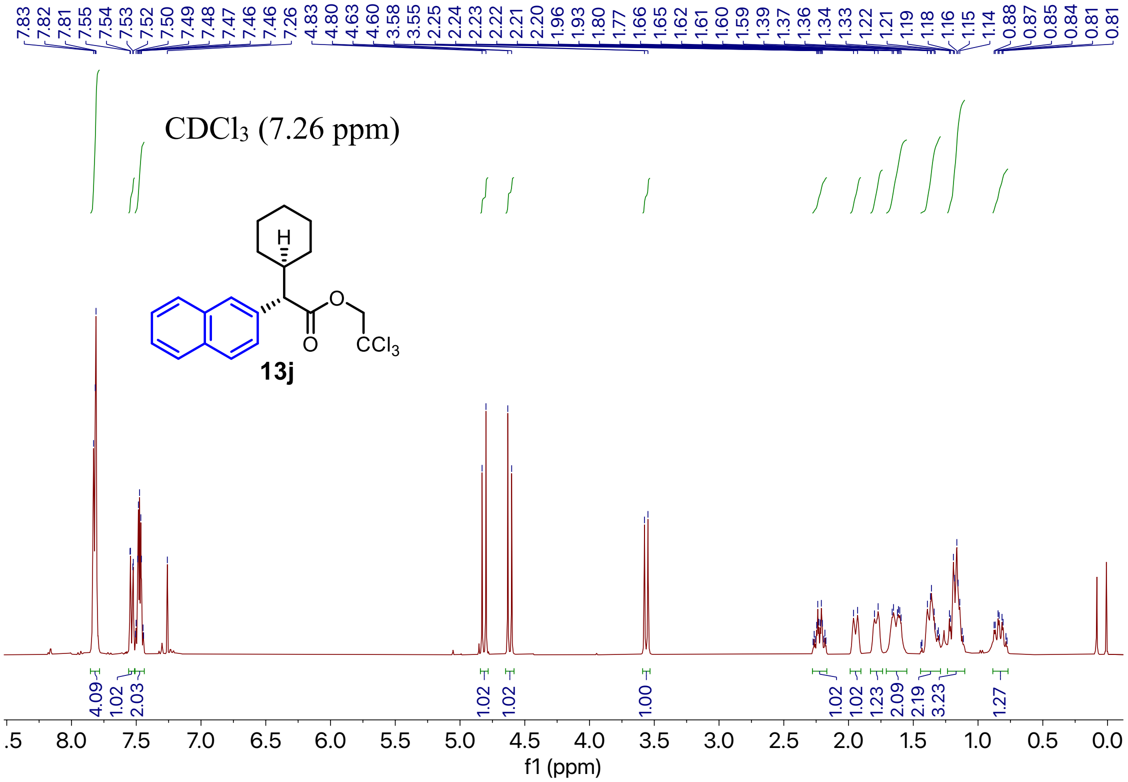
7.83 7.82 7.81 7.55 7.54 7.53 7.52 7.50 7.49 7.48 7.47 7.46 7.26 4.83 4.80 4.63 4.60 3.58 3.55 2.25 2.24 2.23 2.22 2.21 2.20 1.96 1.93 1.80 1.77 1.66 1.65 1.62 1.61 1.60 1.59 1.39 1.37 1.36 1.34 1.33 1.22 1.21 1.19 1.18 1.16 1.15 1.14 0.88 0.87 0.85 0.84 0.81 0.81
CDCl3 (7.26 ppm)
H
O
O
CCl3
13j
4.09 1.02 2.03 1.02 1.02 1.00 1.02 1.02 1.23 2.09 2.19 3.23 1.27
f1 (ppm)

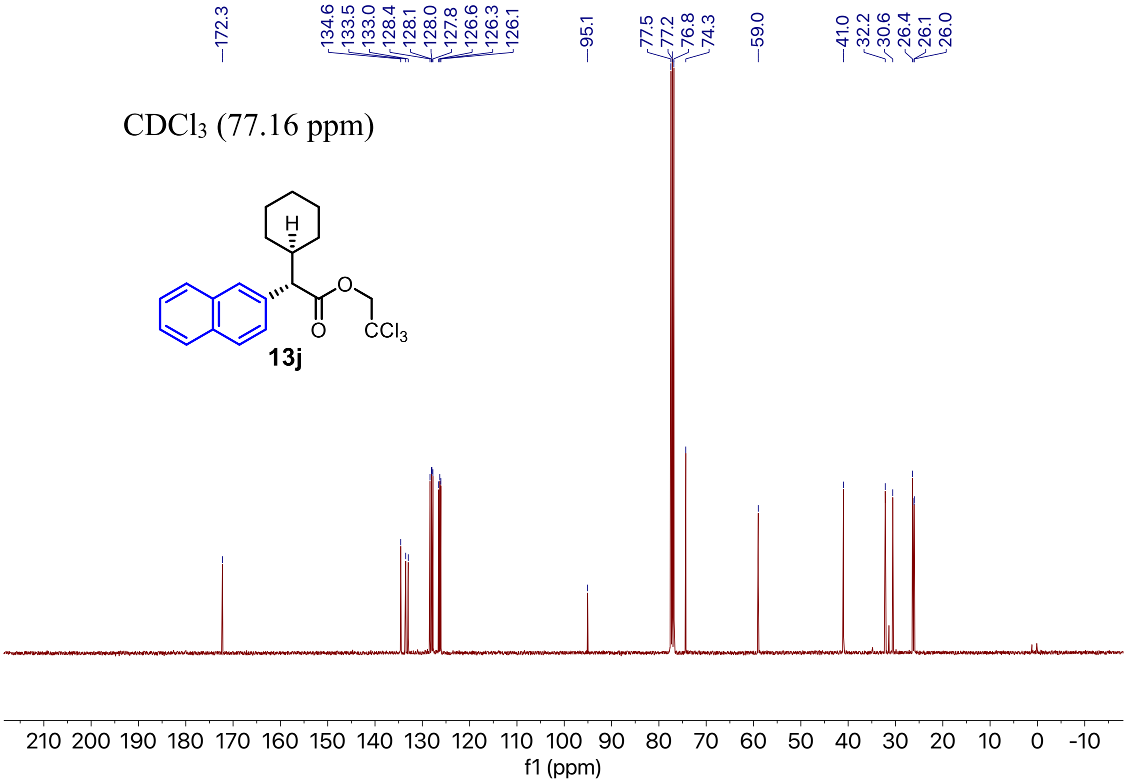
172.3
134.6 133.5 133.0 128.4 128.1 128.0 127.8 126.6 126.3 126.1
95.1
77.5 77.2 76.8 74.3
59.0
41.0 32.2 30.6 26.4 26.1 26.0
CDCl3 (77.16 ppm)
H
O
O
CCl3
13j
f1 (ppm)

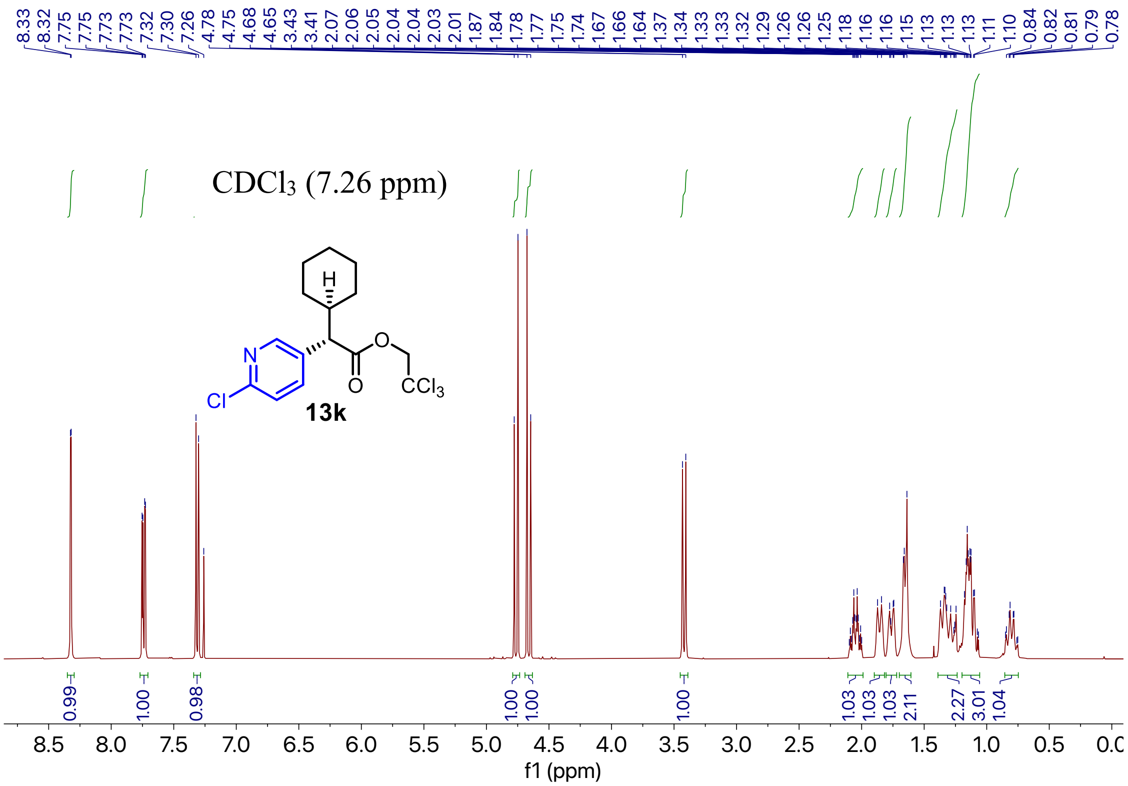
CDCl3 (7.26 ppm)
13k
f1 (ppm)

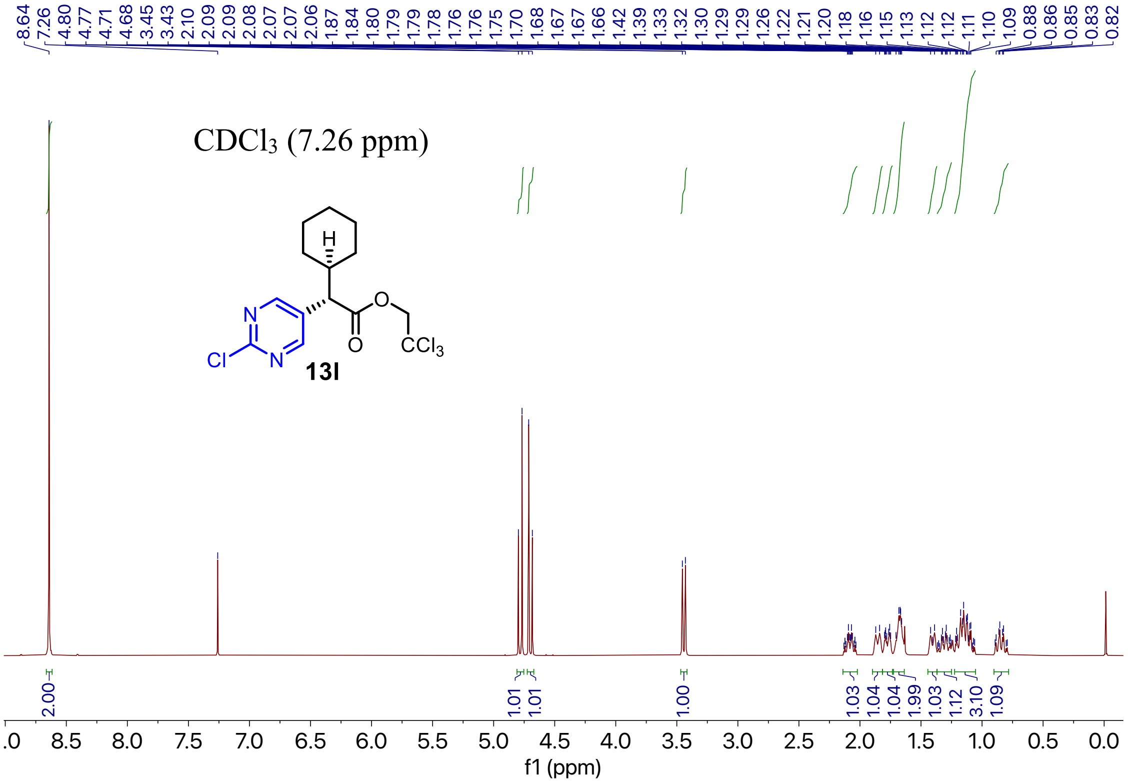
CDCl3 (7.26 ppm)
13l
f1 (ppm)

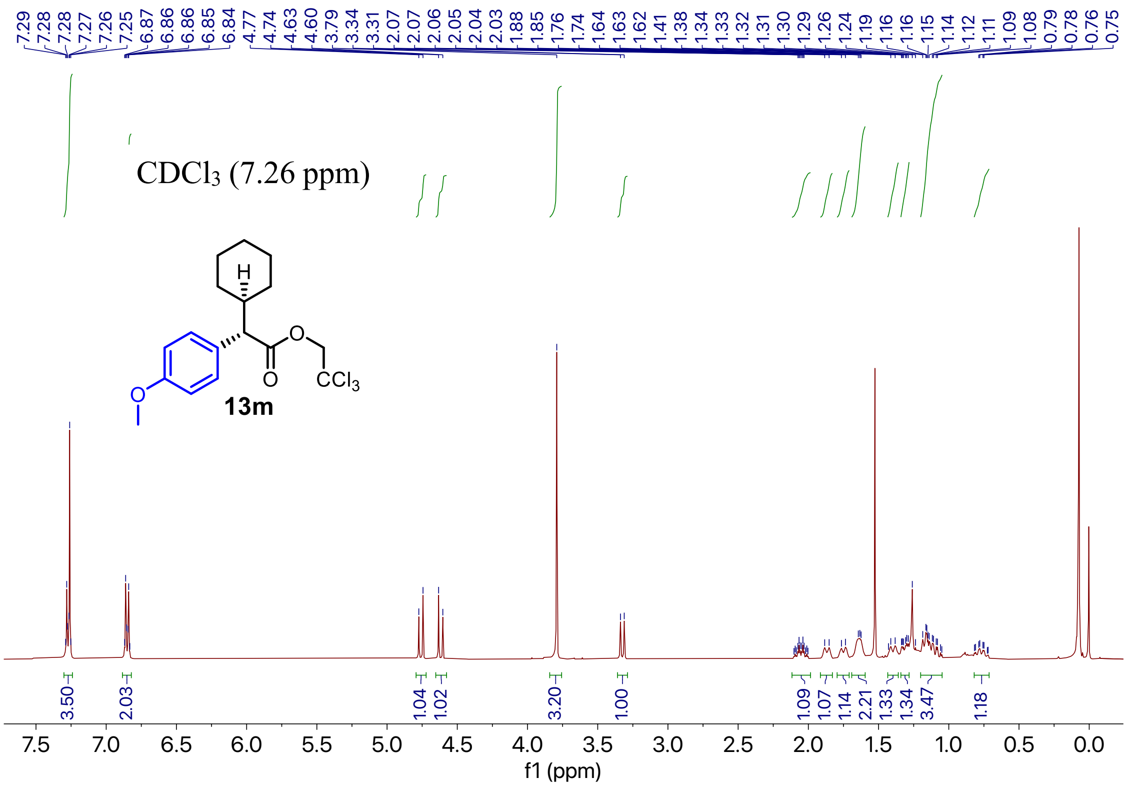
CDCl3 (7.26 ppm)
13m
f1 (ppm)

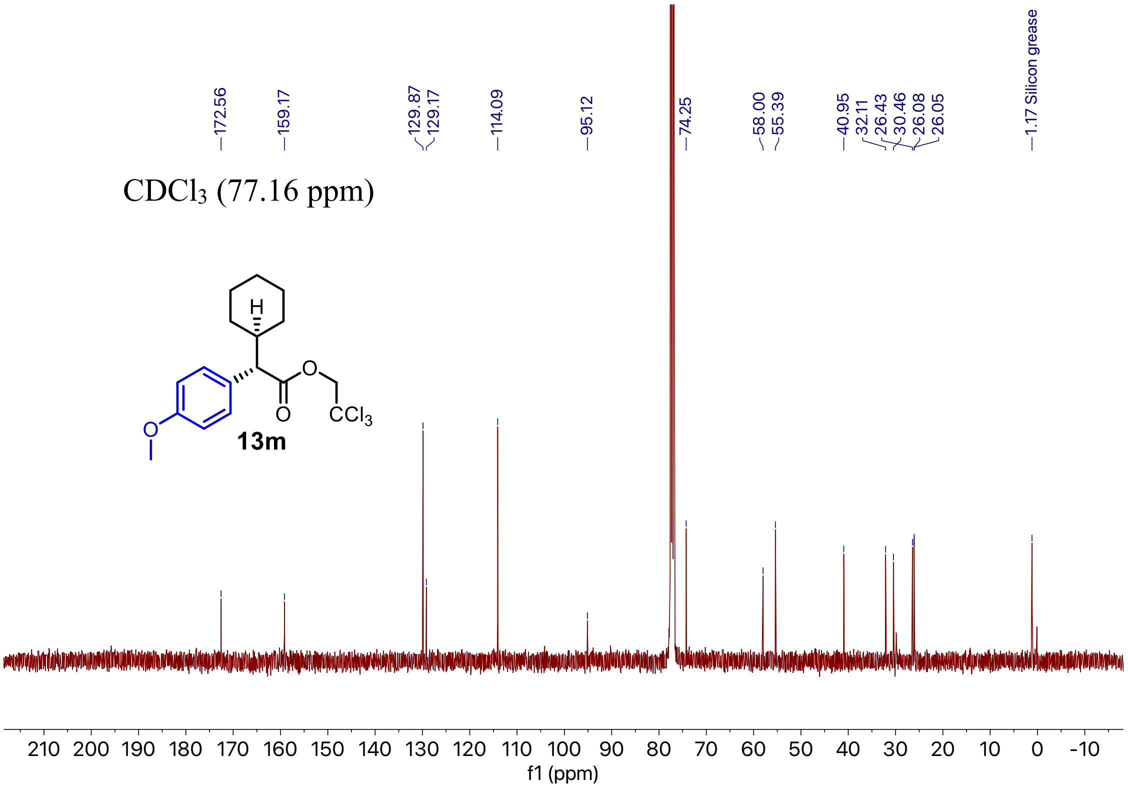
CDCl3 (77.16 ppm)
13m
1.17 Silicon grease
f1 (ppm)

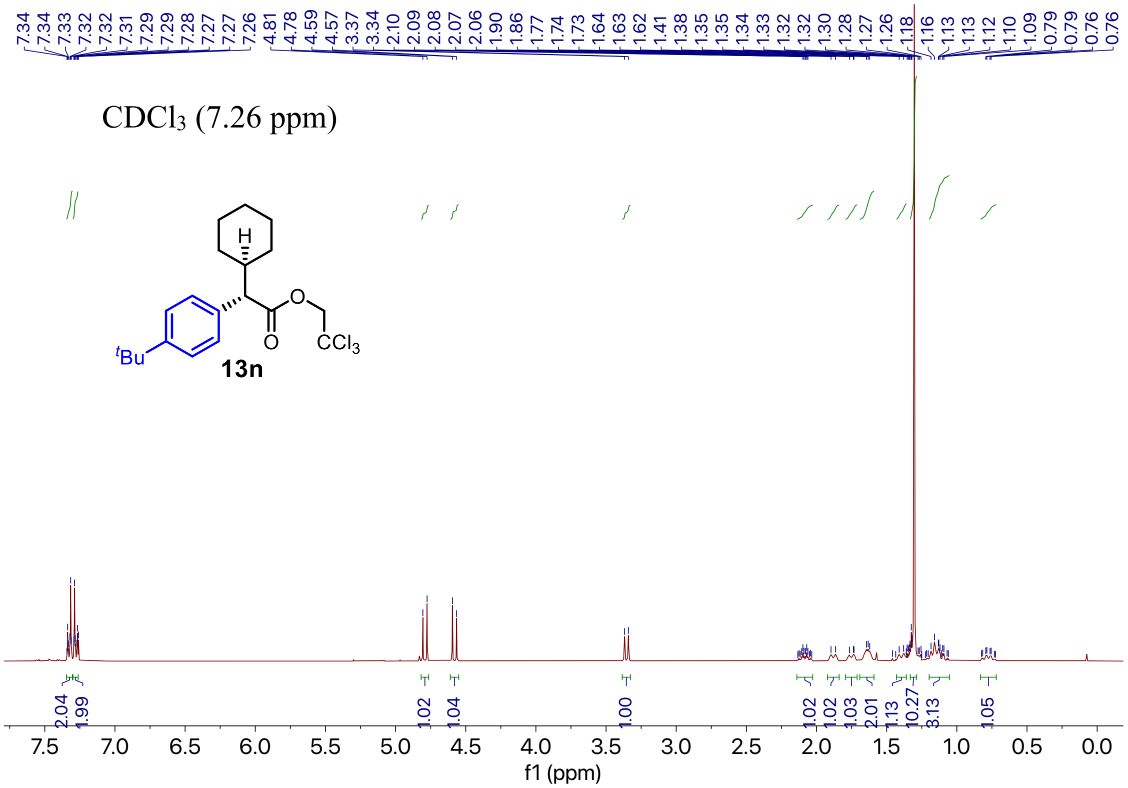
CDCl3 (7.26 ppm)
tBu
CCl3
13n
f1 (ppm)

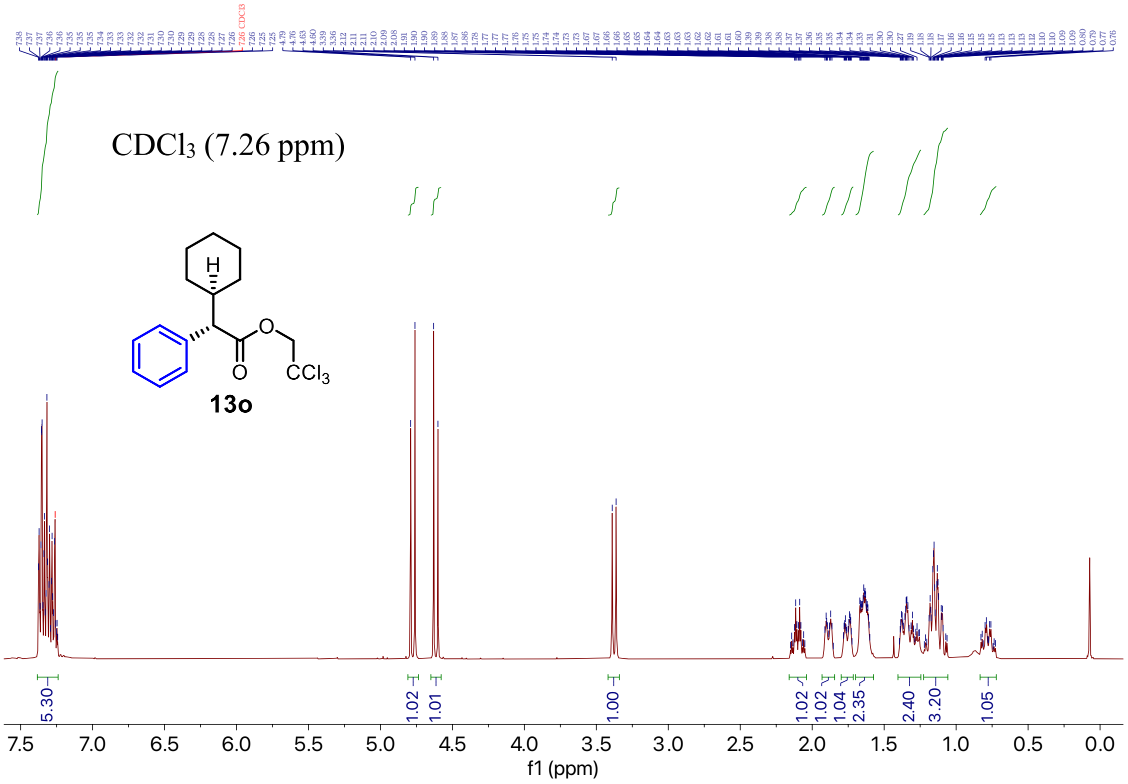
CDCl3 (7.26 ppm)
CCl3
13o
f1 (ppm)

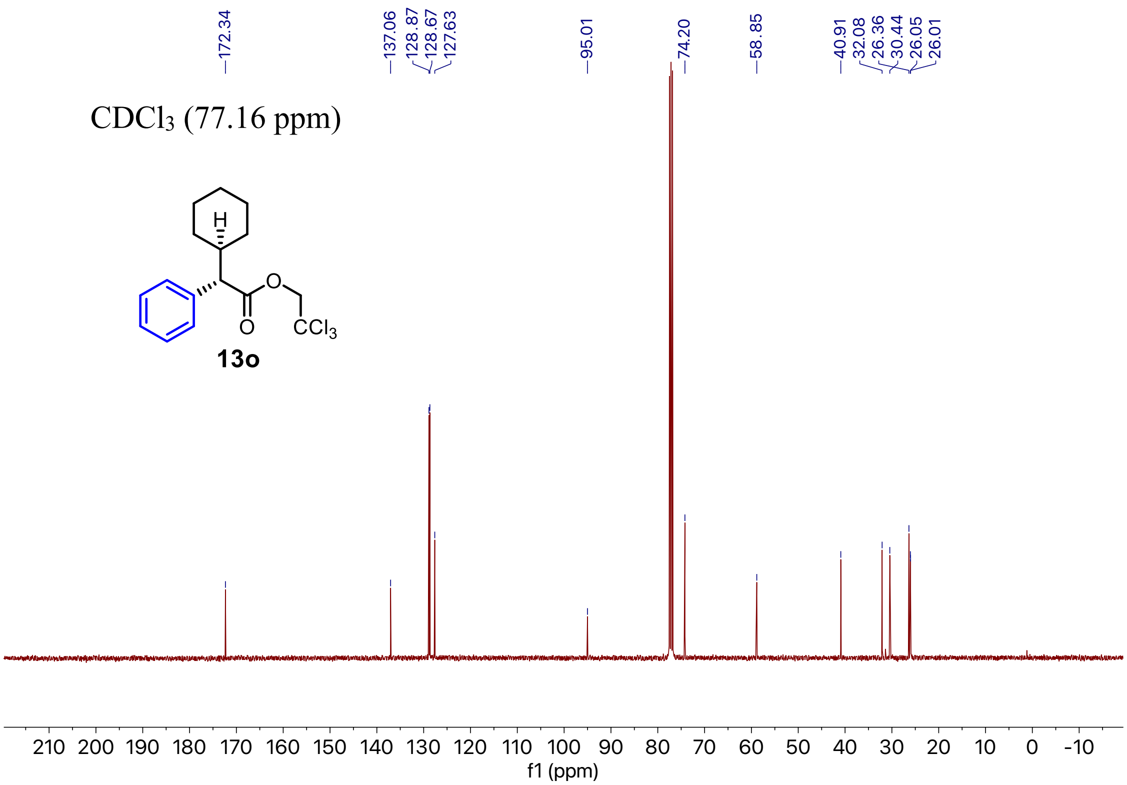
172.34
137.06
128.87
128.67
127.63
95.01
74.20
58.85
40.91
32.08
26.36
30.44
26.05
26.01
CDCl3 (77.16 ppm)
H
O
O
CCl3
13o
210 200 190 180 170 160 150 140 130 120 110 100 90 80 70 60 50 40 30 20 10 0 -10
f1 (ppm)

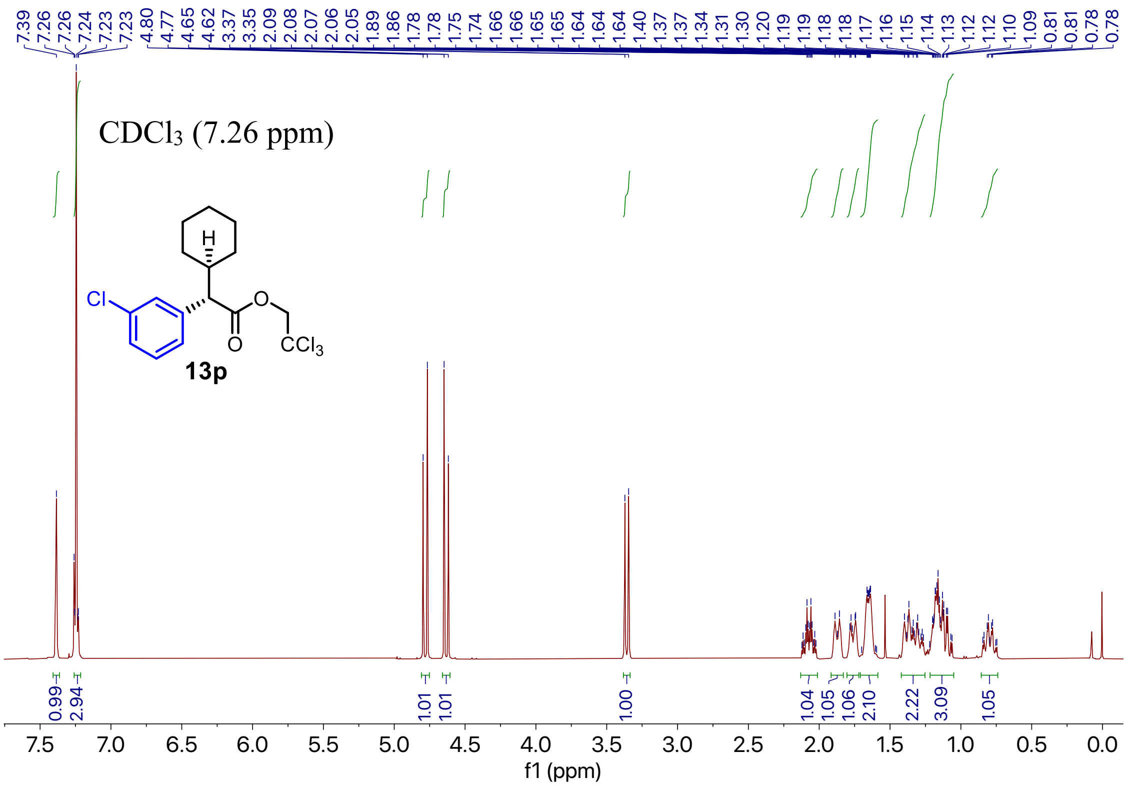
7.39
7.26
7.26
7.24
7.23
7.23
4.80
4.77
4.65
4.62
3.37
3.35
2.09
2.08
2.07
2.06
2.05
1.89
1.86
1.78
1.78
1.75
1.74
1.66
1.66
1.65
1.65
1.64
1.64
1.64
1.40
1.37
1.37
1.34
1.31
1.30
1.20
1.19
1.19
1.18
1.18
1.17
1.16
1.15
1.14
1.13
1.12
1.12
1.10
1.09
0.81
0.81
0.78
0.78
CDCl3 (7.26 ppm)
H
Cl
O
O
CCl3
13p
0.99
2.94
1.01
1.01
1.00
1.04
1.05
1.06
2.10
2.22
3.09
1.05
7.5 7.0 6.5 6.0 5.5 5.0 4.5 4.0 3.5 3.0 2.5 2.0 1.5 1.0 0.5 0.0
f1 (ppm)

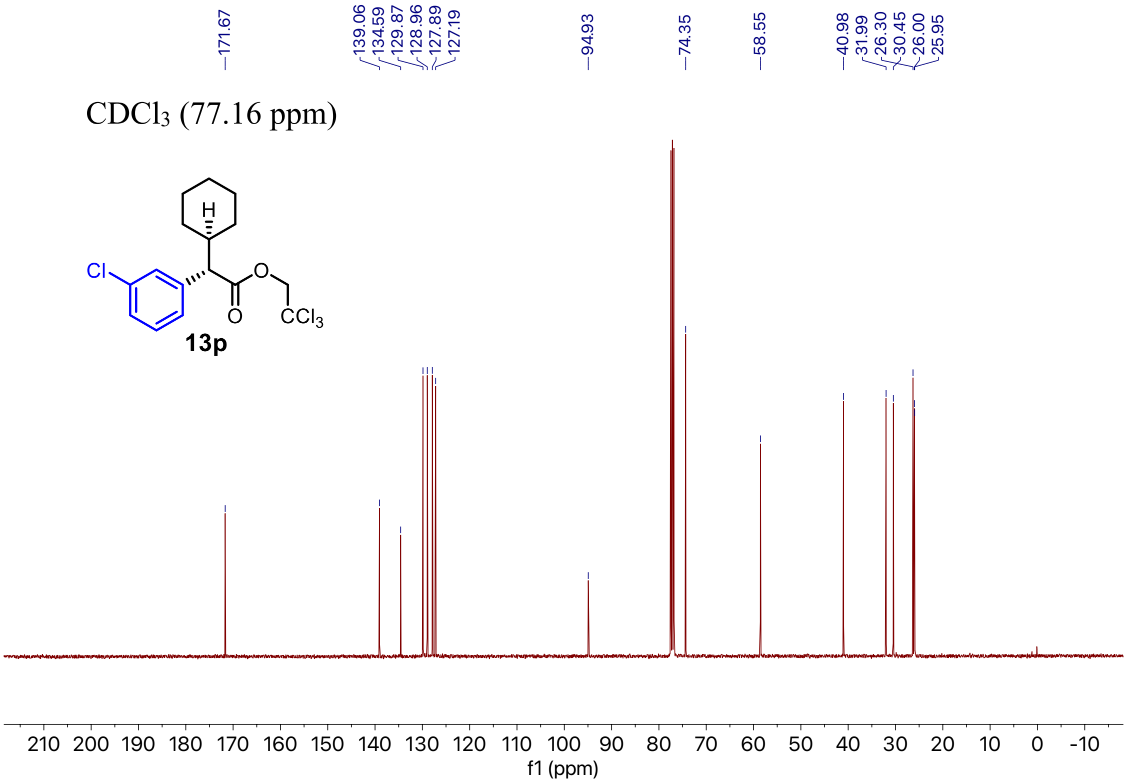
171.67
139.06
134.59
129.87
128.96
127.89
127.19
94.93
74.35
58.55
40.98
31.99
26.30
30.45
26.00
25.95
CDCl3 (77.16 ppm)
H
Cl
O
O
CCl3
13p
210 200 190 180 170 160 150 140 130 120 110 100 90 80 70 60 50 40 30 20 10 0 -10
f1 (ppm)

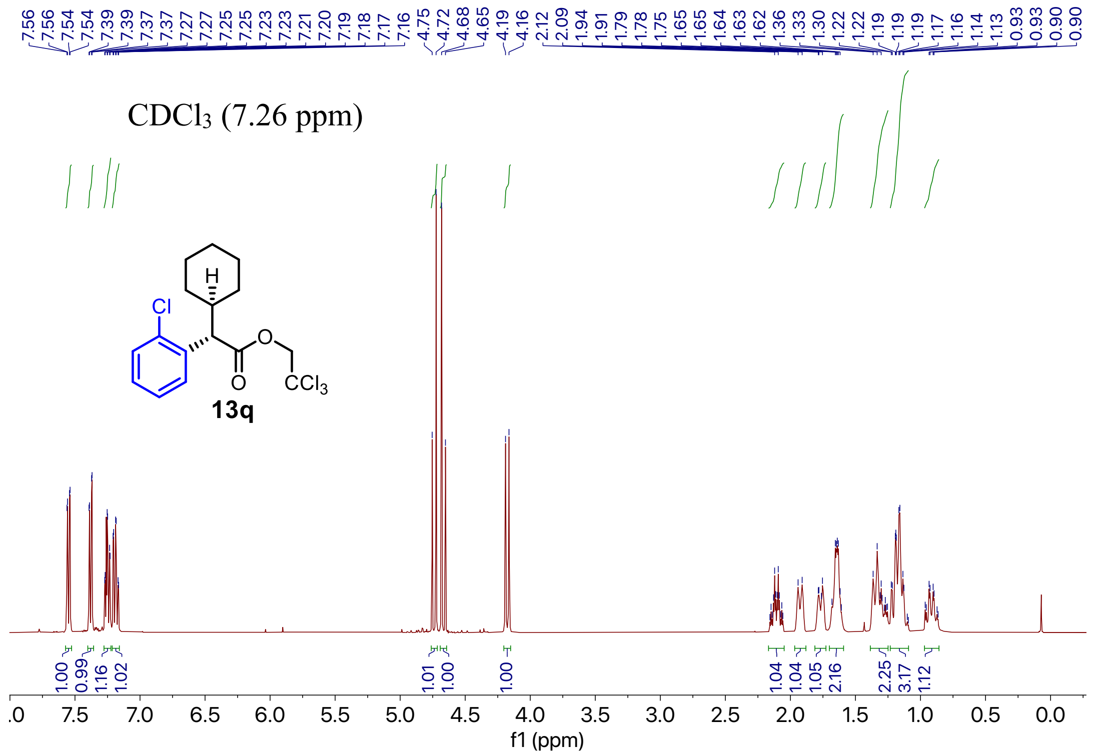
CDCl3 (7.26 ppm)
13q
f1 (ppm)

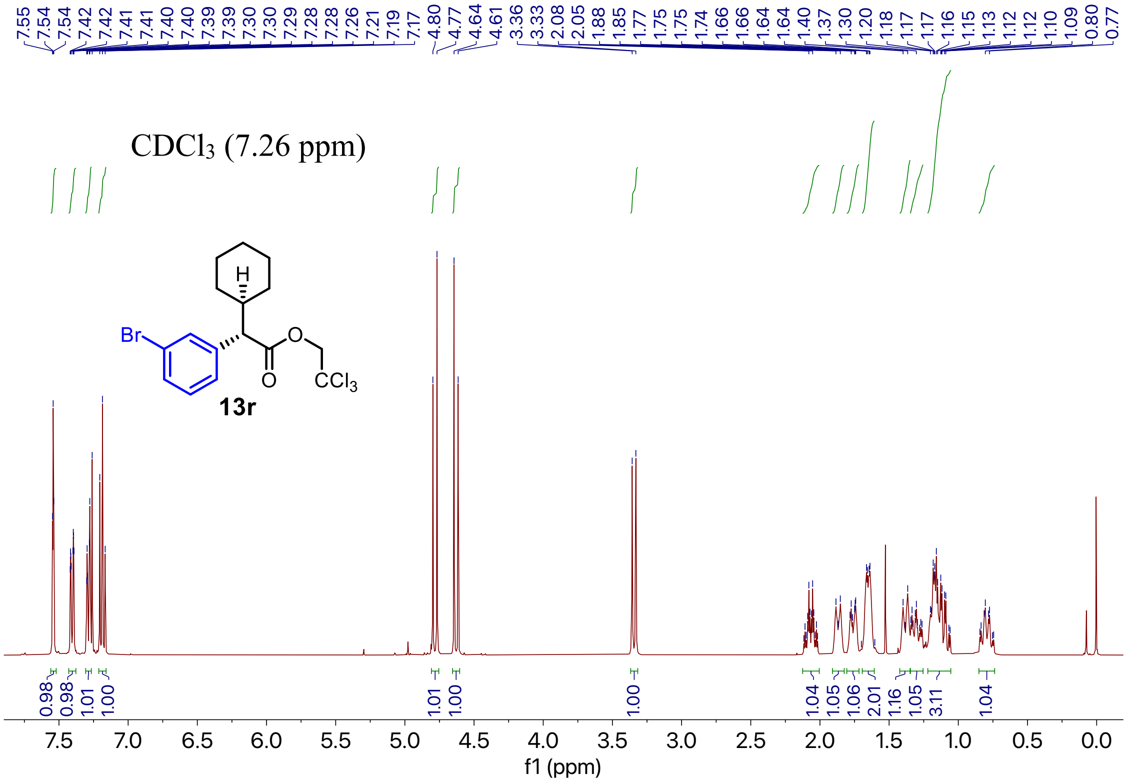
CDCl3 (7.26 ppm)
13r
f1 (ppm)

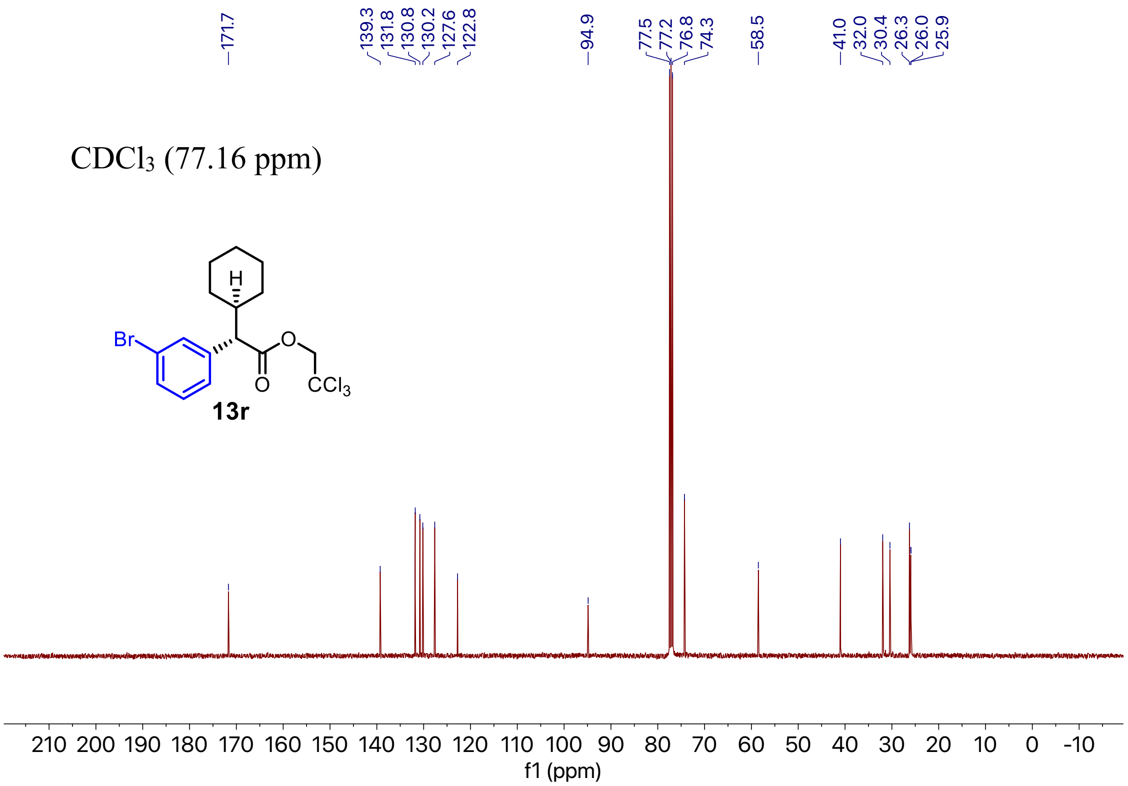
171.7
139.3
131.8
130.8
130.2
127.6
122.8
94.9
77.5
77.2
76.8
74.3
58.5
41.0
32.0
30.4
26.3
26.0
25.9
CDCl3 (77.16 ppm)
Br
O
O
CCl3
f1 (ppm)

13r

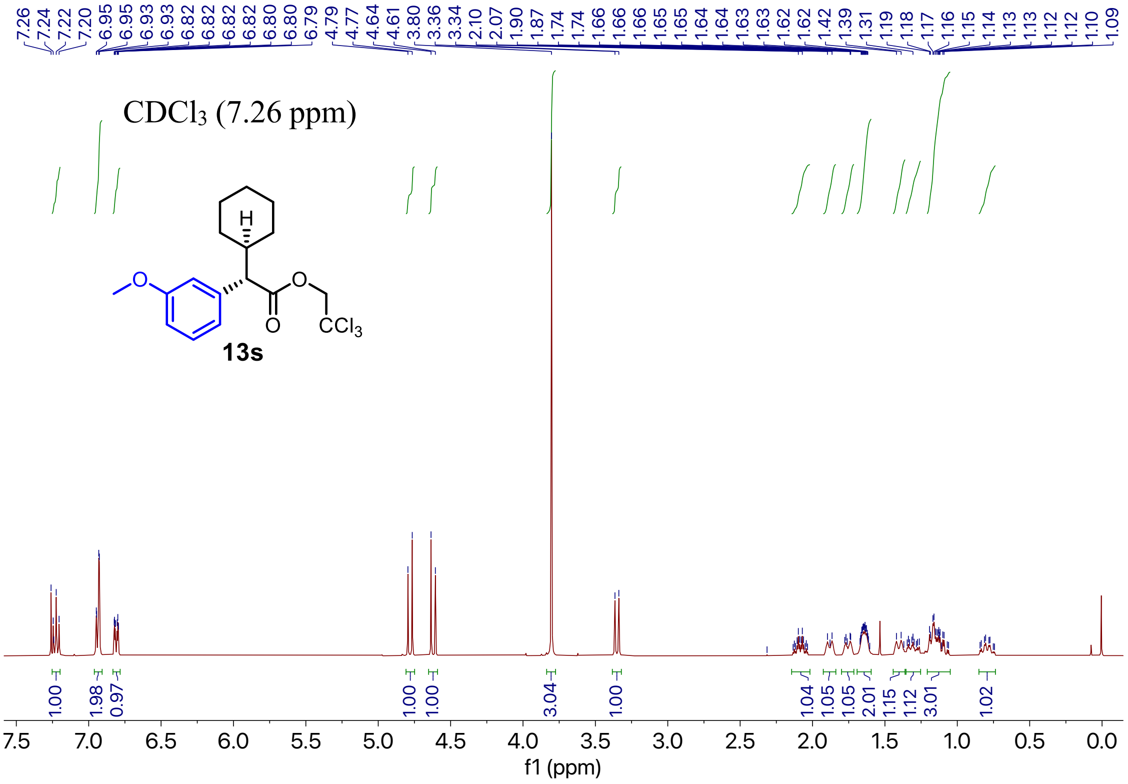
7.26
7.24
7.22
7.20
6.95
6.95
6.93
6.93
6.82
6.82
6.82
6.82
6.80
6.80
6.79
4.79
4.77
4.64
4.61
3.80
3.36
3.34
2.10
2.07
1.90
1.87
1.74
1.74
1.66
1.66
1.65
1.65
1.64
1.64
1.63
1.63
1.62
1.62
1.42
1.39
1.31
1.19
1.18
1.17
1.16
1.15
1.14
1.13
1.13
1.12
1.12
1.10
1.09
CDCl3 (7.26 ppm)
O
O
O
CCl3
1.00
1.98
0.97
1.00
1.00
3.04
1.00
1.04
1.05
1.05
2.01
1.15
1.12
3.01
1.02
f1 (ppm)

13s

CDCl3 (77.16 ppm)

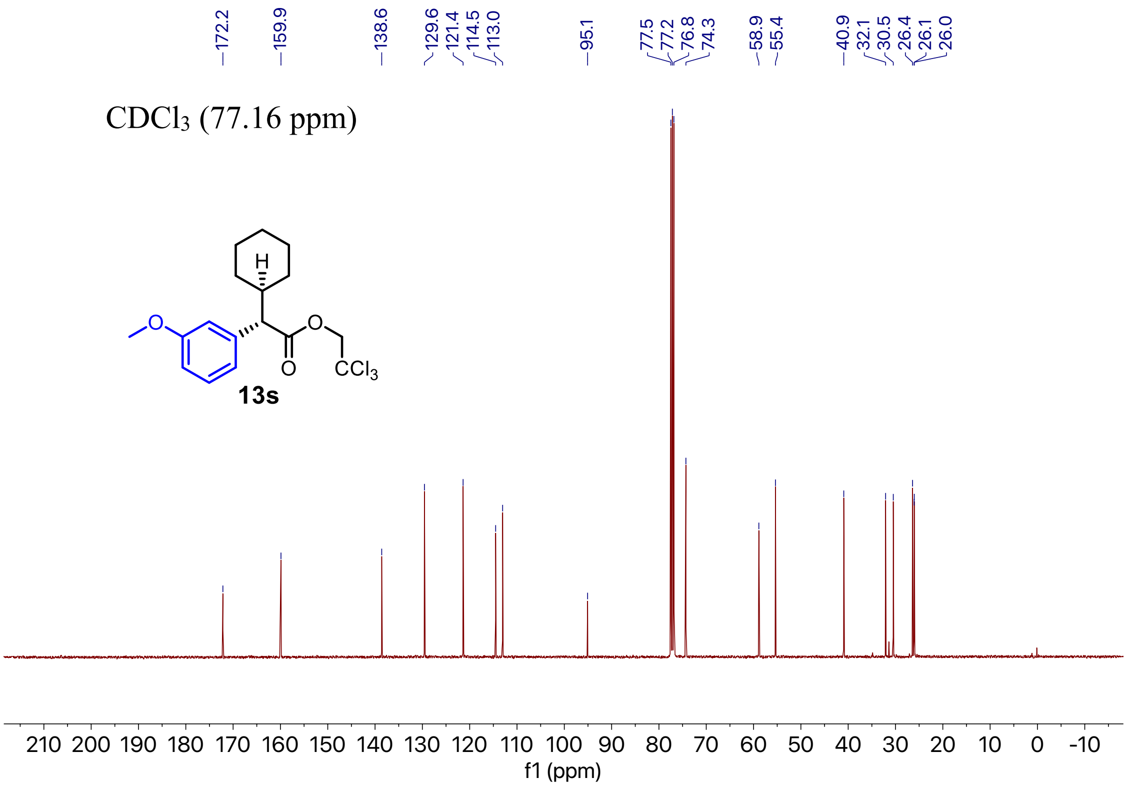

CDCl3 (7.26 ppm)

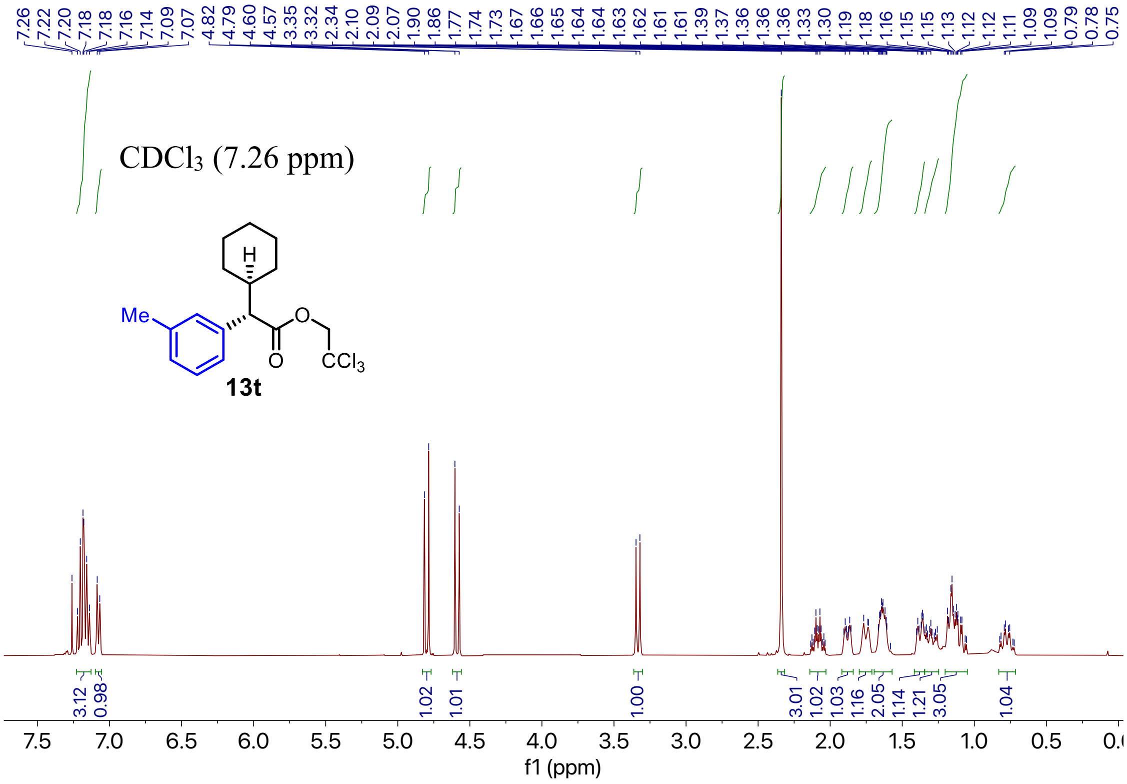

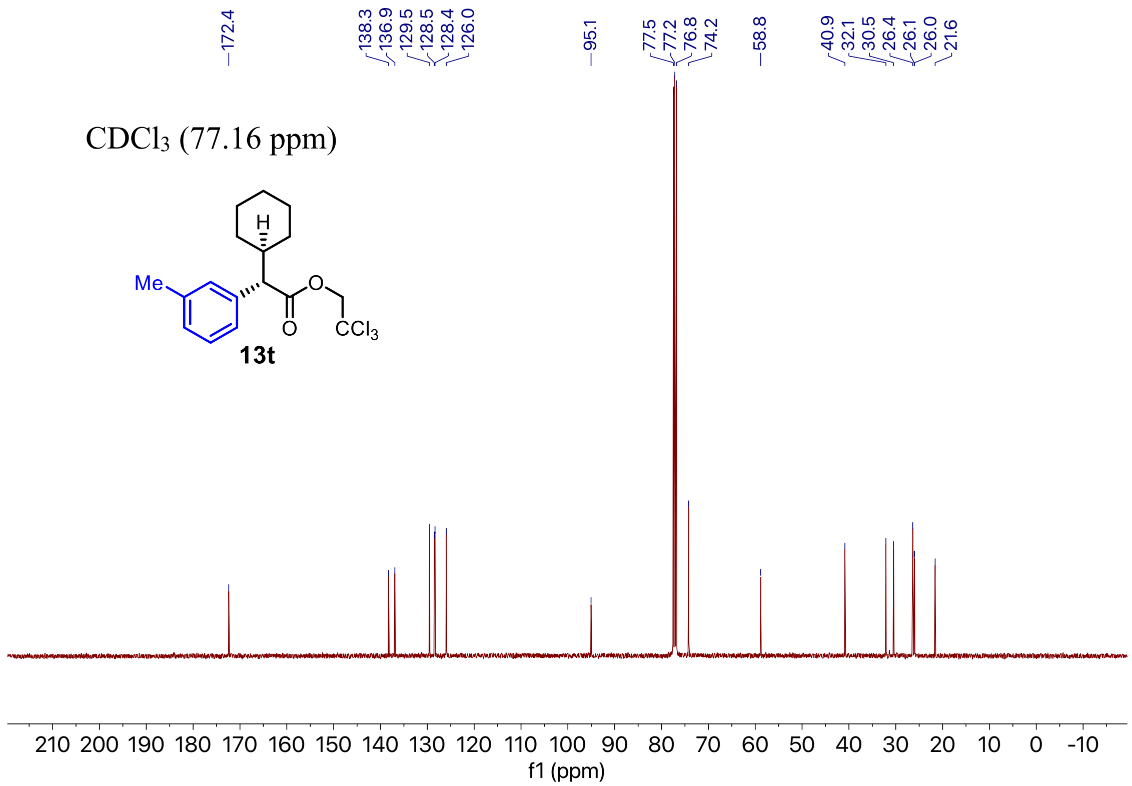

172.4
138.3
136.9
129.5
128.5
128.4
126.0
95.1
77.5
77.2
76.8
74.2
58.8
40.9
32.1
30.5
26.4
26.1
26.0
21.6
CDCl3 (77.16 ppm)
Me
O
O
CCl3
H
13t
210 200 190 180 170 160 150 140 130 120 110 100 90 80 70 60 50 40 30 20 10 0 -10
f1 (ppm)

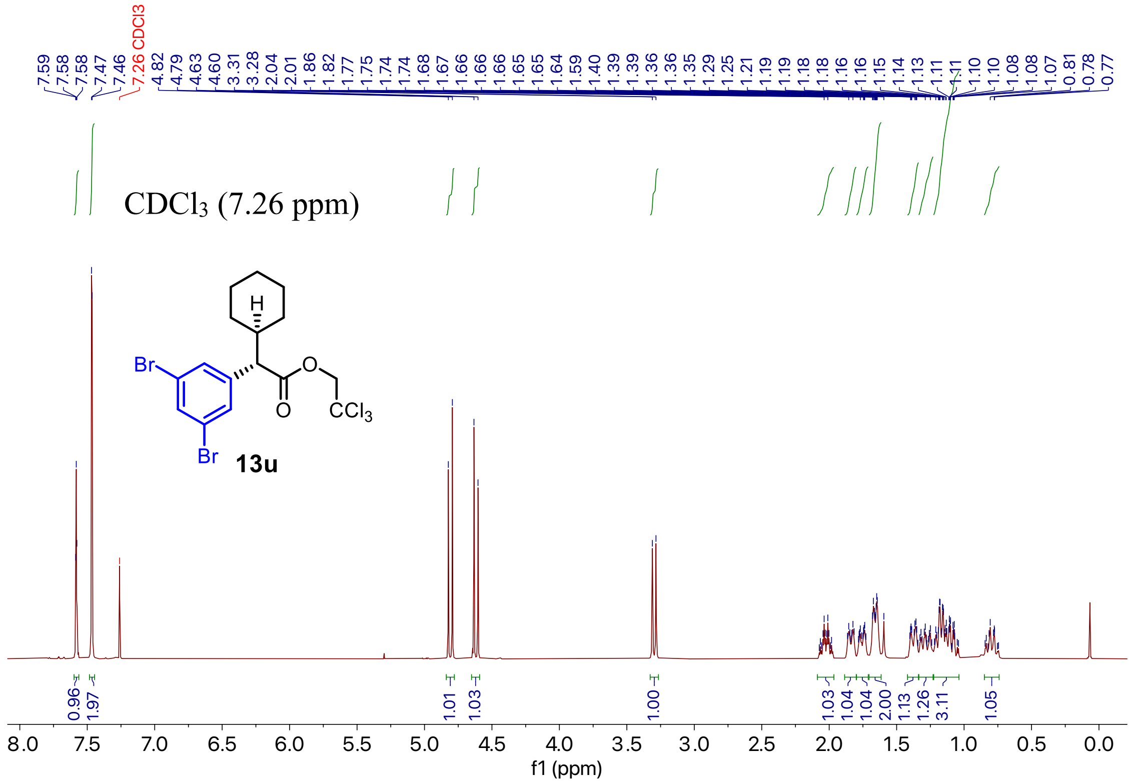

7.59
7.58
7.58
7.47
7.46
7.26 CDCl3
4.82
4.79
4.63
4.60
3.31
3.28
2.04
2.01
1.86
1.82
1.77
1.75
1.74
1.68
1.67
1.66
1.66
1.65
1.65
1.64
1.59
1.40
1.39
1.36
1.36
1.35
1.29
1.25
1.21
1.19
1.19
1.18
1.18
1.16
1.16
1.15
1.14
1.13
1.11
1.11
1.10
1.10
1.08
1.08
1.07
0.81
0.78
0.77
CDCl3 (7.26 ppm)
Br
Br
O
O
CCl3
H
13u
0.96
1.97
1.01
1.03
1.00
1.03
1.04
1.04
2.00
1.13
1.26
3.11
1.05
8.0 7.5 7.0 6.5 6.0 5.5 5.0 4.5 4.0 3.5 3.0 2.5 2.0 1.5 1.0 0.5 0.0
f1 (ppm)

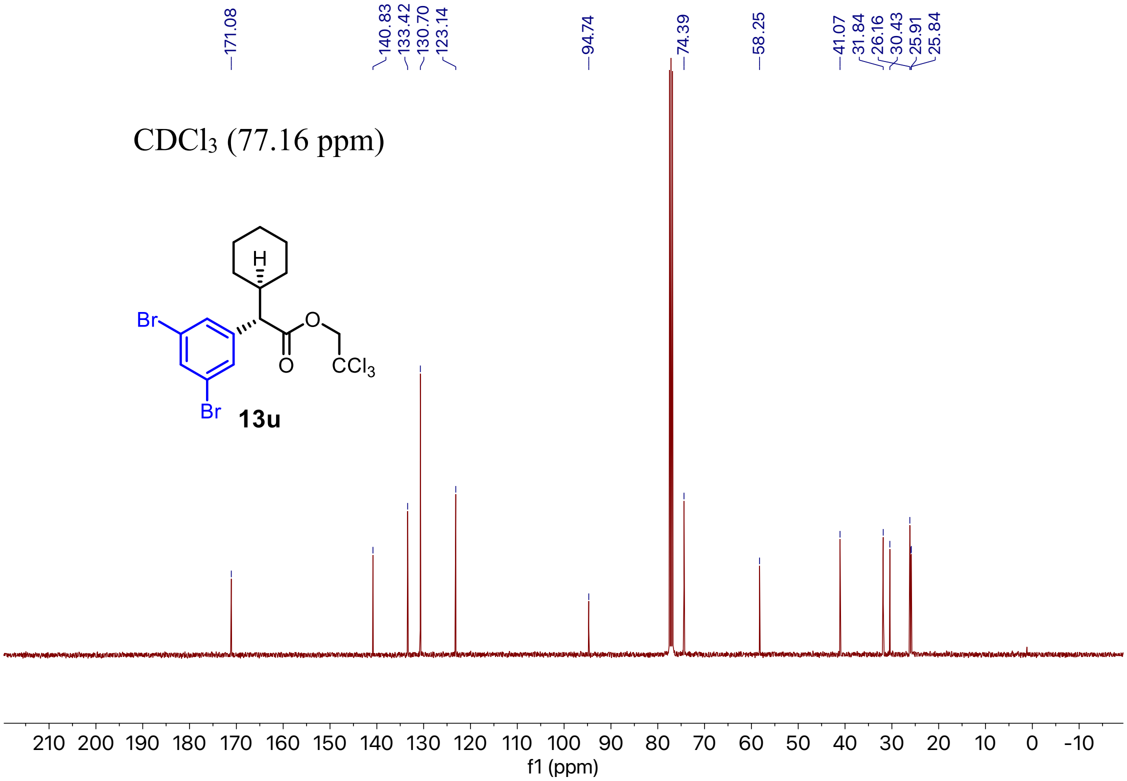
171.08
140.83
133.42
130.70
123.14
94.74
74.39
58.25
41.07
31.84
26.16
30.43
25.91
25.84
CDCl3 (77.16 ppm)
H
Br
Br
O
O
CCl3
13u
210 200 190 180 170 160 150 140 130 120 110 100 90 80 70 60 50 40 30 20 10 0 -10
f1 (ppm)

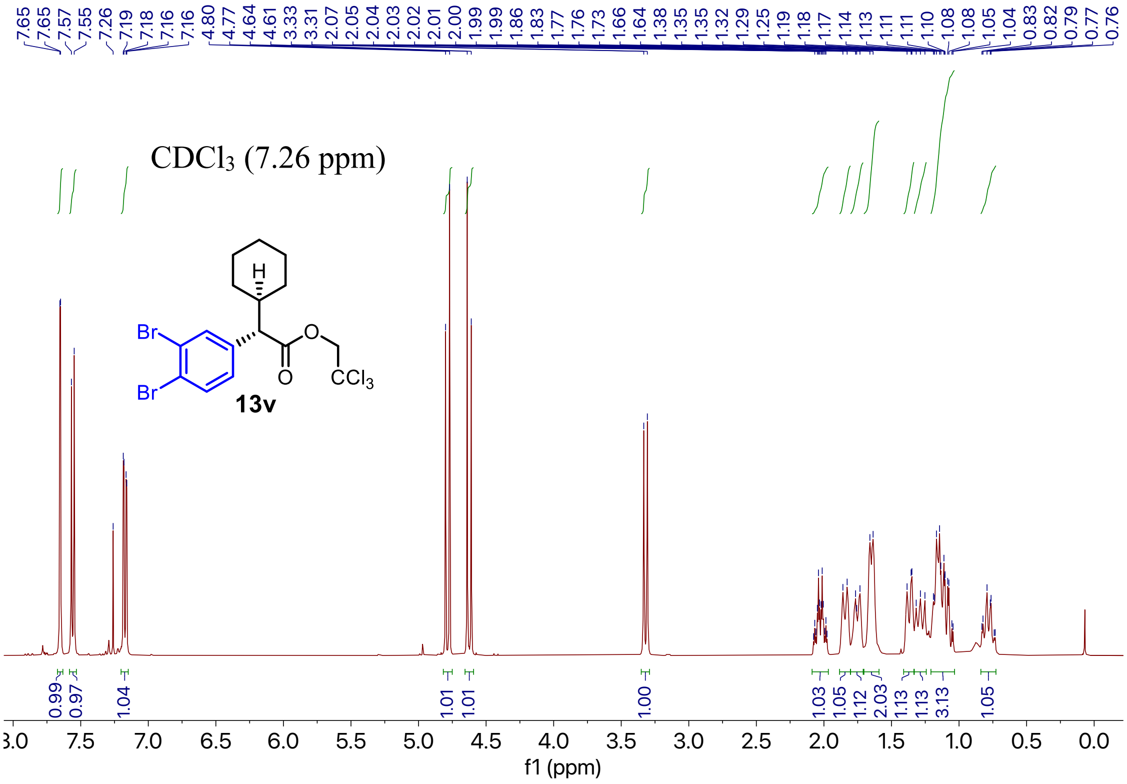
7.65 7.65 7.57 7.55 7.26 7.19 7.18 7.16 7.16 4.80 4.77 4.64 4.61 3.33 3.31 2.07 2.05 2.04 2.03 2.02 2.01 2.00 1.99 1.99 1.86 1.83 1.77 1.76 1.73 1.66 1.64 1.38 1.35 1.35 1.32 1.29 1.25 1.19 1.18 1.17 1.14 1.13 1.11 1.11 1.10 1.08 1.08 1.05 1.04 0.83 0.82 0.79 0.77 0.76
CDCl3 (7.26 ppm)
H
Br
Br
O
O
CCl3
13v
0.99 0.97 1.04 1.01 1.01 1.00 1.03 1.05 1.12 2.03 1.13 1.13 3.13 1.05
8.0 7.5 7.0 6.5 6.0 5.5 5.0 4.5 4.0 3.5 3.0 2.5 2.0 1.5 1.0 0.5 0.0
f1 (ppm)

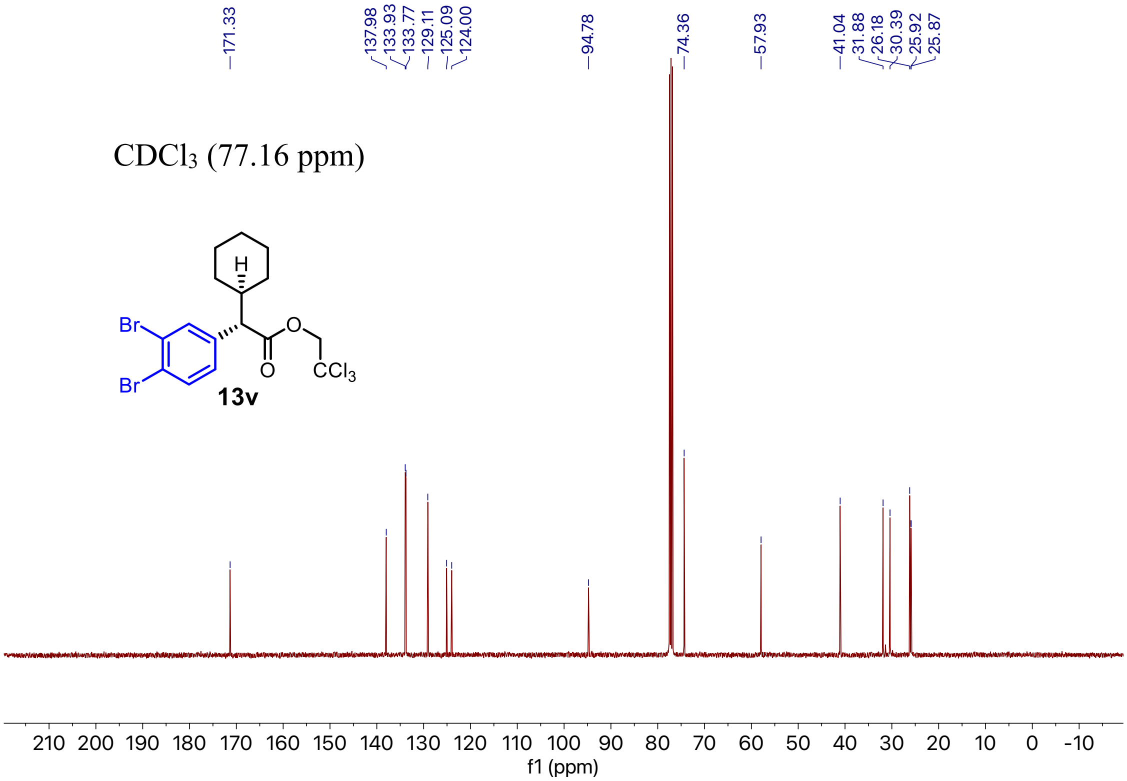
171.33
137.98
133.93
133.77
129.11
125.09
124.00
94.78
74.36
57.93
41.04
31.88
26.18
30.39
25.92
25.87
CDCl3 (77.16 ppm)
Br
Br
O
O
CCl3
H
13v
210 200 190 180 170 160 150 140 130 120 110 100 90 80 70 60 50 40 30 20 10 0 -10
f1 (ppm)

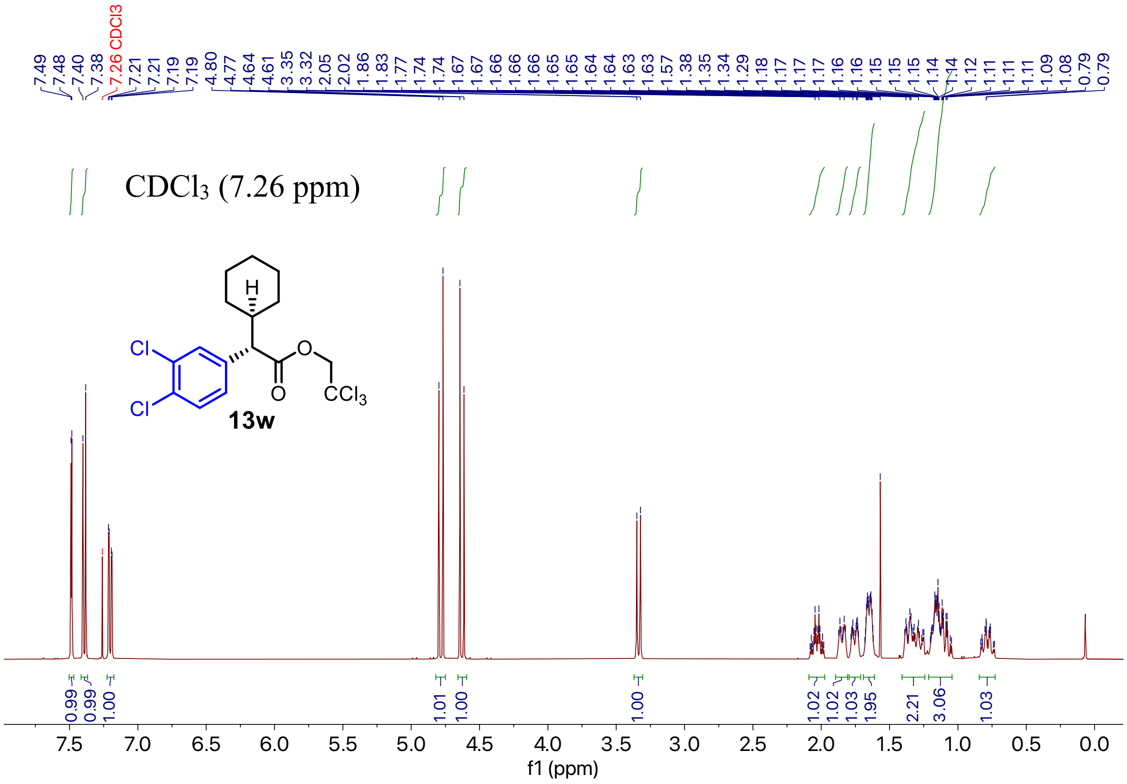
7.49
7.48
7.40
7.38
7.26 CDCl3
7.21
7.21
7.19
7.19
4.80
4.77
4.64
4.61
3.35
3.32
2.05
2.02
1.86
1.83
1.77
1.74
1.74
1.67
1.67
1.66
1.66
1.66
1.65
1.65
1.64
1.64
1.63
1.63
1.57
1.38
1.35
1.34
1.29
1.18
1.17
1.17
1.17
1.16
1.16
1.15
1.15
1.15
1.14
1.14
1.12
1.11
1.11
1.11
1.09
1.08
0.79
0.79
CDCl3 (7.26 ppm)
Cl
Cl
O
O
CCl3
H
13w
0.99
0.99
1.00
1.01
1.00
1.00
1.02
1.02
1.03
1.95
2.21
3.06
1.03
7.5 7.0 6.5 6.0 5.5 5.0 4.5 4.0 3.5 3.0 2.5 2.0 1.5 1.0 0.5 0.0
f1 (ppm)

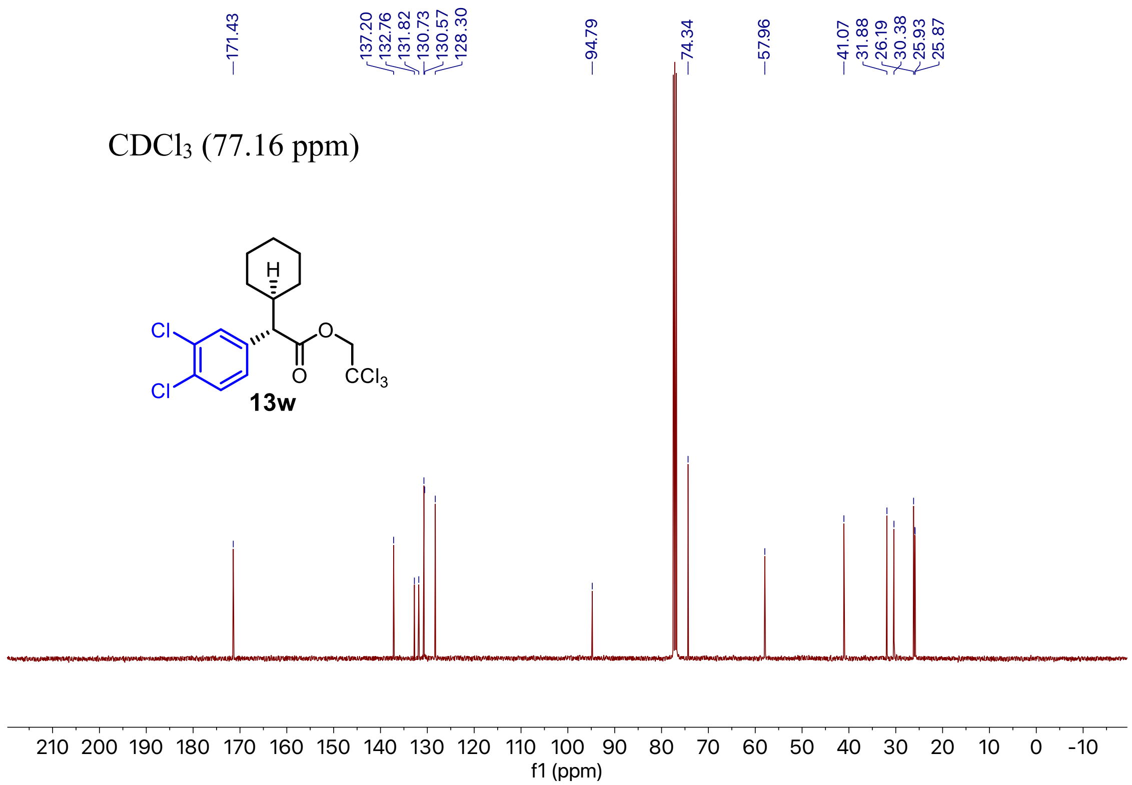

171.43
137.20
132.76
131.82
130.73
130.57
128.30
94.79
74.34
57.96
41.07
31.88
26.19
30.38
25.93
25.87
CDCl3 (77.16 ppm)
H
Cl
Cl
O
O
CCl3
13w
210 200 190 180 170 160 150 140 130 120 110 100 90 80 70 60 50 40 30 20 10 0 -10
f1 (ppm)

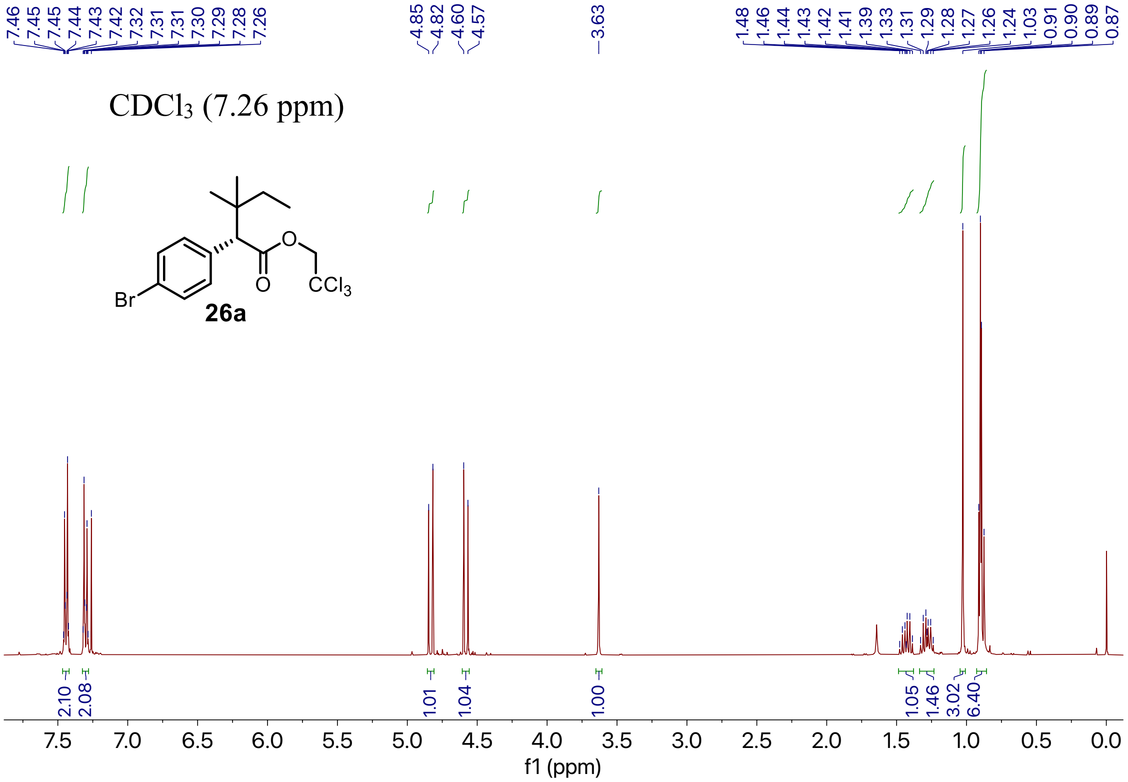
7.46 7.45 7.45 7.44 7.43 7.42 7.32 7.31 7.31 7.30 7.29 7.28 7.26
4.85 4.82 4.60 4.57
3.63
1.48 1.46 1.44 1.43 1.42 1.41 1.39 1.33 1.31 1.29 1.28 1.27 1.26 1.24 1.03 0.91 0.90 0.89 0.87
CDCl3 (7.26 ppm)
Br
O
O
CCl3
26a
2.10 2.08 1.01 1.04 1.00 1.05 1.46 3.02 6.40
7.5 7.0 6.5 6.0 5.5 5.0 4.5 4.0 3.5 3.0 2.5 2.0 1.5 1.0 0.5 0.0
f1 (ppm)

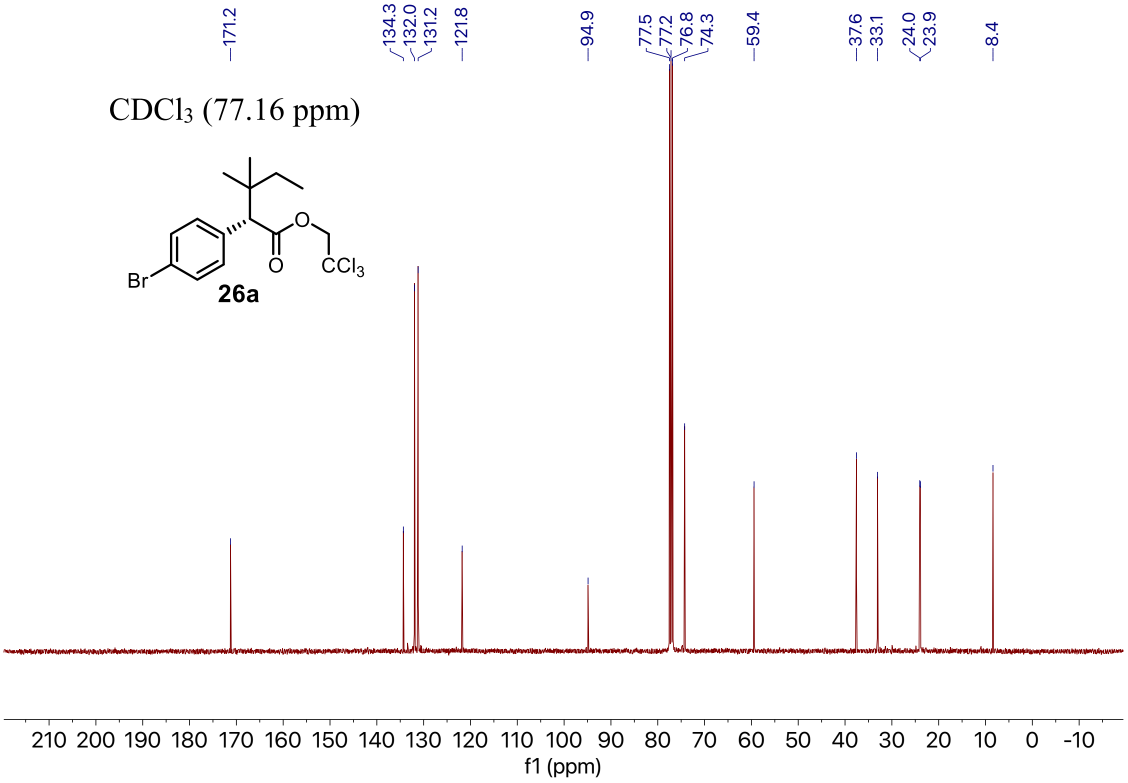
171.2
134.3 132.0 131.2 121.8
94.9
77.5 77.2 76.8 74.3
59.4
37.6 33.1 24.0 23.9
8.4
CDCl3 (77.16 ppm)
Br
O
O
CCl3
26a
210 200 190 180 170 160 150 140 130 120 110 100 90 80 70 60 50 40 30 20 10 0 -10
f1 (ppm)

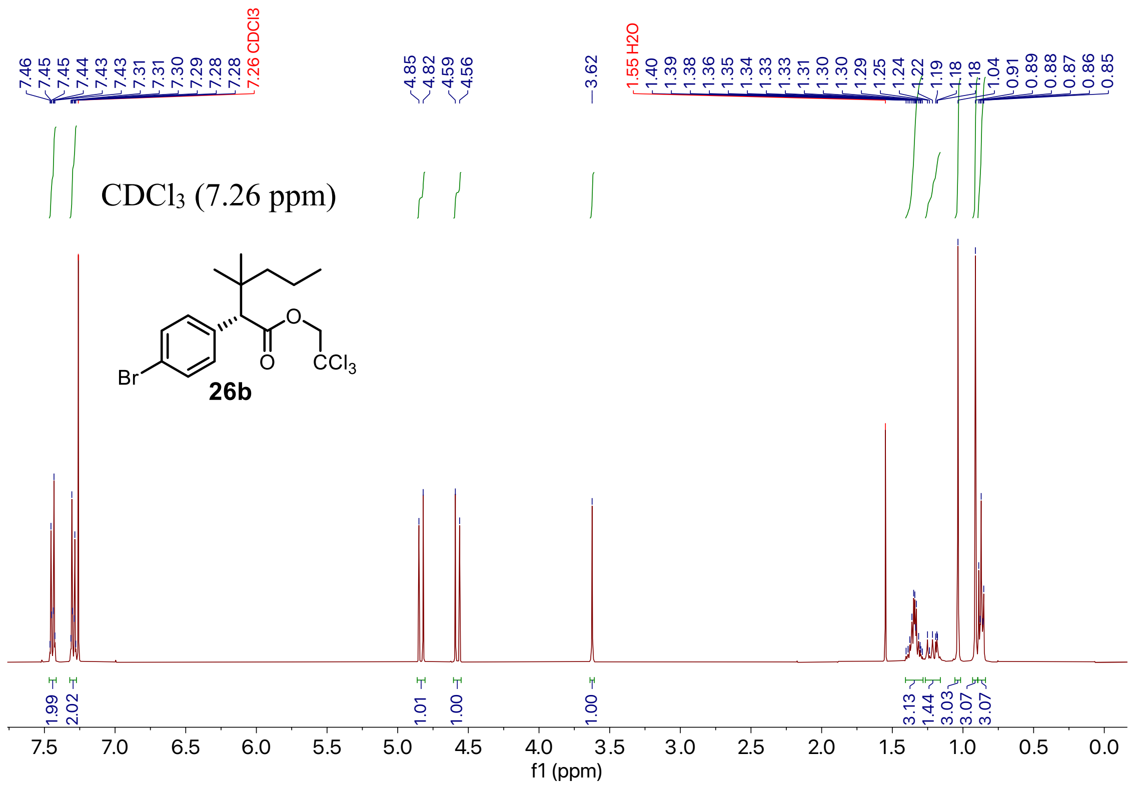

7.46
7.45
7.45
7.44
7.43
7.43
7.31
7.31
7.30
7.29
7.28
7.26 CDCl3
4.85
4.82
4.59
4.56
3.62
1.55 H2O
1.40
1.39
1.38
1.36
1.35
1.34
1.33
1.33
1.31
1.30
1.30
1.29
1.25
1.24
1.22
1.19
1.18
1.18
1.04
0.91
0.89
0.88
0.87
0.86
0.85
CDCl3 (7.26 ppm)
Br
O
O
CCl3
26b
1.99
2.02
1.01
1.00
1.00
3.13
1.44
3.03
3.07
3.07
7.5
7.0
6.5
6.0
5.5
5.0
4.5
4.0
3.5
3.0
2.5
2.0
1.5
1.0
0.5
0.0
f1 (ppm)

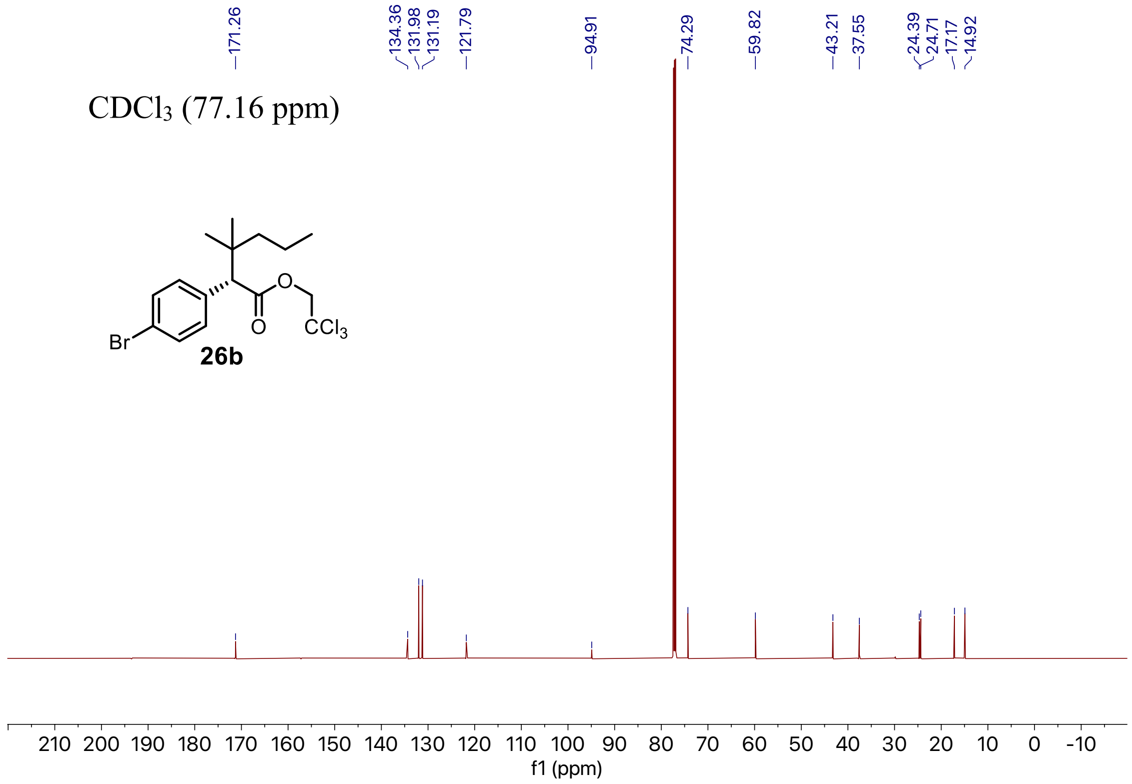

171.26
134.36
131.98
131.19
121.79
94.91
74.29
59.82
43.21
37.55
24.39
24.71
17.17
14.92
CDCl3 (77.16 ppm)
Br
O
O
CCl3
26b
210
200
190
180
170
160
150
140
130
120
110
100
90
80
70
60
50
40
30
20
10
0
-10
f1 (ppm)

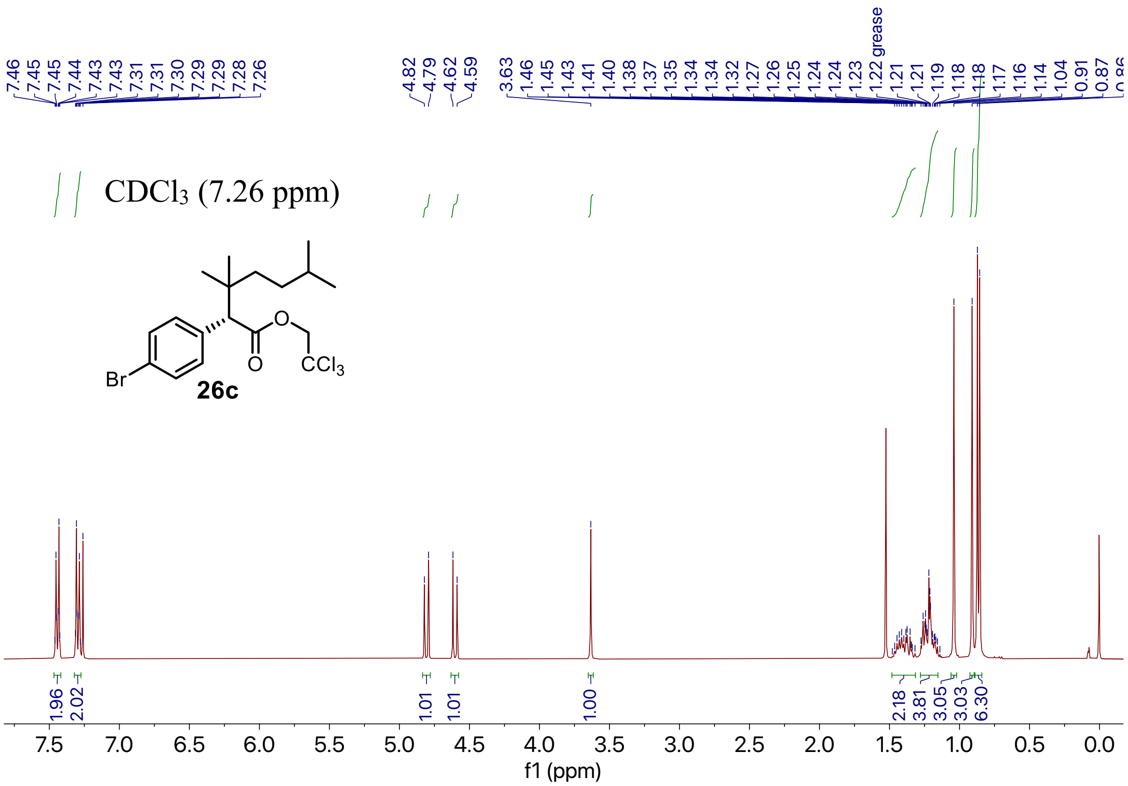

7.46
7.45
7.45
7.44
7.43
7.43
7.31
7.31
7.30
7.29
7.29
7.28
7.26
4.82
4.79
4.62
4.59
3.63
1.46
1.45
1.43
1.41
1.40
1.38
1.37
1.35
1.34
1.34
1.32
1.27
1.26
1.25
1.24
1.24
1.23
1.22 grease
1.21
1.21
1.19
1.18
1.18
1.17
1.16
1.14
1.04
0.91
0.87
CDCl3 (7.26 ppm)
O
Br
26c
O
CCl3
1.96
2.02
1.01
1.01
1.00
2.18
3.81
3.05
3.03
6.30
7.5 7.0 6.5 6.0 5.5 5.0 4.5 4.0 3.5 3.0 2.5 2.0 1.5 1.0 0.5 0.0
f1 (ppm)

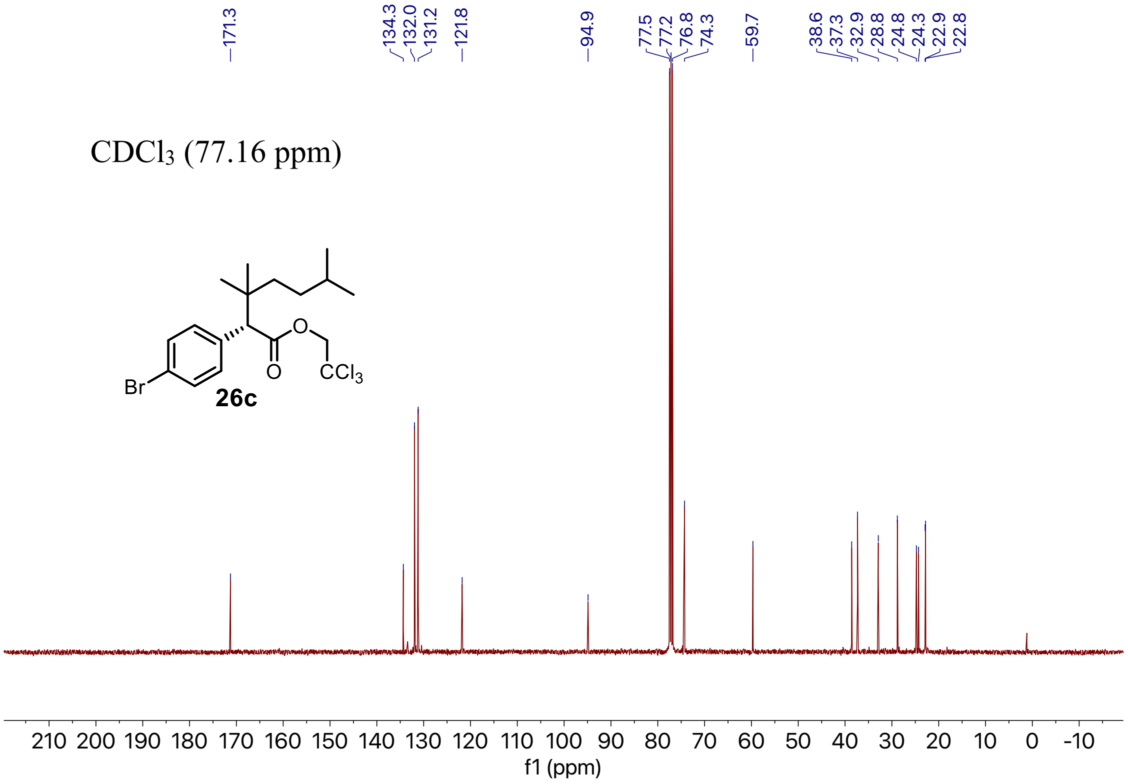

171.3
134.3
132.0
131.2
121.8
94.9
77.5
77.2
76.8
74.3
59.7
38.6
37.3
32.9
28.8
24.8
24.3
22.9
22.8
CDCl3 (77.16 ppm)
O
Br
26c
O
CCl3
210 200 190 180 170 160 150 140 130 120 110 100 90 80 70 60 50 40 30 20 10 0 -10
f1 (ppm)

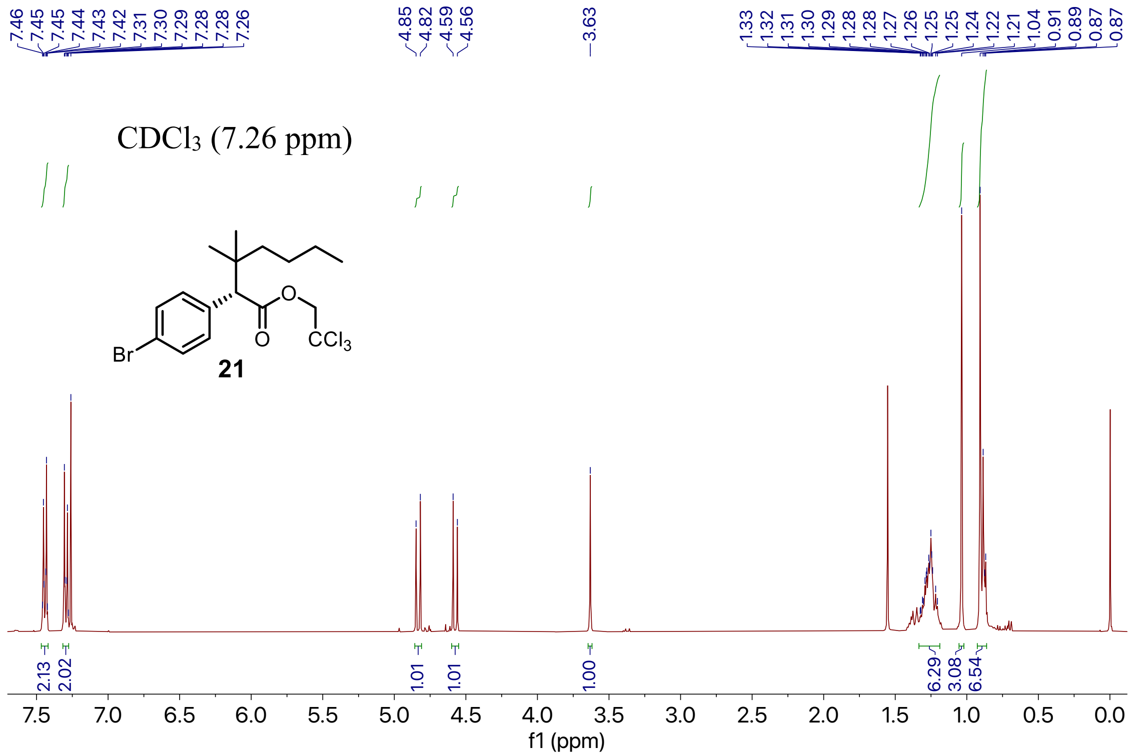
7.46
7.45
7.45
7.44
7.43
7.42
7.31
7.30
7.29
7.28
7.28
7.26
4.85
4.82
4.59
4.56
3.63
1.33
1.32
1.31
1.30
1.29
1.28
1.28
1.27
1.26
1.25
1.25
1.24
1.22
1.21
1.04
0.91
0.89
0.87
0.87
CDCl3 (7.26 ppm)
Br
O
O
CCl3
21
2.13
2.02
1.01
1.01
1.00
6.29
3.08
6.54
f1 (ppm)

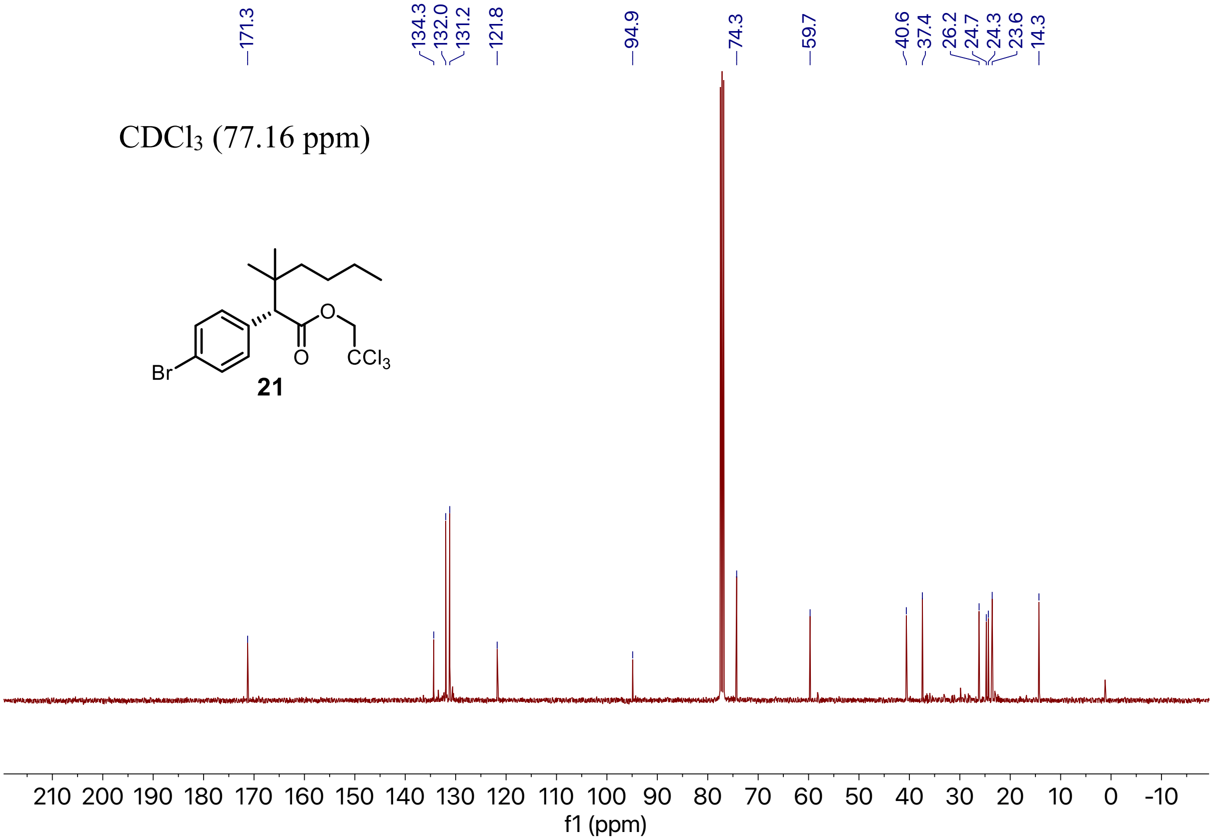
171.3
134.3
132.0
131.2
121.8
94.9
74.3
59.7
40.6
37.4
26.2
24.7
24.3
23.6
14.3
CDCl3 (77.16 ppm)
Br
O
O
CCl3
21
f1 (ppm)

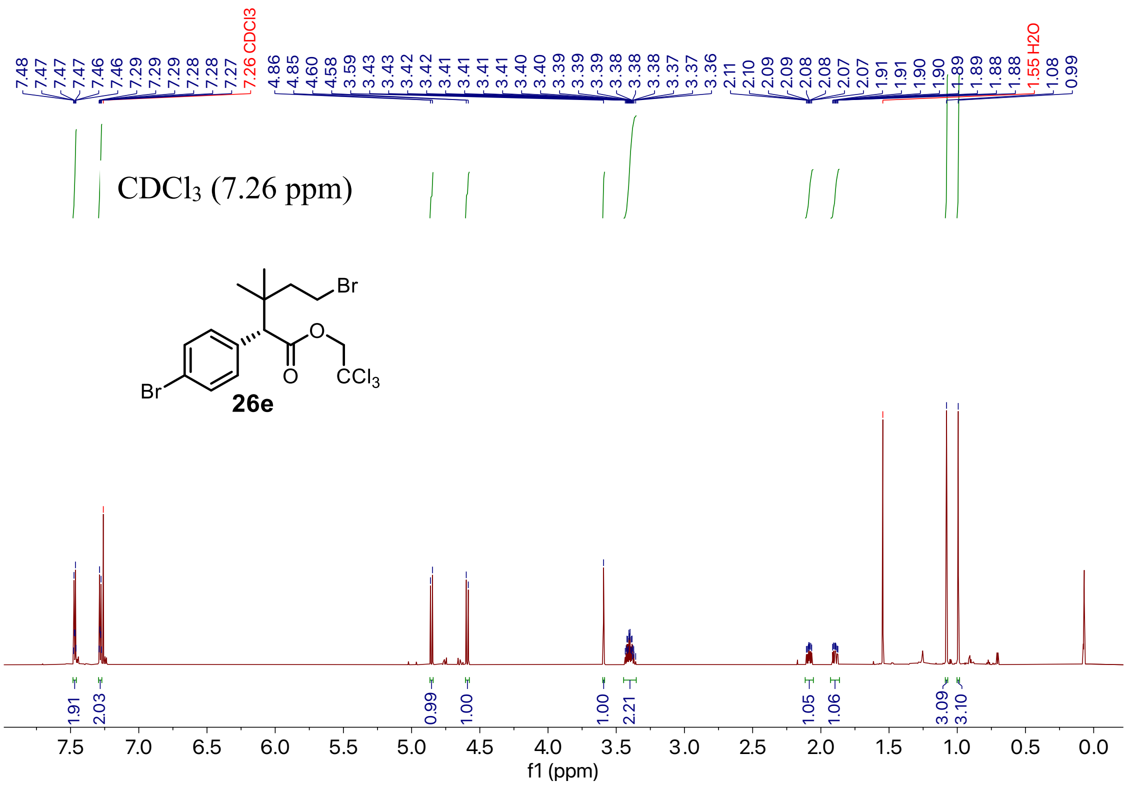

7.48
7.47
7.47
7.47
7.46
7.29
7.29
7.29
7.28
7.28
7.27
7.26 CDCl3
4.86
4.85
4.60
4.58
3.59
3.43
3.43
3.42
3.42
3.41
3.41
3.41
3.40
3.39
3.39
3.39
3.38
3.38
3.38
3.37
3.37
3.36
2.11
2.10
2.09
2.09
2.08
2.08
2.07
2.07
1.91
1.91
1.90
1.90
1.89
1.89
1.88
1.88
1.55 H2O
1.08
0.99
CDCl3 (7.26 ppm)
Br
O
O
CCl3
Br
26e
1.91
2.03
0.99
1.00
1.00
2.21
1.05
1.06
3.09
3.10
7.5
7.0
6.5
6.0
5.5
5.0
4.5
4.0
3.5
3.0
2.5
2.0
1.5
1.0
0.5
0.0
f1 (ppm)

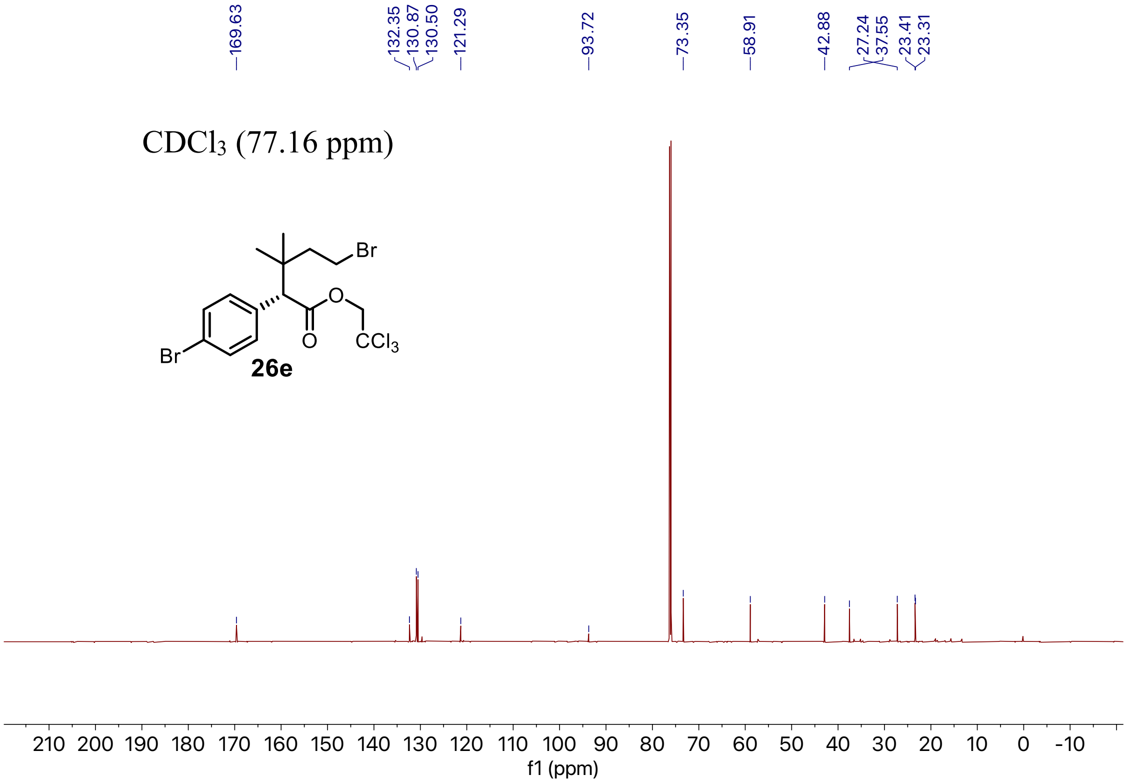

169.63
132.35
130.87
130.50
121.29
93.72
73.35
58.91
42.88
27.24
37.55
23.41
23.31
CDCl3 (77.16 ppm)
Br
O
O
CCl3
Br
26e
210
200
190
180
170
160
150
140
130
120
110
100
90
80
70
60
50
40
30
20
10
0
-10
f1 (ppm)

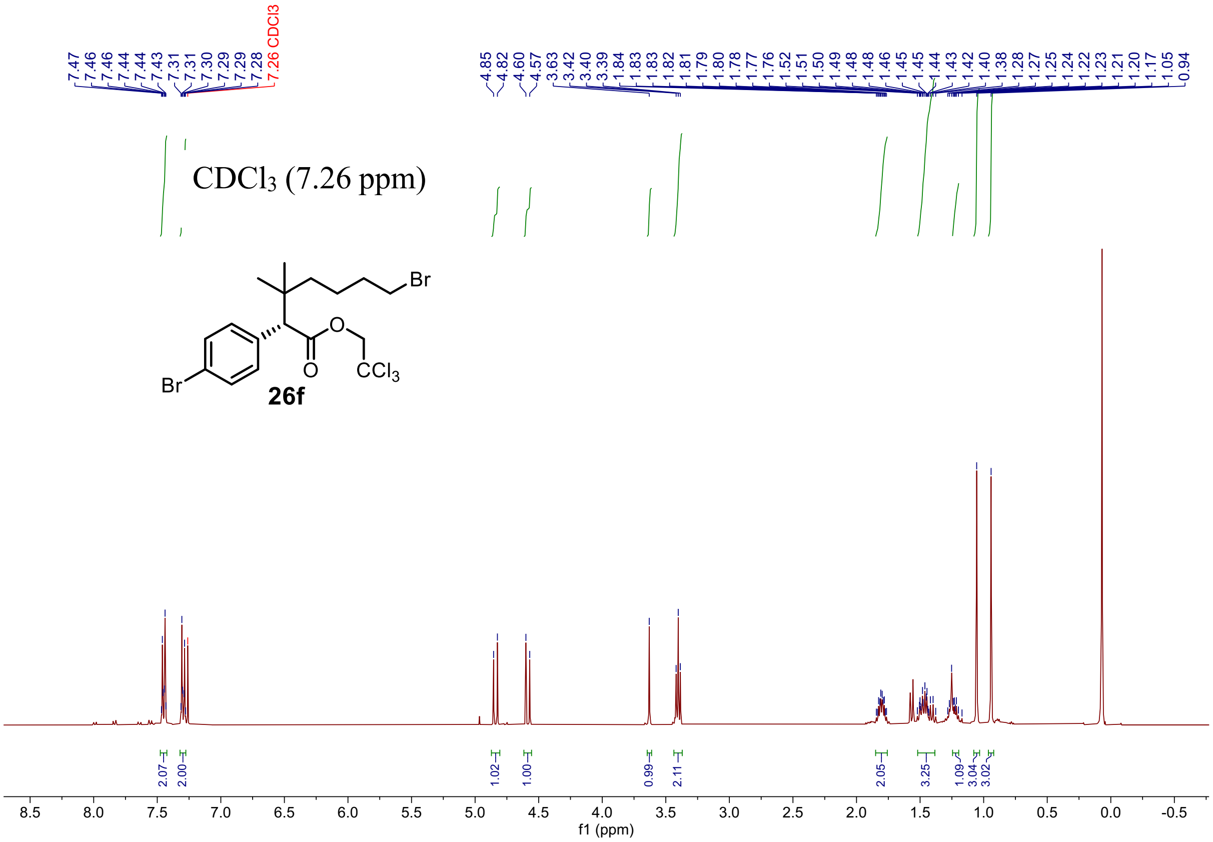
7.47 7.46 7.46 7.44 7.44 7.43 7.31 7.31 7.30 7.29 7.29 7.28 7.26 CDCl3
4.85 4.82 4.60 4.57 3.63 3.42 3.40 3.39 1.84 1.83 1.83 1.82 1.81 1.79 1.80 1.78 1.77 1.76 1.52 1.51 1.50 1.49 1.48 1.48 1.46 1.45 1.45 1.44 1.43 1.42 1.40 1.38 1.28 1.27 1.25 1.24 1.22 1.23 1.21 1.20 1.17 1.05 0.94
CDCl3 (7.26 ppm)
Br
O
O
CCl3
Br
26f
2.07 2.00 1.02 1.00 0.99 2.11 2.05 3.25 1.09 3.04 3.02
f1 (ppm)

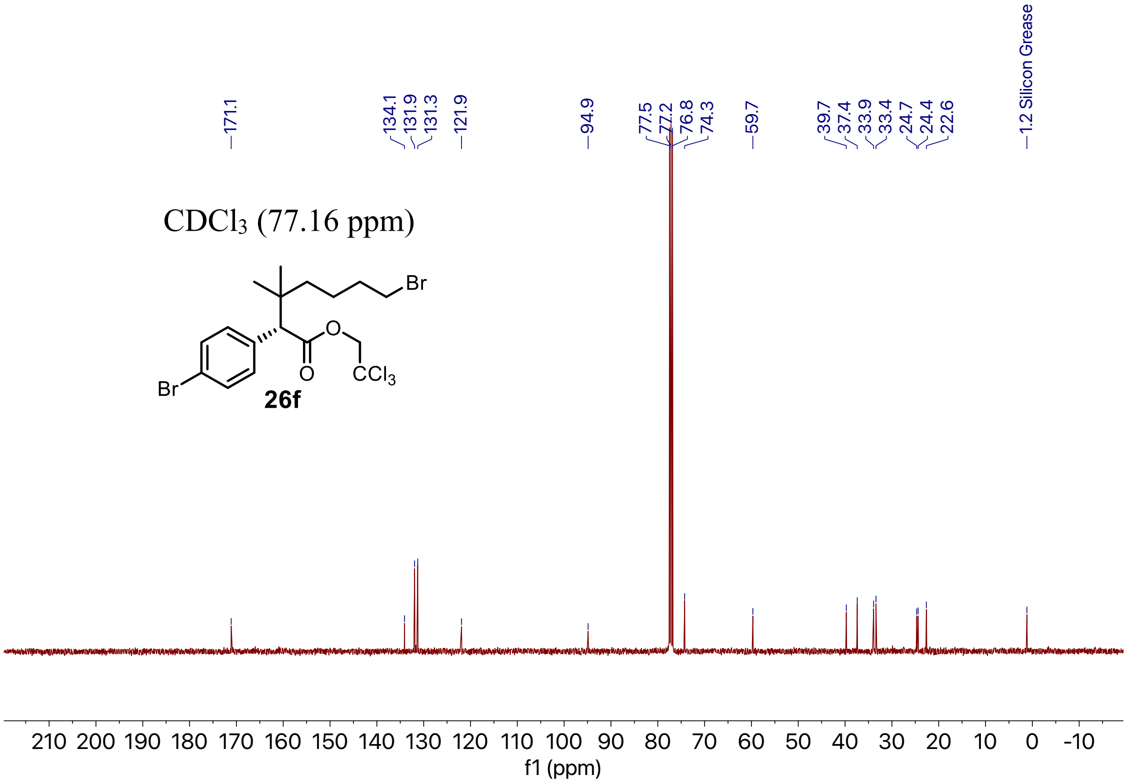
171.1
134.1 131.9 131.3 121.9
94.9
77.5 77.2 76.8 74.3
59.7
39.7 37.4 33.9 33.4 24.7 24.4 22.6
1.2 Silicon Grease
CDCl3 (77.16 ppm)
Br
O
O
CCl3
Br
26f
f1 (ppm)

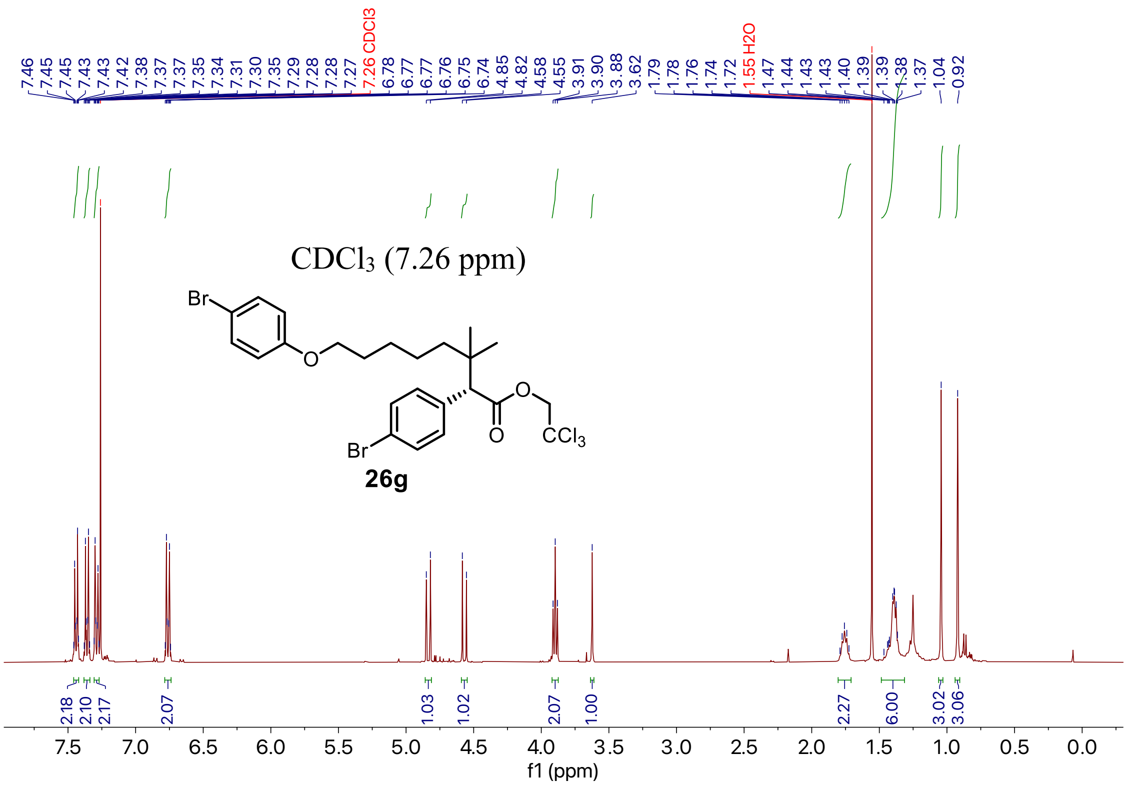
CDCl3 (7.26 ppm)
26g
f1 (ppm)

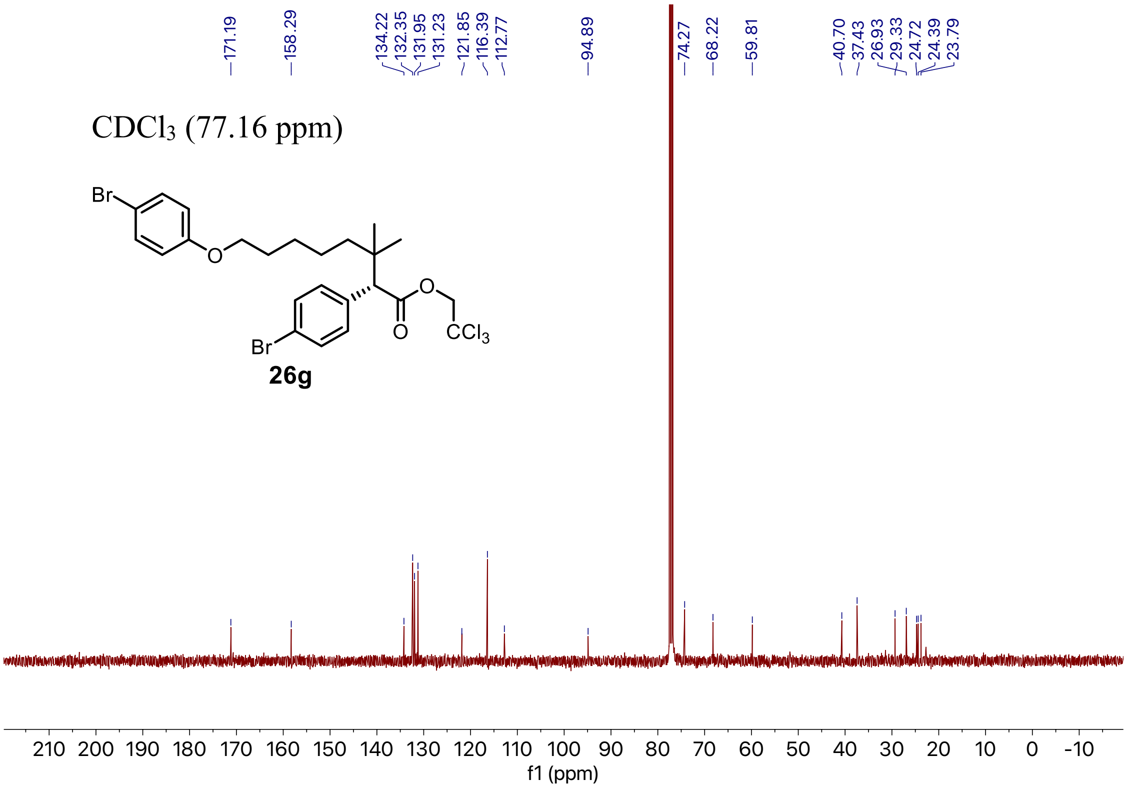
CDCl3 (77.16 ppm)
26g
f1 (ppm)

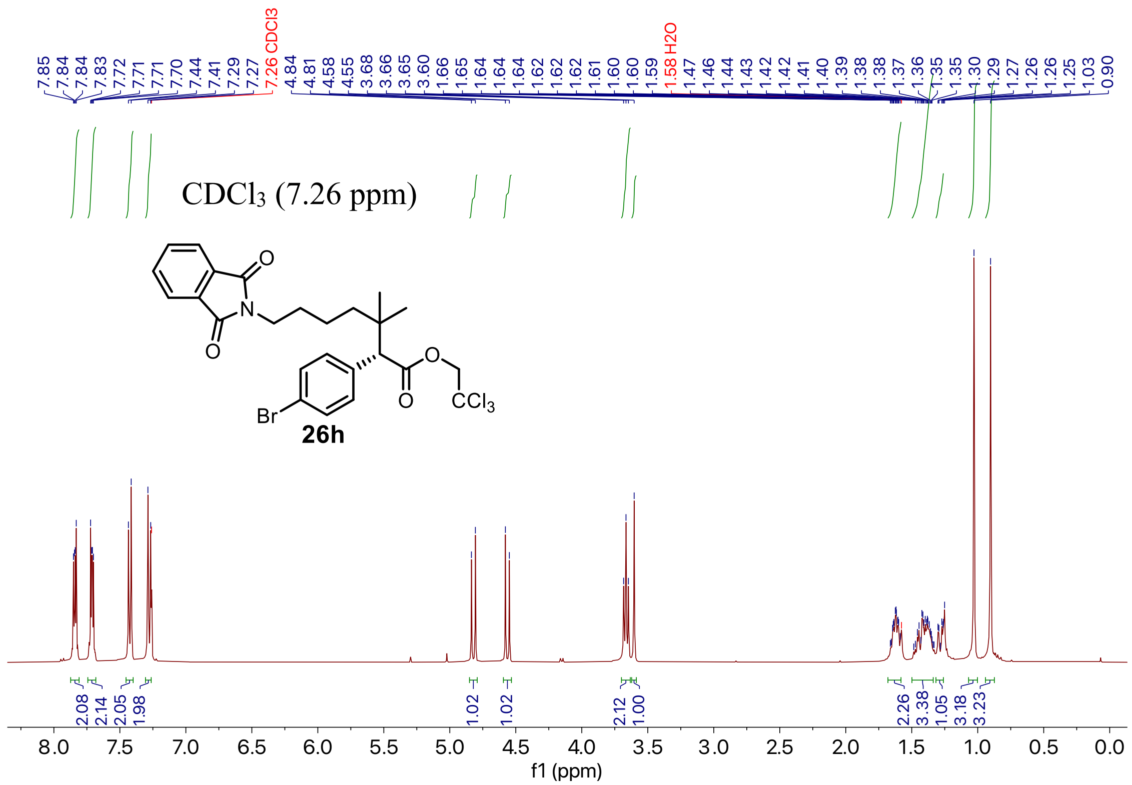
CDCl3 (7.26 ppm)
26h
f1 (ppm)

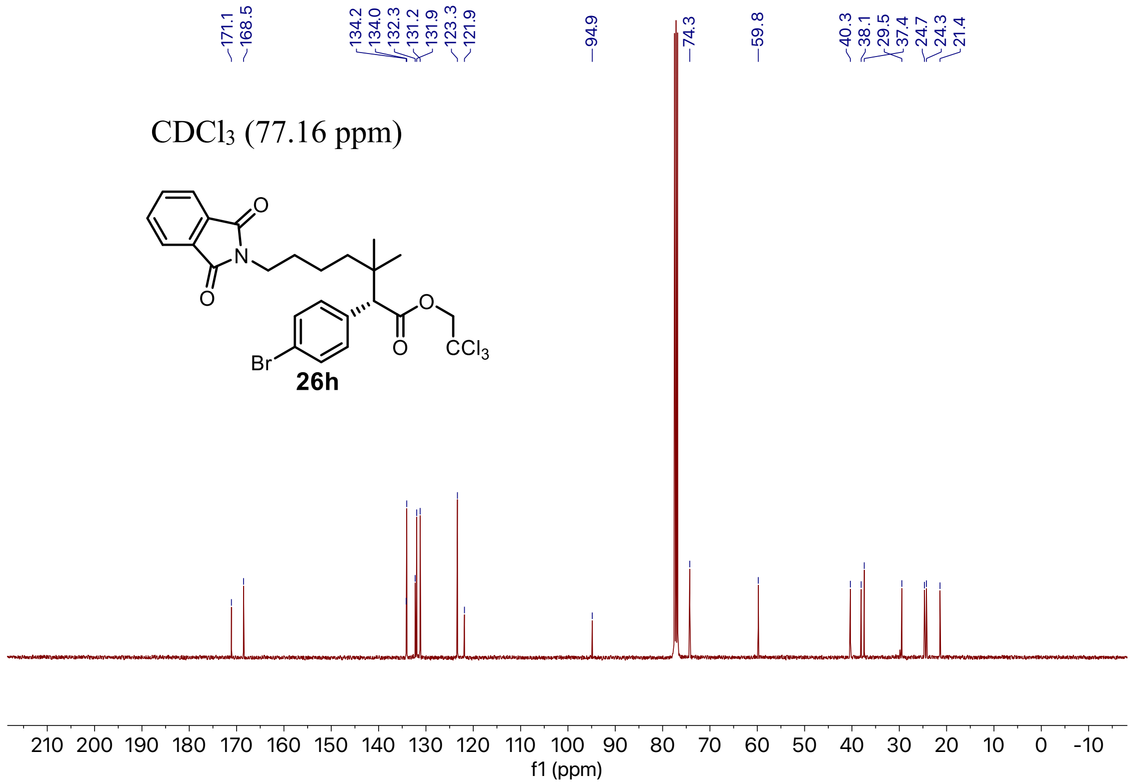
CDCl3 (77.16 ppm)
26h
f1 (ppm)

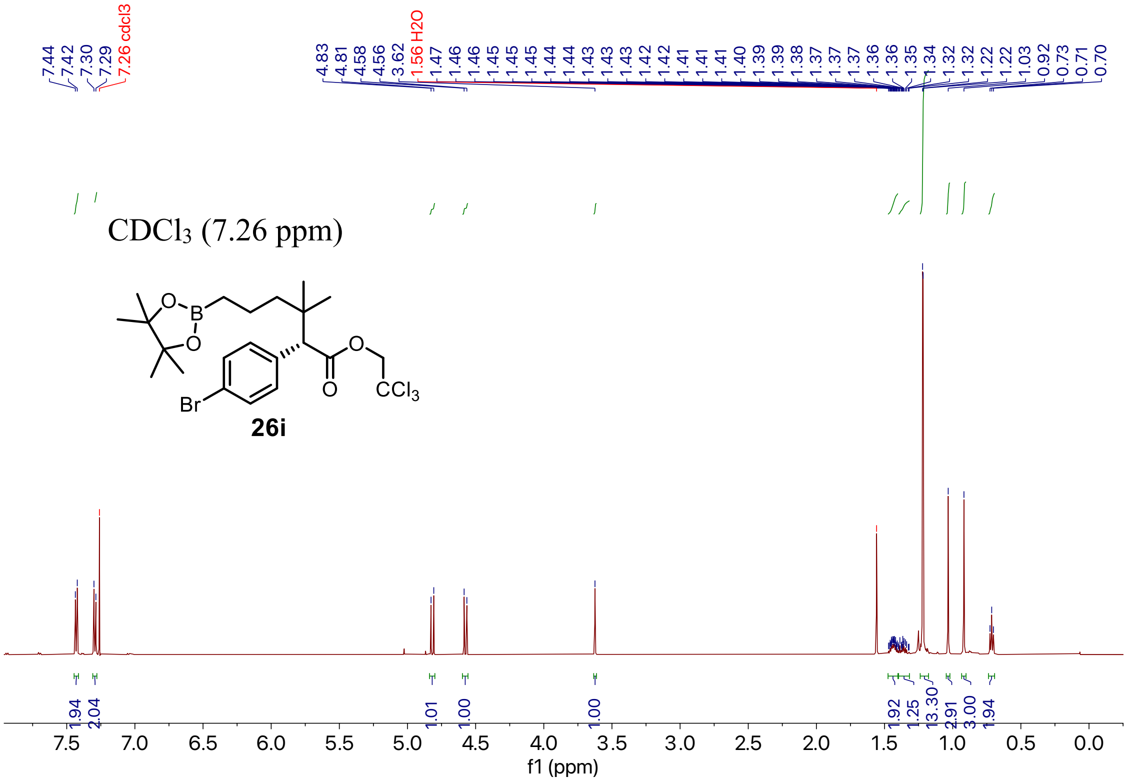

7.44 7.42 7.30 7.29 7.26 cdcl3
4.83 4.81 4.58 4.56 3.62 1.56 H2O 1.47 1.46 1.46 1.45 1.45 1.45 1.44 1.44 1.43 1.43 1.43 1.42 1.42 1.41 1.41 1.41 1.40 1.39 1.39 1.38 1.37 1.37 1.37 1.36 1.36 1.35 1.34 1.32 1.32 1.22 1.22 1.03 0.92 0.73 0.71 0.70
CDCl3 (7.26 ppm)
O B O Br CCl3 O O
26i
1.94 2.04 1.01 1.00 1.00 1.92 1.25 13.30 2.91 3.00 1.94
7.5 7.0 6.5 6.0 5.5 5.0 4.5 4.0 3.5 3.0 2.5 2.0 1.5 1.0 0.5 0.0
f1 (ppm)

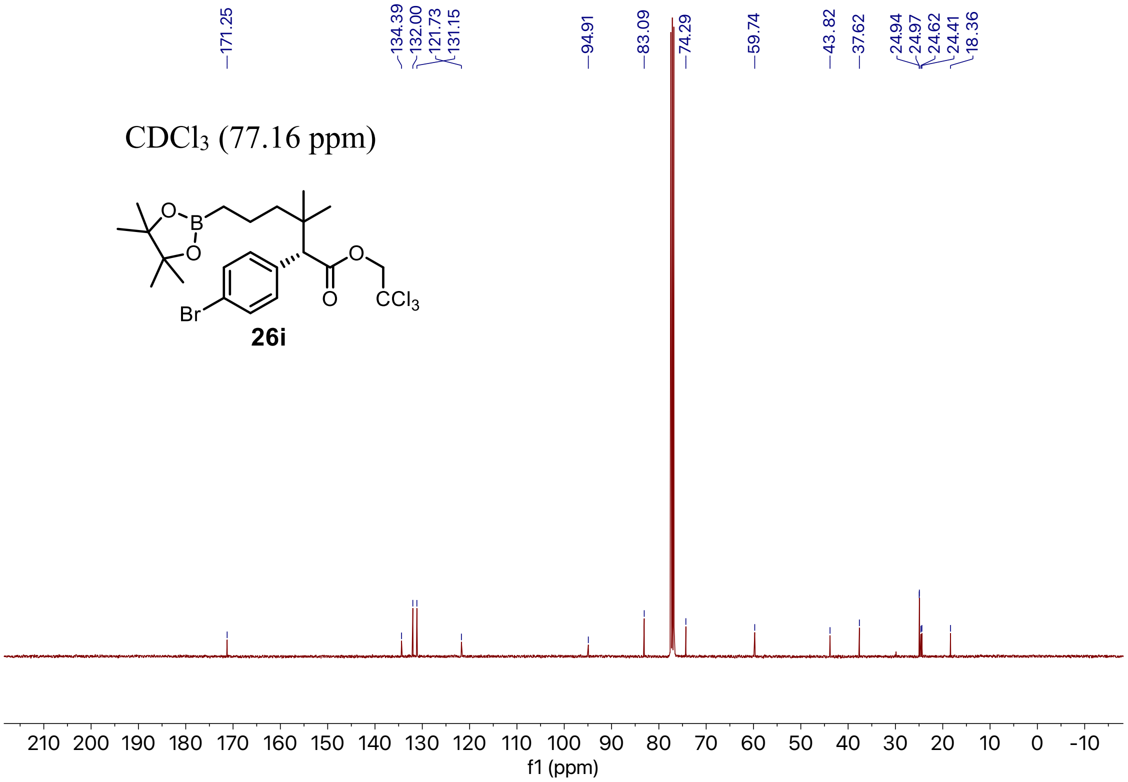

171.25 134.39 132.00 121.73 131.15 94.91 83.09 74.29 59.74 43.82 37.62 24.94 24.97 24.62 24.41 18.36
CDCl3 (77.16 ppm)
O B O Br CCl3 O O
26i
210 200 190 180 170 160 150 140 130 120 110 100 90 80 70 60 50 40 30 20 10 0 -10
f1 (ppm)

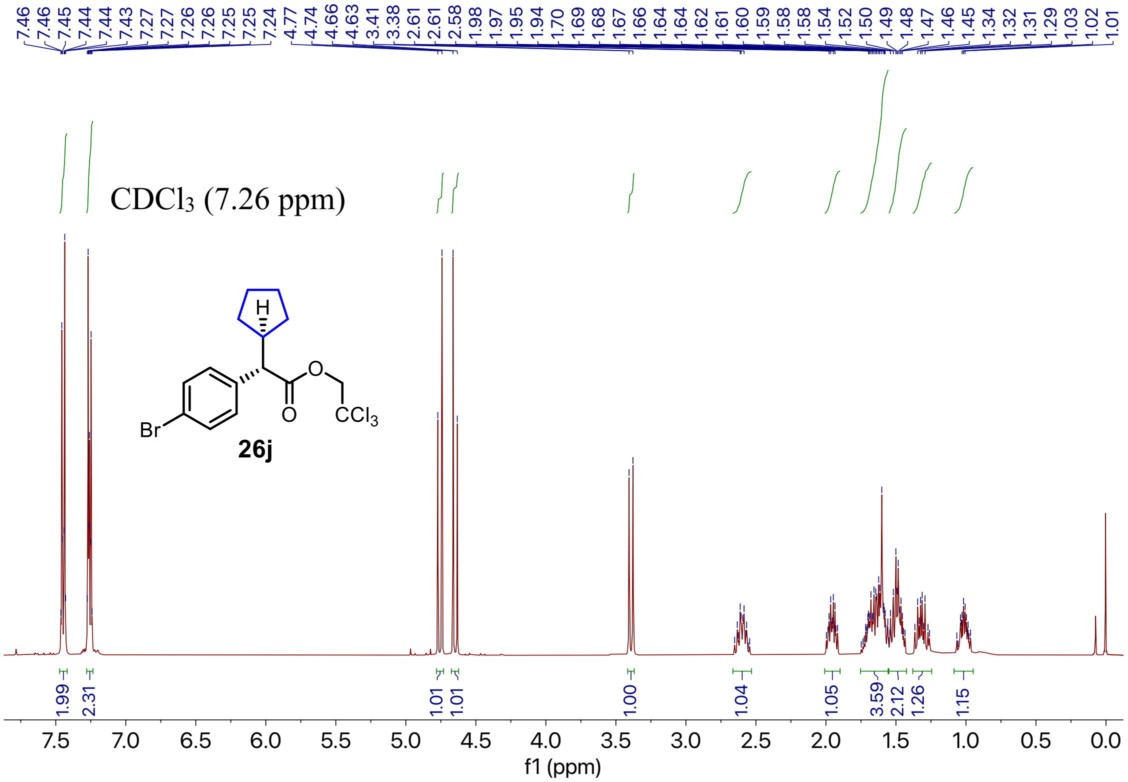
CDCl3 (7.26 ppm)
Br
CCl3
26j
f1 (ppm)

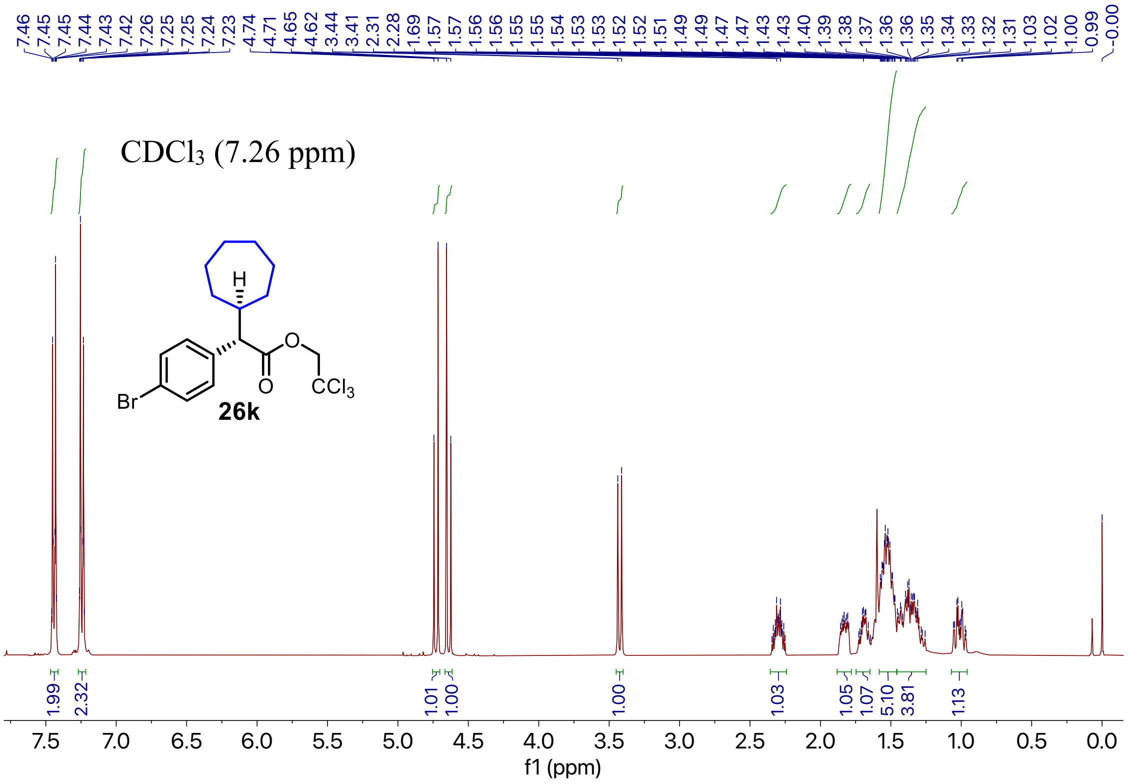
CDCl3 (7.26 ppm)
Br
CCl3
26k
f1 (ppm)

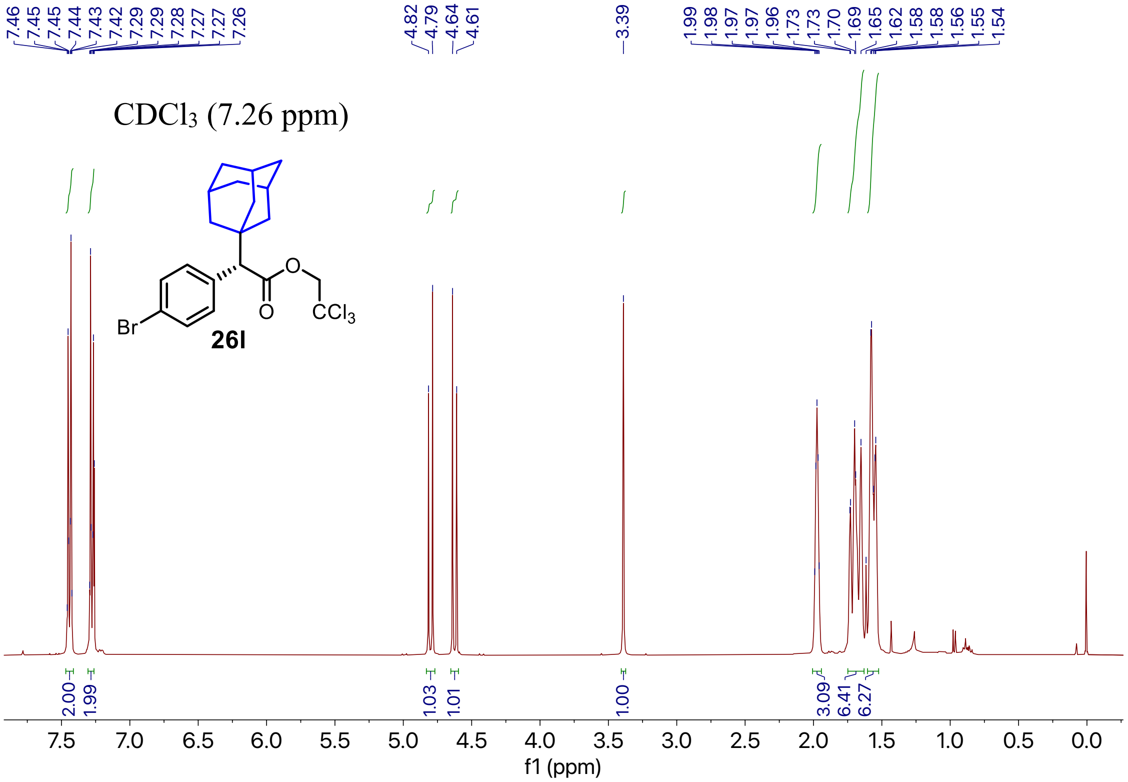
CDCl3 (7.26 ppm)
Br
O
O
CCl3
26l
f1 (ppm)

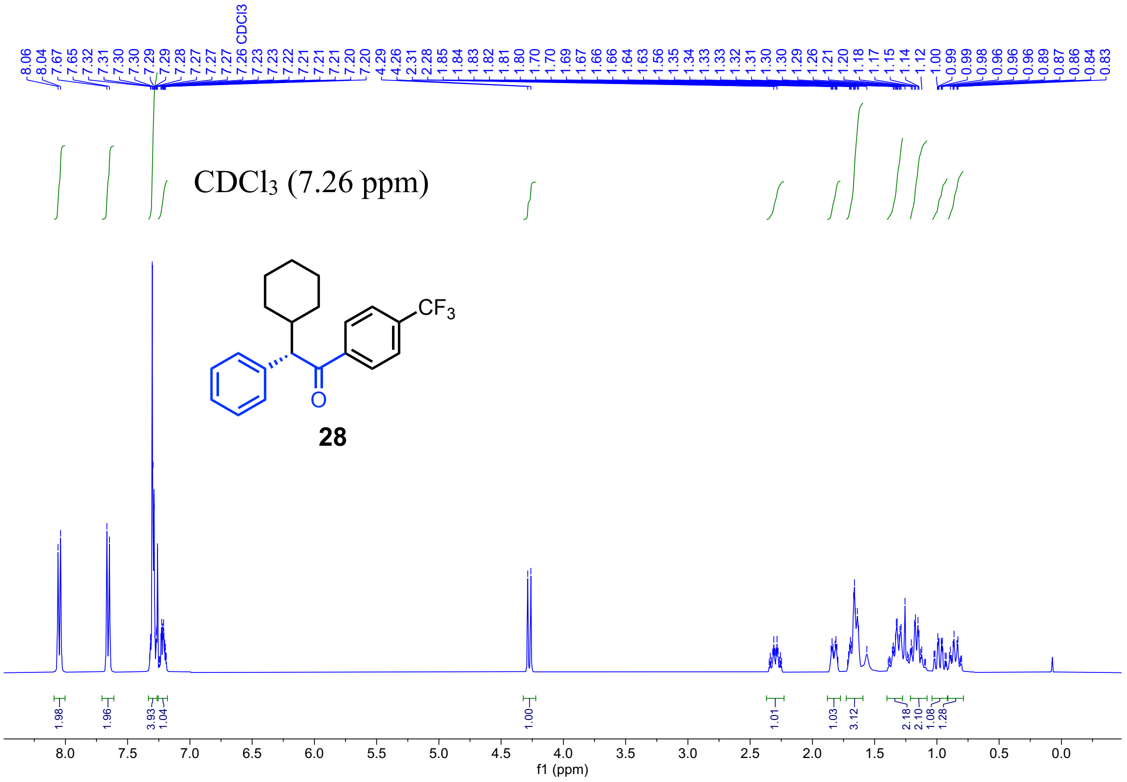
CDCl3 (7.26 ppm)
CF3
O
28
f1 (ppm)

9. HPLC and SFC Chromatograms

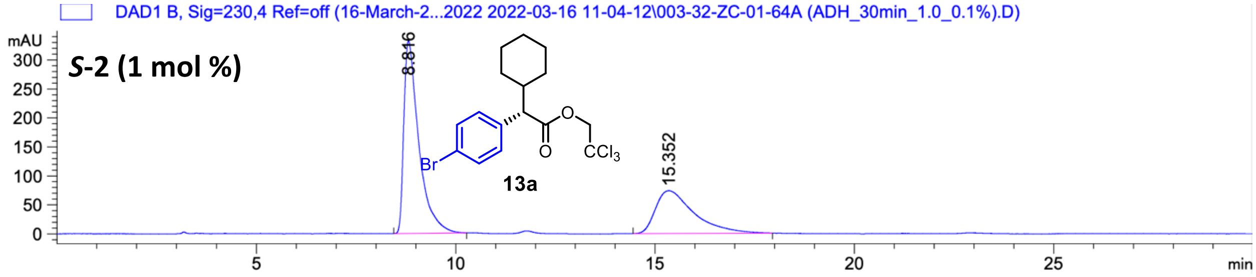

Signal 2: DAD1 B, Sig=230,4 Ref=off

Peak #	RetTime [min]	Type	Width [min]	Area [mAU*s]	Height [mAU]	Area %
1	8.816	BB	0.3506	8657.05273	332.64853	63.6057
2	15.352	BB	0.7824	4953.44189	74.00451	36.3943

Totals : 1.36105e4 406.65304

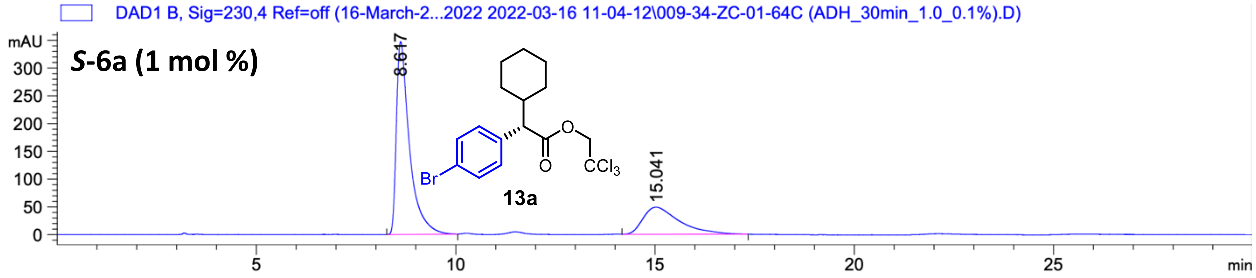

Signal 2: DAD1 B, Sig=230,4 Ref=off

Peak #	RetTime [min]	Type	Width [min]	Area [mAU*s]	Height [mAU]	Area %
1	8.617	BB	0.3284	7928.01123	347.14474	71.9436
2	15.041	BB	0.7408	3091.75098	48.82613	28.0564

Totals : 1.10198e4 395.97087

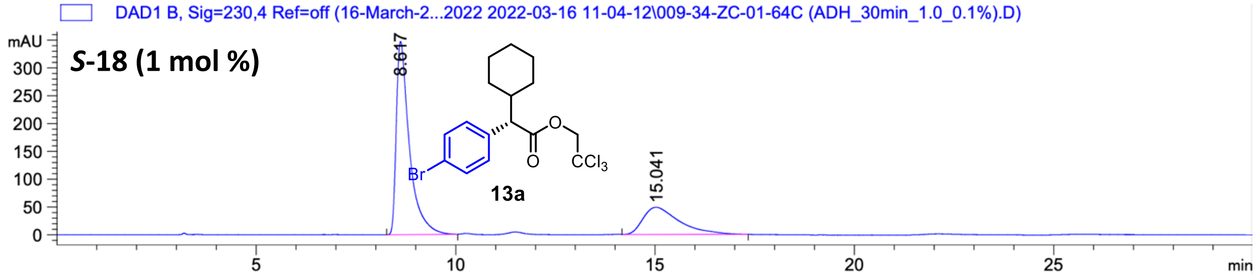

Signal 2: DAD1 B, Sig=230,4 Ref=off

Peak #	RetTime [min]	Type	Width [min]	Area [mAU*s]	Height [mAU]	Area %
1	8.617	BB	0.3284	7928.01123	347.14474	71.9436
2	15.041	BB	0.7408	3091.75098	48.82613	28.0564

Totals : 1.10198e4 395.97087

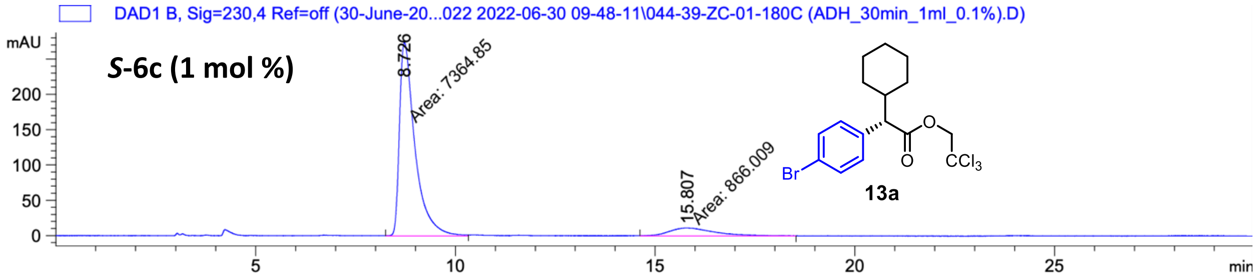

Signal 2: DAD1 B, Sig=230,4 Ref=off

Peak #	RetTime [min]	Type	Width [min]	Area [mAU*s]	Height [mAU]	Area %
1	8.726	MF	0.4481	7364.85254	273.95590	89.4785
2	15.807	FM	1.2928	866.00903	11.16480	10.5215

Totals : 8230.86157 285.12070

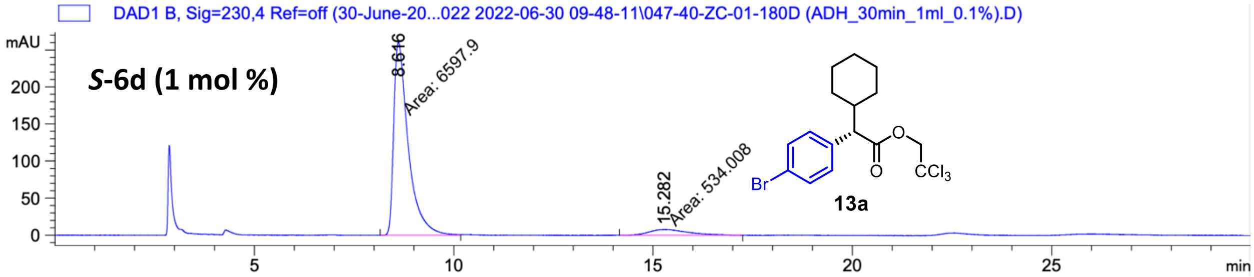

Signal 2: DAD1 B, Sig=230,4 Ref=off

Peak #	RetTime [min]	Type	Width [min]	Area [mAU*s]	Height [mAU]	Area %
1	8.616	MF	0.4220	6597.89648	260.59055	92.5124
2	15.282	MM	1.1337	534.00848	7.85051	7.4876
Totals :				7131.90497	268.44106	

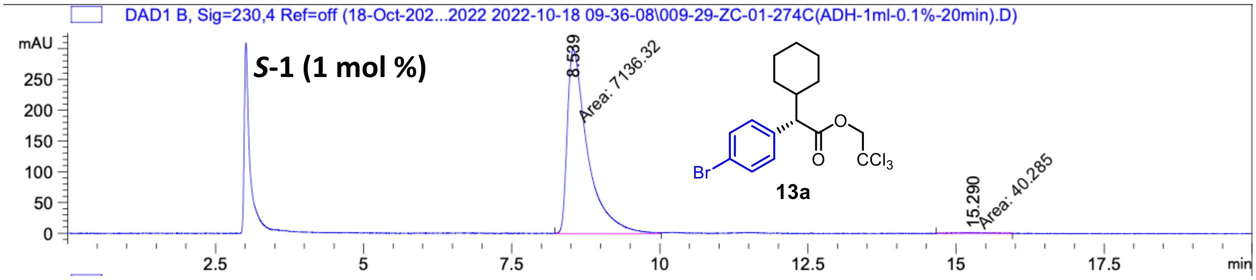

Signal 2: DAD1 B, Sig=230,4 Ref=off

Peak #	RetTime [min]	Type	Width [min]	Area [mAU*s]	Height [mAU]	Area %
1	8.539	MM	0.3970	7136.31641	299.59177	99.4387
2	15.290	MM	0.5161	40.28500	1.30092	0.5613
Totals :				7176.60140	300.89269	

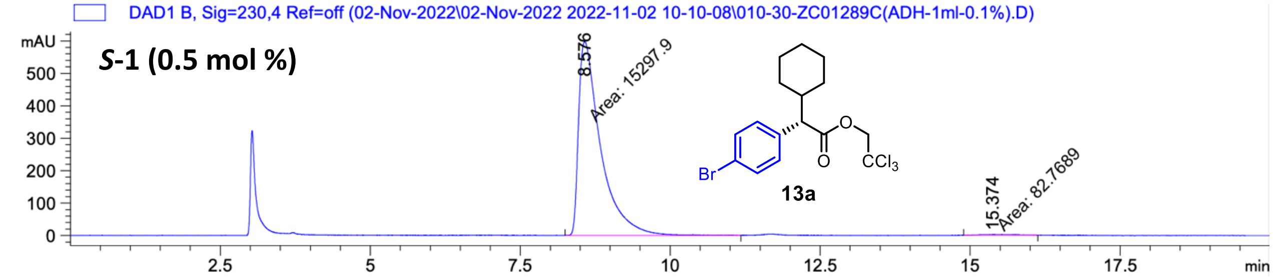

Signal 2: DAD1 B, Sig=230,4 Ref=off

Peak #	RetTime [min]	Type	Width [min]	Area [mAU*s]	Height [mAU]	Area %
1	8.576	MM	0.4264	1.52979e4	597.88763	99.4619
2	15.374	MM	0.6602	82.76888	2.08953	0.5381

Totals : 1.53806e4 599.97717

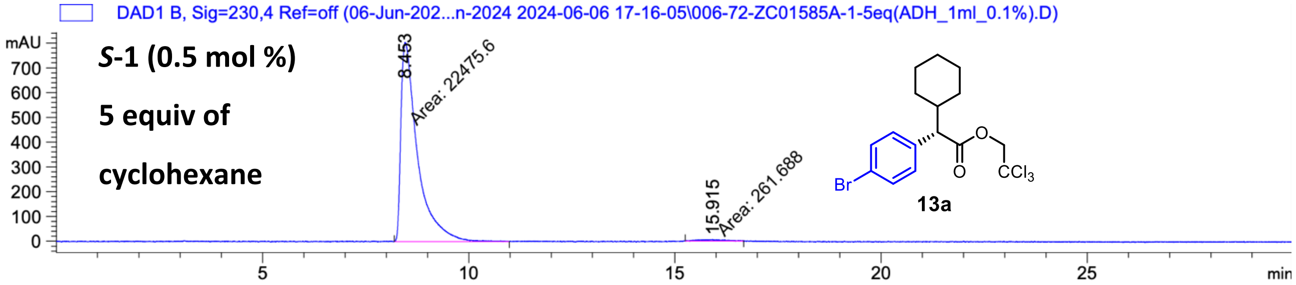

Signal 2: DAD1 B, Sig=230,4 Ref=off

Peak #	RetTime [min]	Type	Width [min]	Area [mAU*s]	Height [mAU]	Area %
1	8.453	MM	0.4662	2.24756e4	803.52441	98.8491
2	15.915	MM	0.6361	261.68787	6.85693	1.1509

Totals : 2.27373e4 810.38135

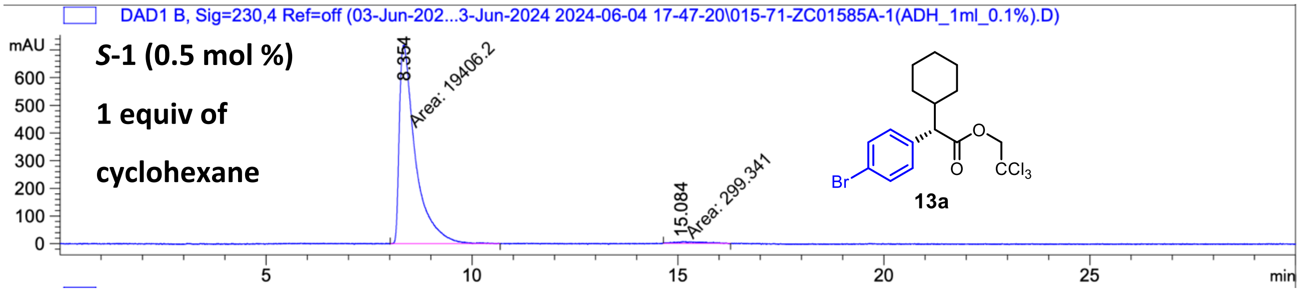

Signal 2: DAD1 B, Sig=230,4 Ref=off

Peak #	RetTime [min]	Type	Width [min]	Area [mAU*s]	Height [mAU]	Area %
1	8.354	MM	0.4505	1.94062e4	717.96234	98.4809
2	15.084	MM	0.7986	299.34125	6.24738	1.5191
Totals :				1.97056e4	724.20972	

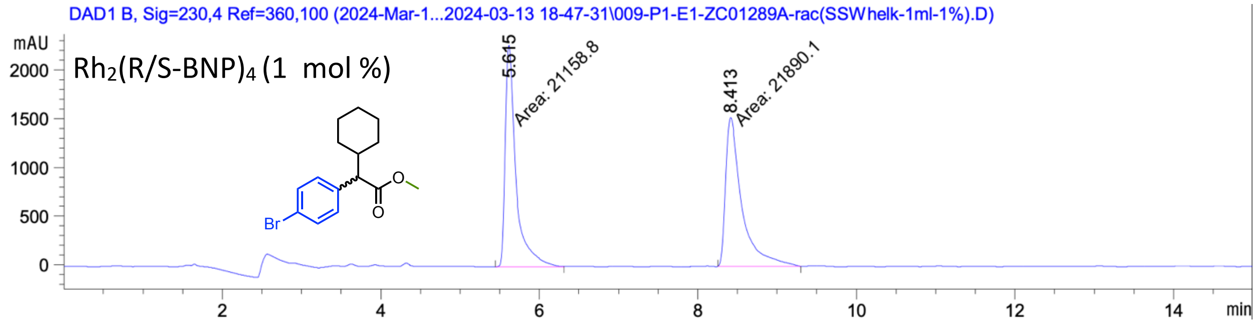

Signal 2: DAD1 B, Sig=230,4 Ref=360,100

Peak #	RetTime [min]	Type	Width [min]	Area [mAU*s]	Height [mAU]	Area %
1	5.615	MM	0.1551	2.11588e4	2273.89258	49.1506
2	8.413	MM	0.2386	2.18901e4	1529.37231	50.8494
Totals :				4.30489e4	3803.26489	

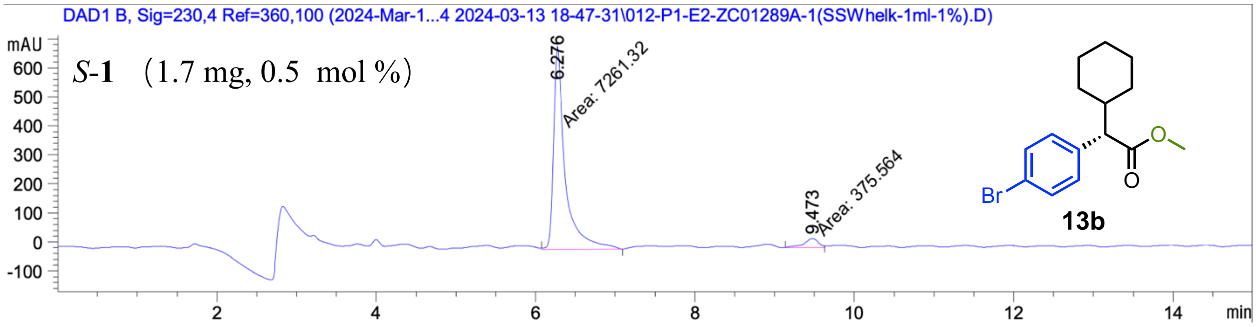

Signal 2: DAD1 B, Sig=230,4 Ref=360,100

Peak #	RetTime [min]	Type	Width [min]	Area [mAU*s]	Height [mAU]	Area %
1	6.276	MM	0.1731	7261.32031	699.11169	95.0822
2	9.473	MM	0.2046	375.56369	30.59856	4.9178
Totals :				7636.88400	729.71026	

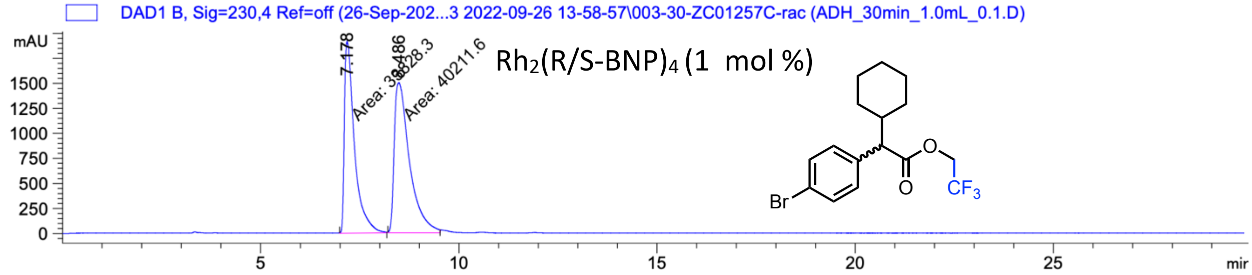

Signal 2: DAD1 B, Sig=230,4 Ref=off

Peak #	RetTime [min]	Type	Width [min]	Area [mAU*s]	Height [mAU]	Area %
1	7.178	MM	0.2941	3.38283e4	1917.33777	45.6893
2	8.486	FM	0.4461	4.02116e4	1502.48047	54.3107

Totals : 7.40399e4 3419.81824

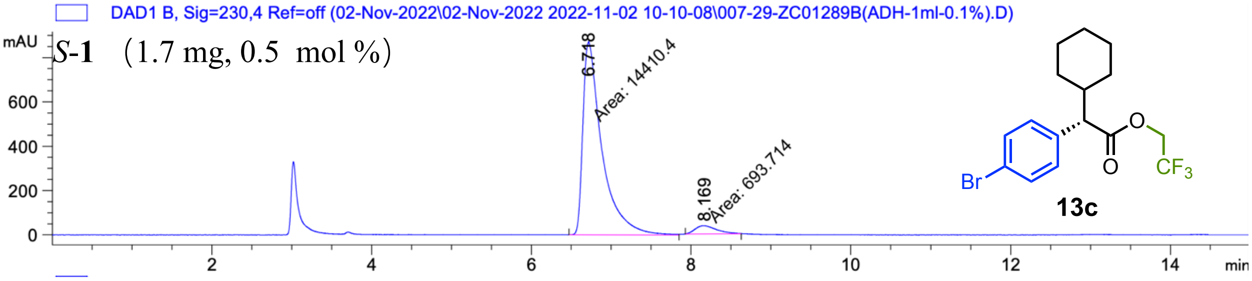

Signal 2: DAD1 B, Sig=230,4 Ref=off

Peak #	RetTime [min]	Type	Width [min]	Area [mAU*s]	Height [mAU]	Area %
1	6.718	MM	0.2753	1.44104e4	872.36676	95.4071
2	8.169	MM	0.3120	693.71362	37.05638	4.5929

Totals : 1.51042e4 909.42314

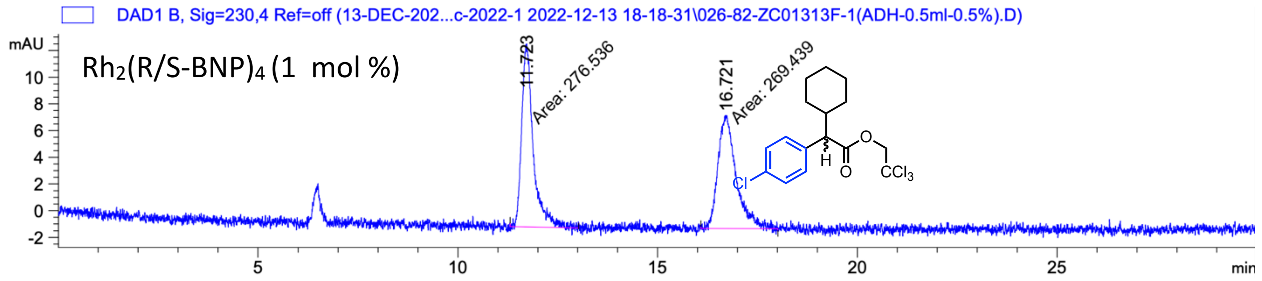

Signal 2: DAD1 B, Sig=230,4 Ref=off

Peak #	RetTime [min]	Type	Width [min]	Area [mAU*s]	Height [mAU]	Area %
1	11.723	MM	0.3359	276.53616	13.72200	50.6499
2	16.721	MM	0.5299	269.43939	8.47415	49.3501

Totals : 545.97556 22.19615

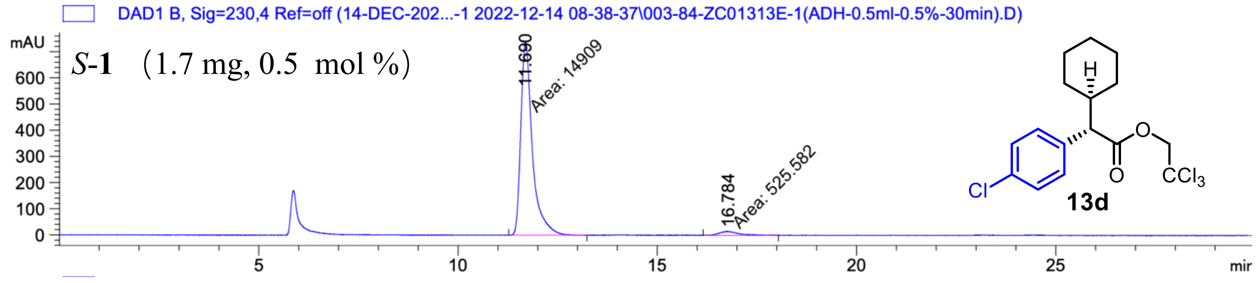

Signal 2: DAD1 B, Sig=230,4 Ref=off

Peak #	RetTime [min]	Type	Width [min]	Area [mAU*s]	Height [mAU]	Area %
1	11.690	MM	0.3373	1.49090e4	736.72101	96.5948
2	16.784	MM	0.5975	525.58185	14.66092	3.4052

Totals : 1.54346e4 751.38192

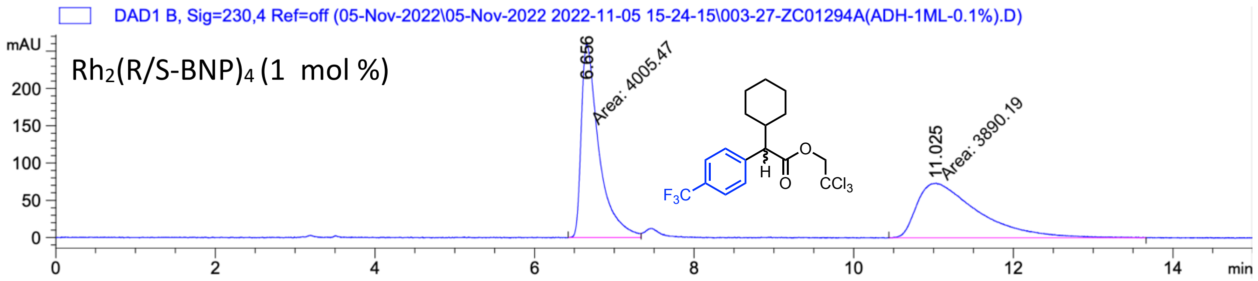

Signal 2: DAD1 B, Sig=230,4 Ref=off

Peak #	RetTime [min]	Type	Width [min]	Area [mAU*s]	Height [mAU]	Area %
1	6.656	MF	0.2576	4005.46729	259.15402	50.7300
2	11.025	MM	0.8841	3890.19043	73.33485	49.2700

Totals : 7895.65771 332.48887

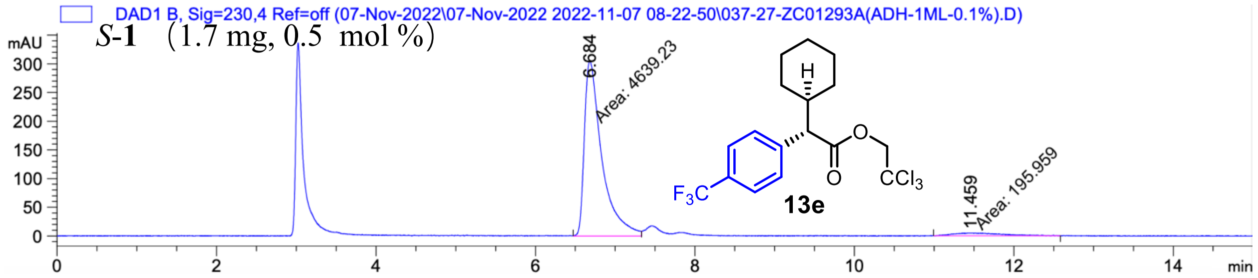

Signal 2: DAD1 B, Sig=230,4 Ref=off

Peak #	RetTime [min]	Type	Width [min]	Area [mAU*s]	Height [mAU]	Area %
1	6.684	MF	0.2531	4639.23438	305.50583	95.9472
2	11.459	MM	0.7024	195.95901	4.64982	4.0528

Totals : 4835.19339 310.15565

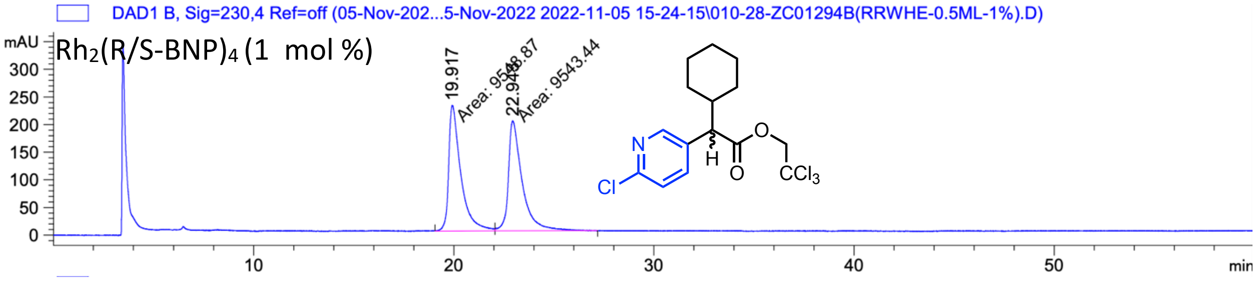

Signal 2: DAD1 B, Sig=230,4 Ref=off

Peak #	RetTime [min]	Type	Width [min]	Area [mAU*s]	Height [mAU]	Area %
1	19.917	MF	0.6999	9548.87305	227.39455	50.0142
2	22.943	FM	0.7978	9543.44238	199.35765	49.9858

Totals : 1.90923e4 426.75220

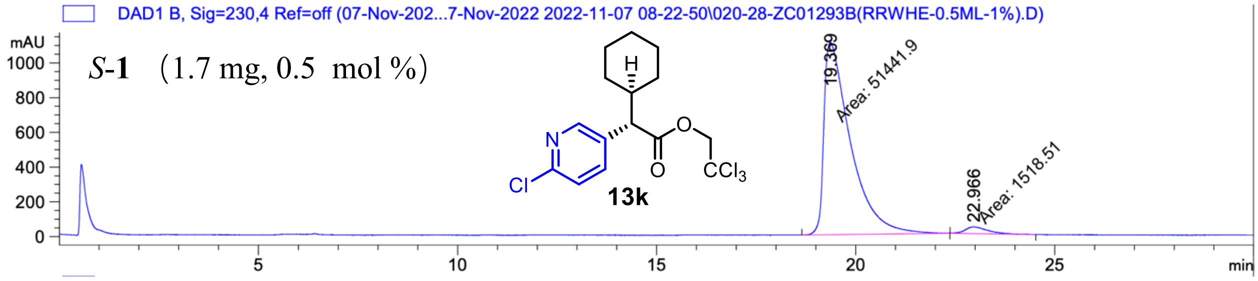

Signal 2: DAD1 B, Sig=230,4 Ref=off

Peak #	RetTime [min]	Type	Width [min]	Area [mAU*s]	Height [mAU]	Area %
1	19.369	MM	0.7736	5.14419e4	1108.27991	97.1327
2	22.966	MM	0.6530	1518.50793	38.75853	2.8673

Totals : 5.29604e4 1147.03844

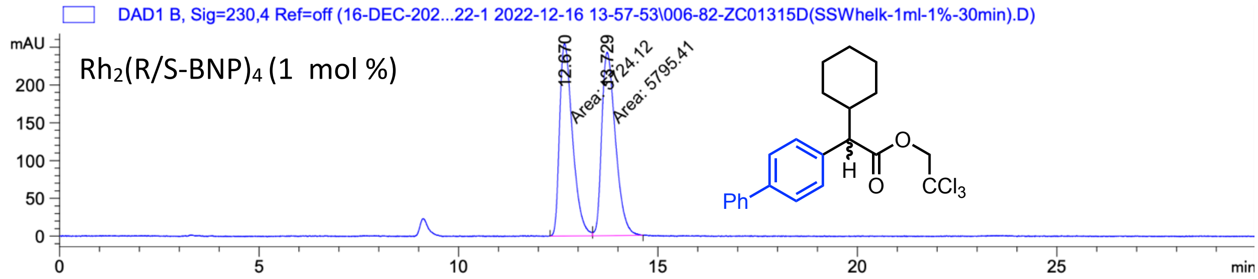

Signal 2: DAD1 B, Sig=230,4 Ref=off

Peak #	RetTime [min]	Type	Width [min]	Area [mAU*s]	Height [mAU]	Area %
1	12.670	MF	0.3746	5724.11621	254.69371	49.6905
2	13.729	FM	0.3983	5795.41357	242.49443	50.3095

Totals : 1.15195e4 497.18814

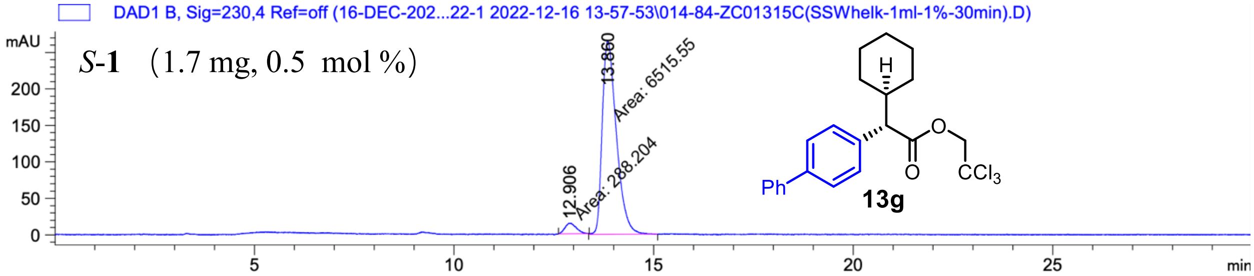


```
Signal 2: DAD1 B, Sig=230,4 Ref=off

Peak RetTime Type  Width     Area      Height     Area
  #   [min]        [min]   [mAU*s]     [mAU]        %
----|-------|----|-------|----------|----------|--------|
   1  12.906 MM     0.3295  288.20389   14.57774   4.2360
   2  13.860 FM     0.4105 6515.55420  264.52066  95.7640

Totals :                   6803.75809  279.09840
```

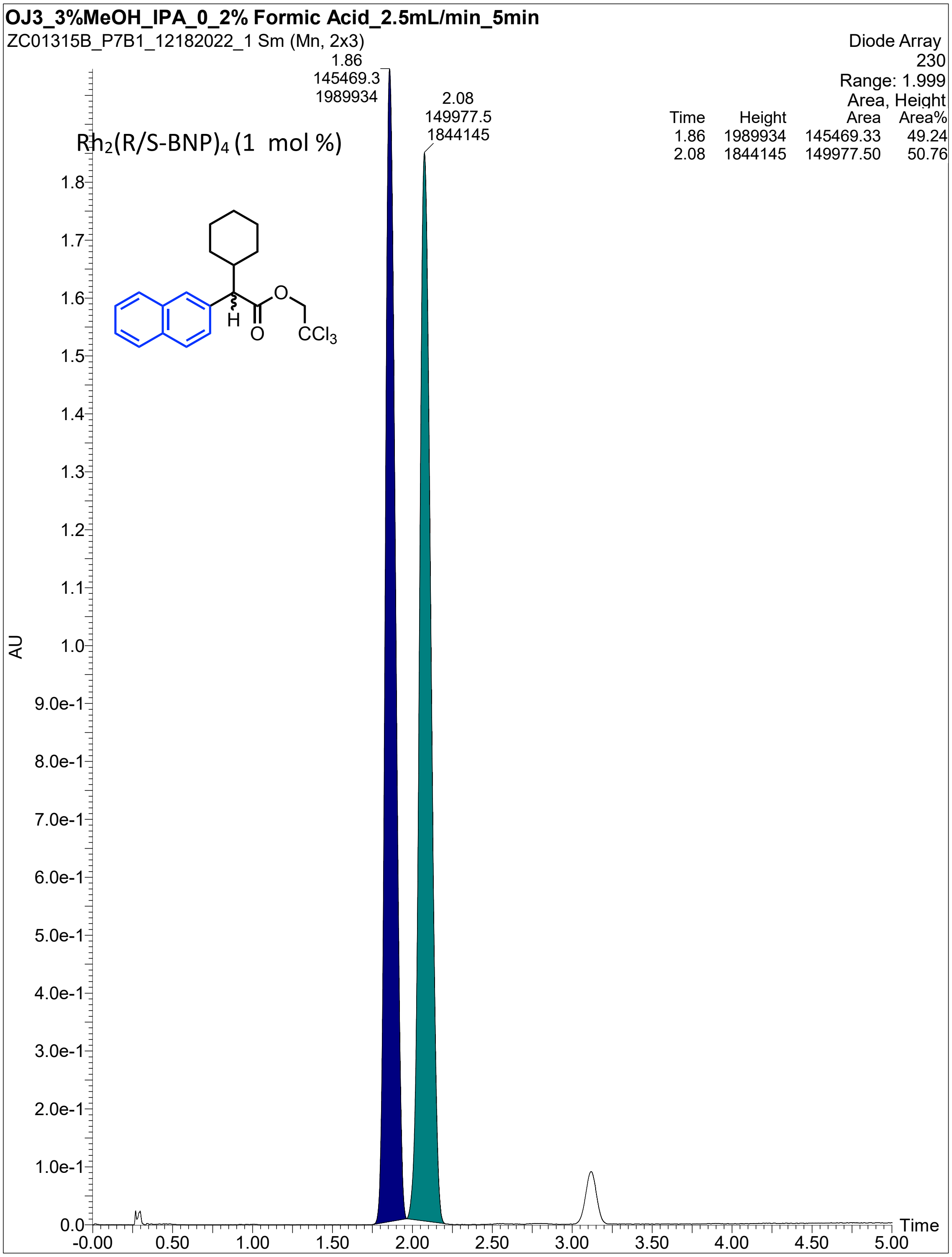

Time	Height	Area	Area%
1.86	1989934	145469.33	49.24
2.08	1844145	149977.50	50.76

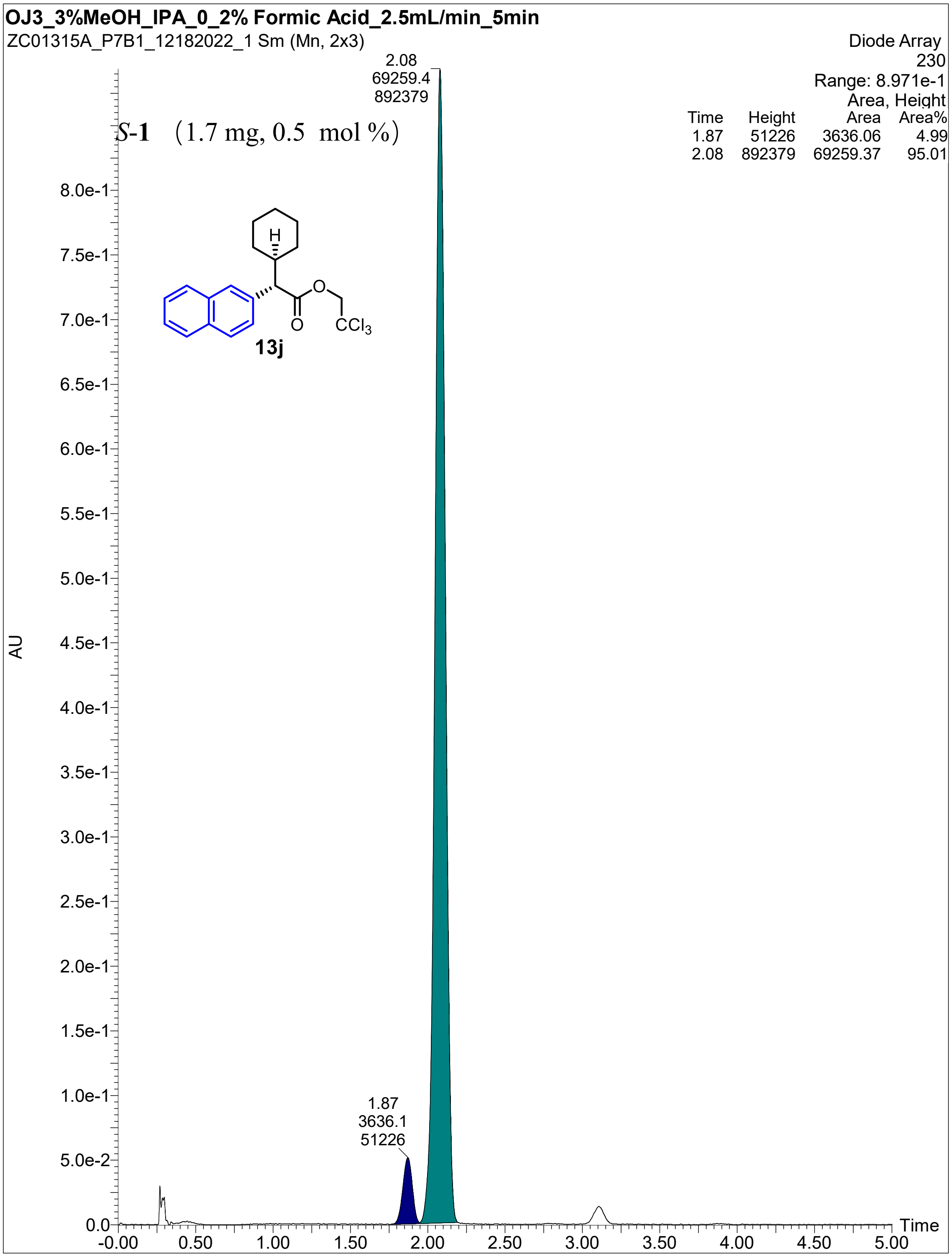

Time	Height	Area	Area%
1.87	51226	3636.06	4.99
2.08	892379	69259.37	95.01

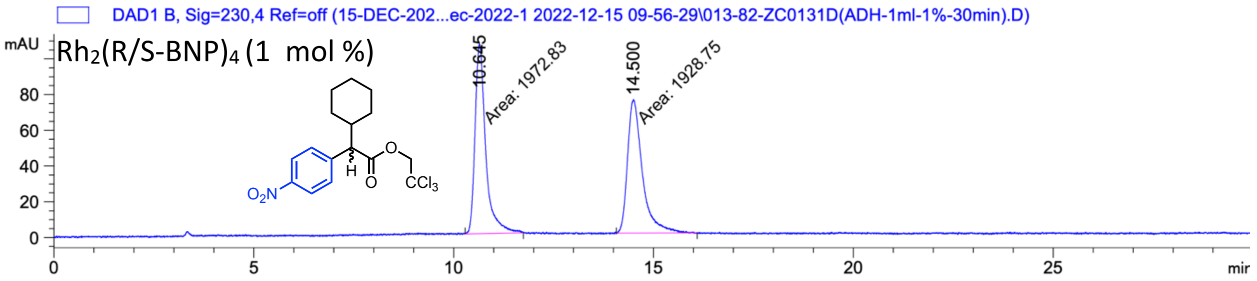

Signal 2: DAD1 B, Sig=230,4 Ref=off

Peak #	RetTime [min]	Type	Width [min]	Area [mAU*s]	Height [mAU]	Area %
1	10.645	MM	0.3096	1972.83044	106.19674	50.5650
2	14.500	MM	0.4304	1928.74609	74.68118	49.4350

Totals : 3901.57654 180.87792

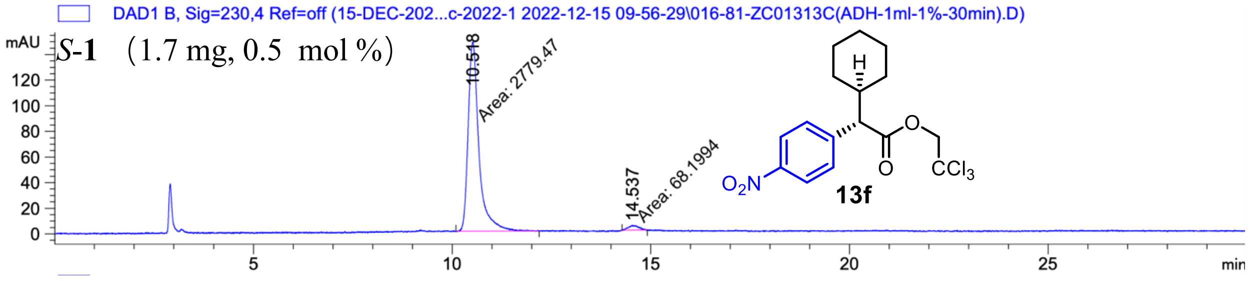

Signal 2: DAD1 B, Sig=230,4 Ref=off

Peak #	RetTime [min]	Type	Width [min]	Area [mAU*s]	Height [mAU]	Area %
1	10.518	MM	0.3139	2779.46729	147.58592	97.6051
2	14.537	MM	0.2936	68.19936	3.87118	2.3949

Totals : 2847.66664 151.45710

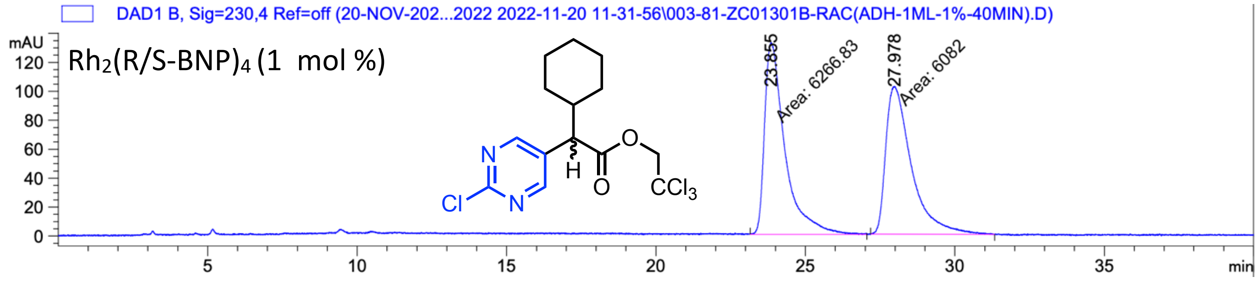

Signal 2: DAD1 B, Sig=230,4 Ref=off

Peak #	RetTime [min]	Type	Width [min]	Area [mAU*s]	Height [mAU]	Area %
1	23.855	MM	0.7888	6266.83350	132.40479	50.7484
2	27.978	MM	0.9920	6082.00049	102.18789	49.2516

Totals : 1.23488e4 234.59267

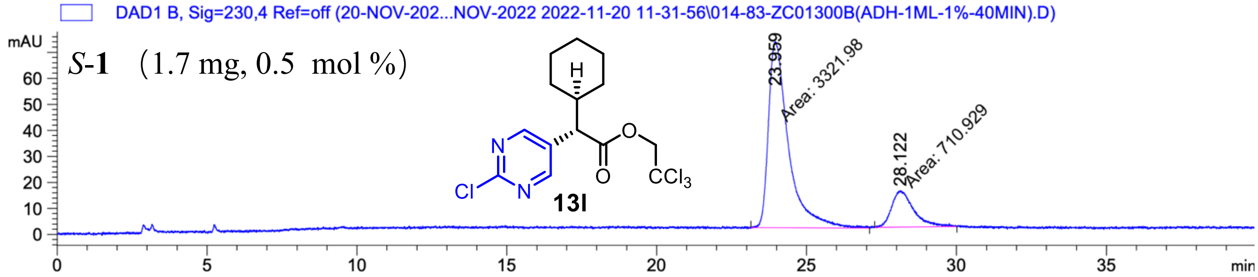

Signal 2: DAD1 B, Sig=230,4 Ref=off

Peak #	RetTime [min]	Type	Width [min]	Area [mAU*s]	Height [mAU]	Area %
1	23.959	MM	0.7734	3321.97729	71.58766	82.3718
2	28.122	MM	0.8478	710.92877	13.97667	17.6282

Totals : 4032.90607 85.56433

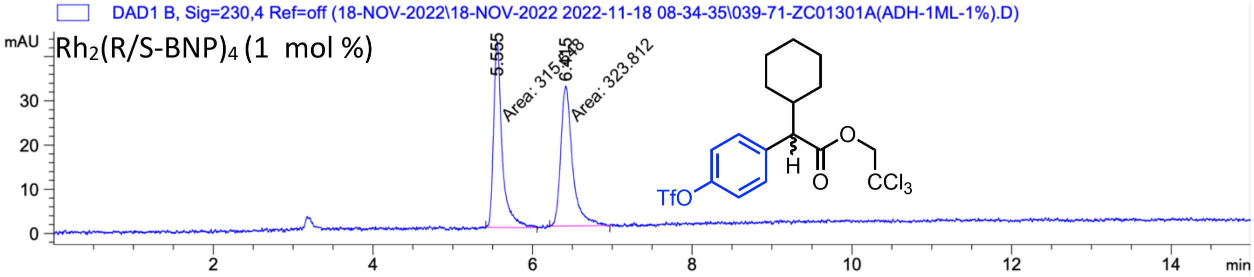

Signal 2: DAD1 B, Sig=230,4 Ref=off

Peak #	RetTime [min]	Type	Width [min]	Area [mAU*s]	Height [mAU]	Area %
1	5.555	MM	0.1249	315.54758	42.11271	49.3537
2	6.415	MM	0.1702	323.81174	31.70454	50.6463

Totals : 639.35931 73.81725

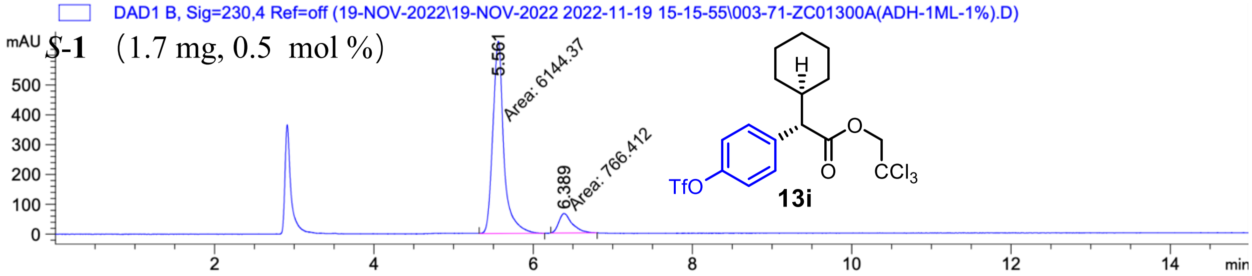

Signal 2: DAD1 B, Sig=230,4 Ref=off

Peak #	RetTime [min]	Type	Width [min]	Area [mAU*s]	Height [mAU]	Area %
1	5.561	MF	0.1591	6144.37012	643.80273	88.9099
2	6.389	MM	0.1958	766.41217	65.25003	11.0901

Totals : 6910.78229 709.05276

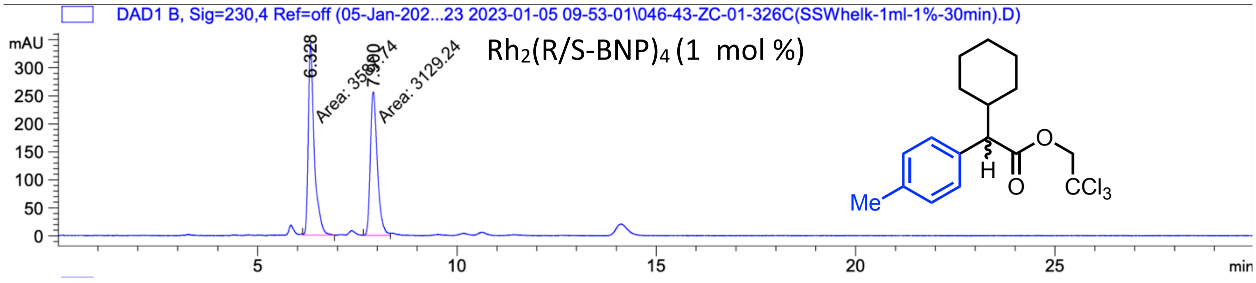

Signal 2: DAD1 B, Sig=230,4 Ref=off

Peak #	RetTime [min]	Type	Width [min]	Area [mAU*s]	Height [mAU]	Area %
1	6.328	MF	0.1743	3587.73706	342.99936	53.4130
2	7.900	MF	0.2037	3129.23730	255.99330	46.5870
Totals :				6716.97437	598.99266	

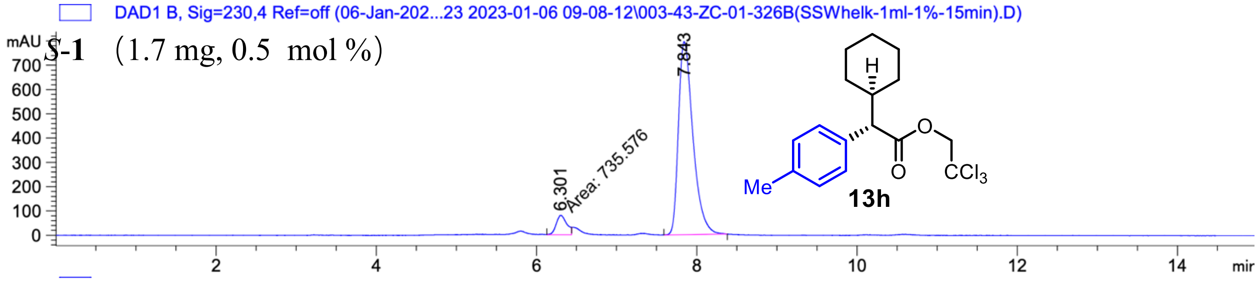

Signal 2: DAD1 B, Sig=230,4 Ref=off

Peak #	RetTime [min]	Type	Width [min]	Area [mAU*s]	Height [mAU]	Area %
1	6.301	MF	0.1539	735.57562	79.67082	6.8719
2	7.843	VV R	0.1541	9968.46680	795.79810	93.1281
Totals :				1.07040e4	875.46892	

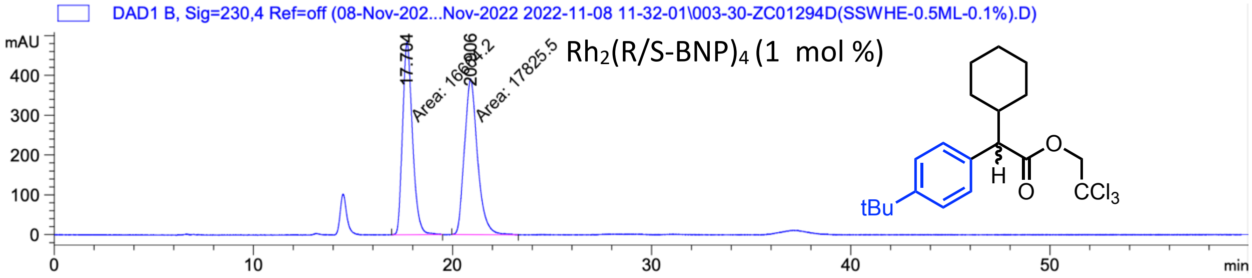

Signal 2: DAD1 B, Sig=230,4 Ref=off

Peak #	RetTime [min]	Type	Width [min]	Area [mAU*s]	Height [mAU]	Area %
1	17.704	MM	0.5737	1.66642e4	484.12158	48.3165
2	20.906	MM	0.7584	1.78255e4	391.71411	51.6835

Totals : 3.44897e4 875.83569

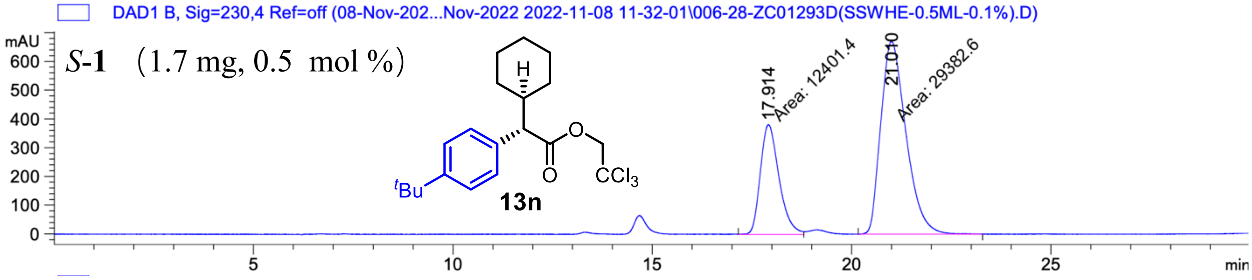

Signal 2: DAD1 B, Sig=230,4 Ref=off

Peak #	RetTime [min]	Type	Width [min]	Area [mAU*s]	Height [mAU]	Area %
1	17.914	MF	0.5414	1.24014e4	381.76724	29.6798
2	21.010	MM	0.7304	2.93826e4	670.48779	70.3202

Totals : 4.17840e4 1052.25504

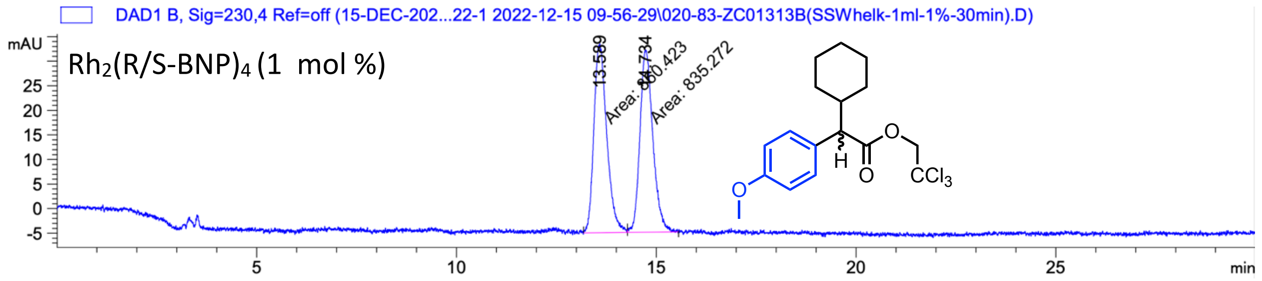

Signal 2: DAD1 B, Sig=230,4 Ref=off

Peak #	RetTime [min]	Type	Width [min]	Area [mAU*s]	Height [mAU]	Area %
1	13.589	MF	0.3730	860.42310	38.44910	50.7416
2	14.734	FM	0.3748	835.27246	37.14481	49.2584
Totals :				1695.69556	75.59391	

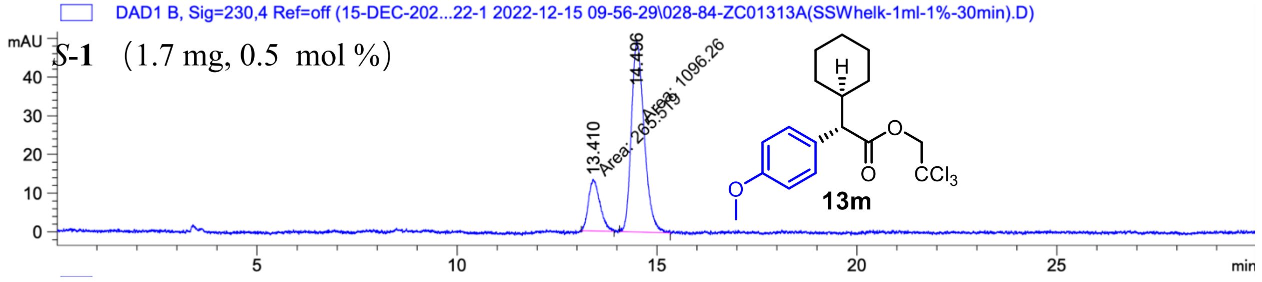

Signal 2: DAD1 B, Sig=230,4 Ref=off

Peak #	RetTime [min]	Type	Width [min]	Area [mAU*s]	Height [mAU]	Area %
1	13.410	MM	0.3315	265.51868	13.35117	19.4980
2	14.496	MM	0.3714	1096.25842	49.19279	80.5020

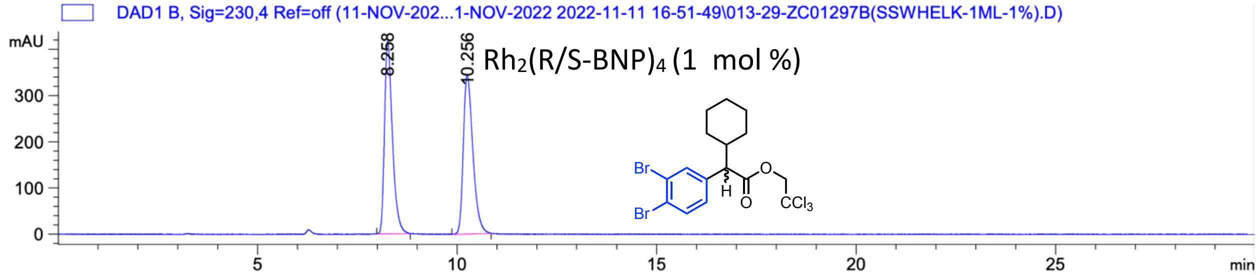

Signal 2: DAD1 B, Sig=230,4 Ref=off

Peak #	RetTime [min]	Type	Width [min]	Area [mAU*s]	Height [mAU]	Area %
1	8.258	VV R	0.1755	5745.78857	416.37619	50.0327
2	10.256	VV R	0.1987	5738.27344	345.66241	49.9673
Totals :				1.14841e4	762.03860	

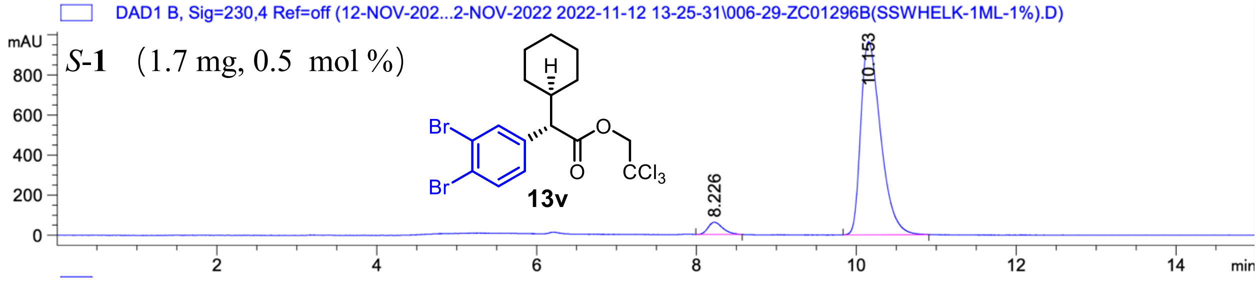

Signal 2: DAD1 B, Sig=230,4 Ref=off

Peak #	RetTime [min]	Type	Width [min]	Area [mAU*s]	Height [mAU]	Area %
1	8.226	VV R	0.1522	780.00122	61.17913	4.4815
2	10.153	VV R	0.2028	1.66248e4	963.69812	95.5185
Totals :				1.74048e4	1024.87725	

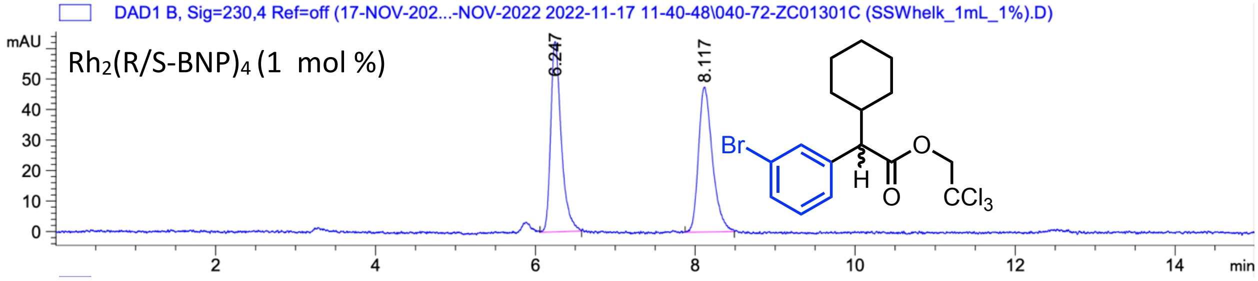

Signal 2: DAD1 B, Sig=230,4 Ref=off

Peak #	RetTime [min]	Type	Width [min]	Area [mAU*s]	Height [mAU]	Area %
1	6.247	VV R	0.1175	572.57837	62.42832	49.9126
2	8.117	VV R	0.1455	574.58264	47.53289	50.0874

Totals : 1147.16101 109.96121

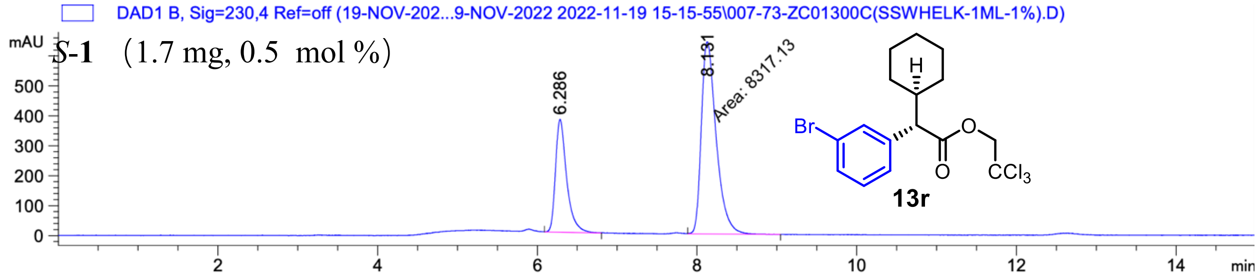

Signal 2: DAD1 B, Sig=230,4 Ref=off

Peak #	RetTime [min]	Type	Width [min]	Area [mAU*s]	Height [mAU]	Area %
1	6.286	VV R	0.1418	3649.03223	377.42004	30.4946
2	8.131	FM	0.2159	8317.13379	641.98505	69.5054

Totals : 1.19662e4 1019.40509

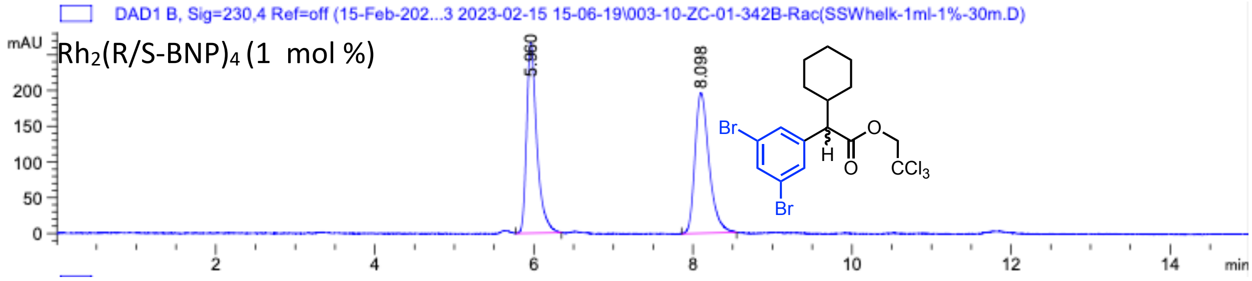

Signal 2: DAD1 B, Sig=230,4 Ref=off

Peak #	RetTime [min]	Type	Width [min]	Area [mAU*s]	Height [mAU]	Area %
1	5.960	VV R	0.1294	2381.18066	267.48785	49.6505
2	8.098	VV R	0.1549	2414.70044	196.49687	50.3495
Totals :				4795.88110	463.98473	

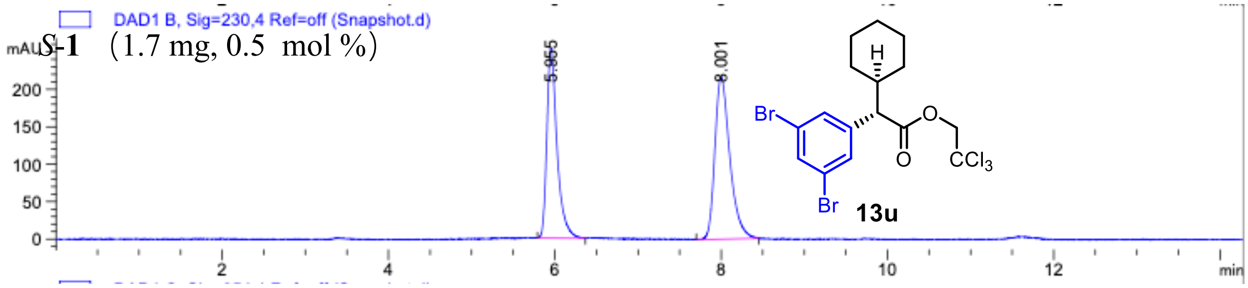

Signal 2: DAD1 B, Sig=230,4 Ref=off

Peak #	RetTime [min]	Type	Width [min]	Area [mAU*s]	Height [mAU]	Area %
1	5.955	VV R	0.1247	2158.93140	253.99509	44.3262
2	8.001	VV R	0.1507	2711.62549	216.33643	55.6738
Totals :				4870.55688	470.33151	

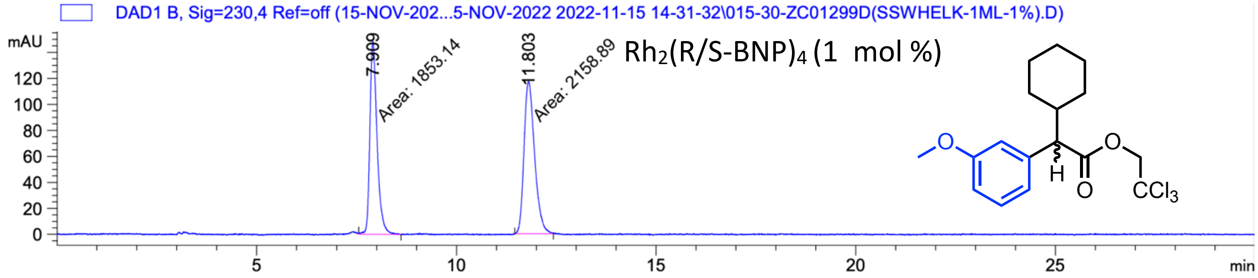

Signal 2: DAD1 B, Sig=230,4 Ref=off

Peak #	RetTime [min]	Type	Width [min]	Area [mAU*s]	Height [mAU]	Area %
1	7.909	MM	0.2082	1853.14453	148.32457	46.1896
2	11.803	MM	0.3073	2158.89136	117.08215	53.8104
Totals :				4012.03589	265.40672	

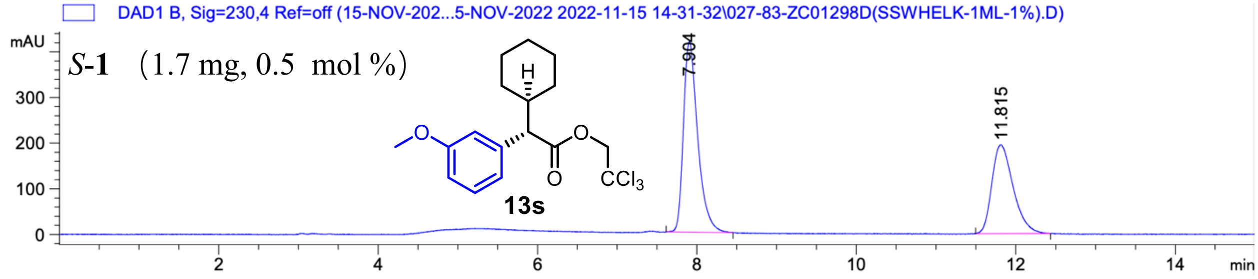

Signal 2: DAD1 B, Sig=230,4 Ref=off

Peak #	RetTime [min]	Type	Width [min]	Area [mAU*s]	Height [mAU]	Area %
1	7.904	VV R	0.1689	5189.76367	417.85678	58.9233
2	11.815	VV R	0.2255	3617.89282	194.53516	41.0767
Totals :				8807.65649	612.39194	

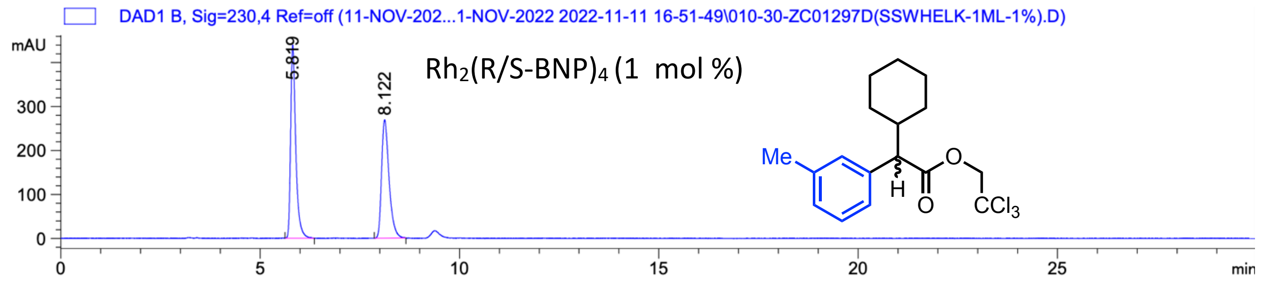

Signal 2: DAD1 B, Sig=230,4 Ref=off

Peak #	RetTime [min]	Type	Width [min]	Area [mAU*s]	Height [mAU]	Area %
1	5.819	VV R	0.1260	3899.34741	439.39630	52.5942
2	8.122	BV R	0.1802	3514.67505	269.45767	47.4058

Totals : 7414.02246 708.85397

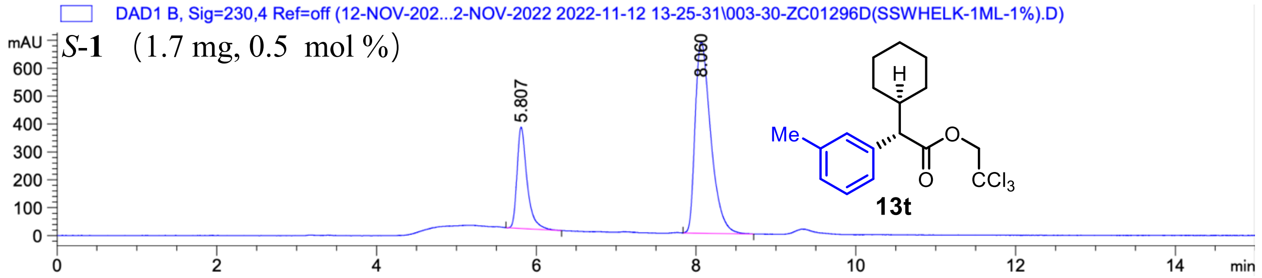

Signal 2: DAD1 B, Sig=230,4 Ref=off

Peak #	RetTime [min]	Type	Width [min]	Area [mAU*s]	Height [mAU]	Area %
1	5.807	VV R	0.1272	3187.38013	363.80576	25.5054
2	8.060	BV R	0.1638	9309.49219	684.00671	74.4946

Totals : 1.24969e4 1047.81247

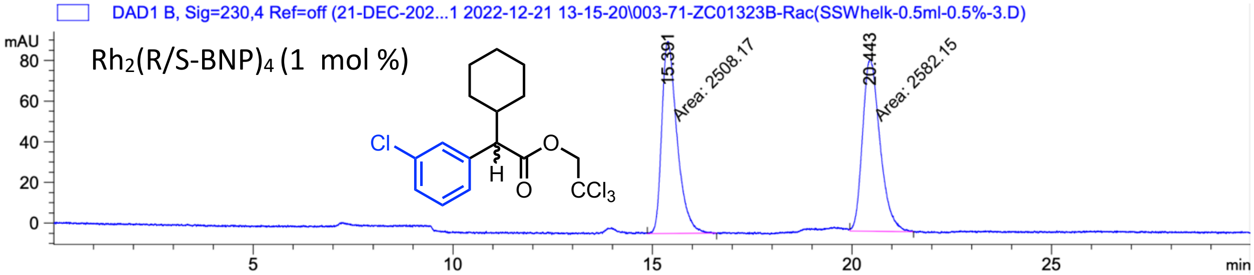

Signal 2: DAD1 B, Sig=230,4 Ref=off

Peak #	RetTime [min]	Type	Width [min]	Area [mAU*s]	Height [mAU]	Area %
1	15.391	MM	0.4426	2508.16650	94.45625	49.2733
2	20.443	MM	0.5083	2582.15430	84.66407	50.7267
Totals :				5090.32080	179.12032	

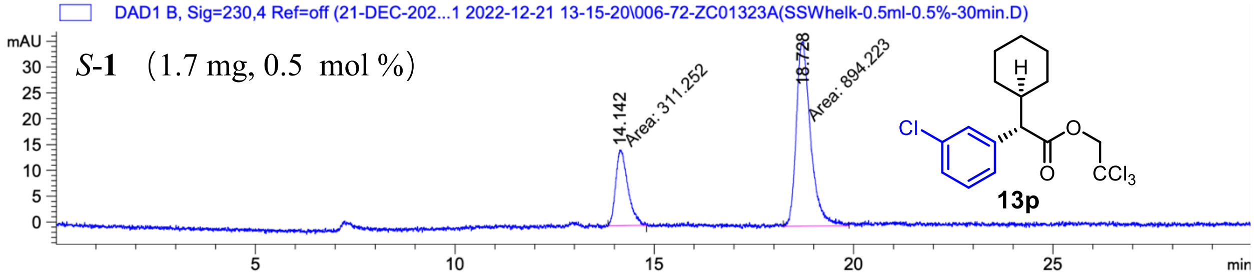

Signal 2: DAD1 B, Sig=230,4 Ref=off

Peak #	RetTime [min]	Type	Width [min]	Area [mAU*s]	Height [mAU]	Area %
1	14.142	MM	0.3542	311.25165	14.64481	25.8198
2	18.728	MM	0.4165	894.22333	35.78009	74.1802
Totals :				1205.47498	50.42490	

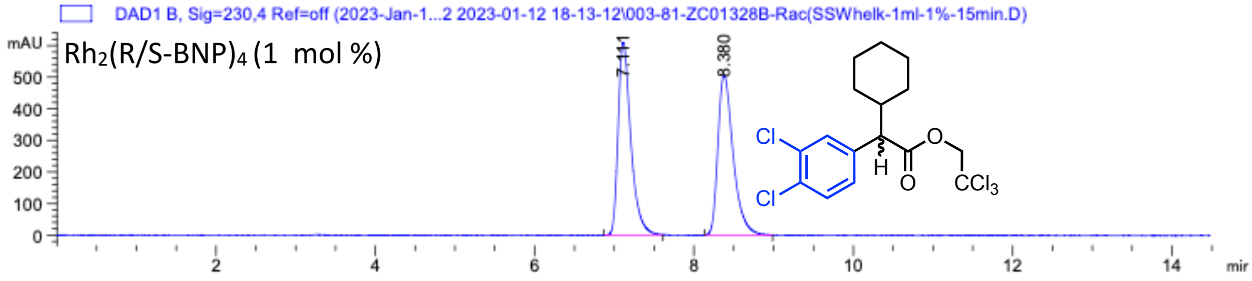

Signal 2: DAD1 B, Sig=230,4 Ref=off

Peak #	RetTime [min]	Type	Width [min]	Area [mAU*s]	Height [mAU]	Area %
1	7.111	VV R	0.1481	6751.95752	610.09143	49.9705
2	8.380	BV R	0.1667	6759.93555	506.53357	50.0295
Totals :				1.35119e4	1116.62500	

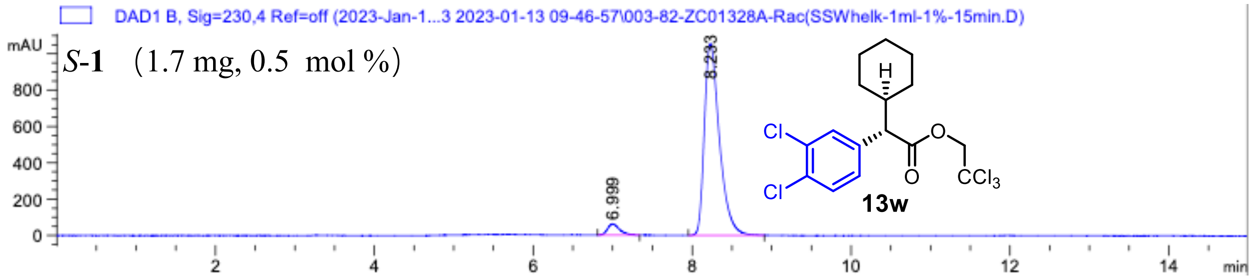

Signal 2: DAD1 B, Sig=230,4 Ref=off

Peak #	RetTime [min]	Type	Width [min]	Area [mAU*s]	Height [mAU]	Area %
1	6.999	VV R	0.1254	660.69763	62.19407	4.5437
2	8.233	VV R	0.1575	1.38802e4	1055.07837	95.4563
Totals :				1.45409e4	1117.27244	

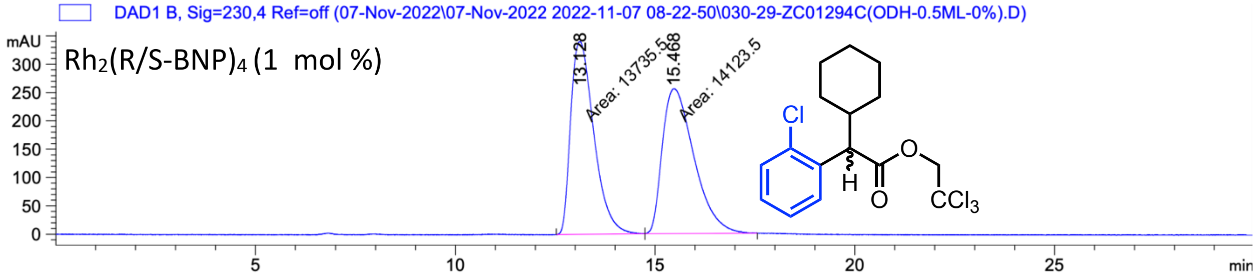

Signal 2: DAD1 B, Sig=230,4 Ref=off

Peak #	RetTime [min]	Type	Width [min]	Area [mAU*s]	Height [mAU]	Area %
1	13.128	MM	0.6722	1.37355e4	340.56323	49.3036
2	15.468	MM	0.9200	1.41235e4	255.85495	50.6964
Totals :				2.78590e4	596.41818	

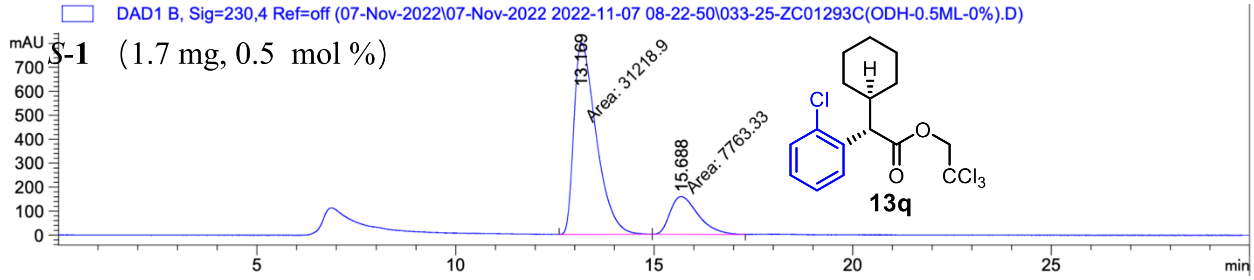

Signal 2: DAD1 B, Sig=230,4 Ref=off

Peak #	RetTime [min]	Type	Width [min]	Area [mAU*s]	Height [mAU]	Area %
1	13.169	MM	0.6493	3.12189e4	801.39178	80.0850
2	15.688	MM	0.8182	7763.33154	158.14745	19.9150

Totals : 3.89823e4 959.53923

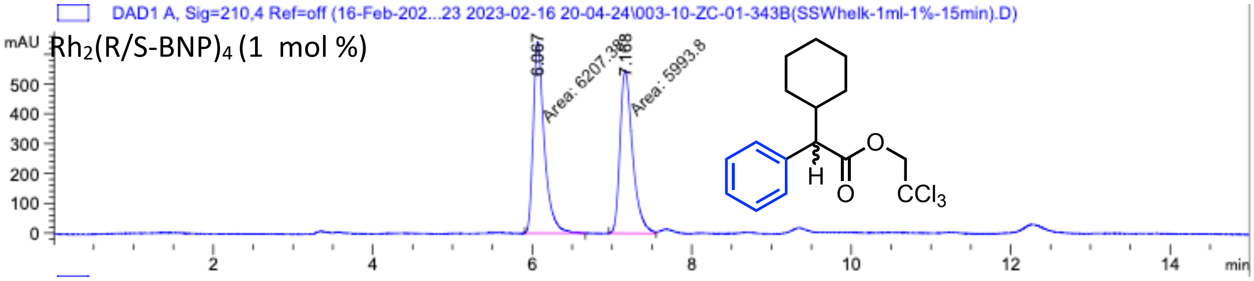

Signal 1: DAD1 A, Sig=210,4 Ref=off

Peak #	RetTime [min]	Type	Width [min]	Area [mAU*s]	Height [mAU]	Area %
1	6.067	MM	0.1609	6207.37695	642.84515	50.8752
2	7.168	MF	0.1829	5993.80420	546.12738	49.1248

Totals : 1.22012e4 1188.97253

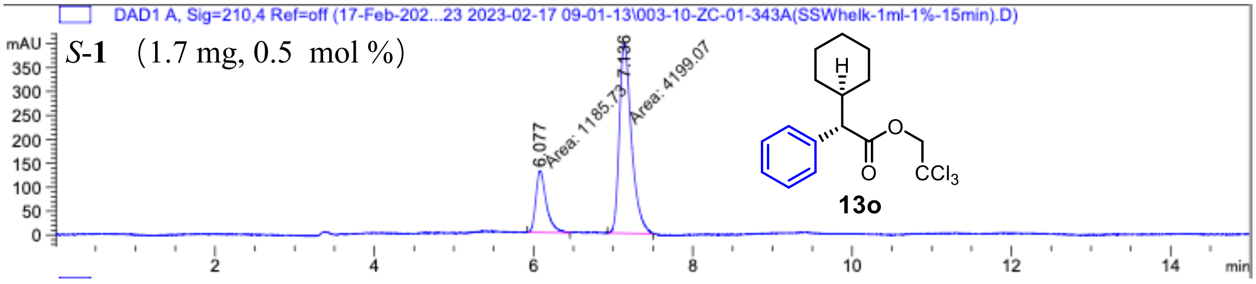

Signal 1: DAD1 A, Sig=210,4 Ref=off

Peak #	RetTime [min]	Type	Width [min]	Area [mAU*s]	Height [mAU]	Area %
1	6.077	MM	0.1528	1185.73352	129.29948	22.0200
2	7.136	MM	0.1763	4199.06689	396.97330	77.9800

Totals : 5384.80042 526.27278

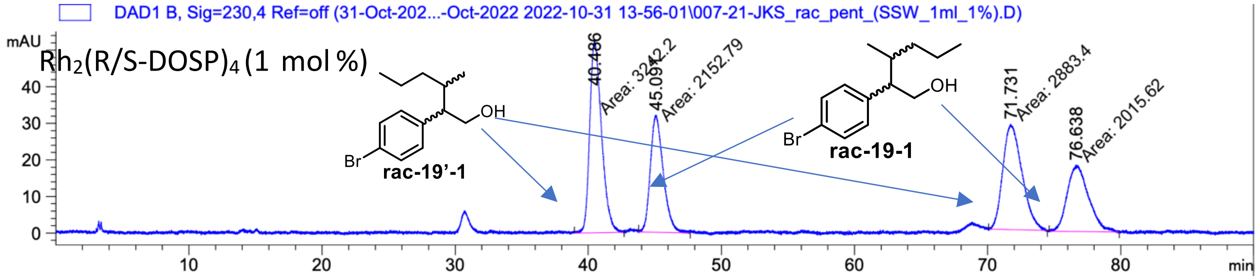

Signal 2: DAD1 B, Sig=230,4 Ref=off

Peak #	RetTime [min]	Type	Width [min]	Area [mAU*s]	Height [mAU]	Area %
1	40.486	MM	1.0320	3242.19922	52.36084	31.4960
2	45.091	MM	1.1185	2152.78516	32.07850	20.9130
3	71.731	MM	1.6665	2883.39941	28.83657	28.0105
4	76.638	MM	1.8445	2015.61963	18.21255	19.5805
Totals :				1.02940e4	131.48845	

For **Rh_2(*R/S*-DOSP)$_4$** catalyzed **racemic** reaction, the **none-shielded** product was the **major** product ***rac*-19'-1**, which corresponds to the **first and third** signals in HPLC analysis. Same NMR analysis was reported.[7]

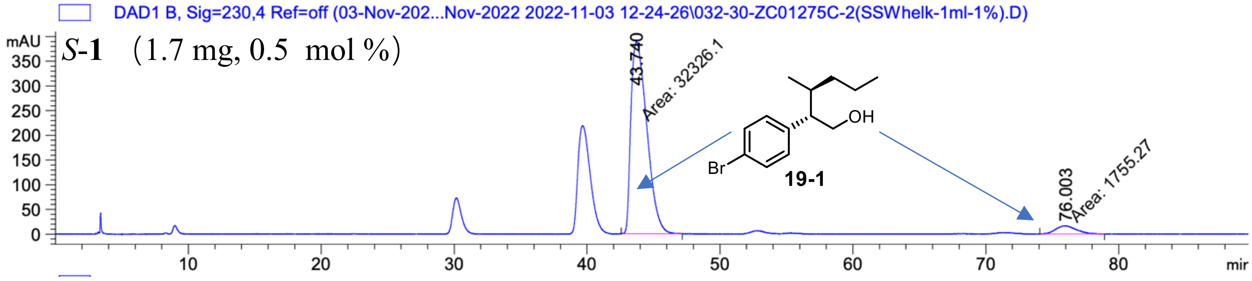

Signal 2: DAD1 B, Sig=230,4 Ref=off

Peak #	RetTime [min]	Type	Width [min]	Area [mAU*s]	Height [mAU]	Area %
1	43.740	MM	1.3828	3.23261e4	389.62701	94.8498
2	76.003	MM	1.7897	1755.26611	16.34590	5.1502
Totals :				3.40814e4	405.97292	

For **1** catalyzed reaction, the **shielded** product was the **major** product **19-1**, which corresponds to the **second and forth** signals in HPLC analysis.

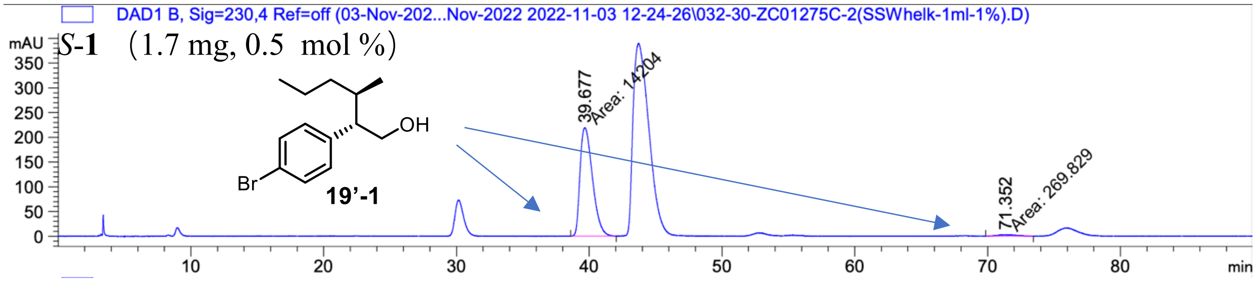


```
Signal 2: DAD1 B, Sig=230,4 Ref=off

Peak RetTime Type  Width     Area       Height     Area
  #   [min]        [min]    [mAU*s]     [mAU]       %
----|-------|----|-------|----------|----------|--------|
   1  39.677 MM    1.0797 1.42040e4   219.26794  98.1357
   2  71.352 MM    1.5045  269.82935    2.98907   1.8643

Totals :                  1.44738e4   222.25701
```

For **1** catalyzed reaction, the **none-shielded** product was the **minor** product **19'-1**, which corresponds to the first and third signals in HPLC analysis.

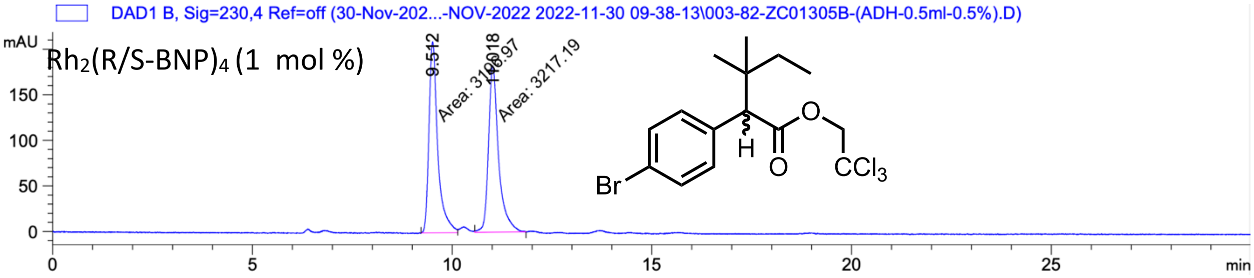

Signal 2: DAD1 B, Sig=230,4 Ref=off

Peak #	RetTime [min]	Type	Width [min]	Area [mAU*s]	Height [mAU]	Area %
1	9.512	MF	0.2542	3196.97021	209.57841	49.8424
2	11.018	FM	0.2941	3217.18750	182.34601	50.1576
Totals :				6414.15771	391.92442	

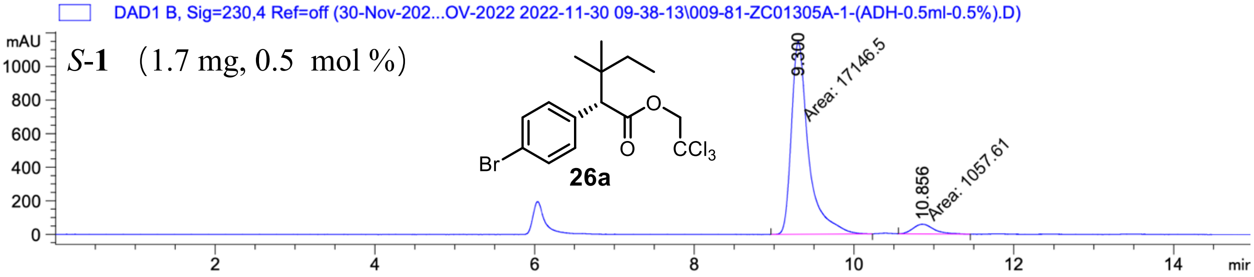

Signal 2: DAD1 B, Sig=230,4 Ref=off

Peak #	RetTime [min]	Type	Width [min]	Area [mAU*s]	Height [mAU]	Area %
1	9.300	MM	0.2483	1.71465e4	1150.82727	94.1903
2	10.856	MM	0.3031	1057.60950	58.14907	5.8097
Totals :				1.82041e4	1208.97634	

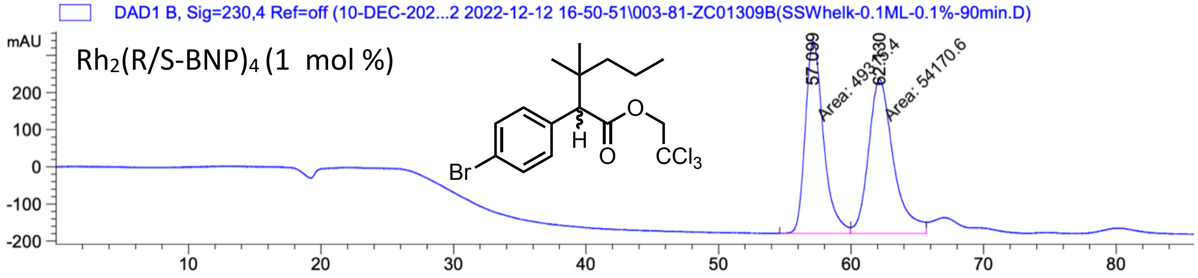


```
Signal 2: DAD1 B, Sig=230,4 Ref=off

Peak RetTime Type  Width     Area       Height      Area
  #   [min]        [min]   [mAU*s]      [mAU]        %
----|-------|----|-------|----------|----------|--------|
   1  57.099 MF     1.5904 4.93154e4  516.80493  47.6542
   2  62.130 MF     2.1682 5.41706e4  416.40005  52.3458

Totals :                   1.03486e5  933.20499
```

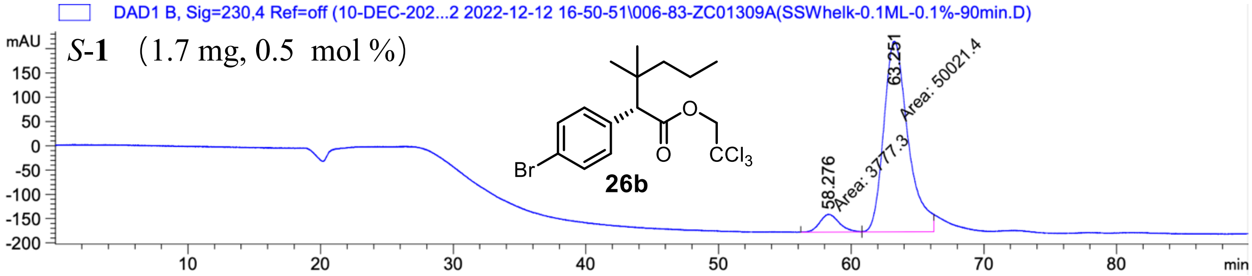


```
Signal 2: DAD1 B, Sig=230,4 Ref=off

Peak RetTime Type  Width     Area       Height      Area
  #   [min]        [min]   [mAU*s]      [mAU]        %
----|-------|----|-------|----------|----------|--------|
   1  58.276 MF     1.6853 3777.29712   37.35490   7.0212
   2  63.251 MF     2.1234 5.00214e4  392.61926  92.9788

Totals :                   5.37987e4  429.97416
```

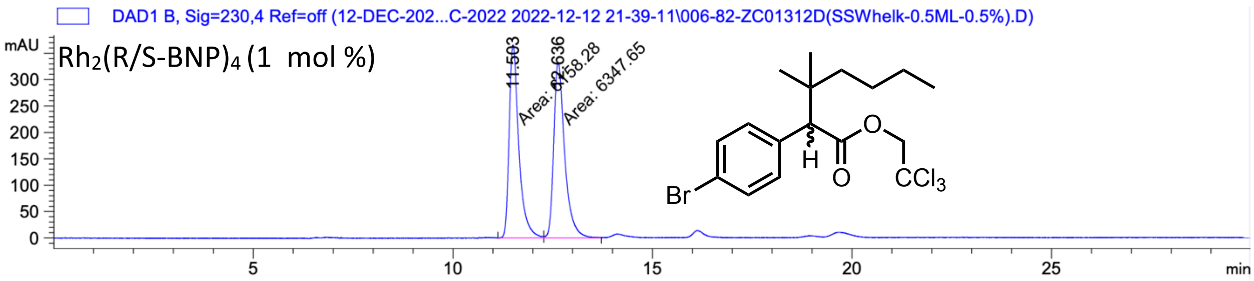

Signal 2: DAD1 B, Sig=230,4 Ref=off

Peak #	RetTime [min]	Type	Width [min]	Area [mAU*s]	Height [mAU]	Area %
1	11.503	MF	0.2809	6158.28223	365.37814	49.2429
2	12.636	FM	0.3188	6347.65332	331.80600	50.7571

Totals : 1.25059e4 697.18414

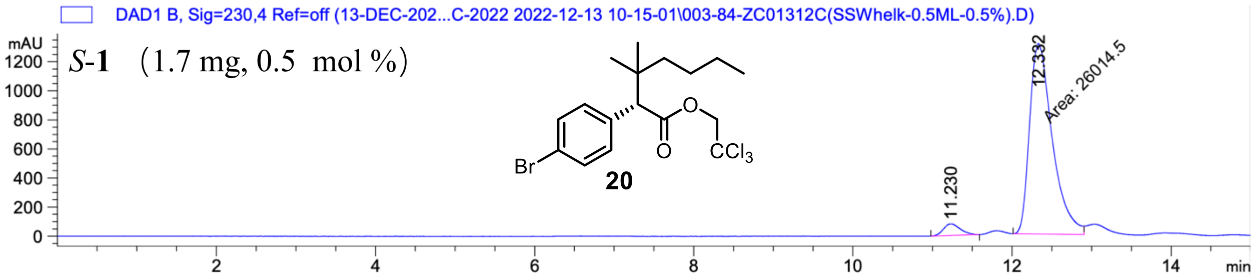

Signal 2: DAD1 B, Sig=230,4 Ref=off

Peak #	RetTime [min]	Type	Width [min]	Area [mAU*s]	Height [mAU]	Area %
1	11.230	VV R	0.1758	1174.03076	79.97372	4.3181
2	12.332	MF	0.3305	2.60145e4	1311.82336	95.6819

Totals : 2.71886e4 1391.79709

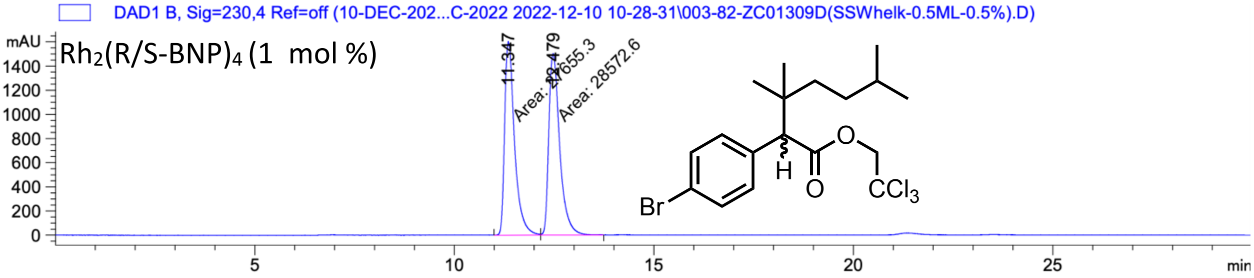

Signal 2: DAD1 B, Sig=230,4 Ref=off

Peak #	RetTime [min]	Type	Width [min]	Area [mAU*s]	Height [mAU]	Area %
1	11.347	MF	0.2874	2.76553e4	1604.03210	49.1843
2	12.479	FM	0.3166	2.85726e4	1504.18420	50.8157

Totals : 5.62280e4 3108.21631

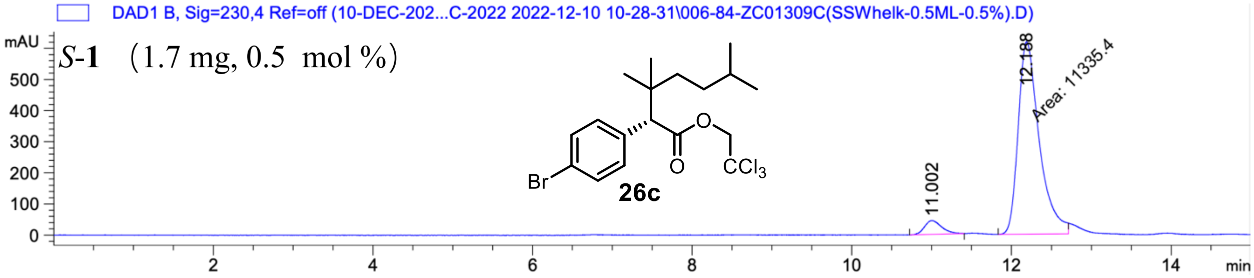

Signal 2: DAD1 B, Sig=230,4 Ref=off

Peak #	RetTime [min]	Type	Width [min]	Area [mAU*s]	Height [mAU]	Area %
1	11.002	VV R	0.1749	662.71100	44.59751	5.5234
2	12.188	MF	0.3055	1.13354e4	618.47815	94.4766

Totals : 1.19981e4 663.07566

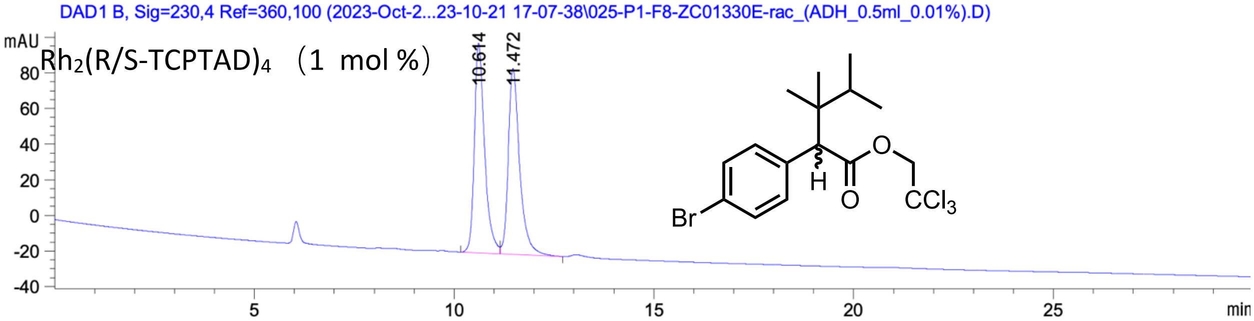

Signal 2: DAD1 B, Sig=230,4 Ref=360,100

Peak #	RetTime [min]	Type	Width [min]	Area [mAU*s]	Height [mAU]	Area %
1	10.614	BV	0.2679	2096.98804	117.69333	50.2802
2	11.472	VB	0.2958	2073.61548	104.36526	49.7198

Totals : 4170.60352 222.05859

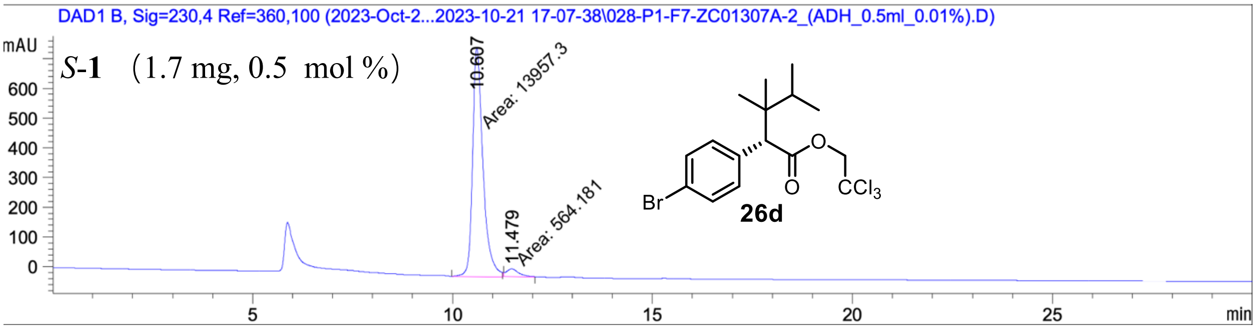

Signal 2: DAD1 B, Sig=230,4 Ref=360,100

Peak #	RetTime [min]	Type	Width [min]	Area [mAU*s]	Height [mAU]	Area %
1	10.607	MM	0.3016	1.39573e4	771.33594	96.1148
2	11.479	MM	0.3611	564.18079	26.04068	3.8852

Totals : 1.45214e4 797.37662

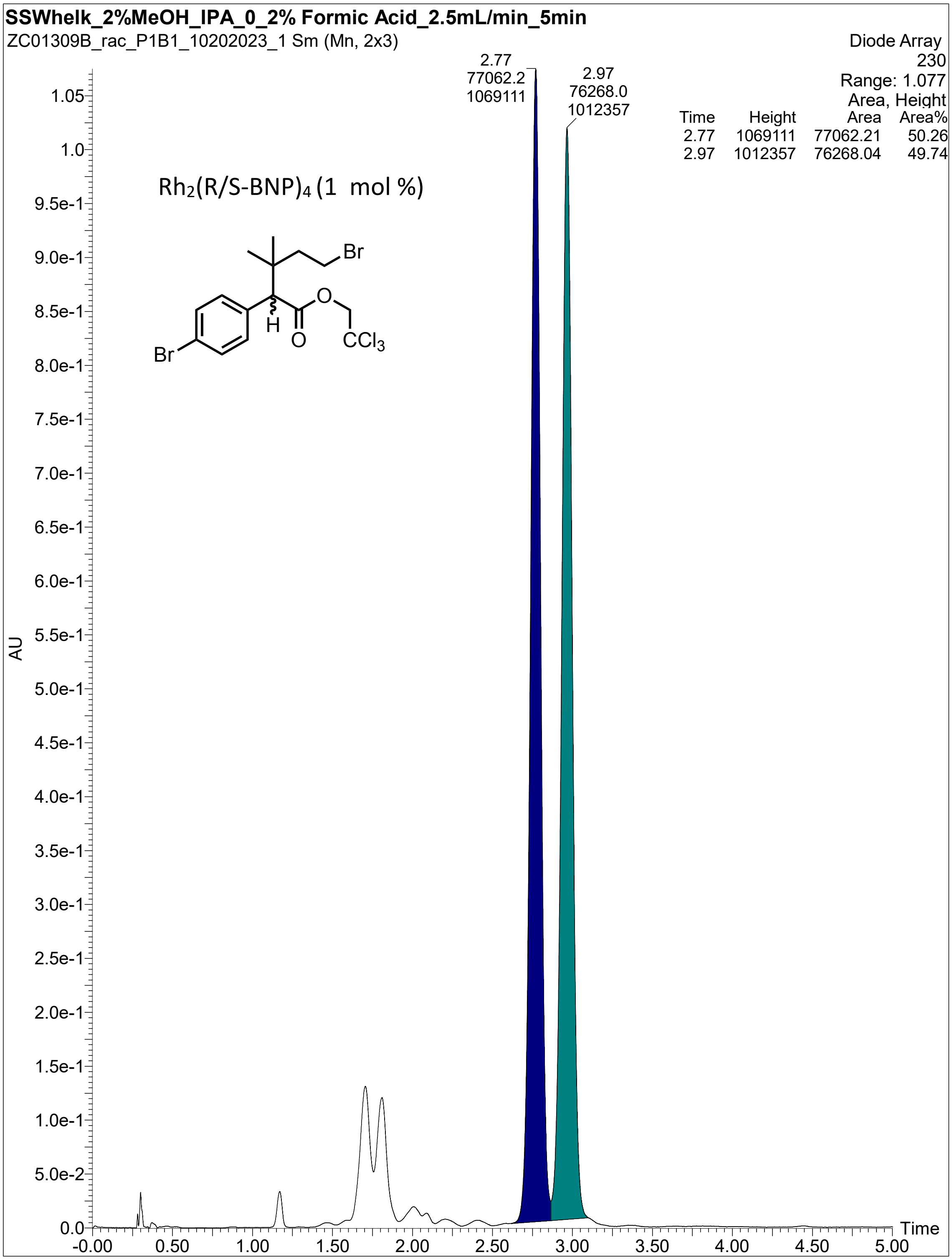

Time	Height	Area	Area%
2.77	1069111	77062.21	50.26
2.97	1012357	76268.04	49.74

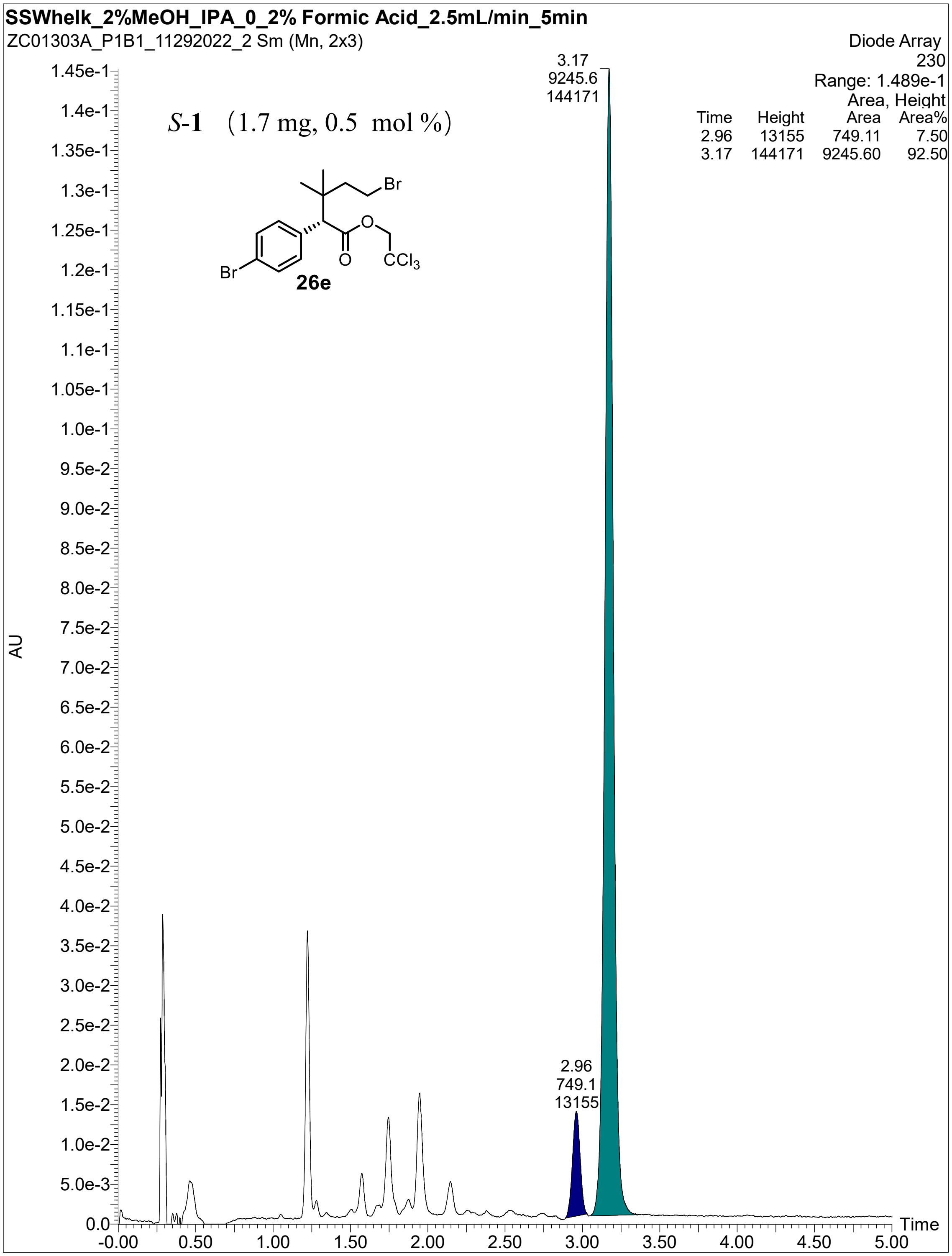

Time	Height	Area	Area%
2.96	13155	749.11	7.50
3.17	144171	9245.60	92.50

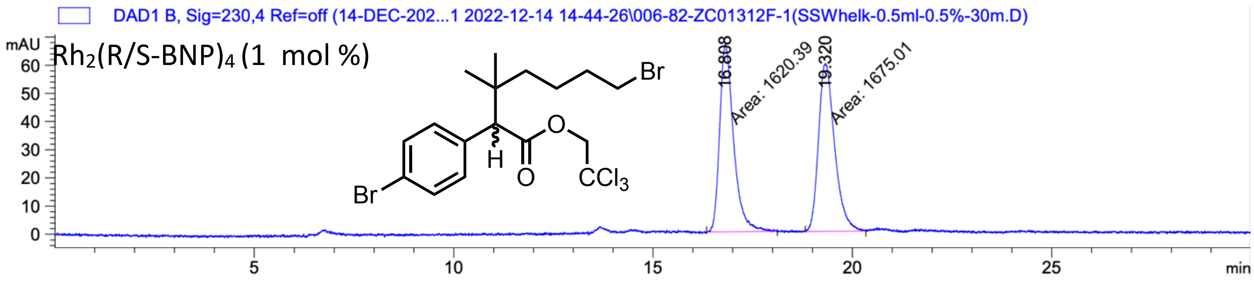

Signal 2: DAD1 B, Sig=230,4 Ref=off

Peak #	RetTime [min]	Type	Width [min]	Area [mAU*s]	Height [mAU]	Area %
1	16.808	MM	0.4061	1620.39368	66.50427	49.1714
2	19.320	MM	0.4693	1675.00537	59.49032	50.8286

Totals : 3295.39905 125.99459

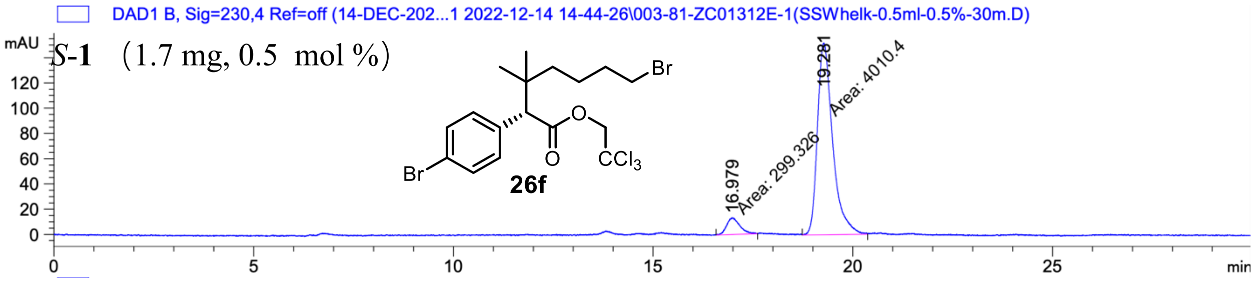

Signal 2: DAD1 B, Sig=230,4 Ref=off

Peak #	RetTime [min]	Type	Width [min]	Area [mAU*s]	Height [mAU]	Area %
1	16.979	MM	0.3738	299.32608	13.34658	6.9454
2	19.281	MM	0.4399	4010.39526	151.95670	93.0546

Totals : 4309.72134 165.30328

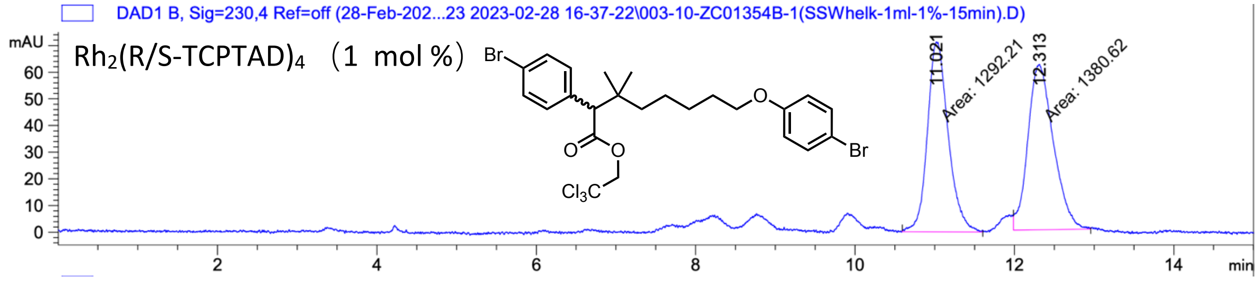

Signal 2: DAD1 B, Sig=230,4 Ref=off

Peak #	RetTime [min]	Type	Width [min]	Area [mAU*s]	Height [mAU]	Area %
1	11.021	MM	0.3026	1292.20630	71.17538	48.3460
2	12.313	MM	0.3711	1380.62341	62.01189	51.6540
Totals :				2672.82971	133.18727	

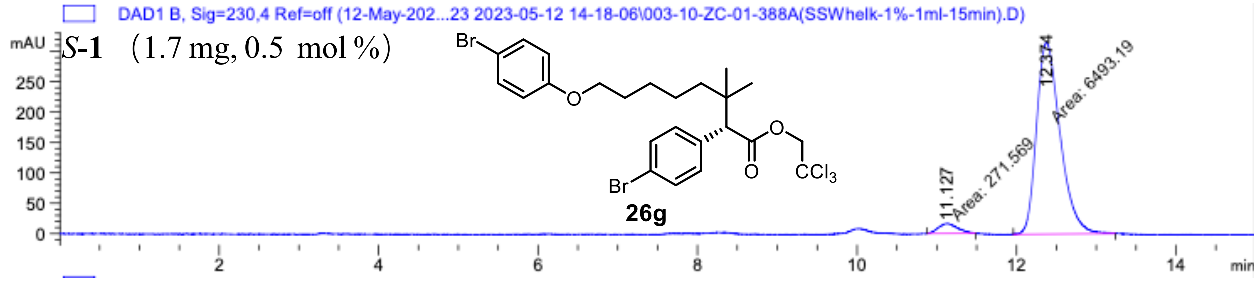

Signal 2: DAD1 B, Sig=230,4 Ref=off

Peak #	RetTime [min]	Type	Width [min]	Area [mAU*s]	Height [mAU]	Area %
1	11.127	MM	0.2773	271.56931	16.32406	4.0145
2	12.374	MM	0.3431	6493.18701	315.42932	95.9855
Totals :				6764.75632	331.75338	

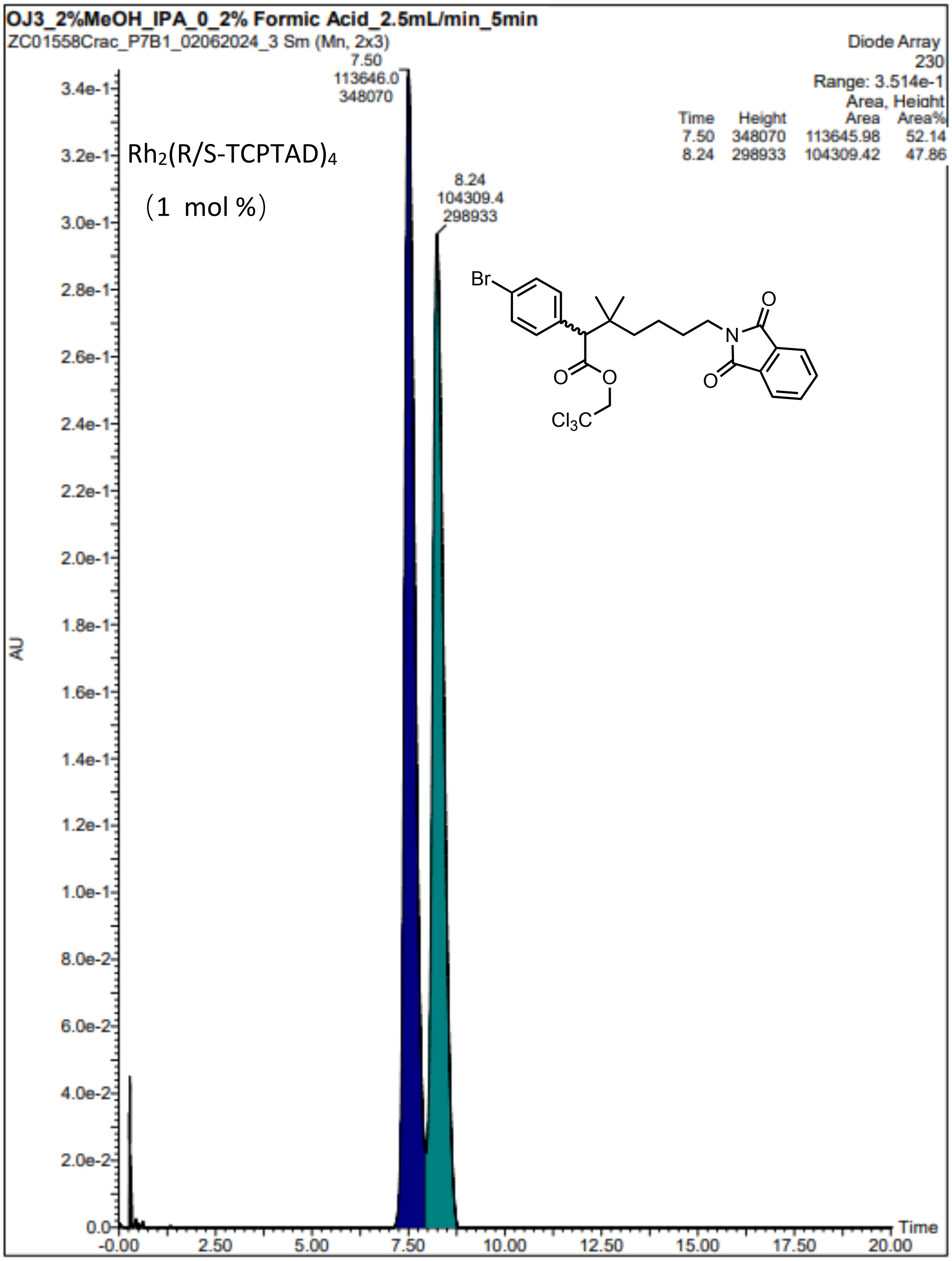
OJ3_2%MeOH_IPA_0_2% Formic Acid_2.5mL/min_5min
ZC01558Crac_P7B1_02062024_3 Sm (Mn, 2x3)
Diode Array
230
Range: 3.514e-1
Area, Height
Time Height Area Area%
7.50 348070 113645.98 52.14
8.24 298933 104309.42 47.86
7.50
113646.0
348070
8.24
104309.4
298933
Rh2(R/S-TCPTAD)4
(1 mol %)
Br
N
O
O
O
O
Cl3C
AU
Time

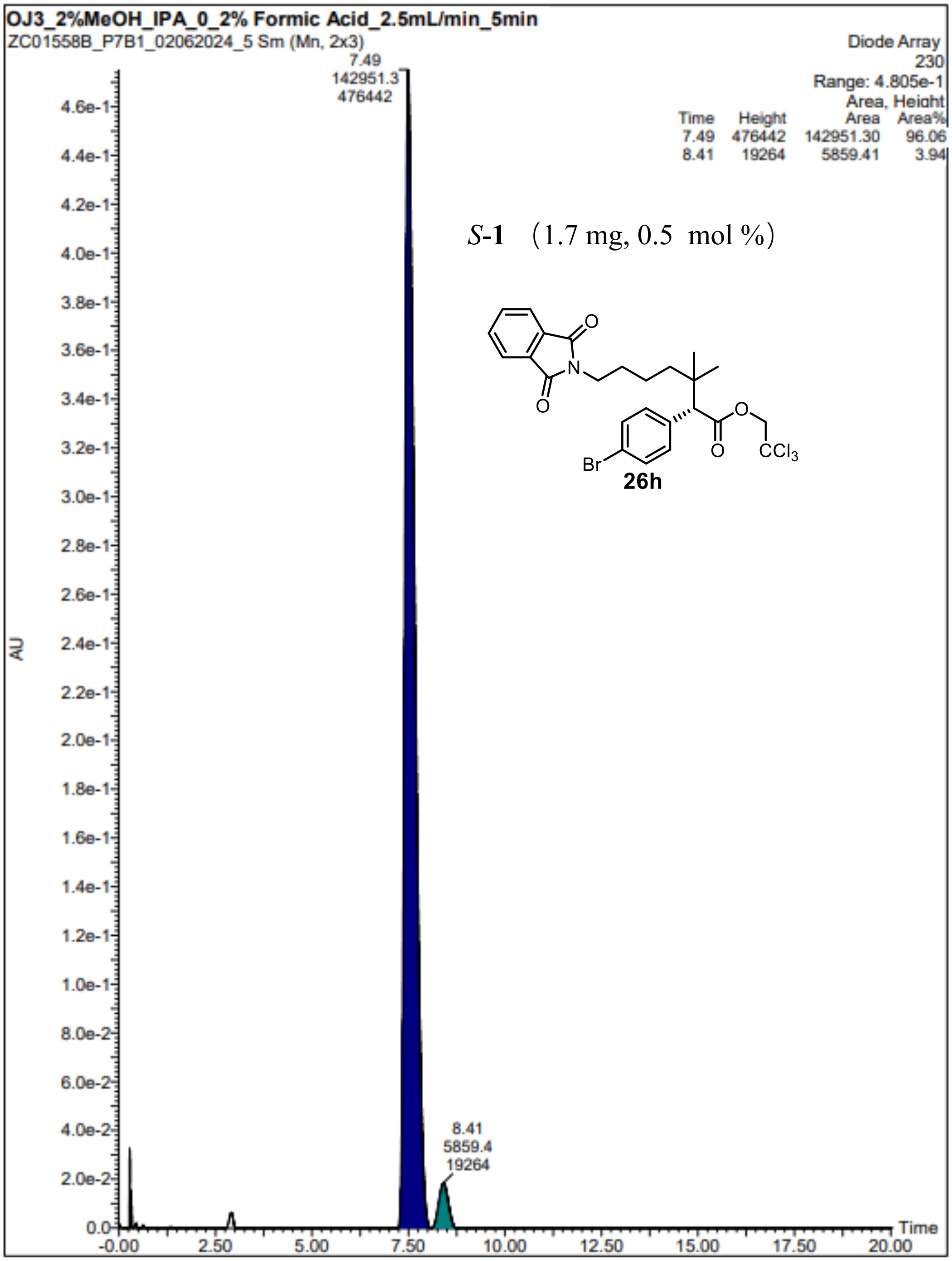
OJ3_2%MeOH_IPA_0_2% Formic Acid_2.5mL/min_5min
ZC01558B_P7B1_02062024_5 Sm (Mn, 2x3)
Diode Array
230
Range: 4.805e-1
Area, Height
Time Height Area Area%
7.49 476442 142951.30 96.06
8.41 19264 5859.41 3.94
7.49
142951.3
476442
8.41
5859.4
19264
S-1 (1.7 mg, 0.5 mol %)
Br
CCl3
26h
AU
Time

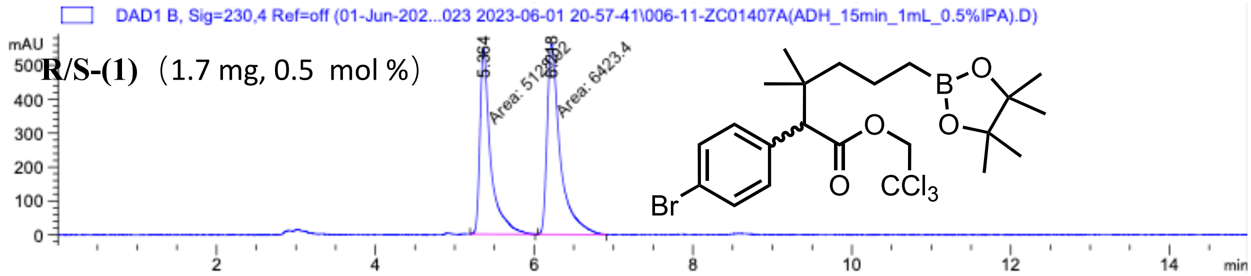

Signal 2: DAD1 B, Sig=230,4 Ref=off

Peak #	RetTime [min]	Type	Width [min]	Area [mAU*s]	Height [mAU]	Area %
1	5.364	MM	0.1569	5128.92188	544.98413	44.3973
2	6.218	MM	0.1905	6423.40283	562.04144	55.6027
Totals :				1.15523e4	1107.02557	

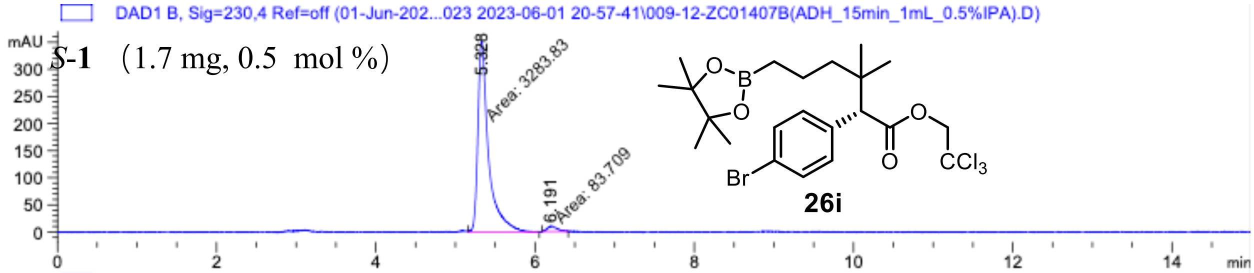

Signal 2: DAD1 B, Sig=230,4 Ref=off

Peak #	RetTime [min]	Type	Width [min]	Area [mAU*s]	Height [mAU]	Area %
1	5.328	MM	0.1554	3283.83081	352.21878	97.5142
2	6.191	MM	0.1412	83.70901	9.88044	2.4858
Totals :				3367.53983	362.09922	

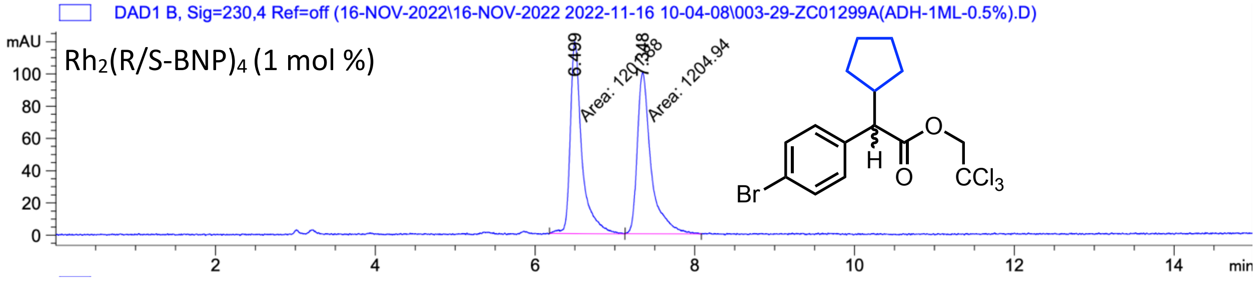

Signal 2: DAD1 B, Sig=230,4 Ref=off

Peak #	RetTime [min]	Type	Width [min]	Area [mAU*s]	Height [mAU]	Area %
1	6.499	MM	0.1682	1201.87817	119.10645	49.9364
2	7.348	MM	0.2007	1204.93994	100.07652	50.0636
Totals :				2406.81812	219.18298	

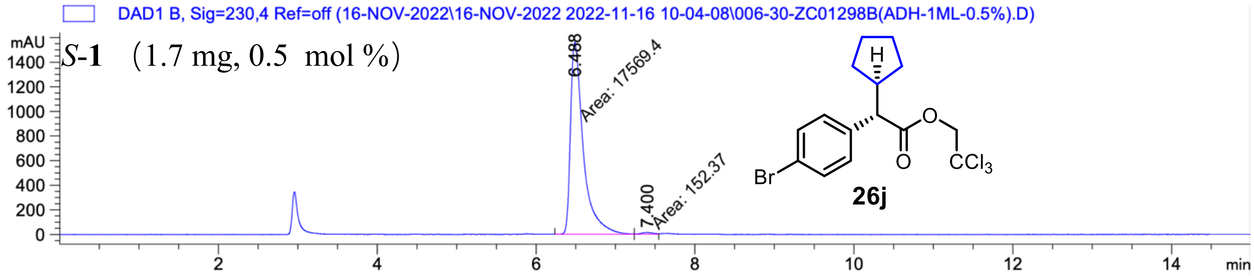

Signal 2: DAD1 B, Sig=230,4 Ref=off

Peak #	RetTime [min]	Type	Width [min]	Area [mAU*s]	Height [mAU]	Area %
1	6.488	MF	0.1876	1.75694e4	1560.88110	99.1402
2	7.400	FM	0.1760	152.37036	14.43132	0.8598
Totals :				1.77218e4	1575.31242	

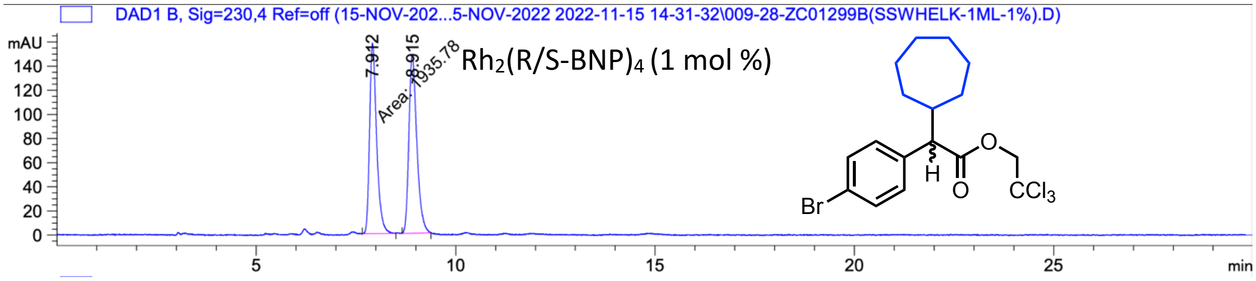

Signal 2: DAD1 B, Sig=230,4 Ref=off

Peak #	RetTime [min]	Type	Width [min]	Area [mAU*s]	Height [mAU]	Area %
1	7.912	MM	0.2042	1935.78381	158.01300	48.6465
2	8.915	BV R	0.1764	2043.49927	147.28694	51.3535
Totals :				3979.28308	305.29994	

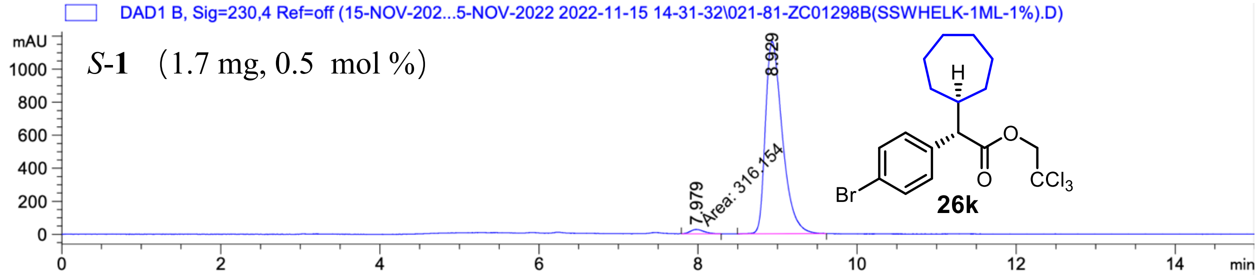

Signal 2: DAD1 B, Sig=230,4 Ref=off

Peak #	RetTime [min]	Type	Width [min]	Area [mAU*s]	Height [mAU]	Area %
1	7.979	MM	0.2016	316.15366	26.13406	1.7967
2	8.929	VV R	0.1791	1.72798e4	1164.75842	98.2033
Totals :				1.75960e4	1190.89248	

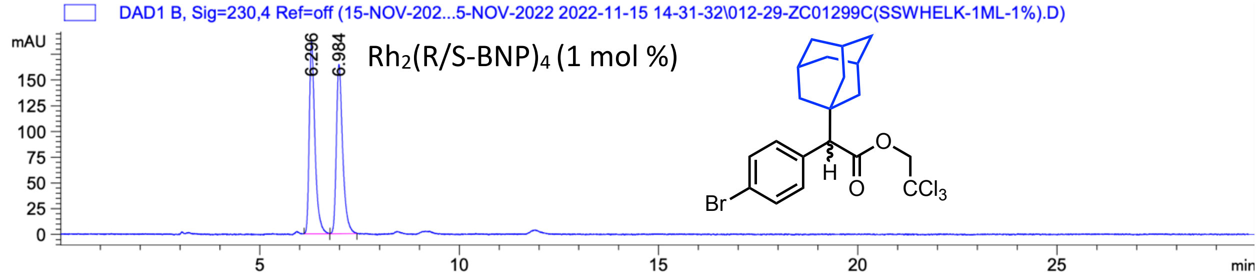

Signal 2: DAD1 B, Sig=230,4 Ref=off

Peak #	RetTime [min]	Type	Width [min]	Area [mAU*s]	Height [mAU]	Area %
1	6.296	BV R	0.1340	1842.88098	186.89391	50.3686
2	6.984	VV R	0.1352	1815.90515	164.64194	49.6314
Totals :				3658.78613	351.53584	

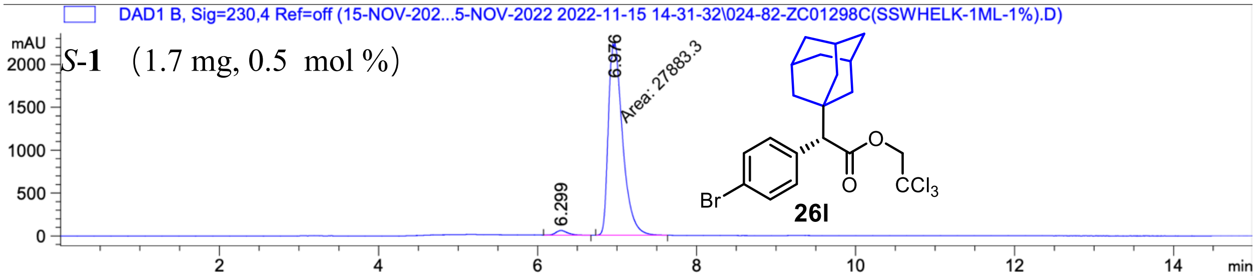

Signal 2: DAD1 B, Sig=230,4 Ref=off

Peak #	RetTime [min]	Type	Width [min]	Area [mAU*s]	Height [mAU]	Area %
1	6.299	VV R	0.1249	578.25439	54.87544	2.0317
2	6.976	MM	0.2072	2.78833e4	2242.32983	97.9683

Totals : 2.84615e4 2297.20528

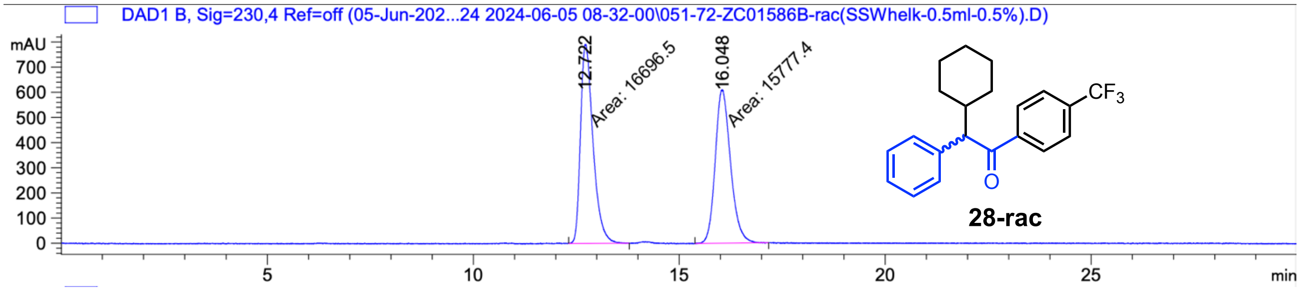

Signal 2: DAD1 B, Sig=230,4 Ref=off

Peak #	RetTime [min]	Type	Width [min]	Area [mAU*s]	Height [mAU]	Area %
1	12.722	MM	0.3515	1.66965e4	791.61670	51.4150
2	16.048	MM	0.4301	1.57774e4	611.34009	48.5850

Totals : 3.24739e4 1402.95679

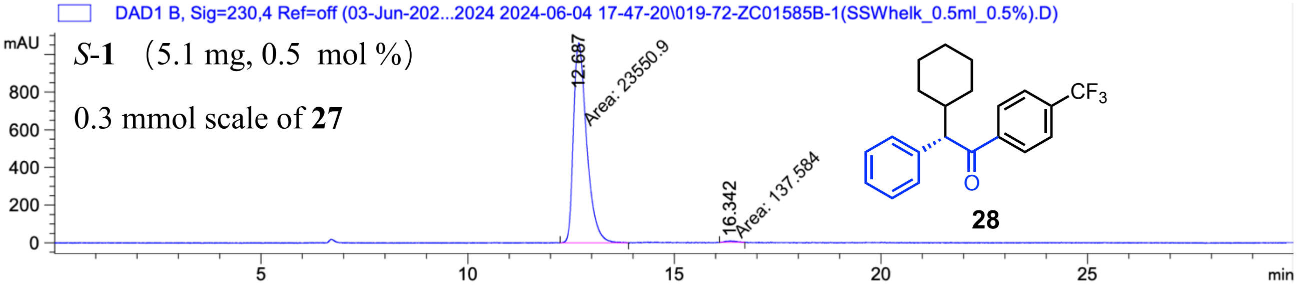

Signal 2: DAD1 B, Sig=230,4 Ref=off

Peak #	RetTime [min]	Type	Width [min]	Area [mAU*s]	Height [mAU]	Area %
1	12.687	MM	0.3702	2.35509e4	1060.38660	99.4192
2	16.342	MM	0.3073	137.58365	7.46306	0.5808

Totals : 2.36885e4 1067.84965

10. X-Ray Crystallographic Data

10.1 X-Ray Crystallographic Data for *S*-**6a**

Crystal Data and Experimental

R_1=6.49%

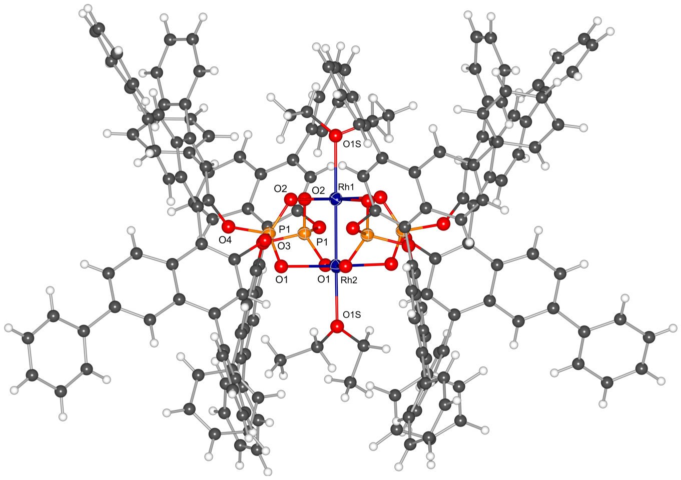

Experimental. Single colorless plate-shaped crystals of *S*-**6a** (ZC-01-51) were crystallized from hexane and DCM. A suitable crystal green, plate-shaped crystal of *S*-**6a** (ZC-01-51) was mounted on the goniometer. The crystal was kept at a steady T = 100 K during data collection. The structure was solved with the ShelXT (Sheldrick, 2015) solution program using dual methods and by using Olex2 1.5-alpha (Dolomanov et al., 2009) as the graphical interface. on a XtaLAB Synergy, Dualflex, HyPix four-circle diffractometer with a micro-focus sealed X-ray tube using a mirror as monochromator and a HyPix detector. The diffractometer was equipped with a low temperature device and used Cu Kα radiation (λ = 1.54184 Å).

Crystal Data. $C_{972}H_{764}Cl_{120}O_{90}P_{20}Rh_{10}$, M_r = 19786.29, orthorhombic, $C222_1$ (No. 20), a = 24.5303(2) Å, b = 65.2899(7) Å, c = 65.3438(7) Å, $\alpha = \beta = \gamma =$ 90°, V = 104653.6(17) Å^3, T = 100(2) K, Z = 4, Z' = 0.5, μ(Cu K_α) = 4.834, 163678 reflections measured, 53799 unique (R_{int} = 0.0639) which were used in all calculations. The final wR_2 was 0.1937 (all data) and R_1 was 0.0649 (I≥2 σ(I)).

Table 10.1.1 Crystal data and structure refinement for *S*-**6a** (ZC-01-51)

CCDC number	**2349408**
Empirical formula	$C_{972}H_{764}Cl_{120}O_{90}P_{20}Rh_{10}$
Formula weight	19786.29
Temperature [K]	100.15
Crystal system	orthorhombic
Space group (number)	$C222_1$ (20)
a/Å	24.5303(2)
b/Å	65.2899(7)
c/Å	65.3438(7)
α/°	90
β/°	90
γ/°	90
Volume [Å^3]	104653.6(17)
Z	4
ρ_{calc} [gcm^{-3}]	1.256
μ [mm^{-1}]	4.834
F(000)	40424
Crystal size [mm^3]	0.700×0.370×0.230
Crystal colour	green
Crystal shape	plate
Radiation	Cu K_α (λ=1.54184 Å)
2θ range [°]	5.41 to 139.49 (0.82 Å)
Index ranges	−20 ≤ h ≤ 26<br>−54 ≤ k ≤ 67<br>−78 ≤ l ≤ 63
Reflections collected	163678
Independent reflections	53799<br>R_{int} = 0.0639<br>R_{sigma} = 0.0618
Completeness to θ = 67.684°	59.2 %
Data / Restraints / Parameters	53799/15495/4520
Absorption correction T_{min}/T_{max} (method)	0.154/1.000 (gaussian)
Goodness-of-fit on F^2	1.006
Final R indexes [I≥2σ(I)]	R_1 = 0.0623<br>wR_2 = 0.1753
Final R indexes [all data]	R_1 = 0.0737<br>wR_2 = 0.1850

Largest peak/hole [eÅ^{-3}]	0.77/–0.36	Flack X parameter	0.097(7)

A green plate-shaped-shaped crystal with dimensions 0.70 × 0.38 × 0.23 mm^3 was mounted on a loop with paratone. Data were collected using a XtaLAB Synergy, Dualflex, HyPix diffractometer equipped with an Oxford Cryosystems low-temperature device operating at T = 100(2) K. Data were measured using ω scans with Cu K_α radiation. The diffraction pattern was indexed and the total number of runs and images was based on the strategy calculation from the program CrysAlisPro 1.171.41.122a (Rigaku OD, 2021). The maximum resolution that was achieved was Θ = 69.745° (0.82 Å). The unit cell was refined using CrysAlisPro 1.171.41.122a (Rigaku OD, 2021) on 65230 reflections, 40% of the observed reflections. Data reduction, scaling and absorption corrections were performed using CrysAlisPro 1.171.41.122a (Rigaku OD, 2021). The final completeness is 59.20 % out to 69.745° in Θ. A numerical absorption correction based on gaussian integration over a multifaceted crystal model was performed using CrysAlisPro 1.171.41.122a (Rigaku Oxford Diffraction, 2021). An empirical absorption correction using spherical harmonics, implemented in SCALE3 ABSPACK scaling algorithm was also applied.The structure was solved and the space group $C222_1$ (# 20) was solved by dual methods using SHELXT and refined by full-matrix least-squares and refined by full matrix least squares minimisation on $\boldsymbol{F^2}$ using version 2018/3 of ShelXL 2018/3 (Sheldrick, 2015). All non-hydrogen atoms were refined with anisotropic displacement parameters. All hydrogen atoms were refined on calculated positions using a riding model with their Uiso values constrained to 1.5 times the Ueq of their pivot atoms for terminal sp^3 carbon atoms and 1.2 times for all other carbon atoms. The formula unit in the crystal structure is very large and consists of 4 dirhodium complexes with diethyl ether at the axial positions (*i.e.* $4[Rh_2(C_{44}H_{28}O_4P)_4\ (C_4H_{10}O)_2]$, and 1 Rh_2 dinuclear complex with water at the axial positions $[Rh_2(C_{44}H_{28}O_4P)_4\ (H_2O)_2]$. The structure is also extremely porous with a very large number of solvent molecules that had to treated using solvent masking. The electron counts in these regions correlate well with 60 dichloromethane molecules (*i.e.* 60 $[CH_2Cl_2]$ in the formula unit). The value of Z' is 0.5. This means that only half of the formula unit is present in the asymmetric unit, with the other half consisting of symmetry equivalent atoms. In particular, the aqua complex resides on a special position.

***Special Details of the Refinement*:** The crystal structure has a very large unit cell of 104,654 Å and a total of 1,976 atoms in the formula unit. The crystals diffracted well but weakly beyond 80° in 2Θ (or a d-spacing of 1.2 Å). The structure was refined as a 2-component inversion twin. Subsequently, a second data set was collected where some higher angle data could be observed. These crystals are very air sensitive and unstable, and unfortunately, we could not collect more data. Also, the exposure times had to be extended to capture the weaker data. Although the data could have been truncated at 80° in 2Θ (as is normal for the other huge catalysts), the extra higher angle data significantly improved the data quality and data to parameter ratio. The other complexes with this ligand (and with Ru as the metal) behave differently: they do not have data present at resolutions above 1 Å. The structure quality is remarkable for one of this size with an R1 of 6% and low residuals. However, some of the peripheral phenyl ring carbons have extreme disorder with very large and elongated ellipsoids for these weakly held, interpenetrating phenyl rings. It is positive however to see the preferred phenyl-phenyl interactions in the structure – π-π stacking and H-π interactions. This is an effect of the size of the large, and bulky molecular structure. The very long and large ligands have rotating groups and are being held by a rigid Rh_2 core. The larger displacement parameters reflect an increase in the uncertainty of the atomic positions, especially for the atoms further away from the Rh-Rh bonds and on the termini of the ligands. The ligands have rotating groups and mean that the phenyl rings can adopt different conformations in the structure. The larger

displacement parameters reflect an increase in the uncertainty of the atomic positions, especially for the atoms further away from the Rh-Rh bonds and on the termini of the ligands.

The Flack parameter was refined to 0.102(7). Determination of absolute structure using Bayesian statistics on Bijvoet differences using the Olex2 results in -0.026(2). The chiral atoms in this structure are: P1(R), P1(R), P1(R), P1(R), P1(R), P1(R).

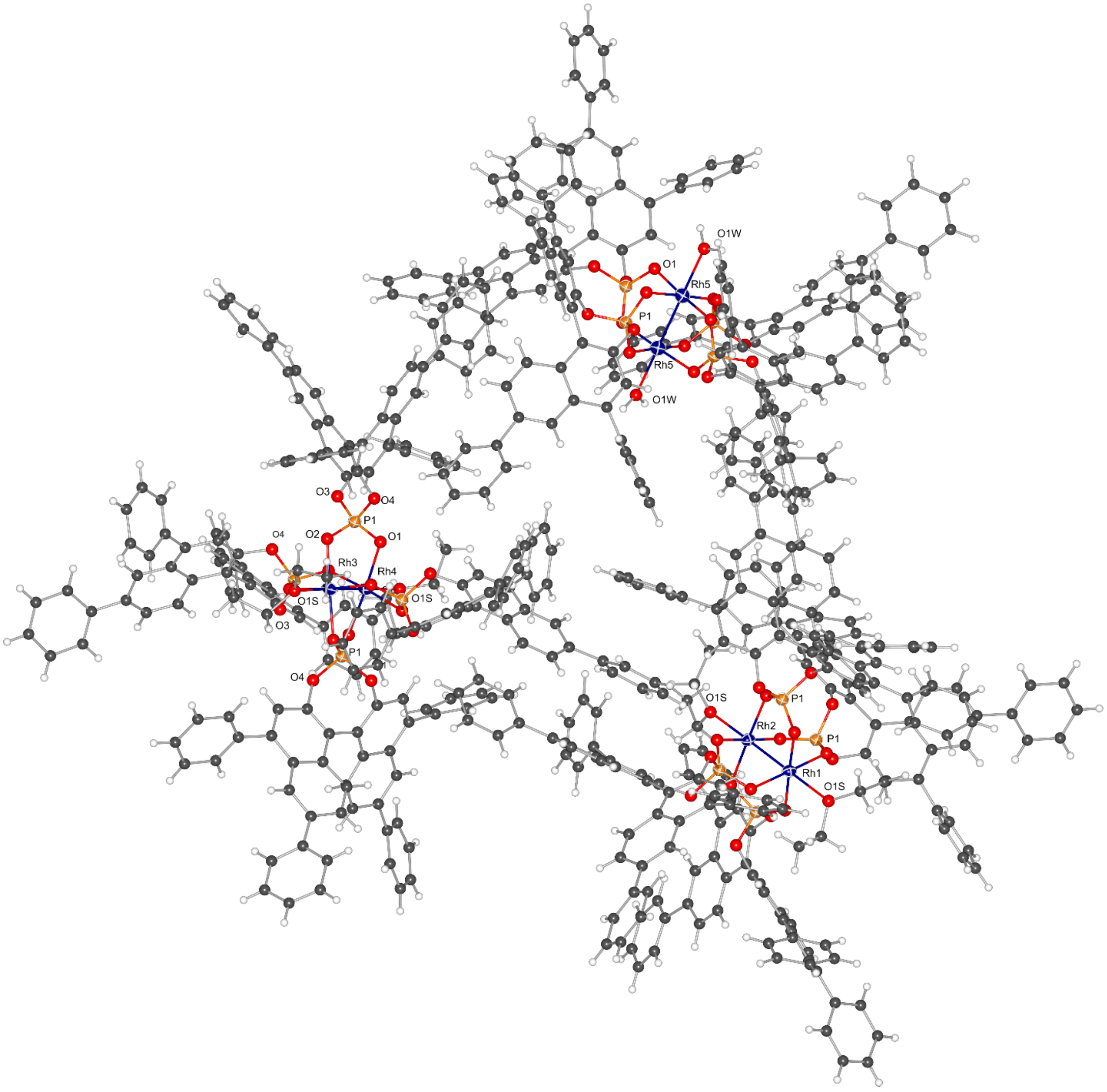

Figure 10.1.1 A representation of the asymmetric showing the chemically distinct dirhodium complexes and the interpenetrating phenyl rings.

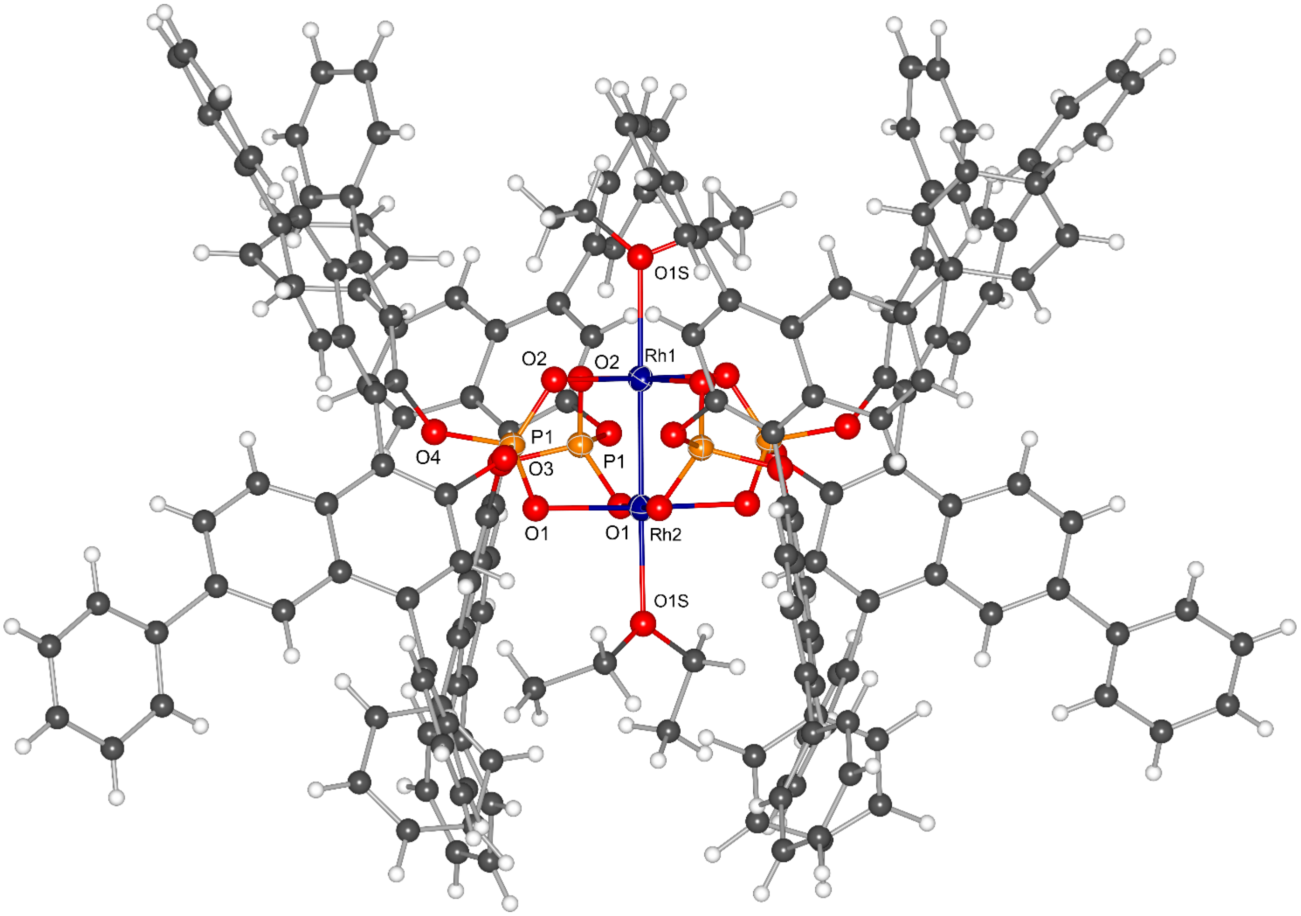

Figure 10.1.2 The molecular structure of one of the three dirhodium complexes in the crystal structure showing the coordination around the rhodium atoms with the low symmetry arrangement of the ligands about the dirhodium center. In this orientation, the bulky phenyl groups appear to be blocking access to the rhodium atoms.

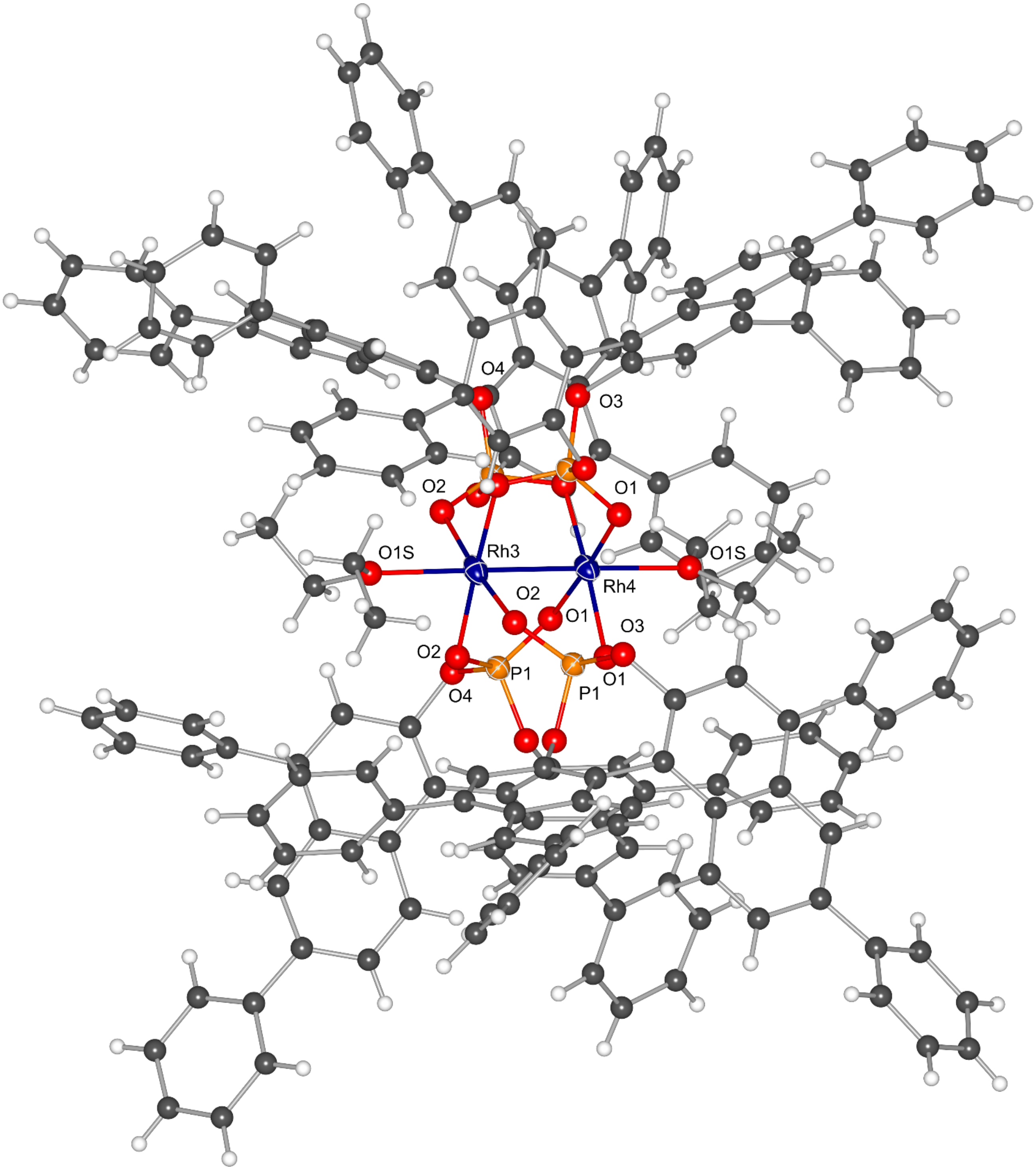

Figure 10.1.3 The molecular structure of the second distinct dirhodium complexes in the crystal structure. The conformational flexibility and the orientation the bulky phenyl groups hinders access to the rhodium atoms.

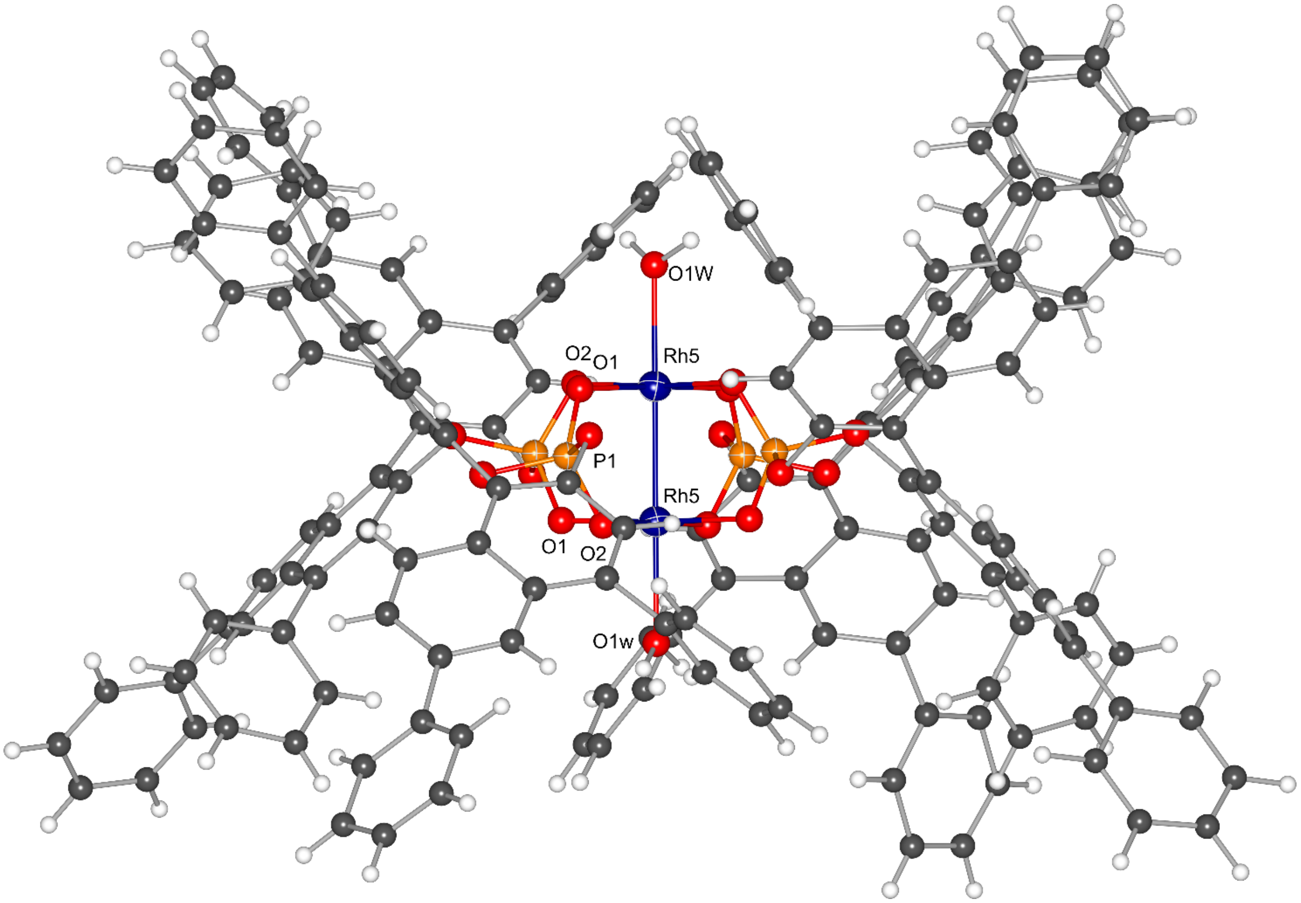

Figure 10.1.4 The molecular structure of the third distinct dirhodium complex with water at the axial positions [Rh2($C_{44}H_{28}O_4P$)$_4$ (H_2O)$_2$]. The conformational flexibility and the orientation the bulky phenyl groups hinders access to the rhodium atoms.

Data Plots: Diffraction Data

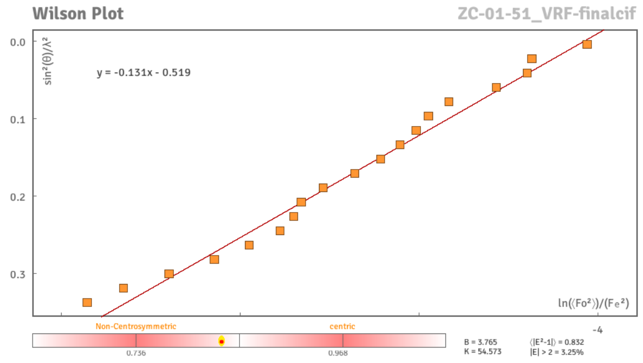

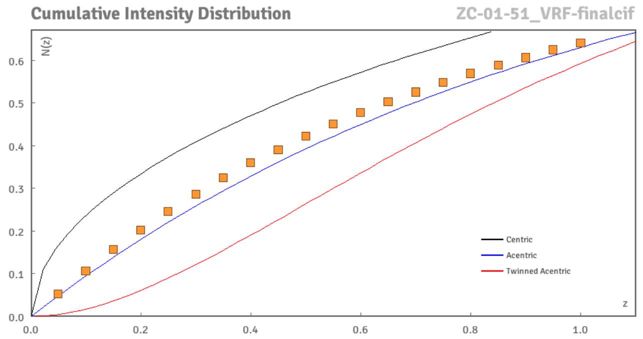

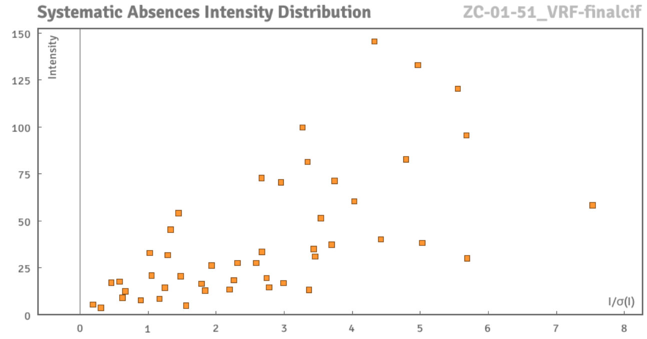

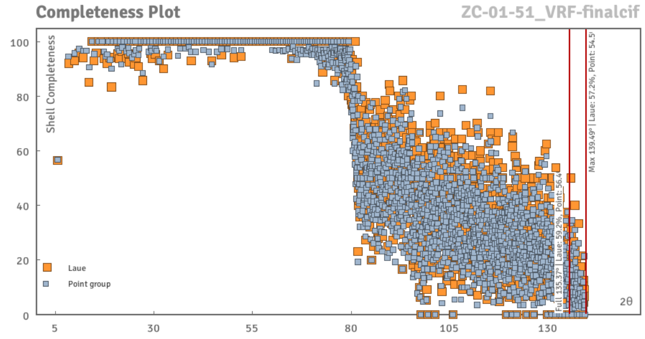

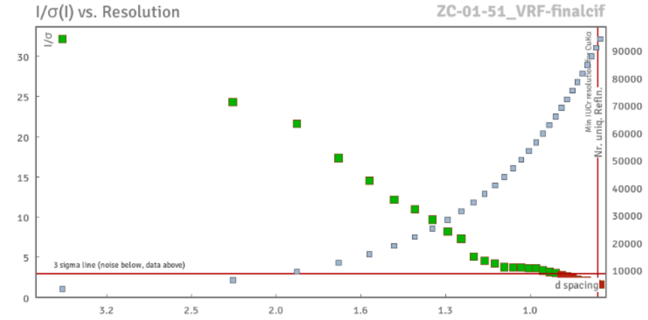

Data Plots: Refinement and Data

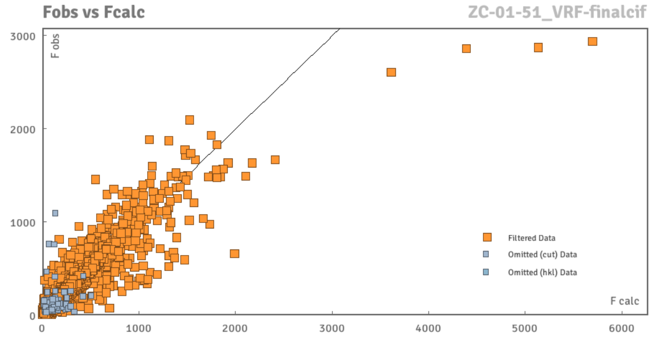

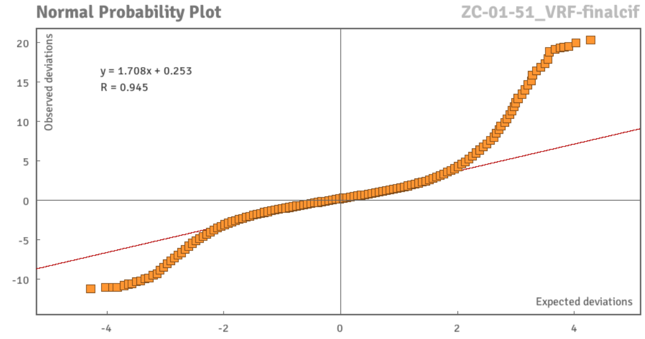

Reflection Statistics

Total reflections (after filtering)	163726	Unique reflections	53799
Completeness	0.545	Mean I/σ	10.91
hkl_{max} collected	(26, 67, 63)	hkl_{min} collected	(-20, -54, -78)
hkl_{max} used	(26, 67, 78)	hkl_{min} used	(-26, 0, 0)
Lim d_{max} collected	100.0	Lim d_{min} collected	0.77
d_{max} used	16.34	d_{min} used	0.82
Friedel pairs	30412	Friedel pairs merged	0
Inconsistent equivalents	1	R_{int}	0.0639
R_{sigma}	0.0618	Intensity transformed	0
Omitted reflections	346	Omitted by user (OMIT hkl)	0
Multiplicity	(77921, 23742, 9385, 2298, 195, 54, 3)	Maximum multiplicity	20
Removed systematic absences	48	Filtered off (Shel/OMIT)	0

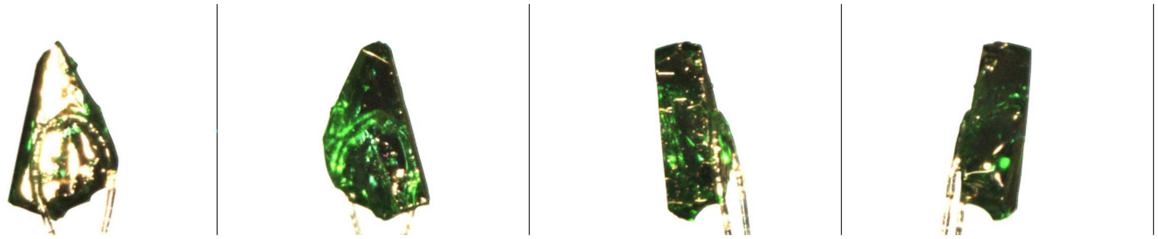

Table 10.1.2: Fractional Atomic Coordinates (×10⁴) and Equivalent Isotropic Displacement Parameters (Å²×10³) for ZC-01-51. U_{eq} is defined as 1/3 of the trace of the orthogonalised U_{ij}.

Atom	x	y	z	U_{eq}
Rh1	57.0(3)	6157.1(2)	5930.5(2)	44.7(2)
Rh2	62.7(3)	6434.1(2)	6192.0(2)	48.2(2)
Rh3	41.3(3)	9274.5(2)	5889.6(2)	53.4(2)
Rh4	77.1(3)	9021.7(2)	6172.0(2)	50.1(2)
Rh5	-4490.8(3)	7774.9(2)	7501.2(2)	57.0(2)
O	-3581(4)	7773.6(17)	7506.6(15)	153(5)
P1_1	-962.8(8)	9266.0(4)	6158.1(4)	51.8(7)
O1_1	-750.6(19)	9056.7(6)	6208.4(8)	57.5(17)
O2_1	-634.1(18)	9400.0(7)	6019.1(7)	51.0(16)
O3_1	-1075.0(16)	9399.3(7)	6356.5(6)	60.8(16)
O4_1	-1556.7(13)	9230.4(6)	6067.1(7)	55.1(16)
C1_1	-2109(2)	9489.3(8)	6215.3(8)	68(3)
C2_1	-2032.2(18)	9385.7(9)	6418.2(8)	58(2)
C3_1	-1521.1(18)	9341.6(9)	6478.4(7)	51(2)
C4_1	-1386(2)	9234.5(12)	6659.3(8)	70(3)
C5_1	-1813(2)	9171.2(13)	6785.1(10)	89.4(19)
C6_1	-2359(2)	9210.8(12)	6731.1(9)	88.7(19)
C7_1	-2473.9(19)	9317.2(11)	6541.8(8)	66(3)
C8_1	-1670(3)	9030.9(11)	6962.7(9)	91(2)
C9_1	-1726(5)	8815.3(12)	6939.1(13)	149(7)
C10_1	-1623(6)	8686.9(12)	7112.2(17)	145(4)
C11_1	-1471(6)	8785.5(18)	7289.8(15)	146(4)
C12_1	-1421(6)	8983.5(18)	7323.0(10)	146(4)
C13_1	-1518(4)	9113.0(13)	7147.1(11)	107(4)
C14_1	-2800(2)	9144.5(13)	6851.1(10)	89(2)
C15_1	-3330(2)	9183.1(14)	6795.2(11)	91(3)
C16_1	-3436(2)	9278.2(13)	6611.7(11)	80(2)
C17_1	-3018(2)	9344.8(11)	6495.1(10)	80(2)
C18_1	-3795(2)	9105.0(14)	6924.9(13)	119(4)
C19_1	-3729(3)	8939.5(16)	7061.2(16)	169(4)
C20_1	-4160(4)	8873.7(18)	7186.6(18)	170(4)
C21_1	-4651(4)	8977(2)	7175(2)	170(4)
C22_1	-4743(3)	9135(2)	7038(2)	172(4)
C23_1	-4305(3)	9206.0(18)	6920(2)	173(4)
C24_1	-1903(2)	9401.4(9)	6048.2(7)	60(3)
C25_1	-1948(3)	9483.8(10)	5849.2(7)	58(2)
C26_1	-2232(4)	9660.9(11)	5821.6(9)	81(3)
C27_1	-2464(3)	9765.2(10)	5988.4(8)	77(3)
C28_1	-2405(3)	9678.7(8)	6191.1(8)	67(2)
C29_1	-2361(4)	9721.4(12)	5608.3(8)	154(5)
C30_1	-2147(6)	9899.0(16)	5519.0(14)	226(7)
C31_1	-2285(7)	9953.9(19)	5318.4(15)	226(7)
C32_1	-2639(7)	9829.3(19)	5205.5(12)	213(8)
C33_1	-2858(7)	9652(2)	5293.3(14)	238(7)
C34_1	-2719(6)	9599.7(17)	5494.3(14)	237(7)
C35_1	-2611(3)	9798.1(9)	6358.8(9)	72(3)
C36_1	-2875(4)	9975.3(11)	6332.7(10)	91(3)
C37_1	-2989(4)	10047.2(10)	6139.6(10)	86(3)
C38_1	-2789(4)	9944.4(11)	5972.0(10)	85(3)
C39_1	-3312(3)	10236.5(8)	6107.0(12)	121(4)
C40_1	-3288(3)	10397.3(11)	6249.0(13)	136(4)
C41_1	-3594(3)	10580.3(9)	6215.3(17)	136(4)

Atom	x	y	z	U_{eq}
C42_1	-3907(4)	10590.7(13)	6040(2)	177(6)
C43_1	-3944(4)	10438.8(15)	5897.9(16)	164(4)
C44_1	-3646(4)	10260.5(13)	5930.7(13)	163(4)
P1_2	-5012.9(13)	7479.8(6)	7203.3(6)	144.4(18)
O1_2	-5535(2)	7588.4(11)	7253.2(12)	188(6)
O2_2	-4511(2)	7534.5(11)	7321.1(10)	130(4)
O3_2	-4851(2)	7499.9(8)	6969.2(7)	137(3)
O4_2	-5149(2)	7242.7(7)	7228.0(9)	136(3)
C1_2	-4775(2)	7081.1(9)	6934.6(8)	98(3)
C2_2	-5182(2)	7196.9(8)	6808.1(10)	101(3)
C3_2	-5197(3)	7402.0(8)	6828.3(9)	116(4)
C4_2	-5564(3)	7532.1(9)	6724.2(14)	138(5)
C5_2	-5941(4)	7441.1(10)	6593.2(17)	151(3)
C6_2	-5941(3)	7227.6(9)	6560.2(15)	150(3)
C7_2	-5567(3)	7101.2(9)	6675.9(14)	123(4)
C8_2	-6364(3)	7579.5(11)	6494.6(14)	153(3)
C9_2	-6765(4)	7675.0(17)	6619.7(16)	182(6)
C10_2	-7189(4)	7789.5(18)	6522(2)	190(4)
C11_2	-7177(5)	7794.0(19)	6312(2)	190(4)
C12_2	-6812(5)	7714(2)	6187.2(16)	190(4)
C13_2	-6380(4)	7599.9(17)	6286.1(14)	163(5)
C14_2	-6361(4)	7130.4(11)	6451.0(17)	149(3)
C15_2	-6428(4)	6921.2(11)	6454(2)	208(9)
C16_2	-6065(4)	6802.2(10)	6562(2)	177(5)
C17_2	-5661(4)	6892.7(10)	6668.4(18)	177(5)
C18_2	-6967(3)	6830.3(14)	6388(2)	296(10)
C19_2	-6994(4)	6662.5(18)	6253(3)	318(7)
C20_2	-7493(5)	6597(2)	6171(3)	319(7)
C21_2	-7961(4)	6702(3)	6224(3)	319(7)
C22_2	-7959(3)	6866(2)	6359(3)	319(7)
C23_2	-7459(4)	6931(2)	6440(3)	319(7)
C24_2	-4792(3)	7100.3(9)	7137.6(8)	109(4)
C25_2	-4460(3)	6988.3(14)	7273.9(9)	117(4)
C26_2	-4080(3)	6857.2(15)	7197.3(9)	117(4)
C27_2	-4042(3)	6821.2(15)	6985.3(9)	118(4)
C28_2	-4388(3)	6938.3(13)	6849.4(8)	124(4)
C29_2	-3709(3)	6754.7(14)	7344.1(11)	162(5)
C30_2	-3216(5)	6842.9(19)	7406(2)	278(8)
C31_2	-2852(5)	6736(3)	7532(3)	278(8)
C32_2	-2987(6)	6538(3)	7599(3)	292(12)
C33_2	-3464(7)	6444(2)	7529(3)	319(9)
C34_2	-3841(5)	6558.8(18)	7416(3)	319(9)
C35_2	-4296(4)	6916(2)	6633.5(9)	130(5)
C36_2	-3879(5)	6808(2)	6557.2(10)	144(6)
C37_2	-3533(4)	6702(2)	6684.6(10)	152(6)
C38_2	-3631(4)	6700.0(19)	6891.6(10)	132(5)
C39_2	-3071(3)	6580.3(16)	6603.0(13)	186(6)
C40_2	-2700(4)	6668.9(19)	6466.2(17)	300(9)
C41_2	-2241(4)	6554(2)	6396(2)	300(9)
C42_2	-2183(5)	6356(2)	6467(2)	243(9)
C43_2	-2533(7)	6263.6(19)	6600(3)	359(11)
C44_2	-2980(6)	6375.5(17)	6669(2)	360(11)
P1_3	1085.0(8)	9022.6(4)	5913.5(3)	57.4(7)
O1_3	901.4(18)	9004.0(8)	6131.9(5)	49.4(17)
O2_3	733.2(17)	9137.6(8)	5766.1(7)	58.6(19)
O3_3	1192.2(15)	8806.5(6)	5810.1(6)	59.4(14)

Atom	x	y	z	U_{eq}
O4_3	1677.7(13)	9120.9(7)	5924.6(6)	51.2(15)
C1_3	2221(2)	8924.6(8)	5696.1(8)	61(2)
C2_3	2147.0(18)	8744.5(8)	5836.1(8)	54(2)
C3_3	1633.9(18)	8694.4(7)	5889.7(8)	53(2)
C4_3	1494(2)	8538.7(10)	6030.3(11)	73(3)
C5_3	1919(2)	8430.2(12)	6121.1(11)	76.8(17)
C6_3	2466(2)	8470.4(10)	6068.2(10)	77.2(17)
C7_3	2586.0(19)	8633.7(10)	5925.6(10)	74(3)
C8_3	1774(3)	8274.0(10)	6284.3(9)	77.5(19)
C9_3	1957(4)	8304.6(13)	6488.7(10)	102(4)
C10_3	1774(5)	8166.6(16)	6644.1(9)	127(3)
C11_3	1423(5)	8014.2(17)	6581.3(14)	129(3)
C12_3	1246(5)	7975.0(15)	6395.2(14)	129(3)
C13_3	1429(5)	8115.5(15)	6238.5(12)	128(5)
C14_3	2899(2)	8354.7(11)	6147.6(11)	78.3(19)
C15_3	3430(2)	8392.4(11)	6091.5(11)	80(3)
C16_3	3543(2)	8550.2(13)	5959.1(13)	86(2)
C17_3	3131(2)	8661.6(11)	5879.8(11)	85(2)
C18_3	3882(3)	8257.9(14)	6171.7(11)	130(5)
C19_3	3888(3)	8188.3(15)	6376.0(10)	118(3)
C20_3	4276(4)	8045.2(16)	6443.4(12)	119(3)
C21_3	4654(4)	7973.2(17)	6304.6(15)	121(2)
C22_3	4674(4)	8039.5(18)	6103.3(14)	121(3)
C23_3	4271(4)	8175.5(18)	6034.6(11)	121(3)
C24_3	2019(2)	9105.7(7)	5752.0(8)	54(2)
C25_3	2110(4)	9289.6(8)	5643.6(10)	80(3)
C26_3	2420(4)	9286.3(9)	5470.9(12)	121(4)
C27_3	2646(4)	9102.0(8)	5397.9(11)	89(3)
C28_3	2549(3)	8916.0(8)	5512.8(8)	77(3)
C29_3	2579(4)	9486.3(9)	5381.3(14)	168(5)
C30_3	2206(4)	9611.1(15)	5278(2)	221(7)
C31_3	2368(6)	9797.3(17)	5191(3)	220(7)
C32_3	2911(7)	9861.2(14)	5210.3(16)	236(9)
C33_3	3293(5)	9735(2)	5308(3)	220(6)
C34_3	3121(4)	9551.1(17)	5397(2)	222(6)
C35_3	2713(4)	8727.1(9)	5417.8(11)	77(3)
C36_3	3007(4)	8720.5(10)	5245.8(11)	78(3)
C37_3	3170(3)	8898.7(9)	5151.5(10)	70(3)
C38_3	2943(4)	9081.4(10)	5212.6(11)	86(3)
C39_3	3530(3)	8895.0(11)	4968.9(9)	94(3)
C40_3	3469(3)	8740.7(11)	4821.3(10)	98(3)
C41_3	3835(3)	8729.3(14)	4652.1(10)	98(3)
C42_3	4244(3)	8874.3(17)	4641.2(12)	124(5)
C43_3	4320(3)	9026.7(15)	4780.4(13)	131(4)
C44_3	3962(3)	9037.9(12)	4945.5(12)	130(4)
P1_5	-218.5(9)	6016.7(4)	6367.0(3)	56.1(7)
O1_5	55(2)	6216.7(6)	6415.8(7)	54.9(15)
O2_5	-273(2)	5955.9(8)	6147.4(5)	59.3(18)
O3_5	-812.6(13)	6002.2(8)	6461.3(5)	58.8(17)
O4_5	114.2(15)	5851.1(6)	6494.3(7)	62.5(16)
C1_5	-541(2)	5663.7(7)	6689.3(8)	69(2)
C2_5	-699(3)	5859.4(8)	6794.2(7)	78(3)
C3_5	-848(2)	6018.4(7)	6675.4(7)	62(2)
C4_5	-1028(3)	6210.3(8)	6748.8(8)	73(3)
C5_5	-1054(5)	6237.2(10)	6960.0(8)	112(2)
C6_5	-902(4)	6078.1(9)	7094.1(8)	113(2)

Atom	x	y	z	U_{eq}
C7_5	-732(4)	5883.1(9)	7010.6(7)	82(3)
C8_5	-1237(4)	6444.9(9)	7039.0(11)	114(3)
C9_5	-845(4)	6580.3(16)	7125(2)	219(10)
C10_5	-1022(6)	6777.8(16)	7193(3)	229(6)
C11_5	-1568(6)	6819.9(16)	7168(3)	228(6)
C12_5	-1951(5)	6703.3(19)	7088(3)	228(6)
C13_5	-1773(4)	6502.2(18)	7023(3)	261(14)
C14_5	-887(5)	6105.5(12)	7307.1(8)	114(3)
C15_5	-716(6)	5951.9(14)	7436.1(9)	295(16)
C16_5	-557(5)	5766.7(13)	7356.3(9)	116(3)
C17_5	-565(4)	5738.3(11)	7151.7(9)	116(3)
C18_5	-688(5)	5990.1(15)	7663.6(7)	258(3)
C19_5	-483(6)	6174.8(17)	7743.5(14)	280.4(11)
C20_5	-431(7)	6205(2)	7955.2(16)	280.4(11)
C21_5	-591(7)	6048(3)	8085.1(10)	280.2(14)
C22_5	-796(7)	5863(3)	8013.7(11)	280.4(11)
C23_5	-842(7)	5835.1(19)	7802.1(12)	280.4(11)
C24_5	-136(2)	5666.8(7)	6553.0(9)	71(3)
C25_5	91(3)	5489.5(7)	6463.1(13)	82(3)
C26_5	-89(3)	5301.5(8)	6522.4(14)	93(3)
C27_5	-527(3)	5282.5(7)	6659.9(15)	101(4)
C28_5	-755(3)	5466.4(7)	6749.0(11)	85(3)
C29_5	235(3)	5121.1(8)	6457.1(12)	122(4)
C30_5	290(4)	5066.6(12)	6251.1(11)	126(3)
C31_5	582(4)	4891.6(14)	6194.2(14)	127(3)
C32_5	819(4)	4768.3(12)	6345.2(19)	128(4)
C33_5	770(5)	4821.4(15)	6551.7(17)	156(4)
C34_5	484(5)	4998.8(15)	6605.9(12)	157(4)
C35_5	-1246(3)	5441.1(9)	6866.3(16)	90(4)
C36_5	-1443(3)	5257.8(9)	6920.2(15)	78(3)
C37_5	-1194(3)	5081.0(8)	6854.6(15)	91(3)
C38_5	-761(4)	5093.7(8)	6721.8(18)	107(4)
C39_5	-1394(3)	4875.3(8)	6915.5(12)	115(5)
C40_5	-1953(3)	4840.9(11)	6944.6(12)	106.8(10)
C41_5	-2142(3)	4645.2(11)	7011.1(13)	106.8(10)
C42_5	-1757(4)	4494.7(11)	7044.7(13)	90(3)
C43_5	-1211(3)	4520.7(9)	7018.6(15)	106.8(10)
C44_5	-1026(3)	4711.6(11)	6953.7(15)	106.8(10)
P1_6	-4989.1(12)	8073.9(6)	7206.3(5)	117.5(14)
O1_6	-5494(2)	8015.8(11)	7321.7(9)	134(4)
O2_6	-4469.0(19)	7964.2(10)	7254.7(10)	131(4)
O3_6	-4852.1(19)	8310.3(7)	7225.1(7)	113(2)
O4_6	-5140.3(18)	8045.7(8)	6971.3(6)	109(2)
C1_6	-4750(2)	8332.5(8)	6803.7(8)	67(2)
C2_6	-5153(2)	8465.2(9)	6914.1(7)	66(2)
C3_6	-5194(2)	8446.0(8)	7118.6(7)	84(3)
C4_6	-5562(3)	8556.9(11)	7242.7(8)	88(3)
C5_6	-5911(3)	8695.1(11)	7146.0(8)	70.8(16)
C6_6	-5880(2)	8727.9(10)	6933.1(8)	71.7(16)
C7_6	-5513(2)	8602.9(10)	6812.6(7)	65(2)
C8_6	-6321(2)	8807.7(10)	7278.6(9)	71.0(18)
C9_6	-6780(3)	8699.7(12)	7356.9(15)	132(6)
C10_6	-7152(3)	8807.0(14)	7487.2(17)	107(3)
C11_6	-7033(4)	9009.7(15)	7525.1(17)	108(3)
C12_6	-6613(4)	9117.4(11)	7460.7(17)	108(3)
C13_6	-6239(4)	9009.0(11)	7328.4(16)	102(4)

Atom	x	y	z	U_{eq}
C14_6	-6255(3)	8853.4(11)	6829.9(8)	70.5(18)
C15_6	-6281(3)	8858.5(11)	6619.9(8)	68(2)
C16_6	-5930(3)	8740.1(12)	6506.6(9)	67(2)
C17_6	-5567(3)	8618.2(11)	6602.7(8)	68(2)
C18_6	-6681(2)	8998.3(10)	6514.0(9)	76(2)
C19_6	-6752(3)	9203.8(10)	6574.6(11)	93(2)
C20_6	-7152(3)	9327.5(10)	6483.0(14)	94(2)
C21_6	-7480(3)	9242.8(13)	6333.5(15)	93(2)
C22_6	-7420(3)	9043.0(14)	6265.5(14)	93(2)
C23_6	-7025(3)	8919.4(11)	6359.2(13)	93(2)
C24_6	-4787(2)	8129.7(8)	6823.7(8)	75(3)
C25_6	-4443(3)	7987.9(8)	6723.8(13)	89(3)
C26_6	-4070(3)	8058.2(9)	6586.6(13)	80(3)
C27_6	-3992(3)	8269.8(8)	6557.8(13)	73(3)
C28_6	-4340(2)	8411.7(8)	6666.7(10)	62(2)
C29_6	-3723(3)	7905.6(10)	6481.0(13)	140(4)
C30_6	-3350(6)	7782(2)	6586.5(18)	313(9)
C31_6	-3026(7)	7640(3)	6482(3)	313(9)
C32_6	-3080(6)	7618(2)	6270(3)	298(12)
C33_6	-3446(7)	7742(3)	6162.2(19)	240(7)
C34_6	-3763(6)	7886(2)	6268.2(13)	239(7)
C35_6	-4218(3)	8625.9(9)	6644.5(16)	76(3)
C36_6	-3809(3)	8695.5(9)	6528.8(13)	62(3)
C37_6	-3485(3)	8562.7(8)	6420.1(12)	57(2)
C38_6	-3595(3)	8356.3(9)	6425.9(13)	71(3)
C39_6	-2999(2)	8632.5(10)	6304.1(10)	76(3)
C40_6	-2667(3)	8789.6(10)	6380.3(11)	95(3)
C41_6	-2225(3)	8866.2(12)	6260.1(14)	96(3)
C42_6	-2144(3)	8779.9(14)	6069.7(14)	131(5)
C43_6	-2455(3)	8627.2(14)	5989.2(10)	94(3)
C44_6	-2885(3)	8552.4(11)	6106.3(10)	93(3)
O1S_7	56(3)	5893.2(13)	5689.5(11)	98(2)
C2S_7	-351(6)	5744(3)	5658(3)	193(6)
C3S_7	-447(12)	5596(4)	5834(4)	282(14)
C4S_7	598(5)	5850(2)	5605(2)	156(5)
C5S_7	743(5)	5989(3)	5426(2)	169(7)
O1S_8	54(3)	9512.5(12)	5627.7(11)	101(2)
C2S_8	-407(5)	9570.1(19)	5512(2)	131(5)
C3S_8	-654(11)	9403(3)	5379(5)	324(16)
C4S_8	570(4)	9615(2)	5579(2)	127(4)
C5S_8	542(8)	9846(2)	5612(4)	251(14)
P1_9	-363.8(9)	8851.1(4)	5778.7(4)	55.4(7)
O1_9	-12(2)	8791.8(8)	5957.4(6)	55.8(16)
O2_9	-445(2)	9073.6(5)	5734.3(9)	63.3(18)
O3_9	-957.0(14)	8755.4(6)	5793.3(7)	61.2(17)
O4_9	-102.4(15)	8736.5(7)	5587.3(5)	49.8(14)
C1_9	-830(2)	8562.0(8)	5422.8(7)	62(2)
C2_9	-897(3)	8438.4(7)	5614.8(8)	61(2)
C3_9	-986(2)	8540.3(7)	5790.8(7)	66(2)
C4_9	-1071(4)	8446.8(8)	5983.1(8)	88(3)
C5_9	-1073(5)	8233.3(9)	5990.1(9)	115(2)
C6_9	-972(4)	8116.1(8)	5812.8(9)	114(2)
C7_9	-873(3)	8220.8(7)	5621.2(8)	77(3)
C8_9	-1191(4)	8134.2(11)	6195.6(9)	117(3)
C9_9	-773(4)	8019.8(18)	6294.4(15)	169(7)
C10_9	-864(6)	7951(2)	6499.2(16)	211(6)

Atom	x	y	z	U_{eq}
C11_9	-1361(7)	8000(3)	6583.6(15)	211(6)
C12_9	-1761(6)	8107(3)	6502.5(16)	211(6)
C13_9	-1669(4)	8177(2)	6295.2(16)	171(7)
C14_9	-935(4)	7901.4(8)	5816.6(11)	115(3)
C15_9	-808(4)	7791.2(9)	5643.9(12)	118(4)
C16_9	-713(4)	7891.8(9)	5462.6(11)	100(3)
C17_9	-749(4)	8097.8(9)	5455.6(10)	100(3)
C18_9	-801(4)	7560.2(8)	5648.8(14)	159(5)
C19_9	-531(4)	7450.8(12)	5804.5(14)	153(3)
C20_9	-472(5)	7237.0(12)	5794(2)	154(3)
C21_9	-681(5)	7135.9(10)	5626(2)	155(3)
C22_9	-956(6)	7235.5(12)	5469.8(19)	156(3)
C23_9	-1013(5)	7448.8(12)	5480.8(16)	157(3)
C24_9	-435(2)	8702.0(8)	5415.1(6)	58(2)
C25_9	-352(3)	8836.5(11)	5248.9(8)	62(3)
C26_9	-671(3)	8818.0(14)	5079.4(10)	105(4)
C27_9	-1100(3)	8675.5(13)	5073.1(9)	82(3)
C28_9	-1187(3)	8543.9(11)	5248.3(8)	80(3)
C29_9	-568(3)	8961.8(13)	4908.2(10)	130(4)
C30_9	-62(3)	8973.3(17)	4809.8(16)	186(5)
C31_9	38(5)	9121(2)	4658.8(19)	186(5)
C32_9	-373(6)	9260.3(19)	4606.4(19)	179(7)
C33_9	-884(5)	9248.7(18)	4701(2)	169(5)
C34_9	-975(4)	9101.7(17)	4853.0(18)	169(5)
C35_9	-1660(3)	8414.0(16)	5243.5(11)	90(3)
C36_9	-2004(3)	8408.3(17)	5084.7(11)	95(4)
C37_9	-1886(3)	8511.9(16)	4906.8(11)	104(4)
C38_9	-1447(3)	8642.9(15)	4902.1(10)	82(3)
C39_9	-2260(3)	8505.8(11)	4728.4(9)	101(3)
C40_9	-2825(3)	8491.1(12)	4758.1(12)	131(4)
C41_9	-3183(3)	8482.7(13)	4586.3(15)	132(4)
C42_9	-2952(4)	8489.7(16)	4393.8(14)	156(6)
C43_9	-2407(4)	8503.9(17)	4357.2(9)	139(4)
C44_9	-2057(3)	8512.1(15)	4524.8(11)	139(4)
P1_10	507.5(9)	9434.2(4)	6289.3(4)	62.1(8)
O1_10	211(2)	9250.8(6)	6372.6(8)	59.9(17)
O2_10	478(2)	9475.3(9)	6064.7(5)	68(2)
O3_10	1136.9(13)	9429.7(8)	6344.0(6)	68.4(19)
O4_10	279.0(16)	9621.9(6)	6418.4(7)	72.9(19)
C1_10	1062(2)	9800.3(8)	6536.0(10)	86(3)
C2_10	1222(3)	9613.9(8)	6656.1(7)	72(3)
C3_10	1266(2)	9435.8(7)	6553.4(7)	62(2)
C4_10	1419(3)	9246.8(8)	6641.0(8)	68(3)
C5_10	1530(3)	9243.6(8)	6850.2(8)	68.2(15)
C6_10	1516(3)	9425.6(8)	6966.1(8)	67.8(16)
C7_10	1329(3)	9612.9(8)	6870.2(7)	66(2)
C8_10	1701(3)	9040.0(8)	6942.4(9)	70.1(17)
C9_10	1366(3)	8947.0(11)	7094.0(11)	72(3)
C10_10	1488(4)	8742.2(11)	7156.7(13)	98(3)
C11_10	1930(4)	8651.1(12)	7063.6(16)	98(3)
C12_10	2246(4)	8725.8(11)	6918.8(16)	98(3)
C13_10	2141(3)	8936.8(12)	6864.3(14)	81(3)
C14_10	1651(3)	9430.7(9)	7174.8(8)	67.0(18)
C15_10	1609(4)	9607.0(9)	7288.9(8)	81(3)
C16_10	1429(4)	9783.8(10)	7196.7(9)	96(3)
C17_10	1301(4)	9782.7(10)	6995.8(9)	97(3)

Atom	x	y	z	U_{eq}
C18_10	1823(3)	9612.9(12)	7505.2(7)	108(4)
C19_10	2313(3)	9514.9(12)	7560.9(11)	133(3)
C20_10	2525(4)	9531.9(15)	7759.9(12)	134(3)
C21_10	2241(4)	9647.6(17)	7901.3(10)	135(3)
C22_10	1758(4)	9747.0(16)	7854.7(9)	136(3)
C23_10	1548(4)	9729.5(14)	7655.9(10)	136(3)
C24_10	591(2)	9801.7(7)	6434.4(10)	74(3)
C25_10	394(3)	9970.2(10)	6321(2)	147(7)
C26_10	709(4)	10141.1(12)	6303(2)	204(9)
C27_10	1212(3)	10154.8(11)	6406(2)	145(7)
C28_10	1399(3)	9981.1(9)	6523.9(14)	97(4)
C29_10	493(4)	10313.0(12)	6179.1(17)	239(8)
C30_10	7(4)	10413.8(18)	6229(2)	218(6)
C31_10	-185(6)	10577.3(19)	6110(3)	218(6)
C32_10	115(7)	10641.9(19)	5940(2)	238(9)
C33_10	599(7)	10541(2)	5886(2)	247(7)
C34_10	786(5)	10379(2)	6007(2)	247(7)
C35_10	1953(3)	9987.3(12)	6595.7(17)	99(4)
C36_10	2275(3)	10150.7(14)	6572(2)	120(5)
C37_10	2099(3)	10317.2(15)	6461(2)	148(6)
C38_10	1564(3)	10327.2(12)	6400(2)	143(6)
C39_10	2465(3)	10491.7(11)	6413.5(14)	163(5)
C40_10	3024(3)	10458.9(15)	6384.1(13)	179(5)
C41_10	3366(3)	10622.6(19)	6316.6(15)	180(5)
C42_10	3123(5)	10810.3(18)	6282.4(19)	293(15)
C43_10	2583(5)	10849.6(12)	6309(2)	195(5)
C44_10	2250(4)	10690.2(12)	6374.5(18)	194(5)
O1S_11	77(3)	6686.7(12)	6423.3(11)	94(2)
C2S_11	-286(5)	6854(2)	6415(3)	171(6)
C3S_11	-804(5)	6831(3)	6542(3)	159(6)
C4S_11	524(6)	6700(4)	6572(3)	233(9)
C5S_11	345(12)	6782(9)	6779(3)	580(40)
O1S_12	99(3)	8785.9(12)	6426.1(10)	82(2)
C2S_12	-209(5)	8603.5(17)	6424.6(18)	116(3)
C3S_12	-724(7)	8606(3)	6554(4)	225(10)
C4S_12	497(4)	8810.1(17)	6592.0(16)	91(3)
C5S_12	979(4)	8665(2)	6570.2(19)	95(4)
P1_13	1143.9(8)	6204.8(4)	6147.2(3)	47.9(7)
O1_13	918.9(19)	6411.8(6)	6198.4(8)	53.9(17)
O2_13	807.1(18)	6063.6(7)	6018.3(7)	49.1(16)
O3_13	1303.9(15)	6075.6(6)	6343.7(6)	49.2(15)
O4_13	1719.7(13)	6246.1(6)	6042.4(6)	47.4(14)
C1_13	2312(2)	5986.7(8)	6168.2(7)	49(2)
C2_13	2275.5(18)	6086.2(10)	6374.8(8)	60(3)
C3_13	1779.3(17)	6136.1(9)	6447.5(7)	53(3)
C4_13	1683(2)	6233.3(13)	6637.3(8)	79(4)
C5_13	2133(2)	6277.9(19)	6759.6(11)	142(3)
C6_13	2666(2)	6233.3(16)	6692.2(10)	141(3)
C7_13	2741.3(18)	6140.9(12)	6493.0(8)	64(3)
C8_13	2018(3)	6357.8(15)	6973.7(9)	144(3)
C9_13	2053(4)	6220.5(17)	7142.5(14)	234(10)
C10_13	1856(5)	6287(2)	7337.4(11)	250(7)
C11_13	1645(6)	6483(3)	7344.7(15)	251(7)
C12_13	1601(6)	6617(2)	7195.5(18)	249(7)
C13_13	1800(5)	6549.4(16)	6998.5(15)	217(8)
C14_13	3131(2)	6287.8(18)	6805.1(12)	141(3)

Atom	x	y	z	U_{eq}
C15_13	3648(2)	6255.9(17)	6730.7(12)	114(4)
C16_13	3717(2)	6166.7(14)	6541.8(11)	80(2)
C17_13	3275(2)	6114.6(12)	6430.8(10)	79(2)
C18_13	4139(2)	6329(2)	6849.0(13)	200(7)
C19_13	4109(4)	6373(3)	7059.8(14)	239(5)
C20_13	4582(5)	6406(3)	7176.4(15)	238(5)
C21_13	5081(4)	6393(3)	7080(2)	237(4)
C22_13	5135(3)	6350(3)	6873(2)	235(4)
C23_13	4661(3)	6322(3)	6756.7(16)	234(5)
C24_13	2066(2)	6077.0(8)	6009.8(7)	44(2)
C25_13	2075(3)	6000.1(10)	5808.0(7)	52(2)
C26_13	2361(3)	5826.4(10)	5766.5(8)	63(3)
C27_13	2647(3)	5723.2(10)	5922.2(7)	53(2)
C28_13	2614(3)	5801.2(8)	6129.2(7)	51(2)
C29_13	2387(3)	5758.3(10)	5550.2(7)	83(3)
C30_13	2233(4)	5560.6(10)	5490.3(10)	111(3)
C31_13	2195(5)	5508.0(12)	5283.0(11)	112(3)
C32_13	2306(6)	5655.4(16)	5133.3(8)	140(6)
C33_13	2476(6)	5852.0(14)	5190.5(9)	116(3)
C34_13	2494(5)	5904.3(11)	5398.3(9)	115(3)
C35_13	2887(3)	5683.9(10)	6284.7(8)	52(2)
C36_13	3190(3)	5519.0(11)	6243.5(9)	54(2)
C37_13	3255(3)	5451.1(10)	6045.7(8)	58(2)
C38_13	2984(3)	5549.4(10)	5890.2(9)	53(2)
C39_13	3599(3)	5270.8(8)	5995.9(10)	67(3)
C40_13	3597(3)	5097.9(10)	6123.1(10)	85(3)
C41_13	3941(3)	4927.4(9)	6077.8(13)	85(3)
C42_13	4269(3)	4940.6(12)	5906.6(15)	102(4)
C43_13	4285(3)	5105.1(13)	5778.6(12)	110(3)
C44_13	3950(3)	5271.3(12)	5822.7(11)	110(3)
P1_14	-1026.0(8)	6380.9(4)	5978.6(3)	55.2(7)
O1_14	-788.0(17)	6447.6(8)	6179.1(6)	52.8(17)
O2_14	-693.0(19)	6244.5(8)	5842.9(7)	61.9(18)
O3_14	-1203.5(16)	6565.7(6)	5836.6(7)	68.4(17)
O4_14	-1591.0(14)	6275.3(7)	6034.6(6)	61.0(17)
C1_14	-2196(2)	6369.1(8)	5766.7(8)	65(2)
C2_14	-2178.6(19)	6584.5(8)	5846.7(10)	77(2)
C3_14	-1688.4(19)	6668.7(7)	5885.9(11)	87(3)
C4_14	-1608(2)	6870.4(8)	5958.6(15)	112(4)
C5_14	-2067(2)	6992.1(10)	5988.5(18)	126.6(14)
C6_14	-2594(2)	6913.3(9)	5954.3(15)	123.4(10)
C7_14	-2654(2)	6702.8(9)	5889.0(14)	102(3)
C8_14	-1976(3)	7211.2(8)	6058.3(14)	133.1(17)
C9_14	-1737(4)	7248.5(13)	6253.1(14)	144(2)
C10_14	-1630(5)	7455.3(15)	6312.8(16)	145(2)
C11_14	-1774(5)	7604.2(13)	6174(2)	145(2)
C12_14	-1987(6)	7580.8(10)	5989.9(18)	144(2)
C13_14	-2099(5)	7371.3(13)	5931.5(15)	144(2)
C14_14	-3064(3)	7025.9(11)	6001.6(16)	123.4(10)
C15_14	-3576(2)	6941.3(12)	5986.1(17)	123.4(10)
C16_14	-3631(2)	6740.7(12)	5926.0(18)	123.4(10)
C17_14	-3182(2)	6630.3(11)	5879.5(15)	123.4(10)
C18_14	-4065(3)	7059.3(16)	6061.8(19)	211(7)
C19_14	-4012(4)	7218(2)	6207(2)	259(5)
C20_14	-4442(6)	7354(2)	6247(3)	260(5)
C21_14	-4923(6)	7329(2)	6139(3)	260(5)

Atom	x	y	z	U_{eq}
C22_14	-4995(4)	7178(3)	5994(3)	260(5)
C23_14	-4571(3)	7039(2)	5959(3)	259(5)
C24_14	-1938(2)	6222.7(7)	5870.9(8)	66(2)
C25_14	-1931(3)	6013.6(7)	5816.1(10)	72(3)
C26_14	-2210(4)	5952.2(9)	5646.9(11)	97(3)
C27_14	-2506(3)	6094.1(8)	5529.0(10)	79(2)
C28_14	-2493(3)	6308.3(8)	5586.1(9)	78(2)
C29_14	-2224(4)	5729.9(8)	5602.0(11)	149(2)
C30_14	-2043(5)	5649.1(12)	5415.5(13)	186.1(11)
C31_14	-2098(7)	5440.1(13)	5372.3(18)	186.1(11)
C32_14	-2350(6)	5310.9(10)	5515(2)	153(2)
C33_14	-2515(7)	5388.0(12)	5705(2)	186.1(11)
C34_14	-2452(7)	5596.9(12)	5746.9(15)	186.1(11)
C35_14	-2775(4)	6448.6(10)	5453.8(11)	97(3)
C36_14	-3031(4)	6389.7(11)	5283.1(12)	117(3)
C37_14	-3084(4)	6185.4(11)	5236.0(12)	122(4)
C38_14	-2831(4)	6042.0(11)	5357.3(13)	111(4)
C39_14	-3399(3)	6114.9(13)	5055.3(9)	153(3)
C40_14	-3467(3)	6243.3(14)	4885.2(11)	149.2(11)
C41_14	-3757(3)	6172.6(17)	4709.5(9)	149.2(11)
C42_14	-3964(3)	5975.9(19)	4715.1(13)	148(3)
C43_14	-3909(4)	5845.6(14)	4876.5(15)	149.2(11)
C44_14	-3626(3)	5914.6(13)	5047.7(13)	149.2(11)
P1_4	336.4(9)	6585.8(4)	5771.3(4)	55.8(7)
O1_4	53.0(19)	6645.9(8)	5965.9(5)	52.6(15)
O2_4	406(2)	6362.7(5)	5727.7(8)	60.0(17)
O3_4	928.4(14)	6683.0(6)	5754.9(8)	64.6(17)
O4_4	11.8(16)	6698.9(7)	5593.9(5)	63.7(17)
C1_4	673(2)	6876.0(8)	5394.3(7)	72(3)
C2_4	809(3)	7000.3(7)	5580.7(8)	74(3)
C3_4	948(2)	6898.2(7)	5752.0(7)	69(2)
C4_4	1085(3)	6992.0(8)	5939.8(8)	83(3)
C5_4	1075(5)	7205.5(8)	5948.0(10)	126(2)
C6_4	939(4)	7322.6(8)	5774.2(9)	125(3)
C7_4	791(3)	7218.1(7)	5587.0(9)	87(3)
C8_4	1154(4)	7304.5(11)	6156.3(9)	128(3)
C9_4	688(4)	7360(2)	6273.7(16)	187(4)
C10_4	767(5)	7431(2)	6478.6(15)	187(4)
C11_4	1299(6)	7439(2)	6545.6(14)	187(4)
C12_4	1747(5)	7390(3)	6445.9(16)	187(4)
C13_4	1667(4)	7323(2)	6237.0(15)	187(4)
C14_4	913(5)	7537.5(8)	5778.2(11)	125(3)
C15_4	753(4)	7648.4(9)	5609.7(11)	105(3)
C16_4	612(4)	7548.1(9)	5432.6(12)	105(3)
C17_4	633(4)	7341.8(9)	5426.0(10)	105(3)
C18_4	732(4)	7879.2(7)	5617.7(12)	100(3)
C19_4	499(4)	7984.8(11)	5784.2(12)	129(3)
C20_4	446(5)	8199.1(11)	5781.4(16)	129(3)
C21_4	631(5)	8305.0(10)	5612.2(19)	129(3)
C22_4	857(6)	8208.5(11)	5443.4(17)	129(3)
C23_4	912(5)	7994.9(11)	5447.6(14)	129(3)
C24_4	272(2)	6738.4(9)	5406.3(6)	61(2)
C25_4	66(3)	6628.9(12)	5236.8(7)	75(3)
C26_4	274(3)	6666.3(14)	5047.2(8)	96(3)
C27_4	715(3)	6801.7(16)	5020.3(8)	129(5)
C28_4	911(3)	6914.7(11)	5195.9(8)	93(3)

Atom	x	y	z	U_{eq}
C29_4	-21(4)	6579.5(13)	4869.2(9)	155(5)
C30_4	-154(5)	6371.4(13)	4856.0(15)	194(6)
C31_4	-470(6)	6296.4(17)	4694.0(19)	195(6)
C32_4	-655(5)	6431(2)	4542.7(16)	183(7)
C33_4	-528(6)	6640(2)	4554.0(16)	202(6)
C34_4	-210(6)	6712.4(15)	4716.3(15)	202(6)
C35_4	1404(3)	7030.6(16)	5168.5(11)	108(4)
C36_4	1663(4)	7044.7(16)	4989.1(11)	114(4)
C37_4	1504(4)	6926.6(18)	4825.3(11)	128(5)
C38_4	1029(4)	6816.1(16)	4838.3(10)	113(4)
C39_4	1798(3)	6933.7(16)	4626.8(10)	150(5)
C40_4	2351(3)	6988.5(16)	4619.9(15)	229(8)
C41_4	2634(4)	6992.4(19)	4429.0(18)	228(8)
C42_4	2344(6)	6941(2)	4255.6(16)	195(7)
C43_4	1808(6)	6887(3)	4254.4(11)	259(9)
C44_4	1532(4)	6883(2)	4440.5(12)	259(9)

Table 10.1.3: Anisotropic Displacement Parameters (×10[4]) for ZC-01-51. The anisotropic displacement factor exponent takes the form: $-2\pi^2[h^2a^{*2} \times U_{11} + ... +2hka^* \times b^* \times U_{12}]$

Atom	U_{11}	U_{22}	U_{33}	U_{23}	U_{13}	U_{12}
Rh1	39.8(4)	52.5(6)	41.9(4)	-2.9(4)	-5.7(3)	4.8(4)
Rh2	42.9(4)	53.6(6)	48.2(4)	-5.0(4)	-2.8(3)	6.6(4)
Rh3	41.8(4)	65.7(6)	52.7(5)	17.6(4)	6.7(4)	9.4(4)
Rh4	41.8(4)	61.4(6)	47.1(4)	11.7(4)	6.6(3)	11.7(4)
Rh5	58.1(5)	65.4(6)	47.5(4)	0.2(6)	-4.7(4)	1.4(4)
O	96(7)	226(12)	136(9)	-147(10)	-29(6)	39(7)
P1_1	44.0(12)	50.7(18)	60.8(16)	9.1(13)	7.5(11)	0.6(11)
O1_1	59(4)	56(2)	57(4)	13(2)	13(3)	10(2)
O2_1	49(3)	53(4)	51(3)	3(3)	2(2)	-10(3)
O3_1	42(2)	72(3)	69(2)	-4(2)	7(2)	1(3)
O4_1	47.2(17)	43(4)	75(4)	5(3)	1.3(19)	1.1(17)
C1_1	73(6)	57(5)	73(3)	-6(3)	6(3)	6(4)
C2_1	43(2)	59(6)	71(3)	-7(4)	7(2)	0(3)
C3_1	43(2)	48(6)	61(3)	-17(3)	7(2)	-1(2)
C4_1	59(3)	82(7)	69(4)	-2(4)	9(3)	10(4)
C5_1	75(3)	112(5)	82(3)	-1(3)	22(2)	1(3)
C6_1	74(3)	107(5)	85(3)	-3(3)	23(2)	3(3)
C7_1	56(2)	64(6)	78(4)	-20(4)	20(2)	-9(3)
C8_1	77(4)	115(5)	80(3)	-1(3)	22(3)	3(4)
C9_1	239(19)	117(5)	93(6)	-1(4)	-17(8)	-10(4)
C10_1	183(9)	150(7)	102(6)	19(5)	-5(7)	-9(8)
C11_1	182(9)	153(7)	102(6)	18(6)	-5(6)	-9(8)
C12_1	182(9)	153(7)	102(5)	17(6)	-4(6)	-9(8)
C13_1	111(9)	126(6)	84(4)	-4(4)	14(4)	-4(6)
C14_1	75(3)	106(5)	85(4)	-5(3)	24(3)	2(3)
C15_1	78(3)	90(8)	104(5)	-32(5)	16(3)	7(4)
C16_1	58(3)	79(6)	103(5)	-35(4)	16(3)	0(3)
C17_1	58(3)	80(6)	102(5)	-35(4)	15(3)	-1(3)
C18_1	113(4)	142(8)	103(7)	-69(5)	34(5)	-41(5)
C19_1	123(5)	203(8)	180(8)	-1(6)	69(5)	-12(5)
C20_1	124(5)	204(8)	181(8)	-1(5)	70(5)	-12(5)
C21_1	125(5)	204(8)	182(8)	0(6)	72(5)	-11(5)

Atom	U_{11}	U_{22}	U_{33}	U_{23}	U_{13}	U_{12}
C22_1	127(5)	205(8)	184(8)	2(6)	72(5)	-11(5)
C23_1	127(5)	206(9)	185(8)	4(6)	72(5)	-11(5)
C24_1	64(5)	50(4)	66(3)	0(3)	-3(3)	13(3)
C25_1	91(6)	20(5)	63(3)	-11(3)	-13(3)	-4(4)
C26_1	111(7)	32(5)	99(4)	2(3)	-1(4)	11(5)
C27_1	86(7)	40(5)	105(4)	-11(3)	-6(4)	-2(4)
C28_1	45(5)	49(4)	107(4)	-6(3)	-3(4)	-10(3)
C29_1	189(11)	167(9)	107(5)	30(5)	-3(5)	67(8)
C30_1	281(13)	219(10)	178(10)	83(7)	-25(9)	17(9)
C31_1	282(13)	219(10)	178(10)	83(7)	-26(9)	17(9)
C32_1	219(14)	220(11)	200(13)	30(9)	3(10)	61(9)
C33_1	298(13)	250(11)	165(9)	33(8)	-55(9)	-6(10)
C34_1	299(13)	249(11)	164(9)	31(8)	-55(9)	-6(10)
C35_1	51(6)	45(5)	121(5)	-10(4)	18(4)	-29(4)
C36_1	77(7)	53(6)	142(6)	-8(5)	15(5)	-15(4)
C37_1	74(6)	42(6)	142(6)	-11(4)	13(5)	-22(4)
C38_1	82(7)	42(5)	131(6)	-2(4)	2(5)	-2(4)
C39_1	69(6)	52(6)	243(9)	33(4)	57(5)	-17(4)
C40_1	55(6)	75(6)	277(9)	4(6)	57(5)	-11(4)
C41_1	55(6)	75(6)	278(9)	5(6)	57(5)	-10(4)
C42_1	127(12)	96(10)	309(10)	1(7)	10(8)	0(9)
C43_1	140(9)	72(8)	280(9)	28(6)	8(7)	1(6)
C44_1	140(9)	72(8)	278(9)	28(6)	8(7)	1(6)
P1_2	59.4(19)	179(4)	195(4)	-128(4)	-17(2)	13(2)
O1_2	78(4)	282(11)	203(11)	-200(10)	-51(4)	56(5)
O2_2	66(4)	120(8)	203(8)	-92(7)	-31(4)	11(4)
O3_2	63(5)	151(6)	197(4)	-123(4)	-14(3)	14(4)
O4_2	60(4)	182(4)	165(7)	-122(4)	-16(4)	11(3)
C1_2	58(5)	117(7)	120(5)	-78(5)	-1(3)	-3(4)
C2_2	64(5)	118(5)	122(7)	-79(5)	-1(4)	3(4)
C3_2	62(6)	118(5)	169(7)	-86(5)	-11(5)	3(4)
C4_2	61(7)	125(6)	227(9)	-58(6)	-30(7)	-12(4)
C5_2	72(4)	110(5)	272(7)	-67(5)	-63(4)	10(3)
C6_2	70(4)	110(5)	269(7)	-68(5)	-64(4)	10(3)
C7_2	72(6)	111(5)	185(8)	-109(5)	-38(6)	38(4)
C8_2	73(4)	110(5)	275(7)	-66(5)	-64(5)	10(4)
C9_2	114(7)	150(13)	282(8)	-44(7)	-45(6)	53(8)
C10_2	137(7)	112(7)	321(10)	-73(9)	-89(8)	45(5)
C11_2	137(7)	112(7)	321(10)	-72(9)	-90(8)	45(5)
C12_2	137(7)	113(8)	319(10)	-72(9)	-90(8)	45(6)
C13_2	110(8)	102(10)	275(7)	-67(6)	-63(5)	31(7)
C14_2	70(5)	110(5)	269(7)	-69(5)	-64(5)	11(4)
C15_2	123(8)	111(5)	390(20)	-59(6)	-138(12)	3(4)
C16_2	119(8)	112(5)	301(14)	-98(6)	-103(8)	32(4)
C17_2	118(8)	112(5)	301(14)	-100(6)	-103(8)	33(4)
C18_2	136(6)	153(12)	600(20)	-115(14)	-177(9)	1(5)
C19_2	145(7)	173(10)	637(18)	-144(10)	-171(9)	-1(6)
C20_2	146(7)	172(9)	638(18)	-144(10)	-172(9)	-1(6)
C21_2	146(7)	172(9)	639(18)	-144(10)	-171(9)	-1(6)
C22_2	146(6)	172(10)	640(18)	-144(10)	-172(9)	2(6)
C23_2	143(6)	171(10)	641(18)	-142(10)	-172(9)	5(6)
C24_2	61(6)	146(7)	120(5)	-82(5)	-2(3)	1(5)
C25_2	62(6)	157(8)	131(6)	-73(6)	-10(5)	-6(5)
C26_2	69(6)	165(9)	116(5)	-70(5)	-15(4)	4(6)
C27_2	51(6)	187(10)	117(5)	-73(5)	-20(4)	22(6)
C28_2	88(7)	181(9)	104(5)	-103(5)	-35(4)	46(6)

Atom	U_{11}	U_{22}	U_{33}	U_{23}	U_{13}	U_{12}
C29_2	91(7)	257(10)	138(8)	-36(8)	-14(6)	35(6)
C30_2	162(9)	346(13)	326(18)	54(12)	-121(9)	-38(9)
C31_2	163(9)	346(12)	326(18)	54(12)	-119(10)	-37(9)
C32_2	257(15)	338(13)	280(20)	28(13)	-43(14)	-32(10)
C33_2	284(14)	319(11)	354(17)	73(10)	-97(11)	-42(8)
C34_2	284(14)	318(10)	354(17)	73(10)	-97(11)	-42(8)
C35_2	88(7)	200(14)	104(5)	-101(6)	-38(4)	73(7)
C36_2	106(8)	227(14)	99(6)	-79(8)	-31(5)	100(9)
C37_2	101(8)	250(14)	106(6)	-61(7)	-23(5)	102(9)
C38_2	68(7)	224(13)	105(6)	-71(7)	-28(5)	51(7)
C39_2	139(9)	326(12)	94(9)	-40(9)	-21(6)	163(8)
C40_2	272(13)	323(12)	304(18)	15(12)	151(13)	220(10)
C41_2	273(13)	325(12)	304(18)	14(12)	151(13)	220(10)
C42_2	295(19)	296(12)	138(15)	-56(10)	57(14)	190(11)
C43_2	355(17)	380(12)	340(20)	76(12)	167(15)	261(9)
C44_2	355(17)	380(12)	340(20)	75(12)	167(15)	261(9)
P1_3	45.7(12)	83(2)	43.7(14)	14.8(13)	4.5(10)	13.9(12)
O1_3	45(3)	60(5)	43.3(17)	13(2)	4.1(17)	16(3)
O2_3	35(3)	93(5)	48(3)	26(3)	9(2)	8(3)
O3_3	43(2)	83(2)	52(3)	13.0(19)	16(2)	9(2)
O4_3	44.6(16)	70(4)	39(3)	13(2)	4.1(16)	18.1(18)
C1_3	55(5)	88(4)	40(4)	0(3)	5(3)	7(3)
C2_3	49(2)	79(4)	32(4)	-8(3)	9(2)	12(3)
C3_3	49(2)	72(5)	40(4)	-7(3)	9(2)	13(2)
C4_3	66(3)	89(5)	64(5)	12(4)	11(3)	9(3)
C5_3	81(3)	98(4)	52(3)	-6(3)	8(2)	27(3)
C6_3	80(3)	100(4)	51(3)	-6(3)	7(2)	27(3)
C7_3	62(3)	105(5)	54(5)	-2(4)	3(3)	28(3)
C8_3	83(3)	96(4)	53(3)	-6(3)	9(3)	26(3)
C9_3	153(9)	93(7)	60(3)	1(3)	-12(3)	1(6)
C10_3	171(9)	135(7)	76(5)	27(5)	-27(5)	-26(5)
C11_3	171(8)	136(7)	79(5)	25(5)	-27(5)	-27(5)
C12_3	171(9)	137(7)	80(5)	24(5)	-28(5)	-27(5)
C13_3	170(10)	137(7)	77(6)	22(5)	-33(6)	-33(7)
C14_3	80(3)	102(5)	52(4)	-6(3)	7(3)	28(3)
C15_3	81(3)	123(6)	35(5)	-23(4)	5(3)	20(3)
C16_3	64(3)	137(6)	56(5)	-5(4)	6(3)	26(3)
C17_3	63(3)	137(6)	56(5)	-5(4)	6(3)	24(3)
C18_3	106(6)	184(10)	100(5)	11(5)	2(4)	51(7)
C19_3	87(5)	168(7)	98(5)	5(5)	-6(4)	30(4)
C20_3	89(5)	168(6)	99(5)	4(4)	-7(4)	31(4)
C21_3	91(5)	170(6)	101(5)	3(5)	-5(4)	32(4)
C22_3	92(4)	170(6)	101(5)	3(5)	-4(4)	34(4)
C23_3	93(5)	169(7)	100(5)	2(5)	-4(4)	35(4)
C24_3	38(4)	86(4)	38(3)	-1(3)	1(3)	1(3)
C25_3	119(8)	85(5)	37(5)	-5(3)	14(4)	-7(4)
C26_3	166(9)	140(5)	58(6)	27(4)	46(6)	41(4)
C27_3	73(7)	132(5)	61(5)	11(3)	17(4)	9(4)
C28_3	62(6)	125(5)	44(4)	0(3)	11(4)	9(4)
C29_3	217(6)	168(6)	120(12)	67(7)	0(6)	4(4)
C30_3	225(8)	195(10)	242(15)	129(10)	-33(9)	-15(7)
C31_3	225(7)	194(10)	242(15)	128(10)	-32(9)	-16(7)
C32_3	222(8)	222(12)	266(18)	29(11)	-4(9)	-15(7)
C33_3	221(6)	217(6)	222(6)	5(3)	3(3)	-8(3)
C34_3	222(6)	218(7)	224(7)	6(4)	4(4)	-9(3)
C35_3	72(6)	123(5)	36(5)	6(4)	14(4)	12(4)

Atom	U_{11}	U_{22}	U_{33}	U_{23}	U_{13}	U_{12}
C36_3	76(6)	118(6)	39(5)	-15(4)	18(4)	-20(4)
C37_3	51(5)	122(6)	38(4)	-9(4)	-4(3)	-23(4)
C38_3	71(6)	122(6)	64(5)	-6(4)	19(4)	-22(5)
C39_3	60(5)	179(7)	44(4)	-6(4)	4(3)	-19(4)
C40_3	57(5)	185(7)	52(5)	-15(4)	5(3)	-14(4)
C41_3	56(5)	185(7)	52(5)	-14(5)	4(3)	-13(4)
C42_3	76(7)	204(8)	94(8)	-31(6)	23(6)	-32(6)
C43_3	90(6)	207(8)	94(7)	-34(5)	38(5)	-47(6)
C44_3	90(6)	207(8)	94(7)	-34(6)	38(5)	-47(6)
P1_5	55.5(13)	72(2)	40.7(14)	2.7(13)	-5.8(10)	-4.0(12)
O1_5	54(3)	71(2)	39(3)	4(2)	-14(3)	-1(2)
O2_5	82(4)	57(5)	38.8(17)	8(2)	-6.5(18)	-11(3)
O3_5	53.3(17)	85(5)	38(2)	6(2)	-8.8(17)	-4(2)
O4_5	59(3)	83(3)	46(3)	11(2)	-2(3)	1(2)
C1_5	78(5)	85(4)	44(5)	10(3)	-1(4)	-7(3)
C2_5	102(8)	90(4)	41(3)	7(3)	-13(3)	7(4)
C3_5	61(6)	86(4)	38(2)	6(2)	-8(2)	-2(4)
C4_5	69(6)	85(5)	65(3)	4(3)	13(4)	-7(4)
C5_5	150(6)	122(5)	65(3)	-1(3)	13(4)	17(4)
C6_5	149(6)	125(5)	65(3)	1(3)	11(4)	16(4)
C7_5	84(7)	120(5)	41(3)	5(3)	-13(3)	7(4)
C8_5	152(6)	122(5)	66(4)	-2(3)	15(4)	17(4)
C9_5	144(7)	191(8)	320(30)	-151(12)	1(8)	39(6)
C10_5	210(11)	174(8)	301(16)	-117(9)	85(12)	27(7)
C11_5	211(11)	172(8)	301(16)	-118(9)	85(12)	28(7)
C12_5	210(11)	173(8)	302(16)	-117(9)	84(12)	27(7)
C13_5	172(6)	211(10)	400(40)	-199(16)	-64(9)	61(6)
C14_5	150(6)	128(5)	65(3)	0(3)	11(4)	17(4)
C15_5	570(40)	224(8)	93(4)	23(4)	-17(6)	173(15)
C16_5	112(7)	168(6)	69(4)	41(4)	-6(5)	26(5)
C17_5	111(7)	167(6)	69(4)	40(4)	-6(5)	26(5)
C18_5	164(8)	512(3)	99(5)	-23(2)	-4(3)	210(4)
C19_5	227(2)	527(2)	87.7(19)	-23.4(19)	-8.9(19)	185(2)
C20_5	227(2)	527(2)	87.7(19)	-23.4(19)	-8.9(19)	185(2)
C21_5	226(3)	527(2)	87(3)	-23(2)	-10(3)	185(2)
C22_5	227(2)	527(2)	87.7(19)	-23.4(19)	-8.9(19)	185(2)
C23_5	227(2)	527(2)	87.7(19)	-23.4(19)	-8.9(19)	185(2)
C24_5	79(5)	87(3)	46(5)	9(3)	1(3)	-8(3)
C25_5	77(7)	89(4)	79(7)	0(4)	13(5)	-13(3)
C26_5	72(6)	92(4)	114(8)	10(4)	10(5)	-13(4)
C27_5	84(6)	87(4)	131(8)	20(5)	24(6)	-5(4)
C28_5	68(5)	87(4)	101(8)	25(4)	4(5)	-1(3)
C29_5	79(7)	94(5)	195(7)	-4(5)	33(5)	-18(5)
C30_5	89(7)	94(8)	195(7)	-4(5)	29(5)	-5(5)
C31_5	91(7)	94(8)	196(7)	-5(5)	29(5)	-4(5)
C32_5	80(8)	100(9)	205(7)	6(6)	27(6)	-14(6)
C33_5	136(9)	127(8)	206(7)	1(6)	23(6)	15(6)
C34_5	136(9)	127(8)	206(7)	1(6)	23(6)	15(6)
C35_5	71(5)	76(4)	122(9)	46(6)	14(5)	13(4)
C36_5	66(6)	78(3)	89(8)	47(5)	-8(5)	9(3)
C37_5	52(5)	79(3)	143(9)	32(4)	0(5)	-1(3)
C38_5	65(6)	90(5)	165(10)	37(6)	17(6)	2(4)
C39_5	88.1(19)	88(3)	170(13)	41(5)	-9(3)	-17(2)
C40_5	87.8(18)	86.4(18)	146(2)	34.7(18)	-10.6(18)	-17.7(16)
C41_5	87.8(18)	86.4(18)	146(2)	34.7(18)	-10.6(18)	-17.7(16)
C42_5	87.5(19)	85(3)	97(9)	22(4)	-14(2)	-18(2)

Atom	U_{11}	U_{22}	U_{33}	U_{23}	U_{13}	U_{12}
C43_5	87.8(18)	86.4(18)	146(2)	34.7(18)	-10.6(18)	-17.7(16)
C44_5	87.8(18)	86.4(18)	146(2)	34.7(18)	-10.6(18)	-17.7(16)
P1_6	60.8(17)	181(3)	111(2)	97(3)	27.8(17)	41(2)
O1_6	69(3)	192(9)	141(7)	119(7)	43(4)	51(4)
O2_6	74(3)	227(9)	92(6)	95(7)	36(4)	69(5)
O3_6	49(4)	184(3)	105(5)	88(4)	20(3)	40(2)
O4_6	67(4)	143(6)	116(3)	93(3)	21(2)	36(3)
C1_6	50(4)	100(4)	52(5)	37(4)	-2(3)	4(3)
C2_6	40(4)	94(5)	63(3)	33(3)	1(2)	-5(3)
C3_6	52(5)	135(7)	64(3)	40(3)	5(2)	30(5)
C4_6	54(5)	132(6)	80(4)	36(4)	15(3)	22(5)
C5_6	38(3)	104(4)	70(3)	22(3)	5(2)	1(2)
C6_6	40(3)	105(4)	70(3)	22(3)	5(2)	-1(2)
C7_6	40(4)	95(5)	59(3)	32(3)	5(2)	-2(3)
C8_6	37(3)	104(4)	72(3)	21(3)	6(2)	0(3)
C9_6	96(6)	125(5)	175(12)	-31(6)	82(8)	-38(5)
C10_6	93(5)	120(6)	107(6)	8(6)	49(4)	-4(4)
C11_6	93(5)	120(6)	109(6)	9(5)	47(4)	-3(4)
C12_6	93(5)	119(6)	113(6)	10(5)	46(4)	-3(4)
C13_6	77(6)	110(4)	118(9)	4(4)	43(6)	-16(3)
C14_6	39(3)	104(4)	68(3)	22(3)	6(2)	-2(3)
C15_6	57(4)	80(5)	68(3)	22(3)	5(2)	-6(4)
C16_6	60(4)	83(6)	58(3)	28(3)	2(3)	2(3)
C17_6	60(4)	84(6)	59(3)	29(3)	3(2)	2(3)
C18_6	60(4)	102(5)	65(5)	34(4)	20(3)	8(3)
C19_6	81(4)	106(5)	92(5)	23(4)	-3(3)	18(3)
C20_6	82(4)	106(5)	92(5)	23(4)	-3(3)	19(3)
C21_6	82(4)	107(5)	92(5)	22(4)	-2(3)	19(3)
C22_6	81(4)	107(5)	91(5)	22(4)	-3(3)	20(4)
C23_6	81(4)	107(5)	90(5)	21(4)	-3(3)	20(4)
C24_6	61(5)	100(4)	63(5)	39(4)	-3(3)	3(3)
C25_6	53(5)	105(5)	109(7)	14(5)	4(4)	-10(4)
C26_6	45(5)	87(4)	107(7)	15(4)	0(4)	-2(3)
C27_6	52(5)	86(4)	81(6)	9(4)	8(4)	-1(3)
C28_6	54(4)	80(4)	52(5)	27(4)	3(3)	6(3)
C29_6	85(7)	105(7)	231(8)	-27(7)	57(6)	-13(6)
C30_6	325(15)	302(15)	313(11)	-52(10)	-19(11)	191(12)
C31_6	325(15)	302(15)	312(11)	-52(10)	-18(11)	191(12)
C32_6	303(19)	283(19)	308(11)	-36(11)	-2(11)	68(15)
C33_6	238(14)	248(14)	234(8)	-46(7)	44(7)	38(10)
C34_6	237(14)	247(14)	233(8)	-45(7)	43(7)	38(10)
C35_6	53(5)	79(4)	95(8)	25(4)	30(5)	9(3)
C36_6	50(5)	77(5)	59(6)	-1(4)	15(4)	-9(3)
C37_6	51(4)	80(5)	42(5)	5(4)	6(3)	-1(3)
C38_6	53(5)	80(5)	79(7)	5(4)	10(4)	-1(3)
C39_6	70(4)	65(6)	92(5)	3(4)	39(4)	4(4)
C40_6	69(5)	84(6)	133(6)	-27(5)	52(4)	-2(4)
C41_6	70(5)	85(6)	134(6)	-27(5)	53(4)	-2(4)
C42_6	106(8)	136(10)	150(7)	-54(6)	74(6)	-32(7)
C43_6	88(6)	93(7)	100(5)	-11(5)	49(4)	-5(5)
C44_6	87(6)	92(7)	99(5)	-11(4)	48(4)	-5(5)
O1S_7	87(4)	136(7)	71(5)	-28(4)	-32(4)	3(4)
C2S_7	154(7)	214(10)	211(15)	-28(8)	-36(8)	-72(7)
C3S_7	310(30)	244(17)	295(19)	24(15)	-47(16)	-160(20)
C4S_7	125(6)	153(11)	189(12)	17(8)	35(7)	29(6)
C5S_7	55(8)	237(17)	214(13)	69(12)	-6(7)	1(8)

Atom	U_{11}	U_{22}	U_{33}	U_{23}	U_{13}	U_{12}
O1S_8	90(5)	148(6)	65(5)	28(5)	2(4)	9(5)
C2S_8	101(5)	174(11)	116(10)	62(7)	-24(6)	-11(6)
C3S_8	320(20)	222(16)	430(30)	-48(14)	-300(20)	84(13)
C4S_8	101(5)	181(7)	98(11)	49(8)	-16(5)	-15(6)
C5S_8	192(15)	187(8)	380(40)	-1(11)	110(20)	-49(8)
P1_9	57.1(13)	59.9(18)	49.3(15)	7.7(14)	2.9(11)	15.9(13)
O1_9	69(3)	41(4)	57(2)	11(3)	-9(2)	2(3)
O2_9	55(4)	60(2)	74(5)	12(2)	-8(3)	12.8(19)
O3_9	58.4(17)	65(3)	60(4)	4(3)	8(2)	14(2)
O4_9	46(3)	56(4)	48.3(19)	5(2)	-0.4(18)	6(2)
C1_9	48(4)	67(5)	72(4)	0(3)	-6(3)	12(3)
C2_9	44(5)	65(3)	75(3)	2(3)	-7(3)	10(3)
C3_9	59(6)	65(3)	75(4)	4(3)	-4(4)	13(2)
C4_9	92(8)	91(4)	81(4)	16(3)	-7(4)	-5(4)
C5_9	144(6)	91(4)	110(4)	17(3)	-11(4)	-5(4)
C6_9	140(6)	92(4)	111(4)	17(3)	-11(4)	-3(4)
C7_9	66(6)	65(3)	100(4)	2(3)	-18(4)	11(3)
C8_9	148(6)	93(5)	111(4)	18(3)	-13(4)	-11(4)
C9_9	148(7)	195(15)	163(7)	101(9)	11(5)	9(8)
C10_9	226(12)	241(14)	167(7)	110(7)	9(6)	-43(10)
C11_9	226(12)	241(14)	166(7)	111(7)	10(6)	-43(10)
C12_9	225(12)	242(14)	165(7)	111(7)	12(6)	-43(10)
C13_9	189(8)	162(16)	162(8)	96(9)	35(6)	48(9)
C14_9	138(6)	92(4)	114(4)	18(3)	-11(4)	-1(5)
C15_9	98(9)	129(5)	127(5)	-4(4)	-18(5)	2(5)
C16_9	84(6)	96(5)	119(5)	-20(4)	-15(5)	12(6)
C17_9	84(6)	96(5)	119(5)	-21(4)	-12(4)	11(6)
C18_9	132(10)	129(5)	215(9)	0(4)	58(7)	7(5)
C19_9	121(7)	128(5)	211(9)	1(5)	67(6)	10(5)
C20_9	120(6)	128(5)	213(8)	0(5)	66(6)	10(5)
C21_9	123(6)	128(5)	215(8)	-1(5)	64(6)	11(5)
C22_9	125(7)	128(5)	216(9)	-2(5)	61(6)	10(5)
C23_9	125(7)	128(5)	216(9)	-3(5)	59(6)	10(5)
C24_9	50(4)	70(5)	53(3)	-2(3)	-5(3)	10(3)
C25_9	58(5)	75(6)	54(4)	1(4)	-10(3)	2(4)
C26_9	103(6)	143(8)	71(4)	25(5)	-37(4)	-40(6)
C27_9	72(5)	104(7)	70(4)	-5(4)	-13(3)	-6(4)
C28_9	56(4)	110(7)	75(4)	1(4)	-11(3)	2(4)
C29_9	145(7)	163(9)	81(6)	38(6)	-38(5)	-52(6)
C30_9	169(7)	219(12)	170(10)	82(8)	8(6)	-32(7)
C31_9	170(7)	219(12)	170(10)	82(8)	9(7)	-31(7)
C32_9	168(7)	186(12)	184(13)	44(10)	-30(7)	-48(7)
C33_9	163(7)	188(10)	157(10)	64(6)	-42(6)	-36(7)
C34_9	162(7)	187(9)	157(10)	63(6)	-42(6)	-36(7)
C35_9	56(5)	110(8)	105(6)	8(5)	-19(4)	2(5)
C36_9	58(6)	120(10)	107(5)	17(5)	-22(4)	-7(6)
C37_9	92(6)	118(9)	103(5)	11(5)	-26(4)	-27(6)
C38_9	78(5)	97(9)	71(5)	0(5)	-17(4)	-10(5)
C39_9	130(5)	47(8)	125(5)	30(5)	-55(4)	-52(5)
C40_9	130(5)	102(8)	163(8)	56(8)	-54(4)	-54(5)
C41_9	130(5)	103(8)	163(8)	56(8)	-53(4)	-55(6)
C42_9	123(6)	184(17)	161(8)	51(8)	-57(5)	-73(7)
C43_9	123(6)	171(10)	123(5)	23(6)	-61(4)	-76(6)
C44_9	123(6)	172(10)	122(5)	24(6)	-60(4)	-75(7)
P1_10	52.3(14)	76(2)	57.9(16)	8.6(14)	5.4(12)	-4.3(13)
O1_10	62(4)	72(3)	45(4)	-2(3)	13(3)	-6(2)

Atom	U_{11}	U_{22}	U_{33}	U_{23}	U_{13}	U_{12}
O2_10	63(4)	82(6)	58.5(17)	12(2)	4.8(18)	-16(4)
O3_10	52.9(16)	78(5)	74(3)	10(3)	3(2)	-5(2)
O4_10	60(3)	81(3)	78(4)	-3(3)	12(3)	-8(2)
C1_10	78(5)	72(4)	109(7)	16(4)	-30(5)	-13(3)
C2_10	69(6)	72(4)	74(3)	5(2)	-2(3)	3(3)
C3_10	43(5)	71(4)	74(3)	7(2)	5(2)	-2(4)
C4_10	67(6)	71(5)	67(3)	2(3)	3(3)	5(4)
C5_10	56(3)	82(3)	66(3)	5(2)	5(2)	-9(3)
C6_10	55(3)	82(3)	67(3)	4(2)	5(3)	-8(3)
C7_10	42(5)	81(4)	74(3)	7(2)	3(3)	-14(3)
C8_10	60(3)	82(3)	68(3)	5(2)	3(3)	-8(3)
C9_10	81(5)	67(4)	70(5)	-9(4)	15(4)	-16(3)
C10_10	141(7)	74(4)	79(6)	-3(4)	7(4)	-2(4)
C11_10	141(7)	75(4)	78(6)	0(4)	7(4)	-1(4)
C12_10	140(7)	76(4)	79(6)	2(4)	7(4)	1(3)
C13_10	69(5)	77(4)	96(8)	10(4)	21(5)	-9(4)
C14_10	53(4)	81(4)	67(3)	2(2)	5(3)	-7(3)
C15_10	102(7)	79(4)	63(3)	5(3)	20(4)	-13(4)
C16_10	134(7)	82(4)	73(4)	5(3)	2(5)	-10(4)
C17_10	135(7)	82(4)	73(4)	6(3)	2(4)	-9(4)
C18_10	157(7)	96(8)	69(3)	4(4)	1(4)	-13(6)
C19_10	178(6)	145(7)	77(4)	-15(4)	-10(4)	19(5)
C20_10	179(6)	145(7)	76(4)	-15(4)	-10(4)	18(5)
C21_10	181(6)	146(7)	77(4)	-15(4)	-8(4)	18(5)
C22_10	182(7)	148(7)	77(4)	-15(4)	-8(4)	19(5)
C23_10	183(7)	149(7)	77(4)	-15(4)	-8(4)	20(5)
C24_10	67(4)	81(4)	76(7)	11(4)	-10(4)	-10(3)
C25_10	122(8)	104(6)	214(15)	66(8)	-98(10)	-45(5)
C26_10	169(8)	132(7)	312(18)	113(10)	-147(10)	-80(6)
C27_10	136(7)	96(6)	204(14)	52(8)	-87(9)	-49(5)
C28_10	96(5)	81(5)	114(10)	15(5)	-30(5)	-26(4)
C29_10	221(11)	202(12)	294(13)	147(11)	-47(9)	17(9)
C30_10	205(10)	185(12)	263(12)	104(9)	-76(8)	-1(8)
C31_10	205(10)	186(12)	262(12)	104(9)	-77(8)	-1(8)
C32_10	232(11)	221(15)	260(13)	99(11)	-71(10)	-24(10)
C33_10	245(11)	212(12)	283(12)	122(8)	-47(9)	-19(9)
C34_10	245(11)	213(12)	282(12)	122(8)	-48(9)	-20(9)
C35_10	91(5)	141(8)	64(8)	41(7)	-13(5)	-37(4)
C36_10	108(7)	152(8)	101(11)	46(7)	-14(7)	-50(5)
C37_10	152(7)	149(8)	143(13)	53(8)	-68(7)	-68(5)
C38_10	154(7)	105(7)	169(15)	37(9)	-75(8)	-62(6)
C39_10	196(7)	182(8)	112(10)	53(7)	-86(7)	-109(6)
C40_10	194(7)	218(10)	127(9)	24(9)	-92(7)	-112(6)
C41_10	194(7)	217(10)	128(9)	23(9)	-92(7)	-113(6)
C42_10	235(10)	240(11)	400(40)	85(14)	-66(13)	-89(8)
C43_10	228(9)	185(8)	172(13)	56(8)	-101(10)	-102(7)
C44_10	228(9)	184(8)	171(13)	56(8)	-101(10)	-102(7)
O1S_11	103(5)	99(6)	79(5)	-17(5)	-9(4)	28(4)
C2S_11	141(7)	142(8)	230(14)	34(8)	43(7)	69(6)
C3S_11	145(9)	130(11)	202(15)	-14(10)	41(9)	57(8)
C4S_11	202(10)	310(20)	193(12)	-132(14)	-113(10)	89(12)
C5S_11	450(40)	1030(90)	249(15)	-380(30)	-215(18)	350(50)
O1S_12	60(4)	109(5)	76(5)	28(4)	0(3)	0(3)
C2S_12	127(7)	139(6)	80(8)	36(6)	-11(5)	-45(5)
C3S_12	177(10)	236(14)	260(20)	-63(15)	88(13)	-136(12)
C4S_12	68(5)	119(6)	86(5)	14(5)	-9(4)	10(4)

Atom	U_{11}	U_{22}	U_{33}	U_{23}	U_{13}	U_{12}
C5S_12	67(5)	126(9)	92(8)	21(7)	-6(5)	13(5)
P1_13	39.6(12)	46.7(17)	57.3(16)	-7.8(12)	-10.2(10)	6.1(10)
O1_13	56(3)	50(2)	56(4)	-13(2)	-20(3)	12(2)
O2_13	47(3)	43(3)	57(3)	-5(3)	-16(2)	6(2)
O3_13	40(2)	47(3)	60(2)	-4(2)	-11.6(18)	2(2)
O4_13	39.7(16)	44(3)	59(3)	-11(2)	-10.2(17)	4.1(17)
C1_13	44(4)	47(4)	55(3)	1(2)	-5(2)	2(3)
C2_13	39(2)	79(7)	63(3)	-15(4)	-13(2)	10(3)
C3_13	39(2)	56(6)	65(4)	-14(4)	-10.9(19)	4(2)
C4_13	61(4)	101(9)	76(4)	-37(5)	-9(3)	7(4)
C5_13	81(3)	227(8)	117(4)	-73(4)	-31(3)	-3(4)
C6_13	81(3)	225(8)	116(4)	-72(4)	-32(3)	-3(4)
C7_13	53(2)	59(7)	79(4)	8(4)	-30(2)	1(3)
C8_13	84(4)	229(8)	118(4)	-76(5)	-33(3)	-5(5)
C9_13	360(30)	224(10)	122(5)	-76(6)	-20(8)	32(11)
C10_13	336(17)	284(14)	130(6)	-102(7)	-26(9)	28(13)
C11_13	336(17)	284(14)	132(6)	-105(7)	-26(9)	28(13)
C12_13	335(17)	282(14)	131(6)	-106(7)	-26(9)	29(13)
C13_13	270(20)	271(9)	106(6)	-86(6)	-73(9)	87(10)
C14_13	83(3)	225(8)	116(4)	-73(5)	-32(3)	-3(4)
C15_13	88(4)	140(11)	114(5)	-10(6)	-23(3)	7(4)
C16_13	57(3)	83(6)	100(5)	18(4)	-25(3)	6(4)
C17_13	56(3)	83(6)	99(5)	19(4)	-24(3)	6(3)
C18_13	140(5)	237(18)	224(7)	-28(8)	-87(5)	-20(7)
C19_13	150(6)	338(10)	228(7)	-48(7)	-85(5)	-24(7)
C20_13	150(6)	337(10)	229(7)	-47(7)	-85(5)	-24(7)
C21_13	147(5)	336(10)	229(7)	-46(7)	-87(5)	-25(7)
C22_13	143(5)	335(10)	229(7)	-45(8)	-87(5)	-26(7)
C23_13	140(5)	334(10)	229(7)	-44(8)	-86(5)	-25(7)
C24_13	39(4)	40(4)	52(3)	-3(3)	-2(2)	2(3)
C25_13	58(5)	45(5)	52(3)	-4(3)	0(3)	-1(3)
C26_13	80(6)	55(5)	55(3)	-7(3)	-2(3)	13(4)
C27_13	54(5)	48(5)	57(3)	2(3)	4(3)	-5(3)
C28_13	49(5)	48(4)	56(3)	2(3)	-1(3)	4(3)
C29_13	91(7)	100(5)	58(3)	-20(3)	-9(3)	38(5)
C30_13	142(8)	105(5)	85(6)	-27(4)	-19(6)	26(5)
C31_13	144(8)	105(5)	86(6)	-27(4)	-19(6)	26(5)
C32_13	212(14)	113(6)	96(7)	-21(5)	1(7)	27(7)
C33_13	176(9)	109(6)	63(4)	-16(4)	9(6)	36(6)
C34_13	174(9)	108(6)	64(4)	-16(4)	9(6)	36(6)
C35_13	52(5)	49(5)	55(4)	0(3)	-2(3)	5(3)
C36_13	51(5)	46(5)	64(4)	4(3)	7(3)	2(3)
C37_13	64(5)	45(5)	65(4)	3(3)	11(3)	-2(3)
C38_13	52(4)	44(5)	64(4)	7(3)	15(3)	-9(3)
C39_13	65(5)	50(5)	88(5)	-13(3)	-1(4)	-1(3)
C40_13	80(6)	60(5)	113(6)	3(4)	10(4)	6(4)
C41_13	80(6)	61(5)	114(6)	3(4)	10(4)	7(4)
C42_13	81(7)	113(8)	112(6)	7(5)	9(5)	10(6)
C43_13	105(7)	113(8)	111(6)	7(5)	30(5)	26(5)
C44_13	105(7)	114(8)	111(6)	6(5)	30(5)	26(5)
P1_14	41.0(12)	83(2)	41.9(14)	-1.3(13)	-0.5(9)	11.9(12)
O1_14	37(3)	78(5)	44(2)	-5(2)	0.5(18)	15(3)
O2_14	45(3)	89(4)	52(3)	-12(3)	1(2)	9(3)
O3_14	58(3)	88(3)	59(3)	8(2)	-20(3)	1(2)
O4_14	42.5(17)	104(4)	36(3)	12(3)	-3.7(18)	7(2)
C1_14	42(5)	105(4)	49(4)	24(3)	-12(3)	-8(3)

Atom	U_{11}	U_{22}	U_{33}	U_{23}	U_{13}	U_{12}
C2_14	77(2)	103(4)	50(6)	26(3)	-10(3)	-8(2)
C3_14	66(3)	92(4)	102(8)	8(5)	-7(3)	5(2)
C4_14	109(3)	100(4)	126(11)	-4(5)	9(4)	-10(3)
C5_14	114.4(18)	131(2)	135(2)	-1.1(19)	6.2(19)	5.4(15)
C6_14	113.3(14)	128.5(15)	128.3(15)	3.3(13)	5.7(13)	6.0(12)
C7_14	106.8(17)	119(2)	81(8)	24(3)	7(2)	13.8(17)
C8_14	126(2)	133(2)	140(2)	-4.3(14)	7.2(17)	1.6(15)
C9_14	145(2)	142(2)	143(2)	0.4(12)	-2.3(13)	1.4(13)
C10_14	147(3)	143(2)	145(3)	-0.3(16)	-2(2)	0.5(16)
C11_14	146(3)	144(3)	144(2)	-0.3(16)	-2.5(15)	0.5(18)
C12_14	145(2)	142(2)	143(2)	0.4(12)	-2.3(13)	1.4(13)
C13_14	145(2)	142(2)	143(2)	0.4(12)	-2.3(13)	1.4(13)
C14_14	113.3(14)	128.5(15)	128.3(15)	3.3(13)	5.7(13)	6.0(12)
C15_14	113.3(14)	128.5(15)	128.3(15)	3.3(13)	5.7(13)	6.0(12)
C16_14	113.3(14)	128.5(15)	128.3(15)	3.3(13)	5.7(13)	6.0(12)
C17_14	113.3(14)	128.5(15)	128.3(15)	3.3(13)	5.7(13)	6.0(12)
C18_14	171(5)	186(10)	278(13)	37(10)	83(6)	53(6)
C19_14	184(6)	239(8)	354(12)	-27(7)	50(7)	85(6)
C20_14	184(6)	240(8)	356(12)	-27(7)	50(7)	86(7)
C21_14	184(6)	240(8)	356(12)	-26(7)	49(7)	86(7)
C22_14	184(6)	240(8)	355(12)	-27(7)	50(7)	86(7)
C23_14	184(6)	239(8)	354(12)	-27(7)	50(7)	85(6)
C24_14	52(4)	107(4)	39(4)	18(3)	-10(3)	1(3)
C25_14	65(6)	107(4)	44(5)	18(3)	-6(4)	2(3)
C26_14	88(7)	147(3)	55(5)	9(2)	-20(4)	-3(2)
C27_14	51(5)	146(4)	40(5)	14(3)	-1(3)	-14(4)
C28_14	39(5)	145(4)	50(4)	14(3)	-10(3)	-9(4)
C29_14	148(4)	149(2)	150(2)	-5.0(16)	-6(2)	-4(2)
C30_14	238.9(19)	152.2(18)	167.1(17)	-11.8(15)	33.5(17)	-19.3(17)
C31_14	238.9(19)	152.2(18)	167.1(17)	-11.8(15)	33.5(17)	-19.3(17)
C32_14	157(4)	151(3)	152(2)	-1.7(17)	1(2)	3(2)
C33_14	238.9(19)	152.2(18)	167.1(17)	-11.8(15)	33.5(17)	-19.3(17)
C34_14	238.9(19)	152.2(18)	167.1(17)	-11.8(15)	33.5(17)	-19.3(17)
C35_14	53(6)	187(6)	50(5)	30(4)	7(4)	21(5)
C36_14	71(7)	228(8)	51(5)	30(5)	4(4)	0(5)
C37_14	60(6)	230(8)	74(4)	22(5)	1(4)	0(5)
C38_14	75(7)	197(8)	59(5)	-6(5)	-20(5)	-9(6)
C39_14	77(8)	288(3)	94(4)	-4(3)	-17(4)	15(4)
C40_14	68.6(19)	286(2)	92.8(19)	-6.0(19)	-12.5(17)	18.9(19)
C41_14	68.6(19)	286(2)	92.8(19)	-6.0(19)	-12.5(17)	18.9(19)
C42_14	67(7)	285(3)	93(4)	-6(3)	-9(4)	21(3)
C43_14	68.6(19)	286(2)	92.8(19)	-6.0(19)	-12.5(17)	18.9(19)
C44_14	68.6(19)	286(2)	92.8(19)	-6.0(19)	-12.5(17)	18.9(19)
P1_4	48.6(13)	60.4(18)	58.4(16)	3.6(14)	-3.6(11)	13.2(12)
O1_4	47(3)	56(4)	55(2)	2(2)	-8(2)	12(3)
O2_4	49(4)	61(2)	70(5)	0(2)	8(3)	10.1(19)
O3_4	48.5(17)	76(3)	69(4)	22(3)	-8(2)	10(2)
O4_4	41(3)	83(4)	67(2)	23(2)	2(2)	11(3)
C1_4	64(5)	71(5)	82(4)	25(3)	11(3)	8(4)
C2_4	63(6)	73(4)	87(4)	22(3)	8(4)	6(4)
C3_4	47(5)	76(3)	84(4)	21(3)	15(4)	9(2)
C4_4	89(8)	77(4)	84(4)	25(3)	13(4)	-12(4)
C5_4	163(5)	77(4)	137(5)	21(3)	-4(4)	-11(4)
C6_4	160(6)	77(4)	137(4)	21(3)	-3(4)	-11(4)
C7_4	60(6)	73(4)	130(5)	21(3)	18(4)	5(4)
C8_4	169(5)	78(4)	137(5)	21(3)	-4(4)	-13(4)

Atom	U_{11}	U_{22}	U_{33}	U_{23}	U_{13}	U_{12}
C9_4	170(5)	232(9)	159(5)	-37(5)	-8(4)	-4(5)
C10_4	170(5)	232(9)	159(5)	-37(5)	-8(4)	-4(5)
C11_4	170(5)	232(9)	159(5)	-37(5)	-8(4)	-4(5)
C12_4	170(5)	232(9)	159(5)	-37(5)	-8(4)	-4(5)
C13_4	170(5)	232(9)	159(5)	-37(5)	-8(4)	-4(5)
C14_4	159(6)	77(4)	139(5)	21(3)	-4(4)	-11(4)
C15_4	87(8)	91(4)	136(5)	30(4)	22(5)	-10(4)
C16_4	94(6)	83(5)	140(5)	29(4)	9(5)	10(5)
C17_4	93(6)	83(5)	140(5)	29(4)	10(5)	9(5)
C18_4	62(7)	91(4)	146(7)	26(4)	-29(5)	-10(4)
C19_4	132(7)	93(5)	160(7)	28(4)	1(5)	-1(5)
C20_4	133(6)	93(5)	161(7)	28(4)	1(5)	-1(5)
C21_4	134(6)	93(5)	161(7)	28(4)	2(5)	-1(5)
C22_4	134(6)	92(5)	161(7)	29(4)	3(5)	-2(5)
C23_4	133(7)	92(5)	160(7)	29(4)	4(5)	-2(5)
C24_4	54(4)	58(5)	71(3)	22(3)	11(3)	19(3)
C25_4	82(6)	74(6)	69(3)	23(4)	11(3)	-1(5)
C26_4	89(6)	124(8)	74(4)	43(4)	19(4)	29(5)
C27_4	123(7)	169(9)	96(5)	29(5)	37(4)	-9(7)
C28_4	80(5)	106(8)	93(4)	46(4)	25(4)	35(5)
C29_4	123(9)	241(8)	101(5)	0(6)	7(6)	10(7)
C30_4	200(13)	246(8)	136(10)	1(6)	-30(8)	-8(7)
C31_4	201(13)	246(8)	138(10)	0(6)	-31(8)	-7(7)
C32_4	171(14)	253(9)	126(11)	-1(7)	-9(10)	3(8)
C33_4	219(13)	254(9)	134(8)	11(8)	-51(8)	-4(8)
C34_4	219(13)	254(9)	134(8)	11(7)	-52(8)	-3(8)
C35_4	88(6)	118(9)	119(7)	28(6)	45(4)	27(5)
C36_4	110(8)	106(10)	127(6)	38(6)	59(5)	48(6)
C37_4	157(8)	108(10)	120(6)	47(6)	62(5)	20(7)
C38_4	146(8)	81(9)	112(6)	52(6)	57(5)	36(6)
C39_4	172(8)	154(14)	125(6)	55(8)	71(5)	34(9)
C40_4	183(8)	330(20)	172(10)	26(12)	86(6)	-11(10)
C41_4	183(8)	330(20)	172(10)	25(12)	86(6)	-11(10)
C42_4	196(9)	202(17)	188(10)	22(12)	65(7)	39(11)
C43_4	214(9)	420(20)	140(7)	11(12)	65(7)	-23(12)
C44_4	214(9)	420(20)	140(7)	11(12)	65(7)	-23(12)

Table 10.1.4: Bond Lengths in Å for S-**6a** (ZC-01-51).

Atom	Atom	Length/Å
Rh1	Rh2	2.4880(10)
Rh1	O2_5	2.095(5)
Rh1	O1S_7	2.334(7)
Rh1	O2_13	2.022(4)
Rh1	O2_14	2.009(5)
Rh1	O2_4	2.071(5)
Rh2	O1_5	2.038(4)
Rh2	O1S_11	2.237(7)
Rh2	O1_13	2.106(5)
Rh2	O1_14	2.090(4)
Rh2	O1_4	2.024(5)
Rh3	Rh4	2.4775(10)
Rh3	O2_1	2.033(5)
Rh3	O2_3	2.081(5)
Rh3	O1S_8	2.312(7)
Rh3	O2_9	2.043(5)
Rh3	O2_10	2.043(5)
Rh4	O1_1	2.057(5)
Rh4	O1_3	2.042(4)
Rh4	O1_9	2.066(5)
Rh4	O1_10	2.016(5)
Rh4	O1S_12	2.265(6)
Rh5	Rh5^{1}	2.4981(15)
Rh5	O	2.232(9)
Rh5	O1_2^{1}	2.016(6)
Rh5	O2_2	1.963(6)

Atom	Atom	Length/Å
Rh5	O1_6[1]	1.953(6)
Rh5	O2_6	2.031(6)
O	HA	0.9990(17)
O	HB	0.9990(15)
P1_1	O1_1	1.499(3)
P1_1	O2_1	1.496(3)
P1_1	O3_1	1.586(3)
P1_1	O4_1	1.591(3)
O3_1	C3_1	1.405(4)
O4_1	C24_1	1.408(4)
C1_1	C2_1	1.500(5)
C1_1	C24_1	1.333(5)
C1_1	C28_1	1.442(5)
C2_1	C3_1	1.345(5)
C2_1	C7_1	1.423(4)
C3_1	C4_1	1.4130(17)
C4_1	H4_1	0.9500
C4_1	C5_1	1.394(4)
C5_1	C6_1	1.410(5)
C5_1	C8_1	1.519(5)
C6_1	C7_1	1.4461(17)
C6_1	C14_1	1.404(5)
C7_1	C17_1	1.382(5)
C8_1	C9_1	1.423(5)
C8_1	C13_1	1.370(5)
C9_1	H9_1	0.9500
C9_1	C10_1	1.431(6)
C10_1	H10_1	0.9500
C10_1	C11_1	1.378(7)
C11_1	H11_1	0.9500
C11_1	C12_1	1.316(6)
C12_1	H12_1	0.9500
C12_1	C13_1	1.447(6)
C13_1	H13_1	0.9500
C14_1	H14_1	0.9500
C14_1	C15_1	1.375(3)
C15_1	C16_1	1.375(3)
C15_1	C18_1	1.509(5)
C16_1	H16_1	0.9500
C16_1	C17_1	1.349(5)
C17_1	H17_1	0.9500
C18_1	C19_1	1.410(3)
C18_1	C23_1	1.415(3)
C19_1	H19_1	0.9500
C19_1	C20_1	1.405(3)
C20_1	H20_1	0.9500
C20_1	C21_1	1.381(3)
C21_1	H21_1	0.9500
C21_1	C22_1	1.385(3)
C22_1	H22_1	0.9500
C22_1	C23_1	1.402(3)
C23_1	H23_1	0.9500
C24_1	C25_1	1.4117(17)
C25_1	H25_1	0.9500
C25_1	C26_1	1.361(5)
C26_1	C27_1	1.407(5)

Atom	Atom	Length/Å
C26_1	C29_1	1.482(5)
C27_1	C28_1	1.4470(17)
C27_1	C38_1	1.419(5)
C28_1	C35_1	1.437(5)
C29_1	C30_1	1.4000(19)
C29_1	C34_1	1.3997(19)
C30_1	H30_1	0.9500
C30_1	C31_1	1.4007(19)
C31_1	H31_1	0.9500
C31_1	C32_1	1.3999(19)
C32_1	H32_1	0.9500
C32_1	C33_1	1.3996(19)
C33_1	H33_1	0.9500
C33_1	C34_1	1.3998(19)
C34_1	H34_1	0.9500
C35_1	H35_1	0.9500
C35_1	C36_1	1.337(5)
C36_1	H36_1	0.9500
C36_1	C37_1	1.375(3)
C37_1	C38_1	1.375(3)
C37_1	C39_1	1.484(5)
C38_1	H38_1	0.9500
C39_1	C40_1	1.402(5)
C39_1	C44_1	1.421(5)
C40_1	H40_1	0.9500
C40_1	C41_1	1.428(5)
C41_1	H41_1	0.9500
C41_1	C42_1	1.380(6)
C42_1	H42_1	0.9500
C42_1	C43_1	1.362(6)
C43_1	H43_1	0.9500
C43_1	C44_1	1.392(6)
C44_1	H44_1	0.9500
P1_2	O1_2	1.500(3)
P1_2	O2_2	1.496(3)
P1_2	O3_2	1.586(3)
P1_2	O4_2	1.592(3)
O3_2	C3_2	1.406(4)
O4_2	C24_2	1.407(4)
C1_2	C2_2	1.501(5)
C1_2	C24_2	1.333(5)
C1_2	C28_2	1.442(5)
C2_2	C3_2	1.346(5)
C2_2	C7_2	1.424(4)
C3_2	C4_2	1.4121(17)
C4_2	H4_2	0.9500
C4_2	C5_2	1.394(4)
C5_2	C6_2	1.411(5)
C5_2	C8_2	1.519(5)
C6_2	C7_2	1.4475(17)
C6_2	C14_2	1.405(5)
C7_2	C17_2	1.381(5)
C8_2	C9_2	1.424(5)
C8_2	C13_2	1.370(5)
C9_2	H9_2	0.9500
C9_2	C10_2	1.430(6)

Atom	Atom	Length/Å
C10_2	H10_2	0.9500
C10_2	C11_2	1.378(7)
C11_2	H11_2	0.9500
C11_2	C12_2	1.316(6)
C12_2	H12_2	0.9500
C12_2	C13_2	1.447(6)
C13_2	H13_2	0.9500
C14_2	H14_2	0.9500
C14_2	C15_2	1.375(3)
C15_2	C16_2	1.376(3)
C15_2	C18_2	1.511(5)
C16_2	H16_2	0.9500
C16_2	C17_2	1.349(5)
C17_2	H17_2	0.9500
C18_2	C19_2	1.409(3)
C18_2	C23_2	1.415(3)
C19_2	H19_2	0.9500
C19_2	C20_2	1.405(3)
C20_2	H20_2	0.9500
C20_2	C21_2	1.380(3)
C21_2	H21_2	0.9500
C21_2	C22_2	1.385(3)
C22_2	H22_2	0.9500
C22_2	C23_2	1.402(3)
C23_2	H23_2	0.9500
C24_2	C25_2	1.4112(17)
C25_2	H25_2	0.9500
C25_2	C26_2	1.361(5)
C26_2	C27_2	1.408(5)
C26_2	C29_2	1.481(5)
C27_2	C28_2	1.4472(17)
C27_2	C38_2	1.420(5)
C28_2	C35_2	1.436(5)
C29_2	C30_2	1.4011(19)
C29_2	C34_2	1.4008(19)
C30_2	H30_2	0.9500
C30_2	C31_2	1.400(2)
C31_2	H31_2	0.9500
C31_2	C32_2	1.4003(19)
C32_2	H32_2	0.9500
C32_2	C33_2	1.4011(19)
C33_2	H33_2	0.9500
C33_2	C34_2	1.401(2)
C34_2	H34_2	0.9500
C35_2	H35_2	0.9500
C35_2	C36_2	1.336(5)
C36_2	H36_2	0.9500
C36_2	C37_2	1.375(3)
C37_2	C38_2	1.374(3)
C37_2	C39_2	1.484(5)
C38_2	H38_2	0.9500
C39_2	C40_2	1.402(5)
C39_2	C44_2	1.422(5)
C40_2	H40_2	1.0780
C40_2	C41_2	1.428(5)
C41_2	H41_2	1.0780

Atom	Atom	Length/Å
C41_2	C42_2	1.381(6)
C42_2	H42_2	1.0780
C42_2	C43_2	1.362(6)
C43_2	H43_2	1.0780
C43_2	C44_2	1.392(6)
C44_2	H44_2	1.0780
P1_3	O1_3	1.501(3)
P1_3	O2_3	1.495(3)
P1_3	O3_3	1.586(3)
P1_3	O4_3	1.591(3)
O3_3	C3_3	1.407(4)
O4_3	C24_3	1.408(4)
C1_3	C2_3	1.501(5)
C1_3	C24_3	1.333(5)
C1_3	C28_3	1.444(5)
C2_3	C3_3	1.347(5)
C2_3	C7_3	1.423(4)
C3_3	C4_3	1.4126(17)
C4_3	H4_3	0.9500
C4_3	C5_3	1.394(4)
C5_3	C6_3	1.409(5)
C5_3	C8_3	1.518(5)
C6_3	C7_3	1.4468(17)
C6_3	C14_3	1.404(5)
C7_3	C17_3	1.381(5)
C8_3	C9_3	1.424(5)
C8_3	C13_3	1.370(5)
C9_3	H9_3	0.9500
C9_3	C10_3	1.430(6)
C10_3	H10_3	0.9500
C10_3	C11_3	1.378(7)
C11_3	H11_3	0.9500
C11_3	C12_3	1.316(6)
C12_3	H12_3	0.9500
C12_3	C13_3	1.447(6)
C13_3	H13_3	0.9500
C14_3	H14_3	0.9500
C14_3	C15_3	1.374(3)
C15_3	C16_3	1.374(3)
C15_3	C18_3	1.509(5)
C16_3	H16_3	0.9500
C16_3	C17_3	1.349(5)
C17_3	H17_3	0.9500
C18_3	C19_3	1.410(3)
C18_3	C23_3	1.415(3)
C19_3	H19_3	0.9500
C19_3	C20_3	1.405(3)
C20_3	H20_3	0.9500
C20_3	C21_3	1.381(3)
C21_3	H21_3	0.9500
C21_3	C22_3	1.385(3)
C22_3	H22_3	0.9500
C22_3	C23_3	1.402(3)
C23_3	H23_3	0.9500
C24_3	C25_3	1.4116(17)
C25_3	H25_3	0.9500

Atom	Atom	Length/Å
C25_3	C26_3	1.362(5)
C26_3	C27_3	1.407(5)
C26_3	C29_3	1.483(5)
C27_3	C28_3	1.4469(17)
C27_3	C38_3	1.420(5)
C28_3	C35_3	1.438(5)
C29_3	C30_3	1.3996(19)
C29_3	C34_3	1.4007(19)
C30_3	H30_3	0.9500
C30_3	C31_3	1.400(2)
C31_3	H31_3	0.9500
C31_3	C32_3	1.4006(19)
C32_3	H32_3	0.9500
C32_3	C33_3	1.3999(19)
C33_3	H33_3	0.9500
C33_3	C34_3	1.401(2)
C34_3	H34_3	0.9500
C35_3	H35_3	0.9500
C35_3	C36_3	1.337(5)
C36_3	H36_3	0.9500
C36_3	C37_3	1.376(3)
C37_3	C38_3	1.375(3)
C37_3	C39_3	1.484(5)
C38_3	H38_3	0.9500
C39_3	C40_3	1.402(5)
C39_3	C44_3	1.421(5)
C40_3	H40_3	0.9500
C40_3	C41_3	1.428(5)
C41_3	H41_3	0.9500
C41_3	C42_3	1.380(6)
C42_3	H42_3	0.9500
C42_3	C43_3	1.361(6)
C43_3	H43_3	0.9500
C43_3	C44_3	1.392(6)
C44_3	H44_3	0.9500
P1_5	O1_5	1.503(3)
P1_5	O2_5	1.494(3)
P1_5	O3_5	1.585(3)
P1_5	O4_5	1.590(3)
O3_5	C3_5	1.406(4)
O4_5	C24_5	1.405(4)
C1_5	C2_5	1.501(5)
C1_5	C24_5	1.334(5)
C1_5	C28_5	1.445(5)
C2_5	C3_5	1.347(5)
C2_5	C7_5	1.424(4)
C3_5	C4_5	1.4121(17)
C4_5	H4_5	0.9500
C4_5	C5_5	1.393(4)
C5_5	C6_5	1.409(5)
C5_5	C8_5	1.519(5)
C6_5	C7_5	1.4472(17)
C6_5	C14_5	1.404(5)
C7_5	C17_5	1.382(5)
C8_5	C9_5	1.424(5)
C8_5	C13_5	1.369(5)
C9_5	H9_5	0.9500
C9_5	C10_5	1.430(6)
C10_5	H10_5	0.9500
C10_5	C11_5	1.378(7)
C11_5	H11_5	0.9500
C11_5	C12_5	1.316(6)
C12_5	H12_5	0.9500
C12_5	C13_5	1.448(6)
C13_5	H13_5	0.9500
C14_5	H14_5	0.9500
C14_5	C15_5	1.375(3)
C15_5	C16_5	1.374(3)
C15_5	C18_5	1.509(5)
C16_5	H16_5	0.9500
C16_5	C17_5	1.349(5)
C17_5	H17_5	0.9500
C18_5	C19_5	1.407(2)
C18_5	C23_5	1.410(2)
C19_5	H19_5	0.9500
C19_5	C20_5	1.402(2)
C20_5	H20_5	0.9500
C20_5	C21_5	1.385(3)
C21_5	H21_5	0.9500
C21_5	C22_5	1.388(3)
C22_5	H22_5	0.9500
C22_5	C23_5	1.400(2)
C23_5	H23_5	0.9500
C24_5	C25_5	1.4125(17)
C25_5	H25_5	0.9500
C25_5	C26_5	1.361(5)
C26_5	C27_5	1.406(5)
C26_5	C29_5	1.483(5)
C27_5	C28_5	1.4473(17)
C27_5	C38_5	1.418(5)
C28_5	C35_5	1.438(5)
C29_5	C30_5	1.3990(16)
C29_5	C34_5	1.3993(16)
C30_5	H30_5	0.9500
C30_5	C31_5	1.3991(16)
C31_5	H31_5	0.9500
C31_5	C32_5	1.3988(16)
C32_5	H32_5	0.9500
C32_5	C33_5	1.3986(16)
C33_5	H33_5	0.9500
C33_5	C34_5	1.3987(16)
C34_5	H34_5	0.9500
C35_5	H35_5	0.9500
C35_5	C36_5	1.337(5)
C36_5	H36_5	0.9500
C36_5	C37_5	1.375(3)
C37_5	C38_5	1.374(3)
C37_5	C39_5	1.484(5)
C38_5	H38_5	0.9500
C39_5	C40_5	1.402(5)
C39_5	C44_5	1.422(5)
C40_5	H40_5	0.9500

Atom	Atom	Length/Å
C40_5	C41_5	1.427(5)
C41_5	H41_5	0.9500
C41_5	C42_5	1.381(6)
C42_5	H42_5	0.9500
C42_5	C43_5	1.361(6)
C43_5	H43_5	0.9500
C43_5	C44_5	1.392(6)
C44_5	H44_5	0.9500
P1_6	O1_6	1.500(3)
P1_6	O2_6	1.497(3)
P1_6	O3_6	1.584(3)
P1_6	O4_6	1.591(3)
O3_6	C3_6	1.405(4)
O4_6	C24_6	1.408(4)
C1_6	C2_6	1.500(5)
C1_6	C24_6	1.334(5)
C1_6	C28_6	1.442(5)
C2_6	C3_6	1.346(5)
C2_6	C7_6	1.424(4)
C3_6	C4_6	1.4130(17)
C4_6	H4_6	0.9500
C4_6	C5_6	1.394(4)
C5_6	C6_6	1.410(4)
C5_6	C8_6	1.518(5)
C6_6	C7_6	1.4468(17)
C6_6	C14_6	1.405(5)
C7_6	C17_6	1.382(5)
C8_6	C9_6	1.424(5)
C8_6	C13_6	1.369(5)
C9_6	H9_6	0.9500
C9_6	C10_6	1.430(6)
C10_6	H10_6	0.9500
C10_6	C11_6	1.378(7)
C11_6	H11_6	0.9500
C11_6	C12_6	1.317(6)
C12_6	H12_6	0.9500
C12_6	C13_6	1.446(6)
C13_6	H13_6	0.9500
C14_6	H14_6	0.9500
C14_6	C15_6	1.374(3)
C15_6	C16_6	1.374(3)
C15_6	C18_6	1.508(5)
C16_6	H16_6	0.9500
C16_6	C17_6	1.349(5)
C17_6	H17_6	0.9500
C18_6	C19_6	1.409(3)
C18_6	C23_6	1.414(3)
C19_6	H19_6	0.9500
C19_6	C20_6	1.405(3)
C20_6	H20_6	0.9500
C20_6	C21_6	1.381(3)
C21_6	H21_6	0.9500
C21_6	C22_6	1.386(3)
C22_6	H22_6	0.9500
C22_6	C23_6	1.401(3)
C23_6	H23_6	0.9500

Atom	Atom	Length/Å
C24_6	C25_6	1.4114(17)
C25_6	H25_6	0.9500
C25_6	C26_6	1.361(5)
C26_6	C27_6	1.407(5)
C26_6	C29_6	1.481(5)
C27_6	C28_6	1.4468(17)
C27_6	C38_6	1.418(5)
C28_6	C35_6	1.437(5)
C29_6	C30_6	1.4002(19)
C29_6	C34_6	1.4003(19)
C30_6	H30_6	0.9500
C30_6	C31_6	1.400(2)
C31_6	H31_6	0.9500
C31_6	C32_6	1.4001(19)
C32_6	H32_6	0.9500
C32_6	C33_6	1.4001(19)
C33_6	H33_6	0.9500
C33_6	C34_6	1.3997(19)
C34_6	H34_6	0.9500
C35_6	H35_6	0.9500
C35_6	C36_6	1.337(5)
C36_6	H36_6	0.9500
C36_6	C37_6	1.374(3)
C37_6	C38_6	1.375(3)
C37_6	C39_6	1.484(5)
C38_6	H38_6	0.9500
C39_6	C40_6	1.401(5)
C39_6	C44_6	1.422(5)
C40_6	H40_6	0.9500
C40_6	C41_6	1.428(5)
C41_6	H41_6	0.9500
C41_6	C42_6	1.380(6)
C42_6	H42_6	0.9500
C42_6	C43_6	1.361(6)
C43_6	H43_6	0.9500
C43_6	C44_6	1.392(6)
C44_6	H44_6	0.9500
O1S_7	C2S_7	1.410(8)
O1S_7	C4S_7	1.467(8)
C2S_7	H2SA_7	0.9900
C2S_7	H2SB_7	0.9900
C2S_7	C3S_7	1.519(4)
C3S_7	H3SA_7	0.9800
C3S_7	H3SB_7	0.9800
C3S_7	H3SC_7	0.9800
C4S_7	H4SA_7	0.9900
C4S_7	H4SB_7	0.9900
C4S_7	C5S_7	1.519(4)
C5S_7	H5SA_7	0.9800
C5S_7	H5SB_7	0.9800
C5S_7	H5SC_7	0.9800
O1S_8	C2S_8	1.410(8)
O1S_8	C4S_8	1.468(8)
C2S_8	H2SA_8	0.9900
C2S_8	H2SB_8	0.9900
C2S_8	C3S_8	1.520(4)

Atom	Atom	Length/Å
C3S_8	H3SA_8	0.9800
C3S_8	H3SB_8	0.9800
C3S_8	H3SC_8	0.9800
C4S_8	H4SA_8	0.9900
C4S_8	H4SB_8	0.9900
C4S_8	C5S_8	1.519(4)
C5S_8	H5SA_8	0.9800
C5S_8	H5SB_8	0.9800
C5S_8	H5SC_8	0.9800
P1_9	O1_9	1.503(3)
P1_9	O2_9	1.494(3)
P1_9	O3_9	1.587(3)
P1_9	O4_9	1.592(3)
O3_9	C3_9	1.406(4)
O4_9	C24_9	1.409(4)
C1_9	C2_9	1.501(5)
C1_9	C24_9	1.334(5)
C1_9	C28_9	1.442(5)
C2_9	C3_9	1.346(5)
C2_9	C7_9	1.423(4)
C3_9	C4_9	1.4122(17)
C4_9	H4_9	0.9500
C4_9	C5_9	1.395(4)
C5_9	C6_9	1.410(5)
C5_9	C8_9	1.519(5)
C6_9	C7_9	1.4467(17)
C6_9	C14_9	1.405(5)
C7_9	C17_9	1.381(5)
C8_9	C9_9	1.424(5)
C8_9	C13_9	1.370(5)
C9_9	H9_9	0.9500
C9_9	C10_9	1.430(6)
C10_9	H10_9	0.9500
C10_9	C11_9	1.378(7)
C11_9	H11_9	0.9500
C11_9	C12_9	1.316(6)
C12_9	H12_9	0.9500
C12_9	C13_9	1.447(6)
C13_9	H13_9	0.9500
C14_9	H14_9	0.9500
C14_9	C15_9	1.374(3)
C15_9	C16_9	1.375(3)
C15_9	C18_9	1.509(5)
C16_9	H16_9	0.9500
C16_9	C17_9	1.348(5)
C17_9	H17_9	0.9500
C18_9	C19_9	1.410(3)
C18_9	C23_9	1.415(3)
C19_9	H19_9	0.9500
C19_9	C20_9	1.405(3)
C20_9	H20_9	0.9500
C20_9	C21_9	1.381(3)
C21_9	H21_9	0.9500
C21_9	C22_9	1.385(3)
C22_9	H22_9	0.9500
C22_9	C23_9	1.401(3)
C23_9	H23_9	0.9500
C24_9	C25_9	1.4114(17)
C25_9	H25_9	0.9500
C25_9	C26_9	1.362(5)
C26_9	C27_9	1.407(5)
C26_9	C29_9	1.482(5)
C27_9	C28_9	1.4475(17)
C27_9	C38_9	1.420(5)
C28_9	C35_9	1.437(5)
C29_9	C30_9	1.4007(19)
C29_9	C34_9	1.4008(19)
C30_9	H30_9	0.9500
C30_9	C31_9	1.4005(19)
C31_9	H31_9	0.9500
C31_9	C32_9	1.4000(19)
C32_9	H32_9	0.9500
C32_9	C33_9	1.4003(19)
C33_9	H33_9	0.9500
C33_9	C34_9	1.3998(19)
C34_9	H34_9	0.9500
C35_9	H35_9	0.9500
C35_9	C36_9	1.337(5)
C36_9	H36_9	0.9500
C36_9	C37_9	1.375(3)
C37_9	C38_9	1.375(3)
C37_9	C39_9	1.485(5)
C38_9	H38_9	0.9500
C39_9	C40_9	1.402(5)
C39_9	C44_9	1.421(5)
C40_9	H40_9	0.9500
C40_9	C41_9	1.427(5)
C41_9	H41_9	0.9500
C41_9	C42_9	1.380(6)
C42_9	H42_9	0.9500
C42_9	C43_9	1.362(6)
C43_9	H43_9	0.9500
C43_9	C44_9	1.392(6)
C44_9	H44_9	0.9500
P1_10	O1_10	1.503(3)
P1_10	O2_10	1.493(3)
P1_10	O3_10	1.585(3)
P1_10	O4_10	1.590(3)
O3_10	C3_10	1.405(4)
O4_10	C24_10	1.405(4)
C1_10	C2_10	1.501(5)
C1_10	C24_10	1.333(5)
C1_10	C28_10	1.444(5)
C2_10	C3_10	1.347(5)
C2_10	C7_10	1.423(4)
C3_10	C4_10	1.4115(17)
C4_10	H4_10	0.9500
C4_10	C5_10	1.394(4)
C5_10	C6_10	1.410(5)
C5_10	C8_10	1.519(5)
C6_10	C7_10	1.4477(17)
C6_10	C14_10	1.405(5)

Atom	Atom	Length/Å
C7_10	C17_10	1.381(5)
C8_10	C9_10	1.424(5)
C8_10	C13_10	1.370(5)
C9_10	H9_10	0.9500
C9_10	C10_10	1.430(6)
C10_10	H10_10	0.9500
C10_10	C11_10	1.378(7)
C11_10	H11_10	0.9500
C11_10	C12_10	1.317(6)
C12_10	H12_10	0.9500
C12_10	C13_10	1.446(6)
C13_10	H13_10	0.9500
C14_10	H14_10	0.9500
C14_10	C15_10	1.375(3)
C15_10	C16_10	1.375(3)
C15_10	C18_10	1.509(5)
C16_10	H16_10	0.9500
C16_10	C17_10	1.349(5)
C17_10	H17_10	0.9500
C18_10	C19_10	1.409(3)
C18_10	C23_10	1.415(3)
C19_10	H19_10	0.9500
C19_10	C20_10	1.404(3)
C20_10	H20_10	0.9500
C20_10	C21_10	1.382(3)
C21_10	H21_10	0.9500
C21_10	C22_10	1.385(3)
C22_10	H22_10	0.9500
C22_10	C23_10	1.402(3)
C23_10	H23_10	0.9500
C24_10	C25_10	1.4128(17)
C25_10	H25_10	0.9500
C25_10	C26_10	1.362(5)
C26_10	C27_10	1.406(5)
C26_10	C29_10	1.482(5)
C27_10	C28_10	1.4473(17)
C27_10	C38_10	1.420(5)
C28_10	C35_10	1.437(5)
C29_10	C30_10	1.4009(19)
C29_10	C34_10	1.4010(19)
C30_10	H30_10	0.9500
C30_10	C31_10	1.4007(19)
C31_10	H31_10	0.9500
C31_10	C32_10	1.4003(19)
C32_10	H32_10	0.9500
C32_10	C33_10	1.4001(19)
C33_10	H33_10	0.9500
C33_10	C34_10	1.4001(19)
C34_10	H34_10	0.9500
C35_10	H35_10	0.9500
C35_10	C36_10	1.337(5)
C36_10	H36_10	0.9500
C36_10	C37_10	1.375(3)
C37_10	C38_10	1.375(3)
C37_10	C39_10	1.483(5)
C38_10	H38_10	0.9500

Atom	Atom	Length/Å
C39_10	C40_10	1.402(5)
C39_10	C44_10	1.422(5)
C40_10	H40_10	0.9500
C40_10	C41_10	1.427(5)
C41_10	H41_10	0.9500
C41_10	C42_10	1.380(6)
C42_10	H42_10	0.9500
C42_10	C43_10	1.362(6)
C43_10	H43_10	0.9500
C43_10	C44_10	1.391(6)
C44_10	H44_10	0.9500
O1S_11	C2S_11	1.411(8)
O1S_11	C4S_11	1.467(8)
C2S_11	H2SA_11	0.9900
C2S_11	H2SB_11	0.9900
C2S_11	C3S_11	1.521(3)
C3S_11	H3SA_11	0.9800
C3S_11	H3SB_11	0.9800
C3S_11	H3SC_11	0.9800
C4S_11	H4SA_11	0.9900
C4S_11	H4SB_11	0.9900
C4S_11	C5S_11	1.520(3)
C5S_11	H5SA_11	0.9800
C5S_11	H5SB_11	0.9800
C5S_11	H5SC_11	0.9800
O1S_12	C2S_12	1.410(8)
O1S_12	C4S_12	1.467(8)
C2S_12	H2SA_12	0.9900
C2S_12	H2SB_12	0.9900
C2S_12	C3S_12	1.520(4)
C3S_12	H3SA_12	0.9800
C3S_12	H3SB_12	0.9800
C3S_12	H3SC_12	0.9800
C4S_12	H4SA_12	0.9900
C4S_12	H4SB_12	0.9900
C4S_12	C5S_12	1.521(4)
C5S_12	H5SA_12	0.9800
C5S_12	H5SB_12	0.9800
C5S_12	H5SC_12	0.9800
P1_13	O1_13	1.498(3)
P1_13	O2_13	1.497(3)
P1_13	O3_13	1.586(3)
P1_13	O4_13	1.593(3)
O3_13	C3_13	1.405(4)
O4_13	C24_13	1.410(4)
C1_13	C2_13	1.501(5)
C1_13	C24_13	1.334(5)
C1_13	C28_13	1.443(5)
C2_13	C3_13	1.347(5)
C2_13	C7_13	1.425(4)
C3_13	C4_13	1.4130(17)
C4_13	H4_13	0.9500
C4_13	C5_13	1.394(4)
C5_13	C6_13	1.409(5)
C5_13	C8_13	1.519(5)
C6_13	C7_13	1.4468(17)

Atom	Atom	Length/Å
C6_13	C14_13	1.405(5)
C7_13	C17_13	1.382(5)
C8_13	C9_13	1.424(5)
C8_13	C13_13	1.370(5)
C9_13	H9_13	0.9500
C9_13	C10_13	1.430(6)
C10_13	H10_13	0.9500
C10_13	C11_13	1.378(7)
C11_13	H11_13	0.9500
C11_13	C12_13	1.316(6)
C12_13	H12_13	0.9500
C12_13	C13_13	1.446(6)
C13_13	H13_13	0.9500
C14_13	H14_13	0.9500
C14_13	C15_13	1.374(3)
C15_13	C16_13	1.375(3)
C15_13	C18_13	1.509(5)
C16_13	H16_13	0.9500
C16_13	C17_13	1.348(5)
C17_13	H17_13	0.9500
C18_13	C19_13	1.410(3)
C18_13	C23_13	1.415(3)
C19_13	H19_13	0.9500
C19_13	C20_13	1.405(3)
C20_13	H20_13	0.9500
C20_13	C21_13	1.380(3)
C21_13	H21_13	0.9500
C21_13	C22_13	1.384(3)
C22_13	H22_13	0.9500
C22_13	C23_13	1.402(3)
C23_13	H23_13	0.9500
C24_13	C25_13	1.4117(17)
C25_13	H25_13	0.9500
C25_13	C26_13	1.361(5)
C26_13	C27_13	1.406(5)
C26_13	C29_13	1.483(5)
C27_13	C28_13	1.4475(17)
C27_13	C38_13	1.419(5)
C28_13	C35_13	1.437(5)
C29_13	C30_13	1.4005(19)
C29_13	C34_13	1.4013(19)
C30_13	H30_13	0.9500
C30_13	C31_13	1.4002(19)
C31_13	H31_13	0.9500
C31_13	C32_13	1.3995(19)
C32_13	H32_13	0.9500
C32_13	C33_13	1.4004(19)
C33_13	H33_13	0.9500
C33_13	C34_13	1.4003(19)
C34_13	H34_13	0.9500
C35_13	H35_13	0.9500
C35_13	C36_13	1.337(5)
C36_13	H36_13	0.9500
C36_13	C37_13	1.375(3)
C37_13	C38_13	1.374(3)
C37_13	C39_13	1.485(5)

Atom	Atom	Length/Å
C38_13	H38_13	0.9500
C39_13	C40_13	1.402(5)
C39_13	C44_13	1.421(5)
C40_13	H40_13	0.9500
C40_13	C41_13	1.428(5)
C41_13	H41_13	0.9500
C41_13	C42_13	1.380(6)
C42_13	H42_13	0.9500
C42_13	C43_13	1.362(6)
C43_13	H43_13	0.9500
C43_13	C44_13	1.391(6)
C44_13	H44_13	0.9500
P1_14	O1_14	1.499(3)
P1_14	O2_14	1.499(3)
P1_14	O3_14	1.583(3)
P1_14	O4_14	1.591(3)
O3_14	C3_14	1.404(4)
O4_14	C24_14	1.409(4)
C1_14	C2_14	1.501(5)
C1_14	C24_14	1.334(5)
C1_14	C28_14	1.442(5)
C2_14	C3_14	1.347(5)
C2_14	C7_14	1.425(4)
C3_14	C4_14	1.4137(17)
C4_14	H4_14	0.9500
C4_14	C5_14	1.392(4)
C5_14	C6_14	1.409(5)
C5_14	C8_14	1.518(5)
C6_14	C7_14	1.4464(17)
C6_14	C14_14	1.404(5)
C7_14	C17_14	1.382(5)
C8_14	C9_14	1.422(5)
C8_14	C13_14	1.368(5)
C9_14	H9_14	0.9500
C9_14	C10_14	1.430(6)
C10_14	H10_14	0.9500
C10_14	C11_14	1.378(7)
C11_14	H11_14	0.9500
C11_14	C12_14	1.317(6)
C12_14	H12_14	0.9500
C12_14	C13_14	1.447(6)
C13_14	H13_14	0.9500
C14_14	H14_14	0.9500
C14_14	C15_14	1.375(3)
C15_14	C16_14	1.374(3)
C15_14	C18_14	1.510(5)
C16_14	H16_14	0.9500
C16_14	C17_14	1.349(5)
C17_14	H17_14	0.9500
C18_14	C19_14	1.411(3)
C18_14	C23_14	1.415(3)
C19_14	H19_14	0.9500
C19_14	C20_14	1.405(3)
C20_14	H20_14	0.9500
C20_14	C21_14	1.381(3)
C21_14	H21_14	0.9500

Atom	Atom	Length/Å
C21_14	C22_14	1.385(3)
C22_14	H22_14	0.9500
C22_14	C23_14	1.402(3)
C23_14	H23_14	0.9500
C24_14	C25_14	1.4113(17)
C25_14	H25_14	0.9500
C25_14	C26_14	1.361(5)
C26_14	C27_14	1.407(5)
C26_14	C29_14	1.481(5)
C27_14	C28_14	1.4478(17)
C27_14	C38_14	1.419(5)
C28_14	C35_14	1.437(5)
C29_14	C30_14	1.4000(19)
C29_14	C34_14	1.4005(19)
C30_14	H30_14	0.9500
C30_14	C31_14	1.4001(19)
C31_14	H31_14	0.9500
C31_14	C32_14	1.4004(19)
C32_14	H32_14	0.9500
C32_14	C33_14	1.4000(19)
C33_14	H33_14	0.9500
C33_14	C34_14	1.3999(19)
C34_14	H34_14	0.9500
C35_14	H35_14	0.9500
C35_14	C36_14	1.337(5)
C36_14	H36_14	0.9500
C36_14	C37_14	1.375(3)
C37_14	C38_14	1.374(3)
C37_14	C39_14	1.484(5)
C38_14	H38_14	0.9500
C39_14	C40_14	1.402(5)
C39_14	C44_14	1.422(5)
C40_14	H40_14	0.9500
C40_14	C41_14	1.428(5)
C41_14	H41_14	0.9500
C41_14	C42_14	1.381(6)
C42_14	H42_14	0.9500
C42_14	C43_14	1.362(6)
C43_14	H43_14	0.9500
C43_14	C44_14	1.391(6)
C44_14	H44_14	0.9500
P1_4	O1_4	1.502(3)
P1_4	O2_4	1.494(3)
P1_4	O3_4	1.589(3)
P1_4	O4_4	1.589(3)
O3_4	C3_4	1.406(4)
O4_4	C24_4	1.407(4)
C1_4	C2_4	1.501(5)
C1_4	C24_4	1.333(5)
C1_4	C28_4	1.444(5)
C2_4	C3_4	1.347(5)
C2_4	C7_4	1.423(4)
C3_4	C4_4	1.4117(17)
C4_4	H4_4	0.9500
C4_4	C5_4	1.395(4)
C5_4	C6_4	1.410(5)

Atom	Atom	Length/Å
C5_4	C8_4	1.519(5)
C6_4	C7_4	1.4467(17)
C6_4	C14_4	1.405(5)
C7_4	C17_4	1.382(5)
C8_4	C9_4	1.424(5)
C8_4	C13_4	1.369(5)
C9_4	H9_4	1.0780
C9_4	C10_4	1.430(6)
C10_4	H10_4	1.0780
C10_4	C11_4	1.378(7)
C11_4	H11_4	1.0780
C11_4	C12_4	1.316(6)
C12_4	H12_4	1.0780
C12_4	C13_4	1.447(6)
C13_4	H13_4	1.0780
C14_4	H14_4	0.9500
C14_4	C15_4	1.375(3)
C15_4	C16_4	1.374(3)
C15_4	C18_4	1.508(5)
C16_4	H16_4	0.9500
C16_4	C17_4	1.348(5)
C17_4	H17_4	0.9500
C18_4	C19_4	1.410(3)
C18_4	C23_4	1.415(3)
C19_4	H19_4	0.9500
C19_4	C20_4	1.405(3)
C20_4	H20_4	0.9500
C20_4	C21_4	1.381(3)
C21_4	H21_4	0.9500
C21_4	C22_4	1.385(3)
C22_4	H22_4	0.9500
C22_4	C23_4	1.401(3)
C23_4	H23_4	0.9500
C24_4	C25_4	1.4120(17)
C25_4	H25_4	0.9500
C25_4	C26_4	1.361(5)
C26_4	C27_4	1.408(5)
C26_4	C29_4	1.482(5)
C27_4	C28_4	1.4474(17)
C27_4	C38_4	1.420(5)
C28_4	C35_4	1.438(5)
C29_4	C30_4	1.3996(19)
C29_4	C34_4	1.4012(19)
C30_4	H30_4	0.9500
C30_4	C31_4	1.4003(19)
C31_4	H31_4	0.9500
C31_4	C32_4	1.3996(19)
C32_4	H32_4	0.9500
C32_4	C33_4	1.4000(19)
C33_4	H33_4	0.9500
C33_4	C34_4	1.3998(19)
C34_4	H34_4	0.9500
C35_4	H35_4	0.9500
C35_4	C36_4	1.336(5)
C36_4	H36_4	0.9500
C36_4	C37_4	1.375(3)

Atom	Atom	Length/Å
C37_4	C38_4	1.374(3)
C37_4	C39_4	1.485(5)
C38_4	H38_4	0.9500
C39_4	C40_4	1.402(5)
C39_4	C44_4	1.422(5)
C40_4	H40_4	0.9500
C40_4	C41_4	1.428(5)
C41_4	H41_4	0.9500
C41_4	C42_4	1.380(6)
C42_4	H42_4	0.9500
C42_4	C43_4	1.362(6)
C43_4	H43_4	0.9500
C43_4	C44_4	1.392(6)
C44_4	H44_4	0.9500

––––

[1]-1-x,+y,3/2-z

Table 10.1.5: Bond Angles in ° for S-**6a** (ZC-01-51).

Atom	Atom	Atom	Angle/°
O2_5	Rh1	Rh2	89.62(12)
O2_5	Rh1	O1S_7	89.6(2)
O1S_7	Rh1	Rh2	179.0(2)
O2_13	Rh1	Rh2	91.12(12)
O2_13	Rh1	O2_5	88.3(2)
O2_13	Rh1	O1S_7	88.2(2)
O2_13	Rh1	O2_4	90.05(19)
O2_14	Rh1	Rh2	89.67(12)
O2_14	Rh1	O2_5	91.0(2)
O2_14	Rh1	O1S_7	91.0(2)
O2_14	Rh1	O2_13	178.9(2)
O2_14	Rh1	O2_4	90.7(2)
O2_4	Rh1	Rh2	88.06(12)
O2_4	Rh1	O2_5	177.10(19)
O2_4	Rh1	O1S_7	92.7(2)
O1_5	Rh2	Rh1	89.24(12)
O1_5	Rh2	O1S_11	91.6(2)
O1_5	Rh2	O1_13	86.96(19)
O1_5	Rh2	O1_14	92.81(19)
O1S_11	Rh2	Rh1	179.0(2)
O1_13	Rh2	Rh1	88.22(11)
O1_13	Rh2	O1S_11	91.3(2)
O1_14	Rh2	Rh1	89.85(11)
O1_14	Rh2	O1S_11	90.7(2)
O1_14	Rh2	O1_13	178.07(18)
O1_4	Rh2	Rh1	89.73(12)
O1_4	Rh2	O1_5	178.42(19)
O1_4	Rh2	O1S_11	89.4(2)
O1_4	Rh2	O1_13	94.21(19)
O1_4	Rh2	O1_14	85.99(19)
O2_1	Rh3	Rh4	89.27(12)
O2_1	Rh3	O2_3	177.76(18)
O2_1	Rh3	O1S_8	92.8(2)
O2_1	Rh3	O2_9	89.41(19)
O2_1	Rh3	O2_10	86.3(2)
O2_3	Rh3	Rh4	88.48(11)
O2_3	Rh3	O1S_8	89.5(2)
O1S_8	Rh3	Rh4	177.2(2)
O2_9	Rh3	Rh4	87.87(13)
O2_9	Rh3	O2_3	90.4(2)
O2_9	Rh3	O1S_8	94.1(2)
O2_10	Rh3	Rh4	89.53(12)
O2_10	Rh3	O2_3	93.7(2)
O2_10	Rh3	O1S_8	88.7(2)
O2_10	Rh3	O2_9	175.0(2)
O1_1	Rh4	Rh3	88.68(12)
O1_1	Rh4	O1_9	93.14(19)
O1_1	Rh4	O1S_12	90.8(2)
O1_3	Rh4	Rh3	88.69(12)
O1_3	Rh4	O1_1	176.78(19)
O1_3	Rh4	O1_9	88.66(19)
O1_3	Rh4	O1S_12	91.8(2)
O1_9	Rh4	Rh3	88.56(12)
O1_9	Rh4	O1S_12	90.4(2)
O1_10	Rh4	Rh3	89.74(13)
O1_10	Rh4	O1_1	90.2(2)
O1_10	Rh4	O1_3	87.93(19)
O1_10	Rh4	O1_9	176.22(19)
O1_10	Rh4	O1S_12	91.4(2)
O1S_12	Rh4	Rh3	178.8(2)
O	Rh5	Rh5[1]	179.4(4)
O1_2[1]	Rh5	Rh5[1]	92.10(15)
O1_2[1]	Rh5	O	87.3(3)
O1_2[1]	Rh5	O2_6	176.7(2)
O2_2	Rh5	Rh5[1]	88.35(16)
O2_2	Rh5	O	91.8(3)
O2_2	Rh5	O1_2[1]	89.7(4)
O2_2	Rh5	O2_6	90.7(3)
O1_6[1]	Rh5	Rh5[1]	89.17(15)
O1_6[1]	Rh5	O	90.7(3)
O1_6[1]	Rh5	O1_2[1]	90.9(3)
O1_6[1]	Rh5	O2_2	177.5(2)
O1_6[1]	Rh5	O2_6	88.9(3)
O2_6	Rh5	Rh5[1]	91.21(14)
O2_6	Rh5	O	89.3(4)
Rh5	O	HA	115.6(16)
Rh5	O	HB	115.6(16)
HA	O	HB	123.4(4)
O1_1	P1_1	O3_1	112.4(2)
O1_1	P1_1	O4_1	105.5(2)
O2_1	P1_1	O1_1	118.6(2)
O2_1	P1_1	O3_1	105.6(2)
O2_1	P1_1	O4_1	110.6(2)
O3_1	P1_1	O4_1	103.1(2)

Atom	Atom	Atom	Angle/°
P1_1	O1_1	Rh4	114.8(3)
P1_1	O2_1	Rh3	117.2(3)
C3_1	O3_1	P1_1	116.8(3)
C24_1	O4_1	P1_1	118.0(3)
C24_1	C1_1	C2_1	118.8(4)
C24_1	C1_1	C28_1	118.0(4)
C28_1	C1_1	C2_1	123.2(4)
C3_1	C2_1	C1_1	118.2(4)
C3_1	C2_1	C7_1	118.4(4)
C7_1	C2_1	C1_1	123.2(4)
O3_1	C3_1	C4_1	115.1(3)
C2_1	C3_1	O3_1	120.2(3)
C2_1	C3_1	C4_1	124.7(4)
C3_1	C4_1	H4_1	121.2
C5_1	C4_1	C3_1	117.6(4)
C5_1	C4_1	H4_1	121.2
C4_1	C5_1	C6_1	120.8(4)
C4_1	C5_1	C8_1	117.1(4)
C6_1	C5_1	C8_1	121.5(4)
C5_1	C6_1	C7_1	119.2(4)
C14_1	C6_1	C5_1	122.4(4)
C14_1	C6_1	C7_1	118.4(4)
C2_1	C7_1	C6_1	119.2(4)
C17_1	C7_1	C2_1	124.7(4)
C17_1	C7_1	C6_1	116.1(4)
C9_1	C8_1	C5_1	119.4(4)
C13_1	C8_1	C5_1	119.9(4)
C13_1	C8_1	C9_1	120.6(4)
C8_1	C9_1	H9_1	120.8
C8_1	C9_1	C10_1	118.5(4)
C10_1	C9_1	H9_1	120.8
C9_1	C10_1	H10_1	122.0
C11_1	C10_1	C9_1	116.1(4)
C11_1	C10_1	H10_1	122.0
C10_1	C11_1	H11_1	115.7
C12_1	C11_1	C10_1	128.5(5)
C12_1	C11_1	H11_1	115.7
C11_1	C12_1	H12_1	122.3
C11_1	C12_1	C13_1	115.3(4)
C13_1	C12_1	H12_1	122.3
C8_1	C13_1	C12_1	121.0(4)
C8_1	C13_1	H13_1	119.5
C12_1	C13_1	H13_1	119.5
C6_1	C14_1	H14_1	119.2
C15_1	C14_1	C6_1	121.6(4)
C15_1	C14_1	H14_1	119.2
C14_1	C15_1	C18_1	120.2(4)
C16_1	C15_1	C14_1	119.5(4)
C16_1	C15_1	C18_1	120.0(4)
C15_1	C16_1	H16_1	120.1
C17_1	C16_1	C15_1	119.7(5)
C17_1	C16_1	H16_1	120.1
C7_1	C17_1	H17_1	117.7
C16_1	C17_1	C7_1	124.5(5)
C16_1	C17_1	H17_1	117.7
C19_1	C18_1	C15_1	121.9(4)

Atom	Atom	Atom	Angle/°
C19_1	C18_1	C23_1	118.1(2)
C23_1	C18_1	C15_1	119.9(4)
C18_1	C19_1	H19_1	119.4
C20_1	C19_1	C18_1	121.1(3)
C20_1	C19_1	H19_1	119.4
C19_1	C20_1	H20_1	120.8
C21_1	C20_1	C19_1	118.4(3)
C21_1	C20_1	H20_1	120.8
C20_1	C21_1	H21_1	118.6
C20_1	C21_1	C22_1	122.7(4)
C22_1	C21_1	H21_1	118.6
C21_1	C22_1	H22_1	120.8
C21_1	C22_1	C23_1	118.4(3)
C23_1	C22_1	H22_1	120.8
C18_1	C23_1	H23_1	119.6
C22_1	C23_1	C18_1	120.8(3)
C22_1	C23_1	H23_1	119.6
O4_1	C24_1	C25_1	115.4(4)
C1_1	C24_1	O4_1	119.9(4)
C1_1	C24_1	C25_1	124.1(4)
C24_1	C25_1	H25_1	120.5
C26_1	C25_1	C24_1	119.0(4)
C26_1	C25_1	H25_1	120.5
C25_1	C26_1	C27_1	121.1(4)
C25_1	C26_1	C29_1	117.4(4)
C27_1	C26_1	C29_1	120.8(4)
C26_1	C27_1	C28_1	118.6(4)
C26_1	C27_1	C38_1	124.6(4)
C38_1	C27_1	C28_1	116.6(4)
C1_1	C28_1	C27_1	119.1(4)
C35_1	C28_1	C1_1	123.9(4)
C35_1	C28_1	C27_1	116.8(4)
C30_1	C29_1	C26_1	122.2(4)
C30_1	C29_1	C34_1	118.9(2)
C34_1	C29_1	C26_1	118.9(4)
C29_1	C30_1	H30_1	119.6
C29_1	C30_1	C31_1	120.8(2)
C31_1	C30_1	H30_1	119.6
C30_1	C31_1	H31_1	120.2
C32_1	C31_1	C30_1	119.6(2)
C32_1	C31_1	H31_1	120.2
C31_1	C32_1	H32_1	119.9
C33_1	C32_1	C31_1	120.2(2)
C33_1	C32_1	H32_1	119.9
C32_1	C33_1	H33_1	120.3
C32_1	C33_1	C34_1	119.5(2)
C34_1	C33_1	H33_1	120.3
C29_1	C34_1	C33_1	121.0(2)
C29_1	C34_1	H34_1	119.5
C33_1	C34_1	H34_1	119.5
C28_1	C35_1	H35_1	118.6
C36_1	C35_1	C28_1	122.9(4)
C36_1	C35_1	H35_1	118.6
C35_1	C36_1	H36_1	119.6
C35_1	C36_1	C37_1	120.7(5)
C37_1	C36_1	H36_1	119.6

Atom	Atom	Atom	Angle/°
C36_1	C37_1	C38_1	119.5(4)
C36_1	C37_1	C39_1	121.7(4)
C38_1	C37_1	C39_1	118.8(4)
C27_1	C38_1	H38_1	118.6
C37_1	C38_1	C27_1	122.8(4)
C37_1	C38_1	H38_1	118.6
C40_1	C39_1	C37_1	120.4(4)
C40_1	C39_1	C44_1	118.5(3)
C44_1	C39_1	C37_1	121.1(4)
C39_1	C40_1	H40_1	119.9
C39_1	C40_1	C41_1	120.2(4)
C41_1	C40_1	H40_1	119.9
C40_1	C41_1	H41_1	121.3
C42_1	C41_1	C40_1	117.5(4)
C42_1	C41_1	H41_1	121.3
C41_1	C42_1	H42_1	117.7
C43_1	C42_1	C41_1	124.5(5)
C43_1	C42_1	H42_1	117.7
C42_1	C43_1	H43_1	121.0
C42_1	C43_1	C44_1	118.0(4)
C44_1	C43_1	H43_1	121.0
C39_1	C44_1	H44_1	119.4
C43_1	C44_1	C39_1	121.3(4)
C43_1	C44_1	H44_1	119.4
O1_2	P1_2	O3_2	112.6(3)
O1_2	P1_2	O4_2	105.0(3)
O2_2	P1_2	O1_2	118.6(3)
O2_2	P1_2	O3_2	105.7(3)
O2_2	P1_2	O4_2	110.6(3)
O3_2	P1_2	O4_2	103.3(2)
P1_2	O1_2	Rh5[1]	115.6(3)
P1_2	O2_2	Rh5	121.3(4)
C3_2	O3_2	P1_2	116.3(3)
C24_2	O4_2	P1_2	118.0(3)
C24_2	C1_2	C2_2	118.7(4)
C24_2	C1_2	C28_2	117.7(4)
C28_2	C1_2	C2_2	123.5(4)
C3_2	C2_2	C1_2	117.7(4)
C3_2	C2_2	C7_2	118.6(4)
C7_2	C2_2	C1_2	123.6(4)
O3_2	C3_2	C4_2	115.3(4)
C2_2	C3_2	O3_2	120.0(4)
C2_2	C3_2	C4_2	124.6(4)
C3_2	C4_2	H4_2	121.2
C5_2	C4_2	C3_2	117.6(4)
C5_2	C4_2	H4_2	121.2
C4_2	C5_2	C6_2	121.0(4)
C4_2	C5_2	C8_2	117.4(4)
C6_2	C5_2	C8_2	121.5(4)
C5_2	C6_2	C7_2	118.9(4)
C14_2	C6_2	C5_2	121.6(4)
C14_2	C6_2	C7_2	118.2(4)
C2_2	C7_2	C6_2	119.1(4)
C17_2	C7_2	C2_2	124.4(4)
C17_2	C7_2	C6_2	116.0(4)
C9_2	C8_2	C5_2	119.3(4)

Atom	Atom	Atom	Angle/°
C13_2	C8_2	C5_2	120.0(4)
C13_2	C8_2	C9_2	120.6(4)
C8_2	C9_2	H9_2	120.8
C8_2	C9_2	C10_2	118.4(4)
C10_2	C9_2	H9_2	120.8
C9_2	C10_2	H10_2	121.9
C11_2	C10_2	C9_2	116.1(4)
C11_2	C10_2	H10_2	121.9
C10_2	C11_2	H11_2	115.7
C12_2	C11_2	C10_2	128.6(6)
C12_2	C11_2	H11_2	115.7
C11_2	C12_2	H12_2	122.4
C11_2	C12_2	C13_2	115.2(4)
C13_2	C12_2	H12_2	122.4
C8_2	C13_2	C12_2	121.1(4)
C8_2	C13_2	H13_2	119.5
C12_2	C13_2	H13_2	119.5
C6_2	C14_2	H14_2	119.0
C15_2	C14_2	C6_2	122.0(4)
C15_2	C14_2	H14_2	119.0
C14_2	C15_2	C16_2	119.3(5)
C14_2	C15_2	C18_2	119.4(4)
C16_2	C15_2	C18_2	119.3(5)
C15_2	C16_2	H16_2	120.2
C17_2	C16_2	C15_2	119.6(5)
C17_2	C16_2	H16_2	120.2
C7_2	C17_2	H17_2	117.5
C16_2	C17_2	C7_2	124.9(5)
C16_2	C17_2	H17_2	117.5
C19_2	C18_2	C15_2	121.6(4)
C19_2	C18_2	C23_2	118.2(2)
C23_2	C18_2	C15_2	119.7(4)
C18_2	C19_2	H19_2	119.4
C20_2	C19_2	C18_2	121.1(3)
C20_2	C19_2	H19_2	119.4
C19_2	C20_2	H20_2	120.8
C21_2	C20_2	C19_2	118.5(3)
C21_2	C20_2	H20_2	120.8
C20_2	C21_2	H21_2	118.6
C20_2	C21_2	C22_2	122.8(4)
C22_2	C21_2	H21_2	118.6
C21_2	C22_2	H22_2	120.7
C21_2	C22_2	C23_2	118.5(3)
C23_2	C22_2	H22_2	120.7
C18_2	C23_2	H23_2	119.5
C22_2	C23_2	C18_2	120.9(3)
C22_2	C23_2	H23_2	119.5
O4_2	C24_2	C25_2	115.9(4)
C1_2	C24_2	O4_2	119.9(4)
C1_2	C24_2	C25_2	124.2(4)
C24_2	C25_2	H25_2	120.4
C26_2	C25_2	C24_2	119.3(4)
C26_2	C25_2	H25_2	120.4
C25_2	C26_2	C27_2	120.8(4)
C25_2	C26_2	C29_2	117.8(4)
C27_2	C26_2	C29_2	121.4(4)

Atom	Atom	Atom	Angle/°
C26_2	C27_2	C28_2	118.5(4)
C26_2	C27_2	C38_2	124.3(4)
C38_2	C27_2	C28_2	116.5(4)
C1_2	C28_2	C27_2	119.4(4)
C35_2	C28_2	C1_2	123.4(4)
C35_2	C28_2	C27_2	117.1(4)
C30_2	C29_2	C26_2	122.2(4)
C34_2	C29_2	C26_2	119.2(4)
C34_2	C29_2	C30_2	118.5(2)
C29_2	C30_2	H30_2	119.5
C31_2	C30_2	C29_2	121.0(2)
C31_2	C30_2	H30_2	119.5
C30_2	C31_2	H31_2	120.2
C30_2	C31_2	C32_2	119.7(2)
C32_2	C31_2	H31_2	120.2
C31_2	C32_2	H32_2	120.0
C31_2	C32_2	C33_2	119.9(2)
C33_2	C32_2	H32_2	120.0
C32_2	C33_2	H33_2	120.4
C32_2	C33_2	C34_2	119.3(2)
C34_2	C33_2	H33_2	120.4
C29_2	C34_2	C33_2	120.9(3)
C29_2	C34_2	H34_2	119.6
C33_2	C34_2	H34_2	119.6
C28_2	C35_2	H35_2	118.6
C36_2	C35_2	C28_2	122.8(4)
C36_2	C35_2	H35_2	118.6
C35_2	C36_2	H36_2	119.7
C35_2	C36_2	C37_2	120.7(5)
C37_2	C36_2	H36_2	119.7
C36_2	C37_2	C39_2	121.5(4)
C38_2	C37_2	C36_2	119.5(4)
C38_2	C37_2	C39_2	118.8(4)
C27_2	C38_2	H38_2	118.5
C37_2	C38_2	C27_2	122.9(4)
C37_2	C38_2	H38_2	118.5
C40_2	C39_2	C37_2	120.3(4)
C40_2	C39_2	C44_2	118.5(3)
C44_2	C39_2	C37_2	121.1(4)
C39_2	C40_2	H40_2	119.9
C39_2	C40_2	C41_2	120.1(4)
C41_2	C40_2	H40_2	119.9
C40_2	C41_2	H41_2	121.2
C42_2	C41_2	C40_2	117.6(4)
C42_2	C41_2	H41_2	121.2
C41_2	C42_2	H42_2	117.8
C43_2	C42_2	C41_2	124.5(5)
C43_2	C42_2	H42_2	117.8
C42_2	C43_2	H43_2	121.0
C42_2	C43_2	C44_2	118.0(4)
C44_2	C43_2	H43_2	121.0
C39_2	C44_2	H44_2	119.3
C43_2	C44_2	C39_2	121.3(4)
C43_2	C44_2	H44_2	119.3
O1_3	P1_3	O3_3	112.5(2)
O1_3	P1_3	O4_3	105.3(2)

Atom	Atom	Atom	Angle/°
O2_3	P1_3	O1_3	118.7(2)
O2_3	P1_3	O3_3	105.5(2)
O2_3	P1_3	O4_3	110.7(2)
O3_3	P1_3	O4_3	103.1(2)
P1_3	O1_3	Rh4	114.5(2)
P1_3	O2_3	Rh3	115.9(3)
C3_3	O3_3	P1_3	115.6(3)
C24_3	O4_3	P1_3	118.6(3)
C24_3	C1_3	C2_3	118.9(4)
C24_3	C1_3	C28_3	118.0(4)
C28_3	C1_3	C2_3	122.9(4)
C3_3	C2_3	C1_3	117.5(3)
C3_3	C2_3	C7_3	118.5(4)
C7_3	C2_3	C1_3	123.8(4)
O3_3	C3_3	C4_3	115.3(3)
C2_3	C3_3	O3_3	119.8(3)
C2_3	C3_3	C4_3	124.8(4)
C3_3	C4_3	H4_3	121.3
C5_3	C4_3	C3_3	117.4(4)
C5_3	C4_3	H4_3	121.3
C4_3	C5_3	C6_3	120.9(4)
C4_3	C5_3	C8_3	117.7(4)
C6_3	C5_3	C8_3	121.4(4)
C5_3	C6_3	C7_3	119.3(4)
C14_3	C6_3	C5_3	122.0(4)
C14_3	C6_3	C7_3	118.7(4)
C2_3	C7_3	C6_3	119.0(4)
C17_3	C7_3	C2_3	125.2(4)
C17_3	C7_3	C6_3	115.7(4)
C9_3	C8_3	C5_3	119.4(4)
C13_3	C8_3	C5_3	119.9(4)
C13_3	C8_3	C9_3	120.5(3)
C8_3	C9_3	H9_3	120.7
C8_3	C9_3	C10_3	118.6(4)
C10_3	C9_3	H9_3	120.7
C9_3	C10_3	H10_3	122.0
C11_3	C10_3	C9_3	116.1(4)
C11_3	C10_3	H10_3	122.0
C10_3	C11_3	H11_3	115.8
C12_3	C11_3	C10_3	128.4(5)
C12_3	C11_3	H11_3	115.8
C11_3	C12_3	H12_3	122.3
C11_3	C12_3	C13_3	115.3(4)
C13_3	C12_3	H12_3	122.3
C8_3	C13_3	C12_3	121.1(4)
C8_3	C13_3	H13_3	119.5
C12_3	C13_3	H13_3	119.5
C6_3	C14_3	H14_3	119.2
C15_3	C14_3	C6_3	121.5(4)
C15_3	C14_3	H14_3	119.2
C14_3	C15_3	C18_3	119.9(4)
C16_3	C15_3	C14_3	119.6(4)
C16_3	C15_3	C18_3	120.5(4)
C15_3	C16_3	H16_3	120.2
C17_3	C16_3	C15_3	119.7(5)
C17_3	C16_3	H16_3	120.2

Atom	Atom	Atom	Angle/°
C7_3	C17_3	H17_3	117.6
C16_3	C17_3	C7_3	124.8(5)
C16_3	C17_3	H17_3	117.6
C19_3	C18_3	C15_3	121.5(4)
C19_3	C18_3	C23_3	118.0(2)
C23_3	C18_3	C15_3	119.8(4)
C18_3	C19_3	H19_3	119.4
C20_3	C19_3	C18_3	121.2(3)
C20_3	C19_3	H19_3	119.4
C19_3	C20_3	H20_3	120.8
C21_3	C20_3	C19_3	118.4(3)
C21_3	C20_3	H20_3	120.8
C20_3	C21_3	H21_3	118.6
C20_3	C21_3	C22_3	122.7(4)
C22_3	C21_3	H21_3	118.6
C21_3	C22_3	H22_3	120.7
C21_3	C22_3	C23_3	118.5(3)
C23_3	C22_3	H22_3	120.7
C18_3	C23_3	H23_3	119.6
C22_3	C23_3	C18_3	120.9(3)
C22_3	C23_3	H23_3	119.6
O4_3	C24_3	C25_3	115.8(4)
C1_3	C24_3	O4_3	120.2(4)
C1_3	C24_3	C25_3	123.9(4)
C24_3	C25_3	H25_3	120.3
C26_3	C25_3	C24_3	119.4(4)
C26_3	C25_3	H25_3	120.3
C25_3	C26_3	C27_3	120.9(4)
C25_3	C26_3	C29_3	117.4(4)
C27_3	C26_3	C29_3	121.1(4)
C26_3	C27_3	C28_3	118.5(4)
C26_3	C27_3	C38_3	124.9(4)
C38_3	C27_3	C28_3	116.5(4)
C1_3	C28_3	C27_3	119.3(4)
C35_3	C28_3	C1_3	123.1(4)
C35_3	C28_3	C27_3	116.8(4)
C30_3	C29_3	C26_3	122.1(4)
C30_3	C29_3	C34_3	118.8(2)
C34_3	C29_3	C26_3	119.1(4)
C29_3	C30_3	H30_3	119.5
C29_3	C30_3	C31_3	121.0(2)
C31_3	C30_3	H30_3	119.5
C30_3	C31_3	H31_3	120.2
C32_3	C31_3	C30_3	119.5(2)
C32_3	C31_3	H31_3	120.2
C31_3	C32_3	H32_3	119.9
C33_3	C32_3	C31_3	120.1(2)
C33_3	C32_3	H32_3	119.9
C32_3	C33_3	H33_3	120.2
C32_3	C33_3	C34_3	119.5(2)
C34_3	C33_3	H33_3	120.2
C29_3	C34_3	C33_3	120.9(2)
C29_3	C34_3	H34_3	119.5
C33_3	C34_3	H34_3	119.5
C28_3	C35_3	H35_3	118.6
C36_3	C35_3	C28_3	122.7(4)

Atom	Atom	Atom	Angle/°
C36_3	C35_3	H35_3	118.6
C35_3	C36_3	H36_3	119.8
C35_3	C36_3	C37_3	120.4(4)
C37_3	C36_3	H36_3	119.8
C36_3	C37_3	C39_3	121.2(4)
C38_3	C37_3	C36_3	119.1(4)
C38_3	C37_3	C39_3	119.2(4)
C27_3	C38_3	H38_3	118.7
C37_3	C38_3	C27_3	122.5(4)
C37_3	C38_3	H38_3	118.7
C40_3	C39_3	C37_3	120.0(4)
C40_3	C39_3	C44_3	118.5(3)
C44_3	C39_3	C37_3	121.3(4)
C39_3	C40_3	H40_3	119.9
C39_3	C40_3	C41_3	120.2(4)
C41_3	C40_3	H40_3	119.9
C40_3	C41_3	H41_3	121.3
C42_3	C41_3	C40_3	117.5(4)
C42_3	C41_3	H41_3	121.3
C41_3	C42_3	H42_3	117.7
C43_3	C42_3	C41_3	124.5(5)
C43_3	C42_3	H42_3	117.7
C42_3	C43_3	H43_3	121.0
C42_3	C43_3	C44_3	118.0(4)
C44_3	C43_3	H43_3	121.0
C39_3	C44_3	H44_3	119.4
C43_3	C44_3	C39_3	121.3(4)
C43_3	C44_3	H44_3	119.4
O1_5	P1_5	O3_5	112.3(2)
O1_5	P1_5	O4_5	104.5(2)
O2_5	P1_5	O1_5	118.3(3)
O2_5	P1_5	O3_5	106.0(2)
O2_5	P1_5	O4_5	111.6(2)
O3_5	P1_5	O4_5	103.2(2)
P1_5	O1_5	Rh2	117.2(3)
P1_5	O2_5	Rh1	116.7(3)
C3_5	O3_5	P1_5	116.1(3)
C24_5	O4_5	P1_5	120.1(3)
C24_5	C1_5	C2_5	119.0(4)
C24_5	C1_5	C28_5	117.7(4)
C28_5	C1_5	C2_5	122.7(4)
C3_5	C2_5	C1_5	117.6(4)
C3_5	C2_5	C7_5	118.2(4)
C7_5	C2_5	C1_5	124.1(4)
O3_5	C3_5	C4_5	115.1(4)
C2_5	C3_5	O3_5	119.9(4)
C2_5	C3_5	C4_5	125.0(4)
C3_5	C4_5	H4_5	121.2
C5_5	C4_5	C3_5	117.5(4)
C5_5	C4_5	H4_5	121.2
C4_5	C5_5	C6_5	120.7(4)
C4_5	C5_5	C8_5	117.6(4)
C6_5	C5_5	C8_5	121.7(4)
C5_5	C6_5	C7_5	119.4(4)
C14_5	C6_5	C5_5	122.0(4)
C14_5	C6_5	C7_5	118.6(4)

Atom	Atom	Atom	Angle/°
C2_5	C7_5	C6_5	119.1(4)
C17_5	C7_5	C2_5	124.9(4)
C17_5	C7_5	C6_5	115.8(4)
C9_5	C8_5	C5_5	119.3(4)
C13_5	C8_5	C5_5	120.1(4)
C13_5	C8_5	C9_5	120.6(4)
C8_5	C9_5	H9_5	120.8
C8_5	C9_5	C10_5	118.5(4)
C10_5	C9_5	H9_5	120.8
C9_5	C10_5	H10_5	122.0
C11_5	C10_5	C9_5	116.0(4)
C11_5	C10_5	H10_5	122.0
C10_5	C11_5	H11_5	115.7
C12_5	C11_5	C10_5	128.7(6)
C12_5	C11_5	H11_5	115.7
C11_5	C12_5	H12_5	122.4
C11_5	C12_5	C13_5	115.2(4)
C13_5	C12_5	H12_5	122.4
C8_5	C13_5	C12_5	121.0(4)
C8_5	C13_5	H13_5	119.5
C12_5	C13_5	H13_5	119.5
C6_5	C14_5	H14_5	119.2
C15_5	C14_5	C6_5	121.6(4)
C15_5	C14_5	H14_5	119.2
C14_5	C15_5	C18_5	119.9(4)
C16_5	C15_5	C14_5	119.7(4)
C16_5	C15_5	C18_5	120.4(4)
C15_5	C16_5	H16_5	120.2
C17_5	C16_5	C15_5	119.5(5)
C17_5	C16_5	H16_5	120.2
C7_5	C17_5	H17_5	117.6
C16_5	C17_5	C7_5	124.9(5)
C16_5	C17_5	H17_5	117.6
C19_5	C18_5	C15_5	121.6(4)
C19_5	C18_5	C23_5	118.2(2)
C23_5	C18_5	C15_5	120.1(4)
C18_5	C19_5	H19_5	119.4
C20_5	C19_5	C18_5	121.17(19)
C20_5	C19_5	H19_5	119.4
C19_5	C20_5	H20_5	120.8
C21_5	C20_5	C19_5	118.5(2)
C21_5	C20_5	H20_5	120.8
C20_5	C21_5	H21_5	118.8
C20_5	C21_5	C22_5	122.5(3)
C22_5	C21_5	H21_5	118.8
C21_5	C22_5	H22_5	120.8
C21_5	C22_5	C23_5	118.5(2)
C23_5	C22_5	H22_5	120.8
C18_5	C23_5	H23_5	119.4
C22_5	C23_5	C18_5	121.1(2)
C22_5	C23_5	H23_5	119.4
O4_5	C24_5	C25_5	114.6(4)
C1_5	C24_5	O4_5	121.3(4)
C1_5	C24_5	C25_5	124.0(4)
C24_5	C25_5	H25_5	120.2
C26_5	C25_5	C24_5	119.5(4)

Atom	Atom	Atom	Angle/°
C26_5	C25_5	H25_5	120.2
C25_5	C26_5	C27_5	120.6(4)
C25_5	C26_5	C29_5	117.4(4)
C27_5	C26_5	C29_5	121.5(4)
C26_5	C27_5	C28_5	118.6(4)
C26_5	C27_5	C38_5	124.6(4)
C38_5	C27_5	C28_5	116.7(4)
C1_5	C28_5	C27_5	119.4(4)
C35_5	C28_5	C1_5	123.5(4)
C35_5	C28_5	C27_5	116.3(4)
C30_5	C29_5	C26_5	122.0(4)
C30_5	C29_5	C34_5	118.76(19)
C34_5	C29_5	C26_5	119.2(4)
C29_5	C30_5	H30_5	119.6
C29_5	C30_5	C31_5	120.9(2)
C31_5	C30_5	H30_5	119.6
C30_5	C31_5	H31_5	120.2
C32_5	C31_5	C30_5	119.7(2)
C32_5	C31_5	H31_5	120.2
C31_5	C32_5	H32_5	119.9
C33_5	C32_5	C31_5	120.14(19)
C33_5	C32_5	H32_5	119.9
C32_5	C33_5	H33_5	120.2
C32_5	C33_5	C34_5	119.5(2)
C34_5	C33_5	H33_5	120.2
C29_5	C34_5	H34_5	119.5
C33_5	C34_5	C29_5	121.0(2)
C33_5	C34_5	H34_5	119.5
C28_5	C35_5	H35_5	118.5
C36_5	C35_5	C28_5	123.1(4)
C36_5	C35_5	H35_5	118.5
C35_5	C36_5	H36_5	119.7
C35_5	C36_5	C37_5	120.6(4)
C37_5	C36_5	H36_5	119.7
C36_5	C37_5	C39_5	121.9(4)
C38_5	C37_5	C36_5	119.3(4)
C38_5	C37_5	C39_5	118.7(4)
C27_5	C38_5	H38_5	118.5
C37_5	C38_5	C27_5	123.0(4)
C37_5	C38_5	H38_5	118.5
C40_5	C39_5	C37_5	120.3(4)
C40_5	C39_5	C44_5	118.5(3)
C44_5	C39_5	C37_5	121.2(4)
C39_5	C40_5	H40_5	119.9
C39_5	C40_5	C41_5	120.2(4)
C41_5	C40_5	H40_5	119.9
C40_5	C41_5	H41_5	121.2
C42_5	C41_5	C40_5	117.6(4)
C42_5	C41_5	H41_5	121.2
C41_5	C42_5	H42_5	117.8
C43_5	C42_5	C41_5	124.4(5)
C43_5	C42_5	H42_5	117.8
C42_5	C43_5	H43_5	121.0
C42_5	C43_5	C44_5	118.1(4)
C44_5	C43_5	H43_5	121.0
C39_5	C44_5	H44_5	119.3

Atom	Atom	Atom	Angle/°
C43_5	C44_5	C39_5	121.3(4)
C43_5	C44_5	H44_5	119.3
O1_6	P1_6	O3_6	112.5(3)
O1_6	P1_6	O4_6	105.3(3)
O2_6	P1_6	O1_6	118.5(3)
O2_6	P1_6	O3_6	105.6(3)
O2_6	P1_6	O4_6	110.3(3)
O3_6	P1_6	O4_6	103.7(2)
P1_6	O1_6	Rh5[1]	121.1(3)
P1_6	O2_6	Rh5	115.9(3)
C3_6	O3_6	P1_6	116.7(3)
C24_6	O4_6	P1_6	118.2(3)
C24_6	C1_6	C2_6	118.8(4)
C24_6	C1_6	C28_6	117.7(4)
C28_6	C1_6	C2_6	123.4(4)
C3_6	C2_6	C1_6	118.2(4)
C3_6	C2_6	C7_6	118.4(4)
C7_6	C2_6	C1_6	123.4(4)
O3_6	C3_6	C4_6	114.9(4)
C2_6	C3_6	O3_6	120.4(4)
C2_6	C3_6	C4_6	124.7(4)
C3_6	C4_6	H4_6	121.2
C5_6	C4_6	C3_6	117.6(4)
C5_6	C4_6	H4_6	121.2
C4_6	C5_6	C6_6	120.9(4)
C4_6	C5_6	C8_6	117.5(4)
C6_6	C5_6	C8_6	121.7(4)
C5_6	C6_6	C7_6	119.0(4)
C14_6	C6_6	C5_6	121.8(4)
C14_6	C6_6	C7_6	118.4(4)
C2_6	C7_6	C6_6	119.2(4)
C17_6	C7_6	C2_6	124.6(4)
C17_6	C7_6	C6_6	116.1(4)
C9_6	C8_6	C5_6	119.3(4)
C13_6	C8_6	C5_6	120.2(4)
C13_6	C8_6	C9_6	120.4(3)
C8_6	C9_6	H9_6	120.8
C8_6	C9_6	C10_6	118.4(4)
C10_6	C9_6	H9_6	120.8
C9_6	C10_6	H10_6	121.8
C11_6	C10_6	C9_6	116.3(4)
C11_6	C10_6	H10_6	121.8
C10_6	C11_6	H11_6	115.8
C12_6	C11_6	C10_6	128.4(5)
C12_6	C11_6	H11_6	115.8
C11_6	C12_6	H12_6	122.4
C11_6	C12_6	C13_6	115.2(4)
C13_6	C12_6	H12_6	122.4
C8_6	C13_6	C12_6	121.2(4)
C8_6	C13_6	H13_6	119.4
C12_6	C13_6	H13_6	119.4
C6_6	C14_6	H14_6	119.2
C15_6	C14_6	C6_6	121.6(4)
C15_6	C14_6	H14_6	119.2
C14_6	C15_6	C16_6	119.7(4)
C14_6	C15_6	C18_6	120.2(4)

Atom	Atom	Atom	Angle/°
C16_6	C15_6	C18_6	120.1(4)
C15_6	C16_6	H16_6	120.2
C17_6	C16_6	C15_6	119.6(5)
C17_6	C16_6	H16_6	120.2
C7_6	C17_6	H17_6	117.7
C16_6	C17_6	C7_6	124.6(5)
C16_6	C17_6	H17_6	117.7
C19_6	C18_6	C15_6	121.8(4)
C19_6	C18_6	C23_6	118.3(2)
C23_6	C18_6	C15_6	119.7(4)
C18_6	C19_6	H19_6	119.5
C20_6	C19_6	C18_6	120.9(3)
C20_6	C19_6	H19_6	119.5
C19_6	C20_6	H20_6	120.7
C21_6	C20_6	C19_6	118.5(3)
C21_6	C20_6	H20_6	120.7
C20_6	C21_6	H21_6	118.6
C20_6	C21_6	C22_6	122.8(4)
C22_6	C21_6	H21_6	118.6
C21_6	C22_6	H22_6	120.8
C21_6	C22_6	C23_6	118.4(3)
C23_6	C22_6	H22_6	120.8
C18_6	C23_6	H23_6	119.5
C22_6	C23_6	C18_6	121.0(3)
C22_6	C23_6	H23_6	119.5
O4_6	C24_6	C25_6	115.4(4)
C1_6	C24_6	O4_6	119.7(4)
C1_6	C24_6	C25_6	124.4(4)
C24_6	C25_6	H25_6	120.5
C26_6	C25_6	C24_6	119.0(4)
C26_6	C25_6	H25_6	120.5
C25_6	C26_6	C27_6	120.7(4)
C25_6	C26_6	C29_6	117.8(4)
C27_6	C26_6	C29_6	121.3(4)
C26_6	C27_6	C28_6	118.9(4)
C26_6	C27_6	C38_6	124.5(4)
C38_6	C27_6	C28_6	116.7(4)
C1_6	C28_6	C27_6	119.1(4)
C35_6	C28_6	C1_6	123.8(4)
C35_6	C28_6	C27_6	116.8(4)
C30_6	C29_6	C26_6	122.2(4)
C30_6	C29_6	C34_6	118.7(2)
C34_6	C29_6	C26_6	119.0(4)
C29_6	C30_6	H30_6	119.5
C29_6	C30_6	C31_6	120.9(2)
C31_6	C30_6	H30_6	119.5
C30_6	C31_6	H31_6	120.2
C32_6	C31_6	C30_6	119.6(2)
C32_6	C31_6	H31_6	120.2
C31_6	C32_6	H32_6	119.9
C31_6	C32_6	C33_6	120.1(2)
C33_6	C32_6	H32_6	119.9
C32_6	C33_6	H33_6	120.2
C34_6	C33_6	C32_6	119.6(2)
C34_6	C33_6	H33_6	120.2
C29_6	C34_6	H34_6	119.5

Atom	Atom	Atom	Angle/°
C33_6	C34_6	C29_6	121.0(2)
C33_6	C34_6	H34_6	119.5
C28_6	C35_6	H35_6	118.5
C36_6	C35_6	C28_6	122.9(4)
C36_6	C35_6	H35_6	118.5
C35_6	C36_6	H36_6	119.6
C35_6	C36_6	C37_6	120.8(4)
C37_6	C36_6	H36_6	119.6
C36_6	C37_6	C38_6	119.4(4)
C36_6	C37_6	C39_6	122.3(4)
C38_6	C37_6	C39_6	118.2(4)
C27_6	C38_6	H38_6	118.6
C37_6	C38_6	C27_6	122.8(4)
C37_6	C38_6	H38_6	118.6
C40_6	C39_6	C37_6	120.7(4)
C40_6	C39_6	C44_6	118.6(3)
C44_6	C39_6	C37_6	120.5(4)
C39_6	C40_6	H40_6	119.9
C39_6	C40_6	C41_6	120.1(4)
C41_6	C40_6	H40_6	119.9
C40_6	C41_6	H41_6	121.2
C42_6	C41_6	C40_6	117.5(4)
C42_6	C41_6	H41_6	121.2
C41_6	C42_6	H42_6	117.8
C43_6	C42_6	C41_6	124.5(5)
C43_6	C42_6	H42_6	117.8
C42_6	C43_6	H43_6	121.0
C42_6	C43_6	C44_6	118.0(4)
C44_6	C43_6	H43_6	121.0
C39_6	C44_6	H44_6	119.4
C43_6	C44_6	C39_6	121.3(4)
C43_6	C44_6	H44_6	119.4
C2S_7	O1S_7	Rh1	127.5(8)
C2S_7	O1S_7	C4S_7	117.0(7)
C4S_7	O1S_7	Rh1	113.4(8)
O1S_7	C2S_7	H2SA_7	108.3
O1S_7	C2S_7	H2SB_7	108.3
O1S_7	C2S_7	C3S_7	116.1(8)
H2SA_7	C2S_7	H2SB_7	107.4
C3S_7	C2S_7	H2SA_7	108.3
C3S_7	C2S_7	H2SB_7	108.3
C2S_7	C3S_7	H3SA_7	109.5
C2S_7	C3S_7	H3SB_7	109.5
C2S_7	C3S_7	H3SC_7	109.5
H3SA_7	C3S_7	H3SB_7	109.5
H3SA_7	C3S_7	H3SC_7	109.5
H3SB_7	C3S_7	H3SC_7	109.5
O1S_7	C4S_7	H4SA_7	109.0
O1S_7	C4S_7	H4SB_7	109.0
O1S_7	C4S_7	C5S_7	112.8(7)
H4SA_7	C4S_7	H4SB_7	107.8
C5S_7	C4S_7	H4SA_7	109.0
C5S_7	C4S_7	H4SB_7	109.0
C4S_7	C5S_7	H5SA_7	109.5
C4S_7	C5S_7	H5SB_7	109.5
C4S_7	C5S_7	H5SC_7	109.5

Atom	Atom	Atom	Angle/°
H5SA_7	C5S_7	H5SB_7	109.5
H5SA_7	C5S_7	H5SC_7	109.5
H5SB_7	C5S_7	H5SC_7	109.5
C2S_8	O1S_8	Rh3	124.4(7)
C2S_8	O1S_8	C4S_8	116.9(6)
C4S_8	O1S_8	Rh3	118.7(6)
O1S_8	C2S_8	H2SA_8	108.3
O1S_8	C2S_8	H2SB_8	108.3
O1S_8	C2S_8	C3S_8	115.8(8)
H2SA_8	C2S_8	H2SB_8	107.4
C3S_8	C2S_8	H2SA_8	108.3
C3S_8	C2S_8	H2SB_8	108.3
C2S_8	C3S_8	H3SA_8	109.5
C2S_8	C3S_8	H3SB_8	109.5
C2S_8	C3S_8	H3SC_8	109.5
H3SA_8	C3S_8	H3SB_8	109.5
H3SA_8	C3S_8	H3SC_8	109.5
H3SB_8	C3S_8	H3SC_8	109.5
O1S_8	C4S_8	H4SA_8	109.1
O1S_8	C4S_8	H4SB_8	109.1
O1S_8	C4S_8	C5S_8	112.5(7)
H4SA_8	C4S_8	H4SB_8	107.8
C5S_8	C4S_8	H4SA_8	109.1
C5S_8	C4S_8	H4SB_8	109.1
C4S_8	C5S_8	H5SA_8	109.5
C4S_8	C5S_8	H5SB_8	109.5
C4S_8	C5S_8	H5SC_8	109.5
H5SA_8	C5S_8	H5SB_8	109.5
H5SA_8	C5S_8	H5SC_8	109.5
H5SB_8	C5S_8	H5SC_8	109.5
O1_9	P1_9	O3_9	112.2(2)
O1_9	P1_9	O4_9	104.9(2)
O2_9	P1_9	O1_9	118.5(3)
O2_9	P1_9	O3_9	105.8(2)
O2_9	P1_9	O4_9	111.0(2)
O3_9	P1_9	O4_9	103.4(2)
P1_9	O1_9	Rh4	113.6(3)
P1_9	O2_9	Rh3	116.7(3)
C3_9	O3_9	P1_9	116.0(3)
C24_9	O4_9	P1_9	118.0(3)
C24_9	C1_9	C2_9	118.7(4)
C24_9	C1_9	C28_9	117.8(4)
C28_9	C1_9	C2_9	123.4(4)
C3_9	C2_9	C1_9	117.8(4)
C3_9	C2_9	C7_9	118.4(4)
C7_9	C2_9	C1_9	123.9(4)
O3_9	C3_9	C4_9	115.4(4)
C2_9	C3_9	O3_9	119.7(4)
C2_9	C3_9	C4_9	124.7(4)
C3_9	C4_9	H4_9	121.2
C5_9	C4_9	C3_9	117.5(4)
C5_9	C4_9	H4_9	121.2
C4_9	C5_9	C6_9	121.0(4)
C4_9	C5_9	C8_9	117.1(4)
C6_9	C5_9	C8_9	121.9(4)
C5_9	C6_9	C7_9	119.0(4)

Atom	Atom	Atom	Angle/°
C14_9	C6_9	C5_9	122.6(4)
C14_9	C6_9	C7_9	118.4(4)
C2_9	C7_9	C6_9	119.3(4)
C17_9	C7_9	C2_9	124.5(4)
C17_9	C7_9	C6_9	116.1(4)
C9_9	C8_9	C5_9	119.1(4)
C13_9	C8_9	C5_9	119.8(4)
C13_9	C8_9	C9_9	120.6(4)
C8_9	C9_9	H9_9	120.7
C8_9	C9_9	C10_9	118.5(4)
C10_9	C9_9	H9_9	120.7
C9_9	C10_9	H10_9	122.0
C11_9	C10_9	C9_9	116.0(4)
C11_9	C10_9	H10_9	122.0
C10_9	C11_9	H11_9	115.7
C12_9	C11_9	C10_9	128.6(6)
C12_9	C11_9	H11_9	115.7
C11_9	C12_9	H12_9	122.4
C11_9	C12_9	C13_9	115.3(4)
C13_9	C12_9	H12_9	122.4
C8_9	C13_9	C12_9	121.0(4)
C8_9	C13_9	H13_9	119.5
C12_9	C13_9	H13_9	119.5
C6_9	C14_9	H14_9	119.2
C15_9	C14_9	C6_9	121.5(4)
C15_9	C14_9	H14_9	119.2
C14_9	C15_9	C16_9	119.7(5)
C14_9	C15_9	C18_9	120.6(4)
C16_9	C15_9	C18_9	119.6(4)
C15_9	C16_9	H16_9	120.2
C17_9	C16_9	C15_9	119.6(5)
C17_9	C16_9	H16_9	120.2
C7_9	C17_9	H17_9	117.7
C16_9	C17_9	C7_9	124.6(5)
C16_9	C17_9	H17_9	117.7
C19_9	C18_9	C15_9	121.8(4)
C19_9	C18_9	C23_9	118.2(2)
C23_9	C18_9	C15_9	119.6(4)
C18_9	C19_9	H19_9	119.5
C20_9	C19_9	C18_9	121.1(3)
C20_9	C19_9	H19_9	119.5
C19_9	C20_9	H20_9	120.8
C21_9	C20_9	C19_9	118.4(3)
C21_9	C20_9	H20_9	120.8
C20_9	C21_9	H21_9	118.6
C20_9	C21_9	C22_9	122.8(4)
C22_9	C21_9	H21_9	118.6
C21_9	C22_9	H22_9	120.7
C21_9	C22_9	C23_9	118.5(3)
C23_9	C22_9	H22_9	120.7
C18_9	C23_9	H23_9	119.5
C22_9	C23_9	C18_9	120.9(3)
C22_9	C23_9	H23_9	119.5
O4_9	C24_9	C25_9	115.5(4)
C1_9	C24_9	O4_9	120.0(4)
C1_9	C24_9	C25_9	124.1(4)

Atom	Atom	Atom	Angle/°
C24_9	C25_9	H25_9	120.4
C26_9	C25_9	C24_9	119.1(4)
C26_9	C25_9	H25_9	120.4
C25_9	C26_9	C27_9	120.9(4)
C25_9	C26_9	C29_9	117.4(4)
C27_9	C26_9	C29_9	121.6(4)
C26_9	C27_9	C28_9	118.7(4)
C26_9	C27_9	C38_9	124.8(4)
C38_9	C27_9	C28_9	116.5(4)
C1_9	C28_9	C27_9	119.2(4)
C35_9	C28_9	C1_9	123.7(4)
C35_9	C28_9	C27_9	116.8(4)
C30_9	C29_9	C26_9	122.1(4)
C30_9	C29_9	C34_9	118.7(2)
C34_9	C29_9	C26_9	119.1(4)
C29_9	C30_9	H30_9	119.5
C29_9	C30_9	C31_9	121.0(2)
C31_9	C30_9	H30_9	119.5
C30_9	C31_9	H31_9	120.2
C32_9	C31_9	C30_9	119.6(2)
C32_9	C31_9	H31_9	120.2
C31_9	C32_9	H32_9	120.0
C33_9	C32_9	C31_9	120.1(2)
C33_9	C32_9	H32_9	120.0
C32_9	C33_9	H33_9	120.2
C34_9	C33_9	C32_9	119.5(2)
C34_9	C33_9	H33_9	120.2
C29_9	C34_9	H34_9	119.5
C33_9	C34_9	C29_9	121.1(2)
C33_9	C34_9	H34_9	119.5
C28_9	C35_9	H35_9	118.6
C36_9	C35_9	C28_9	122.8(4)
C36_9	C35_9	H35_9	118.6
C35_9	C36_9	H36_9	119.7
C35_9	C36_9	C37_9	120.6(5)
C37_9	C36_9	H36_9	119.7
C36_9	C37_9	C39_9	121.4(4)
C38_9	C37_9	C36_9	119.3(4)
C38_9	C37_9	C39_9	118.9(4)
C27_9	C38_9	H38_9	118.5
C37_9	C38_9	C27_9	122.9(4)
C37_9	C38_9	H38_9	118.5
C40_9	C39_9	C37_9	120.3(4)
C40_9	C39_9	C44_9	118.5(3)
C44_9	C39_9	C37_9	121.2(4)
C39_9	C40_9	H40_9	119.9
C39_9	C40_9	C41_9	120.2(4)
C41_9	C40_9	H40_9	119.9
C40_9	C41_9	H41_9	121.2
C42_9	C41_9	C40_9	117.6(4)
C42_9	C41_9	H41_9	121.2
C41_9	C42_9	H42_9	117.8
C43_9	C42_9	C41_9	124.4(5)
C43_9	C42_9	H42_9	117.8
C42_9	C43_9	H43_9	121.0
C42_9	C43_9	C44_9	118.0(4)

Atom	Atom	Atom	Angle/°
C44_9	C43_9	H43_9	121.0
C39_9	C44_9	H44_9	119.3
C43_9	C44_9	C39_9	121.3(4)
C43_9	C44_9	H44_9	119.3
O1_10	P1_10	O3_10	112.0(2)
O1_10	P1_10	O4_10	104.6(2)
O2_10	P1_10	O1_10	118.4(3)
O2_10	P1_10	O3_10	105.8(2)
O2_10	P1_10	O4_10	111.5(3)
O3_10	P1_10	O4_10	103.8(2)
P1_10	O1_10	Rh4	115.8(3)
P1_10	O2_10	Rh3	117.4(3)
C3_10	O3_10	P1_10	116.0(3)
C24_10	O4_10	P1_10	119.4(3)
C24_10	C1_10	C2_10	119.5(4)
C24_10	C1_10	C28_10	117.6(4)
C28_10	C1_10	C2_10	122.8(4)
C3_10	C2_10	C1_10	117.4(4)
C3_10	C2_10	C7_10	118.1(4)
C7_10	C2_10	C1_10	124.5(4)
O3_10	C3_10	C4_10	115.5(4)
C2_10	C3_10	O3_10	119.5(4)
C2_10	C3_10	C4_10	125.0(4)
C3_10	C4_10	H4_10	121.2
C5_10	C4_10	C3_10	117.6(4)
C5_10	C4_10	H4_10	121.2
C4_10	C5_10	C6_10	120.6(4)
C4_10	C5_10	C8_10	117.2(4)
C6_10	C5_10	C8_10	122.1(4)
C5_10	C6_10	C7_10	119.2(4)
C14_10	C6_10	C5_10	122.4(4)
C14_10	C6_10	C7_10	118.4(4)
C2_10	C7_10	C6_10	119.2(4)
C17_10	C7_10	C2_10	124.8(4)
C17_10	C7_10	C6_10	115.9(4)
C9_10	C8_10	C5_10	119.3(4)
C13_10	C8_10	C5_10	120.0(4)
C13_10	C8_10	C9_10	120.3(3)
C8_10	C9_10	H9_10	120.7
C8_10	C9_10	C10_10	118.5(4)
C10_10	C9_10	H9_10	120.7
C9_10	C10_10	H10_10	121.9
C11_10	C10_10	C9_10	116.2(4)
C11_10	C10_10	H10_10	121.9
C10_10	C11_10	H11_10	115.8
C12_10	C11_10	C10_10	128.3(5)
C12_10	C11_10	H11_10	115.8
C11_10	C12_10	H12_10	122.4
C11_10	C12_10	C13_10	115.2(4)
C13_10	C12_10	H12_10	122.4
C8_10	C13_10	C12_10	121.1(4)
C8_10	C13_10	H13_10	119.5
C12_10	C13_10	H13_10	119.5
C6_10	C14_10	H14_10	119.1
C15_10	C14_10	C6_10	121.9(4)
C15_10	C14_10	H14_10	119.1

Atom	Atom	Atom	Angle/°
C14_10	C15_10	C16_10	119.3(4)
C14_10	C15_10	C18_10	120.2(4)
C16_10	C15_10	C18_10	120.1(4)
C15_10	C16_10	H16_10	120.1
C17_10	C16_10	C15_10	119.7(5)
C17_10	C16_10	H16_10	120.1
C7_10	C17_10	H17_10	117.6
C16_10	C17_10	C7_10	124.8(5)
C16_10	C17_10	H17_10	117.6
C19_10	C18_10	C15_10	121.8(4)
C19_10	C18_10	C23_10	118.1(2)
C23_10	C18_10	C15_10	119.9(4)
C18_10	C19_10	H19_10	119.4
C20_10	C19_10	C18_10	121.2(3)
C20_10	C19_10	H19_10	119.4
C19_10	C20_10	H20_10	120.8
C21_10	C20_10	C19_10	118.4(3)
C21_10	C20_10	H20_10	120.8
C20_10	C21_10	H21_10	118.7
C20_10	C21_10	C22_10	122.7(4)
C22_10	C21_10	H21_10	118.7
C21_10	C22_10	H22_10	120.7
C21_10	C22_10	C23_10	118.6(3)
C23_10	C22_10	H22_10	120.7
C18_10	C23_10	H23_10	119.5
C22_10	C23_10	C18_10	120.9(3)
C22_10	C23_10	H23_10	119.5
O4_10	C24_10	C25_10	115.2(4)
C1_10	C24_10	O4_10	120.2(4)
C1_10	C24_10	C25_10	124.3(4)
C24_10	C25_10	H25_10	120.4
C26_10	C25_10	C24_10	119.2(4)
C26_10	C25_10	H25_10	120.4
C25_10	C26_10	C27_10	120.6(4)
C25_10	C26_10	C29_10	117.7(4)
C27_10	C26_10	C29_10	121.7(4)
C26_10	C27_10	C28_10	118.9(4)
C26_10	C27_10	C38_10	124.9(4)
C38_10	C27_10	C28_10	116.2(4)
C1_10	C28_10	C27_10	119.2(4)
C35_10	C28_10	C1_10	123.1(4)
C35_10	C28_10	C27_10	116.9(4)
C30_10	C29_10	C26_10	122.1(4)
C30_10	C29_10	C34_10	118.6(2)
C34_10	C29_10	C26_10	119.2(4)
C29_10	C30_10	H30_10	119.5
C31_10	C30_10	C29_10	121.0(2)
C31_10	C30_10	H30_10	119.5
C30_10	C31_10	H31_10	120.2
C30_10	C31_10	C32_10	119.6(2)
C32_10	C31_10	H31_10	120.2
C31_10	C32_10	H32_10	119.9
C33_10	C32_10	C31_10	120.1(2)
C33_10	C32_10	H32_10	119.9
C32_10	C33_10	H33_10	120.2
C32_10	C33_10	C34_10	119.6(2)

Atom	Atom	Atom	Angle/°
C34_10	C33_10	H33_10	120.2
C29_10	C34_10	H34_10	119.5
C33_10	C34_10	C29_10	121.1(2)
C33_10	C34_10	H34_10	119.5
C28_10	C35_10	H35_10	118.5
C36_10	C35_10	C28_10	122.9(5)
C36_10	C35_10	H35_10	118.5
C35_10	C36_10	H36_10	119.8
C35_10	C36_10	C37_10	120.4(5)
C37_10	C36_10	H36_10	119.8
C36_10	C37_10	C39_10	121.9(4)
C38_10	C37_10	C36_10	119.4(4)
C38_10	C37_10	C39_10	118.7(4)
C27_10	C38_10	H38_10	118.8
C37_10	C38_10	C27_10	122.4(5)
C37_10	C38_10	H38_10	118.8
C40_10	C39_10	C37_10	120.2(4)
C40_10	C39_10	C44_10	118.5(3)
C44_10	C39_10	C37_10	120.9(4)
C39_10	C40_10	H40_10	119.9
C39_10	C40_10	C41_10	120.1(4)
C41_10	C40_10	H40_10	119.9
C40_10	C41_10	H41_10	121.3
C42_10	C41_10	C40_10	117.5(4)
C42_10	C41_10	H41_10	121.3
C41_10	C42_10	H42_10	117.7
C43_10	C42_10	C41_10	124.5(5)
C43_10	C42_10	H42_10	117.7
C42_10	C43_10	H43_10	121.0
C42_10	C43_10	C44_10	118.0(4)
C44_10	C43_10	H43_10	121.0
C39_10	C44_10	H44_10	119.4
C43_10	C44_10	C39_10	121.3(4)
C43_10	C44_10	H44_10	119.4
C2S_11	O1S_11	Rh2	122.4(8)
C2S_11	O1S_11	C4S_11	116.9(7)
C4S_11	O1S_11	Rh2	120.1(7)
O1S_11	C2S_11	H2SA_11	108.4
O1S_11	C2S_11	H2SB_11	108.4
O1S_11	C2S_11	C3S_11	115.5(7)
H2SA_11	C2S_11	H2SB_11	107.5
C3S_11	C2S_11	H2SA_11	108.4
C3S_11	C2S_11	H2SB_11	108.4
C2S_11	C3S_11	H3SA_11	109.5
C2S_11	C3S_11	H3SB_11	109.5
C2S_11	C3S_11	H3SC_11	109.5
H3SA_11	C3S_11	H3SB_11	109.5
H3SA_11	C3S_11	H3SC_11	109.5
H3SB_11	C3S_11	H3SC_11	109.5
O1S_11	C4S_11	H4SA_11	109.0
O1S_11	C4S_11	H4SB_11	109.0
O1S_11	C4S_11	C5S_11	113.1(7)
H4SA_11	C4S_11	H4SB_11	107.8
C5S_11	C4S_11	H4SA_11	109.0
C5S_11	C4S_11	H4SB_11	109.0
C4S_11	C5S_11	H5SA_11	109.5

Atom	Atom	Atom	Angle/°
C4S_11	C5S_11	H5SB_11	109.5
C4S_11	C5S_11	H5SC_11	109.5
H5SA_11	C5S_11	H5SB_11	109.5
H5SA_11	C5S_11	H5SC_11	109.5
H5SB_11	C5S_11	H5SC_11	109.5
C2S_12	O1S_12	Rh4	123.8(6)
C2S_12	O1S_12	C4S_12	116.9(6)
C4S_12	O1S_12	Rh4	119.0(6)
O1S_12	C2S_12	H2SA_12	108.3
O1S_12	C2S_12	H2SB_12	108.3
O1S_12	C2S_12	C3S_12	115.7(7)
H2SA_12	C2S_12	H2SB_12	107.4
C3S_12	C2S_12	H2SA_12	108.3
C3S_12	C2S_12	H2SB_12	108.3
C2S_12	C3S_12	H3SA_12	109.5
C2S_12	C3S_12	H3SB_12	109.5
C2S_12	C3S_12	H3SC_12	109.5
H3SA_12	C3S_12	H3SB_12	109.5
H3SA_12	C3S_12	H3SC_12	109.5
H3SB_12	C3S_12	H3SC_12	109.5
O1S_12	C4S_12	H4SA_12	109.1
O1S_12	C4S_12	H4SB_12	109.1
O1S_12	C4S_12	C5S_12	112.4(6)
H4SA_12	C4S_12	H4SB_12	107.9
C5S_12	C4S_12	H4SA_12	109.1
C5S_12	C4S_12	H4SB_12	109.1
C4S_12	C5S_12	H5SA_12	109.5
C4S_12	C5S_12	H5SB_12	109.5
C4S_12	C5S_12	H5SC_12	109.5
H5SA_12	C5S_12	H5SB_12	109.5
H5SA_12	C5S_12	H5SC_12	109.5
H5SB_12	C5S_12	H5SC_12	109.5
O1_13	P1_13	O3_13	113.0(2)
O1_13	P1_13	O4_13	105.7(2)
O2_13	P1_13	O1_13	118.6(2)
O2_13	P1_13	O3_13	105.3(2)
O2_13	P1_13	O4_13	110.6(2)
O3_13	P1_13	O4_13	102.6(2)
P1_13	O1_13	Rh2	115.2(3)
P1_13	O2_13	Rh1	118.4(3)
C3_13	O3_13	P1_13	116.5(3)
C24_13	O4_13	P1_13	117.9(3)
C24_13	C1_13	C2_13	118.6(4)
C24_13	C1_13	C28_13	117.8(4)
C28_13	C1_13	C2_13	123.5(4)
C3_13	C2_13	C1_13	118.4(3)
C3_13	C2_13	C7_13	118.2(4)
C7_13	C2_13	C1_13	123.3(4)
O3_13	C3_13	C4_13	114.3(3)
C2_13	C3_13	O3_13	120.8(3)
C2_13	C3_13	C4_13	124.7(4)
C3_13	C4_13	H4_13	121.1
C5_13	C4_13	C3_13	117.7(4)
C5_13	C4_13	H4_13	121.1
C4_13	C5_13	C6_13	120.8(4)
C4_13	C5_13	C8_13	116.9(4)

Atom	Atom	Atom	Angle/°
C6_13	C5_13	C8_13	122.1(4)
C5_13	C6_13	C7_13	119.1(4)
C14_13	C6_13	C5_13	122.5(4)
C14_13	C6_13	C7_13	118.3(4)
C2_13	C7_13	C6_13	119.3(4)
C17_13	C7_13	C2_13	124.7(4)
C17_13	C7_13	C6_13	115.9(4)
C9_13	C8_13	C5_13	119.1(4)
C13_13	C8_13	C5_13	119.7(4)
C13_13	C8_13	C9_13	120.4(4)
C8_13	C9_13	H9_13	120.7
C8_13	C9_13	C10_13	118.5(4)
C10_13	C9_13	H9_13	120.7
C9_13	C10_13	H10_13	121.9
C11_13	C10_13	C9_13	116.1(4)
C11_13	C10_13	H10_13	121.9
C10_13	C11_13	H11_13	115.7
C12_13	C11_13	C10_13	128.5(6)
C12_13	C11_13	H11_13	115.7
C11_13	C12_13	H12_13	122.4
C11_13	C12_13	C13_13	115.2(4)
C13_13	C12_13	H12_13	122.4
C8_13	C13_13	C12_13	121.2(4)
C8_13	C13_13	H13_13	119.4
C12_13	C13_13	H13_13	119.4
C6_13	C14_13	H14_13	119.1
C15_13	C14_13	C6_13	121.7(4)
C15_13	C14_13	H14_13	119.1
C14_13	C15_13	C16_13	119.7(4)
C14_13	C15_13	C18_13	120.5(4)
C16_13	C15_13	C18_13	119.7(4)
C15_13	C16_13	H16_13	120.3
C17_13	C16_13	C15_13	119.4(5)
C17_13	C16_13	H16_13	120.3
C7_13	C17_13	H17_13	117.5
C16_13	C17_13	C7_13	124.9(5)
C16_13	C17_13	H17_13	117.5
C19_13	C18_13	C15_13	121.5(4)
C19_13	C18_13	C23_13	118.1(2)
C23_13	C18_13	C15_13	119.6(4)
C18_13	C19_13	H19_13	119.4
C20_13	C19_13	C18_13	121.1(3)
C20_13	C19_13	H19_13	119.4
C19_13	C20_13	H20_13	120.8
C21_13	C20_13	C19_13	118.4(3)
C21_13	C20_13	H20_13	120.8
C20_13	C21_13	H21_13	118.5
C20_13	C21_13	C22_13	123.0(4)
C22_13	C21_13	H21_13	118.5
C21_13	C22_13	H22_13	120.8
C21_13	C22_13	C23_13	118.4(3)
C23_13	C22_13	H22_13	120.8
C18_13	C23_13	H23_13	119.5
C22_13	C23_13	C18_13	121.0(3)
C22_13	C23_13	H23_13	119.5
O4_13	C24_13	C25_13	115.4(4)
C1_13	C24_13	O4_13	120.1(3)
C1_13	C24_13	C25_13	124.1(4)
C24_13	C25_13	H25_13	120.3
C26_13	C25_13	C24_13	119.4(4)
C26_13	C25_13	H25_13	120.3
C25_13	C26_13	C27_13	120.8(4)
C25_13	C26_13	C29_13	117.5(4)
C27_13	C26_13	C29_13	121.7(4)
C26_13	C27_13	C28_13	118.7(4)
C26_13	C27_13	C38_13	124.5(4)
C38_13	C27_13	C28_13	116.8(4)
C1_13	C28_13	C27_13	119.2(4)
C35_13	C28_13	C1_13	124.2(4)
C35_13	C28_13	C27_13	116.6(4)
C30_13	C29_13	C26_13	122.1(4)
C30_13	C29_13	C34_13	118.6(2)
C34_13	C29_13	C26_13	118.7(4)
C29_13	C30_13	H30_13	119.5
C29_13	C30_13	C31_13	121.0(2)
C31_13	C30_13	H30_13	119.5
C30_13	C31_13	H31_13	120.2
C32_13	C31_13	C30_13	119.6(2)
C32_13	C31_13	H31_13	120.2
C31_13	C32_13	H32_13	119.9
C31_13	C32_13	C33_13	120.1(2)
C33_13	C32_13	H32_13	120.0
C32_13	C33_13	H33_13	120.3
C32_13	C33_13	C34_13	119.5(2)
C34_13	C33_13	H33_13	120.3
C29_13	C34_13	H34_13	119.5
C33_13	C34_13	C29_13	121.0(2)
C33_13	C34_13	H34_13	119.5
C28_13	C35_13	H35_13	118.5
C36_13	C35_13	C28_13	123.1(4)
C36_13	C35_13	H35_13	118.5
C35_13	C36_13	H36_13	119.6
C35_13	C36_13	C37_13	120.9(4)
C37_13	C36_13	H36_13	119.6
C36_13	C37_13	C39_13	121.8(4)
C38_13	C37_13	C36_13	119.2(4)
C38_13	C37_13	C39_13	118.9(4)
C27_13	C38_13	H38_13	118.4
C37_13	C38_13	C27_13	123.2(4)
C37_13	C38_13	H38_13	118.4
C40_13	C39_13	C37_13	120.4(4)
C40_13	C39_13	C44_13	118.4(3)
C44_13	C39_13	C37_13	121.1(4)
C39_13	C40_13	H40_13	119.9
C39_13	C40_13	C41_13	120.2(4)
C41_13	C40_13	H40_13	119.9
C40_13	C41_13	H41_13	121.2
C42_13	C41_13	C40_13	117.6(4)
C42_13	C41_13	H41_13	121.2
C41_13	C42_13	H42_13	117.8
C43_13	C42_13	C41_13	124.4(5)
C43_13	C42_13	H42_13	117.8

Atom	Atom	Atom	Angle/°
C42_13	C43_13	H43_13	121.0
C42_13	C43_13	C44_13	118.0(4)
C44_13	C43_13	H43_13	121.0
C39_13	C44_13	H44_13	119.3
C43_13	C44_13	C39_13	121.4(4)
C43_13	C44_13	H44_13	119.3
O1_14	P1_14	O3_14	113.4(3)
O1_14	P1_14	O4_14	105.3(2)
O2_14	P1_14	O1_14	118.5(2)
O2_14	P1_14	O3_14	104.8(2)
O2_14	P1_14	O4_14	110.7(2)
O3_14	P1_14	O4_14	103.0(2)
P1_14	O1_14	Rh2	114.3(2)
P1_14	O2_14	Rh1	119.9(3)
C3_14	O3_14	P1_14	117.6(3)
C24_14	O4_14	P1_14	117.2(3)
C24_14	C1_14	C2_14	118.7(4)
C24_14	C1_14	C28_14	117.4(4)
C28_14	C1_14	C2_14	123.9(4)
C3_14	C2_14	C1_14	118.3(4)
C3_14	C2_14	C7_14	118.2(4)
C7_14	C2_14	C1_14	123.5(4)
O3_14	C3_14	C4_14	113.9(4)
C2_14	C3_14	O3_14	121.2(4)
C2_14	C3_14	C4_14	124.7(4)
C3_14	C4_14	H4_14	121.1
C5_14	C4_14	C3_14	117.7(4)
C5_14	C4_14	H4_14	121.1
C4_14	C5_14	C6_14	120.8(4)
C4_14	C5_14	C8_14	117.5(4)
C6_14	C5_14	C8_14	121.8(4)
C5_14	C6_14	C7_14	119.2(4)
C14_14	C6_14	C5_14	121.9(4)
C14_14	C6_14	C7_14	118.6(4)
C2_14	C7_14	C6_14	119.2(4)
C17_14	C7_14	C2_14	125.0(4)
C17_14	C7_14	C6_14	115.7(4)
C9_14	C8_14	C5_14	119.4(4)
C13_14	C8_14	C5_14	120.3(4)
C13_14	C8_14	C9_14	120.2(3)
C8_14	C9_14	H9_14	120.6
C8_14	C9_14	C10_14	118.8(4)
C10_14	C9_14	H9_14	120.6
C9_14	C10_14	H10_14	122.0
C11_14	C10_14	C9_14	116.0(4)
C11_14	C10_14	H10_14	122.0
C10_14	C11_14	H11_14	115.8
C12_14	C11_14	C10_14	128.4(5)
C12_14	C11_14	H11_14	115.8
C11_14	C12_14	H12_14	122.4
C11_14	C12_14	C13_14	115.2(4)
C13_14	C12_14	H12_14	122.4
C8_14	C13_14	C12_14	121.3(4)
C8_14	C13_14	H13_14	119.3
C12_14	C13_14	H13_14	119.3
C6_14	C14_14	H14_14	119.2

Atom	Atom	Atom	Angle/°
C15_14	C14_14	C6_14	121.6(4)
C15_14	C14_14	H14_14	119.2
C14_14	C15_14	C16_14	119.5(5)
C14_14	C15_14	C18_14	119.8(4)
C16_14	C15_14	C18_14	120.1(4)
C15_14	C16_14	H16_14	120.2
C17_14	C16_14	C15_14	119.6(5)
C17_14	C16_14	H16_14	120.2
C7_14	C17_14	H17_14	117.6
C16_14	C17_14	C7_14	124.9(5)
C16_14	C17_14	H17_14	117.6
C19_14	C18_14	C15_14	121.3(4)
C19_14	C18_14	C23_14	118.1(2)
C23_14	C18_14	C15_14	119.5(4)
C18_14	C19_14	H19_14	119.4
C20_14	C19_14	C18_14	121.2(3)
C20_14	C19_14	H19_14	119.4
C19_14	C20_14	H20_14	120.8
C21_14	C20_14	C19_14	118.4(3)
C21_14	C20_14	H20_14	120.8
C20_14	C21_14	H21_14	118.6
C20_14	C21_14	C22_14	122.8(4)
C22_14	C21_14	H21_14	118.6
C21_14	C22_14	H22_14	120.7
C21_14	C22_14	C23_14	118.5(3)
C23_14	C22_14	H22_14	120.7
C18_14	C23_14	H23_14	119.5
C22_14	C23_14	C18_14	120.9(3)
C22_14	C23_14	H23_14	119.5
O4_14	C24_14	C25_14	114.9(4)
C1_14	C24_14	O4_14	120.0(4)
C1_14	C24_14	C25_14	124.7(4)
C24_14	C25_14	H25_14	120.5
C26_14	C25_14	C24_14	119.0(4)
C26_14	C25_14	H25_14	120.5
C25_14	C26_14	C27_14	120.7(4)
C25_14	C26_14	C29_14	117.5(4)
C27_14	C26_14	C29_14	121.6(4)
C26_14	C27_14	C28_14	118.9(4)
C26_14	C27_14	C38_14	124.5(4)
C38_14	C27_14	C28_14	116.6(4)
C1_14	C28_14	C27_14	119.2(4)
C35_14	C28_14	C1_14	124.0(4)
C35_14	C28_14	C27_14	116.8(4)
C30_14	C29_14	C26_14	122.3(4)
C30_14	C29_14	C34_14	118.7(2)
C34_14	C29_14	C26_14	118.9(4)
C29_14	C30_14	H30_14	119.6
C29_14	C30_14	C31_14	120.8(2)
C31_14	C30_14	H30_14	119.6
C30_14	C31_14	H31_14	120.2
C30_14	C31_14	C32_14	119.7(2)
C32_14	C31_14	H31_14	120.2
C31_14	C32_14	H32_14	120.0
C33_14	C32_14	C31_14	120.1(2)
C33_14	C32_14	H32_14	120.0

Atom	Atom	Atom	Angle/°
C32_14	C33_14	H33_14	120.3
C34_14	C33_14	C32_14	119.5(2)
C34_14	C33_14	H33_14	120.3
C29_14	C34_14	H34_14	119.5
C33_14	C34_14	C29_14	121.1(2)
C33_14	C34_14	H34_14	119.5
C28_14	C35_14	H35_14	118.5
C36_14	C35_14	C28_14	123.0(5)
C36_14	C35_14	H35_14	118.5
C35_14	C36_14	H36_14	119.7
C35_14	C36_14	C37_14	120.7(5)
C37_14	C36_14	H36_14	119.7
C36_14	C37_14	C39_14	121.9(4)
C38_14	C37_14	C36_14	119.3(4)
C38_14	C37_14	C39_14	118.8(4)
C27_14	C38_14	H38_14	118.4
C37_14	C38_14	C27_14	123.1(4)
C37_14	C38_14	H38_14	118.4
C40_14	C39_14	C37_14	120.4(4)
C40_14	C39_14	C44_14	118.4(3)
C44_14	C39_14	C37_14	121.1(4)
C39_14	C40_14	H40_14	119.9
C39_14	C40_14	C41_14	120.2(4)
C41_14	C40_14	H40_14	119.9
C40_14	C41_14	H41_14	121.2
C42_14	C41_14	C40_14	117.6(4)
C42_14	C41_14	H41_14	121.2
C41_14	C42_14	H42_14	117.8
C43_14	C42_14	C41_14	124.4(5)
C43_14	C42_14	H42_14	117.8
C42_14	C43_14	H43_14	121.0
C42_14	C43_14	C44_14	118.0(4)
C44_14	C43_14	H43_14	121.0
C39_14	C44_14	H44_14	119.3
C43_14	C44_14	C39_14	121.4(4)
C43_14	C44_14	H44_14	119.3
O1_4	P1_4	O3_4	112.1(2)
O1_4	P1_4	O4_4	105.3(2)
O2_4	P1_4	O1_4	118.0(3)
O2_4	P1_4	O3_4	105.8(2)
O2_4	P1_4	O4_4	111.8(2)
O3_4	P1_4	O4_4	102.9(2)
P1_4	O1_4	Rh2	115.7(3)
P1_4	O2_4	Rh1	117.5(3)
C3_4	O3_4	P1_4	115.6(3)
C24_4	O4_4	P1_4	119.6(3)
C24_4	C1_4	C2_4	118.7(4)
C24_4	C1_4	C28_4	118.0(4)
C28_4	C1_4	C2_4	122.9(4)
C3_4	C2_4	C1_4	117.6(4)
C3_4	C2_4	C7_4	118.6(4)
C7_4	C2_4	C1_4	123.8(4)
O3_4	C3_4	C4_4	115.5(4)
C2_4	C3_4	O3_4	119.8(4)
C2_4	C3_4	C4_4	124.6(4)
C3_4	C4_4	H4_4	121.2

Atom	Atom	Atom	Angle/°
C5_4	C4_4	C3_4	117.6(4)
C5_4	C4_4	H4_4	121.2
C4_4	C5_4	C6_4	121.0(4)
C4_4	C5_4	C8_4	117.2(4)
C6_4	C5_4	C8_4	121.4(4)
C5_4	C6_4	C7_4	119.0(4)
C14_4	C6_4	C5_4	122.5(4)
C14_4	C6_4	C7_4	118.4(4)
C2_4	C7_4	C6_4	119.2(4)
C17_4	C7_4	C2_4	124.8(4)
C17_4	C7_4	C6_4	116.0(4)
C9_4	C8_4	C5_4	119.2(4)
C13_4	C8_4	C5_4	120.0(4)
C13_4	C8_4	C9_4	120.6(4)
C8_4	C9_4	H9_4	120.8
C8_4	C9_4	C10_4	118.5(4)
C10_4	C9_4	H9_4	120.8
C9_4	C10_4	H10_4	122.0
C11_4	C10_4	C9_4	116.0(4)
C11_4	C10_4	H10_4	122.0
C10_4	C11_4	H11_4	115.7
C12_4	C11_4	C10_4	128.6(6)
C12_4	C11_4	H11_4	115.7
C11_4	C12_4	H12_4	122.4
C11_4	C12_4	C13_4	115.3(4)
C13_4	C12_4	H12_4	122.4
C8_4	C13_4	C12_4	121.0(4)
C8_4	C13_4	H13_4	119.5
C12_4	C13_4	H13_4	119.5
C6_4	C14_4	H14_4	119.2
C15_4	C14_4	C6_4	121.6(4)
C15_4	C14_4	H14_4	119.2
C14_4	C15_4	C18_4	120.6(4)
C16_4	C15_4	C14_4	119.7(5)
C16_4	C15_4	C18_4	119.8(4)
C15_4	C16_4	H16_4	120.2
C17_4	C16_4	C15_4	119.6(5)
C17_4	C16_4	H16_4	120.2
C7_4	C17_4	H17_4	117.6
C16_4	C17_4	C7_4	124.8(5)
C16_4	C17_4	H17_4	117.6
C19_4	C18_4	C15_4	121.9(4)
C19_4	C18_4	C23_4	118.2(2)
C23_4	C18_4	C15_4	119.7(4)
C18_4	C19_4	H19_4	119.5
C20_4	C19_4	C18_4	121.0(3)
C20_4	C19_4	H19_4	119.5
C19_4	C20_4	H20_4	120.7
C21_4	C20_4	C19_4	118.6(3)
C21_4	C20_4	H20_4	120.7
C20_4	C21_4	H21_4	118.6
C20_4	C21_4	C22_4	122.8(4)
C22_4	C21_4	H21_4	118.6
C21_4	C22_4	H22_4	120.8
C21_4	C22_4	C23_4	118.4(3)
C23_4	C22_4	H22_4	120.8

Atom	Atom	Atom	Angle/°
C18_4	C23_4	H23_4	119.5
C22_4	C23_4	C18_4	121.1(3)
C22_4	C23_4	H23_4	119.5
O4_4	C24_4	C25_4	115.4(4)
C1_4	C24_4	O4_4	120.6(4)
C1_4	C24_4	C25_4	124.0(4)
C24_4	C25_4	H25_4	120.4
C26_4	C25_4	C24_4	119.3(4)
C26_4	C25_4	H25_4	120.4
C25_4	C26_4	C27_4	120.9(4)
C25_4	C26_4	C29_4	117.6(4)
C27_4	C26_4	C29_4	121.1(4)
C26_4	C27_4	C28_4	118.5(4)
C26_4	C27_4	C38_4	124.3(4)
C38_4	C27_4	C28_4	116.7(4)
C1_4	C28_4	C27_4	119.2(4)
C35_4	C28_4	C1_4	123.0(4)
C35_4	C28_4	C27_4	116.7(4)
C30_4	C29_4	C26_4	122.2(4)
C30_4	C29_4	C34_4	118.7(2)
C34_4	C29_4	C26_4	118.9(4)
C29_4	C30_4	H30_4	119.5
C29_4	C30_4	C31_4	121.0(2)
C31_4	C30_4	H30_4	119.5
C30_4	C31_4	H31_4	120.2
C32_4	C31_4	C30_4	119.6(2)
C32_4	C31_4	H31_4	120.2
C31_4	C32_4	H32_4	119.9
C31_4	C32_4	C33_4	120.1(2)
C33_4	C32_4	H32_4	119.9
C32_4	C33_4	H33_4	120.2
C34_4	C33_4	C32_4	119.6(2)
C34_4	C33_4	H33_4	120.2
C29_4	C34_4	H34_4	119.5

Atom	Atom	Atom	Angle/°
C33_4	C34_4	C29_4	121.0(2)
C33_4	C34_4	H34_4	119.5
C28_4	C35_4	H35_4	118.5
C36_4	C35_4	C28_4	123.0(5)
C36_4	C35_4	H35_4	118.5
C35_4	C36_4	H36_4	119.7
C35_4	C36_4	C37_4	120.7(5)
C37_4	C36_4	H36_4	119.7
C36_4	C37_4	C39_4	121.6(4)
C38_4	C37_4	C36_4	119.1(4)
C38_4	C37_4	C39_4	118.9(4)
C27_4	C38_4	H38_4	118.4
C37_4	C38_4	C27_4	123.2(4)
C37_4	C38_4	H38_4	118.4
C40_4	C39_4	C37_4	120.4(4)
C40_4	C39_4	C44_4	118.5(3)
C44_4	C39_4	C37_4	121.1(4)
C39_4	C40_4	H40_4	119.9
C39_4	C40_4	C41_4	120.2(4)
C41_4	C40_4	H40_4	119.9
C40_4	C41_4	H41_4	121.3
C42_4	C41_4	C40_4	117.5(4)
C42_4	C41_4	H41_4	121.3
C41_4	C42_4	H42_4	117.8
C43_4	C42_4	C41_4	124.5(5)
C43_4	C42_4	H42_4	117.8
C42_4	C43_4	H43_4	121.0
C42_4	C43_4	C44_4	118.0(4)
C44_4	C43_4	H43_4	121.0
C39_4	C44_4	H44_4	119.3
C43_4	C44_4	C39_4	121.3(4)
C43_4	C44_4	H44_4	119.3

————
[1]-1-x,+y,3/2-z

Table 10.1.6: Torsion Angles in ° for S-**6a** (ZC-01-51).

Atom	Atom	Atom	Atom	Angle/°
Rh1	O1S_7	C2S_7	C3S_7	-65(2)
Rh1	O1S_7	C4S_7	C5S_7	-84.8(15)
Rh2	O1S_11	C2S_11	C3S_11	93.8(16)
Rh2	O1S_11	C4S_11	C5S_11	-146(3)
Rh3	O1S_8	C2S_8	C3S_8	-65(2)
Rh3	O1S_8	C4S_8	C5S_8	-117.6(15)
Rh4	O1S_12	C2S_12	C3S_12	-100.8(15)
Rh4	O1S_12	C4S_12	C5S_12	-103.4(9)
P1_1	O3_1	C3_1	C2_1	-76.0(5)
P1_1	O3_1	C3_1	C4_1	102.9(4)
P1_1	O4_1	C24_1	C1_1	-70.5(6)
P1_1	O4_1	C24_1	C25_1	101.4(6)
O1_1	P1_1	O2_1	Rh3	5.5(4)
O1_1	P1_1	O3_1	C3_1	-70.8(4)
O1_1	P1_1	O4_1	C24_1	166.1(4)
O2_1	P1_1	O1_1	Rh4	22.3(4)

Atom	Atom	Atom	Atom	Angle/°
O2_1	P1_1	O3_1	C3_1	158.4(4)
O2_1	P1_1	O4_1	C24_1	-64.4(4)
O3_1	P1_1	O1_1	Rh4	-101.5(3)
O3_1	P1_1	O2_1	Rh3	132.6(3)
O3_1	P1_1	O4_1	C24_1	48.1(4)
O3_1	C3_1	C4_1	C5_1	-179.3(7)
O4_1	P1_1	O1_1	Rh4	146.9(3)
O4_1	P1_1	O2_1	Rh3	-116.5(3)
O4_1	P1_1	O3_1	C3_1	42.2(4)
O4_1	C24_1	C25_1	C26_1	-173.6(7)
C1_1	C2_1	C3_1	O3_1	2.5(7)
C1_1	C2_1	C3_1	C4_1	-176.3(6)
C1_1	C2_1	C7_1	C6_1	177.3(5)
C1_1	C2_1	C7_1	C17_1	-2.8(9)
C1_1	C24_1	C25_1	C26_1	-2.1(11)
C1_1	C28_1	C35_1	C36_1	178.8(8)
C2_1	C1_1	C24_1	O4_1	-8.7(6)
C2_1	C1_1	C24_1	C25_1	-179.9(7)
C2_1	C1_1	C28_1	C27_1	-179.1(7)
C2_1	C1_1	C28_1	C35_1	6.3(8)
C2_1	C3_1	C4_1	C5_1	-0.5(6)
C2_1	C7_1	C17_1	C16_1	179.4(8)
C3_1	C2_1	C7_1	C6_1	2.6(7)
C3_1	C2_1	C7_1	C17_1	-177.5(6)
C3_1	C4_1	C5_1	C6_1	0.9(11)
C3_1	C4_1	C5_1	C8_1	171.6(5)
C4_1	C5_1	C6_1	C7_1	0.4(11)
C4_1	C5_1	C6_1	C14_1	178.2(7)
C4_1	C5_1	C8_1	C9_1	-95.8(8)
C4_1	C5_1	C8_1	C13_1	87.9(9)
C5_1	C6_1	C7_1	C2_1	-2.2(9)
C5_1	C6_1	C7_1	C17_1	177.9(8)
C5_1	C6_1	C14_1	C15_1	-179.5(9)
C5_1	C8_1	C9_1	C10_1	-176.0(6)
C5_1	C8_1	C13_1	C12_1	175.1(8)
C6_1	C5_1	C8_1	C9_1	74.9(9)
C6_1	C5_1	C8_1	C13_1	-101.4(9)
C6_1	C7_1	C17_1	C16_1	-0.6(6)
C6_1	C14_1	C15_1	C16_1	3.9(10)
C6_1	C14_1	C15_1	C18_1	178.4(8)
C7_1	C2_1	C3_1	O3_1	177.5(6)
C7_1	C2_1	C3_1	C4_1	-1.3(5)
C7_1	C6_1	C14_1	C15_1	-1.7(5)
C8_1	C5_1	C6_1	C7_1	-169.9(5)
C8_1	C5_1	C6_1	C14_1	7.9(10)
C8_1	C9_1	C10_1	C11_1	-0.4(5)
C9_1	C8_1	C13_1	C12_1	-1.2(10)
C9_1	C10_1	C11_1	C12_1	1.9(13)
C10_1	C11_1	C12_1	C13_1	-2.7(16)
C11_1	C12_1	C13_1	C8_1	2.3(13)
C13_1	C8_1	C9_1	C10_1	0.3(5)
C14_1	C6_1	C7_1	C2_1	180.0(7)
C14_1	C6_1	C7_1	C17_1	0.0(3)
C14_1	C15_1	C16_1	C17_1	-4.4(12)
C14_1	C15_1	C18_1	C19_1	-21.6(12)
C14_1	C15_1	C18_1	C23_1	154.7(9)

Atom	Atom	Atom	Atom	Angle/°
C15_1	C16_1	C17_1	C7_1	2.8(10)
C15_1	C18_1	C19_1	C20_1	177.5(9)
C15_1	C18_1	C23_1	C22_1	179.5(12)
C16_1	C15_1	C18_1	C19_1	152.9(8)
C16_1	C15_1	C18_1	C23_1	-30.8(13)
C18_1	C15_1	C16_1	C17_1	-178.9(8)
C18_1	C19_1	C20_1	C21_1	-0.9(7)
C19_1	C18_1	C23_1	C22_1	-4.1(15)
C19_1	C20_1	C21_1	C22_1	3.5(16)
C20_1	C21_1	C22_1	C23_1	-6(2)
C21_1	C22_1	C23_1	C18_1	6.6(19)
C23_1	C18_1	C19_1	C20_1	1.2(7)
C24_1	C1_1	C2_1	C3_1	55.8(7)
C24_1	C1_1	C2_1	C7_1	-118.9(6)
C24_1	C1_1	C28_1	C27_1	-0.2(6)
C24_1	C1_1	C28_1	C35_1	-174.8(8)
C24_1	C25_1	C26_1	C27_1	2.0(13)
C24_1	C25_1	C26_1	C29_1	-168.5(7)
C25_1	C26_1	C27_1	C28_1	-1.1(13)
C25_1	C26_1	C27_1	C38_1	-176.6(10)
C25_1	C26_1	C29_1	C30_1	-115.4(10)
C25_1	C26_1	C29_1	C34_1	66.2(12)
C26_1	C27_1	C28_1	C1_1	0.1(10)
C26_1	C27_1	C28_1	C35_1	175.1(8)
C26_1	C27_1	C38_1	C37_1	-177.0(10)
C26_1	C29_1	C30_1	C31_1	-178.8(7)
C26_1	C29_1	C34_1	C33_1	179.2(10)
C27_1	C26_1	C29_1	C30_1	74.1(11)
C27_1	C26_1	C29_1	C34_1	-104.3(11)
C27_1	C28_1	C35_1	C36_1	4.0(13)
C28_1	C1_1	C2_1	C3_1	-125.3(5)
C28_1	C1_1	C2_1	C7_1	60.0(8)
C28_1	C1_1	C24_1	O4_1	172.3(6)
C28_1	C1_1	C24_1	C25_1	1.2(6)
C28_1	C27_1	C38_1	C37_1	7.4(15)
C28_1	C35_1	C36_1	C37_1	3.3(16)
C29_1	C26_1	C27_1	C28_1	169.1(7)
C29_1	C26_1	C27_1	C38_1	-6.5(13)
C29_1	C30_1	C31_1	C32_1	-0.3(6)
C30_1	C29_1	C34_1	C33_1	0.8(12)
C30_1	C31_1	C32_1	C33_1	0.6(12)
C31_1	C32_1	C33_1	C34_1	-0.3(16)
C32_1	C33_1	C34_1	C29_1	-0.4(16)
C34_1	C29_1	C30_1	C31_1	-0.4(5)
C35_1	C36_1	C37_1	C38_1	-5.3(17)
C35_1	C36_1	C37_1	C39_1	176.1(9)
C36_1	C37_1	C38_1	C27_1	-0.2(17)
C36_1	C37_1	C39_1	C40_1	31.6(12)
C36_1	C37_1	C39_1	C44_1	-149.3(9)
C37_1	C39_1	C40_1	C41_1	179.1(7)
C37_1	C39_1	C44_1	C43_1	-179.1(7)
C38_1	C27_1	C28_1	C1_1	176.1(8)
C38_1	C27_1	C28_1	C35_1	-8.9(11)
C38_1	C37_1	C39_1	C40_1	-147.0(8)
C38_1	C37_1	C39_1	C44_1	32.1(12)
C39_1	C37_1	C38_1	C27_1	178.4(9)

Atom	Atom	Atom	Atom	Angle/°
C39_1	C40_1	C41_1	C42_1	0.00(8)
C40_1	C39_1	C44_1	C43_1	0.02(18)
C40_1	C41_1	C42_1	C43_1	0.00(19)
C41_1	C42_1	C43_1	C44_1	0.0(3)
C42_1	C43_1	C44_1	C39_1	0.0(2)
C44_1	C39_1	C40_1	C41_1	-0.01(8)
P1_2	O3_2	C3_2	C2_2	-74.8(6)
P1_2	O3_2	C3_2	C4_2	101.8(5)
P1_2	O4_2	C24_2	C1_2	-70.9(7)
P1_2	O4_2	C24_2	C25_2	107.7(7)
O1_2	P1_2	O2_2	Rh5	-11.0(6)
O1_2	P1_2	O3_2	C3_2	-66.6(5)
O1_2	P1_2	O4_2	C24_2	162.5(4)
O2_2	P1_2	O1_2	Rh5[1]	-6.7(6)
O2_2	P1_2	O3_2	C3_2	162.4(4)
O2_2	P1_2	O4_2	C24_2	-68.3(5)
O3_2	P1_2	O1_2	Rh5[1]	-130.8(4)
O3_2	P1_2	O2_2	Rh5	116.4(5)
O3_2	P1_2	O4_2	C24_2	44.4(5)
O3_2	C3_2	C4_2	C5_2	-175.7(9)
O4_2	P1_2	O1_2	Rh5[1]	117.5(4)
O4_2	P1_2	O2_2	Rh5	-132.4(4)
O4_2	P1_2	O3_2	C3_2	46.1(4)
O4_2	C24_2	C25_2	C26_2	-175.8(9)
C1_2	C2_2	C3_2	O3_2	-2.1(8)
C1_2	C2_2	C3_2	C4_2	-178.4(7)
C1_2	C2_2	C7_2	C6_2	-179.8(7)
C1_2	C2_2	C7_2	C17_2	9.0(11)
C1_2	C24_2	C25_2	C26_2	2.8(12)
C1_2	C28_2	C35_2	C36_2	168.9(13)
C2_2	C1_2	C24_2	O4_2	-6.4(8)
C2_2	C1_2	C24_2	C25_2	175.1(8)
C2_2	C1_2	C28_2	C27_2	-174.9(8)
C2_2	C1_2	C28_2	C35_2	9.9(12)
C2_2	C3_2	C4_2	C5_2	0.8(7)
C2_2	C7_2	C17_2	C16_2	171.9(11)
C3_2	C2_2	C7_2	C6_2	3.1(9)
C3_2	C2_2	C7_2	C17_2	-168.1(8)
C3_2	C4_2	C5_2	C6_2	-2.4(13)
C3_2	C4_2	C5_2	C8_2	175.3(7)
C4_2	C5_2	C6_2	C7_2	4.4(14)
C4_2	C5_2	C6_2	C14_2	171.3(9)
C4_2	C5_2	C8_2	C9_2	-66.8(10)
C4_2	C5_2	C8_2	C13_2	117.8(10)
C5_2	C6_2	C7_2	C2_2	-4.7(12)
C5_2	C6_2	C7_2	C17_2	167.3(10)
C5_2	C6_2	C14_2	C15_2	-166.1(11)
C5_2	C8_2	C9_2	C10_2	-174.4(7)
C5_2	C8_2	C13_2	C12_2	173.8(9)
C6_2	C5_2	C8_2	C9_2	110.8(10)
C6_2	C5_2	C8_2	C13_2	-64.5(12)
C6_2	C7_2	C17_2	C16_2	0.5(6)
C6_2	C14_2	C15_2	C16_2	-1.9(11)
C6_2	C14_2	C15_2	C18_2	162.1(9)
C7_2	C2_2	C3_2	O3_2	175.2(7)
C7_2	C2_2	C3_2	C4_2	-1.2(6)

Atom	Atom	Atom	Atom	Angle/°
C7_2	C6_2	C14_2	C15_2	0.9(6)
C8_2	C5_2	C6_2	C7_2	-173.2(8)
C8_2	C5_2	C6_2	C14_2	-6.2(13)
C8_2	C9_2	C10_2	C11_2	1.2(7)
C9_2	C8_2	C13_2	C12_2	-1.5(13)
C9_2	C10_2	C11_2	C12_2	-3.4(16)
C10_2	C11_2	C12_2	C13_2	3(2)
C11_2	C12_2	C13_2	C8_2	-0.3(17)
C13_2	C8_2	C9_2	C10_2	0.9(7)
C14_2	C6_2	C7_2	C2_2	-172.1(9)
C14_2	C6_2	C7_2	C17_2	-0.2(3)
C14_2	C15_2	C16_2	C17_2	2.2(13)
C14_2	C15_2	C18_2	C19_2	133.6(10)
C14_2	C15_2	C18_2	C23_2	-37.5(15)
C15_2	C16_2	C17_2	C7_2	-1.5(12)
C15_2	C18_2	C19_2	C20_2	-171.1(11)
C15_2	C18_2	C23_2	C22_2	171.6(14)
C16_2	C15_2	C18_2	C19_2	-62.4(13)
C16_2	C15_2	C18_2	C23_2	126.5(14)
C18_2	C15_2	C16_2	C17_2	-161.8(10)
C18_2	C19_2	C20_2	C21_2	0.5(8)
C19_2	C18_2	C23_2	C22_2	0.3(16)
C19_2	C20_2	C21_2	C22_2	-1.5(17)
C20_2	C21_2	C22_2	C23_2	2(2)
C21_2	C22_2	C23_2	C18_2	-1(2)
C23_2	C18_2	C19_2	C20_2	0.0(7)
C24_2	C1_2	C2_2	C3_2	57.7(8)
C24_2	C1_2	C2_2	C7_2	-119.4(8)
C24_2	C1_2	C28_2	C27_2	0.7(6)
C24_2	C1_2	C28_2	C35_2	-174.5(11)
C24_2	C25_2	C26_2	C27_2	-4.7(15)
C24_2	C25_2	C26_2	C29_2	175.2(7)
C25_2	C26_2	C27_2	C28_2	4.7(15)
C25_2	C26_2	C27_2	C38_2	174.8(12)
C25_2	C26_2	C29_2	C30_2	-88.4(12)
C25_2	C26_2	C29_2	C34_2	94.8(13)
C26_2	C27_2	C28_2	C1_2	-2.6(11)
C26_2	C27_2	C28_2	C35_2	172.9(11)
C26_2	C27_2	C38_2	C37_2	-165.7(14)
C26_2	C29_2	C30_2	C31_2	-174.7(8)
C26_2	C29_2	C34_2	C33_2	169.9(11)
C27_2	C26_2	C29_2	C30_2	91.5(13)
C27_2	C26_2	C29_2	C34_2	-85.3(13)
C27_2	C28_2	C35_2	C36_2	-6(2)
C28_2	C1_2	C2_2	C3_2	-126.8(6)
C28_2	C1_2	C2_2	C7_2	56.2(9)
C28_2	C1_2	C24_2	O4_2	177.8(7)
C28_2	C1_2	C24_2	C25_2	-0.8(6)
C28_2	C27_2	C38_2	C37_2	4.5(19)
C28_2	C35_2	C36_2	C37_2	4(3)
C29_2	C26_2	C27_2	C28_2	-175.3(8)
C29_2	C26_2	C27_2	C38_2	-5.2(15)
C29_2	C30_2	C31_2	C32_2	-0.9(8)
C30_2	C29_2	C34_2	C33_2	-7.0(15)
C30_2	C31_2	C32_2	C33_2	4.6(16)
C31_2	C32_2	C33_2	C34_2	-9(2)

Atom	Atom	Atom	Atom	Angle/˚
C32_2	C33_2	C34_2	C29_2	11(2)
C34_2	C29_2	C30_2	C31_2	2.1(7)
C35_2	C36_2	C37_2	C38_2	3(3)
C35_2	C36_2	C37_2	C39_2	179.4(14)
C36_2	C37_2	C38_2	C27_2	-7(2)
C36_2	C37_2	C39_2	C40_2	51.6(17)
C36_2	C37_2	C39_2	C44_2	-131.4(15)
C37_2	C39_2	C40_2	C41_2	177.1(8)
C37_2	C39_2	C44_2	C43_2	-177.1(8)
C38_2	C27_2	C28_2	C1_2	-173.5(10)
C38_2	C27_2	C28_2	C35_2	2.0(14)
C38_2	C37_2	C39_2	C40_2	-131.6(14)
C38_2	C37_2	C39_2	C44_2	45.4(16)
C39_2	C37_2	C38_2	C27_2	176.0(11)
C39_2	C40_2	C41_2	C42_2	0.00(8)
C40_2	C39_2	C44_2	C43_2	0.01(18)
C40_2	C41_2	C42_2	C43_2	-0.01(19)
C41_2	C42_2	C43_2	C44_2	0.0(3)
C42_2	C43_2	C44_2	C39_2	0.0(2)
C44_2	C39_2	C40_2	C41_2	0.00(8)
P1_3	O3_3	C3_3	C2_3	-77.8(5)
P1_3	O3_3	C3_3	C4_3	99.7(4)
P1_3	O4_3	C24_3	C1_3	-69.1(6)
P1_3	O4_3	C24_3	C25_3	108.9(6)
O1_3	P1_3	O2_3	Rh3	3.5(4)
O1_3	P1_3	O3_3	C3_3	-66.8(4)
O1_3	P1_3	O4_3	C24_3	162.5(4)
O2_3	P1_3	O1_3	Rh4	26.3(4)
O2_3	P1_3	O3_3	C3_3	162.3(3)
O2_3	P1_3	O4_3	C24_3	-68.1(4)
O3_3	P1_3	O1_3	Rh4	-97.6(3)
O3_3	P1_3	O2_3	Rh3	130.7(3)
O3_3	P1_3	O4_3	C24_3	44.4(4)
O3_3	C3_3	C4_3	C5_3	-176.9(7)
O4_3	P1_3	O1_3	Rh4	150.9(3)
O4_3	P1_3	O2_3	Rh3	-118.3(3)
O4_3	P1_3	O3_3	C3_3	46.1(4)
O4_3	C24_3	C25_3	C26_3	-177.8(8)
C1_3	C2_3	C3_3	O3_3	1.7(7)
C1_3	C2_3	C3_3	C4_3	-175.6(5)
C1_3	C2_3	C7_3	C6_3	176.2(5)
C1_3	C2_3	C7_3	C17_3	-7.3(9)
C1_3	C24_3	C25_3	C26_3	0.0(12)
C1_3	C28_3	C35_3	C36_3	178.7(8)
C2_3	C1_3	C24_3	O4_3	-8.8(7)
C2_3	C1_3	C24_3	C25_3	173.4(7)
C2_3	C1_3	C28_3	C27_3	-173.6(7)
C2_3	C1_3	C28_3	C35_3	17.2(9)
C2_3	C3_3	C4_3	C5_3	0.5(6)
C2_3	C7_3	C17_3	C16_3	-177.1(8)
C3_3	C2_3	C7_3	C6_3	0.8(7)
C3_3	C2_3	C7_3	C17_3	177.3(6)
C3_3	C4_3	C5_3	C6_3	-2.2(10)
C3_3	C4_3	C5_3	C8_3	175.4(6)
C4_3	C5_3	C6_3	C7_3	3.2(11)
C4_3	C5_3	C6_3	C14_3	-175.9(7)

Atom	Atom	Atom	Atom	Angle/°
C4_3	C5_3	C8_3	C9_3	-115.9(8)
C4_3	C5_3	C8_3	C13_3	58.3(10)
C5_3	C6_3	C7_3	C2_3	-2.4(8)
C5_3	C6_3	C7_3	C17_3	-179.2(8)
C5_3	C6_3	C14_3	C15_3	178.6(9)
C5_3	C8_3	C9_3	C10_3	174.2(6)
C5_3	C8_3	C13_3	C12_3	-174.5(9)
C6_3	C5_3	C8_3	C9_3	61.7(10)
C6_3	C5_3	C8_3	C13_3	-124.1(9)
C6_3	C7_3	C17_3	C16_3	-0.6(6)
C6_3	C14_3	C15_3	C16_3	1.8(10)
C6_3	C14_3	C15_3	C18_3	-176.8(6)
C7_3	C2_3	C3_3	O3_3	177.5(6)
C7_3	C2_3	C3_3	C4_3	0.2(5)
C7_3	C6_3	C14_3	C15_3	-0.5(6)
C8_3	C5_3	C6_3	C7_3	-174.3(5)
C8_3	C5_3	C6_3	C14_3	6.6(10)
C8_3	C9_3	C10_3	C11_3	-0.7(7)
C9_3	C8_3	C13_3	C12_3	-0.4(13)
C9_3	C10_3	C11_3	C12_3	2.1(16)
C10_3	C11_3	C12_3	C13_3	-2.5(19)
C11_3	C12_3	C13_3	C8_3	1.5(17)
C13_3	C8_3	C9_3	C10_3	0.0(7)
C14_3	C6_3	C7_3	C2_3	176.7(7)
C14_3	C6_3	C7_3	C17_3	-0.1(3)
C14_3	C15_3	C16_3	C17_3	-2.4(12)
C14_3	C15_3	C18_3	C19_3	-40.4(10)
C14_3	C15_3	C18_3	C23_3	130.8(9)
C15_3	C16_3	C17_3	C7_3	1.9(10)
C15_3	C18_3	C19_3	C20_3	172.9(8)
C15_3	C18_3	C23_3	C22_3	-175.8(10)
C16_3	C15_3	C18_3	C19_3	141.0(8)
C16_3	C15_3	C18_3	C23_3	-47.9(11)
C18_3	C15_3	C16_3	C17_3	176.2(7)
C18_3	C19_3	C20_3	C21_3	-0.1(7)
C19_3	C18_3	C23_3	C22_3	-4.3(13)
C19_3	C20_3	C21_3	C22_3	1.4(14)
C20_3	C21_3	C22_3	C23_3	-4.1(18)
C21_3	C22_3	C23_3	C18_3	5.6(17)
C23_3	C18_3	C19_3	C20_3	1.6(6)
C24_3	C1_3	C2_3	C3_3	56.6(7)
C24_3	C1_3	C2_3	C7_3	-118.9(6)
C24_3	C1_3	C28_3	C27_3	0.1(6)
C24_3	C1_3	C28_3	C35_3	-169.2(8)
C24_3	C25_3	C26_3	C27_3	0.8(15)
C24_3	C25_3	C26_3	C29_3	-170.3(7)
C25_3	C26_3	C27_3	C28_3	-1.2(15)
C25_3	C26_3	C27_3	C38_3	175.6(11)
C25_3	C26_3	C29_3	C30_3	-73.0(12)
C25_3	C26_3	C29_3	C34_3	108.2(12)
C26_3	C27_3	C28_3	C1_3	0.7(11)
C26_3	C27_3	C28_3	C35_3	170.7(10)
C26_3	C27_3	C38_3	C37_3	177.9(10)
C26_3	C29_3	C30_3	C31_3	-178.6(8)
C26_3	C29_3	C34_3	C33_3	177.6(12)
C27_3	C26_3	C29_3	C30_3	115.9(11)

Atom	Atom	Atom	Atom	Angle/˚
C27_3	C26_3	C29_3	C34_3	-63.0(13)
C27_3	C28_3	C35_3	C36_3	9.1(14)
C28_3	C1_3	C2_3	C3_3	-129.7(5)
C28_3	C1_3	C2_3	C7_3	54.8(8)
C28_3	C1_3	C24_3	O4_3	177.3(6)
C28_3	C1_3	C24_3	C25_3	-0.5(6)
C28_3	C27_3	C38_3	C37_3	-5.3(15)
C28_3	C35_3	C36_3	C37_3	0.2(16)
C29_3	C26_3	C27_3	C28_3	169.6(7)
C29_3	C26_3	C27_3	C38_3	-13.6(15)
C29_3	C30_3	C31_3	C32_3	-1.0(7)
C30_3	C29_3	C34_3	C33_3	-1.3(16)
C30_3	C31_3	C32_3	C33_3	2.9(15)
C31_3	C32_3	C33_3	C34_3	-4(2)
C32_3	C33_3	C34_3	C29_3	3(2)
C34_3	C29_3	C30_3	C31_3	0.2(7)
C35_3	C36_3	C37_3	C38_3	-12.2(15)
C35_3	C36_3	C37_3	C39_3	175.6(9)
C36_3	C37_3	C38_3	C27_3	14.9(16)
C36_3	C37_3	C39_3	C40_3	37.1(10)
C36_3	C37_3	C39_3	C44_3	-139.0(8)
C37_3	C39_3	C40_3	C41_3	-176.1(7)
C37_3	C39_3	C44_3	C43_3	176.1(7)
C38_3	C27_3	C28_3	C1_3	-176.3(8)
C38_3	C27_3	C28_3	C35_3	-6.4(12)
C38_3	C37_3	C39_3	C40_3	-135.0(8)
C38_3	C37_3	C39_3	C44_3	48.9(10)
C39_3	C37_3	C38_3	C27_3	-172.8(9)
C39_3	C40_3	C41_3	C42_3	0.00(8)
C40_3	C39_3	C44_3	C43_3	0.01(18)
C40_3	C41_3	C42_3	C43_3	0.01(19)
C41_3	C42_3	C43_3	C44_3	0.0(3)
C42_3	C43_3	C44_3	C39_3	0.0(2)
C44_3	C39_3	C40_3	C41_3	-0.01(8)
P1_5	O3_5	C3_5	C2_5	-75.2(5)
P1_5	O3_5	C3_5	C4_5	102.7(5)
P1_5	O4_5	C24_5	C1_5	-67.5(6)
P1_5	O4_5	C24_5	C25_5	115.1(6)
O1_5	P1_5	O2_5	Rh1	1.1(4)
O1_5	P1_5	O3_5	C3_5	-60.0(4)
O1_5	P1_5	O4_5	C24_5	154.6(4)
O2_5	P1_5	O1_5	Rh2	23.1(4)
O2_5	P1_5	O3_5	C3_5	169.4(4)
O2_5	P1_5	O4_5	C24_5	-76.4(4)
O3_5	P1_5	O1_5	Rh2	-100.9(3)
O3_5	P1_5	O2_5	Rh1	128.2(3)
O3_5	P1_5	O4_5	C24_5	37.0(4)
O3_5	C3_5	C4_5	C5_5	-178.0(7)
O4_5	P1_5	O1_5	Rh2	147.9(3)
O4_5	P1_5	O2_5	Rh1	-120.2(3)
O4_5	P1_5	O3_5	C3_5	52.0(4)
O4_5	C24_5	C25_5	C26_5	174.5(8)
C1_5	C2_5	C3_5	O3_5	-4.7(7)
C1_5	C2_5	C3_5	C4_5	177.6(6)
C1_5	C2_5	C7_5	C6_5	-178.5(6)
C1_5	C2_5	C7_5	C17_5	7.5(10)

Atom	Atom	Atom	Atom	Angle/°
C1_5	C24_5	C25_5	C26_5	-2.9(11)
C1_5	C28_5	C35_5	C36_5	179.7(9)
C2_5	C1_5	C24_5	O4_5	-5.1(7)
C2_5	C1_5	C24_5	C25_5	172.1(7)
C2_5	C1_5	C28_5	C27_5	-171.5(8)
C2_5	C1_5	C28_5	C35_5	19.6(10)
C2_5	C3_5	C4_5	C5_5	-0.2(7)
C2_5	C7_5	C17_5	C16_5	174.7(9)
C3_5	C2_5	C7_5	C6_5	-2.3(8)
C3_5	C2_5	C7_5	C17_5	-176.3(7)
C3_5	C4_5	C5_5	C6_5	0.5(12)
C3_5	C4_5	C5_5	C8_5	178.9(6)
C4_5	C5_5	C6_5	C7_5	-1.8(13)
C4_5	C5_5	C6_5	C14_5	175.3(8)
C4_5	C5_5	C8_5	C9_5	-106.0(11)
C4_5	C5_5	C8_5	C13_5	72.7(13)
C5_5	C6_5	C7_5	C2_5	2.7(10)
C5_5	C6_5	C7_5	C17_5	177.2(10)
C5_5	C6_5	C14_5	C15_5	-177.3(11)
C5_5	C8_5	C9_5	C10_5	178.0(7)
C5_5	C8_5	C13_5	C12_5	-176.9(11)
C6_5	C5_5	C8_5	C9_5	72.4(12)
C6_5	C5_5	C8_5	C13_5	-108.9(13)
C6_5	C7_5	C17_5	C16_5	0.6(6)
C6_5	C14_5	C15_5	C16_5	-0.1(12)
C6_5	C14_5	C15_5	C18_5	177.9(10)
C7_5	C2_5	C3_5	O3_5	178.9(6)
C7_5	C2_5	C3_5	C4_5	1.1(5)
C7_5	C6_5	C14_5	C15_5	-0.2(6)
C8_5	C5_5	C6_5	C7_5	179.9(6)
C8_5	C5_5	C6_5	C14_5	-3.0(12)
C8_5	C9_5	C10_5	C11_5	0.1(7)
C9_5	C8_5	C13_5	C12_5	1.8(16)
C9_5	C10_5	C11_5	C12_5	-0.7(18)
C10_5	C11_5	C12_5	C13_5	2(2)
C11_5	C12_5	C13_5	C8_5	-2(2)
C13_5	C8_5	C9_5	C10_5	-0.7(7)
C14_5	C6_5	C7_5	C2_5	-174.5(8)
C14_5	C6_5	C7_5	C17_5	0.0(3)
C14_5	C15_5	C16_5	C17_5	0.6(14)
C14_5	C15_5	C18_5	C19_5	-42.0(13)
C14_5	C15_5	C18_5	C23_5	141.3(11)
C15_5	C16_5	C17_5	C7_5	-0.9(12)
C15_5	C18_5	C19_5	C20_5	-176.7(9)
C15_5	C18_5	C23_5	C22_5	177.1(12)
C16_5	C15_5	C18_5	C19_5	136.0(11)
C16_5	C15_5	C18_5	C23_5	-40.7(15)
C18_5	C15_5	C16_5	C17_5	-177.4(10)
C18_5	C19_5	C20_5	C21_5	-0.4(7)
C19_5	C18_5	C23_5	C22_5	0.2(15)
C19_5	C20_5	C21_5	C22_5	0.3(16)
C20_5	C21_5	C22_5	C23_5	0(2)
C21_5	C22_5	C23_5	C18_5	0(2)
C23_5	C18_5	C19_5	C20_5	0.1(7)
C24_5	C1_5	C2_5	C3_5	57.3(7)
C24_5	C1_5	C2_5	C7_5	-126.5(7)

Atom	Atom	Atom	Atom	Angle/°
C24_5	C1_5	C28_5	C27_5	-0.1(6)
C24_5	C1_5	C28_5	C35_5	-169.1(9)
C24_5	C25_5	C26_5	C27_5	4.9(14)
C24_5	C25_5	C26_5	C29_5	-167.7(7)
C25_5	C26_5	C27_5	C28_5	-4.6(14)
C25_5	C26_5	C27_5	C38_5	176.5(12)
C25_5	C26_5	C29_5	C30_5	-64.8(10)
C25_5	C26_5	C29_5	C34_5	116.5(10)
C26_5	C27_5	C28_5	C1_5	2.1(11)
C26_5	C27_5	C28_5	C35_5	171.8(10)
C26_5	C27_5	C38_5	C37_5	-179.1(11)
C26_5	C29_5	C30_5	C31_5	-177.8(7)
C26_5	C29_5	C34_5	C33_5	176.7(10)
C27_5	C26_5	C29_5	C30_5	122.6(9)
C27_5	C26_5	C29_5	C34_5	-56.1(12)
C27_5	C28_5	C35_5	C36_5	10.5(15)
C28_5	C1_5	C2_5	C3_5	-131.5(5)
C28_5	C1_5	C2_5	C7_5	44.7(9)
C28_5	C1_5	C24_5	O4_5	-176.7(6)
C28_5	C1_5	C24_5	C25_5	0.5(6)
C28_5	C27_5	C38_5	C37_5	2.0(18)
C28_5	C35_5	C36_5	C37_5	-3.7(18)
C29_5	C26_5	C27_5	C28_5	167.7(7)
C29_5	C26_5	C27_5	C38_5	-11.1(16)
C29_5	C30_5	C31_5	C32_5	0.5(7)
C30_5	C29_5	C34_5	C33_5	-2.0(14)
C30_5	C31_5	C32_5	C33_5	-0.9(14)
C31_5	C32_5	C33_5	C34_5	-0.2(18)
C32_5	C33_5	C34_5	C29_5	1.7(18)
C34_5	C29_5	C30_5	C31_5	0.9(7)
C35_5	C36_5	C37_5	C38_5	-4.3(18)
C35_5	C36_5	C37_5	C39_5	178.6(10)
C36_5	C37_5	C38_5	C27_5	5.0(19)
C36_5	C37_5	C39_5	C40_5	33.6(13)
C36_5	C37_5	C39_5	C44_5	-143.7(9)
C37_5	C39_5	C40_5	C41_5	-177.5(7)
C37_5	C39_5	C44_5	C43_5	177.4(7)
C38_5	C27_5	C28_5	C1_5	-178.9(10)
C38_5	C27_5	C28_5	C35_5	-9.2(13)
C38_5	C37_5	C39_5	C40_5	-143.5(9)
C38_5	C37_5	C39_5	C44_5	39.1(12)
C39_5	C37_5	C38_5	C27_5	-177.7(11)
C39_5	C40_5	C41_5	C42_5	0.00(8)
C40_5	C39_5	C44_5	C43_5	0.02(18)
C40_5	C41_5	C42_5	C43_5	0.00(19)
C41_5	C42_5	C43_5	C44_5	0.0(3)
C42_5	C43_5	C44_5	C39_5	0.0(2)
C44_5	C39_5	C40_5	C41_5	-0.01(8)
P1_6	O3_6	C3_6	C2_6	-75.2(6)
P1_6	O3_6	C3_6	C4_6	105.7(5)
P1_6	O4_6	C24_6	C1_6	-69.3(6)
P1_6	O4_6	C24_6	C25_6	103.1(6)
O1_6	P1_6	O2_6	Rh5	-8.2(6)
O1_6	P1_6	O3_6	C3_6	-71.8(4)
O1_6	P1_6	O4_6	C24_6	166.7(4)
O2_6	P1_6	O1_6	Rh5[1]	-9.7(6)

Atom	Atom	Atom	Atom	Angle/˚
O2_6	P1_6	O3_6	C3_6	157.5(4)
O2_6	P1_6	O4_6	C24_6	-64.3(5)
O3_6	P1_6	O1_6	Rh5[1]	-133.5(4)
O3_6	P1_6	O2_6	Rh5	118.9(4)
O3_6	P1_6	O4_6	C24_6	48.3(4)
O3_6	C3_6	C4_6	C5_6	179.6(7)
O4_6	P1_6	O1_6	Rh5[1]	114.2(4)
O4_6	P1_6	O2_6	Rh5	-129.6(4)
O4_6	P1_6	O3_6	C3_6	41.4(4)
O4_6	C24_6	C25_6	C26_6	-176.7(8)
C1_6	C2_6	C3_6	O3_6	2.3(7)
C1_6	C2_6	C3_6	C4_6	-178.6(6)
C1_6	C2_6	C7_6	C6_6	-178.6(5)
C1_6	C2_6	C7_6	C17_6	5.8(9)
C1_6	C24_6	C25_6	C26_6	-4.6(11)
C1_6	C28_6	C35_6	C36_6	174.6(9)
C2_6	C1_6	C24_6	O4_6	-10.1(7)
C2_6	C1_6	C24_6	C25_6	178.2(7)
C2_6	C1_6	C28_6	C27_6	-176.2(7)
C2_6	C1_6	C28_6	C35_6	9.8(10)
C2_6	C3_6	C4_6	C5_6	0.4(6)
C2_6	C7_6	C17_6	C16_6	176.0(8)
C3_6	C2_6	C7_6	C6_6	4.5(7)
C3_6	C2_6	C7_6	C17_6	-171.0(6)
C3_6	C4_6	C5_6	C6_6	-2.3(10)
C3_6	C4_6	C5_6	C8_6	177.6(5)
C4_6	C5_6	C6_6	C7_6	5.3(10)
C4_6	C5_6	C6_6	C14_6	174.6(7)
C4_6	C5_6	C8_6	C9_6	-73.1(8)
C4_6	C5_6	C8_6	C13_6	104.4(9)
C5_6	C6_6	C7_6	C2_6	-6.4(8)
C5_6	C6_6	C7_6	C17_6	169.5(7)
C5_6	C6_6	C14_6	C15_6	-169.0(8)
C5_6	C8_6	C9_6	C10_6	177.8(6)
C5_6	C8_6	C13_6	C12_6	-177.6(9)
C6_6	C5_6	C8_6	C9_6	106.8(8)
C6_6	C5_6	C8_6	C13_6	-75.6(10)
C6_6	C7_6	C17_6	C16_6	0.3(6)
C6_6	C14_6	C15_6	C16_6	-0.6(9)
C6_6	C14_6	C15_6	C18_6	-178.7(6)
C7_6	C2_6	C3_6	O3_6	179.3(6)
C7_6	C2_6	C3_6	C4_6	-1.6(5)
C7_6	C6_6	C14_6	C15_6	0.3(5)
C8_6	C5_6	C6_6	C7_6	-174.6(5)
C8_6	C5_6	C6_6	C14_6	-5.3(9)
C8_6	C9_6	C10_6	C11_6	0.5(6)
C9_6	C8_6	C13_6	C12_6	-0.1(13)
C9_6	C10_6	C11_6	C12_6	-1.8(15)
C10_6	C11_6	C12_6	C13_6	2.0(18)
C11_6	C12_6	C13_6	C8_6	-0.9(16)
C13_6	C8_6	C9_6	C10_6	0.3(6)
C14_6	C6_6	C7_6	C2_6	-176.1(6)
C14_6	C6_6	C7_6	C17_6	-0.2(3)
C14_6	C15_6	C16_6	C17_6	0.7(11)
C14_6	C15_6	C18_6	C19_6	45.3(9)
C14_6	C15_6	C18_6	C23_6	-130.1(8)

Atom	Atom	Atom	Atom	Angle/°
C15_6	C16_6	C17_6	C7_6	-0.6(10)
C15_6	C18_6	C19_6	C20_6	-175.7(6)
C15_6	C18_6	C23_6	C22_6	176.7(8)
C16_6	C15_6	C18_6	C19_6	-132.8(7)
C16_6	C15_6	C18_6	C23_6	51.8(10)
C18_6	C15_6	C16_6	C17_6	178.8(6)
C18_6	C19_6	C20_6	C21_6	0.7(6)
C19_6	C18_6	C23_6	C22_6	1.1(12)
C19_6	C20_6	C21_6	C22_6	-2.1(13)
C20_6	C21_6	C22_6	C23_6	2.9(16)
C21_6	C22_6	C23_6	C18_6	-2.4(15)
C23_6	C18_6	C19_6	C20_6	-0.2(6)
C24_6	C1_6	C2_6	C3_6	56.8(7)
C24_6	C1_6	C2_6	C7_6	-120.0(6)
C24_6	C1_6	C28_6	C27_6	0.2(6)
C24_6	C1_6	C28_6	C35_6	-173.8(9)
C24_6	C25_6	C26_6	C27_6	5.7(14)
C24_6	C25_6	C26_6	C29_6	-178.8(7)
C25_6	C26_6	C27_6	C28_6	-4.0(14)
C25_6	C26_6	C27_6	C38_6	176.8(10)
C25_6	C26_6	C29_6	C30_6	-64.1(13)
C25_6	C26_6	C29_6	C34_6	116.9(12)
C26_6	C27_6	C28_6	C1_6	0.9(11)
C26_6	C27_6	C28_6	C35_6	175.4(9)
C26_6	C27_6	C38_6	C37_6	-171.8(10)
C26_6	C29_6	C30_6	C31_6	-179.8(8)
C26_6	C29_6	C34_6	C33_6	-179.5(12)
C27_6	C26_6	C29_6	C30_6	111.4(12)
C27_6	C26_6	C29_6	C34_6	-67.6(13)
C27_6	C28_6	C35_6	C36_6	0.5(15)
C28_6	C1_6	C2_6	C3_6	-126.9(5)
C28_6	C1_6	C2_6	C7_6	56.3(8)
C28_6	C1_6	C24_6	O4_6	173.3(6)
C28_6	C1_6	C24_6	C25_6	1.6(6)
C28_6	C27_6	C38_6	C37_6	8.9(14)
C28_6	C35_6	C36_6	C37_6	1.4(17)
C29_6	C26_6	C27_6	C28_6	-179.4(7)
C29_6	C26_6	C27_6	C38_6	1.4(14)
C29_6	C30_6	C31_6	C32_6	-0.8(8)
C30_6	C29_6	C34_6	C33_6	1.4(16)
C30_6	C31_6	C32_6	C33_6	1.5(17)
C31_6	C32_6	C33_6	C34_6	-1(2)
C32_6	C33_6	C34_6	C29_6	-1(2)
C34_6	C29_6	C30_6	C31_6	-0.7(7)
C35_6	C36_6	C37_6	C38_6	2.0(15)
C35_6	C36_6	C37_6	C39_6	-174.7(9)
C36_6	C37_6	C38_6	C27_6	-7.4(15)
C36_6	C37_6	C39_6	C40_6	36.7(10)
C36_6	C37_6	C39_6	C44_6	-138.3(8)
C37_6	C39_6	C40_6	C41_6	-175.1(7)
C37_6	C39_6	C44_6	C43_6	175.1(7)
C38_6	C27_6	C28_6	C1_6	-179.8(8)
C38_6	C27_6	C28_6	C35_6	-5.3(12)
C38_6	C37_6	C39_6	C40_6	-140.0(8)
C38_6	C37_6	C39_6	C44_6	44.9(10)
C39_6	C37_6	C38_6	C27_6	169.5(9)

Atom	Atom	Atom	Atom	Angle/°
C39_6	C40_6	C41_6	C42_6	0.01(8)
C40_6	C39_6	C44_6	C43_6	-0.04(18)
C40_6	C41_6	C42_6	C43_6	-0.01(19)
C41_6	C42_6	C43_6	C44_6	0.0(3)
C42_6	C43_6	C44_6	C39_6	0.0(2)
C44_6	C39_6	C40_6	C41_6	0.02(8)
C2S_7	O1S_7	C4S_7	C5S_7	110.7(15)
C4S_7	O1S_7	C2S_7	C3S_7	97(2)
C2S_8	O1S_8	C4S_8	C5S_8	61.2(18)
C4S_8	O1S_8	C2S_8	C3S_8	116(2)
P1_9	O3_9	C3_9	C2_9	-73.5(6)
P1_9	O3_9	C3_9	C4_9	102.2(5)
P1_9	O4_9	C24_9	C1_9	-72.0(6)
P1_9	O4_9	C24_9	C25_9	102.2(6)
O1_9	P1_9	O2_9	Rh3	10.7(4)
O1_9	P1_9	O3_9	C3_9	-62.4(4)
O1_9	P1_9	O4_9	C24_9	158.1(4)
O2_9	P1_9	O1_9	Rh4	20.9(4)
O2_9	P1_9	O3_9	C3_9	166.9(4)
O2_9	P1_9	O4_9	C24_9	-72.7(4)
O3_9	P1_9	O1_9	Rh4	-103.0(3)
O3_9	P1_9	O2_9	Rh3	137.7(3)
O3_9	P1_9	O4_9	C24_9	40.3(4)
O3_9	C3_9	C4_9	C5_9	-176.7(7)
O4_9	P1_9	O1_9	Rh4	145.4(3)
O4_9	P1_9	O2_9	Rh3	-110.8(3)
O4_9	P1_9	O3_9	C3_9	50.2(4)
O4_9	C24_9	C25_9	C26_9	-177.6(8)
C1_9	C2_9	C3_9	O3_9	-6.5(7)
C1_9	C2_9	C3_9	C4_9	178.3(6)
C1_9	C2_9	C7_9	C6_9	-176.7(5)
C1_9	C2_9	C7_9	C17_9	4.6(9)
C1_9	C24_9	C25_9	C26_9	-3.7(11)
C1_9	C28_9	C35_9	C36_9	176.0(10)
C2_9	C1_9	C24_9	O4_9	-2.2(7)
C2_9	C1_9	C24_9	C25_9	-175.9(6)
C2_9	C1_9	C28_9	C27_9	177.7(7)
C2_9	C1_9	C28_9	C35_9	3.4(10)
C2_9	C3_9	C4_9	C5_9	-1.3(7)
C2_9	C7_9	C17_9	C16_9	178.2(8)
C3_9	C2_9	C7_9	C6_9	3.3(8)
C3_9	C2_9	C7_9	C17_9	-175.5(6)
C3_9	C4_9	C5_9	C6_9	2.5(12)
C3_9	C4_9	C5_9	C8_9	-177.2(7)
C4_9	C5_9	C6_9	C7_9	-0.9(13)
C4_9	C5_9	C6_9	C14_9	175.7(7)
C4_9	C5_9	C8_9	C9_9	-113.8(9)
C4_9	C5_9	C8_9	C13_9	57.9(12)
C5_9	C6_9	C7_9	C2_9	-2.1(10)
C5_9	C6_9	C7_9	C17_9	176.8(9)
C5_9	C6_9	C14_9	C15_9	-176.4(11)
C5_9	C8_9	C9_9	C10_9	171.8(7)
C5_9	C8_9	C13_9	C12_9	-171.6(11)
C6_9	C5_9	C8_9	C9_9	66.5(12)
C6_9	C5_9	C8_9	C13_9	-121.8(11)
C6_9	C7_9	C17_9	C16_9	-0.5(6)

Atom	Atom	Atom	Atom	Angle/°
C6_9	C14_9	C15_9	C16_9	0.1(11)
C6_9	C14_9	C15_9	C18_9	-176.6(7)
C7_9	C2_9	C3_9	O3_9	173.6(6)
C7_9	C2_9	C3_9	C4_9	-1.6(5)
C7_9	C6_9	C14_9	C15_9	0.1(6)
C8_9	C5_9	C6_9	C7_9	178.8(7)
C8_9	C5_9	C6_9	C14_9	-4.7(12)
C8_9	C9_9	C10_9	C11_9	0.6(7)
C9_9	C8_9	C13_9	C12_9	0.0(15)
C9_9	C10_9	C11_9	C12_9	-1.6(18)
C10_9	C11_9	C12_9	C13_9	2(2)
C11_9	C12_9	C13_9	C8_9	-0.9(19)
C13_9	C8_9	C9_9	C10_9	0.1(7)
C14_9	C6_9	C7_9	C2_9	-178.7(7)
C14_9	C6_9	C7_9	C17_9	0.1(3)
C14_9	C15_9	C16_9	C17_9	-0.5(13)
C14_9	C15_9	C18_9	C19_9	-48.2(11)
C14_9	C15_9	C18_9	C23_9	139.8(9)
C15_9	C16_9	C17_9	C7_9	0.7(11)
C15_9	C18_9	C19_9	C20_9	-172.3(8)
C15_9	C18_9	C23_9	C22_9	172.6(10)
C16_9	C15_9	C18_9	C19_9	135.1(8)
C16_9	C15_9	C18_9	C23_9	-36.9(12)
C18_9	C15_9	C16_9	C17_9	176.2(8)
C18_9	C19_9	C20_9	C21_9	0.7(7)
C19_9	C18_9	C23_9	C22_9	0.2(13)
C19_9	C20_9	C21_9	C22_9	-1.6(15)
C20_9	C21_9	C22_9	C23_9	1.7(18)
C21_9	C22_9	C23_9	C18_9	-1.0(17)
C23_9	C18_9	C19_9	C20_9	-0.1(6)
C24_9	C1_9	C2_9	C3_9	57.9(7)
C24_9	C1_9	C2_9	C7_9	-122.2(6)
C24_9	C1_9	C28_9	C27_9	0.5(6)
C24_9	C1_9	C28_9	C35_9	-173.8(9)
C24_9	C25_9	C26_9	C27_9	3.8(15)
C24_9	C25_9	C26_9	C29_9	-179.8(7)
C25_9	C26_9	C27_9	C28_9	-1.9(15)
C25_9	C26_9	C27_9	C38_9	-178.7(11)
C25_9	C26_9	C29_9	C30_9	59.1(12)
C25_9	C26_9	C29_9	C34_9	-115.8(11)
C26_9	C27_9	C28_9	C1_9	-0.3(11)
C26_9	C27_9	C28_9	C35_9	174.4(10)
C26_9	C27_9	C38_9	C37_9	-175.5(12)
C26_9	C29_9	C30_9	C31_9	-175.2(8)
C26_9	C29_9	C34_9	C33_9	176.0(10)
C27_9	C26_9	C29_9	C30_9	-124.5(10)
C27_9	C26_9	C29_9	C34_9	60.6(12)
C27_9	C28_9	C35_9	C36_9	1.5(16)
C28_9	C1_9	C2_9	C3_9	-119.3(6)
C28_9	C1_9	C2_9	C7_9	60.6(8)
C28_9	C1_9	C24_9	O4_9	175.2(6)
C28_9	C1_9	C24_9	C25_9	1.5(6)
C28_9	C27_9	C38_9	C37_9	7.6(16)
C28_9	C35_9	C36_9	C37_9	7(2)
C29_9	C26_9	C27_9	C28_9	-178.2(7)
C29_9	C26_9	C27_9	C38_9	4.9(16)

Atom	Atom	Atom	Atom	Angle/°
C29_9	C30_9	C31_9	C32_9	0.6(7)
C30_9	C29_9	C34_9	C33_9	0.9(14)
C30_9	C31_9	C32_9	C33_9	-1.6(15)
C31_9	C32_9	C33_9	C34_9	2.3(19)
C32_9	C33_9	C34_9	C29_9	-1.9(19)
C34_9	C29_9	C30_9	C31_9	-0.2(7)
C35_9	C36_9	C37_9	C38_9	-8(2)
C35_9	C36_9	C37_9	C39_9	179.4(11)
C36_9	C37_9	C38_9	C27_9	0.8(19)
C36_9	C37_9	C39_9	C40_9	32.6(14)
C36_9	C37_9	C39_9	C44_9	-146.6(10)
C37_9	C39_9	C40_9	C41_9	-179.2(7)
C37_9	C39_9	C44_9	C43_9	179.2(7)
C38_9	C27_9	C28_9	C1_9	176.8(9)
C38_9	C27_9	C28_9	C35_9	-8.5(13)
C38_9	C37_9	C39_9	C40_9	-139.5(9)
C38_9	C37_9	C39_9	C44_9	41.2(13)
C39_9	C37_9	C38_9	C27_9	173.1(10)
C39_9	C40_9	C41_9	C42_9	0.00(8)
C40_9	C39_9	C44_9	C43_9	-0.01(18)
C40_9	C41_9	C42_9	C43_9	0.00(19)
C41_9	C42_9	C43_9	C44_9	0.0(3)
C42_9	C43_9	C44_9	C39_9	0.0(2)
C44_9	C39_9	C40_9	C41_9	0.01(8)
P1_10	O3_10	C3_10	C2_10	-75.4(5)
P1_10	O3_10	C3_10	C4_10	102.7(4)
P1_10	O4_10	C24_10	C1_10	-67.0(7)
P1_10	O4_10	C24_10	C25_10	107.2(8)
O1_10	P1_10	O2_10	Rh3	-0.7(5)
O1_10	P1_10	O3_10	C3_10	-62.5(4)
O1_10	P1_10	O4_10	C24_10	157.5(4)
O2_10	P1_10	O1_10	Rh4	24.6(4)
O2_10	P1_10	O3_10	C3_10	167.2(4)
O2_10	P1_10	O4_10	C24_10	-73.5(5)
O3_10	P1_10	O1_10	Rh4	-98.9(3)
O3_10	P1_10	O2_10	Rh3	125.8(3)
O3_10	P1_10	O4_10	C24_10	39.9(4)
O3_10	C3_10	C4_10	C5_10	-177.6(6)
O4_10	P1_10	O1_10	Rh4	149.4(3)
O4_10	P1_10	O2_10	Rh3	-122.0(3)
O4_10	P1_10	O3_10	C3_10	49.8(4)
O4_10	C24_10	C25_10	C26_10	-170.7(11)
C1_10	C2_10	C3_10	O3_10	-3.0(6)
C1_10	C2_10	C3_10	C4_10	179.1(6)
C1_10	C2_10	C7_10	C6_10	-176.0(5)
C1_10	C2_10	C7_10	C17_10	-0.1(9)
C1_10	C24_10	C25_10	C26_10	3.2(15)
C1_10	C28_10	C35_10	C36_10	175.5(11)
C2_10	C1_10	C24_10	O4_10	-7.9(8)
C2_10	C1_10	C24_10	C25_10	178.5(9)
C2_10	C1_10	C28_10	C27_10	-179.6(8)
C2_10	C1_10	C28_10	C35_10	10.7(11)
C2_10	C3_10	C4_10	C5_10	0.3(6)
C2_10	C7_10	C17_10	C16_10	-176.5(8)
C3_10	C2_10	C7_10	C6_10	4.1(7)
C3_10	C2_10	C7_10	C17_10	-180.0(5)

Atom	Atom	Atom	Atom	Angle/°
C3_10	C4_10	C5_10	C6_10	-3.0(10)
C3_10	C4_10	C5_10	C8_10	-179.2(5)
C4_10	C5_10	C6_10	C7_10	6.2(10)
C4_10	C5_10	C6_10	C14_10	-176.8(6)
C4_10	C5_10	C8_10	C9_10	-116.9(7)
C4_10	C5_10	C8_10	C13_10	56.0(9)
C5_10	C6_10	C7_10	C2_10	-6.7(8)
C5_10	C6_10	C7_10	C17_10	177.0(7)
C5_10	C6_10	C14_10	C15_10	-176.6(8)
C5_10	C8_10	C9_10	C10_10	170.7(6)
C5_10	C8_10	C13_10	C12_10	-167.0(8)
C6_10	C5_10	C8_10	C9_10	66.9(9)
C6_10	C5_10	C8_10	C13_10	-120.2(8)
C6_10	C7_10	C17_10	C16_10	-0.5(6)
C6_10	C14_10	C15_10	C16_10	-0.2(10)
C6_10	C14_10	C15_10	C18_10	-172.5(6)
C7_10	C2_10	C3_10	O3_10	176.9(6)
C7_10	C2_10	C3_10	C4_10	-1.0(5)
C7_10	C6_10	C14_10	C15_10	0.5(5)
C8_10	C5_10	C6_10	C7_10	-177.8(5)
C8_10	C5_10	C6_10	C14_10	-0.8(9)
C8_10	C9_10	C10_10	C11_10	0.2(6)
C9_10	C8_10	C13_10	C12_10	5.8(11)
C9_10	C10_10	C11_10	C12_10	-2.4(14)
C10_10	C11_10	C12_10	C13_10	5.8(16)
C11_10	C12_10	C13_10	C8_10	-7.3(14)
C13_10	C8_10	C9_10	C10_10	-2.1(6)
C14_10	C6_10	C7_10	C2_10	176.1(6)
C14_10	C6_10	C7_10	C17_10	-0.1(3)
C14_10	C15_10	C16_10	C17_10	-0.5(12)
C14_10	C15_10	C18_10	C19_10	39.0(9)
C14_10	C15_10	C18_10	C23_10	-144.9(7)
C15_10	C16_10	C17_10	C7_10	0.8(11)
C15_10	C18_10	C19_10	C20_10	176.1(7)
C15_10	C18_10	C23_10	C22_10	-176.2(7)
C16_10	C15_10	C18_10	C19_10	-133.2(8)
C16_10	C15_10	C18_10	C23_10	42.9(10)
C18_10	C15_10	C16_10	C17_10	171.8(7)
C18_10	C19_10	C20_10	C21_10	-0.01(8)
C19_10	C18_10	C23_10	C22_10	0.02(18)
C19_10	C20_10	C21_10	C22_10	0.01(19)
C20_10	C21_10	C22_10	C23_10	0.0(3)
C21_10	C22_10	C23_10	C18_10	0.0(2)
C23_10	C18_10	C19_10	C20_10	-0.01(8)
C24_10	C1_10	C2_10	C3_10	58.8(7)
C24_10	C1_10	C2_10	C7_10	-121.1(7)
C24_10	C1_10	C28_10	C27_10	0.8(7)
C24_10	C1_10	C28_10	C35_10	-168.9(10)
C24_10	C25_10	C26_10	C27_10	-3.3(19)
C24_10	C25_10	C26_10	C29_10	178.7(10)
C25_10	C26_10	C27_10	C28_10	2.3(19)
C25_10	C26_10	C27_10	C38_10	-178.9(15)
C25_10	C26_10	C29_10	C30_10	60.9(14)
C25_10	C26_10	C29_10	C34_10	-119.6(14)
C26_10	C27_10	C28_10	C1_10	-1.0(14)
C26_10	C27_10	C28_10	C35_10	169.3(13)

Atom	Atom	Atom	Atom	Angle/°
C26_10	C27_10	C38_10	C37_10	-164.5(15)
C26_10	C29_10	C30_10	C31_10	179.3(9)
C26_10	C29_10	C34_10	C33_10	-179.7(12)
C27_10	C26_10	C29_10	C30_10	-117.0(13)
C27_10	C26_10	C29_10	C34_10	62.4(15)
C27_10	C28_10	C35_10	C36_10	5.6(17)
C28_10	C1_10	C2_10	C3_10	-120.9(6)
C28_10	C1_10	C2_10	C7_10	59.2(8)
C28_10	C1_10	C24_10	O4_10	171.7(7)
C28_10	C1_10	C24_10	C25_10	-1.9(7)
C28_10	C27_10	C38_10	C37_10	14.2(19)
C28_10	C35_10	C36_10	C37_10	-5(2)
C29_10	C26_10	C27_10	C28_10	-179.8(9)
C29_10	C26_10	C27_10	C38_10	-1.1(19)
C29_10	C30_10	C31_10	C32_10	-0.3(8)
C30_10	C29_10	C34_10	C33_10	-0.2(16)
C30_10	C31_10	C32_10	C33_10	1.1(16)
C31_10	C32_10	C33_10	C34_10	-1(2)
C32_10	C33_10	C34_10	C29_10	1(2)
C34_10	C29_10	C30_10	C31_10	-0.1(7)
C35_10	C36_10	C37_10	C38_10	9(2)
C35_10	C36_10	C37_10	C39_10	-173.5(13)
C36_10	C37_10	C38_10	C27_10	-14(2)
C36_10	C37_10	C39_10	C40_10	32.0(18)
C36_10	C37_10	C39_10	C44_10	-154.9(13)
C37_10	C39_10	C40_10	C41_10	173.3(9)
C37_10	C39_10	C44_10	C43_10	-173.2(10)
C38_10	C27_10	C28_10	C1_10	-179.9(11)
C38_10	C27_10	C28_10	C35_10	-9.5(15)
C38_10	C37_10	C39_10	C40_10	-150.6(11)
C38_10	C37_10	C39_10	C44_10	22.5(16)
C39_10	C37_10	C38_10	C27_10	168.4(13)
C39_10	C40_10	C41_10	C42_10	0.00(8)
C40_10	C39_10	C44_10	C43_10	0.02(18)
C40_10	C41_10	C42_10	C43_10	-0.01(19)
C41_10	C42_10	C43_10	C44_10	0.0(3)
C42_10	C43_10	C44_10	C39_10	0.0(2)
C44_10	C39_10	C40_10	C41_10	-0.01(8)
C2S_11	O1S_11	C4S_11	C5S_11	43(3)
C4S_11	O1S_11	C2S_11	C3S_11	-95.5(15)
C2S_12	O1S_12	C4S_12	C5S_12	70.1(12)
C4S_12	O1S_12	C2S_12	C3S_12	86.0(16)
P1_13	O3_13	C3_13	C2_13	-77.8(6)
P1_13	O3_13	C3_13	C4_13	107.6(4)
P1_13	O4_13	C24_13	C1_13	-71.5(5)
P1_13	O4_13	C24_13	C25_13	101.2(5)
O1_13	P1_13	O2_13	Rh1	-2.3(4)
O1_13	P1_13	O3_13	C3_13	-73.8(4)
O1_13	P1_13	O4_13	C24_13	169.3(3)
O2_13	P1_13	O1_13	Rh2	24.6(4)
O2_13	P1_13	O3_13	C3_13	155.3(3)
O2_13	P1_13	O4_13	C24_13	-61.2(4)
O3_13	P1_13	O1_13	Rh2	-99.2(3)
O3_13	P1_13	O2_13	Rh1	125.3(3)
O3_13	P1_13	O4_13	C24_13	50.7(4)
O3_13	C3_13	C4_13	C5_13	173.2(8)

Atom	Atom	Atom	Atom	Angle/°
O4_13	P1_13	O1_13	Rh2	149.3(3)
O4_13	P1_13	O2_13	Rh1	-124.5(3)
O4_13	P1_13	O3_13	C3_13	39.5(4)
O4_13	C24_13	C25_13	C26_13	-174.3(7)
C1_13	C2_13	C3_13	O3_13	7.2(7)
C1_13	C2_13	C3_13	C4_13	-178.9(6)
C1_13	C2_13	C7_13	C6_13	-179.0(6)
C1_13	C2_13	C7_13	C17_13	1.8(10)
C1_13	C24_13	C25_13	C26_13	-2.0(10)
C1_13	C28_13	C35_13	C36_13	-176.1(7)
C2_13	C1_13	C24_13	O4_13	-8.6(6)
C2_13	C1_13	C24_13	C25_13	179.3(6)
C2_13	C1_13	C28_13	C27_13	-176.9(6)
C2_13	C1_13	C28_13	C35_13	4.0(8)
C2_13	C3_13	C4_13	C5_13	-1.1(7)
C2_13	C7_13	C17_13	C16_13	179.8(8)
C3_13	C2_13	C7_13	C6_13	4.6(8)
C3_13	C2_13	C7_13	C17_13	-174.6(6)
C3_13	C4_13	C5_13	C6_13	2.1(13)
C3_13	C4_13	C5_13	C8_13	-172.6(7)
C4_13	C5_13	C6_13	C7_13	0.3(14)
C4_13	C5_13	C6_13	C14_13	175.8(8)
C4_13	C5_13	C8_13	C9_13	100.4(9)
C4_13	C5_13	C8_13	C13_13	-69.3(10)
C5_13	C6_13	C7_13	C2_13	-3.7(11)
C5_13	C6_13	C7_13	C17_13	175.6(10)
C5_13	C6_13	C14_13	C15_13	-175.4(12)
C5_13	C8_13	C9_13	C10_13	-169.6(6)
C5_13	C8_13	C13_13	C12_13	169.5(7)
C6_13	C5_13	C8_13	C9_13	-74.2(11)
C6_13	C5_13	C8_13	C13_13	116.1(10)
C6_13	C7_13	C17_13	C16_13	0.6(6)
C6_13	C14_13	C15_13	C16_13	-0.6(11)
C6_13	C14_13	C15_13	C18_13	175.8(11)
C7_13	C2_13	C3_13	O3_13	-176.2(6)
C7_13	C2_13	C3_13	C4_13	-2.3(5)
C7_13	C6_13	C14_13	C15_13	0.1(6)
C8_13	C5_13	C6_13	C7_13	174.6(7)
C8_13	C5_13	C6_13	C14_13	-9.8(12)
C8_13	C9_13	C10_13	C11_13	0.00(8)
C9_13	C8_13	C13_13	C12_13	0.00(18)
C9_13	C10_13	C11_13	C12_13	0.0(2)
C10_13	C11_13	C12_13	C13_13	0.0(3)
C11_13	C12_13	C13_13	C8_13	0.0(2)
C13_13	C8_13	C9_13	C10_13	0.00(8)
C14_13	C6_13	C7_13	C2_13	-179.4(7)
C14_13	C6_13	C7_13	C17_13	-0.1(3)
C14_13	C15_13	C16_13	C17_13	1.0(13)
C14_13	C15_13	C18_13	C19_13	19.6(16)
C14_13	C15_13	C18_13	C23_13	-170.8(11)
C15_13	C16_13	C17_13	C7_13	-1.1(11)
C15_13	C18_13	C19_13	C20_13	168.7(14)
C15_13	C18_13	C23_13	C22_13	-167.5(16)
C16_13	C15_13	C18_13	C19_13	-163.9(10)
C16_13	C15_13	C18_13	C23_13	5.7(17)
C18_13	C15_13	C16_13	C17_13	-175.4(10)

Atom	Atom	Atom	Atom	Angle/°
C18_13	C19_13	C20_13	C21_13	-0.4(8)
C19_13	C18_13	C23_13	C22_13	2.4(16)
C19_13	C20_13	C21_13	C22_13	0.5(17)
C20_13	C21_13	C22_13	C23_13	1(2)
C21_13	C22_13	C23_13	C18_13	-2(2)
C23_13	C18_13	C19_13	C20_13	-1.1(7)
C24_13	C1_13	C2_13	C3_13	52.6(7)
C24_13	C1_13	C2_13	C7_13	-123.8(6)
C24_13	C1_13	C28_13	C27_13	1.0(5)
C24_13	C1_13	C28_13	C35_13	-178.2(8)
C24_13	C25_13	C26_13	C27_13	0.0(12)
C24_13	C25_13	C26_13	C29_13	-176.4(7)
C25_13	C26_13	C27_13	C28_13	2.2(12)
C25_13	C26_13	C27_13	C38_13	-175.6(9)
C25_13	C26_13	C29_13	C30_13	-125.8(8)
C25_13	C26_13	C29_13	C34_13	45.1(11)
C26_13	C27_13	C28_13	C1_13	-2.7(10)
C26_13	C27_13	C28_13	C35_13	176.5(8)
C26_13	C27_13	C38_13	C37_13	-179.6(9)
C26_13	C29_13	C30_13	C31_13	171.8(7)
C26_13	C29_13	C34_13	C33_13	-174.5(10)
C27_13	C26_13	C29_13	C30_13	57.8(10)
C27_13	C26_13	C29_13	C34_13	-131.3(9)
C27_13	C28_13	C35_13	C36_13	4.7(12)
C28_13	C1_13	C2_13	C3_13	-129.6(5)
C28_13	C1_13	C2_13	C7_13	54.0(8)
C28_13	C1_13	C24_13	O4_13	173.4(5)
C28_13	C1_13	C24_13	C25_13	1.4(6)
C28_13	C27_13	C38_13	C37_13	2.5(13)
C28_13	C35_13	C36_13	C37_13	-0.5(15)
C29_13	C26_13	C27_13	C28_13	178.4(7)
C29_13	C26_13	C27_13	C38_13	0.6(13)
C29_13	C30_13	C31_13	C32_13	-0.6(7)
C30_13	C29_13	C34_13	C33_13	-3.2(13)
C30_13	C31_13	C32_13	C33_13	2.6(14)
C31_13	C32_13	C33_13	C34_13	-4.8(17)
C32_13	C33_13	C34_13	C29_13	5.2(17)
C34_13	C29_13	C30_13	C31_13	0.9(6)
C35_13	C36_13	C37_13	C38_13	-2.9(14)
C35_13	C36_13	C37_13	C39_13	178.7(8)
C36_13	C37_13	C38_13	C27_13	1.8(14)
C36_13	C37_13	C39_13	C40_13	39.6(10)
C36_13	C37_13	C39_13	C44_13	-138.5(8)
C37_13	C39_13	C40_13	C41_13	-178.1(6)
C37_13	C39_13	C44_13	C43_13	178.1(6)
C38_13	C27_13	C28_13	C1_13	175.3(7)
C38_13	C27_13	C28_13	C35_13	-5.5(10)
C38_13	C37_13	C39_13	C40_13	-138.8(7)
C38_13	C37_13	C39_13	C44_13	43.2(10)
C39_13	C37_13	C38_13	C27_13	-179.8(7)
C39_13	C40_13	C41_13	C42_13	0.00(8)
C40_13	C39_13	C44_13	C43_13	0.00(18)
C40_13	C41_13	C42_13	C43_13	0.00(19)
C41_13	C42_13	C43_13	C44_13	0.0(3)
C42_13	C43_13	C44_13	C39_13	0.0(2)
C44_13	C39_13	C40_13	C41_13	0.00(8)

Atom	Atom	Atom	Atom	Angle/°
P1_14	O3_14	C3_14	C2_14	-75.2(6)
P1_14	O3_14	C3_14	C4_14	110.7(5)
P1_14	O4_14	C24_14	C1_14	-71.6(5)
P1_14	O4_14	C24_14	C25_14	101.2(6)
O1_14	P1_14	O2_14	Rh1	-0.5(5)
O1_14	P1_14	O3_14	C3_14	-76.2(4)
O1_14	P1_14	O4_14	C24_14	171.6(4)
O2_14	P1_14	O1_14	Rh2	22.1(4)
O2_14	P1_14	O3_14	C3_14	153.1(4)
O2_14	P1_14	O4_14	C24_14	-59.2(4)
O3_14	P1_14	O1_14	Rh2	-101.5(3)
O3_14	P1_14	O2_14	Rh1	127.3(3)
O3_14	P1_14	O4_14	C24_14	52.4(4)
O3_14	C3_14	C4_14	C5_14	172.7(8)
O4_14	P1_14	O1_14	Rh2	146.6(3)
O4_14	P1_14	O2_14	Rh1	-122.3(3)
O4_14	P1_14	O3_14	C3_14	37.2(4)
O4_14	C24_14	C25_14	C26_14	-173.4(7)
C1_14	C2_14	C3_14	O3_14	5.6(8)
C1_14	C2_14	C3_14	C4_14	179.0(6)
C1_14	C2_14	C7_14	C6_14	-176.4(6)
C1_14	C2_14	C7_14	C17_14	6.0(10)
C1_14	C24_14	C25_14	C26_14	-1.0(11)
C1_14	C28_14	C35_14	C36_14	177.5(9)
C2_14	C1_14	C24_14	O4_14	-9.4(7)
C2_14	C1_14	C24_14	C25_14	178.5(7)
C2_14	C1_14	C28_14	C27_14	-176.7(7)
C2_14	C1_14	C28_14	C35_14	4.6(9)
C2_14	C3_14	C4_14	C5_14	-1.2(7)
C2_14	C7_14	C17_14	C16_14	177.7(9)
C3_14	C2_14	C7_14	C6_14	5.5(8)
C3_14	C2_14	C7_14	C17_14	-172.2(7)
C3_14	C4_14	C5_14	C6_14	2.3(12)
C3_14	C4_14	C5_14	C8_14	-177.6(7)
C4_14	C5_14	C6_14	C7_14	0.5(13)
C4_14	C5_14	C6_14	C14_14	173.9(8)
C4_14	C5_14	C8_14	C9_14	-64.3(10)
C4_14	C5_14	C8_14	C13_14	112.9(10)
C5_14	C6_14	C7_14	C2_14	-4.4(10)
C5_14	C6_14	C7_14	C17_14	173.4(10)
C5_14	C6_14	C14_14	C15_14	-173.7(11)
C5_14	C8_14	C9_14	C10_14	177.0(7)
C5_14	C8_14	C13_14	C12_14	-176.3(10)
C6_14	C5_14	C8_14	C9_14	115.9(10)
C6_14	C5_14	C8_14	C13_14	-67.0(12)
C6_14	C7_14	C17_14	C16_14	0.0(6)
C6_14	C14_14	C15_14	C16_14	1.0(11)
C6_14	C14_14	C15_14	C18_14	172.8(9)
C7_14	C2_14	C3_14	O3_14	-176.2(7)
C7_14	C2_14	C3_14	C4_14	-2.8(5)
C7_14	C6_14	C14_14	C15_14	-0.3(6)
C8_14	C5_14	C6_14	C7_14	-179.6(6)
C8_14	C5_14	C6_14	C14_14	-6.3(12)
C8_14	C9_14	C10_14	C11_14	0.6(6)
C9_14	C8_14	C13_14	C12_14	0.8(14)
C9_14	C10_14	C11_14	C12_14	-1.9(15)

Atom	Atom	Atom	Atom	Angle/°
C10_14	C11_14	C12_14	C13_14	2.5(18)
C11_14	C12_14	C13_14	C8_14	-1.8(17)
C13_14	C8_14	C9_14	C10_14	-0.1(7)
C14_14	C6_14	C7_14	C2_14	-178.0(8)
C14_14	C6_14	C7_14	C17_14	-0.2(3)
C14_14	C15_14	C16_14	C17_14	-1.1(13)
C14_14	C15_14	C18_14	C19_14	-21.9(14)
C14_14	C15_14	C18_14	C23_14	146.2(11)
C15_14	C16_14	C17_14	C7_14	0.6(11)
C15_14	C18_14	C19_14	C20_14	167.0(11)
C15_14	C18_14	C23_14	C22_14	-165.2(14)
C16_14	C15_14	C18_14	C19_14	149.9(11)
C16_14	C15_14	C18_14	C23_14	-42.0(14)
C18_14	C15_14	C16_14	C17_14	-172.9(9)
C18_14	C19_14	C20_14	C21_14	-0.1(8)
C19_14	C18_14	C23_14	C22_14	3.3(16)
C19_14	C20_14	C21_14	C22_14	-0.6(17)
C20_14	C21_14	C22_14	C23_14	3(2)
C21_14	C22_14	C23_14	C18_14	-4(2)
C23_14	C18_14	C19_14	C20_14	-1.2(7)
C24_14	C1_14	C2_14	C3_14	53.8(7)
C24_14	C1_14	C2_14	C7_14	-124.3(7)
C24_14	C1_14	C28_14	C27_14	1.0(6)
C24_14	C1_14	C28_14	C35_14	-177.7(8)
C24_14	C25_14	C26_14	C27_14	-0.5(14)
C24_14	C25_14	C26_14	C29_14	-175.9(7)
C25_14	C26_14	C27_14	C28_14	2.2(14)
C25_14	C26_14	C27_14	C38_14	-176.4(10)
C25_14	C26_14	C29_14	C30_14	-123.3(10)
C25_14	C26_14	C29_14	C34_14	60.0(12)
C26_14	C27_14	C28_14	C1_14	-2.4(10)
C26_14	C27_14	C28_14	C35_14	176.4(9)
C26_14	C27_14	C38_14	C37_14	-175.1(11)
C26_14	C29_14	C30_14	C31_14	-175.5(8)
C26_14	C29_14	C34_14	C33_14	174.8(12)
C27_14	C26_14	C29_14	C30_14	61.3(12)
C27_14	C26_14	C29_14	C34_14	-115.4(11)
C27_14	C28_14	C35_14	C36_14	-1.2(14)
C28_14	C1_14	C2_14	C3_14	-128.5(5)
C28_14	C1_14	C2_14	C7_14	53.4(8)
C28_14	C1_14	C24_14	O4_14	172.8(5)
C28_14	C1_14	C24_14	C25_14	0.7(6)
C28_14	C27_14	C38_14	C37_14	6.2(16)
C28_14	C35_14	C36_14	C37_14	6.6(19)
C29_14	C26_14	C27_14	C28_14	177.4(7)
C29_14	C26_14	C27_14	C38_14	-1.2(14)
C29_14	C30_14	C31_14	C32_14	1.8(7)
C30_14	C29_14	C34_14	C33_14	-2.0(15)
C30_14	C31_14	C32_14	C33_14	-4.0(15)
C31_14	C32_14	C33_14	C34_14	3(2)
C32_14	C33_14	C34_14	C29_14	0(2)
C34_14	C29_14	C30_14	C31_14	1.2(7)
C35_14	C36_14	C37_14	C38_14	-5(2)
C35_14	C36_14	C37_14	C39_14	175.7(10)
C36_14	C37_14	C38_14	C27_14	-1.2(19)
C36_14	C37_14	C39_14	C40_14	25.9(15)

Atom	Atom	Atom	Atom	Angle/°
C36_14	C37_14	C39_14	C44_14	-156.0(10)
C37_14	C39_14	C40_14	C41_14	178.2(8)
C37_14	C39_14	C44_14	C43_14	-178.2(8)
C38_14	C27_14	C28_14	C1_14	176.3(8)
C38_14	C27_14	C28_14	C35_14	-4.9(12)
C38_14	C37_14	C39_14	C40_14	-153.1(9)
C38_14	C37_14	C39_14	C44_14	25.1(13)
C39_14	C37_14	C38_14	C27_14	177.8(10)
C39_14	C40_14	C41_14	C42_14	0.01(8)
C40_14	C39_14	C44_14	C43_14	0.00(18)
C40_14	C41_14	C42_14	C43_14	-0.04(19)
C41_14	C42_14	C43_14	C44_14	0.0(3)
C42_14	C43_14	C44_14	C39_14	0.0(2)
C44_14	C39_14	C40_14	C41_14	0.00(8)
P1_4	O3_4	C3_4	C2_4	-75.8(5)
P1_4	O3_4	C3_4	C4_4	100.3(5)
P1_4	O4_4	C24_4	C1_4	-69.4(6)
P1_4	O4_4	C24_4	C25_4	112.8(6)
O1_4	P1_4	O2_4	Rh1	4.2(4)
O1_4	P1_4	O3_4	C3_4	-61.7(4)
O1_4	P1_4	O4_4	C24_4	156.7(4)
O2_4	P1_4	O1_4	Rh2	23.0(4)
O2_4	P1_4	O3_4	C3_4	168.4(4)
O2_4	P1_4	O4_4	C24_4	-73.9(4)
O3_4	P1_4	O1_4	Rh2	-100.2(3)
O3_4	P1_4	O2_4	Rh1	130.5(3)
O3_4	P1_4	O4_4	C24_4	39.1(4)
O3_4	C3_4	C4_4	C5_4	-175.9(7)
O4_4	P1_4	O1_4	Rh2	148.6(3)
O4_4	P1_4	O2_4	Rh1	-118.2(3)
O4_4	P1_4	O3_4	C3_4	51.0(4)
O4_4	C24_4	C25_4	C26_4	176.3(7)
C1_4	C2_4	C3_4	O3_4	-4.0(7)
C1_4	C2_4	C3_4	C4_4	-179.7(6)
C1_4	C2_4	C7_4	C6_4	-179.1(6)
C1_4	C2_4	C7_4	C17_4	1.8(10)
C1_4	C24_4	C25_4	C26_4	-1.4(11)
C1_4	C28_4	C35_4	C36_4	171.2(10)
C2_4	C1_4	C24_4	O4_4	-4.9(7)
C2_4	C1_4	C24_4	C25_4	172.7(7)
C2_4	C1_4	C28_4	C27_4	-173.6(8)
C2_4	C1_4	C28_4	C35_4	18.8(10)
C2_4	C3_4	C4_4	C5_4	-0.1(6)
C2_4	C7_4	C17_4	C16_4	179.4(9)
C3_4	C2_4	C7_4	C6_4	1.9(8)
C3_4	C2_4	C7_4	C17_4	-177.2(6)
C3_4	C4_4	C5_4	C6_4	-0.6(12)
C3_4	C4_4	C5_4	C8_4	172.7(6)
C4_4	C5_4	C6_4	C7_4	2.0(13)
C4_4	C5_4	C6_4	C14_4	178.5(7)
C4_4	C5_4	C8_4	C9_4	-94.7(10)
C4_4	C5_4	C8_4	C13_4	79.4(11)
C5_4	C6_4	C7_4	C2_4	-2.6(10)
C5_4	C6_4	C7_4	C17_4	176.6(10)
C5_4	C6_4	C14_4	C15_4	-176.8(11)
C5_4	C8_4	C9_4	C10_4	172.9(7)

Atom	Atom	Atom	Atom	Angle/°
C5_4	C8_4	C13_4	C12_4	-171.4(9)
C6_4	C5_4	C8_4	C9_4	78.6(11)
C6_4	C5_4	C8_4	C13_4	-107.2(11)
C6_4	C7_4	C17_4	C16_4	0.3(6)
C6_4	C14_4	C15_4	C16_4	0.3(11)
C6_4	C14_4	C15_4	C18_4	-179.6(7)
C7_4	C2_4	C3_4	O3_4	175.1(6)
C7_4	C2_4	C3_4	C4_4	-0.6(5)
C7_4	C6_4	C14_4	C15_4	-0.2(6)
C8_4	C5_4	C6_4	C7_4	-171.1(6)
C8_4	C5_4	C6_4	C14_4	5.5(12)
C8_4	C9_4	C10_4	C11_4	-0.3(5)
C9_4	C8_4	C13_4	C12_4	2.8(11)
C9_4	C10_4	C11_4	C12_4	0.3(13)
C10_4	C11_4	C12_4	C13_4	1.2(16)
C11_4	C12_4	C13_4	C8_4	-2.7(14)
C13_4	C8_4	C9_4	C10_4	-1.2(5)
C14_4	C6_4	C7_4	C2_4	-179.3(7)
C14_4	C6_4	C7_4	C17_4	-0.1(3)
C14_4	C15_4	C16_4	C17_4	-0.2(13)
C14_4	C15_4	C18_4	C19_4	-45.4(10)
C14_4	C15_4	C18_4	C23_4	139.6(9)
C15_4	C16_4	C17_4	C7_4	-0.1(11)
C15_4	C18_4	C19_4	C20_4	-175.0(8)
C15_4	C18_4	C23_4	C22_4	174.7(10)
C16_4	C15_4	C18_4	C19_4	134.7(9)
C16_4	C15_4	C18_4	C23_4	-40.3(12)
C18_4	C15_4	C16_4	C17_4	179.7(7)
C18_4	C19_4	C20_4	C21_4	-0.4(7)
C19_4	C18_4	C23_4	C22_4	-0.5(13)
C19_4	C20_4	C21_4	C22_4	1.3(15)
C20_4	C21_4	C22_4	C23_4	-1.8(18)
C21_4	C22_4	C23_4	C18_4	1.3(18)
C23_4	C18_4	C19_4	C20_4	0.0(6)
C24_4	C1_4	C2_4	C3_4	57.4(7)
C24_4	C1_4	C2_4	C7_4	-121.6(6)
C24_4	C1_4	C28_4	C27_4	-0.9(6)
C24_4	C1_4	C28_4	C35_4	-168.6(9)
C24_4	C25_4	C26_4	C27_4	4.4(13)
C24_4	C25_4	C26_4	C29_4	-168.4(7)
C25_4	C26_4	C27_4	C28_4	-5.5(14)
C25_4	C26_4	C27_4	C38_4	165.9(11)
C25_4	C26_4	C29_4	C30_4	-53.0(12)
C25_4	C26_4	C29_4	C34_4	122.1(10)
C26_4	C27_4	C28_4	C1_4	3.7(11)
C26_4	C27_4	C28_4	C35_4	172.1(10)
C26_4	C27_4	C38_4	C37_4	-170.1(12)
C26_4	C29_4	C30_4	C31_4	175.0(8)
C26_4	C29_4	C34_4	C33_4	-174.8(10)
C27_4	C26_4	C29_4	C30_4	134.2(9)
C27_4	C26_4	C29_4	C34_4	-50.7(12)
C27_4	C28_4	C35_4	C36_4	3.3(16)
C28_4	C1_4	C2_4	C3_4	-130.0(5)
C28_4	C1_4	C2_4	C7_4	51.0(8)
C28_4	C1_4	C24_4	O4_4	-177.9(6)
C28_4	C1_4	C24_4	C25_4	-0.3(6)

Atom	Atom	Atom	Atom	Angle/°
C28_4	C27_4	C38_4	C37_4	1.5(17)
C28_4	C35_4	C36_4	C37_4	-8(2)
C29_4	C26_4	C27_4	C28_4	167.0(8)
C29_4	C26_4	C27_4	C38_4	-21.6(15)
C29_4	C30_4	C31_4	C32_4	0.0(6)
C30_4	C29_4	C34_4	C33_4	0.5(12)
C30_4	C31_4	C32_4	C33_4	-0.3(12)
C31_4	C32_4	C33_4	C34_4	0.6(16)
C32_4	C33_4	C34_4	C29_4	-0.7(16)
C34_4	C29_4	C30_4	C31_4	-0.1(5)
C35_4	C36_4	C37_4	C38_4	9(2)
C35_4	C36_4	C37_4	C39_4	-177.7(12)
C36_4	C37_4	C38_4	C27_4	-6(2)
C36_4	C37_4	C39_4	C40_4	26.6(17)
C36_4	C37_4	C39_4	C44_4	-154.3(12)
C37_4	C39_4	C40_4	C41_4	179.1(10)
C37_4	C39_4	C44_4	C43_4	-179.1(10)
C38_4	C27_4	C28_4	C1_4	-168.3(9)
C38_4	C27_4	C28_4	C35_4	0.1(13)
C38_4	C37_4	C39_4	C40_4	-160.5(11)
C38_4	C37_4	C39_4	C44_4	18.6(16)
C39_4	C37_4	C38_4	C27_4	-179.3(11)
C39_4	C40_4	C41_4	C42_4	-0.01(8)
C40_4	C39_4	C44_4	C43_4	0.01(18)
C40_4	C41_4	C42_4	C43_4	0.04(19)
C41_4	C42_4	C43_4	C44_4	0.0(3)
C42_4	C43_4	C44_4	C39_4	0.0(2)
C44_4	C39_4	C40_4	C41_4	-0.01(8)

————

[1]-1-x,+y,3/2-z

Table 10.1.7: Hydrogen Fractional Atomic Coordinates (×10[4]) and Equivalent Isotropic Displacement Parameters (Å[2]×10[3]) for S-**6a** (ZC-01-51). U_{eq} is defined as 1/3 of the trace of the orthogonalised U_{ij}.

Atom	x	y	z	U_{eq}
HA	-3399(10)	7819(12)	7378(6)	229
HB	-3410(10)	7677(8)	7606(7)	229
H4_1	-1016.98	9206.56	6693.87	84
H9_1	-1829.43	8757.94	6811.34	179
H10_1	-1656.49	8542.13	7105.2	174
H11_1	-1391.98	8699.29	7403.12	175
H12_1	-1328.4	9037.55	7453.44	175
H13_1	-1475.08	9257.04	7159.53	128
H14_1	-2729.76	9071.15	6973.83	107
H16_1	-3801.27	9297	6567.34	96
H17_1	-3104.5	9415.55	6372.2	96
H19_1	-3387.46	8871.28	7068.37	202
H20_1	-4115.12	8760.85	7276.86	204
H21_1	-4936.21	8937.88	7265.54	204
H22_1	-5095.53	9193.9	7024.7	206
H23_1	-4350.69	9323.55	6836.43	207
H25_1	-1782.5	9416.63	5736.13	70
H30_1	-1905.44	9983.24	5595.36	272
H31_1	-2139.2	10075.01	5259.22	272

Atom	x	y	z	U_{eq}
H32_1	-2730.98	9865.33	5068.94	255
H33_1	-3099.84	9567.68	5216.94	285
H34_1	-2869.43	9479.77	5554.21	285
H35_1	-2555.99	9749.16	6494.14	87
H36_1	-2986.25	10052.2	6448.9	109
H38_1	-2871.6	9995.55	5839.49	102
H40_1	-3068.19	10384.4	6367.85	163
H41_1	-3582.96	10690.67	6309.97	163
H42_1	-4111.58	10712.14	6017.09	213
H43_1	-4165.72	10454.22	5779.78	196
H44_1	-3665.25	10152.65	5833.33	196
H4_2	-5553.92	7676.38	6742.78	165
H9_2	-6752.14	7662.85	6764.51	218
H10_2	-7463.1	7858.4	6598.4	228
H11_2	-7470.35	7863.83	6247.47	228
H12_2	-6829.71	7730.95	6042.91	228
H13_2	-6104.47	7537.89	6205.05	195
H14_2	-6606.47	7211.47	6372.85	179
H16_2	-6099.61	6657.3	6561.21	212
H17_2	-5422.32	6806.5	6743.95	212
H19_2	-6668.24	6592.59	6216.66	382
H20_2	-7509.39	6483.06	6080.66	382
H21_2	-8297.85	6659.85	6166.37	383
H22_2	-8289.18	6932.34	6395.7	383
H23_2	-7450.19	7045.17	6530.15	382
H25_2	-4501.21	7004.2	7417.59	140
H30_2	-3125.96	6977.47	7362.26	334
H31_2	-2516.31	6796.32	7571.39	334
H32_2	-2754.61	6468.77	7692.65	350
H33_2	-3530.99	6303.42	7557.41	383
H34_2	-4191.17	6503.14	7388.12	383
H35_2	-4541.2	6979.55	6541.08	156
H36_2	-3820.86	6804.89	6413.53	173
H38_2	-3414.94	6613.62	6975.75	159
H40_2	-2760.02	6823.98	6414.26	360
H41_2	-1950.92	6619.58	6290.3	361
H42_2	-1837.79	6269.2	6413.8	292
H43_2	-2465.68	6108.42	6649.89	431
H44_2	-3262.26	6305.4	6773.74	432
H4_3	1124.14	8509.09	6061.71	88
H9_3	2196.47	8414.36	6521.13	122
H10_3	1887.56	8179.27	6782.5	153
H11_3	1292.58	7925.81	6685.86	154
H12_3	1013.11	7862.37	6365.97	155
H13_3	1307.9	8097.55	6101.51	154
H14_3	2824.5	8247.94	6242.28	94
H16_3	3909.64	8580.79	5923.54	103
H17_3	3221.69	8767.03	5785.51	102
H19_3	3623.95	8239.04	6469.64	141
H20_3	4277.61	7999.03	6581.33	143
H21_3	4912.24	7874.09	6348.88	145
H22_3	4953.53	7993.75	6013.83	145
H23_3	4259.44	8212.83	5894.2	145
H25_3	1955.15	9414.49	5690.61	96
H30_3	1836.14	9568.89	5266.63	265
H31_3	2112.69	9879.66	5119.07	264

Atom	x	y	z	U_{eq}
H32_3	3020.2	9990.21	5157.03	284
H33_3	3665.39	9774.47	5313.66	264
H34_3	3376.75	9469.12	5469.48	266
H35_3	2606.69	8601.64	5479.84	92
H36_3	3104.85	8591.99	5188.25	93
H38_3	2987.61	9198.29	5127.66	103
H40_3	3182.29	8643.6	4834.03	118
H41_3	3799.66	8625.85	4550.77	117
H42_3	4488.7	8867.21	4528.77	149
H43_3	4608.65	9122.62	4765.39	157
H44_3	4008.49	9143.07	5044.47	157
H4_5	-1126.99	6317.18	6657.37	88
H9_5	-474.12	6540.33	7137.91	262
H10_5	-777.25	6874.18	7251.3	274
H11_5	-1684.27	6950.69	7214.42	273
H12_5	-2317.57	6747.95	7074.27	274
H13_5	-2031.08	6408.51	6968.83	313
H14_5	-996.91	6233.07	7363.46	137
H16_5	-442.38	5659.3	7444.39	139
H17_5	-448.2	5609.29	7100.99	139
H19_5	-376.89	6281.07	7652.55	336
H20_5	-290.57	6329.38	8008.19	336
H21_5	-558.48	6068.05	8228.66	336
H22_5	-902.17	5758.62	8106.27	336
H23_5	-980.49	5709.17	7750.91	336
H25_5	368.22	5501.43	6362.14	98
H30_5	127.31	5149.56	6148.49	151
H31_5	619.96	4856.65	6053.82	152
H32_5	1012.66	4648.23	6307.31	154
H33_5	929.01	4737.63	6654.42	188
H34_5	459.3	5036.72	6745.98	188
H35_5	-1440.58	5560.12	6907.31	108
H36_5	-1757.68	5249.72	7004.58	94
H38_5	-611.06	4970.35	6668.96	128
H40_5	-2205.98	4948.19	6919.94	128
H41_5	-2519.37	4619.5	7031.59	128
H42_5	-1881.29	4364.23	7089.01	108
H43_5	-962.82	4411.84	7043.85	128
H44_5	-646.01	4732.78	6934.61	128
H4_6	-5572.29	8537.86	7386.76	106
H9_6	-6838.74	8559.82	7323.32	158
H10_6	-7462.73	8742.01	7544.41	128
H11_6	-7287.18	9080.95	7608.55	129
H12_6	-6559.4	9256.48	7498.52	130
H13_6	-5930.08	9078.71	7275.02	122
H14_6	-6496.77	8936.72	6907.22	85
H16_6	-5942.94	8743.96	6361.37	81
H17_6	-5332.43	8537.12	6520.23	81
H19_6	-6525.58	9259.38	6678.68	112
H20_6	-7195.91	9466.44	6523.03	112
H21_6	-7758.51	9324.93	6274.82	112
H22_6	-7640.89	8991.06	6158.03	112
H23_6	-6987.79	8780.54	6318.12	111
H25_6	-4472.34	7845.58	6751.95	107
H30_6	-3316.31	7795.44	6730.72	376
H31_6	-2771.26	7558	6555.29	376

Atom	x	y	z	U_{eq}
H32_6	-2867.18	7519.49	6199.23	358
H33_6	-3479.92	7729.02	6017.94	288
H34_6	-4008.43	7970.61	6194.62	287
H35_6	-4438.88	8722.32	6715.27	91
H36_6	-3740.75	8838.61	6521.7	74
H38_6	-3396.62	8267.84	6337.71	85
H40_6	-2735.55	8845.24	6512.1	114
H41_6	-1996.21	8972.65	6309.35	116
H42_6	-1850.78	8830.45	5989.31	157
H43_6	-2381.08	8573.27	5857.06	113
H44_6	-3107.6	8445.76	6053.01	112
H2SA_7	-253.95	5663.87	5534.87	232
H2SB_7	-698.41	5815.58	5628.7	232
H3SA_7	-736.51	5499.36	5796	422
H3SB_7	-110.51	5520.07	5861.79	422
H3SC_7	-556.3	5672.24	5955.9	422
H4SA_7	610.06	5705.53	5558.46	187
H4SB_7	874.09	5866.78	5713.91	187
H5SA_7	1107.07	5953.39	5375.62	253
H5SB_7	475.64	5970.28	5316.39	253
H5SC_7	739.74	6131.58	5471.87	253
H2SA_8	-304.07	9686.45	5423.22	157
H2SB_8	-691.09	9619.74	5607.88	157
H3SA_8	-970.34	9458.44	5305.7	486
H3SB_8	-382.09	9355.53	5280.38	486
H3SC_8	-770.18	9288.64	5465.54	486
H4SA_8	664.47	9587.69	5434.11	152
H4SB_8	862.63	9557.86	5665.52	152
H5SA_8	894.11	9907.14	5577.09	377
H5SB_8	257.34	9903.88	5523.93	377
H5SC_8	455.51	9874.05	5755.35	377
H4_9	-1123.99	8526.3	6103.13	106
H9_9	-440.41	7990.18	6225.82	203
H10_9	-597.42	7874.8	6572.66	253
H11_9	-1423.67	7950.94	6718.33	253
H12_9	-2087.72	8136.92	6575.03	253
H13_9	-1944.03	8253.35	6227.01	205
H14_9	-1000.22	7830.81	5941.37	138
H16_9	-621.54	7816.69	5342.91	120
H17_9	-685.64	8162.85	5327.81	120
H19_9	-385.28	7522.89	5918.48	184
H20_9	-292.73	7163.77	5900.13	184
H21_9	-633.42	6991.74	5616.73	186
H22_9	-1103.27	7160.9	5357.87	188
H23_9	-1195.32	7519.61	5373.89	188
H25_9	-76.18	8938.63	5255.23	75
H30_9	218.68	8879.67	4846.11	223
H31_9	381.94	9126.39	4592.35	224
H32_9	-304.25	9362.93	4506.59	215
H33_9	-1167.4	9339.99	4662.01	203
H34_9	-1318.81	9096.66	4919.89	203
H35_9	-1731.4	8328.41	5358.04	108
H36_9	-2332.33	8332.1	5094.91	114
H38_9	-1372.39	8714.87	4778.85	99
H40_9	-2969.2	8486.67	4892.96	158
H41_9	-3566.79	8472.77	4603.7	158

Atom	x	y	z	U_{eq}
H42_9	-3189.59	8484.15	4279.07	187
H43_9	-2269.53	8508.15	4221.34	167
H44_9	-1675.49	8522.08	4502.54	167
H4_10	1444.4	9126.07	6560.41	82
H9_10	1067.76	9019.34	7152.18	87
H10_10	1275.8	8672.91	7256.81	118
H11_10	2017.33	8517.09	7109.72	117
H12_10	2522.83	8646.8	6854.89	118
H13_10	2381.03	9005.07	6772.78	97
H14_10	1776.59	9308.96	7239.03	80
H16_10	1394.72	9906.35	7274.03	116
H17_10	1183.02	9907.87	6936.45	116
H19_10	2503.73	9435.88	7461.98	160
H20_10	2855.23	9465.43	7796.37	160
H21_10	2383.04	9659.39	8036.06	162
H22_10	1572.14	9825.48	7955.43	163
H23_10	1217.17	9796.8	7621.74	163
H25_10	46.04	9964.36	6257.06	176
H30_10	-195.78	10370.61	6344.98	262
H31_10	-515.97	10644	6145.32	262
H32_10	-9.88	10754.16	5860.02	285
H33_10	798.07	10582.88	5768.83	296
H34_10	1117.35	10312.06	5971.95	296
H35_10	2096.13	9870.05	6662.96	118
H36_10	2628.09	10151.78	6632.07	144
H38_10	1424.7	10454.08	6351.56	171
H40_10	3176.61	10327.52	6409.27	215
H41_10	3745.62	10603.05	6295.95	216
H42_10	3349.29	10919.55	6237.5	352
H43_10	2437.27	10981.93	6282.8	234
H44_10	1871.6	10714.35	6393.87	233
H2SA_11	-390.91	6876.85	6270.72	205
H2SB_11	-90.49	6978.02	6462.4	205
H3SA_11	-1028.07	6954.58	6526.18	239
H3SB_11	-1008.62	6711.73	6493.78	239
H3SC_11	-707.44	6813.15	6685.94	239
H4SA_11	812.64	6789.83	6515.67	280
H4SB_11	683.72	6561.65	6590.49	280
H5SA_11	659.38	6787.23	6870.96	864
H5SB_11	194.72	6919.94	6761.56	864
H5SC_11	65.73	6691.65	6836.42	864
H2SA_12	27.14	8490.38	6472.92	139
H2SB_12	-311.9	8572.8	6281.41	139
H3SA_12	-907.51	8472.72	6542.77	337
H3SB_12	-627.85	8631.24	6697.27	337
H3SC_12	-967.8	8713.88	6505.24	337
H4SA_12	314.18	8784.13	6724.63	110
H4SB_12	631.11	8953.1	6593.01	110
H5SA_12	1232.74	8687.04	6684.04	143
H5SB_12	849.11	8523.49	6572.09	143
H5SC_12	1166.04	8692.45	6440.47	143
H4_13	1324.34	6267.13	6680.03	95
H9_13	2203.33	6087.49	7126.11	281
H10_13	1869.25	6201.87	7454.92	300
H11_13	1515.13	6526.4	7474.51	301
H12_13	1448.66	6749.58	7215.74	299

Atom	x	y	z	U_{eq}
H13_13	1780.01	6639.28	6884.43	261
H14_13	3087.34	6348.36	6936.27	169
H16_13	4072.88	6141.93	6490.07	96
H17_13	3335.08	6055.4	6299.92	95
H19_13	3761.86	6381.25	7124.12	287
H20_13	4558.79	6437.06	7318.23	286
H21_13	5401.45	6414.01	7158.48	285
H22_13	5485.39	6340.62	6811.85	283
H23_13	4690.58	6297.62	6613.82	281
H25_13	1883.1	6068.85	5702.05	62
H30_13	2154.16	5460.69	5591.69	133
H31_13	2093.9	5373.01	5244.29	134
H32_13	2266.61	5622.07	4992.53	168
H33_13	2578.97	5949.05	5089.29	139
H34_13	2580.43	6040.8	5436.75	138
H35_13	2849.32	5725.57	6423.25	62
H36_13	3363.56	5447.69	6352.24	65
H38_13	3023.58	5498.66	5754.81	64
H40_13	3365.63	5094.2	6239.66	101
H41_13	3943.74	4809.09	6162.4	102
H42_13	4497.84	4827.42	5876.03	123
H43_13	4518.59	5106.35	5662.66	132
H44_13	3955.86	5387.46	5735.28	132
H4_14	-1252.04	6921.12	5986.09	134
H9_14	-1651.27	7138.05	6341.98	172
H10_14	-1468.42	7488.01	6440.83	174
H11_14	-1712.98	7741.65	6215.41	174
H12_14	-2060.34	7693.36	5902.11	172
H13_14	-2262.64	7344.32	5802.4	172
H14_14	-3028.71	7164.03	6045.3	148
H16_14	-3981.83	6680.05	5917.15	148
H17_14	-3233.9	6492.51	5837.12	148
H19_14	-3678.42	7232.31	6279.92	311
H20_14	-4404.07	7459.96	6345.19	312
H21_14	-5216.01	7420.42	6166.87	312
H22_14	-5325.94	7168.96	5918.26	312
H23_14	-4622.44	6928.87	5865.9	311
H25_14	-1733.64	5917.2	5896.02	87
H30_14	-1881.84	5737.24	5317.05	223
H31_14	-1964.84	5386.13	5246.91	223
H32_14	-2408.55	5170.73	5482.67	184
H33_14	-2668.84	5299.28	5804.7	223
H34_14	-2564.85	5649.33	5875.72	223
H35_14	-2778.89	6589.72	5489.14	116
H36_14	-3178.67	6489.98	5193.53	140
H38_14	-2876.93	5901.3	5324.52	133
H40_14	-3319.23	6377.82	4886.9	179
H41_14	-3806.33	6257.67	4593.05	179
H42_14	-4157.12	5928.3	4598.5	178
H43_14	-4058.77	5711.55	4872.29	179
H44_14	-3583.09	5826.02	5161.94	179
H4_4	1179.65	6912.68	6056.28	100
H9_4	283.86	7347.97	6209.66	224
H10_4	430.57	7475.61	6574.78	224
H11_4	1356.2	7492.01	6700.32	224
H12_4	2145.21	7400.06	6514.87	224

Atom	x	y	z	U_{eq}
H13_4	2015.95	7287.08	6143.49	224
H14_4	1007.74	7607.88	5900.36	150
H16_4	500.19	7623.57	5315.56	126
H17_4	532.57	7276.85	5301.5	126
H19_4	375.57	7910.02	5899.99	154
H20_4	285.57	8269.61	5893.47	155
H21_4	602.04	8450.08	5611.43	155
H22_4	971.06	8285.35	5327.68	155
H23_4	1073.32	7926.77	5334.25	154
H25_4	-214.21	6530.21	5254.81	90
H30_4	-28.05	6279.78	4958.57	233
H31_4	-557.02	6154.83	4686.74	234
H32_4	-868.26	6380.85	4432.11	220
H33_4	-657	6731.7	4452.09	243
H34_4	-120.28	6853.76	4723.02	243
H35_4	1553.13	7100.4	5283.24	130
H36_4	1959.52	7137.17	4974.49	137
H38_4	904.08	6746.04	4719.84	136
H40_4	2536.94	7023.09	4742.59	274
H41_4	3008.17	7029.14	4421.71	274
H42_4	2530.76	6942.99	4128.3	235
H43_4	1627.3	6852.47	4130.34	311
H44_4	1157.38	6845.49	4443.03	311

Table 10.1.8: Solvent masking (PLATON/SQUEEZE) information for S-**6a** (ZC-01-51).

No	x	y	z	V	e	Content
1	-0.719	-0.311	-0.581	44903.8	10029.4	240Cl2,240H2,240C1

Citations

CrysAlisPro Software System, Rigaku Oxford Diffraction, (2021).

O.V. Dolomanov and L.J. Bourhis and R.J. Gildea and J.A.K. Howard and H. Puschmann, Olex2: A complete structure solution, refinement and analysis program, *J. Appl. Cryst.*, (2009), **42**, 339-341.

Sheldrick, G.M., Crystal structure refinement with ShelXL, *Acta Cryst.*, (2015), **C71**, 3-8.

Table 10.1.9. Atomic coordinates and U_{eq} [Å^2] for S-6a (ZC-01-51)

Atom	*x*	*y*	*z*	U_{eq}
Rh1	0.00569(3)	0.61571(2)	0.59305(2)	0.0448(2)
Rh2	0.00626(3)	0.64341(2)	0.61920(2)	0.0483(2)
Rh3	0.00413(3)	0.92745(2)	0.58896(2)	0.0535(2)
Rh4	0.00772(3)	0.90216(2)	0.61720(2)	0.0501(2)
Rh5	-0.44908(3)	0.77749(2)	0.75012(2)	0.0571(2)
O	-0.3580(4)	0.77742(16)	0.75059(14)	0.151(5)
HA	-0.3395(9)	0.7826(11)	0.7380(6)	0.227
HB	-0.3405(9)	0.7677(8)	0.7604(7)	0.227
P1_1	-0.09627(8)	0.92660(4)	0.61580(3)	0.0521(6)
O1_1	-0.07508(19)	0.90565(6)	0.62084(8)	0.0580(17)
O2_1	-0.06344(17)	0.93999(7)	0.60188(6)	0.0502(15)
O3_1	-0.10747(15)	0.93993(6)	0.63565(5)	0.0612(15)
O4_1	-0.15567(13)	0.92302(6)	0.60671(7)	0.0548(15)
C1_1	-0.2110(2)	0.94895(8)	0.62153(7)	0.067(2)
C2_1	-0.20326(17)	0.93856(9)	0.64183(7)	0.058(2)
C3_1	-0.15207(17)	0.93417(8)	0.64782(6)	0.051(2)
C4_1	-0.1386(2)	0.92347(11)	0.66592(8)	0.069(3)
H4_1	-0.101696	0.920684	0.669383	0.082
C5_1	-0.1812(2)	0.91714(13)	0.67851(10)	0.0896(18)
C6_1	-0.2359(2)	0.92108(12)	0.67310(8)	0.0888(19)
C7_1	-0.24741(18)	0.93173(10)	0.65419(8)	0.065(2)
C8_1	-0.1668(3)	0.90313(11)	0.69625(8)	0.091(2)
C9_1	-0.1726(5)	0.88155(11)	0.69391(13)	0.150(6)
H9_1	-0.182916	0.875809	0.681130	0.180
C10_1	-0.1624(6)	0.86872(11)	0.71120(16)	0.146(4)
H10_1	-0.165865	0.854251	0.710466	0.176
C11_1	-0.1472(5)	0.87850(17)	0.72899(15)	0.147(4)
H11_1	-0.139362	0.869866	0.740315	0.176
C12_1	-0.1421(6)	0.89836(17)	0.73229(10)	0.147(4)
H12_1	-0.132878	0.903774	0.745325	0.176
C13_1	-0.1517(4)	0.91130(12)	0.71470(11)	0.107(4)
H13_1	-0.147332	0.925704	0.715959	0.129
C14_1	-0.2798(2)	0.91440(13)	0.68512(10)	0.089(2)
H14_1	-0.272773	0.907046	0.697381	0.107
C15_1	-0.3331(2)	0.91826(13)	0.67953(11)	0.093(3)
C16_1	-0.3436(2)	0.92779(13)	0.66112(11)	0.080(2)
H16_1	-0.380122	0.929644	0.656643	0.097
C17_1	-0.3018(2)	0.93446(11)	0.64951(10)	0.080(2)
H17_1	-0.310367	0.941561	0.637237	0.096

C18_1	-0.3795(2)	0.91048(13)	0.69254(12)	0.121(4)
C19_1	-0.3731(3)	0.89381(15)	0.70604(15)	0.169(4)
H19_1	-0.338984	0.886927	0.706695	0.202
C20_1	-0.4162(4)	0.88722(17)	0.71856(18)	0.170(3)
H20_1	-0.411813	0.875866	0.727502	0.204
C21_1	-0.4651(4)	0.8976(2)	0.7175(2)	0.170(3)
H21_1	-0.493650	0.893732	0.726538	0.205
C22_1	-0.4743(3)	0.9135(2)	0.7039(2)	0.172(3)
H22_1	-0.509451	0.919459	0.702575	0.207
C23_1	-0.4303(3)	0.92071(17)	0.69223(19)	0.173(4)
H23_1	-0.434662	0.932585	0.683989	0.207
C24_1	-0.1903(2)	0.94012(8)	0.60481(7)	0.061(2)
C25_1	-0.1948(3)	0.94835(9)	0.58490(7)	0.060(2)
H25_1	-0.178369	0.941618	0.573590	0.072
C26_1	-0.2232(4)	0.96609(10)	0.58216(8)	0.080(3)
C27_1	-0.2464(3)	0.97653(9)	0.59885(8)	0.077(3)
C28_1	-0.2405(3)	0.96784(8)	0.61910(8)	0.066(2)
C29_1	-0.2360(4)	0.97218(11)	0.56082(8)	0.153(5)
C30_1	-0.2145(6)	0.98996(15)	0.55196(14)	0.228(7)
H30_1	-0.190445	0.998367	0.559647	0.274
C31_1	-0.2282(7)	0.99550(18)	0.53190(15)	0.228(7)
H31_1	-0.213576	1.007631	0.526028	0.274
C32_1	-0.2635(7)	0.98307(19)	0.52054(11)	0.218(8)
H32_1	-0.272534	0.986690	0.506877	0.262
C33_1	-0.2855(7)	0.9653(2)	0.52925(14)	0.235(7)
H33_1	-0.309657	0.956917	0.521583	0.282
C34_1	-0.2717(6)	0.96003(17)	0.54935(14)	0.235(7)
H34_1	-0.286719	0.948010	0.555287	0.282
C35_1	-0.2610(3)	0.97979(9)	0.63583(9)	0.072(2)
H35_1	-0.255475	0.974890	0.649363	0.087
C36_1	-0.2876(4)	0.99751(11)	0.63331(10)	0.091(3)
H36_1	-0.298718	1.005171	0.644946	0.109
C37_1	-0.2990(3)	1.00471(10)	0.61394(10)	0.087(3)
C38_1	-0.2787(4)	0.99445(11)	0.59714(10)	0.084(3)
H38_1	-0.286730	0.999598	0.583878	0.100
C39_1	-0.3313(3)	1.02364(8)	0.61065(12)	0.120(3)
C40_1	-0.3290(3)	1.03971(10)	0.62491(12)	0.136(4)
H40_1	-0.306980	1.038408	0.636795	0.164
C41_1	-0.3597(3)	1.05797(9)	0.62159(17)	0.137(4)
H41_1	-0.358567	1.068984	0.631085	0.165
C42_1	-0.3911(4)	1.05906(13)	0.6040(2)	0.181(6)
H42_1	-0.411538	1.071200	0.601766	0.217
C43_1	-0.3945(4)	1.04390(15)	0.58981(16)	0.164(4)
H43_1	-0.416746	1.045434	0.577996	0.197
C44_1	-0.3645(3)	1.02602(13)	0.59306(13)	0.164(4)
H44_1	-0.366402	1.015250	0.583302	0.197
P1_2	-0.50120(13)	0.74799(6)	0.72033(6)	0.1455(17)
O1_2	-0.5535(2)	0.75885(10)	0.72534(11)	0.189(6)
O2_2	-0.4509(2)	0.75342(10)	0.73208(10)	0.130(4)
O3_2	-0.4852(2)	0.75001(8)	0.69690(7)	0.136(3)
O4_2	-0.51484(19)	0.72428(7)	0.72279(8)	0.133(2)
C1_2	-0.4775(2)	0.70810(9)	0.69342(8)	0.099(3)
C2_2	-0.5183(2)	0.71969(8)	0.68078(10)	0.102(3)
C3_2	-0.5197(2)	0.74022(8)	0.68284(9)	0.118(4)

C4_2	-0.5564(3)	0.75320(8)	0.67240(13)	0.136(4)
H4_2	-0.555452	0.767629	0.674256	0.163
C5_2	-0.5941(3)	0.74412(9)	0.65930(16)	0.149(3)
C6_2	-0.5941(3)	0.72275(9)	0.65596(14)	0.148(3)
C7_2	-0.5567(3)	0.71011(8)	0.66753(13)	0.123(4)
C8_2	-0.6364(3)	0.75794(11)	0.64951(13)	0.151(3)
C9_2	-0.6766(4)	0.76746(16)	0.66207(15)	0.183(6)
H9_2	-0.675195	0.766211	0.676548	0.219
C10_2	-0.7189(4)	0.77892(17)	0.6524(2)	0.191(4)
H10_2	-0.746267	0.785783	0.660094	0.230
C11_2	-0.7180(4)	0.77950(18)	0.6313(2)	0.191(4)
H11_2	-0.747281	0.786566	0.624998	0.230
C12_2	-0.6815(5)	0.7715(2)	0.61884(16)	0.191(4)
H12_2	-0.683492	0.773081	0.604414	0.229
C13_2	-0.6382(4)	0.76008(17)	0.62866(14)	0.164(5)
H13_2	-0.610575	0.753978	0.620511	0.197
C14_2	-0.6362(3)	0.71306(10)	0.64509(16)	0.148(3)
H14_2	-0.660735	0.721185	0.637296	0.177
C15_2	-0.6430(3)	0.69210(10)	0.6454(2)	0.208(8)
C16_2	-0.6066(4)	0.68019(10)	0.6563(2)	0.177(5)
H16_2	-0.610037	0.665698	0.656283	0.212
C17_2	-0.5661(4)	0.68929(9)	0.66684(17)	0.177(5)
H17_2	-0.542171	0.680695	0.674391	0.212
C18_2	-0.6969(3)	0.68301(13)	0.6389(2)	0.297(10)
C19_2	-0.6997(4)	0.66608(17)	0.6256(3)	0.319(7)
H19_2	-0.667189	0.658935	0.622116	0.383
C20_2	-0.7496(5)	0.6596(2)	0.6172(2)	0.319(7)
H20_2	-0.751209	0.648072	0.608379	0.383
C21_2	-0.7961(4)	0.6703(2)	0.6223(3)	0.320(7)
H21_2	-0.829769	0.666189	0.616356	0.384
C22_2	-0.7960(3)	0.6868(2)	0.6356(3)	0.320(7)
H22_2	-0.828941	0.693580	0.639113	0.384
C23_2	-0.7460(4)	0.6933(2)	0.6437(3)	0.320(7)
H23_2	-0.745067	0.704833	0.652631	0.383
C24_2	-0.4791(3)	0.71005(9)	0.71374(8)	0.111(3)
C25_2	-0.4458(3)	0.69887(13)	0.72735(8)	0.119(4)
H25_2	-0.449872	0.700461	0.741724	0.143
C26_2	-0.4078(3)	0.68576(14)	0.71966(9)	0.118(4)
C27_2	-0.4042(3)	0.68215(14)	0.69846(8)	0.118(4)
C28_2	-0.4389(3)	0.69385(13)	0.68491(8)	0.125(4)
C29_2	-0.3710(3)	0.67534(13)	0.73434(11)	0.160(5)
C30_2	-0.3216(4)	0.68406(18)	0.7407(2)	0.277(8)
H30_2	-0.312436	0.697509	0.736354	0.333
C31_2	-0.2856(5)	0.6732(3)	0.7533(3)	0.277(8)
H31_2	-0.252018	0.679225	0.757347	0.333
C32_2	-0.2994(6)	0.6535(3)	0.7600(3)	0.293(12)
H32_2	-0.276478	0.646483	0.769343	0.352
C33_2	-0.3471(7)	0.64415(19)	0.7528(3)	0.326(9)
H33_2	-0.353956	0.630091	0.755495	0.391
C34_2	-0.3846(5)	0.65577(18)	0.7415(2)	0.326(9)
H34_2	-0.419686	0.650299	0.738659	0.391
C35_2	-0.4294(4)	0.69171(19)	0.66335(9)	0.130(5)
H35_2	-0.453901	0.698187	0.654136	0.156
C36_2	-0.3877(4)	0.6810(2)	0.65562(9)	0.145(6)

H36_2	-0.381920	0.680776	0.641250	0.174
C37_2	-0.3530(4)	0.6704(2)	0.66840(10)	0.154(6)
C38_2	-0.3633(4)	0.66995(18)	0.68912(10)	0.131(5)
H38_2	-0.342123	0.661106	0.697493	0.157
C39_2	-0.3065(3)	0.65838(16)	0.66024(12)	0.190(6)
C40_2	-0.2684(4)	0.66761(19)	0.64717(17)	0.341(11)
H40_2	-0.273852	0.683298	0.642448	0.409
C41_2	-0.2224(4)	0.6563(3)	0.6401(2)	0.341(11)
H41_2	-0.192625	0.663108	0.630058	0.409
C42_2	-0.2172(5)	0.6362(2)	0.6466(2)	0.250(9)
H42_2	-0.182517	0.627587	0.641293	0.300
C43_2	-0.2532(7)	0.62661(19)	0.6593(3)	0.372(11)
H43_2	-0.247022	0.610913	0.663826	0.446
C44_2	-0.2982(6)	0.63771(17)	0.6662(2)	0.372(11)
H44_2	-0.327215	0.630443	0.676221	0.446
P1_3	0.10852(8)	0.90226(4)	0.59135(3)	0.0577(7)
O1_3	0.09017(17)	0.90041(8)	0.61321(5)	0.0496(16)
O2_3	0.07334(16)	0.91376(7)	0.57660(6)	0.0592(18)
O3_3	0.11921(15)	0.88064(5)	0.58102(6)	0.0591(13)
O4_3	0.16777(12)	0.91211(6)	0.59246(5)	0.0511(14)
C1_3	0.2221(2)	0.89245(8)	0.56957(7)	0.062(2)
C2_3	0.21473(17)	0.87443(8)	0.58361(8)	0.054(2)
C3_3	0.16335(17)	0.86944(7)	0.58896(8)	0.053(2)
C4_3	0.1495(2)	0.85388(9)	0.60304(10)	0.072(3)
H4_3	0.112477	0.850910	0.606187	0.086
C5_3	0.1919(2)	0.84304(11)	0.61213(11)	0.0762(16)
C6_3	0.2466(2)	0.84708(10)	0.60686(10)	0.0764(17)
C7_3	0.25862(18)	0.86337(9)	0.59255(9)	0.073(3)
C8_3	0.1774(3)	0.82741(10)	0.62841(8)	0.0768(18)
C9_3	0.1957(4)	0.83044(12)	0.64888(10)	0.102(4)
H9_3	0.219571	0.841417	0.652150	0.122
C10_3	0.1774(5)	0.81664(15)	0.66438(9)	0.127(3)
H10_3	0.188851	0.817907	0.678216	0.153
C11_3	0.1423(5)	0.80138(16)	0.65816(13)	0.129(3)
H11_3	0.129131	0.792583	0.668626	0.154
C12_3	0.1247(5)	0.79746(15)	0.63947(14)	0.129(3)
H12_3	0.101757	0.786130	0.636496	0.155
C13_3	0.1428(5)	0.81156(14)	0.62385(11)	0.129(5)
H13_3	0.130581	0.809811	0.610166	0.155
C14_3	0.2899(2)	0.83551(10)	0.61482(10)	0.0775(18)
H14_3	0.282391	0.824857	0.624315	0.093
C15_3	0.3431(2)	0.83928(11)	0.60917(11)	0.080(2)
C16_3	0.3543(2)	0.85507(12)	0.59585(12)	0.086(2)
H16_3	0.390970	0.858137	0.592251	0.104
C17_3	0.3130(2)	0.86616(11)	0.58794(10)	0.086(2)
H17_3	0.322018	0.876680	0.578482	0.103
C18_3	0.3883(3)	0.82583(13)	0.61720(10)	0.132(5)
C19_3	0.3888(3)	0.81882(15)	0.63762(10)	0.118(3)
H19_3	0.362406	0.823854	0.646983	0.141
C20_3	0.4277(4)	0.80452(15)	0.64434(11)	0.119(2)
H20_3	0.427849	0.799869	0.658119	0.142
C21_3	0.4656(4)	0.79740(16)	0.63046(14)	0.121(2)
H21_3	0.491443	0.787498	0.634879	0.145
C22_3	0.4676(4)	0.80407(17)	0.61035(14)	0.121(2)

H22_3	0.495697	0.799561	0.601415	0.145
C23_3	0.4272(4)	0.81763(17)	0.60349(11)	0.121(3)
H23_3	0.426022	0.821367	0.589447	0.145
C24_3	0.2019(2)	0.91058(7)	0.57519(8)	0.055(2)
C25_3	0.2110(4)	0.92896(7)	0.56435(10)	0.081(3)
H25_3	0.195702	0.941467	0.569087	0.097
C26_3	0.2419(4)	0.92860(9)	0.54701(12)	0.123(4)
C27_3	0.2645(4)	0.91017(8)	0.53975(10)	0.089(3)
C28_3	0.2548(3)	0.89159(8)	0.55126(8)	0.075(2)
C29_3	0.2576(4)	0.94860(9)	0.53800(13)	0.168(5)
C30_3	0.2202(4)	0.96105(15)	0.5277(2)	0.221(7)
H30_3	0.183237	0.956785	0.526614	0.265
C31_3	0.2362(6)	0.97969(16)	0.5190(3)	0.221(7)
H31_3	0.210580	0.987898	0.511845	0.265
C32_3	0.2904(7)	0.98615(14)	0.52094(16)	0.236(9)
H32_3	0.301199	0.999090	0.515661	0.283
C33_3	0.3288(5)	0.97358(19)	0.5306(3)	0.222(6)
H33_3	0.366033	0.977552	0.531185	0.266
C34_3	0.3119(4)	0.95512(17)	0.5395(2)	0.223(6)
H34_3	0.337570	0.946907	0.546667	0.267
C35_3	0.2713(4)	0.87273(9)	0.54180(10)	0.077(3)
H35_3	0.260705	0.860204	0.548040	0.092
C36_3	0.3008(4)	0.87199(9)	0.52460(11)	0.078(3)
H36_3	0.310569	0.859118	0.518893	0.094
C37_3	0.3171(3)	0.88985(9)	0.51513(9)	0.072(2)
C38_3	0.2944(4)	0.90819(9)	0.52127(11)	0.086(3)
H38_3	0.298966	0.919902	0.512823	0.103
C39_3	0.3531(2)	0.88950(11)	0.49689(8)	0.093(3)
C40_3	0.3468(3)	0.87410(11)	0.48208(9)	0.099(3)
H40_3	0.318160	0.864395	0.483351	0.119
C41_3	0.3834(3)	0.87300(13)	0.46514(9)	0.098(3)
H41_3	0.379652	0.862675	0.454987	0.118
C42_3	0.4244(3)	0.88749(16)	0.46402(12)	0.129(5)
H42_3	0.448764	0.886794	0.452757	0.155
C43_3	0.4321(3)	0.90267(14)	0.47799(13)	0.130(4)
H43_3	0.460930	0.912255	0.476504	0.156
C44_3	0.3963(3)	0.90376(12)	0.49459(12)	0.130(4)
H44_3	0.400936	0.914243	0.504516	0.155
P1_5	-0.02186(8)	0.60166(4)	0.63670(3)	0.0564(7)
O1_5	0.00543(19)	0.62170(5)	0.64162(7)	0.0548(14)
O2_5	-0.0272(2)	0.59559(8)	0.61474(5)	0.0594(17)
O3_5	-0.08127(13)	0.60019(7)	0.64614(5)	0.0593(17)
O4_5	0.01144(15)	0.58513(6)	0.64945(6)	0.0624(15)
C1_5	-0.0541(2)	0.56634(7)	0.66895(8)	0.069(2)
C2_5	-0.0700(3)	0.58593(7)	0.67946(7)	0.078(3)
C3_5	-0.0849(2)	0.60182(7)	0.66754(7)	0.061(2)
C4_5	-0.1027(3)	0.62102(8)	0.67489(8)	0.073(2)
H4_5	-0.112681	0.631695	0.665753	0.088
C5_5	-0.1052(4)	0.62375(10)	0.69599(8)	0.112(2)
C6_5	-0.0902(4)	0.60781(9)	0.70943(8)	0.113(2)
C7_5	-0.0731(3)	0.58831(8)	0.70109(6)	0.081(3)
C8_5	-0.1236(4)	0.64448(9)	0.70387(11)	0.113(2)
C9_5	-0.0845(4)	0.65808(15)	0.7125(2)	0.218(10)
H9_5	-0.047359	0.654121	0.713802	0.262

C10_5	-0.1023(6)	0.67777(15)	0.7193(3)	0.230(6)
H10_5	-0.077802	0.687419	0.725134	0.275
C11_5	-0.1569(6)	0.68199(15)	0.7168(3)	0.229(6)
H11_5	-0.168582	0.695037	0.721560	0.275
C12_5	-0.1951(5)	0.67027(19)	0.7088(3)	0.229(6)
H12_5	-0.231770	0.674709	0.707350	0.275
C13_5	-0.1772(4)	0.65019(18)	0.7023(3)	0.264(14)
H13_5	-0.202975	0.640803	0.696877	0.317
C14_5	-0.0888(4)	0.61058(12)	0.73071(8)	0.114(2)
H14_5	-0.099811	0.623350	0.736330	0.137
C15_5	-0.0718(6)	0.59518(14)	0.74366(8)	0.287(14)
C16_5	-0.0558(5)	0.57661(12)	0.73564(8)	0.118(3)
H16_5	-0.044330	0.565850	0.744430	0.141
C17_5	-0.0566(4)	0.57384(11)	0.71519(8)	0.118(3)
H17_5	-0.044898	0.560940	0.710095	0.141
C18_5	-0.0687(5)	0.59906(15)	0.76639(7)	0.256(3)
C19_5	-0.0483(5)	0.61757(16)	0.77433(14)	0.2792(11)
H19_5	-0.037894	0.628195	0.765193	0.335
C20_5	-0.0430(6)	0.6206(2)	0.79548(15)	0.2792(11)
H20_5	-0.028976	0.633107	0.800736	0.335
C21_5	-0.0585(7)	0.6049(3)	0.80851(10)	0.2790(14)
H21_5	-0.055048	0.606942	0.822856	0.335
C22_5	-0.0791(7)	0.5865(2)	0.80144(10)	0.2792(11)
H22_5	-0.089635	0.575987	0.810735	0.335
C23_5	-0.0839(7)	0.58357(18)	0.78029(12)	0.2792(11)
H23_5	-0.097678	0.570956	0.775212	0.335
C24_5	-0.0136(2)	0.56668(6)	0.65529(8)	0.071(2)
C25_5	0.0091(3)	0.54896(7)	0.64631(12)	0.082(3)
H25_5	0.036764	0.550152	0.636189	0.098
C26_5	-0.0089(3)	0.53014(8)	0.65226(13)	0.092(3)
C27_5	-0.0527(3)	0.52824(7)	0.66597(15)	0.099(4)
C28_5	-0.0754(3)	0.54664(7)	0.67492(11)	0.086(3)
C29_5	0.0236(3)	0.51210(8)	0.64576(11)	0.122(4)
C30_5	0.0292(3)	0.50666(11)	0.62516(11)	0.126(3)
H30_5	0.012955	0.514967	0.614901	0.151
C31_5	0.0583(4)	0.48914(13)	0.61947(14)	0.127(3)
H31_5	0.062096	0.485649	0.605429	0.152
C32_5	0.0819(4)	0.47680(11)	0.63456(18)	0.130(4)
H32_5	0.101364	0.464802	0.630776	0.155
C33_5	0.0770(5)	0.48210(14)	0.65522(17)	0.156(4)
H33_5	0.092936	0.473720	0.665489	0.188
C34_5	0.0484(5)	0.49982(14)	0.66065(12)	0.156(4)
H34_5	0.045787	0.503585	0.674658	0.187
C35_5	-0.1246(3)	0.54410(8)	0.68654(15)	0.088(3)
H35_5	-0.144165	0.556013	0.690539	0.106
C36_5	-0.1444(3)	0.52580(8)	0.69203(14)	0.078(3)
H36_5	-0.175893	0.525012	0.700443	0.093
C37_5	-0.1192(3)	0.50808(8)	0.68553(14)	0.091(3)
C38_5	-0.0760(4)	0.50935(8)	0.67212(17)	0.109(4)
H38_5	-0.061083	0.497025	0.666769	0.131
C39_5	-0.1392(3)	0.48750(8)	0.69159(12)	0.116(5)
C40_5	-0.1952(2)	0.48409(10)	0.69448(12)	0.1075(10)
H40_5	-0.220437	0.494833	0.692031	0.129
C41_5	-0.2142(3)	0.46454(11)	0.70109(13)	0.1075(10)

H41_5	-0.251927	0.462002	0.703118	0.129
C42_5	-0.1757(3)	0.44940(11)	0.70445(13)	0.091(3)
H42_5	-0.188236	0.436356	0.708849	0.109
C43_5	-0.1211(3)	0.45201(9)	0.70185(14)	0.1075(10)
H43_5	-0.096365	0.441104	0.704368	0.129
C44_5	-0.1025(3)	0.47115(11)	0.69538(14)	0.1075(10)
H44_5	-0.064495	0.473269	0.693485	0.129
P1_6	-0.49887(11)	0.80741(5)	0.72063(5)	0.1182(14)
O1_6	-0.54944(19)	0.80162(11)	0.73220(9)	0.133(4)
O2_6	-0.44684(19)	0.79644(10)	0.72546(9)	0.131(4)
O3_6	-0.48517(18)	0.83106(6)	0.72250(7)	0.112(2)
O4_6	-0.51405(17)	0.80457(7)	0.69714(6)	0.107(2)
C1_6	-0.4750(2)	0.83327(8)	0.68035(8)	0.066(2)
C2_6	-0.5153(2)	0.84657(9)	0.69140(7)	0.066(2)
C3_6	-0.5194(2)	0.84462(8)	0.71187(7)	0.081(3)
C4_6	-0.5562(3)	0.85571(11)	0.72425(8)	0.089(3)
H4_6	-0.557226	0.853814	0.738657	0.107
C5_6	-0.5910(3)	0.86951(11)	0.71460(8)	0.0710(16)
C6_6	-0.5879(2)	0.87282(10)	0.69329(7)	0.0717(16)
C7_6	-0.5513(2)	0.86032(9)	0.68125(7)	0.065(2)
C8_6	-0.6320(2)	0.88078(9)	0.72785(9)	0.0712(18)
C9_6	-0.6779(3)	0.87000(11)	0.73574(14)	0.132(5)
H9_6	-0.683744	0.856000	0.732425	0.159
C10_6	-0.7150(3)	0.88072(13)	0.74873(16)	0.106(3)
H10_6	-0.746053	0.874201	0.754463	0.127
C11_6	-0.7033(4)	0.90100(14)	0.75254(16)	0.106(3)
H11_6	-0.728726	0.908124	0.760892	0.128
C12_6	-0.6612(4)	0.91178(10)	0.74602(16)	0.107(3)
H12_6	-0.655828	0.925689	0.749766	0.129
C13_6	-0.6239(3)	0.90094(11)	0.73279(16)	0.102(4)
H13_6	-0.593107	0.907933	0.727402	0.122
C14_6	-0.6255(2)	0.88537(10)	0.68302(8)	0.0704(18)
H14_6	-0.649652	0.893703	0.690760	0.084
C15_6	-0.6281(3)	0.88587(11)	0.66196(8)	0.069(2)
C16_6	-0.5929(3)	0.87397(12)	0.65062(8)	0.067(2)
H16_6	-0.594185	0.874329	0.636092	0.081
C17_6	-0.5567(3)	0.86181(11)	0.66028(8)	0.068(2)
H17_6	-0.533234	0.853684	0.652053	0.082
C18_6	-0.6681(2)	0.89985(9)	0.65139(9)	0.076(2)
C19_6	-0.6751(3)	0.92042(10)	0.65741(11)	0.094(2)
H19_6	-0.652430	0.925985	0.667804	0.113
C20_6	-0.7151(3)	0.93279(10)	0.64826(14)	0.094(2)
H20_6	-0.719455	0.946694	0.652234	0.113
C21_6	-0.7480(3)	0.92429(13)	0.63338(15)	0.094(2)
H21_6	-0.775940	0.932506	0.627524	0.113
C22_6	-0.7421(3)	0.90431(13)	0.62659(13)	0.094(2)
H22_6	-0.764310	0.899111	0.615864	0.113
C23_6	-0.7026(3)	0.89195(11)	0.63595(13)	0.093(2)
H23_6	-0.698966	0.878051	0.631868	0.112
C24_6	-0.4787(2)	0.81297(7)	0.68238(8)	0.075(3)
C25_6	-0.4444(3)	0.79879(8)	0.67239(13)	0.089(3)
H25_6	-0.447286	0.784556	0.675185	0.106
C26_6	-0.4069(3)	0.80584(8)	0.65868(12)	0.079(3)
C27_6	-0.3992(3)	0.82700(8)	0.65581(12)	0.072(3)

C28_6	-0.4340(2)	0.84117(7)	0.66670(10)	0.061(2)
C29_6	-0.3723(3)	0.79056(10)	0.64807(12)	0.138(4)
C30_6	-0.3353(6)	0.7781(2)	0.65863(18)	0.316(9)
H30_6	-0.331882	0.779438	0.673048	0.380
C31_6	-0.3032(7)	0.7638(3)	0.6482(3)	0.316(9)
H31_6	-0.277831	0.755540	0.655518	0.380
C32_6	-0.3086(6)	0.7616(2)	0.6270(3)	0.308(12)
H32_6	-0.287651	0.751650	0.619922	0.370
C33_6	-0.3448(7)	0.7742(3)	0.61619(18)	0.244(7)
H33_6	-0.347884	0.772993	0.601743	0.293
C34_6	-0.3765(6)	0.7885(2)	0.62680(13)	0.244(7)
H34_6	-0.401127	0.796994	0.619458	0.293
C35_6	-0.4219(3)	0.86256(8)	0.66445(15)	0.074(3)
H35_6	-0.444069	0.872185	0.671501	0.089
C36_6	-0.3809(3)	0.86961(9)	0.65292(13)	0.062(2)
H36_6	-0.374126	0.883920	0.652252	0.075
C37_6	-0.3485(2)	0.85628(8)	0.64200(11)	0.057(2)
C38_6	-0.3596(3)	0.83558(8)	0.64255(13)	0.071(3)
H38_6	-0.339929	0.826728	0.633686	0.085
C39_6	-0.2998(2)	0.86321(9)	0.63043(9)	0.076(3)
C40_6	-0.2667(2)	0.87896(10)	0.63805(11)	0.096(3)
H40_6	-0.273561	0.884511	0.651242	0.115
C41_6	-0.2227(3)	0.88668(11)	0.62603(13)	0.097(3)
H41_6	-0.199867	0.897346	0.630962	0.116
C42_6	-0.2145(3)	0.87806(14)	0.60694(14)	0.130(5)
H42_6	-0.185211	0.883155	0.598892	0.157
C43_6	-0.2455(3)	0.86276(13)	0.59893(10)	0.094(3)
H43_6	-0.238104	0.857373	0.585712	0.113
C44_6	-0.2886(3)	0.85520(11)	0.61069(10)	0.093(3)
H44_6	-0.310745	0.844519	0.605385	0.112
O1S_7	0.0057(3)	0.58930(11)	0.56890(10)	0.091(2)
C2S_7	-0.0338(7)	0.5742(3)	0.5651(3)	0.234(8)
H2SA_7	-0.021291	0.565784	0.553391	0.281
H2SB_7	-0.067896	0.581096	0.560857	0.281
C3S_7	-0.0471(12)	0.5599(4)	0.5828(4)	0.302(15)
H3SA_7	-0.074902	0.550010	0.578437	0.453
H3SB_7	-0.014019	0.552490	0.586880	0.453
H3SC_7	-0.060776	0.567852	0.594370	0.453
C4S_7	0.0606(5)	0.5854(2)	0.5609(2)	0.152(5)
H4SA_7	0.063727	0.570786	0.557181	0.182
H4SB_7	0.087551	0.588235	0.571863	0.182
C5S_7	0.0740(5)	0.5985(3)	0.5424(2)	0.184(8)
H5SA_7	0.111024	0.595337	0.537651	0.275
H5SB_7	0.047964	0.595565	0.531379	0.275
H5SC_7	0.071793	0.613019	0.546064	0.275
O1S_8	0.0052(3)	0.95132(11)	0.56281(10)	0.092(2)
C2S_8	-0.0410(5)	0.95684(19)	0.5514(2)	0.134(5)
H2SA_8	-0.031496	0.968811	0.542795	0.161
H2SB_8	-0.069860	0.961253	0.561009	0.161
C3S_8	-0.0646(10)	0.9403(3)	0.5375(4)	0.301(14)
H3SA_8	-0.096454	0.945724	0.530316	0.452
H3SB_8	-0.036941	0.936068	0.527565	0.452
H3SC_8	-0.075420	0.928487	0.545834	0.452
C4S_8	0.0565(4)	0.9616(2)	0.5575(2)	0.141(4)

H4SA_8	0.065196	0.958762	0.543021	0.170
H4SB_8	0.086178	0.955791	0.566016	0.170
C5S_8	0.0540(9)	0.9846(2)	0.5608(4)	0.245(11)
H5SA_8	0.089128	0.990713	0.557102	0.367
H5SB_8	0.025186	0.990451	0.552251	0.367
H5SC_8	0.046171	0.987480	0.575249	0.367
P1_9	-0.03637(9)	0.88510(4)	0.57786(3)	0.0555(7)
O1_9	-0.00117(19)	0.87915(7)	0.59574(6)	0.0552(15)
O2_9	-0.0444(2)	0.90735(5)	0.57340(8)	0.0626(17)
O3_9	-0.09570(13)	0.87553(6)	0.57934(7)	0.0614(16)
O4_9	-0.01023(14)	0.87364(6)	0.55873(5)	0.0500(14)
C1_9	-0.0831(2)	0.85617(8)	0.54226(7)	0.062(2)
C2_9	-0.0897(3)	0.84380(7)	0.56149(7)	0.061(2)
C3_9	-0.0985(2)	0.85403(7)	0.57909(7)	0.065(2)
C4_9	-0.1069(3)	0.84465(8)	0.59831(8)	0.089(3)
H4_9	-0.112286	0.852608	0.610315	0.106
C5_9	-0.1071(5)	0.82332(8)	0.59903(9)	0.114(2)
C6_9	-0.0972(4)	0.81159(8)	0.58129(8)	0.114(2)
C7_9	-0.0873(3)	0.82205(7)	0.56213(8)	0.075(2)
C8_9	-0.1191(4)	0.81344(11)	0.61956(9)	0.117(2)
C9_9	-0.0776(4)	0.80187(18)	0.62946(15)	0.167(6)
H9_9	-0.044352	0.798838	0.622620	0.200
C10_9	-0.0869(6)	0.7949(2)	0.64988(15)	0.219(6)
H10_9	-0.060296	0.787282	0.657205	0.263
C11_9	-0.1365(6)	0.7999(3)	0.65836(15)	0.219(6)
H11_9	-0.142986	0.794858	0.671773	0.262
C12_9	-0.1763(6)	0.8108(3)	0.65024(16)	0.218(6)
H12_9	-0.208812	0.813937	0.657500	0.262
C13_9	-0.1669(4)	0.8178(2)	0.62953(16)	0.170(7)
H13_9	-0.194343	0.825470	0.622716	0.204
C14_9	-0.0936(4)	0.79013(8)	0.58171(10)	0.114(3)
H14_9	-0.099999	0.783081	0.594193	0.137
C15_9	-0.0808(4)	0.77908(8)	0.56438(11)	0.117(4)
C16_9	-0.0712(4)	0.78920(9)	0.54622(11)	0.098(3)
H16_9	-0.061960	0.781717	0.534244	0.118
C17_9	-0.0749(3)	0.80978(9)	0.54558(10)	0.098(3)
H17_9	-0.068590	0.816310	0.532813	0.118
C18_9	-0.0800(4)	0.75598(7)	0.56489(13)	0.160(5)
C19_9	-0.0529(4)	0.74506(12)	0.58046(14)	0.154(3)
H19_9	-0.038287	0.752278	0.591830	0.185
C20_9	-0.0471(5)	0.72368(12)	0.57943(19)	0.154(3)
H20_9	-0.029160	0.716357	0.590048	0.185
C21_9	-0.0680(5)	0.71356(10)	0.5626(2)	0.156(3)
H21_9	-0.063218	0.699146	0.561728	0.187
C22_9	-0.0957(5)	0.72349(12)	0.54705(19)	0.157(3)
H22_9	-0.110551	0.716009	0.535900	0.189
C23_9	-0.1013(5)	0.74482(12)	0.54812(15)	0.158(3)
H23_9	-0.119594	0.751895	0.537437	0.189
C24_9	-0.0435(2)	0.87020(8)	0.54150(6)	0.058(2)
C25_9	-0.0353(3)	0.88368(11)	0.52490(8)	0.062(2)
H25_9	-0.007899	0.893945	0.525554	0.075
C26_9	-0.0671(3)	0.88177(14)	0.50792(10)	0.104(4)
C27_9	-0.1101(3)	0.86751(12)	0.50731(9)	0.082(3)
C28_9	-0.1187(3)	0.85435(11)	0.52484(8)	0.079(3)

C29_9	-0.0569(3)	0.89614(12)	0.49077(10)	0.131(4)
C30_9	-0.0062(3)	0.89732(16)	0.48099(15)	0.184(5)
H30_9	0.021740	0.887908	0.484583	0.221
C31_9	0.0039(5)	0.9122(2)	0.46598(18)	0.185(5)
H31_9	0.038309	0.912695	0.459338	0.222
C32_9	-0.0370(6)	0.92619(18)	0.46082(18)	0.182(6)
H32_9	-0.029996	0.936549	0.450962	0.218
C33_9	-0.0883(5)	0.92498(17)	0.4702(2)	0.169(5)
H33_9	-0.116537	0.934142	0.466313	0.203
C34_9	-0.0976(4)	0.91017(17)	0.48528(17)	0.169(5)
H34_9	-0.131997	0.909632	0.491913	0.203
C35_9	-0.1658(3)	0.84135(14)	0.52435(11)	0.088(3)
H35_9	-0.172838	0.832780	0.535797	0.106
C36_9	-0.2004(3)	0.84074(16)	0.50852(11)	0.096(4)
H36_9	-0.233260	0.833108	0.509580	0.115
C37_9	-0.1886(3)	0.85112(15)	0.49068(11)	0.104(4)
C38_9	-0.1447(3)	0.86430(15)	0.49020(10)	0.083(3)
H38_9	-0.137260	0.871533	0.477896	0.100
C39_9	-0.2261(3)	0.85053(11)	0.47285(9)	0.100(3)
C40_9	-0.2826(3)	0.84901(11)	0.47588(12)	0.133(4)
H40_9	-0.296914	0.848478	0.489374	0.160
C41_9	-0.3185(3)	0.84825(13)	0.45874(15)	0.134(4)
H41_9	-0.356835	0.847224	0.460534	0.161
C42_9	-0.2956(4)	0.84907(15)	0.43942(14)	0.159(6)
H42_9	-0.319406	0.848575	0.427973	0.191
C43_9	-0.2411(4)	0.85055(16)	0.43574(9)	0.138(4)
H43_9	-0.227431	0.851062	0.422151	0.165
C44_9	-0.2059(3)	0.85129(14)	0.45253(10)	0.138(4)
H44_9	-0.167737	0.852328	0.450286	0.166
P1_10	0.05073(9)	0.94344(4)	0.62891(4)	0.0623(7)
O1_10	0.02107(19)	0.92509(6)	0.63727(7)	0.0602(16)
O2_10	0.0478(2)	0.94755(9)	0.60646(5)	0.0681(19)
O3_10	0.11368(13)	0.94297(7)	0.63439(6)	0.0693(18)
O4_10	0.02787(16)	0.96221(6)	0.64182(7)	0.0724(18)
C1_10	0.1062(2)	0.98006(7)	0.65360(9)	0.088(3)
C2_10	0.1222(3)	0.96139(7)	0.66562(7)	0.071(2)
C3_10	0.1266(2)	0.94359(7)	0.65531(7)	0.063(2)
C4_10	0.1419(3)	0.92470(8)	0.66411(8)	0.069(2)
H4_10	0.144499	0.912633	0.656053	0.083
C5_10	0.1530(3)	0.92436(8)	0.68501(8)	0.0685(15)
C6_10	0.1516(3)	0.94258(7)	0.69661(7)	0.0680(15)
C7_10	0.1329(3)	0.96130(7)	0.68702(7)	0.065(2)
C8_10	0.1701(3)	0.90403(7)	0.69421(9)	0.0707(17)
C9_10	0.1365(3)	0.89468(10)	0.70936(10)	0.072(3)
H9_10	0.106693	0.901896	0.715169	0.086
C10_10	0.1487(4)	0.87424(10)	0.71562(12)	0.098(3)
H10_10	0.127479	0.867326	0.725629	0.117
C11_10	0.1929(4)	0.86508(11)	0.70637(15)	0.098(2)
H11_10	0.201649	0.851671	0.710981	0.117
C12_10	0.2247(4)	0.87263(11)	0.69190(15)	0.098(3)
H12_10	0.252503	0.864758	0.685542	0.118
C13_10	0.2141(3)	0.89369(11)	0.68642(13)	0.081(3)
H13_10	0.238089	0.900498	0.677246	0.097
C14_10	0.1652(3)	0.94307(9)	0.71747(8)	0.0671(17)

H14_10	0.177786	0.930889	0.723883	0.080
C15_10	0.1609(3)	0.96074(9)	0.72891(8)	0.081(3)
C16_10	0.1428(4)	0.97846(10)	0.71963(9)	0.095(3)
H16_10	0.139346	0.990727	0.727334	0.114
C17_10	0.1300(3)	0.97826(9)	0.69956(8)	0.096(3)
H17_10	0.118195	0.990764	0.693594	0.115
C18_10	0.1824(3)	0.96134(11)	0.75053(7)	0.108(3)
C19_10	0.2313(3)	0.95148(12)	0.75612(10)	0.133(3)
H19_10	0.250319	0.943565	0.746230	0.159
C20_10	0.2524(3)	0.95314(14)	0.77602(12)	0.133(3)
H20_10	0.285371	0.946458	0.779685	0.160
C21_10	0.2241(4)	0.96473(16)	0.79015(10)	0.135(3)
H21_10	0.238226	0.965887	0.803631	0.162
C22_10	0.1758(4)	0.97474(15)	0.78549(9)	0.135(3)
H22_10	0.157313	0.982597	0.795555	0.163
C23_10	0.1549(3)	0.97302(13)	0.76558(10)	0.136(3)
H23_10	0.121901	0.979797	0.762155	0.163
C24_10	0.0591(2)	0.98019(7)	0.64342(10)	0.073(3)
C25_10	0.0394(3)	0.99705(10)	0.63206(19)	0.144(7)
H25_10	0.004544	0.996501	0.625747	0.172
C26_10	0.0710(4)	1.01413(12)	0.6303(2)	0.212(9)
C27_10	0.1213(3)	1.01547(11)	0.64053(19)	0.144(6)
C28_10	0.1399(3)	0.99809(9)	0.65237(13)	0.096(4)
C29_10	0.0495(3)	1.03131(12)	0.61782(17)	0.245(8)
C30_10	0.0011(4)	1.04147(17)	0.62286(19)	0.217(6)
H30_10	-0.019131	1.037197	0.634511	0.260
C31_10	-0.0181(5)	1.05783(18)	0.6110(2)	0.217(6)
H31_10	-0.051147	1.064539	0.614547	0.260
C32_10	0.0118(7)	1.06422(19)	0.5939(2)	0.241(9)
H32_10	-0.000608	1.075503	0.585972	0.289
C33_10	0.0598(7)	1.0540(2)	0.5884(2)	0.245(7)
H33_10	0.079453	1.058037	0.576566	0.294
C34_10	0.0787(5)	1.0378(2)	0.6006(2)	0.245(7)
H34_10	0.111908	1.031202	0.597094	0.294
C35_10	0.1953(3)	0.99866(12)	0.65944(16)	0.098(4)
H35_10	0.209670	0.986860	0.666028	0.117
C36_10	0.2277(3)	1.01499(13)	0.65721(19)	0.120(4)
H36_10	0.262772	1.015135	0.663371	0.144
C37_10	0.2102(3)	1.03162(14)	0.6460(2)	0.148(6)
C38_10	0.1565(3)	1.03268(11)	0.6398(2)	0.142(6)
H38_10	0.142631	1.045363	0.634975	0.171
C39_10	0.2466(3)	1.04918(11)	0.64137(13)	0.161(5)
C40_10	0.3026(3)	1.04597(15)	0.63840(13)	0.185(5)
H40_10	0.318011	1.032857	0.640924	0.222
C41_10	0.3365(3)	1.06236(19)	0.63160(15)	0.185(5)
H41_10	0.374529	1.060441	0.629503	0.222
C42_10	0.3121(5)	1.08114(18)	0.62815(18)	0.294(13)
H42_10	0.334593	1.092090	0.623627	0.352
C43_10	0.2581(5)	1.08497(12)	0.6308(2)	0.198(5)
H43_10	0.243330	1.098174	0.628231	0.237
C44_10	0.2249(4)	1.06893(12)	0.63749(17)	0.197(5)
H44_10	0.187015	1.071288	0.639450	0.236
O1S_11	0.0077(3)	0.66848(11)	0.64231(10)	0.085(2)
C2S_11	-0.0280(6)	0.6853(2)	0.6415(3)	0.199(7)

H2SA_11	-0.038302	0.687621	0.627065	0.239
H2SB_11	-0.007965	0.697606	0.646226	0.239
C3S_11	-0.0799(6)	0.6834(3)	0.6541(3)	0.171(7)
H3SA_11	-0.101803	0.695838	0.652522	0.256
H3SB_11	-0.100890	0.671535	0.649355	0.256
H3SC_11	-0.070465	0.681549	0.668571	0.256
C4S_11	0.0526(7)	0.6697(4)	0.6570(3)	0.260(11)
H4SA_11	0.081486	0.678654	0.651416	0.311
H4SB_11	0.068392	0.655853	0.658901	0.311
C5S_11	0.0350(13)	0.6779(8)	0.6778(3)	0.56(3)
H5SA_11	0.066525	0.678400	0.686982	0.847
H5SB_11	0.020106	0.691760	0.676121	0.847
H5SC_11	0.007006	0.668947	0.683609	0.847
O1S_12	0.0099(2)	0.87857(10)	0.64261(9)	0.0742(18)
C2S_12	-0.0204(5)	0.86029(18)	0.6425(2)	0.129(4)
H2SA_12	0.003209	0.849137	0.647656	0.154
H2SB_12	-0.029814	0.856988	0.628137	0.154
C3S_12	-0.0727(7)	0.8604(3)	0.6550(3)	0.225(9)
H3SA_12	-0.090565	0.846987	0.653878	0.337
H3SB_12	-0.064103	0.863152	0.669378	0.337
H3SC_12	-0.097226	0.871026	0.649801	0.337
C4S_12	0.0498(4)	0.88100(18)	0.65909(16)	0.096(3)
H4SA_12	0.031684	0.878511	0.672396	0.115
H4SB_12	0.063424	0.895275	0.659082	0.115
C5S_12	0.0978(4)	0.8664(2)	0.65690(18)	0.098(4)
H5SA_12	0.123393	0.868600	0.668223	0.146
H5SB_12	0.084674	0.852252	0.657190	0.146
H5SC_12	0.116415	0.869016	0.643876	0.146
P1_13	0.11439(8)	0.62047(3)	0.61471(3)	0.0482(6)
O1_13	0.09181(18)	0.64119(6)	0.61981(8)	0.0538(16)
O2_13	0.08074(17)	0.60633(7)	0.60182(7)	0.0494(15)
O3_13	0.13033(14)	0.60757(6)	0.63440(5)	0.0492(14)
O4_13	0.17196(12)	0.62461(5)	0.60424(6)	0.0470(13)
C1_13	0.23119(19)	0.59863(7)	0.61681(7)	0.0483(19)
C2_13	0.22759(17)	0.60861(10)	0.63749(7)	0.061(3)
C3_13	0.17789(17)	0.61358(9)	0.64475(7)	0.054(2)
C4_13	0.1684(2)	0.62335(13)	0.66371(8)	0.078(3)
H4_13	0.132546	0.626735	0.668003	0.094
C5_13	0.2134(2)	0.62784(18)	0.67592(11)	0.140(3)
C6_13	0.2667(2)	0.62339(15)	0.66916(9)	0.139(3)
C7_13	0.27419(18)	0.61412(11)	0.64925(8)	0.063(2)
C8_13	0.2019(3)	0.63571(14)	0.69735(9)	0.142(3)
C9_13	0.2057(4)	0.62191(16)	0.71419(13)	0.230(9)
H9_13	0.220996	0.608655	0.712472	0.277
C10_13	0.1859(5)	0.6284(2)	0.73368(11)	0.247(6)
H10_13	0.187494	0.619781	0.745370	0.297
C11_13	0.1645(5)	0.6479(2)	0.73460(14)	0.248(6)
H11_13	0.151376	0.652119	0.747615	0.297
C12_13	0.1597(6)	0.66142(19)	0.71969(18)	0.247(6)
H12_13	0.144277	0.674595	0.721783	0.296
C13_13	0.1798(5)	0.65480(15)	0.69997(14)	0.220(8)
H13_13	0.177519	0.663853	0.688621	0.264
C14_13	0.3132(2)	0.62885(17)	0.68046(11)	0.139(3)
H14_13	0.308770	0.634935	0.693558	0.167

C15_13	0.3650(2)	0.62558(16)	0.67301(12)	0.114(4)
C16_13	0.3718(2)	0.61665(13)	0.65406(11)	0.080(2)
H16_13	0.407329	0.614181	0.648837	0.096
C17_13	0.32748(19)	0.61144(11)	0.64304(10)	0.079(2)
H17_13	0.333380	0.605468	0.629969	0.095
C18_13	0.4141(2)	0.6330(2)	0.68479(13)	0.201(7)
C19_13	0.4109(4)	0.6379(3)	0.70577(14)	0.242(5)
H19_13	0.376206	0.638857	0.712144	0.290
C20_13	0.4582(5)	0.6414(3)	0.71740(15)	0.241(4)
H20_13	0.455819	0.644751	0.731511	0.290
C21_13	0.5081(4)	0.6397(3)	0.7078(2)	0.240(4)
H21_13	0.540132	0.641963	0.715683	0.288
C22_13	0.5137(3)	0.6350(3)	0.6873(2)	0.238(4)
H22_13	0.548764	0.633809	0.681212	0.286
C23_13	0.4663(3)	0.6320(3)	0.67563(16)	0.237(5)
H23_13	0.469342	0.629336	0.661387	0.284
C24_13	0.20659(19)	0.60769(8)	0.60098(6)	0.0427(18)
C25_13	0.2075(3)	0.60001(9)	0.58079(7)	0.053(2)
H25_13	0.188435	0.606923	0.570186	0.064
C26_13	0.2360(3)	0.58259(10)	0.57665(8)	0.063(3)
C27_13	0.2648(3)	0.57235(9)	0.59220(7)	0.053(2)
C28_13	0.2614(2)	0.58013(8)	0.61291(7)	0.051(2)
C29_13	0.2386(3)	0.57583(9)	0.55499(6)	0.082(3)
C30_13	0.2235(4)	0.55604(9)	0.54899(10)	0.111(3)
H30_13	0.215650	0.546036	0.559129	0.133
C31_13	0.2198(4)	0.55079(12)	0.52826(11)	0.112(3)
H31_13	0.209773	0.537276	0.524382	0.134
C32_13	0.2309(6)	0.56554(16)	0.51330(8)	0.146(6)
H32_13	0.227017	0.562194	0.499221	0.175
C33_13	0.2476(5)	0.58522(14)	0.51904(8)	0.117(3)
H33_13	0.257810	0.594945	0.508920	0.140
C34_13	0.2492(5)	0.59046(10)	0.53981(9)	0.116(3)
H34_13	0.257547	0.604128	0.543664	0.139
C35_13	0.2887(3)	0.56842(10)	0.62841(8)	0.052(2)
H35_13	0.284997	0.572620	0.642264	0.062
C36_13	0.3191(3)	0.55190(10)	0.62438(8)	0.054(2)
H36_13	0.336412	0.544794	0.635270	0.065
C37_13	0.3255(3)	0.54509(10)	0.60454(8)	0.058(2)
C38_13	0.2984(3)	0.55498(9)	0.58895(8)	0.053(2)
H38_13	0.302356	0.549926	0.575393	0.064
C39_13	0.3600(2)	0.52708(8)	0.59952(9)	0.067(2)
C40_13	0.3597(3)	0.50976(9)	0.61224(10)	0.084(3)
H40_13	0.336506	0.509372	0.623881	0.101
C41_13	0.3941(3)	0.49274(8)	0.60771(12)	0.085(2)
H41_13	0.394354	0.480909	0.616171	0.102
C42_13	0.4271(3)	0.49407(12)	0.59060(14)	0.101(4)
H42_13	0.450036	0.482757	0.587548	0.121
C43_13	0.4287(3)	0.51054(12)	0.57784(11)	0.110(3)
H43_13	0.452078	0.510692	0.566256	0.132
C44_13	0.3950(3)	0.52719(11)	0.58226(10)	0.109(3)
H44_13	0.395581	0.538830	0.573538	0.131
P1_14	-0.10261(8)	0.63810(4)	0.59786(3)	0.0555(7)
O1_14	-0.07883(17)	0.64478(8)	0.61793(6)	0.0533(16)
O2_14	-0.06928(18)	0.62446(7)	0.58429(7)	0.0621(18)

O3_14	-0.12034(15)	0.65659(6)	0.58367(6)	0.0660(16)
O4_14	-0.15909(13)	0.62754(7)	0.60346(5)	0.0613(17)
C1_14	-0.2197(2)	0.63694(8)	0.57664(8)	0.065(2)
C2_14	-0.21790(18)	0.65850(8)	0.58465(10)	0.074(2)
C3_14	-0.16880(18)	0.66689(7)	0.58856(10)	0.084(3)
C4_14	-0.1608(2)	0.68706(8)	0.59585(14)	0.113(4)
H4_14	-0.125269	0.692124	0.598646	0.136
C5_14	-0.2066(2)	0.69925(10)	0.59878(18)	0.1286(14)
C6_14	-0.2594(2)	0.69133(8)	0.59548(14)	0.1250(10)
C7_14	-0.2654(2)	0.67031(8)	0.58888(13)	0.100(3)
C8_14	-0.1975(3)	0.72112(8)	0.60578(14)	0.1346(17)
C9_14	-0.1739(4)	0.72484(13)	0.62532(14)	0.148(2)
H9_14	-0.165259	0.713780	0.634185	0.178
C10_14	-0.1635(5)	0.74548(15)	0.63138(15)	0.150(2)
H10_14	-0.147508	0.748701	0.644229	0.180
C11_14	-0.1779(5)	0.76046(13)	0.6175(2)	0.150(2)
H11_14	-0.171964	0.774189	0.621810	0.179
C12_14	-0.1990(5)	0.75810(10)	0.59909(18)	0.148(2)
H12_14	-0.206379	0.769377	0.590335	0.178
C13_14	-0.2099(5)	0.73718(12)	0.59315(15)	0.148(2)
H13_14	-0.226053	0.734524	0.580211	0.178
C14_14	-0.3064(2)	0.70261(10)	0.60024(16)	0.1250(10)
H14_14	-0.302821	0.716400	0.604673	0.150
C15_14	-0.3577(2)	0.69413(12)	0.59863(17)	0.1250(10)
C16_14	-0.3631(2)	0.67402(12)	0.59252(17)	0.1250(10)
H16_14	-0.398194	0.667935	0.591598	0.150
C17_14	-0.3182(2)	0.66307(11)	0.58788(15)	0.1250(10)
H17_14	-0.323261	0.649296	0.583592	0.150
C18_14	-0.4067(3)	0.70591(16)	0.60617(19)	0.221(7)
C19_14	-0.4016(4)	0.7216(2)	0.6209(2)	0.262(5)
H19_14	-0.368451	0.722907	0.628329	0.315
C20_14	-0.4446(6)	0.7352(2)	0.6248(3)	0.263(5)
H20_14	-0.440863	0.745776	0.634745	0.316
C21_14	-0.4923(6)	0.7330(2)	0.6138(3)	0.263(5)
H21_14	-0.521631	0.742083	0.616609	0.315
C22_14	-0.4993(4)	0.7181(3)	0.5990(3)	0.263(5)
H22_14	-0.531966	0.717402	0.591252	0.315
C23_14	-0.4569(3)	0.7040(2)	0.5957(3)	0.262(5)
H23_14	-0.462058	0.693127	0.586272	0.315
C24_14	-0.1938(2)	0.62229(7)	0.58709(8)	0.066(2)
C25_14	-0.1931(3)	0.60138(7)	0.58160(10)	0.072(2)
H25_14	-0.173375	0.591738	0.589594	0.087
C26_14	-0.2210(4)	0.59526(9)	0.56466(11)	0.098(3)
C27_14	-0.2507(3)	0.60945(8)	0.55289(10)	0.079(2)
C28_14	-0.2493(3)	0.63087(8)	0.55862(8)	0.077(2)
C29_14	-0.2227(4)	0.57301(8)	0.56022(11)	0.149(2)
C30_14	-0.2047(5)	0.56490(12)	0.54156(13)	0.1866(11)
H30_14	-0.188872	0.573713	0.531644	0.224
C31_14	-0.2099(6)	0.54396(13)	0.53734(17)	0.1866(11)
H31_14	-0.196653	0.538550	0.524794	0.224
C32_14	-0.2347(6)	0.53104(9)	0.5517(2)	0.158(2)
H32_14	-0.240416	0.517009	0.548528	0.189
C33_14	-0.2510(7)	0.53879(12)	0.5707(2)	0.1866(11)
H33_14	-0.266027	0.529918	0.580759	0.224

C34_14	-0.2449(7)	0.55971(12)	0.57481(15)	0.1866(11)
H34_14	-0.256088	0.564969	0.587703	0.224
C35_14	-0.2775(3)	0.64486(10)	0.54538(10)	0.096(3)
H35_14	-0.277805	0.658967	0.548928	0.116
C36_14	-0.3033(4)	0.63904(11)	0.52831(12)	0.117(3)
H36_14	-0.318022	0.649088	0.519377	0.141
C37_14	-0.3085(4)	0.61855(11)	0.52360(12)	0.123(3)
C38_14	-0.2831(4)	0.60418(10)	0.53574(12)	0.110(4)
H38_14	-0.287511	0.590101	0.532465	0.132
C39_14	-0.3399(3)	0.61146(12)	0.50551(9)	0.151(3)
C40_14	-0.3466(3)	0.62431(13)	0.48848(11)	0.1463(11)
H40_14	-0.331865	0.637756	0.488646	0.176
C41_14	-0.3756(3)	0.61724(17)	0.47092(9)	0.1463(11)
H41_14	-0.380468	0.625752	0.459276	0.176
C42_14	-0.3963(3)	0.59753(18)	0.47145(12)	0.146(3)
H42_14	-0.415614	0.592764	0.459783	0.175
C43_14	-0.3908(3)	0.58453(14)	0.48760(14)	0.1463(11)
H43_14	-0.405765	0.571118	0.487188	0.176
C44_14	-0.3624(3)	0.59146(12)	0.50478(12)	0.1463(11)
H44_14	-0.358152	0.582614	0.516211	0.176
P1_4	0.03364(9)	0.65859(4)	0.57712(3)	0.0559(7)
O1_4	0.00520(18)	0.66461(7)	0.59659(5)	0.0525(15)
O2_4	0.04056(19)	0.63627(5)	0.57277(8)	0.0597(16)
O3_4	0.09287(13)	0.66829(6)	0.57550(7)	0.0637(16)
O4_4	0.00118(15)	0.66991(7)	0.55939(5)	0.0639(16)
C1_4	0.0674(2)	0.68761(8)	0.53941(7)	0.073(3)
C2_4	0.0810(3)	0.70005(7)	0.55807(8)	0.075(2)
C3_4	0.0949(2)	0.68980(7)	0.57519(7)	0.069(2)
C4_4	0.1085(3)	0.69922(8)	0.59396(8)	0.083(3)
H4_4	0.118027	0.691288	0.605617	0.099
C5_4	0.1075(5)	0.72054(8)	0.59480(10)	0.125(2)
C6_4	0.0939(4)	0.73227(8)	0.57741(9)	0.124(2)
C7_4	0.0791(3)	0.72181(7)	0.55869(8)	0.086(3)
C8_4	0.1154(4)	0.73042(11)	0.61561(9)	0.128(2)
C9_4	0.0687(4)	0.7360(2)	0.62730(15)	0.192(4)
H9_4	0.028352	0.734887	0.620863	0.230
C10_4	0.0765(5)	0.7432(2)	0.64772(15)	0.192(4)
H10_4	0.042755	0.747808	0.657258	0.230
C11_4	0.1296(6)	0.7440(2)	0.65455(14)	0.191(4)
H11_4	0.135154	0.749385	0.670019	0.230
C12_4	0.1745(5)	0.7391(3)	0.64458(16)	0.192(4)
H12_4	0.214283	0.740059	0.651515	0.230
C13_4	0.1666(4)	0.7323(2)	0.62373(15)	0.192(4)
H13_4	0.201608	0.728722	0.614425	0.230
C14_4	0.0913(4)	0.75376(8)	0.57785(11)	0.124(3)
H14_4	0.100744	0.760786	0.590075	0.149
C15_4	0.0753(4)	0.76487(8)	0.56094(11)	0.105(3)
C16_4	0.0612(4)	0.75478(9)	0.54320(11)	0.106(3)
H16_4	0.049903	0.762291	0.531489	0.127
C17_4	0.0634(3)	0.73417(9)	0.54260(10)	0.105(3)
H17_4	0.053424	0.727645	0.530156	0.126
C18_4	0.0731(3)	0.78795(7)	0.56174(12)	0.101(3)
C19_4	0.0499(4)	0.79852(11)	0.57841(12)	0.128(3)
H19_4	0.037617	0.791044	0.589999	0.154

C20_4	0.0447(5)	0.81995(11)	0.57814(16)	0.129(3)
H20_4	0.028749	0.827005	0.589358	0.154
C21_4	0.0631(5)	0.83052(9)	0.56121(18)	0.129(3)
H21_4	0.060362	0.845029	0.561151	0.155
C22_4	0.0855(5)	0.82088(10)	0.54430(16)	0.129(3)
H22_4	0.096841	0.828557	0.532703	0.154
C23_4	0.0910(5)	0.79951(11)	0.54471(13)	0.128(3)
H23_4	0.107068	0.792689	0.533368	0.153
C24_4	0.0273(2)	0.67385(8)	0.54062(6)	0.061(2)
C25_4	0.0066(3)	0.66291(12)	0.52366(7)	0.076(3)
H25_4	-0.021447	0.653055	0.525462	0.091
C26_4	0.0274(3)	0.66665(13)	0.50469(8)	0.096(3)
C27_4	0.0717(3)	0.68014(15)	0.50203(8)	0.130(5)
C28_4	0.0912(3)	0.69145(11)	0.51961(7)	0.093(3)
C29_4	-0.0021(4)	0.65798(13)	0.48688(9)	0.154(4)
C30_4	-0.0152(5)	0.63716(12)	0.48557(14)	0.194(5)
H30_4	-0.002470	0.628008	0.495815	0.233
C31_4	-0.0467(6)	0.62961(16)	0.46937(19)	0.195(5)
H31_4	-0.055310	0.615446	0.468650	0.234
C32_4	-0.0655(5)	0.6431(2)	0.45426(16)	0.187(7)
H32_4	-0.086788	0.638012	0.443215	0.225
C33_4	-0.0530(6)	0.6640(2)	0.45538(16)	0.203(6)
H33_4	-0.065923	0.673124	0.445195	0.244
C34_4	-0.0212(6)	0.67125(15)	0.47162(15)	0.204(6)
H34_4	-0.012383	0.685403	0.472292	0.244
C35_4	0.1404(3)	0.70303(15)	0.51686(10)	0.110(4)
H35_4	0.155327	0.709966	0.528358	0.132
C36_4	0.1664(4)	0.70455(16)	0.49893(11)	0.114(4)
H36_4	0.195840	0.713886	0.497469	0.137
C37_4	0.1506(4)	0.69264(17)	0.48253(11)	0.128(4)
C38_4	0.1028(3)	0.68160(15)	0.48381(9)	0.113(4)
H38_4	0.090220	0.674654	0.471952	0.136
C39_4	0.1798(3)	0.69341(15)	0.46264(10)	0.143(5)
C40_4	0.2352(3)	0.69866(16)	0.46192(14)	0.229(7)
H40_4	0.254050	0.701986	0.474187	0.275
C41_4	0.2634(4)	0.69900(18)	0.44283(17)	0.229(7)
H41_4	0.300950	0.702528	0.442092	0.274
C42_4	0.2342(5)	0.6940(2)	0.42544(15)	0.190(7)
H42_4	0.252791	0.694232	0.412695	0.229
C43_4	0.1804(5)	0.6889(2)	0.42538(10)	0.249(8)
H43_4	0.162164	0.685563	0.412980	0.299
C44_4	0.1528(4)	0.6885(2)	0.44407(12)	0.249(8)
H44_4	0.115295	0.684947	0.444358	0.299

1. U_{eq} is defined as 1/3 of the trace of the orthogonalized U_{ij} tensor.

Table 10.1.10. Anisotropic displacement parameters [Å^2] for S-6a (ZC-01-51). The anisotropic displacement factor exponent takes the form: $-2\pi^2[\, h^2(a^*)^2U_{11} + k^2(b^*)^2U_{22} + \ldots + 2hka^*b^*U_{12}\,]$

Atom	U_{11}	U_{22}	U_{33}	U_{23}	U_{13}	U_{12}
Rh1	0.0401(4)	0.0525(5)	0.0419(4)	−0.0029(4)	−0.0057(3)	0.0048(3)
Rh2	0.0431(4)	0.0536(5)	0.0482(4)	−0.0049(4)	−0.0027(3)	0.0066(4)
Rh3	0.0418(4)	0.0660(6)	0.0527(4)	0.0176(4)	0.0068(3)	0.0094(4)
Rh4	0.0418(4)	0.0614(6)	0.0472(4)	0.0117(4)	0.0065(3)	0.0117(4)

Rh5	0.0581(4)	0.0656(6)	0.0477(4)	0.0002(5)	−0.0047(4)	0.0013(4)
O	0.095(6)	0.219(11)	0.141(9)	−0.148(10)	−0.026(6)	0.035(6)
P1_1	0.0440(12)	0.0515(17)	0.0608(15)	0.0086(12)	0.0077(10)	0.0004(10)
O1_1	0.058(3)	0.058(2)	0.058(4)	0.016(2)	0.011(3)	0.011(2)
O2_1	0.049(3)	0.050(3)	0.051(3)	0.004(3)	0.001(2)	−0.009(2)
O3_1	0.042(2)	0.074(3)	0.067(2)	−0.005(2)	0.007(2)	0.000(3)
O4_1	0.0477(17)	0.041(3)	0.076(4)	0.006(3)	0.0015(18)	0.0000(17)
C1_1	0.074(5)	0.052(4)	0.076(3)	−0.005(3)	0.006(2)	0.007(4)
C2_1	0.044(2)	0.057(6)	0.072(3)	−0.006(3)	0.007(2)	−0.002(3)
C3_1	0.044(2)	0.048(6)	0.061(3)	−0.017(3)	0.006(2)	−0.004(2)
C4_1	0.059(3)	0.080(7)	0.066(4)	−0.004(4)	0.008(3)	0.007(4)
C5_1	0.079(3)	0.116(4)	0.074(3)	−0.003(3)	0.023(2)	0.000(3)
C6_1	0.078(3)	0.112(5)	0.077(3)	−0.005(3)	0.024(2)	0.002(3)
C7_1	0.054(2)	0.065(6)	0.077(3)	−0.020(4)	0.020(2)	−0.007(3)
C8_1	0.081(4)	0.120(5)	0.073(3)	−0.002(3)	0.022(3)	0.001(3)
C9_1	0.238(18)	0.123(5)	0.089(6)	−0.001(4)	−0.019(8)	−0.014(4)
C10_1	0.181(9)	0.162(7)	0.097(5)	0.020(5)	−0.005(6)	−0.012(8)
C11_1	0.180(9)	0.164(7)	0.097(5)	0.020(5)	−0.005(6)	−0.012(8)
C12_1	0.179(9)	0.165(7)	0.097(5)	0.018(5)	−0.004(6)	−0.013(8)
C13_1	0.110(9)	0.132(6)	0.079(4)	−0.006(4)	0.011(4)	−0.005(6)
C14_1	0.078(3)	0.111(5)	0.078(4)	−0.007(3)	0.025(2)	0.001(3)
C15_1	0.079(3)	0.090(7)	0.110(5)	−0.034(5)	0.017(3)	0.008(4)
C16_1	0.055(3)	0.074(6)	0.113(5)	−0.037(4)	0.016(3)	0.003(3)
C17_1	0.055(2)	0.074(6)	0.111(5)	−0.037(4)	0.015(2)	0.003(3)
C18_1	0.110(4)	0.148(8)	0.105(7)	−0.073(5)	0.036(5)	−0.038(5)
C19_1	0.118(5)	0.206(8)	0.182(7)	−0.006(5)	0.071(5)	−0.014(5)
C20_1	0.120(5)	0.207(8)	0.183(7)	−0.006(5)	0.072(5)	−0.014(5)
C21_1	0.120(5)	0.207(8)	0.184(7)	−0.006(5)	0.073(5)	−0.013(5)
C22_1	0.122(4)	0.208(8)	0.187(8)	−0.004(5)	0.074(5)	−0.013(5)
C23_1	0.122(4)	0.208(8)	0.188(8)	−0.003(6)	0.074(5)	−0.012(5)
C24_1	0.067(5)	0.048(4)	0.069(3)	−0.003(3)	−0.005(3)	0.012(3)
C25_1	0.093(6)	0.020(5)	0.067(3)	−0.013(3)	−0.017(3)	−0.004(4)
C26_1	0.109(7)	0.033(5)	0.098(4)	0.002(3)	−0.008(4)	0.011(4)
C27_1	0.087(6)	0.036(5)	0.107(4)	−0.012(3)	−0.012(4)	−0.003(4)
C28_1	0.046(5)	0.044(4)	0.109(4)	−0.006(3)	−0.005(4)	−0.009(3)
C29_1	0.190(11)	0.160(9)	0.107(4)	0.031(5)	−0.013(5)	0.063(8)
C30_1	0.284(13)	0.220(9)	0.181(10)	0.093(7)	−0.025(9)	0.019(9)
C31_1	0.284(13)	0.219(9)	0.181(10)	0.093(7)	−0.026(9)	0.019(9)
C32_1	0.228(14)	0.229(11)	0.196(13)	0.028(9)	0.005(10)	0.065(10)
C33_1	0.291(13)	0.255(10)	0.160(9)	0.026(8)	−0.064(9)	−0.003(9)
C34_1	0.291(13)	0.254(10)	0.160(9)	0.025(8)	−0.065(8)	−0.003(9)
C35_1	0.045(5)	0.046(5)	0.125(5)	−0.010(4)	0.022(4)	−0.027(3)
C36_1	0.076(6)	0.053(6)	0.143(6)	−0.012(4)	0.018(5)	−0.012(4)
C37_1	0.074(6)	0.040(5)	0.146(6)	−0.013(4)	0.012(5)	−0.022(4)
C38_1	0.080(6)	0.043(5)	0.128(6)	−0.002(4)	−0.005(5)	0.000(4)
C39_1	0.065(6)	0.051(5)	0.244(9)	0.030(4)	0.057(5)	−0.016(4)
C40_1	0.057(6)	0.068(6)	0.284(9)	0.002(6)	0.060(5)	−0.009(4)
C41_1	0.058(6)	0.069(6)	0.285(9)	0.004(6)	0.060(5)	−0.009(4)
C42_1	0.133(11)	0.095(9)	0.315(9)	0.006(7)	0.013(7)	0.002(8)
C43_1	0.132(8)	0.077(8)	0.285(9)	0.029(6)	0.009(6)	0.004(6)
C44_1	0.132(8)	0.077(8)	0.283(9)	0.030(6)	0.008(6)	0.004(6)
P1_2	0.0607(18)	0.179(3)	0.197(4)	−0.131(3)	−0.018(2)	0.012(2)
O1_2	0.081(4)	0.283(11)	0.203(11)	−0.199(10)	−0.054(4)	0.060(5)
O2_2	0.068(4)	0.118(7)	0.204(8)	−0.092(7)	−0.032(4)	0.012(4)

O3_2	0.065(5)	0.145(6)	0.199(4)	−0.120(4)	−0.015(3)	0.013(4)
O4_2	0.056(4)	0.186(4)	0.159(6)	−0.116(4)	−0.011(3)	0.006(3)
C1_2	0.055(5)	0.123(7)	0.118(5)	−0.076(5)	0.001(3)	−0.004(4)
C2_2	0.065(5)	0.121(5)	0.120(6)	−0.081(5)	−0.001(4)	0.005(3)
C3_2	0.062(6)	0.122(5)	0.170(7)	−0.089(5)	−0.010(5)	0.005(3)
C4_2	0.061(6)	0.123(6)	0.224(9)	−0.063(6)	−0.025(6)	−0.010(4)
C5_2	0.069(4)	0.108(5)	0.271(7)	−0.063(5)	−0.061(4)	0.009(3)
C6_2	0.067(4)	0.108(5)	0.268(7)	−0.065(5)	−0.061(4)	0.008(3)
C7_2	0.074(6)	0.114(5)	0.180(8)	−0.106(5)	−0.036(5)	0.037(4)
C8_2	0.071(4)	0.109(5)	0.274(7)	−0.063(5)	−0.062(4)	0.008(3)
C9_2	0.106(7)	0.157(12)	0.284(8)	−0.043(7)	−0.045(6)	0.050(8)
C10_2	0.139(7)	0.108(7)	0.327(10)	−0.069(9)	−0.090(8)	0.044(5)
C11_2	0.139(7)	0.108(7)	0.327(10)	−0.068(9)	−0.091(8)	0.044(5)
C12_2	0.139(7)	0.109(7)	0.326(10)	−0.068(8)	−0.092(8)	0.044(5)
C13_2	0.115(8)	0.103(10)	0.274(7)	−0.062(6)	−0.066(5)	0.030(7)
C14_2	0.067(4)	0.108(5)	0.268(7)	−0.065(5)	−0.062(4)	0.009(3)
C15_2	0.127(8)	0.109(5)	0.39(2)	−0.055(6)	−0.145(11)	0.004(4)
C16_2	0.117(7)	0.114(5)	0.299(14)	−0.091(6)	−0.106(8)	0.032(4)
C17_2	0.117(7)	0.114(5)	0.299(14)	−0.092(6)	−0.106(8)	0.033(4)
C18_2	0.136(6)	0.145(11)	0.61(2)	−0.106(14)	−0.181(8)	0.001(5)
C19_2	0.142(7)	0.158(9)	0.657(18)	−0.133(10)	−0.163(9)	−0.013(6)
C20_2	0.143(6)	0.158(9)	0.658(18)	−0.133(10)	−0.163(9)	−0.013(6)
C21_2	0.143(6)	0.157(9)	0.659(18)	−0.132(10)	−0.163(9)	−0.012(6)
C22_2	0.143(6)	0.158(9)	0.660(18)	−0.132(10)	−0.163(9)	−0.009(6)
C23_2	0.141(6)	0.157(9)	0.661(18)	−0.130(10)	−0.163(9)	−0.006(6)
C24_2	0.062(6)	0.153(6)	0.118(5)	−0.085(5)	−0.001(3)	−0.002(5)
C25_2	0.059(6)	0.172(8)	0.126(6)	−0.078(5)	−0.010(4)	−0.008(5)
C26_2	0.068(6)	0.172(9)	0.113(5)	−0.071(5)	−0.012(4)	−0.001(5)
C27_2	0.052(6)	0.190(10)	0.114(5)	−0.072(5)	−0.017(3)	0.020(6)
C28_2	0.089(6)	0.183(9)	0.103(5)	−0.104(5)	−0.033(4)	0.044(6)
C29_2	0.096(7)	0.253(9)	0.131(8)	−0.041(8)	−0.013(6)	0.029(6)
C30_2	0.166(9)	0.339(12)	0.326(18)	0.045(12)	−0.126(9)	−0.036(9)
C31_2	0.167(9)	0.338(12)	0.326(18)	0.045(12)	−0.124(9)	−0.034(9)
C32_2	0.254(15)	0.345(13)	0.28(2)	0.039(13)	−0.035(14)	−0.027(10)
C33_2	0.293(14)	0.325(10)	0.359(17)	0.085(10)	−0.092(11)	−0.047(8)
C34_2	0.293(14)	0.325(10)	0.359(17)	0.085(9)	−0.092(11)	−0.047(8)
C35_2	0.091(7)	0.196(13)	0.102(5)	−0.105(6)	−0.038(4)	0.071(7)
C36_2	0.102(8)	0.230(14)	0.103(6)	−0.076(7)	−0.032(5)	0.102(8)
C37_2	0.103(8)	0.257(14)	0.103(5)	−0.062(7)	−0.026(4)	0.106(9)
C38_2	0.068(6)	0.220(12)	0.104(5)	−0.069(7)	−0.026(4)	0.047(7)
C39_2	0.135(8)	0.335(12)	0.100(9)	−0.037(9)	−0.027(6)	0.169(8)
C40_2	0.291(13)	0.353(13)	0.38(2)	0.034(13)	0.191(14)	0.237(10)
C41_2	0.291(13)	0.354(13)	0.38(2)	0.034(13)	0.190(14)	0.237(10)
C42_2	0.301(18)	0.317(12)	0.130(14)	−0.066(11)	0.052(13)	0.195(11)
C43_2	0.356(17)	0.375(12)	0.38(3)	0.068(13)	0.170(15)	0.259(9)
C44_2	0.356(17)	0.376(12)	0.38(3)	0.067(13)	0.170(15)	0.259(9)
P1_3	0.0454(12)	0.084(2)	0.0442(13)	0.0149(12)	0.0045(9)	0.0140(12)
O1_3	0.045(3)	0.061(5)	0.0429(16)	0.013(2)	0.0035(16)	0.015(3)
O2_3	0.035(2)	0.095(5)	0.048(3)	0.027(3)	0.009(2)	0.008(3)
O3_3	0.042(2)	0.085(2)	0.050(3)	0.0127(18)	0.015(2)	0.010(2)
O4_3	0.0460(15)	0.069(4)	0.038(3)	0.013(2)	0.0041(16)	0.0160(18)
C1_3	0.054(5)	0.092(4)	0.038(4)	0.000(2)	0.006(3)	0.006(3)
C2_3	0.050(2)	0.081(4)	0.031(4)	−0.010(3)	0.009(2)	0.013(2)
C3_3	0.049(2)	0.068(4)	0.041(4)	−0.009(3)	0.007(2)	0.013(2)

C4_3	0.068(3)	0.083(5)	0.065(5)	0.009(4)	0.008(3)	0.007(3)
C5_3	0.082(3)	0.093(4)	0.053(3)	−0.005(3)	0.008(2)	0.026(2)
C6_3	0.081(3)	0.096(4)	0.053(3)	−0.006(3)	0.007(2)	0.028(2)
C7_3	0.061(2)	0.101(5)	0.056(5)	−0.003(4)	0.005(2)	0.027(3)
C8_3	0.084(3)	0.092(4)	0.054(3)	−0.005(3)	0.009(3)	0.026(3)
C9_3	0.158(9)	0.086(7)	0.062(3)	0.000(3)	−0.011(3)	−0.001(6)
C10_3	0.170(8)	0.134(7)	0.078(5)	0.029(5)	−0.026(5)	−0.026(5)
C11_3	0.170(8)	0.134(7)	0.081(5)	0.028(5)	−0.026(5)	−0.027(5)
C12_3	0.170(8)	0.135(7)	0.083(5)	0.026(5)	−0.028(5)	−0.027(5)
C13_3	0.173(10)	0.135(6)	0.080(5)	0.022(5)	−0.032(5)	−0.035(7)
C14_3	0.081(3)	0.098(4)	0.053(4)	−0.006(3)	0.006(2)	0.028(3)
C15_3	0.080(3)	0.122(6)	0.037(5)	−0.025(4)	0.004(3)	0.022(3)
C16_3	0.061(3)	0.142(6)	0.056(4)	−0.004(4)	0.008(3)	0.029(3)
C17_3	0.061(2)	0.142(6)	0.055(5)	−0.003(4)	0.007(3)	0.027(3)
C18_3	0.101(5)	0.191(10)	0.104(5)	0.014(5)	0.003(4)	0.054(7)
C19_3	0.084(5)	0.169(7)	0.099(5)	0.003(4)	−0.008(3)	0.031(4)
C20_3	0.086(4)	0.169(6)	0.101(5)	0.002(4)	−0.008(3)	0.031(4)
C21_3	0.089(4)	0.171(6)	0.102(5)	0.000(4)	−0.007(3)	0.032(4)
C22_3	0.090(4)	0.171(6)	0.102(5)	0.000(4)	−0.005(4)	0.034(4)
C23_3	0.091(4)	0.170(6)	0.101(5)	−0.001(5)	−0.003(4)	0.036(4)
C24_3	0.038(4)	0.089(4)	0.037(3)	−0.001(3)	0.001(2)	0.000(3)
C25_3	0.118(8)	0.088(4)	0.036(5)	−0.005(3)	0.013(4)	−0.010(4)
C26_3	0.168(9)	0.140(5)	0.060(6)	0.033(3)	0.050(6)	0.040(4)
C27_3	0.073(6)	0.133(5)	0.060(5)	0.013(3)	0.018(4)	0.005(4)
C28_3	0.061(5)	0.124(5)	0.041(4)	0.001(3)	0.012(3)	0.008(4)
C29_3	0.217(6)	0.166(6)	0.122(12)	0.068(7)	0.013(5)	0.007(4)
C30_3	0.222(7)	0.191(10)	0.250(15)	0.132(9)	−0.020(8)	−0.016(7)
C31_3	0.222(7)	0.190(10)	0.250(15)	0.130(9)	−0.019(8)	−0.017(7)
C32_3	0.220(7)	0.222(11)	0.266(18)	0.041(11)	0.005(9)	−0.020(6)
C33_3	0.223(6)	0.219(6)	0.224(6)	0.005(3)	0.002(3)	−0.008(3)
C34_3	0.224(6)	0.219(6)	0.225(7)	0.007(4)	0.004(4)	−0.010(3)
C35_3	0.070(6)	0.125(5)	0.035(4)	0.006(3)	0.014(4)	0.016(4)
C36_3	0.075(6)	0.120(6)	0.039(4)	−0.015(4)	0.018(4)	−0.016(4)
C37_3	0.051(5)	0.125(6)	0.039(4)	−0.010(3)	−0.005(3)	−0.024(4)
C38_3	0.071(6)	0.122(6)	0.064(5)	−0.004(4)	0.020(4)	−0.024(5)
C39_3	0.058(5)	0.177(7)	0.045(4)	−0.007(4)	0.002(3)	−0.020(4)
C40_3	0.056(5)	0.187(7)	0.053(5)	−0.017(4)	0.006(3)	−0.011(4)
C41_3	0.055(5)	0.187(7)	0.053(5)	−0.017(4)	0.005(3)	−0.011(4)
C42_3	0.078(7)	0.211(8)	0.099(8)	−0.036(6)	0.025(6)	−0.035(6)
C43_3	0.088(6)	0.203(8)	0.099(7)	−0.033(5)	0.040(5)	−0.045(5)
C44_3	0.087(5)	0.203(8)	0.099(7)	−0.033(5)	0.040(5)	−0.045(5)
P1_5	0.0561(13)	0.0719(19)	0.0413(13)	0.0028(13)	−0.0058(10)	−0.0036(11)
O1_5	0.054(3)	0.070(2)	0.040(3)	0.002(2)	−0.016(3)	−0.0004(19)
O2_5	0.084(4)	0.058(4)	0.0370(16)	0.009(2)	−0.0077(17)	−0.012(3)
O3_5	0.0516(17)	0.088(5)	0.038(2)	0.006(2)	−0.0098(16)	−0.003(2)
O4_5	0.059(3)	0.083(3)	0.045(3)	0.012(2)	−0.003(2)	0.001(2)
C1_5	0.080(5)	0.085(4)	0.041(4)	0.012(3)	−0.002(3)	−0.005(3)
C2_5	0.103(7)	0.090(4)	0.041(3)	0.007(2)	−0.014(3)	0.006(4)
C3_5	0.059(5)	0.085(4)	0.040(2)	0.006(2)	−0.007(2)	−0.002(4)
C4_5	0.068(6)	0.085(4)	0.067(3)	0.003(3)	0.014(4)	−0.004(4)
C5_5	0.146(5)	0.123(5)	0.067(3)	−0.005(3)	0.012(3)	0.016(4)
C6_5	0.146(5)	0.126(5)	0.066(3)	−0.004(3)	0.010(4)	0.015(4)
C7_5	0.084(7)	0.119(5)	0.040(3)	0.006(2)	−0.012(3)	0.007(4)
C8_5	0.149(5)	0.123(5)	0.068(4)	−0.006(3)	0.014(4)	0.016(4)

C9_5	0.144(7)	0.184(8)	0.33(2)	−0.155(12)	0.006(8)	0.036(6)
C10_5	0.225(11)	0.159(7)	0.304(15)	−0.110(8)	0.091(12)	0.029(7)
C11_5	0.225(11)	0.157(7)	0.304(15)	−0.111(8)	0.092(12)	0.030(7)
C12_5	0.225(11)	0.158(7)	0.305(15)	−0.110(8)	0.091(12)	0.030(7)
C13_5	0.170(6)	0.204(9)	0.42(4)	−0.202(16)	−0.059(9)	0.063(5)
C14_5	0.146(6)	0.130(5)	0.067(3)	−0.005(3)	0.010(4)	0.015(4)
C15_5	0.53(4)	0.237(7)	0.092(4)	0.025(4)	−0.015(6)	0.167(14)
C16_5	0.109(7)	0.178(6)	0.067(4)	0.049(4)	−0.005(5)	0.028(5)
C17_5	0.109(7)	0.177(6)	0.068(4)	0.047(4)	−0.004(5)	0.027(5)
C18_5	0.162(8)	0.512(3)	0.095(4)	−0.019(2)	−0.006(3)	0.206(4)
C19_5	0.228(2)	0.525(2)	0.0853(19)	−0.0208(19)	−0.0082(19)	0.183(2)
C20_5	0.228(2)	0.525(2)	0.0853(19)	−0.0208(19)	−0.0082(19)	0.183(2)
C21_5	0.228(3)	0.525(2)	0.085(3)	−0.021(2)	−0.009(3)	0.183(2)
C22_5	0.228(2)	0.525(2)	0.0853(19)	−0.0208(19)	−0.0082(19)	0.183(2)
C23_5	0.228(2)	0.525(2)	0.0853(19)	−0.0208(19)	−0.0082(19)	0.183(2)
C24_5	0.081(5)	0.086(3)	0.045(5)	0.010(3)	0.000(3)	−0.009(3)
C25_5	0.076(6)	0.088(4)	0.080(7)	0.004(4)	0.011(5)	−0.010(3)
C26_5	0.068(5)	0.091(4)	0.116(7)	0.009(4)	0.007(4)	−0.013(3)
C27_5	0.083(6)	0.086(4)	0.129(8)	0.015(5)	0.021(5)	−0.007(4)
C28_5	0.070(5)	0.086(4)	0.101(7)	0.024(4)	0.006(4)	0.000(3)
C29_5	0.080(7)	0.090(5)	0.195(7)	0.002(5)	0.039(5)	−0.016(5)
C30_5	0.089(7)	0.092(8)	0.197(6)	−0.005(5)	0.032(5)	−0.004(5)
C31_5	0.090(7)	0.093(8)	0.197(7)	−0.005(5)	0.032(5)	−0.003(5)
C32_5	0.080(8)	0.100(9)	0.209(7)	0.007(6)	0.028(6)	−0.013(6)
C33_5	0.134(8)	0.125(8)	0.209(7)	0.003(6)	0.019(6)	0.015(6)
C34_5	0.135(8)	0.125(8)	0.209(7)	0.004(6)	0.019(6)	0.016(6)
C35_5	0.070(5)	0.076(4)	0.119(8)	0.040(5)	0.010(5)	0.009(4)
C36_5	0.066(6)	0.080(3)	0.087(7)	0.046(5)	−0.007(4)	0.007(3)
C37_5	0.054(5)	0.080(3)	0.138(8)	0.034(4)	−0.002(5)	0.000(3)
C38_5	0.066(6)	0.090(4)	0.171(9)	0.041(5)	0.019(6)	0.004(4)
C39_5	0.0885(19)	0.087(3)	0.171(13)	0.038(4)	−0.009(3)	−0.019(2)
C40_5	0.0881(17)	0.0877(18)	0.147(2)	0.0313(18)	−0.0125(18)	−0.0187(16)
C41_5	0.0881(17)	0.0877(18)	0.147(2)	0.0313(18)	−0.0125(18)	−0.0187(16)
C42_5	0.0878(18)	0.091(3)	0.095(8)	0.022(4)	−0.014(2)	−0.017(2)
C43_5	0.0881(17)	0.0877(18)	0.147(2)	0.0313(18)	−0.0125(18)	−0.0187(16)
C44_5	0.0881(17)	0.0877(18)	0.147(2)	0.0313(18)	−0.0125(18)	−0.0187(16)
P1_6	0.0619(17)	0.181(3)	0.112(2)	0.099(3)	0.0285(17)	0.042(2)
O1_6	0.074(3)	0.187(9)	0.139(7)	0.118(7)	0.044(4)	0.049(4)
O2_6	0.072(3)	0.229(9)	0.092(6)	0.091(6)	0.039(4)	0.069(5)
O3_6	0.048(3)	0.186(3)	0.102(4)	0.087(3)	0.018(3)	0.039(2)
O4_6	0.063(4)	0.141(5)	0.118(3)	0.093(3)	0.021(2)	0.033(3)
C1_6	0.052(4)	0.099(4)	0.047(4)	0.035(3)	−0.002(3)	0.002(3)
C2_6	0.039(4)	0.097(5)	0.063(3)	0.032(3)	0.001(2)	−0.004(3)
C3_6	0.053(5)	0.128(6)	0.064(3)	0.039(3)	0.006(2)	0.029(4)
C4_6	0.053(5)	0.133(6)	0.080(4)	0.037(4)	0.018(3)	0.023(5)
C5_6	0.040(3)	0.104(4)	0.070(3)	0.021(3)	0.006(2)	0.002(2)
C6_6	0.041(3)	0.105(4)	0.069(3)	0.020(3)	0.005(2)	0.000(2)
C7_6	0.039(4)	0.098(5)	0.059(3)	0.031(3)	0.006(2)	−0.001(3)
C8_6	0.039(3)	0.104(4)	0.071(3)	0.020(3)	0.006(2)	0.001(3)
C9_6	0.095(6)	0.125(5)	0.177(11)	−0.037(6)	0.079(7)	−0.041(4)
C10_6	0.095(5)	0.116(6)	0.107(6)	0.007(5)	0.048(4)	−0.006(4)
C11_6	0.095(5)	0.115(5)	0.109(6)	0.008(5)	0.047(4)	−0.005(4)
C12_6	0.094(5)	0.114(5)	0.113(6)	0.008(5)	0.045(4)	−0.005(4)
C13_6	0.072(6)	0.108(4)	0.125(9)	0.004(4)	0.041(6)	−0.014(3)

C14_6	0.040(3)	0.104(4)	0.067(3)	0.020(3)	0.006(2)	−0.001(3)
C15_6	0.058(4)	0.081(5)	0.067(3)	0.021(3)	0.005(2)	−0.005(4)
C16_6	0.060(4)	0.084(5)	0.059(3)	0.026(3)	0.001(2)	0.003(3)
C17_6	0.059(4)	0.085(6)	0.059(3)	0.027(3)	0.002(2)	0.003(3)
C18_6	0.060(4)	0.101(5)	0.067(4)	0.034(3)	0.020(3)	0.008(3)
C19_6	0.082(4)	0.106(5)	0.094(5)	0.024(4)	−0.003(3)	0.019(3)
C20_6	0.083(4)	0.106(5)	0.094(5)	0.023(4)	−0.003(3)	0.020(3)
C21_6	0.083(4)	0.107(5)	0.093(5)	0.023(4)	−0.002(3)	0.020(3)
C22_6	0.082(4)	0.107(5)	0.093(4)	0.022(4)	−0.003(3)	0.020(3)
C23_6	0.081(4)	0.107(5)	0.092(4)	0.021(4)	−0.003(3)	0.021(4)
C24_6	0.060(5)	0.100(4)	0.066(5)	0.038(4)	−0.003(3)	−0.001(3)
C25_6	0.057(5)	0.101(4)	0.108(6)	0.019(4)	0.003(4)	−0.010(4)
C26_6	0.043(5)	0.083(4)	0.110(7)	0.020(4)	−0.001(4)	−0.002(3)
C27_6	0.053(5)	0.082(4)	0.081(6)	0.010(4)	0.009(4)	−0.001(3)
C28_6	0.053(4)	0.077(4)	0.053(5)	0.027(4)	0.004(3)	0.007(3)
C29_6	0.083(7)	0.100(6)	0.231(8)	−0.034(7)	0.051(6)	−0.020(5)
C30_6	0.336(15)	0.288(15)	0.325(11)	−0.046(10)	−0.011(11)	0.192(12)
C31_6	0.337(15)	0.288(15)	0.324(11)	−0.046(10)	−0.010(11)	0.191(12)
C32_6	0.31(2)	0.294(19)	0.318(11)	−0.029(11)	0.011(11)	0.070(16)
C33_6	0.245(14)	0.255(14)	0.233(8)	−0.054(7)	0.046(7)	0.036(10)
C34_6	0.245(14)	0.255(14)	0.232(8)	−0.054(7)	0.046(7)	0.036(10)
C35_6	0.052(5)	0.075(4)	0.096(7)	0.025(4)	0.029(4)	0.012(3)
C36_6	0.051(4)	0.077(5)	0.059(5)	0.000(4)	0.014(3)	−0.007(3)
C37_6	0.053(4)	0.077(4)	0.041(5)	0.006(4)	0.007(3)	0.000(3)
C38_6	0.054(5)	0.078(4)	0.080(6)	0.005(4)	0.010(4)	−0.003(3)
C39_6	0.071(4)	0.065(6)	0.092(5)	0.003(4)	0.038(3)	0.004(4)
C40_6	0.068(4)	0.084(6)	0.136(6)	−0.027(5)	0.052(4)	−0.002(4)
C41_6	0.069(4)	0.085(6)	0.137(6)	−0.026(5)	0.053(4)	−0.002(4)
C42_6	0.108(7)	0.131(9)	0.153(7)	−0.051(6)	0.077(5)	−0.032(7)
C43_6	0.089(5)	0.092(7)	0.103(5)	−0.012(4)	0.052(4)	−0.004(4)
C44_6	0.088(6)	0.090(7)	0.101(5)	−0.011(4)	0.051(4)	−0.004(4)
O1S_7	0.085(4)	0.121(6)	0.068(5)	−0.020(4)	−0.032(4)	0.002(4)
C2S_7	0.194(8)	0.247(10)	0.262(19)	−0.027(10)	−0.025(10)	−0.122(8)
C3S_7	0.31(3)	0.246(18)	0.35(2)	0.007(17)	0.018(18)	−0.16(2)
C4S_7	0.122(5)	0.161(12)	0.173(12)	−0.018(8)	0.030(6)	0.021(6)
C5S_7	0.042(7)	0.30(2)	0.212(13)	0.058(12)	0.001(7)	−0.007(8)
O1S_8	0.077(4)	0.125(6)	0.073(5)	0.025(4)	0.005(3)	0.013(4)
C2S_8	0.098(5)	0.173(12)	0.132(10)	0.068(7)	−0.029(6)	−0.007(5)
C3S_8	0.29(2)	0.250(17)	0.36(2)	−0.037(14)	−0.26(2)	0.074(13)
C4S_8	0.110(5)	0.216(8)	0.098(11)	0.060(9)	−0.023(5)	−0.048(6)
C5S_8	0.231(16)	0.229(8)	0.27(3)	−0.018(12)	0.06(2)	−0.117(8)
P1_9	0.0562(13)	0.0599(17)	0.0505(14)	0.0075(13)	0.0028(10)	0.0163(12)
O1_9	0.072(3)	0.041(4)	0.053(2)	0.011(2)	−0.006(2)	0.001(3)
O2_9	0.055(4)	0.060(2)	0.073(5)	0.012(2)	−0.007(3)	0.0113(18)
O3_9	0.0587(17)	0.064(3)	0.061(4)	0.006(3)	0.010(2)	0.012(2)
O4_9	0.047(3)	0.056(3)	0.0474(18)	0.0057(19)	−0.0013(17)	0.006(2)
C1_9	0.046(4)	0.071(5)	0.070(3)	0.000(3)	−0.005(3)	0.011(3)
C2_9	0.044(5)	0.063(3)	0.076(3)	0.003(2)	−0.007(3)	0.009(3)
C3_9	0.057(5)	0.064(3)	0.075(3)	0.005(3)	−0.002(4)	0.012(2)
C4_9	0.093(8)	0.088(4)	0.085(4)	0.020(3)	−0.006(4)	−0.004(4)
C5_9	0.145(5)	0.088(4)	0.109(4)	0.020(3)	−0.015(4)	−0.009(4)
C6_9	0.142(5)	0.089(4)	0.110(4)	0.019(3)	−0.016(4)	−0.006(4)
C7_9	0.065(6)	0.062(3)	0.099(4)	0.001(3)	−0.018(4)	0.009(3)
C8_9	0.150(6)	0.090(4)	0.110(4)	0.021(3)	−0.017(4)	−0.014(4)

C9_9	0.157(7)	0.186(14)	0.156(7)	0.099(9)	−0.002(5)	0.001(8)
C10_9	0.244(12)	0.244(14)	0.168(7)	0.120(7)	0.009(6)	−0.043(10)
C11_9	0.244(12)	0.245(14)	0.167(7)	0.120(7)	0.011(6)	−0.043(10)
C12_9	0.244(12)	0.246(14)	0.166(7)	0.120(7)	0.012(6)	−0.044(10)
C13_9	0.194(8)	0.164(15)	0.151(7)	0.090(8)	0.030(6)	0.041(8)
C14_9	0.140(6)	0.089(4)	0.113(4)	0.020(3)	−0.017(4)	−0.004(4)
C15_9	0.091(8)	0.127(5)	0.132(5)	−0.001(4)	−0.011(5)	0.004(5)
C16_9	0.081(6)	0.093(5)	0.121(5)	−0.021(4)	−0.010(4)	0.008(5)
C17_9	0.081(6)	0.093(4)	0.121(4)	−0.023(4)	−0.008(4)	0.007(5)
C18_9	0.140(10)	0.126(5)	0.214(9)	0.001(4)	0.058(7)	0.010(5)
C19_9	0.123(7)	0.124(5)	0.214(8)	0.003(5)	0.067(6)	0.011(5)
C20_9	0.123(6)	0.124(5)	0.216(8)	0.002(5)	0.066(6)	0.011(5)
C21_9	0.126(6)	0.125(5)	0.217(8)	0.001(5)	0.064(6)	0.012(5)
C22_9	0.129(6)	0.125(5)	0.218(8)	0.000(5)	0.062(6)	0.011(5)
C23_9	0.129(7)	0.125(5)	0.218(8)	−0.002(5)	0.060(6)	0.011(5)
C24_9	0.048(3)	0.072(5)	0.053(3)	−0.002(3)	−0.005(3)	0.009(3)
C25_9	0.058(5)	0.073(6)	0.055(4)	−0.001(4)	−0.009(3)	0.005(4)
C26_9	0.100(6)	0.144(8)	0.070(4)	0.024(5)	−0.035(4)	−0.037(5)
C27_9	0.074(5)	0.103(7)	0.069(4)	−0.006(4)	−0.013(3)	−0.005(4)
C28_9	0.059(4)	0.106(7)	0.072(4)	−0.003(4)	−0.011(3)	0.000(4)
C29_9	0.139(6)	0.173(9)	0.083(6)	0.044(6)	−0.037(5)	−0.051(6)
C30_9	0.165(6)	0.223(11)	0.164(10)	0.081(8)	0.010(6)	−0.037(6)
C31_9	0.167(6)	0.224(11)	0.164(10)	0.081(7)	0.010(6)	−0.036(6)
C32_9	0.164(7)	0.188(12)	0.195(14)	0.050(10)	−0.033(7)	−0.057(7)
C33_9	0.164(6)	0.188(9)	0.156(10)	0.065(6)	−0.042(6)	−0.034(6)
C34_9	0.163(6)	0.188(9)	0.156(10)	0.064(6)	−0.042(6)	−0.034(6)
C35_9	0.055(4)	0.108(7)	0.103(6)	−0.001(5)	−0.019(4)	0.001(4)
C36_9	0.056(5)	0.118(10)	0.112(5)	0.020(5)	−0.026(4)	−0.007(5)
C37_9	0.092(5)	0.113(9)	0.107(5)	0.013(5)	−0.029(4)	−0.023(5)
C38_9	0.082(5)	0.098(8)	0.070(5)	0.001(5)	−0.018(4)	−0.010(5)
C39_9	0.127(5)	0.046(8)	0.125(5)	0.026(5)	−0.054(4)	−0.049(5)
C40_9	0.126(5)	0.103(8)	0.171(8)	0.058(8)	−0.055(4)	−0.056(5)
C41_9	0.126(5)	0.105(8)	0.171(8)	0.058(7)	−0.055(4)	−0.057(5)
C42_9	0.125(6)	0.184(16)	0.168(8)	0.055(8)	−0.060(5)	−0.075(7)
C43_9	0.126(6)	0.165(10)	0.123(5)	0.020(6)	−0.061(4)	−0.075(6)
C44_9	0.126(6)	0.166(10)	0.122(5)	0.021(6)	−0.060(4)	−0.074(6)
P1_10	0.0521(13)	0.077(2)	0.0583(15)	0.0087(14)	0.0052(11)	−0.0040(13)
O1_10	0.065(4)	0.071(3)	0.044(4)	−0.002(3)	0.013(3)	−0.006(2)
O2_10	0.063(4)	0.084(5)	0.0572(17)	0.013(2)	0.0086(18)	−0.016(4)
O3_10	0.0542(15)	0.080(5)	0.073(3)	0.014(3)	0.0046(19)	−0.006(2)
O4_10	0.062(3)	0.077(3)	0.078(4)	−0.002(2)	0.011(3)	−0.009(2)
C1_10	0.081(4)	0.072(4)	0.110(7)	0.018(4)	−0.030(5)	−0.013(3)
C2_10	0.070(6)	0.072(4)	0.073(3)	0.006(2)	−0.001(3)	0.001(3)
C3_10	0.042(5)	0.072(4)	0.074(3)	0.006(2)	0.006(2)	0.000(4)
C4_10	0.068(6)	0.072(4)	0.068(3)	0.002(2)	0.004(3)	0.006(4)
C5_10	0.055(3)	0.082(3)	0.068(3)	0.005(2)	0.006(2)	−0.008(3)
C6_10	0.054(3)	0.082(3)	0.068(3)	0.005(2)	0.005(3)	−0.008(3)
C7_10	0.041(4)	0.081(4)	0.072(3)	0.006(2)	0.002(3)	−0.014(3)
C8_10	0.059(3)	0.084(3)	0.069(3)	0.006(2)	0.004(3)	−0.008(3)
C9_10	0.085(5)	0.059(4)	0.072(5)	−0.014(4)	0.018(4)	−0.021(3)
C10_10	0.141(7)	0.076(4)	0.077(6)	0.002(4)	0.006(4)	−0.001(4)
C11_10	0.140(7)	0.076(4)	0.078(6)	0.005(4)	0.005(4)	0.001(4)
C12_10	0.138(7)	0.077(4)	0.079(6)	0.006(4)	0.005(4)	0.003(3)
C13_10	0.073(5)	0.077(4)	0.093(7)	0.013(4)	0.022(4)	−0.006(4)

C14_10	0.052(4)	0.081(4)	0.068(3)	0.002(2)	0.005(3)	−0.007(3)
C15_10	0.102(7)	0.079(4)	0.063(3)	0.004(3)	0.018(4)	−0.015(4)
C16_10	0.133(7)	0.084(4)	0.069(4)	0.003(3)	−0.001(4)	−0.010(4)
C17_10	0.134(7)	0.084(4)	0.069(4)	0.004(3)	−0.002(4)	−0.009(4)
C18_10	0.162(7)	0.095(8)	0.067(3)	0.005(4)	0.002(4)	−0.009(5)
C19_10	0.182(6)	0.141(7)	0.076(4)	−0.014(4)	−0.010(4)	0.020(5)
C20_10	0.184(6)	0.141(6)	0.076(4)	−0.014(4)	−0.010(4)	0.018(5)
C21_10	0.186(6)	0.142(6)	0.076(4)	−0.014(4)	−0.007(4)	0.018(5)
C22_10	0.187(6)	0.144(6)	0.075(4)	−0.015(4)	−0.007(4)	0.019(5)
C23_10	0.188(7)	0.145(7)	0.075(4)	−0.015(4)	−0.007(4)	0.020(5)
C24_10	0.067(4)	0.074(3)	0.078(7)	0.009(3)	−0.008(4)	−0.009(3)
C25_10	0.113(7)	0.104(6)	0.213(14)	0.068(8)	−0.091(9)	−0.043(5)
C26_10	0.168(8)	0.134(7)	0.334(19)	0.130(10)	−0.152(10)	−0.082(6)
C27_10	0.137(7)	0.096(6)	0.200(13)	0.053(7)	−0.085(8)	−0.049(5)
C28_10	0.098(5)	0.079(5)	0.109(9)	0.013(5)	−0.031(5)	−0.026(4)
C29_10	0.211(10)	0.211(12)	0.313(13)	0.167(10)	−0.042(9)	0.019(9)
C30_10	0.202(9)	0.179(11)	0.269(11)	0.107(9)	−0.074(8)	−0.002(7)
C31_10	0.202(9)	0.180(11)	0.268(11)	0.107(9)	−0.074(8)	−0.002(7)
C32_10	0.237(12)	0.230(16)	0.256(13)	0.102(11)	−0.069(9)	−0.015(11)
C33_10	0.250(11)	0.208(12)	0.277(11)	0.116(8)	−0.041(9)	−0.019(9)
C34_10	0.250(11)	0.208(12)	0.277(11)	0.116(8)	−0.042(9)	−0.020(9)
C35_10	0.091(5)	0.139(8)	0.062(7)	0.039(7)	−0.013(4)	−0.036(4)
C36_10	0.109(7)	0.155(7)	0.095(10)	0.048(6)	−0.012(7)	−0.052(5)
C37_10	0.152(6)	0.154(8)	0.137(12)	0.055(8)	−0.066(7)	−0.070(5)
C38_10	0.156(6)	0.097(7)	0.175(15)	0.028(8)	−0.076(7)	−0.058(5)
C39_10	0.197(7)	0.183(8)	0.104(9)	0.049(7)	−0.082(7)	−0.112(6)
C40_10	0.189(7)	0.240(10)	0.126(9)	0.029(9)	−0.093(7)	−0.121(6)
C41_10	0.190(7)	0.239(10)	0.126(9)	0.028(9)	−0.093(7)	−0.121(6)
C42_10	0.244(10)	0.260(11)	0.38(3)	0.083(13)	−0.069(13)	−0.093(8)
C43_10	0.239(9)	0.187(8)	0.168(12)	0.056(8)	−0.102(10)	−0.105(7)
C44_10	0.239(9)	0.186(8)	0.166(12)	0.056(8)	−0.102(10)	−0.105(7)
O1S_11	0.093(5)	0.084(5)	0.078(5)	−0.008(4)	−0.004(4)	0.018(4)
C2S_11	0.169(8)	0.161(9)	0.267(17)	0.044(9)	0.055(8)	0.094(7)
C3S_11	0.158(10)	0.155(12)	0.200(15)	−0.040(10)	0.032(9)	0.073(9)
C4S_11	0.236(11)	0.33(2)	0.217(13)	−0.157(15)	−0.143(11)	0.096(13)
C5S_11	0.60(5)	0.85(8)	0.246(15)	−0.33(3)	−0.24(2)	0.35(6)
O1S_12	0.059(3)	0.092(5)	0.071(4)	0.029(4)	−0.002(3)	0.000(3)
C2S_12	0.161(8)	0.138(6)	0.087(9)	0.033(6)	−0.005(5)	−0.069(6)
C3S_12	0.173(9)	0.272(15)	0.229(18)	−0.030(16)	0.051(11)	−0.148(12)
C4S_12	0.069(5)	0.136(7)	0.083(5)	0.005(5)	−0.011(4)	0.014(4)
C5S_12	0.060(4)	0.143(10)	0.090(8)	0.023(7)	−0.002(5)	0.013(5)
P1_13	0.0395(11)	0.0474(16)	0.0577(15)	−0.0083(12)	−0.0103(9)	0.0067(10)
O1_13	0.057(3)	0.048(2)	0.056(4)	−0.014(2)	−0.019(3)	0.011(2)
O2_13	0.049(3)	0.044(3)	0.055(3)	−0.005(3)	−0.016(2)	0.007(2)
O3_13	0.041(2)	0.048(3)	0.060(2)	−0.004(2)	−0.0110(17)	0.001(2)
O4_13	0.0408(15)	0.042(3)	0.058(3)	−0.010(2)	−0.0112(16)	0.0040(16)
C1_13	0.044(4)	0.045(4)	0.056(3)	0.003(2)	−0.004(2)	0.002(3)
C2_13	0.039(2)	0.081(7)	0.063(3)	−0.014(4)	−0.013(2)	0.010(3)
C3_13	0.040(2)	0.059(6)	0.064(3)	−0.014(4)	−0.0103(18)	0.004(2)
C4_13	0.063(4)	0.099(8)	0.074(4)	−0.034(5)	−0.010(3)	0.009(4)
C5_13	0.087(3)	0.220(7)	0.114(4)	−0.073(4)	−0.033(3)	−0.006(4)
C6_13	0.086(3)	0.217(7)	0.112(4)	−0.071(4)	−0.033(3)	−0.005(4)
C7_13	0.052(2)	0.059(6)	0.077(3)	0.008(4)	−0.028(2)	0.001(3)
C8_13	0.089(4)	0.222(7)	0.115(4)	−0.076(4)	−0.034(3)	−0.008(4)

C9_13	0.35(3)	0.218(10)	0.120(5)	−0.074(6)	−0.024(7)	0.021(10)
C10_13	0.323(15)	0.285(14)	0.134(6)	−0.101(7)	−0.026(9)	0.026(12)
C11_13	0.323(15)	0.286(13)	0.135(6)	−0.103(7)	−0.027(9)	0.026(12)
C12_13	0.322(15)	0.285(14)	0.134(6)	−0.105(7)	−0.027(9)	0.027(12)
C13_13	0.270(19)	0.279(9)	0.110(6)	−0.088(6)	−0.080(8)	0.100(10)
C14_13	0.088(3)	0.217(8)	0.112(4)	−0.072(5)	−0.034(3)	−0.005(4)
C15_13	0.091(4)	0.137(10)	0.114(5)	−0.008(6)	−0.026(3)	0.005(4)
C16_13	0.054(3)	0.085(6)	0.102(5)	0.019(4)	−0.024(3)	0.006(3)
C17_13	0.052(2)	0.085(6)	0.101(5)	0.020(4)	−0.023(3)	0.005(3)
C18_13	0.141(5)	0.239(17)	0.224(7)	−0.027(8)	−0.089(5)	−0.022(6)
C19_13	0.154(6)	0.340(10)	0.232(7)	−0.052(7)	−0.086(5)	−0.023(7)
C20_13	0.153(6)	0.339(9)	0.232(7)	−0.051(7)	−0.086(5)	−0.024(7)
C21_13	0.150(5)	0.337(9)	0.233(7)	−0.050(7)	−0.088(5)	−0.025(7)
C22_13	0.146(5)	0.336(9)	0.233(7)	−0.050(7)	−0.089(5)	−0.025(6)
C23_13	0.142(5)	0.336(10)	0.233(7)	−0.048(8)	−0.088(5)	−0.024(6)
C24_13	0.042(3)	0.036(3)	0.051(3)	−0.001(2)	−0.002(2)	0.000(3)
C25_13	0.062(5)	0.045(5)	0.053(3)	−0.004(3)	0.002(3)	0.000(3)
C26_13	0.079(5)	0.054(5)	0.055(3)	−0.006(3)	0.000(3)	0.013(4)
C27_13	0.054(4)	0.046(4)	0.058(3)	0.003(3)	0.006(3)	−0.004(3)
C28_13	0.049(4)	0.045(4)	0.057(3)	0.003(3)	−0.001(3)	0.004(3)
C29_13	0.090(7)	0.097(5)	0.059(3)	−0.020(3)	−0.009(3)	0.037(5)
C30_13	0.143(7)	0.102(5)	0.087(6)	−0.030(4)	−0.020(6)	0.029(5)
C31_13	0.145(7)	0.102(5)	0.088(6)	−0.031(4)	−0.020(6)	0.029(5)
C32_13	0.221(14)	0.117(6)	0.100(7)	−0.020(4)	0.001(7)	0.032(7)
C33_13	0.176(8)	0.114(5)	0.061(4)	−0.013(4)	0.012(5)	0.039(6)
C34_13	0.175(8)	0.114(5)	0.060(4)	−0.014(4)	0.012(5)	0.039(5)
C35_13	0.051(5)	0.049(5)	0.055(3)	0.000(3)	−0.004(3)	0.005(3)
C36_13	0.050(5)	0.048(5)	0.066(4)	0.004(3)	0.005(3)	0.003(3)
C37_13	0.061(4)	0.047(5)	0.067(4)	0.004(3)	0.014(3)	−0.002(3)
C38_13	0.054(4)	0.041(4)	0.064(4)	0.004(3)	0.016(3)	−0.008(3)
C39_13	0.066(5)	0.050(4)	0.084(5)	−0.013(3)	−0.001(3)	0.000(3)
C40_13	0.078(5)	0.056(5)	0.119(6)	0.003(4)	0.011(4)	0.006(3)
C41_13	0.078(5)	0.057(5)	0.119(6)	0.003(4)	0.012(4)	0.006(3)
C42_13	0.083(7)	0.107(7)	0.113(6)	0.003(5)	0.010(5)	0.010(5)
C43_13	0.105(6)	0.116(7)	0.108(5)	0.007(4)	0.029(4)	0.032(5)
C44_13	0.105(6)	0.116(7)	0.107(5)	0.007(4)	0.029(4)	0.032(5)
P1_14	0.0401(12)	0.083(2)	0.0431(13)	−0.0016(12)	−0.0009(9)	0.0120(11)
O1_14	0.037(3)	0.078(5)	0.045(2)	−0.007(2)	0.0012(17)	0.015(3)
O2_14	0.046(3)	0.088(4)	0.052(3)	−0.015(3)	0.001(2)	0.007(3)
O3_14	0.049(2)	0.093(3)	0.055(3)	0.012(2)	−0.015(2)	0.001(2)
O4_14	0.0424(17)	0.106(4)	0.035(3)	0.010(2)	−0.0031(17)	0.006(2)
C1_14	0.042(4)	0.105(4)	0.049(4)	0.022(3)	−0.012(3)	−0.009(2)
C2_14	0.069(2)	0.100(3)	0.052(5)	0.029(3)	−0.006(3)	−0.009(2)
C3_14	0.059(2)	0.091(4)	0.103(7)	0.010(5)	−0.001(3)	0.002(2)
C4_14	0.112(3)	0.096(4)	0.132(11)	0.000(5)	0.012(4)	−0.013(2)
C5_14	0.1192(18)	0.131(2)	0.135(2)	−0.0012(19)	0.0057(19)	0.0044(15)
C6_14	0.1174(14)	0.1285(15)	0.1290(15)	0.0023(13)	0.0046(13)	0.0044(12)
C7_14	0.1035(16)	0.114(2)	0.084(7)	0.031(3)	0.009(2)	0.0170(17)
C8_14	0.129(2)	0.134(2)	0.140(2)	−0.0040(14)	0.0066(17)	0.0015(15)
C9_14	0.150(2)	0.147(2)	0.147(2)	0.0003(12)	−0.0022(13)	0.0013(13)
C10_14	0.151(3)	0.148(2)	0.150(3)	−0.0002(16)	−0.001(2)	0.0003(16)
C11_14	0.151(3)	0.149(3)	0.149(2)	−0.0003(16)	−0.0021(15)	0.0005(18)
C12_14	0.150(2)	0.147(2)	0.147(2)	0.0003(12)	−0.0022(13)	0.0013(13)
C13_14	0.150(2)	0.147(2)	0.147(2)	0.0003(12)	−0.0022(13)	0.0013(13)

C14_14	0.1174(14)	0.1285(15)	0.1290(15)	0.0023(13)	0.0046(13)	0.0044(12)
C15_14	0.1174(14)	0.1285(15)	0.1290(15)	0.0023(13)	0.0046(13)	0.0044(12)
C16_14	0.1174(14)	0.1285(15)	0.1290(15)	0.0023(13)	0.0046(13)	0.0044(12)
C17_14	0.1174(14)	0.1285(15)	0.1290(15)	0.0023(13)	0.0046(13)	0.0044(12)
C18_14	0.173(5)	0.197(11)	0.293(13)	0.031(10)	0.081(6)	0.056(6)
C19_14	0.188(6)	0.240(8)	0.360(11)	−0.023(7)	0.048(7)	0.090(6)
C20_14	0.188(6)	0.241(8)	0.361(12)	−0.022(7)	0.048(7)	0.090(7)
C21_14	0.187(6)	0.240(8)	0.361(12)	−0.022(7)	0.048(7)	0.090(7)
C22_14	0.187(6)	0.240(8)	0.360(11)	−0.022(7)	0.048(7)	0.090(7)
C23_14	0.188(6)	0.240(8)	0.360(11)	−0.023(7)	0.048(7)	0.090(6)
C24_14	0.053(4)	0.108(4)	0.038(3)	0.016(3)	−0.009(3)	0.001(3)
C25_14	0.066(5)	0.108(4)	0.043(4)	0.016(3)	−0.004(3)	0.004(3)
C26_14	0.093(7)	0.147(3)	0.054(5)	0.008(2)	−0.018(4)	−0.005(2)
C27_14	0.048(5)	0.148(4)	0.041(4)	0.011(3)	−0.002(3)	−0.018(3)
C28_14	0.039(4)	0.146(4)	0.047(4)	0.015(3)	−0.009(3)	−0.007(4)
C29_14	0.150(4)	0.148(2)	0.150(2)	−0.0042(16)	−0.008(2)	−0.002(2)
C30_14	0.2446(19)	0.1480(18)	0.1674(17)	−0.0111(15)	0.0274(17)	−0.0167(17)
C31_14	0.2446(19)	0.1480(18)	0.1674(17)	−0.0111(15)	0.0274(17)	−0.0167(17)
C32_14	0.162(4)	0.155(3)	0.156(2)	−0.0014(17)	0.000(2)	0.002(2)
C33_14	0.2446(19)	0.1480(18)	0.1674(17)	−0.0111(15)	0.0274(17)	−0.0167(17)
C34_14	0.2446(19)	0.1480(18)	0.1674(17)	−0.0111(15)	0.0274(17)	−0.0167(17)
C35_14	0.051(5)	0.189(6)	0.049(5)	0.031(4)	0.006(3)	0.023(5)
C36_14	0.071(7)	0.231(7)	0.049(5)	0.035(5)	0.001(4)	0.002(5)
C37_14	0.056(6)	0.237(7)	0.075(4)	0.021(5)	−0.001(4)	0.000(5)
C38_14	0.078(7)	0.195(8)	0.059(5)	−0.011(5)	−0.022(4)	−0.013(6)
C39_14	0.078(7)	0.282(3)	0.093(3)	−0.002(3)	−0.020(4)	0.017(3)
C40_14	0.0690(18)	0.278(2)	0.0924(18)	−0.0039(19)	−0.0129(17)	0.0233(19)
C41_14	0.0690(18)	0.278(2)	0.0924(18)	−0.0039(19)	−0.0129(17)	0.0233(19)
C42_14	0.072(7)	0.273(3)	0.093(4)	−0.005(3)	−0.006(4)	0.029(3)
C43_14	0.0690(18)	0.278(2)	0.0924(18)	−0.0039(19)	−0.0129(17)	0.0233(19)
C44_14	0.0690(18)	0.278(2)	0.0924(18)	−0.0039(19)	−0.0129(17)	0.0233(19)
P1_4	0.0485(12)	0.0603(17)	0.0589(15)	0.0036(13)	−0.0035(10)	0.0134(12)
O1_4	0.048(3)	0.056(4)	0.053(2)	0.001(2)	−0.0094(19)	0.011(3)
O2_4	0.048(4)	0.0634(19)	0.067(4)	−0.003(2)	0.007(3)	0.0099(19)
O3_4	0.0464(16)	0.076(3)	0.068(4)	0.021(3)	−0.006(2)	0.012(2)
O4_4	0.044(3)	0.081(4)	0.066(2)	0.023(2)	0.003(2)	0.010(3)
C1_4	0.068(5)	0.070(5)	0.081(4)	0.027(3)	0.012(3)	0.008(4)
C2_4	0.065(6)	0.071(3)	0.090(4)	0.024(3)	0.007(4)	0.004(4)
C3_4	0.046(5)	0.076(3)	0.083(4)	0.022(3)	0.016(4)	0.007(2)
C4_4	0.086(7)	0.075(4)	0.086(4)	0.024(3)	0.010(4)	−0.012(4)
C5_4	0.163(5)	0.075(4)	0.138(4)	0.019(3)	−0.005(4)	−0.013(4)
C6_4	0.160(5)	0.074(4)	0.139(4)	0.019(3)	−0.004(4)	−0.012(4)
C7_4	0.060(6)	0.071(4)	0.129(5)	0.021(3)	0.016(4)	0.003(4)
C8_4	0.169(5)	0.076(4)	0.138(5)	0.020(3)	−0.006(4)	−0.014(4)
C9_4	0.171(5)	0.242(9)	0.161(5)	−0.042(5)	−0.009(4)	−0.007(4)
C10_4	0.171(5)	0.242(9)	0.161(5)	−0.042(5)	−0.009(4)	−0.007(4)
C11_4	0.172(5)	0.242(9)	0.161(5)	−0.041(5)	−0.009(4)	−0.007(5)
C12_4	0.171(5)	0.242(9)	0.161(5)	−0.042(5)	−0.009(4)	−0.007(4)
C13_4	0.171(5)	0.242(9)	0.161(5)	−0.042(5)	−0.009(4)	−0.007(4)
C14_4	0.158(6)	0.074(4)	0.140(5)	0.019(3)	−0.005(4)	−0.012(4)
C15_4	0.081(7)	0.090(4)	0.143(5)	0.030(3)	0.019(5)	−0.010(4)
C16_4	0.093(6)	0.081(4)	0.143(5)	0.032(4)	0.008(4)	0.010(5)
C17_4	0.092(6)	0.080(4)	0.143(5)	0.033(4)	0.010(4)	0.009(5)
C18_4	0.063(6)	0.091(4)	0.149(7)	0.026(4)	−0.029(5)	−0.009(4)

C19_4	0.134(6)	0.091(4)	0.160(6)	0.027(4)	−0.004(5)	0.000(5)
C20_4	0.134(6)	0.091(4)	0.161(6)	0.027(4)	−0.003(5)	−0.001(4)
C21_4	0.135(6)	0.091(5)	0.161(6)	0.027(4)	−0.002(5)	−0.001(4)
C22_4	0.135(6)	0.090(4)	0.161(6)	0.027(4)	−0.002(5)	−0.001(4)
C23_4	0.134(6)	0.090(4)	0.160(7)	0.028(4)	0.000(5)	−0.002(5)
C24_4	0.056(4)	0.057(5)	0.071(3)	0.020(3)	0.012(3)	0.021(3)
C25_4	0.083(6)	0.078(6)	0.066(3)	0.022(4)	0.010(3)	0.001(5)
C26_4	0.094(6)	0.125(8)	0.070(3)	0.040(4)	0.018(4)	0.032(5)
C27_4	0.129(7)	0.169(9)	0.091(5)	0.033(5)	0.037(4)	−0.005(6)
C28_4	0.083(5)	0.102(7)	0.094(4)	0.047(4)	0.028(4)	0.034(4)
C29_4	0.130(9)	0.238(8)	0.093(5)	−0.002(5)	0.008(6)	0.013(7)
C30_4	0.206(13)	0.245(8)	0.131(10)	−0.006(6)	−0.029(8)	−0.008(7)
C31_4	0.207(13)	0.246(8)	0.133(10)	−0.007(6)	−0.029(8)	−0.008(7)
C32_4	0.171(14)	0.264(9)	0.127(11)	−0.003(7)	−0.015(10)	0.001(8)
C33_4	0.220(12)	0.263(9)	0.127(8)	0.014(7)	−0.051(8)	−0.002(8)
C34_4	0.220(12)	0.264(9)	0.128(8)	0.014(7)	−0.051(7)	−0.002(8)
C35_4	0.092(6)	0.115(8)	0.123(6)	0.034(6)	0.052(4)	0.027(5)
C36_4	0.108(8)	0.108(10)	0.126(6)	0.040(5)	0.060(5)	0.046(6)
C37_4	0.159(8)	0.104(10)	0.120(6)	0.050(6)	0.058(5)	0.020(6)
C38_4	0.150(7)	0.080(9)	0.109(5)	0.049(6)	0.057(5)	0.035(6)
C39_4	0.160(7)	0.150(13)	0.118(6)	0.051(7)	0.057(5)	0.028(8)
C40_4	0.169(7)	0.35(2)	0.167(9)	0.041(12)	0.073(6)	−0.011(9)
C41_4	0.169(8)	0.35(2)	0.167(9)	0.041(12)	0.072(6)	−0.012(9)
C42_4	0.194(9)	0.187(15)	0.190(10)	0.014(12)	0.056(7)	0.031(10)
C43_4	0.212(9)	0.41(2)	0.130(6)	0.011(11)	0.052(7)	−0.031(11)
C44_4	0.212(9)	0.41(2)	0.129(6)	0.011(11)	0.052(6)	−0.031(12)

Table 10.1.11. Bond lengths and angles for S-6a (ZC-01-51)

**Atom–Atom**	**Length [Å]**
Rh1–Rh2	2.4880(9)
Rh1–O2_5	2.094(5)
Rh1–O1S_7	2.337(7)
Rh1–O2_13	2.023(4)
Rh1–O2_14	2.009(4)
Rh1–O2_4	2.071(5)
Rh2–O1_5	2.038(4)
Rh2–O1S_11	2.227(7)
Rh2–O1_13	2.104(4)
Rh2–O1_14	2.091(4)
Rh2–O1_4	2.025(4)
Rh3–Rh4	2.4775(9)
Rh3–O2_1	2.032(5)
Rh3–O2_3	2.082(4)
Rh3–O1S_8	2.313(6)
Rh3–O2_9	2.043(5)
Rh3–O2_10	2.043(5)
Rh4–O1_1	2.058(5)
Rh4–O1_3	2.043(4)
Rh4–O1_9	2.067(4)
Rh4–O1_10	2.017(5)
Rh4–O1S_12	2.266(6)
Rh5–Rh5$^{\#1}$	2.4982(14)
Rh5–O	2.235(9)
Rh5–O1_2$^{\#1}$	2.014(6)
Rh5–O2_2	1.965(6)
Rh5–O1_6$^{\#1}$	1.954(6)
Rh5–O2_6	2.032(6)
O–HA	0.9991(15)
O–HB	0.9993(15)
P1_1–O1_1	1.500(3)
P1_1–O2_1	1.496(3)
P1_1–O3_1	1.586(3)
P1_1–O4_1	1.591(3)
O3_1–C3_1	1.404(4)
O4_1–C24_1	1.409(4)
C1_1–C2_1	1.502(5)
C1_1–C24_1	1.335(4)
C1_1–C28_1	1.439(5)
C2_1–C3_1	1.346(4)
C2_1–C7_1	1.423(4)
C3_1–C4_1	1.4129(17)
C4_1–H4_1	0.9500
C4_1–C5_1	1.393(4)
C5_1–C6_1	1.411(4)
C5_1–C8_1	1.518(5)
C6_1–C7_1	1.4459(17)
C6_1–C14_1	1.403(5)
C7_1–C17_1	1.380(5)
C8_1–C9_1	1.424(5)
C8_1–C13_1	1.370(5)

C9_1–H9_1	0.9500
C9_1–C10_1	1.428(6)
C10_1–H10_1	0.9500
C10_1–C11_1	1.378(7)
C11_1–H11_1	0.9500
C11_1–C12_1	1.320(6)
C12_1–H12_1	0.9500
C12_1–C13_1	1.445(6)
C13_1–H13_1	0.9500
C14_1–H14_1	0.9500
C14_1–C15_1	1.379(3)
C15_1–C16_1	1.379(3)
C15_1–C18_1	1.509(5)
C16_1–H16_1	0.9500
C16_1–C17_1	1.348(5)
C17_1–H17_1	0.9500
C18_1–C19_1	1.410(3)
C18_1–C23_1	1.414(3)
C19_1–H19_1	0.9500
C19_1–C20_1	1.405(3)
C20_1–H20_1	0.9500
C20_1–C21_1	1.381(3)
C21_1–H21_1	0.9500
C21_1–C22_1	1.385(3)
C22_1–H22_1	0.9500
C22_1–C23_1	1.403(3)
C23_1–H23_1	0.9500
C24_1–C25_1	1.4117(17)
C25_1–H25_1	0.9500
C25_1–C26_1	1.363(5)
C26_1–C27_1	1.407(5)
C26_1–C29_1	1.483(5)
C27_1–C28_1	1.4469(17)
C27_1–C38_1	1.417(5)
C28_1–C35_1	1.435(5)
C29_1–C30_1	1.3999(19)
C29_1–C34_1	1.3995(19)
C30_1–H30_1	0.9500
C30_1–C31_1	1.4009(19)
C31_1–H31_1	0.9500
C31_1–C32_1	1.3999(19)
C32_1–H32_1	0.9500
C32_1–C33_1	1.3998(19)
C33_1–H33_1	0.9500
C33_1–C34_1	1.3996(19)
C34_1–H34_1	0.9500
C35_1–H35_1	0.9500
C35_1–C36_1	1.338(5)
C36_1–H36_1	0.9500
C36_1–C37_1	1.379(3)
C37_1–C38_1	1.379(3)
C37_1–C39_1	1.484(4)
C38_1–H38_1	0.9500
C39_1–C40_1	1.404(5)
C39_1–C44_1	1.418(5)
C40_1–H40_1	0.9500
C40_1–C41_1	1.427(5)
C41_1–H41_1	0.9500
C41_1–C42_1	1.383(6)
C42_1–H42_1	0.9500
C42_1–C43_1	1.361(6)
C43_1–H43_1	0.9500
C43_1–C44_1	1.396(5)
C44_1–H44_1	0.9500
P1_2–O1_2	1.501(3)
P1_2–O2_2	1.496(3)
P1_2–O3_2	1.586(3)
P1_2–O4_2	1.592(3)
O3_2–C3_2	1.404(4)
O4_2–C24_2	1.407(4)
C1_2–C2_2	1.502(5)
C1_2–C24_2	1.335(4)
C1_2–C28_2	1.440(5)
C2_2–C3_2	1.348(4)
C2_2–C7_2	1.423(4)
C3_2–C4_2	1.4121(17)
C4_2–H4_2	0.9500
C4_2–C5_2	1.392(4)
C5_2–C6_2	1.412(4)
C5_2–C8_2	1.517(5)
C6_2–C7_2	1.4476(17)
C6_2–C14_2	1.404(5)
C7_2–C17_2	1.380(5)
C8_2–C9_2	1.425(5)
C8_2–C13_2	1.370(5)
C9_2–H9_2	0.9500
C9_2–C10_2	1.427(6)
C10_2–H10_2	0.9500
C10_2–C11_2	1.378(7)
C11_2–H11_2	0.9500
C11_2–C12_2	1.320(6)
C12_2–H12_2	0.9500
C12_2–C13_2	1.446(6)
C13_2–H13_2	0.9500
C14_2–H14_2	0.9500
C14_2–C15_2	1.379(3)
C15_2–C16_2	1.380(3)
C15_2–C18_2	1.511(5)
C16_2–H16_2	0.9500
C16_2–C17_2	1.348(5)
C17_2–H17_2	0.9500
C18_2–C19_2	1.409(3)
C18_2–C23_2	1.415(3)
C19_2–H19_2	0.9500
C19_2–C20_2	1.404(3)
C20_2–H20_2	0.9500
C20_2–C21_2	1.380(3)
C21_2–H21_2	0.9500

C21_2–C22_2	1.385(3)
C22_2–H22_2	0.9500
C22_2–C23_2	1.402(3)
C23_2–H23_2	0.9500
C24_2–C25_2	1.4111(17)
C25_2–H25_2	0.9500
C25_2–C26_2	1.363(5)
C26_2–C27_2	1.408(5)
C26_2–C29_2	1.482(5)
C27_2–C28_2	1.4472(17)
C27_2–C38_2	1.418(5)
C28_2–C35_2	1.435(5)
C29_2–C30_2	1.4011(19)
C29_2–C34_2	1.4007(19)
C30_2–H30_2	0.9500
C30_2–C31_2	1.400(2)
C31_2–H31_2	0.9500
C31_2–C32_2	1.4004(19)
C32_2–H32_2	0.9500
C32_2–C33_2	1.4011(19)
C33_2–H33_2	0.9500
C33_2–C34_2	1.401(2)
C34_2–H34_2	0.9500
C35_2–H35_2	0.9500
C35_2–C36_2	1.337(5)
C36_2–H36_2	0.9500
C36_2–C37_2	1.379(3)
C37_2–C38_2	1.378(3)
C37_2–C39_2	1.484(5)
C38_2–H38_2	0.9500
C39_2–C40_2	1.403(5)
C39_2–C44_2	1.418(5)
C40_2–H40_2	1.0780
C40_2–C41_2	1.426(5)
C41_2–H41_2	1.0780
C41_2–C42_2	1.383(6)
C42_2–H42_2	1.0780
C42_2–C43_2	1.361(6)
C43_2–H43_2	1.0780
C43_2–C44_2	1.397(5)
C44_2–H44_2	1.0780
P1_3–O1_3	1.503(3)
P1_3–O2_3	1.496(3)
P1_3–O3_3	1.587(3)
P1_3–O4_3	1.591(3)
O3_3–C3_3	1.406(4)
O4_3–C24_3	1.408(4)
C1_3–C2_3	1.503(5)
C1_3–C24_3	1.335(4)
C1_3–C28_3	1.441(5)
C2_3–C3_3	1.348(4)
C2_3–C7_3	1.422(4)
C3_3–C4_3	1.4126(17)
C4_3–H4_3	0.9500
C4_3–C5_3	1.392(4)
C5_3–C6_3	1.410(4)
C5_3–C8_3	1.517(5)
C6_3–C7_3	1.4467(17)
C6_3–C14_3	1.403(5)
C7_3–C17_3	1.380(5)
C8_3–C9_3	1.425(5)
C8_3–C13_3	1.370(5)
C9_3–H9_3	0.9500
C9_3–C10_3	1.427(6)
C10_3–H10_3	0.9500
C10_3–C11_3	1.378(7)
C11_3–H11_3	0.9500
C11_3–C12_3	1.320(6)
C12_3–H12_3	0.9500
C12_3–C13_3	1.445(6)
C13_3–H13_3	0.9500
C14_3–H14_3	0.9500
C14_3–C15_3	1.378(3)
C15_3–C16_3	1.378(3)
C15_3–C18_3	1.509(5)
C16_3–H16_3	0.9500
C16_3–C17_3	1.349(5)
C17_3–H17_3	0.9500
C18_3–C19_3	1.411(3)
C18_3–C23_3	1.414(3)
C19_3–H19_3	0.9500
C19_3–C20_3	1.405(3)
C20_3–H20_3	0.9500
C20_3–C21_3	1.381(3)
C21_3–H21_3	0.9500
C21_3–C22_3	1.385(3)
C22_3–H22_3	0.9500
C22_3–C23_3	1.402(3)
C23_3–H23_3	0.9500
C24_3–C25_3	1.4115(17)
C25_3–H25_3	0.9500
C25_3–C26_3	1.363(5)
C26_3–C27_3	1.407(5)
C26_3–C29_3	1.484(5)
C27_3–C28_3	1.4467(17)
C27_3–C38_3	1.419(5)
C28_3–C35_3	1.436(5)
C29_3–C30_3	1.3995(19)
C29_3–C34_3	1.4006(19)
C30_3–H30_3	0.9500
C30_3–C31_3	1.400(2)
C31_3–H31_3	0.9500
C31_3–C32_3	1.4005(19)
C32_3–H32_3	0.9500
C32_3–C33_3	1.3999(19)
C33_3–H33_3	0.9500
C33_3–C34_3	1.401(2)
C34_3–H34_3	0.9500

C35_3–H35_3	0.9500
C35_3–C36_3	1.338(5)
C36_3–H36_3	0.9500
C36_3–C37_3	1.380(3)
C37_3–C38_3	1.379(3)
C37_3–C39_3	1.484(4)
C38_3–H38_3	0.9500
C39_3–C40_3	1.404(5)
C39_3–C44_3	1.417(5)
C40_3–H40_3	0.9500
C40_3–C41_3	1.426(5)
C41_3–H41_3	0.9500
C41_3–C42_3	1.383(6)
C42_3–H42_3	0.9500
C42_3–C43_3	1.361(6)
C43_3–H43_3	0.9500
C43_3–C44_3	1.397(5)
C44_3–H44_3	0.9500
P1_5–O1_5	1.504(3)
P1_5–O2_5	1.494(3)
P1_5–O3_5	1.586(3)
P1_5–O4_5	1.590(3)
O3_5–C3_5	1.405(4)
O4_5–C24_5	1.405(4)
C1_5–C2_5	1.503(5)
C1_5–C24_5	1.336(4)
C1_5–C28_5	1.442(5)
C2_5–C3_5	1.348(4)
C2_5–C7_5	1.424(4)
C3_5–C4_5	1.4120(17)
C4_5–H4_5	0.9500
C4_5–C5_5	1.391(4)
C5_5–C6_5	1.410(4)
C5_5–C8_5	1.517(5)
C6_5–C7_5	1.4470(17)
C6_5–C14_5	1.403(5)
C7_5–C17_5	1.380(5)
C8_5–C9_5	1.425(5)
C8_5–C13_5	1.369(5)
C9_5–H9_5	0.9500
C9_5–C10_5	1.428(6)
C10_5–H10_5	0.9500
C10_5–C11_5	1.378(7)
C11_5–H11_5	0.9500
C11_5–C12_5	1.319(6)
C12_5–H12_5	0.9500
C12_5–C13_5	1.446(6)
C13_5–H13_5	0.9500
C14_5–H14_5	0.9500
C14_5–C15_5	1.379(3)
C15_5–C16_5	1.378(3)
C15_5–C18_5	1.509(5)
C16_5–H16_5	0.9500
C16_5–C17_5	1.348(5)
C17_5–H17_5	0.9500
C18_5–C19_5	1.407(2)
C18_5–C23_5	1.410(2)
C19_5–H19_5	0.9500
C19_5–C20_5	1.402(2)
C20_5–H20_5	0.9500
C20_5–C21_5	1.385(3)
C21_5–H21_5	0.9500
C21_5–C22_5	1.388(3)
C22_5–H22_5	0.9500
C22_5–C23_5	1.400(2)
C23_5–H23_5	0.9500
C24_5–C25_5	1.4124(17)
C25_5–H25_5	0.9500
C25_5–C26_5	1.362(5)
C26_5–C27_5	1.406(5)
C26_5–C29_5	1.484(5)
C27_5–C28_5	1.4471(17)
C27_5–C38_5	1.417(5)
C28_5–C35_5	1.436(5)
C29_5–C30_5	1.3989(16)
C29_5–C34_5	1.3992(16)
C30_5–H30_5	0.9500
C30_5–C31_5	1.3991(16)
C31_5–H31_5	0.9500
C31_5–C32_5	1.3988(16)
C32_5–H32_5	0.9500
C32_5–C33_5	1.3985(16)
C33_5–H33_5	0.9500
C33_5–C34_5	1.3987(16)
C34_5–H34_5	0.9500
C35_5–H35_5	0.9500
C35_5–C36_5	1.338(5)
C36_5–H36_5	0.9500
C36_5–C37_5	1.379(3)
C37_5–C38_5	1.378(3)
C37_5–C39_5	1.484(4)
C38_5–H38_5	0.9500
C39_5–C40_5	1.404(5)
C39_5–C44_5	1.418(5)
C40_5–H40_5	0.9500
C40_5–C41_5	1.426(5)
C41_5–H41_5	0.9500
C41_5–C42_5	1.384(6)
C42_5–H42_5	0.9500
C42_5–C43_5	1.361(6)
C43_5–H43_5	0.9500
C43_5–C44_5	1.397(5)
C44_5–H44_5	0.9500
P1_6–O1_6	1.501(3)
P1_6–O2_6	1.497(3)
P1_6–O3_6	1.585(3)
P1_6–O4_6	1.590(3)
O3_6–C3_6	1.404(4)

O4_6–C24_6	1.408(4)
C1_6–C2_6	1.502(5)
C1_6–C24_6	1.335(4)
C1_6–C28_6	1.440(5)
C2_6–C3_6	1.347(4)
C2_6–C7_6	1.423(4)
C3_6–C4_6	1.4129(17)
C4_6–H4_6	0.9500
C4_6–C5_6	1.392(4)
C5_6–C6_6	1.411(4)
C5_6–C8_6	1.517(5)
C6_6–C7_6	1.4467(17)
C6_6–C14_6	1.404(5)
C7_6–C17_6	1.380(5)
C8_6–C9_6	1.425(5)
C8_6–C13_6	1.369(5)
C9_6–H9_6	0.9500
C9_6–C10_6	1.427(6)
C10_6–H10_6	0.9500
C10_6–C11_6	1.378(7)
C11_6–H11_6	0.9500
C11_6–C12_6	1.320(6)
C12_6–H12_6	0.9500
C12_6–C13_6	1.444(6)
C13_6–H13_6	0.9500
C14_6–H14_6	0.9500
C14_6–C15_6	1.378(3)
C15_6–C16_6	1.378(3)
C15_6–C18_6	1.508(5)
C16_6–H16_6	0.9500
C16_6–C17_6	1.348(5)
C17_6–H17_6	0.9500
C18_6–C19_6	1.410(3)
C18_6–C23_6	1.414(3)
C19_6–H19_6	0.9500
C19_6–C20_6	1.405(3)
C20_6–H20_6	0.9500
C20_6–C21_6	1.380(3)
C21_6–H21_6	0.9500
C21_6–C22_6	1.386(3)
C22_6–H22_6	0.9500
C22_6–C23_6	1.402(3)
C23_6–H23_6	0.9500
C24_6–C25_6	1.4115(17)
C25_6–H25_6	0.9500
C25_6–C26_6	1.363(5)
C26_6–C27_6	1.407(5)
C26_6–C29_6	1.482(5)
C27_6–C28_6	1.4467(17)
C27_6–C38_6	1.417(5)
C28_6–C35_6	1.435(5)
C29_6–C30_6	1.4001(19)
C29_6–C34_6	1.4001(19)
C30_6–H30_6	0.9500
C30_6–C31_6	1.400(2)
C31_6–H31_6	0.9500
C31_6–C32_6	1.4001(19)
C32_6–H32_6	0.9500
C32_6–C33_6	1.4003(19)
C33_6–H33_6	0.9500
C33_6–C34_6	1.3996(19)
C34_6–H34_6	0.9500
C35_6–H35_6	0.9500
C35_6–C36_6	1.338(5)
C36_6–H36_6	0.9500
C36_6–C37_6	1.378(3)
C37_6–C38_6	1.379(3)
C37_6–C39_6	1.485(4)
C38_6–H38_6	0.9500
C39_6–C40_6	1.402(5)
C39_6–C44_6	1.419(5)
C40_6–H40_6	0.9500
C40_6–C41_6	1.427(5)
C41_6–H41_6	0.9500
C41_6–C42_6	1.383(6)
C42_6–H42_6	0.9500
C42_6–C43_6	1.361(6)
C43_6–H43_6	0.9500
C43_6–C44_6	1.397(5)
C44_6–H44_6	0.9500
O1S_7–C2S_7	1.405(9)
O1S_7–C4S_7	1.465(8)
C2S_7–H2SA_7	0.9900
C2S_7–H2SB_7	0.9900
C2S_7–C3S_7	1.521(4)
C3S_7–H3SA_7	0.9800
C3S_7–H3SB_7	0.9800
C3S_7–H3SC_7	0.9800
C4S_7–H4SA_7	0.9900
C4S_7–H4SB_7	0.9900
C4S_7–C5S_7	1.521(4)
C5S_7–H5SA_7	0.9800
C5S_7–H5SB_7	0.9800
C5S_7–H5SC_7	0.9800
O1S_8–C2S_8	1.405(9)
O1S_8–C4S_8	1.465(8)
C2S_8–H2SA_8	0.9900
C2S_8–H2SB_8	0.9900
C2S_8–C3S_8	1.521(4)
C3S_8–H3SA_8	0.9800
C3S_8–H3SB_8	0.9800
C3S_8–H3SC_8	0.9800
C4S_8–H4SA_8	0.9900
C4S_8–H4SB_8	0.9900
C4S_8–C5S_8	1.520(4)
C5S_8–H5SA_8	0.9800
C5S_8–H5SB_8	0.9800
C5S_8–H5SC_8	0.9800

P1_9–O1_9	1.504(3)
P1_9–O2_9	1.495(3)
P1_9–O3_9	1.587(3)
P1_9–O4_9	1.591(3)
O3_9–C3_9	1.405(4)
O4_9–C24_9	1.409(4)
C1_9–C2_9	1.502(5)
C1_9–C24_9	1.336(4)
C1_9–C28_9	1.439(5)
C2_9–C3_9	1.347(4)
C2_9–C7_9	1.422(4)
C3_9–C4_9	1.4121(17)
C4_9–H4_9	0.9500
C4_9–C5_9	1.393(4)
C5_9–C6_9	1.411(4)
C5_9–C8_9	1.517(5)
C6_9–C7_9	1.4467(17)
C6_9–C14_9	1.404(5)
C7_9–C17_9	1.380(5)
C8_9–C9_9	1.425(5)
C8_9–C13_9	1.371(5)
C9_9–H9_9	0.9500
C9_9–C10_9	1.427(6)
C10_9–H10_9	0.9500
C10_9–C11_9	1.378(7)
C11_9–H11_9	0.9500
C11_9–C12_9	1.320(6)
C12_9–H12_9	0.9500
C12_9–C13_9	1.445(6)
C13_9–H13_9	0.9500
C14_9–H14_9	0.9500
C14_9–C15_9	1.378(3)
C15_9–C16_9	1.379(3)
C15_9–C18_9	1.509(5)
C16_9–H16_9	0.9500
C16_9–C17_9	1.347(5)
C17_9–H17_9	0.9500
C18_9–C19_9	1.410(3)
C18_9–C23_9	1.415(3)
C19_9–H19_9	0.9500
C19_9–C20_9	1.405(3)
C20_9–H20_9	0.9500
C20_9–C21_9	1.380(3)
C21_9–H21_9	0.9500
C21_9–C22_9	1.385(3)
C22_9–H22_9	0.9500
C22_9–C23_9	1.401(3)
C23_9–H23_9	0.9500
C24_9–C25_9	1.4113(17)
C25_9–H25_9	0.9500
C25_9–C26_9	1.363(5)
C26_9–C27_9	1.407(5)
C26_9–C29_9	1.483(5)
C27_9–C28_9	1.4473(17)
C27_9–C38_9	1.419(5)
C28_9–C35_9	1.435(5)
C29_9–C30_9	1.4005(19)
C29_9–C34_9	1.4007(19)
C30_9–H30_9	0.9500
C30_9–C31_9	1.4006(19)
C31_9–H31_9	0.9500
C31_9–C32_9	1.4001(19)
C32_9–H32_9	0.9500
C32_9–C33_9	1.4003(19)
C33_9–H33_9	0.9500
C33_9–C34_9	1.4000(19)
C34_9–H34_9	0.9500
C35_9–H35_9	0.9500
C35_9–C36_9	1.338(5)
C36_9–H36_9	0.9500
C36_9–C37_9	1.379(3)
C37_9–C38_9	1.379(3)
C37_9–C39_9	1.485(4)
C38_9–H38_9	0.9500
C39_9–C40_9	1.404(5)
C39_9–C44_9	1.418(5)
C40_9–H40_9	0.9500
C40_9–C41_9	1.426(5)
C41_9–H41_9	0.9500
C41_9–C42_9	1.383(6)
C42_9–H42_9	0.9500
C42_9–C43_9	1.361(6)
C43_9–H43_9	0.9500
C43_9–C44_9	1.397(5)
C44_9–H44_9	0.9500
P1_10–O1_10	1.504(3)
P1_10–O2_10	1.494(3)
P1_10–O3_10	1.585(3)
P1_10–O4_10	1.590(3)
O3_10–C3_10	1.404(4)
O4_10–C24_10	1.405(4)
C1_10–C2_10	1.502(5)
C1_10–C24_10	1.334(4)
C1_10–C28_10	1.441(5)
C2_10–C3_10	1.347(4)
C2_10–C7_10	1.422(4)
C3_10–C4_10	1.4113(17)
C4_10–H4_10	0.9500
C4_10–C5_10	1.393(4)
C5_10–C6_10	1.411(4)
C5_10–C8_10	1.517(5)
C6_10–C7_10	1.4477(17)
C6_10–C14_10	1.404(5)
C7_10–C17_10	1.380(5)
C8_10–C9_10	1.425(5)
C8_10–C13_10	1.371(5)
C9_10–H9_10	0.9500
C9_10–C10_10	1.427(6)

C10_10–H10_10	0.9500
C10_10–C11_10	1.378(7)
C11_10–H11_10	0.9500
C11_10–C12_10	1.321(6)
C12_10–H12_10	0.9500
C12_10–C13_10	1.444(6)
C13_10–H13_10	0.9500
C14_10–H14_10	0.9500
C14_10–C15_10	1.379(3)
C15_10–C16_10	1.379(3)
C15_10–C18_10	1.509(5)
C16_10–H16_10	0.9500
C16_10–C17_10	1.348(5)
C17_10–H17_10	0.9500
C18_10–C19_10	1.410(3)
C18_10–C23_10	1.415(3)
C19_10–H19_10	0.9500
C19_10–C20_10	1.404(3)
C20_10–H20_10	0.9500
C20_10–C21_10	1.381(3)
C21_10–H21_10	0.9500
C21_10–C22_10	1.385(3)
C22_10–H22_10	0.9500
C22_10–C23_10	1.403(3)
C23_10–H23_10	0.9500
C24_10–C25_10	1.4128(17)
C25_10–H25_10	0.9500
C25_10–C26_10	1.363(5)
C26_10–C27_10	1.406(5)
C26_10–C29_10	1.483(5)
C27_10–C28_10	1.4474(17)
C27_10–C38_10	1.418(5)
C28_10–C35_10	1.436(5)
C29_10–C30_10	1.4010(19)
C29_10–C34_10	1.4010(19)
C30_10–H30_10	0.9500
C30_10–C31_10	1.4007(19)
C31_10–H31_10	0.9500
C31_10–C32_10	1.4005(19)
C32_10–H32_10	0.9500
C32_10–C33_10	1.4003(19)
C33_10–H33_10	0.9500
C33_10–C34_10	1.4002(19)
C34_10–H34_10	0.9500
C35_10–H35_10	0.9500
C35_10–C36_10	1.338(5)
C36_10–H36_10	0.9500
C36_10–C37_10	1.379(3)
C37_10–C38_10	1.379(3)
C37_10–C39_10	1.484(5)
C38_10–H38_10	0.9500
C39_10–C40_10	1.404(5)
C39_10–C44_10	1.418(5)
C40_10–H40_10	0.9500
C40_10–C41_10	1.426(5)
C41_10–H41_10	0.9500
C41_10–C42_10	1.383(6)
C42_10–H42_10	0.9500
C42_10–C43_10	1.361(6)
C43_10–H43_10	0.9500
C43_10–C44_10	1.396(5)
C44_10–H44_10	0.9500
O1S_11–C2S_11	1.406(9)
O1S_11–C4S_11	1.464(8)
C2S_11–H2SA_11	0.9900
C2S_11–H2SB_11	0.9900
C2S_11–C3S_11	1.522(3)
C3S_11–H3SA_11	0.9800
C3S_11–H3SB_11	0.9800
C3S_11–H3SC_11	0.9800
C4S_11–H4SA_11	0.9900
C4S_11–H4SB_11	0.9900
C4S_11–C5S_11	1.521(3)
C5S_11–H5SA_11	0.9800
C5S_11–H5SB_11	0.9800
C5S_11–H5SC_11	0.9800
O1S_12–C2S_12	1.406(8)
O1S_12–C4S_12	1.464(8)
C2S_12–H2SA_12	0.9900
C2S_12–H2SB_12	0.9900
C2S_12–C3S_12	1.521(4)
C3S_12–H3SA_12	0.9800
C3S_12–H3SB_12	0.9800
C3S_12–H3SC_12	0.9800
C4S_12–H4SA_12	0.9900
C4S_12–H4SB_12	0.9900
C4S_12–C5S_12	1.522(4)
C5S_12–H5SA_12	0.9800
C5S_12–H5SB_12	0.9800
C5S_12–H5SC_12	0.9800
P1_13–O1_13	1.499(3)
P1_13–O2_13	1.498(3)
P1_13–O3_13	1.587(3)
P1_13–O4_13	1.592(3)
O3_13–C3_13	1.405(4)
O4_13–C24_13	1.410(4)
C1_13–C2_13	1.503(5)
C1_13–C24_13	1.336(4)
C1_13–C28_13	1.440(5)
C2_13–C3_13	1.348(4)
C2_13–C7_13	1.424(4)
C3_13–C4_13	1.4129(17)
C4_13–H4_13	0.9500
C4_13–C5_13	1.393(4)
C5_13–C6_13	1.410(4)
C5_13–C8_13	1.518(5)
C6_13–C7_13	1.4465(17)
C6_13–C14_13	1.404(5)

C7_13–C17_13	1.380(5)	C37_13–C39_13	1.485(4)
C8_13–C9_13	1.425(5)	C38_13–H38_13	0.9500
C8_13–C13_13	1.371(5)	C39_13–C40_13	1.403(5)
C9_13–H9_13	0.9500	C39_13–C44_13	1.418(5)
C9_13–C10_13	1.427(6)	C40_13–H40_13	0.9500
C10_13–H10_13	0.9500	C40_13–C41_13	1.426(5)
C10_13–C11_13	1.378(7)	C41_13–H41_13	0.9500
C11_13–H11_13	0.9500	C41_13–C42_13	1.383(6)
C11_13–C12_13	1.320(6)	C42_13–H42_13	0.9500
C12_13–H12_13	0.9500	C42_13–C43_13	1.361(6)
C12_13–C13_13	1.445(6)	C43_13–H43_13	0.9500
C13_13–H13_13	0.9500	C43_13–C44_13	1.396(5)
C14_13–H14_13	0.9500	C44_13–H44_13	0.9500
C14_13–C15_13	1.378(3)	P1_14–O1_14	1.500(3)
C15_13–C16_13	1.379(3)	P1_14–O2_14	1.499(3)
C15_13–C18_13	1.509(5)	P1_14–O3_14	1.583(3)
C16_13–H16_13	0.9500	P1_14–O4_14	1.590(3)
C16_13–C17_13	1.348(5)	O3_14–C3_14	1.403(4)
C17_13–H17_13	0.9500	O4_14–C24_14	1.410(4)
C18_13–C19_13	1.410(3)	C1_14–C2_14	1.503(5)
C18_13–C23_13	1.415(3)	C1_14–C24_14	1.335(4)
C19_13–H19_13	0.9500	C1_14–C28_14	1.440(5)
C19_13–C20_13	1.405(3)	C2_14–C3_14	1.348(4)
C20_13–H20_13	0.9500	C2_14–C7_14	1.424(4)
C20_13–C21_13	1.380(3)	C3_14–C4_14	1.4138(17)
C21_13–H21_13	0.9500	C4_14–H4_14	0.9500
C21_13–C22_13	1.384(3)	C4_14–C5_14	1.391(4)
C22_13–H22_13	0.9500	C5_14–C6_14	1.410(4)
C22_13–C23_13	1.402(3)	C5_14–C8_14	1.516(5)
C23_13–H23_13	0.9500	C6_14–C7_14	1.4460(17)
C24_13–C25_13	1.4115(17)	C6_14–C14_14	1.403(5)
C25_13–H25_13	0.9500	C7_14–C17_14	1.380(5)
C25_13–C26_13	1.362(5)	C8_14–C9_14	1.423(5)
C26_13–C27_13	1.406(5)	C8_14–C13_14	1.368(5)
C26_13–C29_13	1.484(5)	C9_14–H9_14	0.9500
C27_13–C28_13	1.4476(17)	C9_14–C10_14	1.427(6)
C27_13–C38_13	1.418(5)	C10_14–H10_14	0.9500
C28_13–C35_13	1.435(5)	C10_14–C11_14	1.378(7)
C29_13–C30_13	1.4004(19)	C11_14–H11_14	0.9500
C29_13–C34_13	1.4013(19)	C11_14–C12_14	1.320(6)
C30_13–H30_13	0.9500	C12_14–H12_14	0.9500
C30_13–C31_13	1.4002(19)	C12_14–C13_14	1.445(6)
C31_13–H31_13	0.9500	C13_14–H13_14	0.9500
C31_13–C32_13	1.3993(19)	C14_14–H14_14	0.9500
C32_13–H32_13	0.9500	C14_14–C15_14	1.378(3)
C32_13–C33_13	1.4005(19)	C15_14–C16_14	1.378(3)
C33_13–H33_13	0.9500	C15_14–C18_14	1.510(5)
C33_13–C34_13	1.4002(19)	C16_14–H16_14	0.9500
C34_13–H34_13	0.9500	C16_14–C17_14	1.348(5)
C35_13–H35_13	0.9500	C17_14–H17_14	0.9500
C35_13–C36_13	1.337(5)	C18_14–C19_14	1.411(3)
C36_13–H36_13	0.9500	C18_14–C23_14	1.415(3)
C36_13–C37_13	1.379(3)	C19_14–H19_14	0.9500
C37_13–C38_13	1.378(3)	C19_14–C20_14	1.405(3)

C20_14–H20_14	0.9500	C2_4–C7_4	1.422(4)
C20_14–C21_14	1.380(3)	C3_4–C4_4	1.4119(17)
C21_14–H21_14	0.9500	C4_4–H4_4	0.9500
C21_14–C22_14	1.384(3)	C4_4–C5_4	1.393(4)
C22_14–H22_14	0.9500	C5_4–C6_4	1.411(4)
C22_14–C23_14	1.402(3)	C5_4–C8_4	1.517(5)
C23_14–H23_14	0.9500	C6_4–C7_4	1.4466(17)
C24_14–C25_14	1.4112(17)	C6_4–C14_4	1.404(5)
C25_14–H25_14	0.9500	C7_4–C17_4	1.380(5)
C25_14–C26_14	1.362(5)	C8_4–C9_4	1.425(5)
C26_14–C27_14	1.407(5)	C8_4–C13_4	1.370(5)
C26_14–C29_14	1.482(5)	C9_4–H9_4	1.0780
C27_14–C28_14	1.4476(17)	C9_4–C10_4	1.428(6)
C27_14–C38_14	1.417(5)	C10_4–H10_4	1.0780
C28_14–C35_14	1.435(5)	C10_4–C11_4	1.377(7)
C29_14–C30_14	1.3999(19)	C11_4–H11_4	1.0780
C29_14–C34_14	1.4006(19)	C11_4–C12_4	1.320(6)
C30_14–H30_14	0.9500	C12_4–H12_4	1.0780
C30_14–C31_14	1.4002(19)	C12_4–C13_4	1.446(6)
C31_14–H31_14	0.9500	C13_4–H13_4	1.0780
C31_14–C32_14	1.4004(19)	C14_4–H14_4	0.9500
C32_14–H32_14	0.9500	C14_4–C15_4	1.379(3)
C32_14–C33_14	1.4000(19)	C15_4–C16_4	1.378(3)
C33_14–H33_14	0.9500	C15_4–C18_4	1.508(5)
C33_14–C34_14	1.400(2)	C16_4–H16_4	0.9500
C34_14–H34_14	0.9500	C16_4–C17_4	1.347(5)
C35_14–H35_14	0.9500	C17_4–H17_4	0.9500
C35_14–C36_14	1.338(5)	C18_4–C19_4	1.410(3)
C36_14–H36_14	0.9500	C18_4–C23_4	1.415(3)
C36_14–C37_14	1.379(3)	C19_4–H19_4	0.9500
C37_14–C38_14	1.378(3)	C19_4–C20_4	1.405(3)
C37_14–C39_14	1.484(5)	C20_4–H20_4	0.9500
C38_14–H38_14	0.9500	C20_4–C21_4	1.380(3)
C39_14–C40_14	1.404(5)	C21_4–H21_4	0.9500
C39_14–C44_14	1.418(5)	C21_4–C22_4	1.385(3)
C40_14–H40_14	0.9500	C22_4–H22_4	0.9500
C40_14–C41_14	1.426(5)	C22_4–C23_4	1.402(3)
C41_14–H41_14	0.9500	C23_4–H23_4	0.9500
C41_14–C42_14	1.384(6)	C24_4–C25_4	1.4119(17)
C42_14–H42_14	0.9500	C25_4–H25_4	0.9500
C42_14–C43_14	1.361(6)	C25_4–C26_4	1.363(5)
C43_14–H43_14	0.9500	C26_4–C27_4	1.408(5)
C43_14–C44_14	1.396(5)	C26_4–C29_4	1.483(5)
C44_14–H44_14	0.9500	C27_4–C28_4	1.4474(17)
P1_4–O1_4	1.503(3)	C27_4–C38_4	1.418(5)
P1_4–O2_4	1.494(3)	C28_4–C35_4	1.436(5)
P1_4–O3_4	1.589(3)	C29_4–C30_4	1.3995(19)
P1_4–O4_4	1.588(3)	C29_4–C34_4	1.4013(19)
O3_4–C3_4	1.405(4)	C30_4–H30_4	0.9500
O4_4–C24_4	1.407(4)	C30_4–C31_4	1.4003(19)
C1_4–C2_4	1.503(5)	C31_4–H31_4	0.9500
C1_4–C24_4	1.335(4)	C31_4–C32_4	1.3997(19)
C1_4–C28_4	1.442(5)	C32_4–H32_4	0.9500
C2_4–C3_4	1.348(4)	C32_4–C33_4	1.4002(19)

C33_4–H33_4	0.9500
C33_4–C34_4	1.3998(19)
C34_4–H34_4	0.9500
C35_4–H35_4	0.9500
C35_4–C36_4	1.337(5)
C36_4–H36_4	0.9500
C36_4–C37_4	1.379(3)
C37_4–C38_4	1.378(3)
C37_4–C39_4	1.485(5)
C38_4–H38_4	0.9500
C39_4–C40_4	1.403(5)
C39_4–C44_4	1.418(5)
C40_4–H40_4	0.9500
C40_4–C41_4	1.426(5)
C41_4–H41_4	0.9500
C41_4–C42_4	1.383(6)
C42_4–H42_4	0.9500
C42_4–C43_4	1.361(6)
C43_4–H43_4	0.9500
C43_4–C44_4	1.396(5)
C44_4–H44_4	0.9500

**Atom–Atom–Atom**	**Angle [°]**
O2_5–Rh1–Rh2	89.62(11)
O2_5–Rh1–O1S_7	89.7(2)
O1S_7–Rh1–Rh2	179.02(18)
O2_13–Rh1–Rh2	91.16(11)
O2_13–Rh1–O2_5	88.21(19)
O2_13–Rh1–O1S_7	88.2(2)
O2_13–Rh1–O2_4	90.09(19)
O2_14–Rh1–Rh2	89.66(12)
O2_14–Rh1–O2_5	91.1(2)
O2_14–Rh1–O1S_7	91.0(2)
O2_14–Rh1–O2_13	178.90(19)
O2_14–Rh1–O2_4	90.7(2)
O2_4–Rh1–Rh2	88.06(12)
O2_4–Rh1–O2_5	177.09(18)
O2_4–Rh1–O1S_7	92.6(2)
O1_5–Rh2–Rh1	89.32(12)
O1_5–Rh2–O1S_11	91.4(2)
O1_5–Rh2–O1_13	87.05(18)
O1_5–Rh2–O1_14	92.77(18)
O1S_11–Rh2–Rh1	179.1(2)
O1_13–Rh2–Rh1	88.20(11)
O1_13–Rh2–O1S_11	91.2(2)
O1_14–Rh2–Rh1	89.91(11)
O1_14–Rh2–O1S_11	90.7(2)
O1_14–Rh2–O1_13	178.10(17)
O1_4–Rh2–Rh1	89.75(12)
O1_4–Rh2–O1_5	178.40(18)
O1_4–Rh2–O1S_11	89.6(2)
O1_4–Rh2–O1_13	94.22(18)
O1_4–Rh2–O1_14	85.92(18)
O2_1–Rh3–Rh4	89.32(11)
O2_1–Rh3–O2_3	177.82(18)
O2_1–Rh3–O1S_8	92.6(2)
O2_1–Rh3–O2_9	89.43(18)
O2_1–Rh3–O2_10	86.33(19)
O2_3–Rh3–Rh4	88.50(11)
O2_3–Rh3–O1S_8	89.6(2)
O1S_8–Rh3–Rh4	177.25(18)
O2_9–Rh3–Rh4	87.90(12)
O2_9–Rh3–O2_3	90.37(19)
O2_9–Rh3–O1S_8	94.1(2)
O2_9–Rh3–O2_10	175.10(19)
O2_10–Rh3–Rh4	89.59(12)
O2_10–Rh3–O2_3	93.8(2)
O2_10–Rh3–O1S_8	88.5(2)
O1_1–Rh4–Rh3	88.70(12)
O1_1–Rh4–O1_9	93.14(19)
O1_1–Rh4–O1S_12	90.77(19)
O1_3–Rh4–Rh3	88.71(11)
O1_3–Rh4–O1_1	176.79(18)
O1_3–Rh4–O1_9	88.69(18)
O1_3–Rh4–O1S_12	91.85(19)
O1_9–Rh4–Rh3	88.60(11)
O1_9–Rh4–O1S_12	90.3(2)
O1_10–Rh4–Rh3	89.74(12)
O1_10–Rh4–O1_1	90.18(19)
O1_10–Rh4–O1_3	87.91(18)
O1_10–Rh4–O1_9	176.25(18)
O1_10–Rh4–O1S_12	91.4(2)
O1S_12–Rh4–Rh3	178.76(18)
O–Rh5–Rh5$^{\#1}$	179.6(3)
O1_2$^{\#1}$–Rh5–Rh5$^{\#1}$	92.07(15)
O1_2$^{\#1}$–Rh5–O	87.5(3)
O1_2$^{\#1}$–Rh5–O2_6	176.7(2)
O2_2–Rh5–Rh5$^{\#1}$	88.47(15)
O2_2–Rh5–O	91.7(2)
O2_2–Rh5–O1_2$^{\#1}$	89.7(3)
O2_2–Rh5–O2_6	90.7(3)
O1_6$^{\#1}$–Rh5–Rh5$^{\#1}$	89.15(14)
O1_6$^{\#1}$–Rh5–O	90.7(2)
O1_6$^{\#1}$–Rh5–O1_2$^{\#1}$	91.0(3)
O1_6$^{\#1}$–Rh5–O2_2	177.5(2)
O1_6$^{\#1}$–Rh5–O2_6	88.8(3)
O2_6–Rh5–Rh5$^{\#1}$	91.26(13)
O2_6–Rh5–O	89.1(3)
Rh5–O–HA	116.1(14)
Rh5–O–HB	116.1(13)
HA–O–HB	123.3(4)
O1_1–P1_1–O3_1	112.4(2)
O1_1–P1_1–O4_1	105.4(2)
O2_1–P1_1–O1_1	118.7(2)
O2_1–P1_1–O3_1	105.7(2)
O2_1–P1_1–O4_1	110.6(2)
O3_1–P1_1–O4_1	103.1(2)
P1_1–O1_1–Rh4	114.7(3)

P1_1–O2_1–Rh3	117.1(3)
C3_1–O3_1–P1_1	116.8(3)
C24_1–O4_1–P1_1	118.0(3)
C24_1–C1_1–C2_1	118.7(4)
C24_1–C1_1–C28_1	118.1(4)
C28_1–C1_1–C2_1	123.2(4)
C3_1–C2_1–C1_1	118.1(3)
C3_1–C2_1–C7_1	118.6(4)
C7_1–C2_1–C1_1	123.2(4)
O3_1–C3_1–C4_1	115.1(3)
C2_1–C3_1–O3_1	120.3(3)
C2_1–C3_1–C4_1	124.6(4)
C3_1–C4_1–H4_1	121.1
C5_1–C4_1–C3_1	117.7(4)
C5_1–C4_1–H4_1	121.2
C4_1–C5_1–C6_1	120.8(4)
C4_1–C5_1–C8_1	117.1(4)
C6_1–C5_1–C8_1	121.4(4)
C5_1–C6_1–C7_1	119.2(4)
C14_1–C6_1–C5_1	122.3(4)
C14_1–C6_1–C7_1	118.5(4)
C2_1–C7_1–C6_1	119.2(4)
C17_1–C7_1–C2_1	124.7(4)
C17_1–C7_1–C6_1	116.1(4)
C9_1–C8_1–C5_1	119.4(4)
C13_1–C8_1–C5_1	120.0(4)
C13_1–C8_1–C9_1	120.4(3)
C8_1–C9_1–H9_1	120.8
C8_1–C9_1–C10_1	118.6(4)
C10_1–C9_1–H9_1	120.7
C9_1–C10_1–H10_1	121.8
C11_1–C10_1–C9_1	116.3(4)
C11_1–C10_1–H10_1	121.9
C10_1–C11_1–H11_1	115.9
C12_1–C11_1–C10_1	128.2(5)
C12_1–C11_1–H11_1	115.9
C11_1–C12_1–H12_1	122.4
C11_1–C12_1–C13_1	115.4(4)
C13_1–C12_1–H12_1	122.2
C8_1–C13_1–C12_1	121.1(4)
C8_1–C13_1–H13_1	119.5
C12_1–C13_1–H13_1	119.5
C6_1–C14_1–H14_1	119.2
C15_1–C14_1–C6_1	121.5(4)
C15_1–C14_1–H14_1	119.4
C14_1–C15_1–C18_1	120.2(4)
C16_1–C15_1–C14_1	119.4(4)
C16_1–C15_1–C18_1	120.1(4)
C15_1–C16_1–H16_1	120.3
C17_1–C16_1–C15_1	119.6(4)
C17_1–C16_1–H16_1	120.1
C7_1–C17_1–H17_1	117.6
C16_1–C17_1–C7_1	124.7(5)
C16_1–C17_1–H17_1	117.7
C19_1–C18_1–C15_1	121.9(4)
C19_1–C18_1–C23_1	118.1(2)
C23_1–C18_1–C15_1	119.8(4)
C18_1–C19_1–H19_1	119.5
C20_1–C19_1–C18_1	121.1(3)
C20_1–C19_1–H19_1	119.4
C19_1–C20_1–H20_1	120.8
C21_1–C20_1–C19_1	118.4(3)
C21_1–C20_1–H20_1	120.8
C20_1–C21_1–H21_1	118.6
C20_1–C21_1–C22_1	122.8(4)
C22_1–C21_1–H21_1	118.6
C21_1–C22_1–H22_1	120.9
C21_1–C22_1–C23_1	118.3(3)
C23_1–C22_1–H22_1	120.8
C18_1–C23_1–H23_1	119.5
C22_1–C23_1–C18_1	120.8(3)
C22_1–C23_1–H23_1	119.6
O4_1–C24_1–C25_1	115.5(4)
C1_1–C24_1–O4_1	119.9(3)
C1_1–C24_1–C25_1	124.0(4)
C24_1–C25_1–H25_1	120.5
C26_1–C25_1–C24_1	119.0(4)
C26_1–C25_1–H25_1	120.5
C25_1–C26_1–C27_1	121.1(4)
C25_1–C26_1–C29_1	117.4(4)
C27_1–C26_1–C29_1	120.9(4)
C26_1–C27_1–C28_1	118.6(4)
C26_1–C27_1–C38_1	124.4(4)
C38_1–C27_1–C28_1	116.9(4)
C1_1–C28_1–C27_1	119.2(4)
C35_1–C28_1–C1_1	124.0(4)
C35_1–C28_1–C27_1	116.7(4)
C30_1–C29_1–C26_1	122.1(4)
C34_1–C29_1–C26_1	118.9(4)
C34_1–C29_1–C30_1	118.9(2)
C29_1–C30_1–H30_1	119.5
C29_1–C30_1–C31_1	120.8(2)
C31_1–C30_1–H30_1	119.7
C30_1–C31_1–H31_1	120.1
C32_1–C31_1–C30_1	119.6(2)
C32_1–C31_1–H31_1	120.3
C31_1–C32_1–H32_1	119.8
C31_1–C32_1–C33_1	120.2(2)
C33_1–C32_1–H32_1	120.0
C32_1–C33_1–H33_1	120.2
C34_1–C33_1–C32_1	119.5(2)
C34_1–C33_1–H33_1	120.4
C29_1–C34_1–C33_1	121.0(2)
C29_1–C34_1–H34_1	119.6
C33_1–C34_1–H34_1	119.4
C28_1–C35_1–H35_1	118.3
C36_1–C35_1–C28_1	123.2(4)
C36_1–C35_1–H35_1	118.5

C35_1–C36_1–H36_1	119.6
C35_1–C36_1–C37_1	120.4(4)
C37_1–C36_1–H36_1	119.9
C36_1–C37_1–C38_1	119.5(4)
C36_1–C37_1–C39_1	121.7(4)
C38_1–C37_1–C39_1	118.8(4)
C27_1–C38_1–H38_1	118.6
C37_1–C38_1–C27_1	122.7(4)
C37_1–C38_1–H38_1	118.8
C40_1–C39_1–C37_1	120.3(4)
C40_1–C39_1–C44_1	118.6(3)
C44_1–C39_1–C37_1	121.1(4)
C39_1–C40_1–H40_1	119.9
C39_1–C40_1–C41_1	120.2(4)
C41_1–C40_1–H40_1	119.9
C40_1–C41_1–H41_1	121.1
C42_1–C41_1–C40_1	117.5(4)
C42_1–C41_1–H41_1	121.3
C41_1–C42_1–H42_1	117.8
C43_1–C42_1–C41_1	124.3(5)
C43_1–C42_1–H42_1	117.9
C42_1–C43_1–H43_1	121.0
C42_1–C43_1–C44_1	118.1(4)
C44_1–C43_1–H43_1	120.9
C39_1–C44_1–H44_1	119.4
C43_1–C44_1–C39_1	121.2(4)
C43_1–C44_1–H44_1	119.4
O1_2–P1_2–O3_2	112.5(3)
O1_2–P1_2–O4_2	104.9(3)
O2_2–P1_2–O1_2	118.7(3)
O2_2–P1_2–O3_2	105.7(3)
O2_2–P1_2–O4_2	110.6(3)
O3_2–P1_2–O4_2	103.3(2)
P1_2–O1_2–Rh5$^{\#1}$	115.6(3)
P1_2–O2_2–Rh5	121.1(4)
C3_2–O3_2–P1_2	116.4(3)
C24_2–O4_2–P1_2	118.0(3)
C24_2–C1_2–C2_2	118.7(4)
C24_2–C1_2–C28_2	117.7(4)
C28_2–C1_2–C2_2	123.5(4)
C3_2–C2_2–C1_2	117.6(4)
C3_2–C2_2–C7_2	118.7(4)
C7_2–C2_2–C1_2	123.6(4)
O3_2–C3_2–C4_2	115.3(4)
C2_2–C3_2–O3_2	120.2(4)
C2_2–C3_2–C4_2	124.4(4)
C3_2–C4_2–H4_2	121.2
C5_2–C4_2–C3_2	117.7(4)
C5_2–C4_2–H4_2	121.2
C4_2–C5_2–C6_2	121.0(4)
C4_2–C5_2–C8_2	117.4(4)
C6_2–C5_2–C8_2	121.5(4)
C5_2–C6_2–C7_2	118.9(4)
C14_2–C6_2–C5_2	121.5(4)
C14_2–C6_2–C7_2	118.3(4)
C2_2–C7_2–C6_2	119.1(4)
C17_2–C7_2–C2_2	124.3(4)
C17_2–C7_2–C6_2	116.0(4)
C9_2–C8_2–C5_2	119.3(4)
C13_2–C8_2–C5_2	120.1(4)
C13_2–C8_2–C9_2	120.4(3)
C8_2–C9_2–H9_2	120.6
C8_2–C9_2–C10_2	118.5(4)
C10_2–C9_2–H9_2	120.9
C9_2–C10_2–H10_2	121.6
C11_2–C10_2–C9_2	116.3(4)
C11_2–C10_2–H10_2	122.0
C10_2–C11_2–H11_2	115.7
C12_2–C11_2–C10_2	128.3(5)
C12_2–C11_2–H11_2	116.0
C11_2–C12_2–H12_2	122.3
C11_2–C12_2–C13_2	115.3(4)
C13_2–C12_2–H12_2	122.4
C8_2–C13_2–C12_2	121.1(4)
C8_2–C13_2–H13_2	119.6
C12_2–C13_2–H13_2	119.3
C6_2–C14_2–H14_2	119.0
C15_2–C14_2–C6_2	121.9(4)
C15_2–C14_2–H14_2	119.1
C14_2–C15_2–C16_2	119.3(4)
C14_2–C15_2–C18_2	119.4(4)
C16_2–C15_2–C18_2	119.4(4)
C15_2–C16_2–H16_2	120.4
C17_2–C16_2–C15_2	119.5(5)
C17_2–C16_2–H16_2	120.1
C7_2–C17_2–H17_2	117.4
C16_2–C17_2–C7_2	125.1(5)
C16_2–C17_2–H17_2	117.5
C19_2–C18_2–C15_2	121.6(4)
C19_2–C18_2–C23_2	118.1(2)
C23_2–C18_2–C15_2	119.6(4)
C18_2–C19_2–H19_2	119.4
C20_2–C19_2–C18_2	121.1(3)
C20_2–C19_2–H19_2	119.4
C19_2–C20_2–H20_2	120.8
C21_2–C20_2–C19_2	118.5(3)
C21_2–C20_2–H20_2	120.8
C20_2–C21_2–H21_2	118.6
C20_2–C21_2–C22_2	122.8(4)
C22_2–C21_2–H21_2	118.6
C21_2–C22_2–H22_2	120.8
C21_2–C22_2–C23_2	118.5(3)
C23_2–C22_2–H22_2	120.7
C18_2–C23_2–H23_2	119.5
C22_2–C23_2–C18_2	120.9(3)
C22_2–C23_2–H23_2	119.5
O4_2–C24_2–C25_2	115.9(4)
C1_2–C24_2–O4_2	119.9(4)

C1_2–C24_2–C25_2	124.1(4)
C24_2–C25_2–H25_2	120.4
C26_2–C25_2–C24_2	119.3(4)
C26_2–C25_2–H25_2	120.3
C25_2–C26_2–C27_2	120.8(4)
C25_2–C26_2–C29_2	117.8(4)
C27_2–C26_2–C29_2	121.5(4)
C26_2–C27_2–C28_2	118.4(4)
C26_2–C27_2–C38_2	124.2(4)
C38_2–C27_2–C28_2	116.7(4)
C1_2–C28_2–C27_2	119.5(4)
C35_2–C28_2–C1_2	123.3(4)
C35_2–C28_2–C27_2	117.0(4)
C30_2–C29_2–C26_2	122.1(4)
C34_2–C29_2–C26_2	119.3(4)
C34_2–C29_2–C30_2	118.5(2)
C29_2–C30_2–H30_2	119.7
C31_2–C30_2–C29_2	121.0(2)
C31_2–C30_2–H30_2	119.4
C30_2–C31_2–H31_2	120.3
C30_2–C31_2–C32_2	119.7(2)
C32_2–C31_2–H31_2	120.0
C31_2–C32_2–H32_2	120.2
C31_2–C32_2–C33_2	119.9(2)
C33_2–C32_2–H32_2	119.9
C32_2–C33_2–H33_2	120.5
C32_2–C33_2–C34_2	119.2(2)
C34_2–C33_2–H33_2	120.2
C29_2–C34_2–C33_2	120.9(3)
C29_2–C34_2–H34_2	119.4
C33_2–C34_2–H34_2	119.7
C28_2–C35_2–H35_2	118.4
C36_2–C35_2–C28_2	123.1(4)
C36_2–C35_2–H35_2	118.5
C35_2–C36_2–H36_2	119.7
C35_2–C36_2–C37_2	120.4(4)
C37_2–C36_2–H36_2	120.0
C36_2–C37_2–C39_2	121.6(4)
C38_2–C37_2–C36_2	119.6(4)
C38_2–C37_2–C39_2	118.8(4)
C27_2–C38_2–H38_2	118.6
C37_2–C38_2–C27_2	122.7(4)
C37_2–C38_2–H38_2	118.7
C40_2–C39_2–C37_2	120.2(4)
C40_2–C39_2–C44_2	118.6(3)
C44_2–C39_2–C37_2	121.1(4)
C39_2–C40_2–H40_2	119.9
C39_2–C40_2–C41_2	120.1(4)
C41_2–C40_2–H40_2	120.0
C40_2–C41_2–H41_2	121.0
C42_2–C41_2–C40_2	117.7(4)
C42_2–C41_2–H41_2	121.4
C41_2–C42_2–H42_2	117.7
C43_2–C42_2–C41_2	124.3(5)

C43_2–C42_2–H42_2	118.0
C42_2–C43_2–H43_2	121.0
C42_2–C43_2–C44_2	118.0(4)
C44_2–C43_2–H43_2	121.0
C39_2–C44_2–H44_2	119.5
C43_2–C44_2–C39_2	121.3(4)
C43_2–C44_2–H44_2	119.3
O1_3–P1_3–O3_3	112.5(2)
O1_3–P1_3–O4_3	105.2(2)
O2_3–P1_3–O1_3	118.7(2)
O2_3–P1_3–O3_3	105.5(2)
O2_3–P1_3–O4_3	110.7(2)
O3_3–P1_3–O4_3	103.2(2)
P1_3–O1_3–Rh4	114.4(2)
P1_3–O2_3–Rh3	115.9(2)
C3_3–O3_3–P1_3	115.7(3)
C24_3–O4_3–P1_3	118.5(3)
C24_3–C1_3–C2_3	118.8(3)
C24_3–C1_3–C28_3	118.0(4)
C28_3–C1_3–C2_3	122.9(4)
C3_3–C2_3–C1_3	117.4(3)
C3_3–C2_3–C7_3	118.6(4)
C7_3–C2_3–C1_3	123.8(4)
O3_3–C3_3–C4_3	115.4(3)
C2_3–C3_3–O3_3	119.9(3)
C2_3–C3_3–C4_3	124.6(4)
C3_3–C4_3–H4_3	121.2
C5_3–C4_3–C3_3	117.6(4)
C5_3–C4_3–H4_3	121.2
C4_3–C5_3–C6_3	120.8(4)
C4_3–C5_3–C8_3	117.7(4)
C6_3–C5_3–C8_3	121.4(4)
C5_3–C6_3–C7_3	119.3(4)
C14_3–C6_3–C5_3	121.9(4)
C14_3–C6_3–C7_3	118.8(4)
C2_3–C7_3–C6_3	119.0(4)
C17_3–C7_3–C2_3	125.1(4)
C17_3–C7_3–C6_3	115.8(4)
C9_3–C8_3–C5_3	119.4(4)
C13_3–C8_3–C5_3	120.1(4)
C13_3–C8_3–C9_3	120.3(3)
C8_3–C9_3–H9_3	120.7
C8_3–C9_3–C10_3	118.7(4)
C10_3–C9_3–H9_3	120.6
C9_3–C10_3–H10_3	121.8
C11_3–C10_3–C9_3	116.3(4)
C11_3–C10_3–H10_3	121.9
C10_3–C11_3–H11_3	116.0
C12_3–C11_3–C10_3	128.1(5)
C12_3–C11_3–H11_3	115.9
C11_3–C12_3–H12_3	122.4
C11_3–C12_3–C13_3	115.4(4)
C13_3–C12_3–H12_3	122.2
C8_3–C13_3–C12_3	121.2(4)

C8_3–C13_3–H13_3	119.4
C12_3–C13_3–H13_3	119.4
C6_3–C14_3–H14_3	119.3
C15_3–C14_3–C6_3	121.4(4)
C15_3–C14_3–H14_3	119.3
C14_3–C15_3–C18_3	119.9(4)
C16_3–C15_3–C14_3	119.5(4)
C16_3–C15_3–C18_3	120.5(4)
C15_3–C16_3–H16_3	120.4
C17_3–C16_3–C15_3	119.6(4)
C17_3–C16_3–H16_3	120.1
C7_3–C17_3–H17_3	117.4
C16_3–C17_3–C7_3	124.9(5)
C16_3–C17_3–H17_3	117.6
C19_3–C18_3–C15_3	121.6(4)
C19_3–C18_3–C23_3	118.0(2)
C23_3–C18_3–C15_3	119.8(4)
C18_3–C19_3–H19_3	119.5
C20_3–C19_3–C18_3	121.2(3)
C20_3–C19_3–H19_3	119.3
C19_3–C20_3–H20_3	120.9
C21_3–C20_3–C19_3	118.4(3)
C21_3–C20_3–H20_3	120.7
C20_3–C21_3–H21_3	118.7
C20_3–C21_3–C22_3	122.8(4)
C22_3–C21_3–H21_3	118.6
C21_3–C22_3–H22_3	120.8
C21_3–C22_3–C23_3	118.5(3)
C23_3–C22_3–H22_3	120.7
C18_3–C23_3–H23_3	119.5
C22_3–C23_3–C18_3	120.9(3)
C22_3–C23_3–H23_3	119.6
O4_3–C24_3–C25_3	115.8(3)
C1_3–C24_3–O4_3	120.3(3)
C1_3–C24_3–C25_3	123.9(4)
C24_3–C25_3–H25_3	120.4
C26_3–C25_3–C24_3	119.3(4)
C26_3–C25_3–H25_3	120.3
C25_3–C26_3–C27_3	120.9(4)
C25_3–C26_3–C29_3	117.4(4)
C27_3–C26_3–C29_3	121.0(4)
C26_3–C27_3–C28_3	118.5(4)
C26_3–C27_3–C38_3	124.7(4)
C38_3–C27_3–C28_3	116.8(4)
C1_3–C28_3–C27_3	119.4(4)
C35_3–C28_3–C1_3	123.2(4)
C35_3–C28_3–C27_3	116.7(4)
C30_3–C29_3–C26_3	122.0(4)
C30_3–C29_3–C34_3	118.8(2)
C34_3–C29_3–C26_3	119.1(4)
C29_3–C30_3–H30_3	119.4
C29_3–C30_3–C31_3	121.0(2)
C31_3–C30_3–H30_3	119.6
C30_3–C31_3–H31_3	120.2
C30_3–C31_3–C32_3	119.5(2)
C32_3–C31_3–H31_3	120.3
C31_3–C32_3–H32_3	119.8
C33_3–C32_3–C31_3	120.2(2)
C33_3–C32_3–H32_3	120.0
C32_3–C33_3–H33_3	120.1
C32_3–C33_3–C34_3	119.5(2)
C34_3–C33_3–H33_3	120.3
C29_3–C34_3–C33_3	120.9(2)
C29_3–C34_3–H34_3	119.6
C33_3–C34_3–H34_3	119.5
C28_3–C35_3–H35_3	118.4
C36_3–C35_3–C28_3	123.0(4)
C36_3–C35_3–H35_3	118.6
C35_3–C36_3–H36_3	119.8
C35_3–C36_3–C37_3	120.2(4)
C37_3–C36_3–H36_3	120.0
C36_3–C37_3–C38_3	119.1(4)
C36_3–C37_3–C39_3	121.3(4)
C38_3–C37_3–C39_3	119.2(4)
C27_3–C38_3–H38_3	118.7
C37_3–C38_3–C27_3	122.4(4)
C37_3–C38_3–H38_3	118.9
C40_3–C39_3–C37_3	119.9(4)
C40_3–C39_3–C44_3	118.7(3)
C44_3–C39_3–C37_3	121.3(4)
C39_3–C40_3–H40_3	119.9
C39_3–C40_3–C41_3	120.1(4)
C41_3–C40_3–H40_3	119.9
C40_3–C41_3–H41_3	121.1
C42_3–C41_3–C40_3	117.6(4)
C42_3–C41_3–H41_3	121.3
C41_3–C42_3–H42_3	117.8
C43_3–C42_3–C41_3	124.3(5)
C43_3–C42_3–H42_3	117.8
C42_3–C43_3–H43_3	121.0
C42_3–C43_3–C44_3	118.1(4)
C44_3–C43_3–H43_3	120.9
C39_3–C44_3–H44_3	119.4
C43_3–C44_3–C39_3	121.2(4)
C43_3–C44_3–H44_3	119.4
O1_5–P1_5–O3_5	112.2(2)
O1_5–P1_5–O4_5	104.5(2)
O2_5–P1_5–O1_5	118.4(2)
O2_5–P1_5–O3_5	106.1(2)
O2_5–P1_5–O4_5	111.6(2)
O3_5–P1_5–O4_5	103.1(2)
P1_5–O1_5–Rh2	117.1(2)
P1_5–O2_5–Rh1	116.7(3)
C3_5–O3_5–P1_5	116.1(3)
C24_5–O4_5–P1_5	120.0(3)
C24_5–C1_5–C2_5	118.9(4)
C24_5–C1_5–C28_5	117.7(4)
C28_5–C1_5–C2_5	122.8(4)

C3_5–C2_5–C1_5	117.5(3)
C3_5–C2_5–C7_5	118.4(4)
C7_5–C2_5–C1_5	124.1(4)
O3_5–C3_5–C4_5	115.2(3)
C2_5–C3_5–O3_5	120.0(3)
C2_5–C3_5–C4_5	124.8(4)
C3_5–C4_5–H4_5	121.2
C5_5–C4_5–C3_5	117.7(4)
C5_5–C4_5–H4_5	121.2
C4_5–C5_5–C6_5	120.7(4)
C4_5–C5_5–C8_5	117.6(4)
C6_5–C5_5–C8_5	121.6(4)
C5_5–C6_5–C7_5	119.4(4)
C14_5–C6_5–C5_5	121.9(4)
C14_5–C6_5–C7_5	118.7(4)
C2_5–C7_5–C6_5	119.0(4)
C17_5–C7_5–C2_5	124.9(4)
C17_5–C7_5–C6_5	115.8(4)
C9_5–C8_5–C5_5	119.3(4)
C13_5–C8_5–C5_5	120.2(4)
C13_5–C8_5–C9_5	120.4(3)
C8_5–C9_5–H9_5	120.8
C8_5–C9_5–C10_5	118.6(4)
C10_5–C9_5–H9_5	120.6
C9_5–C10_5–H10_5	121.9
C11_5–C10_5–C9_5	116.2(4)
C11_5–C10_5–H10_5	121.9
C10_5–C11_5–H11_5	115.9
C12_5–C11_5–C10_5	128.4(5)
C12_5–C11_5–H11_5	115.7
C11_5–C12_5–H12_5	122.5
C11_5–C12_5–C13_5	115.3(4)
C13_5–C12_5–H12_5	122.2
C8_5–C13_5–C12_5	121.1(4)
C8_5–C13_5–H13_5	119.4
C12_5–C13_5–H13_5	119.5
C6_5–C14_5–H14_5	119.2
C15_5–C14_5–C6_5	121.4(4)
C15_5–C14_5–H14_5	119.3
C14_5–C15_5–C18_5	119.8(4)
C16_5–C15_5–C14_5	119.6(4)
C16_5–C15_5–C18_5	120.5(4)
C15_5–C16_5–H16_5	120.5
C17_5–C16_5–C15_5	119.4(4)
C17_5–C16_5–H16_5	120.1
C7_5–C17_5–H17_5	117.4
C16_5–C17_5–C7_5	125.0(5)
C16_5–C17_5–H17_5	117.5
C19_5–C18_5–C15_5	121.7(4)
C19_5–C18_5–C23_5	118.2(2)
C23_5–C18_5–C15_5	120.0(4)
C18_5–C19_5–H19_5	119.3
C20_5–C19_5–C18_5	121.17(19)
C20_5–C19_5–H19_5	119.5
C19_5–C20_5–H20_5	120.7
C21_5–C20_5–C19_5	118.5(2)
C21_5–C20_5–H20_5	120.9
C20_5–C21_5–H21_5	118.6
C20_5–C21_5–C22_5	122.5(3)
C22_5–C21_5–H21_5	118.8
C21_5–C22_5–H22_5	120.7
C21_5–C22_5–C23_5	118.5(2)
C23_5–C22_5–H22_5	120.9
C18_5–C23_5–H23_5	119.5
C22_5–C23_5–C18_5	121.2(2)
C22_5–C23_5–H23_5	119.3
O4_5–C24_5–C25_5	114.6(4)
C1_5–C24_5–O4_5	121.4(4)
C1_5–C24_5–C25_5	123.9(4)
C24_5–C25_5–H25_5	120.3
C26_5–C25_5–C24_5	119.5(4)
C26_5–C25_5–H25_5	120.2
C25_5–C26_5–C27_5	120.6(4)
C25_5–C26_5–C29_5	117.4(4)
C27_5–C26_5–C29_5	121.6(4)
C26_5–C27_5–C28_5	118.6(4)
C26_5–C27_5–C38_5	124.4(4)
C38_5–C27_5–C28_5	117.0(4)
C1_5–C28_5–C27_5	119.5(4)
C35_5–C28_5–C1_5	123.4(4)
C35_5–C28_5–C27_5	116.2(4)
C30_5–C29_5–C26_5	121.9(4)
C30_5–C29_5–C34_5	118.76(19)
C34_5–C29_5–C26_5	119.3(4)
C29_5–C30_5–H30_5	119.6
C29_5–C30_5–C31_5	120.9(2)
C31_5–C30_5–H30_5	119.6
C30_5–C31_5–H31_5	120.2
C32_5–C31_5–C30_5	119.7(2)
C32_5–C31_5–H31_5	120.2
C31_5–C32_5–H32_5	119.9
C33_5–C32_5–C31_5	120.15(19)
C33_5–C32_5–H32_5	119.9
C32_5–C33_5–H33_5	120.3
C32_5–C33_5–C34_5	119.5(2)
C34_5–C33_5–H33_5	120.2
C29_5–C34_5–H34_5	119.5
C33_5–C34_5–C29_5	121.0(2)
C33_5–C34_5–H34_5	119.5
C28_5–C35_5–H35_5	118.3
C36_5–C35_5–C28_5	123.3(4)
C36_5–C35_5–H35_5	118.4
C35_5–C36_5–H36_5	119.7
C35_5–C36_5–C37_5	120.3(4)
C37_5–C36_5–H36_5	120.0
C36_5–C37_5–C39_5	121.9(4)
C38_5–C37_5–C36_5	119.4(4)
C38_5–C37_5–C39_5	118.6(4)

C27_5–C38_5–H38_5	118.5
C37_5–C38_5–C27_5	122.8(4)
C37_5–C38_5–H38_5	118.7
C40_5–C39_5–C37_5	120.2(4)
C40_5–C39_5–C44_5	118.6(3)
C44_5–C39_5–C37_5	121.2(4)
C39_5–C40_5–H40_5	119.8
C39_5–C40_5–C41_5	120.2(4)
C41_5–C40_5–H40_5	120.0
C40_5–C41_5–H41_5	121.0
C42_5–C41_5–C40_5	117.7(4)
C42_5–C41_5–H41_5	121.4
C41_5–C42_5–H42_5	117.8
C43_5–C42_5–C41_5	124.2(5)
C43_5–C42_5–H42_5	118.0
C42_5–C43_5–H43_5	120.9
C42_5–C43_5–C44_5	118.2(4)
C44_5–C43_5–H43_5	120.9
C39_5–C44_5–H44_5	119.5
C43_5–C44_5–C39_5	121.2(4)
C43_5–C44_5–H44_5	119.3
O1_6–P1_6–O3_6	112.4(3)
O1_6–P1_6–O4_6	105.3(3)
O2_6–P1_6–O1_6	118.5(3)
O2_6–P1_6–O3_6	105.6(3)
O2_6–P1_6–O4_6	110.3(3)
O3_6–P1_6–O4_6	103.7(2)
P1_6–O1_6–Rh5[#1]	121.1(3)
P1_6–O2_6–Rh5	115.8(3)
C3_6–O3_6–P1_6	116.7(3)
C24_6–O4_6–P1_6	118.2(3)
C24_6–C1_6–C2_6	118.7(4)
C24_6–C1_6–C28_6	117.7(4)
C28_6–C1_6–C2_6	123.4(4)
C3_6–C2_6–C1_6	118.1(3)
C3_6–C2_6–C7_6	118.5(4)
C7_6–C2_6–C1_6	123.3(4)
O3_6–C3_6–C4_6	115.0(3)
C2_6–C3_6–O3_6	120.5(3)
C2_6–C3_6–C4_6	124.5(4)
C3_6–C4_6–H4_6	121.1
C5_6–C4_6–C3_6	117.7(4)
C5_6–C4_6–H4_6	121.1
C4_6–C5_6–C6_6	120.9(4)
C4_6–C5_6–C8_6	117.5(4)
C6_6–C5_6–C8_6	121.6(4)
C5_6–C6_6–C7_6	118.9(4)
C14_6–C6_6–C5_6	121.7(4)
C14_6–C6_6–C7_6	118.5(4)
C2_6–C7_6–C6_6	119.2(4)
C17_6–C7_6–C2_6	124.5(4)
C17_6–C7_6–C6_6	116.1(4)
C9_6–C8_6–C5_6	119.4(4)
C13_6–C8_6–C5_6	120.3(4)
C13_6–C8_6–C9_6	120.3(3)
C8_6–C9_6–H9_6	120.8
C8_6–C9_6–C10_6	118.4(4)
C10_6–C9_6–H9_6	120.8
C9_6–C10_6–H10_6	121.6
C11_6–C10_6–C9_6	116.5(4)
C11_6–C10_6–H10_6	121.8
C10_6–C11_6–H11_6	115.9
C12_6–C11_6–C10_6	128.0(5)
C12_6–C11_6–H11_6	116.0
C11_6–C12_6–H12_6	122.4
C11_6–C12_6–C13_6	115.3(4)
C13_6–C12_6–H12_6	122.3
C8_6–C13_6–C12_6	121.3(4)
C8_6–C13_6–H13_6	119.4
C12_6–C13_6–H13_6	119.3
C6_6–C14_6–H14_6	119.2
C15_6–C14_6–C6_6	121.4(4)
C15_6–C14_6–H14_6	119.3
C14_6–C15_6–C18_6	120.1(4)
C16_6–C15_6–C14_6	119.7(4)
C16_6–C15_6–C18_6	120.2(4)
C15_6–C16_6–H16_6	120.4
C17_6–C16_6–C15_6	119.5(4)
C17_6–C16_6–H16_6	120.1
C7_6–C17_6–H17_6	117.6
C16_6–C17_6–C7_6	124.7(5)
C16_6–C17_6–H17_6	117.7
C19_6–C18_6–C15_6	121.8(4)
C19_6–C18_6–C23_6	118.3(2)
C23_6–C18_6–C15_6	119.7(4)
C18_6–C19_6–H19_6	119.6
C20_6–C19_6–C18_6	120.9(3)
C20_6–C19_6–H19_6	119.5
C19_6–C20_6–H20_6	120.8
C21_6–C20_6–C19_6	118.5(3)
C21_6–C20_6–H20_6	120.7
C20_6–C21_6–H21_6	118.6
C20_6–C21_6–C22_6	122.9(4)
C22_6–C21_6–H21_6	118.5
C21_6–C22_6–H22_6	120.8
C21_6–C22_6–C23_6	118.3(3)
C23_6–C22_6–H22_6	120.8
C18_6–C23_6–H23_6	119.5
C22_6–C23_6–C18_6	121.0(3)
C22_6–C23_6–H23_6	119.5
O4_6–C24_6–C25_6	115.4(4)
C1_6–C24_6–O4_6	119.8(4)
C1_6–C24_6–C25_6	124.3(4)
C24_6–C25_6–H25_6	120.5
C26_6–C25_6–C24_6	119.0(4)
C26_6–C25_6–H25_6	120.5
C25_6–C26_6–C27_6	120.7(4)
C25_6–C26_6–C29_6	117.8(4)

C27_6–C26_6–C29_6	121.4(4)
C26_6–C27_6–C28_6	118.9(4)
C26_6–C27_6–C38_6	124.2(4)
C38_6–C27_6–C28_6	116.9(4)
C1_6–C28_6–C27_6	119.2(4)
C35_6–C28_6–C1_6	123.8(4)
C35_6–C28_6–C27_6	116.8(4)
C30_6–C29_6–C26_6	122.1(4)
C30_6–C29_6–C34_6	118.7(2)
C34_6–C29_6–C26_6	119.1(4)
C29_6–C30_6–H30_6	119.6
C29_6–C30_6–C31_6	120.9(2)
C31_6–C30_6–H30_6	119.5
C30_6–C31_6–H31_6	120.3
C30_6–C31_6–C32_6	119.6(2)
C32_6–C31_6–H31_6	120.1
C31_6–C32_6–H32_6	120.0
C31_6–C32_6–C33_6	120.1(2)
C33_6–C32_6–H32_6	119.9
C32_6–C33_6–H33_6	120.3
C34_6–C33_6–C32_6	119.6(2)
C34_6–C33_6–H33_6	120.2
C29_6–C34_6–H34_6	119.4
C33_6–C34_6–C29_6	121.0(2)
C33_6–C34_6–H34_6	119.6
C28_6–C35_6–H35_6	118.3
C36_6–C35_6–C28_6	123.2(4)
C36_6–C35_6–H35_6	118.5
C35_6–C36_6–H36_6	119.6
C35_6–C36_6–C37_6	120.5(4)
C37_6–C36_6–H36_6	119.9
C36_6–C37_6–C38_6	119.5(4)
C36_6–C37_6–C39_6	122.4(4)
C38_6–C37_6–C39_6	118.1(4)
C27_6–C38_6–H38_6	118.6
C37_6–C38_6–C27_6	122.6(4)
C37_6–C38_6–H38_6	118.8
C40_6–C39_6–C37_6	120.6(4)
C40_6–C39_6–C44_6	118.7(3)
C44_6–C39_6–C37_6	120.5(4)
C39_6–C40_6–H40_6	119.9
C39_6–C40_6–C41_6	120.1(4)
C41_6–C40_6–H40_6	120.0
C40_6–C41_6–H41_6	121.0
C42_6–C41_6–C40_6	117.6(4)
C42_6–C41_6–H41_6	121.4
C41_6–C42_6–H42_6	117.8
C43_6–C42_6–C41_6	124.3(5)
C43_6–C42_6–H42_6	117.9
C42_6–C43_6–H43_6	121.0
C42_6–C43_6–C44_6	118.1(4)
C44_6–C43_6–H43_6	120.9
C39_6–C44_6–H44_6	119.5
C43_6–C44_6–C39_6	121.1(4)
C43_6–C44_6–H44_6	119.4
C2S_7–O1S_7–Rh1	129.6(9)
C2S_7–O1S_7–C4S_7	116.7(7)
C4S_7–O1S_7–Rh1	111.5(8)
O1S_7–C2S_7–H2SA_7	107.5
O1S_7–C2S_7–H2SB_7	108.3
O1S_7–C2S_7–C3S_7	116.4(8)
H2SA_7–C2S_7–H2SB_7	107.3
C3S_7–C2S_7–H2SA_7	107.1
C3S_7–C2S_7–H2SB_7	109.8
C2S_7–C3S_7–H3SA_7	108.6
C2S_7–C3S_7–H3SB_7	111.3
C2S_7–C3S_7–H3SC_7	108.5
H3SA_7–C3S_7–H3SB_7	109.5
H3SA_7–C3S_7–H3SC_7	109.5
H3SB_7–C3S_7–H3SC_7	109.5
O1S_7–C4S_7–H4SA_7	109.5
O1S_7–C4S_7–H4SB_7	108.1
O1S_7–C4S_7–C5S_7	112.7(7)
H4SA_7–C4S_7–H4SB_7	107.8
C5S_7–C4S_7–H4SA_7	110.7
C5S_7–C4S_7–H4SB_7	107.8
C4S_7–C5S_7–H5SA_7	108.9
C4S_7–C5S_7–H5SB_7	108.3
C4S_7–C5S_7–H5SC_7	111.2
H5SA_7–C5S_7–H5SB_7	109.5
H5SA_7–C5S_7–H5SC_7	109.5
H5SB_7–C5S_7–H5SC_7	109.5
C2S_8–O1S_8–Rh3	123.8(6)
C2S_8–O1S_8–C4S_8	116.8(7)
C4S_8–O1S_8–Rh3	119.4(6)
O1S_8–C2S_8–H2SA_8	108.6
O1S_8–C2S_8–H2SB_8	108.0
O1S_8–C2S_8–C3S_8	116.1(8)
H2SA_8–C2S_8–H2SB_8	107.3
C3S_8–C2S_8–H2SA_8	109.3
C3S_8–C2S_8–H2SB_8	107.2
C2S_8–C3S_8–H3SA_8	109.3
C2S_8–C3S_8–H3SB_8	108.5
C2S_8–C3S_8–H3SC_8	110.6
H3SA_8–C3S_8–H3SB_8	109.5
H3SA_8–C3S_8–H3SC_8	109.5

H3SB_8–C3S_8–H3SC_8	109.5
O1S_8–C4S_8–H4SA_8	108.7
O1S_8–C4S_8–H4SB_8	109.2
O1S_8–C4S_8–C5S_8	112.6(7)
H4SA_8–C4S_8–H4SB_8	107.8
C5S_8–C4S_8–H4SA_8	109.1
C5S_8–C4S_8–H4SB_8	109.3
C4S_8–C5S_8–H5SA_8	109.1
C4S_8–C5S_8–H5SB_8	109.8
C4S_8–C5S_8–H5SC_8	109.6
H5SA_8–C5S_8–H5SB_8	109.5
H5SA_8–C5S_8–H5SC_8	109.5
H5SB_8–C5S_8–H5SC_8	109.5
O1_9–P1_9–O3_9	112.2(2)
O1_9–P1_9–O4_9	104.9(2)
O2_9–P1_9–O1_9	118.6(2)
O2_9–P1_9–O3_9	105.9(2)
O2_9–P1_9–O4_9	110.9(2)
O3_9–P1_9–O4_9	103.4(2)
P1_9–O1_9–Rh4	113.6(3)
P1_9–O2_9–Rh3	116.8(3)
C3_9–O3_9–P1_9	116.0(3)
C24_9–O4_9–P1_9	118.0(3)
C24_9–C1_9–C2_9	118.6(4)
C24_9–C1_9–C28_9	117.9(4)
C28_9–C1_9–C2_9	123.5(4)
C3_9–C2_9–C1_9	117.7(3)
C3_9–C2_9–C7_9	118.5(4)
C7_9–C2_9–C1_9	123.9(4)
O3_9–C3_9–C4_9	115.5(3)
C2_9–C3_9–O3_9	119.8(3)
C2_9–C3_9–C4_9	124.6(4)
C3_9–C4_9–H4_9	121.2
C5_9–C4_9–C3_9	117.6(4)
C5_9–C4_9–H4_9	121.2
C4_9–C5_9–C6_9	120.9(4)
C4_9–C5_9–C8_9	117.1(4)
C6_9–C5_9–C8_9	121.9(4)
C5_9–C6_9–C7_9	118.9(4)
C14_9–C6_9–C5_9	122.5(4)
C14_9–C6_9–C7_9	118.5(4)
C2_9–C7_9–C6_9	119.4(4)
C17_9–C7_9–C2_9	124.5(4)
C17_9–C7_9–C6_9	116.2(4)
C9_9–C8_9–C5_9	119.2(4)
C13_9–C8_9–C5_9	119.9(4)
C13_9–C8_9–C9_9	120.4(3)
C8_9–C9_9–H9_9	120.8
C8_9–C9_9–C10_9	118.6(4)
C10_9–C9_9–H9_9	120.6
C9_9–C10_9–H10_9	121.9
C11_9–C10_9–C9_9	116.2(4)
C11_9–C10_9–H10_9	121.9
C10_9–C11_9–H11_9	115.9
C12_9–C11_9–C10_9	128.3(5)
C12_9–C11_9–H11_9	115.8
C11_9–C12_9–H12_9	122.4
C11_9–C12_9–C13_9	115.4(4)
C13_9–C12_9–H12_9	122.2
C8_9–C13_9–C12_9	121.1(4)
C8_9–C13_9–H13_9	119.4
C12_9–C13_9–H13_9	119.5
C6_9–C14_9–H14_9	119.2
C15_9–C14_9–C6_9	121.4(4)
C15_9–C14_9–H14_9	119.4
C14_9–C15_9–C16_9	119.7(4)
C14_9–C15_9–C18_9	120.5(4)
C16_9–C15_9–C18_9	119.7(4)
C15_9–C16_9–H16_9	120.4
C17_9–C16_9–C15_9	119.5(4)
C17_9–C16_9–H16_9	120.1
C7_9–C17_9–H17_9	117.6
C16_9–C17_9–C7_9	124.7(5)
C16_9–C17_9–H17_9	117.7
C19_9–C18_9–C15_9	121.8(4)
C19_9–C18_9–C23_9	118.2(2)
C23_9–C18_9–C15_9	119.5(4)
C18_9–C19_9–H19_9	119.4
C20_9–C19_9–C18_9	121.1(3)
C20_9–C19_9–H19_9	119.5
C19_9–C20_9–H20_9	120.8
C21_9–C20_9–C19_9	118.4(3)
C21_9–C20_9–H20_9	120.8
C20_9–C21_9–H21_9	118.5
C20_9–C21_9–C22_9	122.9(4)
C22_9–C21_9–H21_9	118.6
C21_9–C22_9–H22_9	120.7
C21_9–C22_9–C23_9	118.5(3)
C23_9–C22_9–H22_9	120.8
C18_9–C23_9–H23_9	119.6
C22_9–C23_9–C18_9	121.0(3)
C22_9–C23_9–H23_9	119.5
O4_9–C24_9–C25_9	115.6(3)
C1_9–C24_9–O4_9	120.1(3)
C1_9–C24_9–C25_9	124.1(4)
C24_9–C25_9–H25_9	120.5
C26_9–C25_9–C24_9	119.1(4)
C26_9–C25_9–H25_9	120.4
C25_9–C26_9–C27_9	120.9(4)
C25_9–C26_9–C29_9	117.4(4)
C27_9–C26_9–C29_9	121.6(4)
C26_9–C27_9–C28_9	118.6(4)
C26_9–C27_9–C38_9	124.6(4)

C38_9–C27_9–C28_9	116.7(4)
C1_9–C28_9–C27_9	119.3(4)
C35_9–C28_9–C1_9	123.7(4)
C35_9–C28_9–C27_9	116.7(4)
C30_9–C29_9–C26_9	122.0(4)
C30_9–C29_9–C34_9	118.7(2)
C34_9–C29_9–C26_9	119.1(4)
C29_9–C30_9–H30_9	119.5
C29_9–C30_9–C31_9	121.0(2)
C31_9–C30_9–H30_9	119.5
C30_9–C31_9–H31_9	120.2
C32_9–C31_9–C30_9	119.6(2)
C32_9–C31_9–H31_9	120.2
C31_9–C32_9–H32_9	120.0
C31_9–C32_9–C33_9	120.1(2)
C33_9–C32_9–H32_9	119.9
C32_9–C33_9–H33_9	120.3
C32_9–C33_9–C34_9	119.5(2)
C34_9–C33_9–H33_9	120.2
C29_9–C34_9–H34_9	119.4
C33_9–C34_9–C29_9	121.1(2)
C33_9–C34_9–H34_9	119.5
C28_9–C35_9–H35_9	118.3
C36_9–C35_9–C28_9	123.1(4)
C36_9–C35_9–H35_9	118.6
C35_9–C36_9–H36_9	119.6
C35_9–C36_9–C37_9	120.3(4)
C37_9–C36_9–H36_9	120.0
C36_9–C37_9–C39_9	121.4(4)
C38_9–C37_9–C36_9	119.3(4)
C38_9–C37_9–C39_9	118.8(4)
C27_9–C38_9–H38_9	118.6
C37_9–C38_9–C27_9	122.7(4)
C37_9–C38_9–H38_9	118.7
C40_9–C39_9–C37_9	120.2(4)
C40_9–C39_9–C44_9	118.6(3)
C44_9–C39_9–C37_9	121.2(4)
C39_9–C40_9–H40_9	119.8
C39_9–C40_9–C41_9	120.1(4)
C41_9–C40_9–H40_9	120.1
C40_9–C41_9–H41_9	121.0
C42_9–C41_9–C40_9	117.7(4)
C42_9–C41_9–H41_9	121.4
C41_9–C42_9–H42_9	117.7
C43_9–C42_9–C41_9	124.3(5)
C43_9–C42_9–H42_9	118.0
C42_9–C43_9–H43_9	120.9
C42_9–C43_9–C44_9	118.1(4)
C44_9–C43_9–H43_9	121.0
C39_9–C44_9–H44_9	119.5
C43_9–C44_9–C39_9	121.2(4)
C43_9–C44_9–H44_9	119.3
O1_10–P1_10–O3_10	111.9(2)
O1_10–P1_10–O4_10	104.5(2)

O2_10–P1_10–O1_10	118.4(3)
O2_10–P1_10–O3_10	105.8(2)
O2_10–P1_10–O4_10	111.4(2)
O3_10–P1_10–O4_10	103.8(2)
P1_10–O1_10–Rh4	115.7(3)
P1_10–O2_10–Rh3	117.4(3)
C3_10–O3_10–P1_10	116.0(3)
C24_10–O4_10–P1_10	119.4(3)
C24_10–C1_10–C2_10	119.5(4)
C24_10–C1_10–C28_10	117.7(4)
C28_10–C1_10–C2_10	122.8(4)
C3_10–C2_10–C1_10	117.3(4)
C3_10–C2_10–C7_10	118.2(4)
C7_10–C2_10–C1_10	124.4(4)
O3_10–C3_10–C4_10	115.6(3)
C2_10–C3_10–O3_10	119.6(3)
C2_10–C3_10–C4_10	124.8(4)
C3_10–C4_10–H4_10	121.1
C5_10–C4_10–C3_10	117.7(4)
C5_10–C4_10–H4_10	121.1
C4_10–C5_10–C6_10	120.6(4)
C4_10–C5_10–C8_10	117.2(4)
C6_10–C5_10–C8_10	122.1(4)
C5_10–C6_10–C7_10	119.1(4)
C14_10–C6_10–C5_10	122.3(4)
C14_10–C6_10–C7_10	118.5(4)
C2_10–C7_10–C6_10	119.2(4)
C17_10–C7_10–C2_10	124.8(4)
C17_10–C7_10–C6_10	115.9(4)
C9_10–C8_10–C5_10	119.3(4)
C13_10–C8_10–C5_10	120.1(4)
C13_10–C8_10–C9_10	120.1(3)
C8_10–C9_10–H9_10	120.7
C10_10–C9_10–C8_10	118.6(4)
C10_10–C9_10–H9_10	120.7
C9_10–C10_10–H10_10	121.8
C11_10–C10_10–C9_10	116.4(4)
C11_10–C10_10–H10_10	121.9
C10_10–C11_10–H11_10	116.0
C12_10–C11_10–C10_10	128.0(5)
C12_10–C11_10–H11_10	116.0
C11_10–C12_10–H12_10	122.4
C11_10–C12_10–C13_10	115.3(4)
C13_10–C12_10–H12_10	122.3

C8_10–C13_10–C12_10	121.2(4)
C8_10–C13_10–H13_10	119.4
C12_10–C13_10–H13_10	119.4
C6_10–C14_10–H14_10	119.1
C15_10–C14_10–C6_10	121.8(4)
C15_10–C14_10–H14_10	119.2
C14_10–C15_10–C16_10	119.2(4)
C14_10–C15_10–C18_10	120.1(4)
C16_10–C15_10–C18_10	120.2(4)
C15_10–C16_10–H16_10	120.3
C17_10–C16_10–C15_10	119.6(4)
C17_10–C16_10–H16_10	120.0
C7_10–C17_10–H17_10	117.4
C16_10–C17_10–C7_10	125.0(5)
C16_10–C17_10–H17_10	117.6
C19_10–C18_10–C15_10	121.9(4)
C19_10–C18_10–C23_10	118.1(2)
C23_10–C18_10–C15_10	119.9(4)
C18_10–C19_10–H19_10	119.4
C20_10–C19_10–C18_10	121.2(3)
C20_10–C19_10–H19_10	119.4
C19_10–C20_10–H20_10	120.8
C21_10–C20_10–C19_10	118.4(3)
C21_10–C20_10–H20_10	120.8
C20_10–C21_10–H21_10	118.6
C20_10–C21_10–C22_10	122.7(4)
C22_10–C21_10–H21_10	118.6
C21_10–C22_10–H22_10	120.7
C21_10–C22_10–C23_10	118.6(3)
C23_10–C22_10–H22_10	120.7
C18_10–C23_10–H23_10	119.5
C22_10–C23_10–C18_10	120.9(3)
C22_10–C23_10–H23_10	119.6
O4_10–C24_10–C25_10	115.2(4)
C1_10–C24_10–O4_10	120.3(4)
C1_10–C24_10–C25_10	124.3(4)
C24_10–C25_10–H25_10	120.5
C26_10–C25_10–C24_10	119.2(4)
C26_10–C25_10–H25_10	120.3
C25_10–C26_10–C27_10	120.6(4)
C25_10–C26_10–C29_10	117.6(4)
C27_10–C26_10–C29_10	121.7(4)
C26_10–C27_10–C28_10	118.9(4)
C26_10–C27_10–C38_10	124.6(4)
C38_10–C27_10–C28_10	116.5(4)
C1_10–C28_10–C27_10	119.3(4)
C35_10–C28_10–C1_10	123.1(4)
C35_10–C28_10–C27_10	116.8(4)
C30_10–C29_10–C26_10	122.1(4)
C34_10–C29_10–C26_10	119.3(4)
C34_10–C29_10–C30_10	118.6(2)
C29_10–C30_10–H30_10	119.5
C31_10–C30_10–C29_10	121.1(2)
C31_10–C30_10–H30_10	119.5

C30_10–C31_10–H31_10	120.2	C39_10–C40_10–H40_10	120.1
C30_10–C31_10–C32_10	119.6(2)	C39_10–C40_10–C41_10	120.1(4)
C32_10–C31_10–H31_10	120.2	C41_10–C40_10–H40_10	119.8
C31_10–C32_10–H32_10	120.0	C40_10–C41_10–H41_10	121.2
C33_10–C32_10–C31_10	120.1(2)	C42_10–C41_10–C40_10	117.6(4)
C33_10–C32_10–H32_10	119.9	C42_10–C41_10–H41_10	121.2
C32_10–C33_10–H33_10	120.2	C41_10–C42_10–H42_10	118.0
C34_10–C33_10–C32_10	119.6(2)	C43_10–C42_10–C41_10	124.3(5)
C34_10–C33_10–H33_10	120.2	C43_10–C42_10–H42_10	117.7
C29_10–C34_10–H34_10	119.5	C42_10–C43_10–H43_10	121.2
C33_10–C34_10–C29_10	121.1(2)	C42_10–C43_10–C44_10	118.1(4)
C33_10–C34_10–H34_10	119.5	C44_10–C43_10–H43_10	120.8
C28_10–C35_10–H35_10	118.3	C39_10–C44_10–H44_10	119.2
C36_10–C35_10–C28_10	123.2(4)	C43_10–C44_10–C39_10	121.2(4)
C36_10–C35_10–H35_10	118.5	C43_10–C44_10–H44_10	119.5
C35_10–C36_10–H36_10	119.8	C2S_11–O1S_11–Rh2	122.6(9)
		C2S_11–O1S_11–C4S_11	116.8(7)
C35_10–C36_10–C37_10	120.1(4)	C4S_11–O1S_11–Rh2	119.8(7)
C37_10–C36_10–H36_10	120.1	O1S_11–C2S_11–H2SA_11	108.3
C36_10–C37_10–C39_10	121.9(4)	O1S_11–C2S_11–H2SB_11	108.2
C38_10–C37_10–C36_10	119.4(4)	O1S_11–C2S_11–C3S_11	115.9(8)
C38_10–C37_10–C39_10	118.5(4)	H2SA_11–C2S_11–H2SB_11	107.3
C27_10–C38_10–H38_10	118.9	C3S_11–C2S_11–H2SA_11	108.0
C37_10–C38_10–C27_10	122.2(4)	C3S_11–C2S_11–H2SB_11	108.8
C37_10–C38_10–H38_10	118.9	C2S_11–C3S_11–H3SA_11	109.1
C40_10–C39_10–C37_10	120.1(4)	C2S_11–C3S_11–H3SB_11	110.1
C40_10–C39_10–C44_10	118.6(3)	C2S_11–C3S_11–H3SC_11	109.2
C44_10–C39_10–C37_10	120.8(4)	H3SA_11–C3S_11–H3SB_11	109.5

H3SA_11–C3S_11–H3SC_11	109.5
H3SB_11–C3S_11–H3SC_11	109.5
O1S_11–C4S_11–H4SA_11	109.1
O1S_11–C4S_11–H4SB_11	108.6
O1S_11–C4S_11–C5S_11	113.0(7)
H4SA_11–C4S_11–H4SB_11	107.7
C5S_11–C4S_11–H4SA_11	109.8
C5S_11–C4S_11–H4SB_11	108.4
C4S_11–C5S_11–H5SA_11	109.1
C4S_11–C5S_11–H5SB_11	108.9
C4S_11–C5S_11–H5SC_11	110.4
H5SA_11–C5S_11–H5SB_11	109.5
H5SA_11–C5S_11–H5SC_11	109.5
H5SB_11–C5S_11–H5SC_11	109.5
C2S_12–O1S_12–Rh4	124.1(6)
C2S_12–O1S_12–C4S_12	116.7(7)
C4S_12–O1S_12–Rh4	118.7(6)
O1S_12–C2S_12–H2SA_12	108.3
O1S_12–C2S_12–H2SB_12	108.1
O1S_12–C2S_12–C3S_12	116.1(8)
H2SA_12–C2S_12–H2SB_12	107.3
C3S_12–C2S_12–H2SA_12	107.9
C3S_12–C2S_12–H2SB_12	108.8
C2S_12–C3S_12–H3SA_12	109.1
C2S_12–C3S_12–H3SB_12	110.1
C2S_12–C3S_12–H3SC_12	109.2
H3SA_12–C3S_12–H3SB_12	109.5
H3SA_12–C3S_12–H3SC_12	109.5
H3SB_12–C3S_12–H3SC_12	109.5
O1S_12–C4S_12–H4SA_12	109.3
O1S_12–C4S_12–H4SB_12	109.1
O1S_12–C4S_12–C5S_12	112.4(6)
H4SA_12–C4S_12–H4SB_12	107.8
C5S_12–C4S_12–H4SA_12	109.1
C5S_12–C4S_12–H4SB_12	109.1
C4S_12–C5S_12–H5SA_12	109.5
C4S_12–C5S_12–H5SB_12	109.5
C4S_12–C5S_12–H5SC_12	109.5
H5SA_12–C5S_12–H5SB_12	109.5
H5SA_12–C5S_12–H5SC_12	109.5
H5SB_12–C5S_12–H5SC_12	109.5
O1_13–P1_13–O3_13	113.0(2)
O1_13–P1_13–O4_13	105.6(2)
O2_13–P1_13–O1_13	118.5(2)
O2_13–P1_13–O3_13	105.4(2)
O2_13–P1_13–O4_13	110.6(2)
O3_13–P1_13–O4_13	102.70(19)
P1_13–O1_13–Rh2	115.2(3)
P1_13–O2_13–Rh1	118.3(3)
C3_13–O3_13–P1_13	116.5(3)
C24_13–O4_13–P1_13	117.8(3)
C24_13–C1_13–C2_13	118.5(4)
C24_13–C1_13–C28_13	117.8(4)
C28_13–C1_13–C2_13	123.6(4)
C3_13–C2_13–C1_13	118.3(3)
C3_13–C2_13–C7_13	118.4(4)
C7_13–C2_13–C1_13	123.2(3)
O3_13–C3_13–C4_13	114.3(3)
C2_13–C3_13–O3_13	121.0(3)
C2_13–C3_13–C4_13	124.5(4)
C3_13–C4_13–H4_13	121.1
C5_13–C4_13–C3_13	117.9(4)
C5_13–C4_13–H4_13	121.1
C4_13–C5_13–C6_13	120.8(4)
C4_13–C5_13–C8_13	116.9(4)
C6_13–C5_13–C8_13	122.0(4)
C5_13–C6_13–C7_13	119.0(4)
C14_13–C6_13–C5_13	122.4(4)

C14_13–C6_13–C7_13	118.4(4)
C2_13–C7_13–C6_13	119.3(4)
C17_13–C7_13–C2_13	124.7(4)
C17_13–C7_13–C6_13	116.0(4)
C9_13–C8_13–C5_13	119.1(4)
C13_13–C8_13–C5_13	119.8(4)
C13_13–C8_13–C9_13	120.3(3)
C8_13–C9_13–H9_13	120.5
C8_13–C9_13–C10_13	118.6(4)
C10_13–C9_13–H9_13	120.9
C9_13–C10_13–H10_13	121.6
C11_13–C10_13–C9_13	116.3(4)
C11_13–C10_13–H10_13	122.1
C10_13–C11_13–H11_13	115.7
C12_13–C11_13–C10_13	128.2(5)
C12_13–C11_13–H11_13	116.1
C11_13–C12_13–H12_13	122.2
C11_13–C12_13–C13_13	115.3(4)
C13_13–C12_13–H12_13	122.5
C8_13–C13_13–C12_13	121.2(4)
C8_13–C13_13–H13_13	119.6
C12_13–C13_13–H13_13	119.1
C6_13–C14_13–H14_13	119.2
C15_13–C14_13–C6_13	121.6(4)
C15_13–C14_13–H14_13	119.2
C14_13–C15_13–C18_13	120.5(4)
C16_13–C15_13–C14_13	119.6(4)
C16_13–C15_13–C18_13	119.8(4)
C15_13–C16_13–H16_13	120.5
C17_13–C16_13–C15_13	119.3(4)
C17_13–C16_13–H16_13	120.2
C7_13–C17_13–H17_13	117.4

C16_13–C17_13–C7_13	125.1(5)
C16_13–C17_13–H17_13	117.5
C19_13–C18_13–C15_13	121.6(4)
C19_13–C18_13–C23_13	118.2(2)
C23_13–C18_13–C15_13	119.5(4)
C18_13–C19_13–H19_13	119.4
C20_13–C19_13–C18_13	121.1(3)
C20_13–C19_13–H19_13	119.5
C19_13–C20_13–H20_13	120.8
C21_13–C20_13–C19_13	118.3(3)
C21_13–C20_13–H20_13	120.9
C20_13–C21_13–H21_13	118.4
C20_13–C21_13–C22_13	123.1(4)
C22_13–C21_13–H21_13	118.5
C21_13–C22_13–H22_13	120.8
C21_13–C22_13–C23_13	118.3(3)
C23_13–C22_13–H22_13	120.9
C18_13–C23_13–H23_13	119.6
C22_13–C23_13–C18_13	121.0(3)
C22_13–C23_13–H23_13	119.5
O4_13–C24_13–C25_13	115.4(3)
C1_13–C24_13–O4_13	120.1(3)
C1_13–C24_13–C25_13	124.0(4)
C24_13–C25_13–H25_13	120.3
C26_13–C25_13–C24_13	119.4(4)
C26_13–C25_13–H25_13	120.3
C25_13–C26_13–C27_13	120.8(4)

C25_13–C26_13–C29_13	117.4(4)	C36_13–C35_13–C28_13	123.4(4)
C27_13–C26_13–C29_13	121.7(4)	C36_13–C35_13–H35_13	118.4
C26_13–C27_13–C28_13	118.7(4)	C35_13–C36_13–H36_13	119.6
C26_13–C27_13–C38_13	124.3(4)	C35_13–C36_13–C37_13	120.6(4)
C38_13–C27_13–C28_13	117.0(4)	C37_13–C36_13–H36_13	119.8
C1_13–C28_13–C27_13	119.3(3)	C36_13–C37_13–C39_13	121.9(4)
C35_13–C28_13–C1_13	124.2(4)	C38_13–C37_13–C36_13	119.2(4)
C35_13–C28_13–C27_13	116.5(3)	C38_13–C37_13–C39_13	118.8(4)
C30_13–C29_13–C26_13	122.0(4)	C27_13–C38_13–H38_13	118.4
C30_13–C29_13–C34_13	118.6(2)	C37_13–C38_13–C27_13	123.0(4)
C34_13–C29_13–C26_13	118.7(4)	C37_13–C38_13–H38_13	118.6
C29_13–C30_13–H30_13	119.6	C40_13–C39_13–C37_13	120.3(4)
C31_13–C30_13–C29_13	120.9(2)	C40_13–C39_13–C44_13	118.6(3)
C31_13–C30_13–H30_13	119.5	C44_13–C39_13–C37_13	121.1(4)
C30_13–C31_13–H31_13	120.2	C39_13–C40_13–H40_13	120.0
C32_13–C31_13–C30_13	119.7(2)	C39_13–C40_13–C41_13	120.1(4)
C32_13–C31_13–H31_13	120.1	C41_13–C40_13–H40_13	119.9
C31_13–C32_13–H32_13	120.0	C40_13–C41_13–H41_13	121.1
C31_13–C32_13–C33_13	120.1(2)	C42_13–C41_13–C40_13	117.7(4)
C33_13–C32_13–H32_13	119.9	C42_13–C41_13–H41_13	121.2
C32_13–C33_13–H33_13	120.3	C41_13–C42_13–H42_13	117.9
C34_13–C33_13–C32_13	119.4(2)	C43_13–C42_13–C41_13	124.2(5)
C34_13–C33_13–H33_13	120.2	C43_13–C42_13–H42_13	117.9
C29_13–C34_13–H34_13	119.5	C42_13–C43_13–H43_13	121.0
C33_13–C34_13–C29_13	121.0(2)	C42_13–C43_13–C44_13	118.1(4)
C33_13–C34_13–H34_13	119.5	C44_13–C43_13–H43_13	120.8
C28_13–C35_13–H35_13	118.2	C39_13–C44_13–H44_13	119.3

C43_13–C44_13–C39_13	121.3(4)
C43_13–C44_13–H44_13	119.4
O1_14–P1_14–O3_14	113.4(2)
O1_14–P1_14–O4_14	105.3(2)
O2_14–P1_14–O1_14	118.5(2)
O2_14–P1_14–O3_14	104.8(2)
O2_14–P1_14–O4_14	110.7(2)
O3_14–P1_14–O4_14	103.1(2)
P1_14–O1_14–Rh2	114.2(2)
P1_14–O2_14–Rh1	120.0(3)
C3_14–O3_14–P1_14	117.7(3)
C24_14–O4_14–P1_14	117.2(3)
C24_14–C1_14–C2_14	118.6(4)
C24_14–C1_14–C28_14	117.5(4)
C28_14–C1_14–C2_14	123.9(4)
C3_14–C2_14–C1_14	118.2(3)
C3_14–C2_14–C7_14	118.3(4)
C7_14–C2_14–C1_14	123.5(4)
O3_14–C3_14–C4_14	113.9(3)
C2_14–C3_14–O3_14	121.3(4)
C2_14–C3_14–C4_14	124.5(4)
C3_14–C4_14–H4_14	121.1
C5_14–C4_14–C3_14	117.8(4)
C5_14–C4_14–H4_14	121.1
C4_14–C5_14–C6_14	120.7(4)
C4_14–C5_14–C8_14	117.5(4)
C6_14–C5_14–C8_14	121.8(4)
C5_14–C6_14–C7_14	119.1(4)
C14_14–C6_14–C5_14	121.9(4)
C14_14–C6_14–C7_14	118.7(4)
C2_14–C7_14–C6_14	119.2(4)
C17_14–C7_14–C2_14	125.0(4)
C17_14–C7_14–C6_14	115.8(4)
C9_14–C8_14–C5_14	119.4(4)
C13_14–C8_14–C5_14	120.5(4)
C13_14–C8_14–C9_14	120.0(3)
C8_14–C9_14–H9_14	120.5
C8_14–C9_14–C10_14	118.9(4)
C10_14–C9_14–H9_14	120.6
C9_14–C10_14–H10_14	121.7
C11_14–C10_14–C9_14	116.2(4)
C11_14–C10_14–H10_14	122.1
C10_14–C11_14–H11_14	115.8
C12_14–C11_14–C10_14	128.1(5)
C12_14–C11_14–H11_14	116.1
C11_14–C12_14–H12_14	122.3
C11_14–C12_14–C13_14	115.3(4)
C13_14–C12_14–H12_14	122.4
C8_14–C13_14–C12_14	121.4(4)
C8_14–C13_14–H13_14	119.4
C12_14–C13_14–H13_14	119.2
C6_14–C14_14–H14_14	119.2
C15_14–C14_14–C6_14	121.5(4)
C15_14–C14_14–H14_14	119.3
C14_14–C15_14–C16_14	119.5(4)
C14_14–C15_14–C18_14	119.8(4)
C16_14–C15_14–C18_14	120.2(4)
C15_14–C16_14–H16_14	120.5
C17_14–C16_14–C15_14	119.5(5)
C17_14–C16_14–H16_14	120.1
C7_14–C17_14–H17_14	117.4
C16_14–C17_14–C7_14	125.1(5)
C16_14–C17_14–H17_14	117.6
C19_14–C18_14–C15_14	121.4(4)
C19_14–C18_14–C23_14	118.1(2)
C23_14–C18_14–C15_14	119.4(4)
C18_14–C19_14–H19_14	119.4
C20_14–C19_14–C18_14	121.2(3)
C20_14–C19_14–H19_14	119.4
C19_14–C20_14–H20_14	120.8
C21_14–C20_14–C19_14	118.4(3)
C21_14–C20_14–H20_14	120.8

C20_14–C21_14–H21_14	118.6	C29_14–C30_14–H30_14	119.7
C20_14–C21_14–C22_14	122.9(4)	C29_14–C30_14–C31_14	120.8(2)
C22_14–C21_14–H21_14	118.6	C31_14–C30_14–H30_14	119.5
C21_14–C22_14–H22_14	120.7	C30_14–C31_14–H31_14	120.3
C21_14–C22_14–C23_14	118.5(3)	C32_14–C31_14–C30_14	119.7(2)
C23_14–C22_14–H22_14	120.8	C32_14–C31_14–H31_14	120.1
C18_14–C23_14–H23_14	119.6	C31_14–C32_14–H32_14	120.1
C22_14–C23_14–C18_14	120.9(3)	C33_14–C32_14–C31_14	120.1(2)
C22_14–C23_14–H23_14	119.5	C33_14–C32_14–H32_14	119.8
O4_14–C24_14–C25_14	114.8(4)	C32_14–C33_14–H33_14	120.4
C1_14–C24_14–O4_14	120.1(3)	C34_14–C33_14–C32_14	119.5(2)
C1_14–C24_14–C25_14	124.7(4)	C34_14–C33_14–H33_14	120.1
C24_14–C25_14–H25_14	120.6	C29_14–C34_14–H34_14	119.4
C26_14–C25_14–C24_14	118.9(4)	C33_14–C34_14–C29_14	121.1(2)
C26_14–C25_14–H25_14	120.5	C33_14–C34_14–H34_14	119.6
C25_14–C26_14–C27_14	120.7(4)	C28_14–C35_14–H35_14	118.3
C25_14–C26_14–C29_14	117.4(4)	C36_14–C35_14–C28_14	123.3(4)
C27_14–C26_14–C29_14	121.6(4)	C36_14–C35_14–H35_14	118.4
C26_14–C27_14–C28_14	118.9(4)	C35_14–C36_14–H36_14	119.7
C26_14–C27_14–C38_14	124.2(4)	C35_14–C36_14–C37_14	120.4(4)
C38_14–C27_14–C28_14	116.9(4)	C37_14–C36_14–H36_14	119.9
C1_14–C28_14–C27_14	119.3(4)	C36_14–C37_14–C39_14	122.0(4)
C35_14–C28_14–C1_14	124.1(4)	C38_14–C37_14–C36_14	119.3(4)
C35_14–C28_14–C27_14	116.6(4)	C38_14–C37_14–C39_14	118.7(4)
C30_14–C29_14–C26_14	122.2(4)	C27_14–C38_14–H38_14	118.4
C30_14–C29_14–C34_14	118.8(2)	C37_14–C38_14–C27_14	123.0(4)
C34_14–C29_14–C26_14	119.0(4)	C37_14–C38_14–H38_14	118.6

C40_14–C39_14–C37_14	120.4(4)
C40_14–C39_14–C44_14	118.5(3)
C44_14–C39_14–C37_14	121.1(4)
C39_14–C40_14–H40_14	119.9
C39_14–C40_14–C41_14	120.2(4)
C41_14–C40_14–H40_14	119.9
C40_14–C41_14–H41_14	121.1
C42_14–C41_14–C40_14	117.6(4)
C42_14–C41_14–H41_14	121.3
C41_14–C42_14–H42_14	117.8
C43_14–C42_14–C41_14	124.2(5)
C43_14–C42_14–H42_14	117.9
C42_14–C43_14–H43_14	121.0
C42_14–C43_14–C44_14	118.1(4)
C44_14–C43_14–H43_14	120.9
C39_14–C44_14–H44_14	119.4
C43_14–C44_14–C39_14	121.3(4)
C43_14–C44_14–H44_14	119.3
O1_4–P1_4–O3_4	112.1(2)
O1_4–P1_4–O4_4	105.2(2)
O2_4–P1_4–O1_4	118.0(2)
O2_4–P1_4–O3_4	105.8(2)
O2_4–P1_4–O4_4	111.8(2)
O4_4–P1_4–O3_4	102.9(2)
P1_4–O1_4–Rh2	115.6(3)
P1_4–O2_4–Rh1	117.6(3)
C3_4–O3_4–P1_4	115.6(3)
C24_4–O4_4–P1_4	119.5(3)
C24_4–C1_4–C2_4	118.6(4)
C24_4–C1_4–C28_4	118.0(4)
C28_4–C1_4–C2_4	123.0(4)
C3_4–C2_4–C1_4	117.5(3)
C3_4–C2_4–C7_4	118.7(4)
C7_4–C2_4–C1_4	123.8(4)
O3_4–C3_4–C4_4	115.5(3)
C2_4–C3_4–O3_4	119.9(3)
C2_4–C3_4–C4_4	124.4(4)
C3_4–C4_4–H4_4	121.2
C5_4–C4_4–C3_4	117.7(4)
C5_4–C4_4–H4_4	121.1
C4_4–C5_4–C6_4	121.0(4)
C4_4–C5_4–C8_4	117.2(4)
C6_4–C5_4–C8_4	121.4(4)
C5_4–C6_4–C7_4	118.9(4)
C14_4–C6_4–C5_4	122.5(4)
C14_4–C6_4–C7_4	118.5(4)
C2_4–C7_4–C6_4	119.2(4)
C17_4–C7_4–C2_4	124.8(4)
C17_4–C7_4–C6_4	116.0(4)
C9_4–C8_4–C5_4	119.2(4)
C13_4–C8_4–C5_4	120.2(4)
C13_4–C8_4–C9_4	120.4(3)
C8_4–C9_4–H9_4	120.7
C8_4–C9_4–C10_4	118.6(4)
C10_4–C9_4–H9_4	120.7
C9_4–C10_4–H10_4	121.8
C11_4–C10_4–C9_4	116.2(4)
C11_4–C10_4–H10_4	122.0
C10_4–C11_4–H11_4	115.8
C12_4–C11_4–C10_4	128.3(5)
C12_4–C11_4–H11_4	115.9
C11_4–C12_4–H12_4	122.3
C11_4–C12_4–C13_4	115.4(4)
C13_4–C12_4–H12_4	122.3
C8_4–C13_4–C12_4	121.0(4)
C8_4–C13_4–H13_4	119.5
C12_4–C13_4–H13_4	119.4
C6_4–C14_4–H14_4	119.2
C15_4–C14_4–C6_4	121.5(4)
C15_4–C14_4–H14_4	119.3
C14_4–C15_4–C18_4	120.6(4)
C16_4–C15_4–C14_4	119.6(4)
C16_4–C15_4–C18_4	119.8(4)
C15_4–C16_4–H16_4	120.4
C17_4–C16_4–C15_4	119.5(4)
C17_4–C16_4–H16_4	120.1
C7_4–C17_4–H17_4	117.5
C16_4–C17_4–C7_4	124.9(5)
C16_4–C17_4–H17_4	117.6
C19_4–C18_4–C15_4	122.0(4)
C19_4–C18_4–C23_4	118.2(2)
C23_4–C18_4–C15_4	119.7(4)
C18_4–C19_4–H19_4	119.5
C20_4–C19_4–C18_4	121.0(3)
C20_4–C19_4–H19_4	119.5
C19_4–C20_4–H20_4	120.7
C21_4–C20_4–C19_4	118.6(3)
C21_4–C20_4–H20_4	120.7
C20_4–C21_4–H21_4	118.6
C20_4–C21_4–C22_4	122.8(4)

C22_4–C21_4–H21_4	118.6
C21_4–C22_4–H22_4	120.8
C21_4–C22_4–C23_4	118.4(3)
C23_4–C22_4–H22_4	120.8
C18_4–C23_4–H23_4	119.4
C22_4–C23_4–C18_4	121.1(3)
C22_4–C23_4–H23_4	119.5
O4_4–C24_4–C25_4	115.4(4)
C1_4–C24_4–O4_4	120.7(4)
C1_4–C24_4–C25_4	123.9(4)
C24_4–C25_4–H25_4	120.4
C26_4–C25_4–C24_4	119.3(4)
C26_4–C25_4–H25_4	120.3
C25_4–C26_4–C27_4	120.8(4)
C25_4–C26_4–C29_4	117.5(4)
C27_4–C26_4–C29_4	121.2(4)
C26_4–C27_4–C28_4	118.5(4)
C26_4–C27_4–C38_4	124.1(4)
C38_4–C27_4–C28_4	117.0(4)
C1_4–C28_4–C27_4	119.3(4)
C35_4–C28_4–C1_4	123.0(4)
C35_4–C28_4–C27_4	116.6(4)
C30_4–C29_4–C26_4	122.1(4)
C30_4–C29_4–C34_4	118.7(2)
C34_4–C29_4–C26_4	119.1(4)
C29_4–C30_4–H30_4	119.4
C29_4–C30_4–C31_4	121.0(2)
C31_4–C30_4–H30_4	119.6
C30_4–C31_4–H31_4	120.1
C32_4–C31_4–C30_4	119.6(2)
C32_4–C31_4–H31_4	120.3
C31_4–C32_4–H32_4	119.9
C31_4–C32_4–C33_4	120.1(2)
C33_4–C32_4–H32_4	120.0
C32_4–C33_4–H33_4	120.1
C34_4–C33_4–C32_4	119.6(2)
C34_4–C33_4–H33_4	120.3
C29_4–C34_4–H34_4	119.6
C33_4–C34_4–C29_4	121.0(2)
C33_4–C34_4–H34_4	119.4
C28_4–C35_4–H35_4	118.3
C36_4–C35_4–C28_4	123.3(4)
C36_4–C35_4–H35_4	118.4
C35_4–C36_4–H36_4	119.7
C35_4–C36_4–C37_4	120.4(4)
C37_4–C36_4–H36_4	120.0
C36_4–C37_4–C39_4	121.7(4)
C38_4–C37_4–C36_4	119.1(4)
C38_4–C37_4–C39_4	118.8(4)
C27_4–C38_4–H38_4	118.4
C37_4–C38_4–C27_4	123.0(4)
C37_4–C38_4–H38_4	118.6
C40_4–C39_4–C37_4	120.3(4)
C40_4–C39_4–C44_4	118.6(3)
C44_4–C39_4–C37_4	121.1(4)
C39_4–C40_4–H40_4	119.9
C39_4–C40_4–C41_4	120.2(4)
C41_4–C40_4–H40_4	119.9
C40_4–C41_4–H41_4	121.1
C42_4–C41_4–C40_4	117.6(4)
C42_4–C41_4–H41_4	121.3
C41_4–C42_4–H42_4	117.9
C43_4–C42_4–C41_4	124.3(5)
C43_4–C42_4–H42_4	117.8
C42_4–C43_4–H43_4	121.1
C42_4–C43_4–C44_4	118.1(4)
C44_4–C43_4–H43_4	120.9
C39_4–C44_4–H44_4	119.3
C43_4–C44_4–C39_4	121.3(4)
C43_4–C44_4–H44_4	119.4

Symmetry transformations used to generate equivalent atoms:
#1: -1-X, +Y, 1.5-Z;

Bibliography

[1] Crysalispro, *1.171.41.122*, **2021**, Rigaku OD.
[2] Crysalispro, *1.171.41.122*, **2021**, Rigaku OD.
[3] G. M. Sheldrick, *Acta Cryst.* **2015**, *A71*, 3–8, doi:10.1107/S2053273314026370.
[4] G. M. Sheldrick, *Acta Cryst.* **2015**, *C71*, 3–8, doi:10.1107/S2053229614024218.
[5] O. V. Dolomanov, L. J. Bourhis, R. J. Gildea, J. A. K. Howard, H. Puschmann, *J. Appl. Cryst.* **2009**, *42*, 339-341, doi:10.1107/S0021889808042726.
[6] C. R. Groom, I. J. Bruno, M. P. Lightfoot, S. C. Ward, *Acta Cryst.* **2016**, *B72*, 171–179, doi:10.1107/S2052520616003954.
[7] D. Kratzert, *FinalCif*, *V137*, https://dkratzert.de/finalcif.html.

10.2 X-Ray Crystallographic Data for *S*-18

R_1=5.96%

Crystal Data and Experimental

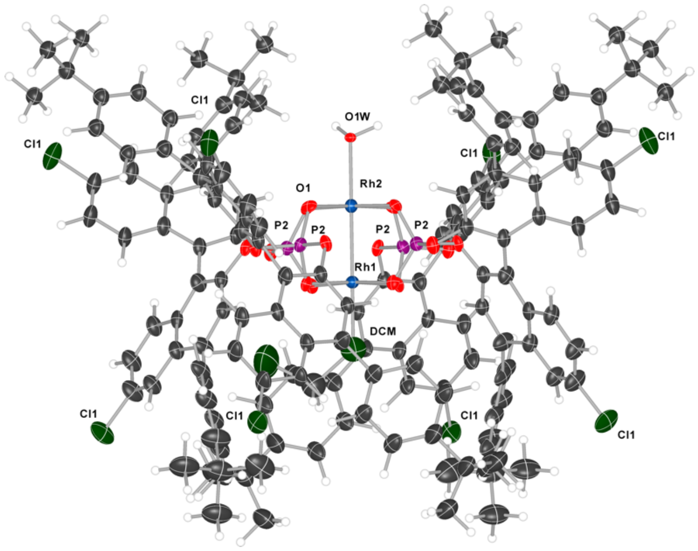

**Compound**	*S*-**18** (ZC-01-57)
**CCDC number**	**2349403**
Formula	$C_{151}H_{134}Cl_{30}O_{17}P_4Rh_2$
$D_{calc.}$/ g cm^{-3}	1.135
μ/mm^{-1}	0.572
Formula Weight	3894.21
Colour	green
Shape	needle-shaped
Size/mm^3	0.28×0.11×0.04
T/K	100.00(10)
Crystal System	tetragonal
Flack Parameter	?
Hooft Parameter	-0.16(2)
Space Group	*P*4
a/Å	25.7605(6)
b/Å	25.7605(6)
c/Å	17.1643(6)
α/°	90
β/°	90
γ/°	90
V/Å^3	11390.3(7)
Z	2
Z'	0.5
Wavelength/Å	0.71073
Radiation type	Mo K_α
Θ_{min}/°	2.501
Θ_{max}/°	24.713
Measured Refl's.	75022
Indep't Refl's	19419
Refl's I≥2 σ(I)	10283
R_{int}	0.1216
Parameters	944
Restraints	1556
Largest Peak	0.382
Deepest Hole	-0.248
GooF	0.887
wR_2 (all data)	0.1424
wR_2	0.1226
R_1 (all data)	0.1215
R_1	0.0596

Experimental. Single green needle-shaped crystals of *S*-**18** (ZC-01-57) were prepared by slow evaporation of a sample solution in dichloromethane and hexane. A suitable crystal with dimensions 0.28 × 0.11 × 0.04 mm^3 was selected and mounted on a loop with paratone on a XtaLAB Synergy, Dualflex, HyPix diffractometer. The crystal was kept at a steady *T* = 100.0(1) K during data collection. The structure was solved with the ShelXT (Sheldrick, 2015) solution program using dual methods and by using Olex2 1.5-alpha (Dolomanov et al., 2009) as the graphical interface. The model was refined with ShelXL 2018/3 (Sheldrick, 2015) using full matrix least squares minimisation on $\boldsymbol{F}^2$.

Crystal Data. $C_{150.85}H_{134.51}Cl_{30}O_{17}P_4Rh_2$, M_r = 3894.21, tetragonal, *P*4 (No. 75), a = 25.7605(6) Å, b = 25.7605(6) Å, c = 17.1643(6) Å, $\alpha = \beta = \gamma = 90°$, V = 11390.3(7) Å^3, T = 100.0(1) K, Z = 2, Z' = 0.5, μ(Mo K_α) = 0.572, 75022 reflections measured, 19419 unique (R_{int} = 0.1216) which were used in all calculations. The final wR_2 was 0.1424 (all data) and R_1 was 0.0596 (I≥2 σ(I)).

Structure Quality Indicators

**Reflections:**	d min (MoKα) 2Θ=49.4° 0.85	I/σ(I) 7.2	Rint m=3.86 12.16%	Full 49.4° 99.7
**Refinement:**	Shift 0.009	Max Peak 0.4	Min Peak -0.2	GooF 0.887

A green needle-shaped-shaped crystal with dimensions 0.28 × 0.11 × 0.04 mm^3 was mounted on a loop with paratone. Data were collected using a XtaLAB Synergy, Dualflex, HyPix diffractometer equipped with an Oxford Cryosystems low-temperature device operating at T = 100.00(10) K.

Data were measured using ω scans with Mo K_α radiation. The diffraction pattern was indexed and the total number of runs and images was based on the strategy calculation from the program CrysAlisPro system (CCD 43.92a 64-bit (release 05-10-2023)). The maximum resolution that was achieved was Θ = 24.713° (0.85 Å).

The unit cell was refined using CrysAlisPro 1.171.43.103a (Rigaku OD, 2023) on 13299 reflections, 18% of the observed reflections.

Data reduction, scaling and absorption corrections were performed using CrysAlisPro 1.171.43.103a (Rigaku OD, 2023). The final completeness is 99.80 % out to 24.713° in Θ. An analytical, numeric absorption correction using a multifaceted crystal model based on expressions derived by R.C. Clark & J.S. Reid. (Clark, R. C. & Reid, J. S. (1995). Acta Cryst. A51, 887-897) was performed using CrysAlisPro 1.171.43.103a (Rigaku Oxford Diffraction, 2023). An empirical absorption correction using spherical harmonics, implemented in SCALE3 ABSPACK scaling algorithm was also applied. The absorption coefficient μ of this material is 0.572 mm^{-1} at this wavelength (λ = 0.71073Å) and the minimum and maximum transmissions are 0.910 and 0.984.

The structure was solved and the space group $P4$ (# 75) determined by the ShelXT (Sheldrick, 2015) structure solution program using using dual methods and refined by full matrix least squares minimisation on $\boldsymbol{F}^2$ using version 2018/3 of ShelXL 2018/3 (Sheldrick, 2015). All non-hydrogen atoms were refined anisotropically. Hydrogen atom positions were calculated geometrically and refined using the riding model. Most hydrogen atom positions were calculated geometrically and refined using the riding model, but some hydrogen atoms were refined freely.

_refine_special_details: Refined as a 4-component inversion twin.

The value of Z' is 0.5. This means that only half of the formula unit is present in the asymmetric unit, with the other half consisting of symmetry equivalent atoms. However, the asymmetric unit in the crystal structure consists of 2 dirhodium complexes with the dirhodium atoms on special positions. The symmetry independent atom consists of half the full molecule and with the other half consisting of symmetry equivalent atoms (i.e. 2[{Rh(L)4}2] complexes), The moiety formula is C162 H142 Cl9.5 O17 P4 Rh2, 10[C1], 10[H2], 10[Cl2].

Determination of absolute structure using Bayesian statistics on Bijvoet differences using the Olex2 results in -0.16(2). The chiral atoms in this structure are: P1(R), P2(R), C5(R), C6(S).

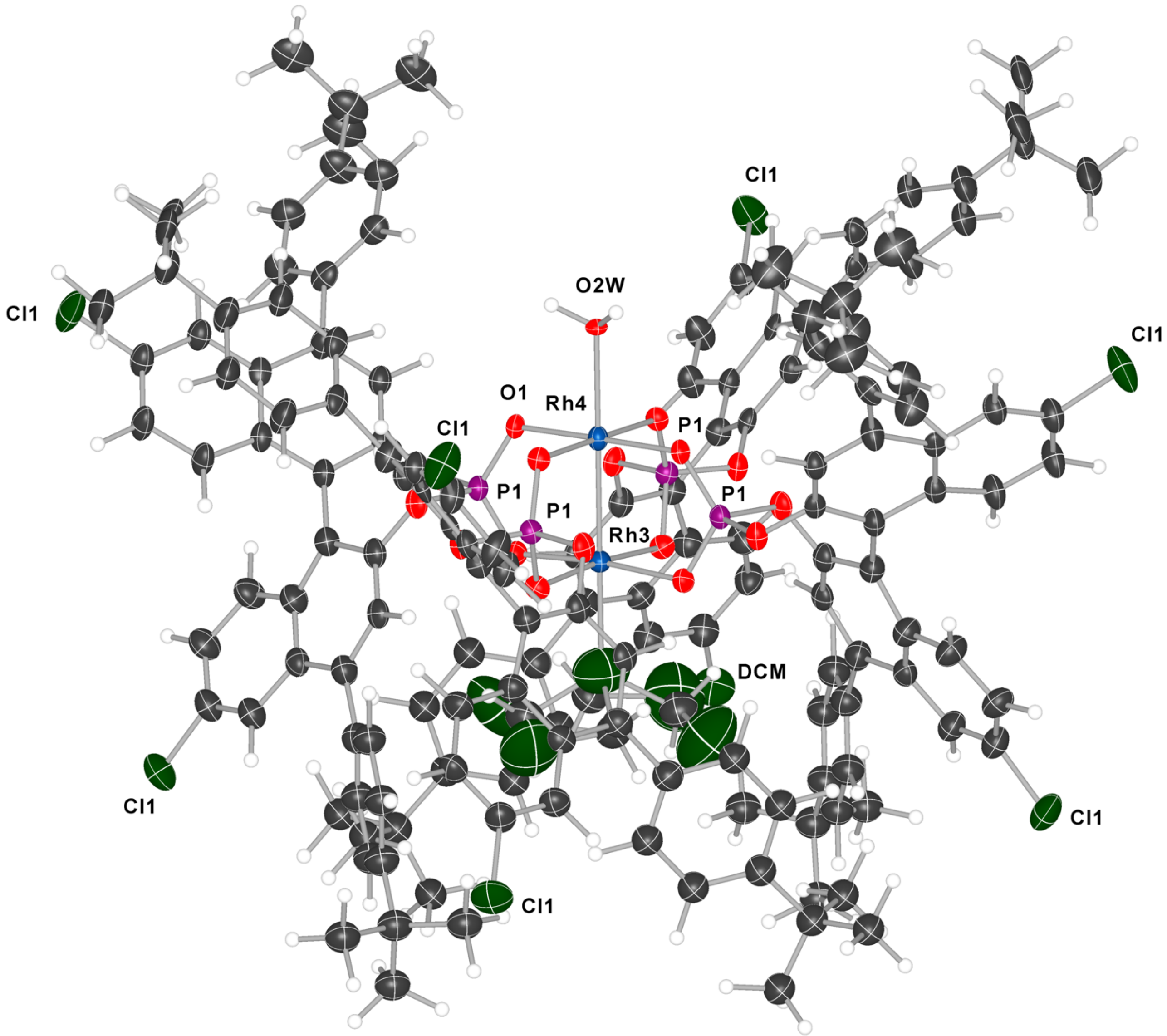

Figure 10.2.2 The molecular structure of one of the two dirhodium complexes in the crystal structure showing the coordination around the rhodium atoms with the 4-fold symmetry arrangement of the ligands about the dirhodium center. There is 4-fold disordered DCM (CH_2Cl_2) coordinated to an axial position and a water molecule at the other axial position

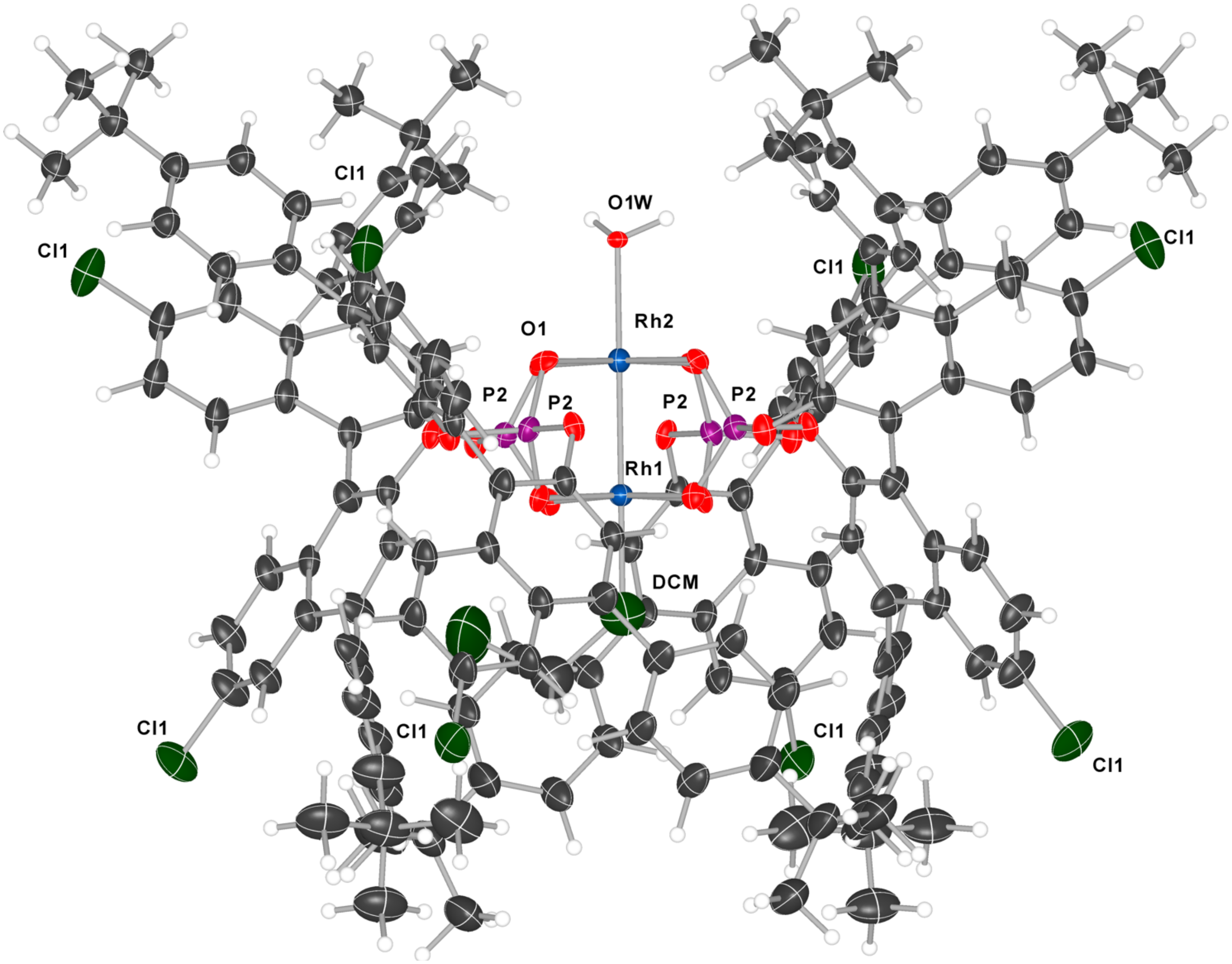

Figure 10.2.3 The molecular structure of the second of the two dirhodium complexes in the crystal structure showing the coordination around the rhodium atoms with the 4-fold symmetry arrangement of the ligands about the dirhodium center.

Data Plots: Diffraction Data

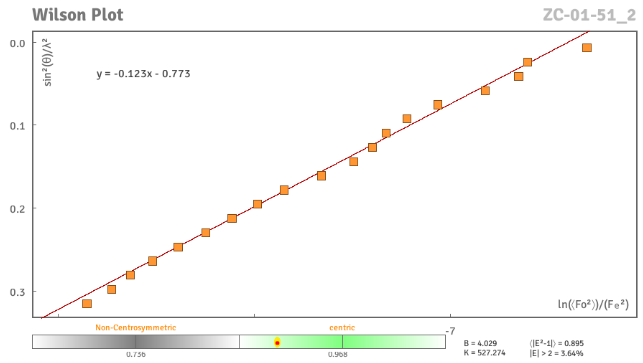

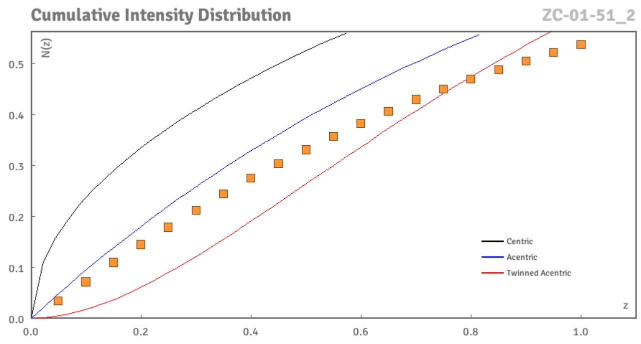

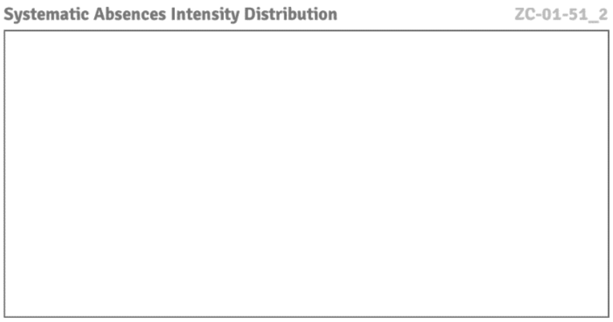

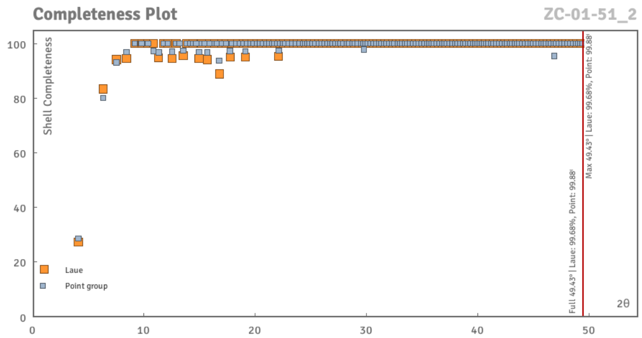

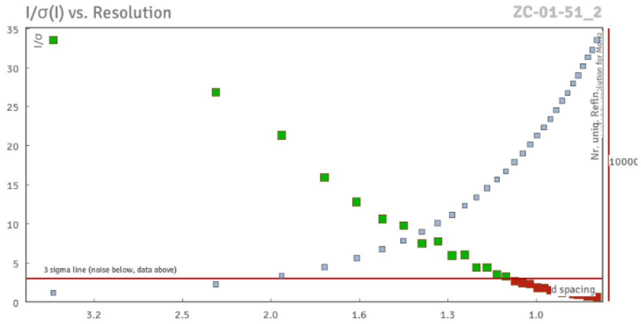

Data Plots: Refinement and Data

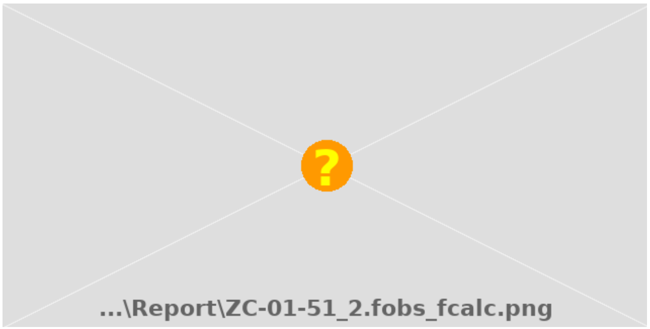

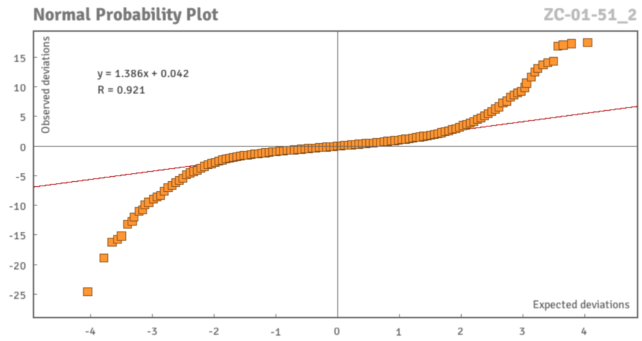

Reflection Statistics

Total reflections (after filtering)	75022	Unique reflections	19419
Completeness	0.999	Mean I/σ	6.47
hkl_{max} collected	(30, 30, 20)	hkl_{min} collected	(-27, -22, -20)
hkl_{max} used	(21, 30, 20)	hkl_{min} used	(-20, 0, -20)
Lim d_{max} collected	20.0	Lim d_{min} collected	0.85
d_{max} used	8.14	d_{min} used	0.85
Friedel pairs	11663	Friedel pairs merged	0
Inconsistent equivalents	16	R_{int}	0.1216
R_{sigma}	0.1384	Intensity transformed	0
Omitted reflections	75	Omitted by user (OMIT hkl)	0
Multiplicity	(24519, 13116, 4824, 1941, 422)	Maximum multiplicity	16
Removed systematic absences	0	Filtered off (Shel/OMIT)	0

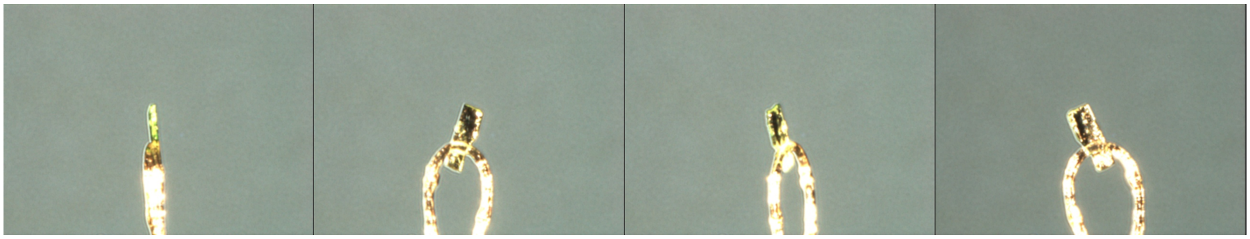

Table 10.2.1: Fractional Atomic Coordinates (×10[4]) and Equivalent Isotropic Displacement Parameters (Å^2×10^3) for *S*-**18** (ZC-01-57). U_{eq} is defined as 1/3 of the trace of the orthogonalised U_{ij}.

Atom	x	y	z	U_{eq}
Rh1	10000	10000	-1098.8(9)	47.5(4)
Rh2	10000	10000	373.8(9)	48.7(4)
P2	9599.6(7)	9011.2(7)	-399.4(15)	51.7(5)
O3	9580.0(17)	9328.2(19)	-1140(3)	49.9(13)
O4	9639.7(19)	9298.3(17)	353(3)	51.6(14)
O1W	10000	10000	1694(2)	33(2)
Rh3	5000	5000	1791.9(9)	50.0(4)
Rh4	5000	5000	3257.1(9)	48.3(4)
P1	6068.5(7)	5100.9(7)	2492.5(14)	52.4(5)
O1	5785.2(16)	4987.9(17)	3247(2)	49.5(12)
O2	5807.3(18)	4971.0(19)	1724(2)	58.0(13)
O2W	5000	5000	4581.0(18)	42(2)
C11_2	6288(4)	6528(4)	-78(5)	97.1(13)
C12_2	6210(4)	6263(4)	-764(6)	100.2(13)
C13_2	5895(4)	6473(3)	-1342(5)	100.2(13)
C14_2	5656(3)	6952(3)	-1233(4)	105.1(15)
C15_2	5740(4)	7213(4)	-536(5)	100.2(13)
C16_2	6056(4)	7001(4)	40(5)	100.2(13)
C17_2	5320(3)	7169(4)	-1875(4)	107.4(17)
C18_2	5658(5)	7453(6)	-2472(7)	107(2)
C19_2	5021(5)	6737(5)	-2292(8)	108.0(17)
C20_2	4928(5)	7556(6)	-1538(7)	108.0(17)
C11_3	7874(2)	4545.3(18)	4506(3)	72.4(17)
C12_3	7553(3)	4322(2)	5051(3)	79(2)
C13_3	7744(2)	3942(2)	5551(3)	95.6(19)
C14_3	8258(2)	3784.5(17)	5502(3)	113(3)
C15_3	8577(2)	4015(2)	4948(3)	95.6(19)
C16_3	8384(3)	4395(2)	4449(3)	107(3)
C17_3	8451(2)	3366(2)	6048(3)	119(2)
C18_3	8799(3)	3608(3)	6675(4)	123(3)
C19_3	7999(3)	3088(3)	6446(5)	134(3)
C20_3	8770(3)	2965(3)	5595(5)	131(3)
C11_4	8236(5)	7611(7)	1509(7)	80.4(18)
C12_4	7794(5)	7330(6)	1353(6)	80.8(19)
C13_4	7418(5)	7265(6)	1928(6)	82.0(17)
C14_4	7485(3)	7484(4)	2660(5)	81.4(17)
C15_4	7906(4)	7813(5)	2775(6)	82(3)
C16_4	8298(4)	7849(6)	2219(7)	78(4)
C17_4	7068(3)	7408(3)	3268(4)	82.7(14)
C18_4	6741(4)	7901(4)	3339(7)	83.2(17)
C19_4	6712(4)	6955(4)	3050(6)	82.2(17)
C20_4	7315(4)	7293(5)	4062(4)	85(2)
Cl1S_6	10000	10000	-2395(11)	176(6)
C1S_6	10502(6)	10124(15)	-3081(9)	168(12)

Atom	x	y	z	U_{eq}
Cl2S_6	11111(5)	10200(20)	-2607(17)	530(40)
Cl1S_7	5000	5000	491(14)	273(9)
C1S_7	4585(7)	5435(7)	-16(14)	134(9)
Cl2S_7	4998(7)	5983(6)	-122(12)	376(9)
C11_8	10791(2)	8228.4(19)	-2952(3)	90(2)
C12_8	10625.2(18)	8301.7(18)	-3716(2)	98.7(18)
C13_8	10966(2)	8471(2)	-4292(2)	104(2)
C14_8	11484(2)	8573(2)	-4122(3)	150(3)
C15_8	11651.9(18)	8500(3)	-3359(3)	111(3)
C16_8	11310.6(19)	8331(2)	-2783(2)	98.7(18)
C17_8	11868(2)	8727(3)	-4758(3)	150(3)
C18_8	12379(3)	8408(4)	-4687(5)	167(3)
C19_8	11639(3)	8619(5)	-5579(3)	169(4)
C20_8	12005(4)	9314(3)	-4697(6)	185(4)
Cl1_9	9721.1(13)	6529.8(12)	-3899.3(18)	136.2(13)
O1_9	10064.8(13)	8594.5(13)	-393(2)	50.9(10)
C1_9	10085(2)	8262(2)	-1001(2)	60.9(17)
C2_9	9731(3)	7853(2)	-1038(3)	78(2)
C3_9	9709(2)	7549.4(19)	-1739(2)	61.4(16)
C4_9	10075(2)	7644(2)	-2345(3)	73(2)
C5_9	10410(3)	8104(2)	-2298(3)	100(2)
C6_9	10407(2)	8378(2)	-1610(3)	61.5(17)
C8_9	9351(2)	7138(2)	-1841(3)	82(2)
C9_9	9345(3)	6825(3)	-2504(3)	102(3)
C10_9	9688(3)	6934(3)	-3103(3)	108(3)
C11_9	10069(3)	7311(2)	-3002(3)	88(3)
Cl1_10	9023.1(12)	6336.1(11)	4422(2)	140.8(14)
O1_10	6616.0(13)	4813.9(14)	2455(3)	58.0(12)
C1_10	7031.9(17)	5008.6(18)	2834(3)	52.2(15)
C2_10	7278(2)	5450.8(19)	2547(3)	66.0(17)
C3_10	7719.0(19)	5651.6(16)	2961(3)	59.6(16)
C4_10	7915.7(17)	5373.9(15)	3616(3)	52.3(13)
C5_10	7668(2)	4885(2)	3846(4)	81.2(19)
C6_10	7234(2)	4730.0(19)	3441(3)	63.6(18)
C8_10	7923(2)	6153.7(18)	2814(4)	81(2)
C9_10	8345(3)	6355(2)	3226(4)	94(3)
C10_10	8532.9(13)	6082.1(14)	3859(2)	80(2)
C11_10	8327(2)	5598.8(17)	4046(3)	72(2)
Cl1_11	8306.1(12)	6246.0(14)	-952.3(18)	137.9(12)
O1_11	6225.6(15)	5705.6(13)	2469(2)	62.9(11)
C1_11	6523.8(15)	5853(3)	1864(3)	68.7(17)
C2_11	7053.5(15)	5738(3)	1868(2)	74(2)
C3_11	7333.7(15)	5793(3)	1148(3)	86(2)
C4_11	7106.9(14)	6086(3)	530(2)	73.7(18)
C5_11	6558.8(14)	6236(3)	582(3)	84.8(14)
C6_11	6304.0(15)	6133(2)	1273(2)	65.0(16)
C8_11	7859.0(17)	5638(3)	1069(3)	99(3)
C9_11	8173.1(15)	5806(3)	457(3)	94(3)
C10_11	7948.4(14)	6086.8(16)	-142(2)	93(3)
C11_11	7432.1(16)	6242(3)	-93(3)	90(2)
Cl1_12	9340.6(11)	5796.7(10)	1484(2)	132.4(12)
O1_12	9102.0(14)	8644.8(12)	-454(3)	61.5(12)
C1_12	9060.7(15)	8175.4(10)	-112(2)	67.6(19)
C2_12	9397.0(15)	7777.2(10)	-332(2)	62.6(16)
C3_12	9375.8(10)	7294.4(9)	83.3(16)	69.8(19)
C4_12	9015.6(10)	7234.4(9)	707.3(17)	61.8(16)

Atom	x	y	z	U_{eq}
C5_12	8670.8(14)	7666.4(12)	909(2)	74.0(16)
C6_12	8719.3(14)	8119.0(11)	493(2)	68.2(19)
C8_12	9683.8(15)	6859.3(11)	-124(2)	83(2)
C9_12	9674.5(17)	6395.3(11)	296(3)	92(3)
C10_12	9347.7(17)	6353.2(11)	932(3)	93(3)
C11_12	9022.3(16)	6762.9(12)	1133(2)	85(2)
C11_1	6264(2)	6496(3)	-88(3)	97.1(13)
C12_1	5894(3)	6866(3)	79(4)	100.2(13)
C13_1	5620(3)	7106(3)	-521(4)	100.2(13)
C14_1	5716(3)	6975(3)	-1292(4)	105.1(15)
C15_1	6091(3)	6600(3)	-1449(3)	100.2(13)
C16_1	6363(3)	6362(3)	-848(3)	100.2(13)
C17_1	5413(3)	7244(3)	-1927(4)	107.4(17)
C18_1	4833(3)	7133(6)	-1830(8)	108.0(17)
C19_1	5497(6)	7832(3)	-1900(8)	108.0(17)
C20_1	5584(6)	7044(6)	-2729(4)	107(2)
C11_5	8219(4)	7544(5)	1455(7)	80.4(18)
C12_5	8195(4)	7901(5)	2051(7)	80.8(19)
C13_5	7776(3)	7897(5)	2565(6)	82(3)
C14_5	7381(3)	7535(4)	2481(6)	81.8(19)
C15_5	7412(4)	7179(5)	1876(7)	82.0(17)
C16_5	7831(5)	7183(5)	1363(7)	80.8(19)
C17_5	6913(3)	7570(4)	3010(5)	82.7(14)
C18_5	6817(6)	7044(4)	3399(8)	82.2(17)
C19_5	6994(5)	7979(5)	3644(7)	83.2(17)
C20_5	6430(4)	7715(6)	2536(8)	85(2)

Table 10.2.2: Anisotropic Displacement Parameters (×10[4]) for *S*-**18** (ZC-01-57). The anisotropic displacement factor exponent takes the form: $-2\pi^2[h^2a^{*2} \times U_{11} + ... + 2hka^* \times b^* \times U_{12}]$

Atom	U_{11}	U_{22}	U_{33}	U_{23}	U_{13}	U_{12}
Rh1	49.4(5)	49.4(5)	43.7(8)	0	0	0
Rh2	46.4(5)	46.4(5)	53.3(8)	0	0	0
P2	43.4(12)	52.4(13)	59.2(13)	2.3(13)	8.2(12)	2.9(10)
O3	32(3)	52(3)	65(3)	-1(3)	12(3)	5(2)
O4	80(4)	33(3)	42(3)	15(2)	9(3)	-11(3)
O1W	37(3)	37(3)	25(4)	0	0	0
Rh3	50.3(5)	50.3(5)	49.5(8)	0	0	0
Rh4	41.0(5)	41.0(5)	62.8(9)	0	0	0
P1	49.4(12)	46.7(13)	60.9(11)	-2.5(11)	0.5(10)	-6.0(10)
O1	38(3)	50(3)	60.0(15)	-0.5(18)	-4.2(15)	-6(2)
O2	48(3)	65(4)	60.6(14)	-5(2)	0.8(16)	-6(3)
O2W	50(4)	50(4)	26(5)	0	0	0
C11_2	97.3(16)	97.5(15)	96.4(14)	2.0(9)	-0.6(9)	0.4(9)
C12_2	103(2)	101.0(17)	96.3(14)	1.0(11)	-2.4(11)	5.4(15)
C13_2	103(2)	101.0(17)	96.3(14)	1.0(11)	-2.4(11)	5.4(15)
C14_2	113(3)	106(2)	96.3(14)	-1.0(13)	-4.8(14)	13(2)
C15_2	103(2)	101.0(17)	96.3(14)	1.0(11)	-2.4(11)	5.4(15)
C16_2	103(2)	101.0(17)	96.3(14)	1.0(11)	-2.4(11)	5.4(15)
C17_2	113(3)	111(3)	98.6(17)	4(2)	-4(2)	13(2)
C18_2	112(4)	111(4)	97.6(17)	5(3)	-5(3)	14(3)
C19_2	112(3)	111(3)	101(3)	4(2)	-4(2)	13(2)
C20_2	112(3)	111(3)	101(3)	4(2)	-4(2)	13(2)

Atom	U_{11}	U_{22}	U_{33}	U_{23}	U_{13}	U_{12}
C11_3	89(3)	38(4)	90(4)	-14(2)	-20(2)	3(3)
C12_3	90(3)	52(4)	96(4)	-3(3)	-18(3)	7(3)
C13_3	104(3)	73(4)	110(4)	13(3)	-8(3)	24(3)
C14_3	109(3)	95(5)	135(5)	38(4)	3(3)	35(3)
C15_3	104(3)	73(4)	110(4)	13(3)	-8(3)	24(3)
C16_3	95(3)	92(5)	133(6)	35(5)	-4(3)	23(3)
C17_3	124(4)	102(5)	132(5)	40(3)	10(3)	48(3)
C18_3	127(6)	98(5)	143(5)	37(5)	-3(5)	63(5)
C19_3	117(4)	121(6)	163(8)	71(5)	7(4)	54(4)
C20_3	140(7)	113(5)	139(6)	32(4)	6(5)	60(5)
C11_4	80(2)	74(4)	87(2)	0(2)	2(2)	-10(2)
C12_4	80(2)	75(4)	88(2)	0(2)	3(2)	-10(2)
C13_4	80(2)	79(3)	88(2)	0(2)	2.5(19)	-10(2)
C14_4	78(2)	79(3)	87(2)	0(2)	2.2(19)	-8(2)
C15_4	79(3)	80(5)	87(3)	0(4)	2(3)	-9(3)
C16_4	78(3)	69(8)	86(3)	3(4)	0(3)	-6(5)
C17_4	79(2)	82(2)	87(2)	2.2(18)	2.5(17)	-6.4(17)
C18_4	80(3)	82(3)	88(3)	2(2)	4(2)	-6(2)
C19_4	77(3)	82(3)	88(4)	2(2)	4(2)	-6(2)
C20_4	82(4)	86(6)	87(2)	1(4)	2(2)	-5(4)
Cl1S_6	180(7)	175(8)	171(7)	-2(4)	-1(4)	3(5)
C1S_6	166(12)	168(13)	168(12)	1(5)	4(5)	-1(5)
Cl2S_6	204(9)	1070(110)	310(30)	190(40)	-70(20)	-170(30)
Cl1S_7	289(18)	323(14)	208(17)	-10(11)	-4(8)	33(17)
C1S_7	132(13)	147(13)	124(13)	3(10)	-12(10)	-7(11)
Cl2S_7	335(13)	393(13)	400(17)	114(12)	-124(13)	5(13)
C11_8	117(3)	51(5)	103(2)	-35(3)	25(2)	-41(3)
C12_8	116(3)	77(4)	103(2)	-29(3)	26(2)	-45(3)
C13_8	122(3)	81(6)	108(3)	-25(3)	31(2)	-43(4)
C14_8	127(3)	210(8)	113(3)	2(4)	28(3)	-72(4)
C15_8	124(3)	101(7)	109(3)	-25(3)	30(2)	-53(4)
C16_8	116(3)	77(4)	103(2)	-29(3)	26(2)	-45(3)
C17_8	137(4)	194(7)	119(3)	-4(4)	38(3)	-70(4)
C18_8	149(5)	233(8)	119(7)	12(7)	47(5)	-49(6)
C19_8	163(7)	229(11)	115(3)	1(5)	32(4)	-62(7)
C20_8	173(8)	199(7)	184(11)	-20(6)	76(7)	-84(5)
Cl1_9	147(3)	116(2)	146(3)	-60(2)	13(2)	-57(2)
O1_9	36(3)	40(2)	77(2)	7.4(18)	-16(2)	7.0(18)
C1_9	53(3)	46(3)	84(2)	0.7(19)	-8(2)	-4(2)
C2_9	73(4)	61(3)	99(2)	-10(2)	5(2)	-22(3)
C3_9	48(3)	42(3)	94(2)	0(2)	-13.9(19)	3(2)
C4_9	63(3)	61(3)	96(3)	-13(2)	-6(2)	-13(3)
C5_9	112(4)	90(4)	99(2)	-29(2)	18(2)	-50(3)
C6_9	55(3)	47(3)	82(2)	-2(2)	-7(2)	-6(3)
C8_9	67(4)	61(4)	117(4)	-19(3)	2(3)	-16(3)
C9_9	88(5)	88(4)	131(4)	-38(3)	13(3)	-36(4)
C10_9	94(5)	101(5)	128(4)	-45(4)	14(3)	-45(4)
C11_9	77(4)	79(4)	107(3)	-28(3)	1(3)	-25(3)
Cl1_10	138(3)	88(2)	196(3)	2(2)	-84(3)	-33.4(18)
O1_10	45(2)	44(2)	85(3)	-6(2)	1(2)	1.5(17)
C1_10	37(2)	45(2)	75(3)	-13(2)	10.6(19)	5.7(18)
C2_10	58(3)	57(3)	83(3)	-6(2)	1(2)	-11(2)
C3_10	49(3)	45(2)	85(3)	-12(2)	4(2)	0.3(19)
C4_10	40(3)	42(2)	75(3)	-19.9(18)	14.7(19)	12.3(18)
C5_10	69(3)	64(3)	111(3)	11(3)	-23(2)	-12(2)
C6_10	51(3)	55(3)	85(3)	-5(2)	-1(2)	1(2)

Atom	U_{11}	U_{22}	U_{33}	U_{23}	U_{13}	U_{12}
C8_10	69(4)	54(2)	119(5)	4(3)	-21(3)	-13(2)
C9_10	83(5)	62(3)	136(5)	14(3)	-36(4)	-23(3)
C10_10	66(4)	55(3)	117(4)	1(3)	-18(3)	-6(3)
C11_10	64(3)	53(3)	99(4)	-5(3)	-10(3)	-3(2)
Cl1_11	118(2)	162(3)	133(3)	24(2)	39(2)	-33(2)
O1_11	66(2)	33(3)	89(3)	-8(2)	3(2)	-7(2)
C1_11	68(2)	48(4)	91(2)	-4(3)	3.5(18)	-9(2)
C2_11	68(2)	70(5)	84(2)	0(3)	4.4(17)	-5(2)
C3_11	79(2)	89(6)	89(3)	3(3)	12(2)	-3(3)
C4_11	76.4(19)	64(5)	81(3)	-9(3)	6.0(17)	-15(2)
C5_11	77.8(18)	85(3)	91.2(15)	1.5(16)	6.7(13)	-9.4(18)
C6_11	69(2)	39(4)	88(2)	-9(2)	2.4(18)	-11(3)
C8_11	83(3)	115(7)	97(5)	15(4)	19(3)	7(3)
C9_11	93(3)	93(6)	97(4)	5(4)	27(3)	9(4)
C10_11	85(3)	88(6)	105(4)	11(4)	20(3)	-9(3)
C11_11	85(3)	90(6)	95(4)	7(4)	16(3)	-9(3)
Cl1_12	87(2)	105(2)	205(3)	64(2)	0(2)	2.4(16)
O1_12	48(3)	47.3(19)	90(3)	4(2)	-8(2)	-6.5(18)
C1_12	63(4)	47(2)	93(4)	5(2)	0(3)	-4.0(19)
C2_12	52(3)	42(2)	94(3)	-2(2)	-7(2)	-11.8(19)
C3_12	58(4)	45(2)	106(4)	4(2)	-2(3)	-9(2)
C4_12	47(3)	42(2)	97(4)	-2(2)	-12(2)	-19(2)
C5_12	78(3)	54(2)	90(3)	9(2)	3(3)	0.6(18)
C6_12	58(4)	56(2)	91(4)	10(3)	-3(3)	-1(2)
C8_12	83(5)	51(2)	113(5)	12(3)	10(4)	4(3)
C9_12	78(5)	60(3)	137(5)	27(3)	14(4)	8(3)
C10_12	76(5)	66(3)	136(5)	31(3)	13(4)	9(3)
C11_12	56(5)	57(3)	142(5)	25(3)	10(3)	-5(2)
C11_1	97.3(16)	97.5(15)	96.4(14)	2.0(9)	-0.6(9)	0.4(9)
C12_1	103(2)	101.0(17)	96.3(14)	1.0(11)	-2.4(11)	5.4(15)
C13_1	103(2)	101.0(17)	96.3(14)	1.0(11)	-2.4(11)	5.4(15)
C14_1	113(3)	106(2)	96.3(14)	-1.0(13)	-4.8(14)	13(2)
C15_1	103(2)	101.0(17)	96.3(14)	1.0(11)	-2.4(11)	5.4(15)
C16_1	103(2)	101.0(17)	96.3(14)	1.0(11)	-2.4(11)	5.4(15)
C17_1	113(3)	111(3)	98.6(17)	4(2)	-4(2)	13(2)
C18_1	112(3)	111(3)	101(3)	4(2)	-4(2)	13(2)
C19_1	112(3)	111(3)	101(3)	4(2)	-4(2)	13(2)
C20_1	112(4)	111(4)	97.6(17)	5(3)	-5(3)	14(3)
C11_5	80(2)	74(4)	87(2)	0(2)	2(2)	-10(2)
C12_5	80(2)	75(4)	88(2)	0(2)	3(2)	-10(2)
C13_5	79(3)	80(5)	87(3)	0(4)	2(3)	-9(3)
C14_5	78(2)	79(3)	88(3)	0(2)	3(2)	-9(2)
C15_5	80(2)	79(3)	88(2)	0(2)	2.5(19)	-10(2)
C16_5	80(2)	75(4)	88(2)	0(2)	3(2)	-10(2)
C17_5	79(2)	82(2)	87(2)	2.2(18)	2.5(17)	-6.4(17)
C18_5	77(3)	82(3)	88(4)	2(2)	4(2)	-6(2)
C19_5	80(3)	82(3)	88(3)	2(2)	4(2)	-6(2)
C20_5	79(2)	89(6)	87(4)	5(4)	3(3)	-6(4)

Table 10.2.3: Bond Lengths in Å for *S*-**18** (ZC-01-57).

Atom	Atom	Length/Å
Rh1	Rh2	2.5276(16)

Atom	Atom	Length/Å
Rh1	O3[1]	2.042(5)

Atom	Atom	Length/Å
Rh1	O3	2.042(5)
Rh1	O3^{2}	2.042(5)
Rh1	O3^{3}	2.042(5)
Rh1	Cl1S_6	2.224(18)
Rh2	O4^{2}	2.032(4)
Rh2	O4	2.032(4)
Rh2	O4^{3}	2.032(4)
Rh2	O4^{1}	2.032(4)
Rh2	O1W	2.265(4)
P2	O3	1.511(5)
P2	O4	1.491(5)
P2	O1_9	1.609(3)
P2	O1_12	1.594(4)
O1W	H1WB	1.0000(15)
O1W	H1WA	0.9981(13)
Rh3	Rh4	2.5149(17)
Rh3	O2^{4}	2.084(5)
Rh3	O2	2.084(5)
Rh3	O2^{5}	2.084(5)
Rh3	O2^{6}	2.084(5)
Rh3	Cl1S_7	2.23(2)
Rh4	O1	2.023(4)
Rh4	O1^{4}	2.023(4)
Rh4	O1^{5}	2.023(4)
Rh4	O1^{6}	2.023(4)
Rh4	O2W	2.272(3)
P1	O1	1.515(3)
P1	O2	1.519(3)
P1	O1_10	1.594(4)
P1	O1_11	1.610(3)
O2W	H2WA	0.9991(14)
O2W	H2WB	1.0000(15)
C11_2	C12_2	1.377(4)
C11_2	C16_2	1.372(4)
C11_2	C5_11	1.528(4)
C12_2	H12_2	1.0780
C12_2	C13_2	1.392(2)
C13_2	H13_2	1.0780
C13_2	C14_2	1.389(4)
C14_2	C15_2	1.389(4)
C14_2	C17_2	1.510(2)
C15_2	H15_2	1.0780
C15_2	C16_2	1.392(2)
C16_2	H16_2	1.0780
C17_2	C18_2	1.531(3)
C17_2	C19_2	1.531(3)
C17_2	C20_2	1.532(3)
C18_2	H18A_2	1.0980
C18_2	H18B_2	1.0980
C18_2	H18C_2	1.0980
C19_2	H19A_2	1.0980
C19_2	H19B_2	1.0980
C19_2	H19C_2	1.0980
C20_2	H20A_2	1.0980
C20_2	H20B_2	1.0980
C20_2	H20C_2	1.0980

Atom	Atom	Length/Å
C11_3	C12_3	1.376(4)
C11_3	C16_3	1.373(4)
C11_3	C5_10	1.526(4)
C12_3	H12_3	1.0780
C12_3	C13_3	1.3930(5)
C13_3	H13_3	1.0780
C13_3	C14_3	1.388(4)
C14_3	C15_3	1.389(4)
C14_3	C17_3	1.512(2)
C15_3	H15_3	1.0780
C15_3	C16_3	1.3930(5)
C16_3	H16_3	1.0780
C17_3	C18_3	1.531(3)
C17_3	C19_3	1.530(3)
C17_3	C20_3	1.533(3)
C18_3	H18A_3	1.0981
C18_3	H18B_3	1.0981
C18_3	H18C_3	1.0981
C19_3	H19A_3	1.0981
C19_3	H19B_3	1.0981
C19_3	H19C_3	1.0981
C20_3	H20A_3	1.0980
C20_3	H20B_3	1.0980
C20_3	H20C_3	1.0980
C11_4	C12_4	1.377(4)
C11_4	C16_4	1.373(4)
C11_4	C5_12	1.528(4)
C12_4	H12_4	1.0780
C12_4	C13_4	1.392(2)
C13_4	H13_4	1.0780
C13_4	C14_4	1.390(4)
C14_4	C15_4	1.390(4)
C14_4	C17_4	1.511(2)
C15_4	H15_4	1.0780
C15_4	C16_4	1.393(2)
C16_4	H16_4	1.0780
C17_4	C18_4	1.531(3)
C17_4	C19_4	1.531(3)
C17_4	C20_4	1.532(3)
C18_4	H18A_4	1.0981
C18_4	H18B_4	1.0981
C18_4	H18C_4	1.0981
C19_4	H19A_4	1.0981
C19_4	H19B_4	1.0981
C19_4	H19C_4	1.0981
C20_4	H20A_4	1.0981
C20_4	H20B_4	1.0981
C20_4	H20C_4	1.0981
Cl1S_6	C1S_6	1.778(3)
C1S_6	H1SA_6	1.0980
C1S_6	H1SB_6	1.0980
C1S_6	Cl2S_6	1.778(3)
Cl1S_7	C1S_7	1.777(3)
C1S_7	H1SA_7	1.0980
C1S_7	H1SB_7	1.0980
C1S_7	Cl2S_7	1.778(3)

Atom	Atom	Length/Å
C11_8	C12_8	1.391(3)
C11_8	C16_8	1.395(3)
C11_8	C5_9	1.527(3)
C12_8	H12_8	1.0780
C12_8	C13_8	1.3930(5)
C13_8	H13_8	1.0780
C13_8	C14_8	1.391(3)
C14_8	C15_8	1.393(3)
C14_8	C17_8	1.525(3)
C15_8	H15_8	1.0780
C15_8	C16_8	1.3929(5)
C16_8	H16_8	1.0780
C17_8	C18_8	1.555(3)
C17_8	C19_8	1.554(3)
C17_8	C20_8	1.555(3)
C18_8	H18A_8	1.0980
C18_8	H18B_8	1.0980
C18_8	H18C_8	1.0980
C19_8	H19A_8	1.0980
C19_8	H19B_8	1.0980
C19_8	H19C_8	1.0980
C20_8	H20A_8	1.0980
C20_8	H20B_8	1.0980
C20_8	H20C_8	1.0980
Cl1_9	C10_9	1.7205(19)
O1_9	C1_9	1.351(3)
C1_9	C2_9	1.394(2)
C1_9	C6_9	1.369(2)
C2_9	C3_9	1.436(2)
C2_9	C2_12	1.499(4)
C3_9	C4_9	1.425(2)
C3_9	C8_9	1.417(2)
C4_9	C5_9	1.467(2)
C4_9	C11_9	1.416(2)
C5_9	C6_9	1.376(2)
C6_9	H6_9	1.0780
C8_9	H8_9	1.0780
C8_9	C9_9	1.394(2)
C9_9	H9_9	1.0780
C9_9	C10_9	1.385(2)
C10_9	C11_9	1.393(2)
C11_9	H11_9	1.0780
Cl1_10	C10_10	1.7196(16)
O1_10	C1_10	1.350(3)
C1_10	C2_10	1.393(2)
C1_10	C6_10	1.369(2)
C2_10	C3_10	1.437(2)
C2_10	C2_11	1.496(4)
C3_10	C4_10	1.426(2)
C3_10	C8_10	1.419(2)
C4_10	C5_10	1.466(2)
C4_10	C11_10	1.416(2)
C5_10	C6_10	1.375(2)
C6_10	H6_10	1.0780
C8_10	H8_10	1.0780
C8_10	C9_10	1.396(2)

Atom	Atom	Length/Å
C9_10	H9_10	1.0780
C9_10	C10_10	1.383(2)
C10_10	C11_10	1.391(2)
C11_10	H11_10	1.0780
Cl1_11	C10_11	1.7182(16)
O1_11	C1_11	1.346(3)
C1_11	C2_11	1.396(2)
C1_11	C6_11	1.368(2)
C2_11	C3_11	1.440(2)
C3_11	C4_11	1.426(2)
C3_11	C8_11	1.417(2)
C4_11	C5_11	1.467(2)
C4_11	C11_11	1.417(2)
C5_11	C6_11	1.381(2)
C5_11	C11_1	1.533(3)
C6_11	H6_11	1.0780
C8_11	H8_11	1.0780
C8_11	C9_11	1.395(2)
C9_11	H9_11	1.0780
C9_11	C10_11	1.384(2)
C10_11	C11_11	1.391(2)
C11_11	H11_11	1.0780
Cl1_12	C10_12	1.7182(16)
O1_12	C1_12	1.348(3)
C1_12	C2_12	1.3949
C1_12	C6_12	1.3690
C2_12	C3_12	1.4347
C3_12	C4_12	1.4255
C3_12	C8_12	1.4184
C4_12	C5_12	1.4655
C4_12	C11_12	1.4172
C5_12	C6_12	1.3728
C5_12	C11_5	1.528(4)
C6_12	H6_12	1.0780
C8_12	H8_12	1.0780
C8_12	C9_12	1.3956
C9_12	H9_12	1.0780
C9_12	C10_12	1.3831
C10_12	C11_12	1.3910
C11_12	H11_12	1.0780
C11_1	C12_1	1.378(4)
C11_1	C16_1	1.373(4)
C12_1	H12_1	1.0780
C12_1	C13_1	1.3929(5)
C13_1	H13_1	1.0780
C13_1	C14_1	1.389(4)
C14_1	C15_1	1.389(3)
C14_1	C17_1	1.509(2)
C15_1	H15_1	1.0780
C15_1	C16_1	1.392(2)
C16_1	H16_1	1.0780
C17_1	C18_1	1.531(3)
C17_1	C19_1	1.531(3)
C17_1	C20_1	1.533(3)
C18_1	H18A_1	1.0980
C18_1	H18B_1	1.0980

Atom	Atom	Length/Å
C18_1	H18C_1	1.0980
C19_1	H19A_1	1.0980
C19_1	H19B_1	1.0980
C19_1	H19C_1	1.0980
C20_1	H20A_1	1.0981
C20_1	H20B_1	1.0981
C20_1	H20C_1	1.0981
C11_5	C12_5	1.377(4)
C11_5	C16_5	1.373(4)
C12_5	H12_5	1.0780
C12_5	C13_5	1.392(2)
C13_5	H13_5	1.0780
C13_5	C14_5	1.389(4)
C14_5	C15_5	1.389(4)
C14_5	C17_5	1.511(2)
C15_5	H15_5	1.0780
C15_5	C16_5	1.392(2)
C16_5	H16_5	1.0780
C17_5	C18_5	1.531(3)
C17_5	C19_5	1.530(3)
C17_5	C20_5	1.533(3)
C18_5	H18A_5	1.0981
C18_5	H18B_5	1.0981
C18_5	H18C_5	1.0981
C19_5	H19A_5	1.0980
C19_5	H19B_5	1.0980
C19_5	H19C_5	1.0980
C20_5	H20A_5	1.0980
C20_5	H20B_5	1.0980
C20_5	H20C_5	1.0980

----

^{1}2-x,2-y,+z; ^{2}+y,2-x,+z; ^{3}2-y,+x,+z; ^{4}1-y,+x,+z; ^{5}+y,1-x,+z; ^{6}1-x,1-y,+z

Table 10.2.4: Bond Angles in ° for *S*-**18** (ZC-01-57).

Atom	Atom	Atom	Angle/°
O3^{1}	Rh1	Rh2	91.96(14)
O3	Rh1	Rh2	91.96(14)
O3^{2}	Rh1	Rh2	91.96(14)
O3^{3}	Rh1	Rh2	91.96(14)
O3^{3}	Rh1	O3	89.935(10)
O3^{1}	Rh1	O3	176.1(3)
O3^{1}	Rh1	O3^{2}	89.933(11)
O3^{2}	Rh1	O3	89.930(10)
O3^{1}	Rh1	O3^{3}	89.933(10)
O3^{3}	Rh1	O3^{2}	176.1(3)
O3^{1}	Rh1	Cl1S_6	88.04(14)
O3^{2}	Rh1	Cl1S_6	88.04(14)
O3^{3}	Rh1	Cl1S_6	88.04(14)
O3	Rh1	Cl1S_6	88.04(14)
Cl1S_6	Rh1	Rh2	180.0
O4^{3}	Rh2	Rh1	88.98(13)
O4^{1}	Rh2	Rh1	88.98(13)
O4^{2}	Rh2	Rh1	88.98(13)
O4	Rh2	Rh1	88.98(13)
O4^{2}	Rh2	O4	89.983(5)
O4^{1}	Rh2	O4^{2}	89.982(6)
O4^{1}	Rh2	O4	178.0(3)
O4^{3}	Rh2	O4	89.980(6)
O4^{3}	Rh2	O4^{2}	178.0(3)
O4^{1}	Rh2	O4^{3}	89.982(6)
O4^{1}	Rh2	O1W	91.02(13)
O4^{2}	Rh2	O1W	91.02(13)
O4	Rh2	O1W	91.02(13)
O4^{3}	Rh2	O1W	91.02(13)
O1W	Rh2	Rh1	180.0
O3	P2	O1_9	113.0(3)
O3	P2	O1_12	104.1(3)
O4	P2	O3	117.5(3)
O4	P2	O1_9	105.9(3)
O4	P2	O1_12	113.6(3)
O1_12	P2	O1_9	101.8(2)
P2	O3	Rh1	114.3(3)
P2	O4	Rh2	119.2(3)
Rh2	O1W	H1WB	114(5)
Rh2	O1W	H1WA	112.82(18)
H1WB	O1W	H1WA	101.7(3)
O2^{4}	Rh3	Rh4	93.23(9)
O2^{5}	Rh3	Rh4	93.23(9)
O2	Rh3	Rh4	93.23(9)
O2^{6}	Rh3	Rh4	93.23(9)
O2^{6}	Rh3	O2	89.820(11)
O2^{4}	Rh3	O2	89.816(11)
O2^{5}	Rh3	O2	173.54(19)
O2^{4}	Rh3	O2^{6}	173.54(19)
O2^{4}	Rh3	O2^{5}	89.818(11)
O2^{5}	Rh3	O2^{6}	89.818(11)
O2^{6}	Rh3	Cl1S_7	86.77(9)
O2^{4}	Rh3	Cl1S_7	86.77(9)
O2	Rh3	Cl1S_7	86.77(9)
O2^{5}	Rh3	Cl1S_7	86.77(9)
Cl1S_7	Rh3	Rh4	180.0
O1	Rh4	Rh3	89.52(10)
O1^{4}	Rh4	Rh3	89.51(10)
O1^{6}	Rh4	Rh3	89.51(10)
O1^{5}	Rh4	Rh3	89.51(10)
O1^{6}	Rh4	O1^{5}	89.996(2)
O1	Rh4	O1^{5}	179.0(2)
O1	Rh4	O1^{4}	89.994(2)
O1	Rh4	O1^{6}	89.998(3)
O1^{4}	Rh4	O1^{5}	89.996(2)
O1^{4}	Rh4	O1^{6}	179.0(2)

Atom	Atom	Atom	Angle/°
O1	Rh4	O2W	90.48(10)
O1[6]	Rh4	O2W	90.49(10)
O1[5]	Rh4	O2W	90.49(10)
O1[4]	Rh4	O2W	90.49(10)
O2W	Rh4	Rh3	180.0
O1	P1	O2	119.2(2)
O1	P1	O1_10	111.8(3)
O1	P1	O1_11	109.2(2)
O2	P1	O1_10	104.8(3)
O2	P1	O1_11	107.6(3)
O1_10	P1	O1_11	103.0(2)
P1	O1	Rh4	119.1(2)
P1	O2	Rh3	112.7(2)
Rh4	O2W	H2WA	116.38(16)
Rh4	O2W	H2WB	119(5)
H2WA	O2W	H2WB	101.7(3)
C12_2	C11_2	C5_11	117.2(8)
C16_2	C11_2	C12_2	120.2(4)
C16_2	C11_2	C5_11	121.8(8)
C11_2	C12_2	H12_2	120.0
C11_2	C12_2	C13_2	120.1(5)
C13_2	C12_2	H12_2	120.0
C12_2	C13_2	H13_2	119.8
C14_2	C13_2	C12_2	120.5(5)
C14_2	C13_2	H13_2	119.8
C13_2	C14_2	C15_2	118.5(3)
C13_2	C14_2	C17_2	119.0(4)
C15_2	C14_2	C17_2	122.5(4)
C14_2	C15_2	H15_2	119.6
C14_2	C15_2	C16_2	120.8(4)
C16_2	C15_2	H15_2	119.6
C11_2	C16_2	C15_2	119.8(4)
C11_2	C16_2	H16_2	120.1
C15_2	C16_2	H16_2	120.1
C14_2	C17_2	C18_2	109.9(4)
C14_2	C17_2	C19_2	111.1(4)
C14_2	C17_2	C20_2	110.2(4)
C18_2	C17_2	C20_2	108.4(3)
C19_2	C17_2	C18_2	108.6(3)
C19_2	C17_2	C20_2	108.6(3)
C17_2	C18_2	H18A_2	109.5
C17_2	C18_2	H18B_2	109.5
C17_2	C18_2	H18C_2	109.5
H18A_2	C18_2	H18B_2	109.5
H18A_2	C18_2	H18C_2	109.5
H18B_2	C18_2	H18C_2	109.5
C17_2	C19_2	H19A_2	109.5
C17_2	C19_2	H19B_2	109.5
C17_2	C19_2	H19C_2	109.5
H19A_2	C19_2	H19B_2	109.5
H19A_2	C19_2	H19C_2	109.5
H19B_2	C19_2	H19C_2	109.5
C17_2	C20_2	H20A_2	109.5
C17_2	C20_2	H20B_2	109.5
C17_2	C20_2	H20C_2	109.5
H20A_2	C20_2	H20B_2	109.5
H20A_2	C20_2	H20C_2	109.5
H20B_2	C20_2	H20C_2	109.5
C12_3	C11_3	C5_10	122.3(5)
C16_3	C11_3	C12_3	120.5(4)
C16_3	C11_3	C5_10	116.2(5)
C11_3	C12_3	H12_3	120.0
C11_3	C12_3	C13_3	120.0(5)
C13_3	C12_3	H12_3	120.0
C12_3	C13_3	H13_3	119.8
C14_3	C13_3	C12_3	120.4(5)
C14_3	C13_3	H13_3	119.8
C13_3	C14_3	C15_3	118.7(3)
C13_3	C14_3	C17_3	119.0(4)
C15_3	C14_3	C17_3	122.3(4)
C14_3	C15_3	H15_3	119.6
C14_3	C15_3	C16_3	120.8(4)
C16_3	C15_3	H15_3	119.6
C11_3	C16_3	C15_3	119.7(4)
C11_3	C16_3	H16_3	120.2
C15_3	C16_3	H16_3	120.2
C14_3	C17_3	C18_3	109.8(4)
C14_3	C17_3	C19_3	111.1(4)
C14_3	C17_3	C20_3	110.0(4)
C18_3	C17_3	C20_3	108.5(3)
C19_3	C17_3	C18_3	108.8(3)
C19_3	C17_3	C20_3	108.6(3)
C17_3	C18_3	H18A_3	109.5
C17_3	C18_3	H18B_3	109.5
C17_3	C18_3	H18C_3	109.5
H18A_3	C18_3	H18B_3	109.5
H18A_3	C18_3	H18C_3	109.5
H18B_3	C18_3	H18C_3	109.5
C17_3	C19_3	H19A_3	109.5
C17_3	C19_3	H19B_3	109.5
C17_3	C19_3	H19C_3	109.5
H19A_3	C19_3	H19B_3	109.5
H19A_3	C19_3	H19C_3	109.5
H19B_3	C19_3	H19C_3	109.5
C17_3	C20_3	H20A_3	109.5
C17_3	C20_3	H20B_3	109.5
C17_3	C20_3	H20C_3	109.5
H20A_3	C20_3	H20B_3	109.5
H20A_3	C20_3	H20C_3	109.5
H20B_3	C20_3	H20C_3	109.5
C12_4	C11_4	C5_12	121.6(7)
C16_4	C11_4	C12_4	120.3(4)
C16_4	C11_4	C5_12	118.1(7)
C11_4	C12_4	H12_4	120.0
C11_4	C12_4	C13_4	120.1(5)
C13_4	C12_4	H12_4	120.0
C12_4	C13_4	H13_4	119.9
C14_4	C13_4	C12_4	120.3(5)
C14_4	C13_4	H13_4	119.9
C13_4	C14_4	C15_4	118.4(3)
C13_4	C14_4	C17_4	118.8(4)
C15_4	C14_4	C17_4	122.4(4)

Atom	Atom	Atom	Angle/°
C14_4	C15_4	H15_4	119.7
C14_4	C15_4	C16_4	120.5(4)
C16_4	C15_4	H15_4	119.7
C11_4	C16_4	C15_4	119.6(4)
C11_4	C16_4	H16_4	120.2
C15_4	C16_4	H16_4	120.2
C14_4	C17_4	C18_4	109.8(4)
C14_4	C17_4	C19_4	111.0(4)
C14_4	C17_4	C20_4	110.2(5)
C18_4	C17_4	C19_4	108.7(3)
C18_4	C17_4	C20_4	108.5(3)
C19_4	C17_4	C20_4	108.6(3)
C17_4	C18_4	H18A_4	109.5
C17_4	C18_4	H18B_4	109.5
C17_4	C18_4	H18C_4	109.5
H18A_4	C18_4	H18B_4	109.5
H18A_4	C18_4	H18C_4	109.5
H18B_4	C18_4	H18C_4	109.5
C17_4	C19_4	H19A_4	109.5
C17_4	C19_4	H19B_4	109.5
C17_4	C19_4	H19C_4	109.5
H19A_4	C19_4	H19B_4	109.5
H19A_4	C19_4	H19C_4	109.5
H19B_4	C19_4	H19C_4	109.5
C17_4	C20_4	H20A_4	109.5
C17_4	C20_4	H20B_4	109.5
C17_4	C20_4	H20C_4	109.5
H20A_4	C20_4	H20B_4	109.5
H20A_4	C20_4	H20C_4	109.5
H20B_4	C20_4	H20C_4	109.5
C1S_6	Cl1S_6	Rh1	131.5(7)
Cl1S_6	C1S_6	H1SA_6	109.4
Cl1S_6	C1S_6	H1SB_6	109.4
H1SA_6	C1S_6	H1SB_6	108.0
Cl2S_6	C1S_6	Cl1S_6	111.0(4)
Cl2S_6	C1S_6	H1SA_6	109.4
Cl2S_6	C1S_6	H1SB_6	109.4
C1S_7	Cl1S_7	Rh3	119.3(12)
Cl1S_7	C1S_7	H1SA_7	111.6
Cl1S_7	C1S_7	H1SB_7	111.6
Cl1S_7	C1S_7	Cl2S_7	101.0(8)
H1SA_7	C1S_7	H1SB_7	109.4
Cl2S_7	C1S_7	H1SA_7	111.6
Cl2S_7	C1S_7	H1SB_7	111.6
C12_8	C11_8	C16_8	117.7(3)
C12_8	C11_8	C5_9	121.6(3)
C16_8	C11_8	C5_9	120.3(3)
C11_8	C12_8	H12_8	119.4
C11_8	C12_8	C13_8	121.2(3)
C13_8	C12_8	H12_8	119.4
C12_8	C13_8	H13_8	119.5
C14_8	C13_8	C12_8	121.0(3)
C14_8	C13_8	H13_8	119.5
C13_8	C14_8	C15_8	118.0(3)
C13_8	C14_8	C17_8	121.5(3)
C15_8	C14_8	C17_8	120.4(3)

Atom	Atom	Atom	Angle/°
C14_8	C15_8	H15_8	119.5
C16_8	C15_8	C14_8	120.9(3)
C16_8	C15_8	H15_8	119.5
C11_8	C16_8	H16_8	119.4
C15_8	C16_8	C11_8	121.1(3)
C15_8	C16_8	H16_8	119.4
C14_8	C17_8	C18_8	110.9(3)
C14_8	C17_8	C19_8	110.8(2)
C14_8	C17_8	C20_8	110.6(3)
C18_8	C17_8	C20_8	108.5(3)
C19_8	C17_8	C18_8	107.3(3)
C19_8	C17_8	C20_8	108.7(3)
C17_8	C18_8	H18A_8	109.5
C17_8	C18_8	H18B_8	109.5
C17_8	C18_8	H18C_8	109.5
H18A_8	C18_8	H18B_8	109.5
H18A_8	C18_8	H18C_8	109.5
H18B_8	C18_8	H18C_8	109.5
C17_8	C19_8	H19A_8	109.5
C17_8	C19_8	H19B_8	109.5
C17_8	C19_8	H19C_8	109.5
H19A_8	C19_8	H19B_8	109.5
H19A_8	C19_8	H19C_8	109.5
H19B_8	C19_8	H19C_8	109.5
C17_8	C20_8	H20A_8	109.5
C17_8	C20_8	H20B_8	109.5
C17_8	C20_8	H20C_8	109.5
H20A_8	C20_8	H20B_8	109.5
H20A_8	C20_8	H20C_8	109.5
H20B_8	C20_8	H20C_8	109.5
C1_9	O1_9	P2	116.5(4)
O1_9	C1_9	C2_9	119.3(3)
O1_9	C1_9	C6_9	118.3(3)
C6_9	C1_9	C2_9	121.85(18)
C1_9	C2_9	C3_9	118.41(19)
C1_9	C2_9	C2_12	115.9(2)
C3_9	C2_9	C2_12	125.7(3)
C4_9	C3_9	C2_9	119.49(17)
C8_9	C3_9	C2_9	122.52(19)
C8_9	C3_9	C4_9	117.92(16)
C3_9	C4_9	C5_9	119.08(17)
C11_9	C4_9	C3_9	118.12(16)
C11_9	C4_9	C5_9	122.59(19)
C4_9	C5_9	C11_8	120.5(3)
C6_9	C5_9	C11_8	121.7(3)
C6_9	C5_9	C4_9	117.41(19)
C1_9	C6_9	C5_9	123.0(2)
C1_9	C6_9	H6_9	118.5
C5_9	C6_9	H6_9	118.5
C3_9	C8_9	H8_9	118.6
C9_9	C8_9	C3_9	122.7(2)
C9_9	C8_9	H8_9	118.6
C8_9	C9_9	H9_9	120.6
C10_9	C9_9	C8_9	118.80(19)
C10_9	C9_9	H9_9	120.6
C9_9	C10_9	Cl1_9	119.9(2)

Atom	Atom	Atom	Angle/°
C9_9	C10_9	C11_9	119.95(19)
C11_9	C10_9	Cl1_9	119.1(2)
C4_9	C11_9	H11_9	119.1
C10_9	C11_9	C4_9	121.9(2)
C10_9	C11_9	H11_9	119.1
C1_10	O1_10	P1	120.7(4)
O1_10	C1_10	C2_10	119.6(3)
O1_10	C1_10	C6_10	118.3(2)
C6_10	C1_10	C2_10	121.65(18)
C1_10	C2_10	C3_10	118.64(18)
C1_10	C2_10	C2_11	120.3(3)
C3_10	C2_10	C2_11	120.8(3)
C4_10	C3_10	C2_10	119.35(16)
C8_10	C3_10	C2_10	122.3(2)
C8_10	C3_10	C4_10	117.77(17)
C3_10	C4_10	C5_10	119.30(15)
C11_10	C4_10	C3_10	118.15(16)
C11_10	C4_10	C5_10	122.45(19)
C4_10	C5_10	C11_3	122.8(3)
C6_10	C5_10	C11_3	119.4(3)
C6_10	C5_10	C4_10	117.82(18)
C1_10	C6_10	C5_10	122.8(2)
C1_10	C6_10	H6_10	118.6
C5_10	C6_10	H6_10	118.6
C3_10	C8_10	H8_10	118.7
C9_10	C8_10	C3_10	122.5(2)
C9_10	C8_10	H8_10	118.7
C8_10	C9_10	H9_10	120.6
C10_10	C9_10	C8_10	118.77(19)
C10_10	C9_10	H9_10	120.6
C9_10	C10_10	Cl1_10	120.4(2)
C9_10	C10_10	C11_10	120.22(16)
C11_10	C10_10	Cl1_10	119.4(2)
C4_10	C11_10	H11_10	118.9
C10_10	C11_10	C4_10	122.12(19)
C10_10	C11_10	H11_10	118.9
C1_11	O1_11	P1	115.8(4)
O1_11	C1_11	C2_11	119.6(2)
O1_11	C1_11	C6_11	119.0(3)
C6_11	C1_11	C2_11	121.39(19)
C1_11	C2_11	C2_10	119.1(3)
C1_11	C2_11	C3_11	117.7(2)
C3_11	C2_11	C2_10	121.6(3)
C4_11	C3_11	C2_11	119.03(19)
C8_11	C3_11	C2_11	122.2(2)
C8_11	C3_11	C4_11	118.04(16)
C3_11	C4_11	C5_11	119.28(16)
C11_11	C4_11	C3_11	117.96(17)
C11_11	C4_11	C5_11	122.68(19)
C4_11	C5_11	C11_2	121.6(5)
C4_11	C5_11	C11_1	123.2(3)
C6_11	C5_11	C11_2	120.9(5)
C6_11	C5_11	C4_11	117.33(18)
C6_11	C5_11	C11_1	119.5(2)
C1_11	C6_11	C5_11	122.8(2)
C1_11	C6_11	H6_11	118.6

Atom	Atom	Atom	Angle/°
C5_11	C6_11	H6_11	118.6
C3_11	C8_11	H8_11	118.8
C9_11	C8_11	C3_11	122.5(2)
C9_11	C8_11	H8_11	118.8
C8_11	C9_11	H9_11	120.7
C10_11	C9_11	C8_11	118.6(2)
C10_11	C9_11	H9_11	120.7
C9_11	C10_11	Cl1_11	120.1(2)
C9_11	C10_11	C11_11	120.34(16)
C11_11	C10_11	Cl1_11	119.5(2)
C4_11	C11_11	H11_11	119.0
C10_11	C11_11	C4_11	121.95(19)
C10_11	C11_11	H11_11	119.0
C1_12	O1_12	P2	124.7(3)
O1_12	C1_12	C2_12	119.5(2)
O1_12	C1_12	C6_12	118.4(2)
C6_12	C1_12	C2_12	121.8
C1_12	C2_12	C2_9	118.7(3)
C1_12	C2_12	C3_12	118.6
C3_12	C2_12	C2_9	122.4(3)
C4_12	C3_12	C2_12	119.5
C8_12	C3_12	C2_12	122.6
C8_12	C3_12	C4_12	117.8
C3_12	C4_12	C5_12	119.3
C11_12	C4_12	C3_12	118.2
C11_12	C4_12	C5_12	122.4
C4_12	C5_12	C11_4	122.2(7)
C4_12	C5_12	C11_5	116.8(6)
C6_12	C5_12	C11_4	119.8(8)
C6_12	C5_12	C4_12	117.8
C6_12	C5_12	C11_5	124.3(6)
C1_12	C6_12	C5_12	122.9
C1_12	C6_12	H6_12	118.5
C5_12	C6_12	H6_12	118.5
C3_12	C8_12	H8_12	118.7
C9_12	C8_12	C3_12	122.6
C9_12	C8_12	H8_12	118.7
C8_12	C9_12	H9_12	120.5
C10_12	C9_12	C8_12	119.0
C10_12	C9_12	H9_12	120.5
C9_12	C10_12	Cl1_12	120.47(17)
C9_12	C10_12	C11_12	120.2
C11_12	C10_12	Cl1_12	119.37(17)
C4_12	C11_12	H11_12	119.0
C10_12	C11_12	C4_12	122.0
C10_12	C11_12	H11_12	119.0
C12_1	C11_1	C5_11	119.2(3)
C16_1	C11_1	C5_11	120.7(3)
C16_1	C11_1	C12_1	120.1(3)
C11_1	C12_1	H12_1	119.9
C11_1	C12_1	C13_1	120.2(5)
C13_1	C12_1	H12_1	119.9
C12_1	C13_1	H13_1	119.8
C14_1	C13_1	C12_1	120.5(5)
C14_1	C13_1	H13_1	119.8
C13_1	C14_1	C15_1	118.5(3)

Atom	Atom	Atom	Angle/°
C13_1	C14_1	C17_1	119.0(4)
C15_1	C14_1	C17_1	122.6(4)
C14_1	C15_1	H15_1	119.6
C14_1	C15_1	C16_1	120.9(4)
C16_1	C15_1	H15_1	119.6
C11_1	C16_1	C15_1	119.9(4)
C11_1	C16_1	H16_1	120.0
C15_1	C16_1	H16_1	120.0
C14_1	C17_1	C18_1	109.9(4)
C14_1	C17_1	C19_1	111.1(5)
C14_1	C17_1	C20_1	110.2(5)
C18_1	C17_1	C20_1	108.4(3)
C19_1	C17_1	C18_1	108.6(3)
C19_1	C17_1	C20_1	108.6(3)
C17_1	C18_1	H18A_1	109.5
C17_1	C18_1	H18B_1	109.5
C17_1	C18_1	H18C_1	109.5
H18A_1	C18_1	H18B_1	109.5
H18A_1	C18_1	H18C_1	109.5
H18B_1	C18_1	H18C_1	109.5
C17_1	C19_1	H19A_1	109.5
C17_1	C19_1	H19B_1	109.5
C17_1	C19_1	H19C_1	109.5
H19A_1	C19_1	H19B_1	109.5
H19A_1	C19_1	H19C_1	109.5
H19B_1	C19_1	H19C_1	109.5
C17_1	C20_1	H20A_1	109.5
C17_1	C20_1	H20B_1	109.5
C17_1	C20_1	H20C_1	109.5
H20A_1	C20_1	H20B_1	109.5
H20A_1	C20_1	H20C_1	109.5
H20B_1	C20_1	H20C_1	109.5
C12_5	C11_5	C5_12	110.6(7)
C16_5	C11_5	C5_12	128.6(7)
C16_5	C11_5	C12_5	120.3(4)
C11_5	C12_5	H12_5	120.0
C11_5	C12_5	C13_5	120.1(5)
C13_5	C12_5	H12_5	120.0
C12_5	C13_5	H13_5	119.8

Atom	Atom	Atom	Angle/°
C14_5	C13_5	C12_5	120.4(5)
C14_5	C13_5	H13_5	119.8
C13_5	C14_5	C15_5	118.6(3)
C13_5	C14_5	C17_5	118.9(4)
C15_5	C14_5	C17_5	122.3(4)
C14_5	C15_5	H15_5	119.6
C14_5	C15_5	C16_5	120.8(4)
C16_5	C15_5	H15_5	119.6
C11_5	C16_5	C15_5	119.8(4)
C11_5	C16_5	H16_5	120.1
C15_5	C16_5	H16_5	120.1
C14_5	C17_5	C18_5	109.8(4)
C14_5	C17_5	C19_5	111.1(5)
C14_5	C17_5	C20_5	110.1(5)
C18_5	C17_5	C20_5	108.5(3)
C19_5	C17_5	C18_5	108.7(3)
C19_5	C17_5	C20_5	108.6(3)
C17_5	C18_5	H18A_5	109.5
C17_5	C18_5	H18B_5	109.5
C17_5	C18_5	H18C_5	109.5
H18A_5	C18_5	H18B_5	109.5
H18A_5	C18_5	H18C_5	109.5
H18B_5	C18_5	H18C_5	109.5
C17_5	C19_5	H19A_5	109.5
C17_5	C19_5	H19B_5	109.5
C17_5	C19_5	H19C_5	109.5
H19A_5	C19_5	H19B_5	109.5
H19A_5	C19_5	H19C_5	109.5
H19B_5	C19_5	H19C_5	109.5
C17_5	C20_5	H20A_5	109.5
C17_5	C20_5	H20B_5	109.5
C17_5	C20_5	H20C_5	109.5
H20A_5	C20_5	H20B_5	109.5
H20A_5	C20_5	H20C_5	109.5
H20B_5	C20_5	H20C_5	109.5

––––

[1]2-x,2-y,+z; [2]2-y,+x,+z; [3]+y,2-x,+z; [4]+y,1-x,+z; [5]1-x,1-y,+z; [6]1-y,+x,+z

Table 10.2.5: Torsion Angles in ° for *S*-**18** (ZC-01-57).

Atom	Atom	Atom	Atom	Angle/°
Rh1	Cl1S_6	C1S_6	Cl2S_6	7(4)
P2	O1_9	C1_9	C2_9	-75.1(8)
P2	O1_9	C1_9	C6_9	96.6(7)
P2	O1_12	C1_12	C2_12	-62.7(5)
P2	O1_12	C1_12	C6_12	111.4(3)
O3	P2	O4	Rh2	-29.2(4)
O3	P2	O1_9	C1_9	-54.4(4)
O3	P2	O1_12	C1_12	152.8(4)
O4	P2	O3	Rh1	33.2(4)
O4	P2	O1_9	C1_9	175.6(3)
O4	P2	O1_12	C1_12	-78.2(4)

Atom	Atom	Atom	Atom	Angle/°
Rh3	Cl1S_7	C1S_7	Cl2S_7	90.6(14)
P1	O1_10	C1_10	C2_10	-73.2(8)
P1	O1_10	C1_10	C6_10	114.0(6)
P1	O1_11	C1_11	C2_11	-77.1(8)
P1	O1_11	C1_11	C6_11	105.9(7)
O1	P1	O2	Rh3	28.3(4)
O1	P1	O1_10	C1_10	-79.4(4)
O1	P1	O1_11	C1_11	173.9(3)
O2	P1	O1	Rh4	-28.8(4)
O2	P1	O1_10	C1_10	150.2(4)
O2	P1	O1_11	C1_11	-55.4(4)
C11_2	C12_2	C13_2	C14_2	0.00(8)
C11_2	C5_11	C6_11	C1_11	176.9(9)
C12_2	C11_2	C16_2	C15_2	-0.01(17)
C12_2	C11_2	C5_11	C4_11	68.2(10)
C12_2	C11_2	C5_11	C6_11	-116.0(9)
C12_2	C13_2	C14_2	C15_2	0.00(5)
C12_2	C13_2	C14_2	C17_2	179.6(5)
C13_2	C14_2	C15_2	C16_2	-0.01(8)
C13_2	C14_2	C17_2	C18_2	-84.3(8)
C13_2	C14_2	C17_2	C19_2	35.9(8)
C13_2	C14_2	C17_2	C20_2	156.3(8)
C14_2	C15_2	C16_2	C11_2	0.01(16)
C15_2	C14_2	C17_2	C18_2	95.3(8)
C15_2	C14_2	C17_2	C19_2	-144.4(8)
C15_2	C14_2	C17_2	C20_2	-24.1(8)
C16_2	C11_2	C12_2	C13_2	0.00(11)
C16_2	C11_2	C5_11	C4_11	-122.3(8)
C16_2	C11_2	C5_11	C6_11	53.5(11)
C17_2	C14_2	C15_2	C16_2	-179.6(6)
C11_3	C12_3	C13_3	C14_3	-0.1(2)
C11_3	C5_10	C6_10	C1_10	-178.8(7)
C12_3	C11_3	C16_3	C15_3	0.03(19)
C12_3	C11_3	C5_10	C4_10	136.4(7)
C12_3	C11_3	C5_10	C6_10	-44.0(10)
C12_3	C13_3	C14_3	C15_3	0.08(18)
C12_3	C13_3	C14_3	C17_3	-179.4(3)
C13_3	C14_3	C15_3	C16_3	-0.04(8)
C13_3	C14_3	C17_3	C18_3	-106.5(4)
C13_3	C14_3	C17_3	C19_3	13.9(5)
C13_3	C14_3	C17_3	C20_3	134.2(4)
C14_3	C15_3	C16_3	C11_3	-0.02(8)
C15_3	C14_3	C17_3	C18_3	74.1(5)
C15_3	C14_3	C17_3	C19_3	-165.5(4)
C15_3	C14_3	C17_3	C20_3	-45.3(4)
C16_3	C11_3	C12_3	C13_3	0.0(2)
C16_3	C11_3	C5_10	C4_10	-55.0(9)
C16_3	C11_3	C5_10	C6_10	124.6(7)
C17_3	C14_3	C15_3	C16_3	179.4(3)
C11_4	C12_4	C13_4	C14_4	0(2)
C11_4	C5_12	C6_12	C1_12	-172.5(7)
C12_4	C11_4	C16_4	C15_4	-1(2)
C12_4	C11_4	C5_12	C4_12	-72.0(18)
C12_4	C11_4	C5_12	C6_12	102.5(16)
C12_4	C13_4	C14_4	C15_4	7(2)
C12_4	C13_4	C14_4	C17_4	179.7(13)

Atom	Atom	Atom	Atom	Angle/˚
C13_4	C14_4	C15_4	C16_4	-11(2)
C13_4	C14_4	C17_4	C18_4	-102.5(12)
C13_4	C14_4	C17_4	C19_4	17.8(13)
C13_4	C14_4	C17_4	C20_4	138.1(12)
C14_4	C15_4	C16_4	C11_4	8(2)
C15_4	C14_4	C17_4	C18_4	70.1(12)
C15_4	C14_4	C17_4	C19_4	-169.6(12)
C15_4	C14_4	C17_4	C20_4	-49.3(12)
C16_4	C11_4	C12_4	C13_4	-3(3)
C16_4	C11_4	C5_12	C4_12	107.8(14)
C16_4	C11_4	C5_12	C6_12	-77.7(17)
C17_4	C14_4	C15_4	C16_4	176.2(12)
C11_8	C12_8	C13_8	C14_8	0.01(4)
C11_8	C5_9	C6_9	C1_9	-177.3(7)
C12_8	C11_8	C16_8	C15_8	-0.01(9)
C12_8	C11_8	C5_9	C4_9	57.9(8)
C12_8	C11_8	C5_9	C6_9	-129.6(7)
C12_8	C13_8	C14_8	C15_8	-0.02(9)
C12_8	C13_8	C14_8	C17_8	176.2(5)
C13_8	C14_8	C15_8	C16_8	0.02(12)
C13_8	C14_8	C17_8	C18_8	-132.9(5)
C13_8	C14_8	C17_8	C19_8	-13.8(6)
C13_8	C14_8	C17_8	C20_8	106.8(5)
C14_8	C15_8	C16_8	C11_8	0.00(13)
C15_8	C14_8	C17_8	C18_8	43.3(5)
C15_8	C14_8	C17_8	C19_8	162.3(5)
C15_8	C14_8	C17_8	C20_8	-77.1(5)
C16_8	C11_8	C12_8	C13_8	0.01(4)
C16_8	C11_8	C5_9	C4_9	-129.4(6)
C16_8	C11_8	C5_9	C6_9	43.1(9)
C17_8	C14_8	C15_8	C16_8	-176.3(5)
Cl1_9	C10_9	C11_9	C4_9	-177.8(7)
O1_9	P2	O3	Rh1	-90.7(3)
O1_9	P2	O4	Rh2	98.2(3)
O1_9	P2	O1_12	C1_12	35.1(4)
O1_9	C1_9	C2_9	C3_9	171.2(7)
O1_9	C1_9	C2_9	C2_12	-7.6(11)
O1_9	C1_9	C6_9	C5_9	-171.5(7)
C1_9	C2_9	C3_9	C4_9	5.4(12)
C1_9	C2_9	C3_9	C8_9	-177.8(8)
C1_9	C2_9	C2_12	C1_12	61.8(8)
C1_9	C2_9	C2_12	C3_12	-123.9(6)
C2_9	C1_9	C6_9	C5_9	-0.1(13)
C2_9	C3_9	C4_9	C5_9	-10.0(11)
C2_9	C3_9	C4_9	C11_9	175.0(8)
C2_9	C3_9	C8_9	C9_9	-176.6(9)
C2_9	C2_12	C3_12	C4_12	-174.5(4)
C2_9	C2_12	C3_12	C8_12	2.5(4)
C3_9	C2_9	C2_12	C1_12	-116.9(7)
C3_9	C2_9	C2_12	C3_12	57.4(10)
C3_9	C4_9	C5_9	C11_8	-177.6(6)
C3_9	C4_9	C5_9	C6_9	9.6(12)
C3_9	C4_9	C11_9	C10_9	6.6(13)
C3_9	C8_9	C9_9	C10_9	-2.9(15)
C4_9	C3_9	C8_9	C9_9	0.2(13)
C4_9	C5_9	C6_9	C1_9	-4.6(12)

Atom	Atom	Atom	Atom	Angle/°
C5_9	C11_8	C12_8	C13_8	172.8(5)
C5_9	C11_8	C16_8	C15_8	-172.9(5)
C5_9	C4_9	C11_9	C10_9	-168.2(9)
C6_9	C1_9	C2_9	C3_9	-0.2(12)
C6_9	C1_9	C2_9	C2_12	-179.0(7)
C8_9	C3_9	C4_9	C5_9	173.0(7)
C8_9	C3_9	C4_9	C11_9	-2.0(11)
C8_9	C9_9	C10_9	Cl1_9	175.6(8)
C8_9	C9_9	C10_9	C11_9	7.3(15)
C9_9	C10_9	C11_9	C4_9	-9.4(15)
C11_9	C4_9	C5_9	C11_8	-2.9(12)
C11_9	C4_9	C5_9	C6_9	-175.7(8)
Cl1_10	C10_10	C11_10	C4_10	178.6(6)
O1_10	P1	O1	Rh4	-151.3(2)
O1_10	P1	O2	Rh3	154.2(2)
O1_10	P1	O1_11	C1_11	55.0(3)
O1_10	C1_10	C2_10	C3_10	179.9(6)
O1_10	C1_10	C2_10	C2_11	5.8(10)
O1_10	C1_10	C6_10	C5_10	177.6(7)
C1_10	C2_10	C3_10	C4_10	4.5(10)
C1_10	C2_10	C3_10	C8_10	-166.2(7)
C1_10	C2_10	C2_11	C1_11	47.1(11)
C1_10	C2_10	C2_11	C3_11	-118.2(8)
C2_10	C1_10	C6_10	C5_10	4.9(12)
C2_10	C3_10	C4_10	C5_10	0.9(10)
C2_10	C3_10	C4_10	C11_10	-175.6(7)
C2_10	C3_10	C8_10	C9_10	178.5(8)
C2_10	C2_11	C3_11	C4_11	-177.9(7)
C2_10	C2_11	C3_11	C8_11	-8.1(12)
C3_10	C2_10	C2_11	C1_11	-126.9(8)
C3_10	C2_10	C2_11	C3_11	67.8(10)
C3_10	C4_10	C5_10	C11_3	175.9(7)
C3_10	C4_10	C5_10	C6_10	-3.6(11)
C3_10	C4_10	C11_10	C10_10	1.5(9)
C3_10	C8_10	C9_10	C10_10	-7.6(12)
C4_10	C3_10	C8_10	C9_10	7.6(12)
C4_10	C5_10	C6_10	C1_10	0.8(13)
C5_10	C11_3	C12_3	C13_3	168.1(5)
C5_10	C11_3	C16_3	C15_3	-168.8(4)
C5_10	C4_10	C11_10	C10_10	-174.9(6)
C6_10	C1_10	C2_10	C3_10	-7.6(11)
C6_10	C1_10	C2_10	C2_11	178.3(7)
C8_10	C3_10	C4_10	C5_10	172.1(7)
C8_10	C3_10	C4_10	C11_10	-4.4(10)
C8_10	C9_10	C10_10	Cl1_10	-175.7(7)
C8_10	C9_10	C10_10	C11_10	4.3(7)
C9_10	C10_10	C11_10	C4_10	-1.4(6)
C11_10	C4_10	C5_10	C11_3	-7.7(12)
C11_10	C4_10	C5_10	C6_10	172.8(7)
Cl1_11	C10_11	C11_11	C4_11	174.7(7)
O1_11	P1	O1	Rh4	95.4(3)
O1_11	P1	O2	Rh3	-96.6(3)
O1_11	P1	O1_10	C1_10	37.8(4)
O1_11	C1_11	C2_11	C2_10	0.3(11)
O1_11	C1_11	C2_11	C3_11	166.1(7)
O1_11	C1_11	C6_11	C5_11	-170.5(7)

Atom	Atom	Atom	Atom	Angle/°
C1_11	C2_11	C3_11	C4_11	16.6(11)
C1_11	C2_11	C3_11	C8_11	-173.6(8)
C2_11	C2_10	C3_10	C4_10	178.6(6)
C2_11	C2_10	C3_10	C8_10	7.8(11)
C2_11	C1_11	C6_11	C5_11	12.6(12)
C2_11	C3_11	C4_11	C5_11	-11.9(11)
C2_11	C3_11	C4_11	C11_11	165.0(8)
C2_11	C3_11	C8_11	C9_11	-162.9(8)
C3_11	C4_11	C5_11	C11_2	-177.1(9)
C3_11	C4_11	C5_11	C6_11	7.0(11)
C3_11	C4_11	C5_11	C11_1	-172.8(6)
C3_11	C4_11	C11_11	C10_11	4.6(11)
C3_11	C8_11	C9_11	C10_11	-7.5(12)
C4_11	C3_11	C8_11	C9_11	7.0(13)
C4_11	C5_11	C6_11	C1_11	-7.1(11)
C4_11	C5_11	C11_1	C12_1	-144.3(7)
C4_11	C5_11	C11_1	C16_1	35.9(8)
C5_11	C11_2	C12_2	C13_2	169.7(8)
C5_11	C11_2	C16_2	C15_2	-169.2(9)
C5_11	C4_11	C11_11	C10_11	-178.6(6)
C5_11	C11_1	C12_1	C13_1	-179.8(3)
C5_11	C11_1	C16_1	C15_1	179.8(3)
C6_11	C1_11	C2_11	C2_10	177.2(7)
C6_11	C1_11	C2_11	C3_11	-16.9(11)
C6_11	C5_11	C11_1	C12_1	35.9(8)
C6_11	C5_11	C11_1	C16_1	-143.9(7)
C8_11	C3_11	C4_11	C5_11	177.8(8)
C8_11	C3_11	C4_11	C11_11	-5.3(11)
C8_11	C9_11	C10_11	Cl1_11	-173.5(7)
C8_11	C9_11	C10_11	C11_11	6.5(7)
C9_11	C10_11	C11_11	C4_11	-5.3(7)
C11_11	C4_11	C5_11	C11_2	6.2(13)
C11_11	C4_11	C5_11	C6_11	-169.8(7)
C11_11	C4_11	C5_11	C11_1	10.5(11)
Cl1_12	C10_12	C11_12	C4_12	179.1(3)
O1_12	P2	O3	Rh1	159.7(2)
O1_12	P2	O4	Rh2	-151.0(3)
O1_12	P2	O1_9	C1_9	56.7(3)
O1_12	C1_12	C2_12	C2_9	-10.6(4)
O1_12	C1_12	C2_12	C3_12	174.9(4)
O1_12	C1_12	C6_12	C5_12	-176.0(4)
C1_12	C2_12	C3_12	C4_12	-0.2
C1_12	C2_12	C3_12	C8_12	176.8
C2_12	C2_9	C3_9	C4_9	-176.0(7)
C2_12	C2_9	C3_9	C8_9	0.9(13)
C2_12	C1_12	C6_12	C5_12	-2.1
C2_12	C3_12	C4_12	C5_12	0.4
C2_12	C3_12	C4_12	C11_12	-176.8
C2_12	C3_12	C8_12	C9_12	178.0
C3_12	C4_12	C5_12	C11_4	173.2(7)
C3_12	C4_12	C5_12	C6_12	-1.4
C3_12	C4_12	C5_12	C11_5	167.1(7)
C3_12	C4_12	C11_12	C10_12	-3.5
C3_12	C8_12	C9_12	C10_12	0.9
C4_12	C3_12	C8_12	C9_12	-5.0
C4_12	C5_12	C6_12	C1_12	2.2

Atom	Atom	Atom	Atom	Angle/°
C4_12	C5_12	C11_5	C12_5	131.3(6)
C4_12	C5_12	C11_5	C16_5	-56.9(11)
C5_12	C11_4	C12_4	C13_4	176.6(15)
C5_12	C11_4	C16_4	C15_4	179.1(14)
C5_12	C4_12	C11_12	C10_12	179.4
C5_12	C11_5	C12_5	C13_5	172.6(11)
C5_12	C11_5	C16_5	C15_5	-171.2(14)
C6_12	C1_12	C2_12	C2_9	175.5(4)
C6_12	C1_12	C2_12	C3_12	0.9
C6_12	C5_12	C11_5	C12_5	-61.0(10)
C6_12	C5_12	C11_5	C16_5	110.9(10)
C8_12	C3_12	C4_12	C5_12	-176.7
C8_12	C3_12	C4_12	C11_12	6.1
C8_12	C9_12	C10_12	Cl1_12	-177.8(3)
C8_12	C9_12	C10_12	C11_12	2.0
C9_12	C10_12	C11_12	C4_12	-0.7
C11_12	C4_12	C5_12	C11_4	-9.7(7)
C11_12	C4_12	C5_12	C6_12	175.7
C11_12	C4_12	C5_12	C11_5	-15.8(7)
C11_1	C5_11	C6_11	C1_11	172.6(6)
C11_1	C12_1	C13_1	C14_1	-0.02(9)
C12_1	C11_1	C16_1	C15_1	0.01(5)
C12_1	C13_1	C14_1	C15_1	0.04(15)
C12_1	C13_1	C14_1	C17_1	-179.8(3)
C13_1	C14_1	C15_1	C16_1	-0.03(14)
C13_1	C14_1	C17_1	C18_1	-61.1(8)
C13_1	C14_1	C17_1	C19_1	59.1(8)
C13_1	C14_1	C17_1	C20_1	179.5(7)
C14_1	C15_1	C16_1	C11_1	0.01(7)
C15_1	C14_1	C17_1	C18_1	119.1(8)
C15_1	C14_1	C17_1	C19_1	-120.7(7)
C15_1	C14_1	C17_1	C20_1	-0.3(8)
C16_1	C11_1	C12_1	C13_1	-0.01(6)
C17_1	C14_1	C15_1	C16_1	179.8(4)
C11_5	C5_12	C6_12	C1_12	-165.4(7)
C11_5	C12_5	C13_5	C14_5	0.00(8)
C12_5	C11_5	C16_5	C15_5	-0.01(19)
C12_5	C13_5	C14_5	C15_5	0.00(18)
C12_5	C13_5	C14_5	C17_5	-175.2(10)
C13_5	C14_5	C15_5	C16_5	0.0(3)
C13_5	C14_5	C17_5	C18_5	-127.7(9)
C13_5	C14_5	C17_5	C19_5	-7.5(10)
C13_5	C14_5	C17_5	C20_5	112.9(9)
C14_5	C15_5	C16_5	C11_5	0.0(3)
C15_5	C14_5	C17_5	C18_5	57.2(10)
C15_5	C14_5	C17_5	C19_5	177.5(9)
C15_5	C14_5	C17_5	C20_5	-62.1(10)
C16_5	C11_5	C12_5	C13_5	0.01(9)
C17_5	C14_5	C15_5	C16_5	175.0(11)

Table 10.2.6: Hydrogen Fractional Atomic Coordinates (×10^4) and Equivalent Isotropic Displacement Parameters (Å^2×10^3) for *S*-**18** (ZC-01-57). U_{eq} is defined as 1/3 of the trace of the orthogonalised U_{ij}.

Atom	x	y	z	U_{eq}
H1WB	10263(4)	9760(20)	1930(40)	50
H1WA	9670	9863.49	1919.01	50
H2WA	5301.9	5172	4839.7	63
H2WB	4700.4(16)	5160(30)	4860(40)	63
H12_2	6392.94	5891.24	-853.98	120
H13_2	5834.75	6263.56	-1878.15	120
H15_2	5558.92	7584.91	-441.37	120
H16_2	6117.69	7208.39	578.25	120
H18A_2	5410.49	7610.35	-2936.95	161
H18B_2	5865.22	7772.31	-2184.39	161
H18C_2	5939.4	7180.82	-2723.6	161
H19A_2	4780.38	6906.29	-2754.45	162
H19B_2	5297.31	6460.84	-2548.59	162
H19C_2	4771.99	6533.8	-1872.11	162
H20A_2	4685.54	7711.36	-2010.24	162
H20B_2	4680.46	7358.69	-1109.69	162
H20C_2	5136.28	7875.88	-1253.53	162
H12_3	7152.63	4442.11	5091.75	95
H13_3	7491.11	3767.89	5977.94	115
H15_3	8977.12	3897.05	4902.56	115
H16_3	8634.54	4571.55	4020.88	128
H18A_3	9123.63	3811.6	6395.95	184
H18B_3	8950.47	3300.28	7055.5	184
H18C_3	8571.5	3883.77	7022.9	184
H19A_3	7823.74	3346.6	6882.61	200
H19B_3	8141.09	2735.06	6733.6	200
H19C_3	7705.69	2983.68	6008.9	200
H20A_3	8908.74	2662.97	5996.15	196
H20B_3	8526.41	2786.92	5143.31	196
H20C_3	9104.15	3156.65	5320.7	196
H12_4	7738.3	7159.94	784.97	97
H13_4	7072.66	7042.81	1803.66	98
H15_4	7929.23	8042.64	3299.68	98
H16_4	8648.02	8063.16	2344.35	93
H18A_4	6994.2	8232.23	3471.55	125
H18B_4	6455.81	7852.91	3809.82	125
H18C_4	6536.63	7972.26	2787.75	125
H19A_4	6447.36	7076.37	2582.69	123
H19B_4	6484.81	6837.81	3562.53	123
H19C_4	6947.28	6625.79	2849.49	123
H20A_4	7703.46	7467.94	4086.63	127
H20B_4	7346.07	6871.78	4143.35	127
H20C_4	7071.8	7458.74	4525.4	127
H1SA_6	10524.18	9801.38	-3496.85	201
H1SB_6	10410.74	10479.16	-3407.3	201
H1SA_7	4237.49	5529.27	326.79	161
H1SB_7	4467.42	5277.81	-583.37	161
H12_8	10225.64	8225.8	-3863.69	118
H13_8	10825.8	8524.5	-4878.78	125
H15_8	12051.48	8576.18	-3211.14	134
H16_8	11450.48	8277.42	-2196.48	118
H18A_8	12648.78	8525.81	-5149.9	250
H18B_8	12557.36	8480.57	-4116.57	250
H18C_8	12291.24	7992.82	-4745.7	250
H19A_8	11922.01	8732.98	-6025.69	253
H19B_8	11551.72	8203.78	-5636.4	253

Atom	x	y	z	U_{eq}
H19C_8	11280.89	8844.83	-5655.32	253
H20A_8	12281.75	9416.43	-5159.08	278
H20B_8	11650.32	9545.63	-4764.67	278
H20C_8	12179.78	9393.24	-4126.33	278
H6_9	10672.26	8699.74	-1547.67	74
H8_9	9071.3	7062.92	-1387.24	98
H9_9	9078.29	6503.73	-2548.91	123
H11_9	10369.32	7351.17	-3435.88	105
H6_10	7045.51	4373.81	3609.88	76
H8_10	7746	6388.38	2366.78	97
H9_10	8520.03	6717.63	3053.25	112
H11_10	8486.13	5388.64	4533.71	86
H6_11	5915.6	6280.48	1349.79	78
H8_11	8021.97	5379.4	1498.54	118
H9_11	8582.48	5719.78	451.21	113
H11_11	7274.82	6488.41	-542.39	108
H6_12	8477.76	8444.34	650.4	82
H8_12	9934.76	6888.17	-624.22	99
H9_12	9918.39	6074.54	126.6	110
H11_12	8767.44	6720.44	1626.61	102
H12_1	5816.72	6970.87	676.35	120
H13_1	5331.54	7395.2	-385.77	120
H15_1	6170.46	6493.08	-2043.97	120
H16_1	6652.41	6072.03	-977.84	120
H18A_1	4615.17	7329.45	-2293.42	162
H18B_1	4765.29	6712.43	-1865.78	162
H18C_1	4702.53	7276.06	-1260.17	162
H19A_1	5272.77	8017.17	-2367.38	162
H19B_1	5367.99	7982.33	-1333.91	162
H19C_1	5911.39	7918.08	-1979.5	162
H20A_1	5714.67	7372.47	-3087.53	161
H20B_1	5255.77	6851.25	-3015.85	161
H20C_1	5904.22	6767.02	-2657.62	161
H12_5	8499.99	8183.06	2120.18	97
H13_5	7759.4	8177.52	3030.7	98
H15_5	7108.92	6894.89	1802.26	98
H16_5	7850.88	6904.43	895.97	97
H18A_5	6679.31	6764.77	2961.48	123
H18B_5	6522.63	7087.38	3856.86	123
H18C_5	7180.23	6901.94	3657.09	123
H19A_5	6648.23	7995.81	4018.5	125
H19B_5	7333.62	7875.49	3997.05	125
H19C_5	7056.59	8359.93	3372.78	125
H20A_5	6092.63	7739.36	2924.97	127
H20B_5	6491.7	8092.7	2253.2	127
H20C_5	6359.83	7418.03	2089.61	127

Table 10.2.7: Atomic Occupancies for all atoms that are not fully occupied in *S*-**18** (ZC-01-57).

Atom	Occupancy
H1WB	0.25
H1WA	0.25
H2WA	0.25

Atom	Occupancy
H2WB	0.25
C11_2	0.5
C12_2	0.5

Atom	Occupancy
H12_2	0.5
C13_2	0.5
H13_2	0.5

Atom	Occupancy	Atom	Occupancy	Atom	Occupancy
C14_2	0.5	C19_4	0.557(7)	C19_1	0.5
C15_2	0.5	H19A_4	0.557(7)	H19A_1	0.5
H15_2	0.5	H19B_4	0.557(7)	H19B_1	0.5
C16_2	0.5	H19C_4	0.557(7)	H19C_1	0.5
H16_2	0.5	C20_4	0.557(7)	C20_1	0.5
C17_2	0.5	H20A_4	0.557(7)	H20A_1	0.5
C18_2	0.5	H20B_4	0.557(7)	H20B_1	0.5
H18A_2	0.5	H20C_4	0.557(7)	H20C_1	0.5
H18B_2	0.5	Cl1S_6	0.125	C11_5	0.443(7)
H18C_2	0.5	C1S_6	0.5	C12_5	0.443(7)
C19_2	0.5	H1SA_6	0.5	H12_5	0.443(7)
H19A_2	0.5	H1SB_6	0.5	C13_5	0.443(7)
H19B_2	0.5	Cl2S_6	0.25	H13_5	0.443(7)
H19C_2	0.5	Cl1S_7	0.125	C14_5	0.443(7)
C20_2	0.5	C1S_7	0.5	C15_5	0.443(7)
H20A_2	0.5	H1SA_7	0.5	H15_5	0.443(7)
H20B_2	0.5	H1SB_7	0.5	C16_5	0.443(7)
H20C_2	0.5	Cl2S_7	0.5	H16_5	0.443(7)
C11_4	0.557(7)	C11_1	0.5	C17_5	0.443(7)
C12_4	0.557(7)	C12_1	0.5	C18_5	0.443(7)
H12_4	0.557(7)	H12_1	0.5	H18A_5	0.443(7)
C13_4	0.557(7)	C13_1	0.5	H18B_5	0.443(7)
H13_4	0.557(7)	H13_1	0.5	H18C_5	0.443(7)
C14_4	0.557(7)	C14_1	0.5	C19_5	0.443(7)
C15_4	0.557(7)	C15_1	0.5	H19A_5	0.443(7)
H15_4	0.557(7)	H15_1	0.5	H19B_5	0.443(7)
C16_4	0.557(7)	C16_1	0.5	H19C_5	0.443(7)
H16_4	0.557(7)	H16_1	0.5	C20_5	0.443(7)
C17_4	0.557(7)	C17_1	0.5	H20A_5	0.443(7)
C18_4	0.557(7)	C18_1	0.5	H20B_5	0.443(7)
H18A_4	0.557(7)	H18A_1	0.5	H20C_5	0.443(7)
H18B_4	0.557(7)	H18B_1	0.5		
H18C_4	0.557(7)	H18C_1	0.5		

Table 10.2.8: Solvent masking (PLATON/SQUEEZE) information for *S*-**18** (ZC-01-57).

No	x	y	z	V	e	Content
1	-0.124	-0.516	-0.255	5012.0	880.0	20C1,20H2,20Cl2

Citations

CrysAlisPro (ROD), Rigaku Oxford Diffraction, Poland (?).

CrysAlisPro Software System, Rigaku Oxford Diffraction, (2023).

O.V. Dolomanov and L.J. Bourhis and R.J. Gildea and J.A.K. Howard and H. Puschmann, Olex2: A complete structure solution, refinement and analysis program, *J. Appl. Cryst.*, (2009), **42**, 339-341.

Sheldrick, G.M., Crystal structure refinement with ShelXL, *Acta Cryst.*, (2015), **C71**, 3-8.

Sheldrick, G.M., ShelXT-Integrated space-group and crystal-structure determination, *Acta Cryst.*, (2015), **A71**, 3-8.

10.3 X-Ray Crystallographic Data for S-1

R_1=5.30%

Crystal Data and Experimental

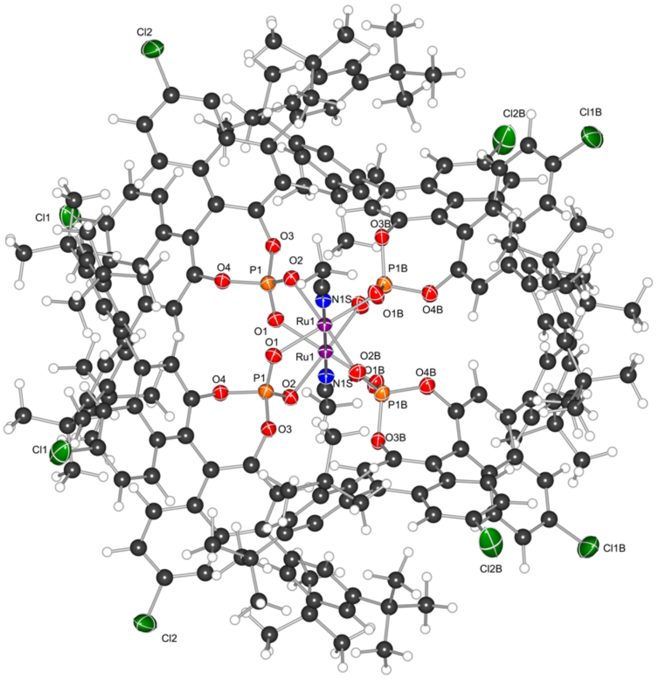

**Compound**	*S*-**1**
**CCDC**	**2349402**
Formula	$C_{196}H_{206}Cl_8N_2O_{16}P_4Rh_2$
$D_{calc.}$/ g cm^{-3}	1.093
μ/mm^{-1}	0.278
Formula Weight	4526.31
Colour	bluish green
Shape	plate-shaped
Size/mm^3	0.33×0.22×0.11
T/K	100.0(4)
Crystal System	orthorhombic
Flack Parameter	0.15(3)
Hooft Parameter	-0.065(11)
Space Group	$P2_12_12$
a/Å	31.9181(10)
b/Å	20.7813(5)
c/Å	20.7296(6)
α/°	90
β/°	90
γ/°	90
V/Å^3	13750.0(7)
Z	2
Z'	0.5
Wavelength/Å	0.71073
Radiation type	Mo K_α
Θ_{min}/°	2.911
Θ_{max}/°	24.710
Measured Refl's.	68215
Indep't Refl's	20587
Refl's I≥2 σ(I)	14808
R_{int}	0.0557
Parameters	1047
Restraints	1477
Largest Peak	0.309
Deepest Hole	-0.325
GooF	0.968
wR_2 (all data)	0.1241
wR_2	0.1197
R_1 (all data)	0.0654
R_1	0.0530

Experimental. Single bluish green plate-shaped crystals of *S*-**1** were prepared via slow evaporation of a sample solution of dichloromethane and hexane. A suitable crystal with dimensions 0.33 × 0.22 × 0.11 mm^3 was selected and mounted on a loop with paratone on a XtaLAB Synergy, Dualflex, HyPix diffractometer. The crystal was kept at a steady T = 100.0(4) K during data collection. The structure was solved with the ShelXT (Sheldrick, 2015) solution program using dual methods and by using Olex2 1.5-alpha (Dolomanov et al., 2009) as the graphical interface. The model was refined with ShelXL 2018/3 (Sheldrick, 2015) using full matrix least squares minimisation on F^2.

Crystal Data. $C_{196}H_{206}Cl_8N_2O_{16}P_4Rh_2$, M_r = 4526.31, orthorhombic, $P2_12_12$ (No. 18), a = 31.9181(10) Å, b = 20.7813(5) Å, c = 20.7296(6) Å, $\alpha = \beta = \gamma$ = 90°, V = 13750.0(7) Å^3, T = 100.0(4) K, Z = 2, Z' = 0.5, μ(Mo K_α) = 0.278, 68215 reflections measured, 20587 unique (R_{int} = 0.0557) which were used in all calculations. The final wR_2 was 0.1241 (all data) and R_1 was 0.0530 (I≥2 σ(I)).

Structure Quality Indicators

**Reflections:**	d min (MoKα) 2Θ=49.4° 0.85	I/σ(I) 14.2	Rint m=3.31 5.57%	Full 49.4° 94.2
**Refinement:**	Shift 0.002	Max Peak 0.3	Min Peak -0.3	GooF 0.968

A bluish green plate-shaped-shaped crystal with dimensions 0.33 × 0.22 × 0.11 mm^3 was mounted on a loop with paratone. Data were collected using a XtaLAB Synergy, Dualflex, HyPix diffractometer equipped with an Oxford Cryosystems low-temperature device operating at T = 100.0(4) K.

Data were measured using ω scans with Mo K_{α} radiation. The diffraction pattern was indexed and the total number of runs and images was based on the strategy calculation from the program CrysAlisPro 1.171.42.78a (Rigaku OD, 2022). The maximum resolution that was achieved was Θ = 24.710° (0.85 Å).

The unit cell was refined using CrysAlisPro 1.171.42.78a (Rigaku OD, 2022) on 14791 reflections, 22% of the observed reflections.

Data reduction, scaling and absorption corrections were performed using CrysAlisPro 1.171.42.78a (Rigaku OD, 2022). The final completeness is 93.90 % out to 24.710° in Θ. A gaussian absorption correction was performed using CrysAlisPro 1.171.42.78a (Rigaku Oxford Diffraction, 2022). A numerical absorption correction based on gaussian integration over a multifaceted crystal model was performed using CrysAlisPro 1.171.41.122a (Rigaku Oxford Diffraction, 2021). An empirical absorption correction using spherical harmonics, implemented in SCALE3 ABSPACK scaling algorithm was also applied. The absorption coefficient μ of this material is 0.278 mm^{-1} at this wavelength (λ = 0.71073Å) and the minimum and maximum transmissions are 0.766 and 1.000.

The structure was solved and the space group $P2_12_12$ (# 18) determined by the ShelXT (Sheldrick, 2015) structure solution program using using dual methods and refined by full matrix least squares minimisation on $\boldsymbol{F}^2$ using version 2018/3 of ShelXL 2018/3 (Sheldrick, 2015). All non-hydrogen atoms were refined anisotropically. Hydrogen atom positions were calculated geometrically and refined using the riding model. Hydrogen atom positions were calculated geometrically and refined using the riding model.

_refine_special_details: Refined as a 2-component inversion twin.

The value of Z' is 0.5. This means that only half of the formula unit is present in the asymmetric unit, with the other half consisting of symmetry equivalent atoms. The moiety formula is C196 H206 Cl8 N2 O16 P4 Rh2, 26[C2], 26[H3], 26[N1].

The Flack parameter was refined to 0.15(3). Determination of absolute structure using Bayesian statistics on Bijvoet differences using the Olex2 results in -0.065(11). The chiral atoms in this structure are: P1(R). Note: The Flack parameter is used to determine chirality of the crystal studied, the value should be near 0, a value of 1 means that the stereochemistry is wrong and the model should be inverted. A value of 0.5 means that the crystal consists of a racemic mixture of the two enantiomers.

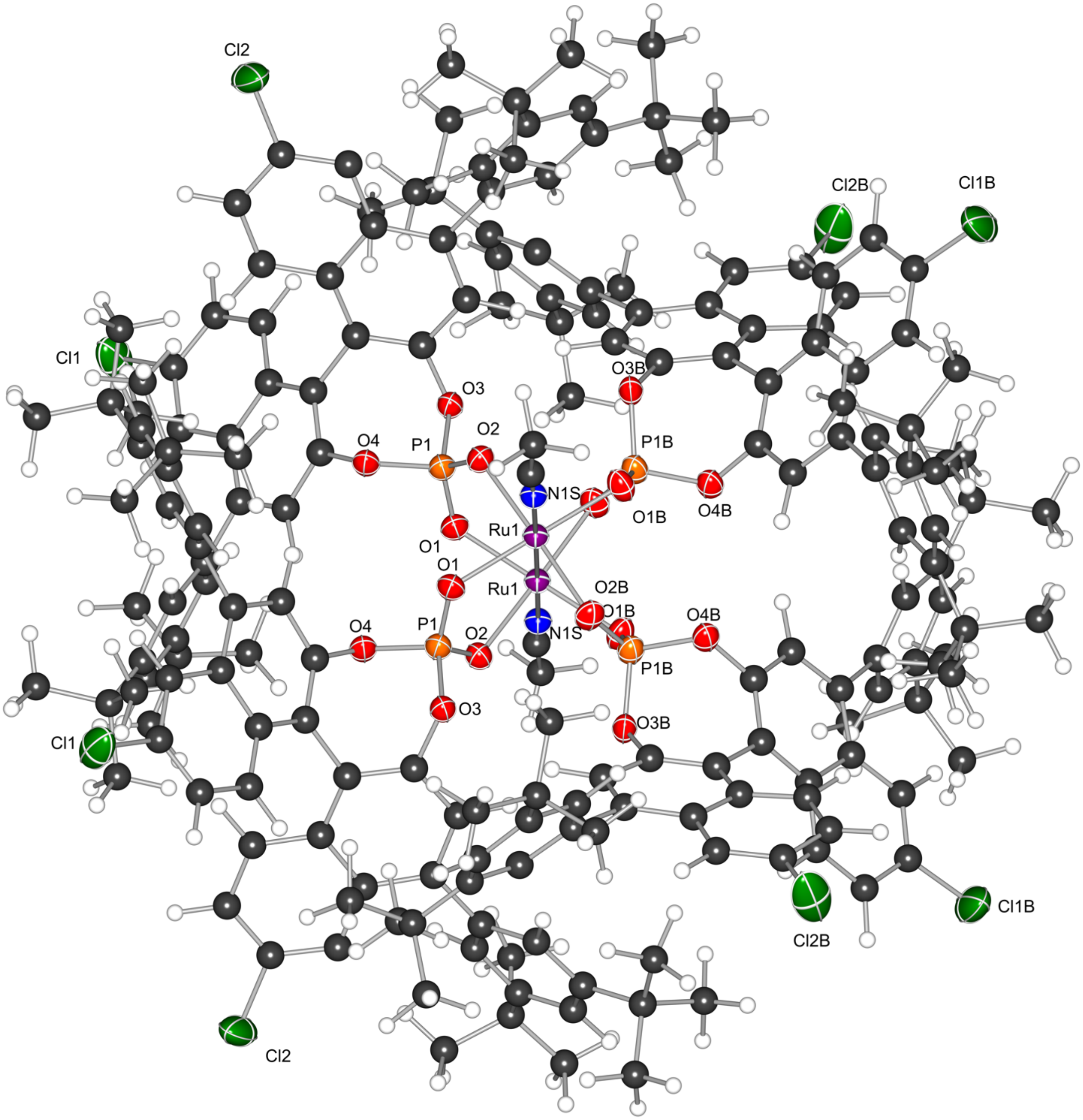

Figure 10.3.4 Molecular structure of the dirhodium complex *S*-**1**

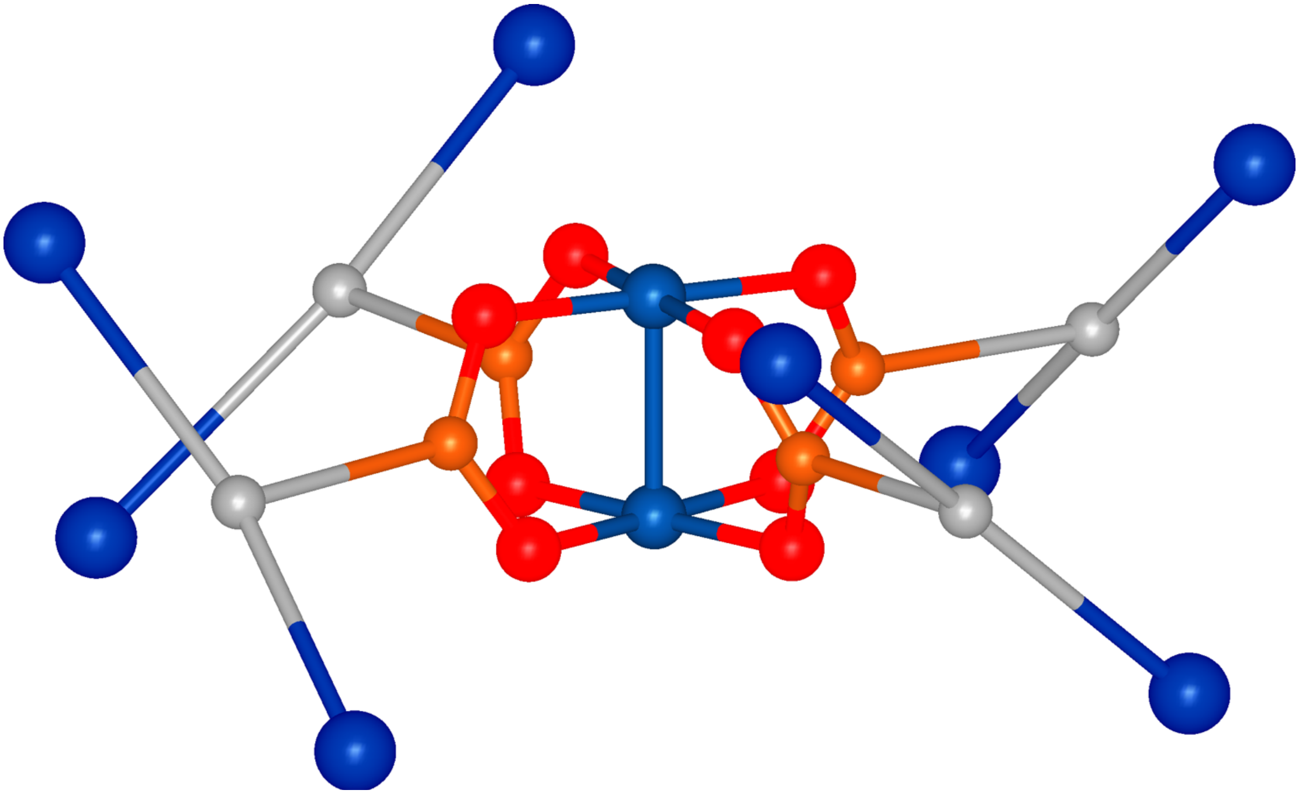

Figure 10.3.5 Simplified cluster, with centroids representing the centroids of the ligand rings showing the underlying symmetry of the complex. The complex has near-perfect D4 (and perfect C2) crystallographic symmetry.

Data Plots: Diffraction Data

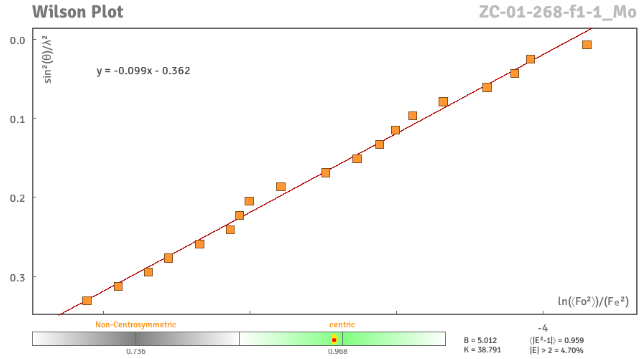

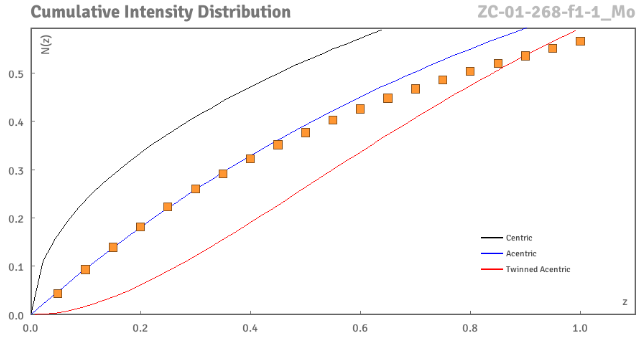

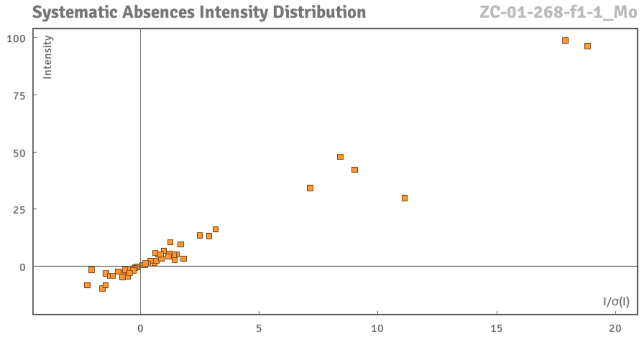

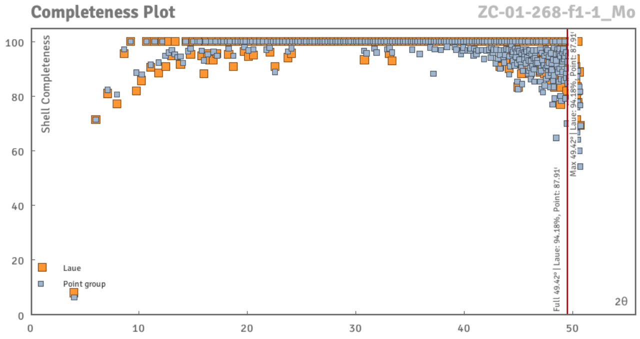

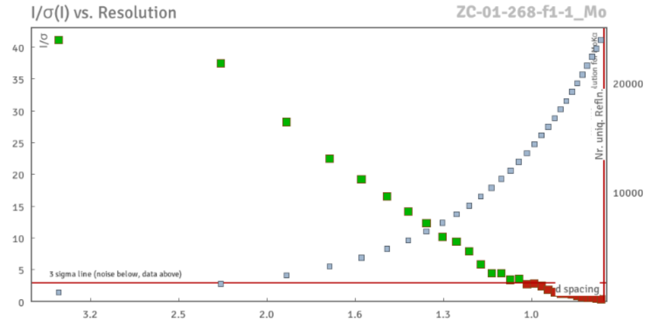

Data Plots: Refinement and Data

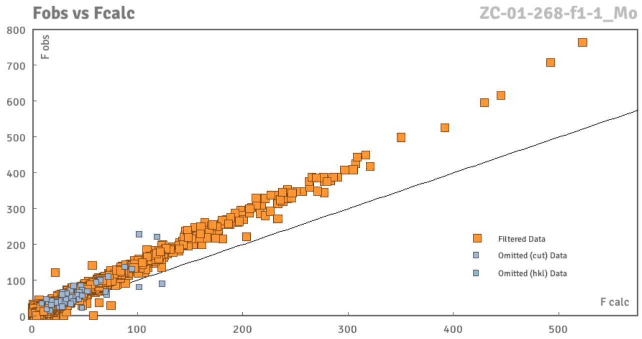

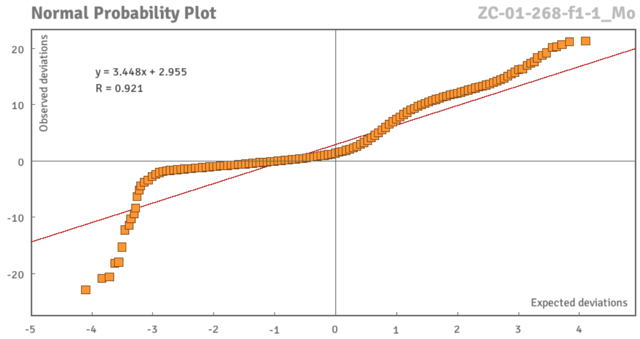

Reflection Statistics

Total reflections (after filtering)	87155	Unique reflections	24266
Completeness	0.965	Mean I/σ	8.85
hkl_{max} collected	(38, 25, 24)	hkl_{min} collected	(-36, -24, -24)
hkl_{max} used	(38, 25, 24)	hkl_{min} used	(-38, 0, 0)
Lim d_{max} collected	100.0	Lim d_{min} collected	0.36
d_{max} used	8.03	d_{min} used	0.83
Friedel pairs	18512	Friedel pairs merged	0
Inconsistent equivalents	13	R_{int}	0.0733
R_{sigma}	0.09	Intensity transformed	0
Omitted reflections	252	Omitted by user (OMIT hkl)	0
Multiplicity	(38226, 18429, 3559, 375, 28, 1)	Maximum multiplicity	10
Removed systematic absences	66	Filtered off (Shel/OMIT)	0

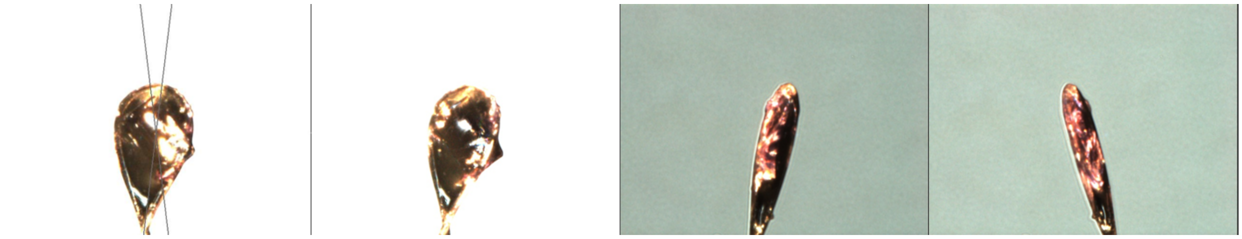

Table 10.3.9: Fractional Atomic Coordinates (×10⁴) and Equivalent Isotropic Displacement Parameters (Å²×10³) for *S*-**1**. U_{eq} is defined as 1/3 of the trace of the orthogonalised U_{ij}.

Atom	x	y	z	U_{eq}
Rh1	5392.9(2)	4985.9(2)	5635.7(2)	71.01(15)
N1S	6108.8(14)	4939(2)	5617(2)	82.5(12)
C1S	6439.9(18)	4938(4)	5608(4)	127(3)
C2S	6892.7(18)	4866(7)	5588(6)	241(7)
Cl1	6356.5(8)	7774.5(10)	1417.0(11)	162.7(10)
Cl2	3802.1(9)	9276.5(9)	2772.8(11)	164.7(10)
P1	5005.5(5)	5941.6(6)	4687.1(6)	75.2(4)
O1	5394.2(10)	5537.0(13)	4810.0(15)	84.3(9)
O2	4617.5(10)	5817.3(13)	5073.6(14)	76.3(9)
O3	5152.5(9)	6669.0(12)	4753.4(14)	76.8(10)
O4	4869.4(10)	5893.4(12)	3951.6(13)	85.3(11)
C1	5145.1(13)	6151.7(17)	3492.0(19)	73.1(14)
C2	5178.5(14)	6804.2(18)	3398(2)	80.0(15)
C3	5467.0(17)	7048.1(17)	2951(2)	91.5(17)
C4	5700.2(15)	6606.9(17)	2576(2)	78.5(14)
C5	5686.1(14)	5929.5(16)	2700.6(19)	77.0(13)
C6	5395.0(18)	5717.1(18)	3157(2)	93.7(17)
C7	4901.5(14)	7146.3(18)	4479.3(18)	77.7(15)
C8	4899.9(15)	7219.1(19)	3817.0(19)	81.9(15)
C9	4630.8(17)	7708(2)	3569.5(17)	81.1(14)
C10	4388.1(16)	8092(2)	3979.8(19)	88.4(16)
C11	4397.3(13)	7971.3(18)	4658.8(17)	78.0(14)
C12	4658.8(14)	7500(2)	4908.2(19)	80.7(15)
C13	5557.5(16)	7722.5(18)	2881(2)	89.5(17)
C14	5826.8(19)	7933(2)	2393(2)	104(2)
C15	6010.8(18)	7495.1(17)	1986(3)	116(2)
C16	5974.9(16)	6842.6(18)	2079(2)	85.9(17)
C17	4587.8(19)	7777(2)	2879.5(18)	102(2)
C18	4311.0(19)	8251(2)	2658(2)	114(2)
C19	4090(2)	8646(2)	3072(2)	126(3)
C20	4095(2)	8556(3)	3732(2)	123(2)
C21	5937.4(13)	5452.2(17)	2341.2(19)	84.8(12)
C22	5743.6(14)	4920(2)	2064(2)	91.6(11)
C23	5970.9(13)	4419(2)	1783(3)	104.0(12)
C24	6405.5(13)	4482(2)	1765(3)	111.9(18)
C25	6619.2(13)	4978(2)	2077(3)	104.0(12)
C26	6374.2(13)	5462(2)	2353(2)	91.6(11)
C27	5733.9(14)	3845(2)	1446(2)	111.8(16)
C28	5294.4(16)	3740(3)	1720(3)	125(2)
C29	5990(2)	3231(2)	1540(4)	168(4)
C30	5693(2)	4031(4)	736(2)	166(4)
C31	7089.9(18)	5061(3)	2096(3)	180(3)
C32	7233(2)	5200(6)	2785(4)	325(9)
C33	7197(3)	5631(4)	1661(6)	333(8)

Atom	x	y	z	U_{eq}
C34	7304(2)	4452(4)	1847(5)	214(5)
C35	4133.3(13)	8305(2)	5140(2)	103.0(19)
C36	4300.3(14)	8605(3)	5675(2)	97.0(18)
C37	4049.2(13)	8899(2)	6142.3(19)	91.5(16)
C38	3616.1(13)	8912(3)	6043(3)	115(2)
C39	3431.9(13)	8634.5(19)	5499(2)	129(2)
C40	3700.3(14)	8338(3)	5059(3)	116(2)
C41	4244.7(16)	9204(2)	6745(2)	111.9(19)
C42	4691.9(18)	8968(4)	6870(3)	152(3)
C43	4024(2)	9027(4)	7379(2)	146(3)
C44	4266(3)	9935(2)	6651(3)	162(3)
C45	2960.7(16)	8635(5)	5359(6)	174(3)
C46	2717(7)	8003(8)	5430(15)	281(9)
C47	2892(8)	8885(16)	4670(8)	235(8)
C48	2789(7)	9137(11)	5832(12)	228(11)
C45A	2957.4(17)	8648(6)	5386(9)	174(3)
C46A	2817(10)	7946(7)	5319(17)	281(9)
C47A	2817(9)	9023(16)	4788(12)	235(8)
C48A	2681(8)	8815(13)	5967(11)	228(11)
Cl1B	6345.9(10)	9204.4(11)	8429.8(11)	190.8(13)
Cl2B	3820.6(10)	7870.8(11)	9944.0(11)	183.7(12)
P1B	5001.0(6)	5943.2(6)	6579.2(6)	78.8(4)
O1B	5389.2(10)	5802.1(14)	6180.6(14)	83.1(10)
O2B	4607.6(9)	5568.6(14)	6456.5(14)	78.1(9)
O3B	5144.8(9)	5881.8(13)	7310.1(12)	85.9(11)
O4B	4872.8(10)	6679.0(12)	6520.8(12)	82.5(11)
C1B	5146.1(14)	7135(2)	6786.8(18)	83.2(16)
C2B	5171.9(15)	7235(2)	7440.6(19)	92.4(18)
C3B	5468.0(18)	7666(2)	7689.9(17)	91.8(17)
C4B	5712.7(17)	8028(2)	7253.3(18)	92.5(18)
C5B	5674.9(14)	7945(2)	6568.8(16)	80.0(15)
C6B	5391.2(15)	7482.2(19)	6353.9(18)	73.2(14)
C7B	4893.8(15)	6157.3(18)	7786.5(19)	89.9(18)
C8B	4891.1(15)	6818.3(19)	7855(2)	85.8(17)
C9B	4620(2)	7066.6(17)	8341(2)	103(2)
C10B	4376.0(18)	6659.5(19)	8726(2)	101(2)
C11B	4398.2(14)	5979.5(17)	8627(2)	85.2(16)
C12B	4657.4(14)	5730.2(18)	8151.7(19)	70.7(13)
C13B	5536(2)	7759(3)	8369.9(19)	112(2)
C14B	5822(2)	8224(3)	8583(2)	137(3)
C15B	5997(2)	8645(3)	8146.3(18)	136(3)
C16B	5975.0(17)	8538(2)	7491.8(19)	94.3(19)
C17B	4591(2)	7755.1(18)	8425(3)	132(3)
C18B	4310(3)	7978(2)	8895(4)	176(4)
C19B	4087(2)	7567(2)	9289(3)	143(3)
C20B	4105(2)	6906(2)	9223(3)	154(4)
C21B	5928.4(12)	8307.1(19)	6097.4(18)	81.8(15)
C22B	5739.1(14)	8596(2)	5566(2)	89.7(12)
C23B	5970.5(13)	8892(3)	5078(2)	108.7(14)
C24B	6404.1(13)	8916(3)	5157(2)	99.8(17)
C25B	6613.7(13)	8600(3)	5654(2)	108.7(14)
C26B	6365.0(13)	8313(2)	6126(2)	89.7(12)
C27B	5741.0(14)	9235(2)	4501(2)	93.0(16)
C28B	5301.4(17)	8970(3)	4380(3)	135(3)
C29B	6005(2)	9143(5)	3892(3)	228(6)
C30B	5703(2)	9943(2)	4691(4)	175(4)

Atom	x	y	z	U_{eq}
C31B	7083.5(18)	8594(2)	5754(2)	181(3)
C32B	7228(2)	7913(2)	5918(3)	215(4)
C33B	7178(3)	9047(3)	6316(3)	252(4)
C34B	7304(2)	8830(3)	5145(3)	237(4)
C35B	4127.9(14)	5500(2)	8952(2)	97.2(18)
C36B	4289.8(14)	4971(2)	9266(2)	88.8(15)
C37B	4034.0(14)	4518(2)	9572(2)	111.3(18)
C38B	3603.9(14)	4639(3)	9599(3)	138(3)
C39B	3425.2(13)	5187(3)	9323(3)	142(3)
C40B	3695.1(14)	5587(3)	8974(3)	117(2)
C41B	4222.9(15)	3912.1(18)	9879(2)	105.5(16)
C42B	4665.9(18)	3767(3)	9634(3)	146(3)
C43B	3990(3)	3286(2)	9715(5)	251(7)
C44B	4254(3)	4012(4)	10611(2)	184(4)
C45B	2951.6(15)	5291(6)	9247(7)	190(4)
C46B	2728(5)	4966(10)	8679(8)	193(6)
C47B	2867(7)	6016(6)	9209(13)	225(7)
C48B	2774(7)	5038(13)	9886(8)	281(12)
C45C	2946.6(15)	5248(8)	9352(9)	190(4)
C46C	2799(7)	5661(11)	8784(9)	193(6)
C47C	2890(8)	5629(12)	9980(9)	225(7)
C48C	2675(8)	4641(9)	9392(14)	281(12)

Table 10.3.10: Anisotropic Displacement Parameters (×10[4]) for *S*-**1**. The anisotropic displacement factor exponent takes the form: $-2\pi^2[h^2a^{*2} \times U_{11} + \ldots + 2hka^* \times b^* \times U_{12}]$

Atom	U_{11}	U_{22}	U_{33}	U_{23}	U_{13}	U_{12}
Rh1	97.1(3)	55.4(2)	60.5(2)	-1.0(2)	-0.8(2)	-12.5(3)
N1S	86(2)	70(2)	91(3)	-44(3)	-11(2)	-15(2)
C1S	86(2)	134(6)	162(7)	36(6)	-8(2)	-12(2)
C2S	86(2)	357(16)	280(14)	-100(15)	-23(4)	0(4)
Cl1	241(3)	122.7(15)	124.9(17)	7.8(13)	87.3(18)	-43.5(16)
Cl2	253(3)	102.3(14)	138.2(18)	-27.5(13)	-79.6(18)	72.3(16)
P1	102.4(9)	58.6(7)	64.6(7)	2.1(6)	6.5(8)	-2.6(7)
O1	116.4(18)	49.4(17)	87(2)	10.0(17)	15.8(19)	6.1(15)
O2	97.9(16)	54.7(18)	76(2)	16.4(16)	4.6(15)	-1.6(15)
O3	105(3)	60.9(10)	65(2)	-3.4(15)	6.3(18)	-5.9(12)
O4	143(3)	41.1(17)	71.5(11)	-8.1(15)	-8.3(14)	-16.3(18)
C1	107(4)	51(2)	61(3)	1(2)	-16(2)	2(2)
C2	116(4)	59(3)	66(3)	-7(2)	-1(3)	-14(2)
C3	145(5)	68(3)	62(3)	-6(3)	16(3)	2(3)
C4	108(4)	69(3)	59(3)	1(2)	-3(3)	2(3)
C5	119(4)	65(2)	46(3)	0(2)	-7(2)	-7(2)
C6	140(5)	59(3)	82(4)	0(3)	17(3)	-7(3)
C7	112(5)	58(3)	63(3)	-9(2)	-2(3)	-5(2)
C8	134(5)	44(3)	67(3)	-2(2)	-1(3)	-13(3)
C9	127(4)	47(3)	69(3)	-5(2)	-18(3)	-15(3)
C10	113(4)	67(3)	86(3)	-32(3)	-34(3)	-11(3)
C11	105(4)	43(3)	86(3)	-6(2)	-20(3)	-11(2)
C12	80(4)	72(3)	91(4)	5(3)	18(3)	-3(2)
C13	123(5)	69(3)	76(4)	1(3)	5(3)	-2(3)
C14	178(7)	71(4)	64(4)	-16(3)	13(3)	-20(4)
C15	140(6)	87(3)	120(6)	-3(4)	39(4)	-25(4)

Atom	U_{11}	U_{22}	U_{33}	U_{23}	U_{13}	U_{12}
C16	145(5)	71(3)	42(3)	5(3)	-11(3)	-18(3)
C17	175(6)	62(3)	68(3)	1(3)	-25(4)	22(4)
C18	194(7)	57(4)	90(4)	-19(3)	-58(4)	23(4)
C19	182(7)	98(5)	97(4)	-29(4)	-57(5)	48(4)
C20	150(6)	125(6)	95(4)	-3(4)	-42(4)	32(4)
C21	143(3)	74(2)	37(2)	10.3(18)	-3(2)	12(2)
C22	143(3)	78.3(19)	53(2)	4.2(17)	-5.5(19)	11.1(19)
C23	145(2)	83.3(18)	84(3)	-6.3(19)	2(2)	9.5(18)
C24	146(2)	86(3)	104(4)	-10(3)	-1(2)	7(2)
C25	145(2)	83.3(18)	84(3)	-6.3(19)	2(2)	9.5(18)
C26	143(3)	78.3(19)	53(2)	4.2(17)	-5.5(19)	11.1(19)
C27	173(4)	93(3)	69(3)	-10(2)	0(3)	0(3)
C28	178(4)	95(5)	101(5)	-19(4)	11(4)	-6(3)
C29	214(7)	83(3)	206(9)	-46(4)	-66(7)	7(4)
C30	239(9)	181(7)	79(3)	19(4)	-33(4)	-97(7)
C31	146(2)	195(6)	200(7)	-47(6)	4(3)	-3(3)
C32	142(7)	590(20)	245(8)	-190(10)	-38(5)	186(12)
C33	140(9)	351(12)	508(18)	168(14)	-39(10)	-50(9)
C34	106(6)	216(7)	321(14)	-104(8)	-15(7)	-11(5)
C35	133(4)	65(4)	111(4)	-26(3)	-10(4)	-1(3)
C36	122(4)	74(4)	95(4)	-29(3)	22(3)	-26(3)
C37	134(4)	65(3)	75(3)	4(3)	31(3)	0(3)
C38	125(4)	71(4)	150(6)	-14(4)	32(4)	22(4)
C39	134(5)	101(5)	152(7)	-17(5)	30(4)	3(4)
C40	122(4)	107(5)	120(5)	-26(4)	4(4)	-19(4)
C41	161(5)	69(3)	105(4)	-31(4)	24(4)	-5(4)
C42	192(6)	180(8)	84(5)	-39(5)	1(5)	59(6)
C43	193(8)	172(7)	72(4)	-31(5)	14(4)	-7(6)
C44	287(10)	88(4)	111(6)	-19(4)	-84(6)	-11(5)
C45	161(4)	180(5)	182(5)	-16(4)	4(4)	14(4)
C46	147(13)	260(12)	440(20)	-78(13)	69(14)	-33(10)
C47	118(10)	400(20)	188(9)	-27(12)	10(9)	115(11)
C48	94(12)	330(20)	265(15)	-135(18)	-23(10)	32(14)
C45A	161(4)	180(5)	182(5)	-16(4)	4(4)	14(4)
C46A	147(13)	260(12)	440(20)	-78(13)	69(14)	-33(10)
C47A	118(10)	400(20)	188(9)	-27(12)	10(9)	115(11)
C48A	94(12)	330(20)	265(15)	-135(18)	-23(10)	32(14)
Cl1B	334(4)	125.7(17)	113.2(16)	-3.3(14)	-68(2)	-110(2)
Cl2B	300(3)	139.0(18)	112.4(17)	11.2(15)	80(2)	54(2)
P1B	114.3(11)	62.0(8)	60.0(8)	-3.1(7)	0.3(10)	-16.5(9)
O1B	101(3)	75(2)	74(2)	-22.7(19)	6(2)	-17(2)
O2B	75(2)	89(2)	70(2)	-18(2)	8(2)	-6(2)
O3B	132(3)	69(2)	57(2)	-5.7(18)	1.9(19)	-11(2)
O4B	139(3)	67(2)	41.8(18)	-5.3(16)	-5.8(19)	-13.3(19)
C1B	112(5)	78(3)	60(3)	4(3)	-7(3)	-12(3)
C2B	144(5)	74(3)	60(3)	18(3)	-15(3)	-17(3)
C3B	136(5)	70(3)	70(3)	5(3)	9(3)	-17(3)
C4B	137(5)	78(4)	63(3)	-11(3)	0(3)	-28(3)
C5B	130(4)	54(3)	56(3)	-5(2)	-2(3)	-8(3)
C6B	108(4)	44(3)	68(3)	14(2)	-13(3)	6(2)
C7B	140(6)	70(3)	59(3)	3(3)	13(3)	5(3)
C8B	136(5)	67(3)	55(3)	3(2)	-9(3)	-10(3)
C9B	189(6)	63(3)	58(3)	0(3)	6(4)	9(4)
C10B	180(6)	70(3)	54(3)	3(3)	-16(3)	4(3)
C11B	119(5)	78(3)	58(3)	2(3)	9(3)	-5(3)
C12B	94(4)	63(3)	55(3)	1(2)	-11(3)	-1(3)

Atom	U_{11}	U_{22}	U_{33}	U_{23}	U_{13}	U_{12}
C13B	182(7)	78(4)	77(4)	4(3)	4(4)	-37(4)
C14B	239(9)	90(5)	82(5)	-14(4)	-15(5)	-53(5)
C15B	241(9)	80(5)	87(4)	-3(4)	-14(5)	-27(5)
C16B	155(6)	38(3)	90(4)	-5(3)	-14(4)	-14(3)
C17B	265(9)	68(3)	64(4)	5(3)	35(5)	-8(5)
C18B	319(13)	93(5)	116(7)	-7(5)	73(7)	38(6)
C19B	268(10)	89(4)	72(4)	22(4)	48(5)	71(5)
C20B	287(11)	105(4)	69(5)	22(4)	31(5)	55(6)
C21B	105(4)	55(3)	86(4)	-26(3)	8(3)	-22(3)
C22B	112(3)	56(2)	101(3)	-15(2)	-9(2)	-5(2)
C23B	104(3)	103(3)	119(4)	5(3)	6(2)	-3(2)
C24B	121(4)	97(4)	81(4)	-11(3)	10(3)	0(4)
C25B	104(3)	103(3)	119(4)	5(3)	6(2)	-3(2)
C26B	112(3)	56(2)	101(3)	-15(2)	-9(2)	-5(2)
C27B	118(4)	78(3)	82(4)	-16(3)	3(3)	-2(3)
C28B	163(6)	112(5)	131(6)	53(5)	-50(5)	-54(5)
C29B	225(11)	328(15)	131(7)	34(9)	55(7)	153(11)
C30B	227(9)	53(4)	246(10)	-19(5)	-54(8)	-10(5)
C31B	105(3)	240(6)	200(5)	28(5)	-7(3)	-11(3)
C32B	80(5)	245(6)	320(11)	56(6)	31(6)	-9(4)
C33B	218(7)	290(6)	248(6)	-9(5)	-40(5)	-39(5)
C34B	128(7)	348(11)	234(6)	86(7)	12(5)	8(7)
C35B	133(4)	79(4)	80(4)	13(3)	21(4)	-2(3)
C36B	139(4)	74(3)	53(3)	-2(3)	10(3)	6(3)
C37B	176(4)	102(3)	56(3)	0(2)	9(3)	-27(3)
C38B	178(4)	143(6)	93(5)	23(5)	15(4)	-19(3)
C39B	126(5)	187(8)	114(6)	52(6)	18(5)	-12(4)
C40B	125(4)	126(5)	98(5)	17(4)	13(4)	-6(4)
C41B	160(4)	90(3)	66(3)	-1(2)	13(3)	-38(3)
C42B	185(5)	166(7)	85(5)	51(5)	41(4)	6(4)
C43B	234(9)	86(4)	434(18)	6(6)	-169(11)	-33(5)
C44B	285(11)	199(9)	69(3)	-17(4)	-11(4)	118(8)
C45B	131(5)	215(9)	223(11)	-4(8)	24(7)	-26(6)
C46B	169(8)	193(9)	217(9)	-9(8)	-5(7)	4(8)
C47B	218(10)	226(9)	231(11)	-5(8)	9(8)	16(8)
C48B	221(18)	340(30)	286(19)	40(20)	90(20)	-100(20)
C45C	131(5)	215(9)	223(11)	-4(8)	24(7)	-26(6)
C46C	169(8)	193(9)	217(9)	-9(8)	-5(7)	4(8)
C47C	218(10)	226(9)	231(11)	-5(8)	9(8)	16(8)
C48C	221(18)	340(30)	286(19)	40(20)	90(20)	-100(20)

Table 10.3.11: Bond Lengths in Å for *S*-**1**.

Atom	Atom	Length/Å
Rh1	Rh1[1]	2.5088(8)
Rh1	N1S	2.287(4)
Rh1	O1	2.059(3)
Rh1	O2[1]	2.036(3)
Rh1	O1B	2.038(3)
Rh1	O2B[1]	2.055(3)
N1S	C1S	1.057(6)
C1S	C2S	1.453(4)
C2S	H2SA	1.0900
C2S	H2SB	1.0900
C2S	H2SC	1.0900
Cl1	C15	1.716(3)
Cl2	C19	1.716(3)
P1	O1	1.520(3)
P1	O2	1.498(3)
P1	O3	1.589(3)
P1	O4	1.589(3)
O3	C7	1.396(4)

Atom	Atom	Length/Å
O4	C1	1.404(4)
C1	C2	1.374(5)
C1	C6	1.390(4)
C2	C3	1.401(5)
C2	C8	1.513(5)
C3	C4	1.414(5)
C3	C13	1.438(3)
C4	C5	1.432(4)
C4	C16	1.439(3)
C5	C6	1.398(5)
C5	C21	1.477(2)
C6	H6	1.0770
C7	C8	1.381(5)
C7	C12	1.389(4)
C8	C9	1.426(5)
C9	C10	1.401(5)
C9	C17	1.444(3)
C10	C11	1.430(4)
C10	C20	1.439(3)
C11	C12	1.387(3)
C11	C35	1.479(3)
C12	H12	1.0770
C13	H13	1.0770
C13	C14	1.398(4)
C14	H14	1.0770
C14	C15	1.374(3)
C15	C16	1.375(3)
C16	H16	1.0770
C17	H17	1.0770
C17	C18	1.402(4)
C18	H18	1.0770
C18	C19	1.381(3)
C19	C20	1.382(3)
C20	H20	1.0770
C21	C22	1.392(4)
C21	C26	1.395(3)
C22	H22	1.0770
C22	C23	1.396(2)
C23	C24	1.394(2)
C23	C27	1.574(6)
C24	H24	1.0770
C24	C25	1.396(2)
C25	C26	1.395(2)
C25	C31	1.512(6)
C26	H26	1.0770
C27	C28	1.529(3)
C27	C29	1.528(3)
C27	C30	1.527(3)
C28	H28A	1.0880
C28	H28B	1.0880
C28	H28C	1.0880
C29	H29A	1.0880
C29	H29B	1.0880
C29	H29C	1.0880
C30	H30A	1.0880
C30	H30B	1.0880

Atom	Atom	Length/Å
C30	H30C	1.0880
C31	C32	1.5278(19)
C31	C33	1.5282(19)
C31	C34	1.5275(19)
C32	H32A	1.0880
C32	H32B	1.0880
C32	H32C	1.0880
C33	H33A	1.0880
C33	H33B	1.0880
C33	H33C	1.0880
C34	H34A	1.0880
C34	H34B	1.0880
C34	H34C	1.0880
C35	C36	1.380(3)
C35	C40	1.394(3)
C36	H36	1.0770
C36	C37	1.397(2)
C37	C38	1.398(2)
C37	C41	1.535(3)
C38	H38	1.0770
C38	C39	1.395(2)
C39	C40	1.395(2)
C39	C45	1.532(3)
C39	C45A	1.533(3)
C40	H40	1.0770
C41	C42	1.532(3)
C41	C43	1.537(3)
C41	C44	1.532(3)
C42	H42A	1.0880
C42	H42B	1.0880
C42	H42C	1.0880
C43	H43A	1.0880
C43	H43B	1.0880
C43	H43C	1.0880
C44	H44A	1.0880
C44	H44B	1.0880
C44	H44C	1.0880
C45	C46	1.534(2)
C45	C47	1.534(2)
C45	C48	1.534(2)
C46	H46A	1.0880
C46	H46B	1.0880
C46	H46C	1.0880
C47	H47A	1.0900
C47	H47B	1.0900
C47	H47C	1.0900
C48	H48A	1.0880
C48	H48B	1.0880
C48	H48C	1.0880
C45A	C46A	1.533(3)
C45A	C47A	1.531(3)
C45A	C48A	1.532(3)
C46A	H46D	1.0900
C46A	H46E	1.0900
C46A	H46F	1.0900
C47A	H47D	1.0900

Atom	Atom	Length/Å
C47A	H47E	1.0900
C47A	H47F	1.0900
C48A	H48D	1.0900
C48A	H48E	1.0900
C48A	H48F	1.0900
Cl1B	C15B	1.714(4)
Cl2B	C19B	1.722(4)
P1B	O1B	1.518(3)
P1B	O2B	1.499(3)
P1B	O3B	1.588(3)
P1B	O4B	1.588(3)
O3B	C7B	1.395(4)
O4B	C1B	1.401(4)
C1B	C2B	1.374(5)
C1B	C6B	1.392(4)
C2B	C3B	1.401(5)
C2B	C8B	1.513(5)
C3B	C4B	1.412(5)
C3B	C13B	1.439(3)
C4B	C5B	1.435(4)
C4B	C16B	1.439(3)
C5B	C6B	1.394(5)
C5B	C21B	1.475(3)
C6B	H6B	1.0770
C7B	C8B	1.381(5)
C7B	C12B	1.389(4)
C8B	C9B	1.425(5)
C9B	C10B	1.400(6)
C9B	C17B	1.444(3)
C10B	C11B	1.430(4)
C10B	C20B	1.439(3)
C11B	C12B	1.388(3)
C11B	C35B	1.480(3)
C12B	H12B	1.0770
C13B	H13B	1.0770
C13B	C14B	1.399(4)
C14B	H14B	1.0770
C14B	C15B	1.377(3)
C15B	C16B	1.377(3)
C16B	H16B	1.0770
C17B	H17B	1.0770
C17B	C18B	1.401(4)
C18B	H18B	1.0770
C18B	C19B	1.381(3)
C19B	C20B	1.380(3)
C20B	H20B	1.0770
C21B	C22B	1.392(3)
C21B	C26B	1.395(3)
C22B	H22B	1.0770
C22B	C23B	1.395(2)
C23B	C24B	1.394(2)
C23B	C27B	1.574(6)
C24B	H24B	1.0770
C24B	C25B	1.394(2)
C25B	C26B	1.394(2)
C25B	C31B	1.514(6)

Atom	Atom	Length/Å
C26B	H26B	1.0770
C27B	C28B	1.528(3)
C27B	C29B	1.529(3)
C27B	C30B	1.529(3)
C28B	H28D	1.0880
C28B	H28E	1.0880
C28B	H28F	1.0880
C29B	H29D	1.0880
C29B	H29E	1.0880
C29B	H29F	1.0880
C30B	H30D	1.0880
C30B	H30E	1.0880
C30B	H30F	1.0880
C31B	C32B	1.527(2)
C31B	C33B	1.527(2)
C31B	C34B	1.526(2)
C32B	H32D	1.0880
C32B	H32E	1.0880
C32B	H32F	1.0880
C32B	C33B	2.501(4)
C32B	C34B	2.501(4)
C33B	H33D	1.0880
C33B	H33E	1.0880
C33B	H33F	1.0880
C33B	C34B	2.501(4)
C34B	H34D	1.0880
C34B	H34E	1.0880
C34B	H34F	1.0880
C35B	C36B	1.378(4)
C35B	C40B	1.394(4)
C36B	H36B	1.0770
C36B	C37B	1.399(2)
C37B	C38B	1.3968(17)
C37B	C41B	1.534(2)
C38B	H38B	1.0770
C38B	C39B	1.3974(17)
C39B	C40B	1.398(2)
C39B	C45B	1.535(2)
C39B	C45C	1.534(2)
C40B	H40B	1.0770
C41B	C42B	1.533(3)
C41B	C43B	1.536(3)
C41B	C44B	1.533(3)
C42B	H42D	1.0880
C42B	H42E	1.0880
C42B	H42F	1.0880
C43B	H43D	1.0880
C43B	H43E	1.0880
C43B	H43F	1.0880
C44B	H44D	1.0880
C44B	H44E	1.0880
C44B	H44F	1.0880
C45B	C46B	1.533(2)
C45B	C47B	1.533(2)
C45B	C48B	1.533(2)
C46B	H46G	1.0900

Atom	Atom	Length/Å
C46B	H46H	1.0900
C46B	H46I	1.0900
C47B	H47G	1.0900
C47B	H47H	1.0900
C47B	H47I	1.0900
C48B	H48G	1.0900
C48B	H48H	1.0900
C48B	H48I	1.0900
C45C	C46C	1.533(3)
C45C	C47C	1.533(3)
C45C	C48C	1.533(3)
C46C	H46J	1.0900
C46C	H46K	1.0900
C46C	H46L	1.0900
C47C	H47J	1.0900
C47C	H47K	1.0900
C47C	H47L	1.0900
C48C	H48J	1.0900
C48C	H48K	1.0900
C48C	H48L	1.0900

----
[1]1-x,1-y,+z

Table 10.3.12: Bond Angles in ° for *S*-**1**.

Atom	Atom	Atom	Angle/°
N1S	Rh1	Rh1[1]	178.52(10)
O1	Rh1	Rh1[1]	89.37(9)
O1	Rh1	N1S	90.40(15)
O2[1]	Rh1	Rh1[1]	90.17(9)
O2[1]	Rh1	N1S	88.37(13)
O2[1]	Rh1	O1	88.86(12)
O2[1]	Rh1	O1B	178.21(13)
O2[1]	Rh1	O2B[1]	90.81(10)
O1B	Rh1	Rh1[1]	88.55(9)
O1B	Rh1	N1S	92.91(13)
O1B	Rh1	O1	89.88(10)
O1B	Rh1	O2B[1]	90.45(12)
O2B[1]	Rh1	Rh1[1]	90.70(9)
O2B[1]	Rh1	N1S	89.51(15)
O2B[1]	Rh1	O1	179.67(13)
C1S	N1S	Rh1	177.7(6)
N1S	C1S	C2S	174.3(10)
C1S	C2S	H2SA	109.5
C1S	C2S	H2SB	109.5
C1S	C2S	H2SC	109.5
H2SA	C2S	H2SB	109.5
H2SA	C2S	H2SC	109.5
H2SB	C2S	H2SC	109.5
O1	P1	O3	105.72(17)
O1	P1	O4	110.44(17)
O2	P1	O1	119.33(16)
O2	P1	O3	111.23(16)
O2	P1	O4	106.04(18)
O4	P1	O3	102.94(15)
P1	O1	Rh1	116.44(18)
P1	O2	Rh1[1]	117.46(18)
C7	O3	P1	118.1(3)
C1	O4	P1	117.1(2)
C2	C1	O4	121.5(3)
C2	C1	C6	121.7(3)
C6	C1	O4	116.7(3)
C1	C2	C3	120.1(3)
C1	C2	C8	115.8(3)
C3	C2	C8	124.1(3)
C2	C3	C4	118.4(3)
C2	C3	C13	123.4(4)
C4	C3	C13	118.1(4)
C3	C4	C5	121.4(3)
C3	C4	C16	119.6(3)
C5	C4	C16	118.9(3)
C4	C5	C21	123.5(3)
C6	C5	C4	117.0(3)
C6	C5	C21	119.4(3)
C1	C6	C5	120.9(3)
C1	C6	H6	119.5
C5	C6	H6	119.5
C8	C7	O3	119.0(3)
C8	C7	C12	125.2(4)
C12	C7	O3	115.8(3)
C7	C8	C2	120.4(4)
C7	C8	C9	116.0(4)
C9	C8	C2	123.6(4)
C8	C9	C17	119.0(4)
C10	C9	C8	121.4(3)
C10	C9	C17	119.5(4)
C9	C10	C11	119.1(3)
C9	C10	C20	121.7(4)
C11	C10	C20	118.8(4)
C10	C11	C35	124.7(3)
C12	C11	C10	120.2(3)
C12	C11	C35	115.0(3)
C7	C12	H12	121.0
C11	C12	C7	118.1(3)
C11	C12	H12	121.0
C3	C13	H13	120.0
C14	C13	C3	120.1(4)
C14	C13	H13	120.0
C13	C14	H14	120.0
C15	C14	C13	120.0(4)
C15	C14	H14	120.0
C14	C15	Cl1	118.2(3)
C14	C15	C16	122.1(3)
C16	C15	Cl1	119.0(3)

Atom	Atom	Atom	Angle/°
C4	C16	H16	120.4
C15	C16	C4	119.2(3)
C15	C16	H16	120.4
C9	C17	H17	121.5
C18	C17	C9	117.0(4)
C18	C17	H17	121.5
C17	C18	H18	118.8
C19	C18	C17	122.5(4)
C19	C18	H18	118.8
C18	C19	Cl2	120.2(3)
C18	C19	C20	122.0(4)
C20	C19	Cl2	117.8(3)
C10	C20	H20	121.6
C19	C20	C10	116.8(4)
C19	C20	H20	121.6
C22	C21	C5	120.1(3)
C22	C21	C26	117.5(3)
C26	C21	C5	121.6(3)
C21	C22	H22	118.8
C21	C22	C23	122.3(4)
C23	C22	H22	118.8
C22	C23	C27	120.0(3)
C24	C23	C22	117.2(3)
C24	C23	C27	122.5(3)
C23	C24	H24	118.5
C23	C24	C25	123.0(3)
C25	C24	H24	118.5
C24	C25	C31	125.5(3)
C26	C25	C24	116.6(3)
C26	C25	C31	117.7(3)
C21	C26	C25	122.9(4)
C21	C26	H26	118.5
C25	C26	H26	118.5
C28	C27	C23	112.6(3)
C29	C27	C23	108.6(4)
C29	C27	C28	108.9(4)
C30	C27	C23	106.1(4)
C30	C27	C28	108.4(3)
C30	C27	C29	112.3(4)
C27	C28	H28A	109.5
C27	C28	H28B	109.5
C27	C28	H28C	109.5
H28A	C28	H28B	109.5
H28A	C28	H28C	109.5
H28B	C28	H28C	109.5
C27	C29	H29A	109.5
C27	C29	H29B	109.5
C27	C29	H29C	109.5
H29A	C29	H29B	109.5
H29A	C29	H29C	109.5
H29B	C29	H29C	109.5
C27	C30	H30A	109.5
C27	C30	H30B	109.5
C27	C30	H30C	109.5
H30A	C30	H30B	109.5
H30A	C30	H30C	109.5

Atom	Atom	Atom	Angle/°
H30B	C30	H30C	109.5
C25	C31	C32	109.9(4)
C25	C31	C33	107.2(5)
C25	C31	C34	110.0(4)
C32	C31	C33	109.8(3)
C34	C31	C32	109.8(3)
C34	C31	C33	110.0(3)
C31	C32	H32A	109.5
C31	C32	H32B	109.5
C31	C32	H32C	109.5
H32A	C32	H32B	109.5
H32A	C32	H32C	109.5
H32B	C32	H32C	109.5
C31	C33	H33A	109.5
C31	C33	H33B	109.5
C31	C33	H33C	109.5
H33A	C33	H33B	109.5
H33A	C33	H33C	109.5
H33B	C33	H33C	109.5
C31	C34	H34A	109.5
C31	C34	H34B	109.5
C31	C34	H34C	109.5
H34A	C34	H34B	109.5
H34A	C34	H34C	109.5
H34B	C34	H34C	109.5
C36	C35	C11	122.3(3)
C36	C35	C40	117.3(3)
C40	C35	C11	120.4(3)
C35	C36	H36	118.9
C35	C36	C37	122.2(3)
C37	C36	H36	118.9
C36	C37	C38	118.3(3)
C36	C37	C41	120.8(3)
C38	C37	C41	121.0(3)
C37	C38	H38	119.1
C39	C38	C37	121.9(3)
C39	C38	H38	119.1
C38	C39	C40	116.9(4)
C38	C39	C45	124.5(6)
C38	C39	C45A	122.2(8)
C40	C39	C45	118.6(6)
C40	C39	C45A	121.0(9)
C35	C40	C39	123.4(4)
C35	C40	H40	118.3
C39	C40	H40	118.3
C37	C41	C43	114.3(4)
C42	C41	C37	112.6(4)
C42	C41	C43	101.9(4)
C44	C41	C37	109.0(4)
C44	C41	C42	107.3(4)
C44	C41	C43	111.5(5)
C41	C42	H42A	109.5
C41	C42	H42B	109.5
C41	C42	H42C	109.5
H42A	C42	H42B	109.5
H42A	C42	H42C	109.5

Atom	Atom	Atom	Angle/°
H42B	C42	H42C	109.5
C41	C43	H43A	109.5
C41	C43	H43B	109.5
C41	C43	H43C	109.5
H43A	C43	H43B	109.5
H43A	C43	H43C	109.5
H43B	C43	H43C	109.5
C41	C44	H44A	109.5
C41	C44	H44B	109.5
C41	C44	H44C	109.5
H44A	C44	H44B	109.5
H44A	C44	H44C	109.5
H44B	C44	H44C	109.5
C39	C45	C46	118.5(10)
C39	C45	C47	108.5(11)
C39	C45	C48	103.3(11)
C47	C45	C46	108.0(7)
C48	C45	C46	109.8(7)
C48	C45	C47	108.3(8)
C45	C46	H46A	109.5
C45	C46	H46B	109.5
C45	C46	H46C	109.5
H46A	C46	H46B	109.5
H46A	C46	H46C	109.5
H46B	C46	H46C	109.5
C45	C47	H47A	109.5
C45	C47	H47B	109.5
C45	C47	H47C	109.5
H47A	C47	H47B	109.5
H47A	C47	H47C	109.5
H47B	C47	H47C	109.5
C45	C48	H48A	109.5
C45	C48	H48B	109.5
C45	C48	H48C	109.5
H48A	C48	H48B	109.5
H48A	C48	H48C	109.5
H48B	C48	H48C	109.5
C39	C45A	C46A	106.6(15)
C47A	C45A	C39	115.0(13)
C47A	C45A	C46A	109.0(8)
C47A	C45A	C48A	110.6(8)
C48A	C45A	C39	116.9(15)
C48A	C45A	C46A	96.8(4)
C45A	C46A	H46D	109.5
C45A	C46A	H46E	109.5
C45A	C46A	H46F	109.5
H46D	C46A	H46E	109.5
H46D	C46A	H46F	109.5
H46E	C46A	H46F	109.5
C45A	C47A	H47D	109.5
C45A	C47A	H47E	109.5
C45A	C47A	H47F	109.5
H47D	C47A	H47E	109.5
H47D	C47A	H47F	109.5
H47E	C47A	H47F	109.5
C45A	C48A	H48D	109.5

Atom	Atom	Atom	Angle/°
C45A	C48A	H48E	109.5
C45A	C48A	H48F	109.5
H48D	C48A	H48E	109.5
H48D	C48A	H48F	109.5
H48E	C48A	H48F	109.5
O1B	P1B	O3B	105.54(18)
O1B	P1B	O4B	110.79(17)
O2B	P1B	O1B	119.41(16)
O2B	P1B	O3B	111.23(16)
O2B	P1B	O4B	105.74(18)
O4B	P1B	O3B	102.98(15)
P1B	O1B	Rh1	117.86(18)
P1B	O2B	Rh1[1]	115.62(17)
C7B	O3B	P1B	118.5(3)
C1B	O4B	P1B	117.5(3)
C2B	C1B	O4B	121.8(3)
C2B	C1B	C6B	121.6(4)
C6B	C1B	O4B	116.5(3)
C1B	C2B	C3B	120.0(4)
C1B	C2B	C8B	116.0(3)
C3B	C2B	C8B	123.8(3)
C2B	C3B	C4B	118.5(3)
C2B	C3B	C13B	123.3(4)
C4B	C3B	C13B	118.2(4)
C3B	C4B	C5B	121.6(3)
C3B	C4B	C16B	119.6(3)
C5B	C4B	C16B	118.5(3)
C4B	C5B	C21B	123.2(3)
C6B	C5B	C4B	117.0(3)
C6B	C5B	C21B	119.8(3)
C1B	C6B	C5B	121.1(3)
C1B	C6B	H6B	119.5
C5B	C6B	H6B	119.5
C8B	C7B	O3B	119.0(3)
C8B	C7B	C12B	125.2(3)
C12B	C7B	O3B	115.8(3)
C7B	C8B	C2B	120.5(4)
C7B	C8B	C9B	115.9(4)
C9B	C8B	C2B	123.6(4)
C8B	C9B	C17B	118.9(4)
C10B	C9B	C8B	121.5(3)
C10B	C9B	C17B	119.6(4)
C9B	C10B	C11B	119.2(3)
C9B	C10B	C20B	121.8(4)
C11B	C10B	C20B	119.0(4)
C10B	C11B	C35B	124.8(3)
C12B	C11B	C10B	120.0(3)
C12B	C11B	C35B	114.8(3)
C7B	C12B	H12B	120.9
C11B	C12B	C7B	118.2(3)
C11B	C12B	H12B	120.9
C3B	C13B	H13B	120.0
C14B	C13B	C3B	120.0(4)
C14B	C13B	H13B	120.0
C13B	C14B	H14B	120.1
C15B	C14B	C13B	119.7(4)

Atom	Atom	Atom	Angle/°
C15B	C14B	H14B	120.1
C14B	C15B	Cl1B	118.0(3)
C16B	C15B	Cl1B	118.7(3)
C16B	C15B	C14B	121.6(4)
C4B	C16B	H16B	120.4
C15B	C16B	C4B	119.2(3)
C15B	C16B	H16B	120.4
C9B	C17B	H17B	121.6
C18B	C17B	C9B	116.9(4)
C18B	C17B	H17B	121.6
C17B	C18B	H18B	118.8
C19B	C18B	C17B	122.5(4)
C19B	C18B	H18B	118.8
C18B	C19B	Cl2B	119.6(3)
C20B	C19B	Cl2B	117.7(3)
C20B	C19B	C18B	122.3(4)
C10B	C20B	H20B	121.6
C19B	C20B	C10B	116.8(4)
C19B	C20B	H20B	121.6
C22B	C21B	C5B	120.4(3)
C22B	C21B	C26B	117.6(3)
C26B	C21B	C5B	121.6(3)
C21B	C22B	H22B	118.9
C21B	C22B	C23B	122.3(4)
C23B	C22B	H22B	118.9
C22B	C23B	C27B	120.3(3)
C24B	C23B	C22B	117.2(3)
C24B	C23B	C27B	122.2(3)
C23B	C24B	H24B	118.5
C25B	C24B	C23B	123.0(3)
C25B	C24B	H24B	118.5
C24B	C25B	C26B	116.6(3)
C24B	C25B	C31B	125.5(3)
C26B	C25B	C31B	117.7(3)
C21B	C26B	H26B	118.6
C25B	C26B	C21B	122.8(4)
C25B	C26B	H26B	118.6
C28B	C27B	C23B	112.9(3)
C28B	C27B	C29B	109.0(4)
C28B	C27B	C30B	108.4(3)
C29B	C27B	C23B	108.4(4)
C30B	C27B	C23B	106.0(4)
C30B	C27B	C29B	112.1(4)
C27B	C28B	H28D	109.5
C27B	C28B	H28E	109.5
C27B	C28B	H28F	109.5
H28D	C28B	H28E	109.5
H28D	C28B	H28F	109.5
H28E	C28B	H28F	109.5
C27B	C29B	H29D	109.5
C27B	C29B	H29E	109.5
C27B	C29B	H29F	109.5
H29D	C29B	H29E	109.5
H29D	C29B	H29F	109.5
H29E	C29B	H29F	109.5
C27B	C30B	H30D	109.5
C27B	C30B	H30E	109.5
C27B	C30B	H30F	109.5
H30D	C30B	H30E	109.5
H30D	C30B	H30F	109.5
H30E	C30B	H30F	109.5
C25B	C31B	C32B	109.8(4)
C25B	C31B	C33B	107.1(5)
C25B	C31B	C34B	109.9(4)
C32B	C31B	C33B	110.0(3)
C34B	C31B	C32B	110.0(3)
C34B	C31B	C33B	110.0(3)
C31B	C32B	H32D	109.5
C31B	C32B	H32E	109.5
C31B	C32B	H32F	109.5
C31B	C32B	C33B	35.02(14)
C31B	C32B	C34B	34.98(14)
H32D	C32B	H32E	109.5
H32D	C32B	H32F	109.5
H32E	C32B	H32F	109.5
C33B	C32B	H32D	76.3
C33B	C32B	H32E	111.7
C33B	C32B	H32F	133.3
C34B	C32B	H32D	133.9
C34B	C32B	H32E	76.6
C34B	C32B	H32F	110.9
C34B	C32B	C33B	60.001(6)
C31B	C33B	C32B	35.01(14)
C31B	C33B	H33D	109.5
C31B	C33B	H33E	109.5
C31B	C33B	H33F	109.5
C31B	C33B	C34B	34.98(14)
C32B	C33B	H33D	123.7
C32B	C33B	H33E	122.2
C32B	C33B	H33F	74.5
H33D	C33B	H33E	109.5
H33D	C33B	H33F	109.5
H33E	C33B	H33F	109.5
C34B	C33B	C32B	59.999(6)
C34B	C33B	H33D	121.4
C34B	C33B	H33E	74.5
C34B	C33B	H33F	124.4
C31B	C34B	C32B	35.00(14)
C31B	C34B	C33B	35.01(14)
C31B	C34B	H34D	109.5
C31B	C34B	H34E	109.5
C31B	C34B	H34F	109.5
C32B	C34B	H34D	86.5
C32B	C34B	H34E	94.1
C32B	C34B	H34F	143.9
C33B	C34B	C32B	60.000(7)
C33B	C34B	H34D	144.1
C33B	C34B	H34E	87.1
C33B	C34B	H34F	93.4
H34D	C34B	H34E	109.5
H34D	C34B	H34F	109.5
H34E	C34B	H34F	109.5

Atom	Atom	Atom	Angle/°
C36B	C35B	C11B	122.2(4)
C36B	C35B	C40B	117.3(3)
C40B	C35B	C11B	120.4(4)
C35B	C36B	H36B	118.9
C35B	C36B	C37B	122.2(3)
C37B	C36B	H36B	118.9
C36B	C37B	C41B	120.8(3)
C38B	C37B	C36B	118.1(3)
C38B	C37B	C41B	121.1(3)
C37B	C38B	H38B	119.0
C37B	C38B	C39B	122.1(3)
C39B	C38B	H38B	119.0
C38B	C39B	C40B	116.4(4)
C38B	C39B	C45B	123.9(6)
C38B	C39B	C45C	117.2(7)
C40B	C39B	C45B	118.1(6)
C40B	C39B	C45C	125.8(7)
C35B	C40B	C39B	123.4(4)
C35B	C40B	H40B	118.3
C39B	C40B	H40B	118.3
C37B	C41B	C43B	114.4(4)
C42B	C41B	C37B	112.7(4)
C42B	C41B	C43B	101.9(4)
C42B	C41B	C44B	107.2(4)
C44B	C41B	C37B	108.9(3)
C44B	C41B	C43B	111.4(5)
C41B	C42B	H42D	109.5
C41B	C42B	H42E	109.5
C41B	C42B	H42F	109.5
H42D	C42B	H42E	109.5
H42D	C42B	H42F	109.5
H42E	C42B	H42F	109.5
C41B	C43B	H43D	109.5
C41B	C43B	H43E	109.5
C41B	C43B	H43F	109.5
H43D	C43B	H43E	109.5
H43D	C43B	H43F	109.5
H43E	C43B	H43F	109.5
C41B	C44B	H44D	109.5
C41B	C44B	H44E	109.5
C41B	C44B	H44F	109.5
H44D	C44B	H44E	109.5
H44D	C44B	H44F	109.5
H44E	C44B	H44F	109.5
C46B	C45B	C39B	118.4(10)
C47B	C45B	C39B	108.5(11)
C47B	C45B	C46B	108.1(7)
C47B	C45B	C48B	108.4(7)
C48B	C45B	C39B	103.2(11)
C48B	C45B	C46B	109.9(7)
C45B	C46B	H46G	109.5
C45B	C46B	H46H	109.5
C45B	C46B	H46I	109.5
H46G	C46B	H46H	109.5
H46G	C46B	H46I	109.5
H46H	C46B	H46I	109.5
C45B	C47B	H47G	109.5
C45B	C47B	H47H	109.5
C45B	C47B	H47I	109.5
H47G	C47B	H47H	109.5
H47G	C47B	H47I	109.5
H47H	C47B	H47I	109.5
C45B	C48B	H48G	109.5
C45B	C48B	H48H	109.5
C45B	C48B	H48I	109.5
H48G	C48B	H48H	109.5
H48G	C48B	H48I	109.5
H48H	C48B	H48I	109.5
C46C	C45C	C39B	108.8(13)
C47C	C45C	C39B	101.1(14)
C47C	C45C	C46C	109.1(8)
C47C	C45C	C48C	108.2(8)
C48C	C45C	C39B	119.9(15)
C48C	C45C	C46C	109.1(8)
C45C	C46C	H46J	109.5
C45C	C46C	H46K	109.5
C45C	C46C	H46L	109.5
H46J	C46C	H46K	109.5
H46J	C46C	H46L	109.5
H46K	C46C	H46L	109.5
C45C	C47C	H47J	109.5
C45C	C47C	H47K	109.5
C45C	C47C	H47L	109.5
H47J	C47C	H47K	109.5
H47J	C47C	H47L	109.5
H47K	C47C	H47L	109.5
C45C	C48C	H48J	109.5
C45C	C48C	H48K	109.5
C45C	C48C	H48L	109.5
H48J	C48C	H48K	109.5
H48J	C48C	H48L	109.5
H48K	C48C	H48L	109.5

––––
[1]1-x,1-y,+z

Table 10.3.13: Torsion Angles in ° for *S*-**1**.

Atom	Atom	Atom	Atom	Angle/°
Cl1	C15	C16	C4	177.1(5)
Cl2	C19	C20	C10	-170.4(5)
P1	O3	C7	C8	-74.1(5)

Atom	Atom	Atom	Atom	Angle/°
P1	O3	C7	C12	103.7(4)
P1	O4	C1	C2	-75.8(5)
P1	O4	C1	C6	101.5(4)
O1	P1	O2	Rh1[1]	-9.9(3)
O1	P1	O3	C7	160.8(3)
O1	P1	O4	C1	-65.5(3)
O2	P1	O1	Rh1	-13.1(3)
O2	P1	O3	C7	-68.2(3)
O2	P1	O4	C1	163.8(3)
O3	P1	O1	Rh1	113.02(19)
O3	P1	O2	Rh1[1]	-133.40(18)
O3	P1	O4	C1	46.9(3)
O3	C7	C8	C2	-2.3(6)
O3	C7	C8	C9	178.9(4)
O3	C7	C12	C11	-178.7(4)
O4	P1	O1	Rh1	-136.30(17)
O4	P1	O2	Rh1[1]	115.39(19)
O4	P1	O3	C7	44.9(3)
O4	C1	C2	C3	177.8(4)
O4	C1	C2	C8	-0.2(6)
O4	C1	C6	C5	-178.5(5)
C1	C2	C3	C4	3.9(8)
C1	C2	C3	C13	-172.1(5)
C1	C2	C8	C7	52.9(6)
C1	C2	C8	C9	-128.5(5)
C2	C1	C6	C5	-1.2(8)
C2	C3	C4	C5	-8.0(8)
C2	C3	C4	C16	175.3(5)
C2	C3	C13	C14	-175.4(6)
C2	C8	C9	C10	-178.0(5)
C2	C8	C9	C17	6.1(8)
C3	C2	C8	C7	-125.1(5)
C3	C2	C8	C9	53.6(7)
C3	C4	C5	C6	7.4(8)
C3	C4	C5	C21	-177.3(5)
C3	C4	C16	C15	1.1(8)
C3	C13	C14	C15	-1.1(10)
C4	C3	C13	C14	8.6(9)
C4	C5	C6	C1	-2.7(8)
C4	C5	C21	C22	-128.0(5)
C4	C5	C21	C26	62.2(6)
C5	C4	C16	C15	-175.7(5)
C5	C21	C22	C23	-172.7(5)
C5	C21	C26	C25	172.9(5)
C6	C1	C2	C3	0.7(8)
C6	C1	C2	C8	-177.4(5)
C6	C5	C21	C22	47.2(6)
C6	C5	C21	C26	-122.6(6)
C7	C8	C9	C10	0.7(7)
C7	C8	C9	C17	-175.1(5)
C8	C2	C3	C4	-178.2(5)
C8	C2	C3	C13	5.8(9)
C8	C7	C12	C11	-1.0(8)
C8	C9	C10	C11	-2.8(8)
C8	C9	C10	C20	-175.7(5)
C8	C9	C17	C18	178.3(5)

Atom	Atom	Atom	Atom	Angle/°
C9	C10	C11	C12	3.1(8)
C9	C10	C11	C35	-175.0(4)
C9	C10	C20	C19	-5.6(10)
C9	C17	C18	C19	0.7(10)
C10	C9	C17	C18	2.4(9)
C10	C11	C12	C7	-1.2(7)
C10	C11	C35	C36	-126.7(6)
C10	C11	C35	C40	51.9(7)
C11	C10	C20	C19	-178.4(6)
C11	C35	C36	C37	-177.5(5)
C11	C35	C40	C39	178.8(4)
C12	C7	C8	C2	-179.9(4)
C12	C7	C8	C9	1.3(7)
C12	C11	C35	C36	55.0(7)
C12	C11	C35	C40	-126.3(6)
C13	C3	C4	C5	168.2(5)
C13	C3	C4	C16	-8.5(8)
C13	C14	C15	Cl1	-177.2(5)
C13	C14	C15	C16	-6.9(11)
C14	C15	C16	C4	6.9(10)
C16	C4	C5	C6	-175.9(5)
C16	C4	C5	C21	-0.6(7)
C17	C9	C10	C11	173.0(5)
C17	C9	C10	C20	0.1(9)
C17	C18	C19	Cl2	172.6(6)
C17	C18	C19	C20	-6.6(11)
C18	C19	C20	C10	8.7(11)
C20	C10	C11	C12	176.2(5)
C20	C10	C11	C35	-2.0(8)
C21	C5	C6	C1	-178.2(5)
C21	C22	C23	C24	-2.5(8)
C21	C22	C23	C27	-177.0(4)
C22	C21	C26	C25	2.8(8)
C22	C23	C24	C25	7.5(10)
C22	C23	C27	C28	-26.2(6)
C22	C23	C27	C29	-146.9(6)
C22	C23	C27	C30	92.2(6)
C23	C24	C25	C26	-7.2(10)
C23	C24	C25	C31	177.8(6)
C24	C23	C27	C28	159.6(6)
C24	C23	C27	C29	38.8(7)
C24	C23	C27	C30	-82.1(7)
C24	C25	C26	C21	1.8(9)
C24	C25	C31	C32	-132.9(7)
C24	C25	C31	C33	107.8(7)
C24	C25	C31	C34	-11.8(8)
C26	C21	C22	C23	-2.5(7)
C26	C25	C31	C32	52.1(7)
C26	C25	C31	C33	-67.2(6)
C26	C25	C31	C34	173.2(6)
C27	C23	C24	C25	-178.1(5)
C31	C25	C26	C21	177.2(5)
C35	C11	C12	C7	177.1(4)
C35	C36	C37	C38	-3.0(9)
C35	C36	C37	C41	177.6(5)
C36	C35	C40	C39	-2.5(8)

Atom	Atom	Atom	Atom	Angle/°
C36	C37	C38	C39	0.9(8)
C36	C37	C41	C42	-18.1(7)
C36	C37	C41	C43	-133.7(6)
C36	C37	C41	C44	100.8(6)
C37	C38	C39	C40	0.4(6)
C37	C38	C39	C45	-179.6(6)
C37	C38	C39	C45A	-179.9(6)
C38	C37	C41	C42	162.5(6)
C38	C37	C41	C43	46.9(7)
C38	C37	C41	C44	-78.5(7)
C38	C39	C40	C35	0.5(6)
C38	C39	C45	C46	-107.5(14)
C38	C39	C45	C47	128.9(14)
C38	C39	C45	C48	14.2(12)
C38	C39	C45A	C46A	-122.6(14)
C38	C39	C45A	C47A	116.5(16)
C38	C39	C45A	C48A	-15.8(13)
C40	C35	C36	C37	3.8(9)
C40	C39	C45	C46	72.5(14)
C40	C39	C45	C47	-51.1(13)
C40	C39	C45	C48	-165.8(12)
C40	C39	C45A	C46A	57.1(14)
C40	C39	C45A	C47A	-63.8(16)
C40	C39	C45A	C48A	163.9(13)
C41	C37	C38	C39	-179.7(4)
C45	C39	C40	C35	-179.5(7)
C45A	C39	C40	C35	-179.3(7)
Cl1B	C15B	C16B	C4B	174.2(5)
Cl2B	C19B	C20B	C10B	-171.7(6)
P1B	O3B	C7B	C8B	-73.8(5)
P1B	O3B	C7B	C12B	105.1(4)
P1B	O4B	C1B	C2B	-75.5(5)
P1B	O4B	C1B	C6B	104.4(4)
O1B	P1B	O2B	Rh1[1]	-11.1(3)
O1B	P1B	O3B	C7B	162.1(3)
O1B	P1B	O4B	C1B	-66.8(3)
O2B	P1B	O1B	Rh1	-12.9(3)
O2B	P1B	O3B	C7B	-67.0(3)
O2B	P1B	O4B	C1B	162.5(3)
O3B	P1B	O1B	Rh1	113.15(19)
O3B	P1B	O2B	Rh1[1]	-134.44(17)
O3B	P1B	O4B	C1B	45.6(3)
O3B	C7B	C8B	C2B	-2.9(7)
O3B	C7B	C8B	C9B	178.6(5)
O3B	C7B	C12B	C11B	-179.2(4)
O4B	P1B	O1B	Rh1	-136.05(18)
O4B	P1B	O2B	Rh1[1]	114.47(18)
O4B	P1B	O3B	C7B	45.9(3)
O4B	C1B	C2B	C3B	175.9(5)
O4B	C1B	C2B	C8B	0.6(7)
O4B	C1B	C6B	C5B	-179.6(4)
C1B	C2B	C3B	C4B	4.9(9)
C1B	C2B	C3B	C13B	-175.1(6)
C1B	C2B	C8B	C7B	52.4(7)
C1B	C2B	C8B	C9B	-129.3(6)
C2B	C1B	C6B	C5B	0.2(8)

Atom	Atom	Atom	Atom	Angle/˚
C2B	C3B	C4B	C5B	-2.3(9)
C2B	C3B	C4B	C16B	171.1(6)
C2B	C3B	C13B	C14B	-176.5(7)
C2B	C8B	C9B	C10B	-177.9(5)
C2B	C8B	C9B	C17B	3.7(9)
C3B	C2B	C8B	C7B	-122.8(6)
C3B	C2B	C8B	C9B	55.6(8)
C3B	C4B	C5B	C6B	-1.2(9)
C3B	C4B	C5B	C21B	-179.2(5)
C3B	C4B	C16B	C15B	3.0(10)
C3B	C13B	C14B	C15B	8.1(12)
C4B	C3B	C13B	C14B	3.5(11)
C4B	C5B	C6B	C1B	2.3(8)
C4B	C5B	C21B	C22B	-132.8(6)
C4B	C5B	C21B	C26B	54.4(7)
C5B	C4B	C16B	C15B	176.6(6)
C5B	C21B	C22B	C23B	-174.0(5)
C5B	C21B	C26B	C25B	173.9(5)
C6B	C1B	C2B	C3B	-3.9(8)
C6B	C1B	C2B	C8B	-179.3(4)
C6B	C5B	C21B	C22B	49.3(6)
C6B	C5B	C21B	C26B	-123.5(5)
C7B	C8B	C9B	C10B	0.5(9)
C7B	C8B	C9B	C17B	-177.9(6)
C8B	C2B	C3B	C4B	179.9(5)
C8B	C2B	C3B	C13B	-0.1(9)
C8B	C7B	C12B	C11B	-0.4(8)
C8B	C9B	C10B	C11B	-0.2(10)
C8B	C9B	C10B	C20B	179.3(6)
C8B	C9B	C17B	C18B	178.0(7)
C9B	C10B	C11B	C12B	-0.4(9)
C9B	C10B	C11B	C35B	-173.6(5)
C9B	C10B	C20B	C19B	2.0(11)
C9B	C17B	C18B	C19B	3.4(14)
C10B	C9B	C17B	C18B	-0.4(11)
C10B	C11B	C12B	C7B	0.7(8)
C10B	C11B	C35B	C36B	-128.8(6)
C10B	C11B	C35B	C40B	48.4(8)
C11B	C10B	C20B	C19B	-178.4(7)
C11B	C35B	C36B	C37B	-180.0(5)
C11B	C35B	C40B	C39B	-173.7(6)
C12B	C7B	C8B	C2B	178.3(5)
C12B	C7B	C8B	C9B	-0.2(8)
C12B	C11B	C35B	C36B	57.7(7)
C12B	C11B	C35B	C40B	-125.0(6)
C13B	C3B	C4B	C5B	177.6(6)
C13B	C3B	C4B	C16B	-8.9(9)
C13B	C14B	C15B	Cl1B	-179.9(6)
C13B	C14B	C15B	C16B	-14.6(13)
C14B	C15B	C16B	C4B	9.0(11)
C16B	C4B	C5B	C6B	-174.7(5)
C16B	C4B	C5B	C21B	7.3(8)
C17B	C9B	C10B	C11B	178.2(6)
C17B	C9B	C10B	C20B	-2.3(10)
C17B	C18B	C19B	Cl2B	168.7(8)
C17B	C18B	C19B	C20B	-3.8(15)

Atom	Atom	Atom	Atom	Angle/˚
C18B	C19B	C20B	C10B	1.0(13)
C20B	C10B	C11B	C12B	-180.0(6)
C20B	C10B	C11B	C35B	6.9(9)
C21B	C5B	C6B	C1B	-179.6(4)
C21B	C22B	C23B	C24B	-3.1(9)
C21B	C22B	C23B	C27B	-177.7(4)
C22B	C21B	C26B	C25B	0.8(8)
C22B	C23B	C24B	C25B	7.4(9)
C22B	C23B	C27B	C28B	-24.7(7)
C22B	C23B	C27B	C29B	-145.6(6)
C22B	C23B	C27B	C30B	93.8(6)
C23B	C24B	C25B	C26B	-7.4(10)
C23B	C24B	C25B	C31B	178.2(6)
C24B	C23B	C27B	C28B	160.9(6)
C24B	C23B	C27B	C29B	40.1(7)
C24B	C23B	C27B	C30B	-80.5(6)
C24B	C25B	C26B	C21B	3.1(9)
C24B	C25B	C31B	C32B	-135.9(6)
C24B	C25B	C31B	C33B	104.8(6)
C24B	C25B	C31B	C34B	-14.7(7)
C25B	C31B	C32B	C33B	-117.5(6)
C25B	C31B	C32B	C34B	121.1(5)
C25B	C31B	C33B	C32B	119.2(5)
C25B	C31B	C33B	C34B	-119.4(5)
C25B	C31B	C34B	C32B	-121.0(5)
C25B	C31B	C34B	C33B	117.7(6)
C26B	C21B	C22B	C23B	-0.9(8)
C26B	C25B	C31B	C32B	49.8(6)
C26B	C25B	C31B	C33B	-69.6(6)
C26B	C25B	C31B	C34B	170.9(5)
C27B	C23B	C24B	C25B	-178.1(5)
C31B	C25B	C26B	C21B	178.0(5)
C32B	C31B	C33B	C34B	121.3(6)
C32B	C31B	C34B	C33B	-121.3(6)
C33B	C31B	C32B	C34B	-121.3(6)
C33B	C31B	C34B	C32B	121.3(6)
C34B	C31B	C32B	C33B	121.3(6)
C34B	C31B	C33B	C32B	-121.3(6)
C35B	C11B	C12B	C7B	174.5(4)
C35B	C36B	C37B	C38B	-5.0(9)
C35B	C36B	C37B	C41B	176.1(5)
C36B	C35B	C40B	C39B	3.6(10)
C36B	C37B	C38B	C39B	1.0(11)
C36B	C37B	C41B	C42B	-17.9(7)
C36B	C37B	C41B	C43B	-133.6(7)
C36B	C37B	C41B	C44B	101.0(6)
C37B	C38B	C39B	C40B	4.7(12)
C37B	C38B	C39B	C45B	170.1(8)
C37B	C38B	C39B	C45C	176.8(10)
C38B	C37B	C41B	C42B	163.2(6)
C38B	C37B	C41B	C43B	47.5(8)
C38B	C37B	C41B	C44B	-78.0(7)
C38B	C39B	C40B	C35B	-7.2(11)
C38B	C39B	C45B	C46B	-80.6(14)
C38B	C39B	C45B	C47B	155.8(12)
C38B	C39B	C45B	C48B	41.0(13)

Atom	Atom	Atom	Atom	Angle/°
C38B	C39B	C45C	C46C	-153.1(11)
C38B	C39B	C45C	C47C	92.2(12)
C38B	C39B	C45C	C48C	-26.6(16)
C40B	C35B	C36B	C37B	2.7(8)
C40B	C39B	C45B	C46B	84.5(13)
C40B	C39B	C45B	C47B	-39.0(13)
C40B	C39B	C45B	C48B	-153.8(12)
C40B	C39B	C45C	C46C	18.2(17)
C40B	C39B	C45C	C47C	-96.6(14)
C40B	C39B	C45C	C48C	144.7(14)
C41B	C37B	C38B	C39B	180.0(6)
C45B	C39B	C40B	C35B	-173.5(8)
C45C	C39B	C40B	C35B	-178.5(10)

----

[1]1-x,1-y,+z

Table 10.3.14: Hydrogen Fractional Atomic Coordinates (×10^4) and Equivalent Isotropic Displacement Parameters (Å^2×10^3) for *S*-**1**. U_{eq} is defined as 1/3 of the trace of the orthogonalised U_{ij}.

Atom	x	y	z	U_{eq}
H2SA	6996.82	4845.09	5088.14	361
H2SB	6981.94	4423.92	5835.47	361
H2SC	7038.85	5275.94	5827.44	361
H6	5363.91	5209.67	3251.49	112
H12	4673.22	7410.46	5419.46	97
H13	5417.72	8065.76	3205.62	107
H14	5890.03	8439.12	2336.42	125
H16	6150.32	6511.87	1783.92	103
H17	4760.57	7476.59	2549.76	122
H18	4269.41	8311.01	2145.99	136
H20	3888.7	8820.96	4046.94	148
H22	5406.66	4892.75	2066.6	110
H24	6584.42	4132.01	1499.07	134
H26	6529.24	5859.18	2585.52	110
H28A	5125.44	3400.01	1417.71	187
H28B	5126.96	4195.92	1730.73	187
H28C	5318.06	3549.15	2207.84	187
H29A	6068.45	3176.4	2048.17	251
H29B	6276.92	3261.61	1258.05	251
H29C	5807.72	2818.32	1381.38	251
H30A	5606.43	3608.15	455.99	249
H30B	5990.22	4218.16	563.03	249
H30C	5451.63	4397.2	684.26	249
H32A	7552.89	5378.93	2778.55	488
H32B	7218.56	4759.43	3068.25	488
H32C	7028.1	5559.86	2999.45	488
H33A	7197.09	6071.42	1944.35	500
H33B	6965.02	5668.72	1278.05	500
H33C	7505.72	5558.96	1449.52	500
H34A	7624.52	4563.88	1707.49	321
H34B	7133.9	4269.44	1430.32	321
H34C	7306.19	4089.1	2225.02	321
H36	4635.39	8612.83	5735.31	116
H38	3419.03	9142.48	6395.19	138

Atom	x	y	z	U_{eq}
H40	3565.78	8123.9	4633.4	139
H42A	4683.25	8468.54	7028.18	228
H42B	4835.85	9262.23	7243.41	228
H42C	4874.2	9004.26	6428.39	228
H43A	3771.81	9367.95	7471.5	219
H43B	4248.01	9044.19	7774.22	219
H43C	3894.51	8544.12	7341.77	219
H44A	4435.61	10042.51	6208.86	242
H44B	4428.14	10151.7	7058.86	242
H44C	3950.33	10128.79	6619.74	242
H46A	2866.07	7631.28	5139.84	422
H46B	2396.38	8071.89	5268.26	422
H46C	2717.26	7856.14	5934.38	422
H47A	2557.87	8889.02	4562.34	353
H47B	3016.06	9372.49	4630.64	353
H47C	3053.5	8572.43	4329.5	353
H48A	2458.84	9221.36	5732.01	342
H48B	2961.8	9584.92	5777.38	342
H48C	2824.22	8960.79	6323.52	342
H46D	2915.52	7675.65	5743.41	422
H46E	2476.45	7927.3	5278	422
H46F	2957.97	7736.46	4889.42	422
H47D	3016.88	8897.66	4381.13	353
H47E	2492.92	8900.74	4675.1	353
H47F	2840.41	9537.83	4882.73	353
H48D	2786.25	8543.96	6386.99	342
H48E	2704.14	9328.95	6067.59	342
H48F	2356.64	8691.85	5859.96	342
H6B	5361.34	7391.39	5844.47	88
H12B	4674.77	5219.49	8067.72	85
H13B	5367.21	7471.49	8714.88	135
H14B	5903.74	8252.91	9085.82	164
H16B	6151.78	8833.06	7162.44	113
H17B	4775.2	8082.2	8139.36	159
H18B	4267.95	8488.82	8951.28	211
H20B	3925.5	6590.91	9528.59	185
H22B	5402.49	8590.75	5530.62	108
H24B	6585.44	9192.42	4816.83	120
H26B	6516.52	8085.93	6530.94	108
H28D	5310.57	8446.61	4386.22	203
H28E	5189.77	9134.53	3912.48	203
H28F	5090.86	9140.22	4755.92	203
H29D	6315.7	9342.81	3968.5	342
H29E	5855.23	9388.32	3489.08	342
H29F	6030.65	8631.98	3783.63	342
H30D	5416.66	10013.92	4966.87	263
H30E	5693.36	10238.62	4258.09	263
H30F	5971.55	10080.03	4983.26	263
H32D	7261.87	7866.31	6438.25	322
H32E	7528.26	7819.73	5687.12	322
H32F	6997.7	7567.73	5745.74	322
H33D	6887.79	9265.85	6482.87	378
H33E	7392.56	9420.85	6156.67	378
H33F	7319.87	8775.83	6708.01	378
H34D	7266.51	8474.93	4763.27	356
H34E	7635.63	8897.97	5244.68	356

Atom	x	y	z	U_{eq}
H34F	7166.79	9284.33	4992.73	356
H36B	4624.31	4903.7	9274.91	107
H38B	3403.54	4298.44	9840.99	166
H40B	3561.59	5981.62	8707.54	140
H42D	4881.33	4131.7	9812.92	218
H42E	4666.86	3772.08	9108.93	218
H42F	4763.84	3296.03	9805.03	218
H43D	4210.01	2885.51	9722.82	377
H43E	3851.98	3325.85	9237.16	377
H43F	3744.55	3202.71	10069.04	377
H44D	4541.76	3795.55	10788.7	276
H44E	3986.35	3786.6	10845.38	276
H44F	4254.75	4524.64	10717.29	276
H46G	2783.55	4449.13	8695.2	289
H46H	2392.31	5059.31	8710.58	289
H46I	2848.36	5160.08	8226.83	289
H47G	2991.04	6204.47	8757.28	337
H47H	2530.06	6101.72	9229.38	337
H47I	3018.58	6256.59	9613.3	337
H48G	2831.07	4522.48	9921.75	422
H48H	2926.07	5283.95	10286.69	422
H48I	2437.54	5129.08	9902.77	422
H46J	2837.74	5393.21	8335.71	289
H46K	2469.04	5783.06	8847.83	289
H46L	2983.76	6101.58	8762.85	289
H47J	3081.36	6063.57	9961.74	337
H47K	2561.84	5761.15	10035.87	337
H47L	2987.77	5333.73	10387.42	337
H48J	2709.91	4362.49	8950.18	422
H48K	2773.24	4351.27	9803.21	422
H48L	2347.3	4778.69	9451.66	422

Table 10.3.15: Atomic Occupancies for all atoms that are not fully occupied in *S*-**1**.

Atom	Occupancy	Atom	Occupancy	Atom	Occupancy
C45	0.5	C47A	0.5	H48G	0.5
C46	0.5	H47D	0.5	H48H	0.5
H46A	0.5	H47E	0.5	H48I	0.5
H46B	0.5	H47F	0.5	C45C	0.5
H46C	0.5	C48A	0.5	C46C	0.5
C47	0.5	H48D	0.5	H46J	0.5
H47A	0.5	H48E	0.5	H46K	0.5
H47B	0.5	H48F	0.5	H46L	0.5
H47C	0.5	C45B	0.5	C47C	0.5
C48	0.5	C46B	0.5	H47J	0.5
H48A	0.5	H46G	0.5	H47K	0.5
H48B	0.5	H46H	0.5	H47L	0.5
H48C	0.5	H46I	0.5	C48C	0.5
C45A	0.5	C47B	0.5	H48J	0.5
C46A	0.5	H47G	0.5	H48K	0.5
H46D	0.5	H47H	0.5	H48L	0.5
H46E	0.5	H47I	0.5		
H46F	0.5	C48B	0.5		

Table 10.3.16: Solvent masking (PLATON/SQUEEZE) information for *S*-**1**.

No	x	y	z	V	e	Content
1	-0.420	-0.853	-0.191	6313.3	1265.2	52C2,52H3,52N1

Citations

CrysAlisPro Software System, Rigaku Oxford Diffraction, (2022).

O.V. Dolomanov and L.J. Bourhis and R.J. Gildea and J.A.K. Howard and H. Puschmann, Olex2: A complete structure solution, refinement and analysis program, *J. Appl. Cryst.*, (2009), **42**, 339-341.

Sheldrick, G.M., Crystal structure refinement with ShelXL, *Acta Cryst.*, (2015), **C71**, 3-8.

Sheldrick, G.M., ShelXT-Integrated space-group and crystal-structure determination, *Acta Cryst.*, (2015), **A71**, 3-8.

10.4 X-Ray Crystallographic Data for 26e

R_1=3.45%

Crystal Data and Experimental

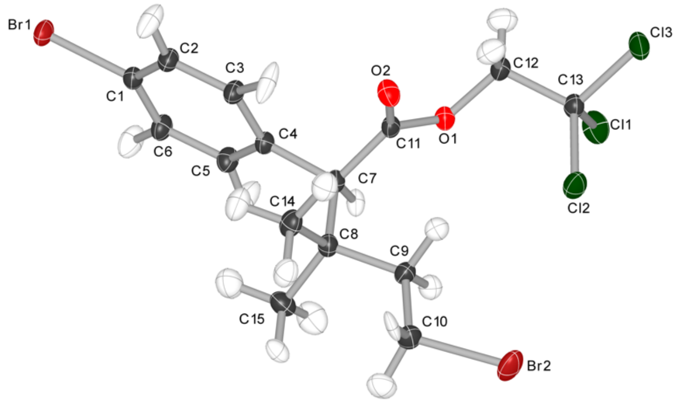

Experimental. Single colourless prism-shaped crystals of **26e** were recrystallized from a mixture of dichloromethane and hexane by solvent layering. A suitable crystal with dimensions 0.46 × 0.19 × 0.11 mm³ was selected and mounted on a XtaLAB Synergy, Dualflex, HyPix diffractometer. The crystal was kept at a steady T = 100.03(11) K during data collection. The structure was solved with the ShelXT (Sheldrick, 2015) solution program using dual methods and by using Olex2 1.5-alpha (Dolomanov et al., 2009) as the graphical interface. The model was refined with olex2.refine 1.5-alpha (Bourhis et al., 2015) using full matrix least squares minimisation on F^2.

Crystal Data. $C_{15}H_{17}Br_2Cl_3O_2$, M_r = 495.466, orthorhombic, $P2_12_12_1$ (No. 19), a = 9.07826(19) Å, b = 10.3751(2) Å, c = 19.1988(6) Å, $\alpha = \beta = \gamma =$ 90°, V = 1808.30(8) Å³, T = 100.03(11) K, Z = 4, Z' = 1, μ(Mo K_α) = 4.929, 66352 reflections measured, 9239 unique (R_{int} = 0.0712) which were used in all calculations. The final wR_2 was 0.0618 (all data) and R_1 was 0.0345 (I≥2 σ(I)).

**Compound**	**26e**
**CCDC number**	**2335091**
Formula	$C_{15}H_{17}Br_2Cl_3O_2$
$D_{calc.}$/ g cm^{-3}	1.820
μ/mm^{-1}	4.929
Formula Weight	495.466
Colour	colourless
Shape	prism-shaped
Size/mm³	0.46×0.19×0.11
T/K	100.03(11)
Crystal System	orthorhombic
Flack Parameter	0.006(3)
Hooft Parameter	0.006(3)
Space Group	$P2_12_12_1$
a/Å	9.07826(19)
b/Å	10.3751(2)
c/Å	19.1988(6)
α/°	90
β/°	90
γ/°	90
V/Å³	1808.30(8)
Z	4
Z'	1
Wavelength/Å	0.71073
Radiation type	Mo K_α
Θ_{min}/°	2.89
Θ_{max}/°	37.64
Measured Refl's.	66352
Indep't Refl's	9239
Refl's I≥2 σ(I)	7043
R_{int}	0.0712
Parameters	397
Restraints	324
Largest Peak	0.7792
Deepest Hole	-0.6151
GooF	1.0114
wR_2 (all data)	0.0618
wR_2	0.0563
R_1 (all data)	0.0599
R_1	0.0345

Structure Quality Indicators

**Reflections:**	d min (MoKα) 2Θ=75.3° 0.58	I/σ(I) 20.7	Rint m=7.09 7.12%	Full 50.5° 97% to 75.3° 96.3	
**Refinement:**	Shift 0.001	Max Peak 0.8	Min Peak -0.6	GooF 1.011	Hooft .006(3)

A colorless prism-shaped-shaped crystal with dimensions 0.46 × 0.19 × 0.11 mm^3 was mounted. Data were collected using a XtaLAB Synergy, Dualflex, HyPix diffractometer equipped with an Oxford Cryosystems low-temperature device operating at T = 100.03(11) K.

Data were measured using ω scans with Mo K_{α} radiation. The diffraction pattern was indexed and the total number of runs and images was based on the strategy calculation from the program CrysAlisPro system (CCD 42.79a 64-bit (release 16-12-2022)). The maximum resolution that was achieved was Θ = 37.64° (0.58 Å).

The unit cell was refined using CrysAlisPro 1.171.43.103a (Rigaku OD, 2023) on 8215 reflections, 12% of the observed reflections. Data reduction, scaling and absorption corrections were performed using CrysAlisPro 1.171.43.103a (Rigaku OD, 2023). The final completeness is 97.88 % out to 37.64° in Θ. An analytical, numeric absorption correction using a multifaceted crystal model based on expressions derived by R.C. Clark & J.S. Reid. (Clark, R. C. & Reid, J. S. (1995). Acta Cryst. A51, 887-897) was performed using CrysAlisPro 1.171.43.103a (Rigaku Oxford Diffraction, 2023). An empirical absorption correction using spherical harmonics, implemented in SCALE3 ABSPACK scaling algorithm was also applied. The absorption coefficient μ of this material is 4.929 mm^{-1} at this wavelength (λ = 0.71073Å) and the minimum and maximum transmissions are 0.197 and 0.678.

The structure was solved and the space group $P2_12_12_1$ (# 19) determined by the ShelXT (Sheldrick, 2015) structure solution program using dual methods and refined by full matrix least squares minimisation on $\boldsymbol{F}^2$ using version of olex2.refine 1.5-alpha (Bourhis et al., 2015). All atoms (including all hydrogen atoms) were refined anisotropically. Hydrogen atom positions were refined using the Hirshfeld model. Refinement was by using NoSpherA2, an implementation of non-spherical atom-form-factors (F. Kleemiss, H. Puschmann, O. Dolomanov, S.Grabowsky - https://doi.org/10.1039/D0SC05526C – 2020). NoSpherA2 implementation of HAR makes use of tailor-made aspherical atomic form factors calculated from a Hirshfeld-partitioned electron density (ED) not from spherical-atom form factors. The ED was calculated from a Gaussian basis set single determinant SCF wavefunction from DFT using selected functionals for a fragment of this crystal. This fragment was embedded in an electrostatic crystal field by employing cluster charges. The following options were used: SOFTWARE: ORCA PARTITIONING: NoSpherA2 INT ACCURACY: Normal METHOD: PBE BASIS SET: x2c-SVP CHARGE: 0 MULTIPLICITY: 1 SOLVATION: CH2CL2 RELATIVISTIC: DKH2 DATE: 2024-02-24_17-38-49

There is a single formula unit in the asymmetric unit, which is represented by the reported sum formula. In other words: Z is 4 and Z' is 1. The moiety formula is C15 H17 Br2 Cl3 O2.

The Flack parameter was refined to 0.006(3). Determination of absolute structure using Bayesian statistics on Bijvoet differences using the Olex2 results in 0.006(3). The chiral atoms in this structure are: C7(S). Note: Note: The Flack parameter is used to determine chirality of the crystal studied, the value should be near 0, a value of 1 means that the stereochemistry is wrong and the model should be inverted. A value of 0.5 means that the crystal consists of a racemic mixture of the two enantiomers.

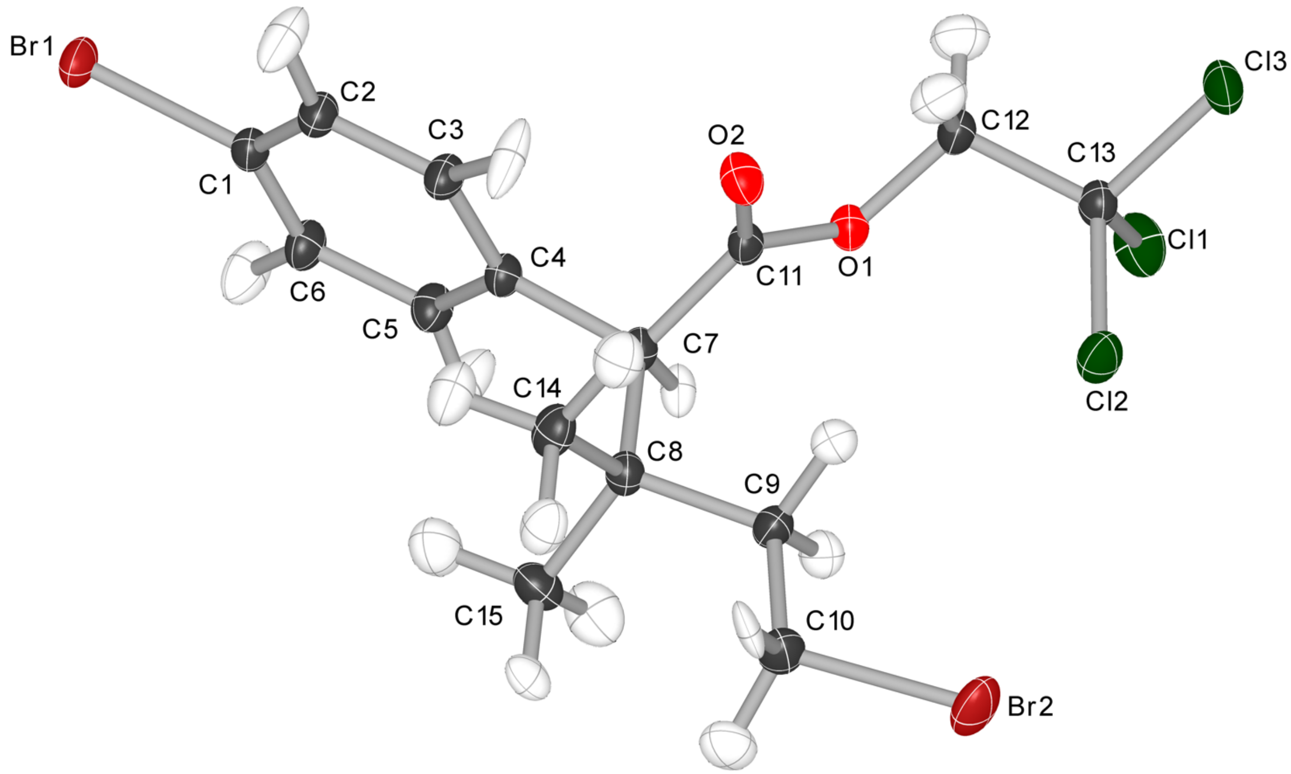

Figure 10.4.6 Thermal ellipsoidal representation of the independent molecule in the crystal structure (50% probabilities).

Data Plots: Diffraction Data

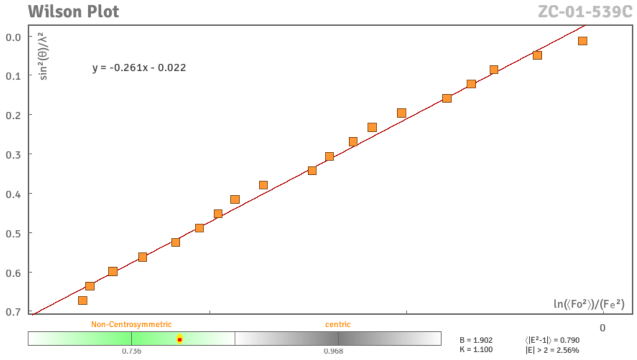

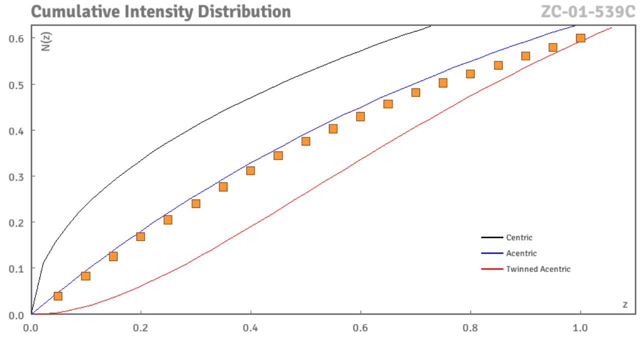

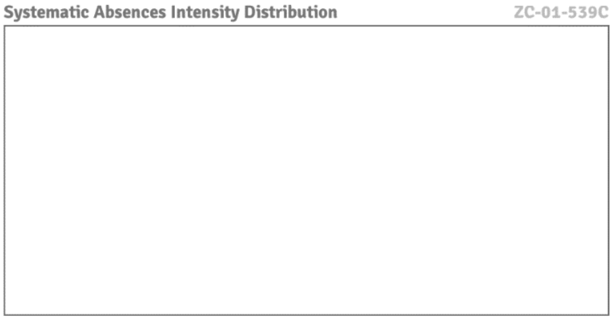

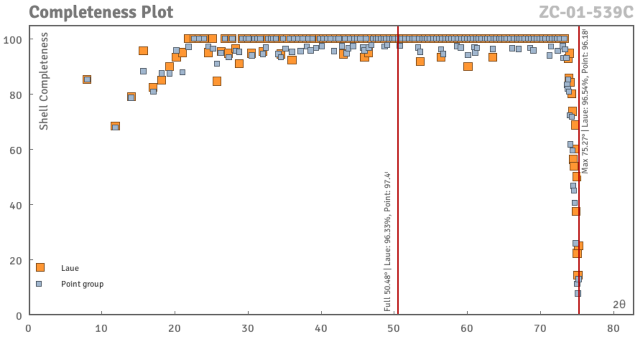

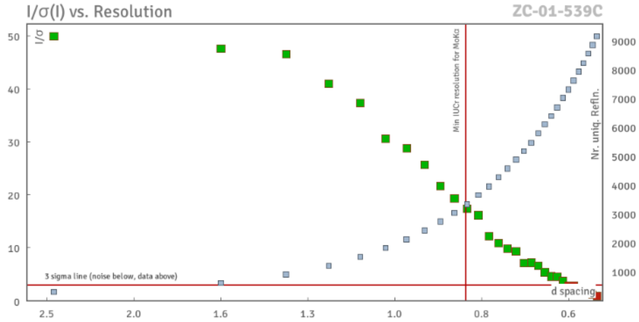

Data Plots: Refinement and Data

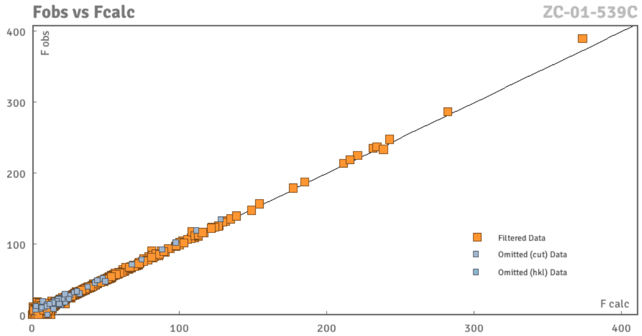

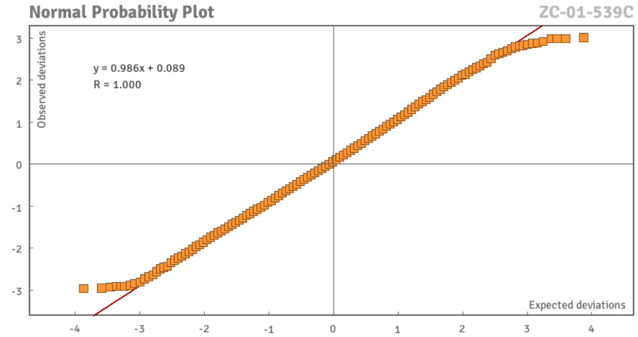

Reflection Statistics

Total reflections (after filtering)	65466	Unique reflections	9239
Completeness	0.962	Mean I/σ	16.24
hkl_{max} collected	(15, 17, 31)	hkl_{min} collected	(-15, -17, -32)
hkl_{max} used	(15, 17, 32)	hkl_{min} used	(-15, 0, 0)
Lim d_{max} collected	100.0	Lim d_{min} collected	0.36
d_{max} used	7.05	d_{min} used	0.58
Friedel pairs	11957	Friedel pairs merged	0
Inconsistent equivalents	0	R_{int}	0.0715
R_{sigma}	0.0482	Intensity transformed	0
Omitted reflections	886	Omitted by user (OMIT hkl)	0
Multiplicity	(11324, 7686, 6177, 3330, 1137, 264, 72, 4)	Maximum multiplicity	17
Removed systematic absences	0	Filtered off (Shel/OMIT)	0

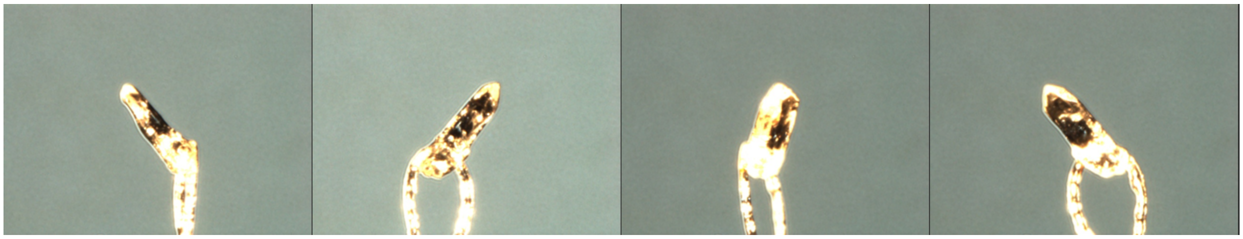

Table 10.4.17: Fractional Atomic Coordinates (×10[4]) and Equivalent Isotropic Displacement Parameters (Å[2]×10[3]) for **26e**. U_{eq} is defined as 1/3 of the trace of the orthogonalised U_{ij}.

Atom	x	y	z	U_{eq}
Br1	12467.6(4)	11177.5(4)	3540.1(2)	23.99(12)
Br2	1953.0(5)	5418.4(4)	3682.6(3)	35.16(17)
Cl1	7815.2(6)	2054.5(5)	3311.0(3)	30.14(11)
Cl2	5452.6(5)	3274.2(4)	4063.4(3)	25.8(1)
Cl3	7481.1(7)	1631.4(4)	4791.6(2)	26.71(9)
O1	8062.5(14)	4931.3(11)	3810.0(7)	20.2(2)
O2	7677.5(15)	6278.9(12)	4709.0(7)	23.8(3)
C1	10952.1(18)	9924.2(15)	3531.3(10)	18.6(3)
C2	10328.8(19)	9564.7(18)	4160.7(10)	20.3(3)
C3	9223(2)	8643.5(18)	4167.0(10)	20.0(3)
C4	8724.0(17)	8081.0(15)	3543.6(9)	16.9(3)
C5	9377(2)	8466.1(18)	2921.2(10)	20.3(3)
C6	10496(2)	9386.9(17)	2910.3(10)	20.9(3)
C7	7473.5(19)	7104.0(14)	3529.1(8)	16.9(2)
C8	5885.7(17)	7707.7(15)	3582.4(10)	18.0(3)
C9	4794.3(17)	6575.2(16)	3616.8(10)	19.9(3)
C10	3191.2(19)	6956.3(17)	3638.4(12)	24.9(4)
C11	7731.1(16)	6098.5(15)	4091.0(9)	17.2(3)
C12	8305.0(19)	3908.0(17)	4290.4(10)	22.2(3)
C13	7302.9(19)	2773.8(16)	4112.4(9)	20.0(3)
C14	5703(2)	8588.8(18)	4217.1(11)	23.7(4)
C15	5635(2)	8496.1(19)	2917.1(11)	24.6(4)

Table 10.4.18: Anisotropic Displacement Parameters (×10[4]) for **26e**. The anisotropic displacement factor exponent takes the form: $-2\pi^2[h^2a^{*2} \times U_{11} + ... +2hka^* \times b^* \times U_{12}]$

Atom	U_{11}	U_{22}	U_{33}	U_{23}	U_{13}	U_{12}
Br1	24.3(2)	20.99(19)	26.6(2)	-9.89(19)	-1.90(19)	3.44(16)
Br2	18.9(2)	29.4(2)	57.2(4)	-8.23(17)	-0.5(2)	-3.6(2)
Cl1	40.7(3)	23.5(2)	26.3(2)	3.16(18)	6.99(18)	-3.06(16)
Cl2	19.23(18)	22.1(2)	36.1(3)	-2.37(15)	1.14(17)	-1.80(18)
Cl3	38.1(2)	15.42(16)	26.6(2)	-2.93(19)	-4.6(2)	1.88(14)
O1	20.5(5)	15.8(5)	24.4(6)	-1.6(4)	-0.5(5)	1.2(4)
O2	31.9(7)	18.6(5)	21.0(6)	-0.9(5)	1.1(5)	2.0(4)
C1	17.7(6)	15.5(6)	22.5(8)	-1.7(5)	-0.4(5)	1.2(5)
C2	21.0(7)	18.5(7)	21.4(8)	-4.1(6)	-1.3(6)	0.1(6)
H2	42(12)	42(13)	22(3)	-26(7)	0(2)	-2(2)
C3	21.6(7)	20.2(8)	18.3(8)	-5.8(6)	-0.5(6)	1.0(6)
H3	56(13)	35(13)	20(4)	-32(7)	9(3)	-9(3)
C4	16.9(6)	15.8(6)	18.0(7)	-2.6(5)	1.2(5)	0.7(5)
C5	23.9(8)	20.4(8)	16.6(8)	-4.4(6)	2.0(6)	-0.1(6)
H5	52(12)	69(15)	18(3)	-39(7)	1(2)	-1(2)

Atom	U_{11}	U_{22}	U_{33}	U_{23}	U_{13}	U_{12}
C6	21.7(7)	20.8(8)	20.2(8)	-6.1(6)	3.1(6)	0.2(6)
H6	34(12)	37(11)	22(4)	-17(6)	5(3)	1(3)
C7	17.8(5)	13.6(5)	19.4(6)	-1.3(5)	1.2(5)	-0.2(4)
H7	27(8)	14(6)	19(3)	-4(3)	3(2)	-0.2(19)
C8	17.8(6)	15.3(6)	20.9(8)	-0.8(4)	0.9(5)	-0.3(5)
C9	17.4(6)	17.6(7)	24.7(9)	-1.9(5)	-2.4(6)	-0.7(6)
H9a	26(8)	19(6)	26(3)	1(3)	-3(2)	-1(2)
H9b	21(8)	23(6)	28(3)	-2(3)	-3(2)	2.8(19)
C10	17.8(7)	23.2(8)	33.6(11)	-0.6(5)	-1.7(7)	1.6(7)
H10a	21(10)	33(10)	36(5)	3(5)	0(3)	7(3)
H10b	34(10)	9(8)	31(4)	1(4)	3(3)	9(2)
C11	16.1(7)	14.3(6)	21.2(7)	-2.4(5)	0.5(5)	1.8(4)
C12	20.0(7)	16.2(7)	30.5(9)	-3.3(5)	-4.1(6)	0.5(6)
H12a	23(3)	33(8)	34(9)	2.6(18)	-4.4(18)	0(4)
H12b	25(8)	33(8)	33(3)	-1(4)	-3.9(19)	-4.4(19)
C13	20.9(8)	15.1(6)	24.1(8)	-1.0(5)	0.0(6)	-0.8(5)
C14	23.7(8)	19.0(9)	28.6(10)	-1.6(6)	2.0(7)	-6.5(6)
H14a	30(9)	26(7)	30(4)	-2(4)	1(2)	-3(2)
H14b	36(6)	29(5)	46(10)	-12(3)	-2(3)	-2(3)
H14c	25(3)	22(8)	34(9)	-0.9(19)	2(2)	-6(4)
C15	24.2(9)	24.2(10)	25.5(10)	3.6(7)	1.1(7)	6.6(6)
H15a	30(6)	32(5)	43(10)	-2(3)	1(3)	9(3)
H15b	45(10)	33(6)	31(5)	5(3)	3(3)	0(2)
H15c	25(3)	20(7)	19(9)	1.9(19)	1(2)	7(4)

Table 10.4.19: Bond Lengths in Å for **26e**.

Atom	Atom	Length/Å
Br1	C1	1.8931(16)
Br2	C10	1.9537(18)
Cl1	C13	1.7720(19)
Cl2	C13	1.7607(17)
Cl3	C13	1.7695(18)
O1	C11	1.360(2)
O1	C12	1.423(2)
O2	C11	1.202(2)
C1	C2	1.385(3)
C1	C6	1.380(3)
C2	H2	1.05(2)
C2	C3	1.386(3)
C3	H3	1.16(3)
C3	C4	1.406(3)
C4	C5	1.392(3)
C4	C7	1.522(2)
C5	H5	0.98(2)
C5	C6	1.394(3)
C6	H6	1.07(2)
C7	H7	1.10(2)

Atom	Atom	Length/Å
C7	C8	1.575(2)
C7	C11	1.519(2)
C8	C9	1.538(2)
C8	C14	1.532(3)
C8	C15	1.534(3)
C9	H9a	1.0992(12)
C9	H9b	1.0992(12)
C9	C10	1.509(2)
C10	H10a	1.0972(19)
C10	H10b	1.0973(19)
C12	H12a	1.097(2)
C12	H12b	1.111(18)
C12	C13	1.526(2)
C14	H14a	1.11(3)
C14	H14b	1.07(2)
C14	H14c	1.11(2)
C15	H15a	1.087(4)
C15	H15b	1.087(4)
C15	H15c	1.085(4)

Table 10.4.20: Bond Angles in ° for **26e**.

Atom	Atom	Atom	Angle/°
C12	O1	C11	116.20(14)
C2	C1	Br1	118.30(13)
C6	C1	Br1	120.22(13)
C6	C1	C2	121.48(15)
H2	C2	C1	124.7(13)
C3	C2	C1	119.28(16)
C3	C2	H2	116.0(13)
H3	C3	C2	125.5(12)
C4	C3	C2	120.79(16)
C4	C3	H3	113.7(12)
C5	C4	C3	118.30(14)
C7	C4	C3	122.13(15)
C7	C4	C5	119.54(15)
H5	C5	C4	121.2(14)
C6	C5	C4	121.30(17)
C6	C5	H5	116.9(14)
C5	C6	C1	118.85(16)
H6	C6	C1	118.6(13)
H6	C6	C5	122.4(13)
H7	C7	C4	106.4(11)
C8	C7	C4	114.61(12)
C8	C7	H7	108.4(13)
C11	C7	C4	109.25(14)
C11	C7	H7	106.1(11)
C11	C7	C8	111.59(13)
C9	C8	C7	106.77(12)
C14	C8	C7	112.84(14)
C14	C8	C9	110.59(15)
C15	C8	C7	107.10(14)
C15	C8	C9	110.33(15)
C15	C8	C14	109.14(14)
H9a	C9	C8	108.52(10)
H9b	C9	C8	108.52(10)
H9b	C9	H9a	107.5
C10	C9	C8	114.98(14)
C10	C9	H9a	108.52(11)
C10	C9	H9b	108.52(11)
C9	C10	Br2	110.01(12)
H10a	C10	Br2	105.2(12)
H10a	C10	C9	116.1(12)
H10b	C10	Br2	101.9(11)
H10b	C10	C9	114.0(12)
H10b	C10	H10a	108.4(3)
O2	C11	O1	122.64(15)
C7	C11	O1	111.35(14)
C7	C11	O2	126.01(15)
H12a	C12	O1	114.7(13)
H12b	C12	O1	107.8(12)
H12b	C12	H12a	107.3(13)
C13	C12	O1	109.75(14)
C13	C12	H12a	108.0(13)
C13	C12	H12b	109.2(13)
Cl2	C13	Cl1	109.16(10)
Cl3	C13	Cl1	109.50(9)
Cl3	C13	Cl2	108.91(10)
C12	C13	Cl1	111.26(13)
C12	C13	Cl2	110.68(12)
C12	C13	Cl3	107.27(12)
H14a	C14	C8	112.5(12)
H14b	C14	C8	109.0(15)
H14b	C14	H14a	115(2)
H14c	C14	C8	106.8(13)
H14c	C14	H14a	104.9(19)
H14c	C14	H14b	108.3(18)
H15a	C15	C8	110.7(11)
H15b	C15	C8	106.1(11)
H15b	C15	H15a	109.7(5)
H15c	C15	C8	111.0(10)
H15c	C15	H15a	109.8(5)
H15c	C15	H15b	109.6(5)

Table 10.4.21: Torsion Angles in ° for **26e**.

Atom	Atom	Atom	Atom	Angle/°
Br1	C1	C2	C3	-179.66(14)
Br1	C1	C6	C5	179.99(14)
Br2	C10	C9	C8	-179.91(12)
Cl1	C13	C12	O1	67.87(12)
Cl2	C13	C12	O1	-53.69(13)
Cl3	C13	C12	O1	-172.39(11)
O1	C11	C7	C4	-111.02(14)
O1	C11	C7	C8	121.20(13)
O2	C11	C7	C4	68.07(17)
O2	C11	C7	C8	-59.71(18)
C1	C2	C3	C4	-0.4(2)
C1	C6	C5	C4	-0.2(2)
C2	C3	C4	C5	0.5(2)
C2	C3	C4	C7	-177.69(16)

Atom	Atom	Atom	Atom	Angle/°
C3	C4	C5	C6	-0.12(19)
C3	C4	C7	C8	78.98(17)
C3	C4	C7	C11	-47.09(17)
C4	C7	C8	C9	-175.69(15)
C4	C7	C8	C14	-54.00(16)
C4	C7	C8	C15	66.13(16)
C7	C8	C9	C10	-177.45(15)

Table 10.4.22: Hydrogen Fractional Atomic Coordinates (×10[4]) and Equivalent Isotropic Displacement Parameters (Å[2]×10[3]) for **26e**. U_{eq} is defined as 1/3 of the trace of the orthogonalised U_{ij}.

Atom	x	y	z	U_{eq}
H2	10660(30)	9910(20)	4650(13)	35(6)
H3	8590(30)	8290(30)	4656(13)	37(7)
H5	8970(30)	8200(30)	2469(13)	46(7)
H6	10970(30)	9730(20)	2435(13)	31(6)
H7	7560(30)	6595(19)	3031(11)	20(3)
H9a	4973.0(17)	5955.1(17)	3160.3(11)	23(4)
H9b	5042.5(18)	5999.9(17)	4083.0(11)	24(3)
H10a	2780(20)	7503(16)	3189(5)	30(5)
H10b	2870(20)	7468(15)	4115(5)	25(4)
H12a	9442(10)	3550(20)	4309(11)	30(4)
H12b	8030(20)	4270(20)	4819(11)	30(4)
H14a	5730(30)	8050(20)	4715(13)	29(4)
H14b	6480(30)	9360(20)	4182(15)	37(4)
H14c	4580(30)	9000(20)	4187(13)	27(4)
H15a	6451(14)	9258(12)	2872(11)	35(4)
H15b	5755(19)	7825(15)	2487(8)	37(4)
H15c	4536(8)	8909(15)	2910(10)	21(4)

Citations

CrysAlisPro (ROD), Rigaku Oxford Diffraction, Poland (?).

CrysAlisPro Software System, Rigaku Oxford Diffraction, (2023).

L.J. Bourhis and O.V. Dolomanov and R.J. Gildea and J.A.K. Howard and H. Puschmann, The Anatomy of a Comprehensive Constrained, Restrained, Refinement Program for the Modern Computing Environment - Olex2 Disected, *Acta Cryst. A*, (2015), **A71**, 59-71.

O.V. Dolomanov and L.J. Bourhis and R.J. Gildea and J.A.K. Howard and H. Puschmann, Olex2: A complete structure solution, refinement and analysis program, *J. Appl. Cryst.*, (2009), **42**, 339-341.

Sheldrick, G.M., ShelXT-Integrated space-group and crystal-structure determination, *Acta Cryst.*, (2015), **A71**, 3-8.

11. Reference

(1) (a) Green, S. P; Wheelhouse, K. M.; Payne; A. D.; Hallett, J. P.; Miller, P. W.; & Bull, J. A. Thermal Stability and Explosive Hazard Assessment of Diazo Compounds and Diazo Transfer Reagents. *Org. Proc. Res. Dev.* **2019**. (b) Ackerman, L. K. G.; Martinez Alvarado, J. I.; Doyle, A. G. Direct C–C Bond Formation from Alkanes Using Ni-Photoredox Catalysis. *Journal of the American Chemical Society* **2018**, *140* (43), 14059-14063.

(2) Wieting, J. M.; Fisher, T. J.; Schafer, A. G.; Visco, M. D.; Gallucci, J. C.; Mattson, A. E. Preparation and Catalytic Activity of BINOL-Derived Silanediols. *European Journal of Organic Chemistry* **2015**, *2015* (3), 525-533.

(3) Valente, C.; Choi, E.; Belowich, M. E.; Doonan, C. J.; Li, Q.; Gasa, T. B.; Botros, Y. Y.; Yaghi, O. M.; Stoddart, J. F. Metal–organic frameworks with designed chiral recognition sites. *Chemical Communications* **2010**, *46* (27), 4911-4913.

(4) Lee, S. J.; Lin, W. A Chiral Molecular Square with Metallo-Corners for Enantioselective Sensing. *Journal of the American Chemical Society* **2002**, *124* (17), 4554-4555.

(5) Hodgson, D. M.; Selden, D. A.; Dossetter, A. G. Synthesis and evaluation of 4,4′,6,6′-tetrasubstituted binaphtholphosphate dirhodium(II) complexes as catalysts in enantioselective carbonyl ylide formation–cycloaddition reactions. *Tetrahedron: Asymmetry* **2003**, *14* (24), 3841-3849.

(6) Pirrung, M. C.; Zhang, J. Asymmetric dipolar cycloaddition reactions of diazocompounds mediated by a binaphtholphosphate rhodium catalyst. *Tetrahedron Letters* **1992**, *33* (40), 5987-5990.

(7) Liao, K.; Negretti, S.; Musaev, D. G.; Bacsa, J.; Davies, H. M. L. Site-selective and stereoselective functionalization of unactivated C–H bonds. *Nature* **2016**, *533* (7602), 230-234.

(8) Fu, J.; Ren, Z.; Bacsa, J.; Musaev, D. G.; Davies, H. M. L. Desymmetrization of cyclohexanes by site- and stereoselective C–H functionalization. *Nature* **2018**, *564* (7736), 395-399.

(9) Davies, H. M. L.; Hansen, T.; Churchill, M. R. Catalytic Asymmetric C−H Activation of Alkanes and Tetrahydrofuran. *Journal of the American Chemical Society* **2000**, *122* (13), 3063-3070.

(10) Wei, B.; Sharland, J. C.; Blackmond, D. G.; Musaev, D. G.; Davies, H. M. L. In Situ Kinetic Studies of Rh(II)-Catalyzed C–H Functionalization to Achieve High Catalyst Turnover Numbers. *ACS Catalysis* **2022**, *12* (21), 13400-13410.

(11) Liao, K.; Negretti, S.; Musaev, D. G.; Bacsa, J.; Davies, H. M. Site-selective and stereoselective functionalization of unactivated C-H bonds. *Nature* **2016**, *533* (7602), 230-234.

(12) Kurland, R. J.; Rubin, M. B.; Wise, W. B. Inversion Barrier in Singly Bridged Biphenyls. *The Journal of Chemical Physics* **1964**, *40* (8), 2426-2427.

(13) Kost, D.; Carlson, E. H.; Raban, M. The validity of approximate equations for kc in dynamic nuclear magnetic resonance. *Journal of the Chemical Society D: Chemical Communications* **1971**, (13), 656-657.

(14) *Gaussian 16,* Revision A.03, M. J. Frisch, G. W. Trucks, H. B. Schlegel, G. E. Scuseria, M. A. Robb, J. R. Cheeseman, G. Scalmani, V. Barone, G. A. Petersson, H. Nakatsuji, X. Li, M. Caricato, A. Marenich, J. Bloino, B. G. Janesko, R. Gomperts, B. Mennucci, H. P. Hratchian, J. V. Ortiz, A. F. Izmaylov, J. L. Sonnenberg, D. Williams-Young, F. Ding, F. Lipparini, F. Egidi, J. Goings, B. Peng, A. Petrone, T. Henderson, D. Ranasinghe, V. G. Zakrzewski, J. Gao, N. Rega, G. Zheng, W. Liang, M. Hada, M. Ehara, K. Toyota, R. Fukuda, J. Hasegawa, M. Ishida, T. Nakajima, Y. Honda, O. Kitao, H. Nakai, T. Vreven, K. Throssell, J. A. Montgomery, Jr., J. E. Peralta, F. Ogliaro, M. Bearpark, J. J. Heyd, E. Brothers, K. N. Kudin, V. N. Staroverov, T. Keith, R. Kobayashi, J. Normand, K. Raghavachari, A. Rendell, J. C. Burant, S. S. Iyengar, J. Tomasi, M. Cossi, J. M. Millam, M. Klene, C. Adamo,

R. Cammi, J. W. Ochterski, R. L. Martin, K. Morokuma, O. Farkas, J. B. Foresman, and D. J. Fox, *Gaussian, Inc., Wallingford CT*, **2016**.

(15). (a) Hay, P. J.; Wadt, W. R. Ab Initio Effective Core Potentials for Molecular Calculations. Potentials for the Transition Metal Atoms Sc to Hg. *J. Chem. Phys.* **1985**, *82*, 270-283. (b) Hay, P. J.; Wadt, W. R. Ab Initio Effective Core Potentials for Molecular Calculations. Potentials for K to Au Including the Outermost Core Orbitals. *J. Chem. Phys.* **1985**, *82*, 299-310. (c) Wadt, W. R.; Hay, P. J. Ab Initio Effective Core Potentials for Molecular Calculations. Potentials for Main Group Elements Na to Bi. *J. Chem. Phys*. **1985**, *82*, 284-298.

(16). (a) Becke, A. D. Density-Functional Exchange-Energy Approximation with Correct Asymptotic Behavior. *Phys. Rev. A* **1988,** *38*, 3098-3100. (b) Lee, C.; Yang, W.; Parr, R. G. Development of The Colle-Salvetti Correlation-Energy Formula into a Functional of the Electron Density. *Phys. Rev. B* **1988,** *37*, 785-789. (c) Becke, A. D. A New Mixing of Hartree–Fock and Local Density‐Functional Theories. *J. Chem. Phys.* **1993,** *98*, 1372-1377.

(17). Grimme, S., Hansen, A., Brandenburg, J. G. & Bannwarth, C. Dispersion-Corrected Mean-Field Electronic Structure Methods. *Chem. Rev.* **116**, 5105-5154 (2016).

(18). (a) Grimme, S.; Antony, J.; Ehrlich, S.; Krieg, H. A Consistent and Accurate Ab Initio Parametrization of Density Functional Dispersion Correction (DFT-D) for the 94 Elements H-Pu. *J. Chem. Phys.* **2010,** *132*, 154104-154122. (b) Becke, A. D.; Johnson, E. R. A Density-Functional Model of the Dispersion Interaction. *J. Chem. Phys.* **2005,** *123*, 154101-154106. (c) Becke, A. D.; Johnson, E. R. Exchange-Hole Dipole Moment and the Dispersion Interaction. *J. Chem. Phys.* **2005,** *122*, 154104-154109. (d) Johnson, E. R.; Becke, A. D. A Post-Hartree-Fock Model of Intermolecular Interactions: Inclusion of Higher-Order Corrections. *J. Chem. Phys.* **2006,** *124*, 174104-174112.

(19). (a) Barone, V.; Cossi, M. Quantum Calculation of Molecular Energies and Energy Gradients in Solution by a Conductor Solvent Model. *J. Phys. Chem. A* **1998,** *102*, 1995-2001. (b) Cossi, M.; Rega, N.; Scalmani, G.; Barone, V. Energies, structures, and electronic properties of molecules in solution with the C-PCM solvation model. *J. Comput. Chem.* **2003,** *24*, 669-681.

(20). McLarney, B. D.; Hanna, S. R.; Musaev, D. G.; France, S. A Predictive Model for the [$Rh_2(esp)_2$]-catalyzed Intermolecular C(sp^3)-H bond insertion of β-carbonyl ester carbenes: Interplay Theory and Experiment", *ACS Catal*, **2019**, 9, 4526-4538.

(21). Nguyen, T-T. H.; Bosse, A. T.; Ly, D.; Suarez, C. A.; Fu, J.; Shimabukuro, K.; Musaev, D. G.; Davies, H. M. L. Diaryldiazoketones as Effective Carbene Sources for Highly Selective Rh(II)-Catalyzed Intermolecular C–H Functionalization *J. Am. Chem. Soc.* **2024**, *146*, 8447–8455.
